# Creation of the HIV-1 antisense gene *asp* coincided with the emergence of the pandemic group M and is associated with faster disease progression

**DOI:** 10.1128/spectrum.03802-23

**Published:** 2024-01-17

**Authors:** Angelo Pavesi, Fabio Romerio

**Affiliations:** 1 Department of Chemistry, Life Sciences and Environmental Sustainability, University of Parma, Parma, Italy; 2 Department of Molecular and Comparative Pathobiology, Johns Hopkins University School of Medicine, Baltimore, Maryland, USA; National Institute of Allergy and Infectious Diseases, Baltimore, Maryland, USA

**Keywords:** HIV-1, antisense gene *asp*, disease progression, viral spread, pathogenesis

## Abstract

Despite being first identified more than three decades ago, the antisense gene *asp* of HIV-1 remains an enigma. *asp* is present uniquely in pandemic (group M) HIV-1 strains, and it is absent in all non-pandemic (out-of-M) HIV-1 strains and virtually all non-human primate lentiviruses. This suggests that the creation of *asp* may have contributed to HIV-1 fitness or worldwide spread. It also raises the question of which evolutionary processes were at play in the creation of *asp*. Here, we show that HIV-1 genomes containing an intact *asp* gene are associated with faster HIV-1 disease progression. Furthermore, we demonstrate that the creation of a full-length *asp* gene occurred via the evolution of codon usage in *env* overlapping *asp* on the opposite strand. This involved differential use of synonymous codons or conservative amino acid substitution in *env* that eliminated internal *stop* codons in *asp*, and redistribution of synonymous codons in *env* that minimized the likelihood of new premature *stops* arising in *asp*. Nevertheless, the creation of a full-length *asp* gene reduced the genetic diversity of *env*. The Luria-Delbruck fluctuation test suggests that the interrupted *asp* open reading frame (ORF) is the progenitor of the intact ORF, rather than a descendant under random genetic drift. Therefore, the existence of group-M isolates with a truncated *asp* ORF indicates an incomplete transition process. For the first time, our study links the presence of a full-length *asp* ORF to faster disease progression, thus warranting further investigation into the cellular processes and molecular mechanisms through which the ASP protein impacts HIV-1 replication, transmission, and pathogenesis.

**IMPORTANCE:**

Overlapping genes engage in a tug-of-war, constraining each other’s evolution. The creation of a new gene overlapping an existing one comes at an evolutionary cost. Thus, its conservation must be advantageous, or it will be lost, especially if the pre-existing gene is essential for the viability of the virus or cell. We found that the creation and conservation of the HIV-1 antisense gene *asp* occurred through differential use of synonymous codons or conservative amino acid substitutions within the overlapping gene, *env*. This process did not involve amino acid changes in ENV that benefited its function, but rather it constrained the evolution of ENV. Nonetheless, the creation of *asp* brought a net selective advantage to HIV-1 because *asp* is conserved especially among high-prevalence strains. The association between the presence of an intact *asp* gene and faster HIV-1 disease progression supports that conclusion and warrants further investigation.

## INTRODUCTION

Viral genomes increase the amount of genetic information by encoding overlapping genes on multiple reading frames. This strategy is especially impactful for small genomes, often serving as a source of novel accessory proteins (1, 2). Sense overlapping genes are encoded on the same DNA strand, whereas antisense overlapping genes are encoded on opposite DNA strands.

Although the majority of overlapping genes are of same-strand type, two antisense overlaps have been found in human retroviruses. The pX region of the human T-lymphotropic virus 1 (HTLV-1) genome encodes the N-terminal half of p30 in the sense strand and HBZ in the antisense strand (3–5). The pX region is a fascinating case of “gene nursery” also containing a triple same-strand overlap that includes Rex, Tax, and the C-terminal half of p30 (6).

The HIV-1 genome encodes ENV in the sense strand, while the antisense strand is predicted to encode an antisense protein (ASP) of 150–190 residues possibly associated with cellular membranes (7, 8). *In vitro* studies documented ASP expression on the surface of productively infected cells and on the viral envelope (8). Direct demonstration that ASP is expressed *in vivo* is still lacking, partly because ASP is difficult to detect due to low expression, rapid turnover, physical properties, and binding to putative partners (9, 10). Currently, only indirect evidence is available in the form of humoral and cellular immune responses against ASP during natural infection (11–15). Moreover, the role of ASP in HIV-1 replication and spread remains largely unknown.

Theoretical evidence suggesting that the *asp* open reading frame (ORF) is a real protein-coding gene came from the finding that a full-length ORF (>150 codons) is present exclusively in pandemic HIV-1 strains of group M, while it is virtually absent in all other human and non-human lentiviruses (non-pandemic HIV-1 groups N, O, and P, HIV-2, and SIV infecting African apes and Old-World monkeys) (16). Among the immediate progenitors of HIV-1 group M strains, a full-length *asp* ORF is found only in strain MB66 of SIV infecting chimpanzees (SIVcpz) (16, 17). The frequency of HIV-1 strains within each subtype that contains a full-length *asp* ORF correlates with the prevalence of the subtype itself. Subtype A represents a special case, where an early *stop* codon downstream of the canonical *start* interrupts the ORF, and the creation of a novel *start* codon following the premature *stop* reopened the ASP reading frame (16).

However, those studies did not prove whether the *asp* ORF originated recently when the pandemic group M diverged from SIVcpz or rather it originated in a progenitor lentivirus and later disappeared in all descendants except group-M strains. Resolving this point could shed light on a possible role of ASP in the worldwide spread of group M. On one hand, the lack of a full-length *asp* ORF in 25%–30% of group-M subtypes (or 16% of strains from the high-prevalence subtypes A, B, C, and G plus CRF01-AE) is not negligible (16, 18), and it may suggest an ancient origin of ASP, which later became obsolete and dispensable. On the other hand, our recent analyses on a broad range of human and non-human primate lentiviruses found evidence of selective pressure leading to the creation of an intact *asp* ORF in the pandemic HIV-1 group M through conservation of the *start* codon and avoidance of internal *stop* codons, which suggests a recent origin of ASP (18). However, our recent report did not include rigorous analyses that would confirm or disprove that preliminary conclusion (18). Moreover, it is difficult to reconcile the high frequency (~70%–75%) of group-M strains containing an intact *asp* ORF with the notion that this is a pseudogene or a gene encoding a protein with no function. Furthermore, systematic studies on *de novo* accessory proteins expressed by sense overlapping genes showed that “accessory” does not mean “dispensable,” because most novel proteins provide a selective advantage during viral pathogenesis or spread (1, 19). The higher frequency of intact *asp* ORF over the truncated one, especially in high-prevalence clades, is consistent with that. Finally, the frequency of HIV-1 strains in high prevalence subtypes that lack an intact *asp* ORF (15%) is similar to the frequency of strains lacking an intact *nef* gene [13.5% (16, 20)], which indicates that the absence of an intact accessory gene in a non-negligible percentage of viral isolates is not unique to *asp*.

Here, we addressed the hypothesis that the truncated *asp* ORF is the progenitor of the intact ORF, rather than a descendant under random genetic drift. Consequently, progressive removal of internal *stop* codons from the pre-existing interrupted *asp* ORF would be an ongoing evolutionary process leading to the creation and fixation of an intact ORF in pandemic group-M strains. The peculiar distribution of *stop* codons in the truncated *asp* ORF that we identified in our study strongly supports the view that it is ancestral, not descendant, of the intact one. We found a significantly higher frequency of intact *asp* ORF in rapid progressor compared to long-term non-progressor individuals. Altogether, our studies support a recent origin of the *asp* ORF and its role in promoting spread or pathogenesis.

## RESULTS

### The presence of a full-length *asp* ORF is associated with faster HIV-1 disease progression

A previous report investigated the evolution of the *asp* ORF in human and non-human primate lentiviruses by analyzing >23,000 *env* sequences from ~4,000 people living with HIV-1 (PLWH) (16). However, studies have shown that as many as 90% of HIV-1 proviral sequences from PLWH under suppressive antiretroviral therapy (ART) are mutated and replication-defective (21).

Here, we sought to investigate the association between an intact *asp* ORF and disease progression by analyzing exclusively sequences from intact, replication-competent HIV-1 isolates (see Materials and Methods for detailed description). We used the Los Alamos HIV Database to build a data set of *env* sequences covering the entire *asp* ORF, from canonical *start* codon to canonical *stop* codon. These sequences were from three groups of PLWH not undergoing antiretroviral therapy: rapid progressors (RP; progress to AIDS in <3 years after primary infection), slow progressors (SP; progress to AIDS in 7–10 years), and long-term non-progressors (LTNP; progress to AIDS in >12 years). To ensure that the sequences were derived from intact, replication-competent isolates, we restricted our search to (i) RNA sequences from patient plasma samples, (ii) DNA sequences from *in vitro* viral outgrowth assays, and (iii) DNA sequences from peripheral blood mononuclear cell (PBMC) samples of PLWH off antiretroviral therapy, which reflect recent infection events and are less likely to belong to defective proviruses. This selection yielded 1,222 sequences from 32 RP (File S1), 1,015 from 76 SP (File S2), and 814 from 75 LTNP (File S3). For 14 of 32 RP, we found only one *env* sequence, and for the remaining 18 RP, the number of *env* sequences ranged from 3 to 391. For 30 of 76 SP, we found only one *env* sequence, and for the remaining 46 SP, we retrieved a range of 2–125 *env* sequences. Finally, for 30 out of 75 LTNP, we found only one *env* sequence and for the remaining 45 LTNP, we retrieved a number of *env* sequences ranging from 2 to 186. The percentage of donors in each of the three groups of PLWH for whom only one sequence was available (43.8% for RP, 39.5% for SP, and 40% for LTNP) was not significantly different (RP vs SP, χ^2^ = 0.17; *P* = 0.68; RP vs LTNP, χ^2^ = 0.13; *P* = 0.72; SP vs. LTNP, χ^2^ = 0.04; *P* = 0.95).

**Fig 1:**
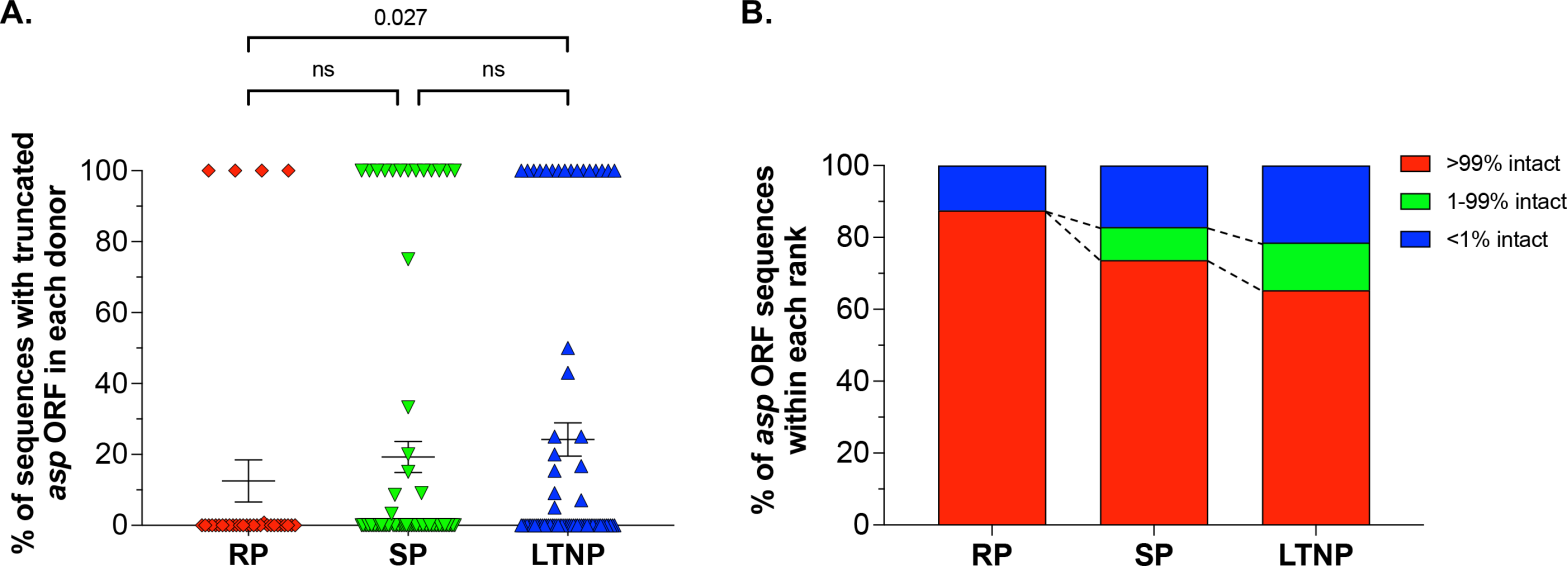
Frequency of truncated *asp* ORF in 32 rapid progressors (1,222 sequences), 76 slow progressors (1,015 sequences), and 75 long-term non-progressors (814 sequences). All sequences encompass the genomic region containing the *asp* ORF from canonical *start* to canonical *stop*. (A) Frequency of interrupted *asp* ORFs found in each PLWH in the three cohorts (red rhombi, RP; green inverted triangles, SP; and blue triangles, LTNP), as well as the mean and standard error of the mean (SEM) of each cohort. (B) Frequencies of truncated *asp* ORF sequences in each patient of the three cohorts grouped in three ranks: 0%–1% truncated *asp* ORFs (red), 1%–99% truncated *asp* ORFs (green); and 99%–100% truncated *asp* ORFs (blue).

We determined the percentage of *env* sequences that contain a truncated *asp* ORF for each individual PLWH in the data set (i.e., not the overall percentage of sequences with a truncated *asp* ORF for each of the three groups of PLWH). Then, we compared the distribution of the percentage of sequences with interrupted *asp* ORF for the PLWH within each group (percentage distribution for RP vs SP vs LTNP). Using the Mann-Whitney *U* test, we found a significantly lower percentage of sequences with truncated *asp* ORFs among RP compared to LTNP (12.5% vs 24.2%; *U* = 978.5; *P* = 0.027) (Fig. 1A). No statistically significant difference was found between RP and SP, and between SP and LTNP. Importantly, >95% of sequences in all three data sets are from subtypes B and C. These subtypes have very high global prevalence (12% and 48%, respectively), and a high frequency of isolates with intact *asp* (85% and 84%, respectively) (16). Therefore, these results are not due to different subtype compositions of the three data sets. We also did not observe significant differences in terms of ART regimen among the three data sets. Sequences from all LTNPs, from 75 of 76 SP, and from 27 of 32 RP were obtained from samples collected while the individuals were off ART.

We then distributed the sequences from PLWH of each group into three ranks: sequences with <1% intact *asp* ORF, 1%–99% intact *asp* ORF, and >99% intact *asp* ORF. We found a gradual decline of sequences with intact *asp* ORF in PLWH with slow disease progression and long-term non-progression. This decline was compensated by a gradual increase of sequences with truncated *asp* ORF (Fig. 1B).

Overall, these results identify an association between the frequency of sequences containing an intact *asp* ORF and disease progression, and they highlight a trend toward increased frequency of truncated *asp* ORF in individuals who typically progress to AIDS more slowly. Thus, our results suggest a role for full-length ASP protein in promoting intra-host viral spread or pathogenesis, as previously suggested (16).

### The region of *env* overlapping *asp* is under strong selection pressure when compared to the region of *env* outside the overlap

To test whether the *env* region overlapping *asp* is under selection pressure, we recovered (18) two data sets containing the entire nucleotide sequence of *env* from our previous study. The first data set includes 3,725 *env* sequences in which the region of *env* overlaps a full-length *asp* ORF (no premature *stops* in *asp*; ENV_FL-ASP_ data set). The second data set includes 1,376 *env* sequences in which the region of *env* overlaps a truncated *asp* ORF having a number of premature *stop* codons from 1 to 5 (ENV_ΔASP_ data set). All the sequences belong to HIV-1 group M, and B, C, and CRF01-AE are the most prevalent subtypes in both data sets. We merged the two data sets into a single data set of 5,101 complete *env* sequences. They are aligned over a total of 1,274 codon positions, including gapped positions, with the region overlapping *asp* located between positions 606 and 894, and the *asp* ORF delimited by canonical *start* and *stop* codons.

We analyzed all codon positions, except those with a frequency of gaps >5%, and identified 595 positions with at least one CAN or TAN codon (where N represents any nucleotide). The CAN and TAN codons in *env* are potential hotspots for the appearance of *stop* codons in the –2 antisense reading frame (reverse complements are TG and TA, respectively). Since the first, second, and third codon positions of *env* overlap the second, first, and third codon positions of *asp*, respectively, a substitution yielding T or C in the third base of the codon immediately upstream of the CAN or TAN codon in *env* would result in TGA, TAA, or TAG *stop* codons in the antisense frame.

**Fig 2:**
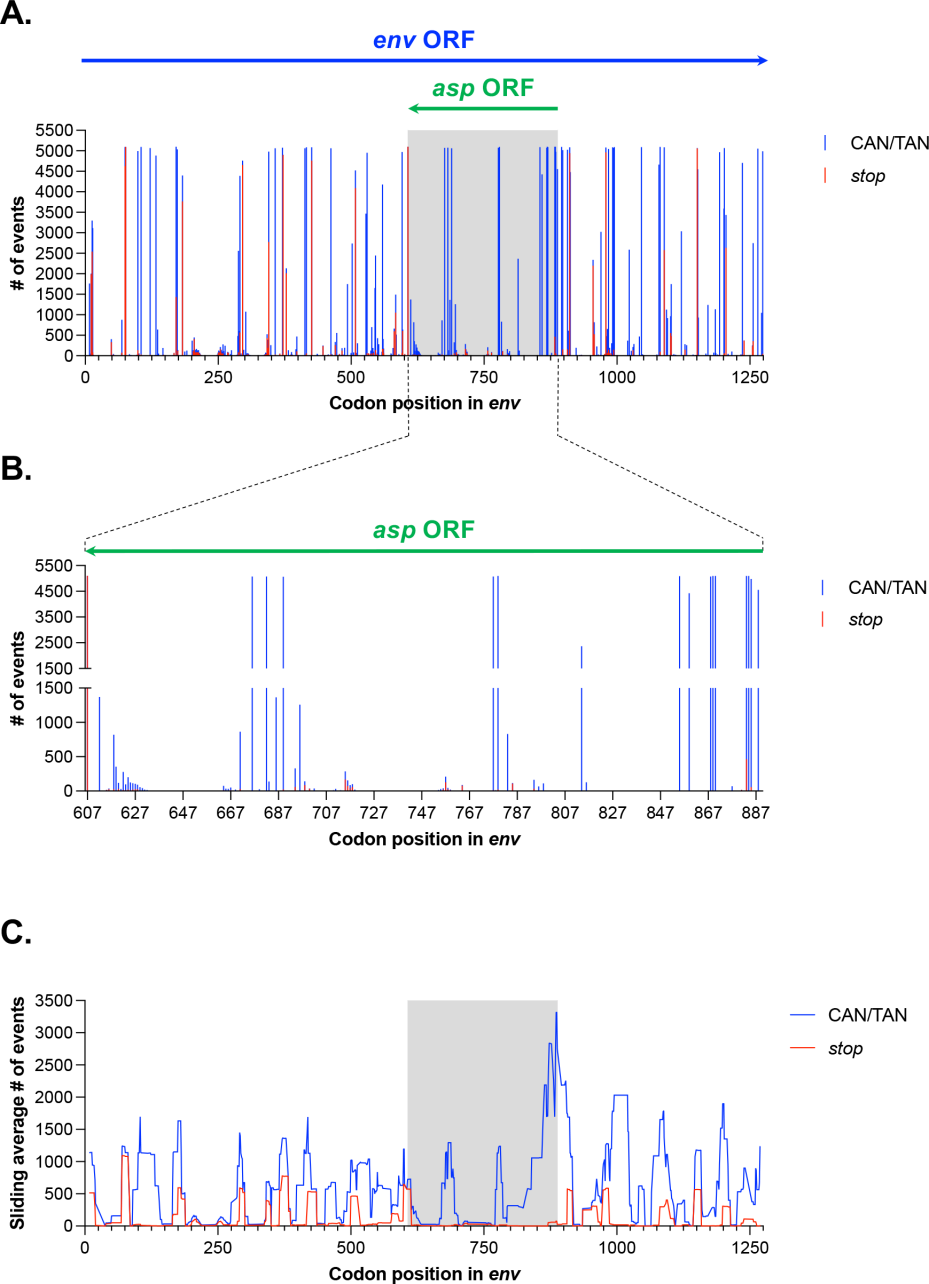
(A) Distribution of the number of CAN/TAN codons (blue peaks) and the number of *stop* codons in the –2 reading frame (red peaks) along 5,101 *env* sequences aligned over a total of 1,274 codon positions. (B) Enlarged view of the distribution of CAN/TAN codons in *env* (blue peaks) and *stop* codons (red peaks) within the *asp* ORF. (C) Moving average (window scanning of 10 codon positions and steps of one position) of the number of CAN/TAN codons (blue profile) and *stop* codons (red profile) in the –2 reading frame.

By moving along the aligned *env* sequences, we counted the number of CAN/TAN codons in each of the 595 codon positions (blue columns in Fig. 2A) and the respective number of *stop* codons in the –2 reading frame (red columns in Fig. 2A). In the region of *env* outside the overlap, we identified 480 positions with at least one CAN or TAN codon and calculated a mean number of *stops* in the –2 reading frame of 161.7. In the region of *env* overlapping *asp,* we identified 115 codon positions with at least one CAN or TAN codon. Leaving out position 607, which coincides with the canonical *stop* codon of *asp* (see inset of Fig. 2A), we calculated a mean number of *stops* in the –2 reading frame of 14.1 (one order of magnitude lower than that outside the overlap; Student’s *t* = 4.38; *P* = 10^−5^). Figure 2B provides an enlarged view of the CAN/TAN codons in *env* (blue peaks) and corresponding *stop* codons in *asp* (red peaks) inside the region of overlap. Importantly, the regions within and outside the overlap show very similar mean content of CAN/TAN codons (mean = 14.8%, sd = 33% and mean = 12.3%, sd = 29.0%, respectively; Student’s *t* = 0.75; *P* = 0.45). Therefore, the significantly lower mean number of *stops* in the overlap compared to the region outside the overlap is not the consequence of a lower frequency of CAN/TAN codons within the overlap.

By moving along the aligned *env* sequences with a window scanning of 10 codon positions and a step of one position, we calculated the moving average of the number of CAN/TAN codons and that of *stop* codons in the –2 reading frame (see, respectively, the blue peaks and the read peaks in Fig. 2C). We observed a remarkably high difference between the distribution of blue and red peaks in the *env* overlapping region (gray box) and that in the *env* non-overlapping region. Altogether, these results support the existence of selection pressures that drive the creation of an antisense ORF encoding a full-length, functional product.

### Identification of the codon positions that are the main determinants of the difference between *env* overlapping a full-length *asp* and *env* overlapping a truncated *asp*

We extracted 3,725 gene regions of *env* overlapping a full-length *asp* from the ENV_FL-ASP_ data set (File S4) and 1,376 gene regions of *env* overlapping a truncated *asp* from the ENV_ΔASP_ data set (File S5). They are aligned over a total of 289 codon positions, which are located from position 606 to 894 in the aligned entire gene *env*. Based on the classification in The Los Alamos HIV database, the great majority of *env* sequences in the two data sets (97% for ENV_FL-ASP_ data set and 88% for ENV_ΔASP_) belong to 59 common subtypes, in which B, C, A1, CRF01-AE, and CRF02_AG are the most prevalent.

In the ENV_ΔASP_ data set, we detected a total of 92 codon positions having at least one premature *stop* in the antisense *asp* ORF. Under the rule of a frequency of *stops* >1.5%, we identified 13 codon positions, with position 883 accounting for the highest number of *stops* (465 out of 1,994; 23.3%) and position 794 for the lowest number of *stops* (35 out of 1,994; 1.8%). Taken together, the 13 codon positions account for 70% of the total number of *stops* (1,390 out of 1,994). We examined these codon positions in the ENV_FL-ASP_ and found that 11 of them show a substantial decrease in the frequency of CAN codons when compared to the ENV_ΔASP_ data set (Table 1).

**TABLE 1:** Codon positions that are the main determinants of the difference between *env* overlapping an intact *asp* ORF (ENV_FL-ASP_) and *env* overlapping an interrupted *asp* ORF (ENV_ΔASP_)

Codon position in *env*	*Stop* codons in the *asp* ORF^*a*^ number (%)	% frequency of CAN codons in ENV_FL-ASP_ data set	% frequency of CAN codons in ENV_ΔASP_ data set	Difference frequency of CAN codon between ENV_FL-ASP_ data set and ENV_ΔASP_ data set
715	168 (8.4)	1.9	11.6	–9.7
757	126 (6.3)	1.9	11.7	–9.8
785	109 (5.5)	0.1	8.0	–7.9
698	86 (4.3)	0.4	7.9	–7.5
764	77 (3.9)	0.2	5.7	–5.5
716	76 (3.8)	1.3	6.4	–5.1
694	59 (3.0)	2.9	15.9	–13.0
718	59 (3.0)	0.8	4.6	–3.8
717	40 (2.0)	1.1	3.1	–2.0
700	35 (1.8)	0.0	2.3	–2.3
794	35 (1.8)	0.9	8.3	–7.4
883^*b*^	465 (23.3)	100.0	100.0	0.0
885^*b*^	55 (2.8)	97.1	98.4	–1.3

^*a*^ Data in parenthesis are the percent frequency of *stop* codons over a total of 1,994 *stop* codons.

^*b*^ For codon positions 883 and 885, the loss of a *stop* in *asp* and creation of a full-length ORF are due to a nucleotide substitution in the third base of the preceding codon of *env* (882 and 884, respectively). See text for details.

**Fig 3:**
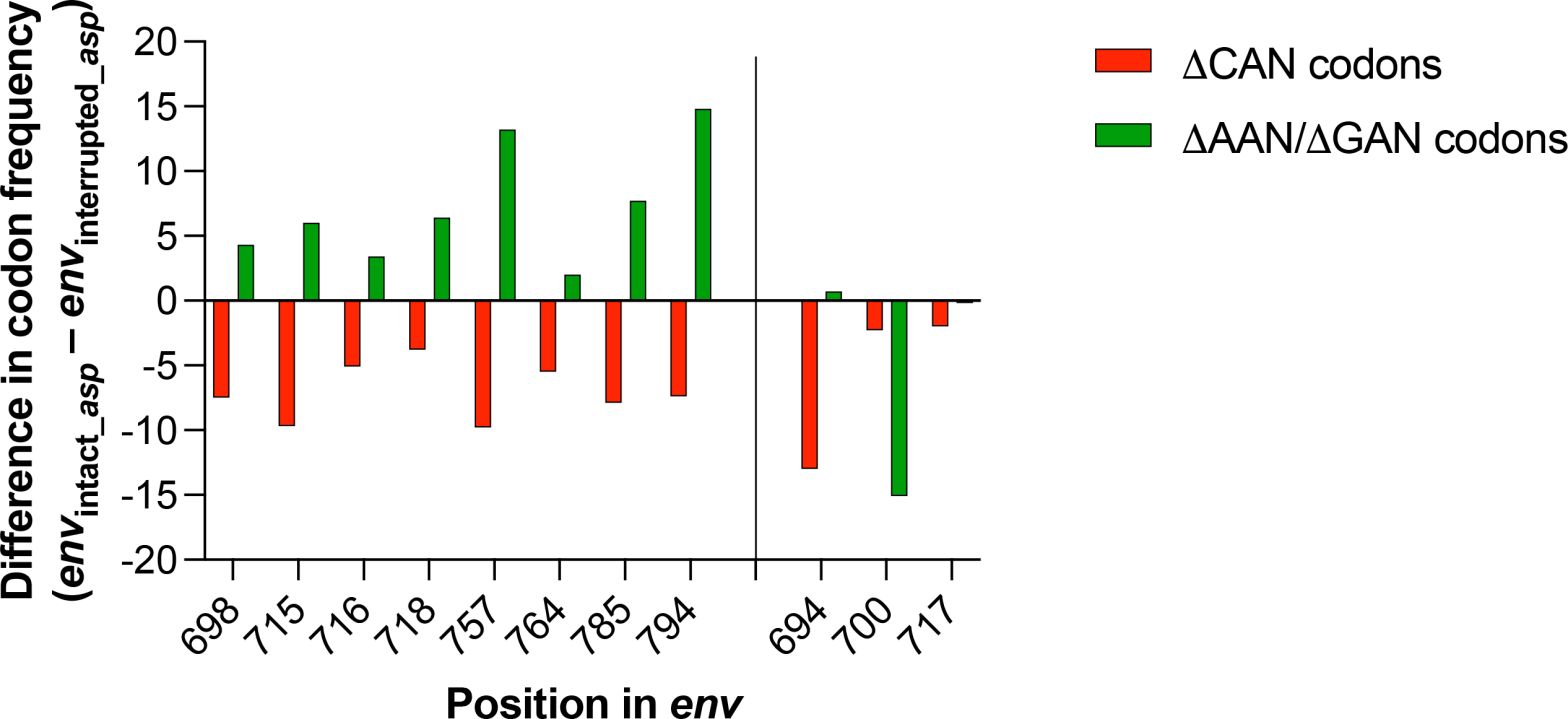
Eleven codon positions largely responsible for the difference between *env* overlapping and intact *asp* ORF and *env* overlapping a truncated *asp* ORF through the loss of CAN codons (ΔCAN). For eight positions (698, 715, 716, 718, 757, 764, 785, and 794), the loss of CAN codons (red columns) in *env* overlapping an intact *asp* ORF is counterbalanced by a significant increase in the content of AAN/GAN codons (green columns). For the remaining three positions (694, 700, and 717), the CAN codon is replaced by codons other than AAN/GAN.

The CAN codons encode *Gln* using CAA and CAG and *His* using CAT and CAC. The codons that differ from CAN at first codon position encode *Asn* (AAT and AAC), *Lys* (AAG and AAA), *Asp* (GAT and GAC), and *Glu* (GAA and GAG). Based on the PAM240 scoring matrix (22), substitutions of *Gln* or *His* with *Asn*, *Lys*, *Asp*, and *Glu* are of conservative type. Indeed, the PAM240 score of these amino acid substitutions is always greater than zero, with the exception of the substitution of *His* with *Lys* (score = 0). Based on these notions, we expect to find that the decrease in CAN codons observed in the 11 codon positions of ENV_FL-ASP_ (Table 1) should be paired with an increase in the content of AAN and GAN codons when compared to ENV_ΔASP_. Using the contingency χ^2^ test, we found that 8 of the 11 codon positions show: (i) a statistically significant decrease in the content of CAN codons (*P* = 10^−5^ in all cases) and (ii) a significant increase in the content of AAN/GAN codons (*P* = 0.03 at position 716; *P* = 0.01 at positions 698 and 764, *P* = 10^−4^ at positions 715 and 718, and *P* = 10^−5^ at positions 757, 785, and 794) (Fig. 3). For three positions (694, 700, and 717), the loss of CAN codon in *env* overlapping an intact *asp* ORF is due to replacement with codons other than AAN/GAN (Fig. 3).

In the case of codon positions 883 and 885 with a frequency of *stops* in the antisense *asp* ORF > 1.5%, they account, respectively, for 23.3% and 2.8% of the total amount of *stops* in *asp*. In ENV_FL-ASP_ and ENV_Δ-ASP_, these two positions show similar content in CAN codons, equal to 100% (Table 1). Indeed, the main difference between *env* with full-length *asp* and *env* with truncated *asp* concerned the codon position –1. At codon position 882, we found a strong decrease (−33%) of T-ending codons in ENV_FL-ASP_, when compared to ENV_ΔASP_. Specifically, we found that the frequency of GCT (*Ala*) in ENV_FL-ASP_ drops to zero, while that of GCG (*Ala*) increases to 97.1%. Therefore, the transition to a full-length *asp* ORF is due to a replacement of GCT with the synonym GCG. Since myeloid and lymphoid cells show a bias for GCT over GCG (23), the use of the latter is not expected to provide an advantage for the expression of *env* but rather only to remove a *stop* codon in the *asp* ORF. At codon position 884, we found a substantial decrease (−4.0%) of T-ending codons in ENV_FL-ASP_, when compared to ENV_ΔASP_. It was due to the loss of CAT (*His*), which was replaced by the synonym CAC and the quasi-synonymous CAG (*Gln*). Therefore, the loss of T-ending codons in *env* inhibits the appearance of the TGA *stop* codon in the antisense frame.

It should be noted that codon positions 882 and 884 fall within the RRE element, but neither one is critical for Rev-RRE interaction. Indeed, the portion of RRE that overlaps both *asp* and *env* covers codon positions 819 through 894 of *env*, while the portion of RRE that overlaps *env* outside of *asp* covers *env* positions 895 through 913 (plus the first two nucleotides of *env* codon 914). Codon positions 882 and 884 of *env* map in Stem IV of RRE, whereas the domains of RRE that contain high- and low-affinity Rev-binding sites are located in Stems IIB and IA, respectively (Fig. S1) (24, 25). Thus, the two nucleotide substitutions at positions 882 and 884 of *env* inside RRE that eliminate *stops* in *asp* do not appear to be driven by modulation of the affinity for Rev.

No appreciable difference in the frequency of TAN codons was observed between ENV_FL-ASP_ and ENV_ΔASP_ (Table S1).

Altogether, these results identify 13 codon positions in *env* that are critical for the presence of a full-length *asp*. In 10 out of 13, the creation of an antisense ORF not interrupted by premature *stops* occurred via nucleotide substitutions yielding a conservative amino acid change in ENV (eight codon positions; Table 1; Fig. 3) or via synonymous substitutions (two codon positions; Table 1).

### Creation of a new *start* codon in the *asp* ORF of subtype A strains

We analyzed the special case of 377 *env* sequences from HIV-1 subtype A (File S6) where the *asp* ORFs contain *start* and *stop* codons at canonical positions, but they are interrupted by a premature *stop* codon. However, the creation of a new *start* codon downstream of the premature *stop* reopened the *asp* ORF that expresses a protein with an N-terminal deletion (16).

In 97% of the sequences, the premature *stop* in *asp* and the new start codon reopening the *asp* ORF coincide, respectively, with positions 882–883 and 865–866 in *env*. We found that the new *start* codon was created by 100% frequency of the GGC (*Gly*) codon at position 865 of *env* followed by 100% ATA (*Ile*) at position 866, which in the reverse complementary gives rise to ATG. In the ENV_FL-ASP_ and ENV_Δ-ASP_ data sets, we found at position 865 of *env* only 4.7% and 11.3%, respectively, of GGC, and 95.3% and 88.7%, respectively, of the synonymous GGT, GGA, or GGG codons. Therefore, in 97% of subtype A strains, the new *start* codon reopening the *asp* ORF is due to a different use of synonyms for glycine (GGC instead of GGT, GGA, or GGG) at position 865 of *env*. This nucleotide substitution does not impact the amino acid sequence of ENV, and it appears to benefit exclusively the expression of ASP, albeit with an N-terminal deletion. Importantly, the position of the new *start* codon preserves both transmembrane domains and the extracellular loop of ASP, suggesting that the truncated protein is still able to associate with cellular membranes and viral envelope (8).

### Intact *asp* ORF: ancestral or descendant of truncated *asp* ORF?

To test the hypothesis that the truncated *asp* ORF originated from nucleotide substitutions occurring in a pre-existing intact *asp* ORF, we extracted a first subset of 1,625 gene regions of *env* overlapping an intact *asp* from the ENV_FL-ASP_ data set, and a second subset of 339 gene regions of *env* overlapping a truncated *asp* from the ENV_Δ-ASP_ data set. All the selected sequences belong to subtype B, and they are aligned over a total of 289 codon positions, which are located from position 606 to 894 in the aligned entire gene *env*.

We found 12 codon positions with a frequency of CAN/TAN codons higher than 95% in both subsets. They are the codon positions 676, 777, 779, 814, 855, 868, 869, 870, 883, 884, 885, and 888. As reported above, these positions are potential hotspots for the appearance of *stops* in the –2 antisense reading frame, because their reverse complements are TG and TA, respectively, and a substitution yielding T or C in the third base of codon position −1 would result in a synonymous or quasi-synonymous substitution in *env* and TGA, TAA, or TAG in *asp*.

If the truncated *asp* ORF originated from an intact one that lost the ability to encode a full-length ASP due to a T or C substitution in the third base of codon position –1, we should expect to find a poor fluctuation in the number of *stop* codons in the antisense frame at the 12 codon positions. In contrast to expectation, we found a fluctuation ranging from 0 to 22 *stops* (Table 2). Using the Luria-Delbruck fluctuation test (21, 22), we found that fluctuation around the mean number of *stops* (3.5) is statistically significant (χ^2^ = 124.9; *P* = 10^−5^).

**TABLE 2:** Luria-Delbruck fluctuation test for sequences from HIV-1 group M subtypes B and C

Subtype B		Subtype C	
Codon positions in the *env* region overlapping *asp* with CAN/TAN codon frequency >95%^*a*^	Number of *stop* codons in the antisense frame of 339 *env* sequences overlapping an interrupted *asp* ORF^*b*^	Codon positions in the *env* region overlapping *asp* with CAN/TAN codon frequency >95%^*c*^	Number of *stop* codons in the antisense frame of 198 *env* sequences overlapping an interrupted *asp* ORF^*d*^
676	0	676	0
777	8	777	0
779	5	779	3
814	0	855	1
855	0	859	0
868	0	868	0
869	1	869	0
870	2	870	0
883	1	883	4
884	2	884	4
885	22	885	8
888	1	888	0

^*a*^ 1,625 *env* sequences overlapping an intact *asp* ORF and 339 *env* sequences overlapping an interrupted *asp* ORF.

^*b*^ x^2^ = 124.9; *P* = 10^-5^.

^*c*^ 968 *env* sequences overlapping an intact *asp* ORF and 198 *env* sequences overlapping an interrupted *asp* ORF.

^*d*^ x^2^ = 43.6; *P* = 10^-5^.

We carried out the same analysis using a first subset of 968 gene regions of *env* overlapping an intact *asp* and a second subset of 198 gene regions of *env* overlapping a truncated *asp*. In this case, all the selected sequences belong to subtype C. We found 12 codon positions with a frequency of CAN/TAN codons higher than 95% in both subsets: 676, 777, 779, 855, 859, 868, 869, 870, 883, 884, 885, and 886. As in the case of subtype B strains, we also found a substantial fluctuation in the number of *stop* codons in the antisense for subtype C strains (from 0 to 8, see Table 2). Indeed, the Luria-Delbruck test revealed that fluctuation around the mean number of *stops* (1.7) is statistically significant (χ^2^ = 43.6; *P* = 10^−5^).

Altogether, these results do not support the hypothesis that the truncated *asp* ORF originated from the intact one. Rather, they suggest that the truncated *asp* ORF is ancestral and the intact one is a descendant. In agreement with this view, the progressive removal of internal *stop* codons in a pre-existing interrupted *asp* ORF is an evolutionary process leading to the appearance of an antisense ORF encoding a full-length, functional product in pandemic group-M strains of HIV-1.

### Codon permutation test suggests a process of stabilization of the intact *asp* ORF

We carried out a first codon permutation test using 1,625 complete *env* sequences overlapping a full-length *asp* ORF and 339 complete *env* sequences overlapping a truncated *asp* ORF (all belonging to subtype B). After removing the gaps, we obtained 1,625 *env* sequences with a mean length of 190.9 codons (sd = 4.1) and 339 *env* sequences with a mean length of 191.0 codons (sd = 3.9). For each *env* sequence, we carried out 100 permutations in the order of synonymous codons such that the amino acid sequence of ENV did not change. After each round of 100 permutations, we counted the total number of new internal *stop* codons that appeared in the antisense *asp* ORF. We found that in the set of 1,625 *env* sequences, the mean number of *stops* in the antisense ORF for each round of 100 permutations (89.1; sd = 36.5) was significantly smaller than that in the 339 sequences (152.8; sd = 60.2; Student’s *t* = 18.78; *P* = 10^−5^).

We then carried out a similar test using sequences of subtype C: 968 *env* sequences overlapping a full-length *asp* ORF and 198 *env* sequences overlapping a truncated *asp*. After removing the gaps, we obtained 968 *env* sequences with a mean length of 186.7 codons (sd = 5.0) and 198 *env* sequences with a mean length of 187.4 codons (sd = 5.4). We found that in the set of 968 *env* sequences, the mean number of stops in the antisense ORF for each round of 100 permutations (85.8; sd = 35.8) was almost half of that in the 198 sequences (164.8; sd = 76.9; Student’s *t* = 14.15; *P* = 10^−5^).

Altogether, these results suggest a process of stabilization of the intact *asp* ORF, after its origin. It consisted of a change in the order of synonymous codons in *env* such that it minimized the likelihood of new premature stops arising in *asp*.

### Codon diversity in the ENV_FL-ASP_ data set is significantly lower than in the ENV_ΔASP_ data set

**Fig 4:**
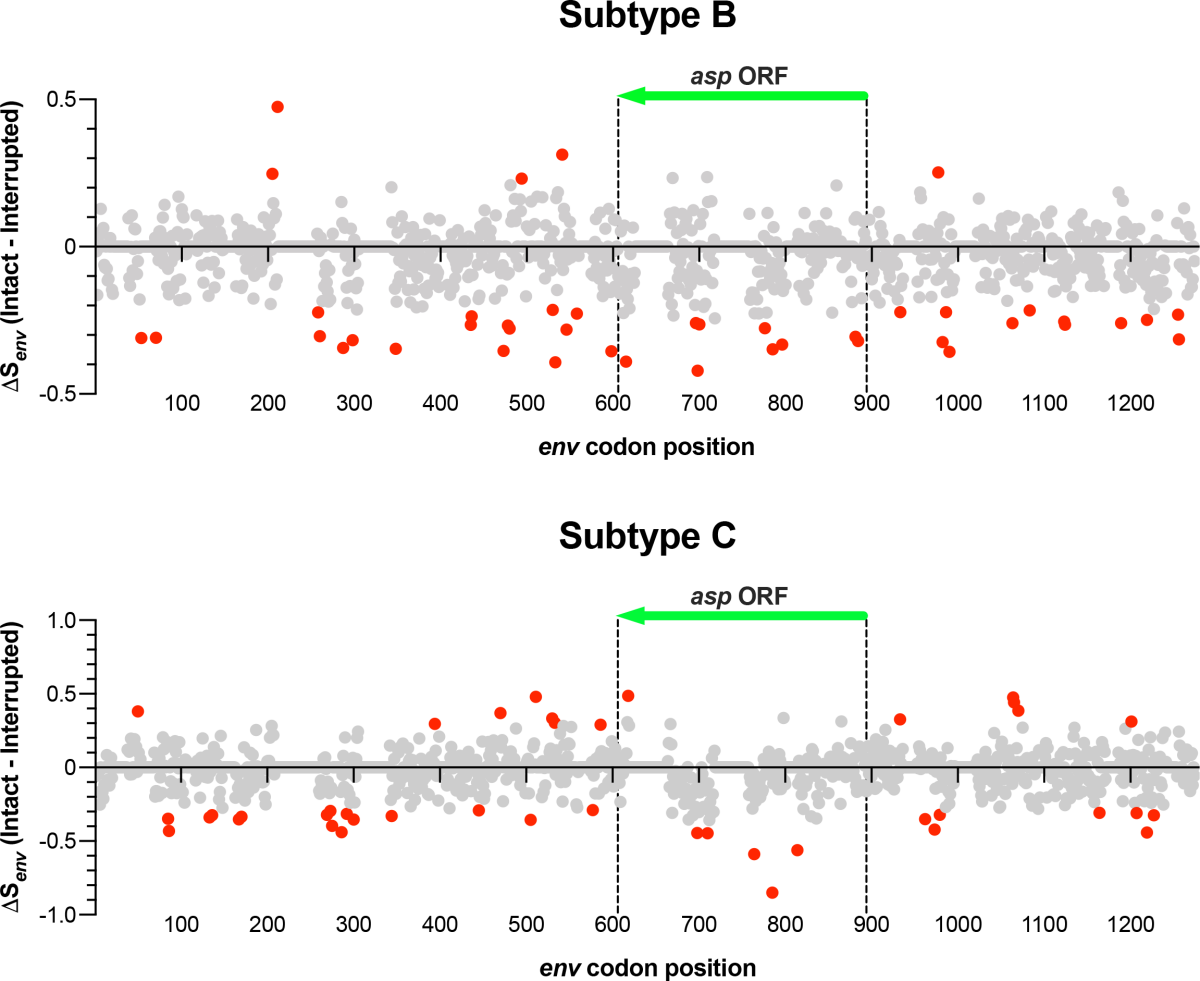
Difference in entropy at each codon position of *env* (ΔS*_env_*) between sequences overlapping an intact *asp* ORF (1,625 from subtype B and 968 from subtype C isolates) and sequences overlapping an interrupted *asp* ORF (339 from subtype B and 198 from subtype C isolates). The two vertical dashed lines comprise the region of overlap and the green arrow marks the orientation of the *asp* ORF. The red circles indicate positions with a ΔS*_env_* value significantly higher or lower than the mean value along the entire *env* sequence. Subtype B: inside the overlap, nine codon positions with ΔS*_env_* significantly lower than the mean value; outside the overlap, 29 codon positions with ΔS*_env_* significantly lower and five codon positions with ΔS*_env_* significantly higher than the mean value. Subtype C: inside the overlap, five codon positions with ΔS*_env_* significantly lower and one with ΔS*_env_* significantly higher than the mean value; outside the overlap, 23 codon positions with ΔS*_env_* significantly lower and 12 codon positions with ΔS*_env_* significantly higher than the mean value.

To evaluate the extent of codon diversity (number and abundance of codons) in the region of *env* comprising the overlap with the *asp* ORF (289 positions from 606 to 894 in the multiple alignments of *env* with 1,274 codon positions), we analyzed a data set of 1,964 *env* sequences from subtype B: 1,625 *env* sequences overlapping an intact *asp* ORF and 339 overlapping an interrupted *asp* ORF. We first removed the codon positions having a frequency of gap >5% (which shortened the alignment to 183 codon positions), and then we calculated the difference between the entropy in ENV_FL-ASP_ and ENV_ΔASP_ – ΔS*_env_*(Intact − Interrupted)—at each codon position using the Shannon entropy index (26). The mean difference was –0.05, significantly lower than 0 (Student’s *t* for paired data = 5.68; *P* = 10^−5^), indicating that ENV_FL-ASP_ has a codon diversity significantly smaller than ENV_ΔASP_. Using the zeta test (cutoff value = 1.96; *P* < 0.05), we detected nine codon positions in which ΔS*_env_*(Intact − Interrupted) is significantly lower than the −0.05 mean difference (Fig. 4A).

We also evaluated the extent of diversity in the number and abundance of synonymous codons in the region of *env* outside the antisense overlap (649 codon positions after the removal of codon positions with a frequency of gaps >5%). For each position, we calculated the difference between the entropy in the ENV_FL-ASP_ and ENV_ΔASP_ data sets. We then evaluated the significance of the difference using the Student’s *t* test for paired data. The mean difference was −0.04, significantly lower than 0 (Student’s *t* = 8.17; *P* = 10^−5^). Using the zeta test (cutoff value = 1.96; *P* < 0.05), we found that the *env* region outside the overlap has 29 codon positions where ΔS*_env_*(Intact − Interrupted) is significantly lower than the −0.04 mean difference (Fig. 4A).

A similar analysis was performed with 1,166 *env* sequences from subtype C: 968 sequences overlapping an intact *asp* ORF and 198 overlapping an interrupted *asp* ORF. We found a mean ΔS*_env_*(Intact − Interrupted) of −0.08, significantly lower than 0 (Student’s *t* for paired data = 5.67; *P* = 10^−5^), indicating that ENV_FL-ASP_ has a codon diversity significantly smaller than ENV_ΔASP_. Using the zeta test (cutoff value = 1.96; *P* < 0.05), we detected five codon positions in which ΔS*_env_*(Intact − Interrupted) is significantly lower than the −0.08 mean difference (Fig. 4B). In the region of *env* outside the antisense overlap (642 codon positions after the removal of codon positions with a frequency of gaps >5%), the mean ΔS*_env_*(Intact − Interrupted) was –0.023, significantly lower than 0 (Student’s *t =* 3.96; *P* = 10^−4^). Using the zeta test (cutoff value = 1.96; *P* < 0.05), we found that the *env* region outside the overlap contains 23 codon positions where ΔS*_env_*(Intact − Interrupted) is significantly lower than the −0.04 mean difference (Fig. 4B).

The smaller genetic diversity of *env* in ENV_FL-ASP_ compared to ENV_ΔASP_ suggests that maintenance of a full-length *asp* ORF likely affected the evolution of *env* both inside and outside the region of overlap. Thus, the presence of an intact *asp* ORF encoding a functional ASP protein affects the genetic evolution of the *env* gene and the functional evolution of the ENV protein. This also suggests that maintenance of an intact *asp* ORF likely provides a net selective advantage to the virus.

## DISCUSSION

Overprinting is a process through which point mutations within an existing or “ancestral” open reading frame led to the creation (“birth”) of a novel protein-coding gene in a different reading frame while preserving the expression of the ancestral one (1, 27). When ancestral and novel genes are encoded on the same DNA strand, they form a sense overlap, whereas when encoded on opposite DNA strands, they form an antisense overlap. Since ancestral and novel genes share the same nucleotide sequence, they constrain each other’s evolution. Unlike the ancestral one, the novel protein usually lacks remote homologs and is said to have been created *de novo* (28). The birth of novel genes by overprinting is driven by selection pressures, and it provides the virus with a fitness advantage that leads to their fixation in the population (19, 29, 30). Examples include a sense overlap in the plant *Tymoviridae*, where a novel overprinting gene is found in 20 different virus species despite a 30%–50% genomic sequence diversity (1). Similarly, the antisense overlapping gene *hbz* of HTLV-1 shows a very high degree of conservation (4, 31).

In contrast, the *env/asp* antisense overlap of HIV-1 investigated in this study is an evolutionary enigma. Although an antisense *asp* ORF of >150 amino acids is a unique feature of pandemic HIV-1 strains of group M, it is absent in ~25%–30% of virus isolates due to premature *stop* codons (16, 18). While *asp* ORFs of <150 codons are found in other HIV-1 groups (N, O, and P), the significantly lower combined global prevalence of these three groups compared to group M argues against the hypothesis that a shorter *asp* ORF may provide a more pronounced fitness advantage than a full-length *asp* ORF. At the same time, the existence of longer *asp* ORF with N-terminal extensions due to the emergence of upstream *start* codons is not possible or unlikely. Indeed, the closest alternative *start* codon is located 20 codon positions upstream of the canonical *start*. However, the presence of a *stop* codon between canonical and alternative *start* codons does not allow the expression of a protein with an N-terminal extension.

As previously hypothesized (16), the loss of a functional ASP protein does not compromise the viability of the virus, because *asp* is an accessory gene. Using the Luria-Delbruck fluctuation test, we found that the distribution of *stop* codons in the control data set of HIV-1 sequences from subtypes B and C (Table 2) does not support the view that the truncated *asp* descended from the intact *asp* through random genetic drift, which is in line with the finding reported in our previous study (18). Therefore, we hypothesize the opposite evolutionary process, with the truncated *asp* as the progenitor of the intact one. While the *asp* ORF is not necessary for efficient viral replication, we cannot exclude *a priori* the hypothesis that *asp* is something more than a dispensable accessory gene. Indeed, the presence of an intact *asp* exclusively in pandemic HIV-1 strains (group M) together with the strong selection pressure that we found in the *env* region overlapping *asp* (Fig. 2) suggests the possibility that *asp* may have played a role in transforming group-M HIV-1 strains into pandemic viruses.

Several endogenous and exogenous retroviruses express antisense RNAs even when these transcripts do not carry an open reading frame (32). In the case of HIV-1, antisense transcription is driven by a negative-sense promoter in the U3 region of the 3′ long terminal repeat (LTR) in a Tat-independent manner (33–35), and it has been confirmed by us and other groups (36) in multiple cell systems, including cells from infected individuals under suppressive antiretroviral therapy [for a review see reference (37)]. On the other hand, only indirect evidence in the form of humoral and cellular immune responses exists for the expression of the protein (11–15, 38). Whether HIV-1 strains with an interrupted *asp* ORF (i.e., containing early *stops*) are capable of expressing antisense transcripts and a truncated ASP protein remains to be determined. The evidence we present here and reported previously (18) suggests that the expression of a longer ASP protein (compared to a hypothetical truncated one) via progressive removal of premature *stops* may have led to acquiring additional functions and provided a selective advantage to the virus. Indeed, a significantly higher percentage of strains within group M (especially among high prevalence subtypes) contains a full-length *asp* ORF, which is completely absent in non-pandemic strains of groups N, O, and P.

An alternative hypothesis is that the creation of *asp* is the consequence of adaptation to specific host populations. However, this interpretation is not supported by the evidence that both high-prevalence subtypes and CRFs (i.e., A, B, C, G, CRF01_AE; total prevalence ~82%; on average, 85% isolates with an intact *asp*) and low-prevalence subtypes and CRFs (i.e., D, F, H, J, K, and CRF02_AG; total prevalence ~10%; on average, 30% isolates with intact *asp*) are not confined to specific geographic areas or populations. Rather, they show very diverse worldwide distributions and often circulate in the same geographic region and within the same populations; for instance, that is the case of subtypes C and D in East Africa or B and F in South America.

Through the identification of the codon positions that are the main determinants of the difference between *env* with intact *asp* and *env* with truncated *asp* (Table 1), we found that the loss of internal *stops* in *asp* is due to nucleotide substitutions yielding a conservative amino acid change in ENV (Fig. 3) or to synonymous nucleotide substitutions that do not change the amino acid sequence of ENV. This suggests that the creation of the *asp* ORF is not merely a “byproduct” of *env* evolution. An alternative hypothesis is that the maintenance of an intact *asp* ORF may be due to primary RNA sequence requirements to generate specific secondary structures in the viral RNA genome. However, secondary and tertiary RNA structures can be generated via alternative primary sequences. For example, several long noncoding RNAs (lncRNA) interact with the same chromatin remodeling enzymes (e.g., the polycomb repressor complex 2, PRC2) despite having highly divergent sequences (39). That is because PRC2 does not recognize specific nucleotide sequences but rather specific tertiary structures [G-quadruplexes (40)] that can be achieved via alternative primary sequences. Indeed, published (36) and unpublished studies from our lab have shown that the HIV-1 antisense ASP RNA interacts with PRC2 with affinity comparable to the host lncRNA HOTAIR, despite these two transcripts having very different nucleotide sequences. In light of that, the HIV-1 genomic RNA may not be limited to a specific primary sequence to fold into the same secondary and tertiary structure, but rather that could be achieved via different primary sequences that could also involve the presence of early *stops* in *asp* ORF. Yet, the frequency of early *stops* in *asp* is low. Furthermore, this hypothesis is also not in line with the evidence that viral strains with early *stops* in *asp* are also found in high-prevalence clades (16) without any substantial evidence of an impact on secondary or tertiary structures of the viral genomic RNA.

The view that the transition process from truncated to intact *asp* is driven by fine-tuning in the use of synonymous codons was also supported by the codon permutation test. It suggests a stabilization of the intact *asp* ORF after its birth through a change in the order of synonyms in *env* that minimized the likelihood of new premature *stops* arising in *asp*. Finally, this evolutionary process is also consistent with the fact that the “birth” of an intact *asp* ORF is a very recent event. Indeed, *asp* is unique to HIV-1 strains of group M (16), which diverged from SIVcpz around 100 years ago (41–43).

Using the Shannon entropy index (44), we found that the region of *env* overlapping intact *asp* ORF has a codon diversity significantly smaller than the one observed in *env* overlapping truncated *asp*. This is due to selection pressure on *env* to maintain a full-length *asp* ORF. More interestingly, we found that the selection pressure acting on *env* overlapping an intact *asp* extends to regions of *env* outside the overlap, thus reducing codon diversity along the entire *env* gene. Altogether, the presence of an intact *asp* ORF encoding a functional ASP protein significantly affects the diversity of the *env* gene and the functional evolution of the ENV protein. Lower entropy (thus greater conservation) of *env* overlapping an intact *asp* ORF both within and outside the region of overlap is readily apparent in strains of subtype B and—to lower extent—subtype C (Fig. 4). Therefore, the evolutionary constraints associated with maintenance of an intact *asp* ORF must be offset by a fitness advantage that HIV-1 derives from the expression of ASP. In line with this, co-evolution of the ASP and ENV proteins was proposed in a study showing that the diversity of ASP is associated with that of the V3 loop of ENV (mapping in a region of *env* outside the *asp* overlap), leading to hypothesize that ASP mutations are linked to viral tropism and co-receptor usage (45). Previous studies from our group and other groups demonstrated that ASP is present on the envelope surrounding HIV-1 viral particles, which lends support to the hypothesis that ASP may participate in the early steps of HIV-1 infection (8, 46).

It should be underscored that the fluctuation test, the permutation test, and the Shannon entropy analysis were carried out separately on sequences from viral isolates of subtypes B and C. This approach allowed us to avoid the “background noise” deriving from the extremely high number of genotypes within group M HIV-1. Moreover, subtypes B and C show two of the highest prevalence rates worldwide (16).

The finding that 16% of high prevalence group-M strains lack an intact *asp* ORF raises the hypothesis that the removal of internal *stops* in a pre-existing ORF to generate an intact one is a process still in progress. Therefore, the *env* sequence overlapping a truncated *asp* ORF could be viewed as a pre-antisense overlapping coding region. Pre-overlapping coding regions have been previously used as controls to investigate the birth of sense-overlapping genes in viruses (47). It is also possible that the introduction of antiretroviral therapy in clinical practice in the mid-1990s may have slowed down or stopped the transition from truncated to intact *asp* ORF. This is supported by absent or reduced intra-host viral evolution in ART-treated PLWH (48–52). However, the low number of sequences deposited in the Los Alamos Database, which were obtained from samples collected prior to 1983 (a total of 22, of which 0 in all years prior to 1976, 2 from 1976, 0 from 1977, 2 from 1978, 5 from 1979, 0 from 1980, 7 from 1981, and 6 from 1982), does not allow to draw statistically sound comparisons with sequences from more recent isolates. Moreover, the length of the available sequences from the earliest known 1959 isolate, ZR59 (53), that covers the *env* gene range from 152 to 197 bp, while the ones from another early 1960 isolate, DRC60 (54),are ~50 bp long. Altogether, sequences from the samples collected prior to 1983 do not allow us to determine whether early isolates already contained an intact *asp* gene.

Finally, we tested the hypothesis that the presence of an intact asp ORF in the pandemic HIV-1 group M provides a selective advantage during viral pathogenesis or spread. The finding of a trend toward increased frequency of intact *asp* ORF in individuals with faster disease progression (Fig. 1) supports the hypothesis that ASP promotes viral transmission or pathogenesis, which warrants further investigation. We chose not to include sequences from elite controllers (EC) in our study because these individuals harbor a very different reservoir than other chronically infected individuals (55, 56). The viral reservoir in EC is highly enriched in infectious proviruses integrated in non-genic regions of the host genome, characterized by dense heterochromatin where viral transcription is suppressed. Therefore, intra-host viral evolution in EC is significantly reduced compared to RP, SP, and LTNP.

In summary, this *in silico* study provides strong evidence in support of the notion that the *asp* ORF of HIV-1 is an actual protein-coding gene and that the product encoded by *asp* provided a fitness advantage to HIV-1 that may have facilitated its worldwide spread. Further biochemical studies are needed to identify and elucidate the mechanisms through which ASP supports HIV-1 transmission, infection, replication, and persistence.

## MATERIALS AND METHODS

### Sequence data sets from rapid progressors, slow progressors, and long-term non-progressors

The Los Alamos HIV Database (www.hiv.lanl.gov/content/index) contains comprehensive data on HIV-1 genetic sequences from patients with a variable rate of HIV-1 disease progression. In detail, the database reports a total of 4,266 sequences from 385 people living with HIV-1 (PLWH) classified as “rapid progressor” (progress to AIDS in <3 years from primary infection), 7,307 sequences from 390 PLWH classified as “slow progressor” (progress to AIDS in 7–10 years after primary infection), and 11,804 sequences from 745 PLWH classified as “long-term non-progressor” (progress to AIDS in >12 years after primary infection). From the Los Alamos Database (updated to September 2022), we extracted sequences containing the region of the *env* gene covering the entire *asp* ORF from canonical *start* to canonical *stop*. We then restricted this data set to a subset that contains only sequences derived from intact, replication-competent HIV-1 isolates. Therefore, we built a data set of viral and proviral genomes restricted to (i) RNA sequences from patient plasma samples, (ii) DNA sequences from *in vitro* viral outgrowth assays, and (iii) DNA sequences from PBMC samples of PLWH off antiretroviral therapy, which reflect recent infection events and are less likely to be associated with defective proviruses. Using the criteria described above, we obtained a data set containing 1,222 sequences from 32 RP, 1,015 from 76 SP, and 814 from 75 LTNP. For 14 of 32 RP, we found only one *env* sequence, and for the remaining 18 RP, we retrieved a number of *env* sequences ranging from 3 to 391 (File S1). For 30 of 76 SP, we found only one *env* sequence, and for the remaining 46 SP, we retrieved a number of *env* sequences ranging from 2 to 125 (File S2). Finally, for 30 out of 75 LTNP, we found only one *env* sequence and for the remaining 45 LTNP, we retrieved a number of *env* sequences ranging from 2 to 186 (File S3). The percentage of donors in each of the three groups of PLWH for whom only one sequence was available (43.8% for RP, 39.5% for SP, and 40% for LTNP) was not significantly different (RP vs SP, χ^2^ = 0.17; *P* = 0.68; RP vs LTNP, χ^2^ = 0.13; *P* = 0.72; SP vs LTNP, χ^2^ = 0.04; *P* = 0.95). For each donor in the three groups of PLWH, we determined the percentage of *env* sequences encompassing a complete antisense *asp* ORF that were either intact (no premature *stops*) or truncated (one or more premature *stops*). The percentage of sequences with interrupted *asp* ORF for each PLWH was then distributed in three ranks: <1%, 1%–99%, and >99%.

### Sample and control data sets of *env* sequences overlapping intact or interrupted *asp* ORF

We used two data sets from our previous study (18), which contain 3,725 and 1,376 *env* sequences, respectively. The first data set includes *env* sequences that overlap an intact *asp* ORF (presence of canonical *start* and *stop* codons, and absence of internal *stops*), whereas the second includes *env* sequences that overlap an *asp* interrupted by premature *stops* (presence of canonical *start* and *stop* codons and presence of one or more internal *stops*). For further details, see the supplementary material.

## Data Availability

The present study was conducted using sequences previously deposited in the National Center for Biotechnology Information (NCBI) database at the National Library of Medicine (https://www.ncbi.nlm.nih.gov/). From the NCBI database, we compiled six sequence data sets that are contained in Files S1 to S6 within the supplementary material. Each supplementary file contains all the NCBI accession numbers of the sequences included in each of the six data sets. The sequences included in three of the data sets (see Files S4 and S5) were also included in our previous study (18).

## SUPPLEMENTAL MATERIAL

Supplemental material spectrum.03802-23-s0001.pdf Supplemental information, Table S1, Figure S1, and Supplemental Files 1-6.

ASM does not own the copyrights to Supplemental Material that may be linked to, or accessed through, an article. The authors have granted ASM a non-exclusive, world-wide license to publish the Supplemental Material files. Please contact the corresponding author directly for reuse.
