# Supplementary material for: Creation of the HIV-1 antisense gene asp coincided with the emergence of the pandemic group M and is associated with faster disease progression
Source: Microbiol Spectr. 2024 Jan 17;12(2):e03802-23. doi: 10.1128/spectrum.03802-23 (PMC10846101; doi:10.1128/spectrum.03802-23)
Supplement: Supplemental material — Supplemental information, Table S1, Figure S1, and Supplemental Files 1-6. [file spectrum.03802-23-s0001.pdf]

Creation of the HIV-1 antisense gene *asp* coincided with the emergence of the pandemic group M and is associated with faster disease progression

Angelo Pavesi and Fabio Romerio

Supporting Information

Materials and Methods

Sequence datasets from rapid progressors, slow progressors, and long-term non-progressors. The Los Alamos HIV database (www.hiv.lanl.gov/content/index) contains comprehensive data on HIV-1 genetic sequences from patients with a variable rate of HIV-1 disease progression. In detail, the database reports a total of 4266 HIV-1 nucleotide sequences from 385 people living with HIV-1 (PLWH) classified as "rapid progressor" (RP; progress to AIDS in <3 years from primary infection), 7307 HIV-1 sequences from 390 PLWH classified as "slow progressor" (SP; progress to AIDS in 7-10 years after primary infection), and 11804 HIV-1 sequences from 745 PLWH classified as "long-term non-progressor" (LTNP; progress to AIDS in >12 years after primary infection). For each volunteer donor, the database contains a variable number of HIV-1 sequences ranging from 1 to 391 in RPs (mean = 38.2), from 1 to 125 in SPs (mean = 13.4) and from 1 to 186 in LTNPs (mean = 10.9). Some sequences cover the complete viral genome, but in the great majority of cases it includes a genomic region.

We carefully examined the sequence data of each donors, and we selected the sequences in which the *env* gene covers the entire genomic region containing the *asp* ORF from canonical *start* to canonical *stop* codon. To ensure that our dataset included only sequences from replication-competent genomes, we restricted the dataset to 1) sequences derived from plasma RNA; 2) sequences of genomic DNA from cells of healthy donor infected during viral outgrowth assay involving peripheral blood mononuclear cells (PBMC) of PLWH; and 3) sequences of genomic

DNA from cells of PLWH off antiretroviral therapy (likely to reflect recent infection events). For each PLWH donor, we counted the frequency of *env* sequences encompassing a complete anti-sense ORF (from canonical *start* to canonical *stop*) that were either intact (no premature *stops*) or truncated (one or more premature *stops*).

This selection yielded 1222 sequences from 32 RP, 1015 from 76 SP, and 814 from 75 LTNP. The NCBI accession numbers of the selected sequences are given in **Supplementary File 1** for RP, in **Supplementary File 2** for SP, and in **Supplementary File 3** for LTNP. For 14 of 32 RP (43.8%) only 1 *env* sequence was available, and for the remaining 18 RP (56.2%) we retrieved a number of *env* sequences ranging from 3 to 391 (**Supplementary File 1**). For 30 of 76 SP (39.5%), we found only 1 *env* sequence, and for the remaining 46 SP (60.5%) we retrieved a number of *env* sequences ranging from 2 to 125 (**Supplementary File 2**). Finally, for 30 of 75 LTNP (40%) we found only 1 *env* sequence and for the remaining 45 LTNP (60%) we found a number of *env* sequences ranging from 2 to 186 (**Supplementary File 3**). The percentage of donors in each group of PLWH for whom only one sequence was available (43.8% for RP, 39.5% for SP, and 40% for LTNP) were not significantly different (RP vs. SP, χ^2 = 0.171; P = 0.68, RP vs. LTNP, χ^2 = 0.130; P = 0.72, SP vs. LTNP, χ^2 = 0.004; P = 0.95). The hypothesis we tested was that there is a significantly higher frequency of intact *asp* ORFs in samples from RP compared to SP and LTNP, which would suggest a correlation between the presence of intact *asp* ORF and disease progression.

Sample and control datasets of *env* sequences overlapping intact or interrupted *asp* ORF. From our previous study (1), we recovered two datasets containing the entire *env* gene from strains of HIV-1 group M and Simian Immunodeficiency Virus (SIV) infecting chimpanzee (SIVcpz). The first dataset includes 3725 env/asp overlaps (Supplementary File S1 in (1)) where *env* comprises an "intact" *asp* ORF (>150 codons) delimited by *start* and *stop* codons at canonical

positions, and without premature stops (ENV_{FL-ASP} in this study and **Supplementary File 4**). All the sequences in this dataset belong to HIV-1 group M strains, except one from SIVcpz (acc. number DQ373063). The second dataset (Supplementary File S2 in (1)) was subdivided into two datasets. The first includes 1376 *env/asp* overlaps ($ENV_{\Delta ASP}$ in this study and **Supplementary File 5**), where *env* comprises a “truncated” *asp* ORF (<150 codons) delimited by *start* and *stop* codons at canonical position and comprising one or more premature *stop* codons ($ENV_{\Delta ASP}$). More specifically, this dataset includes 871, 390, 98, 11, and 6 *env* sequences overlapping an *asp* ORF with 1, 2, 3, 4, or 5 internal *stop* codons, respectively. All the sequences included in this dataset belong to strains from HIV-1 group M. The other dataset includes 377 *env/asp* overlaps from subtype A strains (**Supplementary File 6** in this study). Subtype A represents a special case, where the *asp* ORF contains *start* and *stop* codons at canonical positions, but it is interrupted by a premature *stop* codon. However, the creation of a new *start* codon downstream of the premature *stop* reopened the *asp* ORF that expresses a protein with an N-terminal deletion.

The mean length of all *env* sequences is 2574 nt (sd = 39 nt), while the multiple alignment covers 3822 nt positions (1274 codon positions) due to the presence of several gaps. The region of the alignment that overlaps the *asp* ORF (*env/asp*) is comprised between nt positions 1816 and 2682 of *env*. The nt positions 2679-2681 (CAT) and 1818–1820 (CTA) of *env* correspond, in the reverse frame, to the canonical *start* and *stop* codons of *asp* ORF, respectively. In the reference $HIV\text{-}1_{HXB2}$ genome (GenBank K03455) the region of *env* overlapping *asp* is comprised between nt 7371–7943, while in the reference *env* sequence between nt 1147–1715.

Statistical analyses. For our studies, we used the Shannon entropy index (2, 3), the Luria-Delbruck fluctuation test (4, 5), and the codon permutation test (6). Data were analyzed using the Mann-Whitney U test, the contingency table chi-square test, the T-Student test for paired data, and the Zeta test, as appropriate.

References

1. A. Pavesi, F. Romerio, Extending the Coding Potential of Viral Genomes with Overlapping Antisense ORFs: A Case for the De Novo Creation of the Gene Encoding the Antisense Protein ASP of HIV-1. *Viruses* **14** (2022).
2. G. P. Patil, C. Taillie, Diversity as a concept and its measurement. *J Am Statist Assoc* **77**, 548-563 (1982).
3. C. E. Shannon, W. Weaver, *The mathematic theory of communication* (University of Illinois Press, 1949).
4. S. E. Luria, M. Delbruck, Mutations of Bacteria from Virus Sensitivity to Virus Resistance. *Genetics* **28**, 491-511 (1943).
5. M. E. Jones, S. M. Thomas, A. Rogers, Luria-Delbruck fluctuation experiments: design and analysis. *Genetics* **136**, 1209-1216 (1994).
6. T. E. Schlub, J. P. Buchmann, E. C. Holmes, A Simple Method to Detect Candidate Overlapping Genes in Viruses Using Single Genome Sequences. *Molecular biology and evolution* **35**, 2572-2581 (2018).

Supplementary Table S1. Codon positions that are the main determinants of the difference between *env* overlapping an intact *asp* ORF ($ENV_{FL\text{-}ASP}$) and *env* overlapping an interrupted *asp* ORF ($ENV_{\Delta ASP}$).

Codon position in *env*	*Stop* codons in the *asp* ORF[1] number (%)	% frequency of TAN codons in $ENV_{FL\text{-}ASP}$ dataset	% frequency of TAN codons in $ENV_{\Delta ASP}$ dataset	Difference frequency of TAN codon between $ENV_{FL\text{-}ASP}$ dataset and $ENV_{\Delta ASP}$ dataset
715	168 (8.4%)	0.4	3.2	–2.8
757	126 (6.3%)	0.0	0.5	–0.5
785	109 (5.5%)	0.0	0.0	0.0
698	86 (4.3%)	0.0	1.2	–1.2
764	77 (3.9%)	0.1	0.8	–0.7
716	76 (3.8%)	0.0	0.0	0.0
694	59 (3.0%)	0.0	0.0	0.0
718	59 (3.0%)	0.0	0.4	–0.4
717	40 (2.0%)	0.0	0.0	–0.3
700	35 (1.8%)	0.0	0.4	–0.4
794	35 (1.8%)	0.0	1.2	–1.2
883*	465 (23.3%)	0.0	0.0	0.0
885*	55 (2.8%)	0.1	0.3	–0.2

[1] Data in parenthesis is the percent frequency of *stop* codons over a total of 1994 *stop* codons.

* For codon positions 883 and 885, the loss of a *stop* in *asp* and creation of a full-length ORF is due to a nucleotide substitution in the third base of the preceding codon of *env* (882 and 884, respectively). See main text for details.

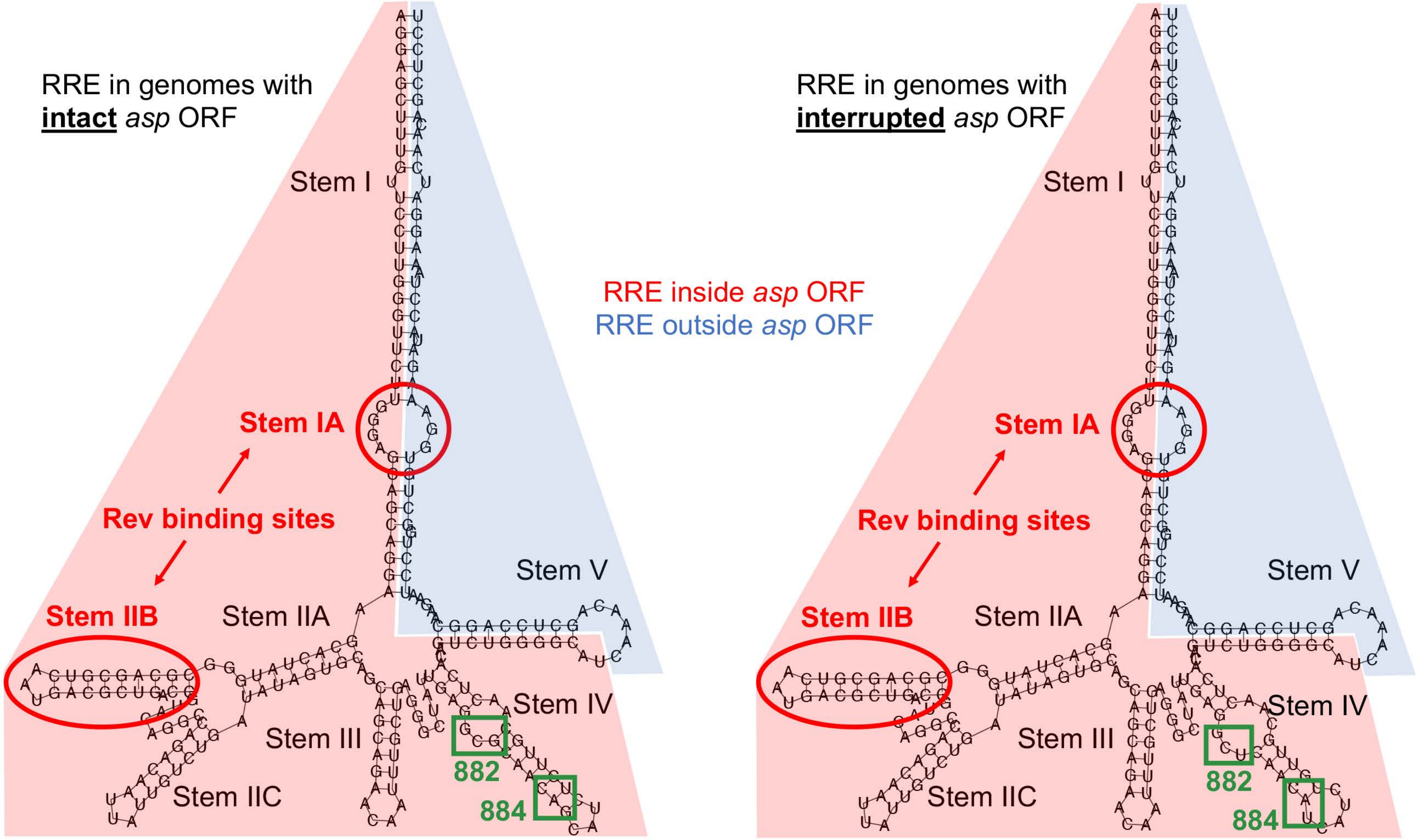

Figure S1. Two-dimensional structure of RRE from strains with an intact (left) or truncated *asp* ORF (right). The portion shaded in red maps within *asp*, and the portion shaded in blue maps outside *asp*. The location of Stems I, IA, IIA, IIB, IIC, III, IV and V is indicated. High- and low-affinity Rev binding sites (Stem IIB and IA, respectively) are encircled in red and indicated with red, bold letters. The *env* codons 882 and 884 in Stem IV are enclosed in green rectangles. Removal of *stops* in *asp* at these two positions involved G>T substitutions in third base of the two codons.

Supplementary File 1
Group of people living with HIV-1 and classified as rapid progressors (RP; progress to AIDS in <3 years after primary infection)

N. patient	*N. intact asp ORF*	*N. truncated asp ORF*	*% truncated asp ORF*
1	1	0	0.0
2	22	0	0.0
3	1	0	0.0
4	4	0	0.0
5	388	3	0.8
6	0	7	100.0
7	1	0	0.0
8	1	0	0.0
9	1	0	0.0
10	1	0	0.0
11	4	0	0.0
12	66	0	0.0
13	147	0	0.0
14	100	0	0.0
15	1	0	0.0
16	1	0	0.0
17	0	3	100.0
18	99	0	0.0
19	1	0	0.0
20	22	0	0.0
21	183	0	0.0
22	0	14	100.0
23	20	0	0.0
24	21	0	0.0
25	20	0	0.0
26	3	0	0.0
27	1	0	0.0
28	1	0	0.0
29	1	0	0.0
30	1	0	0.0
31	0	1	100.0
32	82	0	0.0

NCBI accession number of the HIV-1 sequences from each patient RP
Patient 1: 1 sequence
U43141
Patient 2: 22 sequences
AF544008
AY043173
AY529671
DQ411851
DQ904337
DQ904338
DQ904339
KF146938
KF146939

KF146940
KF146941
KF146942
KF146943
KF146944
KF146945
KF146946
KF146947
KF146948
KF146949
KF146950
KF146951
KF146952
Patient 3: 1 sequence
AY805330
Patient 4: 4 sequences
AJ535612
AJ535613
AJ535614
AJ535615
Patient 5: 391 sequences
AY357338
AY357340
AY357341
AY357342
AY357343
AY357344
AY357345
AY357347
AY357348
AY357349
AY357350
AY357352
AY357353
AY357354
AY357355
AY357356
AY357357
AY357358
AY357359
AY357361
AY357362
AY357365
AY357366
AY357367
AY357368
AY357369
AY357371
AY357373
AY357374
AY357376
AY357377

AY357378
AY357398
AY357399
AY357401
AY357403
AY357404
AY357405
AY357407
AY357408
AY357409
AY357410
AY357411
AY357413
AY357414
AY357415
AY357416
AY357417
AY357418
AY357419
AY357420
AY357422
AY357423
AY357424
AY357425
AY357427
AY357428
AY357429
AY357431
AY357435
AY357436
AY357438
AY357439
AY357440
AY357441
AY357444
AY357445
AY357446
AY357447
AY357448
AY357450
AY357451
AY357452
AY357453
AY357454
AY357455
AY357456
AY357457
AY357458
AY357459
AY357460
AY357461
AY357462

AY357464
AY357465
AY357466
AY357467
AY357468
AY357469
AY357471
AY357474
AY357475
AY357476
AY357477
AY357478
AY357479
AY357480
AY357481
AY357482
AY357483
AY357484
AY357485
AY357486
AY357487
AY357488
AY357489
AY357490
AY357491
AY357494
AY357495
AY357497
AY357498
AY357500
AY357501
AY357503
AY357504
AY357505
AY357506
AY357507
AY357508
AY357510
AY357511
AY357512
AY357515
AY357516
AY357517
AY357518
AY357519
AY357520
AY357521
AY357524
AY357526
AY357527
AY357528
AY357529

AY357530
AY357532
AY357533
AY357534
AY357535
AY357536
AY357537
AY357538
AY357540
AY357542
AY357543
AY357544
AY357545
AY357546
AY357550
AY357551
AY357552
AY357553
AY357554
AY357555
AY357557
AY357558
AY357559
AY357560
AY357562
AY357563
AY357564
AY357565
AY357566
AY357571
AY357572
AY357573
AY357575
AY357576
AY357580
EF367180
EF367181
EF367182
EF367183
EF367184
EF367185
EF367187
EF367188
EF367189
EF367190
EF367191
EF367192
EF367193
EF367194
EF367195
EF367196
EF367197

EF367198
EF367199
EF367200
EF367201
EF367202
EF367203
EF367205
EF367206
EF367207
JQ707955
JQ707956
JQ707957
JQ707958
JQ707959
JQ707960
JQ707961
JQ707962
JQ707963
JQ707964
JQ707965
JQ707966
JQ707967
JQ707968
JQ707969
JQ707970
JQ707971
JQ707972
JQ707973
JQ707974
JQ707975
JQ707976
JQ707977
JQ707978
JQ707979
JQ707980
JQ707981
JQ707982
JQ707983
JQ707984
JQ707985
JQ707986
JQ707987
JQ707988
JQ707989
JQ707990
JQ707991
JQ707992
JQ708005
JQ708006
JQ708007
JQ708010
JQ708011

JQ708012
JQ708013
JQ708014
JQ708015
JQ708016
JQ708018
JQ708019
JQ708021
JQ708022
JQ708024
JQ708025
JN176460
JN176461
JN176462
JN176463
JN176464
JN176465
JN176466
JN176467
JN176468
JN176469
JN176470
JN176471
JN176472
JN176473
JN176474
JN176475
JN176476
JN176477
JN176478
JN176479
JN176480
JN176481
JN176482
JN176483
JN176484
JN176485
JN176486
JN176487
JN176488
JN176489
JN176490
JN176491
JN176492
JN176493
JN176494
JN176495
JN176496
JN176497
JN176498
JN176499
JN176500

JN176501
JN176502
JN176503
JN176504
JN176505
JN176506
JN176507
JN176508
JN176509
JN176510
JN176511
JN176512
JN176513
JN176514
JN176515
JN176516
JN176517
JN176518
JN176519
JN176520
JN176521
JN176522
JN176523
JN176524
JN176525
JN176526
JN176527
JN176528
JN176529
JN176530
JN176531
JN176532
JN176533
JN176534
JN176535
JN176536
JN176537
JN176538
JN176539
JN176540
JN176541
JN176542
JN176543
JN176544
JN176545
JN176546
JN176547
JN176548
JN176549
JN176550
JN176551
JN176552

JN176553
JN176554
JN176555
JN176556
JN176557
JN176558
JN176559
JN176560
JN176561
JN176562
HQ591389
HQ591390
HQ591391
HQ591392
HQ591393
HQ591394
HQ591395
HQ591398
HQ591399
HQ591400
HQ591401
HQ591402
HQ591403
HQ591404
HQ591405
HQ591406
HQ591407
HQ591408
HQ591409
HQ591410
HQ591411
HQ591412
HQ591413
HQ591414
HQ591415
FJ713453
FJ713454
FJ713455
FJ713456
FJ713457
FJ713458
JQ924495
JQ924496
JQ924497
JQ924498
JQ924499
JQ924500
JQ924501
Patient 6: 7 sequences
DQ447271
AY529666
FJ846658

FJ846659
FJ846660
FJ846661
FJ846662
Patient 7: 1 sequence
DQ447266
Patient 8: 1 sequence
DQ447272
Patient 9: 1 sequence
DQ447270
Patient 10: 1 sequence
DQ447269
Patient 11: 4 sequences
AY608576
AY608577
GQ994936
GQ994937
Patient 12: 66 sequences
EF203976
EF203977
FJ443492
HQ625596
HQ625597
GQ999972
FJ443222
FJ443223
FJ443224
FJ443225
FJ443226
FJ443227
FJ443228
FJ443229
FJ443230
FJ443231
FJ443232
FJ443233
FJ443234
FJ443235
FJ443236
FJ443237
FJ443238
FJ443239
KC863554
KC863555
KC863556
KC863557
KC863558
KC863559
KC863560
KC863561
KC863562
KC863563

KC863564
KC863565
KC863566
KC863567
KC863568
KC863571
MK205683
MK205693
MK205704
MK205715
MK205718
MK205729
MK205740
MK205751
MK205761
MK205771
MK205780
MK205790
MK205801
MK205825
MK205827
MK205996
MK206007
MK206025
MK206026
MK206035
MK206046
MK206057
MK206064
MK206065
MK206066
MK206067
Patient 13: 147 sequences
EF203965
EF203966
EF203967
FJ443356
FJ443357
FJ443358
FJ443359
FJ443360
FJ443361
FJ443362
FJ443363
FJ443364
FJ443365
FJ443366
KC863376
KC863377
KC863378
KC863379
KC863380

KC863381
KC863382
KC863383
KC863384
KC863385
KC863386
KC863387
KC863388
KC863389
KC863390
KC863391
KC863392
FJ443501
FJ443502
FJ443503
FJ443504
FJ443505
FJ443506
FJ443507
FJ443508
MK205828
MK205830
MK205831
MK205832
MK205833
MK205834
MK205835
MK205836
MK205837
MK205838
MK205839
MK205840
MK205841
MK205842
MK205843
MK205844
MK205845
MK205846
MK205847
MK205848
MK205849
MK205850
MK205851
MK205852
MK205853
MK205854
MK205855
MK205856
MK205857
MK205858
MK205859
MK205860

MK205861
MK205862
MK205863
MK205864
MK205865
MK205866
MK205867
MK205868
MK205869
MK205870
MK205871
MK205872
MK205873
MK205874
MK205875
MK205876
MK205877
MK205878
MK205879
MK205880
MK205881
MK205882
MK205883
MK205884
MK205885
MK205886
MK205887
MK205889
MK205890
MK205891
MK205892
MK205893
MK205894
MK205895
MK205896
MK205897
MK205898
MK205899
MK205900
MK205901
MK205902
MK205903
MK205904
MK205905
MK205906
MK205907
MK205908
MK205909
MN097563
MN097564
MN097565
MN097566

MN097567
MN097568
MN097569
MN097570
MN097571
MN097572
MN097573
MN097574
MN097575
MN097576
MN097577
MN097578
MN097579
MN097580
MN097581
MN097582
MN097583
HQ625598
HQ625599
KJ700457
FJ443519
FJ444632
GQ999982
MK205824
Patient 14: 100 sequences
DQ435683
FJ443509
GQ999983
KC894136
EF203987
EF203988
EF203989
FJ443186
FJ443187
FJ443188
FJ443189
FJ443190
FJ443191
FJ443192
FJ443193
FJ443195
FJ443308
FJ443309
FJ443310
FJ443311
FJ443312
FJ443313
FJ443314
FJ443315
KC863271
KC863272
KC863273

KC863274
KC863275
KC863276
KC863277
KC863278
KC863279
KC863280
KC863281
KC863282
KC863283
KC863284
KC863285
KC863286
KC863287
JX976632
JX976633
JX976634
JX976635
JX976636
JX976637
JX976638
JX976639
JX976640
JX976641
JX976642
JX976643
JX976644
JX976646
JX976647
JX976648
JX976649
JX976650
JX976651
JX976652
JX976653
JX976654
JX976655
JX976656
JX976657
JX976658
JX976659
JX976660
JX976661
JX976662
JX976663
JX976664
JX976665
JX976666
JX976667
JX976668
JX976669
OK500140

OK500141
OK500142
OK500143
OK500144
OK500145
OK500146
OK500147
OK500148
OK500149
OK500150
OK500151
OK500152
OK500153
OK500154
OK500155
OK500156
OK500157
OK500158
OK500159
OK500160
OK500161
Patient 15: 1 sequence
EF531328
Patient 16: 1 sequence
EF531337
Patient 17: 3 sequences
MH460464
MK086126
MK086128
Patient 18: 99 sequences
EF203973
EF203974
EF203975
GQ999976
FJ443215
FJ443216
FJ443218
FJ443219
FJ443220
FJ443396
FJ443397
FJ443398
FJ443399
FJ443400
FJ443401
FJ443402
FJ443403
FJ443404
FJ443405
FJ443406
FJ443407
FJ443408

MN635324
MN635325
MN635326
MN635327
MN635328
MN635329
MN635330
MN635331
MN635332
MN635333
MN635334
MN635335
MN635336
MN635337
MN635338
MN635339
MN635340
MN635341
MN635342
MN635343
MN635344
MN635345
MN635346
MN635347
MN635348
MN635349
MN635350
MN635351
MN635352
MN635353
MN635354
MN635355
MN635356
MN635357
MN635358
MN635359
MN635360
MN635361
MN635362
MN635363
MN635364
MN635365
MN635366
MN635367
MN635368
MN635369
MN635370
MN635371
MN635372
MN635373
MN635374
MN635375

MN635376
MN635377
MN635378
MN635379
MN635380
MN635381
MN635382
MN635383
MN635384
MN635385
MN635386
MN635387
MN635388
MN635389
MN635390
MN635391
MN635392
MN635393
MN635394
MN635395
MN635396
MN635397
MN635398
MN635399
MN635400
Patient 19: 1 sequence
JN681227
Patient 20: 22 sequences
FJ443253
FJ443254
FJ443255
FJ443256
FJ443257
FJ443258
FJ443259
FJ443260
FJ443261
FJ443262
FJ443263
FJ443264
FJ443265
FJ443266
FJ443267
FJ443269
FJ443270
FJ443271
FJ443272
FJ443273
GQ999981
JN967791
Patient 21: 183 sequences
EF203980

EF203981
GQ999989
HQ625604
KF241776
KC863461
KC863462
KC863463
KC863464
KF996576
KF996577
KF996578
KF996579
KF996580
KF996581
KF996582
KF996583
KF996584
KF996585
KF996586
KF996587
KF996588
KF996589
KF996590
KF996591
KF996592
KF996593
KF996594
KF996595
KF996596
KF996598
KF996599
KF996600
KF996601
KF996603
KF996604
KF996605
KF996606
KF996607
KF996608
KF996609
KF996610
KF996611
KF996612
KF996613
KF996614
KF996615
KF996616
KF996617
KF996618
KF996619
KF996620
KF996621

KF996622
KF996623
KF996624
KF996625
KF996626
KF996627
KF996628
KF996629
KF996630
KF996632
KF996633
KF996634
KF996635
KF996636
KF996637
KF996638
KF996639
KF996640
KF996641
KF996642
KF996643
KF996644
KF996645
KF996646
KF996647
KF996648
KF996649
KF996650
KF996651
KF996652
KF996653
KF996654
KF996655
KF996656
KF996657
KF996658
KF996659
KF996660
KF996661
KF996662
KF996663
KF996664
KF996665
KF996666
KF996667
KF996668
KF996669
KF996670
KF996671
KF996672
KF996673
KF996674

KF996675
KF996676
KF996677
KF996678
KF996679
KF996680
KF996681
KF996682
KF996683
KF996684
KF996685
KF996686
KF996687
KF996688
KF996689
KF996690
KF996691
KF996692
KF996693
KF996694
KF996695
KF996696
KF996697
KF996698
KF996699
KF996700
KF996701
KF996702
KF996703
KF996704
KF996705
KF996706
KF996707
KF996708
KF996709
KF996710
KF996711
KF996712
KF996713
KF996714
KF996715
KF996716
KT698223
KT698224
KT698225
KT698226
KT698227
MF572809
MF572810
MF572811
MF572812
MF572813

MF572814
MF572815
MF572816
MF572817
MF572818
MF572819
MF572820
MF572821
MF572822
MF572823
MF572824
MF572825
MF572826
MF572827
MF572828
MF572829
MK205498
MK205499
MK205500
MK205501
MK205502
MK205503
MK205504
MK205505
MK205506
MK205507
Patient 22: 14 sequences
GQ999977
KC863515
KC863516
KC863517
KC863518
KC863519
KC863520
KC863521
KC863522
KC863523
KC863524
KC863525
KC863526
KC863527
Patient 23: 20 sequences
FJ443177
FJ443178
FJ443179
FJ443180
FJ443181
FJ443182
FJ443184
FJ443185
FJ443298
FJ443299

FJ443300
FJ443302
FJ443303
FJ443304
FJ443305
FJ443306
FJ443494
FJ443495
JN967795
JX131327
Patient 24: 21 sequences
FJ443159
FJ443160
FJ443161
FJ443162
FJ443163
FJ443164
FJ443165
FJ443418
FJ443419
FJ443420
FJ443421
FJ443422
FJ443423
FJ443424
FJ443425
FJ443426
FJ443427
FJ443428
FJ443429
FJ443493
JN681230
Patient 25: 20 sequences
FJ443455
FJ443456
FJ443457
FJ443458
FJ443459
FJ443460
FJ443461
FJ443462
FJ443463
FJ443464
FJ443465
FJ443466
FJ443467
FJ443468
FJ443469
FJ443470
FJ443471
FJ443472
FJ443473

JN967793
Patient 26: 3 sequences
KF990605
KF990606
KF990607
Patient 27: 1 sequence
KT200352
Patient 28: 1 sequence
KT200356
Patient 29: 1 sequence
KT200355
Patient 30: 1 sequence
KT200354
Patient 31: 1 sequence
KT200357
Patient 32: 82 sequences
MT793911
MT793912
MT793913
MT793914
MT793915
MT793916
MT793917
MT793918
MT793919
MT793920
MT793921
MT793922
MT793923
MT793924
MT793925
MT793926
MT793927
MT793928
MT793929
MT793930
MT793931
MT793932
MT793933
MT793934
MT793935
MT793936
MT793937
MT793938
MT793939
MT793940
MT793941
MT793942
MT793943
MT793944
MT793945
MT793946

MT793947
MT793948
MT793949
MT793950
MT793951
MT793952
MT793953
MT793954
MT793955
MT793956
MT793957
MT793958
MT793959
MT793960
MT793961
MT793962
MT793963
MT793964
MT793965
MT793966
MT793967
MT793969
MT793971
MT793972
MT793973
MT793975
MT793978
MT793980
MT793981
MT793982
MT793983
MT793984
MT793985
MT793986
MT793988
MT793989
MT793991
MT793993
MT793996
MT793997
MT793998
MT793999
MT794000
MT794001
MT794002
MT794003

Supplementary File 2
Group of people living with HIV-1 and classified as slow progressors (SP; progress to AIDS in 7-10 years after primary infection)

N. patient	*N. intact asp ORF*	*N. truncated asp ORF*	*% truncated asp ORF*
1	4	0	0.0
2	4	1	20.0
3	7	0	0.0
4	5	0	0.0
5	10	1	9.1
6	1	0	0.0
7	1	0	0.0
8	1	0	0.0
9	0	1	100.0
10	4	0	0.0
11	1	0	0.0
12	15	0	0.0
13	2	0	0.0
14	2	0	0.0
15	2	0	0.0
16	1	0	0.0
17	1	0	0.0
18	6	0	0.0
19	2	1	33.3
20	2	0	0.0
21	0	1	100.0
22	1	0	0.0
23	0	1	100.0
24	25	0	0.0
25	2	0	0.0
26	2	0	0.0
27	99	0	0.0
28	0	3	100.0
29	3	0	0.0
30	100	0	0.0
31	21	0	0.0
32	19	0	0.0
33	0	18	100.0
34	22	0	0.0
35	23	0	0.0
36	1	0	0.0
37	1	0	0.0
38	0	1	100.0
39	1	0	0.0
40	0	4	100.0
41	1	0	0.0
42	3	0	0.0
43	1	0	0.0
44	1	0	0.0
45	0	1	100.0
46	1	0	0.0

47	0	2	100.0
48	0	1	100.0
49	1	0	0.0
50	11	0	0.0
51	11	0	0.0
52	53	5	8.6
53	9	0	0.0
54	88	3	3.3
55	20	60	75.0
56	37	0	0.0
57	56	10	15.1
58	0	1	100.0
59	1	0	0.0
60	1	0	0.0
61	1	0	0.0
62	12	0	0.0
63	125	0	0.0
64	7	0	0.0
65	20	0	0.0
66	9	0	0.0
67	6	0	0.0
68	2	0	0.0
69	1	0	0.0
70	0	1	100.0
71	2	0	0.0
72	0	1	100.0
73	1	0	0.0
74	1	0	0.0
75	12	0	0.0
76	13	0	0.0

NCBI accession number of the HIV-1 sequences from each patient SP

Patient 1: 4 sequences
AJ535616
AJ535617
AJ535618
AJ535619
Patient 2: 5 sequences
DQ295192
KY820495
OK075244
OK514753
OK514755
Patient 3: 7 sequences
JQ316130
KU869598
KU869599
MN043596
OK075241
OK075242
OK075243
Patient 8: 5 sequences

DQ295196
KX960979
MN043580
OK075270
OK075271
Patient 5: 11 sequences
DQ339425
JQ316133
KJ140253
KU869549
KU869620
KU896072
MN043599
MN043600
MT582421
MW405271
MW405272
Patient 6: 1 sequence
AF538304
Patient 7: 1 sequence
AF538305
Patient 8: 1 sequence
AF538306
Patient 9: 1 sequence
AF538307
Patient 10: 4 sequence
AY624304
AY624305
AY624306
AY624307
Patient 11: 1 sequence
AY751406
Patient 12: 15 sequence
AY901966
GU216748
GU216749
GU216750
GU216751
GU216752
GU216753
GU216754
GU216755
GU216756
GU216757
GU216758
GU216759
GU216760
GU216761
Patient 13: 2 sequences
AY522733
EU293448
Patient 14: 2 sequence

AY522734
EU293449
Patient 15: 2 sequences
AY522735
EU293445
Patient 16: 1 sequence
AY529678
Patient 17: 1 sequence
AY529676
Patient 18: 6 sequence
AY529676
FJ846653
FJ846654
FJ846655
FJ846656
FJ846657
Patient 19: 2 sequences
DQ295195
MN043581
OK514756
Patient 20: 2 sequence
DQ295193
OK075236
Patient 21: 1 sequence
AY529679
Patient 22: 1 sequence
DQ447268
Patient 23: 1 sequence
DQ447267
Patient 24: 25 sequences
HQ143536
HQ143537
HQ143538
HQ143539
HQ143540
HQ143541
HQ143542
HQ143543
HQ143544
HQ143545
HQ143546
HQ143547
HQ143548
HQ143549
HQ143550
HQ143551
HQ143552
HQ143553
HQ143554

HQ143555
HQ143556
HQ143557
HQ143558
HQ143559
HQ143560
Patient 25: 2 sequences
AY522726
EU293446
Patient 26: 2 sequences
AY522731
EU293447
Patient 27: 99 sequences
DQ435682
GQ999974
OK500139
EF203960
EF203961
EF203962
FJ443149
FJ443150
FJ443151
FJ443152
FJ443153
FJ443154
FJ443155
FJ443156
FJ443157
FJ443158
FJ443292
FJ443293
FJ443294
FJ443295
FJ443296
FJ443297
JX976698
JX976699
JX976700
JX976701
JX976702
JX976703
JX976704
JX976705
JX976706
JX976707
JX976708
JX976709
JX976710
JX976711
JX976712
JX976713
JX976714

JX976715
JX976716
JX976717
JX976718
JX976719
JX976720
JX976722
JX976723
JX976724
JX976725
JX976726
JX976728
JX976729
JX976730
KC863476
KC863477
KC863478
KC863479
KC863480
KC863481
KC863482
KC863483
KC863484
KC863485
KC863486
KC863487
KC863488
KC863489
KC863490
KC863491
KC863492
KC863493
KC863494
KC863495
KC863496
KC863497
MK205656
MK205657
MK205658
MK205659
MK205660
MK205661
MK205662
MK205663
MK205664
MK205665
MK205666
MK205667
MK205668
MK205669
MK205670
MK205671

MK205672
MK205673
MK205674
MK205675
MK205676
MK205677
MK205678
MK205679
Patient 28: 3 sequences
MH460467
MH460468
MH460469
Patient 29: 3 sequences
EU161643
EU161644
EU161645
Patient 30: 100 sequence
EF203968
EF203969
GQ999984
MT260181
MT260182
MT260183
MT260184
MK205450
MK205451
MK205452
MK205453
MK205454
MK205455
MK205456
MK205457
MK205458
MK205459
MK205460
MK205461
MK205462
MK205463
MK205464
MK205465
MK205466
MK205467
MK205468
MK205469
MK205470
MK205471
MK205472
MK205473
MK205474
MK205476
MK205477
MK205478

MK205479
MK205480
MK205481
MK205482
MK205483
MK205484
MK205485
MK206155
MK206156
MK206158
MK206159
MK206160
MK206161
MK206162
MK206163
MK206164
MK206165
MK206166
MK206167
MK206168
MK206169
MK206170
MK206171
MK206172
MK206173
MK206174
MK206175
MK206176
MK206177
MK206178
MK206179
MK206180
MK206181
MK206182
MK206183
MK206184
MK206185
MK206186
MK206187
MK206188
MK206189
MK206190
MK206191
MK206192
MK206193
MK206194
MK206195
MK206196
MK206197
MK206198
MK206199
MK206200

MK206201
MK206202
MK206203
MK206204
MK206205
MK206206
MK206207
MK206208
MK206209
MK206210
MK206211
MK206212
MK206213
Patient 31: 21 sequences
KC863498
KC863499
KC863500
KC863501
KC863502
KC863503
KC863504
KC863505
KC863506
KC863507
KC863508
KC863509
KC863510
KC863511
KC863512
KC863513
KC863514
GQ999975
EF203957
EF203958
EF203959
Patient 32: 19 sequences
FJ443210
FJ443211
FJ443212
FJ443214
FJ443430
FJ443431
FJ443432
FJ443433
FJ443434
FJ443435
FJ443436
FJ443437
FJ443438
FJ443439
FJ443440
FJ443441

FJ443442
FJ443514
FJ443515
Patient 33: 18 sequences
FJ443906
FJ443907
FJ443908
FJ443909
FJ443910
FJ443911
FJ443912
FJ443913
FJ443914
FJ443915
FJ443916
FJ443917
FJ443918
FJ443919
FJ443920
FJ443921
FJ443922
JN967794
Patient 34: 22 sequences
DQ396381
GU216762
GU216763
GU216764
GU216765
GU216766
GU216767
GU216768
GU216769
GU216770
GU216771
GU216772
GU216773
GU216774
GU216775
GU216776
GU216777
GU216778
GU216779
GU216780
GU216781
GU216782
Patient 35: 23 sequences
GU216724
GU216725
GU216726
GU216727
GU216728
GU216729

GU216730
GU216731
GU216732
GU216733
GU216734
GU216735
GU216736
GU216737
GU216739
GU216740
GU216741
GU216742
GU216743
GU216744
GU216745
GU216746
GU216747
Patient 36: 1 sequence
Eu293450
Patient 37: 1 sequence
GU367406
Patient 38: 1 sequence
GU367403
Patient 39: 1 sequence
GU367407
Patient 40: 4 sequences
GU367399
GU367400
GU367401
GU367402
Patient 41: 1 sequence
GU367409
Patient 42: 3 sequences
GU367395
GU367396
GU367397
Patient 43: 1 sequence
GU367408
Patient 44: 1 sequence
GU367398
Patient 45: 1 sequence
GU367412
Patient 46: 1 sequence
GU367405
Patient 47: 2 sequences
GU367410
GU367411
Patient 48: 1 sequence
GU367404
Patient 49: 1 sequence
KJ941152
Patient 50: 11 sequences

FJ515874
FJ515875
FJ515876
FJ515877
FJ515878
FJ515879
JN400538
JN400539
JN400540
JN400542
JN400545
Patient 51: 11 sequences
GU216837
GU216838
GU216839
GU216840
GU216841
GU216842
GU216843
GU216844
GU216845
GU216846
GU216847
Patient 52: 58 sequences
JN251812
JN251813
JN251814
JN251815
JN251816
JN251819
JN251820
JN251821
JN251822
JN251823
JN251824
JN251825
JN251826
JN251827
JN251829
JN251830
JN251831
JN251832
JN251833.
JN251834
JN251836
JN251837
JN251841
JN251842
JN251844
JN251845
JN251846
JN251847

JN251848
JN251849
JN251850
JN251851
JN251853
JN251854
JN251856
JN251857
JN251858
JN251859
JN251860
JN251861
JN251863
JN251864
JN251865
JN251866
JN251867
JN251868
JN251869
JN251871
JN251872
JN251873
JN251879
JN251880
JN251881
JN251882
JN251883
JN251885
JN251886
JN251888
Patient 53: 9 sequences
JN400529
JN400530
JN400531
JN400532
JN400533
JN400534
JN400535
JN400536
JN400537
Patient 54: 91 sequences
JQ609870
JQ609871
JQ609872
JQ609873
JQ609874
JQ609875
JQ609876
JQ609877
JQ609878
JQ609879
JQ609880

JQ609881
JQ609882
JQ609883
JQ609884
JQ609885
JQ609886
JQ609887
JQ609888
JQ609889
JQ609890
JQ609891
JQ609892
JQ609893
JQ609894
JQ609895
JQ609896
JQ609897
JQ609898
JQ609899
JQ609900
JQ609901
JQ609902
JQ609903
JQ609904
JQ609905
JQ609906
JQ609907
JQ609908
JQ609909
JQ609910
JQ609911
JQ609912
JQ609913
JQ609914
JQ609915
JQ609916
JQ609917
JQ609918
JQ609919
JQ609920
JQ609921
JQ609922
JQ609923
JQ609924
JQ609925
JQ609926
JQ609927
JQ609928
JQ609929
JQ609930
JQ609931
JQ609932

JQ609933
JQ609934.
JQ609935
JQ609936
JQ609937
JQ609938
JQ609939
JQ609940
JQ609941
JQ609942
JQ609943
JQ609944
JQ609945
JQ609946
JQ609947
JQ609948
JQ609949
JQ609950
JQ609951
JQ609952
JQ609953
JQ609954
JQ609955
JQ609956
JQ609957
JQ609958
JQ609959
JQ609960
Patient 55: 80 sequences
JQ609973
JQ609974
JQ609975
JQ609976
JQ609977
JQ609978
JQ609979
JQ609980
JQ609981
JQ609982
JQ609983
JQ609984
JQ609985
JQ609986
JQ609987
JQ609988
JQ609989
JQ609990
JQ609991
JQ609992
JQ609993
JQ609994
JQ609995

JQ609996
JQ609997
JQ609998
JQ609999
JQ610000
JQ610001
JQ610002
JQ610003
JQ610004
JQ610005
JQ610006
JQ610007
JQ610008
JQ610009
JQ610010
JQ610011
JQ610012
JQ610013
JQ610014
JQ610015
JQ610016
JQ610017
JQ610018
JQ610019
JQ610020
JQ610021
JQ610022
JQ610023
JQ610024
JQ610025
JQ610026
JQ610027
JQ610028
JQ610029
JQ610030
JQ610031
JQ610032
JQ610033
JQ610034
JQ610035
JQ610036
JQ610037
JQ610038
JQ610039
JQ610040
JQ610041
JQ610042
JQ610043
JQ610044
JQ610045
JQ610046
JQ610047

JQ610048
JQ610049
JQ610050
JQ610051
JQ610052
Patient 56: 37 sequences
JQ610120
JQ610121
JQ610122
JQ610123
JQ610124
JQ610125
JQ610126
JQ610127
JQ610128
JQ610129
JQ610130
JQ610131
JQ610132
JQ610133
JQ610134
JQ610135
JQ610136
JQ610137
JQ610138
JQ610139
JQ610140
JQ610141
JQ610142
JQ610143
JQ610144
JQ610145
JQ610146
JQ610147
JQ610148
JQ610149
JQ610150
JQ610151
JQ610152
JQ610153
JQ610154
JQ610155
JQ610156
Patient 57: 66 sequences
JQ610053
JQ610054
JQ610055
JQ610056
JQ610057
JQ610058
JQ610059
JQ610060

JQ610061
JQ610062
JQ610063
JQ610064
JQ610065
JQ610066
JQ610067
JQ610068
JQ610069
JQ610070
JQ610071
JQ610072
JQ610073
JQ610074
JQ610075
JQ610076
JQ610077
JQ610078
JQ610079
JQ610080
JQ610081
JQ610082
JQ610083
JQ610084
JQ610085
JQ610086
JQ610087
JQ610088
JQ610089
JQ610090
JQ610091
JQ610092
JQ610093
JQ610094
JQ610095
JQ610096
JQ610097
JQ610098
JQ610099
JQ610100
JQ610101
JQ610102
JQ610103
JQ610104
JQ610105
JQ610107
JQ610108
JQ610109
JQ610110
JQ610111
JQ610112
JQ610113

JQ610114
JQ610115
JQ610116
JQ610117
JQ610118
JQ610119
Patient 58: 1 sequence
KF384814
Patient 59: 1 sequence
KF384813
Patient 60: 1 sequence
KF384812
Patient 61: 1 sequence
KJ948660
Patient 62: 12 sequences
KM516886
KM516887
KM516888
KM516889
KM516890
KM516891
KM516892
KM516893
KM516894
KM516895
KM516896
KM516897
Patient 63: 125 sequences
MF591581
MF591582
MF591583
MF591584
MF591585
MF591586
MF591587
MF591588
MF591589
MF591590
MF591591
MF591592
MF591593
MF591594
MF591595
MF591596
MF591597
MF591598
MF591599
MF591600
MF591601
MF591602
MF591603
MF591604

MF591605
MF591606
MF591607
MF591608
MF591609
MF591610
MF591611
MF591612
MF591613
MF591614
MF591615
MF591616
MF591617
MF591618
MF591619
MF591620
MF591621
MF591622
MF591623
MF591624
MF591625
MF591626
MF591627
MF591628
MF591629
MF591631
MF591633
MF591634
MF591635
MF591636
MF591637
MF591638
MF591639
MF591640
MF591641
MF591642
MF591643
MF591644
MF591645
MF591646
MF591647
MF591648
MF591649
MF591650
MF591651
MF591652
MF591653
MF591654
MF591655
MF591656
MF591657
MF591658

MF591659
MF591660
MF591661
MF591662
MF591663
MF591664
MF591665
MF591666
MF591667
MF591668
MF591669
MF591670
MF591671
MF591672
MF591673
MF591674
MF591675
MF591676
MF591677
MF591678
MF591679
MF591680
MF591681
MF591682
MF591683
MF591684
MF591685
MF591686
MF591687
MF591688
MF591689
MF591690
MF591691
MF591692
MF591693
MF591694
MF591695
MF591696
MF591697
MF591698
MF591699
MF591700
MT436178
MT436179
MT436180
MT436181
MT436182
MT436183
MT436184
Patient 64: 7 sequences
MT033672
MT033673

MT033675
MT033686
MT033688
MT033699
MT033701
Patient 65: 20 sequences
MT795076
MT795077
MT795078
MT795079
MT795080
MT795081
MT795082
MT795083
MT795084
MT795085
MT795086
MT795087
MT795088
MT795089
MT795090
MT795091
MT795093
MT795094
MT795095
MT795096
Patient 66: 9 sequences
MT033713
MT033716
MT033717
MT033719
MT033720
MT033721
MT033726
MT033727
MT033735
Patient 67: 6 sequences
MT033853
MT033855
MT033857
MT033865
MT033867
MT033870
Patient 68: 2 sequences
MW924797
MW924798
Patient 69: 1 sequence
MW924802
Patient 70: 1 sequence
MW924804
Patient 71: 2 sequences
MW924805

MW924806
Patient 72: 1 sequence
MW924809
Patient 73: 1 sequence
MW924812
Patient 74: 1 sequence
MW924814
Patient 75: 12 sequences
MZ397027
MZ397028
MZ397029
MZ397030
MZ397031
MZ397032
MZ397033
MZ397034
MZ397035
MZ397036
MZ397037
MZ397038
Patient 76: 13 sequences
MZ397064
MZ397065
MZ397066
MZ397068
MZ397069
MZ397070
MZ397071
MZ397072
MZ397073
MZ397074
MZ397075
MZ397076
MZ397077

Supplementary File 3
Group of people living with HIV-1 and classified as long-term non-progressors (LTNP; progress to AIDS in >12 years after primary infection)

N. patient	*N. intact asp ORF*	*N. truncated asp ORF*	*% truncated asp ORF*
1	4	4	50.0
2	17	0	0.0
3	2	0	0.0
4	22	4	15.4
5	0	14	100.0
6	1	0	0.0
7	1	0	0.0
8	8	2	20.0
9	1	0	0.0
10	2	0	0.0
11	1	0	0.0
12	0	2	100.0
13	0	2	100.0
14	1	0	0.0
15	0	1	100.0
16	1	0	0.0
17	3	1	25.0
18	0	1	100.0
19	1	0	0.0
20	1	0	0.0
21	1	0	0.0
22	2	0	0.0
23	0	2	100.0
24	0	2	100.0
25	2	0	0.0
26	2	0	0.0
27	0	1	100.0
28	1	0	0.0
29	0	1	100.0
30	5	0	0.0
31	1	0	0.0
32	2	0	0.0
33	2	0	0.0
34	2	0	0.0
35	0	2	100.0
36	1	0	0.0
37	2	0	0.0
38	1	0	0.0
39	0	1	100.0
40	4	0	0.0
41	8	0	0.0
42	1	0	0.0
43	11	0	0.0
44	0	1	100.0
45	0	2	100.0

46	0	20	100.0
47	15	0	0.0
48	18	0	0.0
49	15	0	0.0
50	86	0	0.0
51	19	1	5.0
52	12	0	0.0
53	1	0	0.0
54	1	0	0.0
55	106	80	43.0
56	1	0	0.0
57	1	0	0.0
58	3	1	25.0
59	1	0	0.0
60	2	0	0.0
61	7	0	0.0
62	2	0	0.0
63	0	1	100.0
64	10	1	9.1
65	1	0	0.0
66	153	0	0.0
67	4	0	0.0
68	47	0	0.0
69	6	0	0.0
70	1	0	0.0
71	26	2	7.1
72	5	1	16.7
73	5	0	0.0
74	0	1	100.0
75	1	0	0.0

NCBI accession number of the HIV-1 sequences from each patient LTNP

Patient 1: 8 sequences
AF224507
DQ295194
OK075248
OK075249
OK075250
OK075251
OK075252
OK075253
Patient 2: 17 sequences
AJ418478
AJ418479
AJ418480
AJ418481
AJ418482
AJ418483
AJ418484
AJ418485
AJ418486
AJ418487

AJ418488
AJ418489
AJ418490
AJ418491
AJ418492
AJ418493
AJ418494
Patient 3: 2 sequences
AF042102
AF042106
Patient 4: 26 sequences
DQ837381
KU869570
MW405290
MW405291
MW405293
MW405294
MW405296
MW405297
MW405298
MW405330
MW405331
MW405332
MW405333
MW405334
MW405335
MW405336
MW405337
MW881608
MW881646
MW881647
MW881648
MW881649
OK075284
OK075285
OK075286
OK075287
Patient 5: 14 sequences
JQ316136
JQ316137
JQ316138
KF561436
KF561437
KF561438
KF561439
KY820470
KY820473
MT877447
MT877448
MW405300
MW405304
MW405338

Patient 6: 1 sequence
AY228556
Patient 7: 1 sequence
KC595159
Patient 8: 10 sequence
U69584
U69585
U69586
U69587
U69588
U69589
U69590
U69591
U69592
U69593
Patient 9: 1 sequence
AF538302
Patient 10: 2 sequences
AF538303
GQ995528
Patient 11: 1 sequence
AF128126
Patient 12: 2 sequences
AY322184
AY322185
Patient 13: 2 sequences
AY322193
EU110085
Patient 14: 1 sequence
AY445524
Patient 15: 1 sequence
AY322191
Patient 16: 1 sequence
AY818644
Patient 17: 4 sequence
AY779550
AY779551
AY779552
AY779564
Patient 18: 1 sequence
DQ367256
Patient 19: 1 sequence
KC595186
Patient 20: 1 sequence
AF042103
Patient 21: 1 sequence
AF042104
Patient 22: 2 sequences
DQ141341
KC595191
Patient 23: 2 sequences
DQ141342

KC595192
Patient 24: 2 sequences
DQ141343
KC595193
Patient 25: 2 sequences
DQ141344
KC595198
Patient 26: 2 sequences
DQ141345
KC595199
Patient 27: 1 sequence
DQ448819
Patient 28: 1 sequence
AF042105
Patient 29: 1 sequence
AY322189
Patient 30: 5 sequences
AY970946
AY970947
AY970948
AY970949
AY970950
Patient 31: 1 sequence
AY228557
Patient 32: 2 sequences
DQ487190
DQ487191
Patient 33: 2 sequences
DQ487188
DQ487189
Patient 34: 2 sequences
EF531330
KC595179
Patient 35: 2 sequences
EF531331
KC595180
Patient 36: 1 sequence
EF531332
Patient 37: 2 sequences
EF531333
KC595181
Patient 38: 1 sequence
EF531334
Patient 39: 1 sequence
EF531335
Patient 40: 4 sequences
MH595839
MH595840
MH595841
MH595842
Patient 41: 8 sequences
DQ054367

MW881609
MW881610
MW405339
MW405340
MW405341
MW405342
MW405305
Patient 42: 1 sequence
KC595184
Patient 43: 11 sequences
EF121404
EF121405
EF121406
EF121407
EF121408
EF121409
EF121410
EF175209
EF175210
EF175211
EF175212
Patient 44: 1 sequence
ay322190
Patient 45: 2 sequences
MH460465
MN153476
Patient 46: 20 sequences
FM165626
FM165627
FM165628
FM165629
FM165630
FM165631
FM165632
FM165633
FM165634
FM165635
FM165636
FM165637
FM165638
FM165639
FM165640
FM165641
FM165642
FM165643
FM165644
FM165647
Patient 47: 15 sequences
FJ147124
FJ147125
FJ147126
FJ147127

FJ147128
FJ147129
FJ147130
FJ147131
FJ147132
FJ147133
FJ147134
FJ147135
FJ147136
FJ147137
FJ147138
Patient 48: 18 sequences
FJ147106
FJ147107
FJ147108
FJ147109
FJ147110
FJ147111
FJ147112
FJ147113
FJ147114
FJ147115
FJ147116
FJ147117
FJ147118
FJ147119
FJ147120
FJ147121
FJ147122
FJ147123
Patient 49: 15 sequences
FJ147091
FJ147092
FJ147093
FJ147094
FJ147095
FJ147096
FJ147097
FJ147098
FJ147099
FJ147100
FJ147101
FJ147102
FJ147103
FJ147104
FJ147105
Patient 50: 86 sequences
GU455425
GU455426
GU455427
GU455428
GU455429

GU455430
GU455431
GU455432
GU455433
GU455434
GU455435
GU455436
GU455437
GU455438
GU455439
GU455440
GU455441
GU455442
GU455443
GU455444
GU455445
GU455446
GU455447
GU455448
GU455449
GU455450
GU455451
GU455452
GU455453
GU455454
GU455455
HQ231091
HQ231092
HQ231093
HQ231094
HQ231095
HQ231096
HQ231097
HQ231098
HQ231099
HQ231100
HQ231101
HQ231102
HQ231103
HQ231104
HQ231105
HQ231106
HQ231107
HQ231108
HQ231109
HQ231110
HQ231111
HQ231112
HQ231113
HQ231114
HQ231115
HQ231116

HQ231117
HQ231118
HQ231119
HQ231120
HQ231121
HQ231122
HQ231123
HQ231124
HQ231125
HQ231126
HQ231127
HQ231128
HQ231129
HQ231130
HQ231131
HQ231132
HQ231133
HQ231134
HQ231135
HQ231136
HQ231137
HQ231138
HQ231139
HQ231140
HQ231141
HQ231143
HQ231144
HQ902005
Patient 51: 20 sequences
GU455494
GU455495
GU455496
GU455497
GU455498
GU455499
GU455500
GU455501
GU455502
GU455503
GU455504
GU455505
GU455506
GU455507
GU455508
GU455509
GU455510
GU455511
GU455512
GU455513
Patient 52: 12 sequences
GU455514
GU455515

GU455516
GU455517
GU455518
GU455519
GU455520
GU455521
GU455522
GU455523
GU455524
GU455525
Patient 53: 1 sequence
FJ152546
Patient 54: 1 sequence
FJ152547
Patient 55: 186 sequence
JQ609684
JQ609685
JQ609686
JQ609687
JQ609688
JQ609689
JQ609690
JQ609691
JQ609692
JQ609693
JQ609694
JQ609695
JQ609696
JQ609697
JQ609698
JQ609699
JQ609700
JQ60970
JQ609702
JQ609703
JQ609704
JQ609705
JQ609706
JQ609707
JQ609708
JQ609709
JQ609710
JQ609711
JQ609712
JQ609713
JQ609714
JQ609715
JQ609716
JQ609717
JQ609718
JQ609719
JQ609720

JQ609721
JQ609722
JQ609723
JQ609724
JQ609725
JQ609726
JQ609727
JQ609728
JQ609729
JQ609730
JQ609731
JQ609732
JQ609733
JQ609734
JQ609735
JQ609736
JQ609737
JQ609738
JQ609739
JQ609740
JQ609741
JQ609742
JQ609743
JQ609744
JQ609745
JQ609746
JQ609747
JQ609748
JQ609749
JQ609750
JQ609751
JQ609752
JQ609753
JQ609754
JQ609755
JQ609756
JQ609757
JQ609758
JQ609759
JQ609760
JQ609761
JQ609762
JQ609763
JQ609764
JQ609765
JQ609766
JQ609767
JQ609768
JQ609769
JQ609770
JQ609771
JQ609772

JQ609773
JQ609774
JQ609775
JQ609776
JQ609777
JQ609778
JQ609779
JQ609780
JQ609781
JQ609782
JQ609783
JQ609784
JQ609785
JQ609786
JQ609787
JQ609788
JQ609789
JQ609790
JQ609791
JQ609792
JQ609793
JQ609794
JQ609795
JQ609796
JQ609797
JQ609798
JQ609799
JQ609800
JQ609801
JQ609802
JQ609803
JQ609804
JQ609805
JQ609806
JQ609807
JQ609808
JQ609809
JQ609810
JQ609811
JQ609812
JQ609813
JQ609814
JQ609815
JQ609816
JQ609817
JQ609818
JQ609819
JQ609820
JQ609821
JQ609822
JQ609823
JQ609824

JQ609825
JQ609826
JQ609827
JQ609828
JQ609829
JQ609830
JQ609831
JQ609832
JQ609833
JQ609834
JQ609835
JQ609836
JQ609837
JQ609838
JQ609839
JQ609840
JQ609841
JQ609842
JQ609843
JQ609844
JQ609845
JQ609846
JQ609847
JQ609848
JQ609849
JQ609850
JQ609851
JQ609852
JQ609853
JQ609854
JQ609855
JQ609856
JQ609857
JQ609858
JQ609859
JQ609860
JQ609861
JQ609862
JQ609863
JQ609864
JQ609865
JQ609866
JQ609867
JQ609868
JQ609869
Patient 56: 1 sequence
KC595197
Patient 57: 1 sequence
KC595202
Patient 58: 4 sequences
KC595189
KC595190

MH605986
MH605987
Patient 59: 1 sequence
KC595195
Patient 60: 2 sequences
KC595150
KC595224
Patient 61: 7 sequences
KC595149
KC595221
KC595225
KC595226
KC595227
MH605989
MH605990
Patient 62: 2 sequences
KC595168
MH605992
Patient 63: 1 sequence
KC595194
Patient 64: 11 sequences
KX595119
KX595120
KX595121
KX595122
KX595123
KX595124
KX595125
KX595126
KX595127
KX595128
KX595129
Patient 65: 1 sequence
MK757421
Patient 66: 153 sequences
MK053935
MK053936
MK053937
MK053938
MK053939
MK053940
MK053941
MK053942
MK053943
MK053944
MK053945
MK053946
MK053947
MK053948
MK053949
MK053950
MK053951

MK053952
MK053953
MK053954
MK053955
MK053956
MK053957
MK053958
MK053959
MK053960
MK053961
MK053962
MK053963
MK053964
MK053965
MK053966
MK053967
MK053968
MK053969
MK053970
MK053971
MK053972
MK053973
MK053974
MK053975
MK053976
MK053977
MK053978
MK053979
MK053980
MK053981
MK053982
MK053983
MK053984
MK053985
MK053986
MK053987
MK053988
MK053989
MK053990
MK053991
MK053992
MK053993
MK053994
MK053995
MK053996
MK053997
MK053998
MK053999
MK054000
MK054001
MK054002
MK054003

MK054004
MK054005
MK054006
MK054007
MK054008
MK054009
MK054010
MK054011
MK054012
MK054013
MK054014
MK054015
MK054016
MK054017
MK054018
MK054019
MK054020
MK054021
MK054022
MK054023
MK054024
MK054025
MK054026
MK054027
MK054028
MK054029
MK054030
MK054031
MK054032
MK054033
MK054034
MK054035
MK054036
MK054037
MK054038
MK054039
MK054040
MK054041
MK054042
MK054043
MK054044
MK054045
MK054046
MK054047
MK054048
MK054049
MK054050
MK054051
MK054052
MK054053
MK054054
MK054055

MK054056
MK054057
MK054058
MK054059
MK054060
MK054061
MK054062
MK054063
MK054064
MK054065
MK054066
MK054067
MK054068
MK054069
MK054070
MK054071
MK054072
MK054073
MK054074
MK054075
MK054076
MK054077
MK054078
MK054079
MK054080
MK054081
MK054082
MK054083
MK054084
MK054085
MK054086
MK054087
Patient 67: 4 sequences
MH605968
MH605969
MH605970
MH605971
Patient 68: 47 sequences
MT530694
MT530695
MT530696
MT530697
MT530698
MT530699
MT530700
MT530701
MT530702
MT530703
MT530704
MT530705
MT530706
MT530707

MT530708
MT530709
MT530710
MT530711
MT530712
MT530713
MT530714
MT530715
MT530716
MT530717
MT530718
MT530719
MT530720
MT530721
MT530722
MT530723
MT530724
MT530725
MT530726
MT530727
MT530731
MT530732
MT530733
MT530734
MT530735
MT530736
MT530737
MT530738
MT530739
MT530740
MT530741
MT530742
MT530744
Patient 69: 6 sequences
MN871328
MN871329
MN871330
MN871331
MN871332
MN871333
Patient 70: 1 sequence
MH605991
Patient 71: 28 sequences
JQ341411
KF561440
KF561441
KU869605
KY820508
KY820515
KY820520
KY820521
KY820522

KY820523
KY820524
KY820525
KY820526
KY820527
KY820528
MG461319
MG461320
MG461321
MG461322
MK871374
MK871374
MN043579
MT877450
MT877451
MT877452
MT877455
MW405288
MW405329
Patient 72: 6 sequences
JQ316127
KU869587
KU869588
MN043583
OK075245
OK075246
Patient 73: 5 sequences
JQ316134
OK075272
OK075273
OK075274
OK075275
Patient 74: 1 sequence
KF561435
Patient 75: 1 sequence
AY819715

Supplementary File 4

Sample dataset of 3725 env sequences (ENV_{FL-ASP} in the text) that overlap an intact asp ORF (presence of canonical start and stop codons and absence of internal stops). Each env sequence has a length of 289 codon positions, including positions with gap. The reverse complementary of triplet CTA (third base at codon position 1 and first and second base at codon position 2 in env) corresponds to the TAG termination codon of the antisense asp ORF. The reverse complementary of triplet CAT (third base at codon position 288 and first and second base at codon position 289 in env) corresponds to the ATG start codon of the antisense asp ORF. The 3725 env sequences are first reported in the alignment format with gaps and then in the format without gaps. In the reference genome sequence of HIV-1 (NCBI accession number K03455) the region of env overlapping asp is comprised between nucleotides 7371-7943.

```
>K03455
TTCTACTGTAATTCAACACAACTGTTTAATAGTACTTGGTTTAATAGTACTTGGAGTACTGAAGGGTCAAATA
ACACT--------------------------------------------------------------------
-------------------------
GAAGGAAGTGACACAATCACCCTCCCATGCAGAATAAAACAAATTATAAACATGTGGCAGAAAGTAGGAAAAG
CAATGTATGCCCCTCCCATCAGTGGACAAATTAGATGTTCATCAAAT---
ATTACAGGGCTGCTATTAACAAGAGATGGTGGTAATAGCAACAAT----------------------------
-------------------------------------------------------------------------
-------------
GAGTCCGAGATCTTCAGACCTGGAGGAGGAGATATGAGGGACAATTGGAGAAGTGAATTATATAAATATAAAG
TAGTAAAAATTGAACCATTA---GGAGTAGCACCCACCAAGGCAAAGAGAAGAGTGGTG--------------
-------------------------CAGAGAGAAAAAAGAGCA---GTGGGA---ATAGGAGCT---------
TTGTTCCTTGGGTTCTTGGGAGCA---GCAGGAAGCACTATG---GGCGCAGCCTCA---
ATGACGCTGACGGTACAG---GCCAGACAA---TTATTG---TCTGGTATAGTGCAGCAGCAGAAC---
AATTTG---CTGAGG---GCTATTGAGGCGCAACAGCATCTGTTGCAACTCACAGTCTGGGGCATC
>KF725913
TTCTATTGTAATACATCAGGACTGTTTAATAGCACCTGGAGGAATGGAACCATGCAGGAGCCAAACAGCACAC
AG-----------------------------------------------------------------------
----------------------------
TTAAATGACACTATAACTCTCAAGTGCAGAATAAGGCAAATTATAAATATGTGGCAGAGAGCAGGAAAAGCAA
TGTATGCCCCTCCCATCCAAGGAAAAATACAGTGTAAATCAAAT---
ATTACAGGATTACTATTGACAAGAGATGGTGGTGCGGAAAATAAT----------------------------
-------------------------------------------------------------------------
-------
GCAAGTGTACCTGAAATCTTCAGACCTGGAGGGGGAGATATGAGAGACAATTGGAGAAGTGAATTATATAGGT
ATAAAGTAGTAAAAATTGAACCACTA---GGAGTAGCACCCACCAAGGCAAAGAGAAGAGTGGTG--------
-------------------------------GAGAGAGAAAAAAGAGCACTAATTGGA---CTAGGAGCT---
------GTCTTCATTGGGTTCTTAGGAGCA---GCAGGAAGCACTATG---GGCGCGGCGTCA---
ATAACGCTGACGGTACAG---GCCAGGCAA---TTATTG---TCTGGCATAGTGCAACAGCAAGCC---
AACTTG---CTGAGG---GCTATAGAGGCGCAACAACACCTGTTGAGACTCACGGTCTGGGGCATT
>KY968401
TTCTACTGTAATACAACACAGCTGTTTAATTTTAATAGTACTTGGAATAATAATGACACTGAAGGAAATTTCA
CTGAAGAT-----------------------------------------------------------------
----------------------------
AATAAAACTACTATCACACTCCCATGCAGAATAAAACAAATTATAAACAGGTGGCAGGAAGTAGGAAAAGCAA
TGTATGCCCCTCCCATCAGAGGACAAATTAGCTGTTCATCAAAT---
ATTACAGGGCTGCTATTAACAAGAGATGGTGGTAACAACACTAAC----------------------------
-------------------------------------------------------------------------
-------------
```

AACAATGAAACTTTCAGACCTGGAGGAGGAGATATGAGGGACAATTGGAGAAGTGAATTATATAAATATAAAG
TAGTAAGAATTGAGCCATTG---GGAATAGCACCCACCAAGGCAAAGAGAAGAGTGGTG--------------
-------------------------CAGAGAGAAAGAAGAGCA---GTGGGA---ATGGGAGCC---------
ATGTTCCTTGGGTTCTTGGGAGCA---GCAGGAAGCACTATG---GGCGCAGCGTCA---
ATAACGCTGACGGTACAG---GCCAGACAA---TTATTG---TCTGGTATAGTGCAACAGCAGAAC---
AATCTG---CTGAGA---GCTATTGATGCGCAACAGCATCTGTTGCAACTCACAGTCTGGGGCATC
>KY968403
TTTTACTGTAATACATCAAAGCTGTTTAATAACACTTGGAATGGCACTGCATGGCCAAGCACTGAA-------
----

GAAAATGAAACTATCACACTTCCATGCAGAATAAAACAAATTATAAACAGGTGGCAGGAAGTAGGAAAGGCAA
TGTATGCCCCTCCCATCAAAGGTGTAATTAGCTGTTCATCAAAT---
ATCACAGGGCTGTTATTAACAAGAGATGGGGATAACAATAACGGG----------------------------
----

TCCGAGACTTTCAGACCTGGAGGAGGAGATATGAGGGACAATTGGAGAAGTGAATTATATAAATATAAAGTAG
TAAGAATTGAGCCATTA---GGAATAGCACCCACCGAGGCAAAGAGAAGAGTGGTG-----------------
----------------------CAGAGAGAAAAAAGAGCA---GTGGGA---ATGGGAGCT---------
ATGTTCCTTGGGTTCTTGGGAGCA---GCAGGAAGCACTATG---GGCGCAGCGTCC---
ATGACGCTGACGGTACAG---GCCAGACAA---TTATTG---TCTGGTATAGTGCAACAGCAGAAC---
AACCTG---CTGAGA---GCTATTAAGGCGCAACAGCATCTGTTGCAGCTCACAGTCTGGGGCATC
>AY037269
TTCTACTGTAATACAACAAAGCTGTTTAATAGTACTTGGGTAAATGGCACTTTAAATAACACTGCAGGAAATG
AC---

AGTGACACAAGCATCATACTCCCATGCAGAATAAAACAAATTATAAACAGATGGCAGGAAGTAGGAAAAGCAA
TGTATGCCCCTCCCATTAGCGGAGTAATTAGCTGTGTATCAAAT---
ATTACAGGGCTGCTATTAGCAAGAGATGGTGGTGGGAACACGAGC----------------------------
----

AGAGACAACGAAACCTTCAGACCTGTAGGGGGAGATATGAGGGACAATTGGAGAAGTGAATTATATAAATATA
AAGTAGTAAGAATTGAGCCAGTA---GGAATAGCACCCACCAAGGCAAAGAGAAGAGTGGTG-----------
----------------------------CAGAGAGAAAAAAGAGCA---GTGGGA---TTAGGAGCT------
---ATGTTCCTTGGGTTCTTGGGAGCA---GCAGGAAGCACTATG---GGCGCAGCGTCA---
GTGACGTTGACGGTACAG---GCCAGACAA---TTGTTG---TCTGGTATAGTGCAACAGCAGAAC---
AATCTG---CTGAGA---GCTATTGAGGCGCAACAACACCTGTTGCAACTCACAGTCTGGGGCATC
>KY968394
TTCTACTGTAATTCATCACAACTGTTTAATAGCACTTGGGCTTTGAATAGTACTAACACTACTGCTGAA----
----

GGAAATGAAACTATCATACTCCCATGCAGAATAAAACAAATTATAAACATGTGGCAACAAGTAGGAAGAGCAA
TGTATGCCCCTCCTATCAAAGGACTAATTAGATGCTCATCAAAT---
ATTACAGGGATACTATTAACAAGAGATGGTGATAGTAATAGAACT----------------------------
----

AATAATGAGACCTTCAGACCTGGAGGAGGAAATATGAAGGATAATTGGAGAAGTGAATTATATAAATATAAAG
TAGTAAAGATTGAGCCATTA---GGACTAGCACCCACCAAGGCAAAAAGAAGAGTGGTG--------------
-------------------------CAGAGAGAGAAAAGAGCA---GTGGGA---TTAGGAGCT---------
ATGTTCCTTGGGTTCTTGGGAGCA---GCAGGAAGCACTATG---GGCGCAGCGTCA---
TTGACGCTGACGGTACAG---GCCAGACTA---TTATTG---TCTGGTATAGTGCAACAGCAGAAC---
AATCTG---CTGAGA---GCTATTGAGGCGCAACAACATATGTTGCAACTCACAGTCTGGGGCATT
>KY968407
TTCTACTGTAATTCATCAAAGCTGTTTAACAGTATTTGGTGGTCTAATAATACTAATACTACAGAGGGAATT-
----

GCAGGAAAAAATATCACACTCCCATGCAGAATAAAACAAATTATAAACAGATGGCAGGAAGTAGGAAAAGCAA
TGTATGCCCCTCCCATCAGTGGACTAGTTAGATGTTCATCAAAT---
ATTACAGGACTGCTATTAACAAGAGATGGGGGTAAAGATAATGAAAATAGCAAT-------------------
----

AATAGGACTGAAACCTTCAGACCTGGAGGAGGAAATATGAAGGACAATTGGAGAAGTGAATTATATAAATATA
AAGTAGTGAAGATTGAGCCATTA---GGAGTAGCACCCACCAGGGCAAAGAGAAGAGTGGTG-----------
----------------------------CAGAGAGAAAAAAGAGCA---GTAACA---ATAGGAGCT------
---GTGTTCCTTGGGTTCTTGGGAGCA---GCAGGAAGCACTATG---GGCGCAGCGTCA---
ATGACGCTGACGGTACAG---GCCAGACAA---TTACTG---TCTGGTATAGTGCAACAGCAGAAC---
AATTTG---CTGAGG---GCTATTGAGGCGCAACAGCATCTGTTGCAACTCACAGTCTGGGGCATC
>DQ383746
TTCTACTGTAATACAACACAACTGTTCAATAGTACTTGGAATTCAACACAACTTAATCCTAATAGCTCTTTGA
GTACC--

AATGATACTGCTACTATCACACTCCCATGCAGAATAAAACAAATTATAAACATGTGGCAGAGAGTAGGAAAAG
CAATGTATGCCCCTCCCATTGAAGGACACATTAGGTGTTCATCAAAT---
ATTACAGGACTGCTATTAACAAGAGATGGTGGTAACGAGAGCGAG----------------------------
----

TCTGAGATCTTCAGGCCTGGAGGAGGAGATATGAGGGATAATTGGAGAAGCGAATTATATAAATATAAAGTAG
TAAAAGTTGAACCATTA---GGAATAGCACCCACCAAGGCAAAGAGAAGAGTGGTG-----------------
----------------------CAGAGAGAAAAAAGAGCA---GTGGGA---CTAGGAGCT---------
ATGTTCCTTGGGTTCTTGGGAGCA---GCAGGAAGCACTATG---GGCGCAGCGTCA---
ATGGCGCTGACGGTACAG---GCCAGACAA---TTATTG---TCTGGTATAGTGCAACAGCAAAAC---
AATTTG---CTGATG---GCTATTGAGGCGCAACAGCATATGTTGCGACTCACAGTCTGGGGCATT
>KY968396
TTCTACTGTAATACAACACAGCTATTTAATAGTACGTGGAACGAGACATGGGCAAATGACACAGAA-------
----

GGAAATGACACAATCACACTCCCATGCAGAATAAAACAAATTATAAACATGTGGCAGGAAGTAGGAAAAGCAA
TGTATGCCCCTCCCATCAGCGGACCAATTAACTGTACATCCAAT---
ATTACAGGGCTGCTATTAACAAGAGATGGTGGGAATAGGAATACAAGCGAG----------------------
----

CTCCTCGAGACCTTCAGACCTGGAGGAGGAGATATGAGGGACAATTGGAGAAGTGAATTATATAAATATAAAG
TAGTAAAGATTGAACCATTA---GGAGTAGCGCCCACCAAGGCAAAGAGAAGAGTGGTG--------------
-------------------------CAAAGAGAAAAAAGAGCA---GTGGGA---TTTGGAGCT---------
ATATTCCTTGGGTTCTTGGGAGCA---GCAGGAAGCACTATG---GGCGCAGCGTCA---
GTGACGCTGACGGTACAG---GCCAGACAA---TTATTG---TCTGGTATAGTGCAGCAGCAGAAC---
AATCTG---CTGAGG---GCTATTGAGGCGCAACAACACCTGTTGCAACTCACAGTCTGGGGCATC
>DQ383748
TTCTACTGTAATACAACAAAACTGTTTGATAATAGTACTTGGAATAACACTGAGGAGTCA-------------
----

AATAGCACTATATTCACACTCCCATGCAGAATAAAACAAATTATAAACATGTGGCAGGAAGTAGGAAAAGCAA
TGTATGCCCCTCCCATCAGAGGACAAATTAACTGTTCATCAAAT---
ATTACAGGGCTGCTATTAACAAGAGATGGTGGGACCGAGGAGAAG----------------------------
----

GAGATCTTCAGACCTGGAGGAGGAGATATGAGGGACAATTGGAGAAGTGAATTATATAAATACAAAGTAGTAA
AAATTGAACCATTA---GGAGTAGCACCCACCAAGGCAAAGAGAAGAGTGGTG--------------------
-------------------CAAAGAGAGAGAAGAGCA---GTGGGA---ATAGGAGCC---------
CTGTTCCTTGGGTTCTTAGGAGCA---GCAGGAAGCACTATG---GGCGCAGCGTCG---
CTGACGCTGACGGTACAG---GCCAGACAA---TTATTG---TCTGGTATAGTGCAACAGCAGAAC---
AATATG---CTGAGG---GCTATTGAGGCGCAACAGCATATGTTGCAACTCACAGTCTGGGGCATC
>DQ383751
TTCTACTGTAATACAACACAACTGTTTAATAGTGCTTGGAATGATACTAATATAAACAGAACTGAAGGGTTAA
ACAAC--

ACAGGAAGTGGCACTATCACACTCCCATGCAGAATAAAACAAATTATAAACCTGTGGCAGGAAGTAGGAAAAG
CAATGTATGCCCCTCCCATCAGTGGACAAATTAATTGTTCATCAAAT---
ATTACAGGGTTGCTATTAACAAGAGATGGGGGTAATGGTAATAACACA-------------------------
----

AACAAGACAGAGATCTTCAGACCTGGAGGAGGAAATATGAAGGACAATTGGAGAAGTGAATTATATAAATATA
AAGTAGTAAAAGTTGAACCATTA---GGAATAGCACCCACCATGGCAAAGAGAAGAGTGGTG-----------
----------------------------CAAAGAGAAAAAAGAGCA---GTGGGA---ATAGGAGCG------
---TTGTTCCTTGGGTTCTTGGGAGCA---GCAGGAAGCACTATG---GGCGCAGCGTCA---
ATAACGCTGACGGTACAG---GCCAGACAA---CTATTG---TCTGGTATAGTGCAACAGCAAAAT---
AATTTG---CTGAGA---GCTATTGAAGCGCAACAGCATATGTTGCAGCTCACAGTCTGGGGCATC
>KY968399
TTCTACTGTAATACAACACAACTGTTTAATAGTACTTGGAGTACTTGGAATAGTACTAAAGGGGTAAATGGCA
CT---

GAAGAAAAGAACATCACACTCCCATGCAGAATAAAACAAATTATAAACATGTGGCAGGAAGTAGGAAGAGCAA
TGTATGCCCCTCCCATCAATGGACAAATTAGATGTTCATCAAAA---
ATTACAGGGCTACTATTAACAAGAGATGGTGGTACTAACAGTACC----------------------------
----

AACGAGACCTTCAGACCTGGAGGAGGAGATATGAGGGACAATTGGAGAAGTGAATTATATAAATACAAAGTAG
TAAAAATTGAACCATTA---GGAGTAGCACCCACCGAGGCAAAGAGAAGAGTGGTG-----------------
----------------------CAGAGAGAAAAAAGAGCA---CTGGGA---CTAGGAGCT---------
CTGTTCCTTGGGTTCTTGGGAGCA---GCAGGAAGCACTATG---GGCGCAGCGTCA---
CTGACGCTGACGGTACAG---GCCAGACAA---TTATTG---TCTGGTATAGTGCAACAGCAGAAC---
AATTTG---CTGAGG---GCTATTGAGGCGCAACAGCATATGTTGCAACTCACAGTCTGGGGCATC
>KY658686
TTCTACTGTAATATATCACAACTGTTTAATAGTACTTGGAATCCAAATACTACAGAATCAGACAACACAAAT-
----

AACACTGAACTTATCATACTCCCATGTAGAATAAAACAAATTATAAACTTGTGGCAGGAAGTAGGAAAAGCAA
TGTATGCTCCTCCCATCCAAGGAAACATTACCTGTTCATCAAAT---
ATTACAGGGCTAGTATTAACAAGAGATGGTGGTGATAATGCTACT----------------------------
----

CTTGAGGTCTTCAGACCTGGAGGAGGAAATATGAAGGACAATTGGAGAAGTGAATTATATAAATATAAAGTAG
TAAGAATTGAACCATTA---GGAGTAGCACCCACCAAGGCAAAGAGAAGAGTGGTG-----------------
----------------------CAGAGAGAAAGAAGAGCA---GTGGGAGCGCTAGGAGCT---------
ATGTTCCTTGGGTTCTTGGGAACA---GCAGGAAGCACTATG---GGCGCAGCGGCA---
ATGACGCTGACGGTACAG---GCCAGACAA---TTATTG---TCTGGTATAGTGCAACAGCAAAAC---
AATTTG---CTGAGA---GCTATTGAGGCGCAACAGCATCTGTTGCAACTCACAGTCTGGGGCATC
>KY658683
TTCTACTGTAATACAACACAGTTGTTTAATAGTACTTGGAATGCAAATAATACCCAAGGGTCAAATGGA----
----

ACTGGAGAAATTATCACACTCCCATGCAGAATAAAACAAATTATAAACAAATGGCAGGAAGTAGGAAAAGCAA
TGTATGCCCCTCCCATCAAAGGAAACATTAGTTGTTCCTCAAAT---
ATCACAGGGCTGCTACTAACAAGGGATGGTGGTAGCAATAGCAGC----------------------------
----

AACGAAACCTTCAGACCTGGAGGAGGAGATATGAGGGACAATTGGAGAAGTGAACTATATAAATATAAAGTGG
TAAAAATTGAACCATTA---GGAGTAGCACCTACCAGGGCAAGGAGAAGAGTGGTG-----------------
----------------------CAAAGAGAAAAAAGAGCA---CTAGGGATTGTGGGAGCT---------
GGGCTCCTTGGGTTCTTAGCAGCA---GCAGGAAGCACTATG---GGCGCAGCGTCG---
GTGACGCTGACGGTACAG---GCCAGACTA---TTATTG---TCTGGTATAGTGCAACAGCAGAAC---
AATCTG---CTGAGG---GCTATTGAGGCGCAACAGCACCTGTTGCAACTCACAGTCTGGGGCATC
>MH078530
TTCTACTGTAATACAACAAAGCTGTTTAATAGTAATAGTAATTGGCATTCCAATAAGACTTGGGAGGGG----
----

CTAAATGACACTATCACACTCCCATGCAGAATAAAACAAATTATAAACACATGGCAGGAAGTAGGAAAAGCAA
TGTATGCCCCTCCCATCAGAGGACGAATTAATTGTTCATCAAAT---
ATTACAGGGCTGCTACTAACAAGAGATGGTAGTGGCAGCAATGACACT-------------------------
----

AATAATACCGAGATCTTCAGACCTGGAGGAGGAAATATGAGGGACAATTGGAGAAGTGAATTATATAAATATA
AAGTAGTGAAAATTGAACCAATA---GGAATAGCACCCACCAAGGCAAAGAGAAGAGTGGTG-----------
----------------------------CAGAGAGAAAAAAGAGCG---ATAACG---CTGGGAGCT------
---ATGTTCCTTGGGTTCTTGGGAACA---GCAGGAAGCACTATG---GGCGCAGCGTCG---
CTGACGCTGACGGTACAA---GCCAGACAA---TTGTTG---TCTGGTATAGTGCAACAGCAGAAC---
AATCTG---CTGAGA---GCTATTGAGGCGCAACAGCATCTGTTGCAGCTCACAGTCTGGGGCATC
>KY658685
TTCTACTGTAACACAACACAACTGTTTAATAGTACTTGGAACAGTACTTGGAATTGGAACAGTACTACTGAGA
ACTCA--

ACAAACTTTAACATAACAATTCCATGCAAAATAAAACAAATTGTAAACAGGTGGCAAGAGGTAGGAAAAGCAA
TGTATGCCCCTCCCATCAGAGGAATAATCCAATGCACATCAAAC---
ATTACAGGGTTGCTATTAACAAGAGATGGTGGTAATGAGAATAAT----------------------------
----

ATTAGTGAGATTTTCAGACCTGGAGGAGGAGATATGAGGGACAATTGGAGAAGTGAATTATATAAATACAAAG
TAGTACAAATTGAACCAATA---GGAATAGCACCCACCAAGGCAAAGAGAAGAGTGGTG--------------
-------------------------CAGAGAGAGAAAAGAGCA---GTGGGA---ATAGGAGCT---------
ATGTTCCTTGGGTTCTTGAGCGCA---GCAGGAAGCACTATG---GGCGCAGCGTCA---
GTGACGCTGACGGTACAG---GCCAGACAA---TTATTG---TCTGGTATAGTGCAGCAGCAGAAC---
AATCTG---CTGAAG---GCTATTGAGGCACAACAGCATCTGTTGCAACTCACAGTCTGGGGCATC
>MH078533
TTCTACTGTAATTCAACACCATTGTTTAATAGTACTTGGAATAGTAGTACTTGGAATAATAGTAATATTGAAC
AGGTTAAT---
-------------------GGA---
ACTGAACAGGATACTATCACACTCCACTGCAGAATAAAACAAATAGTAAACATGTGGCAGGAAGTAGGAAAAG
CAATGTATGCCCCTCCTATCAGAGGACAAATTAGATGTTCATCAAAG---
ATTACGGGGCTACTGTTAGTAAGAGATGGGGGTAAAAACAACACA----------------------------
----

CAGAACGAGACCTTCAGGCCTACAGGAGGAAACATGAAGGACAATTGGAGAAGTGAATTATATAAATATAAAG
TAGTAGAAATTAAGCCATTA---GGAATAGCACCAACCAAGGCAAAGAGAAGAGTGGTG--------------
-------------------------CAGAGAGAAAAAAGAGCA---GTGGGA---GTAGGAGCT---------
ATGATCCTTGGTTTCTTGAGTACA---GCAGGAAGCACTATG---GGCGCAGCGTCA---
GTGACGCTGACGGTACAA---GCCAGACAA---TTATTG---TCTGGTATAGTGCAGCAGCAAAGC---
AATTTG---CTGAGG---GCTATTGAGGCGCAACAACATATGTTGCAACTCACAGTCTGGGGCATC
>AY037268
TTCTACTGTAATACAACACAACTGTTTAGTAGTACTTGGAATGAGACTAATATTTGGAATGAGACTAATACA-
----

GATAATAGCACAATCACACTCCAATGCAGAATAAAGCAATTTATAAACATGTGGCAGAAAGTAGGAAAAGCAA
TGTATGCCCCTCCCATCAGAGGACACATTAGATGTTCATCAAAT---
ATTACAGGGCTGCTATTAACAAGAGATGGTGGTATTATCAACACC----------------------------
----

ACCGAGACCTTCAGACCTGGAGGAGGAGATATGAGGGACAATTGGAGAAGTGAATTATATAAATACAAAGTAG
TAAGAATTGAACCATTA---GGAGTAGCACCCACCAAGGCAAAGAGAAGAGTGGTG-----------------
----------------------CAGAGAGAAAAAAGAGCA---ATAGGGGTAATAGGAGCT---------
TTGTTCCTTGGGTTCTTGGGAGCA---GCAGGAAGCACTATG---GGCGCAGCGTCA---
GTGACGTTGACGGTACAG---GCCAGACAA---TTATTG---TCTGGTATAGTGCAACAGCAGAAC---
AATCTG---CTAAGG---GCTATTGAGGCGCAACAGCATCTGTTGCAACTCACAGTCTGGGGCATT
>AY037282
TTCTACTGTGATACAACAAAACTGTTTAATAGTACTTGGACTACTGAAGGGTTAAATAGCACTGAAGGAAAT-
----

AGCACAAAAATAATCACACTCCCATGTAGAATAAGACAAATTATAAACATGTGGCAGGAAGTAGGAAAAGCAA
TGTATGCCCCTCCCATCAGTGGACAAATTAACTGTTCATCAAAT---
ATTACAGGGCTGCTATTAACAAGAGATGGTGGTAATGACACTAACAGT-------------------------
----

AGCAATACCGAGATATTCAGACCTGGAGGAGGAAATATGAAGGACAATTGGAGAAGTGAATTATATAAATACA
AAGTAGTACAAATTGAACCAATA---GGAGTAGCGCCCACCAAGGCAAAGAGAAGAGTGGTG-----------
----------------------------CAGAGAGAAAAAAGAGCA---GTGGGA---CTAGGAGCC------
---TTGTTCCTTGGGTTCTTGGGAGCA---GCAGGAAGCACTATG---GGCGCAGCGTCA---
ATAACGCTGACGGTACAG---GCCAGACAA---TTATTG---TCTGGTATAGTGCAACAGCAGAAC---
AATCTG---CTGAGA---GCTATTGAGGCGCAACAACATCTGTTGCAACTCACAGTCTGGGGCATC
>FJ952154
TTCTACTGTAACACAACAAAATTGTTTAATAGTACTTGGAATAGTAATGCTCCTTGGAATAGTACTGAATGG-
----

TTAAATGACACTATCACACTCCCATGCAGAATAAAACAAATTATAAACATGTGGCAGAAAGTAGGAAAAGCAA
TGTATGCCCCTCCCATCAGGGGACAAATTAGATGCTCATCAAAT---
ATTACAGGGCTAATATTAACAAGAGATGGTGGCAGAAATGGTAACAGTAGC----------------------
----

AACAACGGGAGCGAGACCTTCAGACCTGGAGGAGGAGATATGAGGGACAATTGGAGAAGTGAATTATATAAAT
ATAAAGTAGTAAAAATTGAGCCATTA---GGACTAGCACCCACCAAGGCAAAGAGAAGAGTGGTG--------
-------------------------------CAGAGAGAAAAAAGAGCA---GTGGGA---CTAGGAGCT---
------GTGTTCCTTGGGTTCTTGGGAGCA---GCAGGAAGCACTATG---GGCGCAGCGTCA---
GTAACGCTGACGGTACAG---GCCAGACTA---TTATTG---TCTGGTATAGTGCAACAGCAAAGC---
AATTTG---CTGAGG---GCTATTGAGGCGCAACAGCATCTGTTGCAACTCACAGTCTGGGGCATC
>DQ676886
TTCTACTGTAATTCAACACCACTGTTTAATAGTACTTGGGTGCGAGATGGTAATACTTGGAGGGAGTCTAATA
GAACTAAT---
-------------AGTAGT------
ACTTCAAGTGTTAATATCACACTCCCATGCAGAATAAAACAAATTATAAACATGTGGCAGGAAGTAGGAAAAG
CAATGTATGCCCCTCCTATCAAAGGACTAATTAGATGTTCATCAAAT---
ATTACAGGGATACTATTAACAAGAGATGGTGGTAATAACAATACC----------------------------
----

ACCAATGAAACTTTCAGACCTGGAGGAGGAGATATGAGAGACAATTGGAGAAGTGAATTATATAAATATAAAG
TAGTAAAAATTGAACCATTA---GGGATAGCACCCACCAGGGCAAAGAGAAGAGTGGTG--------------
-------------------------CAGAGAGAAAAAAGAGCA---GTAACA---TTAGGAGCT---------
ATGTTCCTTGGGTTCTTGGGAGCA---GCAGGAAGCACTATG---GGCGCAGCGTCA---
CTGACGCTGACGGTACAG---GCCAGACTA---TTATTG---TCTGGTATAGTGCAACAGCAGAAC---
AATCTG---CTGAGG---GCTATTGAGGCGCAACAACATCTGTTGCAACTCACAGTCTGGGGCATT
>AF042100
TTCTACTGTAATTCAACACAACTGTTTAATAGTACTTGGAATATTAATAGTACTTGGAATGATACTGGAGAGT
CAAATAAC---
-------------------ACT---
GTAGGCAATGTCACACTCACACTCCCATGCAGAATAAAACAAATTGTAAACCTGTGGCAGGAAGTAGGAAAAG
CAATGTATGCCCCTCCCATCAGCGGACTAATTAGATGTTCATCAAAT---
ATTACAGGGCTGCTATTAACAAGAGATGGGGGTAAGGGTAATGAC----------------------------
----

ACGGTCGAGACCTTCAGACCTGGAGGAGGAGATATGAGGGACAATTGGAGAAGTGAATTATATAAATATAAAG
TAGTAAAAATTGAACCATTA---GGAGTAGCACCCACTAAGGCTAAGAGAAGAGTGGTG--------------
-------------------------CAGAGAGAAAAAAGAGCA---GTGGGAATAGGAGCTGTG---------
TTGTTCCTTGGGTTCTTGGGAGCA---GCAGGAAGCACTATG---GGCGCAGCGTCA---
ATAACGCTGACGGTACAG---GCCAGACAA---TTATTG---TCTGGTATAGTGCAACAGCAGAAC---
AATTTG---CTGAGG---GCTACTGAGGCGCAACAGCATCTGTTGCAACTCACAGTCTGGGGCATC
>AF042101
TTTTACTGTAATTCAACACAACTGTTTAATGGTACTTGGAATGATACTGAAAGGTCATGGTCATATAACACTG
CA---

GAAAATAATGACACAATCACGCTCCCATGTAAAATAAAACAAATTATAAACAGGTGGCAGGAAGTAGGAAAAG
CAATATATGCCCCTCCCATCAGAGGACAAATTAAATGTTCATCAAAC---
ATTACAGGGCTGCTCTTAACAAGAGATGGTGGCCCTGACAATAAA----------------------------
----

```
GACACTGAGATCTTCAGACCTGGAGGAGGAGATATGAGGGACAATTGGAGAAGTGAATTATATAAATATAAAG
TAGTAAAAATTGAACCATTA---GGAATAGCACCCACTAAGGCAAAGAGAAGAGTGGTG--------------
-------------------------CAGAGAGAAAAAAGAGCA---GTGGGA---CTAGGAGCT---------
TTGTTCCTTGGGTTCTTGGGAGCA---GCAGGAAGCACTATG---GGCGCAGCGTCA---
ATAACGCTGACGGTACAG---GCCAGACAA---TTATTG---TCTGGTATAGTGCAGCAGCAGAAC---
AATCTG---CTGAGG---GCTATTGAGGCGCAACAGCATCTGTTGCAACTCACAGTCTGGGGCATC
>AF538304
TTCTACTGTAATACAACACAACTGTTTAATAGTACTTGGAATGTTACTGAAAGGTTAAATAACACTGAAAGA-
-------------------------------------------------------------------------
----------------------------
GATAATACTACAATCACACTCCCATGCAGAATAAAACAACTTATAAACATGTGGCAGGAGGTAGGAAAAGCAA
TGTATGCCCCTCCCATTAGCGGACGAATTAGATGTTCATCAAAT---
ATTACAGGGCTGATATTAACAAGAGATAGTGGTAGCGGGAACGGG----------------------------
-------------------------------------------------------------------------
----------------
ACCGAGACCTTCAGACCTGGAGGAGGAAATATGAAGGACAATTGGAGAAGTGAATTATATAAATATAAAGTAG
TAAGAATTGAACCATTA---GGAGTAGCACCCACCAAGGCAAAGAGAAGAGTGGTG-----------------
----------------------CAGAGAGAAAAAAGAGCA---TTAGGGGGACTAGGAGCT---------
ATGTTCCTTGGGTTCTTGGGAGCA---GCAGGAAGCACTATG---GGCGCAGCGTCA---
ATGGCGCTGACGGTACAG---GCCAGACAA---TTATTG---TCTGGTATAGTGCAACAGCAGAAC---
AATCTG---CTGAGA---GCTATTGAGGCGCAACAGCATCTGTTGCAACTCACAGTCTGGGGCATT
>AF538306
TTCTACTGTAATTCAACACCACTGTTTAATAGTACTTGGAATGTTACTACAGGGTCAAGTAACATGGAAGAGG
AT-----------------------------------------------------------------------
----------------------------
AGTCCAGAAAATATTACACTCACATGCAGAATAAAACAAATTATAAACATGTGGCAGGAAGTAGGAAAAGCAA
TGTATGCCCCTCCCATCAAAGGACTAATTAGCTGTTCATCAAAC---
ATTACAGGGCTGCTATTAACAAGAGATGGTGGTAATAATAGCGAGACC-------------------------
-------------------------------------------------------------------------
----------
AAGACCACCGAGACCTTCAGACCTGGAGGAGGAGATATGAGGGACAATTGGAGAAGTGAATTATATAAATACA
AAGTAGTAAAAATTGAACCATTA---GGAGTAGCACCCACCAGGGCAAAGAGAAGAGTGGTG-----------
----------------------------CAGAGAGAAGAGAGAGCA---GTGGGA---ATAGGAGCT------
---TTGTTCCTTGGGTTCTTGGGAGCA---GCAGGAAGCGCTATG---GGCGCAGCATCA---
ATGACGCTGACGGTACAG---GCCAGACAA---TTATTG---TCTGGTATAGTGCAGCAGCAGAAC---
AATTTG---CTGTGG---GCTATTGAGGCGCAACAGCATCTGTTGCAACTCACAGTCTGGGGCATC
>AF042103
TTCTACTGTGATTCAACACGACTGTTTAATAGTACTTGGATTAATGGTACTCGGAATGAAACTGAAGGC----
-------------------------------------------------------------------------
----------------------------
AATGGAAGCACTATCACACTCCCCTGCAAAATAAAACAAATTATAAACCTGTGGCAGGAAGTAGGAAAAGCAA
TGTATGCCCCTCCCATCAGAGGACAAATTAGATGTACATCAAAT---
ATTACAGGGCTGATATTAACAAGAGATGGTGGTAATAACACGAAC----------------------------
-------------------------------------------------------------------------
-------------
GAGACCGAGATCTTCAGACCTGGAGGAGGAGATATGAGGGACAATTGGAGAAGTGAATTATATAGGTATAAAG
TAGTAAAAATTGAACCATTA---GGAGTAGCACCCACCAAGGCAAAGAGAAGAGTGGTG--------------
-------------------------CAGAGAGAAAAAAGAGCA---GTGGGA---ATAGGAGCT---------
GTGTTCCTTGGGTTCTTAGGAGCA---GCAGGAAGCACTATG---GGCGCAGCGTCA---
CTGACGCTGACGGTACAA---GCCAGACAA---TTATTG---TCTGGTATAGTGCAGCAGCAGAGC---
AATTTG---CTGAGG---GCTATTGAGGCGCAACAGCATCTGTTGCAACTCACAGTCTGGGGCATT
>AF042104
TTCTACTGTAATTCAACACAACTGTTTAATAGTATTTGGAAAAATGATACTTGGAATAATAGTACTTGGGATG
AC-----------------------------------------------------------------------
----------------------------
ACTGCAGGACATATCACACTTCCATGCAGAATAAAACACATTGTAAACATGTGGCAGGAAGTAGGAAAAGCCA
TGTATGCCCCTCCCATCAGAGGACAAATTAGATGTACATCAAAT---
ATTACAGGGATGCTACTAACAAGAGATGGTGGTAATAGCGATAACGCG-------------------------
-------------------------------------------------------------------------
----------
```

AACGGGACCGAGACCTTCAGACCTGGAGGCGGAGATATGAGGGACAATTGGAGATGTGAACTATATAGATATA
AAGTAGTAAAAATTGAACCATTA---GGGATAGCACCCACCAAGGCAAAGAGAAGAGTGGTG-----------
----------------------------CAGAGAGAAAAAAGAGCA---GTGGGA---ATAGGAGCT------
---GTGTTCCTTGGGTTCTTGGGAGCA---GCAGGAAGCACTATG---GGCGCAGCGTCA---
ATGACGCTGACGGTACAG---GCCAGACTA---TTATTG---TCTGGTATAGTGCAACAGCAGATC---
ATTTTG---CTGAGG---GCTATTGAGGCGCAACAGCATCTGTTGCAACTCACAGTCTGGGGCATC
>AF042105
TTCTACTGTAATACAACACCACTGTTTAATAGAACATGGGTTAATGGTACCTGGGATAATACTTGGGATAATA
CTACA--

GACATTAACGGAAATATCATACTTCCATGCAGAATAAAACAAATTATAAACATGTGGCAGAAAGTAGGAAAAG
CAATGTATGCCCCTCCCATCAGAGGAATAATTAACTGTACATCAAAT---
ATTACAGGGCTGATATTAACAAGAGATGGTGGTTTGAATAGTACA----------------------------
----

AACGAGACTTTCAGACCTGGAGGAGGAGATATGAGGGACAATTGGAAAAGTAAATTATATAAGTATAAAGTAG
TAGAAATTGAACCATTA---GGAGTAGCACCCACCAAGGCAAAAAGAAGAGTGGTG-----------------
----------------------CAGAGAGAAAAAAGAGCG---GTGACA---CTAGGAGCT---------
ATGTTCCTTGGGTTCTTAGGAGCA---GCAGGAAGCACTATG---GGCGCAGCGTCA---
ATAACGCTGACGGCACAA---GCCAGACAA---TTATTG---TCTGGTATAGTGCAGCAGCAGAAC---
ACTCTG---CTGAGG---GCTATTAAGGCGCAACAGCATCTGTTGCAACTCACAGTCTGGGGCATC
>AF538302
TTCTATTGTAATTCAACAGGATTGTTTAATAGTACCTGGAATAATAGTACCTGGAATGATACTGAAAAGTCAA
ATAGC--
-------------------ACT---
GGAGGAAATGATACAATCACACTCCAATGCAGAATAAAACAAATTATAAACTTGTGGCAGGAAGTAGGAAAAG
CAATGTATGCCCCTCCCATCAGAGGAGAAATTAGATGTACATCAAAT---
ATTACAGGGCTGTTATTAACAAGAGATGGTGGTATTGTCAAGGAG----------------------------
----
----------------AACGAGACCTTCAGACCTGGAGGAGGC---GATATGAGG---
GACAATTGGAGAAGTGAATTATATAAAGTAGTACAAATTGAACCATTA---
GGAGTAGCACCCACCGAGGCAAAGAGAAGAGTGGTG-------------------------------------
--CAGAGAGAAAAAAGAGCA---ATAGGA---ATAGGAGCT---------
GTGTTCCTTGGGTTCTTGGGAGCA---GCAGGAAGCACTATG---GGCGCAGCGTCA---
ATGACGCTGACGGTACAG---GCCAGACTA---TTATTG---TCTGGTATAGTGCAACAGCAGAAC---
AATTTG---CTGAGG---GCTATTGAGGCGCAACAGCATCTGTTGCAACTCACAGTCTGGGGCATC
>AY624305
TTCTACTGTAATACATCAAAACTGTTTAATAGCACTTGGAATAGTAATGGGACTTGGAATGGTGATAAGGAA-
----

ACTAAAGGAATTATCGTACTCCCATGTAGAATAAAACAAATTATAAACATGTGGCAGGGAGTAGGAAAAGCAA
TGTATGCCCCTCCCGTCAAAGGAAAAATTGAATGTTCATCAAAT---
ATTACAGGGCTGCTATTAACAAGAGATGGTGGTATTAACAGCAGC----------------------------
----

GATAGCGAAACCTTCAGACCTGGAGGAGGAAATATGAAGGACAATTGGAGAAGTGAATTATATAAATATAAAG
TAGTAGAAATTGAACCATTA---GGAGTAGCACCCACCAAGGCAAAGAGAAGAGTGGTG--------------
-------------------------CAGAGAGAAAAAAGAGCA---GTGGGAATGATAGGAGCT---------
ATGTTCCTTGGGTTCTTGGGAGCA---GCAGGAAGCACTATG---GGCGCAGCGTCA---
ATGGCGCTGACGGTACAG---ACCAGACAA---TTATTG---TCTGGTATAGTGCAACAGCAGAAC---
AACTTG---CTGAAG---GCTATTGAGGCGCAACAGCATCTGTTGCAACTCACAGTCTGGGGCATC
>JN002002
TTCTACTGTAACACAACACAGCTGTTTAATAGTACTTGGAATTCAACACAACTGTTTAATAGTACTGGGGATG
ATACT--
-------------------GGA---
GAGTCAAATAAAATAATCACACTCCCATGTAGAATAAAACAAATTATAAACATGTGGCAGGAAGTAGGAAAAG
CAATGTATGCCCCTCCCATCAGAGGACAAATTAGATGTTTATCAAAT---
ATTACAGGGCTGATATTAACAAGAGATGGTGGTAATAACAGTGACACG-------------------------
----

AACACCACCGAGATCTTCAGACCGGGAGGAGGAGATATGAGGGACAATTGGAGAAGTGAACTATATAAATATA
AAGTAGTAAAAATTGAACCATTA---GGAGTAGCACCTACCAAGGCAAAGAGAAGAGTGGTG-----------
----------------------------CAGAGAGAAAAAAGAGCA---GTGGGAACGCTGGGAGCT------
---ATGTTCCTTGGGTTCTTGGGAGCA---GCAGGAAGCACTATG---GGCGCAGCGTCA---
ATAACGCTGACGGTACAG---GCCAGACTA---TTATTG---ACTGGTATAGTGCAACAGCAGAAC---
AACCTG---CTGAGG---GCTATTGAGGCGCAACAGCATCTGTTGCAACTCACAGTCTGGGGCATC
>JN002009
TTCTACTGTAATTCAACACAACTGTTTAATAGTACTTGGTTTAATAGTATTTGGAAAGTTAATGATACTGAAG
AGTCAAAT---
-------------AGCACT------
ACAGAAAATATTACAATCACACTCCCATGTAGAATAAAACAAATTATAAATATGTGGCAGGAAGTAGGAAAAG
CAATGTATGCCCCTCCCATCAGAGGACAAATTAGCTGTTTATCAAAT---
ATTACAGGGCTGTTATTAACAAGAGATGGTGGTAAGAATAACCAGAGC-------------------------
----

AACACCACCGAGACCTTCAGACCAGGAGGAGGAAATATGAAGGACAATTGGAGAAGTGAATTATATAAATATA
AAGTAGTAAGAATTGAACCATTA---GGAGTAGCACCCACCAAGGCAAAGAGAAGAGTGGTG-----------
----------------------------CAGAGAGAAAAAAGAGCA---GTGGGAACGATAGGAGCT------
---ATGTTCCTTGGGTTCTTGGGAGCA---GCAGGAAGCACTATG---GGCGCAGCGTCG---
CTGACGCTGACGGTACAG---GCCAGACTA---TTATTG---TCTGGTATAGTGCAGCAGCAGAAC---
AATCTG---CTGAGG---GCTATTGAGGCGCAACAGCATCTGTTGCAACTCACAGTCTGGGGCATC
>GU367406
TTCTACTGTAATTCAACACAACTGTTTGATAGTACATGGAATGATACTGAAAGTCCAAATAGCAAT-------
----

GACACCACAAATATCACACTCCCATGTAGAATAAAACAAATTATAAACATGTGGCAGAAAGTAGGAAAAGCAA
TGTATGCCCCTCCCATCAGCGGAATAATCAAATGTTCATCAAGT---
ATTACAGGGCTGCTTCTAACAAGAGATGGTGGTAGAAGTAACAGCAATGAC----------------------
----

ACTAATAACACTGAGATCTTCAGACCTGCAGGGGGAGATATGAAGGACAATTGGAGAAGTGAATTATATAAGT
ATAAAGTAGTAAAAATTGAACCATTA---GGAGTAGCACCCACCAAGGCAAAGAGAAGAGTGGTG--------
-------------------------------CAGAGAGAAAAAAGAGCA---GTGGGAATGCTAGGAGCT---
------ATGTTCCTCGGGTTCTTGGGAGCA---GCAGGAAGCACTATG---GGCGCAGCGTCA---
ATGACGCTGACGGTACAG---GCCAGACAA---TTATTG---TCTGGTATAGTGCAGCAGCAGAAC---
AACTTG---CTGAAG---GCTATTGAGGCGCAACAGCATCTGTTGCAACTCACAGTCTGGGGCATC
>GU367395
TTCTACTGTGATACATCAAAACTGTTTAATAGTACTTGGAATGGTCGGAATGAAAGTATCACAAAT-------
----

AGCACTGCAAATTTCACACTCCCATGTAGAATAAAACAGATTATAAACATGTGGCAGGGAGTAGGAAAAGCAA
TGTATGCCCCTCCCATCAGAGGACAAATTAGATGTTTATCAAAT---
ATTACAGGGATACTATTAACAAGAGATGGTGGTAATACTGGGAAC----------------------------
----

AACACCACCGAGATATTCAGACCTGGAGGAGGAGATATGAGGGACAATTGGAGAAGTGAACTATATAAATATA
AAGTAGTAGAAATTAAACCATTA---GGAGTAGCACCCACCCAGGCAAAGAGAAGAGTGGTG-----------
----------------------------CAAAGAGAAAAAAGAGCA---GTGGGAGTAATAGGAGCT------
---ATGTTCCTTGGGTTCTTGGGAGCA---GCAGGAAGCACTATG---GGCGCAGCGTCA---
ATGACACTGACGGTACAA---GCCAGACAA---TTATTG---TCTGGTATAGTGCAGCAGCAGAAC---
AATCTG---CTGAGA---GCTATTGAGGCACAACAGCATCTGTTGCAACTCACAGTCTGGGGCATC
>FJ653258
TTCTACTGTAATACAATACAATTGTTCAACAGTACTTGGAATGAAAATGATACTGAGAATGACAATAGTACTG
AA---

GGATCAAATGACACTATCACACTCCAATGCAAAATAAAGCAAATTGTAAACATGTGGCAGGAAGTAGGAAAAG
CAATGTATGCCCCTCCCATCAGCGGGAACATTACATGCTCATCACAC---
ATTACAGGGTTGCTATTAACAAGAGATGGTGGTGCGAACAGCAAC----------------------------
----

AATACCGAGATCTTCAGACCTGGAGGAGGAGATATGAGGGACAATTGGAGAAGTGAATTATATAAATATAAAG
TAGTAAGAATTGAACCATTA---GGAGTAGCACCCACCAAGGCAAAAAGAAGAGTGGTG--------------
-------------------------CAGAGAGAAAAAAGAGCA---GTGGGA---TTAGGAGTT---------
GTGTTCCTTGGGTTCTTGGGAGCA---GCAGGAAGCATTATG---GGCGCAGCGTCA---
CTAACGCTGACGGTACAA---GCCAGACAA---TTATTG---TCTGGTATAGTGCAACAGCAGGAC---
AACCTG---CTGAGA---GCTATTGAGGCGCAACAGCACCTGTTGCAACTCACAGTCTGGGGCATC
>MN485973
TTCTACTGTAATTCAACACAGCTGTTTAATAATAGTACTTGG-------------------------------
----
-------------------AAT---
GAAACTGATAACATTATCACACTCCCATGCAGAATAAAACAAATTATAAACAGATGGCAGGAAGTAGGAAAAG
CAATGTATGCCCCTCCCATTGCAGGACAAATTAGATGTTCATCAAAC---
ATTACAGGGCTGCTATTAACAAGAGATGGTGGTGAGAACGCAAGC----------------------------
----

GCCGAGGTCTTCAGACCGGGAGGAGGAGATATGAGGGATAATTGGAGAAGTGAATTATATAAATATAAGGTAG
TAAAAATCGAACCATTA---GGGGTAGCACCCACCAAGGCAAAGAGAAGAGTGGTG-----------------
----------------------CAGAGAGAAAAAAGAGCA---GTGGGA---ATAGGAGCC---------
ATGTTCCTTGGGTTCTTGGGAGCA---GCAGGGAGCACTATG---GGCGCAGCGTCA---
ATGACGCTGACGGTACAG---GCCAGACTA---TTATTG---TCTGGTATAGTGCAACAGCAGAAC---
AATCTG---CTGAGG---GCTATTGAGGCACAACAGCATATATTGCAACTCACAGTCTGGGGCATC
>MN485981
TTCTACTGTAATTCATCACAACTGTTTAATAGTACTTGGCAGTCTAATGATACTTGGCAGAGTAATAGTGAG-
----

AATGATACTATCATCACGCTTCCATGCAGAATAAAACAAATTATAAACAGATGGCAGGAAGTAGGAAAAGCAA
TATATGCCCCTCCCATTAGCGGACTCATTAACTGTACATCAAAC---
ATTACAGGGCTAATACTAACAAGAGATGGTGGTAATGTTAGCGAC----------------------------
----

AACCCTGAGACCTTCAGACCTACAGGAGGAGATATGAAGGACAATTGGAGGAGTGAACTATATAAATATAAAG
TAGTAAAAATTGAGCCATTA---GGAATAGCACCCACCAAGGCAAAGAGAAGAGTGGTG--------------
-------------------------CAGAGAGAGAAAAGA------GTGGGAGCACTAGGAGCT---------
GTGTTCCTTGGGTTCTTGGGAACA---GCAGGAAGCACTATG---GGCGCAGCGTCA---
TTGACGCTGACGGTACAG---GCCAGAACA---TTATTG---TCTGGTATAGTGCAACAGCAGAAC---
AATTTG---CTGAAG---GCTATTGAGGCGCAACAACATCTGTTGCAACTCACAGTCTGGGGCATC
>MN485993
TTCTACTGTAATACAACACAACTATTTAATAGTACTTGGAGTGGTAATGAGACTAAGACTGCAGAAAAC----
----

AATACAGAAACTATCACGCTCCCATGCAGAATAAAACAAATTGTGCGCCTGTGGCAGGAAGTAGGAACAGCAA
TGTATGCCCCTCCCATCAGTGGACTAATTCGGTGTTCATCAAAT---
ATTACAGGACTGTTATTGGTAAGAGATGGTGGTAATATCAATGGAACC-------------------------
----

AATAACACTGAGATATTCAGACCTGGGGGAGGAGATATGAGGGACAATTGGAGAAGTGAATTATATAAATATA
AAGTAGTAAAAATTGAACCAATA---GGAGTAGCACCCACCAAGGCGAAGAGAAGAGTGGTG-----------
----------------------------CAGAGAGAAAAAAGAGCA---GTGACA---CTGGGAGCT------
---ATGTTCCTTGGGTTCTTGGGAGCA---GCCGGAAGCACTATG---GGCGCAGCATCA---
GTAACGCTGACGGTACAA---GCCAGACAA---TTATTG---TCTGGTATAGTGCAACAGCAGAAC---
AATCTG---CTGAGG---GCTATTGAGGCGCAACAGCATCTGTTGCAACTCACAGTCTGGGGCATC
>MN485996
TTCTATTGTAATACAACGCAGCTGTTTAATAGTACTTGGAATAGTACTTTGGAAAATAGTACTAGGAATAAT-
----

ACAAATGCCACCATTACACTCCCATGCAGAATAAAGCAAATTGTAAACCTGTGGCAAGAAGTAGGAAAAGCAA
TGTATGCCCCTCCCATCAGAGGACAAATTAGATGTATATCAAAT---
ATTACAGGGATGCTATTAACAAGAGATGGTGGTACAGGTAATAACACT-------------------------
----

AACAATACTGAGATCTTCAGACCTGGAGGAGGAGATATGAGAGACAATTGGAGAAGTGAACTATATAAATATA
AAGTAGTAAAAATTGAACCATTA---GGAGTAGCACCCACCAAGGCAAAGAGAAGAGTGGTG-----------
----------------------------CAGAGAGAAAAAAGAGCA---GTGGGA---CTAGGAGCT------
---TTGTTCCTTGGGTTCTTGGGAGCA---GCAGGAAGCACTATG---GGCGCAGCGTCA---
GTGACGCTGACGGTACAG---GCTAGACAA---TTATTG---TCTGGTATAGTGCAGCAGCAGAAC---
AACCTG---CTGAGG---GCTATTGAGGCGCAACAACATCTGTTGCAACTCACAGTCTGGGGCATC
>MN485998
TTCTACTGTAATACATCACAGCTGTTTAATAGTACTTGGAGTAGTAGCAATTGGACTGATAATAGCAATAACA
GCACACAA---

GGGTCAAATGACACTATAACTCTCCCATGCAGAATAAAACAAATTATAAACAAATGGCAAGAAGTAGGAAGAG
CAATGTATGCCCCTCCCATTAGAGGACAAATTAGATGCGAATCAAAT---
ATTACAGGGCTGCTATTAACAAGAGATGGAGGGATCCTGAACAGT----------------------------
----

ACCAATGAGACTTTCAGACCTGAAGGAGGAGATATGAGGAACAATTGGAGGAGTGAATTATATAAATATAAAG
TAGTAGAAATTGAACCATTA---GGAATAGCACCCACCAAGGCAAAGAGAAGAGTGGTG--------------
-------------------------CAGAGAGAAAAAAGAGCA---GTGGGA---ATAGGAGCT---------
CTGTTCCTTGGGTTCTTGGGAGCA---GCAGGAAGCACTATG---GGCGCAGCATCA---
ATGACGCTGACGGTACAG---GCCAGGCAA---TTACTG---TCTGGTATAGTGCAACAGCAGAAC---
AATTTG---CTGAGG---GCTATTGAGGCGCAACAGCATATGTTGCAACTCACAGTCTGGGGCATC
>MN486009
TTCTATTGTAATACATCACAACTGTTTAATAGCACTTGGGGGGTTAATAGTACCAGGAATGGTACACTTGCTT
GGTGGGTTAAT--
-GGTACCAGGAATGATACA------
CTTGTAAATGATACTATCACACTCCCATGCAGAATAAAACAATTTATAAACCTGTGGCAGGAAGTAGGAAAAG
CAATGTATGCCCCTCCCATCAAAGGACTAATTAAATGCTCATCAAAC---
ATTACAGGGTTACTATTAACAAGAGATGGTGGTAAAACCAATGAAACA-------------------------
----

AATAACACCGAGATCTTCAGACCGGGAGGAGGAAATATGAAGGATAATTGGAGAAGTGAATTATATAAATATA
AAGTAGTAAAAATTGAACCATTA---GGAGTAGCACCCACCAAGGCAAAGAGAAGAGTGGTG-----------
----------------------------CAAAGAGAAAAAAGAGCA---GTGGGA---ATAGGAACA------
---TTGTTCCTTGGGTTCTTGGGAGCA---GCAGGAAGCACTATG---GGCGCAGCATCA---
ATAACGCTGACGGTACAG---GCCAGACAA---TTATTG---TCTGGTATAGTGCAGCAGCAGAAC---
AACCTG---CTGAGA---GCTATTGAGGCGCAACAACATATGTTGCAACTCACAGTCTGGGGCATC
>MN486010
TTCTACTGTAATACATCACAGCTGTTTAATAGTACTTGGGAGTTTAATGATACAAGTAAAACTTGGTACATCA
ACAATACAATT--
-------------AGCAAC------
AACACAAATAGCACAATCATACTCCCATGCAGAATAAAACAAATTATAAACAAATGGCAGGAAGTAGGAAGAG
CAATATATGCCCCTCCCATCGAAGGACAAATTAGATGCGAATCAAAT---
ATTACAGGGCTGCTATTAACAAGAGATGGAGGGATGAATAACACC----------------------------
----

AATGAGACCTTCAGACCTGAAGGAGGAGATATGAGGAACAATTGGAGGAGTGAATTATATAAATATAAAGTAG
TAGAAATTGAACCATTA---GGAGTAGCACCCACCAAGGCAAAGAGAAGAGTGGTG-----------------
----------------------CAGAGAGAAAAAAGAGCA---GTGGGA---ATAGGAGCT---------
TTGTTCCTTGGGTTCTTGGGAGCA---GCAGGAAGCACTATG---GGCGCAGCGTCA---
TTAACGCTGACGGTACAG---GCCAGACAA---CTACTG---TCTGGTATAGTGCAACAGCAGAAC---
AATTTG---CTGAGA---GCTATTGAGGCGCAACAGCATATGTTGCAACTCACAGTCTGGGGCATC
>MN486013
TTCTACTGTAATACAACAAAACTGTTT--

----------ACTGAAAAC------
ATAAATAGCACAAATATCACACTCCCATGCAGAATAAAACAAATTATAAACATGTGGCAAAAAGTTGGAAAAG
CAATGTATGCCCCTCCCATCAGTGGAATAATTAGGTGTTCATCAAAT---
ATCACAGGGCTACTATTAATGAGAGATGGTGGTAATAGTAGTGGT----------------------------
----

AATGAGTCCGAGGTCTTCAGACCTGGAGGAGGAAATATGAAGGACAATTGGAGAAGTGAATTATATAAATATA
AAGTAGTAAGAGTTGAACCATTA---GGAATAGCACCCACCACGGCAAAGAGAAGAGTGGTG-----------
----------------------------CAGAGAGAAAAAAGAGCA---GTAGGA---CTAGGAGCT------
---GTGTTCCTTGGGTTCTTGGGAGCA---GCAGGAAGCACTATG---GGCGCAGCGTCA---
ATAACGCTGACGGTACAG---GCCAGACAA---TTATTG---TCTGGTATAGTGCAACAGCAAAAC---
AATATG---CTGAGA---GCTATTGAGGCGCAACAACATATGTTGCAACTCACAGTCTGGGGCATC
>MN486014
CTCTATTGTAATACATCACAGCTGTTTAATAGTACTTGGTTTAATGGTACTTGGAATGAGACTGGAGAGTCAA
AT---

AAAACAGAACTTATCATACTCCCATGCAGAATAAAACAAATCATAAACATGTGGCAGGAAGTAGGAAAAGCAA
TGTATGCCCCTCCCATCAAAGGAAACATTTCCTGTTCATCAAAT---
ATTACAGGACTGCTATTAACAAGGGATGGTGGTAACCTCAACGGGAGT-------------------------
----

AATGAGACCGAGATCTTCAGACCTGCAGGGGGAGATATGAGGGACAATTGGAGAAGTGAATTATATAAATATA
AAGTAGTAAGAATTGAACCATTA---GGAGTAGCACCCACCAGGGCAAAAAGAAGAGTGGTG-----------
----------------------------CAGAGAGAAAAAAGAGCA---TTGGGA---TTTGGAGCT------
---GTGTTCATTGGGTTCTTGGGAGCA---GCAGGAAGCACTATG---GGCGCAGCGTCA---
TTGACGCTGACGGTACAG---GCCAGAAAC---TTATTA---TCTGGTATAGTGCAGCAGCAGAAC---
AATCTG---CTGAGG---GCTATTGAGGCGCAACAGCATCTGTTGCAACTCACAGTCTGGGGCATC
>MN486022
TTCTACTGTAATACAACACAACTGTTTAATAGTACTTGGTATGCCAATAATACTTGGAATAATACTGGAGAG-
----

TCAAACATCACAATCACACTCCCCTGCAGAATAAGACAAATTGTAAACCTGTGGCAGGAAGTAGGAAAAGCAA
TGTATGCCCCTCCCATTAGAGGACCAATTACCTGTAGATCAAAT---
ATTACAGGGCTAATATTAACAAGAGATGGTGGTGAGAGTAACAATACC-------------------------
----

AAGAACAATGAGACCTTCAGACCCGGAGGAGGAGATATGAGGGACAATTGGAGAAGTGAATTATATAAATATA
AAGTAGTAAAAATTGAACCATTA---GGAGTAGCACCTACCAAGGCAAAGAGAAGAGTGGTG-----------
----------------------------CAGAGAGAAAGAAGAGCA---ATAGGA---TTAGGAGCT------
---ATGTTCCTTGGTTTCTTGGGAGCA---GCAGGAAGCACTATG---GGCGCAGCGTCA---
GTGACGCTGACGGTACAG---GCCAGACAA---TTATTG---TCTGGTATAGTGCAACAGCAGAGC---
AATCTG---CTGCAG---GCTATTGAGGCACAACAGCATCTGTTGAAACTCACAGTCTGGGGCATC
>DQ313249
TTCTACTGTAATACAACAAAACTGTTTAATAGTACTTGGAATGGTACTGAAGGGTCAAACAACACTGGAGGA-
----

GAAAATGACACGATCACACTCCCATGCAGAATAAAACAAATTGTAAACATGTGGCAGGAAGTAGGAAAAGCAA
TGTATGCACCTCCCATCAGAGGACAAATTAGATGTTCATCAAAT---
ATTACAGGGCTGATATTAACAAGAGATGGTGGTAATAATAATAAC----------------------------
----

ACGAACGAGACCTTCAGACCTGGAGGAGGAGATATGAGGGACAATTGGAGAAGTGAATTATATAAATATAAAG
TAGTAAAAATTGAACCATTA---GGAGTAGCACCCACCAGGGCAAAGAGAAGAGTGGTG--------------
-------------------------CAGAGAGAAAAAAGAGCA---ATAGCG------GGAGCT---------
GTGTTCCTTGGGTTCTTGGGAGCA---GCAGGAAGCACTATG---GGCGCAGCGTCA---
GTGGCGCTGACGGTACAG---GCCAGACTA---TTATTA---TCTGGTATAGTGCAACAGCAGAAC---
AATTTG---CTGAGG---GCTATTGAGGCGCAACAGCATCTGTTGCAACTCACAGTCTGGGGCATC
>L07421
TTCTACTGTAATTCAACAAAGCTGTTTAATAGTACTTGGAATGATACTAGTACTTGGAATGATACTACAGGAT
CA---

AATAAAGGAAATATCACACTCCTATGCAGAATAAAACAAATTATAAACAGGTGGCAAGAAGTAGGAAAAGCAA
TGTATGCCCCTCCCATCGAAGGACATATTAGATGTTCATCAAAT---
ATTACAGGGCTGTTACTAACAAGAGATGGTGGTAAGAACGAGAGC----------------------------
----

GAGACCACCGAGACCTTCAGACCTGGAGGAGGAGATATGAGGGATAATTGGAGAAGTGAATTATACAAATATA
AAGTGGTGAAGATTGAGCCATTA---GGAGTAGCACCCACCAAGGCAAAGAGAAGAGTGGTG-----------
----------------------------CAGAGAGAAAAAAGAGCA---GTGGGACTGATAGGAGCT------
---TTGTTCCTTGGATTCTTGGGAGCA---GCAGGAAGCACTATG---GGCGCGGCGTCA---
ATGACGCTGACGGTACAG---GCCAGACAA---TTATTG---TCTGGTATAGTGCAACAGCAGAGC---
AATTTG---CTGAGG---GCTATTGAGGCGCAACAGCATCTGTTGCAACTCACAGTCTGGGGCATC
>DQ313250
TTCTACTGTAATACATCACAACTGTTTAATACTACTTGGAGTGATACTACTACTTGGAATAATACTAACAAC-
----

ACAAATGGCAATATCACACTCCCATGCAGAATAAAACAAATTATAAACATGTGGCAGGGAGTAGGAAAAGCAA
TGTATGCTCCTCCCATCAGTGGACAAATTAGATGTTCATCAAAT---
ATTACAGGGCTGATATTAACAAGAGATGGTGGTCTCGCGAACAGG----------------------------
----

ACCAAAGAGACCTTCAGACCTGGAGGAGGAGATATGAGGGACAATTGGAGAAGTGAATTATATAAATATAAAG
TAGTAAAAATTGAACCATTA---GGAGTAGCACCCACCAAGGCAAAGAGAAGAGTGGTG--------------
-------------------------CAGAGAGAAAAAAGAGCA---GTGGGAATGCTAGGAGCT---------
GTGTTCCTTGGGTTCTTGGGAGCA---GCAGGAAGCACTATG---GGCGCAGCGTCA---
ATAACGCTGACGGTACAG---GCCAGACAA---TTATTG---TCTGGTATAGTGCAACAGCAGAAC---
AATCTG---CTGAAG---GCTATTGAGGCGCAACAGCATCTGTTGCAACTCACAGTCTGGGGCATC
>DQ313253
TTCTACTGTGATACAACACAACTGTTTAATAGTACTTGGCCTGTAGGGTCAAATACAAATGCCACAAATACC-
----

ACTGAAGAAATACTCACGCTCCCATGCAAAATAAAACAGATTATAAACAGGTGGCAGGAAGTAGGAAAAGCAA
TGTATGCCCCTCCCATCAGAGGAAAAATTAGATGTTCATCAAAT---
ATTACAGGGCTGCTAATAACAAGAGATGGTGGTAATAACAATAAC----------------------------
----

AATACGAACGAGACCTTCAGACCTGGAGGGGGAGATATGAGGGACAATTGGAGAAGTGAATTATATAAATATA
AAGTAGTAAAAATTGAACCATTA---GGAATAGCACCCACCAAGGCAAAGAGAAGAGTGGTG-----------
----------------------------CAGAGAGAAAAAAGGACA---GTGGGA---ATAGGAGCT------
---TTGTTCCTTGGTTTCTTGGGAGCA---GCAGGAAGCACTATG---GGCGCAGCGTCA---
CTGACGCTGACGGTACAG---GCCAGACAA---TTATTG---TCTGGTATAGTGCAACAGCAGAAC---
AATTTG---CTGAGG---GCTATTGAGGCGCAACAGCATATGTTGCAACTCACAGTCTGGGGCATC
>DQ313252
TTCTACTGTAATTCAACACCACTGTTTAATAGTACTTGGAATGATACTTGGAATGATAATGAACAGTCAAATG
AC---

ACTGAAGACACAATCACACTCCAATGCAGAATAAAACAAATTATAAACATGTGGCAGGAAGTAGGAAAAGCAA
TGTATGCCCCTCCCATCAGAGGACAAATTAGATGTTCATCAAAT---
ATTACAGGGCTACTATTAACAAGAGATGGTGGTAAAAATGATACCAATGAT----------------------
----

ACCGAGACCATCGAGGTCTTCAGACCTGGAGGAGGAGATATGAGGGACAATTGGAGAAGTGAATTATATAAAT
ATAAAGTAGTAAAAATTGAACCATTA---GGAGTAGCACCCACCAAGGCAAAGAGAAGAGTGGTG--------
-------------------------------CAGAGAGAAAAAAGAGCA---GTGGGA---ATAGGAGCT---
------GTGTTCCTTGGGTTCTTGGGAGCA---GCAGGAAGCACTATG---GGCGCAGCGTCA---
ATAACGCTGACGGTACAG---GCCAGACTA---TTATTG---TCTGGTATAGTGCAACAGCAGAAC---
AATTTG---CTGAGG---GCTATTGAGGCGCAACAACATCTGTTGCAACTCACAGTCTGGGGCATC
>EU191616
TTCTACTGTAATACAGCACAACTGTTTAATAGTACTTGGCATGCAAATGGCACTGTAGAGTCAACT-------
----

ACCACTGGAAATATCACACTCCCATGCAGAATAAAACAAATTATAAACATGTGGCAGGGAGTAGGAAAAGCAA
TGTATGCCCCTCCCATCAGAGGACAAATAAGATGTTCATCAAAT---
ATTACAGGGCTGCTCTTAACGAGAGATGGGGGGGATAATAACACG----------------------------
----

GACGAACCCGAAACCTTCAGACCTGCAGGAGGAGATATGAGGGACAATTGGAGAAGTGAACTATATAAATATA
AAGTAGTACAAATTGAACCATTA---GGAGTAGCACCCACCAAGGCAAAGAGGAGAGTGGTG-----------
----------------------------CAGAGAGAAAAAAGAGCA---ATAGGAACGATAGGAGCT------
---ATGTTCCTTGGGTTCTTGGGAGCA---GCAGGAAGCACTATG---GGCGCAGCGTCA---
ATAACGCTGACGGTACAG---GCCAGACAA---TTATTG---TCTGGTATAGTGCAACAGCAGAAC---
AACTTG---CTGAGG---GCTATTGAGGCGCAACAGCATCTGTTGCAACTCACAGTCTGGGGCATC
>MH746266
TTTTACTGTAATTCAACACAGCTATTTAATAGTACTTGGCTATTTAATAGTACTTGGAATGATACCAATTGGA
AGGGTACC---

GAAGGGAATGGTACCATCATACTCCCATGCAGAATAAAACAAATTATAAACAGATGGCAGGAAGTAGGACAAG
CAATGTATGCCCCTCCCATCAGCGGACTAATTAAATGTACATCAAAC---
ATTACAGGGCTATTACTAACAAGAGATGGTGGTAATAATGGAAGT----------------------------
----

AGTAACAATGAGACCTTCAGACCTGGAGGAGGAGACATGAGGGACAATTGGAGAAGTGAATTATATAAATATA
AGGTAGTAAAAATTGAGCCATTA---GGAGTAGCACCCACCAAGGCAAAGAGAAGAGTGGTG-----------
----------------------------CAGAGAGAAAAAAGAGCG---GTGGGAGCAATAGGAGCT------
---ATGTTCCTTGGGTTCCTGGGAGCA---GCAGGAAGCACTATG---GGCGCAGCGTCG---
ATGACGCTGACGGTACAG---GCCAGACAA---TTATTG---TCTGGTATAGTGCAACAGCAGAAC---
AACCTG---CTGAGG---GCTATTGAGGCGCAACAGCATCTGTTGCAACTCACAGTCTGGGGCATC
>MH746265
TTCTACTGTAATTCATCACAACTGTTTAATAGTACTTGGGATAGGAATAGTACTTGGAATGATCCTAAT----
----

AATAGCACTACTATCACACTCCCATGCAGAATAAAACAAATTATAAACATGTGGCAGGAAGTAGGAAAAGCAA
TGTATGCTCCTCCCATCAGTGGACTAATTAAATGCTCATCAAAT---
ATTACAGGACTACTATTGACAAGAGATGGTGGTAATAATAATAACACA-------------------------
----

AATGAGACAGAGGTCTTCAGACCTGGAGGAGGAGATATGAGGGACAATTGGAGAAGTGAATTATATAAATATA
AAGTAGTACAAATTGAACCATTA---GGAGTAGCACCCACTAAGGCAAAAAGAAGAGTGGTG-----------
----------------------------CAGAGAGAAAAAAGAGCT---CTGGGA---ATGGGAGCT------
---TTGTTCCTTGGGTTCTTGGGAGCA---GCAGGAAGCACTATG---GGCGCAGCGTCA---
ATAACGCTGACGGTACAA---GCCAGACAA---CTATTG---TCTGGTATAGTGCAACAGCAGAAC---
AATCTG---CTGAGG---GCTATTGAGGCGCAACAGCATATGTTGCAACTCACAGTCTGGGGCATC
>MH746264
TTCTACTGTAATTCAACACAACTGTTTAATAGCAGTTGGAGTTGGAATGGTACTAATGGTAATTGGAGTACTG
AAGAGCTAAAT--

GGCACTGAAAATATTACACTCCCATGTAGAATAAAACAAATAATAAACATGTGGCAAACAGTAGGAAAAGCAA
TGTATGCCCCTCCCATCAGAGGACAAATTAGATGTGCATCAAAT---
ATTACAGGGATGATACTAATAAGAGATGGTGGAAATGATGGAGGA----------------------------
----

GCCAACGAGACCTTCAGACCTGGAGGAGGAAATATGAGGGATAATTGGAGAAGTGAATTATATAAATATAAAG
TAGTACAAATTGAACCATTA---GGAGTAGCACCCACCAAGGCAAAGAGAAGAGTGGTG--------------
-------------------------CAGAGAGAAAAAAGAGCA---GTGGGAACAATAGGAGCT---------
ATGTTCCTTGGGTTCTTGGGAGCA---GCAGGAAGCACTATG---GGCGCAGCGTCA---
ATGACACTGACGGTACAG---GCCAGACAA---TTATTG---TCTGGTATAGTGCAACAGCAGAAC---
AATCTG---CTGAGG---GCTATTGAGGCGCAACAGCATCTGTTGCAACTCACAGTCTGGGGCATT
>MH746261
TTCTATTGTAATACATCAGAGTTGTTTAATAGCACTTGGAATGAGACTACTACAAATTTAACTGGTATA----
----

AATGACTCAACTATCATACTCCCATGTAGAATAAAACAAATTATAAATATGTGGCAGGGAGTAGGAAAAGCAA
TGTATGCCCCTCCCATCAACGGAAAAATTATGTGTAACTCAACT---
ATTACAGGACTGCTCTTAACAAGAGATGGTGGTAGTAATAATGAGACC-------------------------
----

AACACCACCGAGATCTTCAGACCTGCAGGAGGAGATATGAGGGACAATTGGAGAAGTGAATTATATAAATATA
AAGTAGTAAAAATTGAGCCAATA---GGAATAGCACCCACCAAGGCAAAGAGAAGAGTGGTG-----------
----------------------------CAGAGAGAAAAAAGAGCA---GTGGGAGTACTGGGAGCT------
---ATGTTCCTTGGGTTCTTGGGAGCA---GCAGGAAGCACTATG---GGCGCAGCGGCA---
ACAGCGCTGACGGTACAG---ACCAGACAA---TTATTG---TCTGGTATAGTGCGACAGCAGGAC---
AATCTG---CTGAAG---GCTATTGAGGCGCAACAGCACATGTTGCAACTCACAGTCTGGGGCATC
>MH746254
TTCTACTGTAATACAACAAAACTGTTTAATAGTACTTGGTATAGGAATAATACCAGTGACAGTAAAGAA----
----

GGAAATGGCACTATCACAATCCAATGCAGAATAAGACAAGTGATAAACATGTGGCAGGAAGTTGGCAAAGCAA
TGTATGCCCCTCCTATCAGAGGAAAAATTATCTGTTCATCAAAT---
ATCACAGGAATAATATTGACAAGAGATGGTGGTGAGAATAACACA---------------------------
----

AATAACAACGAGACCTTCAGACCTGGAGGAGGAGATATGAGGGACAATTGGAGAAGTGAATTATACAAATATA
AAGTAGTAAAAATTGAACCACTA---GGAGTAGCACCCACCAAGGCAAAGAGAAGAGTGGTG-----------
----------------------------CAGAGAGAAAAAAGAGCA---ATAACA---CTAGGAGCT------
---ATGTTCCTTGGGTTCTTGGGAGCA---GCAGGAAGCACTATG---GGCGCAGCGTCA---
CTGACGCTGACGGTACAG---GCCAGACAA---TTATTG---TCTGGTATAGTGCAGCAGCAGAAC---
AATCTG---CTGAGG---GCTATTGAGGCGCAACAACATCTGTTGCAACTCACGGTCTGGGGCATC
>DQ358809
TTTTACTGTAATACAACAAAGCTGTTTAATAGTACTTGGAATGATAATAGTACTGGAGAGGCTACTAGTACCT
GGAATAGTACT---
----------GAAGAGCCA------
GAAAATAGCGCAATTATCATACTCCCATGCATAATACAACTCATCATAAACAGGTGGCAGGAAGTAGGAAAAG
CAATGTATGCCCCTCCCATCAAGGGAATAATTAGCTGCTCATCAAAT---
ATTACAGGGTTGCTATTAACAAGAGATGGTGGTAACAATAGCGAG---------------------------
----

ACCAATGAGACCTTCAGACCTGGAGGAGGAGATATGAGGGACAATTGGAGAAGTGAATTATATAAATATAAAG
TAGTAAAAATTGAGCCATTA---GGAGTAGCACCCACCAAGGCAAAGAGAAGAGTGGTG--------------
-------------------------CAGAGAGAAAAAAGAGCA---GTGGGAACGCTAGGAGCT---------
ATGTTCCTTGGGTTCTTGGGAGCA---GCAGGAAGCACTATG---GGCGCAGCGTCA---
GTGACGCTGACGGTACAG---GCCAGACAA---TTATTG---TCTGGTATAGTGCAACAGCAAAAC---
AATTTG---CTGAAG---GCTATTGAGGCGCAACAACATTTGTTGCAACTCACAGTCTGGGGCATC
>DQ358810
TTCTACTGCAATACATCACGGCTGTTTAATACTACTTGGAATACTACTACTTGGAACAGTACTGAGCTGGCTA
ATATTAGT--
-------------GCTATT------
AACGATAATGAAACTATCACACTCCAGTGCAGGATAAAACAAATTATAAACATGTGGCAGGCGGTAGGAAAAG
CAATGTATGCCCCTCCCATCCAAGGACTAATTAGATGTTCATCAAAT---
ATAACAGGGCTGCTATTAACAAGGGATGGTGGTAATAATAGTATTAAGTGGAAC------------------
----
-
CAGAGCACATCCGACTCCGAGGTCTTCAGACCCCTAGGAGGAAATATGAAGGACAATTGGAGAAGTGAATTAT
ATAAATATAAAGTAGTACAAATTGAGCCATTA---GGAATAGCACCCACCAAGGCAAAGAGAAGAGTGGTG--
-------------------------------------CAGAGAGAGAAAAGAGCA---GTGGGA---
CTAGGAGCT---------GTGTTCATTGGGTTCTTGGGAGCA---GCAGGAAGCACTATG---
GGCGCAGCGTCA---ATAACGCTGACGGTACAA---GCCAGACAA---TTGTTG---
TCTGGTATAGTGCAACAGCAAAAC---AATTTG---CTGAAG---
GCTATTGAGGCGCAACAGCATCTGTTGCAACTCACAGTCTGGGGCATT
>JN692435
TTTTACTGTAATTCAACACAGCTGTTTAATAGTACTTGGCTGCATAATAACACTTGGAATGGTACTACAGGAT
CA--

AATAACAATGACACTATCACACTCCCATGCAGAATAAGACAAATTATAAACAGGTGGCAAGAAGTAGGAAAAG
CAATGTATGCCCCTCCCATCGCAGGACTAATTAGGTGTTCATCAAAT---
ATTACAGGGTTACTCTTAACAAGAGATGGTGGTCCGAACGTGACC---------------------------
----

ACCGAAATCTTTAGACCTGGAGGAGGAGATATGAGGGACAATTGGAGAAGTGAATTATATAAATATAAAGTAG
TAAGAATTGAGCCATTA---GGAGTAGCACCCACCAAGGCAAGGAGAAGAGTGGTG-----------------
----------------------CAGAGAGAAAAAAGAGCG---GTGACG---CTGGGAGCT---------
ATGTTCCTTGGGTTCTTGGGAGCA---GCAGGAAGCACTATG---GGCGCAGCGTCG---
ATAACGCTGACGGTACAG---GCCAGACAA---TTATTG---TCTGGTATAGTGCAACAGCAAAAC---
AATTTG---CTGAGA---GCTATTGAGGCGCAACAGCATCTGTTGCAGCTCACAGTCTGGGGCATC
>JN692440
TTCTACTGTGATACAACAAAGCTGTTTAATAGTACTTGGAATACTACTAGGAATACAACTAAAAGTGAAGAAG
AA---

GGAGATAGCACAATCATACTCCCATGCAGAATAAAACAAATTGTAAATATGTGGCAGGAAGTAGGAAACGCAA
TGTATGCCCCTCCCATCAGCGGACTAATTAGATGTTCATCAAAT---
ATTACAGGGCTGCTATTAACAAGAGATGGTGGGGGTGGTAACAGG----------------------------
----

TCGAACGAGGAGACCTTCAGACCTATAGGAGGAGATATGAGGGACAATTGGAGAAGTGAATTATATAAATATA
AAGTAGTAAAAATTGAACCACTA---GGAATAGCACCCACCAAGGCAAAGAGAAGAGTGGTG-----------
----------------------------CAGAGAGAAAAAAGAGCA---GTGGGA---CTAGGAGCT------
---TTGTTCCTTGGGTTCTTGGGAGCA---GCAGGAAGCACTATG---GGCGCAGCGGCA---
ATGACGCTGACGGTACAG---ACCAGACAA---TTGTTG---TCTGGTATAGTGCAACAGCAGAAC---
AATTTG---CTGAGA---GCTATTGAGGCGCAACAGCATCTGTTGCAACTCACAGTCTGGGGCATC
>JN692443
TTCTACTGTAATACAACAAAACTGTTCAATAGTAGTTGGCAGATTAATGGTACTGGTAGTGGAGAGCCAGATG
AT---

GAGAATGGCACTATCACACTCCCATGCAGAATAAAACAAATTATAAACAGGTGGCAGGAAGTAGGAAAAGCAA
TATATGCTCCTCCCATCAGAGGACTAATTAGATGTTCCTCAAAT---
ATTACAGGGCTGCTACTCACAAGAGATGGTGGTAATAGTAACACG----------------------------
----

AGCAATAATGAGACATTCAGACCTGGAGGAGGAAATATGAAGGACAATTGGAGAAGTGAATTATATAAATATA
AAGTAGTACAAATTGAGCCATTA---GGAGTAGCACCCACTAAGGCAAAAAGAAGAGTGGTG-----------
----------------------------CAGAGAGAAAAAAGAGCA---GTAACG---CTGGGAGCT------
---ATGTTCCTTGGGTTCTTGGGAGCA---GCAGGAAGCACTATG---GGCGCAGCATCG---
GTGACGCTGACGGTACAG---GCCAGACTA---TTATTG---TCTGGTATAGTGCAACAGCAAAAC---
AATTTG---CTGAGG---GCTATTGAGGCGCAACAGCACCTGTTGCAACTCACAGTCTGGGGCATC
>JN692444
TTCTATTGTAATACAACACGGCTGTTTAATAGTACTTGGCCTATTAATGATACAAATATGAATAATACAAATA
ACTCA--

AATAGCACTAGAATTATCACACTCCAATGCAAAATAAAACAAATTATAAACAGGTGGCAGGAAGTAGGAAAAG
CAATGTATGCCCCTCCCATTAAAGGACCAATTAGCTGTTCATCAAAT---
ATTACAGGGCTATTATTAACAAGAGATGGTGGTAATACTAGTAGAACT-------------------------
----

AATGGTACTGAAATCTTCAGACCTGGAGGAGGAAATATGAAGGACAATTGGAGAAGTGAATTATACAAATATA
AAATAGTACAAATTAACCCATTA---GGAGTAGCACCCACCAAGGCAAAGAGAAGAGTGGTG-----------
----------------------------CAGAGAGAGAAAAGAGCA---GTGGGAGCGCTGGGAGCT------
---ATGTTCCTTGGGTTCTTGGGAGCA---GCAGGAAGCACTATG---GGCGCAGCGTCA---
CTGACGCTGACGGTACAG---GCCAGACAA---TTATTG---TCTGGTATAGTGCAACAGCAAAAC---
AATTTG---CTGAAA---GCTATTGAGGCGCAGCAGCATCTGTTGCAACTCACAGTCTGGGGCATC
>JN692445
TTCTACTGTAACACAACAAAGCTGTTTAATAGTGATTGGGACAATGGTACTTGGAATGATACTAAC-------
----

AAGGGAGAAAATATCACAATCCCATGCAGAATAAAACAAATCATAAACAGATGGCAGGAAGTAGGAAAAGCAA
TGTATGCCCCTCCCATCCAAGGACAAATTAGATGTTCATCAAAT---
ATTACAGGGCTGTTATTGACAAGAGATGGTGGCCTTAACACTAGCGAG-------------------------
----

ACTAACAGGACCGAAATCTTCAGGCCTACAGGAGGAGATATGAGGGACAATTGGAGAAGTGAATTATATAAAT
ATAAAGTAGTAAGAATTGAGCCACTA---GGAATAGCACCCACCAAGGCAAAGAGAAGAGTGGTG--------
-------------------------------CAGAGAGAAAAAAGAGCA---GTGGGAACATTAGGAGCT---
------ATGTTCCTTGGGTTCTTGGGAGCA---GCAGGAAGCACTATG---GGCGCAGCGTCA---
ATGACGCTGACGGTACAG---GCCAGACTA---TTATTG---TCTGGTATAGTGCAACAGCAGAAC---
AATTTG---CTGAAG---GCTATTGAGGCGCAACAGCATATGTTGCAACTCACAGTCTGGGGCATT
>JN692447
TTCTATTGTAACACAACACAATTGTTTAATTGGGATGATAATAGTACTTGG----------------------
----

ACAGAGGGCAAAATCATACTCCCATGCAGAATAAAACAAATAGTAAACAGGTGGCAGGAAGTAGGCAAAGCAA
TGTATGCCCCTCCCATCAGAGGATCAATTAACTGTACATCAAAC---
ATTACAGGGTTGCTATTAACAAGAGATGGTGGTAATGGAACCAAC----------------------------
----

AATGAGACCTTCAGACCTGGAGGAGGAGATATGAGGGACAGTTGGAGAAGTGAATTATATAAATATAAAGTAG
TAAAAATTGAACCTTTA---GGAGTAGCACCCACCAAGGCAAAGAGAAGGGTGGTG-----------------
----------------------CAGAGAGAAAAAAGAGCA---GTGGGAGCAATAGGAGCT---------
GTGTTCCTTGGGTTCCTGGGAACA---GCAGGAAGCACTATG---GGCGCAGCGTCA---
CTAACGCTGACGGTACAA---GCCAGACTA---TTACTG---TCTGGTATAGTGCAACAGCAGAAC---
AATCTG---CTGAGA---GCTATTGAGGCGCAACAACATCTGTTGCAACTCACGGTCTGGGGCATT
>EF637056
TTCTACTGTAATACAACAAGGCTGTTTAATGGCACTTGGGATGGTACTAAATGGAATAATACTGTATGG----
----

GGAAATAACACGCTTATACTTCCATGCAGAATAAAACAAATTATAAATAAGTGGCAGGAAGTAGGAAAAGCAA
TGTATGCCCCTCCCATTAGAGGACAAATTAGATGTTCATCAAAT---
ATTACAGGGCTGCTATTAACAAGAGATGGTGGTAACAGTAACAAT----------------------------
----

AAGTCTGAAATTTTCAGACCAGGAGGAGGATATATGAAGGACAATTGGAGAAGTGAATTATATAAATATAAAG
TAGTAAGAATCGAGCCATTA---GGAATAGCACCCAACAGGGCAAGGAGAAGAGTGGTG--------------
-------------------------CAAAGAGAAAAAAGGGCA---GTGGGG---ACCGATAGG---------
ATGTTCCTTGGGTTCTTGGGAGCA---GCAGGAAGCACTATG---GGCGCAGCGTCA---
GTGACGCTGACGGTACAG---GCCAGACAA---TTATTG---TCTGGTATAGTGCAGCAGCAAAAC---
AATTTG---CTGAGA---GCTATTGAGGCGCAACAACATATGTTGCAACTCACAGTCTGGGGCATC
>EF637054
TTCTACTGTGATACAGCGAAGCTGTTCAATAGCACGTGGATTTGGAATAGTACTCGGAATGATTCTAGCACTA
AT---

GAAACCAGCATAATCACACTCCCATGCAAAATAAAACAAATTATAAACAGGTGGCAGGAAGTAGGAAAAGCAA
TGTATGCCCCTCCTATCAGAGGACAAATTGAATGTTCCTCCAAT---
ATTACAGGGTTGCTATTAACCAGAGATGGTGGTAACAGTAGTAAC----------------------------
----

AACGAGACCTTCAGACCTGGAGGAGGAGATATGAGGGACAATTGGAGAAGTGAATTATATAAATATAAGGTAG
TAAAAATTGAACCATTA---GGATTAGCACCCACCAAGGCAAAGAGAAGAGTGGTG-----------------
----------------------CAGAGAGAAAAAAGAGCA---GTGGGAACAATGGGAGCT---------
ATGTTCCTTGGGTTCTTGGGAGCA---GCAGGAAGCACTATG---GGCGCAGCGTCA---
CTGACGCTGACGGTACAG---GCCAGACTA---TTATTG---TCTGGTATAGTGCAACAGCAGAAC---
AATTTG---CTGAGA---GCTATTGAGGCGCAACAGCATATGTTGCAGCTCACAGTCTGGGGCATC
>EF637051
TTCTACTGTAATACAACACAGCTGTTTAATAGTACTTGGGGGTTAAATAGTAATGGCACAAATAGTAATGGCA
CAAAT--

AGTAATGGCACAAATATCACACTCCCATGCAGAATAAAACAAATTATAAACAGGTGGCAGGAAGTAGGAAAAG
CAATGTATGCTCCTCCCATCAGGGGAAATATTAGCTGTACATCAAAT---
ATCACAGGGATACTATTAACAAGGGATGGTGGTACCAACCAGACC----------------------------
----

AGTGAAACCGAAACCTTCAGACCTGGAGGGGGCGATATGAGGGATAATTGGAGAAATGAATTATATAAATATA
AAGTAGTAAAAATTGAGCCATTA---GGAGTAGCACCCACCACGGCAAAGAGAAGAGTGGTG-----------
----------------------------CAGAGAGAAAAAAGAGCA---GTGGGG---ATAGGAGCT------
---TTGTTCCTTGGGTTCTTGGGAGCA---GCGGGAAGCACTATG---GGCGCAGCGTCA---
ATGACGCTGACGGTACAG---GCCAGACTA---TTATTG---TCTGGTATAGTGCAACAGCAGAAC---
AATCTG---CTGAGG---GCTATTGAGGCGCAACAGCATATGTTGCAACTCACAGTCTGGGGCATC
>EF637050
TTCTACTGTAATACAACACAGCTGTTTAATAGTACTTGGTATTCTAATGGTACTTTGAATGATACTGGTACTT
GGAATGGT---
-------------ACTGAG------
GGGTTAAATGACACTATCACACTCCCATGCAAAATAAAACAATTTATAAACAGGTGGCAAGAAGTAGGAAAAG
CAATGTATGCCCCTCCCATCAGAGGACAAATTAGATGCTCATCAAAT---
ATTACAGGGCTGCTATTAACAAGAGATGGAGGGAAAGTGAACGGGTCT-------------------------
----

AACGGGACTGAGATCTTCAGACCTGGAGGAGGAAATATGAGGGACAATTGGAGAAGGGAATTATATAAATATA
AAGTAGTAAAAATTGAACCATTA---GGAGTAGGACCCACCAGAGCAAAAAGAAGAGTGGTG-----------
----------------------------CAGAGAGAGAAAAGAGCA---GTGGGA---ATAGGAGCT------
---CTGTTCCTTGGGTTCTTGGGAGCA---GCAGGAAGCACTATG---GGCGCAGCGTCA---
TTGACGCTGACGGTACAG---GCCAGACAA---TTATTG---TCTGGTATAGTGCAACAGCAAAAC---
AACTTG---CTGATG---GCTATTGAAGCGCAACAGCATCTGTTGAAGCTCACAGTCTGGGGCATC
>EF637048
TTCTACTGTAATACAACACAGTTGTTTAATAGTACTTGGGTTAATGGTGCTTGGATTAATGGTACTTGGTCC-
----

AATAATACTAATATCATACTCCCATGCAGAATAAAACAAATTATAAACAGGTGGCAGGAAGTAGGAAGAGCAA
TGTATGCCCCTCCCATCAGAGGACAAATTAGCTGTTCATCAAAT---
ATTACAGGGCTGCTATTAATGAGAGATGGTGGTAAGGACAACAAT----------------------------
----

ATTTCCGAAATCTTTAGACCTGCAGGAGGAGATATGAGGGACAATTGGAGAAGTGAATTATATAAATATAAAG
TAGTAAAAATTGAGCCATTA---GGAATAGCACCCACCAAGGCAAGGAGAAGGGTGGTG--------------
-------------------------CAGAGAGAAAAAAGAGCA---GTGACA---CTAGGAGCT---------
GTGTTCCTTGGGTTCTTGGGAGCA---GCAGGAAGCACTATG---GGCGCAGCGTCA---
GTGACGCTGACGGTACAG---GCCAGGCTA---TTATTG---TCTGGTATAGTGCAACAGCAAAAC---
AACTTG---CTGAGG---GCTATTGAGGCGCAACAGAGTATGTTGCAACTCACCGTCTGGGGCATC
>EF637047
TTTTACTGTAATACATCAAAGCTGTTTAATAGTACTTGGACTTGGAATGGTACTGGGCCTGAAATGGTAAATA
ACAAC--
-------------------ACA---
GAAAACACAACAGAAATTACACTCCCATGCAGAATAAAACAAATTATAAACAGGTGGCAGGAAGTAGGAAAAG
CAATGTATGCCCCTCCCATCAGTGGAATAATTAGATGTACATCAAAT---
ATTACAGGGCTACTGTTAACAAGAGATGGTGGTAATGTAAGTAAT----------------------------
----

ACCACTGATATCTTCAGACCTGGAGGAGGAGATATGAGGGACAATTGGAGAAGTGAACTATATAAATATAAAG
TAGTAAAAATTGAGCCATTA---GGAGTAGCACCCACCAAGGCAAGGAGAAGAGTGGTG--------------
-------------------------CAGAGAGAAAAAAGAGAA---GTGGGAACATTCGGAGCT---------
ATGTTCCTTGGGTTCTTAGGAGCA---GCAGGAAGCACTATG---GGCGCAGCGTCA---
GTGACGCTGACGGTACAG---GCCAGACAA---TTATTG---TCTGGTATAGTGCAACAGCAGAAC---
AATTTG---CTGAGG---GCTATTGAGGCGCAACAGCATATGTTGCAACTCACAGTCTGGGGCATC
>EF637046
TTCTACTGTAATACAACACCGCTGTTTAATAATAGTAAGTGGGTGAATGGTGGGTGGAATAGTACTATG----
----

AAGAATGGAACTATCACCCTCCCATGCAGAATAAAACAAATTGTAAACAGGTGGCAGGAAGTAGGAAAAGCAA
TGTATGCCCCTCCCATCAGAGGACAAATTAACTGTACATCACAA---
ATTACAGGGCTGCTCTTAACAAGAGATGGTGGTATTAACCAGACC----------------------------
----

AACAATGAAACCTTCAGACCAGCAGGAGGAGATATGAGGGACAATTGGAGAAGTGAATTATATAAATATAAAG
TAGTGAAAATTGAGCCATTA---GGAGTAGCACCCACCAAGGCAAGGAGAAGAGTGGTG-------------
-------------------------CAAAGAGAAAAAAGAGCA---GTGGGAACAATAGGAGCT---------
ATGTTCCTTGGGTTCTTGGGAGCA---GCAGGAAGCACTATG---GGCGCAGCGGCA---
ATGACGCTGACGGTACAG---GCCAGACTA---TTATTG---TCTGGTATAGTGCAACAGCAGAAC---
AACTTG---CTGAGA---GCTATTGAGGCGCAACAGCATATGTTGCAGCTCACAGTCTGGGGCATC
>JN692450
TTCTACTGCAATACAACAGATCTGTTTAATAGTGCTTGGGATTCTACAAATGATACTGACAATCTTACAGATG
ATACTGGC---
-------------ATGGGA------
GGAGATGGTTCAGTTATCATACTCCCATGCAGAATAAAACAAATTATAAACAGATGGCAGGAAGTAGGAAAAG
CAATGTATGCCCCTCCCATCAGAGGACAAATTAGCTGTTCATCAAAT---
ATTACAGGGCTGATATTAACAAGAGATGGTGGTAAAAATGGTAATAAC-------------------------
----

ACAAACGGGACTGAAACCTTCAGACCTGGAGGAGGAGATATGAGGGACAATTGGAGAAGTGAATTATATAAAT
ATAAAGTAGTAAAAATTGAGCCATTA---GGGAGTACACCCACCAAGGCAAAGAGGAGAACGGTG--------
-------------------------------CAGAGAGAAAAAAGAGCA---GTGGGAACGCTAGGAGCT---
------ATGTTCCTTGGGTTCTTGGGAACA---GCAGGAAGCACTATG---GGCGCAGCGTCA---
ATAACGCTGACGGTACAG---GCCAGACAA---TTATTG---TCTGGTATAGTGCAACAGCAGAAC---
AATTTG---CTGAGG---GCTATTGAGGCGCAACAGCATATGTTGCAACTCACAGTCTGGGGCATT
>JN692451
TTCTACTGTAATACAACACCACTGTTTAATAGCACATGGCATGATAATAATAATGTAAGCATATGGCATGTTA
ATAGAACTGTA--
-AATAGTACTGAAGGG---------
TTAAAGGGAGAAAATATCACACTCCAATGCAGAATAAAACAGATTATAAACAGGTGGCAGGAAGTAGGAAAAG
CAATGTATAACCCTCCCATTAGGGGCTTAATTAGATGTTCATCAAAT---
ATTACAGGGATACTATTAATGAGAGATGGTGGTGGTCCTAACAAC----------------------------
----

CGTACTAACGAAACCTTCAGACCAGGAGGAGGAAATATGAAGGACAATTGGAGAAGTGAATTATATAAATACA
AAGTAGTAAAAATTGAGCCATTA---GGACTAGCACCCACAAGGGCAAAGAGAAGAGTGGTG-----------
----------------------------CAGAGAGAAAAAAGAGCA---GTGGGAACAATAGGAGCT------
---ATGTTCCTTGGGTTCTTGGGAGCA---GCAGGAAGCACTATG---GGTGCAGCGGCA---
GTGACGCTGACGGTACAG---GCCAGGCTA---TTGTTG---TCTGGTATAGTGCAACAGCAGAAC---
AATCTG---CTGAAG---GCTATTGAGGCGCAACAACGTATGTTGCAACTCACAGTCTGGGGCATT
>JN692452
TTCTACTGTAATACATCACAACTGTTTAATAGTACTTGGGGGCCTAATGGTACTTGGCTGTCAAATAGTACTG
AGAATAGG---
-------------GCTAAT------
GGGTCTAATGACATTATCATACTCCCATGCAGAATAAAACAAATTATAAACATGTGGCAGGAAGTAGGAAAAG
CAATGTATGCCCCTCCCATCCAAGGACAAATTAACTGTTCATCACAT---
ATAACAGGACTGCTATTAACAAGAGATGGTGGAAATAGTAATAGAACAAAC----------------------
----

GGGTCCGAAACCGAAATCTTCAGACCTGGAGGAGGAGATATGAGGGACAATTGGAGAAGTGAATTATATAAAT
ATAAAGTAGTAAAAATTGAGCCATTA---GGAATAGCACCCACCAAGGCAAGGAGAAGAGTGGTG--------
-------------------------------CAGAGAGAAAGAAGAGCA---ATAACA---CTAGGAGCT---
------ATGTTCCTTGGGTTCTTGGGAGCA---GCAGGAAGCACTATG---GGCGCAGTGTCG---
ATGACGCTGACGGTACAG---GCCAGACAA---TTATTG---TCTGGAATAGTGCAACAGCAGAAC---
AATCTG---CTGAAG---GCTATTGAGGCGCAACAGCATATGTTGAAACTCACAGTCTGGGGCATC
>JN692453
TTCTATTGTAATTCATCACAGCTGTTTAATAGCACTTGGAATAATACCAATTGGAATGATACTAATATACAGG
CA---

AATGACACTGAAAATATCACACTCCCATGCAGGATAAAACAAATTATAAACAGATGGCAGGAAGTAGGAAAAG
CAATGTACGCCCCTCCCATCAGCGGAATAATTAAATGTACATCAAAT---
ATTACAGGGCTACTATTAACAAGAGATGGTGGTAACAAGAATGGGACG-------------------------
----

GAAAGGAATGAGACCTTCAGACCTATAGGAGGAAATATGAAGGACAATTGGAGAAGTGAATTATATAAATATA
AAGTAGTAAAAATTGAGCCATTA---GGAATAGCACCCACCAAGGCAAAGAGAAGAGTGGTG-----------
----------------------------CAGAGAGAAAAAAGAGCA---GTCACA---ATAGGAGCT------
---ATGTTCCTTGGGTTCTTGGGAGCA---GCAGGAAGCACTATG---GGCGCAGCGTCA---
ATGACGCTGACGGTACAG---GCCAGACTA---TTATTG---TCTGGTATAGTGCAACAGCAGAAC---
AATTTG---CTGAGA---GCTATTGAGGCGCAACAGCATATATTGCAACTCACAGTCTGGGGCATT
>JN692457
TTCTACTGTAATACAACAAAGCTGTTTAATAGTACTTGGCATATTAATGGTACTTGGAATGACACTAAA----
----

GGTAATGAGACTATCACACTCCCATGCAGAATAAAACAAATTATAAACAGGTGGCAGGAGGTAGGAAGAGCAA
TGTATGCCCCTCCCATTAGTGGATGGATCAACTGTTCATCAAAT---
ATTACAGGGCTGATATTAACAAGAGATGGTGGTAACCAAAGCGGG----------------------------
----

CCCGACTCCGAAACCTTCAGACCTGGAGGAGGAGATATGAGGGACAATTGGAGAAGTGAATTATATAAATATA
AAGTAGTAAAAATTGAGCCATTA---GGAATAGCACCCACCAAGGCAAAGAGAAGAGTGGTG-----------
----------------------------CAAAGAGAAAAAAGAGAA---CTAACA---CTAGGAGCG------
---TTGTTTCTTGGGTTCTTGGGAGCA---GCAGGAAGCACTATG---GGCGCAGCGTCA---
TTAACGCTGACGGTACAG---GCCAGACAA---TTATTG---TCTGGTATAGTGCAACAGCAGAAC---
AATCTG---CTGAGG---GCTATTGAGGCGCAACAGCATATGTTGCAACTCACAGTCTGGGGCATC
>FJ195090
TTCTACTGTAATACAACACAACTATTTAATTTCACTTGGAATGGTACTGAGATTGGAAATAATAGTACT----
----

AAAGAAAAAAATATCACACTCCCATGTAGAATAAAACAAATTGTAAACAGATGGCAGGAAGTAGGAAAAGCAA
TGTATGCCCCTCCCATCAGTGGATACATTAAATGTGTATCAAAT---
ATTACAGGGCTGCTATTAGCAAGAGATGGTGGTAATACTAGTGAAAAT-------------------------
----

AACAACACCGAGATCTTTAGACCTTTAGGAGGAAATATGAAGGACAATTGGAGAAATGAATTATATAAATATA
AAGTAGTAAAAATTGAGCCATTA---GGAGTAGCACCCACCTTGGCAAAGAGAAGAGTGGTG-----------
----------------------------CAAAGAGAAAAAAGAGCA---GTGGGAACAATAGGAGCT------
---ATGTTCCTTGGGTTCTTGGGAGCA---GCAGGAAGCACTATG---GGCGCAGCATCA---
CTAACGCTGACGGTACAA---GCCAGGCTA---CTACTG---TCTGGTATAGTGCAACAGCAGAAC---
AATCTG---CTGAGG---GCTATTGAGGCGCAACAACATCTGTTGCAACTCACAGTCTGGGGCATC
>JN692459
TTCTACTGTAATACAACACAGCTATTTAAGGATACTTGGAATGGCACTTGGATTGATAATATTTGGAAGGAGA
ATGATACT---
-------------GAAAAG------
ACAGATAACACTACTATCACACTCCCATGCAGAATAAAACAAATTATAAACAGGTGGCAGGACGTAGGAAAAG
CAATGTATGCCCCTCCTATCAGAGGATACATTAACTGTTCATCAAAT---
ATTACAGGGCTAATATTAACAAGAGATGGGGGTGACAGCAACTTGAATAAGACC------
TCGAATGGGACCTGCTTGAAGGGGACC---

TGCTCGAATGAGACTGAGACCTTTAGACCTGTAGGAGGAGATATGAGGGATAATTGGAGAAGTGAATTATATA
AATATAAAGTAGTAAAAATTGAGCCATTA---GGGATAGCACCCACCAAGGCAAAGAGAAGAGTGGTG-----
----------------------------------CAAAGAGAAAAAAGAGCA---
GTGGGAACGATAGGAGCT---------ATGTTCCTTGGGTTCTTGGGAGCA---GCAGGAAGCACTATG---
GGCGCAGCGTCA---CTGACGCTGACGGTACAG---GCCAGACTA---TTATTG---
TCTGGTATAGTGCAACAGCAGAAT---AATTTG---CTGAGG---
GCTATTAAGGCGCAACAACATCTGTTGCAACTCACAGTCTGGGGCATC
>JN692460
TTCTACTGTAATACATCAGGGCTGTTTAATAGTACTTGGCTAAATAATGGTACTAGGAATGATACTAACTCTA
CAAGT--
-------------------GAT---
AATAGCCCAAAGAATATCACACTCCCATGCAGAATAAAACAAATTATAAACAGGTGGCAGGAAACGGGAAGAG
CAGTATATGCCCCTCCCATCAAAGGAATAATTAGCTGTTCATCCAAT---
ATTACAGGGCTGCTATTAACAAGAGATGGTGGCAGCGGTGGGAACGACACC----------------------

----

GGAAACGCCACCGAGGTCTTCAGACCTGGGGGAGGAGATATGAGGGACAATTGGAGAAGTGAATTATATAAAT
ATAAAGTAGTAAGAATTGAACCATTA---GGAATGGCACCCACCGAGGCAAAGAGAAGAGTGGTG-------
------------------------------CAGAGAGAAAAAAGAGCA---GTGGGA---ATAGGAGCT---
------TTGTTCCTTGGGTTCTTGGGAGCA---GCAGGAAGCACTATG---GGCGCAGCGGCA---
ATGACGCTGACGGTACAG---GCCAGACTA---TTATTG---TCTGGTATAGTGCAACAGCAAAAT---
AATTTG---CTGAGA---GCTATTGAAGCACAACAGCATATGTTGCAACTCACAGTCTGGGGCATC
>JN692463
TTCTACTGTAATACGACACAACTGTTTAATAGTACTTGGGGTATGGATGACAAGGATGTGAATGGTACTACA-
----

GGAAATGAAACTATCATACTTCCATGCAGAATAAAACAAATTGTAAACATGTGGCAGGAAGTAGGAAAAGCAA
TGTATGCCCCTCCCATCAGAGGACAAATTAGATGTTCATCAAAT---
ATCACAGGGCTGCTATTAACAAGAGATGGTGGTGGCAATAAAAAC---------------------------
----

AGAAGTAATGAGACCTTCAGACCTGGAGGAGGAGATATGAAGGACAATTGGAGAAGTGAATTATATAAATATA
AAGTAGTAAAAATTGAACCATTA---GGAGTAGCACCCACCAAGGCAAGGAGAAGAGTGGTG-----------
---------------------------CAGAGAGAAAAAAGAGCA---GTGGGAACACTAGGAGCT------
---CTGTTCCTTGGGTTCTTGGGAGCA---GCAGGAAGCACTATG---GGCGCAGCGTCA---
ATGACGCTGACGGTACAG---GCCAGACTA---TTATTG---TCTGGTATAGTGCAACAGCAGAAC---
AATTTG---CTGAGG---GCTATTGAGGCGCAACAGCATATGTTGCAACTCACAGTCTGGGGCATC
>JN692467
TTCTACTGTAATTCAACACAACTGTTTGATACTTGGAATGATACTACCCATGGA------------------
----

GGAAATGACACAATCATACTCCCATGCAGAATAAAACAAATTATAAACAGGTGGCAGGAAGTAGGAAAAGCAA
TGTATGCCCCTCCCATCAAAGGACAAATTAATTGTGTATCAAAT---
ATCACAGGGCTGATATTAACAAGAGATGGTGGTAACACGAATGACCAG------------------------
----

AACGGGTCCGAGATCTTCAGACCTGGAGGAGGAAACATGAAGGACAATTGGAGAAGTGAATTATATAAATATA
AAGTAGTAAAAATTGAACCATTA---GGAGTAGCACCCACCAGGGCAAAAAGAAGAGTGGTG-----------
---------------------------CAGAGAGAAAAAAGAGCA---GTGGGA---CTAGGAGCT------
---TTGTTCCTTGGGTTCTTGGGAGCA---GCAGGAAGCACTATG---GGCGCAGCGTCA---
ATGACGCTGACGGTACAG---GCCAGACAA---TTATTG---TCTGGTATAGTGCAACAGCAAAAC---
AACTTG---CTGAGG---GCTATTGAGGCGCAACAGCGTCTGTTGCAACTCACAGTCTGGGGCATC
>JN692475
TTCTACTGTAATACAACACAGCTGTTTAATAGTACTTGGAAGTTTAATGGTACCTGGAGTATGTCCAATGGGA
AT--

AGTACTGAAAAGAATATCACACTCCCATGCAGAATAAAACAAATTATAAATAAGTGGCAAGAAGTAGGAAAGG
CAATGTATGCCCCTCCCATCAGCGGAATAATTAAATGTTCATCAAAT---
ATTACAGGGCTGCTATTAACAAGAGATGGGGGTGATAAT---------------------------------
----

TCGACAGAAACCTTCAGACCGGGAGGAGGAGATATGAGGGACAATTGGAGAAGTGAATTATATAAATATAAAG
TAATAAGGATTGAGCCATTA---GGAATAGCACCCACCAAGGCAAAGAGAAGAGTGGTG--------------
-------------------------CAGAGAGAAAAAAGAGCA---ATAGGA---ATAGGAGCT---------
GTGTTCCTTGGGTTCTTGGGAGCA---GCAGGAAGCACTATG---GGCGCAGCGTCA---
GTAACGCTGACGGTACAG---GCCAGACAA---TTATTG---TCTGGTATAGTGCAACAGCAGAAC---
AATTTG---CTGAAG---GCTATTGAGGCGCAGCAGCATATGTTGCAACTCACTGTCTGGGGCATC
>FJ195091
TTCTACTGTGATACAACTAGGCTGTTTAATAATGAAACACAG------------------------------
----

AATGACACTATCACACTCCCATGCAAAATAAGACAAATTATAAACAGATGGCAGGAAGTAGGAAAATCAATGT
ATGCCCCTCCCATCAGAGGAGAAATTAAATGTTCATCAAAT---
ATTACAGGGCTGATATTAACAAGAGATGGTGGTAACAGTACCAGC---------------------------

----

ACCATTGAGACCTTCAGACCTGGAGGAGGAAATATGAAGGACAATTGGAGGAGTGAATTATATAAATATAAAG
TAGTAAAAATTGAGCCACTA---GGAATAGCACCCACCAAGGCAAGGAGAAGAGTGGTG--------------
-------------------------CAAAGAGAAAAAAGGGCA---GTGGGAACGTTTGGAGCT---------
ATGTTCCTTGGATTCTTGGGAGCA---GCAGGAAGCACTATG---GGCGCAGCGTCA---
CTGACGCTGACGGTACAG---GCCAGACTA---TTATTG---TCTGGTATAGTGCAACAGCAAAAC---
AATTTG---CTGAGA---GCTATTGAGGCGCAACAACATATGTTGCAACTCACAGTCTGGGGCATC
>FJ195088
TTCTACTGTAATACAACACAGTTATTTAATAGTACTTGGGAGTTTAATAGTACTACGCAGTTGAATATT----
----

ACAGGTAAAACTATCACACTTCCATGCAGAATAAAACAAATTATAAACAGGTGGCAGGAAGTAGGAAAAGCAA
TGTATGCCCCTCCCATCGGAGGACAAATTAGCTGTTCATCAAAA---
ATTACAGGGCTGCTATTAACAAGAGATGGTGGAAATGATGGTAACAAT-------------------------
----

ACTGGGAACGAGACCTTCAGACCTGGAGGAGGAGATATGAGGGACAATTGGAGAAGTGAATTATATAAATATA
AAGTAGTAAAAATTGAACCATTA---GGAGTAGCACCCACCACGGCAAAGAGAAGAGTGGTG-----------
----------------------------CAGAGAGAAAAAAGAGCA---GTGGGAACGATAGGAGCT------
---ATGTTCCTTGGGTTCTTGGGAGCA---GCAGGAAGCACTATG---GGCGCAGCGTCA---
ATAACGCTGACGGTACAG---GCCAGACTA---TTATTG---TCTGGTATAGTGCAGCAGCAGAAC---
AATTTG---CTGAGG---GCTATTGAGGCGCAACAGCATATGTTGCAACTCACAGTCTGGGGCATC
>FJ195089
TTCTACTGTAACACAACAAAGCTGTTTAATAGTATTTGGAAGCATAATAGTACTACTAATATTAAAGGAAATT
CC---

ACTAGTAATGACACTATTACACTCCCATGCAGAATAAAACAAATTATAAACAAGTGGCAGGAAGTAGGAAAAG
CAATGTATGCCCCTCCCATCAGAGGACAAATTAAATGTAAATCAAAT---
ATTACAGGGCTGCTATTAACAAGAGATGGTGGTAACACCAACAATAGTGAA----------------------
----

GCCAACAGCAGTGAAACCTTCAGACCTGGAGGAGGAGATATAAGGGACAATTGGAGAAGTGAATTATATAAAT
ATAAAGTAGTAAGAATTGAGCCATTA---GGAATAGCACCCACCAAGGCAAAGAGAAGAGTGGTG--------
-------------------------------CAGAGAGAAAAAAGAGCA---GTGGGAACATTAGGAGCT---
------GTGTTCCTTGGGTTCTTGGGAACA---GCAGGAAGCACGATG---GGCGCAGCGTCA---
ATAACGCTGACGGTACAG---GCCAGACTA---TTATTG---TCTGGTATAGTGCAACAGCAGAAC---
AATTTG---CTGAGG---GCTATTGAGGCGCAACAGCATATGTTGCAACTCACAGTCTGGGGCATC
>JN692479
TTCTACTGTGATTCAACACAGTTGTTTAATAGTACTTGGCATAATACTACAGACAATAATACTACA-------
----

GACAATAGCAATATCACACTCCCATGCAGAATAAAACAAATTATAAACAGGTGGCAAAAAGTAGGAAAAGCAA
TGTATGCCCCTCCCATCGCAGGACCAATTAGCTGTACATCAAAC---
ATTACAGGGCTGCTATTAACAAGAGATGGTGGAGAAAGCAATGGG----------------------------
----

ACTGAGACCTTCAGACCTGGAGGGGGAGATATGAGGGACAATTGGAGAAGTGAATTATATAAATACAAAGTAG
TAAAAATTGAGCCATTA---GGAATAGCACCCACCAAGGCAAGGAGGAGAGTGGTG-----------------
----------------------CAAAAAGAGAAAAGAGCA---GTGACG---CTGGGAGCT---------
ATGTTCCTTGGGTTCTTGGGAGCA---GCAGGAAGCACTATG---GGCGCAGCGTCA---
CTGACGCTGACGGTACAG---GCCAGACTA---TTGTTG---TCTGGTATAGTGCAACAGCAAAAT---
AATTTG---CTGAAT---GCTATTGAGGCGCAACAACATCTGTTGCAACTCACAGTCTGGGGCATC
>HQ377443
TTCTACTGCAATACATCACAACTGTTTAATAGTACTTGGAATATTGATGGTACTTGGAATGATACTGGA----
----

GGAAAGCAAAATATCACACTCCCATGCAGAATAAGACAAATTGTAAACAGATGGCAAGAAGTAGGGAAGGCAA
TGTATGCCCCTCCCATCCCAGGACAAATTACATGTAGATCAAAT---
ATTACAGGGATACTATTAACAAGAGATGGTGGCGGCAAAAATGAT----------------------------

----

AGCCTTAATGAAACCTTCAGACCTGGAGGAGGAGATATGAGGGACAATTGGAGAAGTGAATTATATAAATATA
AAGTAGTAAGAATTGAACCATTA---GGAATAGCACCCACCAAGGCAAAGAGAAGAGTGGTG-----------
----------------------------CAGAGAGAAAAAAGAGCA---GTGACA---TTAGGAGCT------
---ATGTTCCTTGGGTTCTTGGGAGCA---GCAGGAAGCACTATG---GGCGCAGCGGCA---
ACAACGCTGACGGTACAG---GCCAGACAA---TTATTG---TCTGGTATAGTGCAACAGCAGAAC---
AATCTG---CTGATG---GCTATTGAGGCGCAACAGCATATGTTGCAACTCACAGTCTGGGGCATC
>HQ377487
TTCTACTGCAATACAACACAGCTGTTTAATAGTACCTGGACCCAGGTAAACGGTACTACAGTTAGGAATGGTA
CAAATAGC---
-------------ACTGCT------
AACACAAATGGCACTATCACACTCCCATGCAGAATAAAACAAATTGTAAACACGTGGCAGGCAGTAGGGAAAG
CAATGTATGCCCCTCCCATCAGAGGACAGATTAGCTGTAACTCAAGT---
ATTACAGGGCTGTTATTAACAAGAGATGGTGGTAGTGGTGTCAATGTC-------------------------
----

ACGAACGACACCGAAATCTTCAGACCTGCAGGAGGAGATATGAGGGACAATTGGAGAAGTGAATTATATAAAT
ATAAAGTAGTGAAAATTGAGCCACTA---GGAATAGCACCCACCAAGGCAAGGAGAAGAGTGGTG--------
-------------------------------CAAAGAGAAAAAAGAGCA---GTGGGA---ATAGGAGCT---
------GTGTTCCTTGGGTTCTTGGGAGCA---GCAGGAAGCACTATG---GGCGCAGCGTCA---
GTGACGCTGACGGTACAG---GCCAGACTA---TTATTG---TCTGGTATAGTGCAGCAGCAGAAC---
AATTTG---CTGAGA---GCTATTGAGGCGCAACAGCACATGTTGCAACTCACAGTCTGGGGCATC
>JN687739
TTCTACTGTAATACAACAAAACTGTTTAATAGTATTTGGACTTGGAATGATACACAGCTGTTGAATGATACTG
GAGAG--

CCAGACAATGAAACTATCACACTCCCATGCAGAATAAGACAAATTATAAACAGGTGGCAGGAAGTAGGAAAAG
CAATGTATGCCCCTCCCATCAGAGGACATATTAACTGTTCATCAAAT---
ATTACAGGGCTGCTATTAACAAGGGATGGTGGTAATAACACGAAC----------------------------
----

GGGACCGAGACCTTCAGACCTGGGGGAGGAGATATGAGGGACAATTGGAGAAGTGAATTATATAAATATAAAG
TAGTACAAATTGAGCCAGTA---GGAGTAGCACCCTCCAAGGCAAAGAGAAGAGTGGTG-------------
-------------------------CAGAGAGAGAAAAGAGCA---GTGGGAGCAATAGGAGCT---------
ATGTTCCTTGGGTTCTTGGGAGCA---GCAGGAAGCACTATG---GGCGCAGCGTCA---
CTCACGCTGACGGTACAG---GCCAGACAA---TTATTG---TCTGGTATAGTGCAACAGCAGAAC---
AATATG---CTGAGG---GCTATTGAGGCGCAACAACATCTGTTGCAACTCACAGTCTGGGGCATC
>JF680907
TTCTACTGTAATACAACAAAACTGTTTAATAGTAACCGGACTATAGGGTCGAATGACACTAAAGAG-------
----

AATGACACAGACATCACACTCCCATGCAAAATAAAACAAATTGTAAACATGTGGCAGGAAGTAGGAAAAGCAA
TGTATGCCCCTCCTATCAAGGGAAAAATTGAATGTTCATCAAAT---
ATTACAGGGATACTATTAACAAGAGATGGAGGTGATAATAGAACA----------------------------
----

AACGAGACCTTCAGACCAGCAGGAGGAGATATGAGGGACAATTGGAGAAGTGAATTATATAAATATAAAGTAG
TAAGAATTGAACCATTA---GGAGTAGCACCTACCAGGGCAAGGAGAAGAGTGGTG-----------------
----------------------CAGAGAGAAAAAAGAGCA---GTGGGAACAATAGGAGCT---------
ATGTTCCTTGGGTTCCTAGGAGCA---GCAGGAAGCACTATG---GGCGCAGCGTCA---
GTAACGCTGACGGTACAG---GCCAGACTA---TTATTG---TCTGGTATAGTGCAACAGCAAAAC---
AATTTG---CTGAAG---GCTATTGAGGCGCAACAGCATCTGTTGCAACTCACGGTCTGGGGCATC
>KU749389
TTCTACTGTAACACAACAAAACTGTTTAATAGTAGTTGGCCTCTTAATGGTTCAGCAAATATAACT-------
----

GCAGAAGACCCTATCAGACTCCCATGCAGAATTAGACAAATTGTAAACATGTGGCAGAAAGTAGGAAAAGCAA
TGTATGCCCCTCCCATCGAAGGACAGATTAGATGTTCATCAAAT---
ATTACAGGACTGCTATTAGTAAGAGATGGTGGTAATGGTGGTAATGAC-------------------------

----

ACAAACACGACCGAGATCTTCAGACCTGAAGGAGGAAATATGAAGGACAATTGGAGAAGTGAATTATATAAAT
ATAAAGTAGTAAGAATTGAACCCTTA---GGAATAGCACCCACCAAGGCAAAGAGAAGAGTGGTG--------
--------------------------------CAGAGAGAAAAAAGAGCA---TTGGGA---TTCGGAGCG---
------TTGTTCCTTGGGTTCTTGGGAGCA---GCAGGAAGCACTATG---GGCGCAGCGTCA---
GTGACGCTGACGGTACAG---GCCAGACAA---TTATTG---TCTGGTATAGTGCAACAGCAAAAC---
AACTTG---CTGAGG---GCTATTGAGGCGCAACAACATCTGTTGCAACTCACAGTCTGGGGCATC
>KT427690
TTCTACTGTAATACCACAACGCTATTTAATAGTACGTGGAATAAGAATGATACTAGGAATGATACTACT----
----

AGGAATGATACTATCACACTCCAATGCAGAATAAAACAAATTATAAACAGGTGGCAGGAAGTAGGAAAAGCAA
TATATGCCCCTCCCATCCCAGGACAAATTAAATGCTCATCCAAT---
ATTACAGGGCTGCTACTAACAAGAGATGGTGGTAACAGTACCGAG----------------------------
----

AGTGAAACCTTCAGACCTGGAGGAGGAGATATAAGGGACAATTGGAGAAGTGAATTATATAAATATAAAGTAG
TACAGATTGAGCCATTA---GGAGTAGCACCCACCAGGGCAAAAAGACAAGTGGTG-----------------
----------------------AGGAGAGAAAAAAGAGCA---GTGGGA---CTGGGAGCT---------
TTGTTCCTTGGGTTCTTGGGAGCA---GCAGGAAGCACTATG---GGCGCGGCGTCA---
CTAACGCTGACGGTACAG---GCCAGGCAA---TTATTG---TCTGGGATAGTGCAACAGCAGAAC---
AATCTG---CTGAGG---GCTATTGAAGCGCAACAGCACCTGTTGCAGCTCACAGTCTGGGGCATT
>KT427688
TTCTACTGTAATACATCAAAACTGTTTAATAGTACTGAGAATGGTAATGACACCAATAGTACTGGGCAA----
----

TCTAACAGTACCATCACACTCCAATGCAGAATAAAACAAATTATTAATAGGTGGCAGGAAGTAGGAAAAGCAA
TGTATGCCCCTCCCATCAGAGGAAACATTAGCTGTTCATCAAAC---
ATTACAGGGCTACTATTAACAAGAGATGGGGGTAACACAACTGAC----------------------------
----

AATGAGACCTTCAGGCCAACAGGAGGAGATATGAAGGACAATTGGAGAAGTGAGTTATATAAATATAAAGTAG
TAAGAATTGAACCAATA---GGAGTAGCCCCCACCAAGGCAAAGAGAAGAGTGGTG-----------------
----------------------CAGAGAGAAAAAAGAGCA---GTAACA---CTGGGAGCT---------
GTGTTCCTTGGGTTCTTGGGAGCA---GCAGGAAGCACTATG---GGCGCAGCGTCA---
ATAACGCTGACGGCACAG---GCCAGACAA---TTGTTG---TCTGGTATAGTGCAACAGCAGAGC---
AATCTG---CTGAGA---GCTATTGAGGCGCAACAGCATCTGTTGCAACTCACAGTCTGGGGCATC
>KT427686
TTCTACTGTGATACAACACAGCTGTTTAATAGTACTTGGGATGGAAATAGTACTGAAAACAATAACACAGGA-
----

GGAAATGAAACTATCATACTCCCATGCAGAATAAAACAAATTATAAATACATGGCAGGGAGTAGGAAAAGCAA
TATATGCCCCTCCCATCAGTGGAAAGATTAATTGTTTATCAAAT---
ATTACAGGGCTGCTATTAACAAGAGATGGTGGG--

TCCAATGAGAGTGAGACCTTCAGACCCACAGGAGGAAATATGATGGATAATTGGAGAAGTGAATTATATAAAT
ATAAAGTAGTAAGAATTGAGCCCTTA---GGAGTAGCACCCACCAAGGCAAAGAGAAGAGTGGTG--------
--------------------------------CAAAGAGAAAAAAGAGCT---GTGGGAGCAATAGGAGCT---
------ATGTTCCTTGGGTTCTTGGGAGCA---GCAGGAAGCACTATG---GGCGCAGCGTCG---
CTGACGCTGACGGTACAG---GCCAGACTA---TTATTG---TCTGGTATAGTGCAACAGCAAAAC---
AACTTG---CTGAGG---GCTATTGAGGCGCAACAGCACCTGTTGCAACTCACAGTCTGGGGCATT
>KT427685
TTCTACTGTAATACAACACAACTGTTTAACAGGACCTGGAATGAGACTACCTTGAATGGTACTAACCTTAATG
TAACT--

TCCAATGAAAATATCACAATTCCATGCAGAATAAAACAAATTATAAACAGATGGCAGGAAGTGGGAAAAGCAA
TGTATGCCCCTCCCATCAGAGGACAAATTAGGTGCTCATCAAAT---
ATTACAGGACTGTTATTAACAAGAGATGGTGGCGGAGAGAACAGG----------------------------

----

TCCAATGAAACATTCAGACCTGGAGGAGGAGATATGAGGGACAATTGGAGAAGTGAATTATATAAATATAAAG
TGGTAAAAATTGAGCCATTA---GGAATAGCACCCACCAAGGCAAAGAGAATAGTGGTG--------------
-------------------------CAGAGAGAAAAAAGAGCA---GTAACA---CTAGGAGCT---------
ATGTTCCTTGGGTTCTTGGGAACA---GCAGGAAGCACTATG---GGCGCAGCGTCA---
CTAACGCTGACGGTACAG---GCCAGACTA---TTATTG---TCTGGTATAGTGCAACAGCAGAAC---
AATTTG---CTGAGA---GCTATTGAGGCGCAACAGCATATGTTGAAACTCACAGTCTGGGGCATC
>KT427683
TTCTACTGTAATACATCAGGACTGTTTAATAGTACTTGGTTGGTTAATGGCACCAGCAATACAGAT-------
----

TCAAATGAAACAATCGAACTTCCATGTAGAATAAAACAAATTATAAACAGATGGCAGGAAGTAGGAAAAGCAA
TGTATGCCCCTCCCATCAGAGGACAAATTAGCTGTTCATCAAAC---
ATTACAGGGCTGCTATTAATAAGAGATGGCGGTAATGGGTCTGGG----------------------------
----

AACAATGAGACCTTCAGACCTGGTGGAGGAGATATGAGGGACAATTGGAGAAGTGAATTATATAAATATAAAG
TAGTAAAAATTGAGCCATTG---GGAGTAGCGCCTTCCAAGGCAAAGAGAAGAGTGGTG--------------
-------------------------CAGAGAGAAAAAAGAGCG---GTGGGAACACTAGGAGCC---------
ATGTTCCTTGGGTTCTTGGGAGCA---GCAGGAAGCACTATG---GGCGCAGCGTCA---
ATAACGCTGACGGTACAG---GCCAGACAA---TTACTG---TCTGGTATAGTGCAACAGCAGAAC---
AATCTG---CTGAGA---GCTATTGAGGCGCAACAACACCTGTTGCAACTCACAGTCTGGGGCATC
>KT427682
TTTTACTGTAATACAACACAGCTGTTTAATAGTACTTGGAATGGGACACAGCTGTTTAATAGTACTTGGAATG
GGACGGAATCA---TCA------GAT---

AACTCAAATGACACTATCATACTCCCATGCAGAATAAAACAAATTATAAATAGGTGGCAGGAAGTAGGAAAAG
CAATGTATGCCCCTCCCATCAAAGGAATAATTAGATGTTCATCAAAT---
ATTACAGGGCTACTACTAACAAGAGATGGTGGTAGCGACAGTAGT----------------------------
----

AATGGCACCGAAACCTTCAGGCCTATAGGAGGAGATATGAGGGACAATTGGAGAAGTGAATTATATAAATATA
AAGTAGTAAGAATTGAGCCATTA---GGAATAGCACCCACCAAGGCAAGGAGAAGAGTGGTG-----------
----------------------------CAGAGAGAAAAAAGAGCA---GTGGGC---ATAGGAGCT------
---GTGTTCCTTGGGTTCTTGGGAGCA---GCAGGAAGCACTATG---GGCGCAGCGTCA---
CTGACGCTGACGGTACAA---GCCAGACAA---TTACTG---TCTGGTATAGTGCAACAGCAAAAC---
AATTTG---CTGAGA---GCTATTGAGGCGCAACAACATCTGTTGCAACTCACAGTCTGGGGCATC
>KT427680
TTCTACTGTAAGACAACAAACCTATTTAATAGTAGTTGGTCAAAGAATAATAGTATTTGGTCAAAGGATAATA
CTGAGTCA---

GATAATAATGATACCATCACACTCCAGTGTAGAATAAAGCAAATTATAAACAGGTGGCAGGAAGTAGGAAAAG
CAATGTATGCCCCTCCCATCAGCGGAGTAATTAACTGTGTATCAAAC---
ATTACAGGGCTGATATTAACAAGAGATGGTGGCAACGATGATAATAACGCCACC-------------------
----

AAGAACGGGCCTGAAATCTTCAGACCTGGAGGAGGAAATATGAAGGACAATTGGAGAAGTGAATTATACAAAT
ATAAAGTAGTAAAAATTGAGCCATTA---GGAATAGCACCCACCAAGGCAAAGAGAAGAGTGGTG--------
-------------------------------CAGAAAGAAAAAAGAGCA---GTAACA---CTAGGAGCT---
------GTGTTCCTTGGGTTCTTGGGAGCA---GCAGGAAGCACTATG---GGCGCAGCGTCA---
ATAACGCTGACGGCACAG---GCCAGACAA---TTACTG---TCTGGTATAGTGCAACAGCAGAAC---
AATCTG---CTGAGA---GCTATTGAGGCGCAACAGCATATGTTGCAACTCACGATCTGGGGCATC
>KT427675
TTCTATTGTAATACAACACAACTATTTAATAGTACCTGGGTCTGGAATGCTACTAGGAATGATACTGGAGGAC
TAAATAAC---

ATCACACTTCCATGCAGAATAAAACAAATTATAAACATGTGGCAGGAAGTAGGAAAAGCAATGTATGCCCCTC
CCATCAGAGGACAAATTAGATGTTCATCAAAT---
ATCACAGGGCTACTATTAACAAGAGATGGTGATAATGGGACTAAC----------------------------

----

AATGAGACCTTCAGACCTGGAGGAGGAGATATGAGGGACAATTGGAGAAGTGAATTATATAAATATAAAGTAG
TAAAAATTGAACCATTG---GGAGTAGCACCCACCAAGGCAAGGAGAAGAGTGGTG-----------------
----------------------CAGAGAGAAAAAAGAGCA---GTAGGAGCGCTAGGAGCT---------
ATGTTCCTTGGGTTCTTGGGAGCA---GCAGGAAGCACTATG---GGCGCAGCGTCA---
CTGACGCTGACGGTACAG---GCCAGACGA---TTATTG---TCTGGTATAGTGCAACAGCAAAAC---
AATTTG---CTGAGA---GCTATTGAGGCGCAACAGCATATGTTGCAACTCACAGTCTGGGGCATC
>KT427671
TTCTACTGTAATACAACACCATTATTTAATAAGACTTGGAATGGTACATCGACAGGGGACTGGAAAAT-----
----

GCCACAGGAAGTATCACACTCCATTGCAGGATAAAACAAATCATAAACAGGTGGCAGGAAGTAGGAAAAGCAA
TGTATGCCCCTCCCATCAGAGGAGAGATTAGCTGTTCATCAAAT---
ATTACAGGGCTGCTATTAACAAGAGATGGTGGGTCTAATAATGGGACT-------------------------
----

AAAAATGACAATGAGACCTTCAGACCTGGAGGAGGAGATATGAGGGACAATTGGAGAAGTGAATTATATAAAT
ATAAAGTAGTAAAAATTGAGCCATTA---GGAATAGCACCCACCAGGGCAAGGAGAAGAGTGGTG--------
-------------------------------CAGAGAGAAAAAAGAGCG---GTAACG---CTGGGAGCT---
------ATGTTCCTTGGGTTCTTGGGAGCA---GCAGGAAGCACTATG---GGCGCAGCGTCA---
ATAACGCTGACGGTACAG---GCTAGACAA---TTATTG---TCTGGTATAGTGCAACAGCAGAGC---
AATCTG---CTGAAT---GCTATTGAGGCGCAACAGCGTCTGTTGCAGCTCACAGTCTGGGGCATC
>KT427745
TTCTACTGTAATACATCAAGGCTGTTTAATAGTGCTTGGCTGAATAATGGTACTAGGAATGGTACTAACTCTA
ATTCA--

ACAGATAATAAGATCACACTCCCATGCAGAATAAAACAAATTATAAACAGGTGGCAGGAAACAGGAAAAGCAA
TGTATGCCCCTCCCATCAGAGGAACAATTAGATGTTCATCCAAC---
ATTACAGGGCTGCTATTAACAAGAGATGGTGGCAGTGGTGGGAACGACACCGGG-------------------
----

AACGCCACCGAGGTCTTCAGACCTGGAGGAGGAGATATGAGGGACAATTGGAGAAGTGAATTATATAAATATA
AAGTAGTAAGAATTGAACCATTA---GGAATAGCACCCACCGAGGCAAAGAGAAGAGTGGTG-----------
----------------------------CAGAGAGAAAAAAGAGCA---GTAGGGGGACTAGGAGCT------
---TTGTTCCTTGGGTTCTTGGGAGCA---GCAGGAAGCACTATG---GGCGCAGCGGCA---
ATGACGCTGACGGTACAG---GCCAGAATA---TTATTG---TCTGGTATAGTGCAACAGCAAAAT---
AATTTG---CTGAGA---GCTATTGAAGCACAACAGCATATGTTGCAACTCACAGTCTGGGGCATC
>KJ849788
TTCTACTGTAATACAACACCACTGTTTAATAGTACTTGGCATAATAATGGTACT-------------------
----
-AGGCCTAATAGTACT---------
TGGTCAAATAACACAATCACACTCCAATGCAGAATAAAACAAATTGTAAACCTGTGGCAGCAAGTAGGAAAAG
CAATGTATGCCCCTCCCATTAGTGGATATATTAATTGTTCATCAAAT---
ATTACAGGGCTACTATTAACAAGAGATGGTAGT--

ATCAGCAGTAGCAGTAAT---
AACACTGAAGTCTTTAGGCCTAGAGGAGGAGATATGAAGGACAATTGGAGAAGTGAATTATATAAATATAAAG
TAGTACAACTTGAACCACTA---GGAGTAGCACCAACCAAGGCAAAGAGGAGAACGGTG--------------
-------------------------CAGAGAGAAAAAAGAGCA---GTGGGA---CTGGGAGCT---------
CTGTTCATTGGGTTCTTGGGAGCA---GCAGGAAGCACTATG---GGCGCAGCGTCA---
GTGACGCTGACGGTACAA---GCCAGACAA---TTATTG---TCTGGTATAGTGCAGCAGCAAAAC---
AATTTG---CTGAGG---GCTATTGAGGCGCAACAGCATCTGTTGCAACTCACAGTCTGGGGCATT
>KT427740
TTCTACTGTAATACAACACAGCTGTTTAATAGTACTTGGAATGAAACACAGCTGGATGATTGGAATAAAAGTA
AT---

GATACAAGTAATATCACACTCCCATGCAGAATAAGACAAATTGTAAATAAGTGGCAGGAAGTAGGAAAAGCAA
TGTATGCCCCTCCCATCCAAGGACAAATTAAATGTACATCAAAT---
ATTACAGGGATGCTATTGACAAGAGATGGGGGTAGTAAAAATGACACG-------------------------

----

AACGGGAGCGAAACCTTCAGACCTGCAGGAGGAGATATGAGGGACAATTGGAGAAGTGAATTATATAAATACA
AAGTGGTAAAAATTGAACCATTA---GGAATAGCACCCACCAAGGCAAGGAGAATAGTGGTG-----------
----------------------------CAAAGAGAAAAAAGAGCA---GTGGGA---ATAGGAGCT------
---GTGTTCCTTGGGTTCTTGGGAGCA---GCGGGAAGCACTATG---GGCGCAGCGTCA---
ATAGCGCTGACGGCACAG---GCCAGACAA---TTATTG---TCTGGTATAGTGCAACAGCAAAAC---
AATTTG---CTGAGG---GCTATTGAGGCGCAACAGCATATGTTGCAACTCACAGTTTGGGGCATC
>KT427739
TTCTACTGTAATACATCAGGGCTGTTTAATAGTACTTGGCTAAATAATGGTACTAGGAATGATACTAACTCTA
CAAGTGAT---

ACTAACTCTAATATCACACTCCCATGCAGAATAAAACAAATTATAAACAGGTGGCAGGAAACGGGGAAAGCAA
TATATGCCCCTCCCATCAGAGGAATGATTAGCTGTTCATCCAAC---
ATTACAGGGCTGCTATTAACAAGGGATGGTGGCAGCGGTGGGAACGCCACCGGG-------------------
----

AACGCCACCGAGATCTTCAGACCTGGAGGGGGAGATATGAGAGACAATTGGAGAAGTGAATTATACAAATATA
AAGTAGTAAGAATTGAACCATTA---GGAATAGCACCCACTGAGGCAAAGAGAAGAGTGGTG-----------
----------------------------CAAAGAGAAAAAAGAGCA---GTGGGA---ATAGGAGCT------
---TTGTTCCTTGGGTTCTTGGGAACA---GCAGGAAGCACTATG---GGCGCAGCGGCA---
ATGACGCTGACGGTACAG---GCCAGACTA---TTATTG---TCTGGTATAGTGCAACAGCAAAAT---
AATTTG---CTGAGA---GCTATTGAGGCACAACAGCATATATTGCAACTCACAGTCTGGGGCATC
>KT427738
TTCTACTGTAATACAACACAACTGTTTAATAGTACCTGGCAAAGTAATGGTACCTGGAATAATAGTAATAATA
AA---

GAGAATGACACATACACACTCCCATGCAGAATAAAACAAATTATAAACAGGTGGCAGGAAGTAGGAAGAGCAA
TATATGCCCCTCCCATCAGTGGACAAATTAACTGTTCATCAAAT---
ATTACAGGGATAATATTAACAAGAGATGGTGGTAGGAACGAAAGT----------------------------
----

AATGGGACAGAAATCTTCAGACCTGGAGGAGGAGATATGAGGGACAATTGGAGAAGTGAATTATATAAATATA
AAGTAGTAAAAATTGAGCCATTA---GGAGTAGCACCCACCAAGGCAAGGAGAAGAGTGGTG-----------
----------------------------CAGAGAGAAAAAAGAGCA---GTAACA---CTAGGAGCT------
---GTGTTCATTGGGTTCTTGGGAGCA---GCAGGAAGCACTATG---GGCGCAGCGTCA---
GTGACGCTGACGGTACAG---GCCAGACAA---TTATTG---TCTGGTATAGTGCAACAGCAGAGC---
AATTTG---CTGAGG---GCTATTGAGGCGCAACACCACATGTTGCAACTCACAGTCTGGGGCATT
>KT427735
TTCTACTGTAATACAGCACAGTTGTTTAATTCTGATTGGAATTCTACTTGGACTTGGAGTGATAATACAAATA
ATACTGGGTTT--

GAAAACAAAACTATCACACTCCTGTGTAGAATAAAACAAATCATAAACAGGTGGCAGGGAGTAGGAAAAGCAA
TGTACGCCCCTCCAATCCAAGGAAATATTAGCTGTTCATCAAAT---
ATTACAGGGCTGCTATTAACAAGAGATGGAGGCAACAATACCGGT----------------------------
----

AACGATACCGAGACCTTCAGACCTGGAGGAGGAAATATGAAGGACAATTGGAGAAGTGAATTATATAAATACA
AAGTAGTAAGAATTAAGCCATTA---GGAGTAGCACCCACCAAGGCAAAGAGAAGAGTGGTG-----------
----------------------------CAGAGAGAAAAAAGAGCA---GTGGGAACATTAGGAGCT------
---GTGTTCCTTGGGTTCTTGGGAGCA---GCAGGAAGCACTATG---GGCGCAGCGTCA---
CTGACGCTGACGGTACAG---GCCAGACAA---TTATTG---TCTGGTATAGTGCAACAGCAGAAC---
AATCTG---CTGAGG---GCTATTGAGGCGCAACAGCACCTGTTGCAACTCACAGTCTGGGGCATC
>KT427734
TTCTACTGTAATACAACACAACTGTTTAATAGTACCTGGAATAATGGTACTGAAAAG----------------
----

ACCAATGGCACAATAACACTCCCATGCAGAATAAAACAAATTGTGAACAAGTGGCAGGAAGTAGGAAAAGCAA
TGTATGCCCCTCCCATCAGGGGATACATTAACTGTTCATCAAAT---
ATTACAGGGCTGCTATTAACAAGAGATGGTGGCAACAATAATAAC----------------------------

----

AGCCAGAACGAGACCTTCAGACCAGGAGGAGGAGATATGAGGGACAATTGGAGAAGTGAATTATATAAATATA
AAGTAGTAAGGATTGAACCATTA---GGAATAGCACCCACCAGGGCAAAGAGAAGAGTGGTG-----------
------------------------------CAAAGAGAAAAAAGAGCA---GTGGGAGCACTAGGAGCT------
---ATGTTCCTTGGGTTCTTGGGAGCA---GCAGGAAGCACTATG---GGCGCAGCGTCA---
ATAACGCTGACGGTACAG---GCCAGACAA---TTATTG---TCTGGTATAGTGCAACAGCAAAAT---
AATTTG---CTGAGA---GCTATTGAGGCGCAGCAGCATCTGCTGCAACTCACGGTCTGGGGCATT
>KT427733
TTCTACTGTAATACAACAAAGCTGTTTAATAGTACTTGGGGGTCTAATGGTACTTGGAAGGGTACAAATGAC-
----

AGTAATAGTAATATCACAATACAATGCAGAATAAAACAAATTATAAACAGGTGGCAAGAAGTAGGAAAAGCAA
TGTATGCCCCTCCCGTCAAAGGACCAATCAAATGTTCATCAAAT---
ATTACAGGACTACTATTAACAAGAGATGGTAGTAGTAGTAGTAAT----------------------------
----

GATACTGAGACCTTCAGACCTGGAGGAGGAAATATGAAGGACAATTGGAGAAGTGAATTATATAAATACAAAG
TAGTAAAAATTGAGCCATTA---GGAGTAGCACCCACCAGGGCAAGGAGAAGAGTGGTG--------------
---------------------------CAGAGAGAAAGAAGAGCG---GTAACA---CTAGGAGCT---------
ATGTTCCTTGGGTTCTTGGGAGCA---GCAGGAAGCACTATG---GGCGCAGCGTCA---
ATGACGCTGACGGTACAG---GCCAGACTA---TTATTG---TCTGGTATAGTGCAGCAGCAGAGC---
AATTTG---CTGAGG---GCTATTGAGGCGCAACAGCATCTGTTGCAACTCACAGTCTGGGGCATC
>KJ849803
TTCTACTGTAATACATCAGGGCTGTTTAATAGTACCTGGTATGGGAATGAG----------------------
----
-ACTGCAGAGGCAAAT---------
AGTACAAATACTAATATCACACTCCCATGTAGAATAAAGCAAATTATAAACAGGTGGCAGGAAGTAGGAAAAG
CAATGTATGCCCCTCCCATCAGGGGACAAATAAGATGTTCATCAAAT---
ATTACAGGGATACTACTAACAAGAGATGGTGGT--

-
GATAACAATACCGAGATCGAGACCTTCAGACCTGGAGGGGGAGATATGAGGGACAATTGGAGAAGTGAATTAT
ATAAATATAAAGTAGTAGAAATTGAGCCATTA---GGAATAGCACCTACCAAGGCAAAAAGAAGAGTGGTG--
--CAGAGAGAAAAAAGAGCA---ATAACA---
CTGGGAGCT---------ATGTTCCTTGGGTTCTTGGGAGCA---GCAGGAAGCACTATG---
GGCGCAGCGTCA---GTAACGCTGACGGTACAG---GCCAGACAA---TTATTG---
TCTGGTATAGTGCAACAGCAGAAC---AATCTG---CTGAAG---
GCTATTGAGGCGCAACAACATCTGTTGCAACTCACAGTCTGGGGCATC
>KT427730
TTCTACTGTAATACAACACAGCTGTTTAATAGTACTTGGGTGGGGAATAGTACTATAAATGATACTGCC----
----

AAGACAGAAACTATCACACTCCCATGCAGAATAAAACAAATTATAAACAGATGGCAGGAAGTAGGAAAAGCAA
TGTATGCCCCTCCCATCAGGGGACCAATTAACTGTTCATCAAAT---
ATTACAGGGATACTATTACTAAGAGATGGTGGTGTTAATACCACG----------------------------
----

AATGAGACCTTCAGACCTGGAGGAGGAGATATGAGGGACAATTGGAGAAGTGAATTATATAAATATAAAATAG
TAAAAATTGAGCCATTA---GGAGTAGCACCCACCAAGGCAAAAAGAAGAGTGGTG-----------------
------------------------CAGAGAGAAAAAAGAGCA---GTAACA---TTAGGAGCT---------
ATGTTCCTTGGGTTCTTGGGAGCA---GCAGGAAGCACTATG---GGCGCAGCGTCA---
CTGACGCTGACGGTACAG---GCCAGACAA---TTATTG---TCTGGTATAGTGCAACAGCAGAGC---
AATATG---CTGAGA---GCTATTGAGGCGCAACAGCATCTGTTGCAACTCACAGTCTGGGGCATT
>KT427728
TTCTATTGTAATACAACAAAACTGTTTAATAGTACGTGGCCGTGGCCGCATAACAGTACTATAAATAGGACTG
AAGAG--

TCAGATAATAGCACAATCACACTCCCATGCAAAATAAAACAAATTATAAACAGATGGCAGGAAATAGGAAAAG
CAATGTATGCCCCTCCCATCAGGGGACAAATTAGATGTTCATCAAAT---

ATTACAGGGCTGCTATTAACAAGAGATGGTGGAAATGTTGGTAAT----------------------------
----

AAGACCAGTGAGACCTTCAGACCTGGAGGAGGAGATATGAGGGACAATTGGAGAAGTGAGTTATATAAATATA
AAGTAGTACAAATTGAACCATTA---GGAGTAGCACCCACCAAGGCAAAGAGAAGAGTGGTG-----------
----------------------------CAGAGAGAAAAAAGAGCA---TTGGGAACGCTGGGAGCT------
---ATGTTCCTTGGGTTCTTGGGAGCA---GCAGGAAGCACTATG---GGCGCAGCGTCA---
ATGACGCTGACGGTACAG---GCCAGGCAA---TTATTG---TCTGGTATAGTGCAACAGCAGAAC---
AATCTG---CTGAGG---GCTATTGAGGCGCAACAACATATGTTGCAACTCACAGTCTGGGGCATC
>KT427727
TTCTACTGTAATACATCAAAACTGTTTAATAGTACTTGGACTAATGATACTTTGGATACCAGGTTCAATATG-
----

ACAGATGATAATATCACACTCCCATGCAGAATAAGACAAATTATAAACAGGTGGCAGGAAGTAGGAAAAGCAA
TGTATGCCCCTCCAATCAGTGGACCAATTAGATGTTCATCAAAT---
ATTACAGGGCTATTATTAACAAGAGATGGTGGTAATAGTAATAGC----------------------------
----

ACGAATGAGACCTTCAGACCTGGAGGAGGAGATATGAGGGACAATTGGAGAAGTGAATTATATAAATATAAAG
TGGTACAAATTGAGCCATTA---GGAGTAGCACCCACCAGGGCAAAGAGAAGAGTGGTG--------------
-------------------------CAGAGAGAAAAAAGAGAA---ATAACAACACTAGGAGTT---------
ATGTTCCTTGGGTTCTTGGGAGCA---GCAGGAAGCACTATG---GGCGCAGCGTCA---
ATGACGCTGACGGTACAG---GCCAGACAA---TTATTG---TCTGGTATAGTGCAACAGCAGAAC---
AATTTG---CTGAGA---GCTATTGAGGCGCAACAGCATCTGTTGCAACTCACAGTCTGGGGCATC
>KT427725
TTCTACTGCAATACATCCAGACTGTTTAATAGTACATGGAATAGCACTGAACTGGAGGAGAGGAATAAC----
----

ACTGATAAAATTATCACACTCCCATGCAAAATAAAACAAATTATAAATATGTGGCAAGAAGTAGGAAAAGCAA
TGTATGCCCCTCCTATCAGAGGACAGCTTAACTGTTCATCAAAT---
ATTACAGGGCTGATACTAACAAGAGATGGTGGCCAAGGGAACGAG----------------------------
----

ACCAATAATGAAACATTCAGACCTGGAGGAGGAGATATGAGGGACAATTGGAGAAGTGAATTATATAAATACA
AAGTAGTAAAAATTGAACCACTA---GGAATAGCACCCACCAAGGCAAAGAGAAGAGTGGTG-----------
----------------------------CAGAGAGAAAAAAGAGCC---GTGGGAACGCTAGGAGCT------
---ATGTTCCTTGGGTTCTTGGGAGCA---GCAGGAAGCACTATG---GGCGCAGCGTCA---
ATGACGCTGACGGTACAG---GCCAGGCAA---TTATTG---TCTGGTATAGTGCAGCAGCAGAGC---
AATCTG---CTGAGA---GCTATTGAGGCGCAGCAGCATATGTTGCAACTCACAGTCTGGGGCATC
>KT427724
TTCTACTGTAATACAACACAACTGTTTACAACACAACTGTTTAATAGGACATGGGAGTTTAATAGTACTGGAC
CAAT---

GCTACAGGTAATATCACACTCCCATGCAGAATAAAACAAATTATAAATAGGTGGCAAGAAGTAGGAAAAGCAA
TGTATGCCCCTCCCATCAGCGGATATATTAACTGTTCATCAAAT---
ATTACAGGACTCCTATTAATAAGAGATGGGGGCAATGAAACCGAT----------------------------
----

GGGAGCGAGACCTTTAGACCTATAGGAGGAAATATGAGGGATAATTGGAGAAGTGAATTATATAAATATAAAG
TAGTAAGAATTGAGCCATTA---GGAGTAGCACCCACCAAGGCAAGGCGAAGAGTGGTG--------------
-------------------------CAGAGAGAAAAAAGAGCC---ATAACA---ATGGGAGCT---------
ATGTTCCTTGGGTTCTTGGGAGCA---GCAGGAAGCACTATG---GGCGCAGCGTCA---
ATGGCGCTGACGGTACAG---GCCAGATTA---TTATTA---TCTGGTATAGTGCAACAGCAGAGC---
AATTTG---CTGAGG---GCTATTGAGGCGCAACAACATCTGTTGCAACTCACAGTCTGGGGCATC
>KT427723
TTCTACTGTAATACATCAGGGCTGTTTAATAGTACTTGGATACATAATGGTACTCGGCTAAATAATGGTACT-
----

AGGAATGGTACTATCACACTCCCATGCAGAATAAAACAAATTATAAACAGGTGGCAGGAAACGGAAAAGCAA
TGTATGCCCCTCCCATCAGAGGAATAATTAGATGTTCATCCAAC---

ATTACAGGGCTGCTATTAACAAGAGATGGTGGTGGGAATGACACC----------------------------
----

GACAACTCCACCGAGGTCTTCAGACCCGGAGGAGGAGATATGAGGGACAATTGGAGAAGTGAATTATATAAAT
ATAAAGTAGTAAAAATTGAACCATTA---GGAATAGCACCCAACAAGGCAAGGAGAAGAGTGGTG--------
-------------------------------CAGAGAGAAAAAAGAGCA---GTGGGAACGCTAGGAGCT---
------CTGTTCCTTGGGTTCTTGGGAACA---GCAGGAAGCACTATG---GGCGCAGCGTCA---
GTCACGCTGACGGTACAG---GCCAGACTA---TTATTG---TCTGGTATAGTGCAACAGCAGAAC---
AATATG---CTGAGA---GCTATTGAGGCGCAACAGCACATGTTGCAACTCACAGTCTGGGGCATC
>KT427720
TTCTATTGTAATACAACAAAGCTATTTAATGATACATGGTTGAATGAAAATGAAACT----------------
----

AGCACTAGCAATATCACACTCCCATGCAGAATAAAACAAATTATAAACAGATGGCAGGAAGTAGGAAGAGCAA
TGTATGCCCCTCCCATCCAAGGACAAATTAGCTGTTCATCAAAT---
ATTACAGGGCTGCTATTAACAAGAGATGGTGGTTCAGGAGCCACT----------------------------
----

AATGAAACCTTCAGACCTGTGGGAGGAGATATGAGAGACAACTGGAGAAGTGAATTATATAAATATAAAGTAG
TAAAAATTGAGCCATTA---GGACTGGCACCCACCAAGTCAAGGAGAGTAGTGGTG-----------------
----------------------CAAAGAGAAAAAAGAGCA---GTGGGA---ATGGGAGCT---------
ATGTTCCTTGGGTTCTTGGGAGCA---GCAGGAAGCACTATG---GGCGCAGCGGCA---
GTGACGCTGACGGTACAG---GCCAGACAA---TTATTG---TCTGGTATAGTGCAACAGCAAAAC---
AATTTG---CTGAGA---GCTATTGAGGCGCAACAGCACATGTTGCAACTCACAGTCTGGGGCATC
>KT427716
TTCTACTGTAATACAACAGGGCTGTTTAATAGTACGTGGAACTCTACTACAAAGATAAATACT----------
----

ACAAGGTCAAATATCATACTCCCATGCAGAATAAAACAAATTATAAACAGGTGGCAGGAAGTAAGAAAAGCAA
TGTATAACCCTCCCATCAAAGGAAGAATTAAGTGTTTATCAAAT---
ATTACAGAGATACTATTAACAAGAGATAGTAGTAGTAACAATGACACG-------------------------
----

AACAGGACCGAGATCTTCAGACCAGGAGGAGGAGATATGAGGGACAATTGGAGAAGTGAATTATATAAATATA
AAGTAGTAAGAATTAAGCCATTA---AGAATAGCACCCACCAAGGCAAAGAGAAGAGTGGTG-----------
----------------------------CAGAGAGAAAAAAGAGCA---GTAACA---CTAGGAGCT------
---ATGTTCCTTGGGTTCTTGGGAACA---GCAGGAAGCACTATG---GGCGCAGCGGCA---
CTGACGCTGACGGTACAG---GCCAGGCTA---TTATTG---TCTGGTATAGTGCAACAGCAGAAC---
AATTTG---CTGAGA---GCTATTGAGGCGCAACAGCATATGTTGCAACTCACAGTCTGGGGCATC
>KJ849767
TTCTACTGTAATACAACACAACTGTTTAATAGCAGGTGGAATATGACTGCTAAT-------------------
----
TGGACTATGAATGTTACT---------
ACAGGGACAGATAATATCACACTCCCATGCAGAATAAAACAAATTATAAATAGATGGCAGGAAGTAGGAAAAG
CAATGTATGCCCCTCCCATCGCAGGACAAATCAAATGCTTATCAAAT---
ATTACAGGGCTACTATTAACAAGAGATGGTAGT--

-
AACCAGACTAATGGGACAGAGACCTTCAGACCAGGAGGAGGAGATATGAGGGACAATTGGAGAAGTGAATTAT
ATAAATATAAAGTAGTAAAAATTGAGCCATTA---GGAGTAGCACCCACCAAGGCAAAGAGAAGAGTGGTG--
-------------------------------------CAGAGAGAAAGAAGAGCA---ATAACA---
ATGGGAGCT---------ATGTTCCTTGGGTTCTTGGGAGCA---GCAGGAAGCACTATG---
GGCGCAGCGTCA---TTGACGCTGACGGTACAG---GCCAGACAA---TTATTG---
TCTGGTATAGTGCAACAGCAGAAC---AATTTG---CTGAGG---
GCTATTGAGGCGCAACAGCACCTGTTGCAACTCACAGTCTGGGGCATC
>KT427847
TTCTACTGTAATACAACACAGCTGTTTAATAATGCTTGGAGTGAGACTGAGCTGTTTAATAGTACTGAGAATG
AT---

GCAAACAGCACACTCACACTCCCATGCAGAATAAAACAAATTATAAACAGGTGGCAGGAAGTTGGAAAAGCAA

TGTATGCCCCTCCCATCAAAGGACCAATTAGGTGTGTATCAAAC---
ATTACAGGGCTACTATTAACAAGAGATGGTGGTAACAATTCTTCT----------------------------
----

GAAACCAATTCTTCTGAAATCTTCAGACCTGGAGGAGGAGATATGAGGGACAATTGGAGAAGTGAATTATATA
AATATAAAGTAGTAAAAATTGAGCCATTA---GGAGTAGCACCCACCAAGGCAAAGAGAAGAGTGGTG-----
----------------------------------CAGAGAGAAAAAAGAGCA---
GTGGGGACAATAGGAGCT---------ATGTTCCTTGGGTTCTTGGGAGCA---GCAGGAAGCACTATG---
GGCGCAGCGTCA---ATGACGCTGACGGTACAA---GCTAGACAA---TTATTG---
TCTGGTATAGTGCAACAGCAGAAC---AATCTG---CTGAGG---
GCTATTGAGGCGCAACAGCATATGTTGCAACTCACAGTCTGGGGCATC
>KT427853
TTCTATTGTAATACAACACAGCTGTTTAATAGTACTTGGATGGGGAATGATACCATATCAAATACTACAGGA-
----

TTAAATGACACTATCACACTCCCATGCAGAATAAAACAAATTATAAACAGGTGGCAGGAAGTAGGAAAAGCAA
TATATGCCCCTCCCATCAGCGGACAGATTAACTGTTTATCAAAT---
ATTACAGGGCTGCTATTAACAAGAGATGGTGGTGATAACACCACC----------------------------
----

ATCGAGACCTTCAGACCTGGAGGAGGAAATATGAAGGACAATTGGAGAAGTGAATTATATAAATATAAAGTAG
TAAAAATTGAGCCAGTA---GGAATAGCGCCCACCAAGGCAAAGAGAAGAGTGGTG-----------------
----------------------CAGAGAGAAAGAAGAGCA---GTAACG---CTAGGAGCT---------
ATGTTCCTTGGGTTCTTGGGAGCA---GCAGGAAGCACTATG---GGCGCAGCGTCA---
ATGACGCTGACGGTACAG---GCCAGACAA---CTATTG---TCTGGTATAGTGCAACAGCAGAAC---
AATTTG---CTGAGG---GCTATTGAGGCGCAACAGCATCTGTTGCAACTCACAGTATGGGGCATC
>KT427709
TTCTATTGTAATACAACAGACCTGTTTAATAGTGCTTGGCTGTCTAACAGTACTGGGAATGAGACT-------
----

GGACAGTCAAATATTACACTCCCATGCAGAATAAAACAAATTATAAACATGTGGCAGGAGGTAGGAAAAGCAA
TGTATGCCCCCCCCATCCAAGGACAAATTAGGTGTTCATCAAAT---
GTTACAGGGCTAATATTAGTAAGAGATGGTGGTGACAATACCAAC----------------------------
----

GGGACCGAGACCTTCAGACCTGGAGGAGGAGATATGAGGGACAATTGGAGAAGTAAATTATATAAATATAAAG
TAGTAAAAATTGAGCCAGTA---GGATTAGCACCCACCAAGGCAAGGAGAAGAGTGGTG--------------
-------------------------CAGAGAAAAAAAAGAGCG---GTGGGAACGCTGGGAGCT---------
GTGTTCCTTGGGTTCTTGGGAGCA---GCAGGAAGCACTATG---GGCGCAGCGTCA---
ATGACACTGACGGTACAG---GCCAGACAA---TTATTG---TCTGGTATAGTGCAGCAGCAGAAC---
AATTTG---CTGAAG---GCTATTGAGGCGCAACACCATCTGTTGCAACTCACAGTCTGGGGCATC
>KT427702
TTCTACTGTAATTCAACACAACTGTTTAATAGTACTTGGGGGGTTAATGGTACTTGGAATGATACTAGG----
----

TCAAATGACACTATCACACTCCCATGCAGAATAAAACAAATTATAAACATGTGGCAGGAAGTAGGAAAAGCAA
TGTATGCCCCTCCCATCAGTGGACAAATTAACTGTTCATCAAAT---
ATTACAGGGTTGCTATTAACAAGAGATGGAGGTAATAACGGGACG----------------------------
----

AACGAGACCTTCAGACCTGGAGGAGGAAATATGAGGGACAATTGGAGAAGTGAATTATATAAATATAAAGTAG
TAAAAATTGAACCATTA---GGAATAGCACCCACCAAGGCAAGAAGAAGAGTGGTG-----------------
----------------------CAAAGAGAAAAAAGAGCA---ATAGGA---TTCGGAGCT---------
ATGTTCCTTGGGTTCTTGGGAGCA---GCAGGAAGCACTATG---GGCGCAGCGTCG---
GTGACGCTGACGGTACAG---GCCAGACAA---TTATTG---TCTGGTATAGTGCAACAGCAGAGC---
AATTTG---CTGAAG---GCTATTGAGGCGCAACAGCATCTGTTGCAACTCACAGTCTGGGGCATC
>KT427700
TTCTACTGTAATACAACAAAGCTGTTTAATAGTACCTGGCCTTTGAATGATACTACTTTGAATGATACTACAG
GG---

TCAGATAAAAATATCACTCTCCCATGCAGAATAAAACAAATTATAAACAGGTGGCAAGAAGTAGGAAAAGCAA
TGTATGCCCCTCCCATCAGAGGACAAATTAGGTGTTCATCAAAT---
ATTACAGGTCTATTATTAACAAGAGATGGTGGTAATGTCAACGGGACC-------------------------
----

ACCACCACCGAGACCTTCAGACCTGGAGGAGGAGATATGAGGGACAATTGGAGAAGTAAATTATATAAATATA
AAGTAGTGAAAATTGAGCCATTA---GGAGTAGCACCTACCAAGGCAAAGAGAAGAGTGGTG-----------
----------------------------CAGAGGGAAAAAAGAGCA---ATAACAGGGCTAGGAGCT------
---GTGTTCCTTGGGTTCTTGGGAGCA---GCAGGAAGCACTATG---GGCGCAGCGTCG---
GTAACGCTGACGGTACAG---GCCAGACAA---TTATTG---TCTGGCATAGTGCAGCAGCAGAAC---
AATCTG---CTGAGG---GCTATTGAGGCGCAACAGCATATGTTGCAACTCACAGTCTGGGGCATC
>KT427699
TTCTACTGTAATACAAACCGACTGTTTAACATAAATGAAACTACTCTAAATAAAACTATAAATGAAACTAGA-
----

GTCAATGAGACAATCACACTCCCATGTAGAATAAAGCAAATTATAAACATGTGGCAGACAGTAGGAAAAGCAA
TGTATGCCCCTCCCATCAACGGACAAATTAATTGTACATCAAAT---
ATTACAGGGCTACTATTAACAAGAGATGGTGGTAAAACTAATGAGACC-------------------------
----

AATGATACCGAGGTCTTCAGACCTGGAGGAGGAGATATGAGGGATAATTGGAGAAGTGAATTATATAAATATA
AAGTAGTAAAAATTGAACCATTA---GGAATAGCACCTACCAAGGCCAGGAGAAGAGTGGTG-----------
----------------------------CAGAGAGAAAAAAGAGCA---GTGGGAATGCTAGGAGCT------
---ATGTTCCTTGGGTTCTTGGGAGCA---GCAGGAAGCACTATG---GGCGCAGCGTCA---
ATGGCGCTGACGGTACAG---ACCAGACAA---TTATTG---TCTGGTATAGTGCAACAGCAGAAT---
AATCTG---CTGAGG---GCTATTGAGGCGCAACAACATATGTTGCAACTCACAGTCTGGGGCATC
>KT427692
TTCTACTGTAATACATCAGGGCTGTTTAATAGTACTTGGCTAACTAATAGTACTAGGAATAGTACTAACCAA-
----

GGCAATAGTACTATCACACTCCCATGCAGAATAAAACAAATTATAAACAGGTGGCAGGAAACGAGAAAAGCAA
TGTATGCCCCTCCCATCAGAGGAATAATTAGATGTTCATCCAAC---
ATTACAGGGCTGCTATTAACAAGAGATGGTAGTAGTAAGAACGGCACC-------------------------
----

GACAACTCCACCGAGGTCTTCAGACCTAGAAGAGGAGATATGAAGGACAATTGGAGAAGTGAATTATATAAAT
ATAAAGTAGTAAAAATTGAACCATTA---AGAATAGCACCCAACAAGGCAAGGAGAAGAGTGGTG--------
-------------------------------CAGAGAGAAAAAAGAGCA---GTGACA---CTAGGAGCT---
------TTGTTCCTTGGGTTCTTGGGAACA---GCAGGAAGCACTATG---GGCGCAGCGGCA---
CTGACGCTGACGGTACAG---GCCAGAACA---TTACTG---TCTGGTATAGTGCAACAGCAAAAT---
AATTTG---CTGAGA---GCTATTGAAGCACAACAGCATATGTTGCAACTCACAGTCTGGGGCATC
>KT427792
TTCTACTGCAGTACAACAAAGCTGTTTAATAGTACTTGGTCTGAGAATAATACCGCAGAGAATAATACCGCAG
GG---

GTAAATGAAACTATCACACTCCCATGCAGAATAAAACAAATCATAAACAGATGGCAGGGAGTAGGAAGAGCAA
TGTATGCCCCTCCCATCAGAGGATATATTAACTGTTCAGCAAAT---
ATTACAGGGATGCTATTAACAAGAGATGGTGGCAATAATAGCAGC----------------------------
----

CCCAACGAGACTTTCAGACCTGAAGGGGGAAATATGAAGGACAATTGGAGAAGTGAGTTATATAAATATAAGG
TAGTCCAAATTGAGCCATTA---GGAATAGCACCCACCAAGGCGAAGAGAAGAGTGGTG--------------
-------------------------CAGAAAGAAAAAAGAGCA---GTGACA---CTGGGAGCT---------
ATGTTCCTTGGGTTCTTGGGAGCA---GCAGGAAGCACTATG---GGCGCAGCGTCA---
CTGACGCTGACGGTACAG---GCCAGACAA---TTATTG---TCTGGTATAGTGCAACAGCAGAAC---
AATCTG---CTGAGG---GCTATTGAGGCGCAACAGCATCTGTTGCAACTCACAGTCTGGGGCATT
>KT427791
TTCTACTGTAATACAACACAGCTATTTAATAGTACCTGGTATGCTAATGGTACTACTGAAAATAGTACT----
----

GGAAACGAAACTATCACACTCCCATGCAGAATAAAACAAATTATAAACATGTGGCAAGAAGTAGGAAGAGCAA
TGTATGCCCCTCCTATCAAGGGACAAATTAACTGTACATCAAAT---
ATTACAGGGCTGATATTAACAAGAGATGGTGGTAAAAACGACACA----------------------------
----

GAGACAGAGACCTTCAGACCTGGAGGAGGAAATATGAAGGACAATTGGAGAAGTGAATTATATAAATATAAAG
TAGTAAGAATTGAACCATTA---GGAGTAGCACCCACCAAGGCAAAGAGAAGAGTGGTG--------------
-------------------------CAGAGAGAAAAAAGAGCA---GTGGGAACACTGGGAGCC---------
ATGTTCCTTGGGTTCTTGGGAGCA---GCAGGAAGCACTATG---GGCGCAGCGTCA---
ATAACGCTGACGGTACAG---GCCAGACAA---TTATTG---TCTGGTATAGTGCAACAGCAGAAC---
AATTTG---CTGAGG---GCTATTGAGGCGCAACAACATCTGTTGCAACTCACAGTCTGGGGCATC
>KT427787
TTCTACTGTGACACAGCAGAACTGTTTAATAGTACTTGGAATAATACTGAAGAGGCAGATAACATGGGA----
----

GGAAATGATACCATCATACTCCCATGTAGAATAAGACAAATTATAAACAGGTGGCAGGAAGTAGGAAAAGCAA
TGTATGCCCCTCCCATCCAAGGACATATTAATTGCTTATCAAAT---
ATTACAGGGCTACTATTAATGAGAGATGGTGGTAATAACGTGAGC----------------------------
----

GGGCCCGAGATCTTCAGACCTGGAGGAGGAGATATGAGGGACAATTGGAGAAGTGAATTATATAAATATAAAG
TAGTAAAAATTAAACCATTA---GGAATAGCACCCACTAAAGCAAAGAGAAGAGTGGTG--------------
-------------------------CAGAGAGAAAAAAGAGCA---GTGGGA---ATAGGAGCT---------
GTGTTCCTTGGGTTCTTGGGAGCA---GCAGGAAGCACTATG---GGCGCAGCGTCA---
ATGACGCTGACGGTACAA---GCCAGACTA---TTATTG---TCTGGTATAGTGCAGCAGCAAAAC---
AATTTG---CTGCAG---GCTATTGAGGCGCAACAGCGTCTGTTGCAACTCACAGTCTGGGGCATC
>KT427786
TTCTACTGTAATACAACACGACTGTTTAATAGTACTTGGCCCGCTGGGGACACTTGGAATGATACTGAA----
----

AACACAGCCAATATCACACTCCCATGTAGAATAAAACAAATTATAAACAGGTGGCAGGCAGTAGGAAAAGCAA
TGTATGCCCCTCCCATCAGAGGACAAATTAGCTGCTCATCAAAC---
ATTACAGGGTTGCTATTAACAAGAGATGGTGGTAATGATAATGCTACA-------------------------
----

AGGAACGAGACCTTCAGACCGGGAGGAGGAGATATGAGGGACAATTGGAGAAGTGAATTATATAAATATAAAG
TAGTAAAAATTGAACCATTG---GGAATAGCACCCACCAAGGCAAAGAGAAGAGTGGTG--------------
-------------------------CAGAGAGAAAAAAGAGCA---GTGGGAGTACTAGGAGCT---------
ACGTTCCTTGGGTTCTTGGGAGCA---GCAGGAAGCACTATG---GGCGCAGCGTCA---
ATGACGCTGACGGTACAG---GCCAGACTA---TTATTG---TCTGGTATAGTGCAACAGCAGAAC---
AATTTG---CTGAAG---GCTATTGAGGCGCAACAGCATCTGTTGCAACTCACAGTCTGGGGCATT
>KT427784
TTTTACTGTAATACAACAAAGCTGTTTAATAGTAGGTGGAATATTAGTGGAGAGTCAAATAACACA-------
----

GAAGGAGAAACTATCATACTCCCATGCAGAATAAAACAAATTATAAACAGATGGCAGGAAGTAGGAAAAGCAA
TGTATGCCCCTCCAATCAGCGGATATATTAGATGTTCATCAAAT---
ATTACAGGGCTACTATTAACAAGAGATGGTGGTAATAATGGAACA----------------------------
----

AATGAGACCTTCAGACCAGGAGGAGGAGACATAAAGGATAATTGGAGAAGTGAATTATATAAATATAAAGTAG
TAAAAATTGAACCATTA---GGAGTAGCACCCACCAAGGCAAAGAGAAGAGTGGTG-----------------
----------------------CAGAGAGAAAAAAGAGCA---GTGGGA---ATAGGAGCT---------
ATGTTCCTTGGGTTCTTGGGAGCA---GCAGGAAGCACTATG---GGCGCAGCGTCA---
CTAACGCTGACGGTACAG---GCCAGACTA---TTATTG---TCTGGTATAGTGCAACAGCAGAAC---
AATTTG---CTGAGA---GCTATTGAGGCGCAACAGCATCTGTTGCAACTCACAGTCTGGGGCATC
>KT427781
TTCTACTGTAATACAACACAGCTGTTTAATAGTATTTGGCCTTGGAATGATACTGATTCAAATAGTACTAAAG
GG---

TCAAATAGCAATATCACACTCCCATGCAGAATAAAACAAATCATAAACAGGTGGCAGGAAGTAGGAAAAGCAA
TGTATGCCCCTCCCATCAGAGGAGAAATCAGGTGTACATCAAAT---
ATTACAGGACTACTATTAACAAGAGATGGTGGTTCTGAGGACAACACT-------------------------
----

TCTATATCTGAGATCTTCAGACCTGGAGGAGGAGATATGAGGGATAATTGGAGAAGTGAATTATATAAATATA
AAGTAGTACAAGTTGAGCCATTA---GGAATAGCACCCACCAGGGCAAGGAGAAGAGTGGTG-----------
----------------------------CAGAGAGAAAGAAGAGCA---GTAACA---ATGGGAGCT------
---CTGTTCCTTGGGTTCTTGGGAGCA---GCAGGAAGCACTATG---GGCGCAGCGTCA---
ATGACGCTGACGGTACAG---GCCAGACAA---TTATTG---TCTGGTATAGTGCAGCAGCAGAGC---
AATTTG---CTGAAG---GCTATTGAGGCGCAACAGCACCTGTTGCAACTCACAGTCTGGGGCATC
>KT427656
TTCTACTGTAATTCATCACAGCTGTTTAATAGTACTTGGTATACTAATGGTACTTGGAATGCTAATGGCACTG
AAAAAAAT---

TTCAATGACACCATTACGCTCCCATGCAGAATAAAACAAATTATAAACAGGTGGCAGGACATAGGAAGAGCAA
TATATGCTCCTCCCATCAGAGGATACATTAATTGTTCATCAAAT---
ATTACAGGAATGATATTAACAAGAGATGGGAGTGACAATGACTCT----------------------------
----

AGTCCTGAAATCTTCAGACCTGGAGGAGGAAATATGAAGGACAATTGGAGAAGTGAATTATATAAATATAAAG
TAGTAAAATTGAGCCATTA---GGAATAGCACCCACCAAGGCAAAGAGAAGAGTGGTG---------------
-------------------------CAGAGAGAAAAAAGAGCA---GTGGGAACGTTGGGAGCT---------
ATGTTCCTTGGGTTCTTGGGAACA---GCCGGAAGCACTATG---GGCGCAGCGGCA---
GTAACGCTGACGGTACAG---GCCAGACAA---TTATTG---TCTGGTATAGTGCAACAGCAGAAC---
AATCTG---CTGAGG---GCTATTGAGGCGCAACAGCATATGTTGCAACTCACAGTTTGGGGCATC
>KT427650
TTCTACTGTAATACATCACAGTTGTTTAACAGTACCTGGAGTCTTAACAGTACTTGGAATGGTACTAATACT-
----

AACAATGGCACTATCACACTCCCATGCAGAATAAAACAAATCATAAACAGGTGGCAGGAAGTAGGAAGATCAA
TGTATGCCCCTCCCATCAAAGGACTAATTAATTGTTTGTCAAAT---
ATTACAGGGCTCCTGTTAACAAGAGATGGTAGTAATGGGTCCGAT----------------------------
----

CCCGAAATCTTCAGACCTGGAGGAGGAGATATGAGGGACAATTGGAGAAGTGAATTATATAAATATAAAATAG
TAAAAATCGAGCCATTA---GGAATAGCACCCACCAAGGCAAAGAGAAGAGTGGTG-----------------
----------------------CAGAGAGAGAAAAGAGGA---GTAACA---TTAGGAGCT---------
GTGTTCCTTGGGTTCTTGGGAACG---GCAGGAAGCACTATG---GGCGCAGCGGCC---
ACGACGCTGACGGTACAG---GCCAGACAA---TTATTG---TCTGGTATAGTGCAACAGCAGAAC---
AATTTG---CTGAGA---GCTATTGAGGCGCAACAGCATATGTTGCAACTCACAGTCTGGGGCATT
>KJ849811
TTCTACTGTAATACAACAGAACTATTTAATTCCACTTGGAATGGTACTGCTGGT-------------------
----
GAGAACAATAGTACTAGT---------
ACTGAGGGAGGAAACATCACACTCCCATGTAGAATAAAACAAATTGTAAACAGATGGCAGGAAGTAGGAAAAT
CAATGTATGCCCCTCCCATCAGTGGATACATCAAGTGTGTATCAAAT---
ATTACAGGGCTGCTATTAACAAGAGATGGTGGT--

ATTACTAATGAAACTAAC---
CACAACGAGACTTTTAGACCTTTAGGAGGAAATATGAAGGACAATTGGAGAAATGAATTATATAAATATAAAG
TAGTAAAATTGAGCCATTA---GGAGTAGCACCCACCAAGGCAAAGAGAAGAGTGGTG---------------
-------------------------CAAAGAGAAAAAAGAGCA---GTGGGAGCAATAGGAGCT---------
ATGTTCCTTGGGATCTTGGGAGCA---GCAGGAAGCACTATG---GGCGCAGCGTCA---
ATAACGCTGACGGTACAA---GCCAGGCTA---CTCTTG---TCTGGTATAGTGCAACAGCAGAAC---
AATCTG---CTGAGG---GCTATTGAGGCGCAACAACATCTGTTGCAACTCACAGTCTGGGGCATC
>KJ849825
TTCTATTGTAATACAACAAAACTGTTTAATAATACTTGGAATAGTACTGCTGGAAATTGGACT----------
---GGAAAT---
TGGACTGTGACTAAAGGGCCA---------------

AGTGGTACTGATATTATCATACTCCCATGCAGAATGAAACAAATCATAAACAGGTGGCAGGAAGTAGGAAAAG
CAATGTATGCTCCTCCCATCAGTGGAAGAATTAGCTGTTTATCAAAT---
ATCACAGGGCTGATATTAACAAGAGATGGTGGT--

AATAACAAGAACAGGAAC---
CGGACCGACAATTTCAGACCTGAAGGAGGAGATATGAGGAATAATTGGAGAAGTGAATTATATAAATACAAAG
TAGTAAAAATTGAGCCATTA---GGAATAGCACCCACCAAGGCAAGGAGAAGAGTGGTG--------------
-------------------------CAGAGAGAAAAAAGAGCA---ATGGGAACACTAGGAGCT---------
GTGTTCCTTGGGTTCTTGGGAACA---GCAGGAAGCACTATG---GGCGCAGCGTCA---
CTGACGCTGACGGTACAG---GCCAGACAA---CTATTG---ACTGGTATAGTGCAACAGCAGAAC---
AATTTG---CTGAGG---GCTATTGAGGCGCAACAGCATCTGTTGCAGCTCACAGTCTGGGGCATC
>KT427775
TTCTACTGTAATACAACACAGCTGTTTAATAGTACTTGGCTGAATAGTACTTGGAATGATAATGAGACAAAAT
CA---

GACAATAGCACTATCACTCTTCCATGCAGAATAAAACAAATTATAAATAGATGGCAGGAAGTAGGAAAAGCAA
TGTATGCCCCTCCCATCGCAGGAAAAATTAGATGTTCATCAAAT---
ATTACAGGGCTGCTATTAACAAGAGATGGAGGCAATACCAGTAAT----------------------------
----

GGCCACAGTGAGATCTTCAGGCCTGCAGGAGGAGATATGAGGGACAATTGGAGAAGTGAATTATATAAATATA
AAGTAGTAAAAATTGAGCCTTTA---GGAATAGCACCCACCAAGGCAAGGAGAAGAGTGGTG-----------
-----------------------------CAGAGAGAAAAAAGAGCA---GTGGGA---ATAGGAGCT------
---GTGTTCCTTGGGTTCTTGGGAGCA---GCAGGAAGCACTATG---GGCGCAGCGTCA---
GTGACGCTGACGGTACAG---GCCAGGCAA---TTATTG---TCTGGTATAGTGCAACAGCAGAAC---
AATTTG---CTGAAG---GCTATTGAGGCGCAACAGCATCTGTTGCAACTCACAGTCTGGGGCATC
>KT427769
TTTTACTGTAATACAACAAAGCTGTTTAATAGGACTTGGATTAGAAATGGGGGATTTTGGAATACTACTGAAG
AG---

GAAAATAACACTGAAATCACACTCCCATGCAAAATAAAGCAAATCATAAACAGGTGGCAGGAAGTAGGAAAAT
CGATATATGCCCCTCCCATCAGAGGACAAATTAGATGTGTATCAAAT---
ATTACAGGGCTGCTATTAACAAGAGATGGTGGTATTAACAATACTACT-------------------------
----

AACGAGACCGAGATCTTTAGACCTGCAGGGGGAGATATGAGGGACAATTGGAGAAGTGAATTATATAAATATA
AAGTAGTAAAAATTGAACCATTA---GGAGTAGCACCCACCAAGGCAAAGAGAAGAGTGGTG-----------
-----------------------------CAGAGAGAAAAAAGGGCA---GTGGGAGTAATAGGAGCT------
---ATGTTCCTTGGGTTCCTGGGAGCA---GCAGGAAGCACTATG---GGCGCAGCGTCA---
GTGACGCTGACGGTACAG---GCCAGACAA---TTACTG---TCTGGTATAGTGCAACAGCAGAAC---
AATCTG---CTGAGG---GCTATTGAGGCGCAACAACATCTGTTGCAACTCACAGTCTGGGGCATC
>KT427767
TTCTACTGTAATACAACACAACTGTTTAATAGTACTTGGTTTAATAGTACATGGACTACTGCAGAGTCAAATA
ACACT--

GAAGGAAGTGACTCAATCACCCTCCCATGCAGAATAAAGCAAGTAGTAAACATGTGGCAGGAAGTAGGAAAAG
CAATGTATGCCCCTCCCATCAGCGGACGAATTAACTGTTCATCAAAC---
ATTACAGGGTTGCTATTAACAAGAGATGGTGGTAATGGGAGTGAA----------------------------
----

CCTATAGAGATCTTCAGACCTGGAGGAGGAGATATGAGGGACAATTGGAGGAGTGAATTATATAAATATAAAG
TAGTAAAAATAGAACCATTA---GGAATAGCACCTACCAAGGCAAAGAGAAGAGTGGTG--------------
-------------------------CAGAGAGAAAAGAGAGCA---GTGACA---CTAGGAGCT---------
GTGTTCCTTGGGTTCTTGGCAGCA---GCAGGAAGCACTATG---GGCGCAGCGTCA---
ATGACGCTGACGGTACAG---GCCAGACTA---TTATTG---TCTGGTATAGTGCAACAGCAGAAC---
AATTTG---CTGAGG---GCTATTGAGGCGCAACAACATCTGTTGCAACTCACAGTCTGGGGCATC
>KT427764
TTCTACTGTAATACATCACAGCTGTTTAACAGTACTTGGATTAATGATACTAATACAGAA-------------
----

AATAACACCCATATCACCCTCCCATGCAGAATAAGACAAATCATAAACAGGTGGCAAGAAGTAGGAAAAGCAA
TGTATGCCCCTCCCATTAGCGGACAGATTAAATGTTCATCAAAT---
ATTACAGGGTTGCTATTAACAAGAGATGGTGGTGTCAATGGTACC-------------------------
----

AATGGAACTGAGACCTTCAGACCTGGAGGAGGAGATATGAGGGACAATTGGAGAAGTGAATTATATAAATATA
AAGTAGTAAAAATTGAACCATTA---GGAATAGCACCCACCAAGGCAAGGAGAAGAGTGGTG-----------
---------------------------CAAAGAGAAAAAAGAGCG---GTGGGAGCACTAGGAGCT------
---ATGTTCCTTGGATTCTTGGGCACA---GCAGGAAGCACTATG---GGCGCAGCGTCA---
ATAACGCTGACGGTACAG---GCCAGACAA---TTATTG---TCTGGTATAGTGCAACAGCAGAAC---
AATCTG---CTGAGA---GCTATTGAGGCGCAACAGCACTTGTTGCAACTCACAGTCTGGGGCATC
>KT427761
TTCTACTGTAATACAACAAAACTGTTTAATAGTACTTGGAAAAATAATAATGTTAGTTGGAATGTTGCGGCT-
----

GAAAATGACACTATCACACTCCCATGTAGAATCAAACAAATTATAAACCTGTGGCAGGAAGTGGGAAAAGCAA
TGTATGCCCCTCCCATCCAAGGACAAATTAACTGTTCATCAAAT---
ATTACAGGGCTGTTATTAATAAGAGATGGTGGTATTAACAACCAGACC----------------------
----

AAGGACACCGAGACCTTCAGACCTGGAGGAGGAGATATGAGGGACAATTGGAGAAGTGAATTATATAAATATA
AAGTAGTAAAAATTGAACCAGTA---GGAATAGCACCCACCAAGGCAAAGAGAAGAGTGGTG-----------
---------------------------CAAAGAGAAAAAAGAGCA---GTGGGAATGGTAGGAGCT------
---ATGTTCCTTGGGTTCTTGGGAGCA---GCAGGAAGCACTATG---GGCGCAGCGTCA---
ATAACGCTGACGGTACAG---GCCAGACAA---CTATTG---TCTGGTATAGTGCAACAGCAGAAC---
AATATG---CTGAGA---GCTATTGAGGCGCAACAGCATCTGTTGCAACTCACAGTCTGGGGCATC
>KT427757
TTCTACTGCAATACAACACAGCTGTTTAATAGTACTTGGCCTGAGAATAATATTAGTACTACAGGGGTAAAT-
----

GTAAGTGATACTATCACAATTCCATGCAGAATAAAACAATTCATAAACAGGTGGCAGGCAGTAGGAAGAGCAA
TGTATGCCCCTCCCATCAGAGGATATATTAACTGTTCATCAAAT---
ATTACAGGGCTGCTATTAACAAGAGATGGTGGTAGTAACAGCTCC-------------------------
----

TTAAATGAGACTTTCAGACCTGAAGGGGGAAATATAAAGGACAATTGGAGAAGTGAGTTATATAAATATAAGG
TAGTCCAAATTGAGCCATTA---GGAGTAGCACCCACCAAGGCAAAGAGAAGAGTGGTG-------------
------------------------CAGAGAGAAAGAAGAGCA---GTGACA---CTAGGAGCT---------
ATGTTCCTTGGGTTCTTGGGAGCA---GCAGGAAGCACTATG---GGCGCAGCGTCA---
CTGACGCTGACGGTACAG---GCCAGACAA---TTGTTG---TCTGGTATAGTGCAACAGCAGAAC---
AATCTG---CTGAGG---GCTATTGAGGCGCAACAGCATCTGTTGCAACTCACAGTCTGGGGCATT
>KT427756
TTCTACTGCAATACATCAAATCTGTTTAATAAGTCTTGGACTAATAGTTCTTGGTCTGGTAATGAAGGGTCAA
ATAAC---
------------------AAT---
ACAGAAAATGACACAATCACACTCCAATGCAGAATAAAACAAATTATAAACATGTGGCAGAAAGTAGGAAAAG
CAATGTATGCCCCTCCTATCCGAGGAACAATTAAATGTTCATCAAAT---
ATCACAGGGCTGCTATTAACAAGAGATGGTGGCAATAACAGCACC-------------------------
----

AATGAGACCTTCAGACCTGGAGGAGGAGATATGAGAGATAATTGGAGAAGTGAATTATATAAATATAAAGTAG
TAAAAATTGAACCATTA---GGAGTAGCACCCACCAGGGCAAAAAGAAGAGTGGTG-----------------
---------------------CAGAAAGAAAAAAGAGCA---GTGGGA---ATAGGAGCT---------
TTATTCCTTGGGTTCTTGTCGGCA---GCAGGAAGCACTATG---GGCGCAGCGTCA---
ATGACGCTGACGGTACAG---GCCAGACAA---TTATTG---ACTGGTATAGTGCAACAGCAAAAC---
AATTTG---CTGAGA---GCTATTGAGGCGCAACAGCATATGTTGCAACTCACAGTCTGGGGCATA
>KT427755
TTCTACTGTAATACATCAAAACTGTTTAATAGTACGTGGAACAGTGGGAATTTAGCAGTAGATAATAGTACTG
AT--

GATAATGGGACTATCATACTCCCATGTAGAATAAAACAAATCATAAACATGTGGCAGGAAGTAGGAAAAGCAA
TGTATGCCCCTCCTATTCGAGGAATAATTAAATGTTTATCAAAT---
ATCACAGGGATAATTTTAACAAGAGATGGTGGTAATAACAATAATAGCAACGAG-------------------
----

ACCACCGAAATCTTCAGACCTGGAGGAGGAGACATGAGGGACAATTGGAGAAGTGAATTATATAAATATAAAG
TAGTAAAAATTGAACCATTA---GGAGTAGCACCCACTGAGGCAAGGAGGAGAGTGGTG--------------
-------------------------CAGAGAGAAAAAAGAGCA---ATAACG---ATGGGAGCT---------
ATGTTCCTTGGGTTCTTGGGAGCA---GCAGGAAGCACTATG---GGCGCAGCATCA---
ATAACGCTGACGGTACAG---GCCAGACAA---TTATTG---TCTGGAAAAGGGCGACAGCAGAAC---
AATCTG---CTGAGG---GCTATTGAGGCGCAACAGCATCTGTTGCAACTCACGGTCTGGGGCATT
>KT427754
TTCTACTGTAATACATCACAGCTGTTTAACAGTACTTGGCCTTGGAACAGTGCTGGGAATGAGAGT-------
----

GATAGTACTAATATCACACTCCCATGCAGAATAAGACAAATCATAAACAGATGGCAGGAAGTAGGAAAAGCAA
TGTATGCCCCTCCCATCAAAGGACCAATTAGATGCTCATCAAAT---
ATTACAGGGATACTATTAACAAGAGATGGTGGTAATACCAACCAG----------------------------
----

ACCACGGAGATCTTTAGACCGGGAGGAGGAAATATGAAGGACAATTGGAGAAGTGAATTATATAAATATAAAG
TAGTAAAAATTGAGCCATTA---GGAGTAGCACCCACCAAGGCAAGGAGAAGAGTGGTG--------------
-------------------------CAGAGAGACAAAAGAGCA---GTAACA---CTAGGAGCT---------
ATGTTCCTTGGGTTCTTGGGAGCA---GCAGGAAGCACTATG---GGCGCAGCGTCA---
ATGACGCTGACGGTACAG---GCCAGACTA---TTATTG---TCTGGTATAGTGCAACAGCAGAAC---
AATTTG---CTGAAG---GCTATTGAGGCGCAACAGCGTCTGTTGCAACTCACAGTCTGGGGCATC
>KT427750
TTTTACTGTAATACAACACTGCTATTTAACAGAACTTGGAAGTTTAATGGCACTTGGAATAGTGTGAAGAATA
GTACTGGA---

GAGTCAAACAATATTACACTCCCATGCAGAATAAAACAAATCATAAACAGGTGGCAGGAAGTAGGAAAAGCAA
TGTATGCCCCTCCCATCAGAGGACTAATTAACTGTTTATCAAAT---
ATTACAGGGCTACTATTAACAAGAGATGGTGGTAATGCGAATAGC----------------------------
----

AGCAATGAGACCTTCAGACCGGGAGGGGGAGATATGAGGGACAATTGGAGAAGTGAATTATATAAATATAAAG
TAGTAAAAATTGAACCATTA---GGAGTAGCACCCACCAAGGCAAAGAGAAGAGTGGTG--------------
-------------------------CAGAGAGAAAAAAGAGCA---ATAGGAACAATAGGAGCT---------
ATGTTCCTTGGGTTCTTGGGAGCA---GCAGGAAGCACTATG---GGCGCAGCGTCA---
GTGACGCTGACGGTACAG---GCCAGACAA---TTATTG---TCTGGTATAGTGCAACAGCAGAGC---
AATCTG---CTGAGA---GCTATTGAGGCGCAACAACATATGTTGCAACTCACAGTCTGGGGCATC
>KT427830
TTCTACTGTGATACAACTAAACTGTTTAACAGTACTTGGAATAATAGCATTGATACTTGGGAGAATAGCACT-
----

AATACTGGGAATATCACACTCCCATGTAGAATAAAACAAATTATAAACATGTGGCAGGAAGTAGGAAAAGCAA
TGTACGCCCCTCCTATCAGAGGACAAATTAGATGCTCATCAAAC---
ATTACAGGGCTGCTACTAGTGAGAGATGGTGGTACAAATAACACA----------------------------
----

AACAGCTCCGAGATTTTCAGACCTGGAGGAGGAGACATGAGGGACAATTGGAGAAGTGAATTATACAGGTATA
AAGTGGTAAAAATTGAACCATTA---GGAGTAGCACCCACCAAGGCAAAGAGAAGAGTGGTG-----------
----------------------------CAGAGAGAAAAAAGAGCA---GTGGGAACGCTGGGAGCT------
---ATGTTCCTTGGGTTCTTGGGAGCA---GCAGGAAGCACTATG---GGCGCAGCGTCA---
ATAACGCTGACGGCACAG---GCCAGGCAA---TTATTG---TCTGGTATAGTGCAGCAGCAGAAC---
AATTTG---CTGAGA---GCTATTGAGGCGCAACAGCATCTGTTGCAACTCACAGTCTGGGGCATC
>KT427829
TTCTACTGTAATACAACACAACTGTTTAATAGTACTTGGCATAATAATGGTAGTACTTGGTATACTAATGATA
GTATTTGG---
TATACTAATGGTACTGAA---------

GAACTAAATGACACTATCACCCTTCCATGCAGAATAAAACAAATTGTAAACATGTGGCAGGAAGTAGGAAAAG
CAATGTATGCCCCTCCCATCAAAGGACAAATTAGATGTTCATCAAAT---
ATCACAGGGCTGCTATTAACAAGAGATGGTGGTAATACTAATGGCACA-------------------------
----

AATGGTACTGAGATTTTCAGACCTGGAGGAGGAGATATGAGGGACAATTGGAGAAGTGAATTATATAAATATA
AAGTAATAAAAATTGAACCATTA---GGAATAGCACCCACCAAGGCAAGGAGAAGAGTGGTG-----------
----------------------------CAGAGAGAAAAAAGAGCA---CTGGGAACGCTAGGAGCT------
---ATGTTCCTTGGGTTCTTGGGAACA---GCAGGAAGCACTATG---GGCGCAGCGTCA---
CTGACGCTGACGGTACAG---GCCAGACAA---TTATTG---TCTGGTATAGTGCAGCAGCAGAAC---
AATTTG---CTGAGG---GCTATTGAGGCGCAACAGCATATGTTACAACTCACAGTCTGGGGCATC
>KT427824
TTCTACTGTAACACAACACCACTGTTTAATAGTAGTTGGCAGAAAAATAATGGAACTTGGAGTACCAATATTA
CTGGAAAT---
TGGAGTACTGATACTACTGGG------------
AATAATAGCACTAATATCACACTCCAATGTAGAATAAAACAAATTATAAACTTGTGGCAGGAAGTAGGAAAAG
CAATGTATGCCCCTCCCATCAGGGGACCAATTAAATGTGAATCAAAT---
ATTACAGGACTGCTATTAACAAGAGATGGGGGAGGAGATGGGGGTAATAAA----------------------
----
AGTAATGACCATAAC---
AAGACCGAGATCTTCAGACCTGGAGGAGGAGATATGAGGGACAATTGGAGAAGTGAATTATATAAATATAAAG
TAGTAAAAATTGAACCATTA---GGAGTAGCACCCACCAAGGCAAAGAGAAGAGTGGTG--------------
-------------------------CAGAGAGAAAAAAGAGCA---ATAGGG---CTGGGAGCT---------
ATGTTCCTTGGGTTCTTGGGAACA---GCAGGAAGCACTATG---GGCGCAGCGTCA---
ATAACGCTGACGGTACAG---GCCAGACAA---TTATTG---TCTGGTATAGTGCAACAGCAAAAT---
AATTTG---CTGAAG---GCTATTGAGGCGCAACAGCATCTGTTGCAACTCACAGTCTGGGGCATC
>KJ849790
TTCTACTGCAATACAACAAAGCTGTTTAGTAATTTTAATAGTACTTGG-------------------------
----
-------AATAATACTGAA------
GGGTCAAATGACACAATCATACTCCCATGCAGAATAAAACAAATTGTAAACAGGTGGCAGGAAGTAGGAAAAG
CAATGTATGCCCCTCCCATCAAAGGACAAATTAAATGTTCATCAAAT---
ATTACAGGGCTGCTATTAACAAGAGATGGTGGTATA-------------------------------------
----
AGGAATGAGTCTGGAGGAAAT---------
GAGTCTGAGACCTTCAGACCTGCAGGAGGGGATATGAAAGACAATTGGAGAAGTGAATTATATAAATATAAAG
TAGTACAAATTGAACCATTA---GGAGTAGCACCCACCAAGGCAAGAAGAAGAGTGGTG--------------
-------------------------CAGAGAGAAAAAAGAGAA---GTAACA---CTAGGGGCT---------
TTATTCCTTGGGTTCTTGGGAGCA---GCAGGAAGCACTATG---GGCGCAGCGTCA---
GTGACGCTGACGGTACAA---GCCAGACAA---TTATTG---TCTGGTATAGTGCAACAGCAGAAC---
AATTTG---CTGAGA---GCTATTGAGGCGCAACAGCATCTGTTGCAACTCACAGTCTGGGGCATC
>KT427819
TTCTACTGTAATACATCACAACTGTTTAATAGTACTTGGCTGCCTAATGGTACTTGGCTGTCTAATAGTACTA
GGAATGAG---

ACAAATGGAAATATCACACTCTCATGCAGAATAAAACAAATTATAAACAGGTGGCAGGAAGTAGGAAAAGCAA
TGTATGCCCCTCCCATCGCAGGGAAAATTAGCTGTTCATCAAAC---
ATTACAGGGCTGATATTAACAAGAGATGGTAGCAGCACAAATAGC----------------------------
----

ACGTCTGAAACCTTCAGACCTGGAGGAGGAGACATGAGGGACAATTGGAGAAGTGAATTATATAAATATAAAG
TAGTAAGAGTTGAGCCAGTA---GGAATAGCACCCACCAAGGCAAAGAGAAGAGTGGTG--------------
-------------------------CAGAGAGAAAAAAGAGCA---ATAGGA---TTAGGAGCT---------
ATGTTCCTTGGGTTCTTGGGAGCA---GCAGGAAGCACTATG---GGCGCAGCATCA---
GTGACGCTGACGGTACAA---GCCAGACAA---TTATTG---TCTGGTATAGTGCAACAGCAGAAT---
AATCTG---CTGAGA---GCTATTGAGGCGCAACAACATATGTTGCAACTCACAGTCTGGGGCATC
>KJ849796
TTCTACTGTAATTCAACACAGCTGTTTAATAGTTCTTGGCATTTTAATGGTTCTTGG----------------
----
TATGGTAATGAACCTGAAAGA------------

AATAAAACTGTCAATATCACACTCCCATGCAGAATAAAACAAATTATAAACATGTGGCAGGAAGTAGGAAAAG
CAATGTATGCCCCTCCCATCAGGGGACAAATTATGTGCAAATCAAAT---
ATTACAGGGCTGTTACTAGCAAGAGATGGTGGT--

AATAATGAATCTGAAATCTTCAGACCTGGAGGAGGAGATATGAGGGACAATTGGAGAAGTGAATTATATAAAT
ATAAAGTAGTACAAATTGAGCCATTA---GGAATAGCACCCACCACGGCAAGGAGAAGAGTGGTG--------
-------------------------------CAGAGAGAAAAAAGAGCA---GTGGGG---ATAGGAGCT---
------ATGATCCTTGGGTTCTTGGGAGCA---GCCGGAAGCACTATG---GGCGCAGCGTCA---
ATAACGCTGACGGTACAG---GCCAGACTA---TTATTG---TCTGGTATAGTGCAACAGCAGAAC---
AATTTG---CTGAGG---GCTATTGAGGCGCACCAGCATATGTTGCAACTCACAGTCTGGGGCATT
>KT427815
TTCTACTGTAATACATCACGACTGTTTAATAGTACTTGGGCTACAAATGAAACTAATGAGAGA----------
----

AATGAATCTACAATCACGCTCCCATGCAGAATAAGACAAATTATAAACAGGTGGCAAGAAGTAGGAAAAGCGA
TGTATGCCCCTCCCATTGCAGGACAAATTAACTGTTCATCAAAT---
ATTACAGGGCTACTATTAACAAGAGATGGTGGTACTGATAATAGT----------------------------
----

ACCGAGGTCTTCAGACCTGGAGGAGGAGATATGAGGGACAATTGGAGAAGTGAATTATATAAATACAAAGTAG
TACAAATTGAACCAATA---GGAATAGCACCCACCAAGGCAAGGAGAAGAGTGGTG-----------------
----------------------CAGAGAGAAAAAAGAGCA---ATAGGA---ATAGGAGCT---------
CTGTTCGTTGGGTTCTTGTCAGCA---GCAGGAAGCACTATA---GGCGCAGCGTCA---
GTGACGCTGACGGTACAG---GCCAGACAA---TTACTG---TCTAGTATAGTGCAACAGCAAAAC---
AATTTG---CTGAGG---GCTATTGAGGCGCAACAGCATCTGTTGCAACTCACAGTCTGGGGCATC
>KJ849807
TTCTACTGTAATACAACACAACTGTTTAATAGTACTTGGAATGATACATGGGTATAT----------------
----
AACATAGCTACAGGAAACATA------------
ACTACAGATAATAAAATCACACTCCCATGCAGAATAAAACAAATTATAAACAGGTGGCAGGAAGTAGGAAAAG
CAATGTATGCCCCTCCCATCGAAGGACTAATTAGATGCTCATCAAAT---
ATTACAGGACTACTATTAACAAGAGATGGGGGT--

GTTAACAGCAGCAAC---
CAGTATGAGATCTTCAGACCTGGAGGAGGAGATATGAGAGATAATTGGAGAAGTGAATTATATAAATACAAAG
TAGTAAGAATTGAACCGTTA---GGAATAGCACCCACCAAGGCAAGGAGAAGAGTGGTG--------------
-------------------------CAGAGGGAAAAAAGAGCA---GTGGGA---ATAGGAGCT---------
TTGTTCCTTGGGTTCTTGGGAGCA---GCAGGAAGCACTATG---GGCGCAGCGTCA---
ATGACGCTGACGGTACAG---GCCAGACAA---TTATTG---TCTGGTATAGTGCAACAGCAAAGC---
AATCTG---CTGAGA---GCTATTGAGGCGCAACAGCATATGTTGCAGCTCACAGTCTGGGGCATC
>KT427805
TTCTACTGTGAAACAACACAGCTATTTAATAGTACTTGGACTTTTAATAGGACGTCTAATAGTACCGGG----
----

AATGATACTATTATCACACTCCCATGCAGGATAAAACAAATTATAAACAGGTGGCAGGAAGTAGGAAAAGCAA
TGTATGCCCCTCCCATCAGAGGATCAATTAACTGCACATCAAAC---
ATCACAGGGCTAATACTAACAAGAGATGGTGGGATTAACAGGACT----------------------------
----

GAGAATGAAACCTTCAGACCTGGAGGAGGAGATATGAGGGACAATTGGAGAAGTGAATTATATAAATATAAAG
TAGTAAAAATTGAACCATTA---GGATTAGCACCCACCAAGGCAAAGAGAAGAGTGGTA--------------
-------------------------CAGAGAGAAAAAAGAGCA---GTAACG---CTGGGAGCT---------
ATGTTCCTTGGGTTCTTGGGAACA---GCAGGAAGCACTATG---GGCGCAGCGTCA---
TTGACGCTGACGGTACAG---GCCAGACAA---TTATTG---TCTGGTATAGTGCAACAGCAGAAC---
AATATG---CTGAGG---GCTATTGAGGCGCAACAGCATATGTTGCAACTCACAGTCTGGGGCATC
>KJ849808
TTCTACTGTAACACAACACAGCTGTTTAATGGCACATGGTCCAATGGTACA----------------------
----
-------TGGACAACACCG------

CTGCTTAATGGGACTATCACACTCCCATGCAGAATAAAACAAATTATAAACAGATGGCAGGAAGTAGGAAAAG
CAATATATGCCCCTCCCATCAGAGGACAAATTAGCTGTACATCAAAT---
ATCACAGGACTGCTATTAACAAGAGATGGGGGG--

-
GTTGGCCAGAGAGAGAATGAAACCTTCAGACCTGGAGGAGGAAATATGAAAGACAATTGGAGAAGTGAACTAT
ATAAATATAAAGTAGTAAAAATTGAACCATTA---GGAGTAGCACCCACCCCAGCAAAGAGAAGAGTGGTG--
-------------------------------------CAGAGAGAAAAAAGAGCA---
GTGGGAACAATAGGAGCT---------ATGTTCCTTGGGTTCTTAGGAGCA---GCAGGAAGCACTATG---
GGCGCAGCGTCG---CTGACGCTGACGGTACAG---GCCAGACTA---TTATTG---
TCTGGTATAGTGCAACAGCAAAAC---AATTTG---CTGAGG---
GCTATTGAGGCGCAACAGCATCTGTTGCAACTCACAGTCTGGGGCATC
>KT427799
TTCTACTGTAATACAACACAGTTATTTAATAGTACTTGGCATGCTAATAGCACTTGGAATGAGACTACAGGAT
CA---

GGGTCAAATGACACTATCTCACTCCCATGCAGAATAAAACAAATTATAAACAGATGGCAGGAAGTAGGAAAAG
CAATGTATGCCCCTCCCATCGGAGGACAAATTAGATGTTCATCAAAT---
ATTACAGGATACTATTAACAAGAGATGGTGGTACAGAGAACAAC-----------------------------
----

ACGTCCGAGACTTTCAGACCTGGAGGAGGAAATATGAAGGACAATTGGAGAAGTGAATTATATAAATATAAAG
TAGTAAGAATTGAGCCATTA---GGAGTAGCACCCACCAAGGCAAAGAGAAGAGTGGTG--------------
-------------------------CAGAGAGAAAAAAGAGCA---GTGACG---TTTGGAGCT---------
TTTTTCCTTGGGTTCTTAGGAACA---GCAGGAAGCACTATG---GGCGCAGCATCA---
ATAACGCTGACGGTACAG---GCCAGACAA---TTATTG---TCTGGTATAGTGCAACAGCAGAGC---
AACCTG---CTGAGA---GCTATTGAGGCGCAACAACGTATGTTGCAACTCACAGTCTGGGGCATC
>KT427667
TTCTACTGTAACACAACACAACTGTTTAACAGTACCTGGACTAATGGTACCTGGGAGACGACTAGTACTGGG-
----

AATATAAGTAATATCACACTCCCATGCAGGATAAAACAAATTATAAATAGGTGGCAGGAAGTAGGAAAAGCAA
TGTATAACCCTCCCATCAGGGGAAAAATTAACTGTTTATCAAAC---
ATTACAGGAATACTATTAACAAGAGATGGAGGAGGTAACGGGTCT----------------------------
----

AGTAACGAGACCTTCAGACCTGGAGGAGGAAATATGAAGGATAATTGGAGAAGTGAACTATATAAATATAAAG
TAGTAAAAATTGAACCATTA---GGACTAGCGCCCACCAGGGCAAGGAGAAGAGTGGTG--------------
-------------------------CAAAGAGAAAAAAGAGCA---GTGGGAACACTAGGAGCT---------
ATGTTCCTTGGATTCTTGGGAGCA---GCAGGAAGCACTATG---GGCGCAGCGTCA---
ATGGCGCTGACGGTACAA---GCCAGACAA---TTATTG---GCTGGTATAGTGCAACAGCAGAAC---
AATATG---CTGAGA---GCTATTGAGGCGCAACAGCATATGTTGCAACTCACAGTCTGGGGCATC
>KT427794
TTCTACTGTAATACAACAAAGTTGTTTAATAGTACTTGGAAGTACAACGATACTAGCAACACTTGGACTGAAG
GAAACCAT---
ACTAAAGAAGGAAACCATACT------------
GAAGGAAATAACACAATCACACTCCCATGTAGAATAAAGCAAATTATAAACAGGTGGCAGGAGGTAGGAAAAG
CAATGTATGCCCCTCCCATTAGGGGACTAATTAAATGTACATCAAAT---
ATTACAGGGATGATATTAACAAGAGATGGTGGTAAGGAGAATGAGACCGAG----------------------
----

AATACCGAGACCTTCAGACCTGGAGGAGGAGATATGAGGGACAACTGGAGAAGTGAATTATATAAATATAAAA
TAGTAAAAATTGAGCCATTA---GGAATAGCACCCACCAAGGCAAGGAGAAGAGTGGTG--------------
-------------------------CAGAGAGAAAAAAGAGCA---GTGGGAACAATAGGAGCT---------
ATGTTCCTTGGGTTCTTGGGAACA---GCAGGAAGCACTATG---GGCGCAGCGTCA---
ATCACGCTGACGGTACAG---GCCAGACAA---TTATTG---TCTGGTATAGTGCAGCAGCAGAAC---
AATTTG---CTGAAG---GCTATTGAGGCGCAACAACATCTGTTGCAACTCACAGTCTGGGGCATC
>KU749390
TTCTACTGTAATACAACACAATTGTTTAACAGTACTTGGAATGCTACTGGGAATGGTACTATAGAGGGA----
----

GATAGCGATAACATCACACTCCAATGCAAAATAAAACAAATTATAAACATGTGGCAGGAAGTAGGAAAAGCAA
TGTATGCCCCTCCCATCAGAGGAGAAATTAGATGTTCATCAAAT---
ATTACAGGGCTGCTATTAACAAGAGATGGTGGTGATAATGAGTCC----------------------------
----

AGTACTGATGAGACCTTTAGACCTGGAGGAGGAGATATGAAGGATAATTGGAGAAATGAATTATATAAATATA
AAGTAGTAAAAATTGAACCATTA---GGAATAGCACCCACCAAGGCAAAAAGAAGAGTGGTG-----------
----------------------------CAAAGAGAAAAAAGAGCA---GTGGGA---ATAGGAGCT------
---TTGTTCCTTGGGTTCTTGGGAGCA---GCAGGAAGCACTATG---GGCGCAGCGTCC---
ATGACGCTGACGGTACAG---GCCAGACAA---TTACTG---TCTGGTATAGTGCAACAGCAGAAC---
AATTTG---CTGAGA---GCTATTGAGGCGCAACAGCACCTGTTGCAACTCACAGTCTGGGGCATC
>KX181912
TTCTACTGTAATACAACATCACTGTTTAATAGTACTTGGAATAATACTAAAGAGTGGCATGGTACTACAGGG-
----

AGAAACAACACTATCACACTCCCATGTAAAATAAAACAAATCATAAACAGATGGCAGGAAGTAGGACAAGCTA
TGTATGCCCCTCCCATCAGTGGAAAAATTAACTGCTTATCAAAT---
ATTACAGGGCTGTTATTAATAAGAGATGGTGGTGTTAACGTTAGC----------------------------
----

AACACCACAGAGACCTTCAGGCCTGGGGGGGGAAATATAAAGGACAATTGGAGAAGTGAATTATATAAATATA
AAGTAGTAAAAATTGAACCCTTT---GGAGTAGCCCCCCCCCGGTCAAAGAGAAGAGTGGGG-----------
----------------------------CAGAGAGAAAAAAGAGCA---GTGACG---CTAGGAGCT------
---ATGTTCCTTGGGTTTTTGGGAGCA---GCAGGAAGCACTATG---GGCGCAGCGTCA---
ATGGGGCTGACGGTACAG---GCAAAACAA---TTATTG---TCTGGTATAGTGCAGCAGCAGAAT---
AATTTT---CTGAGG---GCTATTGAGGCGCAACAGCATATGTTGCAACTCACAGTCTGGGGCATT
>KX181906
TTCTACTGTAACACATCACAGCTGTTTAATAGTACTTGGTATTGGAATGGATCAAATTTCAATGAAACAAATC
TCAAT--

GAATCTGAAACTATCACAATCCCATGCAGAATAAAACAAATTATAAACAGGTGGCAGGAAGTGGGAAAAGCAA
TGTATGCCCCTCCCATCAGAGGAATGATTAACTGCACATCAAAT---
ATTACAGGGCTGCTATTAACATGGGATGGTGGTAACAATAACAGA----------------------------
----

ACGAACGAGACCTTTAGACCTGGAGGAGGAGATATGAGGGACAATTGGAGAAGTGAATTATATAAATATAAAG
TAGTAAAAATTGAGCCATTA---GGACTAGCACCCACCAAGGCAAAGAGAAGAGTGGTG--------------
-------------------------CAGAGAGAAAAACGAGCA---GTGGGAACAATAGGAGCT---------
ATGTTCCTTGGGTTTTTGGGAGCA---GCAGGAAGCACTATG---GGCGCAGCGTCA---
GTAACGCTGACGGTACAG---GCCAGACTA---TTATTG---TCTGGTATAGTGCAACAGCAAAAC---
AATTTG---CTGAGG---GCTATTGAGGCGCAACAACATATGTTGCAACTCACAGTCTGGGGCATT
>KX181900
TTCTACTGTAATACAACAAAACTGTTTAATAGTACTTGGAATAGTAGTAGTACTTGGTATAATAAAAGTGCTG
AA---

GGAAATTCCACTATTATACTCCCATGCAGAATAAAACAAATTATAAATAGGTGGCAGGAAGTAGGGAAAGCAA
TGTATGCCCCTCCCATCCCAGGACACATTAACTGTTTATCAAAT---
ATTACAGGGCTACTATTGACAAGAGATGGTGGAAACAACAACAGT----------------------------
----

AGCTCCGAAACTTTTAGACCTGGAGGAGGAGATATGAGGGACAATTGGAGAAGTGAATTATATAAGTATAAAG
TAGTAAGAATTGAACCATTA---GGAGTAGCACCCACCAAGGCAAGGAGAAGAGTGGTG--------------
-------------------------CAGAGAGAAAAAAGAGCA---GTGGGAACAATAGGAGCT---------
ATGTTCCTTGGGTTCTTGGGAGCA---GCAGGAAGCACTATG---GGCGCAGCATCA---
ATAACGCTGACGGTACAA---GCCAGACTA---TTATTG---TCTGGTATAGTGCAACAGCAGAAC---
AATCTG---CTGAGA---GCTATTGAGGCGCAACAGCATATGTTGCAACTCACAGTCTGGGGCATT
>KX181901
TTCTACTGTGATTCAACACAGCTGTTTAATAGTACTTGGCTGTTTAATGATACTCGGAATGGTACTGACATTG
AA---

GGTAATGAAACTATCACACTCCCATGTAGAATAAAACAAATTATAAACAGGTGGCAAGAAGTAGGAAAAGCAA
TATATGCCCCTCCCATCGAAGGACAAATTAGATGTACATCAAAT---
ATTACAGGGCTGTTATTAACAAGAGATGGTGGT-------------------------------------
----

AACAGTGGGACAGAAACTTTCAGACCTGGAGGAGGAGATATGAGGGACAATTGGAGAAGTGAATTATATAAAT
ATAAAGTAGTGAAAATTGAGCCATTA---GGAATAGCACCCACCAAGGCAAAGAGAAGAGTGGTG--------
------------------------------CAGAGAGAAAAAAGAGCA---GTGGGAACGATAGAAGCT---
------ATGTTCCTTGGGTTCTTGGGAGCA---GCAGGAAGCACTATG---GGCGCAGCGTCA---
GTAACGCTGACGGTACAG---GCCAGACTA---TTATTG---TCTGGTATAGTGCAACAGCAAAAC---
AATTTG---CTGAAG---GCTATTGAGGCGCAACAGCATATGTTGCAACTCACAGTCTGGGGCATT
>KX181891
TTCTACTGTGATACAACAAAGCTGTTCAATAGTACTTGGGTGATCAATAATACTTGGCAAAGAGTAAATAACA
CCAAA---

AATGAATCAAAGATCATACTCCCATGCAAAATAAAACAAATTATAAACAGGTGGCAGGAGGTAGGAAAAGCAA
TGTATGCCCCTCCCATCAGAGGATACATTAACTGTTCATCGAAT---
ATTACAGGGCTGCTATTAACAAGAGATGGGGGAGACAATAATAAT---------------------------
----

AATAATGAGACCTTCAGACCCCAAGGGGGAAATATGAAGGATAATTGGAGAAGTGAATTATATAAATATAAAG
TAATAAAAATTGAGCCATTA---GGAGTAGCACCCACCGAGGCAAAGAGAAGAGTGGTG--------------
------------------------CAGAGAGAAAAAAGAGCA---ATAGGAGTGCTAGGAGCT---------
GTGTTCCTTGGGTTCTTAGGAGCA---GCAGGAAGCACTATG---GGCGCAGCGTCA---
ATAACGCTGACGGTACAG---GCCAGACAA---TTATTG---TCTGGTATAGTGCAACAGCAGAGC---
AATCTG---CTGCAG---GCTATTGAGGCGCAACAGCATCTGTTGCAACTCACAGTCTGGGGCATC
>KX181907
TTCTATTGTAATACAACACAACTGTTCAATAGCACCTGGAATAATACAGAAATGGTTAATGGTACTACTAATG
GTACT---

ACTAATGATACAATTATACTCCCATGCAGAATAAAACAAATTGTAAACATGTGGCAGGAAGTAGGAAAAGCAA
TGTATGCCCCTCCCCTCAGGGGAAAAATTAACTGTGTATCAAAT---
ATTACAGGGCTAATATTAACAAGAGATGGGGGAATAAATAGGACG---------------------------
----

AAGAACGAGACCGAGACCTTCAGACCTGGGGGGGGAGATATGAGGGACAATTGGAGAAGTGAATTATATAAAT
ATAAAGTAGTAAAAATTGAACCCTTA---GGGGTAGCCCCCCCCGGGCAAAGAGAAGAGTGGTG--------
------------------------------CAAAGAGAAAAAAGAGCA---GTAACC---CTAGGAGCT---
------ATGTTCCTTGGGTTTTTGGGAGCA---GCAGGAAGCAATATG---GGCGCAGCGTCA---
ATAACGCTGACGGTACAG---GCCCGACAA---TTATTG---TCTGGTATAGTGCAACAGCAAAAC---
AATTTG---CTGAGA---GCTATTGAGGCGCAACAGCATCTGTTGCAACTCACGGTCTGGGGCATC
>KX181910
TTCTACTGTAATACATCACAGCTGTTTAATAGTACTTGGTTTGCGAATGGTACTAGAAAAGAAAATGGAACTG
CA--

GGAAACAACACATTCATCACACTCCCATGCAGAATAAAACAGATCATAAACAGGTGGCAGGAAGTAGGAAAAG
CAATGTATGCCCCTCCCATCAGTGGATACATTAATTGTTCATCAAAT---
ATTACAGGGCTGTTATTAACAAGAGATGGTGGTACCGGAAACAAA---------------------------
----

ACCGAGCCCGAGATCTTCAGACCTAGAAGGGGAGATATGAAGGACAATTGGAGAAGTGAATTATATAAATACA
AAGTAGTAGAAATTGAGCCATTA---GGAATAGCCCCCACCAAGGCAAAGAGAAGAGTGGTG-----------
---------------------------CAGAGAGAAAAAAGAGCA---GTAGAAATGCTAGGAGCT------
---ATGTTCCTTAGGTCCTTAGGAGCA---GCAGGAAGCACTATA---GGCGCAGCGTCG---
GTGGCGCTGACGGTACAG---GCCAGACAA---TTATTG---TCTGGTATAGTGCAACAGCAGAGC---
AATTTG---CTGAAG---GCTATTGAAGCGCAACAACATATGTTGCAACTCACAGTCTGGGGCATT
>KX181911
TTCTACTGTAATTCAACACAGCTGTTTAATAGTAGTTGGACTAATGGTAGTTGGACTGGCATTGAAGGAAATA
ATACA---

AGTAATGAAAGTATCACACTCCCATGCAGAATAAAACAAATAATAAATAGGTGGCAGGAAGTAGGAAGAGCAA
TATATGCCCCTCCCATCAGTGGACAACTTAACTGTTCATCAAAT---
ATTACAGGGCTGCTATTAACAAGAGATGGTGGTAATAGGACCAATGGG-------------------------
----

GAGGCCGAAGTCTTCAGACCTGGAGGAGGAAATATGAAAGACAATTGGAGAAGTGAATTATATAAATATAAAG
TAGTAAAAATTGAGCCATTA---GGAATAGCACCCACCAAGGCGAAGAGAAGAGTGGTG--------------
-------------------------CAGAGAGAAAAAAGAGCA---GTGGGAACAATAGGAGCT---------
ATGTTCCTTGGGTTCTTGGGAGCA---GCAGGAAGCACTATG---GGCGCAGCGTCA---
ATAACGCTGACGGTACAG---GCCAGACTA---TTATTG---TCTGGTATAGTGCAACAGCAGAAC---
AATCTG---CTGAGA---GCTATTGAGGCGCAACAGCATATGTTGCAACTCACTGTCTGGGGCATC
>KX181895
TTTACTGTAATACAACACGGCTGTTTAATAGTTTTTGGGTGAAAGGTACTTGGGGGAATAGTACTTTACAG-
----

AACAATGGTACTATCATACTTCCCTGCAGAATAAAACAGATTATAAACAGGTGGCAAGAAGTAGGAAAAACAA
AGTACGCCCCCCCCCTCAGAGGACACCTTAACTGTTTTTCAAAC---
ATTACAGGGGTGATATTAGCAAGGGATGGTGGTATTAATGTGACC----------------------------
----

AACAATACCGAGACCTTCAGACCTGGGGGAGGGGACAAGAGGGGCAATTGGGGAAGTGAATTATACAAATATA
AAATAGTAAAAAATGAGCCATTA---GGAGTAGCACCCACCAAGGCAAAGAGAAGAGTGGTG-----------
----------------------------CAGAGAGAAAAAAGAGCA---GTGGGAACTATAGGAGCT------
---ATGTTCCTTGGGTTCTTGGGAGCA---GCAGGAAGCACTATG---GGCGCAGCATCA---
GTGACGCTGACGGTACAG---GCCAGACTA---TTATTG---TCTGGTATAGTGCAACAGCAGAGC---
AATCTG---CTGAGG---GCTATTGAGGCGCAACAGCATTTGTTGCAACTCACAGTCTGGGGCATC
>KX181897
TTCTATTGTAATACAACACAGCTGTTTAATAGTACTTGGAATGGCACACATCTGTCTAATAGTATT-------
----

GGGAATGAGACTATCACACTCCCATGCAGAATAAAACAAATTATAAACAGATGGCAGGAAGTAGGAAAGGCAA
TGTATGCCCCTCCCATCAGTGGACAAATTAGCTGTTCATCAAAT---
ATAACAGGGCTGATATTAACAAGAGATGGTAGTAATGATAATAGC----------------------------
----

AGCACGAATGAAACTTTCAGACCGGGAGGAGGAAATATGAAAGACAATTGGAGAAGTGAATTATATAAATATA
AAGTAGTAGAAATTGAGCCAGTA---GGATTAGCACCCACCAAGGCAAAAAGAAGAGTGGTG-----------
----------------------------CAGAGAGAAAAAAGAGCA---GTAGGAACGCTAGGAGCT------
---ATGTTCCTTGGGTTCTTGGGAACA---GCAGGAAGCACTATG---GGCGCAGCGTCA---
TTGACGCTGACGGTACAG---GCCAGACAA---TTAATG---TCTGGTATAGTGCAACAGCAGAAC---
AATCTG---CTGAGG---GCTATTGAGGCGCAACAACATATGTTGCAACTCACAGTCTGGGGCATC
>KX181915
TTTTACTGTAATACAACAAACCTGTTTAATAATGGTACCCAGCTGCTGCCTAATGATACTTGGCAGTTTAATG
GAAGTAAAATT--

GAAAATGGGACTATCATACTCCCATGCAGAATAAAACAAATTATAAACAGGTGGCAGGAAGTAGGAAAAGCAA
TGTATGCCCCTCCCATCAGTGGACTAATTAGATGTTCATCAAAC---
ATTACAGGGCTGATATTAACAAGAGATGGTGGGTACAACAACAAGTCT-------------------------
----

AACAATACTGAGACCTTTAGACCTGGAGGAGGAGATATGAGGGACAATTGGAGAAGTGAATTATATAAATATA
AAGTAGTAAAAATTAAGCCATTA---GGGGTAGCACCCACCAAGGCAAAGAGAAGAGTGGTG-----------
----------------------------CAAAGAGAAAAAAGAGCA---GTAACA---ATAGGAGCT------
---ATGTTCCTTGGGTTTTTGGGAGCA---GCAGGAAGCACTATG---GGCGCAGCATCA---
CTAACGCTGACGGTACAG---GCCAGACAA---TTATTG---ACTGGTATAGTGCAACAGCAAAAC---
AATTTG---TTGAGG---GCTATTGAGGCGCAACAGCATTTGTTGCAACTCACAGTCTGGGGCATC
>KX181916
TTTTACTGTAATTCAACACAGCTGTTCAACAGTAGTTGGAGGTTTAATAATAATACTTGGAAGGTTGTTAATA
ATAATACT---

-------------------GGG---
AATTCAAATGACAACATCACACTCCCATGCAGAATAAAACAAATTATAAACAGGTGGCAGGATGTAGGAAAAG
CAATGTATGCCCCTCCCATCAGGGGACAAATTAGCTGTTCATCAAGT---
ATTACAGGGCTGCTATTAACAAGAGATGGTGGTAATAATAAAGAG----------------------------
----

TCCCCGGAGGCCTTCAGACCTGGAGGGGGGGATATGAAGGGCAATTGGGGAAGTGAATTATATAAATATAAAG
TAGTAAAAAATAAGCCATTA---GGAATAGCCCCCCCAAGGCAAAGAGAAGAGTGGTG--------------
-------------------------CAAAGAGAAAAAAGAGCA---GTAGGAACGATAGGAGGT---------
ATGTTCCTTGGGTTTTTGGGAGCA---GCAGGAAGCAGTATG---GGCGCAGCGTCC---
TTGACGCTGACGGTACAG---GCCAGACTA---TTATTG---TCTGGTATAGTGCAACAGCAGAAT---
AATTTG---CTGAGG---GCTATTGAGGCGCAACAACATCTGTTGCAACTCACAGTCTGGGGCATC
>MK041584
TTCTACTGTAACACAACAAAGCTGTTTAATAGTACTTGGAATAGTACTGACTGGTCAAATACCACTGAT----
----

GATAATGACACCATCACACTCCCATGCAGAATAAAACAAATTATAAACAGGTGGCAGGAAGTAGGAAAAGCAA
TGTATGCCCCTCCCATTGCAGGACAGATTATGTGTTCATCAAAT---
ATTACAGGGCTAATATTAACAAGAGATGGTGGTAACAGTAACGGGTCG-------------------------
----

ACTAATGAGACCTTCAGACCTGCAGGAGGAGATATGAGGGACAATTGGAGAAGTGAATTATATAAATATAAAG
TAGTAAAAATAGAGCCATTA---GGAATAGCACCTACCAAGGCAAGGAGAAGAGTGGTG--------------
-------------------------CAAAGAGAGAAAAGAGCA---GTGGGA---CTAGGAGCT---------
CTGTTTCTTGGGTTCTTGGGAGCA---GCAGGAAGCACTATG---GGCGCAGCGTCA---
ATGACGCTGACGGTACAG---GCCAGACAA---CTATTG---TCTGGTATAGTGCAACAGCAAAAC---
AATTTG---CTGATG---GCTATTGAGGCGCAACAGCGTATGTTGCAACTCACAGTCTGGGGCATC
>MK041586
TTCTACTGCAATACAACACAGCTGTTTAATTCAACTTGGGATACTAATTCAACTTGGACTGCTGAGGAAGCAG
AT---

GGAAATATCACTATCACACTCCCATGTAGAATAAAACAATTTATAAACAGATGGCAGGAGGTAGGAAAAGCAA
TGTATGCCCCTCCCATCAGAGGAATAATTAACTGTGTATCAAAT---
ATTACAGGACTACTATTAGTGAGAGATGGTGGTGAGAACAATGCA----------------------------
----

ACCAATGAGACCTTCAGGCCTGGAGGAGGCGATATGAGGGACAATTGGAGAAGTGAACTATATAAATATAAAG
TAGTACAGATTGAGCCGTTA---GGAATAGCACCCACCAGGGCAAGGAGAAGAGTGGTG--------------
-------------------------CAGAGAGAAAAAAGAGCA---GTGGGAACAATAGGAGCT---------
ATGTTCCTTGGGTTCTTGGGAGCA---GCAGGAAGCACTATG---GGCGCAGCGTCA---
ATGACGCTGACGGTACAG---GCCAGACTA---CTACTG---TCTGGTATAGTGCAACAGCAGAAC---
AACTTG---CTGAGA---GCTATTGAGGCGCAACAACATCTGTTGCAACTCACAGTCTGGGGCATC
>MK041551
TTTTACTGTGATACAACACAACTGTTTAATAGTACTTGGTCACTTAATAAGACTTCTTGGTATAATACTACTA
CT---

GAAGGAATAGATATCACACTCCCATGCAAAATAAAACAAATCATAAACAGGTGGCAGGGAGCAGGAAAAGCAA
TGTATGCCCCTCCCATCAGAGGAAAAATTTTCTGTTCATCAAAA---
ATTACAGGAATACTATTAACAAGAGATGGTGGGTTTAACAATGCCAGT-------------------------
----

AACAATAGCGAGACCTTCAGACCTGGAGGGGGAGATATGAGGGACAATTGGAGAAGTGAATTATACAAATATA
AAGTAGTAAAAATTGAGCCATTA---GGAGTAGCACCCACCAAGGCAAAAAGAAGAGTGGTG-----------
----------------------------CAAAGAGACAAAAGAGCA---GTGGGAACGTTAGGAGCT------
---ATGTTCCTTGGGTTCTTGGGAGCA---GCAGGAAGCACTATG---GGCGCAGCGTCA---
ATGGCGCTGACGGTACAG---GCCAGACAA---TTATTG---TCTGGTATAGTGCAGCAGCAAAAC---
AATTTG---CTGAGA---GCTATTGAGGCGCAACAACATCTGTTGCAACTCACAGTCTGGGGCATC
>MK041554
TTCTACTGTAATACATCACAGCTGTTTAATGAAACTTTTTTGAAGAAGAT----------------------
----

GATACTGAACCTATCACACTCCAATGCAGAATAAAACAAATTATAAACAGGTGGCAGGAAGTGGGAAAAGCAA
TGTATGCCCCTCCCATCAGTGGACAAATTAACTGTTCATCAAAT---
ATTACAGGGTTGATACTAACGAGAGATGGTGGCATCAATAATACC----------------------------
----

ATGAATGAGACTTTCAGACCTGGAGGAGGAGACATGAGGGACAATTGGAGAAGTGAATTATATAAATATAAAG
TAGTAAAAATTGAGCCATTA---GGAGTAGCACCCACCAAGGCAAAGAGAAGAGTGGTG--------------
-------------------------CAGAGAGAAAAAAGAGCA---GTGGGAACGATAGGAGCT---------
ATGTTCCTTGGGTTCTTGGGAGCA---GCAGGAAGCACTATG---GGCGCAGCGTCG---
CTGACGCTGACGGTACAG---GCCAGACTA---TTATTG---TCTGGTATAGTGCAACAGCAAAAC---
AATTTG---CTGAGG---GCTATTGAGGCGCAACAGCATCTGTTGCAACTCACAGTCTGGGGCATC
>MK041558
TTCTACTGTAATACAACACAGTTGTTTAATAGTACTTGGAATAGTACTGACACTAACTGGATAAATAATGCTA
GTTCG--

ACAAAGGGTAATATCACACTCCCATGCAGGATAAAACAAATTATAAACAGGTGGCAAGAAGTAGGAAAAGCAA
TGTATGCCCCTCCCATCAGCGGACAAATTAAGTGTTCATCAAAT---
ATTACAGGACTGCTATTAACAAGAGATGGTGGTCCTGAAAATGAC----------------------------
----

TCTGCGACCTTCAGACCTGGAGGAGGAGATATGAGGAATAATTGGAGAAGTGAATTATATAAATACAAAGTAG
TAAAAATTGAGCCATTA---GGAGTAGCACCCACCAAGGCAAAGAGAAGAGTGGTG-----------------
----------------------GAGAGAGAAAAAAGAGCA---GTGACA---CTAGGAGCA---------
ATGTTCCTTGGGTTCTTGGGAGCA---GCAGGAAGCACTATG---GGCGCAGCGTCA---
ATAACGCTGACGGTACAG---GCCAGACAA---TTATTG---TCTGGTATAGTGCAACAGCAGAAC---
AATCTG---CTGAAG---GCTATTGAGGCGCAACAGCACCTGTTGCAACTCACAGTCTGGGGCATC
>MK041556
TTCTACTGTAATACATCACAGCTGTTTAATAGTATGGAGTTGGAGGCTTTGAGGAATAACAATAAA-------
----

ACTAATGACACTATCACACTCCAATGCAGAATAAAACAAATTATAAACAGGTGGCAGGAAGTAGGAAAAGCAA
TGTATGCCCCTCCCATCAGTGGACAAATTAACTGTGTATCAAAT---
ATTACAGGGTTGATACTAACAAGAGATGGTGGTAACGGACTAAC----------------------------
----

GGGACTGAGACTTTCAGACCAGGAGGAGGAGATATGAGGGACAATTGGAGAAGTGAATTATATAAATATAAAG
TAGTAAAAATTGAGCCATTA---GGAATAGCACCCACCAAGGCAAAGAGAAGAGTGGTG--------------
-------------------------CAGAGAGAAAAAAGAGCA---TTGGGAACGATAGGAGCT---------
ATGTTCCTTGGGTTCTTGGGAGCA---GCAGGAAGCACTATG---GGCGCAGCGTCA---
CTGACGCTGACGGTACAG---GCCAGACAA---TTATTG---TCTGGTATAGTGCAACAGCAAAAC---
AATTTG---CTGAGG---GCTATTGAGGCGCAACAGCATCTGTTGCAACTCACAGTCTGGGGCATC
>MK041562
TTCTATTGTGATTCAGCACAGCTGTTTAATAGTACTTGGAATGAAACTGATAGTTGGAATAATACTTCT----
----

GAAGAATCAACTATCACACTCCCATGCAGAATAAAACAAATTGTAAACCTGTGGCAGGAAGTAGGAAAAGCAA
TGTATGCCCCTCCCAGAGAAGGGGTTATTAGCTGTTTATCAAAT---
ATCACAGGGCTGCTCTTAACAAGAGATGGTGGTAATGAGAACACGAACTCT----------------------
----

AGCACTGAAACCTTCAGACCTGGAGGAGGCAATATGAAAGACAATTGGAGAAGTGAATTATACAAATATAAAG
TAGTAAGAATTGAACCATTA---GGAGTAGCACCTACCCTGGCAAAAAGAAGAGTGGTG--------------
-------------------------CAGAGAGAAAAAAGAGCA---GTGGGA---ATAGGAGCT---------
GTGTTCCTTGGGTTCTTGGGAGCA---GCAGGAAGCACTATG---GGCGCAGCGTCG---
GTGACGCTGACGGTACAA---GCCAGACAA---TTATTG---TCTGGTATAGTGCAACAGCAGAAC---
AATTTG---CTGATG---GCTATTGAGGCGCAACAGCGTCTGTTGCAACTCACAGTCTGGGGCATC
>MG571981
TTCTACTGTAATACAACACAGCTGTTTAATAGTACTTGGAATGAAACACAGCTGTTTAATAATATTAATAATA
GTACTAAA---

GAGAATGAGAATATCACACTCCCATGCAGAATAAAACAAATTGTAAACAGGTGGCAGGAAGTAGGAAAAGCAA
TGTATGCCCCTCCCATCAGAGGACAAATTAGCTGTTCATCAAAC---
ATTACAGGGCTGATACTAACAAGAGATGGTAGCGGTAACGGGTCTGCG-------------------------
----

AGCAATGAGACTTTCAGACCTGGAGGAGGAAATATGAGGGACAATTGGAGAAGTGAATTATATAAATATAAAG
TAGTAAAAATAGAGCCATTA---GGAGTAGCACCCACCAGGGCAAAGAGAAGAGTGGTG--------------
-------------------------CAGAGAGAAAAAAGAGCA---GTGGGGACGTTAGGAGCT---------
ATGTTCCTTGGGTTCTTGGGAGCA---GCAGGAAGCACTATG---GGCGCAGCGTCA---
GTGACGCTGACGGTACAG---GCCAGACAA---TTATTG---TCTGGTATAGTGCAACAGCAGAAT---
AATCTG---CTGAAG---GCTATTGAGGCGCAACAACATCTGTTGCAACTCACAGTCTGGGGCATC
>MG571996
TTCTACTGCAATACAACACAGCTGTTTAATTCAACTTGGGATACTAATTCAACTTGGACTGCTGAGGAAGCAG
AT---

GGAAATATCACTATCACACTCCCATGTAGAATAAAACAATTTATAAACAGATGGCAGGAGGTAGGAAAAGCAA
TGTATGCCCCTCCCATCAGAGGAATAATTAACTGTGTATCAAAT---
ATTACAGGACTACTATTAGTGAGAGATGGTGGTGAGAACAATGCA----------------------------
----

ACCAATGAGACCTTCAGGCCTGGAGGAGGCGATATGAGGGACAATTGGAGAAGTGAACTATATAAATATAAAG
TAGTACAGATTGAGCCGTTA---GGAATAGCACCCACCAGGGCAAGGAGAAGAGTGGTG--------------
-------------------------CAGAGAGAAAAAAGAGCA---GTGGGAACAATAGGAGCT---------
ATGTTCCTTGGGTTCTTGGGAGCA---GCAGGAAGCACTATG---GGCGCAGCGTCA---
ATGACGCTGACGGTACAG---GCCAGACTA---CTACTG---TCTGGTATAGTGCAACAGCAGAAC---
AACTTG---CTGAGA---GCTATTGAGGCGCAACAACATCTGTTGCAACTCACAGTCTGGGGCATC
>MG571999
TTCTACTGTAATACAACAAAGCTGTTTAATGGTACTTGGAATGGTACGGATTTCAATAATACTGGAGAA----
----

TCAAATGACACTATCACACTCCCCTGCAGAATAAGACAAATTATAAATAGATGGCAGGAAGTAGGAAAAGCAA
TGTATGCACCTCCCATCAGTGGACCAATTAGCTGTTCATCAAAT---
ATTACAGGGCTAATGTTAACAAGAGATGGGGGTGAGGATAACAGT----------------------------
----

TCTACTGAAACCTTCAGACCTGGAGGAGGAAATATGAAAGACAATTGGAGAAGTGAATTATATAAATATAAAA
TAGTAAAAATTGAGCCATTA---GGAGTAGCACCCACCACGGCAAAGAGAAGAGTGGTG--------------
-------------------------CAGAGAGAAAAAAGAGCA---GTGGGAACGCTAGGAGCT---------
ATGTTCCTTGGGTTCTTGGGAGCA---GCAGGAAGCACTATG---GGCGCAGCGTCA---
GTGACGCTGACGGTACAG---GCCAGACAA---TTATTG---TCTGGTATAGTGCAACAGCAGAAC---
AATTTG---CTGAGA---GCTATTGAGGCGCAACAGCACATGTTGCAACTCACAGTCTGGGGCATT
>MG572006
TTCTACTGTAATACAACACAGCTGTTTAATAGTACTTGGGGTAATAATACTTGGAATGGTACTGAAGGAAAT-
----

GGAAATGACACTATCACACTTCCATGTAGAATAAAACAAATTATAAACAGATGGCAGGAAGTAGGAAAAGCAA
TATATAACCCTCCAGTCAGAGGAATAATTAACTGTGCATCAAAT---
ATTACAGGGCTGCTATTAACAAGAGATGGTGGTAACACAAACACAACC-------------------------
----

AATAGGTCTGAAATCTTCAGACCTGGAGGAGGAAATATGAAGGACAATTGGAGAAGTGAATTATATAAATATA
AAGTAGTAAAAATAGAACCAATA---GGAATAGCACCCACCGCGGCAAAGAGAAGAGTGGTG-----------
----------------------------CAGAGAGAAAAAAGAGCA---CTGCCG---CTGGGAGCT------
---ATGTTCCTTGGGTTCTTGGGAGCA---GCAGGGAGCACTATG---GGCGCAGCGTCA---
ATGACGCTGACGGTACAG---ACCAGACAA---TTATTG---TCTGGTATAGTGCAACAGCAGAGC---
AATCTG---CTGAGG---GCTATTGAGGCGCAACAACATATGTTGCAACTCACAGTCTGGGGCATC
>AY173956
TTCTACTGTAATTCAACACAACTGTTTAACAGTACTTGGAATAGTACTTGGAATGTTGATGAAAGATCAAAT-
----

GACACTGCAACACTCAAACTCCCATGCAGAATAAAACAATTTGTAAACATGTGGCAGGAAGTAGGAAAAGCAA
TGTATGCCCCTCCCATCGATGGGCAAATTAGTTGTTCATCAAAT---
ATTACAGGGTTACTATTAACAAGAGATGGTGGTAATAACACAAAC---------------------------
----

GAGACTGAGACCTTCAGACCTGGAGGAGGAGATATGAGGGACAATTGGAGAAGTGAATTATATAAATATAAAG
TAGTAAAAGTTGAACCATTA---GGAGTAGCACCCACCAGGGCAAAGAGAAGAGTGGTG-------------
-------------------------CAAAGAGAAAAAAGAGCA---GTGGGA---ATAGGAGCT--------
GTGCTCCTTGGGTTCTTGGGAGCA---GCAGGAAGCACTATG---GGCGCAGCGTCA---
CTGACGCTGACGGTACAG---GCCAGACAA---TTATTG---TCTGGTATAGTGCAACAGCAAAAC---
AATTTG---CTGAGG---GCTATTGAGGCGCAACAGCATCTGTTGCAACTCACAGTCTGGGGCATC
>AY779552
TTCTACTGTAATTCAACACCACTGTTTAATAGTACTTGGAATAGTACTACTTGGAACAGCACTAAAGGGTCAC
ATAAC---
-------------------AGT---
ACAGGAAATGAAACTATCACACTCCAATGCATAATAAAACAAATTATAAACCTGTGGCAGGAAGTAGGAAAAG
CAATGTATGCCCCTCCCATCAAAGGACAAATTAAATGCTCATCAAAT---
ATTACAGGGCTGCTATTAACAAGAGATGGTGGTAGGGGCAATGACACG------------------------
----

GACAATACCACCGAGATCTTCAGACCTGGAGGAGGAGATATGAGGGATAATTGGAGAAGTGAATTATACAAAT
ATAAAGTAGTAAGAATTGAGCCATTA---GGGGTAGCACCTACCAAGGCAAAGAGAAGAGTGGTG--------
-------------------------------CAGAGAGAAAAAAGAGCA---GTGGGA---ATAGGAGCC---
------TTGTTCCTTGGGTTCTTGGGAGCA---GCAGGAAGCACTATG---GGCGCAGCGTCA---
ATAACGCTGACGGTACAG---GCCAGACTA---TTATTG---TCTGGTATAGTGCAGCAGCAGAAC---
AATCTG---CTGAGG---GCTATTGAGGCGCAACAGCACCTGTTGCAACTCACAGTTTGGGGCATC
>GU562135
TTCTACTGCAACTCATCAAAACTGTTTAATTGGAATTCAACACATTGGAATTCAACACATTGGAATTCAACAC
AACAGGGT--
-------------AATAGT------
ACTGGAAATGATACTATTACACTCACATGTAGGATAAAACAAATTATAAACATGTGGCAGGAAGTAGGAAAAG
CAATGTATGCCCCTCCCATCAAAGGACAAATTAGATGTTCATCAAAT---
ATTACAGGGATACTATTAACAAGAGATGGTGGAAATAACAATGACACC------------------------
----

AACAAAACCGAGATCTTCAGACCAGGAGGAGGAGATATGAGGGACAATTGGAGAAGTGAATTATATAAATATA
AAGTAGTAAAAATTGAACCATTA---GGAGTAGCACCCACCAAGGCAAAGAGAAGAGTGGTG-----------
----------------------------CAGAGAGAAAAAAGAGCG---GTAACG---CTAGGAGCT------
---ATGTTCCTTGGGTTCTTGGGAGCA---GCAGGAAGCACTATG---GGCGCAGCGTCA---
CTAACGCTGACGGTACAG---GCCAGACTA---TTATTG---TCTGGTATAGTGCAACAGCAGAAC---
AATCTG---CTGAGG---GCTATTGAGGCGCAGCAGCATCTGTTGCAACTCACAGTCTGGGGCATC
>GU562272
TTCTACTGTAATTCAACACCACTGTTTAATAGTACTTGGCCTAGAAATTCAACAACACAACAGTCTAAT----
----

AATAGTACACCCATTGTACTCAAATGTAGAATAAAGCAAATTATAAACATGTGGCAGGAAGTAGGAAAAGCAA
TGTATGCCCCTCCCATTAAAGGAGTAATTAGGTGTTCATCAAAT---
ATCACAGGGATAATATTAACAAGAGATGGTGGTGCTAATAATACTAAC------------------------
----

CATAATAATACCGAGACTTTCAGACCTGCAGGAGGAGATATGAGGGACAATTGGAGAAGTGAATTATATAAAT
ATAAAGTAGTAAAAAATTGAACCATTA---GGAGTAGCACCCACCAGGGCAAAGAGAAGAGTGGTG--------
-------------------------------CAAAGAGAAAAAAGAGCA---GTGGGAGTAGTAGGAGCA---
------ATGTTCCTTGGGTTCTTGGGAGCA---GCAGGAAGCACTATG---GGCGCAGCGTCA---
ATAACGCTGACGGTACAG---GCCAGACAA---TTACTG---TCTGGTATAGTGCAACAGCAGAAC---
AATCTG---CTGAAG---GCTATTGAGGCGCAACAGCATCTGTTGCAACTCACAGTCTGGGGCATA
>GU562155
TTTTACTGTAATTCAACACACCTATTTAATTCAACTTGGAATAAGAATGATTCAACTTGGAATAAGAATGATT
CAACTTGGACT---

-AAGAATGATTCAACA--------
GTAAATAACGAAACTATCACCCTCCCATGCAGAATAAAACAAATTATAAACAAGTGGCAGGAAGTAGGAAAAG
CAATGTATGCCCCTCCCATCAAAGGACAAATTAGATGCTCATCAAAT---
ATTACAGGGCTAATATTAGTAAGAGATGGTGGGAACACGAACACC-------------------------
----

ACCGAGACCTTCAGACCTGCAGGAGGAGATATGAGGGACAATTGGAGAAGTGAATTATATAAATATAAAGTAG
TAAAAATTGAACCATTA---GGAGTAGCACCTACCAAGGCAAAGAGAAGAGTGGTG--------------
---------------------CAGAGAGAAAAAAGAGCA---GTGGGA---CTAGGAGCT-------
GTGTTCTTTGGGTTCTTGGGAGCA---GCAGGAAGCACTATG---GGCGCAGCGTCA---
GTGACGCTGACGGTACAG---GCCAGACAA---TTATTG---TCTGGTATAGTGCAACAGCAGAAT---
AATTTG---CTGAGG---GCTATTGAGGCGCAACAGCATATGTTGCAACTCACAGTCTGGGGCATC
>JF320413
TTCTACTGTGACTCAACACCACTGTTTAATAGTACTTGGAATAAGACTGAAAATGAGTGGAATAGTACTACTG
AC--

ATACCAGAGAACATCACCCTCAGATGTAGAATAAGACAAATTATAAACATGTGGCAGGAAGTAGGAAAAGCAA
TGTATGCCCCTCCTATCAGTGGCCCAATTAACTGTACATCAAAT---
ATTACAGGGCTGCTATTAACAAGAGATGGTGGTAACCTCAGCAGC-------------------------
----

ACTAATGAGACCTTCAGACCTGGAGGAGGAAATATGAGGGATAATTGGAGAAGTGAATTATATAAATATAAAG
TAGTAAAAATTGAACCATTA---GGAGTAGCACCCACCAAGGCAAGGAGGAGAGTGGTG-----------
------------------------CAGAGAGAGAAAAGAGCA---GTGGGA---ATAGGAGCA-------
TTGTTCCTTGGGTTCTTGGGAGCA---GCAGGAAGCACTATG---GGCGCAGCGTCA---
ATGACGCTGACGGTACAA---GCCAGGCTA---TTATTG---TCTGGTATAGTGCAACAGCAGAAT---
AACTTG---CTGAGA---GCTATTGAGGCGCAACAGCATATGCTGCAACTCACAGTCTGGGGCATC
>GU562266
TTCTACTGTAATACATCACAATTGTTTAATGGTACTTGGAATAGTACTACATGGTCAAATTCAATTAAC----
----

AGCACTGAAAATATCACACTCCCATGCAGAATAAAACAATTTATAAACATGTGGCAGGAAGTAGGAAAAGCAA
TGTATGCCCCTCCCATCCAAGGACCAATTAGATGTTTATCAAAT---
ATTACAGGGCTACTATTAACAAGAGATGGTGGTAATAATAATGACATG----------------------
----

AATAACACCGAGACTTTCAGACCTGGAGGAGGAGATATGAGGGACAATTGGAGAAGTGAATTATATAAATATA
AAGTAGTACAAATTGAACCATTA---GGAGTAGCACCCACCAAGGCAAGGAGAAGAGTGGTG--------
---------------------------CAGAGAGAAAAAAGAGCA---GTAATGGACTAGGAGCT-----
---ATGTTCCTTGGGTTCTTGGGAGCA---GCAGGAAGCACTATG---GGCGCAGCATCA---
ATAACGCTGACGGTACAG---GCCAGACAA---TTATTG---TCTGGCATAGTGCAACAGCAGAGC---
AATTTG---CTGAGG---GCTATTGAGGCGCAACAGCACATGTTGCAACTCACAGTCTGGGGCATC
>AY314061
TTCTACTGTAATACAACATCACTGTTTAACAGTACTTGGAATGGTACTGAAGGGTCAATTAACACTACA----
----

GAAAATGACCCACTCACACTCCCATGCAGGATAAAACAAATTATAAATATGTGGCAGGAAGTAGGAAAAGCAA
TGTATGCCCCTCCCATCGCAGGACAAATTAGATGTACATCAAAT---
ATTACAGGGCTGCTACTAACAAGAGATGGTGGTAATGACACCATCAAT----------------------
----

AAGGGGACCGAGGTCTTCAGACCTGGAGGAGGAGATATGAGGGACAATTGGAGAAGTGAATTATATAAATATA
AAGTAGTAAAAATTGAACCATTA---GGAGTAGCACCCACCAGGGCAAAGAGAAGAGTGGTG--------
---------------------------CAAAGAGAAAAGAGAGCA---GCGGGA---ATAGGAGCT----
---GTGCTCCCTGGGTTCTTGGGAGCA---GCAGGAAGCACTAAG---GGCGAA---TCC---
AGCACACTGACGGTACAA---GCCAGACAA---TTATTG---TCTGGTATAGTGCAACAGCAGAGT---
AATCTG---CTGAGG---GCTATTGAGGCGCAACAGCATCTGTTGCAACTCACAGTCTGGGGCATC
>GU562033
TTCTACTGTAATTCAACACAACTGTTTAATAGTACTTGGAATGTTAGTAGTACTTGGAATAGTACTGAAGAG-
----

```
----------------------------
TCAAATGACACTATTATACTCCCATGTAGGATAAAACAAATTATAAACATGTGGCAGAAAGTAGGAAAAGCCA
TGTATGCCCCTCCCATCAGAGGACAAGTTAAATGTTCATCAAAA---
ATTACAGGGATACTATTAACAAGAGATGGTGGTAATAGTGGTAATAAC-------------------------
-------------------------------------------------------------------------
-------
ACGAACGAGACCGAGACCTTCAGGCCTGGAGGAGGAGATATGAGGGACAATTGGAGAAGTGAATTATATAAAT
ATAAAGTAGTAAAAATTAAACCATTA---GGAGTAGCACCCACCAAGGCAAAGAGAAGAGTGGTG--------
-------------------------------CAGAGAGAAAAAAGAGCA---GTGGGAACAATAGGAGCT---
------ATGTTCCTTGGGTTCTTGGGAGCA---GCAGGAAGCACTATG---GGCGCAGCGTCA---
ATGACGCTGACGGTACAG---GCCAGACAA---TTATTG---TCTGGTATAGTGCAACAGCAAAAC---
AATTTG---CTGAGG---GCTATTGAGGCGCAACAGCATCTGTTGCAACTCACAGTCTGGGGCATC
>GU562058
TTCTACTGTAATTCAACACAGCTGTTTAATAGTACTTGGTTTAAGAATAATACTTGGATAGGGTCAAAT----
-------------------------------------------------------------------------
----------------------------
GGCACTGAAAATATCACACTCCCATGCAGAATAAAACAAATTATAAACAGGTGGCAGGAAGTAGGAAAAGCAA
TGTATGCCCCTCCCATCAGTGGAGTAATTAGCTGTTCATCAAAT---
ATTACAGGGCTGATATTAACAAGAGATGGGGGAAGAGGTGATGTTAATAAC----------------------
-------------------------------------------------------------------------
-------
ACCAACGAGACTGAGATCTTCAGGCCTGGAGGAGGAGATATGAGGGACAATTGGAGAAGTGAATTATATAAAT
ATAAAGTAGTAAAAATTGAGCCATTA---GGAATAGCACCCACCAAGGCAAAGAGAAGAGTGGTG--------
-------------------------------CAAAGAGAAAAAAGAGCA---GTAACG---TTTGGAGCT---
------TTGTTCCTTGGGTTCTTGGGAGCA---GCAGGAAGCACTATG---GGCGCAGCGTCA---
ATAACGCTGACGGCACAG---GCCAGACTA---TTATTG---TCTGGTATAGTGCAACAGCAGAAC---
AATTTG---CTGAGA---GCTATTGAGGCGCAACAGCATCTGTTGCAACTCACAGTCTGGGGCATC
>GU562001
TTCTACTGTACTACAACACAGATGTTTAATAGTACTTGGAAGGATGGTACTTGGACTGAAGGATCAAATAAC-
-------------------------------------------------------------------------
----------------------------
ACTGGCGGAAATATCATACTCCCATGCAGAATAAAACAAATTATAAACATGTGGCAGAAAGTAGGAAAAGCAA
TATATGCCCCTCCCATTAGTGGACAAATTAACTGTTCATCAAAT---
ATTACAGGGCTGCTATTAACAAGAGATGGTGGTAACGAGGGCAATGCT-------------------------
-------------------------------------------------------------------------
-------
ACTAATAATACTGAAACCTTCAGACCTGGTGGAGGAGATATGAGGGACAATTGGAGAAGTGAATTATATAAAT
ATAAAGTAGTAAAAATTGAACCAATA---GGAATAGCACCCACCAAGGCAAAGAGAAGAGTGGTG--------
-------------------------------CAGAGAGAAAAAAGAGCA---GTGGGA---ATAGGAGCT---
------GTGTTCCTTGGGTTCTTGGGAGCA---GCAGGAAGCACTATG---GGCGCAGCGTCA---
ATAACGCTGACGGTACAG---GCCAGACAA---TTATTG---TCTGGTATAGTGCAACAGCAGAAC---
AATTTG---CTGAGG---GCTATTGAGGCGCAACAGCACCTGTTGCAACTCACAGTCTGGGGCATC
>GU562080
TTCTACTGTAATACAACACAACTATTTAATTCAACTTGGAATAATAACTCAACTTGGAGTAGTAATTCAACGG
GAGAT--------------------------------------------------------------------
-------------------------
AATGAAAATATCACTATCACCCTCCCATGCAGAATAAAACAAATTATAAACATGTGGCAGGAAGTAGGAAAAG
CAATGTATGCCCCTCCCATCGGAGGGCAAATTAGATGCTCATCAAAT---
ATTACAGGGATGATTTTAACAAGAGATGGTGGTGAGAACAACACC----------------------------
-------------------------------------------------------------------------
----------------
ACTGAGATCTTCAGACCTGGAGGAGGAGATATGAGGGACAATTGGAGAAGTGAATTATATAAATATAAAGTAG
TAAAAATTGAACCAATA---GGAGTAGCACCCACCAAGGCAAAGAGAAGAGTGGTG-----------------
----------------------CAGAGAGAAAAAAGAGCA---GTGGGA---ATGGGAGCT---------
TTGTTCCTTGGGTTCTTGGGAGCA---GCAGGAAGCACTATG---GGCGCAGCATCA---
ATAACGCTGACGGTACAG---GCCAGGCAA---TTATTG---TCTGGTATAGTGCAACAGCAGAAT---
AATTTG---CTGAGA---GCTATTGAGGCGCAACAGCATCTGTTGCAACTCACAGTCTGGGGCATC
>DQ322223
TTCTACTGTAATGTAACAGAACTGTTTAATAGTACTTGGAATGGAACTGAACAGAATAGTACCCAG-------
-------------------------------------------------------------------------
```

AATGAGGATACTATCACACTCCCATGCAGAATAAAACAAATTATAAACATGTGGCAGGAAGTAGGAAAAGCAA
TGTATGCCCCTCCCATCAGAGGAAATATTACTTGTTCATCAAAT---
ATTACAGGGCTGCTATTGACAAGAGATGGTGGAAACAATGAGAGC----------------------------
----

ACGAATGAAACCTTCAGACCTGGAGGAGGAGATATGAGGGACAATTGGAGAAGTGAACTATATAAATATAAAG
TAGTAAAACTTGAACCATTA---GGAGTAGCACCCACCAAGGCAAAGAGAAGAGTGGTG--------------
-------------------------CAAAGAGAAAAAAGAGCA---GTGGGA---ATAGGAGCT---------
ATGTTCCTTGGGTTCTTGGGAGCA---GCAGGAAGCACTATG---GGCGCAGCGTCA---
ATAACGCTGACGGTACAG---GCCAGACAA---TTACTG---TCTGGTATAGTGCAACAGCAGAGC---
AATTTG---CTGAGA---GCTATTGAGGCACAACAGCATATGTTGCAACTCACAGTCTGGGGCATC
>DQ322227
TTCTATTGTAATACAACAAAACTGTTTAATAGTACTTGGCCTAGAAACAACACTGAAGGGTCTGAA-------
----

GGAAATGACACAATCACACTCCCATGCAGAATAAAACAAATTATAAACATGTGGCAGGAAGTAGGAAAAGCAA
TGTATGCCCCTCCCATCAGAGGACAAATTAAATGTTCATCAAAT---
ATTACAGGGCTGTTATTAACAAGAGATGGGGGTAATAATGAGACC----------------------------
----

AATGGGATTGAGACCTTCAGACCTGGAGGAGGAAATATGAAGGACAATTGGAGAAGTGAATTATATAAATATA
AAGTAGTACAAATTGAACCATTA---GGAATAGCACCCACCAAGGCAAAGAGAAGAGTGGTG-----------
----------------------------CAGAGAGAAAAAAGAGAC---CTAGGA---CTAGGAGCT------
---TTGTTAATCGGGTTCTTGGGAGCA---GCAGGAAGCACTATG---GGCGCAGCGTCA---
GTGACGCTGACGGTACAG---GCCAGGCAA---TTAATG---TCTGGTATAGTGCAACAGCAGAAC---
AATCTG---CTGAGA---GCTATTGAGGCGCAACAGCACATGTTGCAACTCACAGTCTGGGGCATT
>DQ322225
TTCTACTGTAATACAACAAAACTGTTCAATAGTACTTGGAATAGTACTGAGAATGGGAATACTTGGAATAAAA
CTACATGGTTA--
-------------GATAAC------
CCAGAAAATGACACAATCACACTCCCATGCAGAATAAGACAAATTATAAACATGTGGCAGGAAGTAGGAAAAG
CAATGTATGCCCCTCCCATCCAAGGACAGATTAACTGTTCATCAAAT---
ATTACAGGGCTGCTATTAACAAGAGATGGTGGTGAGGATCGTAACAATAGC----------------------
----

GAGAATGAGGAGAATGAGATCTTCAGACCTGGAGGAGGAGATATGAGGGATAATTGGAGAAGTGAATTATATA
AATATAAAGTAGTAAAAATTGAACCATTA---GGAGTAGCACCCACCAAGGCAAAGAGAAGAGTGGTG-----
----------------------------------CAAAGAGAAAGAAGAGCA---GTGGGA---
CTAGGAGCT---------ATGTTCCTTGGGTTCTTGGGAGCA---GCAGGAAGCACTATG---
GGCGCAGCGTCA---ATAACGCTGACGGTACAG---GCCAGACAA---TTATTG---
TCTGGTATAGTGCAGCAGCAAAAC---AATTTG---CTGAGA---
GCTATTGAGGCGCAACAGCATTTGTTGCAACTCACAGTCTGGGGCATC
>DQ322239
TTTTACTGTGATACAAAACCGCTGTTTAATAGTACTTGGAATCCAAAACAACCAGTTAATAGTACTGGGAATG
GT---

ACTGACGGAGATATCATACTCCAATGTAGAATAAAACAAATTATAAACATGTGGCAGAAAGTAGGAAAAGCAA
TGTATGCCCCTCCCATCAGAGGACCAATTAGTTGTTTATCAAAT---
ATTACAGGGCTACTATTAACAAGAGATGGTGGTAACGTGAGTGATAGT-------------------------
----

AATATCACCGAGATCTTCAGACCTGTAGGAGGAGATATGAAGGACAATTGGAGAAGTGAATTATATAAATATA
AAGTAGTAAAAATTGAACCATTA---GGAATAGCACCCACCAAGGCAAAAAGAAGAGTGGTG-----------
----------------------------CAAAGAGAAAAAAGAGCA---GTGGGAGTGATAGGAGCT------
---ATGTTCCTTGGGTTCTTGGGAGCA---GCAGGAAGCACTATG---GGCGCAGCGTCA---
TTGACGCTGACGGTACAA---GCCAGACTA---TTATTG---TCTGGTATAGTGCAACAGCAGAAC---
AATTTG---CTGAGA---GCTATTGAGGCGCAACAGCATCTGTTGCAACTCACAGTCTGGGGCATC
>JQ403022

TTCTACTGCAATACATCACAACTATTTAATAGTACCTGGAATAGTACTTGGTCTGATACTTGGAATAGTACTG
GAGGA---

AAGAACGGCACAATCACACTCCCATGTAGAATAAAACAAATTATAAACAGGTGGCAGGAAGTAGGAACAGCAA
TGTATGCCCCTCCCATCAGAGGACTAATTAAGTGTACATCAAAT---
ATTACAGGGCTGTTATTAACAAGAGATGGTGGTAATACCACCAAC---------------------------
----

AACAACAGCGAGACCTTCAGACCTGGAGGAGGAGATATGAGAGACAATTGGAGAAGTGAATTATATAAATATA
AAGTAGTAAGAATTGAACCATTA---GGAGTAGCACCCACCAAGGCAAAGAGAAGAGTGGTG-----------
----------------------------CAGAGAGAAAAAAGGGCA---GCA------CTAGGAGCT------
---ATGTTCCTTGGGTTCTTGGGAGCA---GCAGGAAGCACTATG---GGCGCAGCGTCA---
GTAGCGCTGACGGTACAA---GCCAGACAA---TTATTG---TCTGGTATAGTGCAACAGCAGAAC---
AATTTG---CTGAGA---GCTATTGAGGCGCAAACAAATATGTTGCGACTCACGGTCTGGGGCATT
>JQ403023
TTCTATTGTGATTCAACAAAATTGTTTAATAGTAATTGGACTATAGGTGAGGATACTGATTGGTTCAATAATA
CTGATTGG--

TTAAATGGCACAATCACACTCCCATGCAGAATAAAACAAATTGTAAACATGTGGCAGGAAGTAGGAAAAGCAA
TGTATGCCCCTCCCATCAGCGGACAAATTAGCTGTAATTCAACT---
ATTACAGGGCTGTTATTAACAAGAGATGGTGGTGTGGATAATAACACC------------------------
----

GATAATAACACTGAGATCTTCAGGCCAGGAGGAGGAGATATGAGGGACAATTGGAGAAGTGAATTGTATAAAT
ATAAAGTAGTACAAATTGAACCATTA---GGAATAGCACCCACTAAGGCAAAGAGAAGAGTGGTG--------
-------------------------------CAGAGAGAAAAAAGGGGC------------ATAGGAGCT---
------GTGTTCCTTGGGTTCTTGGGAGCA---GCAGGAAGCACTATG---GGCGCAGCGTCA---
GTGACGCTGACGGTACAG---GCCAGACAA---TTATTG---TCTGGTATAGTGCAACAGCAAAAC---
AATTTG---CTGAAG---GCTATTGATGCGCAACAGCATCTGTTGCAACTCACAGTCTGGGGCATC
>KC797171
TTCTACTGTAATACAACACAACTGTTTAATAGTACCTGGTCCAATAGTAGTGGTACTTGGAATTCAACACATA
ATAGTACT--
-------------GAGGAG------
TTAGATAACGACACAATCACACTCCCATGCAGAATAAAACAAATTATAAACATGTGGCAGGAAGTAGGAAAAG
CAATGTATGCCCCTCCCATCAAGGGAAAAATTAGATGTTCATCAAAC---
ATTACAGGTCTGCTATTAACAAGAGATGGTGGCAATACTAATAATACTAAT---------------------
----

AATACCATTGAAACCTTCAGACCTGGAGGAGGGGATATGAGGGACAATTGGAGAAGTGAATTATTTAAATATA
AAGTAGTACAAATTGAACCATTA---GGAGTAGCACCTTCCAGGGCAAAGAGAAGAGCGGTG-----------
----------------------------CAGAGAGAAAAAAGAGCA---GTAGGAACACTAGGAGCT------
---ATGTTCCTTGGGTTCTTGGGAGCA---GCCGGAAGCACTATG---GGCGCAGTGTCA---
ATGACGCTGACGGTACAG---GCCAGACAA---TTATTG---TCTGGTATAGTGCAACAGCAGAGC---
AATCTG---CTGAGG---GCTATTGAGGCGCAACAGCATATGTTGCAACTCACAGTCTGGGGCATC
>JQ403025
TTCTACTGTAATTCAACAAAACTGTTTAATAGTACTTGGGATACTAATACTACAGGGTTAGATAATGACACTA
AAGGA---

GATAATGACACATTCATACTCCCATGTAGAATAAAACAGATTATAAACCTGTGGCAGGAAGTAGGAAAAGCAA
TGTATGCCCCTCCCATCAAAGGACAAATTAGATGTTCCTCAAAT---
ATTACAGGACTGCTAATAACAAGAGATGGTGGTAGTAACGGGACT---------------------------
----

GATGAGATCTTCAGACCTGGAGGAGGAGATATGAGGGACAATTGGAGAAGTGAATTATATAAATATAAAGTAG
TAAAAATTGAACCATTA---GGATTAGCACCCACCAAGGCAAAGAGAAGAGTGGTG-----------------
----------------------CAAAGAGAAAAAAGAGCA---GTGGGA---TTCGGAGCT---------
CTGTTCCTTGGGTTCTTGGGAGCA---GCAGGAAGCACTATG---GGCGCAGCGTCA---
GTGACGCTGACGGTACAG---GCCAGACAA---TTATTG---TCTGGTATAGTGCAACAGCAAAAC---
AATTTG---CTGAAG---GCTATTGAGGCGCAACAGCATCTGTTGCAACTCACAGTCTGGGGCATC
>JQ403026

TTCTACTGTAATACAACACATTTGTTTAAGGGTACTTGGAATGGTACTGAATGGTCAAATAACACTGAAGGGT
CAAAT--
-------------GACACT------
GCAAGAAATGACACAATCATACTCCCATGCAGAATAAGACAATTTATAAACATGTGGCAGGAAGTAGGAAAAG
CAATGTATGCCCCTCCCATCAAAGGACAAATTAGTTGTTCATCAAAT---
ATTACAGGGCTGATATTAACAAGAGATGGTGGTAATGAGACCGAAAAG-------------------------
----

AATGAGACTGAGACCTTCAGACCTGGAGGGGGAGATATGAGGGACAATTGGAGAAGTGAATTATATAAATATA
AAGTAGTAAAAATTGAACCATTA---GGAGTAGCACCCACCAAGGCAAAGAGAAGAGTGGTG-----------
----------------------------CAGAGAGAAAAAAGAGCA---GTGGGA---ATAGGAGCT------
---CTGTTCCTTGGGTTCTTGGGAGCA---GCAGGAAGCACTATG---GGCGCAGCGTCA---
ATGACGCTGACGGTACAG---GCCAGACTA---TTATTG---TCTGGTATAGTGCAGCAGCAGAAC---
AATCTG---CTGAGG---GCTATTGAGGCGCAACAACATCTGTTGCAACTCACAGTCTGGGGCATC
>EU023916
TTCTACTGTAATACAACACAGCTATTTAATAGTACCTGGGTAAATGGTACTAATACTGGGAATAGTACT----
----

AATAGCACCGATATCACACTCCCGTGCAGAATAAAACAAATTGTAAACAGGTGGCAGGAAGTAGGAAAAGCAA
TGTATGCCCCTCCCATCAGAGGACAGATTAGATGTAACTCAAGT---
ATTACAGGGCTGCTATTAACAAGAGATGGTGGAGGTAACAGGACCGAG-------------------------
----

AACAGGACCGAAACCTTCAGACCTGGAGGAGGAGATATGAGGGACAATTGGAGAAGTGAATTATACAAATATA
AAGTAGTAAAAATTGAGCCATTA---GGAGTAGCACCCACCAAGGCAAAGAGAAGAGTGGTG-----------
----------------------------CAGAGAGAAAAAAGAGCA---GCACTA------GGAGCC------
---ATGTTCCTTGGGTTCTTGGGAGCA---GCAGGAAGCACTATG---GGCGCAGCGTCA---
GTGACGCTGACGGTACAG---GCCAGACTA---TTATTG---TCTGGTATAGTGCAACAGCAGAAC---
AATCTG---CTGAGA---GCTATTGAGGCGCAACAGCATATGTTGCAACTCACAGTCTGGGGCATC
>JQ403019
TTCTACTGTAATACAACACAACTGTTTAATAGTACCTGGTCTGATAATAGTACCGGGAATGGTACTGAA----
----

GGAGGAGAGGTCATCACAATCCCATGCAGAATAAAACAAATTGTAAACATGTGGCAGAGAGTAGGAAAAGCAA
TATATGCCCCTCCTATTAAGGGACAAATTAACTGTACTTCAAAT---
ATTACAGGGTTGCTCTTAACAAGAGATGGTGGTAATACTAGTAGG----------------------------
----

GACAACGAGACCTTCAGACCTGGAGGAGGAAATATGAGAGACAATTGGAGAAGTGAATTATATAAATACAAAG
TAGTAAGAATTGAGCCATTA---GGAGTAGCACCCACCAAGGCAAAGAGAAGAGTGGTG--------------
-------------------------CAAAGAGAAAAAAGAGCA---GTGGGAACAATAGGAGCT---------
ATGTTCCTTGGGTTCTTGGGAGCA---GCAGGAAGCACTATG---GGCGCAGCGTCG---
ATGGCGCTGACGGTACAG---GCCAGACAA---TTATTG---TCTGGTATAGTGCAACAGCAAAAC---
AATTTG---CTGAGA---GCTATTGAGGCGCAACAACATATGTTGCAACTCACAGTCTGGGGCATT
>JQ403029
TTCTACTGTAATACAGCACAACTGTTTAATAGTACTTGGGGGGTTGCTAATAGTACTTGGAAT----------
----

GTTAGTGACAATATCACACTCCCATGCAGAATAAAACAAATTATAAACATGTGGCAAGAAGTAGGAAAAGCAA
TGTATGCCCCTCCCATCCAAGGACAAATTAGATGTTCATCAAAT---
ATTACAGGGATAATATTAACAAGAGATGGTGGTAACGGAAATGGGACC-------------------------
----

GACAACACCACCGAGATCTTCAGACCTGGAGGGGGAGATATGAGGGACAATTGGAGAAGTGAATTATATAAAT
ATAAAGTAGTAAAAATTAAACCATTA---GGAGTAGCACCCACCAAGGCAAAGAGAAGAGTGGTG--------
-------------------------------CAGAGAGAAAAAAGAGCA---GTAACG---TTAGGAGCT---
------ATGTTCCTTGGGTTCTTGGGAGCA---GCAGGAAGCACTATG---GGCGCAGCGTCA---
ATAACGCTGACGGTACAG---GCCAGACAA---TTGTTG---TCTGGTATAGTGCAACAGCAGAGC---
AATTTG---CTGAGA---GCTATTGAGGCGCAACAACATCTGTTGCAACTCACAGTCTGGGGCATC
>JQ403044

TTCTACTGTGATACAGCAAAACTGTTTGATAGTACTTGGCATAATAGTACTTGGAATGATACTACAGGAGTCA
ATAACACT---
-------------GAGGGA------
AATAGCACAGACCCAATCGAACTCCCATGCAGAATAAAACAAATTATAAACATGTGGCAGGGAGTAGGAAGAG
CAATGTATGCCCCTCCCATCAGTGGACGAATTAGCTGTTCATCAAAT---
ATTACAGGACTGCTATTAACAAGAGATGGTGGCACAAATGATACA----------------------------
----

AAAGATAATGAGACCTTCAGACCTGGAGGAGGAGATATGAGAGACAATTGGAGAAGTGAATTATGGAAATATA
AAGTAGTAAAAGTTGAACCATTA---GGACTAGCACCTACCAAGGCAAAGAGAAGAGTGGTG-----------
----------------------------CAGAGGGAAAAAAGAGCA---ATAACA---CTAGGAGCT------
---ATGTTCCTTGGGTTCTTGGGAGCA---GCAGGAAGCACTATG---GGCGCAGCGTCA---
ATGACGCTGACGGTACAG---GCCAGACAA---TTATTG---TCTGGTATAGTGCAACAGCAAAAT---
AATTTG---CTGAGA---GCTATTGAGGCGCAGCAGCATATGTTGCAACTCACAGTCTGGGGCATC
>KX792621
TTCTATTGTAATACAACAGGACTGTTTAACAGCACTTGGAATTCAACACAACTGTCTAATGATACTGGGAATG
AAACTGAGGGG---TCA------GGT---

AACACTACTACAGAGATCATACTCCAATGCAGAATAAAACAAATTATAAACATGTGGCTGGAAGTAGGAAAAG
CAATGTATGCCCCTCCCATCAGAGGACAAATCAGATGTTCATCTAAT---
ATTACAGGGCTATTATTAACACGGGATGGTGGTAATACTAATAATACA-------------------------
----
GAAGGGAATGGCACCGAGACT---------
TTCACTGAGATCTTCAGACCTGAGGGAGGAGACATGAGGGACAATTGGAGAAGTGAATTATATAAATATAAAG
TAGTAAAAATTGAACCATTA---GGAGTAGCACCCACCAAGGCAAAGAGAAGAGTGGTG--------------
-------------------------CAGAGAGAAAAAAGAGCG---GCA------ATAGGAGCT---------
TTGTTCCTTGGGTTCTTGGGAGCA---GCAGGAAGCACTATG---GGCGCAGCGTCA---
ATGACGCTGACGGTACAG---GCCAGACAA---TTGTTG---TCTGGTATAGTGCAACAGCAGAGC---
AACTTG---CTGAGA---GCTATTGAGGCGCAACAGCACCTGTTGCAACTCACAGTCTGGGGCATC
>JQ403043
TTCTACTGTAATTCAACAAAACTGTTCAATAGTACTTGGTATAGGAATGATACAGGAACTTGGAATATTACAG
GAACTGGA---

GAGAATGACACACTCACACTCCCATGCAGAATAAAACAAATTATAAACAGGTGGCAGGAAGTAGGAAAAGCAA
TGTATGCCCCTCCCATCAGAGGACAAATTAGATGTACATCAAAT---
ATTACTGGGATACTATTAACAAGAGATGGTGGTAATACAAATAACAATAATACC------------ATG----
----

AACGAGACTGAGATCTTCAGACCTGGAGGAGGAGATATGAGGGACAATTGGAGAAGTGAATTATATAAATATA
AAGTAGTAAAAATTGAACCATTA---GGACTAGCACCCACCAGGGCAAAGAGAAGAGTGGTG-----------
----------------------------CGGATAGAAAAAAGAGCA---GTGACA---CTAGGAGCT------
---ATGTTCCTTGGGTTCTTGGGAGCA---GCAGGAAGCACTATG---GGCGCAGCGTCA---
GTAACGCTGACGGTACAG---GCCAGACAA---TTATTG---ACTGGTATAGTGCAACAGCAGAAT---
AATTTG---CTGAAG---GCTATTGAGGCGCAACAACATCTGTTGCAACTCACAGTCTGGGGCATC
>JQ403045
TTCTACTGTAATACAACACAACTGTTTAATAGTAGCTGGGATAATAGTACTGAGAATGGAAATGATACTGAAG
GG---

TCAAATAACCTTATCATACTCCCATGCAGAATAAAACAAATTATAAACATGTGGCAGAAAGTAGGAAAAGCAA
TGTATAACCCTCCCATCCAGGGACAAATTAGCTGTTCATCAAAT---
ATTACAGGGCTGCTATTAACACGAGATGGTGGTAAGAGTGAGAACAAGACT----------------------
----

GAGAACAAGACCACTGAGATCTTCAGACCGGGAGGAGGAGATATGAGGGACAATTGGAGAAGTGAATTATATA
AATATAAAGTAGTAAAAATTGAACCATTA---GGAGTAGCACCCACCAAGGCAAAGAGAAGAGTGGTG-----
----------------------------------CAGAGAGAAAAAAGAGCA---GTGGGA---
TTAGGAGCT---------CTGTTCCTTGGGTTCTTGGGAGCA---GCAGGAAGCACTATG---
GGCGCAGCGTCA---GTGACGCTGACGGTACAG---GCCAGACAA---TTATTG---
TCTGGTATAGTGCAGCAGCAAAAC---AATTTG---CTGAGG---
GCTATTGAGGCGCAGCAGCATCTGTTGCAACTCACAGTCTGGGGCATC

>JQ403046
TTCTACTGTGATACAACACAGCTGTTTAATAGTACTTGGAACGGTACTGAAGGAAATTTC-------------
----

ACTGAAACTAACATCACACTCCCATGCAGAATAAAACAAATTGTAAACAGGTGGCAGGAAGTAGGGAGAGCGA
TGTATGCCCCTCCCATCAGCGGAATCATTAGATGTTCATCAAAT---
ATTACAGGGCTGCTACTAACAAGAGATGGTGGTATCAGCAACAATGGG-------------------------
----

TCCGAAAATGGGTCCGAAACCTTCAGACCTGGAGGAGGAGATATGAGGGACAATTGGAGAAGTGAATTATATA
AATATAAAGTAGTAAAAATTGAGCCATTA---GGATTAGCACCCACCAAGGCAAAGAGAAGAGTGGTG-----
----------------------------------CAAAGAGAAAAAAGAGCA---GTAACG---
CTGGGAGCT---------GTGTTCCTTGGGTTCTTGGGAGCA---GCAGGAAGCACTATG---
GGCGCAGCGTCA---ATAACGCTGACGGTACAA---GCCAGACTC---TTATTG---
TCTGGTATAGTGCAGCAGCAGAAC---AATCTG---CTGAAA---
GCTATTGAGGCGCAACAGCATATGTTGCAACTCACAGTCTGGGGCATC
>EU023919
TTCTACTGTAATACAACACAACTGTTTAATAGTACTTGGGAGGTTAATAATACTGAGAGTAATACTACCATA-
----

TCAAATGACACTATTACTCTCCCATGCAGAATAAAACAAATTATAAACATGTGGCAGAAGGTAGGAAAAGCAA
TGTATGCCCCTCCCATCAAAGGACTAATTAGATGTTCATCAAAT---
ATTACAGGGCTGCTATTAACAAGAGATGGTGGTAATGGCCCAAAC----------------------------
----

GGGACAAATGAGGCCTTCAGACCTGCAGGAGGAGATATGAGGGACAATTGGAGAAGTGAACTATATAAATATA
AAGTAGTACAACTTGAACCATTA---GGAATAGCACCCACCAAGGCAAAGAGGAGAGTGGTG-----------
----------------------------CAGAGAGAAAAAAGAGCA---GTGGGAATGATAGGAGCT------
---ATGTTCCTTGGGTTCTTGGGAGCA---GCAGGAAGCACTATG---GGCGCAGCGTCA---
TTGACGCTGACGGTACAG---GCCAGACAG---TTACTG---TCTGGTATAGTGCAACAGCAGAAT---
AATCTG---CTGAGG---GCTATTGAGGCGCAACAGCATCTGTTGCAACTCACAGTCTGGGGCATC
>EU023922
TTCTACTGTAATACAACACAACTGTTTAATAGTACTTGGAATGGGACTAATACTGAAGGCACTGAA-------
----

GGAAATGACACAATCACACTCCCATGCAGAATAAGACAGATTATAAACATGTGGCAGGAAGTAGGAAAAGCAA
TGTATGCCCCTCCCATCAGAGGACAAATTAGATGTTCATCAAAT---
ATTACAGGGCTGCTATTAACAAGGGATGGTGGTACGAACGAGACC----------------------------
----

AACGAGACCGAGATCTTCAGACCAGGAGGAGGAGATATGAGGGACAATTGGAGAAGTGAATTATATAAATATA
AAGTAGTAAAAATTGAACCATTA---GGAGTAGCACCCACCAAGGCAAAGAGAAGGGTGGTG-----------
----------------------------CAGAGAGAAAAAAGAGCA---GTGGGA---CTAGGAGCT------
---ATGTTCCTTGGGTTCTTGGGAGCA---GCAGGAAGCACTATG---GGCGCAGCGTCA---
GTGACGCTGACGGTACAG---GCCAGACAA---TTATTG---TCTGGTATAGTGCAACAGCAGAAT---
AATCTG---CTGAAG---GCTATTAAGGCGCAACAACATCTGTTGCAACTCACAGTCTGGGGCATC
>KX792557
TTCTACTGTAATACAACACAGCTGTTTAACAGTACGTGGACTTGGAATAATACTGATAGTGCTGGGAACGAG-
----

ACTGAAAAAATTATCACACTCCCGTGCAGGATAAGACAAATTGTAAATAGATGGCAAGAAGTAGGAAAAGCAA
TGTATGCCAATCCCATTAGTGGACCAATTAGCTGTTCATCAAAT---
ATTACAGGGCTGCTATTAACAAGAGATGGTGGTATTAACAACGAGACC-------------------------
----

AATACCACCGAGACCTTCAGACCTTGGGGGGGAGATATGAGGGACAATTGGAGAAGTAAATTATATAAATATA
AAGTAGTAAAAATTAAACCATTA---GGACTAGCACCCACCAAGGCAAAGAGAAGAGTGGTG-----------
----------------------------CAGAGAGAAAAAAGAGCA---GTAACA---TTGGGAGCT------
---ATGTTTCTTGGGTTCTTGGGAACA---GCAGGAAGCACTATG---GGCGCAGCGTCA---

CTGACGCTGACGGTACAG---GCCAGACTA---TTATTG---TCTGGTATAGTGCAACAGCAGAAC---
AACCTG---CTGAGG---GCTATTGAGGCGCAACAGCATCTGTTGCAACTCACAGTCTGGGGCATC
>KX792831
TTCTACTGTAATACAACAAAACTGTTTGATAGTACCTGGTATCCGAATGGTACTTGGTATTCGAATGGTATTA
CTTGGTATCCG---AATGGTACTACAAGGTATCCGAATGGTACTACAAAGGCAAATAGC---
ACAGGGTCAAT-------------------------
AGCACTGAGGATACGATCACACTCCCATGCAGAATAAAACAAATTATAAACAGGTGGCAAGAAGTAGGAAAAG
CAATGTATGCCCCTCCCGCCCGAGGACAAATTAAATGCTCATCAAAT---
ATTACAGGGCTGCTATTGACAAGAGATGGTGGTAAGAGCAATACCAGTGAG----------------------
----

AACGAGGAGACCTTCAGACCGGGAGGAGGAAATATGAGGGACAATTGGAGAAGTGAATTATATAAATATAAAG
TAGTAAAAATTGAACCATTA---GGAATAGCACCCACCAAGGCAAGGAGAAGAGTGGTG--------------
-------------------------CAGAAAGAAAAAAGAGCA---GTAACA---CTAGGAGCT---------
GTGTTCCTTGGGTTCTTGGGAGCA---GCAGGAAGCACTATG---GGCGCAGCGTCA---
ATGACGCTGACGGTACAA---GCCAGACAA---TTATTG---TCTGGTATAGTGCAACAGCAGAGC---
AATCTG---CTGAGG---GCTATTGAGGCGCAACAGCACCTGTTGCAACTCACAGTCTGGGGCATC
>JQ403042
TTCTACTGTAATACAGCACAACTGTTTAATAGTACTTGGAATTGGAATGATACTACCAAATCAAATAATACA-
----

GGGCCAGATAACATCACACTCCCATGCAGAATTAAACAAATTATAAACAGGTGGCAGGAAGTAGGAAAAGCAA
TGTATGCCCCTCCCATCAGCGGACTAGTTAGATGTGTATCAAAT---
ATTACAGGGCTACTATTAACAAGGGATGGTGGTACGGACAACACC----------------------------
----

ACCGAGATCTTCAGACCTGGAGGAGGAAATATGAAGGACAATTGGAGAAGTGAATTATATAAATATAAAGTAG
TAAAAATTGAGCCATTA---GGAATAGCACCCACCAAGGCAAAAAGAAGAGTGGTG-----------------
----------------------CAGAGAGAAAAAAGAGCA---ATAACG---ATAGGAGCT---------
ATGTTCCTTGGGTTCTTGGGAGCA---GCAGGAAGCACTATG---GGCGCAGCGTCA---
ATGACGCTGACGGTACAG---GCCAGACTA---TTATTG---TCTGGTATAGTGCAACAGCAGAAC---
AATCTG---CTGAGG---GCTATTGAGGCGCAACAGCATCTGTTGCAACTCACAGTCTGGGGCATC
>KX792664
TTCTACTGTAATTCATCACGACTGTTTAATTGGACAGGGAATGGGACTAATATAACATCAAATAGCAATATCA
CA---

TCAAATATCAATATCACAATCCCATGCAGGATAAAACAAATTATAAACATGTGGCAGGAAGTAGGAAAAGCAA
TGTATGCCCCTCCTATCATGGGACAAATCAAGTGTTCAACAAAT---
ATTACAGGGATAATATTAACAAGAGATGGTGGGAACAACGAAACT----------------------------
----

AACGCCACCGAGATCTTCAGACCTGGAGGAGGAAATATGAAGGACAATTGGAGAAGTGAATTATATAGATATA
AAGTAGTAAAAATTGAACCATTA---GGAGTAGCACCCACCAAGGCAAAGAGAAGAGTGGTG-----------
----------------------------CAGAGAGAAAAAAGAGCA---ATACCA---TTAGGAGCT------
---ATGTTCCTTGGGTTCTTGGGAGCA---GCAGGAAGCACTATG---GGCGCAGCGTCA---
ATGGCGCTGACGGTACAA---GCCAGACAA---TTATTG---TCTGGTATAGTGCAGCAGCAGAAC---
AATTTG---CTGAGG---GCTATTGAGGCGCAACAGCATCTGTTGCAACTCACAGTCTGGGGCATC
>KX792688
TTCTACTGTAATACATCACAACTGTTTAATAGCACTTGGTATTTTAATAGCACTGGGGATAACAATAGTACTG
GGAATACT---

ACTAACAAAAATATCACACTCCCATGTAGAATAAAACAAATTATAAACATGTGGCAGGCAGTAGGAAAAGCAA
TATATGCCCCTCCCATCAGTGGACAAATTAGTTGTTCATCAAAC---
ATTACAGGGCTAATATTAACAAGAGATGGTGGTACAAACACCACA----------------------------
----

AATGATACCTTCAGACCTGCAGGGGGGAATATGAAAGACAATTGGAGAAGTGAATTATATAAATATAAAGTAG
TAAAAATTGAACCATTA---GGAGTGGCACCCACCAAGGCAAAGAGAAGAGTGGTG-----------------
----------------------CAGAGAGAAAAAAGAGCC---GTGGGA---CTAGGAGCT---------
TTGTTCCTTGGGTTCTTGGGAGCA---GCAGGAAGCACTATG---GGCGCAGCGTCA---

ATGACGCTGACGGTACAG---GCCAGACAA---TTATTG---TCTGGTATAGTGCAGCAGCAGAAC---
AATATG---CTGAGG---GCTATTGAGGCGCAACAGCATATGTTGCAACTCACAGTCTGGGGCATC
>KX792771
TTCTACTGTGATTCAACACCACTGTTTAACAGTACTTGGAACAGTACTTGGAGTGGTAATGATACGGGAGTAA
ACAAC--

ACTGAAAAGACTATCACACTCCAATGTAGAATAAAACAAATTATAAACATGTGGCAGAGAGTAGGAAAAGCAA
TGTATAACCCTCCCATCAGTGGACAAATTAATTGCTCATCAAAT---
ATTACAGGGATGATATTATCAAGAGATGGTGGTAGTATGAACAGG----------------------------
----

AGTAACGAGACCTTCAGACCTGGAGGAGGAGATATGAGGGACAATTGGAGAAGTGAATTATATAAATATAAAG
TAGTAAAAGTTGAACCATTA---GGAGTAGCACCCACCAAGGCAAAGAGAAGAGTGGTG--------------
-------------------------CAGAGAGAAAAAAGAGCA---ATGGGA---ATAGGAGCT---------
TTGTTCCTTGGGTTCTTGGGAGCA---GCAGGAAGCACTATG---GGCGCAGCGTCA---
ATAACGCTGACGGTACAG---GCCAGACAA---TTATTG---TCTGGTATAGTGCAACAGCAGAAC---
AATTTG---CTGAAA---GCTATTGAGGCGCAACAGCATCTGTTGCAACTCACAGTCTGGGGCATC
>KX792725
TTCTACTGTAATACAACACAACTATTTAATAGTACTTGGCTGTTTAATAGTACATGGAATAGTACT-------
--GAA---
AGATTAGGGAATGATACTAAA---------------
AGGACAAATGACACTATCACACTCCCATGCAAAATAAAACAAGTTATAAACATGTGGCAGACAGTAGGAAAAG
CAATGTATGCTCCTCCCATCAGAGGACTAATTAGATGTTCATCAAAT---
ATTACAGGGCTGATATTGACAAGAGATGGTAGTGGTAACACCACC----------------------------
----

GGTAACGAGACCTTTAGGCCTGGAGGAGGAAATATGAAGGACAATTGGAGAAGTGAATTATATAAATATAAAG
TGGTAAAAATTGAACCCTTA---GGAGTAGCACCCACTAGGGCAAAGAGAAGAGTGGTG--------------
-------------------------CAGAGAGAAAAAAGAGCA---GCGGGA---CTAGGAGCT---------
TTGTTCCTTGGGTTCTTGGGAATG---GCAGGAAGCACTATG---GGCGCAGCGTCA---
CTAACGCTGACGGTACAG---GCCAGACAA---TTATTG---TCTGGTATAGTGCAACAGCAGAAC---
AATCTG---CTGAGG---GCTATTGAGGCGCAACAGCATCTGTTGCAACTCACAGTCTGGGGCATC
>KX792808
TTCTACTGTAATTCAACACAACTGTTTAATAGTAGTCGGGATAATAATAGAAGGTCAGATAACTCTGAA----
----

GGAAATAAAACAATCACACTCCCATGCAGAATAAAACAAATTATAAACATGTGGCAGAAAGTAGGAAAAGCAA
TGTATGCCCCTCCCATCAGAGGACAAATTAGATGTTCATCAAAT---
ATTACAGGGATAATATTAACAAGAGATGGTGGTAATGGTAATGAGTCCAAT----------------------
----

AACGAGACCGAGACCTTCAGACCTGGAGGAGGGGATATGAGGGACAATTGGAGAAGTGAATTATATAAATATA
AAGTAGTAAGAATTGAACCATTA---GGATTGGCACCCACCAAGGCAAAGAGAAGAGTGGTA-----------
----------------------------CAGAGAGAAAAAAGAGCA---GTGGGA---ATAGGAGCT------
---GTGTTCCTTGGGTTCTTGGGAGCA---GCAGGAAGCACTATG---GGCGCAGCCTCG---
ATGACGCTGACGGTACAA---GCCAGACTA---TTATTG---TCTGGTATAGTGCAACAGCAGAAC---
AATCTG---CTGAGA---GCTATTGAGGCGCAACAACATCTGTTGCAACTCACAGTCTGGGGCATC
>EU023929
TTCTACTGCAATACAACACAACTGTTTAATAGTACTTGGTATAGTAATAGTACTTGGAATAGTACTGAAGGGG
CAAAT--

AACACTGCAGGAAATATCACACTCCCATGCAGAATAAAACAAATTGTAAACAGGTGGCAGGGAGTAGGAAAAG
CAATGTATGCCCCTCCCATCAGAGGACAAATTAGGTGTTCATCAAAT---
ATTACAGGGCTAATATTAACAAGAGATGGGGGGCCTAACAACACG----------------------------
----

ACAGAGACCTTCAGACCTGGAGGAGGAGATATGAGGGACAATTGGAGAAGTGAATTATATAAATATAAAGTAG
TACAAATTGAACCATTA---GGAGTAGCACCCACCAAGGCAAAAAGAAGAGTGGTG-----------------
----------------------CAGAGAGAAAAAAGAGCA---GTGGGA---ATAGGAGCT---------
GTGTTCCTCGGGTTCTTGGGAGCA---GCAGGAAGCACTATG---GGCGCAGCGTCA---

CTGACGCTGACGGTACAG---GCCAGACAA---TTATTG---TCTGGTATAGTGCAACAGCAGAAC---
AATCTG---CTGAGG---GCTATTGAGGCGCAACAGCATCTGTTGCAACTCACAGTCTGGGGCATC
>EU023933
TTCTACTGTAACACATCACAGCTGTTTAATAGTACTTGGAACGGTACTGAGAGTGGTATTACAAACCTTACAA
AC---

CCAGATGAGACTATCTCACTCCCATGCAGAATAAGACAAATTGTAAACAGGTGGCAGGAAGTAGGAAAAGCAA
TGTATGCCCCTCCAATCAGTGGACAAATTAATTGTTCATCAAAT---
ATTACAGGGCTGCTATTAGTAAGAGACGGTGGTGTGTATAATAGTACA-------------------------
----

AGTGATAACGAGACTTTCAGACCTGAAGGAGGAAATATGAAAGACAATTGGAGAAGTGAATTATATAAATATA
AAGTAGTAAAAATTGAGCCATTA---GGAATAGCACCCACCAAGGCAAGGAGAAGAGTGGTG-----------
----------------------------CAAAGAGAAAAAAGAGCA---GTGGGAACGTTAGGAGCT------
---ATGTTCCTTGGTTTCTTGGGAGCA---GCAGGAAGCACTATG---GGCGCAGCGTCA---
CTGACGCTGACGGTACAA---GCCAGATTA---TTATTA---TCTGGTATAGTGCAACAGCAAAAC---
AATTTG---CTGAGA---GCTATTGAGGCGCAACAGCATCTGTTGCAGCTCACAGTCTGGGGCATT
>EU023927
TTCTACTGTAATGTAACACCACTATTTAATAGTACTTGGAAGCCTGGTAATGAGACTGTGAATGATACT----
----

AAAGAAGAAGTCATCACACTCCCATGCAGGATAAAACAAATTATAAACCTGTGGCAGGAAGTAGGAAAAGCAA
TGTATGCCCCTCCCATCAGCGGAAAAATTAGATGTTCATCAAAT---
ATTACAGGGCTATTATTAACAAGAGATGGTGGTGTAAACGTGAGC----------------------------
----

GAAAATACTGAGATCTTCAGACCTGGAGGAGGAGATATGAGGGATAATTGGAGAAGTGAATTATATAAATATA
AAGTAGTAAAAATTGAACCATTA---GGAGTAGCACCCACCAGGGCAAAGAGAAGAGTGGTG-----------
----------------------------CAGAGAGAAAAAAGAGCA---ATGGGA---ATAGGAGCT------
---CTGTTCCTTGGGTTCTTGGGAGCA---GCAGGAAGCACTATG---GGCGCAGCGTCA---
GTAACGCTGACGGTACAG---GCCAGACTA---TTATTG---TCTGGTATAGTGCAACAGCAGAAC---
AACCTG---CTGAGG---GCTATTGAGGCGCAACAGCATGTGTTGCAGCTCACAGTCTGGGGCATC
>MH714351
TTCTACTGTAATACAACACAACTGTTTAATAGTACTTGGGATAGTACTGAAGGGTTGAATAAAACTGAAAGAA
ATGAA--

AATGACACAAAGATCATACTCCCATGCAGAATAAAACAATTTATAAACATGTGGCAGGAAGTAGGAAAAGCAA
TGTATGCCCCTCCCATCAGAGGACAAATTAAATGTATATCAAAT---
ATTACAGGGCTGATATTAACAAGAGATGGTGGTAATAACAATAATGGG-------------------------
----

AATAATGAGACCTTCAGACCTGTAGGAGGAGATATGAGGGACAATTGGAGAAGTGAATTATATAAATATAAAG
TAGTAAAAATTGAACCATTA---GGATTAGCACCCACCAGGGCAAAGAGAAGAGTGGTG--------------
-------------------------CAGAGAGAAAAAAGAGCA---GTGGGA---TTAGGAGCT---------
ATGTTCCTTGGGTTCTTGGGAGCA---GCAGGAAGCACTATG---GGCGCAGCGTCA---
GTAACGCTGACGGTACAG---GCCAGACTA---TTATTG---TCTGGTATAGTGCAACAGCAAAAC---
AATTTG---CTGAGG---GCTATTGAGGCGCAACAGCATCTGTTGCAACTCACAGTCTGGGGCATC
>MH714324
TTCTACTGTAATACATCACAACTGTTTAATAGTACTTGGAATACCTGGAATAGTACTGACTGGTCAAAAAAC-
----

ACTACAGAAAATATCACACTCCCATGCAGAATAAAACAAATTATAAACATGTGGCAGGAAGTAGGAAAAGCAA
TGTATGCCCCTCCCATCAGGGGACGAATTAAATGTTCATCAAAT---
ATTACAGGGTTGTTATTAACAAGAGATGGTGGTAACGGTACCAATACT-------------------------
----

ACCACCGAGGTCTTCAGACCTATAGGAGGAAATATGAAGGACAATTGGAGAAGTGAATTGTATAAATATAAAG
TGGTACAAATTGAACCATTA---GGAATAGCACCCACCAAGGCAAAGAGAAGAGTGGTG--------------
-------------------------CAGAGAGAAAAGCGAGCA---GTGGGA---ATAGGAGCT---------
ATGTTCCTTGGGTTCTTGGGAGCA---GCAGGAAGCACTATG---GGCGCAGCATCA---

ATGATGCTGACGGTACAG---GCCAGACAA---TTATTG---TCTGGTATAGTGCAACAGCAAAAC---
AATTTG---CTGAGA---GCCATTGAGGCGCAACAACATCTGTTGCAACTCACAGTCTGGGGCATC
>MH714343
TTCTACTGTAATACATCAAAACTGTTTGATAGTATTTGGAATGGTACTGAAAGGATAAATAACACTGAAGAA-
----

AATAGCACAATAATCACACTCCCATGCAGAATAAAACAAATTATAAACATGTGGCAGGAAGTAGGAAAAGCAA
TGTATGCCCCTCCCATCAGAGGACAAATTAGCTGTTCATCAAAC---
ATTACAGGGCTGATATTAACAAGAGATGGTGGTAATAGAACGGAGAATGGGACT-------------------
----

GAGAATGATAAAGAGATCTTCAGACCTGTAGGAGGAGATATGAGGGACAATTGGAGAAGTGAATTATATAAAT
ATAAAGTAGTAAAAATTGAACCATTA---GGAGTAGCACCCACCGAGGCAAAGAGAAGAGTGGTG--------
-------------------------------CAAAGAGAAAAAAGAGCA---GTGGGA---TTAGGAGCT---
------ATGTTCCTTGGGTTCTTGGGAGCA---GCAGGAAGCACTATG---GGCGCAGCGTCA---
ATAACGCTGACGGTACAG---GCCAGACTA---TTATTG---ACTGGTATAGTGCAACAGCAGAAC---
AATCTG---CTGAGG---GCTATTGAGGCGCAACAGCATCTGTTGCAACTCACAGTCTGGGGCATC
>MH714329
TTCTACTGTAACACAACAAAACTGTTTAACAGTACTTGGGATGCTAACAGTACTTGGGATGAAAATAATACTA
CAAATGAC---

AATGATGAAAATATCACACTCCCATGTAGAATAAAACAAATTATAAACCTGTGGCAGGAAGTAGGAAAAGCAA
TGTATGCCCCTCCCATCAGTGGACCAATTATCTGTAACTCAAGT---
ATTACAGGGCTCCTAATAACAAGGGATGGTGGTAATGACACAAAC----------------------------
----

CAGAACGAGACCTTCAGACCTGGAGGAGGAGATATGAGGGATAATTGGAGAAGTGAATTATATAAATATAAAG
TAGTAAAAATTGAACCATTA---GGAATAGCACCCACCAAGGCAAAAAGAAGAGTGGTG--------------
-------------------------CAGAGAGAAAAAAGAGCA---GTGGGA---ATAGGAGCT---------
GTGTTCCTTGGGTTCTTGTCAGCA---GCAGGAAGCACTATG---GGCGCAGCGTCA---
GTGACGCTGACGGTACAG---GCCAGACTA---TTATTG---TCTGGTATAGTGCAACAGCAGAAT---
AATTTG---CTGAGG---GCTATTGAGGCGCAGCAGCGTATGTTGCAACTCACAGTCTGGGGCATC
>KU600814
TTCTACTGTAATTCAACACCACTGTTTAATAGTACTTGGTATGGTAATAATACTGGGAATGGTACTGACGAAT
TAGGGAAC---

AGTGGAAATAACACAATTACACTACAATGCAGAATAAAACAAATTATAAACATGTGGCAGGAAGTAGGAAAAG
CAATGTATGCCCCTCCCATCAGAGGACGTATTAACTGTACATCAAAT---
ATTACAGGGTTGCTATTAACAAGAGATGGGGGAAATACTAATAATGATAGT----------------------
----

AGCGAGCCCGAGATCTTCAGACCTGGAGGAGGAAATATGAAGGACAATTGGAGAAGTGAATTATATAAATATA
AAGTAGTAAAAATTGAACCATTA---GGAGTAGCACCCACCAAGGCAAAGAGAAGAGTGGTG-----------
----------------------------CAGAGAGAAAAAAGAGCA---GTGGGA---ATAGGAGCT------
---CTGTTCCTTGGGTTCTTGGGAGCA---GCAGGAAGCACTATG---GGCGCAGCGTCA---
CTGACGCTGACGGTACAG---GCCAGACAA---TTATTG---TCTGGTATAGTGCAACAGCAGAAC---
AATTTG---CTGAGG---GCTATTGAGGCGCAACAGCATCTGTTGCAACTCACAGTCTGGGGCATC
>KU600818
TTCTACTGTAATACAACACAACTGTTTAATAGTACTTGGGAAAATCCTGAAAGGTCAAATAGCACTGAA----
----

GGAAATAGCACAATCACACTCCCATGCAGAATAAAACAAATTATAAACATGTGGCAGGAAGTAGGAAAAGCAA
TGTATGCCCCTCCCATCAGAGGACAAATTAAGTGTATATCAAAT---
ATTACAGGGTTGCTACTAACAAGAGATGGTGGTAATAATGGTACC----------------------------
----

ACGAACGAGACCTTCAGACCTGGAGGAGGAGATATGAGGAACAATTGGAGAAGTGAATTATATAAATATAAAG
TAGTAAAAATTGAACCATTA---GGAGTAGCACCCACCAAGGCAAAGAGAAGAGTGGTG--------------
-------------------------CAAAGAGAAAAAAGAGCA---GTGGGA---ATAGGAGCT---------
TTGTTCCTTGGGTTCTTGGGAGCA---GCAGGAAGCACTATG---GGCGCAGCGTCA---

ATGACGCTGACGGTACAG---GCCAGACAA---TTATTG---TCTGGTATAGTGCAACAGCAGAAT---
AATCTG---CTGAGG---GCTATTGAGGCGCAACAGCATATGTTGCAACTCACAGTCTGGGGCATC
>KU600815
TTCTACTGTAATACAACACAACTGTTTAATAGTACTTGGGATAAGGAGTCAAATAATACTTCGAAT-------
----

GAGACTGAAATAATCACACTCCCATGCAGAATAAAACAAATTATAAACATGTGGCAGGAAGTAGGAAAAGCAA
TGTATGCACCTCCCATCAGAGGAAACATTAGCTGTTCATCAAAT---
ATTACAGGGCTGCTATTAACAAGAGATGGTGGGGTGGTGAATGGTAGT-------------------------
----

AGCAATGAGACAGAGACCTTCAGACCTGGAGGAGGAAATATGAAGGACAATTGGAGAAGTGAATTATATAAAT
ATAAGGTAGTACAAATTGAACCATTA---GGACTAGCACCCACCACGGCAAAAAGAAGAGTGGTG--------
-------------------------------CAAAGAGAAAAAAGAGCA---GTGGGA---TTAGGAGCT---
------ATGTTCCTTGGGTTCTTGGGAGCA---GCAGGAAGCACTATG---GGCGCAGCGTCA---
ATAACGCTGACGGTACAG---GCCAGACAA---TTATTG---TCTGGTATAGTGCAACAGCAGAGC---
AATTTG---CTGAGG---GCTATTGAGGCGCAACAGCATCTGTTGCAACTCACAGTCTGGGGCATC
>AY180905
TTCTACTGTAATACATCACGACTGTTTAATAGTACTTGGAATAGTACTAGGAATAATATA-------------
----

GGAAATGGCACTATCACGCTCCCTTGCAGAATAAAACAAATTATAAACATGTGGCAGGAAGTAGGGAAAGCAA
TGTATGCCCCTCCCATTGCAGGAAATATTAGCTGTTCATCAAAT---
ATTACAGGGTTGTTATTAACAAGAGATGGTGGTAAGAATGAGAGC----------------------------
----

AACACCACCGAGACCTTCAGACCTGCAGGAGGAGATATGAGGGACAATTGGAGAAGTGAATTATATAAATATA
AAGTAGTAAGAATTGAACCATTA---GGAGTAGCACCCACCAAGGCAAAGAGAAGAGTGGTG-----------
----------------------------CAGAGAGAAAGAAGAGCA---GTGGGAGCGATAGGAGCT------
---ATGTTCCTTGGGTTCTTGGGAGCA---GCAGGAAGCACTATG---GGCGCAGCGTCA---
ATAACGCTGACGGTACAG---ACCAGACAA---TTGTTG---TCTGGTATAGTGCAACAGCAGAGA---
AATCTG---CTGAGG---GCTATTGAGGCGCAACAGCATCTGTTGCAACTCACAGTCTGGGGCATC
>EU363827
TTCTACTGTAATACATCACGACTGTTTAATAGTACTTGGAATGAGACTAGTGCTTGGAATGATACTACA----
----

GGAAATGACACTATCACGCTCCCTTGCAGAATAAAACAAATTGTAAACATGTGGCAGGAGGTAGGGAAAGCAA
TGTATGCCCCTCCCATTAGTGGACGCATTAAATGCTCATCAAAT---
ATTACAGGGTTACTATTGACAAGAGATGGTGGTAGGAATAACGAGAGC-------------------------
----

AACACCACCGAGACCTTCAGACCTGTAGGAGGAAATATGAGAGACAATTGGAGAAGTGAATTATATAAATATA
AAGTAGTAAAAATTGAACCATTA---GGATTAGCACCCACCAGGGCAAAGAGAAGAGTGGTG-----------
----------------------------CAGAGAGAAAAAAGAGCA---GTGGGA---ATAGGAGCT------
---ATGTTCCTTGGGTTCTTGGGAGCA---GCAGGAAGCACTATG---GGCGCAGCGTCA---
ATAACGCTGACGGTACAG---GCCAGACAA---TTGCTG---TCTGGTATAGTGCAACAGCAGAGA---
AATCTG---CTGAGT---GCTATTGAGGCGCAACAACATTTGTTGCAACTCACAGTCTGGGGCATC
>AY905493
TTCTATTGTAATACATCACAACTGTTCAATAGTACTTGGAATGGTAATACTACTTGGAATAATACTGCA----
----

GGAAATAGCAATATCACGCTCCCTTGCAGAATAAAACAAATTGTAAACATGTGGCAGGAAGTAGGGAAAGCAA
TGTATGCCCCTCCCATTAGAGGACAAATTAGATGTTCATCAAAT---
ATTACAGGGTTGCTATTAACAAGAGATGGTGGTACTAATAAGAGC----------------------------
----

GAGAACACCGAGACATTCAGACCTGGAGGAGGAGATATGAAAGACAATTGGAGAAGTGAATTATATAAATATA
AAGTAGTAAAAATTGAACCATTA---GGAATAGCACCCACCAAGGCAAAGAGAAGAGTGGTG-----------
----------------------------CAGAGAGAAAAAAGAGCA---GTGGGAACAATAGGAGCT------
---ATGTTCCTTGGGTTCTTGGGAGCA---GCAGGAAGCACTATG---GGCGCAGCGTCA---

ATAACGCTGACGGTACAG---GCCAGACAA---TTGTTG---TCTGGTATAGTGCAACAGCAGAGA---
AATTTG---CTGAGG---GCTATTGAGGCGCAACAGCATATGTTGCAACTCACAGTCTGGGGCATC
>JF932492
TTCTACTGTAATACATCACAACTGTTTAATAGTATTTGGTATAATAATGATACTTGGAATGGTACTACA----
----

GGAGATGACAATATCACACTCCCTTGCAGAATAAGACAAATTGTAAACCTATGGCAGGAAGTAGGGAAAGCAA
TGTATGCCCCTCCCATTAAAGGACAAATTAGATGTTCATCAAAT---
ATTACAGGGTTGCTATTAACAAGAGATGGTGGGACGAATGAGGGC----------------------------
----

AATACCAATGAGACCTTCAGACCTGGAGGAGGAGATATGAGAGACAATTGGAGAAGTGAATTATATAAATATA
AAGTAGTAAAAATTGAACCATTA---GGAGTAGCACCCACCAAGGCAAAGAGAAGAGTGGTG-----------
----------------------------CAGAGAGAAAAAAGAGCA---GTGGGAATGATGGGAGCT------
---ATGTTCCTTGGGTTCTTGGGAGCA---GCAGGAAGCACTATG---GGCGCAGCGTCA---
ATGACGCTGACGGTACAG---GCCAGACAA---TTATTG---TCTGGTATAGTGCAACAGCAAAGA---
AATTTG---CTGAGG---GCTATTGAGGCGCAACAGCATCTGTTGCAACTCACAGTCTGGGGCATT
>AY905497
TTCTACTGTAATACATCACAACTGTTTAATAGTATTAATGGTACCTGGAATGGTACTACA-------------
----

AAAAATGGCAATATCACGCTCCCTTGCAGAATAAGACAAATTGTAAACAGGTGGCAGGAAGTAGGGAAAGCAA
TGTATGCTCCTCCCATTAGCGGGCAAATTAGATGTTCATCAAAT---
ATTACAGGGTTACTATTAACAAGAGATGGTGGTACTAATGAGAGC----------------------------
----

AGCAACAACGAGACCTTCAGACCTGGAGGAGGAGATATGAGGGACAATTGGAGAAGTGAATTATATAAATATA
AAGTAGTAAAAATTGAACCATTA---GGAGTAGCACCCACCAAGGCAAGGAGAAGAGTGGTG-----------
----------------------------CAGAGAGAAAAAAGAGCA---GTGGGAACGATAGGAGCT------
---ATGTTCCTTGGGTTCTTGGGAGCA---GCAGGAAGCACTATG---GGCGCAGCGTCA---
ATAACGCTGACGGTACAG---GCCAGACAA---TTGTTG---TCTGGTATAGTGCAACAGCAGAGA---
AATTTG---CTGAGG---GCTATTGAGGCGCAACAGCATATGTTGCAACTCACAGTCTGGGGCATC
>AY905495
TTCTACTGTAATACAACAAAACTGTTTAATAGTATTTGGAATACTAATGGTACTTGGAATGATACTAGTACTT
GGAATAAT---
-------------------AGT---
ACAGGGAATGATACTATCACGCTCCCTTGCAGAATAAAACAAATTGTAAACATGTGGCAGGAAGTAGGGAAAG
CAATGTATGCCCCTCCCATTAGAGGACAAATTAGATGTTCATCAAAT---
ATTACAGGGCTGCTATTAACAAGAGATGGTGGTAATAATGGGACCAAC-------------------------
----

ACCACCGACACAGAGATCTTCAGACCTGCAGGAGGAGATATGAGGGACAATTGGAGAAGTGAATTATATAAAT
ATAAAGTAGTAAAAATTGAACCATTA---GGAGTAGCACCCACCAGGGCAAAGAGAAGAGTGGTG--------
-------------------------------CAAAGAGAAAAAAGAGCA---GTGGGAACAATAGGAGCT---
------ATGTTCCTTGGGTTCTTGGGAGCA---GCAGGAAGCACTATG---GGCGCAGCGTCA---
GTAACGCTGACGGTACAG---GCCAGACAA---TTGTTG---TCTGGTATAGTGCAACAGCAGAGA---
AATTTG---CTGAGG---GCTATTGAGGCGCAACAGCATATGTTGCAACTCACAGTCTGGGGCATT
>AY905494
TTCTACTGTAATACGACACAACTGTTTAATAGTACTTGGAAGTTTAATAGTACTTGGAATGATACTAGTACA-
----

GAGAATGGTACTATCATACTCCCTTGCAGAATAAAACAAATTGTAAACATGTGGCAGGAAGTAGGGAAAGCAA
TGTATGCCCCTCCCATTAGTGGGCAAATTAGATGTTCATCAAAT---
ATTACAGGGTTGCTATTAACAAGAGATGGTGGTAAGGAGGAGAAC----------------------------
----

AACACCACCGAGACCTTCAGACCTGGAGGAGGAGATATGAGAGATAATTGGAGAAGTGAATTATATAAATATA
AAGTAGTAAAAATTGAACCATTA---GGAGTAGCACCCACCAAGGCAAAGAGAAGAGTGGTG-----------
----------------------------CAGAGAGAAAAAAGAGCA---GTGGGAACGATAGGAGCT------
---ATGTTCCTTGGGTTTTTGGGAGCA---GCAGGAAGCACTATG---GGCGCAGCGTCA---

ATAACGCTGACGGTACAG---GCCAGACAA---TTGTTG---TCTGGTATAGTGCAACAGCAGAGA---
AATTTG---CTGAGG---GCTATTGAGGCGCAACAGCATATGTTGCAACTCACAGTCTGGGGCATC
>HM215397
TTCTACTGTAATACATCACAACTGTTTAATAGTACTTGGAATGTTACTGGTACTGTGAATGGTACTACA----
----

GGAAATGGCACTATCACGCTCCCTTGCAGCATAAAACAAATTGTAAACAGGTGGCAGGAAGTAGGGAGAGCAA
TATATGCCCCTCCCATTAGAGGACAAATTAGATGTTCATCAAAT---
ATTACAGGGTTGCTATTAACAAGAGATGGTGGTAATGAGAGCACGACC-------------------------
----

ACCGAGACCGAGACCTTCAGACCTGCAGGAGGAGATATGAGAGACAATTGGAGAAGTGAATTATATAAATATA
AAGTAGTAAAAATTGAACCATTA---GGAGTAGCACCCACCAAGGCAAAGAGAAGAGTGGTG-----------
----------------------------CAGAGAGAAAAAAGAGCA---GTGGGAACGATAGGAGCT------
---ATGTTCCTTGGGTTCTTGGGAGCA---GCAGGAAGCACTATG---GGCGCAGCGTCA---
ATCACGCTGACGGTACAG---GCCAGACAA---TTGTTG---TCTGGTATAGTGCAACAGCAGAGA---
AATTTG---CTGAGG---GCTATTGAGGCGCAACAGCATCTGTTGCAACTCACAGTCTGGGGCATC
>HQ699990
TTCTATTGTGATACATCACAACTGTTTAATAATATTTGGTTTAATAGTACTTGGAATGATAGTACTTGGAATA
AT---
-------------------ACT---
ACAGGAGATAGCAACATCACGCTCCCCTGCAGAATAAAACAAATTGTAAACATGTGGCAGGGAGTAGGGAAAG
CAATGTATGCCCCTCCCATTAAAGGACAAATTAAATGTTCATCAAAT---
ATTACAGGGTTGCTATTAACAAGAGATGGTGGTAAAAATGTGAGC----------------------------
----

AATACCAGCGAGACCTTCAGACCTGGAGGAGGAGATATGAAAGACAATTGGAGAAGTGAATTATATAAATATA
AAGTAGTAAAAATTGAACCATTA---GGAGTAGCACCCACCAAGGCAAAGAGAAGAGTGGTG-----------
----------------------------CAGAGAGAAAAAAGAGCA---GTGGGA---ATGGGAGCT------
---ATGTTCCTTGGGTTCTTGGGAGCA---GCAGGAAGCACTATG---GGCGCAGCGTCA---
ATAACGCTGACGGTACAG---GCCAGACAA---TTGTTG---TCTGGTACAGTGCAGCAGCAGAGA---
AATTTG---CTGAGG---GCTACTGAGGCGCGACAGCATCTGTTGCAACTCACGGTCTGGGGCATC
>HQ699996
TTCTACTGTAATACATCACAACTGTTTAATAGTACTTGGAGTTGGAATAATACTAATACTGAGAATAATACTA
CAGAA--

AACAGCAATATCACTATCACGCTCCCTTGCAGAATAAAACAAATTGTAAACATGTGGCAGGAAGTAGGGAAAG
CAATGTATGCCCCTCCCATTGCAGGACAAATTAGATGTTCATCAAAT---
ATTACAGGGTTGCTATTAACAAGAGATGGTGGCAACAGTACCAAC----------------------------
----

GAGACCTTCAGGCCTGAAGGAGGAAATATGAGAGACAATTGGAGAAGTGAATTATATAAATATAAAGTAGTAA
GAATTGAACCATTA---GGAGTAGCACCCACCAAGGCAAAGAGAAGAGTGGTG--------------------
-------------------CAGAGAGAAAAAAGAGCA---GTGGGAACGATAGGAGCT---------
ATGTTCCTTGGGTTCTTGGGAGCA---GCAGGAAGCACTATG---GGCGCAGCGTCA---
ATGACGCTGACGGTACAG---GCCAGACAA---TTGTTG---TCTGGTATAGTGCAACAGCAAAGA---
AATTTG---CTGAGG---GCTATTGAGGCGCAACAGCATCTGTTGCAACCCACAGTCTGGGGCATC
>EU131797
TTCTACTGTAATACATCACAACTGTTTAATAGTATTTGGTATAATAATGGTACTTGGAATGGTACTACA----
----

GGAAATGACACTATCACGCTCCCTTGCAGAATAAAACAAATTGTAAACATGTGGCAGGAAGTAGGGAAAGCAA
TGTATGCCCCTCCCATTGAAGGACAAATTAGTTGTAAATCAAAT---
ATTACAGGGTCGCTATTAACAAGAGATGGTGGTAATAATGGGAGC----------------------------
----

AGGACCAACGAGACCTTCAGACCTACAGGAGGAGATATGAGAGACAACTGGAGAAGTGAATTATATAAATATA
AAGTAGTAAAAGTTGAACCATTA---GGAGTAGCACCCACCAAGGCAAAGAGAAGAGTGGTG-----------
----------------------------CAGAGAGAAAAAAGAGCA---GTGGGAACGATAGGAGCT------
---ATGTTCCTTGGGTTCTTGGGAGCA---GCAGGAAGCACTATG---GGCGCAGCGTCA---

ATGACGCTGACGGTACAG---GCCAGACAA---TTGTTG---TCTGGTGTAGTGCAACAGCAGAGA---
AATTTG---CTGAGG---GCTATTGAGGCGCAACAGCATATGTTGCAACTCACAGTCTGGGGCATC
>EU131791
TTCTACTGTAATACATCACAACTGTTTAAGAGTACTTGGAATGTTACTACTGGTACTCCGAATGGTACTACA-
----

GGAAATGACAATATCACGCTCCCTTGCAGAATAAGACAAATTGTAAACATGTGGCAGGAAGTAGGGAAAGCAA
TGTATGCCCCTCCCATTACAGGAGTAATTAGCTGTTCATCAAAT---
ATTACAGGGTTACTATTAACAAGAGATGGTGGTAAAAATGTGACCAAT-------------------------
----

AATGACACCGAGACCTTCAGACCTGGAGGAGGAGATATGAGAGGCAATTGGAGAAGTGAATTATATAAATATA
AAGTAGTAAAAATTGAACCATTA---GGAATAGCACCCACCAAGGCAAAGAGAAGAGTGGTG-----------
----------------------------CAGAGAGAAAAAAGAGCA---GTGGGAGTAATAGGAGCT------
---ATGTTCCTTGGGTTCTTGGGAGCA---GCAGGAAGCACTATG---GGCGCGGCGTCA---
ATAACGCTGACGGTACAG---GCCAGACAA---ATGTTG---TCTGGTATAGTGCAACAGCAGAGA---
AATTTG---CTGAGG---GCTATTGAGGCGCAACAGCACATGTTGCAACTCACAGTCTGGGGCATC
>EU131790
TTCTACTGTAATACATCACAACTGTTTAATAGTACTTGGCATAATAATGGTACTTGGGGTGATACTACA----
----

GGAAATAGCTCTATCACGCTCCCTTGCAGAATAAAACAAATTGTAAACATGTGGCAGGAAGTAGGGAAAGCAA
TGTATGCTCCTCCCATTGAAGGGCAAATTAGATGTTCATCAAAT---
ATTACAGGGTTGATATTAACAAGAGATGGTGGTAAAAATGAGAGC----------------------------
----

GCCACCGAGACCTTCAGACCTGAGGGAGGAGATATGAGAGACAATTGGAGAAGTGAATTATATAAATATAAAG
TAGTAAAAATTGAACCATTA---GGACTAGCACCCACCAAGGCAAAGAGAAGAGTGGTG--------------
-------------------------CAAAGAGAAAAAAGAGCA---GTGGGAACAATAGGAGCT---------
ATGTTCCTTGGGTTCTTGGGAGCA---GCAGGAAGCACTATG---GGCGCAGCGTCA---
ATGACGCTGACGGTACAG---GCCAGACAA---TTGTTG---TCTGGTATAGTGCAACAGCAGAGA---
AATCTG---CTGAGG---GCTATTGAGGCGCAACAGCATATGTTGCAACTCACAGTCTGGGGCATC
>EU131788
TTCTACTGTAATACATCACAACTGTTTAATAGTACTTGGAATAATAATGGTACTTGGAATGATACTACA----
----

GACAATAGCACTATCACACTCCCTTGCAGAATAAAACAAATTATAAATAGGTGGCAGGAAGTAGGGAAAGCAA
TGTATGCCCCTCCCATTAGTGGACTAATTAGATGCTCATCAAAT---
ATTACAGGGTTACTATTAACAAGAGATGGTGGTACTAATAAGAGC----------------------------
----

AGCACCACCGAGACCTTCAGACCTGCAGGAGGAAATATGAGAGACAATTGGAGAAGTGAATTATATAAATATA
AAGTAGTACAAATTGAACCATTA---GGAGTAGCACCCACCAAGGCTAAGAGAAGAGTGGTG-----------
----------------------------CAAAGAGAAAAAAGAGCA---GTGGGAGCAATAGGAGCT------
---ATGTTCCTTGGGTTCTTGGGAGCA---GCAGGAAGCACAATG---GGCGCAGCGTCA---
ATAACGCTGACGGTACAG---GCCAGGCAA---TTGCTG---TCTGGTATAGTGCAACAGCAGAGA---
AATTTG---CTGAGG---GCTATTGAGGCGCAACAGCATATGTTGCAACTCACAGTCTGGGGCATA
>EU131808
TTCTACTGTAATACAACACAACTGTTTAGTAGTACTTGGTATGAGAATGGTACTTGGAATGGGACTAGTAATT
GGAATGGG---
-------------------ACT---
ACAGGAAATGACATTATCACACTCCCTTGCAGAATAAAACAAATTGTAAACATGTGGCAGGAAGTAGGGAAAG
CAATGTATGCCCCTCCTATTGAAGGACAAATTAGATGTTCATCAAAT---
ATTACAGGGCTGCTATTAACAAGAGATGGTGGCAATAATGAGAGC----------------------------
----

AGTGCCAATGAGACCTTCAGACCTGCAGGAGGAAATATGAAAGACAATTGGAGAAGTGAATTATATAAATATA
AAGTAGTAAAAATTGAACCATTA---GGAGTAGCACCCACCAAGGCAAAAAGAAGAGTGGTG-----------
----------------------------CAGAGAGGAAAAAGAGCA---GTGGGAACGATAGGAGCT------
---ATGTTCCTTGGGTTCTTGGGAGCA---GCAGGAAGCACTATG---GGCGCAGCGTCA---

ATAACGCTGACGGTACAG---GCCAGACAA---ATGTTG---TCTGGTATAGTGCAACAGCAAAGA---
AATCTG---CTGAGG---GCTATTGAGGCGCAACAGCATCTGTTGCAACTCACAGTCTGGGGCATC
>EU131789
TTCTACTGTAATACATCACAACTGTTTAATAGTACTTGGGATAGAAATAGTAGTACTTGGAGTAATACTACAG
GA---

CATAACAATAGCACTATCACACTCCCTTGCAGAATAAAACAAATTGTAAACAGGTGGCAGGAAGTAGGGAAAG
CAATGTATGCCCCTCCCATTAGCGGACTAATTAGATGCTCATCAAAG---
ATTACAGGGTTATTATTAACAAGAGATGGTGGTACTAATGAGAACAACACC----------------------
----

ATCGAGAACACCACCGAGATCTTCAGACCTGGAGGAGGAGATATGAGAGACAATTGGAGAAGTGAATTATATA
AATATAAAGTAGTACAAATTGAACCATTA---GGAGTAGCACCCACCAAGGCAAAGAGAAGGGTGGTG-----
----------------------------------CAGAGAGAAAAAAGAGCA---
GTGGGAACAATAGGAGCT---------ATGTTCCTTGGGTTCTTGGGAGCA---GCAGGAAGCACTATG---
GGCGCAGCGTCA---ATAACGCTGACGGTACAG---GCCAGACAA---TTATTG---
TCTGGTATAGTGCAACAGCAGAGA---AATTTG---CTGAGG---
GCTATTGAGGCGCAACAGCATATGTTGCAACTCACAGTCTGGGGCATA
>MK053935
TTCTACTGTAATACAACACAACTGTTTAATAGTACTTGGAATGAGAATAGTACTTGGAATGAGACTAGTACT-
----

GAGAATAATATTATCACACTCCCTTGCAGAATAAAACAAATTGTAAACATGTGGCAGGAAGTAGGGAAAGCAA
TGTATGCCCCTCCCATTAGCGGACAAATTAGATGTTCATCAAAT---
ATTACAGGATTACTATTAACAAGAGATGGTGGTAATGAGACCAAC----------------------------
----

ACCACCGAGATTTTCAGACCTGGAGGAGGAGATATGAGAGACAATTGGAGAAGTGAATTATATAAATATAAAG
TAGTAAAAATTGAACCATTA---GGAGTAGCACCCACCAAGGCAAAGAGAAGAGTGGTG--------------
-------------------------CAGAGAGAAAAAAGAGCA---GTAACA---CTTGGAGCT---------
ATGTTCCTTGGGTTCTTGGGAGCA---GCAGGAAGCACTATG---GGCGCAGCGTCA---
ATAACGCTGACGGTACAG---GCCAGACAA---TTGTTG---TCTGGTATAGTGCAACAGCAGAGA---
AATTTG---CTGAGG---GCTATTGAGGCGCAACAGCATCTGTTGCAACTCACAGTCTGGGGCATC
>DQ990880
TTCTACTGTAATACCTCACGACTGTTTAATAGTACTTGGTATAATAATGGTACTTGGAATAATGCTAGTACTG
GG---

AATGATAGCACTATTATACTCCCTTGCAGAATAAAACAAATTGTAAACAGGTGGCAGGAAGTAGGGAAAGCAA
TATATGCCCCTCCCATTGCAGGACCAATTAATTGTTCATCAAAA---
ATTACAGGGTTACTATTAACAAGAGATGGTGGGATTAATAAGACT----------------------------
----

GACACCGAGACCTTCAGACCTGGAGGAGGAGATATGAGAGACAATTGGAGAAGTGAATTATATAAATATAAAG
TAGTGAAAATTGAACCATTA---GGAGTAGCACCCACCAGGGCAAAGAGAAGAGTGGTG--------------
-------------------------CAAAGAGAAAAAAGAGCA---GTGGGAGCAATAGGAGCG---------
ATGTTCCTTGGGTTCTTGGGAGCA---GCAGGAAGCACTATG---GGCGCAGCGTCA---
ATAACGCTGACGGTACAG---GCCAGACAA---TTGTTG---ACTGGTATAGTGCAACAGCAAAGA---
AATTTG---CTGAGA---GCTATTGAGGCGCAACAGCATCTGTTGCAACTCACAGTCTGGGGCATC
>HM215420
TTTTATTGTAATACATCACAACTGTTTAATGAAACTTGGACTTGGAATAATAGTACTAGGAATGATAATACA-
----

GGAAATGGCACTATCACGCTCCCTTGCAGAATAAAACAAATTGTAAACATGTGGCAGGAAGTAGGGAAAGCAA
TGTATGCCCCTCCCATTAGAGGACAAATTAGATGTTCATCAAAT---
ATTACAGGGTTACTATTAACAAGAGATGGTGGTAATAATACCGAC----------------------------
----

AAAAACACCGAGATCTTCAGACCTGGAGGAGGAAACATGAGAGACAATTGGAGAAGTGAATTATATAAATATA
AAGTAGTAAAAATTGAACCATTA---GGAGTAGCACCCACCAAGGCAAAGAGAAGAGTGGTG-----------
----------------------------CAGAGAGAAAAAAGAGCA---GTGGGA---ATAGGAGCT------

---GTGTTCCTTGGGTTCTTGGGAGCA---GCAGGAAGCACTATG---GGCGCAGCGTCA---
ATAACGCTGACGGTACAG---GCCAGACAA---TTGTTG---TCTGGTATAGTGCAGCAGCAGAGA---
AATTTG---CTGATG---GCTATTGAGGCGCAACAGCATCTGTTGCAACTCACAGTCTGGGGCATC
>HQ699995
TTCTACTGTAATACATCACAACTGTTTAATAGTACTTGGTATAATAATGGCACTTGGGATGGTACTACA----
----

GAAAATGGCAATATCACGCTCCCTTGCAGAATAAAACAATTTGTAAACATGTGGCAGGGAGTAGGGAGAGCAA
TGTATGCCCCTCCCATTACAGGAAAGATTACATGTTTATCAAAT---
ATTACAGGGTTGATATTAACAAGAGATGGTGGGAATAATAAGACC----------------------------
----

AACACCACCGAGACCTTCAGACCTGAAGGAGGAAATATGAAAGACAATTGGAGAAGTGAATTATATAAATATA
AAGTAGTAAAAATTGAACCATTA---GGAGTAGCACCCACCAAGGCAAAGAGAAGAGTGGTG-----------
----------------------------CAGAGAGAAAAAAGAGCA---GTGGGAGCACTAGGAGCT------
---ATGTTCCTTGGGTTCTTGGGAGCA---GCAGGAAGCACTATG---GGCGCAGCGTCA---
ATAACGCTGACGGTACAG---GCCAGACAA---TTGTTG---TCTGGTATAGTGCAGCAGCAAAGA---
AATTTG---CTGAGA---GCTATTGAGGCGCAACAGCATCTGTTGCAACTCACAGTCTGGGGCATC
>HM215428
TTCTACTGTAATACATCACAACTGTTTAACAGTACTTGGTATGTGAATGGTACTTGGAGTGGTACTACA----
----

GAAAATGGCACTATCACACTCCCTTGCAGAATAAAACAAATTGTAAACATGTGGCAGGCAGTAGGGAAAGCAA
TGTATGCCCCTCCCATTGCAGGAAAAATTAGATGTTCATCAAAT---
ATTACAGGGCTGCTATTAACAAGAGATGGTGGTATTAATGTGAGC----------------------------
----

AATGACACAGAGACCTTCAGACCTGGAGGAGGAAATATGAAAGACAATTGGAGAAGTGAATTATATAAATATA
AAGTAGTAAGAATTGAACCATTA---GGAGTAGCACCCACCAAGGCAAAGAGAAGAGTGGTG-----------
----------------------------CAGAGAGAAAAAAGAGCA---GTGGGAACAATAGGAGCT------
---ATGTTCCTTGGGTTCTTGGGAGCA---GCAGGAAGCACTATG---GGCGCAGCGTCA---
ATAACGCTGACGGTACAG---GCCAGACAA---TTGTTG---TCTGGTATAGTGCAACAGCAGAGA---
AATTTG---CTGATG---GCTATTGAGGCGCAACAGCATATGTTGCAACTCACAGTCTGGGGCATC
>EU363828
TTCTACTGTAATACATCACAACTGTTTAATAGTACTTGGAATGTTACTACAGAAAATAGCAATATTACA----
----

GGAAATGACACTATCACGCTCCCTTGCAGAATAAAACAAATTATAAACATGTGGCAGGAAGTAGGGAGAGCAA
TGTATGCCCCTCCCATTAGTGGACAAATTAGATGCTCATCAAAT---
ATTACAGGGTTGCTATTAACAAGGGATGGTGGTAATAAGACCAACGAG-------------------------
----

ACCGAAGACACCGAGATCTTCAGACCTGGAGGAGGAGATATGAGAGACAATTGGAGAAGTGAATTATATAAAT
ATAAAGTAGTACAAATTGAACCATTA---GGCGTAGCACCCACCAAGGCAAAGAGAAGAGTGGTG--------
-------------------------------CAGAGAGAGAAAAGAGCA---GTGGGAACGATAGGAGCT---
------ATGTTCCTTGGGTTCTTGGGAGCA---GCAGGAAGCACTATG---GGCGCAGCGTCA---
ATGACGCTGACGGTACAG---GCCAGACAA---TTGTTG---TCTGGTATAGTGCAACAGCAGAGA---
AATTTG---CTGAGG---GCTATTGAGGCGCAACAACATCTGTTGCAACTCACAGTCTGGGGCATC
>MF591612
TTCTACTGTAATACATCAAAACTGTTTAATAGTACTTGGAATGGTAATAGTACTTGGAATGATACTACA----
----

GGAGATGATCCTATCACGCTCCCTTGCAGAATAAAACAAATTGTAAACATGTGGCAGGAAGTAGGGAAAGCAA
TGTATGCCCCTCCCATTAGAGGACAAATTAGATGCTCATCAAAT---
ATTACAGGGTTGCTATTAACAAGAGATGGTGGTAATAATACGAACGAGACCACC-------------------
----

GAGGAGACCGAGACCTTCAGACCTGGAGGAGGAGATATGAGAGACAATTGGAGAAGTGAACTATATAAATATA
AAGTAGTAAAAATTGAACCATTA---GGAGTAGCACCCACCAAGGCAAAGAGAAGAGTGGTG-----------
----------------------------CAGAGAGAAAAAAGAGCA---GTGGGAACGATAGGAGCT------

---ATGTTCCTTGGGTTCTTGGGAACA---GCAGGAAGCACTATG---GGCGCAGCGTCA---
ATAACGCTGACGGTACAG---GCCAGACAG---TTGTTA---TCTGGTATAGTGCAACAGCAGAGA---
AATTTG---CTGAGG---GCTATTGAGGCGCAACAGCATCTGTTGCAACTCACAGTCTGGGGCATC
>JF932482
TTCTACTGTAATACATCACAACTGTTTAATAGTACTTGGGATTCAAATGGTACTAATACTTGGACA-------
----

GAAAATGGCACTATCACGCTCCCTTGCAGAATAAAACAGATTGTAAACATGTGGCAGGAAGTAGGGAAAGCAA
TGTATGCCCCTCCCATTAGCGGACAAATTAACTGTTCATCAAAT---
ATTACAGGGTTGCTATTAACAAGAGATGGTGGTAAAAATGAGAGC----------------------------
----

AATACCACCGAGACCTTCAGACCTGGAGGAGGAGATATGAGAGACAATTGGAGAAGTGAATTATATAAATATA
AAGTAGTAAAAATTGAACCATTA---GGAGTAGCACCCACCAAGGCAAAGAGAAGAGTGGTG----------
----------------------------CAGAGAGAAAAAAGAGCA---GTGGGAACGATAGGAGCT------
---ATGTTCCTTGGGTTCTTGGGAGCA---GCAGGAAGCACTATG---GGCGCAGCGTCA---
ATAACGCTGACGGTACAG---GCCAGACAA---TTGTTG---TCTGGTATAGTGCAACAGCAGAGA---
AATTTG---CTGATG---GCTATTGAGGCGCAACAGCATCTGTTGCAACTCACAGTCTGGGGCATC
>HQ699949
TTCTACTGTAATACATCACAACTGTTTGATAGTACTTGGTGGAATAATGATACTAGTACTTGGAATGGTACTG
CA---

GAAAATAATACTATCACGCTCCCTTGCAGAATAAAACAAATTGTAAACATGTGGCAGGAAGTAGGAAGAGCAA
TGTATGCCCCTCCCATTGAAGGACGAATTAGATGTTCATCAAAT---
ATTACAGGGTTGCTATTAACAAGAGATGGTGGTGATGATGAGAAC----------------------------
----

ACCACTGAGATCTTCAGACCTGGAGGAGGAAATATGAGGGACAATTGGAGAAGTGAATTATATAAATATAAAG
TAGTAAAAATTGAACCATTA---GGAGTAGCACCCACCAAGGCAAAGAGAAGAGTGGTG--------------
-------------------------CAGAGAGAAAAAAGAGCA---GTGGGAACGATAGGAGCT---------
ATGTTCCTTGGGTTCTTGGGAGCA---GCAGGAAGCACTATG---GGCGCAGCGTCA---
ATAACGCTGACGGTACAG---GCCAGACAA---TTGTTG---TCTGGTATAGTGCAACAGCAGAGA---
AATCTG---CTGAGA---GCTATTGAGGCGCAACAACATATGTTGCAACTCACAGTCTGGGGCATC
>HM215423
TTCTACTGTAATACATCACAACTATTTAATAGTACTTGGCATGTTAATGGTACTTGGAATGGTACTACA----
----

GAAAATGGCACTATCACGCTCCCTTGCAGAATAAAACAAATTGTAAACATGTGGCAGGAAGTAGGAAAAGCAA
TGTATGCCCCCCCCATTAGCGGGAAAATCAGATGTTCATCAAAA---
ATTACAGGGTTGCTATTAACAAGAGATGGTGGTGCTAATGAAAGC----------------------------
----

GACCTCATCTTCAGACCTACAGGAGGAGATATGAGGGACAATTGGAGAAGTGAATTATATAAATATAAAGTAG
TAAAAATTGAACCATTA---GGAATAGCACCCACCAAGGCAAAGAGAAGAGTGGTG-----------------
----------------------CAGAGAGAAAAAAGAGCA---GTGGGAACAATAGGAGCT---------
ATGTTCCTTGGGTTCTTGGGAGCA---GCAGGAAGCACTATG---GGCGCAGCGTCG---
GTAACGCTGACGGTACAG---GCCAGACAA---TTGTTG---TCTGGTATAGTGCAACAGCAGAGA---
AATCTG---CTGATG---GCTATTGAGGCGCAACAGCATTTGTTGCAACTCACCGTCTGGGGCATC
>EU131805
TTCTACTGTAATACATCAAAACTGTTTAGTAGTATTTGGCATGACAATGGTACTAGTATTTGGAATGATACTA
TA---

AAAGATGAAAATATCACACTCCCTTGCAGAATAAAACAAATTATAAACATGTGGCAGCAAGTAGGAAAAGCAA
TGTATGCCCCTCCCATTAGAGGACAAATTAGATGTTCATCAACT---
ATTACAGGGTTGCTATTAACAAGAGATGGTGGCAATGATACCAGT----------------------------
----

AACAACGAGACCTTCAGACCAACAGGAGGAAATATGAGAGACAATTGGAGAAGTGAATTATATAAATATAAAG
TAGTAAAAATTGAACCATTA---GGAATAGCACCCGCCAGGGCAAAGAGAATAGTGGTG--------------
-------------------------CAGAGAGAAAAAAGAGCA---GTGGGAACAATAGGAGCT---------

ATGTTCCTTGGGTTCTTGGGAGCA---GCCGGAAGCACTATG---GGCGCAGCGTCA---
ATAACGCTGACGGTACAG---GCCAGACAA---ATGTTG---TCTGGTATAGTGCAACAGCAGAGA---
AATTTG---CTGATG---GCTATTGAGGCGCAACAGCATATGTTGCAACTCACAGTCTGGGGCATC
>EU131800
TTCTACTGTAATACATCACAACTGTTTAATAGTACTTGGACTTGGAATGATACTAATAATTGGACTAATACTA
CA---

GAAAAGGGCAATATCACACTCCCATGCAAAATAAAACAAATTGTAAACATGTGGCAGGAAGTAGGGAAAGCAA
TGTATGCCCCTCCCATTGCAGGACAGATTAGATGTTCATCAAAT---
ATTACAGGGTTGCTATTAACAAGAGATGGTGGTAAGAATGAGAGC----------------------------
----

AACACCACCGAGACCTTCAGACCTGGAGGAGGAGAGATGAGAGATAATTGGAGAAGTGAATTATATAAATATA
AAGTAGTAAAAATTGAACCTTTA---GGAGTAGCACCCACCAAGGCAAAGAGAAGAGTGGTG-----------
----------------------------CAGAGAGAAAAAAGAGCA---GCGGGAACGTTAGGAGCT------
---ATGTTCCTTGGGTTCTTGGGAGCA---GCAGGAAGCACTATG---GGCGCAGCGTCA---
ATGACGCTGACGGTACCG---GCCAGACAA---TTGTTG---TCTGGTATAGCGCAACAGCACAGA---
AATTTG---CTGAGA---GCTATTGAGGCCCAACAGCATATGCTGCAACTCACAGCCTGGGGCATC
>EU131794
TTCTACTGTGATACATCACAATTGTTTATTAGTACTTGGAATAATACTAGTACTTGGGAGAATACTACA----
----

GGAAATAGCGATATCACGCTCCCTTGCAGAATAAAACAAATTATAAATATGTGGCGGGAAGTAGGGAAAGCAA
TGTATGCCCCTCCCATTGAAGGACAAATTAGATGTTCATCAAAT---
ATTACAGGGTTACTATTAACAAGAGATGGTGGTAATAAGAACGAGACC-------------------------
----

ACCAAGACGGAGACCTTCAGGCCTGGAGGAGGAGATATGAGAGATAATTGGAGAAGTGAATTATATAAGTATA
AAGTAGTAAAAATTGAACCACTA---GGAGTAGCACCCACCAAGGCAAAGAGAAGAGTGGTG-----------
----------------------------CAGAGAGAAAAAAGAGCA---GTGGAAACGATAGGAGCT------
---GTGTTCCTTGGGTTCTTGGGAGCA---GCAGGAAGCACTATG---GGCGCAGCGTCA---
ATAACGCTGACGGCACAG---GCCAGACAA---TTGTTG---TCTGGTATAGTGCAACAGCAGAGA---
AATTTG---CTGAGG---GCTATTGAGGCGCAACAGCATCTGTTGCAACTCACAGTCTGGGGCATC
>EU131807
TTCTACTGCAATACATCACAACTGTTTAATAGTACTTGGAATAATAATACTAGTACTTGGACCAATACTACA-
----

GACAATAGCACTATCACACTTCCTTGCAGAATAAAACAAATTGTAAACATGTGGCAGGAAGTAGGGAAAGCAA
TGTATGCCCCTCCCATTGAAGGACAAATCAGATGTTCATCAAAT---
ATTACAGGGTTACTATTAACAAGAGATGGTGGTGATAATAAGGAC----------------------------
----

AGTACCACTGAGACCTTCAGATCTGGAGGAGGAGATATGAGAGACAATTGGAGAAGTAAATTATATAAATATA
AAATAGTAAAAATTGGACCACTA---GGAGTAGCACCCACCAAGGCAAAGAGAAGAGTGGTG-----------
----------------------------CAGAGAGAAAAAAGAGCA---GTGGGAACAATAGGAGCT------
---ATGTTCCTTGGGTTCTTGGGAGCA---GCAGGAAGCACGATG---GGCGCAGCGTCA---
ATAACGCTAACGGTACAG---GCCAGACAA---TTGTTG---TCTGGTATAGTGCAACAGCAGAGA---
AATTTG---CTGAGG---GCTATTGAGGCGCAACAGCATCTGTTGCAACTCACAGTCTGGGGCATC
>EU131809
TTCTACTGTAATACAACAAAGCTATTTAATAATACTTTGCAGGATAATAATACTTGGAATAGTCCTTGGCAAG
GG---

TCAAATGACACTATCACACTCCAATGCAGAATAAAACAAATTATAAACAGATGGCAGGAAGTAGGAAAAGCAA
TGTATGCCCCTCCCATCAGGGGACAAATTAGATGCTCATCAAAT---
ATTACAGGACTGCTACTAACAAGAGATGGTGGTAACGAAAGCGGG----------------------------
----

TCCGAAATCTTCAGACCTGGAGGAGGAAATATGAGGGACAATTGGAGAAGTGAATTATATAAATATAAAGTAG
TAAAAATTGAGCCATTA---GGACTAGCACCCACCAAGGCAAAAAGAAGAGTGGTG-----------------
----------------------CAGAGAGAAAAAAGAGCA---GTGGGAACACTAGGAGCT---------

CTGTTCCTTGGGTTCTTGGGAGCA---GCAGGAAGCACTATG---GGCGCAGCGTCA---
ATCACGCTGACGGTACAG---GCCAGACTA---TTACTG---TCTGGTATAGTGCAACAGCAGAAC---
AATTTG---CTGAGA---GCTATTGAGGCGCAACAGCATATGTTGCAACTCACAGTCTGGGGCATC
>MH479968
TTCTACTGTAATACATCAGAACTGTTT------AATTGGAATGATACTAGTCCTTGGAAGAAT----------
----

ATCACAAGAAATATCACGCTCCCTTGCAGAATAAGACAAATTGTAAACATGTGGCAGGAAGTAGGGAAAGCAA
TGTATGCCCCTCCCATTAGAGGAAAAATTAGATGTTCATCAAAT---
ATTACAGGGTTGCTATTAACAAGAGATGGTGGTGAGAATGAGAGC----------------------------
----

AATGTCACCGAGACCTTCAGACCTGGAGGAGGAAATATGAAGGACAATTGGAGAAGTGAATTATATAAATATA
AAGTAGTAAAAATTGAACCATTA---GGAGTAGCACCCACCAAGGCAAAGAGAAGAGTGGTG-----------
----------------------------CAGAGAGAAAAAAGAGCA---GTGGGAACAATAGGAGCT------
---ATGTTCCTTGGGTTCTTGGGAGCA---GCAGGAAGCACTATG---GGCGCAGCGTCA---
ATAACGCTGACGGTACAG---GCCAGACAA---TTATTG---TCTGGTATAGTGCAACAGCAGAGA---
AATTTG---CTGAGG---GCTATTGAGGCGCAACAGCATCTGTTGCAACTCACAGTCTGGGGCATC
>JF932468
TTCTACTGTAATACATCACAACTGTTTAATGGTACTTGGAATAATAATGGTACTTGGAGTGGTACTGCTTGGA
ATACTACA---

GGAAATAACACTATCACGCTCCCTTGCAGAATAAAACAAATTGTAAACATGTGGCAGGAAGTAGGGAAAGCAA
TATACGCCCCTCCCATTAGAGGACAAATCAACTGTTCATCAATT---
ATTACAGGGTTACTATTAACAAGAGATGGTGGTAAGAATGTGAGC----------------------------
----

AACACCACCGAGATCTTCAGACCTGCAGGGGGAGATATGAGAGACAATTGGAGAAGTGAATTATATAAATATA
AAGTAGTAAAAATTGAACCATTA---GGAATAGCACCCACCAAGGCAAAGAGAAGAGTGGTG-----------
----------------------------CAAAGAGAAAAAAGAGCA---GTGGGAACGATAGGAGCT------
---ATGTTCCTTGGGTTCTTAGGAGCA---GCAGGAAGCACTATG---GGCGCAGCGTCA---
ATAACGCTGACGGTACAG---GCCAAACAA---TTGTTG---TCTGGTATAGTGCAGCAGCAGAGA---
AATTTG---CTGAAT---GCTATTGAGGCGCAACAACGTCTGTTGCAACTCACAGTCTGGGGCATC
>JF932470
TTCTACTGTAATACAACAAAATTGTTTAATAGTACTTGGAATGATACTAGTACTGAGAATGTTACTACA----
----

GAAAATAGCACTATCGTACTCCATTGCAGAATAAGACAAATTGTAAACATGTGGCAGGAAGTAGGGAAAGCAA
TGTATGCCCCTCCCATTGAAGGACGAATTAGATGTTCATCAAAC---
ATTACAGGATTGCTATTAACAAGAGATGGTGGTAATAATAAAGGC----------------------------
----

AACACCACCGAGACCTTCAGACCTGGAGGAGGAAATATGAAAGACAATTGGAGAAGTGAATTATATAAATATA
AAGTAGTAAAAATTGAACCATTA---GGAGTAGCACCCACCAAGGCAAAGAGAAGAGTGGTG-----------
----------------------------CAAAGAGAAAAAAGAGCA---GTGGGAACGATAGGAGCT------
---ATGTTCCTTGGGTTCTTGGGAGCA---GCAGGAAGCACTATG---GGCGCAGCGTCA---
ATAACGCTGACGGTACAG---GCCAGACAA---CTGTTG---TCTGGTATAGTGCAACAGCAGAGA---
AATTTG---CTGAGG---GCTATTGAGGCGCAACAGCATCTGTTGCAACTCACAGTCTGGGGCATC
>JF932471
TTCTATTGTAATACAGCAAAACTATTTAATAGTACTTGGCATGCTAATAATACTTGGACTGGTGATAGTACTA
TA---

GGAAATAGCAATATCACGCTCCCTTGCAAAATAAAACAATTTATAAACAAGTGGCAGGAAGTAGGGAAAGCAA
TGTATGCCCCTCCCATTAGCGGACTCATTAAATGCCCATCAAAG---
ATCACAGGGTTGCTATTAACAAGAGATGGTGGTAAGGAGACCAATACC-------------------------
----

ACCGACATTGAGATCTTTAGACCTGGAGGAGGAGATATGAGAGACAATTGGAGAAGTGAATTATATAAATATA
AAGTAGTAAAAATTGAACCATTA---GGGGTAGCACCCACCAAGGCAAAGAGAAGAGTGGTG-----------
----------------------------CAGAGAGAAAAAAGAGCA---GTGGGAACACTAGGAGCT------

---ATGTTCCTTGGGTTCTTGGGAGCA---GCAGGAAGCACTATG---GGCGCAGCGTCA---
ATGACGCTGACGGTACAG---GCCAGACAA---TTGCTG---TCTGGTATAGTGCAACAGCAGAGA---
AATCTG---CTGAGG---GCTATTGAGGCGCAACAGCGTCTGTTGCAACTCACAGTCTGGGGCATC
>JF932473
TTCTACTGTAATACATCACAACTGTTTAATAGTACTTGGAGTAATAATAGTACCAGTACTTGGAATAATACTA
CA---

GGAAATGACACTATCACGCTTCCTTGCAGAATAAAACAAATTGTAAATATGTGGCAGGAAGTAGGGAAAGCAA
TGTATGCCCCTCCCATTGCAGGACAAATTAGATGTTCATCAAAT---
ATTACAGGGTTGCTATTAACAAGAGATGGTGGTAATAATGGGAGC----------------------------
----

GACATTGAGATCTTCAGACCTGGAGGAGGAGACATGAGAGACAATTGGAGAAGTGAATTATATAAATATAAAG
TAGTAAAAATTGAACCATTA---GGAGTAGCACCCACCAAGGCAAAGAGAAGAGTGGTG--------------
-------------------------CAGAGAGAAAAAAGAGCA---GTGGGAACAATAGGAGCT---------
ATGTTCCTTGGGTTCTTGGGAGCA---GCAGGAAGCACTATG---GGCGCAGCGTCA---
ATAACGCTGACGGTACAG---GCCAGACAA---TTGTTG---TCTGGTATAGTGCAACAGCAGAGA---
AATTTG---CTGAGG---GCTATTGAGGCGCAACAGCATATGTTGCAACTCACAGTCTGGGGCATC
>KM217584
TTCTACTGCAATACAACAAAGCTGTTTAATAATACCTTGTTGGCTAATGACACTTGGAATAGAACTTGGCAAC
AG---

TCAAATGATACTATCATACTCCAATGCAGAATAAAACAAATTATAAACAGGTGGCAGGAAGTAGGAAAAGCAA
TGTATGCTCCTCCCATCAGGGGACAAATTAGCTGTTCATCAAAT---
ATTACAGGGCTGCTATTAACAAGAGATGGTGGTGACACGAATGACACG-------------------------
----

AATAGGTCTGAAATCTTCAGACCTGGAGGAGGAGATATGAGGGACAATTGGAGAAGTGAATTATATAAATATA
AAGTAGTAAAACTTGAGCCACTA---GGAATAGCACCTACCAAGGCAAAGAGAAGAGTGGTG-----------
----------------------------CAGAGAGAAAGAAGAGCA---GTGGGAACAATAGGAGCT------
---GTGTTCCTTGGGTTCTTGGGAGCA---GCAGGAAGCACTATG---GGCGCAGCGTCA---
GTGACGCTGACGGTACAG---GCCAGACTA---TTACTG---TCTGGTATAGTGCAACAGCAGAAC---
AATCTG---CTGAGA---GCTATTGAGGCGCAACAGAACATGTTGCAACTCACAGTCTGGGGCATC
>KM217662
TTCTACTGTAATACATCAAAGCTGTTTAATAATACTTGGACTAATAATACTTGGAATGGTACTTGGCCA----
----

AATAGTACCATCATAATCATCCCATGCAGAATAAAACAAATTATAAACAAGTGGCAGGAAGTAGGAAGGGCAA
TGTATGCCCCTCCCATCAGTGGACAAATTAGATGTTCATCAAAT---
ATTACAGGGTTGCTATTAACAAGGGATGGTGGTGGTGGTAATAACTCAACTAAC------TCAACTGAG---
CCA--

ACTGAGGAAATCTTCAGACCTGCAGGAGGAGATATGAGGGACAATTGGAGAAGTGAATTATATAAATATAAAG
TGGTAAAAATTGAGCCAATA---GGACTAGCACCCACCAAGGCAAAGAGAAGAGTGGTG--------------
-------------------------CAGAGAGAAAAAAGAGCA---GTGGGAACTTTAGGAGCT---------
GTGTTCCTTGGGTTCTTGGGAGCA---GCAGGAAGCACTATG---GGCGCAGCGTCA---
TTGACGCTGACGGTACAG---GCCAGACTA---TTACTG---TCTGGTATAGTGCAACAGCAGAAC---
AATCTG---CTGAGA---GCTATTGAGGCGCAACAACACATGTTGCAACTCACAGTCTGGGGCATC
>KM217802
TTTTACTGTAATACAACACAGCTGTTTAATAGTACTTGGAATGGTACTGAAGGGGTAAATACCACTGAAGGAG
AG---

ATCATACTCCCATGCAGAATAAAACAAATTATAAACAGGTGGCAAGAAGTAGGAAAAGCAATATATGCCCCTC
CCATCAGCGGACAGATCAATTGTTCATCAAAT---
ATTACAGGGCTATTATTAACAAGAGATGGTGGTATTAATAACGAGACC-------------------------
----

CAGACTACTGAGACCTTCAGACCTGGAGGAGGAGATATGAGGGACAATTGGAGAAGTGAATTATATAAATATA
AAGTAGTAAAAATTGAACCATTA---GGAGTAGCACCCACCAAGGCAAAGAGAAGAGTGGTG-----------
----------------------------CAAAGAGAAAAAAGAGCA---GTGGGA---ATAGGAGCT------

---GTGTTCCTTGGGTTCTTGGGAGCA---GCAGGAAGCACTATG---GGCGCAGCGTCG---
ATAACGCTGACGGTACAG---GCCAGACAA---TTATTG---TCTGGTATAGTGCAACAGCAGGGT---
AACTTG---CTGAGA---GCTATTGAGGCGCAACAACATCTGTTGCAACTCACAGTCTGGGGCATC
>KM217995
TTCTACTGTAATACAACAAAGTTGTTTAATAATACTTTGCTGACTAATGATACTTGGCGAGATACTTGGCAAG
AGTCA--
-------------AATAAT------
ACTGAAGGAAATGTCATTACACTCCAATGCAGAATAAAACAAATTATAAACAGATGGCAGGAAGTAGGAAAAG
CAATGTATGCCCCTCCCATCAGGGGACAAATTAGTTGTTCATCAAAT---
ATTACAGGGCTTCTATTAACAAGAGATGGTGGCAGCATGAATGAGTCC-------------------------
----

ATAAATGAAACCTTCAGACCTGGAGGAGGAAATATGAGGGACAATTGGAGGAGTGAATTATATAAATATAAAG
TAGTAAAAATTGAGCCACTA---GGAATAGCACCCACCAAGGCAAAGAGAAGAGTGGTG--------------
-------------------------CAGAGGGAAAAAAGAGCA---GTGGGAACAATAGGAGCT---------
ATGTTCCTTGGTTTCTTGGGAGCA---GCAGGAAGCACTATG---GGCGCAGCGTCA---
ATGACGCTGACGGTACAG---GCCAGACTA---TTACTG---TCTGGTATAGTGCAACAGCAGAAC---
AATCTG---CTGAAA---GCTATTGAGGCGCAACAGCACCTGTTGCAACTCACAGTCTGGGGCATC
>JF932475
TTCTACTGTAATACATCACAACTGTTTAATAGTACTTGGTATGGTAATGATACTTGGAATAATACTACA----
----

AAAGATAGCAATATCACGCTCCATTGCAGAATAAAACAAGTTGTAAACATGTGGCAGGAAGTAGGGAAAGCAA
TGTATGCCCCTCCCATTGCAGGACTAATTAGATGTACATCAAAC---
ATTACCGGGTTGCTACTAACAAGAGATGGGGGTAATAATAGGAGCGGG-------------------------
----

GACACCGAGATCTTCAGACCTGGAGGAGGAGATATGAGAGACAATTGGAGAAGTGAATTATATAAATATAAAG
TAGTGAAAATTGAACCATTA---GGAGTAGCACCCACCAGGGCACAGAGAAGAGTGGTG--------------
-------------------------CAGAGAGAAAAAAGGGCA---GTGGGAACGATAGGAGCT---------
ATGTTCCTTGGGTTCTTGGGAGCA---GCAGGAAGCACTATG---GGCGCAGCGTCA---
ATGACGCTGACGGTACAG---GCCAGACAA---CTGTTG---TCTGGTATAGTGCAACAGCAGAGA---
AATTTG---CTGATG---GCTATTGAGGCGCAACAGCATCTGTTGCAACTCACAGTCTGGGGCATC
>JF932476
TTTTACTGTAATACATCAAAACTGTTTAATAGTACTTGGGATAGGACTGATAATAATACTCGAAATGAT----
----

AATAATAATACAATCACGCTCCCTTGCAGAATAAAACAACTTATAAACATGTGGCAGGAAGTAGGGAAAGCAA
TGTATGCCCCTCCCATTAAAGGACAAATTAACTGTTCATCACAT---
ATTACGGGATTGCTATTAACAAGAGATGGTGGTACTAATGAGAGC----------------------------
----

AACACCACCGAGACCTTCAGACCTGGAGGAGGAGATATGAGAGACAATTGGAGAAGTGAATTATATAAATATA
AAGTAGTAAAAATTGAACCATTA---GGAGTAGCACCCACCAAGGCAAAGAGAAGAGTGGTG-----------
----------------------------CAGAGAGAAAAAAGAGCA---GTGGGAGCGTTAGGAGCT------
---ATGTTCCTTGGGTTCTTGGGAGCA---GCAGGAAGCACGATG---GGCGCAGCGTCA---
ATAGCGCTGACGGTACAG---GCCAGACAA---TTATTG---TCTGGTATAGTGCAGCAGCAGAGA---
AATTTG---CTGAGG---GCTATTGAGGCGCAACAGCATATGTTGCAACTCACAGTCTGGGGCATC
>JF932477
TTCTACTGTAATACATCACAACTGTTTAATAGTACTTGGGATGGTAATGATACTAGTACTTGGACTAACGCTA
CA---

GAAAATCGCACTATCACACTCCCTTGCAGAATAAAACAAATTGTAAACATGTGGCAGGAAGTAGGGAAAGCAA
TGTATGCCCCTCCCATTAGAGGACAAATTAGATGTTCATCAAAT---
ATTACAGGGCTGATATTAATAAGAGATGGTGGTAATAATGAGAGCACG-------------------------
----

ACCAACAACGAGACCTTTAGACCTGCAGGAGGAAATATGAAAGATAATTGGAGAAGTGAATTATATAAATATA
AAGTAGTAAAAATTGAACCATTA---GGAGTAGCACCCACCAAGGCAAAGAGAAGAGTGGTG-----------
----------------------------CAGAGAGAAAAAAGAGCA---GTGGGAACGATAGGAGCT------

---ATGTTCCTTGGGTTCTTGGGAGCA---GCAGGAAGCACTATG---GGCGCAGCGTCA---
ATAACGCTGACGGTACAG---GCCAGACAA---TTGATG---TCTGGTATAGTGCAACAGCAGAGA---
AATTTG---CTGAGG---GCTATTGAGGCGCAACAGCATATGTTGCAACTCACAGTCTGGGGCATC
>JF932483
TTCTACTGTAATACATCAAAACTGTTTAATGATACTATTGCTTGGAATGATACAGAACTAGAACTGTTTAACG
ATACTAGTACT--

GAGAATGATACTATCACACTCCCTTGCAGAATAAAACAAGTTGTAAATATGTGGCAGGAAGTAGGGAAAGCAA
TGTATGCCCCTCCCATTAGTGGACAAATTAGATGTTCATCAAAT---
ATTACAGGGATGATATTAACAAGAGATGGTGGTAATGTGACCAAC----------------------------
----

AACAAGACTGAGGTCTTCAGACCTGGAGGAGGAGATATGAGAGACAATTGGAGAAGTGAATTATATAAATATA
AAGTAGTAAAAATTGAACCATTA---GGAGTAGCACCCACCAAGGCAAAG-----------------------
-------------------------------AGAAAAAAAAGAGCA---GTGGGAACAAAAGGAGCT------
---ATGTTCCTTGGGTTCTTGGGAGCA---GCAGGAAGCACTATG---GGCGCAGCATCA---
ATAACGCTGACGGTACAG---GCCAGACAA---TTATTG---TCTGGTATAGTGCAACAGCAGAGA---
AATTTG---CTGAGG---GCTATTGAGGCGCAACAGCATCTGTTGCAACTCACAGTCTGGGGCATC
>JF932484
TTCTACTGTAATACCTCACAACTGTTTAATAGTACTTGGTATGCTAATAGGACTTGGAAGGGTGATACA----
----

GGAAATGACACTATCACACTCCCTTGCAGAATAAAACAAATTGTAAACATGTGGCAGGAAGTAGGAAAAGCAA
TGTATGCCCCTCCCATTAAAGGACAAATTAGATGTTCATCAAAT---
ATTACAGGGTTGTTATTAACAAGAGATGGTGGTAATGGGAACAAGACC-------------------------
----

AACAACACTGAGACCTTCAGACCGGGAGGAGGAGACATGAGAGACAATTGGAGAAGTGAATTATATAAATATA
AAGTAGTACAAATTGAACCATTA---GGAGTAGCACCCACCAAGGCAAAGAGAAGAGTGGTG-----------
----------------------------CAAAGAGAAAAAAGAGCA---GTGGGAACGATAGGAGCT------
---ATGTTCCTTGGGTTCTTGGGAGCA---GCAGGAAGCACTATG---GGCGCAGCGTCA---
ATACCGCTGACGGTACAG---GCCAGACAA---TTGTTG---TCTGGTATAGTGCAACAGCAGAGA---
AATTTG---CTGAGG---GCTATTGAGGCGCAACAGCATATGTTGCAACTCACAGTCTGGGGCATT
>JF932485
TTCTACTGTAATACATCACAACTGTTTAATAGTACTTGGCATAATAAGGGTACTTGGAATAGTACTCGGAATG
ATACTGCA---

GAAAATGACACTATCACGCTCCCTTGCAGAATAAAACAAATTGTAAACATGTGGCAGGAAGTAGGAAAAGCAA
TGTATGCCCCTCCCATTAAAGGACAAATTAGATGTTCATCAAAT---
ATTACAGGGTTGCTATTAACAAGAGATGGTGGTAATAATGAGAACAAGACCACC-------------------
----

AACGAGACCGAGACCTTCAGACCTGGAGGAGGAGATATGAGAGACAATTGGAGAAGTGAATTATATAAATATA
AAGTAGTAAAAATTGAACCATTA---GGAGTAGCACCCACCAAGGCAAAAAGAAGAGTGGTG-----------
----------------------------CAGAGAGAAAAAAGAGCA---GTGGGAACGATAGGAGCT------
---ATGTTCCTTGGGTTCTTGGGAGCA---GCAGGAAGCACTATG---GGCGCAGCGTCA---
ATAACGCTGACGGTACAG---GCCAGACAA---TTGTTG---TCTGGTATAGTGCAACAGCAGAGA---
AATTTG---CTGAGG---GCTATTGAGGCGCAACAACATCTGTTGCAACTCACAGTCTGGGGCATC
>JF932486
TTCTACTGTGACACATCACAACTGTTTAATAGTTCTTGGAATAGTAATACTAGTACTTGGAATAAGACA----
----

GGAGATAGTAATATCACGCTCCCTTGCAGAATAAAACAAATTATAAACATGTGGCAGAAAGTAGGGAAAGCAA
TGTATGCCCCTCCTATTAGAGGACAAATTAGATGTTCATCAAAT---
ATTACAGGGTTGCTATTAACAAGAGATGGTGGTGAGGGGAACAGG----------------------------
----

ACCAACGAGACCTTCAGACCTGGAGGAGGAGATATGAGAGACAATTGGAGAAGTGAATTATATAAATATAAAG
TAGTAAGAATTGAACCATTA---GGAGTAGCACCCACCAAGGCAAAGAGAAGAGTGGTG--------------
-------------------------CAGAGAGAAAAAAGAGCA---GTGGGAACGCTAGGAGCT---------

ATGTTCCTTGGGTTCTTGGGAGCA---GCAGGAAGCACTATG---GGCGCAGCGTCA---
ATAACGCTGACGGTACAG---GCCAGACAA---TTGTTG---TCTGGTATAGTGCAACAGCAGAGA---
AATTTG---CTGAGG---GCTATTGAGGCGCAACAGCATCTGTTGCAACTCACAGTCTGGGGCATC
>MH479957
TTCTACTGTAATACATCACAACTGTTTAACAGTACTTGGAATAATACCAGTACTGGGAATGATACTAATACT-
----

ACAGATAGAATTATCACGCTCCCTTGCAGGATAAAACAAATTGTAAACATGTGGCAGGAAGTAGGAAAAGCAA
TGTATGCCCCTCCCATTGGAGGACAGATTAGGTGCTCATCAAAT---
ATTACAGGGTTGCTATTAACAAGAGATGGTGGTAATACCACCGAGTCTACCGAG-------------------
----

TCTACCGAGACCTTCAGACCTGCAGGAGGAAATATGAAGGACAATTGGAGAAGTGAATTATATAAATATAAAG
TAGTAAAAATTGAACCATTA---GGAGTAGCACCCACCAGGGCAAAGAGAAGAGTGGTG--------------
-------------------------CAAAGAGAAAAAAGAGCA---GTGGGAACGATAGGAGCT---------
ATGTTCCTTGGGTTCTTGGGAGCA---GCAGGAAGCACTATG---GGCGCAGCGTCA---
ATAACGCTGACGGTACAG---GCCAGACAA---TTGCTG---TCTGGTATAGTGCAACAGCAGAGA---
AATTTG---CTGAGG---GCTATTGAGGCGCAACAACACCTGTTGCAACTCACAGTCTGGGGCATC
>JF932487
TTCTACTGTAATACATCAAAACTGTTTAACAGTACTTGGCATGCTAATGGTACTTGGACTAATAGTACA----
----

GTAGATAGCAATATCACGCTCCATTGTAGAATAAAACAAATTGTAAACATGTGGCAAGAAGTAGGGAAAGCAA
TGTATGCCCCTCCCATTGCAGGAAAAATTAGATGTTCATCAAAT---
ATTACAGGGTTGCTATTAACAAGAGATGGGGGTAATACTGGGAAC----------------------------
----

ACTGACAACGAGACCTTCAGACCTGGAGGAGGAGATATGAGAGACAATTGGAGAAGTGAATTATATAAATATA
AAGTAGTTAAAATTGAGCCAATA---GGAGTAGCACCCACCAAGGCAAAGAGAAGAGTGGTG-----------
----------------------------CAGAGAGAAAAAAGAGCA---GTGGGAACAATAGGAGCT------
---ATGTTCCTTGGGTTCTTGGGAGCA---GCAGGAAGCTCTATG---GGCGCAGCGTCA---
ATAACGCTGACGGTACAG---GCCAGACAA---TTGTTG---TCTGGTATAGTGCAACAGCAGAGA---
AATTTG---CTGAGG---GCTATTGAGGCGCAACAGCATATGTTGCAACTCACAGTCTGGGGCATC
>JF932489
TTCTACTGTAATACATCACAACTGTTTAATGGGAATGGTACTAGTACTGGGAATAAGACTACA----------
----

GGAAATGACACTATCACACTCCCTTGCAGAATAAAACAAATTGTAAACATGTGGCAGGAGGTAGGGAAAGCAA
TGTATGCCCCTCCCATTTCAGGACGAATTAGATGTGTATCAAAC---
ATTACAGGGTTGCTATTAACAAGAGATGGTGGTAATGAGACCGAT----------------------------
----

ACCACCGAGACCTTCCGACCTTGGGGAGGAGATATGAGAGACAATTGGAGAAGTGAATTATATAAATATAAAG
TAGTAAAAATTGAACCATTA---GGAGTAGCACCCACCAAGGCAAAGAGAAGAGTGGTG--------------
-------------------------CAGAGAGAAAAAAGAGCA---GTGGGA---CTAGGAGCT---------
ATGTTCCTTGGGTTCTTGGGAGCA---GCAGGAAGCACGATG---GGCGCAGCGTCA---
ATAACGCTGACGGTACAG---GCCAGACAA---TTGATG---TCTGGTATAGTGCAACAGCAGAGA---
AATTTG---CTGAGG---GCTATTGAGGCGCAACAGCATATGTTGCAACTCACAGTCTGGGGCATC
>JF932490
TTCTACTGTAATACATCACAACTGTTTAATAGTACTTGGTATGAGAATGGTACTATTACTACTAAGAATTTT-
----

ACTACAGGAACTATCACACTCCCTTGCAGAATAAAACAAATTGTAAACATGTGGCAGCAAGTAGGGAAAGCAA
TGTATGCCCCTCCCATTGCAGGAAAAATTAGATGTTCATCAAAT---
ATTACAGGATTGCTATTAACAAGAGATGGGGGTACGGGTAATGAGACCAACAAC-------------------
----

AATGACACTGAGACTTTCAGACCTGAAGGAGGAAATATGAAAGACAATTGGAGAAGTGAATTATATAAATATA
AAGTAGTAAGAATTGAACCATTA---GGACTAGCACCCACCAAGGCAAAGAGAAGAGTGGTG-----------
----------------------------CAGAGAGAAAAAAGAGCA---GTGGGAACAATAGGAGCT------

---ATGTTCCTTGGGTTCTTGGGAGCA---GCAGGAAGCACTATG---GGCGCAGCGTCA---
ATAACGCTGACGGTACAG---GCCAGACAA---TTGTTG---TCTGGTATAGTGCAACAGCAAAGA---
AATTTG---CTGAGG---GCTATTGAGGCGCAACAGCATCTGTTGCAACTCACAGTCTGGGGCATC
>JF932491
TTCTACTGTAATACAACACAACTGTTTAATAGTACTTGGCCTGGGAATAATACTAGTACTAAGACA-------
----

GAAAATGATAATATCACGCTCCCTTGCAAAATAAGACAAATTGTAAACATGTGGCAGAAAGTAGGGAAAGCAA
TGTATGCCCCTCCCATTAAAGGACAAATTAGATGTTCATCAAAT---
ATTACAGGGTTGCTGTTAACAAGAGATGGTGGTAGTAATGAGAACACG-------------------------
----

ATCAACGAGACCTTCAGACCTGGAGGAGGAGATATGAGAGACAATTGGAGAAGTGAATTATATAGATATAAAG
TAGTAAAAATTGAACCTTTA---GGAGTAGCACCCACCAAGGCAAAGAGAAGAGTGGTG--------------
-------------------------CAGAGAGAAAAAAGAGCA---GTGGGAACGCTAGGAGCT---------
ATGTTCCTTGGGTTCTTGGGAGCA---GCAGGAAGCACTATG---GGCGCAGCGTCA---
ATAACGCTGACGGTACAG---GCCAGACAA---TTGCTG---TCTGGTATAGTGCAACAGCAGAGA---
AATTTG---CTGATG---GCTATTGAGGCGCAACAACATCTGTTGCAACTCACAGTCTGGGGCATC
>JF932493
TTCTACTGTAATACAACACAACTGTTTAATAGTACTTGGAATAATACTAGTATTTGGAATAATGTTACA----
----

GAAAATGGCACTATCACGCTTCCTTGCAGAATAAAACAAATTGTAAACATGTGGCAGGGAGTAGGGAAAGCAA
TGTATGCCCCTCCCATTGCAGGACAAATTAACTGTTCATCAAAT---
ATTACAGGGTTGCTATTAACAAGAGATGGTGGTGTTAATAAGAGC----------------------------
----

GAGGGCAACACCACCGAGACCTTCAGACCTGCAGGAGGAAATATGAGAGACAATTGGAGAAGTGAATTATATA
AATATAAAGTAGTAAAAATTGAACCATTA---GGATTAGCACCCACCAAGGCAAAGAGAAGAGTGGTG-----
-----------------------------------CAAAGAGAAAAAAGAGCA---
GTGGGAACGATAGGAGCT---------ATGTTCCTTGGGTTCTTGGGAGCA---GCAGGAAGCACGATG---
GGCGCAGCGTCG---ATGACGCTGACGGTACAG---GCCAGGCAA---TTGTTG---
TCTGGTATAGTGCAACAGCAAAGA---AATTTG---CTGAGG---
GCTATTGAGGTGCAACAGCATCTGTTGCAACTCACAGTCTGGGGCATC
>JF932499
TTCTACTGTAATACATCACAACTGTTTAATAGTTCTTGGAATGATACTAGTACTTGGAATAATACTACA----
----

GAAAATAGCACTATCATGCTCCCTTGCAGAATAAAACAAATTGTAAACATGTGGCAGGAAGTAGGGAAAGCAA
TGTATGCCCCTCCCATTAGTGGAATAATTAACTGCAAATCAAAT---
ATTACAGGGTTGCTATTAACGAGAGATGGTGGTGGTAATGAGACT----------------------------
----

AACACTGAGATCTTTAGACCAGGAGGAGGAAATATGAAAGACAATTGGAGAAGTAAATTATATAAATATAAAG
TAGTAAAAATTGAACCATTA---GGAGTAGCACCCACCAAGGCAAAGAGAAGAGTGGTG--------------
-------------------------CAGAGAGAAAAAAGAGCA---GTGGGAACGATAGGAGCT---------
ATGTTCCTTGGGTTCTTGGGAACA---GCAGGAAGCACTATG---GGCGCAGCGTCA---
ATAACGCTGACGGTACAG---GCCAGACAA---TTGCTG---TCTGGTATAGTGCAACAGCAGAGA---
AATTTG---CTGAGG---GCTATTGAGGCGCAACAGCATCTGTTGCAACTCACAGTCTGGGGCATC
>JF932500
TTCTACTGTAATACATCACAACTGTTTAATAGTACTTGGCCTAATAATGGTACTTGGAATGATACTGCA----
----

AAAAATGACAATATCACTCTCCCTTGCAGAATAAAACAAATTATAAACATGTGGCAGGAAGTAGGGAAAGCAA
TGTATGCCCCTCCCATTGGAGGACAAATTAGATGTTCATCAAAT---
ATTACAGGGTTGCTATTAACAAGAGATGGTGGTAGGAATGAGAGT----------------------------
----

AACACCACCGAGACCTTCAGACCTGGAGGAGGAGACATGAGAGACAATTGGAGAAGTGAATTATATAAATATA
AAGTAGTAAAAATTGAACCATTA---GGAGTAGCACCCACCAAGGCAAAGAGAAGAGTGGTG-----------

----------------------------CAGAGAGAAAAAAGAGCA---GTGGGAACGATAGGAGCT------
---ATGTTCCTTGGGTTCTTGGGAGCA---GCAGGAAGCACTATG---GGCGCAGCGTCA---
ATAACGCTGACGGTACAG---GCCAGACAA---TTGTTG---TCTGGTATAGTGCAACAGCAGAGA---
AATTTG---CTGATG---GCTATTGAGGCGCAACAGCATCTGTTGCAACTCACAGTCTGGGGCATC
>KM218168
TTCTACTGTAATACAACAAAGCTATTTAGTAATACTTGGCAGACTAATAACTCTTGGAATGGTACTTGGCAAG
GGTCAAATAAT--

AGTGATAGAAATGTCCTTATACTCCAATGCAGAATAAAACAAATTATAAACAGATGGCAGGAAGTAGGAAAAG
CAATGTATGCCCCTCCCATCAGGGGAGAAATTAAATGTTCATCAAAT---
ATTACAGGGCTTCTATTAACAAGAGATGGTGGTAACACAACTGAGTCCAGA----------------------
----

AATGGGACTGAAATCTTCAGACCTGGAGGAGGAGATATGAGGGACAATTGGAAAAGTGAATTATATAAATATA
AAGTAGTAAAAATTGAGCCACTA---GGAATAGCACCCACCAAGGCAAAGAGAAGAGTGGTG-----------
----------------------------CAGAGAGACAAAAGAGCG---GTAGGAACACTAGGAGCT------
---ATGTTCCTTGGGTTCTTGGGAGCA---GCAGGAAGCACTATG---GGCGCAGCGTCG---
ATGACGCTGACGGTACAG---GCCAGACTA---TTACTG---TCTGGTATAGTGCAACAGCAGAAC---
AATATG---CTGAAA---GCTATTGAGGCGCAACAGCACCTGTTGCAACTCACAGTCTGGGGCATC
>KM218228
TTCTACTGTAATACATCAAAATTGTTTAATAATACTTTACAGCCTAATAATACTTGGAATAGTACTTGGCAAA
CAGAT--

ATTAACGGAACCATCACACTCCCATGCAGAATAAAACAAATTATAAACAGGTGGCAGGAAGTAGGAAGAGCAA
TGTATGCCCCTCCCATCAGTGGACAAATTAAATGTTTATCAAAT---
ATTACAGGGCTGCTATTAACAAGGGATGGTGGTAAAACGAATGGGTCCGAA----------------------
----

AATGAGACCGAAATCTTCAGGCCTGGAGGAGGAGATATGAGAGACAATTGGAGAAGTGAATTATATAAATATA
AAGTAGTAAAAATTGAGCCACTA---GGAATAGCACCCACCAAGGCAAAGAGAAGAGTGGTG-----------
----------------------------CAGAGAGAAAAGAGAGCG---GTGGGAACACTGGGAGCT------
---GTGTTCCTCGGGTTCTTGGGAGCA---GCAGGAAGCACTATG---GGCGCAGCGTCA---
ATGACGCTGACGGTACAG---GCCAGACTA---TTGCTG---TCTGGTATAGTGCAACAGCAGAAC---
AATATG---CTGAGA---GCTATTGAGGCGCAACAGCACATGTTGCAACTCACAGTCTGGGGCATC
>JF932479
TTCTACTGTAATACATCACAACTGTTTAATAGTGTTAATGGTACTTGGAATGGTACTGGTACA----------
----

GGAAATAACACTATCACGCTCCCTTGCAGAATAAAACAAATTGTAAACATGTGGCAGGAAGTAGGGAAAGCAA
TGTATGCCCCTCCCATTAGTGGACAAATTAGATGTTTATCAAAT---
ATTACAGGGTTGCTATTAACAAGAGATGGTGGTAATAATGGGAGC----------------------------
----

AACACCACCGAGACCTTCAGACCAGGAGGAGGAGATATGAGAGACAATTGGAGAAGTGAATTATATAAATATA
AAGTAGTACAAATTGAACCATTA---GGAGTAGCACCCACCAGGGCAAAGAGAAGAGTGGTG-----------
----------------------------CAGAGAGAAAAGAGAGCA---GTGGGAACAATAGGAGCT------
---ATGTTCCTTGGGTTCTTGGGAGCA---GCAGGAAGCACTATG---GGCGCAGCATCG---
ATAACGCTGACGGTACAG---GCCAGACAA---TTGTTG---TCTGGTATAGTGCAACAGCAAAGA---
AATTTG---CTGAGG---GCTATTGAGGCGCAACAGCACATGTTGCAACTCACAGTCTGGGGCATC
>JF932480
TTTTACTGTAATACATCACAACTGTTTAATAGTTCTTGGCATGCTAATAGTACTAGGAATAACACAGATAAG-
----

ATCATACTTCCTTGTAGAATAAAACAAATTGTAAACATATGGCAGGGAGTAGGGAAAGCAATATATGCCCCTC
CCATTGAAGGACAAATTAGTTGTTTATCAAAG---
ATTACAGGGTTACTATTAACAAGAGATGGTGGTGGTGGTAGAAATGCGAGT----------------------
----

GAGAACAACACCGAGACCTTCAGACCTGGAGGGGGAGATATGAGAGACAATTGGAGAAGTGAATTATATAAAT
ATAAAGTAGTAAAAATTAAACCATTA---GGAATAGCACCCACCAAGGCAAAAAGAAGAGTGGTG--------

-------------------------------CAAAGAGAAAAAAGAGCA---GTGGGAACGATAGGAGCT---
------ATGTTCCTTGGGTTCTTGGGAACA---GCAGGAAGCACTATG---GGCGCAGCGTCA---
ATGACGCTGACGGTACAG---GCCAGACAA---TTGTTG---TCTGGTATGGTGCAACAGCAGAGA---
AATTTG---CTGAGG---GCTATTGAGGCGCAACAGCATCTGTTGCAACTCACAGTCTGGGGCATC
>JF932481
TTCTACTGTGATACATCACAACTGTTTAATAGTACTTGGATTTGGAATGATACTAGTACTTGGAATGGTACTG
CA---

GAAGATAGCACTATCACGCTCCCTTGCAGAATAAGACAAATTGTAAACATGTGGCAGGAAGTAGGGAAAGCAA
TGTATGCCCCTCCCATAGCAGGACAAATTAGATGTTCATCAAAT---
ATTATAGGGTTGCTATTAACAAGAGATGGTGGTAATGAGAACAAC----------------------------
----

AAGACCACCGAGACCTTCAGACCTGGAGGAGGAGATATGAGAGACAATTGGAGGAGTGAATTATATAAATATA
AAGTAGTAAAAATTGAACCATTA---GGAGTAGCACCCACCAGGGCAAAGAGAAGAGTGGTG-----------
----------------------------CAGAGAGAAAAAAGAGCA---GTGGGA---ATAGGAGCT------
---ATGTTCCTTGGGTTCTTGGGAGCA---GCAGGAAGCACTATG---GGCGCAGCGTCA---
ATAACGCTGACGGTACAG---GCCAGACAA---TTGTTG---TCTGGTATAGTGCAGCAGCAGAGA---
AATCTG---CTGAGG---GCTATTGAGGCGCAACAGCATCTGTTGCAACTCACAGTCTGGGGCATC
>JF346919
TTCTACTGTAATACATCACTACTGTTTAATAGTACTTGGTATGAGAATGATACTAGTAATTGGAATGCT----
----

ACTAAAGAAAAAATCACGCTCCCTTGCAGAATAAGACAAATTGTAAACATGTGGCAGGAAGTAGGGAAAGCAA
TGTATGCCCCTCCCATTGCAGGACAACTTAACTGCTCATCAAAC---
ATTACAGGGTTGCTATTAACAAGAGATGGTGGTGGTGATGGTAAT----------------------------
----

AGTACGGACGAGATCTTCAGACCTGGAGGAGGAGATATGAGGGACAATTGGAGAAGTGAATTATATAAATATA
AAGTAGTAAAAATTGAACCATTA---GGAGTAGCACCCACCAAGGCAAAGAGAAGAGTGGTG-----------
----------------------------CAGAGAGAAAAAAGAGCA---GTGGGAACGATAGGAGCT------
---ATGTTCCTTGGGTTCTTGGGAGCA---GCAGGAAGCACTATG---GGCGCAGCGTCA---
ATAACGCTGACGGTACAG---GCCAGACAA---TTATTG---TCTGGTATAGTGCAACAGCAGAGA---
AATTTG---CTGAGG---GCTATTGAGGCGCAACAGCATTTGTTGCAACTCACAGTCTGGGGCATC
>JF932498
TTCTACTGTAATACATCACGCTTGTTTAACAGTATTTGGAATGGTACTAGTACTTGGAATGATACTACA----
----

GAAAATGACACTATCATACTCCCTTGCAGAATAAAACAATTTGTAAACATGTGGCAGAAAGTAGGGAAGGCAA
TGTATGCCCCTCCCATTAGCGGACCGATTAGCTGTTCATCAAAA---
ATTACAGGGTTGCTATTAACAAGAGATGGTGGTAATGAGAGCCAG----------------------------
----

ATTTCCGAGACCTTCAGACCTGAAGGAGGGGATATGAGAGATAATTGGAGAAGTGAATTATATAAATATAAAG
TAGTAAAAATTGAACCATTA---GGAATAGCACCCACCAGGGCAAAAAGAAGAGTGGTG--------------
-------------------------CAGAGAGAAAAAAGAGCA---GTGGGAACGATAGGAGCT---------
ATGTTCCTTGGGTTCTTAGGAGCA---GCGGGAAGCACTATG---GGCGCAGCGTCA---
ATAACGCTGACGGTACAG---GCCAGACAA---TTGCTG---TCTGGTATAGTGCAACAGCAGAGA---
AATTTG---CTGAGG---GCTATTGAGGCGCAACAGCATCTGTTGCAACTCACAGTCTGGGGCATT
>JX960597
TTCTACTGTAACTCAACACCACTGTTTAACAGTACTTGGGAGAATGGGACACAGGAAGAATGGACACAGCTTA
ATGAG--
-------------AATAAT------
ACTGAAGAGTCAAATATCACCCTCCAATGCAGAATAAAACAATTTATAAACATGTGGCAGGAAGTAGGAAAAG
CAATGTATGCCCCTCCCATCAGTGGACAAATCAGGTGTTCATCAAAT---
ATTACAGGACTGCTATTAACAAGAGATGGGGGAGGG-------------------------------------
----

GAGAACGAGACCTTCAGGCCTGAAGGAGGAGATATGAGGGACAATTGGAGAAGTGAATTATATAAATATAAAG
TAGTGAAAATTGAACCATTA---GGAGTAGCACCCACTAAGGCAAAGAGAAGAGTGGTG--------------

-------------------------CAGAGAGAAAAAAGAGCA---GCG------ATAGGAGCT---------
ATGTTCCTTGGGTTCTTGGGAGCA---GCAGGAAGCACTATG---GGCGCAGCGTCA---
GTAACGCTGACGGTACAG---GCCAGACAA---TTATTG---TCTGGTATAGTGCAACAGCAGAAT---
AATTTG---CTGAGA---GCTATTGAGGCGCAACAGCATCTGTTGCAACTCACAGTCTGGGGCATC
>JX960598
TTCTACTGTAATACAACACAACTGTTTAATAGTACTTGGAATGACACTCGTATTGAGAATAGTACTGGGAACG
CA---

AATGGAAGCCATATCACACTCCCATGCAGAATAAAACAAATTATAAACATGTGGCAGGAAGTAGGAAAAGCAA
TGTATGCCCCTCCCATCAAAGGACTACTTAGATGTTCATCAAAT---
ATTACAGGGCTGATATTAGTAAGAGATGGTGGTGCTGTCAATACT----------------------------
----

ACCACCGAGACCTTCAGACCTGGAGGAGGAGATATGAGGGACAATTGGAGAAGTGAATTATATAAATATAAAG
TAGTAAAAATTGAACCATTA---GGACTAGCACCCACCAAGGCAAAAAGAAGAGTGGTG--------------
-------------------------CAAAGAGAAAAAAGAGCA---GTGGGA---ATAGGAGCT---------
CTGTTCCTTGGGTTCTTGGGAGCA---GCAGGAAGCACTATG---GGCGCAGCGTCG---
ATGACGTTGACGGTACAG---GCCAGACAA---TTATTG---TCTGGTATAGTGCAACAGCAAAAC---
AACTTG---CTGAAG---GCTATTGAGGCGCAACAGCATATGTTGCAACTCACAGTATGGGGCATC
>KC899011
TTTTACTGTAATACATCAAAACTGTTTAATAGTACTTGGAATGATACTAAA----------------------
----

GGAAATGGCACTATCACGCTCGATTGCAGAATAAAACAAATTGTAAATATGTGGCAGGGAGTAGGAAAAGCAA
TATATGCCCCTCCCATTGAAGGACAAATTAGATGTTCATCAAAG---
ATTACAGGGTTACTATTAACAAGAGATGGTGGCAATAATGGGAACAACGAG----------------------
----

ACCACCGAGACCTTCAGACCTGGAGGAGGAAATATGAAAGACAATTGGAGAAGTGAATTATATAAATATAAAG
TAGTAAAACTTGAACCATTA---GGAGTGGCACCCACCAAGGCAAAGAGAAGGGTGGTG--------------
-------------------------CAGAGAGAAAAAAGAGCA---GTGGGAACGATAGGAGCT---------
ATGTTCCTTGGGTTCTTGGGAGCA---GCAGGAAGCACTATG---GGCGCAGCGTCA---
ATAACGCTGACGGTACAG---GCCAGACAA---TTGTTG---TCTGGTATAGTGCAACAGCAGAGA---
AATTTG---CTGATG---GCAATTGAGGCGCAACAGCATCTGTTGCAACTCACCGTCTGGGGCATC
>HQ215556
TTCTACTGTAATACAACAAAGCTGTTTAATAATACTTTTAATAGTGCCTGGCAAGAGTCAAAAGATAATGGC-
----

ACCGAAGGAGTCATCAAACTTCCATGCAAAATAAAACAAATAATAAATAGATGGCAGGAAGTAGGAAAAGCAA
TGTATGCTCCTCCCATCGAGGGACAAATTAGATGTTCATCAAAT---
ATTACAGGATTACTATTAACACGAGATGGTGGTAACAAGAGTGAG----------------------------
----

ACTGAGCCTGAAACCTTCAGACCTGGGGGAGGAGATATGAGGGACAATTGGAGAAGTGAATTATATAAATATA
AAGTAATAAAAATTGAGCCATTA---GGACTGGCACCCACCAGGGCAAAGAGAAGAGTGGTG-----------
----------------------------CAGAGACAAAAAAGAGCA---GTGGGAGTACTAGGAGCT------
---ATGTTCCTTGGGTTCTTGGGAGCA---GCAGGAAGCACTATG---GGCGCAGCGTCA---
GTCACGCTGACGGTACAG---GCCAGACTA---TTGCTG---TCTGGTATAGTGCAACAGCAGAAC---
AATTTG---CTGAGA---GCTGTTGAGGCGCAACAACACCTGTTGCAACTCACAGTCTGGGGCATC
>JF346916
TTCTACTGTAATACATCACAACTGTTTAATAGTACTTGGTATGCTAATGGTACTTGGGAGGGTAATTCA----
----

AGAAATGACAATATCACGCTCCCTTGCAGAATAAGACAAATTGTAAACATGTGGCAGGAAGTAGGGAAAGCAA
TGTATGCCCCTCCCATTGCAGGACAAATTAGTTGTTTATCAAAT---
ATTACAGGGTTGCTATTAACAAGAGATGGTGGTAATGAGACCAAT----------------------------
----

ACCACCGAGACCTTCAGACCTGGAGGAGGAGATATGAGAGACAATTGGAGAAGTGAATTATATAAATATAAAG
TAGTAAAAATTGAACCATTA---GGAGTAGCACCCACCAAGGCAAAGAGAAGAGTGGTG--------------

-------------------------CAGAGAGAAAAAAGAGCA---GTGGGAATGATAGGAGCT---------
ATGTTCCTTGGGTTCTTGGGAGCA---GCAGGAAGCACTATG---GGCGCAGCGTCA---
ATAACGCTGACGGTACAA---GCCAGACAA---TTGTTG---TCTGGCATAGTGCAACAGCAGAGA---
AATTTG---CTGAGG---GCTATTGAGGCGCAACAGCATCTGTTGCAACTCACAGTCTGGGGCATC
>JF932494
TTCTACTGTAATACAACAAAACTGTTTGATAGCACTTGGCATAAAAGTAATAATTGGACA-------------
----

GAAAATGGCACTATCGAGCTCCCCTGCAGAATAAAACAAATTGTAAACATGTGGCAGGAAGTAGGAAAAGCAA
TGTATGCCCCTCCCATCAGTGGACGAATTAGCTGTTTATCAAAT---
ATTACAGGGTTGCTCTTAACAAGAGATGGTGGTGGTAATGAGACCAAT-------------------------
----

ACGACCACCGAGATCTTTAGACCTGGAGGAGGAGATATGAGGGACAATTGGAGAAGTGAATTATATAAATATA
AAGTAGTAAAAATTGAACCATTA---GGAGTAGCACCCACCCAGGCAAAGAGAAGAGTGGTG-----------
----------------------------CAGAGAGAAAAAAGAGCA---GTGGGAACGATAGGAGCT------
---ATGTTCCTTGGGTTCTTGGGAGCA---GCAGGAAGCACTATG---GGCGCAGCGTCA---
ATAACGCTGACGGTACAG---GCCAGACAA---TTATTG---TCTGGTATAGTGCAACAGCAGAGA---
AATTTG---CTGAGG---GCTATTGAGGCGCAACAGCATCTGTTGCAACTCACAGTCTGGGGCATC
>JX140658
TTCTACTGTAATACATCAAAACTGTTTAATAGCACTTGGCCAAGTGATGGTACTGATGGTAATGTTACTGGAG
GG---

TCAAATGACACTATCACACTCCAATGCAGAATAAGACAAATTATAAACAGATGGCAGGAAGTAGGAAAAGCAA
TGTATGCTCCTCCCATCAGAGGAGTAATCAACTGTACATCAAAT---
ATTACAGGACTACTGTTAGTAAGAGATGGTGGGATTAACAACAGC----------------------------
----

ACTAATGAGACCTTCAGACCTGGAGGAGGAGATATGAGAGACAATTGGAGAAGTGAATTATACAAATACAAAG
TAGTAAGACTTGAGCCATTA---GGAGTAGCACCCACCGAGGCAAAGAGAATAGTGGTG--------------
-------------------------CAGAGAGAAAAAAGAGCA---GTAGGA---CTAGGAGCT---------
ATGTTCCTTGGGTTCTTGGGAGCA---GCAGGAAGCACGATG---GGCGCAGCGTCA---
ATAACGCTGATGGTACAA---GCCAGACAA---TTATTG---ACTGGTATAGTGCAACAGCAAAGC---
AATTTG---CTGAGG---GCTATTGAGGCGCAACAGCATATGTTGCAACTCACAGTCTGGGGCATC
>KX692916
TTCTACTGTAAGACATCACAACTGTTTAATAGTATTTGGTATAGTAATAGTACTTGGAATGATACTACAGGAA
AT---

AGTACTGGGAATATCACGCTCCCTTGCAGAATAAAACAAATTATAAACATGTGGCAGGAAGTAGGGAAAGCAA
TGTATGCCCCTCCCATTAGAGGACAAATTAGCTGTTTATCAAAT---
ATTACAGGGTTGCTATTAACAAGAGATGGTGGTAAGAATGAGAGC----------------------------
----

AATACCACCGAGACCTTTAGACCTGCAGGAGGAGATTTGAGAGACAATTGGAGAAGTGAATTATATAAATATA
AAGTAGTAAAAATTGAACCATTA---GGAGTAGCACCCACCAAGGCAAGGAGAAGAGTGGTG-----------
----------------------------CAAAGAGAAAAAAGAGCA---GTGGGAGCGCTAGGAGCT------
---ATGTTCCTTGGGTTCTTGGGAGCA---GCAGGAAGCACTATG---GGCGCAGCGTCA---
ATAACGCTGACGGTACAG---GCCAGACAA---TTGTTG---TCTGGTATAGTGCAACAGCAGAGA---
AATTTG---CTGAGG---GCTATTGAGGCGCAACAGCATCTGTTGCAACTCACAGTCTGGGGCATC
>KX692929
TTCTACTGTAATACATCACAACTGTTTAGTAGTACTTGGAATAATACTGGTACTCGGACTGGTACTACT----
----

GGAAATGACACTATCTCGCTCCCTTGCAGAATAAGACAAATTGTAAACATGTGGCAGGAAGTAGGGAAAGCAA
TGTATGCCCCTCCCATTGCAGGACAAATTAATTGTTCATCAAAT---
ATTACAGGGTTGCTATTAACAAGAGATGGTGGGGAGGGGAACGCGACCAGT----------------------
----

ACTAACGACACAGAGATCTTCAGACCTACAGGAGGAAATATGAGAGACAATTGGAGAAGTGAATTATATAAAT
ATAAAGTAGTAAAAATTGAACCATTA---GGAGTAGCACCCACCAGGGCAAAGAGAAGAGTGGTG--------

--------------------------------CAGAGAGAAAAAAGAGCA---GTGGGAACGATAGGAGCT---
------ATGTTCCTTGGGTTCTTGGGAGCA---GCAGGAAGCACTATG---GGCGCAGCGTCA---
ATAACGCTGACGGTACAG---GCCAGACAA---TTGTTG---TCTGGTATAGTGCAACAGCAGAGA---
AATTTG---CTGAGG---GCTATTGAGGCGCAACAGCATCTGTTGCAACTCACAGTCTGGGGCATC
>KX692941
TTCTACTGTAATACATCACAACTGTTTAATAGTAGGTGGGATGATGTGGATGAGAATGAGACTAGTACTAGTA
ATGGCACT---

ACAGGGAATGATACTATCACGCTCCCTTGCAGAATAAAACAAATTATAAACATGTGGCAGGAAGTAGGAAAAG
CAATGTATGCCCCTCCCATTGCAGGACTAATTAGATGCTCATCAAAT---
ATTACAGGGTTGCTATTAACAAGGGATGGTGGTAATATTAATGCTACT-------------------------
----
GGGAACAATACCAACAAGAATAAT---------
AACACCGAGATCTTCAGACCTGCAGGGGGAAATATGAAAGACAATTGGAGAAGTGAATTATATAGATATAAAG
TAGTAAAAGTTGAACCATTA---GGAGTAGCACCCACCAGGGCACAGAGAAGAGTGGTG--------------
-------------------------CAAAGAGAAAAAAGAGCA---GTGGGAATGATAGGAGCT---------
ATGTTCCTTGGGTTCTTGGGAACA---GCAGGAAGCACTATG---GGCGCAGCGTCA---
CTAACGCTGACGGTACAG---GCCAGACAA---TTGTTG---TCTGGTATAGTGCAACAGCAGAGA---
AATCTG---CTGAGG---GCTATTGAGGCGCAACAACATCTGTTGCAACTCACAGTCTGGGGCATC
>KX692973
TTCTACTGTAATACATCAAAACTATTTGATAGTACTTGGAATGCTACTAGTGATTGGAATGATACTGAG----
----

ACTACAAAAAAATATCACGCTCCCTTGCAGAATAAAACAAATTGTAAACATGTGGCAGGAAGTAGGGAAAGCAA
TGTATGCCCCTCCCATTGAAGGACAAATTGGATGTTCATCAAAT---
ATTACAGGGTTGCTATTAGCAAGAGATGGTGGTAATAGTGAGAAC----------------------------
----

ACCACCGAGACCTTCAGACCTGCAGGAGGAAACATGAGAGACAATTGGAGAAGTGAATTATATAGATATAAAA
TAGTAAAAATTGAACCATTA---GGAGTAGCACCCACCAAGGCAAAGAGAAGAGTGGTG--------------
-------------------------CAGAGAGAAAAAAGAGCA---GTGGGAACAATAGGAGCT---------
ATGTTCCTTGGGTTCTTGGGAGCA---GCAGGAAGCACTATG---GGCGCAGCGTCA---
ATAACGCTGACGGTACAG---GCCAGACAA---TTGTTG---TCTGGTATAGTGCAACAGCAAAGA---
AATTTG---CTGATG---GCTATTGAGGCGCAACAGCATCTGTTGCAACTCACAGTCTGGGGCATC
>KX692981
TTCTACTGTAATACATCACAACTGTTTAATAGTACTTGGTATAAAAACAGTACTTGGAATGGTGCTACA----
----

GGAAATGGCACTATCACGCTCCCTTGCAGAATAAAACAAATTGTAAACATGTGGCAGGAAGTAGGAAAAGCAA
TGTATGCCCCTCCCATTAGCGGACAAATTAGATGTTCATCAAAT---
ATTACAGGGTTGCTATTAACAAGAGATGGTGGTAATAATGGGAACAAT-------------------------
----

ACCACTGAGACTGAGACCTTCAGACCTGGAGGAGGAGATATGAGAGACAATTGGAGAAGTGAATTATATAAAT
ATAAAGTAGTAAAAATTGAACCATTA---GGAGTAGCACCCACCAAGGCAAAGAGAAGAGTGGTG--------
--------------------------------CAGAGAGAAAAAAGAGCA---GTGGGATTGGTAGGAGCT---
------ATGTTCCTTGGGTTCTTGGGAGCA---GCAGGAAGCACTATG---GGCGCAGCGTCA---
ATAACGCTGACGGTACAG---GCCAGACAA---TTGTTG---TCTGGTATAGTGCAACAGCAAAGA---
AATTTG---CTGAGG---GCTATTGAGGCGCAACAGCATCTGTTGCAACTCACAGTCTGGGGCATC
>KX693001
TTCTACTGTAATACATCACAACTGTTTAATAGTACTTGGAATAATAATGGCACTTGGAATGGTGCTACA----
----

GGAAACGACACTGTCACGCTCCCTTGCAGAATAAAACAAATTGTAAACATGTGGCAGGAAGTAGGGAAAGCAA
TGTATGCCCCTCCCATTAGAGGACTAATTAACTGTTCATCAAAT---
ATTACAGGGTTGCTATTAACAAGAGATGGTGGTACAAATGAGAGC----------------------------
----

AATACCACCGAGACCTTCAGACCTGGAGGAGGAGATATGAGAGACAATTGGAGAAGTGAATTATATAAATATA
AAGTAGTAAAAATTGAACCATTA---GGAGTAGCACCCACCAAGGCAAAGAGAAGAGTGGTG-----------

----------------------------CAGAGAGAAAAAAGAGCA---GTGGGAACGATAGGAGCT------
---ATGTTCCTTGGGTTCTTGGGAGCA---GCAGGAAGCACTATG---GGCGCAGCGTCA---
ATAACGCTGACGGTACAG---GCCAGACAA---TTGTTG---TCTGGTATAGTGCAACAGCAGAGA---
AATTTG---CTGAGA---GCTATTGAGGCGCAACAGCATATGTTGCAACTCACAGTCTGGGGCATC
>KX693029
TTCTACTGTAATACATCACAACTGTTTAACAGTACTTGGCCTTGGAATGATACTAGTACTGGGAATGATACTA
CA---

GGAAATAGCACTATCACGCTCCCTTGCAAAATAAGACAAATTGTAAACAGGTGGCAGGAAGTAGGGAAAGCAA
TGTATGCCCCTCCCATTGCAGGACCAATTAGCTGCTCATCAAAT---
ATTACAGGGTTGCTATTAACAAGAGATGGTGGTAATGGGAGCGCG----------------------------
----

GACAACGAGACCTTCAGACCTGGAGGAGGAGATATGAGAGACAATTGGAGAAGTGAATTATATAAATATAAAG
TAGTACAAATTGAACCATTA---GGAGTAGCACCCACCAAGGCAAAGAGAAGAGTGGTG--------------
-------------------------CAGAGAGAAAAAAGAGCA---GTGGGAACGATAGGAGCT---------
ATGTTCCTTGGGTTCTTGGGAGCA---GCAGGAAGCACTATG---GGCGCAGCGTCA---
ATAACGCTGACGGTACAG---GCCAGACAA---TTGTTG---TCTGGTATAGTGCAACAGCAGAGA---
AATTTG---CTGAGG---GCTATTGAGGCGCAACAGCATCTGTTGCAACTCACAGTCTGGGGCATC
>KX693036
TTCTACTGTAATACATCACAACTATTTAATAGTACTTGGAATAATAATAGTACTTGGAATGGTACCAGTTTT-
----

AAGAATGATACTATCACGCTCCCTTGTAGAATAAAACAAATTGTAAACATGTGGCAGGGAATAGGGAAAGCAA
TGTATGCCCCTCCCATTGCAGGACAAATTAACTGCTCATCAAAA---
ATTACAGGGTTGCTACTAACAAGAGATGGTGGTGGTAAGAATGAGAGC-------------------------
----

AATACCACCGAGACCTTCAGACCTGGAGGAGGAGATATGAAAGACAATTGGAGAAGTGAATTATATAAATATA
AAGTAGTAAAAATTGAACCATTA---GGATTAGCACCCACCAAGGCAAAGAGAAGAGTGGTG-----------
----------------------------CAGAGAGAAAAAAGAGCA---GTGGGAACGATAGGAGCT------
---ATGTTCCTTGGGTTCTTGGGAGCA---GCAGGAAGCACTATG---GGCGCAGCGTCA---
CTAACGCTGACGGTACAG---GACAGACAA---TTGTTG---TCTGGTATAGTGCAACAGCAAAGA---
AATTTG---CTGAGG---GCTATTGAGGCGCAACAGCATCTGTTGCAACTCACAGTCTGGGGCATC
>KX693575
TTCTACTGTAATACATCAAAACTGTTTAATAGTACTTGGAGTAATACTAGTGCTTGGAATGATACTACA----
----

GGAAATGACACTATCACACTCCCTTGCAGAATAAAACAAATTGTAAACATGTGGCAGGAAGTAGGGAAAGCAA
TGTATGCCCCTCCCATTAGAGGAAAAATTAGCTGTTCATCAAAC---
ATTACAGGGTTGCTATTAACAAGAGATGGTGGTAATAATGGGAGC----------------------------
----

AACACCAACGAGACCTTCAGACCTGCAGGAGGAGATATGAGAGACAATTGGAGAAGTGAATTATATAAATATA
AAATAGTAAAAATTGAACCATTA---GGAGTAGCACCCACCAAGGCAAAGAGAAGAGTGGTG-----------
----------------------------CAGAGAGAAAAAAGAGCA---GTGGGAACACTAGGAGCT------
---ATGTTCCTTGGGTTCTTGGGAGCA---GCAGGAAGCACTATG---GGCGCAGCGTCA---
ATAACGCTGACGGTACAG---GCCAGACAA---ATGTTG---TCTGGTATAGTGCAACAGCAAAGA---
AATTTG---CTGAGG---GCTATTGAGGCGCAACAGCATATGTTGCAACTCACAGTCTGGGGCATC
>KX693068
TTCTACTGTAATACATCAAAACTGTTTAATAGTACTTGGCCTAGTACTGGGAATGATACTCCTAGTACTGGGA
ATGATACT---

ACAGGAAATGGCACTATCACACTCCCTTGCAGAATAAAACAAATTGTAAACATGTGGCAGGAAGTAGGGAAAG
CAATGTATGCCCCTCCCATTAAAGGGCAAATTAGCTGTGTATCAAAT---
ATTACAGGGTTGCTATTAATGAGAGATGGTGGTAAT-------------------------------------
----

AATGAGAACGAGACCTTCAGACCTGGAGGAGGAGATATGAGAGACAATTGGAGAAGTGAATTATATAAATATA
AAGTAGTAAAAATTGAACCATTA---GGAGTAGCACCCACCAAGGCAAAGAGAAGAGTGGTG-----------

----------------------------CAGAGAGAAAAAAGAGCA---GTGGGA---ATAGGAGCT------
---ATGTTCCTTGGGTTCTTGGGAGCA---GCAGGAAGCACTATG---GGCGCAGCGTCA---
ATAACGCTGACGGTACAG---GCCAGGCAA---TTGTTG---TCTGGTATAGTGCAACAGCAGAGA---
AATTTG---CTGATG---GCTATTGAGGCGCAACAGCATCTGTTGCAACTCACAGTCTGGGGCATC
>KX693091
TTCTACTGTAATACATCACAATTGTTTAATAGTACCTGGAACAGTAATGGTACTCGGAATGGTACCACA----
----

GGAAATGACATTATCACGCTCCCTTGCAGAATAAAACAAATTGTAAACATGTGGCAGGAAGTAGGGAAAGCAA
TGTATGCCCCTCCCATTAGTGGACAAATTAGATGTTCATCAAAT---
ATTACAGGGTTGCTATTAACAAGAGATGGTGGTAAAAATGAGACC----------------------------
----

AACACCACCGAGACCTTCAGACCCGCAGGAGGAGATATGAGAGACAATTGGAGAAGTGAATTATATAAATATA
AAGTAGTAAAAATTGAACCATTA---GGAGTAGCACCCACCAAGGCAAAGAGAAGAGTGGTG-----------
----------------------------CAGAGAGAAAAAAGAGCA---GTGGGAACAATAGGAGCT------
---ATGTTCCTTGGGTTCTTGGGAGCA---GCAGGAAGCACTATG---GGCGCAGCGTCA---
ATAACGCTGACGGTACAG---GCCAGACAA---TTGTTG---TCTGGTATAGTGCAACAGCAGAGA---
AATCTG---CTGAGG---GCTATTGAGGCGCAACAGCATATATTGCAACTCACAGTCTGGGGCATT
>KX693093
TTCTACTGTAATACATCAAGACTGTTTAATGAGACTTGGGATAATATTACTACTGGGAATATTGGGAGTAACA
TTACA--

GAAGATGATATTATCACGCTCCCTTGCAGAATAAAACAAATTGTAAACAGGTGGCAGGAAGTAGGGAAAGCAA
TGTATGCCCCTCCCATTAGCGGACTAGTTAGATGTTCATCAAAT---
ATTACAGGGTTGCTATTAACAAGAGATGGTGGTACTAGTAAGGCCAATGACACC-------------------
----
AATACTGGGACCAATAAT---
AACACAGAAATTTTCAGACCTGGAGGAGGAGATATGAGAGACAATTGGAGAAGTGAATTATATAAATATAAAG
TAATAAAACTTGAACCATTA---GGAGTAGCACCCACCAAGGCAAAGAGAAGAGTGGTG--------------
-------------------------CAGAGAGAAAAAAGAGCA---GTGGGA---ATCGGAGCT---------
ATGTTCCTTGGGTTCTTGGGAGCA---GCAGGAAGCACCATG---GGCGCAGCGTCA---
ATAACGCTGACGGTACAG---GCCAGACAA---TTGTTG---TCTGGTATAGTGCAACAGCAGAGT---
AATTTG---CTGAGG---GCTATTGAGGCGCAACAACATCTGTTGCAACTCACAGTCTGGGGCATA
>KX693114
TTCTACTGTAACACATCAAAACTGTTTAATAGTACTTGGAGTAATACTAGTGCTTGGAATGATACTGCA----
----

GAAAATGACACTGTCACGCTCCCTTGCAGAATAAAACAAATTATAAACATGTGGCAGGGAGTAGGGAAAGCAA
TGTATGCCCCTCCCATTAGTGGACAAATTAGATGTTCATCAAAT---
ATTACAGGATTGCTATTAACAAGAGATGGTGGTAATGGTGGTAATAATGGGAGC-------------------
----

AATACCACCGAAACCTTCAGACCTGCAGGAGGAAATATGAAAGACAATTGGAGAAGTGAATTATATAAATATA
AAGTAGTACAAATTGAACCATTA---GGAGTAGCACCCACCAAGGCAAAGAGAAGAGTGGTG-----------
----------------------------CAAAAAGAAAAAAGAGCA---GTGGGAACAATAGGAGCT------
---ATGTTCCTTGGGTTCTTGGGAGCA---GCAGGAAGCACTATG---GGCGCAGCGTCA---
TTAACGCTGACGGTACAG---GCCAGACAA---CTGTTG---TCTGGTATAGTGCAACAGCAGAGA---
AATCTG---CTGAGG---GCTATTGAGGCGCAACAGCATCTGTTGCAACTCACAGTCTGGGGCATC
>KX693689
TTCTACTGTGATACATCACAACTGTTTAACAGTACTTGGGACAAGAATGGTACTTGGGAAGGTACTGTAGGAA
AT---

GGAAATGGCACTATCACTCTCCCCTGCAGGATAAAACAAATTGTAAACATGTGGCAGGAAGTAGGGAAAGCAA
TGTATGCCCCTCCCATTGCAGGACAAATTAAATGTTCATCAAAT---
ATTACAGGGTTACTATTAACAAGAGACGGTGGTAGTGGTAAAAAT----------------------------
----
GAGAGCAATACCACC---
GAGACCGAGACCTTCAGGCCTGCAGGAGGAGATATGAGAGACAATTGGAGAAGTGAATTATATAAATATAAAG
TAGTAAGAATTGAACCATTA---GGAGTAGCACCCACCAAGGCAAAGAGAAGAGTGGTG--------------

-------------------------CAAAGAGAAAAAAGAGCA---GTGGGAACACTAGGAGCT---------
ATGTTCCTTGGGTTCTTGGGAGCA---GCAGGAAGCACTATG---GGCGCAGCGTCA---
ATAACGCTGACGGTACAG---GCCAGACAG---TTATTG---TCTGGTATAGTGCAACAGCAGAGA---
AATTTG---CTGAGG---GCTATTGAGGCGCAACAGCATATGTTGCAACTCACAGTCTGGGGCATC
>KX693720
TTCTACTGTAATACATCACAACTGTTTAATAGTACTTGGAATGTTAATGGTAATTGGGATGGTACTACAGGAA
ATGATACT---

ACAGGAAATGGCACTATCATGCTCCCTTGCAGAATAAAACAAATTATAAACATGTGGCAGGAAGTAGGGAAAG
CAATGTATGCCCCTCCCATTAAAGGAAAGATTAAATGTTCATCAAAT---
ATTACAGGGTTGCTATTAACAAGAGATGGTGGTGAAAATGTGACC----------------------------
----

AACACCACCGAGACCTTCAGACCTGGAGGGGGAGATATGAGAGACAATTGGAGAAGTGAATTATATAAATACA
AAGTAGTAAAAATTGAACCATTA---GGAGTAGCACCCACTAAGGCAAAGAGAAGAGTGGTG-----------
----------------------------CAGAGAGAAAAAAGAGCA---GTGGGAACGATAGGAGCT------
---ATGTTCCTTGGGTTCTTGGGAGCA---GCAGGAAGCACTATG---GGCGCAGCGTCA---
ATAACGCTGACGGTACAG---GCCAGACAA---TTGTTG---TCTGGTATAGTGCAACAGCAGAGA---
AATTTG---CTGATG---GCTATTGAGGCGCAACAGCATCTGTTGCAACTCACAGTCTGGGGCATT
>KX693770
TTCTACTGTAATACATCAAAACTGTTTAGTAGTACTTGGTATAATAATGGTACTTGGACTAGTACTTGGAATG
AGACTATA---

GAAAATGACACTATCACACTCCCTTGCAGAATAAAACAAATTGTAAACATGTGGCAGGAAGTAGGGAGAGCAA
TGTATGCCCCTCCCATTGCAGGAAAAATTAGATGTTCATCAAAC---
ATTACAGGGTTGCTATTAACAAGAGATGGTGGTAATAATAATGAGACCAACGCC-------------------
----

ACCACCACCGAGGAGATCTTCAGACCTGCAGGAGGAGATATGAGAGACAATTGGAGAAGTGAATTATATAAAT
ATAAAATAGTAAAAATTGAACCATTA---GGAATAGCACCCACCAAGGCAAAGAGAAGAGTGGTG--------
-------------------------------CAGAGAGAAAAAAGAGCA---GTGGGAACGCTAGGAGCT---
------ATGTTCCTTGGGTTCTTGGGAGCA---GCAGGAAGCACTATG---GGCGCAGCGTCA---
ATAACGCTGACGGTACAG---GCCAGACAA---TTGTTG---TCTGGTATAGTGCAACAGCAAAGA---
AATTTG---CTGAGG---GCTATTGAGGCGCAACAGCATATGTTGCAACTCACAGTCTGGGGCATC
>KX693223
TTCTACTGTAATTCATCACAATTGTTTAATAGTACTTGGTATGTTAATGGTACTCGGATTGGTACTACA----
----

GGAAATGACACTATCACGCTCCCTTGCAGAATAAGACAAATTGTAAACATGTGGCAGGAAGTAGGGAAGGCAA
TGTATGCCCCTCCCATTGCAGGACCAATTAGATGTTTATCAAAT---
ATTACAGGGTTGCTATTAACAAGAGATGGTGGTAATAATACAGAT----------------------------
----

AATACCACCGAGACCTTCAGACCTGGAGGAGGAGATATGAGAGACAATTGGAGAAGTGAATTATATAAATATA
AAGTAGTAAAAATTGAGCCATTA---GGAGTAGCACCCACCAAGGCAAAGAGAAGAGTGGTG-----------
----------------------------CAGAGAGAAAAAAGAGCA---GTGGGAACAATAGGAGCT------
---ATGTTCCTTGGGTTCTTGGGAGCA---GCAGGAAGCACCATG---GGCGCAGCGTCA---
ATAACGCTGACGGTACAG---GCCAGACAA---TTGTTG---TCTGGTATAGTGCAACAGCAGAGA---
AATTTG---CTGAGG---GCTATTGAGGCGCAACAACATCTGTTGCAACTCACAGTCTGGGGCATC
>KX693238
TTCTATTGTAATACATCACAGCTGTTTAATAGTAATTGGAATGATAGTACTTTGAATGATAATAGTACTTGG-
----

AATGAAACTACTATCACGCTCCCTTGCAGAATAAAACAAATTGTAAACATGTGGCAGGAAGTAGGAAAAGCAA
TGTATGCCCCTCCCATTGCAGGACAAATTAACTGTTCATCAGAT---
ATTACAGGGTTGCTATTAACAAGAGATGGTGGTAAGAAGAATGAGAGCAAT----------------------
----

AATAACACTGAGACCTTCAGACCTGAAGGAGGAGATATGAGAGACAATTGGAGAAGTGAATTATACAAATATA
AAGTAGTAAAAATTGAACCATTA---GGAGTAGCACCCACCAAGGCAAAGAGAAGAGTGGTG-----------

---------------------------CAGAGAGAAAAAAGAGCA---GTGGGAACGATAGGAGCT------
---ATGTTCCTTGGGTTCTTGGGAGCA---GCAGGAAGCACTATG---GGCGCAGCGTCA---
ATAACGCTGACGGTACAG---GCCAGACAA---TTGTTG---TCTGGTATAGTGCAACAGCAGAGA---
AATTTG---CTGAGG---GCTATTGAGGCGCAACAGCATCTGTTGCAACTCACAGTCTGGGGCATC
>KX693247
TTCTACTGTAATACATCACAACTGTTTAATAGTACTTGGGATGATACTAATATTTGGAATGATACTAGTACTG
GGAATGGTACT--

ACAGAAAATAGCACTATCACGCTCCCTTGCAGAATAAAACAAATTGTAAACATGTGGCAGGAAGTAGGGAAAG
CAATGTATGCCCCTCCCATTGAAGGACGAATTAGATGTTCATCAAAT---
ATTACAGGGTTGCTACTAACAAGAGATGGTGGTAATGGGAGCGATACC------------------------
----

ACCAATAACACCGAGACCTTCAGACCTGGAGGAGGAGATATGAGAGACAATTGGAGAAGTGAATTATATAAAT
ATAAAGTAGTAAAAATTGAACCATTA---GGAGTAGCACCCACCAAGGCAAAGAGAAGAGTGGTG--------
------------------------------CAGAGAGAAAAAAGAGCA---GTGGGAACGATAGGAGCT---
------ATGTTCCTTGGGTTCTTGGGAGCA---GCAGGAAGCACTATG---GGCGCAGCGTCA---
ATAACGCTGACGGTACAG---GCCAGACAA---TTATTG---TCTGGTATAGTGCAACAGCAGAGA---
AATTTG---CTGAGG---GCTATTGAGGCGCAACAGCATCTGTTGCAACTCACAGTCTGGGGCATC
>KX693268
TTCTATTGTAATACATCACAACTGTTTAATAGTACTTGGTATACTAATAATACTTGGAATGGTACTACA----
----

GGAAATGATACTATCACGCTCCCTTGCAGAATAAAACAAATTGTAAACATGTGGCAGGAAGTAGGGAAAGCAA
TGTATGCCCCTCCTATTGCAGGAAAAATTAAATGTTCATCAAAT---
ATTACAGGGTTGCTATTAACAAGAGATGGTGGTATAAACAATACC---------------------------
----

AATACCACTGAGACCTTCAGACCTGGAGGAGGAGATATGAGAGACAATTGGAGAAGTGAATTATATAAATATA
AAGTAGTAAAAATTGAACCATTA---GGAATAGCACCCACCAAGGCAAAAAGAAGAGTGGTG-----------
---------------------------CAGAGAGAAAAAAGAGCA---GTAGGAGCACTAGGAGCT------
---ATGTTCCTTGGGTTCTTGGGAGCA---GCAGGAAGCACTATG---GGCGCAGCGTCA---
ATAACGCTGACGGTACAG---GCCAGACAA---TTGTTG---TCTGGTATAGTGCAGCAGCAGAGG---
AATCTG---CTGAGG---GCTATTGAGGCGCAACAGCATCTGTTGCAACTCACAGTCTGGGGCATA
>KX693886
TTCTACTGTAATACATCACAACTGTTTAATAGTACTTGGAACAATAGTATTTGGAATAATAATATTACTTGGA
GTGAT--
-------------------AAA---
ACAGGAAATGACACTATCTCGCTCCCTTGCAGAATAAAACAAATTATAAACACGTGGCAGAAAGTAGGAAAAG
CAATATATGCCCCTCCCATTGCAGGACAAATTAAATGTTCATCAAAT---
ATTACAGGGTTAATATTAACAAGAGATGGTGGTAATGAAAACAAGACC------------------------
----

ACCGAGACCGAGACCTTCAGACCTGGAGGAGGAGAGATGAGAGACAATTGGAGAAGTGAATTATATAAATATA
AAGTAGTAAAAATTGAACCATTA---GGAGTAGCACCTACCAAGGCAAAGAGAAGAGTGGTG-----------
---------------------------CAAAGAGAGAAAAGAGCA---GTGGGA---ATAGGAGCT------
---ATGTTCCTTGGGTTCTTGGGAGCA---GCAGGAAGCACTATG---GGCGCAGCATCA---
ATAACGCTGACGGTACAG---GCCAGACAA---TTGTTG---TCTGGTATAGTGCAACAGCAGAGA---
AATTTG---CTGAGG---GCTATTGAGGCGCAACAACAACTGTTGCAACTCACAGTCTGGGGCATC
>KX693314
TTCTACTGTAATACAACACGGCTGTTTAATAGTACTTGGAATGAGACTAGTACTTGGAATGATACTGCA----
----

GAAAATGACAATATCACGCTCCCTTGCAGAATAAGACAAATTATAAACAGGTGGCAGGAAGTAGGGAAGGCAA
TATATGCCCCTCCCATTGCAGGACAAATTAGATGTTCATCAAAT---
ATTACAGGGTTGCTATTAACAAGAGATGGTGGTAATAATGAGAAC---------------------------
----

GAGACCACCGAGATCTTCAGACCTGCAGGAGGAGATATGAAAGACAATTGGAGAAGTGAATTGTATAAATATA
AAGTAGTAAAAATTGAACCATTA---GGAGTAGCACCCACCAAGGCAAAGAGAAGAGTGGTG-----------

----------------------------CAGAGAGAAAAAAGAGCA---GTGGGAACGATAGGAGCT------
---ATGTTCCTTGGGTTCTTGGGAGCA---GCAGGAAGCACTATG---GGCGCAGCGTCA---
ATAACGCTGACGGTACAG---GCCAGACAA---TTGTTG---TCTGGTATAGTGCAACAGCAAAGA---
AATTTG---CTGAGA---GCTATTGAGGCGCAACAGCATCTGTTGCAACTCACAGTCTGGGGCATC
>KX693326
TTCTACTGTAATACATCAAAACTGTTCAATAGTACTTGGTATGGGAATGGTAATAGTACTTTGAATGATACCA
CA---

GAAAATAGCACTATCACGCTCCCTTGCAGAATAAAACAAATTGTAAACATGTGGCAGGAGGTAGGGAAAGCAA
TGTATGCCCCTCCCATTAAAGGACCAATTAGTTGTTCATCAAAT---
ATTACAGGGCTGCTATTAACAAGAGATGGTGGTAAAAATAATGATACTGAGAGC-------------------
----

AATACCACCGAGATCTTCAGACCTGGAGGAGGAGATATGAGAGACAATTGGAGAAGTGAATTATATAAATATA
AAGTAGTAAAAATTGAACCATTA---GGAGTAGCACCCACCAAGGCAAAGAGAAGAGTGGTG-----------
----------------------------CAGAGAGAAAAAAGAGCA---GTGGGAACGATAGGAGCT------
---ATGTTCCTTGGGTTCTTGGGAGCA---GCAGGAAGCACTATG---GGCGCAGCGTCA---
ATAACGCTGACGGTACAG---GCCAGACAA---TTGTTG---TCTGGTATAGTGCAACAGCAGAGA---
AATTTG---CTGAGG---GCTATTGAGGCGCAACAGCAGATGTTGCAACTCACAGTCTGGGGCATC
>KX693339
TTCTACTGTAATACATCAAAACTGTTTACTAATATTACAGGAAAT----------------------------
----

AGCACTGACCCTATCATGCTCCCTTGCAGAATAAAACAAATTGTAAACATGTGGCAGGAAGTAGGGAGAGCAC
TGTATGCCCCTCCCATTGAAGGACCAATTAATTGTTCATCAAAG---
ATTACAGGGTTGCTATTAACAAGAGATGGTGGTAATAGTGAGAAC----------------------------
----

AAGACCGAGACCTTCAGACCTGGAGGAGGAAATATGAGAGACAATTGGAGAAGTGAATTATATAAATATAAAG
TAGTAAAAATTGAACCATTA---GGAGTAGCACCCACCAAGGCAAAGAGAAGAGTGGTG--------------
-------------------------CAGAGAGAAAAAAGAGCA---GTGGGAACGATAGGAGCT---------
ATGTTCCTTGGGTTCTTGGGAGCA---GCAGGAAGCACTATG---GGCGCAGCGTCA---
ATAACGCTGACGGTACAG---GCCAGACAA---TTGTTG---TCTGGTATAGTGCAACAGCAGAGA---
AATTTG---CTGAGG---GCTATTGAGGCGCAACAGCATCTGTTGCAACTCACAGTCTGGGGCATC
>KX693374
TTCTACTGTAATACATCACAACTGTTTGATAGTACTTGGAATGATAGTAGTACTTGGAATGATACTAGTACTT
GGAATGTTACT--

ACAAAAAATGACACTATCACGCTCCCTTGCAGAATAAAACAAATTGTAAACATGTGGCAGGAAGTAGGGAAAG
CAATGTATGCCCCTCCTATTAGAGGACTAATTAGTTGTTCATCAAAA---
ATTACAGGGTTGTTATTAACAAGAGATGGTGGTAATAAGAATGATAGCAAT----------------------
----

ACCACCGAGATCGAGATCTTCAGACCTGGAGGAGGAGATATGAGAGACAATTGGAGAAGTGAATTATATAAAT
ATAAAGTAGTAAAAATTGAACCATTA---GGAGTAGCACCCACCAGGGCAAAGAGAAGAGTGGTG--------
-------------------------------CAAAGAGAAAAAAGAGCA---GTGGGAACGATAGGAGCT---
------ATGTTCCTTGGATTCTTGGGAGCA---GCAGGAAGCACTATG---GGCGCAGCGTCA---
TTAACGCTGACGGTACAG---GCCAGACAA---TTGCTG---TCTGGTATAGTGCAACAGCAGAGA---
AATTTG---CTGATG---GCTATTGAGGCGCAACAGCATCTGTTGCAACTCACAGTCTGGGGCATC
>KX693390
TTCTACTGTGATACATCACAACTGTTTAATAGTACTTGGGATGAAAATAGTACTAAGAATGATAATAATACTT
GGAAA--
-------------GATACT------
ACAGGAAATGACACTATCACACTCCATTGCAGAATAAAACAAGTTATAAACATGTGGCAGGAAGTAGGGAGAG
CAATGTATGCCCCTCCCATTAAAGGACAAATTAGATGTTCATCAAAT---
ATTACAGGGTTGCTATTAACAAGAGATGGGGGTACGAATGTGAGC----------------------------
----

AATACCAACGAGACCTTCAGACCTGGGGGAGGAAATATGAAAGACAATTGGAGAAGTGAATTATATAAATATA
AAGTAGTAAAAATTGAACCATTA---GGAGTAGCACCCACCAAGGCAAAGAGAAGAGTGGTG-----------

```
----------------------------CAGAGAGAAAAAAGAGCA---GTGGGG---CTCGGAGCT------
---ATGTTCCTTGGGTTCTTGGGAGCA---GCAGGAAGCACTATG---GGCGCAGCGTCA---
ATAACGCTGACGGTACAG---GCCAGACAA---TTGTTG---TCTGGTATAGTGCAACAGCAGAGA---
AATTTG---CTGAGG---GCTATTGAGGCGCAACAGCGTATGTTGCAACTCACAGTCTGGGGCATC
>KX693437
TTCTACTGTAATACATCACAACTGTTTAACAGTACTTGGAATGATAATAGTACTTGGAAATGGAACAGGAAAA
ATACT--------------------------------------------------------------------
-------------------------
ACAGATAATGACACTCTCACGCTCCCTTGCAGGATAAAACAAATTGTAAACATGTGGCAGGAAGTAGGGAAAG
CAATGTATGCCCCTCCCATTAGAGGACCAATTAAATGTAGATCAAAT---
ATTACAGGGCTGCTATTAACAAGAGATGGTGGCAATAATGGGACC----------------------------
-------------------------------------------------------------------------
----------
AGTAACAACGAGACCTTCAGACCTGGAGGAGGAGATATGAGAGACAATTGGAGAAGTGAATTATATAAATATA
AAGTAGTACAAATTGAACCATTA---GGAGTAGCACCCACCAGGGCAAAGAGAAGAGTGGTG-----------
----------------------------CAGAGAGAAAAAAGAGCA---GTGGGAACGATAGGAGCT------
---ATGTTCCTTGGGTTCTTGGGAGCA---GCAGGAAGCACGATG---GGCGCAGCGTCA---
ATAACGCTGACGGTACAG---GCCAGACAA---TTGCTG---TCTGGTATAGTGCAACAGCAGAGA---
AATTTG---CTGAGG---GCTATTGAGGCGCAACAACATCTGTTGCAACTCACCGTCTGGGGCATC
>KX693455
TTCTACTGCAATACATCACAACTGTTTAATAGTACTTGGAATGATACTAGTACTTGGAATGAGACTACA----
-------------------------------------------------------------------------
----------------------------
GGAAATAGCACTATCACGCTCCCTTGCAGAATAAGACAATTTGTAAACATGTGGCAGGAAGTAGGAAAAGCAA
TGTATGCCCCTCCCATTAGAGGACAAATTAGATGTTCATCAAAT---
ATTACAGGGTTGCTATTAACAAGAGATGGTGGTATTAATGGGAGC----------------------------
-------------------------------------------------------------------------
----------
GAGACCACCGAGACCTTCAGACCTGGAGGAGGAAATATGAGAGACAATTGGAGAAGTAAATTATATAAATATA
AAGTAGTAAAAATTGAACCATTA---GGAGTAGCACCCACCAAGGCAAAGAGAAGAGTGGTG-----------
----------------------------CAGAGAGAAAAAAGAGCA---GTGGGAACGATAGGAGCT------
---ATGTTCCTTGGGTTCTTGGGAGCA---GCAGGAAGCACTATG---GGCGCAGCATCA---
ATAACGCTGACGGTACAG---GCCAGACAA---TTGTTG---TCTGGTATAGTGCAACAGCAGAGA---
AATTTG---CTGAGA---GCTATTGAGGCGCAACAGCATCTGTTGCAGCTCACAGTCTGGGGCATC
>KX693469
TTCTACTGTGATACATCAAAACTGTTTAGTAGTACTTGGACTAATACTAGTACTTGGAATAAGACTGAGACTT
GGAATGTTTCT--------------------------------------------------------------
-------------------------
GCAGAACATGACACTATCACGCTCCATTGCAGAATAAAACAAATTGTAAACATGTGGCAGGAAGTAGGGAAAG
CAATGTATGCCCCTCCCATTGCAGGACAAATTAACTGTTCATCAAAT---
ATTACAGGGTTGCTATTAACAAGAGATGGTGGTAATAATACCAAT----------------------------
-------------------------------------------------------------------------
-------------
AACACCGAGACCTTCAGACCTGGAGGAGGAAATATGAGAGACAATTGGAGAAGTGAATTATATAAATATAAAG
TAGTAAAAATTGAACCATTA---GGAGTAGCACCCACCAAGGCAAAGAGAAGAGTGGTG--------------
-------------------------CAGAGAGAAAAAAGAGCA---GTGGGAACGCTAGGAGCT---------
ATGTTCCTTGGGTTCTTGGGAGCA---GCAGGAAGCACTATG---GGCGCAGCGTCA---
CTAACGCTGACGGTACAG---GCCAGACAA---TTGCTG---TCTGGTATAGTGCAACAGCAGAGA---
AATTTG---CTGAGG---GCTATTGAGGCGCAACAGCGTATGTTGCAACTCACAGTCTGGGGCATC
>KX693475
TTCTATTGTAATACATCAAAGCTGTTTGATAGTACTTGGAATAATACTAGTACTGAGAATAAT----------
-------------------------------------------------------------------------
----------------------------
AATGATACTAATATCACGCTCCCTTGCAGAATAAGACAAATTATAAACAGGTGGCAGGAAGTAGGGAAAGCAA
TGTATGCCCCTCCCATTGCAGGACAAATTAGATGTTCATCAAAT---
ATCACAGGGTTAATATTAACAAGAGATGGTGGTAATAATGGGACC----------------------------
-------------------------------------------------------------------------
----------
AACAACACTGAGATCTTCAGACCTGCAGGAGGAGATATGAGGGACAATTGGAGAAGTGAATTATATAAATATA
AAGTAGTAAAAATTGAACCATTA---GGAGTAGCACCCACCAAGGCAAAGAGAAGAGTGGTG-----------
```

----------------------------CAGAGAGAAAAAAGAGCA---GTGGGA---TTAGGAGCT------
---ATGTTCCTTGGGTTCTTGGGAGCA---GCAGGAAGCACTATG---GGCGCAGCGTCA---
ATAACGCTGACGGTACAG---GCCAGACAA---TTGTTG---TCTGGTATGGTGCAACAGCAGAGA---
AATTTG---CTGAGG---GCTATTGAGGCGCAACAACATCTGTTGCAACTCACAGTCTGGGGCATC
>KX693489
TTCTACTGTAATACATCACAATTGTTTAATAGTACTTGGACTTGGAATGATACTAGTGCTTGGAATAATAGTA
CA---

GAAAATGACACTATCACGCTCCCTTGCAGAATAAAACAAATTGTAAACATGTGGCAGGAAGTAGGGAAAGCAA
TGTATGCCCCTCCTATTGCAGGACAAATTAGATGTTTATCAAAT---
ATTACAGGGTTGCTATTAACAAGAGATGGTGGTAAGAATGAGAGC----------------------------
----

AACACCACCGAGACCTTCAGACCTGCAGGAGGAGATATGAGAGACAATTGGAGGAGTGAATTATATAAATATA
AAGTAGTAGAAATTGAACCATTA---GGAGTAGCACCCACCAAGGCAAAGAGAAGAGTGGTG-----------
----------------------------CAGAGAGAAAAAAGAGCA---GTGGGAACGATAGGAGCT------
---ATGTTCCTTGGGTTCTTGGGAGCA---GCAGGAAGCACTATG---GGCGCAGCGTCA---
ATAACGCTGACGGCACAG---GCCAGACAA---TTGTTG---TCTGGTATAGTGCAACAGCAGAGA---
AATTTG---CTGAGG---GCTATTGAGGCGCAACAGCATCTGTTGCAACTCACAGTCTGGGGCATC
>KX693491
TTCTACTGTGATACATCACAACTGTTTAACAGTACTTGGCCTTGGAATGATAATAGTACTTGGAATAATTCTA
CA---

GGAAATGGCACTATCACGCTCCCTTGCAGAATAAAACAAATTATAAACATGTGGCAGGAAGTAGGGAAAGCAA
TGTATGCCCCTCCCATTGCAGGGCAAATTAACTGTTCATCAAAA---
ATTACAGGGTTGCTATTAACAAGAGATGGTGGTATTGATAATACTACGAAC----------------------
----
GATACCACCAAGAAC---
AACACCGAGACCTTCAGACCTGCAGGAGGAGATATGAGGGACAATTGGAGAAGTGAATTATATAAATATAAAG
TAGTAAAAATTGAACCATTA---GGAGTAGCACCCACCAAGGCAAAGAGAAGAGTGGTG--------------
-------------------------CAGAGAGAAAAAAGAGCA---GTAGGAACGATAGGAGCT---------
ATGTTCCTTGGGTTCTTGGGAGCA---GCAGGAAGCACTATG---GGCGCAGCGTCA---
ATAACGCTGACGGTACAG---GCCAGACAA---TTATTG---TCTGGTATAGTGCAACAGCAGAGA---
AATTTG---CTGAGG---GCTATTGAGGCGCAACAGCATATATTGCAACTCACAGTCTGGGGCATC
>KX693496
TTCTACTGTAATACATCACAATTATTTAATAGTACTTGGAATTGGAATGATACTATTCCTTGGAATGATACTA
CA---

GGAAATGACACTATCGTGCTCCCTTGCAAAATAAAACAAATTGTAAACATGTGGCAAGAGGTAGGGAAAGCAA
TGTATGCCCCTCCCATTAGTGGACAAATTAGATGTTCCTCAAAT---
ATTACAGGGTTACTATTAACAAGAGATGGTGGTATTAATGAGAGC----------------------------
----

AACACCGCCGAGACCTTCAGACCTGGAGGAGGAGATATGAGAGACAATTGGAGAAGTGAATTATATAAATATA
AAGTAGTAAAAATTGAACCATTA---GGAGTAGCACCCACCAAGGCAAAGAGAAGAGTGGTG-----------
----------------------------CAGAGAGAAAAAAGA---------------CTAGGAGCT------
---ATGTTCCTTGGGTTCTTGGGAGCA---GCAGGAAGCACTATG---GGCGCAGCGTCA---
ATAACGCTGACGGTACAG---GCCAGACAA---TTATTG---TCTGGTATAGTGCAACAGCAGAGA---
AATTTG---CTGAGG---GCTATTGAGGCGCAACAACATCTGTTGCAACTCACAGTCTGGGGCATC
>KX693539
TTCTACTGTGATACATCACGACTGTTCAATAGTACTTGGACTAATACTGGTACTTGGAATGCTACTACAGGAA
ATAATGAT---

ACTACAGGAAATATTATCAGACTCCCTTGTAGGATAAAACAAATTGTAAACATGTGGCAGGAAGTAGGGAGGG
CAATGTATGCCCCTCCCATTGAAGGACAAATTAGATGTTCATCTAAT---
ATTACAGGGTTGCTATTAACAAGAGATGGTGGTGAGAATGAGAGC----------------------------
----

AATACCACCGAAACCTTCAGACCTGAAGGAGGAGATATGAGGGACAATTGGAGAAGTGAATTATATAAATATA
AAGTAGTAAAAATTGAACCATTA---GGAGTAGCACCCACCAAGGCAAAGAGAAGAGTGGTG-----------

----------------------------CAGAGAGAAAAAAGAGCA---GTGGGAACGATAGGAGCT------
---ATGTTCCTTGGGTTCTTGGGAGCA---GCAGGAAGCACTATG---GGCGCAGCGTCA---
ATAACGCTGACGGTACAG---GCCAGACAA---TTGTTG---TCTGGTATAGTGCAACAGCAGAGA---
AATTTG---CTGAGG---GCTATTGAGGCGCAACAGCATCTGTTGCAACTCACAGTCTGGGGCATC
>KP109512
TTCTACTGTAATACAACACAACTGTTTAATAGTACTTGGAATGGTACTATGTTTAATGGTAGTAGTACTTGGA
ATAATACT--
----------GGGAATAGT------
ACTGAAAATGACACTATCACCCTCCCATGCAGAATAAAACAAATTATAAACATGTGGCAGGAAGTAGGAAAAG
CAATGTATGCCCCTCCCATCAGTGGACTAATCAGATGTTCATCAAAT---
ATTACAGGACTGATATTAACAAGAGATGGGGGGGTGGAACAGAACAGCACG---------------------
----

AACGACACCGAGACCTTCAGGCCTGGAGGAGGAGATATGAGGGACAATTGGAGAAGTGAATTATATAAATACA
AAGTAGTGAAACTTGAACCATTA---GGAGTAGCACCCACTAGGGCAAAAAGAAGAGTGGTG----------
----------------------------CAGAGAGAAAAAAGAGCA---TTGGGA---CTAGGAGCT------
---ATGTTCCTTGGGTTCTTGGGAACA---GCCGGAAGCACTATG---GGCGCAGCGTCA---
ATAACGCTGACGGTACAG---GCCAGACAA---TTATTG---TCTGGTATAGTGCAACAGCAGAAC---
AATTTG---CTGAGG---GCTATTGAGGCGCAACAGCATCTGTTGCAACTCACAGTCTGGGGCATC
>JF932495
TTCTACTGTAATACATCAAAACTGTTTAATAGTACTTGGAATGAGACTAGTGCTTGGAATGATACTGCA----
----

GAAAATGACACTATCACGCTCCCTTGCAGAATAAAACAAATTGTAAACATGTGGCAGGAAGTAGGAAGAGCAA
TGTATGCCCCTCCCATCAGAGGACAAATTAGATGTTCATCAAAC---
ATTACAGGGTTGCTATTAACAAGAGATGGTGGTAAGAATGAGAGC---------------------------
----

AATACCACCGAGACCTTCAGACCTGGAGGAGGAGATATGAAGGACAATTGGAGAAGTGAATTATATAAATATA
AAGTAGTAAAAATTGAACCATTA---GGAGTAGCACCCACCAAGGCAAAGAGAAGAGTGGTG----------
----------------------------CAGAGAGAAAAAAGAGCG---GTGGGAGCGATAGGAGCT------
---ATGTTCCTTGGGTTCTTGGGAACG---GCAGGAAGCGCTATG---GGCGCAGCGTCA---
CTGACGCTGACGGTACAG---GCCAGACAA---TTGTTG---TCTGGTATAGTGCAACAGCAAAGA---
AATTTG---CTGAGG---GCTATTGAGGCGCAACAGCATCTGTTGCAACTCACCGTCTGGGGCATC
>JF932496
TTCTACTGTAATACAACACAATTGTTTAATAGTACTTGGACTTGGAATGATATTAGTACTTGGAATAATACTA
CA--

GGAAATAGCACTATCACGCTTCCTTGCAGAATAAAACAAATTATAAACATGTGGCAGGAAGTAGGAACAGCAA
TGTATGCCCCTCCCATCGAAGGAAAAATTAGATGTTCATCAAAT---
ATTACAGGGTTACTATTAACAAGAGATGGTGGTAATAAGAACGAGACC------------------------
----

CCCGACACCGAGACCTTCAGACCTACAGGAGGAAATATGAGGGACAATTGGAGAAGTGAATTATATAAATATA
AAGTAGTAAAAATTGAACCATTA---GGAGTAGCACCCACCAGGGCAAAGAGAAGAGTGGTG----------
----------------------------CAAAGAGAAAAAAGAGCA---GTGGGAACGATAGGAGCT------
---ATGTTCCTTGGGTTCTTGGGAGCA---GCAGGAAGCACTATG---GGCGCAGCGTCA---
ATAACGCTGACGGTACAG---GCCAGACAA---TTGCTG---TCTGGTATAGTGCAACAGCAGAGA---
AATTTG---CTGAGA---GCTATTGAGGCGCAACAGCATATGTTGCAACTCACCGTCTGGGGCATC
>GU647196
TTCTACTGTAATACAGCACAACTGTTTAATAGTACTTGGAATAATACTAGTACTTGGAATGGTACTACA----
----

GGAAATAGCATTATTACGCTCCCTTGCAGGATAAGACAAATTGTAAACATGTGGCAGAAAGTAGGGAAAGCAA
TGTATGCCCCTCCCATTGAAGGACCAATTAAATGCTCATCAAAT---
ATTACAGGGTTGCTATTAACAAGAGATGGTGGACATAATGAGAGC---------------------------
----

AATACCACTGAGACCTTCAGACCTGGAGGAGGAGATATGAGAGACAATTGGAGGAGTGAATTATATAAATATA
AAGTAGTAAAAATTGAACCATTA---GGAGTAGCACCCACCAAGGCAAAGAGAAGAGTGGTG----------

----------------------------CAGAGAGAAAAAAGAGCA---GTGGGAATGATAGGAGCT------
---ATGTTCCTTGGGTTCTTGGGAGCA---GCAGGAAGCACTATG---GGCGCAGCGTCA---
ATAACGCTGACGGTACAG---GCCAGACAG---TTGTTG---TCTGGTATAGTGCAACAGCAGAGA---
AATTTG---CTGAGG---GCTATTGAGGCGCAACAGCATCTGTTGCAACTCACAGTCTGGGGCATC
>EU363830
TTCTACTGTAATACATCACAATTGTTTAACAGTACTTGGACTCCTAATAGTACTTGGAATGGTACTGGTACT-
----

TGGAATGATACTATCACACTCCCTTGCAGGATAAAACAAATTGTAAACATGTGGCAGGAAGTAGGGAAAGCAA
TGTATGCCCCTCCCATTGAAGGACCAATTAGATGTTCATCAAAT---
ATTACAGGGTTATTATTAACAAGAGATGGTGGTAAAAATGAGAACAACACT----------------------
----

GAGAACAACACCGAGACCTTCAGACCTGCAGGAGGAGATATGAGAGACAATTGGAGAAGTGAATTATATAAAT
ATAAAGTAGTAAAAATTGAACCATTA---GGAGTAGCACCCACCAGGGCAAAGAGAAGAGTGGTG--------
-------------------------------CAGAGAGAAAAAAGAGCA---GTGGGAACGATAGGAGCT---
------ATGTTCCTTGGGTTCTTGGGAGCA---GCAGGAAGCACTATG---GGCGCAGCATCA---
ATAACGCTGACGGTACAG---GCCAGACAA---TTATTG---TCTGGTATAGTGCAACAGCAGAGA---
AATTTG---CTGAGG---GCTATTGAGGCGCAACAGCATCTGTTGCAACTCACAGTCTGGGGCATC
>AY561236
TTCTACTGTAATACAACACAACTGTTTAATAGTACTTGGAATGAGAATAGTACTTGGAATGGGACTATTACTG
AAAAT--

TCAAATAACACTAATATCACACTCCCATGTAAAATAAAGCAAATTATAAATATGTGGCAGAGAGTAGGAAAAG
CAATGTATGCCCCTCCCATCAGAGGAGTAATAAGATGTGTATCAAAC---
ATCACAGGGCTACTATTAACAAGAGATGGTGGGAACAACGATACT----------------------------
----

AACAACAATGAGACCTTCAGACCTGGAGGAGGAAACATGAGGGATAATTGGAGAAGTGAATTATATAAATATA
AAGTAGTAAAAATTGAACCATTA---GGAGTAGCACCCACCAAGGCAAAGAGAAGAGTGGTG-----------
----------------------------CAGAGAGAAAAAAGAGCA---GTAACA---CTAGGAGCT------
---ATGTTCCTTGGGTTCTTGGGAGCA---GCAGGAAGCACTATG---GGCGCAGCGTCA---
ATAACGCTGACGGTACAG---GCCAGACAA---TTATTG---TCTGGTATAGTGCAACAGCAGAAC---
AATCTG---CTGAGG---GCTATTGAGGCGCAACAACATCTGTTGCAACTCACAGTCTGGGGCATT
>AY561239
TTCTACTGTAATACAGCACAACTGTTTAATAGTACTTGGAATGCTACTGATACTTGGAATACTACTACAGGGT
TAAAT--

GATACTGGTAAAAATATCACACTCCCATGCAGAATAAAACAAATTATAAACATGTGGCAGACAGTAGGAAAAG
CAATGTATGCCCCTCCCATCAGAGGACAAATTAGGTGTTCATCAAAT---
ATCACAGGGTTGTTATTAACAAGAGATGGAGGTAATAACAGCATA----------------------------
----

AACACAGAGATCTTCAGACCTGGAGGAGGAGATATGAGGGACAATTGGAGAAGTGAATTATATAAATATAAAG
TAGTAAAAATTGAACCATTA---GGAGTAGCACCCACCAAGGCAAAGAGAAGAGTGGTG--------------
-------------------------CAGAGGGAAAAAAGAGCA---GTAACA---CTAGGAGCT---------
ATGTTCCTTGGGTTCTTGGGAGCA---GCAGGAAGCACTATG---GGCGCAGCATCA---
ATAACGCTGACGGCACAG---GCCAGACAA---TTATTG---TCTGGTATAGTGCAACAGCAGAAC---
AATCTG---CTGAGG---GCTATTGAGGCGCAACAGCATATGTTGCAGCTCACAGTCTGGGGCATC
>HQ377377
TTCTACTGTAATACAACAAAATTGTTTAATAGTACTTGGTATAGTAATGGAACGCGACTTGCAAATGACAGTG
AT---

ACAAGTGAAAACATCACACTCCCATGCAGAATAAAACAAATTGTAAACATGTGGCAGGAAGTAGGAAAAGCAA
TGTATGCCCCTCCTATCAGCGGACAAATTAGATGTGTATCAAAT---
ATAACAGGGCTACTATTAACAAGGGATGGTGGGAAAGGGAATGGGAACGGA----------------------
----

ACGAACAATAACACCGAGACCTTCAGACCTGAAGGAGGAAATATGAAGGATAATTGGAGAAGTGAATTATACA
AATATAAAGTAGTAAAAGTTGAGCCATTA---GGAGTAGCACCCACCAAGGCAAAGAGAAGAGTGGTG-----

```
----------------------------------CAGAGAGAAAAAAGAACA---GTGGGA---
CTGGGAGCT---------TTGTTCCTTGGGTTCTTGGGAGCA---GCAGGAAGCACTATG---
GGCGCAGCGTCA---GTGACGCTGACGGTACAA---GCCAGACAA---TTATTG---
TCTGGTATAGTGCAACAGCAGAAC---AACTTG---CTGATG---
GCTATTGAGGCGCAACAAAATATGTTGCGACTCACAGTCTGGGGCATC
>AY586542
TTCTACTGTAATACAACAAAACTGTTTAATAGTACTTGGGATGTTAATAATAATATTAGCGGGCAAAATAAC-
-------------------------------------------------------------------------
----------------------------
ACTGAAGCTAATATTACACTCCCATGTAGAATAAAACAAATTGTAAACCTGTGGCAGGAAGTAGGAAAAGCAA
TGTATGCCCCTCCCATCAAAGGACAAATTAGCTGTTCATCAAAT---
ATTACAGGGCTGCTATTAACAAGAGATGGTGGTAATAACGGGACC----------------------------
-------------------------------------------------------------------------
----------
AATAACACCGAGACCTTCAGACCTGGAGGAGGAGATATGAGGGACAATTGGAGAAGTGAATTATATAAATATA
AAGTAGTAAAAATTGAACCATTA---GGAGTAGCACCCACCAAGGCAAAGAGAAGAGTGGTG-----------
----------------------------CAAAGAGAAAAAAGAGCA---GTGGGAATGGTAGGAGCT------
---ATGTTCCTTGGGTTCTTGGGAGCA---GCAGGAAGCACTATG---GGCGCAGCGGCA---
ATGACGCTGACGGTACAG---ACCAGACAA---TTATTG---TCTGGTATAGTGCAGCAGCAAAAC---
AATTTG---CTGAGG---GCTATTGAGGCGCAACAACATCTGTTGCAACTCACAGTCTGGGGCATC
>EU668963
TTCTACTGTAATACAACAAGGCTGTTTAATAGTACTTGGAATGGGAATAATACTTGGAATAATACTGATGACG
CTGCTACT-----------------------------------------------------------------
-------------------ATC---
ACTGAGAATGCGACTATCACACTCCCATGTAGAATAAAACAAATTATAAACAGGTGGCAGGAAGTAGGAAAAG
CAATGTATGCCCCTCCCATCCGAGGACATATTAGCTGTTTATCAAAC---
ATTACAGGGCTGATACTAACAAGAGATGGTGGTAAGGGCAATGACACC-------------------------
-------------------------------------------------------------------------
-------
AACAACAACACTGAGGTCTTCAGACCTGGAGGAGGAGATATGAGGGACAATTGGAGAAGTGAATTATATAAAT
ATAAAGTAGTAAGAATTGAGCCATTA---GGAATAGCACCCACCAAGGCAAGGAGAAGAGTGGTG--------
-------------------------------CAGAGAGAAAAAAGAGCA---GTAACA---ATAGGAGCG---
------ATGTTCCTTGGGTTCTTGGGAGCA---GCAGGAAGCACTATG---GGCGCAGCGTCA---
ATAACGCTGACGGTACAG---GCCAGACTA---TTATTG---TCTGGTATAGTGCAGCAGCAGAGC---
AATATG---CTGAGA---GCTATTGAGGCGCAACAACACCTGTTGCAACTCACAGTTTGGGGCATC
>FJ388890
TTCTACTGTAATACAACAGCACTGTTTAATAGTACTTGGAATAATACTGGATGGTCAGCTGACACAAATGGCA
CTAAT--------------------------------------------------------------------
-------------------------
GGCACTGAAGGAAATATCACACTCAAATGCAGAATAAAACAATTTATAAACCTGTGGCAGCAAGTAGAAAAAG
CAATGTACGCCCCTCCCATCGAAGGACTAATTAGATGCTCATCAAAC---
ATTACCGGGATACTATTAACAAGAGATGGGGGCAATAATAATGGGACC-------------------------
-------------------------------------------------------------------------
----------
AACACGACAGAGGTATTCAGACCTGGAGGAGGAAATATGAAAGATAATTGGAGGAGTGAATTATATAAATATA
AAGTAGTACAAATTGAACCCTTA---GGAATAGCACCCAACAAGGCACAGAGAAGAGTGGTG-----------
----------------------------CAGAGAGAAAAAAGAGCA---GTAGGA---ATAGGAGCT------
---GTGTTCCTTGGGTTCTTGGGAGCA---GCAGGAAGCACTATG---GGCGCAGCATCA---
GTGATGCTGACGGTACAA---GCCAGACAA---TTATTG---TCTGGTATAGTGCAACAGCAAAAC---
AATTTG---CTGAGA---GATAGGGAGGCGCAACAACATCTGTTGCAACTCCCTGTCTGGGGCATC
>FJ388895
TTCTACTGTAACACAACACCACTGTTCAATAGTACTTGGAATCCTAATGGTATTTGGGAGGGTACTACAGGGT
CAAATGAC-----------------------------------------------------------------
-------------------ACT---
GGGTCAAATGACACTGTCACACTCCAATGCAGAATAAAACAAATTGTAAACCTGTGGCAGGAAGTAGGAAAAG
CAATGTATGCCCCTCCCATCGAAGGACCAATTAAATGTTCATCAAAT---
ATTACAGGGATGCTATTAACAAGAGATGGTGGTAATAGTACTAAT----------------------------
-------------------------------------------------------------------------
----------------
AATGAGACCTTCAGACCTGGAGGAGGAGATATGAGGGACAATTGGAGAAGTGAACTATATAAATATAAAGTAG
```

TAAAAATTGAACCATTA---GGAATAGCACCCACCAAGGCAAAGAGAAGAGTGGTG-----------------
----------------------CAGAGAGACAAAAGAGCA---GTGGGAATAATGGGAGCT---------
ATGTTCCTTGGGTTCTTGGGAACA---GCAGGAAGCACTATG---GGCGCAGCGGCA---
CTGACGCTGACGGTACAG---GCCAGACAA---TTATTG---TCTGGTATAGTGCAACAGCAGAAT---
AATTTG---CTGAGG---GCTATTGAGGCGCAGCAGCATATGTTGCGACTCACTTTTTTTGGCATC
>FJ388898
TTCTACTGTAATTCAACACCACTGTTTAATAATACCTGGAATGGTACCGAATTGACAAATAATAATAATAATG
AAGAAAAT---
-------------ATCACA------
AATAATGAAGAAAATATCACACTCCAATGCAGAATAAAACAAATTATAAACAGGTGGCAGGAAGTAGGAAAAG
CAATGTATGCCCCTCCCATTGCAGGACAAATTAGATGCTCATCAAAC---
ATTACAGGGCTGCTATTGACAAGAGATGGTGGTAATGATAGTAAC-----------------------------
----

ACAACCGAAATCTTCAGACCTGGAGGAGGAGATATGAGGGACAATTGGAGAAGTGAATTATATAAATATAAAG
TAGTAAAGCTTGAACCATTA---GGAATAGCACCCACCAAGGCAAAGAGAAGAGTGGTG--------------
-------------------------CAAAGAGAAAAAAGAGCA---GTGGGA---ATAGGAGCT---------
ATGTTCCTTGGGTTCTTGGGAGCA---GCAGGAAGCACTATG---GGCGCAGCGTCA---
ATAACGCTGACGGTACAG---GCCAGACAA---TTATTG---TCTGGTATAGTGCAACAGCAGAGC---
AATCTG---CTGAGG---GCTATTGAGGCGCAACAGCATATGTTGCAACTCACAGTTTGGGGCATC
>FJ388905
TTCTACTGTAATACAACATCACTGTTTAATAGTACTTTGAAACCAAAAAATAGCACTATCCAAAGATATAGAG
ATAAT--

AGAGATAATGACACTATTGTACTCCAATGCAGAATAAAACAATTTATAAACATGTGGCAGGAAGTAGGAAAGG
CAATGTATGCCCCTCCCATCAGAGGACAGCTTAACTGCTCATCAAAT---
ATTACAGGGCTGCTATTAACAAGAGATGGTGATGTTAATATCACCGAG-------------------------
----

AACGGGACTGAGACCTTCAGACCTATAGGAGGAGATATGAGGGACAATTGGAGAAGTGAATTATATAAATATA
AAGTAATAAAACTTGAACCATTA---GGAGTAGCACCCACCAAGGCACAGAGAAGAGTGGTG-----------
-----------------------------CAGAGAGAAAAAAGAGCA---GCGATA------GGAGCT------
---ATGATCCTTGGGTTCTTGGGAGCA---GCAGGAAGCACTATG---GGCGCAGCGTCA---
GTGACGCTGACGGTACAG---GCCAGACTA---TTATTG---TCTGGTATAGTGCAGCAGCAGAGC---
AATCTG---CTGAGG---GCTATTGAGGCGCAACAACATCTGTTGCAACTCACAGTCTGGGGCATC
>FJ388910
TTCTACTGTAATACAACACAACTGTTTAATAGTACCTGGAATGCTGATGGTACTCGGAACGATACTGAAGGGT
CAGATAAC---
--------------------AAC---
AACAATACAGAAACAATCACACTCCCATGCAGAATAAAGCAAGTCATAAACCTGTGGCAGGAAGTAGGAAAAG
CAATGTATGCCCCTCCCATCAGAGGACAAATTAACTGTTCCTCAAGC---
ATTACAGGGCTACTATTAACAAGAGACGGTGGTGACAACTCCACT-----------------------------
----

AGCAGCCCTGAGATCTTCAGACCTGGAGGAGGAGATATGAGGGACAATTGGAGAAGTGAATTATATAAATATA
AAGTAGTAAGAGTTGAACCATTA---GGAGTAGCACCCACCAAGGCAAAGAGAAGAGTGGTG-----------
-----------------------------CAGAGAGAAAAAAGAGCA---GTGACA---CTAGGAGCT------
---GTGTTCCTTGGGTTCTTGGGAGCA---GCAGGAAGCACTATG---GGCGCAGCGTCA---
TTGACGCTGACGGTACAG---GCCAGACAA---TTATTG---TCTGGTATAGTGCAACAGCAAAAC---
AATTTG---CTGAGG---GCTATTGAGGCGCAACAGCACCTGTTGCAACTCACAGTCTGGGGCATC
>FJ388911
TTCTACTGTAATACAACACAGCTGTTTAATAGTACTTGGTGGGCTAATAGTACTGGGCCTGGTAATGGAAATG
GAAATATC---
--------------------ACA---
ATCACATCTGAAAGCATCATAACTCCATGCAGAATAAAACAACTTATAATCAGAAGACCGCAAGAAGTAAGAA
CAACATCTGCCCCTCCTATAGCAGCAGAAAATATTTGTTGTTCAAAA---
ATTACAAGAGTGCTATTAACAACAGAAGGTGGTGATAATACAAAC-----------------------------
----

GGGAGTGAGACCTTCAGACCTGGAGGAGGAAATATGAGGGACAATTGGAGAAGTGAATTATATAAATATAAAG

TAGTAAGAATTGAACCATTA---GGAGTAGCACCCACCAGGGCAAAGAGAAGAGTGGTG------------
------------------------CAAAGAGAAAAAGAGCA---GTGGGAACAATAGGAGCT---------
ATGTTCCTTGGGTTCTTGGGAGCA---GCAGGAAGCACTATG---GGCGCAGCGTCA---
ATAACGCTGACGGTACAG---GCCAGACAA---TTATTG---TCTGGTATAGTGCAGCAGCAGAAC---
AATTTG---CTGAGA---GCTATTGAGGCGCAACAGCATCTGTTGCAACTCACAGTCTGGGGCATC
>FJ388912
TTCTACTGTAATTCAACACAACTGTTTAATAGTACTTGGAATGTTAATAGTACTTGGAATGATACCGAAAGGT
CAAATATC---
------------------ACA---
GAAGAAAATTTCAATATCACATTCCCATTCAGAATAATTCTAATTATAAGCAGGGGACTGGAAATAGCAAAAT
CAGCCCCTCCCCCCACCGGCATAAGTAAAATTTCATGTTCTTCAACG---
AGTACGGGGTTGACAAGAGCAAGAGGTGAAGGTAATGATAACAAC--------------------------
----

GGGACCGAGACCTTCAGACCTGGAGGAGGAGACATGAGGGACAATTGGAGAAGTGAATTATATAAATATAAAG
TAGTAAAAATTGAACCATTA---GGAGTAGCACCCACCAGGGCAAAAAGAAGAGTGGTG------------
------------------------CAAAGAGAAAAAGAGGA---GTGGGA---CTAGGAGCT---------
TTGTTCCTTGGGTTCTTGGGAGCA---GCAGGAAGCACTATG---GGCGCAGCGTCG---
ATAACGCTGACGGTACAG---GCCAGGCAA---TTATTG---TCTGGTATAGTGCAACAACAAAAC---
AATTTG---TTGAGA---GCTATTGAGGCGCAACAGCATCTGTTGCAGCTCACAGTCTGGGGCATC
>FJ388960
TTCTACTGTAATACAACACAACTGTTTACTAGTAAGTGGAAGAATGGTACTAGATTGTCAGAGAACAAAACTA
AC---

ACAGAAGAAAATATCACACTCCCATGCAGAATAAAACAAATTATAAACATGTGGCAGGAAGTAGGAAAAGCAA
TGTATGCCCCTCCCATCAGAGGACAAATTAGATGTTCTTCAAAT---
ATTACTGGGTTGCTATTAACAAGAGATGGGGGTGATACCGGGGAG--------------------------
----

AACGAGACAGAGGTCTTCAGACCTGGAGGAGGAAATATGAGGGACAATTGGAGAGGTGAATTATATAAATATA
AAGTGGTACAAATTGAACCCTTA---GGAGTAGCACCCACCAAGGCAAAGAGAAGAGTGGTG---------
---------------------------CAGAGAGAAAAAGAGCA---GTAATA------GGAGCT------
---ATGTTCCTTGGGTTCTTGGGAGCA---GCAGGAAGCACTATG---GGCGCAGCATCA---
GTGGTGCTGACGGTACAG---GCCAGACAA---TTATTG---TCTGGTATAGTGCAACAGCAAAAC---
AATTTG---CTGAAG---GCTATTGAGGCGCAACAACATCTGTTGCAACTCACAGTCTGGGGCATC
>FJ388914
TTCTACTGTAATACAACACCACTGTTTAATAGTACTTGGAATAGTACTAGCTGGCCAAATAACACAAATAACA
CAAATGAC---
------------------ACT---
AACACAAATGACACTATCACACTCAGATGCAGAATAAAACAAATTATAAACCTGTGGCAGGAAGTAGGAAAAG
CAATGTATGCCCCTCCCATCAGAGGACAAATTAATTGTACATCAAAT---
ATTACCGGGTTGCTATTAACAAGAGATGGGAGTGATGCGAACAGC--------------------------
----

ACGATCGAGGTCTTCAGACCTATAGGAGGAAATATGAAGGACAATTGGAGAAGTGAATTATATAAATATAAAG
TGGTAAAAATTGAACCATTA---GGAATAGCACCCACCACGGCAAGGAGAAGAGTGGTG------------
------------------------CAGAGAGAAAAAGAGCA---GTGGGA---CTAGGAGCT---------
TTGTTCCTTGGGTTCTTGGGAGCA---GCAGGAAGCACTATG---GGCGCAGCATCA---
ATGGGGCTGACGGTACAG---GCCAGACAA---TTATTG---TCTGGTATAGTGCAACAGCAAAAC---
AATTTG---CTGAGG---GCTATTGAGGCGCAACATAATCTGTTGCAACTCACAGTCTGGGGCATC
>FJ388915
TTCTACTGTAACACAACAAAACTGTTTAACAGTACTTGGACTTGGAATAGTGGTACTTGGAATTCAACAAATA
GTAGT--
------------------ACT---
AATGGAATTGACACTATCACTATCACATGCCCAATAAGGCTAAGTGTAAACGCGTGGCAGGAAGTAGGAATAG
CAATGTCTATCTCTGCCCTCAGAGGCCGAATCAAGAGTTTATCAACG---
TTTACAGGTACAGGATTAACAAGAACAAGTGGTGGCGCCAACGAGGAT-----------------------
----

GAGAACAGGAATGAGACCTTCAGACCTGGGGGAGGAAATATGAAGGACAATTGGAGAAGTGAATTATATAAAT

ATAAAGTAGTAGAAATTGAACCTTTA---GGAATAGCACCCACCAAGGCAAAGAGAAGAGTGGTG--------
-------------------------------CAGAGAGAAAAAAGAGCA---GCATTA------GGAGCT---
------TTGTTCCTTGGGTTCTTGGGAGCA---GCAGGAAGCACTATG---GGCGCAGCGTCA---
ATAACGCTGACGGTACAA---GCCAGACTA---TTGTTG---TCTGGTATAGTGCAACAGCAAAGT---
AACTTG---CTGAAA---GCTATTGAGGCGCAACAGCATCTGTTGCAACTCACAGTCTGGGGCATC
>FJ388918
TTCTACTGTAACACAACACAACTGTTCAATAGTACTTGGAATTTAACACAATCGTTTAATAGTAGTACTGAAG
GGTCAAGT---
-------------GGCACT------
GGGTCAAGTGGCACTATCACACTCCCATGCAGAATAAGACAAATTGTACACTTGTGGCAGGAAGTAGGAAAAG
CAATGTATGCCCCTCCCATCAGAGGACTAATCAGGTGTTCATCAAAT---
ATTACAGGGCTGCTGTTAACAAGAGATGGTGGTAATGATGGTAATGAC-------------------------
----

TCCAATACTGAGGTCTTCAGACCTGGGGGAGGAAATATGAAGGACAATTGGAGAAGTGAATTATATAAATATA
AAGTAGTAGAAATTGAACCATTA---GGAGTAGCACCCACCAGGGCAAAGAGAAGAGTGGTG-----------
----------------------------CAGAGAGAAAAAAGAGCA---GTGCTA------GGAGCT------
---TTGCTCCTTGGGTTCTTGGGAGCA---GCAGGAAGCACTATG---GGCGCAGCGTCA---
ATAGCGCTGACGGCACAA---GCCAGACAA---TTGATG---TCTGGTATAGTGCAACAGCAAAAC---
AACTTG---CTGAGA---GCTATTGAGGCGCAACAGCATATGTTGCAACTCACAGTCTGGGGCATC
>FJ388920
TTCTACTGTAGTACAACACCACTGTTTAATAGTACTTGGGATAATAATACTGAATGGTCAAATAACACAAGTA
AAAAT--

AAAAATGACAGTAATATCACACTCCCATGCAGAATAAAACAAATTATAAACCTGTGGCAGGAAGTAGGAAAAG
CAATGTATGCCCCTCCCATCAAAGGACAAATTAACTGTTCATCAAAT---
ATTACTGGACTACTATTAACAAGAGATGGGGGTGGAAACGAGAGC----------------------------
----

AATATCGAGACCTTCAGACCCGCAGGGGGAGATATGAGGGATAATTGGAGAAGTGAATTATATAAATACAAAG
TGGTACAAATTGAACCATTA---GGAGTAGCACCCACCAAGGCAAAGAGAAGAGTGGTG--------------
-------------------------CAGAGAGAAAAAAGAGCA---GTGGGA---CTAGGAGCT---------
GTGCTCTTTGGGTTCTTGGGAGCA---GCAGGAAGCACTATG---GGCGCAGCATCA---
GTGATGCTGACGGTACAG---GCCAGACAA---TTATTG---TCTGGTATAGTGCAGCAGCAAAAC---
AATTTG---CTGAGA---GCTATTGAGGCGCAACAACATCTGTTGCAACTCACAGTCTGGGGCATC
>FJ388923
TTCTACTGTAATACAACACCACTGTTTAATAGTACTTGGAATGATACTAGTCGGCCAAATATCACAGGTGAGG
AAGAT--
-------------------AAT---
GGTGAGGAAGATAATATGATACTCACATGCAGAATAAAGCAAATTATAAACCTGTGGCAGGAAGTAGGAAAAG
CAATGTATGCCCCTCCCATCAGAGGACAAATTAGATGTTCATCAAAT---
ATTACCGGGTTGCTCTTAACAAGAGATGGGGGTAATGACAGCAGC----------------------------
----

AGGACCGAGGTCTTCAGACCTATGGGAGGAAATATGAGGGACAATTGGAGAAGTGAATTATATAAATATAAAG
TGGTGCAAGTTGAACCATTA---GGAGTAGCACCTACCACAGCAAAGAGAAGAGTGGTG--------------
-------------------------CAGAGAGAAAAAAGAGCA---GTGGGA---ATAGGAGCT---------
GTGTTCCTTGGGTTCTTGGGAGCA---GCAGGAAGCACTATG---GGCGCAGCATCA---
GTGATGCTGACGGTACAA---GCCAGACAA---TTATTG---TCTGGTATAGTGCAGCAGCAAAAC---
AATTTG---CTGAGG---GCTATTGAGGCGCAACAACATCTGTTGCAACTCACAGTCTGGGGCATC
>FJ388963
TTCTACTGTAATACAACAAAACTGTTTAATAGTACTTGGAATGTTAGTACTGGGAATGGAAATAAAACTGGAG
AGGGCACAAAT--
-------------AGTAAT------
GGCACAAATAGGAATATCACACTTCCATGCAGAATAAAACAAATTATAAACATGTGGCAGGAAGTAGGAAAAG
CAATGTATGCCCCTCCCATCAAAGGACAAATTAAATGCTCATCAAAT---
ATTACAGGGCTGTTATTACCAAGAGATGGTGGTTTAAATAATAGC----------------------------
----

ACTAATGAGACCTTCAGACCTGGAGGAGGAAATATGAGGGACAATTGGAGAAGTGAATTATATAAATATAAAG

TAGTAAAAATTGAACCATTA---GGAGTAGCACCTTCCAGGGCAAAGAGAAGAGTGGTG--------------
-------------------------CAGAGAGAAAAAAGAGCA---GTAGGG---CTAGGAGCT---------
GTGTTCATTGGGTTCTTAGGAGCA---GCCGGAAGCACTATG---GGCGCAGCGTCA---
ATAACGCTGACGGTACAG---GCCAGACAA---TTATTG---TCTGGTATAGTGCAACAGCAGAGC---
AACCTG---CTGAGG---GCTATTGAGGCGCAACAGCATCTGTTGCAACTCACAGTCTGGGGCATC
>FJ388924
TTCTACTGTAATACAACACCACTGTTTAATAGTACTTGGGGTAATGGTACTGAATGGTCAAATAACACAAATA
ACCCT--
-------------------AAT---
AACCCTGACAGAGATATCACACTCACATGCAGAATAAAACAAATTATAAACCTGTGGCAGGAAGTAGGAAAAG
CAATGTATGCCCCTCCCATCAGAGGACAAATTAGATGTTCATCAAAT---
ATTACTGGGTTACTATTAACAAGAGATGGGGGTAAAAACGAGAGC----------------------------
----

GAGGCCACCGAGGTCTTCAGACCTGCAGGGGGAGATATGAGGGACAATTGGAGAAGTGAATTATATAAATATA
AAGTGGTACAAATTGAACCATTA---GGAGTAGCACCCACCAAGGCAAAGAGAAGAGTGGTG-----------
----------------------------CAAAGAGAAAAAAGAGCA---GTGGGA---CTAGGAGCT------
---ATGCTCTTTGGGTTCTTGGGAGCA---GCAGGAAGCACTATG---GGCGCAGCATCA---
GTGATGCTGACGGTACAG---GCCAGACAA---TTATTG---TCTGGTATAGTGCAACAGCAAAAC---
AATTTG---CTGAGG---GCTATTGAGGCGCAACAACATCTGTTGCAACTCACAGTCTGGGGCATC
>FJ388964
TTCTACTGTGACACAAAACCACTGTTTAATAGTACCTGGAATAGTAATAGTACTTGGAATGTTACAAATGACA
CT---

GACACTGCAGAAAATATCACACTCCAATGCAAGATAAAACAAATTATAAACTTGTGGCAGAGAGTAGGAAGAG
CAATGTATGCCCCTCCCATCAGAGGACAAATTAGATGTTCATCAAAT---
ATTACAGGGCTGCTACTAACAAGAGATGGTGGTAAGGAAAATAACACG-------------------------
----

AACTCTACTGAGATCTTCAGACCTGGAGGAGGAGACATGAGGGACAATTGGAGAAGTGAATTATATAAATATA
AAGTAGTAAAACTTGAACCATTA---GGAGTAGCACCTTCCAAGGCAAAGAGAAGAGTGGTG-----------
----------------------------CAGAGAGAAAAAAGAGCA---GTGGGA---CTAGGAGCT------
---ATGTTCCTTGGGTTCTTGGGAGCA---GCAGGAAGCACTATG---GGCGCAGCGTCA---
ATGACGCTGACGGTACAG---GCCAGGCAA---TTACTG---TCTGGTATAGTGCAGCAGCAGAGT---
AATTTG---CTGCGG---GCTATTGAGGCGCAACAGCATCTGCTGCAACTCACAGTTTGGGGCATC
>FJ388927
TTCTACTGTAATTCAACAGAACTGTTTAATAGTATTTGGTATGCAAATGGTACTAGGAATAGTACTGAAGGGG
CACATAAC---
-------------------GAA---
TCACATAACGAAACTATCACGCTCCCATGCAGAATAAAACAAATTATAAATATGTGGCAAGAAGTAGGAAAAG
CAATGTGTGCCCCTCCCATCAGAGGACAGATTAGGTGTTCATCAAAT---
ATTACAGGGCTACTATTAACAAGAGATGGTGGTAACGAGAACAAC----------------------------
----

ACCAACACAGAGACCTTCAGACCGGGAGGAGGAGATATGAGGGACAATTGGAGAAGTGAATTATATAAATATA
AAGTAGTAAAAATTGAACCGTTA---GGAGTAGCACCTACCAAGGCAAAGAGAAGAGTGGTG-----------
----------------------------CAAAGAGAAAAAAGAGCA---ATAGGA---ATGGGAGCT------
---ATGTTCCTTGGGTTCTTGGGAGCG---GCAGGAAGCACTATG---GGCGCAGCGTCA---
CTGACGCTGACGGTACAG---GTCAGACAA---TTATTG---TCTGGTATAGTGCAACAGCAGAAC---
AATCTG---CTGATG---GCTATTGAGGCGCAACAACATCTGTTGCAACTCACAGTCTGGGGCATC
>FJ388933
TTCTACTGTAATACAACAAAATTGTTTAATAGTACTTGGAATGGTACTGAAGGGTCAAGTAACGGTACTGACA
GTGAAAATAAC--
-------------ATCAAT------
AATAACAACACTGAAATCGTACTCCCATGCAAAATAAAACAAATTATAAACATGTGGCAGGAAGTAGGAAAAG
CAATGTATGCCCCTCCCATCCAAGGACAAATTAATTGTACATCAAAT---
ATTACAGGGCTGCTACTAACAAGAGATGGTGGTAATAGTAGCGGG----------------------------
----

ACTAATGAGACCTTCAGACCTGGAGGAGGAGATATGAGGGACAATTGGAGAAGTGAATTATATAAATATAAAG

TAGTAAGAATTGAACCATTA---GGAGTAGCACCCACTAGGGCAAAGAGAAGAGTGGTG--------------
-------------------------CAGAGAGAAAAAAGAGCA---GCGATA------GGAGCT---------
ATGTTCCTTGGGTTCTTGGGAGCA---GCAGGAAGCACTATG---GGCGCAGCGTCA---
ATAACGCTGACGGTACAG---GCCAGACTA---TTATTG---TCTGGTATAGTGCAACAGCAGAAC---
AATCTG---CTGAGG---GCTATTGAGGCGCAACAGCATCTGTTGCAACTCACAGTTTGGGGCATT
>FJ388935
TTTTACTGTAATACATCACAACTGTTTAATAGTAGTTGGGATGAGAATGCTAATGCTGGGAATAATACTCCTG
GGAATGAT---
-------------------ACT---
CCTGGGAATGATACTATCACGCTTCCATGCAGAATAAAACAAATTATAAACATGTGGCAGGAAGTAGGAAAAG
CAATGTATGCCCCTCCCATCAGAGGACAAATTAAATGTGTATCAAAT---
ATTACAGGGCTGTTATTGACAAGAGATGGTGGTAAGAACAACAGTAAT-------------------------
----

GATAACAATACTGAGATCTTCAGACCTGAAGGAGGAAATATGAAGGATAATTGGAGAAGTGAATTATATAAAT
ATAAAGTAGTGAAGATTGAACCATTA---GGAATAGCACCCACCAAGGCAAAGAGAAGAGTGGTG--------
-------------------------------CAAAGAGAAAAAAGAGCA---GTGGGAACAATAGGAGCT---
------ATGTTCCTAGGGTTCTTGGGAGCA---GCAGGAAGCACTATG---GGCGCAGCGTCA---
ATGACGCTGACGGTACAG---GCTAGACAA---TTATTG---TCTGGTATAGTGCAACAGCAAAAC---
AACTTG---CTGAAG---GCTATTGATGCGCAACAGCATATGTTGCAGCTCACAGTTTGGGGCATC
>FJ388936
TTCTACTGTAATACAACGCCACTGTTTAATAGTACTTGGAATTGGAATAATACTGGAGAGCCAGAAACCACAA
ATGAC--
-------------------ACT---
GACACTAATAAGAATATCACACTCAAATGCAGAATAAAACAAATTATAAACTTGTGGCAGGAAGTAGGAAAAG
CAATGTATGCCCCTCCCATCAGAGGACAAATTAGATGTTCATCAAAC---
ATTACTGGGTTACTATTAACAAGAGATGGGGGTAGTACCAAGAACGGGACC----------------------
----

CAGAACGAGACTGAGGTCTTCAGACCTGAAGGAGGAAATATGAAGGACAATTGGAGAAGTGAATTATATAAAT
ATAAAGTGGTACAAATTGAACCATTA---GGAATAGCACCCACCAAGGCAAAGAGAAGAGTGGTG--------
-------------------------------CAGAGAGAAAAAAGAGCA---GTGGGA---ATAGGAGCT---
------ATGTTCCTTGGGTTCTTGGGAGCA---GCAGGAAGCACTATG---GGCGCAGCGGCA---
GTGACGCTGACGGTACAG---GCCAGACAA---TTGTTG---TCTGGTATAGTGCAACAGCAAAAC---
AATTTG---CTGAGA---GCTATTGAGGCGCAACAACATATGTTGCAACTCACAGTCTGGGGCATC
>FJ388937
TTCTACTGTAATTCATCACAACTGTTTAATAGTACTTGGAATAGTACTAAATGGTTAAATAGCACTGAAGGAG
AAAACATC---
-------------------ACA---
ACTGAAGGAGAAAACATCACACTCCCATGCAGAATAAAACAAATTATAAACATGTGGCAGACAGTAGGAAAAG
CAATGTATGCCCCTCCCATCAGTGGACTAATTAGATGTTCATCAAAT---
ATTACAGGGCTACTATTAGTAAGGGATGGTGGTAATACCACTAGTAGC-------------------------
----

AATTCCACCGAGACCTTCAGACCGGCAGGTGGAGATATGAGGGACAATTGGAGAAGTGAATTGTATAAATACA
AAGTAGTAAAAATTGAACCAATA---GGAATAGCACCCACCAGGGCAAGGAGAAGAATGGTG-----------
----------------------------CAGAGAGAAAAAAGAGCA---GCAATAGGAGTAGGAGCT------
---GTGTTCCTTGGGTTCTTGAGTGCA---GCAGGAAGCACTATG---GGCGCAGCGTCA---
CTGACGCTGACGGTACAG---GCCAGGCAG---TTATTG---TCTGGTATAGTGCAACAGCAGAAC---
AACTTG---CTGAGG---GCTATTGAGGCGCAACAGCATCTGTTGCAACTCACAGTTTGGGGCATC
>FJ388939
TTCTACTGTAATACAACACAACTGTTTAATAGTATTTGGAATCGGGATGATATTTGGAATACTACTGGGGGGA
ATGGT--
-------------------ACT---
GAGTCAAATAACAATATTACTCTCCCATGCAGAATAAGACAAATTGTAAATAGGTGGCAGGAAGTAGGAAAAG
CAATGTATGCCCCTCCCATCGCAGGACACATTAGTTGTTTATCAAAT---
ATTACAGGGCTACTATTAACAAGAGATGGTGGTGTTAATAAAAGT----------------------------
----

GCCATCGAGACCTTCAGACCATTAGGAGGAAATATAAAGGATAATTGGAGAAGTGAATTATATAAATATAAAG

TAGTAAAAATTGAACCATTA---GGGGTAGCACCCACCAAGGCAAAGAGAAGAGTGGTG-------------
-------------------------CAGAGAGAAAAAAGAGCG---GCACTA------GGAGCG---------
CTGTTACTCGGGTTCTTGGGAGCA---GCAGGAAGCACTATG---GGCGCAGCGTCA---
CTGACGCTGACGGTACAG---GCCAGACAA---TTATTG---TCTGGTATAGTGCAACAGCAGAAC---
AACTTG---CTGAGA---GCTATTGAGGCGCAACAACATCTGTTGCAGCTCACAGTCTGGGGCATT
>FJ388955
TTCTACTGTAATACATCACAACTGTTCAATAGTACTTGGAACACCACTGAGCTTGAGTCAAATATCACAGATG
AAAAT--

ATCACAGATGAAAATATCACACTCCCATGCAGAATAAAACAAATCATAAATATGTGGCAGGAAGTAGGAAAAG
CAATGTACGCCCCTCCCATTAAAGGACAAATTAGATGTTCATCAAAT---
ATTACAGGGCTGCTATTAACAAGAGATGGTGGTAGTAATACCACA----------------------------
----

AATGGAACCGAGATTTTCAGACCTGGAGGAGGAGATATGAGGGACAATTGGAGAAGTGAATTATATAAATACA
AAGTAGTAAAAATTGAACCATTA---GGAATAGCACCCACCAAGGCAAAGAGAAGAGTGGTG-----------
----------------------------CAGAGAGAAAAAAGAGCA---GCAATA------GGAGCT------
---TTGTTCCTTGGGTTCTTGGGAGCA---GCAGGAAGCACTATG---GGCGCAGCGTCA---
ATGACGCTGACGGTACAG---GCCAGACTA---TTATTG---TCTGGTATAGTGCAACAGCAAAAC---
AATTTG---CTGAAA---GCTATTGAGGCACAACAGCATCTGTTGCAACTCACAGTCTGGGGCATT
>JF683736
TTCTACTGTAATTCAACTGCACTGTTTAATAGTACTTGGAATAATACTGCAGGGTCAAATAACACTACAGGAA
ATAAC--
-------------------ACT---
ACAGGAAATGACACAATCACACTCCAATGCAGAATAAAACAAATTGTAAACATGTGGCAGGAAGTAGGAAGAG
CAATGTATGCCCCTCCCATCAGAGGACCAATTAGATGTTCATCAAAT---
ATTACAGGGATACTATTAACAAGAGATGGTGGTAATAATAATAGCACAAAC----------------------
----

AAGTCTGAGTCTGAGACCTTCAGACCTGGAGGAGGAAATATGAAGGACAATTGGAGAAGTGAATTATATAAAT
ATAAAGTAGTACAAATTGAACCATTA---GGATTAGCACCCACCAAGGCAAAGAGAAGAGTGGTG--------
-------------------------------CAGAGAGAAAAAAGAGCA---ATAGGA---CTAGGAGCT---
------ATGTTCCTTGGGTTCTTGGGAGCA---GCAGGAAGCACTATG---GGCGCAGCATCA---
GTAACGCTGACGGTACAG---GCCAGACAA---TTATTG---TCTGGTATAGTGCAGCAGCAGAAT---
AATCTG---CTGAGA---GCTATTGAGGCGCAACAGCACATGTTGCAACTCACAGTCTGGGGCATT
>JF683741
TTCTACTGTAATTCAGCGGGACTGTTTAATAGTACTTGGGGGACTACTAATGGTACTTGGAAGGGGACTGAAG
GGTCA--

GCTAACAATAACACTCTCATACTCCCATGCAGAATAAAACAAATTATAAACATGTGGCAGAGAGTAGGAAAAG
CAATGTATGCCCCTCCTATTAGAGGAAATATTAGCTGTTCATCAAAT---
ATTACAGGGTTACTGTTGACAAGAGATGGTGGTAATAATAATAAT----------------------------
----

AGTGGGACGGAGACTTTCAGACCTGGAGGAGGAGATATGAGAGACAATTGGAGAAGTGAATTATATAAATATA
AAGTAGTAAAAATTGAACCATTA---GGAGTAGCACCCACTAAGGCAAAGAGAAGAGTGGTG-----------
----------------------------CAGAGAGAAAAAAGAGCA---GTGGGA---TTAGGAGCT------
---TTGTTCCTTGGGTTCTTGGGAGCA---GCAGGAAGCACTATG---GGCGCAGCGTCA---
GTGACGCTGACGGTACAG---GCCAGACTA---TTATTG---TCTGGTATAGTGCAACAGCAGAAC---
AATTTG---CTGATG---GCTATTGAGGCGCAACAGCATCTGTTGCAACTCACAGTCTGGGGCATC
>JF683743
TTCTACTGTAATACAACACCATTGTTTAGTAGTATTTGGAATGGTACTAAGAGGGTAAATGGAACTAACGAAA
CT---

GAAACTGGAGACAATATCACACTCACATGCAGAATAAAACAAATTATAAACATGTGGCAGGAAGTAGGAAAAG
CAATGTATGCCCCTCCCATCGCAGGACAAATTAACTGTTCATCAAAT---
ATTACAGGGCTGTTACTAACAAGAGATGGTGGTGTTAGTAATAACACT-------------------------
----

ACAGGGAATGAAACCTTCAGACCTGGAGGAGGAAATATGAAGGACAATTGGAGAAGTGAATTATATAAATATA

AAGTAGTAAGAATTGAACCATTA---GGAATAGCACCCACTAAGGCAAAGAGAAGAGTGGTG-----------
----------------------------CAGAGAGAAAAAAGAGCA---GCA------TTTGGAGCC------
---TTGTTCCTTGGGTTCTTGGGAGCA---GCAGGAAGCACTATG---GGCGCAGCGTCA---
GTAACGCTGACGGTACAG---GCCAGACAA---TTATTG---TCTGGTATAGTGCAACAGCAGAAC---
AATTTG---CTGAGA---GCTATTGAGGCGCAACAGCATCTGTTGCAACTCACAGTCTGGGGCATC
>JF683747
TTCTACTGTAATACATCACAACTGTTTAATAGTACTTGGAATAGTACTTTGGGGGTGAATAGTACTTTGGGGG
TTAATAAT--
-------------GGTACT------
GACAATGACACTATCATCTCACTCCCATGTAGAATAAAACAAATTATAAACATGTGGCAGGAAGTAGGAAAAG
CAATGTATGCCCCTCCCATCAGAGGACTAATTAGATGTTCATCAAAT---
ATTACAGGGCTGATATTAACAAGAGATGGTGGGAGTAGCAAGAATAAC-------------------------
----

AGCACGAATGAGACTTTCAGACCTGGAGGAGGAGATATGAGGGACAATTGGAGAAGTGAATTATATAAATATA
AAGTAGTAAAAATTGAACCATTA---GGAATAGCACCCACCCAGGCAAAGAGAAGAGTGGTG-----------
----------------------------CAGAGAGAAAAAAGAGCA---GTGGGG---ATAGGAGCT------
---GTGTTCCTTGGGTTCTTGGGAGCA---GCAGGAAGCACTATG---GGCGCAGCGTCT---
GTGACGCTGACGGTACAG---GCCAGACTA---TTATTG---TCTGGTATAGTGCAACAGCAGAAC---
AATCTG---CTGAAG---GCTATTGAGGCGCAACAACATCTGTTGCAACTCACAGTCTGGGGCATT
>JF683751
TTCTACTGTAATTCAACACAACTGTTTAATAGTACTTGGACTTGGAATGGTACTGAAGGGTCAAATAACACTG
AA---

GGAAATGGCACAATCACACTCCCATGCAAAATAAAACAAATTGTAAACATGTGGCAGGAAGTAGGAAAAGCAA
TGTATGCCCCTCCCATCAGGGGACAAATTAAATGTTCATCAAAT---
ATTACAGGGCTGCTATTAACAAGAGATGGGGGTAATAGCACCATC----------------------------
----

AACGAGACCTTCAGACCTGGAGGAGGAGATATGAGGGATAATTGGAGAAGTGAGTTATATAAATATAAAGTAG
TAAAAATTGAACCATTA---GGGGTAGCACCCACCAAGGCAAAGAGAAGAGTGGTG-----------------
----------------------CAGAGAGAAAAAAGAGTG---GCAGGA---CTAGGAGCT---------
ATGTTCCTTGGGTTCTTGGGAGCA---GCAGGAAGCACTATG---GGCGCAGCGTCA---
ATAACGCTGACGGTACAG---GCCAGACAG---TTATTG---TCTGGTATAGTGCAACAGCAGAAC---
AATCTG---CTGAGG---GCTATTGAGGCGCAACAGCATCTGTTGCAACTCACAGTCTGGGGCATC
>JF683753
TTCTACTGTAATACAACAAAACTATTTAATAGTGCTTGGATTAATGGTACTTGGAATGGTACTGAAGGGTCA-
----

AATAAATCAACCACCATCACACTCCCATGCAGAATAAAACAAGTTATAAACCTGTGGCAGGAAGTAGGAAAAG
CAATGTATGCCCCTCCCATCAGGGGATACATTAATTGTTCATCAAAT---
ATTACAGGACTGCTATTAACAAGGGATGGTGGTAATAAT----------------------------------
----

GAGACCGAGACCTTCAGACCTGGAGGAGGAAATATGAAGGACAATTGGAGAAGTGAATTATATAAATATAAAG
TAGTAAAAATTGAACCATTA---GGAATAGCACCCACCAAGGCAAGGAGACGAGTGGTG--------------
-------------------------CAGAGAGAAAAAAGAGCA---GTCACA---CTAGGAGCT---------
ATGTTCCTTGGGTTCTTGGGGGCA---GCAGGAAGCACTATG---GGCGCAGCGTCA---
CTGACGCTGACGGTACAA---GCCAGACTA---TTATTG---TCTGGTATAGTGCAGCAGCAAAAC---
AATTTG---CTGAGG---GCTATTGGGGCGCAACAACATCTGTTGCAACTCACAGTCTGGGGCATC
>JF683754
TTCTACTGTAATACAACACCACTATTTAATAGTACCTGGAATGATACTAGCTGGTCAGTGGCCACTAAG----
----

AATGATACAAATATCACACTCGCATGCAGAATAAAACAAATTATAAACCTGGGGCAGGAAGTAGGAAGAGCAA
TGTATGCCCCTCCCATCAGAGGACTAATTAGATGTTCATCAAAT---
ATTACAGGGTTGCTATTAACAAGAGATGGGGGTAGCAACGAGAAC----------------------------
----

AATGGAACTGAGGTCTTCAGACCTGCAGGAGGAAATATGAAGGACAATTGGAGAAGTGAATTATACAAATATA

```
AAGTGGTACAAATTGAACCATTA---GGAGTAGCACCCACCAGGGCACGGAGAAGAGTGGTG-----------
----------------------------CAAAGAGGAAAAAGAGCA---GTGGGA---ATAGGAGCT------
---GTTCTCCTTGGGTTCTTGGGAGCA---GCAGGAAGCACTATG---GGGGCAGCTTCA---
ATTGGGCTGACGGTACAG---GCCAGACAA---TTATTG---TCTGGTATAGTGCAGCAGCAAAGC---
AATTTG---CTGAGA---GCTATTGAGGCGCAACAACATCTGTTGCAACTCACAGTCTGGGGCATC
>JF683769
TTCTACTGTAATACAACATCACTGTTTAATAGTACTTGGAATGAAACACAATTGCATAATAGCACTTGGAATG
AG-----------------------------------------------------------------------
-------------------------
ACTAGAAATGACAATATCACACTCCCATGCAAAATAAAACAAATTATAAACATGTGGCAGGAAGTAGGAAAAG
CAATGTATGCCCCTCCCATCAGAGGACTAATTAAATGTACATCAAAT---
ATTACAGGAATGTTACTGACAAGAGATGGGGGTAGTAACCCTAACAAT-------------------------
-------------------------------------------------------------------------
-------
ACTAACAATACTGAGATTTTCAGACCTGGAGGAGGAGATATGAGGGACAATTGGAGAAGTGAATTATATAAAT
ATAAAGTAGTACAAATTGAACCATTA---GGAATAGCACCCACCAAGGCAAAGAGAAGAGTGGTG--------
-------------------------------CAGAGAGAAAAAAGAGCA---CTGGGA---ATAGGAGCT---
------ATGTTCCTTGGGTTCTTGGGAGCA---GCAGGAAGCACTATG---GGCGCAGCGTCA---
CTGACGCTGACGGTACAG---GCCAGGCAA---TTGTTG---TCTGGTATAGTGCAACAGCAAAAC---
AATTTG---CTGAGA---GCTGTTGAGGCGCAACAGCATCTGTTGCAACTCACAGTCTGGGGCATT
>JF683775
TTCTACTGTAATACAACACAACTATTTAATAGTAATTGGACAGTAGTTAATAGTACTTGGTCAGCTAATAGTA
CTTGGAATAGT--------------------------------------------------------------
-------------ACTACA------
GGGTCAAATAACACTATCACACTCCAATGCAGAATAAAACAAATTATAAACATGTGGCAGGAAGTAGGAAAAG
CAATGTATGCCCCTCCCATCAGAGGAAATATTAGTTGTTCATCAAAT---
ATTACAGGGCTGCTCCTAGTAAGAGGAGGT---AATGAATCAAAA----------------------------
-------------------------------------------------------------------------
-------------
TCACAAGAGATCTTCAGACCTATAGGAGGAAACATGAAGGACAATTGGAGAAGTGAATTATACAAATATAAAG
TAGTAAAAATTGAGCCATTA---GGAGTAGCACCTACCAAGGCAAAGAGAAGAGTGGTG--------------
-------------------------CAAAGAGAAAAAAGAGCT---GTGGGA---CTAGGAGCT---------
TTGTTCCTTGGGTTCTTGGGAGCA---GCAGGAAGCACTATG---GGCGCAGCGTCA---
ATAACGCTGACGGTACAG---GCCAGACAA---TTATTG---TCTGGTATAGTGCAACAGCAGAAC---
AACCTG---CTGAGA---GCTATTGAGGCGCAACAGCATATGTTGCAACTCACAGTCTGGGGCATT
>JF683778
TTCTACTGTAATACAACACAACTGTTTAATAGTACTTGGATTTTTACTGGGAATACCACACAACTGGTTAATG
GG-----------------------------------------------------------------------
-------------------------
AGTGTGAATGAGAATATCACACTCCCATGCAAAATAAAACAAATTGTAAACATGTGGCAGAAAGTAGGAAAAG
CAATGTATGCCCCTCCAATCAGAGGACCAATTAGCTGTACATCAAAT---
ATTACAGGGCTGCTATTAATAAGAGATGGTGGTGTTGGCAATGAGACC-------------------------
-------------------------------------------------------------------------
----------
AATAGCACCGAGATCTTTAGGCCTGGAGGAGGAGATATGAGGGACAATTGGAGAAGTGAATTATATAAATATA
AAGTAGTAAGAATTGAGCCAATA---GGAGTAGCACCCACTAAGGCAAAGAGAAGAGTGGTG-----------
----------------------------CAGAGAGAAAAAAGAGCA---GTGGGA---CTAGGAGCT------
---GTGCTCTTTGGGTTCTTGGGAGCA---GCAGGAAGCACTATG---GGCGCAGCGTCA---
GTGACGCTGACGGTACAG---GCCAGACAA---TTATTG---TCTGGTATAGTGCAACAGCAGAAC---
AATTTG---CTGAAG---GCTATTGAGGCGCAACAGCATTTGTTGCAACTCACAGTCTGGGGCATC
>JF683785
TTCTACTGTAATTCATCACAACTGTTTGATAGGTCTTGGCATACTAGTGGTACTCGGAAGGATAATAATACAA
GT-----------------------------------------------------------------------
----------------------------
ATAAATGGAACTATCATACTCCCATGTAGAATAAAACAAATTATACATATGTGGCAGGAAGTAGGGAAAGCAA
TGTACGCCCCTCCCATCAGAGGAGCAATTAACTGTACATCAAAT---
ATTACAGGGCTGCTATTAACAAGAGATGGTGGTAATGGCAGTAAT----------------------------
-------------------------------------------------------------------------
----------
AATGAAACTGAAACTTTCAGACCTATAGGAGGGAATATGAAGGACAATTGGAGAAGTGAATTATATAAATATA
```

AAGTGGTAAAAATTGAACCATTA---GGAGTAGCACCCACCAAGGCAAAGAGAAGAGTGGTG-----------
----------------------------CAAAGAGAAAAAAGAGCA---GTGGGAGTAATAGGAGCT------
---ATGTTCCTCGGGTTCTTGGGAGCA---GCAGGAAGCACTATG---GGCGCAGCGTCA---
CTGACGCTGACGGTACAG---GCCAGACAA---TTATTG---TCTGGTATAGTGCAGCAGCAGAAC---
AATCTG---CTGAGG---GCTATTGAGGCGCAACAGCATCTGTTGCAACTCACAGTCTGGGGCATC
>JF683787
TTCTACTGTAATACAACACAATTGTTTAATAGTACTTGGAATACAACAGGGAATACAACAGAATGGGGTAATA
GTACTGAA---
-------------AGTAAT------
AGTACTGAAGAAACTATCACACTCCCATGCAGAATAAAACAAATTATAAACAGATGGCAGGAAGTAGGAAAAG
CAATGTATGCCCCTCCCATCAGCGGAAAAATTAAATGCTCATCAAAT---
ATTACAGGGTTACTATTAACAAGAGATGGGGGTGATAATAACGAGACC-------------------------
----

AACGGAACTGAGGTCTTCAGACCTGGAGGAGGAGATATGAGGGACAATTGGAGAAGTGAATTATATAAATATA
AAGTAGTAAAAATTGAACCATTA---GGAGTAGCACCCACCCAGGCAAGGAGAAGAGTGGTG-----------
----------------------------CAGAGAGAAAAAAGAGGA---GTAGGA---CTAGGAGCT------
---TTGTTCCTTGGGTTCTTGGGAGCA---GCAGGAAGCACTATG---GGCGCAGCGTCA---
ATGACGCTGACGGTACAG---GCCAGACAA---CTATTG---TCTGGTATAGTGCAACAGCAAAAC---
AATTTG---CTGAGA---GCTATTGAGGCGCAACAGCGTCTGTTGCAACTCACAGTCTGGGGCATC
>JF683788
TTCTACTGTAATACAACACAGCTGTTTAATAGTACCTGGTATAATATTGATAATGCTACAGGGACAGAT----
----

GGTAATGAGACTATCACACTCCCATGCAGAATAAAACAAATTATAAACAGATGGCAGGAAGTAGGAAAAGCAA
TGTATGCTCCTCCCATCAGTGGACAAATTAATTGTTCTTCAAAT---
ATTACAGGGTTACTATTAACAAGAGATGGTGGTAATAGTAATAACAGT-------------------------
----

AATGAGAGTGAGACCTTCAGACCTGGAGGAGGAGATATGAAGGACAATTGGAGAAGTGAATTATATAAATATA
AAGTAGTAAGAATTGAACCATTA---GGAGTAGCACCCACCAAGGCAAGGAGAAGAGTGGTG-----------
----------------------------CAGAGAGAAAAAAGAGCA---GTGGGAACGCTAGGAGCT------
---ATGTTCCTTGGGTTCTTGGGAGCA---GCAGGAAGCACTATG---GGCGCAGCATCA---
ATAACGCTGACGGTACAG---GCCAGACAA---TTATTG---TCTGGTATAGTGCAACAGCAGAAC---
AATTTG---CTGAGA---GCTATTGAGGCGCAACAGCATCTGTTGCAACTCACAGTCTGGGGCATC
>JF683790
TTCTACTGTAATACAACAAAACTGTTTAATAGTACTTGGCCTTGGAATAGTACTGATACTGGGGGTGAA----
----

ACTAATGAAAATATCACACTCCCTTGCAGAATAAGACAAATTGTAAACAGGTGGCAGGAAGTAGGAAAGGCAA
TGTATGCCCCTCCCATCAGCGGAGTAATTAATTGTCTATCAAAT---
ATTACAGGGATACTATTAACAAGAGATGGTGGTAATAATAATGAGACT-------------------------
----

AACAACACTGAGATCTTCAGACCTGGAGGAGGAGATATGAGGGACAATTGGAGAAGTGAATTATATAAATACA
AAGTAGTAAGAATTGAACCATTA---GGAATAGCACCCACCAAGGCAAAGAGAAGAGTGGTG-----------
----------------------------CAGAGAGAAAGAAGAGCA---GTGGGA---TTTGGAGCT------
---TTTTTCCTTGGGTTCTTGGGAGCA---GCAGGAAGCACTATG---GGCGCAGCGTCA---
GTAACGCTGACGGTACAG---GCCAGACAA---TTATTG---TCTGGTATAGTGCAACAGCAGAAC---
AACTTG---CTGAGA---GCTATTGAGGCGCAACAACATCTGTTGCAACTCACAGTCTGGGGCATC
>JF683794
TTCTACTGTGATACATCAGAACTGTTTAATAGTACTTGGAATAATACTAAAAGGGTAAATAACACGAATAAC-
----

ACAAATGAAAATATCACACTTCCATGCAGAATAAAACAAATTATAAACATGTGGCAGGGAGTAGGAAAAGCAA
TGTATGCCCCTCCCATCAGTGGACCAATTAAATGTACATCAAAT---
ATTACAGGGCTGTTATTGACTAGAGATGGTGGTAAGAATAACACG----------------------------
----

AATGGGACTGAGATCTTCAGACCAGGAGGAGGAGATATGAGGGACAATTGGAGAAGTGAATTATATAAATATA

AAGTAGTACAAATTGAACCATTA---GGAATAGCACCCACCAAGGCAAAAAGAAGAGTGGTG-----------
----------------------------CAAAGAGAAAAAAGAGAA---CTAACG---ATGGGAGCT------
---CTGTTCCTTGGGTTCTTGAGCGCA---GCAGGAAGCACTATG---GGCGCAGCGTCA---
ATAACGCTGACGGTACAG---GCCAGACAA---TTATTG---TCTGGTATAGTGCAACAGCAGAAC---
AATCTG---CTGAGA---GCTATTGAGGCGCAACAGCATATGTTGCAACTCACAGTCTGGGGCATC
>JF683801
TTCTACTGTAAGACATCACAACTGTTTAATAGTACTTGGCAGTCTAATGATACTTGGCTGCCTAATGGTAACG
AG---

AGTGATGCCAAGATCACACTCCCATGCAGAATAAAACAAATTATAAATAGGTGGCAGGAAGTAGGAAAAGCAA
TGTATGCCCCTCCCATAGCAGGACAAATTAGCTGTTCATCAAAT---
ATTACAGGGCTGCTATTAACAAGAGATGGTGGCAACAACAATGAC----------------------------
----

AATAACAGCGAGATCTTCAGGCCTGCAGGAGGAGATATGAGGGACAATTGGAGAAGTGAATTATATAAATATA
AAGTAGTAAAAATTGAGCCATTA---GGAGTAGCACCCACCAAGGCAAAGAGAAGAGTGGTG-----------
----------------------------CAAAGAGAAAAAAGAGCA---GTGGGAGCAATAGGAGCT------
---ATGTTCCTTGGGTTCTTGGGAACA---GCAGGAAGCACTATG---GGCGCAGCGTCA---
CTAACGCTGACGGTACAG---GCCAGACAA---TTATTG---TCTGGTATAGTGCAACAGCAGAAC---
AATCTG---CTGAGG---GCTATTGAGGCGCAACAGCATCTGTTGCAACTCACAGTCTGGGGCATC
>JF683804
TTCTACTGTAATTCAACACGACTATTTAATAGTACTTGGTATAATAATGGTACTTGGAAAGGTACTGAAGGGA
TAAATAAC---
-------------------ACC---
GGGATAAATAACACCATCATACTCCCATGCAGAATAAAACAAATTATAAACATGTGGCAGGAAGTAGGAAAAG
CAATGTATGCCCCTCCCATCGAAGGACAAATTAAATGTACATCAAAT---
ATTACAGGGCTGTTATTAACAAGAGATGGTGGTACTAACGGGACGACCGATGGT------
ACTAACGGGACGACCGAT---GGTACTAAC---

GGGACCGAGATCTTCAGACCTATAGGAGGAAATATGAGGGACAATTGGAGAAGTAAATTATATAAATATAAAG
TAATAAAAATTGAACCATTA---GGAGTAGCACCCACCAAGGCAAAGAGAAGAGTGGTG--------------
-------------------------CAGAGAGAAAAAAGAGGA---GCA------CTAGAAGTT---------
TTTTTCCTTGGGTTCTTGGGAGCA---GCAGGAAGCACTATG---GGCGCAGCGTCA---
ATGGCGCTGACGGTACAG---GCCAGACTA---TTGTTG---TCTGGTATAGTGCAGCAGCAGAAC---
AATTTG---CTGAGA---GCCATTGAGGCGCAACAGCATATGTTGCAACTCACAGTCTGGGGCATC
>JQ403048
TTCTACTGTAATACAGCACAACTGTTCAACAGTACGTGGACAAATGATACTGGGGAAGTGAATGATACTAAAG
AG---

TCAAATGACACTATCACACTCCCATGCAGGATAAAACAATTCATAAATATGTGGCAGGAAGTAGGAAAAGCAA
TGTATGCCCCTCCCATCCAAGGATCAATTAGGTGTTTATCAAAT---
ATTACAGGGCTGCTACTAACAAGAGATGGTGGTAATGGTAATAGGTCC-------------------------
----

GATAATGAGACCTTCAGACCTGGAGGAGGAGATATGAGGGACAATTGGAAAAGTGAATTATATAAATATAAAG
TAGTAAAAATTGAACCATTA---GGAGTAGCACCCACCAAGGCAAAGAGAAGAGTGGTG--------------
-------------------------CAGAGAGAAAAAAGAGCA---GTGGGA---GTAGGAGCT---------
TTGTTCCTTGGGTTCTTGGGAGCA---GCAGGAAGCACTATG---GGCGCAGCATCA---
GTGATGCTGACGGTACAG---GCCAGACAA---TTATTG---TCTGGTATAGTGCAACAGCAGAAC---
AATTTG---CTGAGG---GCTATTGAGGCGCAACAGCATATGTTGCAACTCACAGTCTGGGGCATC
>KT124812
TTTTACTGTAATACAACAAACCTGTTTAATAGTACTTGGTCTAAAAATGATACTAATGTGACAAACAAAACT-
----

GATAATGACATTATAACACTCCCATGCAGAATAAAACAAATTATAAACATGTGGCAGGAAGTAGGAAAAGCAA
TGTATGCCCCTCCCATCAGGGGACAAATTAGATGTTCCTCAAAT---
ATTACAGGGTTGCTATTAACAAGAGATGGTGGCAATAATAGCACC----------------------------
----

AATGGGTCCGAGATCTTCAGACCTGGAGGAGGAGATATGAGGGACAATTGGAGAAGTGAACTATATAAATATA

AAGTAGTAAAAATTGAACCATTA---GGAATAGCACCCACCAGGGCAAAGAGAAGAGTGGTG-----------
----------------------------CAGAGAGAGAAAAGAGCA---GTGGGA---ATAGGAGCG------
---TTGTTCCTTGGGTTCTTGGGAGCA---GCAGGAAGCACTATG---GGCGCAGCGTCA---
ATGACGCTGACGGTACAG---GCCAGACTA---TTACTG---TCTGGTATAGTGCAACAGCAGAAC---
AATTTG---CTGAGA---GCTATTGAGGCGCAACAGCATATGTTGCAACTTACAGTCTGGGGCATC
>KT124752
TTCTACTGTAATACAACAAAATTGTTTAGTAGTAATTGGACTTGGGATAATAATAATACGGGTAATTGGACTG
GT---
ACTTGGAATAGTGTTAAT---------
AACACTGAACATAATATCACACTCATATGCAGAATAAAACAAATTATAAACATGTGGCAGGGAGTAGGAAAAG
CGATGTATGCCCCTCCCATCAGAGGACAAATTAAATGTTCATCAAAT---
ATTACAGGGCTGCTGTTAACAAGAGATGGTGGTATTACTAACACTAGC-------------------------
----

AATAATACAGAGACCTTCAGACCTGGAGGAGGAGATATGAGGGACAATTGGAGAAGTGAATTATACAAATATA
AAGTAGTACAAATTGAACCATTA---GGAGTAGCACCCACCAAGGCAAAGAGAAGAGTGGTG-----------
----------------------------CAAAGAGAAAAAAGAGAT---TTGGGA---ATAGGAGCT------
---TTGTTCCTTGGGTTCTTGGGAGCA---GCAGGAAGCACTATG---GGCACAGCATCA---
ATAATGCTGACGGTACAG---GCCAGACAA---TTACTG---TCTGGTATAGTGCAGCAGCAGAAC---
AATCTG---CTGAGA---GCTATTGAGGCGCAACAGCATATGTTGCAACTCACAGTCTGGGGCATC
>KT124755
TTCTATTGTAATACAACACAACTGTTTAATAGTACTTGGAATAGTACTGAACGTTTAAATAGCACTGAA----
----

AGTAATGATAATATCACTATCCCATGCAGAATAAAACAAATTATAAACTTGTGGCAGGAAGTAGGAAAAGCAA
TGTATGCCCCTCCCATCAGAGGACAAATTAGATGCTCATCAAAT---
ATTACAGGGCTGCTATTAACAAGAGATGGTGGTAATAACGAAAAC----------------------------
----

AATACCATCGAGATCTTCAGACCTGGAGGAGGAGATATGAGGGACAATTGGAGAAATGAACTATATAAATATA
AAGTAGTAAAAATTGAACCATTA---GGAATAGCACCCACCAAGGCAAAGAGAAGAGTGGTG-----------
----------------------------CAGAGAGAAAAAAGAGCA---GTGGGA---CTAGGAGCT------
---TTGTTCCTTGGGTTCTTGGGAGCA---GCAGGAAGCACTATG---GGCGCAGCGTCA---
ATAACGCTGACGGTACAG---GCCAGACAA---TTACTG---TCTGGTATAGTGCAACAGCAGAAC---
AATCTG---CTGAGG---GCTATTGAGGCGCAACACCAGCTGTTGCAACTCACAGTCTGGGGCATT
>KT124757
TTCTACTGTAATACATCACGATTGTTTAATAGTACTTGGAATAACACTAAAAACAGAGATATAGATATGACTG
GAGAC--

ATAAATGGAACCATCACGCTCCAATGCAGAATAAAACAATTTATAAACATGTGGCAGGGAGTAGGAAAAGCAA
TGTATGCCCCTCCCATCCCGGGACTAATTAGATGTTCATCAAAT---
ATTACAGGGCTGCTATTAACAAGAGATGGGGGTAATGATACTAAG----------------------------
----

AACAATACTACTGAGATCTTCAGACCTGGAGGAGGAGATATGAGGGACAATTGGAGAAGTGAATTATATAAAT
ACAAAGTAGTAAAAATTGAACCATTA---GGACTAGCACCCACCAGGGCAAAGAGAAGAGTGGTG--------
-------------------------------CAGAGAGAAAAAAGAGCA---GTGACA---CTAGGAGCT---
------GTGTTCCTTGGGTTCTTGGGAACA---GCAGGAAGCACTATG---GGCGCAGCGTCA---
CTAACGCTGACGGTACAG---GCCAGACAA---TTACTG---TCTGGTATAGTGCAACAGCAGAGC---
AACCTG---CTGAGA---GCTATTGAGGCGCAACAGCACATGTTGCAACTCACGGTCTGGGGCATC
>KT124794
TTCTACTGTAATTCAACAAAGCTGTTTAACAGTACTTGGTATCCTAAAGGTAATAGTAGTAATAGTACTAGTG
GTACTGAG---

AATACTACTGAGATCATCACGCTCCCATGCAGAATAAAGCAAGTTATAAACATGTGGCAGACAGTAGGAAAAG
CAATGTATGCCCCTCCCATCAGAGGAATAATTAGCTGTTCATCAAAT---
ATTACAGGGATACTATTAACAAGAGATGGTGGTACAAACCAAAAT----------------------------
----

GAAAATGAGACCTTCAGACCTGCAGGAGGAAATATGAGGGACAATTGGAGAAGTGAATTATATAAATATAAAG

TAGTAAAAGTTGAACCATTA---GGAGTAGCACCCACCAAGGCAAGGAGAAGAGTGGTG--------------
-------------------------CAGAGAGAAAGAAGAGCAGTAGTGGGA---ATAGGAGCT---------
GTGTTCCTTGGGTTCTTGTCAGCA---GCAGGAAGCACTATG---GGCGCAGCGGCA---
GTGACGCTGACGGTACAG---GCCAGACAA---TTATTG---TCTGGTATAGTGCAACAGCAGAAT---
AATCTG---CTGAGA---GCTATTGAGGCGCAACAGCATCTGTTGCAACTCACAGTCTGGGGCATC
>KT124800
TTCTACTGTAATACAACACAACTGTTTAACAGTAATTGGACGACTAATAATACTGGGAGTAATGATACGGAGA
TTAATGATACT--
-------------------ATG---
GAGATTAATGATACTATCACCCTCCCATGCAGAATAAAACAAATTATAAACAGGTGGCAGGAAGTAGGAAAAG
CAATGTTTGCCCCTCCTATTAGAGGACAAATCAGCTGTTCATCAAAT---
ATTACAGGGCTGCTATTAACAAGAGATGGTGGCGACAACAACGAGACAGAA----------------------
----

ACCACCGAAATCTTTAGGCCTGGAGGAGGAGATATGAGGGACAATTGGAGAAGTGAATTATATAAATATAAAG
TAGTAAGGGTTGAACCATTA---GGAGTAGCACCCACCAAGGCAAAGAGAAGAGTGGTG--------------
-------------------------CAGAGAGAAAAAAGAGCA---GCG------ATAGGAGCT---------
GTGTTCCTTGGGTTCTTGGGAGCA---GCAGGAAGCACTATG---GGCGCAGCGTCA---
GTGGCGCTGACGGTACAG---GCCAGACAA---TTATTG---TCTGGTATAGTGCAGCAGCAGAAC---
AATCTG---TTGAGA---GCTATTGAGGCGCAACAGCACCTGTTGCAACTCACAGTCTGGGGCATT
>KT124810
TTCTACTGTGAGTCAACACCATTATTTAATAATAGTTCAGAAGGGAACTTTAGTAGTTCAGAAGGGAGCTTTA
GTAGAAGC---
-------------TCAAGT------
AACAACAACAGCACAATCACGCTGTCATGTAAAATAAGACAAATTATAAACATGTGGCAGGAAGTAGGAAAAG
CAATGTATGCCCCTCCCATCAGAGGGCAAATTAGATGCTCATCAAAT---
ATTACAGGGCTGTTATTAACAAGAGATGGTGGTAAGGAGACAGAA----------------------------
----

AATAATACTGAGATCTTCAGACCAGGAGGAGGAAATATGAAGGACAATTGGAGAAGTGAATTATATAAATATA
AAGTAGTAAAAATTGAACCATTA---GGAGTAGCACCCACCAAGGCAAAGAGAAGAGTGGTG-----------
----------------------------CAGAGAGAAAAAAGAGCA---ATAGGAACACTAGGAGCT------
---ATGTTCCTTGGTTTCTTGGGAGCA---GCAGGAAGCACTATG---GGCGCAGCGTCA---
ATAACGCTGACGGTACAG---GCCAGACAA---TTATTG---TCTGGTATAGTGCAACAGCAGAAC---
AATTTG---CTGAGG---GCTATTGAGGCGCAACAACATCTGTTGCAACTCACAGTCTGGGGCATC
>KT124749
TTCTACTGTGATACAACAAAACTGTTTAATAGTACTTGGAATAGTACTTGGAATAATACTACTAGGAATGACA
CT---

GGAGGTGAAGATAAAATTACACTCCCATGCAGAATAAAACAAATTGTAAACCTGTGGCAGGAAGTAGGAAAAG
CAATGTATGCCCCTCCCATCAGTGGACAAATTAGTTGTTCATCAAAT---
ATTACAGGGATACTATTAACAAGAGATGGAGGTAATACAAGCAGT----------------------------
----

ACTATTGAGACCTTCAGACCTGGAGGAGGAGATATGAGGGACAATTGGAGAAGTGAATTATATAAATATAAAG
TAGTAAAAATTGAACCATTA---GGAATAGCACCCACCAAGGCAAAGAGAAGAGTGGTG--------------
-------------------------CAAAGAGAAAAAAGAGCA---TTGGGA---ATAGGAGCT---------
GTGTTCCTTGGGTTCTTGGGAGCA---GCAGGAAGCACTATG---GGCGCAGCATCA---
ATAACGCTGACGGTACAG---GCCAGACTA---TTATTG---TCTGGTATAGTGCAACAGCAAAAC---
AATTTG---CTGAGA---GCTATTGAGGCGCAACAGCATCTGTTGCAACTCACAGTCTGGGGCATT
>KT124753
TTCTACTGTAATTCAACACCACTGTTCAATAGTACTTGGAATGAGACTAGTATTTGGAATGATCCTAATAATA
ATACTTGG---
-------------AATGCT------
AATGCTACTGAATTTATCATACTCCCATGCAGAATAAAACAAATTATAAACATGTGGCAGGAAGTAGGAAAAG
CAATGTATGCCCCTCCCATCAGGGGAATAATTAGATGTTCATCAAAC---
ATTACAGGACTGATATTAACAAGAGATGGTGGTAATAACAATGACACA-------------------------
----

AATGACACCGAGATCTTCAGACCTGGAGGAGGAGATATGAGGGACAATTGGAGAAGTGAATTATATAAATATA

AAGTGGTAGAAATTGAACCATTA---GGAATAGCACCCACCAAGGCAAAGAGAAGAGTGGTG-----------
----------------------------CAGAGAGAAAAAAGAGCA---ATAGGAGCACTAGGAGCT------
---GTGTTCCTTGGGTTCTTGGGAGCA---GCAGGAAGCACTATG---GGCGCAGCGTCA---
CTGACGCTGACGGTACAG---GCCAGACAA---TTATTG---TCTGGTATAGTGCAACAGCAGAAC---
AATTTG---CTGAGG---GCTATTGAGGCGCAACAACATCTGTTGCAACTCACAGTCTGGGGCATC
>KT124754
TTCTACTGTAATACAGCAGCACTGTTTAATAGTACTTGGAATAGTACTGAAGGGTTAAATAGCACTAACGAAA
AT---

GGTACTATCCCTATCACACTCCCATGCAAGATAAAACAAATTATAAACAGGTGGCAGGAAGTAGGAAAAGCAA
TGTATGCCCCTCCCATTAGTGGAAGAATTAACTGTTTATCAAAT---
ATTACAGGGCTGCTACTAACAAGAGATGGTGGTAATAATAGAGAG----------------------------
----

AGCAATGAAACCTTTAGACCTGGAGGAGGAGATATGAGGGACAATTGGAGAAGTGAATTATATAAATATAAAG
TAGTAAGAATTGAACCATTA---GGAGTAGCACCCACCAGGGCAAAGAGAAGAGTGGTG--------------
-------------------------CAGAGAGAAAAAAGAGCA---GTAGGA---CTAGGAGCT---------
TTGTTCCTTGGGTTCTTGGGAGCA---GCAGGAAGCGCTATG---GGCGCAGCGTCA---
ATAACGCTGACGGTACAG---GCCAGACAA---TTATTG---TCTGGAATAGTGCAACAGCAGAAC---
AATCTG---CTAAGA---GCTATTGAGGCGCAACAGCATCTGTTGCAACTCACAGTCTGGGGCATC
>KC247955
TTCTACTGTAATACATCAAAACTGTTTAATGTGAGCACTAGACAGACAAATGAC-------------------
----

ACAAATAGAAATATCACACTCCCATGCAGAATAAAACAAATTATAAACAGGTGGCAGGAGGTAGGAAAAGCAA
TGTATGCCCCTCCCATCAGAGGACAAATTAGATGTTCATCAAAC---
ATTACAGGGCTACTATTAACAAGAGATGGTGGTAACAGCAGCAAC----------------------------
----

AATGAGACCTTCAGACCTGGAGGAGGAGATATGAGAGACAATTGGAGAAGTGAATTATATAAATATAAAGTAG
TAAAAATTGAACCATTA---GGAATAGCACCCACCAAGGCAAGGAGAAGAGTGGTG-----------------
----------------------CAGAGAGAAAAAAGAGCA---GTGACA---CTGGGAGCT---------
ATGTTCCTTGGGTTCTTAGGCATG---GCAGGAAGCACTATG---GGCGCAGCGTCA---
GTGACGCTGACGGTACAG---GCCAGACAA---TTATTG---TCTGGTATAGTGCAACAGCAAAAC---
AATTTG---CTGAGA---GCTATTGAGGCGCAACAGCATCTGTTGCAACTCACGGTCTGGGGCATT
>KT124772
TTCTACTGTAATACATCAAAATTGTTCAATAGTACTTGGCATAGGAATAATAATACAGTCTCAGGAAAT----
----

GTCACAGGGAATATCACACTCCAATGTAGAATAAAACAATTTATAAACATGTGGCAGGAAGTAGGAAAAGCAA
TGTATGCCCCTCCCGTCAGCGGAAAAATTAGTTGTGTATCAAAT---
ATTACAGGGATACTTTTAACAAGAGATGGAGGTAATAACAACACA----------------------------
----

GAGAACAGCACAGAGACCTTCAGACCTGGAGGAGGAGATATGAGGGACAATTGGAGAAGTGAATTATATAAAT
ATAAAGTAGTAAAAATTGAACCATTA---GGACTGGCACCCACCAAGGCAAAGAGAAGAGTGGTG--------
-------------------------------CAGAGAGAAAAAAGAGCA---ATAACG---CTGGGAGCT---
------ATGTTCCTTGGGTTCTTGGGAGCA---GCAGGAAGCACTATG---GGCGCAGCGTCG---
ATGACGCTGACGGTACAG---GCCAGACTA---TTATTG---TCTGGTATAGTGCAACAGCAAAAC---
AACTTG---CTGAGA---GCTATTGAGGCGCAACAACATCTGTTGCAGCTCACAGTCTGGGGCATC
>KT124797
TTCTACTGTGATACATCAAAACTGTTTAATAGTACCTGGATTAGTAATGGTACCTGGATTATAAATAGTACTG
GTAATGGGACT--
-------------AATGAT------
ACTGAAGGGTCAGATATCACGCTCCCATGCAGAATAAGACAAATTGTAAATAGGTGGCAGGAAGTAGGAAAAG
CAATGTATGCCCCTCCTATCAGTGGATATATTAGATGTTCATCAAAT---
ATTACAGGGATACTATTAACAAGAGATGGTGGTAATGACGAGAATACC-------------------------
----

ACCGATACCGAGACCTTCAGACCTGGAGGAGGAGATATGAGGGACAATTGGAGAAGTGAATTATATAAATATA

```
AAGTAGTAAAACTTGAACCCTTA---GGAATAGCACCCACCAAGGCAAAGAGGAGAGTGGTG-----------
----------------------------CAGAGAGAAAAAAGAGCA---GTGGGA---CTAGGAGCT------
---ATGTTCCTTGGGTTCTTGGGAGCA---GCAGGAAGCACTATG---GGCGCAGCGTCA---
ATAACGCTGACGGTACAG---GCCAGACTA---TTATTG---TCTGGTATAGTGCAGCAGCAAAAC---
AACTTG---CTGCAG---GCTATTGAGGCGCAACAACATCTGTTGCAACTCACAGTCTGGGGCATC
>KC248026
TTCTATTGTGACACAACACAACTGTTTAATAGCACTTGGATTAATGGTACCAGGAATGATACTGAAGGG----
-------------------------------------------------------------------------
----------------------------
TCAAATAATACTATCACACTCCAATGCAGAATAAAACAGATTATAAACATGTGGCAGGAAGTAGGAAAAGCAA
TGTATGCCCCTCCCATCAAAGGACAAATTAGATGTTCATCAAAT---
ATTACAGGGTTACTATTAACAAGAGATGGTGGTACGGACAATGACACAAGT----------------------
-------------------------------------------------------------------------
----------
AATGAGACCGAGATCTTCAGACCGGGAGGAGGAAATATGAAGGACAATTGGAGAAGTGAATTATATAAATATA
AAGTTGTAAAAATTGAACCATTA---GGAGTAGCACCCACCAAGGCAAAGAGAAGAGTGGTG-----------
----------------------------CAAAGAGAAAAAAGAGCA---GTGGGA---ATAGGAGCA------
---TTGTTCCTTGGGTTCTTGGGAGCA---GCAGGAAGCACTATG---GGCGCAGCATCA---
ATAACGCTGACGGTACAG---GCCAGACAA---TTATTG---TCTGGGATAGTGCAACAGCAGAAC---
AACTTG---CTGAGA---GCTATTGAGGCGCAACAACATATGTTGCAACTCACAGTCTGGGGCATC
>KC248015
TTCTACTGTGATTCATCAAAACTGTTTAATAGTACTTGGAATGATTCTACTTCAGGGAATACCAAT-------
-------------------------------------------------------------------------
----------------------------
GCAAATGACACTATCATACTCCCATGCAGAATAAGGCAAATTATAAACATGTGGCAGGAAGTAGGAAAAGCAA
TGTATGCCCCTCCCATTAGTGGAATAATTAGATGTTCATCAAAT---
ATTACAGGGCTGCTATTAACAAGAGATGGTGGTATTAACAAGAGTGAA-------------------------
-------------------------------------------------------------------------
-------------
ACCACCGAGATCTTCAGACCTGGAGGAGGAGATATGAGGGATAATTGGAGAAGTGAATTATATAAATATAAAG
TAGTAAAAATTGAACCAGTA---GGAGTAGCACCTACCAAGGCAAAGAGAAGAGTGGTG--------------
-------------------------CAGAGAGAAAAAAGAGCA---GTGGGA---CTAGGAGCT---------
TTGTTCCTTGGGTTCTTGGGAGCA---GCAGGAAGCACTATG---GGCGCAGCGTCA---
ATAACGCTGACGGTACAG---GCCAGACAA---GTATTG---TCTGGTATAGTGCAACAGCAGAAC---
AATCTG---CTGAGA---GCTATTGAGGCGCAACAGCACCTGTTGCAACTCACAGTCTGGGGCATC
>KT124802
TTCTACTGTAATACTTCACAACTGTTTAATAGTTCCTGGAACGGGACACAGAGCGAGACAAATAACACCAAC-
-------------------------------------------------------------------------
----------------------------
AGAAATGACACACTCACACTCCAATGCAAAATAAGACAAATTGTAAACATGTGGCAGGAAGTAGGAAAAGCAA
TGTATGCCCCTCCCATCAGGGGACCAATTAGATGTTCATCAAAC---
ATTACAGGGCTACTATTAACAAGAGATGGTGGTAGTGGTAATACAACC-------------------------
-------------------------------------------------------------------------
-------
AGCAACACTACTGAGATCTTCAGACCTGGAGGAGGGAATATGAGGGACAATTGGAGAAGTGAATTATATAAAT
ATAAAGTAGTAAGAATTGAACCATTA---GGAGTAGCACCCACCAAGGCAAAGAGAAGAGTGGTG--------
-------------------------------CAGAGAGAAAAAAGAGCA---GTGGGA---CTGGGAGCT---
------CTGTTCCTTGGGTTCTTGGGAGCA---GCAGGGAGCACTATG---GGCGCAGCGTCA---
TTGACGCTGACGGTACAG---GCCAGACAA---TTATTG---TCTGGTATAGTGCAACAGCAGAAC---
AATTTG---CTGAAG---GCTATTGAGGCGCAACAGCATCTGTTGCAACTCACAGTCTGGGGCATC
>KT124765
TTTTACTGTAATACAACAGAACTGTTTAATAGTATTTGGGTTAATGGTTCTTGGGTTAATGGGACTAATAATG
ATACTGAA-----------------------------------------------------------------
----------------------------
TCAAATTCCAATATCACACTTCCATGCAGAATAAAACAAATTATAAACATGTGGCAAAAAGTAGGAAAAGCAA
TATATGCCCCTCCTATCAGAGGACAAATTAGATGTTCATCAAAT---
ATTACAGGGCTGCTACTAACAAGGGATGGTGGGATAGACAATAAC----------------------------
-------------------------------------------------------------------------
----------
AAAACCAATGAGACCTTCAGGCCTGGAGGAGGAGATATGAGAGACAATTGGAGAAGTGAATTATACAAATATA
```

AAGTAGTAAAAATTGAACCATTA---GGAGTAGCACCCACCAGGGCAAAGAGAAGAGTGGTG-----------
----------------------------CAGAGAGAAAAAAGAGGA------------CTAGGACTT------
---GTGTTCCTTGGGTTCTTGGGAGCA---GCAGGAAGCACTATG---GGCGCAGCGTCA---
GTGACGCTGACGGTACAG---GCCAGACAA---TTATTG---TCTGGTATAGTGCAACAGCAAAAC---
AATTTG---CTGAAG---GCTATTGAGGCGCAACAGCATCTGTTGCAACTCACAGTCTGGGGCATT
>KT124811
TTCTACTGTAATACATCACAGTTGTTTAATGGTACTTGGAATAGTACTGTGGGATGGATAACTGAAGGATCAA
ATAAC--

ACTGGAGGAATTATCACACTCCCGTGCAGAATAAAACAAATTGTAAACAGATGGCAGGAAGTAGGAAAGGCAA
TGTATGCCCCTCCTATCAGAGGAAATATTAGCTGTCTATCCAAT---
ATCACAGGGCTATTATTGACAAGAGATGGTGGTAGCACAAACAGTAGT-------------------------
----

AATGATACAGAGACCTTCAGACCTGGAGGAGGAAATATGATGGATAATTGGAGAAGTGAATTATATAAATATA
AAGTAGTAAAAATCGAACCATTA---GGAGTAGCACCCACCGAGGCAAAGAGAAGAGTGGTG-----------
----------------------------CAAAGAGACAAAAGAGCA---GTGGGAACAATAGGAGCT------
---ATGTTCCTTGGGTTCTTGGGAGCA---GCAGGAAGCACTATG---GGCGCAGCGTCA---
ATAACGCTGACGGTACAG---GCCAGACAA---TTGTTG---TCTGGTATAGTGCAACAGCAGAGC---
AATCTG---CTGAAG---GCTATTGAGGCGCAACAGCGTATGTTGCAACTCACAGTCTGGGGCATC
>KT124767
TTCTACTGTAATACAACAGAACTGTTTAATAGTGTTTGGAATAATAGTGGAAGGGTAACTGGCACTGGA----
----

AATAGTACTAACATCACACTCCCATGCAGAATAAAACAATTTATAAATATGTGGCAAGAAGTAGGAAAAGCAA
TGTATGCCCCTCCCATCAGTGGACAAATTAAATGTTCATCAAAT---
ATTACAGGGCTGCTACTAACAAGAGATGGTGGTCCTAACAAAACG----------------------------
----

GAGAATGATACCTTCAGACCTACAGGAGGAGATATGAGAAATAATTGGAGAAGTGAATTATATAAATATAAAG
TAGTACAAATTGAACCATTA---GGAATAGCACCCACCACGGCAAAGAGAAGAGTGGTG--------------
-------------------------CAGAGAGAAAAAAGAGAC---CTAGGA---CTAGGAGCT---------
TTGTTCATTGGGTTCTTGGGAGCA---GCAGGAAGCACTATG---GGCGCAGCGTCA---
GTGACGCTGACGGTACAG---GCCAGACAA---TTATTG---TCTGGTATAGTGCAACAGCAGAAC---
AATTTG---CTGAGA---GCTATTGAGGCGCAACAACATCTGTTGCAACTCACAGTCTGGGGCATC
>KY324301
TTCTACTGTAATACAACACAACTGTTTAATAGTGACTGGACGATTAATAATGGTACTGGGAATGATACTGAAG
GGTCATTA---

AATAGCAGTGACATCACACTCCCATGCAGAATAAGACAAATTATAAATATGTGGCAGAAAGTAGGAAAAGCAA
TGTATGCCCCTCCTATCAGAGGACAAATTAGATGCTCATCAAAT---
ATTACAGGGCTGCTATTAACAAGAGATGGTGGACAGGGTAGTAATGCT-------------------------
----

ACAAATGAAACCTTCAGACCTGCAGGAGGAAATATGAAAGATAATTGGAGAAGTGAATTATATAAATATAAAG
TAGTAAAAATTGAACCATTA---GGAGTAGCACCCACCAAGGCAAAGAGAAGAGTGGTG--------------
-------------------------CAGAGAGAAAAAAGAGCA---GCA------CTAGGAGCT---------
ATGTTCCTTGGGTTCTTGGGAGCA---GCAGGAAGCACTATG---GGCGCAGCGTCA---
ATAACGCTGACGGTACAG---GCCAGACTG---TTATTG---TCTGGTATAGTGCAACAGCAAAAC---
AATTTG---CTGAGG---GCTATTGAGGCGCAACAGCATCTGTTGCAACTCACAGTCTGGGGCATC
>KY324379
TTTTATTGTAACACAACACAACTGTTTAATAGTACATGGGATAATGGTACATGGCATAATGAGAATAATGAGA
ATAATGGAACA---TTT--
-------------AATGCA------
ACAAGCCATGAAAATATCACAATCCCATGCAGAATAAAACAAATTATAAACATGTGGCAGACAGTAGGAAAAG
CAATGTATGCCCCTCCCATTAGTGGACTAATCAGATGTTCATCAAAT---
ATTACAGGGCTGCTATTAACAAGAGATGGTGGTAGTAATAACAGC----------------------------
----

CTTAATGAGACCTTCAGACCTGGAGGAGGAGATATGAGGGACAATTGGAGAAGTGAATTATATAAATATAAAG

TAGTAAAAATTGAACCATTA---GGAATAGCACCCTCCAAGGCAAAGAGAAGAGTGGTG--------------
-------------------------CAGAGAGAAAAAAGAGCA---GTGGGA---CTAGGAGCC---------
ATGTTCCTTGGGTTCTTGGGAGCA---GCAGGAAGCACTATG---GGCGCAGCGTCA---
ATGACGCTGACGGTACAG---GCCAGACAA---TTATTG---TCTGGTATAGTGCAACAGCAGAGC---
AATTTG---CTGAGA---GCTATTGAGGCGCAACAGCACATGTTGCAGCTAACAGTCTGGGGCATC
>KY324630
TTTTACTGTAATACAACCCAGCTATTTAATAGCAGTTGGAATGCAACACAACTATGGAATACTGGGATGGAGA
ATAATAGCTTT--
-------GGGATGGAGAAT------
AATAGCTTAGAAATTATCACACTCCCATGTAGAATAAAACAAATTATAAACATGTGGCAGGAAGTAGGAAAAG
CAATGTATGCCCCTCCCATCAGCGGATTAATTAGATGTTCATCAAAT---
ATCACAGGGCTGCTATTAACAAGAGATGGTGGGATGCACAATAAGAGT-------------------------
----

AATGAGACTGAGACCTTCAGACCTGCAGGGGGAGATATGAAGGACAATTGGAGAAGTGAATTATATAAATATA
AAGTAGTAAAAATTGAACCTTTA---GGGATAGCACCCACCAAGGCAAAGAGAAGAGTGGTG-----------
----------------------------CAAAGAGAAAAAAGAGCA---GTGACG---CTGGGAGCT------
---ATGTTCCTTGGGTTCTTGGGAGCA---GCAGGAAGCACTATG---GGCGCAGCGTCA---
ATGACGCTGACGGTACAA---GCCAGACAA---TTATTG---TCTGGTATAGTGCAACAGCAGAAC---
AATTTG---CTGAGA---GCTATTGAGGCGCAACAGCATCTGTTGCAACTCACGGTCTGGGGCATC
>KY324641
CTCTACTGTAATACAACACAACTGTTTAACAGTACTTGGTTTAACAGTACCAACATTACTGACACTAGAGAAA
AT---

AGCACAGAAATAATCACACTCCCATGTAGAATAAAACAAATTGTAAACATGTGGCAGGAGGTAGGAAAAGCAA
TGTATGCCCCTCCCATCAAAGGAAACATTAGCTGCATATCGAAT---
ATTACAGGGCTGCTGTTAATAAGAGATGGTGGTAACATCACCAAT----------------------------
----

AGTACATCCGAGACCTTCAGACCAGGAGGAGGAGATATGAGGGACAATTGGAGAAGTGAATTATACAGATATA
AAGTAGTAAAAATTGAACCATTA---GGAGTAGCCCCCACCAAGGCAAGGAGAAGAGTGGTG-----------
----------------------------CAGAGAGAAAAAAGAGCA---ATAGGGACATTTGGAGCT------
---ATGTTCCTTGGTTTCTTGGGAGCA---GCAGGAAGCACTATG---GGCGCAGCGTCA---
ATGGCGCTGACGGTACAG---GCCAGACTA---TTATTG---TCTGGTATAGTGCAACAGCAGAAC---
AATTTG---CTGAGA---GCTATTGAGGCGCAACAGCACATGTTGCAACTCACAGTCTGGGGCATC
>KY324761
TTCTACTGTAATACAACAAAATTGTTTAATAGTACTTGGAATTTTAATAGTACTTGGAATACAACACAATTGC
TTAATAGT---
-AATTGGAATACAACT---------
AGTACTGAGAAGAACATCACACTCCCATGTAGAATAAAACAAATTATAAACAGGTGGCAGGACGTAGGAAAAG
CAATGTATGCCCCTCCCATCAGAGGACAAATTAGATGTTCATCAAAC---
ATTACAGGGCTACTATTAACAAGAGATGGTGGTGTTGAGAACACC----------------------------
----

AACACCACAGAAGTCTTCAGACCTGGAGGAGGAGATATGAGAGACAATTGGAGAAGTGAATTATATAAATATA
AAATAGTAAAAATTGAACCATTA---GGAATAGCACCCACCAAGGCAAAGAGAAGAGTGGTG-----------
----------------------------CAAAGAGAAAAAAGAGCT---GTAACA---TTAGGAGCT------
---CTGTTCCTTGGGTTCTTGGGAGCA---GCAGGAAGCACTATG---GGCGCAGCGTCA---
ATGACGCTGACGGTACAG---GCCAGACTA---TTATTG---TCTGGTATAGTGCAGCAGCAAAAC---
AACTTG---CTGCAG---GCTATTGAGGCGCAACAGCATCTGTTGCAACTCACAGTCTGGGGCATC
>KY324808
TTCTACTGTAATACAACACCACTGTTTAATAGTACTTGGGGGACTAATACTACAAAGCCAGAAATC-------
----

GCAAATGGAACCATCACACTCCAATGCAGAATAAAACAAATTATAAACATGTGGCAGGAAGTAGGAAAAGCAA
TGTATGCCCCTCCCATCAGGGGAGAAATTAGATGTTCATCAAAT---
ATTACAGGGCTGCTATTAACAAGAGATGGTGGTGTTAACAAGACAAATGAT----------------------
----
ACAAATGACACAAAT---
AACACAGAGACCTTTAGACCTGGAGGAGGAGATATGAGGGACAATTGGAGAAATGAGTTATATAAATATAAAG

TAGTAAAAATTGAACCATTA---GGAATAGCACCCACCAAGGCAAAGAGAAGAGTGGTG-------------
-------------------------CAGAGAGAAAAAAGAGCG---GCA------CTAGGAGCT---------
CTTGTCCTTGGGTTCTTGGGAGCA---GCAGGAAGCACTATG---GGCGCAGCGTCA---
GTGACGCTGACGGTACAG---GCCAGACTA---TTATTG---TCTGGTATAGTGCAACAACAGAAC---
AATCTG---TTGAGA---GCTATTGAGGCGCAACAGCATATGTTGCAACTCACAGTCTGGGGCATC
>MK115683
TTCTACTGTGATTCAACGCAACTGTTTAATAGTACCTGGCTGCCTAATAATGAGAGTAATTGGTATAATAATG
GGAAT---
-AATACTACAGGGTCAAAC------
AACAGTACAGCCAATATCACACTCCCATGCAGAATAAAACAAATTATAAACCTGTGGCAAGAAGTAGGAAAAG
CAATGTATGCCCCTCCCATCAGAGGAGCAATCAATTGCTTGTCAAAT---
ATTACAGGGTTGTTACTAACAAGAGATGGTGGGAATAATAACGAG---------------------------
----

ACAACCGACCCCACCGAGATCTTTAGACCTGGAGGAGGAAATATGAAGGACAATTGGAGAAGTGAATTATATA
GATATAAAGTAGTAAAAATTGAACCATTA---GGAGTAGCACCCACCACGGCAAAGAGAAGAGTGGTG-----
----------------------------------CAGAGAGAAAAAAGAGCA---GTGGGT---
CTCGGAGCC---------TTGTTCCTTGGGTTCTTGGGAGCA---GCAGGAAGCACTATG---
GGCGCAGCGTCA---GTAACGCTGACGGTACAG---GCCAGACAA---TTATTG---
TCTGGTATAGTGCAGCAGCAGAAC---AATTTG---CTGAGG---
GCTATTGAGGCGCAACAGCATATGTTGCAACTCACAGTCTGGGGCATC
>MK115918
TTTTACTGTAATACAACACAACTGTTTAATAGTACTTGGAATAGTACTGGGGATAGTACTTGGAAT-------
----

AATACAGATAATATCACACTCCCATGTAGAATAAGGCAAATTGTAAACATGTGGCAGGAAGTAGGAAAGGCAA
TGTATGCTCCTCCCATCCGTGGACAAATTAAATGTTCATCAAAT---
ATTACAGGGCTGTTATTAACAAGGGATGGTGGTAACGGGAATGAGACG------------------------
----

AGAGAAACAGAAACCTTCAGACCTGGAGGAGGAGATATGAGGGACAATTGGAGAAGTGAATTATATAAATATA
AAGTAGTAAGAATTGAACCATTA---GGAATAGCACCCACTAAGGCAAAGAGAAGAGTGGTG-----------
----------------------------CAAAGAGAAAAAAGAGCA---ATAGGAACACTGGGAGCT------
---GTGTTCCTTGGGTTCTTGGGAGCA---GCAGGAAGCACTATG---GGCGCAGCGTCA---
ATGACGCTGACGGTACAG---GCCAGGCAA---TTATTG---ACTGGTATAGTGCAGCAGCAGAAC---
AATCTG---CTGAAG---GCTATTGAGGCGCAACAGCATCTGTTGCAACTCACAGTCTGGGGCATT
>MK116058
TTCTATTGTAATACAACACAATTGTTTAATAGCACTTGGGATAATGGTACTGAAAAGTCAAGTAACCTTGGAG
GA--

AATGATACAACCATCATTCTCCCATGCAGAATAAAACAAATTATAAACATGTGGCAGGAAGTAGGAAAAGCAA
TGTATGCCCCTCCCATCAAAGGAATAATTAGATGTTCATCAAAT---
ATTACAGGGCTGCTATTGACAAGAGATGGAGGTAATAGTACTGACACA------------------------
----

AATGGGAGCGAGATCTTCAGACCTGGAGGAGGAGATATGAGGGACAATTGGAGAAGTGAATTATATAAGTATA
AAGTAGTAAAAATTGAACCATTA---GGAGTAGCACCTACCAAGGCAAAGAGAAGAGTGGTG-----------
----------------------------CAGAGAGAAAAAAGAGCA---GTGGGA---CTAGGAGCT------
---TTGTTCCTTGGGTTCTTGGGAGCA---GCAGGAAGCACTATG---GGCGCAGCGTCT---
CTGACGCTGACGGTACAG---GCCAGACAA---TTGTTG---TCTGGTATAGTGCAACAGCAGAAC---
AATTTG---CTGAGG---GCAATTGAGGCGCAACAGCATCTGTTGCAACTCACAGTCTGGGGCATC
>MK116212
TTTTACTGTAATTCAACACAACTGTTTAATAGTACTTGGAATAATAATGGTACTAATACTTGGAAT-------
----

AGTACAGGTAATATCACACTCCCATGTAAAATAAGGCAAATTGTAAACATGTGGCAGAAAGTAGGAAAAGCAA
TGTATGCTCCTCCCATCCGTGGACAAATTAAATGTTCATCAAAT---
ATTACAGGGCTGCTATTAACAAGAGATGGTGGTAACGAGAGTGAG---------------------------
----

AGCGAAACCTTCAGACCTGGCGGAGGAGATATGAGGGACAATTGGAGAAGTGAATTATACAAATATAAAGTAG
TAAGAATTGAACCATTA---GGACTAGCACCCACTAAGGCAAAGAGAAGAGTGGTG-----------------
----------------------CAGAGAGAAAAAAGAGCA---ATAGGAACACTGGGAGCT---------
GTGTTCCTTGGGTTCTTGGGAACA---GCAGGAAGCACTATG---GGCGCAGCGTCA---
ATGACGCTGACGGTACAG---GCCAGGCAA---TTATTG---TCTGGTATAGTGCAGCAGCAGAAC---
AATCTG---CTGAAG---GCTATTGAGGCGCAACAGCATCTGTTGCAACTCACAGTCTGGGGCATT
>MK086129
TTCTACTGTAATACAACAAAACTGTTTAATAGGACCTTTAATAGTACTTGGAATAGTACTGAAGGGTCA----
----

AATAGTACAACTATTACACTCCCATGCAGAATAAAACAAATTATAAACATGTGGCAGGAAATAGGAAAAGCAA
TGTATGCCCCTCCCATCAGCGGACAAATTAGATGTTCATCAAAT---
ATCACAGGGCTGATATTAACAAGAGATGGTGGTAATGACACTAAC----------------------------
----

AATGAGACCTTCAGACCTGGAGGAGGAAATATGAAAGATAATTGGAGAAGTGAATTATATAAATATAAAGTAG
TAAAAATTGAACCATTA---GGAGTAGCACCCACCAGGGCAAAGAGGAGAGTGGTG-----------------
----------------------CAGAGAGAAAAAAGAGCA---GTGGGAGTAATAGGAGCT---------
ATGTTCCTTGGGTTTCTGGGAGCA---GCAGGAAGCACTATG---GGCGCAGCATCA---
ATAACGCTGACGGTACAG---ACCAGACGA---TTAGTG---TCTGGTATAGTGCAACAGCAGAAC---
AATCTG---CTGAGA---GCTATTGAGGCGCAACACAATCTGTTGCAACTCACAGTCTGGGGCATC
>MN090868
TTTTACTGTAATTCAACACAACTGTTTAATAGTACTTGGAATAATAATGGTACTAATACTTGGAAT-------
----

AGTACAGGTAATATCACACTCCCATGTAAAATAAGGCAAATTGTAAACATGTGGCAGAAAGTAGGAAAAGCAA
TGTATGCTCCTCCCATCCGTGGACAAATTAAATGTTCATCAAAT---
ATTACAGGGCTGCTATTAACAAGAGATGGTGGTAACGAGAGTGAG----------------------------
----

AGCGAAACCTTCAGACCTGGCGGAGGAGATATGAGGGACAATTGGAGAAGTGAATTATATAAATATAAAGTAG
TAAGAATTGAACCATTA---GGACTAGCACCCACTAAGGCAAAGAGAAGAGTGGTG-----------------
----------------------CAGAGAGAAAAAAGAGCA---ATAGGAACACTGGGAGCT---------
GTGTTCCTTGGGTTCTTGGGAACA---GCAGGAAGCACTATG---GGCGCAGCGTCA---
ATGACGCTGACGGTACAG---GCCAGGCAA---TTATTG---TCTGGTATAGTGCAGCAGCAGAAC---
AATCTG---CTGAAG---GCTATTGAGGCGCAACAGCATCTGTTGCAACTCACAGTCTGGGGCATT
>AF490512
TTCTACTGTAATACAACAAAACTGTTTAATAGTACTTGGAAGTTTAATGGTACTTGGAAGGTTGTTGAAGGAT
CAAGT--

GACACTGCAGAAAATATCACACTCCCATGCAGAATAAAACAAATTATAAACATGTGGCAGGAAGTAGGAAAAG
CAATGTATGCCCCTCCCATCAGAGGACAAATTAGATGTTCATCAAAT---
ATTACAGGGTTACTATTAACAAGAGATGGTGGTAATAACACAAAC----------------------------
----

GAGACCCCCGAGACCTTCAGACCTGGAGGAGGAAATATGAAGGACAATTGGAGAAGTGAATTATATAAATATA
AAGTAGTAAAAATTGAACCATTA---GGAGTAGCACCCACCAAGGCAAAGAGAAGAGTGGTG-----------
----------------------------CAGAGAGAAAAAAGAGCA---GTGGGA---ATAGGAGCT------
---TTGTTCCTTGGGTTCTTGGGAGCA---GCAGGAAGCACTATG---GGCGCAGCGTCA---
ATAACGCTGACGGTACAG---GCCAGACAA---TTATTG---TCTGGTATAGTGCAACAGCAAAAC---
AATTTG---CTGAGG---GCTATTGAGGCGCAACAGCATCTGTTGCAACTCACAGTCTGGGGCATC
>U43096
TTCTACTGTAATTCAGCACAACTGTTTAATAGTACTTGGAATGATACTAAAGAGTCAAATAAC----------
----

ACAAATGGAACTATCACACTCCCATGCAGAATAAAACAAATTATAAACATGTGGCAGGAAGTAGGAAAAGCAA
TGTATGCCCCTCCCATCAGTGGACAAATTAGATGTTCATCAAAT---
ATTACAGGGCTGCTATTAACAAGAGATGGTGGTAAAAATAAGGAC----------------------------
----

AACGAGACCGAGACCTTCAGACCTGGAGGAGGAAATATGAGGGACAATTGGAGAAGTGAATTATATAAATATA
AAGTAGTAAAAATTGAACCATTA---GGAGTAGCACCCACCAAGGCAAAGAGAAGAGTGGTG-----------
----------------------------CAGAGAGAAAAAAGAGCA---GTGGGACTGCTGGGAGCT------
---GTGTTCCTTGGGTTCTTGGGAGCA---GCAGGAAGCACTATG---GGCGCACGGTCA---
ATGGCGCTGACGGTACAG---GCCAGACAG---TTATTG---TCTGGTATAGTGCAACAGCAGAAC---
AATTTG---CTGAGG---GCTATTGAGGCGCAACAGCATCTGTTGCAACTCACAGTCTGGGGCATC
>U43141
TTCTACTGTAATTCAACAAAACTGTTTAATAGTACTTGGAATAATACTAGTACTTGGAATGATAAT-------
----

GGAAATGACACTATCATACTGCCATGTAGAATAAAACAAATTATAAACATGTGGCAGGAAGTAGGAAAAGCAA
TGTATGCCCCTCCCATTGGAGGACTAATTAGATGTTCATCAAAT---
ATTACAGGGCTGATATTAACAAGAGATGGTGGTAATGATAACAGT----------------------------
----

AGTACCACCGAGATCTTCAGACCTGGAGGGGGAAATATGAGGGACAATTGGAGAAATGAATTATATAAATATA
AAGTAGTAAAAATTGAACCATTA---GGAGTAGCACCCACCAAGGCAAAGAGAAGAGTGGTG-----------
----------------------------CAGAGAGAAAAAAGAGCG---GTGGGAATGCTAGGAGCT------
---ATGTTCCTTGGGTTCTTGGGAGCA---GCAGGAAGCACTATG---GGCGCACGGTCA---
CTGACGCTGACGGTACAG---GCCAGACAA---TTATTG---TCTGGTATAGTGCAACAGCAAAAC---
AATTTG---CTGAGA---GCTATTGAGGCGCAACAACATCTGTTGCAACTCACAGTCTGGGGCATC
>EF514704
TTCTACTGTAGTACAACACAATTGTTTAATAGTACTTGGAGTAGTACTGGAGAGTCAAATAACACCAATGGCA
CA---

GGAACAAATGGCAATATCATACTCCCATGCAGAATAAGACAAATTGTAAACTTGTGGCAGGAAGTAGGAAAAG
CAATGTATGCCCCTCCCATCAGAGGACTAATTAGATGTTCATCAAAT---
ATTACAGGGCTGCTATTAACAAGAGATGGTGGTAGTGGTAATAACACG-------------------------
----

GACAATGCCACCGAGATCTTCAGACCTGGAGGAGGAAATATGAGGGACAATTGGAGAAGTGAATTATATAAAT
ATAAAGTAGTAAAAATTGAACCACTA---GGAGTAGCACCCACCAAGGCAAAGAGAAGAGTGGTG--------
-------------------------------CAGAGAGAAAAAAGAGCA---GTGGGAGTAATGGGAGCT---
------ATGTTCCTTGGGTTCTTGGGAGCA---GCAGGAAGCACTATG---GGCGCAGCATCA---
ATGACGCTGACGGTACAG---GCCAGACAA---TTATTG---TCTGGTATAGTGCAACAGCAAAAC---
AATTTG---CTGAAG---GCTATTGAGGCGCAGCAGAATCTGTTGCAACTCACAGTCTGGGGCATC
>EF514705
TTCTACTGTAACACTTCACAACTGTTTAATAGTACTTGGTATAGCACTTGGAATAGTACTTGGTATAGCAATA
GGAATAGTACT--
-------GGAGGGTCAACT------
AACGAGAGCGACATAATCTCAATCCCATGCAAGATAAAACAAATTATAAACATGTGGCAGGAAGTAGGAAAAG
CAATGTATGCCCCTCCCATTAGAGGACAAATTAACTGTTTCTCAAAC---
ATTACAGGGCTACTATTAACAAGAGATGGTGGTCTAAATGTTACC----------------------------
----

AATACCAGTGAGACCTTCAGACCTGGAGGAGGAGATATGAGGGACAATTGGAGAAGTGAATTATATAAATATA
AAGTAGTAAAAATTGAACCATTA---GGAATAGCACCTACCCAGGCAAAGAGAAGAGTGGTG-----------
----------------------------CAGAGAGAAAAAAGAGCA---GTGGGA---ATAGGAGCT------
---GTATTCCTTGGGTTCTTGGGAGCA---GCAGGAAGCACTATG---GGCGCAGCGTCA---
GTGGCGCTGACGGTACAA---GCCAGACAA---TTATTG---TCTGGTATAGTGCAACAGCAAAAT---
AATTTG---CTGAGG---GCTATTGAGGCGCAACAGCACCTGTTGCAACTCACAGTCTGGGGCATC
>EF514706
TTCTACTGTAATACAACACTACTGTTTAATAGTACTTGGAATGATACTACAGGAAGGTTAAAT----------
----

ACCACTGAAGACATCACACTCCCATGCAGAATAAAACAAATTATAAACATGTGGCAGGAAGTAGGAAAAGCAA
TGTATGCCCCTCCCATCAGAGGACAAATTAGCTGTTCATCAAAT---
ATTACAGGAATACTATTAACAAGAGATGGTGGTAATAATAATAAT----------------------------
----

```
ACCAACGAGACCTTCAGACCTGCAGGAGGAAATATGAAGGATAATTGGAGAAGTGAATTATATAAATATAAAG
TAGTAAAAATTGAACCATTA---GGAGTAGCACCCACCGAGGCAAAGAGAAGAGTGGTG--------------
-------------------------CAAAGAGAAAAAAGAGCA---GTGGGA---CTAGGAGCT---------
CTGTTCCTTGGGTTCTTGGGAGCA---GCAGGAAGCACTATG---GGCGCAGCGTCA---
ATGACGCTGACGGTACAA---GCCAGACAA---TTATTG---TCTGGTATAGTGCAACAGCAGAAC---
AATTTG---CTGAGG---GCTATTGAGGCGCAACAGCGTATGTTGCAACTCACAGTCTGGGGCATC
>EF514707
TTCTACTGTAATACAACACCACTGTTTAATAGTACGTGGAATACTAATACTACAGAGCTAAATAACACTGATG
AC-----------------------------------------------------------------------
----------------------------
AACAATGGCACATTCACACTCTCATGCAGAATAAAACAAATTATAAACATGTGGCAGGAAGTAGGAAAAGCAA
TGTATGCCCCTCCAATCAAAGGACGAATTAGCTGTTCATCAAAT---
ATTACAGGGCTGCTACTAACAAGAGATGGTGGTAACCCAAACAGTACC-------------------------
-------------------------------------------------------------------------
----------
AGTACCAACGAGACCTTCAGACCTGCAGGAGGAGATATGAGAGATAATTGGAGAAGTGAATTATATAAATATA
AAGTAGTAAGAATTGAACCATTA---GGAGTAGCACCCACCAAGGCAAAGAGAAGAGTGGTG-----------
----------------------------CAGAGAGAAAAAAGAGGA------------ATAGGAGTT------
---ATGCTCCTTGGGTTCTTGGGAGCA---GCAGGAAGCACTATG---GGCGCAGCGTCA---
ATGGCGCTGACGGTACAG---GCCAGACGA---TTATTG---TCTGGTATAGTGCAACAGCAGAAC---
AATTTG---CTGAGA---GCTATTGAGGCGCAACAGCATATGTTGCAACTCACAGTCTGGGGCATC
>EF514709
TTCTACTGTAATACAACACAACTGTTTAATAGTACTTGGAATAGTACTACAGGGTCAAATAACACTGAA----
-------------------------------------------------------------------------
----------------------------
AGAAATGACACAATCACACTCCCTTGCAGAATAAAACAAATTGTAAACAGGTGGCAGGAAGTAGGAAAAGCAA
TGTATGCCCCTCCCATCAAAGGACAAATTAGATGTTCATCAAAT---
ATTACAGGGCTGCTATTAACAAGAGATGGTGGTAATAACGCAAGC----------------------------
-------------------------------------------------------------------------
-------------
GGGCCTGAGATCTTCAGACCTGGAGGAGGAGATATGAGGGATAATTGGAGAAGTGAGTTATATAAATATAAAG
TAGTAAAAATTGAACCATTA---GGAGTAGCACCCACCAAGGCAAAGAGAAGAGTGGTG--------------
-------------------------CAGAGAGAAAAAAGAGCG---GCAGGA---CTAGGAGCT---------
ATGTTCCTTGGGTTCTTGGGAGCA---GCAGGAAGCACTATG---GGCGCAGCGTCA---
ATAACGCTGACGGTACAG---GCCAGACAA---TTATTG---TCTGGTATAGTGCAACAGCAGAAC---
AATCTG---CTGAGG---GCTATTGAGGCGCAACAGCATCTGTTGCAACTCACAGTCTGGGGCATC
>EF514710
TTCTACTGTAATACAACACAACTGTTTAATAGTACTTGGAATGATACTAGCAACTCAAATACC----------
-------------------------------------------------------------------------
----------------------------
ACAAATGGCACTATCACACTCCCATGTAGAATAAGACAAATTGTAAATTTGTGGCAGGAAGTAGGAAAAGCAA
TGTATGCCCCTCCCATCAGGGGACTAATTAAATGCTCATCAAAT---
ATTACAGGGCTGATATTAACAAGAGATGGTGGTAATAACATAAGC----------------------------
-------------------------------------------------------------------------
----------
AATACCACCGAGATCTTCAGACCTGGAGGAGGGGATATGAGGGACAATTGGAGAAGTGAATTATATAAGTATA
AAGTAGTAAAAATTGAACCATTA---GGAGTAGCACCCACCAAGGCAAAGAGAAGAGTGGTG-----------
----------------------------CAGAGAGAAAAAAGAGCA---GTGGGA---ATAGGAGCT------
---GTGTTCCTTGGGTTCTTGGGAGCG---GCAGGAAGCACTATG---GGCGCAGCGTCA---
CTGACGCTGACGGTACAG---GCCAGACAA---TTATTG---TCTGGTATAGTGCAACAGCAAAAC---
AATTTG---CTGAAG---GCTATTGAGGCGCAGCAGCATCTGTTGCAACTCACAGTCTGGGGCATC
>EF514711
TTTTACTGTAACACAACACAATTGTTTAATAGTACTTGGAATGATACTAGAGAGTCAAATAATAATGTA----
-------------------------------------------------------------------------
----------------------------
GGAAATGACACAATCACACTCCCATGTAGAATAAAACAAATTATAAACATGTGGCAGGAAGTAGGAAAAGCAA
TGTATGCCCCTCCCATCAGAGGACAAATTAGATGCTCATCAAAT---
ATTACAGGGCTGATATTAACAAGAGATGGTGGTAAAGAAGGGAAC----------------------------
-------------------------------------------------------------------------
----------
```

GGGACGACCGAGACCTTCAGACCAGGAGGAGGAGATATGAGGGACAATTGGAGAAGTGAATTATATAAATATA
AAGTAGTAAAAATTGAACCTCTA---GGAGTAGCACCCACCAAGGCAAGGAGAAGAGTGGTG-----------
----------------------------CAGAGAGAAAAAAGAGCA---GCGGGA---CTAGGAGCT------
---TTGTTCCTTGGGTTCCTGGGAGCG---GCAGGAAGCACTATG---GGCGCAGCGTCA---
GTGACGCTGACGGTACAG---GCCAGACAA---TTACTG---TCTGGTATAGTGCAACAGCAAAAC---
AATTTG---CTGATG---GCTATTGAGGCGCAACAGCATCTGTTGAAACTCACAGTCTGGGGCATC
>EF514712
TTCTACTGTAATACAACACAACTGTTTAGTAGTACTTGGAATGAGAGTACTTGGAATGAGAGTACATGGAATA
ATACAGGGAAT--
----------ACTACCCCT------
GAAAGAAATGAAACTATCACACTCTCATGCAGAATAAAACAAATTATAAACATGTGGCAGGAAGTAGGAAAAG
CAATGTATGCCCCGCCCATAGAAGGACACATTAACTGTTCATCAAAT---
ATTACAGGGCTGCTATTAGTAAGAGATGGGGGTAACAGGAGCAAGAGCGAG----------------------
----

AATAACGGGAACGAGACCTTCAGACCTGGAGGAGGAGATATGAGGGATAATTGGAGAAGTGAATTATATAAAT
ATAAAGTAGTAAAAATTGAACCATTA---GGAATAGCACCCACCAAGGCAAAGAGAAGAGTGGTG--------
-------------------------------CAGAGAGAAAAAAGAGGA---GTA------ATAGGAGCT---
------ATGTTCCTTGGGTTCTTGGGAGCA---GCAGGAAGCACTATG---GGCGCAGCGTCA---
ATGACGCTGACGGTACAG---GCCAAACAA---TTATTG---TCTGGCATAGTGCAACAGCAGAAC---
AATTTG---CTGAAG---GCTATTGAGGCGCAACAGCATCTGTTGCAACTCACAGTCTGGGGCATC
>EF514697
TTCTACTGTAATACAGCAAAGCTGTTTAATAGTACTCTGAATAATACTGAATGGTCAAATATCACTGCAGAAA
AT---

AATGAAGACAAAATCATACTCCAATGCAGAATAAAACAAATTATAAACATGTGGCAGGAAGTAGGAAAAGCAA
TGTATGCCCCTCCCATTAGCGGACCAATTAACTGTTCATCAAGT---
ATTACAGGGCTGATATTAACAAGAGATGGTGGTAATATTAATAATAATAGC----------------------
----

ATGAGAGATAATGACACCTTCAGACCTGGAGGAGGAGATATGAGGGATAATTGGAGAAGTGAATTATATAAAT
ATAAAGTAGTAAGAATTGAACCATTA---GGAGTAGCACCCACCAAGGCAAAGAGAAGAGTGGTG--------
-------------------------------CAGAGAGAAAAAAGAGCA---GTGGGA---ATAGGAGCT---
------GTACTCCTTGGGTTCTTGGGAGCA---GCAGGAAGCACTATG---GGCGCAGCATCA---
GTGATGCTGACGGTACAA---GCCAGGCAA---TTATTG---TCTGGTATAGTGCAACAGCAGAAC---
AATCTG---CTGAGA---GCTATTGAGGCGCAACAACATCTGTTGCAACTCACAGTCTGGGGCATC
>EF514701
TTCTACTGTAATTCATCACAACTGTTTAATAGTACTTGGAACAGTAACAGTACCTTAACTTTAACTGGTCCTG
AAGAGTCA---
-------------------AAC---
AGCACTGATAATAATATCACACTCCCATGCAGAATAAAGCAGTTTATAAACCTGTGGCAGGGAGTAGGAAAAG
CAATGTATGCCCCTCCCATCCAAGGACGAATTAAATGTACATCAAAT---
ATTACAGGGCTGATATTAACAAGAGATGGTGGGAATAGCAGCAAC----------------------------
----

ACCACCACCGAAATCTTCAGACCTGGAGGAGGAGATATGAGGGACAATTGGAGAAGTGAATTATATAAATATA
AAGTAGTAAAAATTGAACCATTA---GGAGTAGCACCCACCAAGGCAAAGAGAAGAGTGGTG-----------
----------------------------CAGAGAGAAAAAAGAGCA---GTGGGG---CTAGGAGCT------
---CTGTTCCTTGGGTTCTTGGGAGCA---GCGGGAAGCACTATG---GGCGCAGCGTCA---
GTGACGCTGACGGTACAG---GCCAGACAA---TTGTTG---TCTGGTATAGTGCAACAGCAAAAC---
AACTTG---CTGATG---GCTATTGAGGCGCAACAGCATCTGTTGCAACTCACAGTCTGGGGCATT
>FJ694790
TTCTACTGTAATACGACACGACTGTTCAATAGTATGTTCAATGGTACTTTGAAGAATACTACAGGGTCAAATA
ACACA--

GGGAATAATGACACAATCATACTCCCATGCAGAATAAAACAAATTATAAACATGTGGCAGGAAGTAGGAAAAG
CAATGTATGCCCCTCCCATCGAAGGACTAATTAACTGTGTGTCAAAT---
ATTACAGGGCTGATATTAACAAGAGATGGGGGTAAGAGTAGTAAC----------------------------
----

GAAAACGAGACCTTCAGACCTATAGGAGGAAATATGAGGGACAACTGGAGAAGTGAATTATATAAATATAAAG
TAGTAAGAATTGAACCATTA---GGAGTAGCACCCACTGCGGCAAAGAGAAGAGTGGTG--------------
-------------------------CAGAGAGAAAAAAGAGCA---GTGGGA---ATAGGAGCT---------
GTGTTCCTTGGGTTCTTGGGAGCA---GCAGGAAGCACTATG---GGCGCAGCGTCA---
ATGACGCTGACGGTACAG---GCCAGACAA---TTATTG---TCTGGTATAGTGCAACAGCAGAAT---
AATCTG---CTGAGA---GCTATTGAGGCGCAACAGCATCTGTTGCAACTCACAGTCTGGGGCATC
>EU839597
TTCTACTGTAATGCAACAGAGCTGTTTAATAGTACTTGGAATGATACTACTGGGAGTGTTACTGAA-------
----

GGTAATGACACAATCACACTTCCATGCAGAATAAAACAAATTATAAACATGTGGCAAGAAGTAGGAAAAGCAA
TGTATGCCCCTCCCATCAGGGGATATATTAACTGTTCATCAAAC---
ATTACAGGGCTGCTATTAATAAGAGATGGGGGTATTAATAATACA----------------------------
----

GGCAATGAGACCTTTAGACCTGGAGGGGGAAATATGAAGGACAATTGGAGAAGTGAATTATATAAATATAAAG
TAGTAAGAATTGAACCATTA---GGAGTAGCACCCACCAAGGCAAAGAGAAGAGTGGTG--------------
-------------------------CAGAGAGAAAAAAGAGCA---GTGGGA---ATAGGAGCT---------
GTGTTCCTTGGGTTCTTGGGAGCA---GCAGGAAGCACTATG---GGCGCAGCGTCA---
ATGACGCTGACGGTACAG---GCCAGACAA---TTATTG---TCTGGTATAGTGCAACAGCAAAAC---
AATTTG---CTGAGA---GCTATTGAGGCGCAACAGCATCTGTTGCAACTCACAGTCTGGGGCATC
>EU839596
TTCTACTGTAATTCAACACAACTGTTTAATAGTACTTGGCTTGCTAATAGTACTTGGAATGATAATGATACTA
GT---

ACTGATAATAGTACTATCACACTCCCCTGTAGAATAAAACAAATTATAAACATGTGGCAGGAAGTAGGCAAAG
CAATGTATGCCCCTCCCATCAGTGGACAAATTAACTGTTCATCAAAT---
ATCACAGGGCTACTATTAACAAGAGATGGTGGTAATGAAAGTGAG----------------------------
----

TTCGAGATCTTCAGACCTGGAGGAGGAAATATGAAGGACAATTGGAGAAGTGAATTGTATAAATATAAAGTAG
TAAAAATTGAACCATTA---GGAGTAGCACCCACCAGGGCAAGGAGAAGAGTGGTG-----------------
----------------------CAGAGAGAAAAAAGAGCA---GTGGGA---ATAGGAGCT---------
GTGTTCCTTGGGTTCTTAGGAGCA---GCAGGAAGCACTATG---GGCGCAGCGTCC---
ATAACGCTGACGGTACAA---GCCAGACAA---TTATTG---TCTGGTATAGTGCAGCAGCAAAAC---
AATTTG---CTGAGG---GCTATTGAGGCGCAACAACATTTGTTGCAACTCACAGTCTGGGGCATC
>FJ817365
TTCTACTGTAATACAACACAACTGTTTAATAGTAGTTGGAATGTAAATAAAACTGTAAATGACACT-------
----

GAAAAGGAAAATATCACACTCCCATGTAGAATAAAACAAATTATAAACCTGTGGCAGGAAGTAGGAAAAGCAA
TGTATGCCCCTCCCATCAGTGGACAAATTAGATGTTCATCAAAT---
ATTACAGGGTTACTACTAATGAGAGATGGTGGTAAAGAGGAAAAC----------------------------
----

GGGACCATCATCTTCAGACCTGGAGGAGGAAATATGAGGGACAATTGGAGAAGTGAATTATACAAATATAAAG
TAGTAAAACTTGAACCATTA---GGAATAGCACCCACCAGGGCAAAGAGAAGAGCGGTG--------------
-------------------------CAGAGAGAAAAAAGAGCA---GTGGGC---ATAGGAGCT---------
CTGTTCCTTGGGTTCTTGGGAGCA---GCAGGAAGCACTATG---GGCGCAGCGTCG---
ATGACGCTGACGGTACAG---GCCAGACAA---TTATTG---TCTGGTATAGTGCAACAGCAAAAC---
AATCTG---CTGAGG---GCTATTGAGGCGCAACAACATCTGTTGCAACTCACAGTCTGGGGCATC
>KY658702
TTCTACTGTAATACAACACCATTGTTTAATAGTACTTGGAATAGTACTACTTGGAATAGTAATAATACTGCAA
ATGAA--

AATGATACAACCATCACACTGCAATGTAGAATAAAACAAATCATAAACATGTGGCAGGAAGTAGGAAAAGCAA
TGTATGCTCCTCCCATCAGGGGAAATATTACATGTATGTCAAAT---
ATTACAGGAGTATTATTAATAAGAGATGGTGGCAATACAAGTGAGGCA-------------------------
----

AACAGCACTGAGATCTTCAGACCAGGAGGAGGAGATATGAGGGACAATTGGAGAAGTGAATTATATAAATATA
AGGTAGTAAAAATTGAACCATTA---GGTATAGCACCCACCAAGGCAAGAAGAAGAGTGGTG-----------
----------------------------CAGAGAGAAAGAAGAGCA---GTGGGAACACTGGGAGCT------
---CTGTTCCTTGGGTTCTTGGGAGCA---GCAGGAAGCACTATG---GGCGCAGCGTCA---
GTGGCGCTGACGGTACAG---GCCAGACAA---TTATTG---TCTGGTATAGTGCAACAGCAGAAC---
AATTTG---CTGAGA---GCTATTGAGGCGCAACAGCATATGTTGCAGCTCACGGTCTGGGGCATC
>AY173960
TTCTACTGTGATACAACAAACCTGTTTAATAGTACTTGGAATGAAACACAACTATTTAATAGTACTTGGAATA
AT---

ACTGGGAATGAAAATATCACACTTCCATGCAGAATAAAACAAATTATAAACAGGTGGCAGGAAGTAGGAAGAG
CAATGTATGCCCCTCCCATCCAAGGACAAATAAACTGCTCCTCAAAT---
ATTACAGGGCTGCTATTGACAAGAGATGGTGATAATAGTACAAAT----------------------------
----

AATGAAACCTTCAGACCTATAGGAGGAGATATGAAGGATAATTGGAGAAGTGAATTATATAAATATAAAGTAG
TACAAATTGAGCCATTA---GGACTAGCACCCACCAAGGCAAGGAGAAGAGTGGTG-----------------
----------------------CAAAGAGAAAAAAGAGCA---GTGGGAACATTAGGAGCT---------
ATGTTCCTTGGGTTCTTGGGAGCA---GCAGGAAGCACTATG---GGCGCAGCGTCA---
ATGGCGCTGACGGTACAG---GCCAGACAA---TTACTG---TCTGGTATAGTGCAACAGCAAAAC---
AATTTG---CTGAGA---GCTATTGAGGCGCAGCAGCATATGTTGCAACTCACAGTCTGGGGCATT
>KC595150
TTCTACTGTAATTCATCACAACTGTTTAATAGTACTTGGAATAATAATACTAAAGGGTTAAATAACACTGAA-
----

GGAAATGGCACAATCACACTCCCATGCAGAATAAAACAAATTATAAACATGCGGCAGGAAGTAGGAAAAGCAA
TGTATGCCCCTCCTATCAGAGGACAAATTAGATGTTCATCAAAT---
ATTACAGGGCTAATATTAACAAGAGATGGTGGTAATAACACGAAC----------------------------
----

GGGTCCGAGATCTTCAGACCTGGAGGAGGAAATATGAGGGACAATTGGAGAAGTGAATTATATAAATATAAAG
TAGTAGAAATTGAACCATTA---GGAGTAGCACCCACCAAGGCAAAGAGAAGAGTGGTG--------------
-------------------------CAGAGAGAAAAAAGAGCA---GTGGGA---ATAGGAGCT---------
ATGTTCCTTGGGTTCTTGGGAGCA---GCAGGAAGCACTATG---GGCGCAGCGTCA---
ATGACGCTGACGGTACAG---GCCAGACTG---TTATTG---TCTGGTATAGTGCAACAGCAGAAT---
AATTTG---CTGAGG---GCTATTGAGGCGCAACAGCATCTGTTGCAACTCACAGTCTGGGGCATC
>KC595152
TTCTACTGTAATTCAACACAACTGTTTAATAGTACTTGGAATAATACTCGGAATAATACTAAAGGG-------
----

TCAAATAACACTATCACACTCCCATGTAGAATAAAACAAATTGTAAACATGTGGCAGGAGGTAGGAAAAGCAA
TGTATGCCCCTCCCATCAGAGGACAAATTAGATGTTCATCAAAT---
ATTACAGGGCTGCTATTAACAAGAGATGGTGGTAAAAACGATAAC----------------------------
----

GAGACTGAGATCTTCAGGCCTGGAGGAGGAGATATGAGGGACAATTGGAGAAGTGAATTATATAAATATAAAG
TAGTAAAAATTGAACCATTA---GGAGTAGCACCCACCAGGGCAAAGAGAAGAGTGGTG--------------
-------------------------CAGAGAGAAAAAAGAGCA---GTGGGA---ATAGGAGCT---------
GTGTTCCTTGGGTTCTTGGGAGCA---GCAGGAAGCACTATG---GGCGCAGCGTCA---
ATGACGCTGACGGTACAG---GCCAGACAA---TTATTG---TCTGGTATAGTGCAACAGCAGAAC---
AATCTG---CTGAGG---GCTATTGAGGCGCAACAGCATCTGTTGCAACTCACAGTCTGGGGCATC
>KC595154
TTCTACTGTAATACATCACAACTGTTTAATAGTATTTGGAATAATAATAATAGTACCTGGAATAGGACGGGGG
AGGGGTCA---
-------------------AAC---
AACACTGAAGAAATTATTATGCTCCCATGCAGAATAAAGCAAATTATAAACATGTGGCAGGAAGTAGGAAAAG
CAATGTATGCCCCTCCCATCCGTGGAATAATTAGATGCTCATCAAAT---
ATTACAGGGCTACTATTAACAGGAGATGGTGGTAATAACACTGACACT-------------------------
----

AACGCCAACGAGACTTTCAGACCTGGGGGAGGAGATATGAGGGACAATTGGAGAAGTGAATTATATAAATATA
AAGTAGTAAAAATTGAACCATTA---GGAGCAGCACCCACCAAGGAAAAGAGAAGAGTGGTG-----------
----------------------------CAGAGAGAAAAAAGAGCA---GGGGGG---ATAGTCTTT------
---TTGTTCCTTGGGTTCTTGGGAGCA---ACAGGAAGCACTATG---CCCGCAACGTCA---
TTGACGTTGACGGTACAG---GCCAGACTA---TTATTG---TCTGGTATAGTGCAACAGCAGAAC---
AACCTG---CTGAGG---GCTATTGAGGCGCAACAGCATCTATTGCAACTCACAGTCTGGGGCATC
>KC595157
TTCTACTGTAACTCAACACCGTTGTTTAATAGTACTTGGAGTAATACTACCGAGTCAAATAGC----------
----

ACAGCCAATAATATCACACTCCAATGCAAAATAAAACAAATTATAAACCTGTGGCAAGAAGTAGGAAAAGCAA
TGTATGCCCCTCCCATCAGAGGACAGATTAGATGTTCATCAAAT---
ATTACAGGACTGTTATTAACAAGAGATGGTGGTGACAAGAATGAC----------------------------
----

ACCGAGGTCTTCAGACCGGGAGGAGGAAATATGAAGGACAATTGGAGAAGTGAATTATATAAATATAAAGTAG
TAAAAATTGAACCATTA---GGAGTAGCACCCACCAAGGCAAAGAGAAGAACGGTG-----------------
----------------------CAGAGAGAAAAAAGAGCA---GTA------ATAGGAGCA---------
ATGTTCCTTGGGTTCTTGGGAGCA---GCAGGAAGCACTATG---GGCGCAGCGTCA---
GTGACGTTGACGGTACAG---GCCAGACTA---TTATTG---TCTGGTATAGTGCAACAGCAGAGC---
AATCTG---CTGAGG---GCTATTGAGGCGCAACAGCATCTGTTGCAACTCACAGTCTGGGGCATC
>KC595160
TTCTACTGTAATTCAACACAACTGTTTAATAGTACTTGGAATAATAATAGTACTTGGAAGACTTGGGAGATTG
GAGGG--

CCAAATGACACACTCACACTCCCATGCAGAATAAAACAAATTATAAACATGTGGCAGGAAGTAGGAAAAGCAA
TGTATGCCCCGCCCATCAGAGGACAAATTAGATGCTCATCAACT---
ATTACAGGGTTGCTATTAACAAGAGATGGAGGTAGTAACACTAAC----------------------------
----

GAGACCGAGATTTTCAGACCTGGAGGAGGAGATATGAGGGACAATTGGAGAAGTGAATTGTATAAATATAAAG
TAGTAAAAATTGAACCATTA---GGAGTAGCACCCACCAGGGCAAAGAGAAGAGTGGTG--------------
-------------------------CAGAGAGAAAAAAGAGCA---GCA------ATAGGAGCT---------
TTGTTCCTTGGGTTCTTGGGAGCA---GCAGGAAGCACTATG---GGCGCAGCGTCA---
ATGACGCTGACGGTACAG---GCCAGACTA---TTATTG---TCTGGTATAGTGCAACAGCAGAAC---
AATTTG---CTGAGG---GCTATTGAGGCGCAACAGCATCTGTTGCAACTCACAGTCTGGGGCATC
>KC595161
TTCTACTGTAATACAACACAACTGTTTAATAGTACTTGGAGTAATACTGGAGCGTCAAATGGCACTGAA----
----

GGAAATGACACAATCACACTCCCATGCAAGATAAGACAAATTATAAACATGTGGCAGGAAGTAGGAAAAGCAA
TGTATGCTCCTCCCATCAGAGGACAAATTAGATGTTCATCAAAT---
ATTACAGGGCTGCTATTAACAAGAGATGGTGGTAATAACACGAAC----------------------------
----

GGGACCGAGATCTTCAGACCTGGGGGAGGAGATATGAGGGACAATTGGAGAAGTGAATTATATAAATATAAAG
TAGTAAAAATTGAACCATTA---GGAGTAGCACCCACCAAGGCAAAGAGAAGAGTGGTG--------------
-------------------------CAGAGAGAAAAAAGAGCG------GGA---ATAGGAGCT---------
TTGTTCCTTGGGTTCTTGGGAGCA---GCAGGAAGCACTATG---GGCGCAGCGTCA---
ATAACGCTGACGGTACAG---GCCAGACTA---TTATTG---TCTGGTATAGTGCAACAGCAGAAT---
AATTTG---CTGAGG---GCTATTGAGGCGCAACAGCACCTGTTGCAACTCACAGTCTGGGGCATC
>KC595162
TTCTACTGTAATTCAACACAACTGTTTAATAGTACTTGGAATGCTAATAGTACTTGGAATGGTACTGAAGAGC
CAAGTAAC---

TTTGCAGAAAATATCACACTCCCATGCAAAATAAAACAAATTATAAACATGTGGCAGGAAGTAGGAAAAGCAA
TGTATGCCCCTCCCATCAGAGGACAAATTAGATGCTCATCAAAT---
ATTACAGGGTTGCTATTAACAAGAGATGGTGGTAATAACAAAAGC----------------------------
----

GAGGCCACCGAGACCTTCAGACCTGGAGGAGGAGATATGAGGGACAATTGGAGAAGTGAATTATATAAATATA
AAGTAGTAAAAATTGAACCATTA---GGAGTAGCACCCACCAGGGCAAAGAGAAGAGTGGTG-----------
----------------------------CAGAGAGAAAAAAGAGCA---GTGGGA---ATAGGAGCG------
---TTGTTCCTTGGGTTCTTGGGAGCA---GCAGGAAGCACTATG---GGCGCAGCGTCA---
ATGACGTTGACGGTACAG---GCCAGACTA---TTATTG---TCTGGTATAGTGCAACAGCAGAAC---
AATTTG---CTGAGG---GCTATTGAGGCGCAACAGCATCTGTTGCAACTCACAGTCTGGGGCATC
>KC595163
TTCTACTGTGATACAACACAACTGTTTAATAGTACTTGGAATGATACTACAAATGACACTGGCAAAAAT----
----

GACAATGACACAATCATTCTCCAATGCAGAATAAAACAAATTATAAACATGTGGCAGGAAGTAGGAAAAGCAA
TATATGCCCCTCCCATCCAAGGACAAATTAACTGTTCATCAAAT---
ATTACAGGGCTGCTACTAACAAGAGATGGTGGTAATACTAATAACACG-------------------------
----

AACGGGACTGAGACCTTCAGACCTGGAGGAGGAGATATGAGGGACAATTGGAGAAGTGAATTATATAAATATA
AAGTAGTAAAAATTGAACCATTA---GGAGTAGCACCCACCAAGGCAAAGAGAAGAGTGGTG-----------
----------------------------CAGAGAGAAAAAAGAGCA---GTGGGA---ATAGGAGCT------
---GTGTTCCTTGGGTTCTTGGGAGCA---GCAGGGAGCACTATG---GGCGCAGCGTCA---
ATGACGCTGACGGTACAG---GCCAGACAA---TTATTG---TCTGGTATAGTGCAACAGCAGAAT---
AATTTG---CTGAGA---GCTATTGAGGCGCAACAACATATGTTGCAACTCACAGTCTGGGGCATC
>KC595165
TTCTACTGTAATTCAACACAATTGTTTAATAGTACTTGGAACCGGACTGGTACTGAGGGGTTAAATACCACTG
AA---

GGAAATGGCACATTCACACTTCCATGTAGAATAAAACAATTTATAAACTTGTGGCAGGGAGTAGGAAAAGCAA
TGTATGCCCCTCCCATCAGGGGACAAATTAGATGTTCATCAAAC---
ATTACAGGGCTACTATTAACAAGAGATGGTGGTAATAAAATGAAC----------------------------
----

GAGACAAGCGAGACCGAGACCTTCAGACCTGGAGGAGGAAATATGAGGGACAATTGGAGAAGTGAATTATATA
AATATAAAGTAGTAAGAATTGAACCATTA---GGAATAGCACCCACCAAGGCAAAGAGAAGAGTGGTG-----
----------------------------------CAGAGAGAAAAAAGAGCA---GTGGGA---
ATAGGAGCT---------GTGTTCCTTGGGTTCTTGGGAGCA---GCAGGAAGCACTATG---
GGCGCAGCGTCA---ATGACGCTGACGGTACAG---GCTAGACTA---TTATTG---
TCTGGTATAGTGCAACAGCAGAAT---AATTTG---CTGAGA---
GCTATTGAGGCGCAACAACATCTGTTGCAACTCACAGTCTGGGGCATC
>KC595197
TTCTACTGCAATACAACACAACTGTTTAATAGTACTTGGAATAGTACTACTAGGAATGAC-------------
----

ACTGAAGGAAATATTACACTCCCATGCAGAATAAAACAAATTATAAACATGTGGCAGGAAGTAGGAAAAGCAA
TGTATGCCCCTCCCATCAGTGGACAAATTAGTTGTTCGTCAAAT---
ATTACAGGGATACTATTAACAAGAGATGGGGGTAATACGAACGGT----------------------------
----

AATAATACTGAAACCTTCAGACCTGGAGGAGGAGATATGAGGGACAATTGGAGAAGTGAATTATATAAATATA
AAGTAGTAAAAATTGAACCATTA---GGAATAGCACCCACCAGGGCAAAGAGAAGAGTGGTG-----------
----------------------------CAGAAAGAAAAAAGAGCA---GTGGGG---ATAGGAGCT------
---GTGTTCCTTGGGTTCTTGGGAGCA---GCAGGAAGCACTATG---GGCGCAGCATCA---
ATGACGCTGACGGTACAG---GCCAGACTA---TTATTG---TCTGGTATAGTGCAACAGCAAAAC---
AATTTG---CTGAGA---GCTATTGAGGCGCAACAGCATCTGTTGCAACTCACAGTCTGGGGCATC
>KC595202
TTCTACTGTAATACAACACAACTGTTTAATAGTACTTGGAATGCTACTGAAGAGACAAATAAC----------
----

ACTGAAGGCACAATCACACTTCCATGCAGAATAAAACAAATTATAAACATGTGGCAGGAAGTAGGAAAAGCAA
TGTATGCCCCTCCCATCAGAGGAAATATTAGCTGCTCATCAAAT---
ATTACAGGGGTGATATTAACAAGAGATGGTGGCACAGACAATAGC----------------------------
----

GAGACCGAGACCTTTAGACCGGGAGGAGGAGATATGAAGGACAATTGGAGAAGTGAATTGTATAAATATAAAG
TAGTAAAAATTGAGCCATTA---GGAATAGCACCCACCAAGGCAAAAAGAAGAGTGGTG--------------
-------------------------CAGAGAGAAAAAAGAGCA---GTGGGA---ATAGGAGCT---------
GTGTTCCTTGGGTTCTTGGGAGCA---GCAGGAAGCACTATG---GGCGCAGCGTCA---
ATGACGTTGACGGTACAG---GCCAGACTA---TTATTG---TCTGGTATAGTGCAACAGCAGAAC---
AATTTG---CTGAGG---GCTATTGAGGCGCAACAGCATCTGTTGAAACTCACAGTCTGGGGCATC
>KC595203
TTCTACTGTAATTCAACACAACTGTTTAATAGTACCTGGGATAATAGTAGTATTGAGAATAATACAGAGTCA-
----

GGAAATGAAACTATCACACTCCCATGCAGGATAAAACAAATTATAAACTTGTGGCAGGAAGTAGGAAAAGCAA
TGTATGCCCCTCCCATCAGAGGGCCAATTAATTGCTCATCAGAT---
ATTACAGGGCTGCTATTAACAAGAGATGGTGGTACTAATGGCCCT----------------------------
----

AACGATACTGAGACCTTCAGACCTGGAGGAGGAAACATGAAGGACAATTGGAGAAGTGAATTATATAAATATA
AAGTAGTAAGAATTGAACCACTA---GGAATAGCACCCACCAATGCAAGGAGAAGAGTGGTG-----------
----------------------------CAGAGAGAAAAAAGAGCA---GTGGGG---CTAGGAGCC------
---TTGTTCCTTGGGTTCTTGGGAGCA---GCAGGAAGCACTATG---GGCGCAACGTCA---
ATGGCGTTGACGGTACAG---GCCAGACAA---TTATTG---TCTGGTATAGTGCAACAGCAAAAC---
AATTTG---CTGAGG---GCCATTGAGGCGCAACAGCAACTGTTGCAACTCACAGTTTGGGGCATC
>KC595158
TTCTACTGTAATACAACACAACTGTTTAATAGTACTTGGGGTAATAATAGTACTTGGAGTAATACTACCGAGT
CAGGTGGC--

AATGGAACAAATATCATACTTCCATGCAGAATAAAACAAATTATAAACATGTGGCAGGAAGTAGGAAAAGCAA
TGTATGCCCCTCCCATCAGAGGACAAATTAGCTGCTCATCAAAT---
ATTACAGGGCTGCTATTAACAAGAGATGGTGGTAACAGTAACACG----------------------------
----

ACTGAGACCTTCAGACCTGGAGGAGGAAATATGAAGGACAATTGGAGAAGTGAATTATATAAATATAAAGTAG
TAAAAATTGAACCATTA---GGAGTAGCACCCACCAGGGCAAAGAGAAGAGTGGTG-----------------
----------------------CAAAGAGAAAAAAGAGCA---GTGGGA---ATAGGAGCT---------
GTGTTCCTTGGGTTCTTGGGAGCA---GCAGGAAGCACTATG---GGCGCAGCGTCC---
ATGACGTTGACGGTACAG---GCCAGACTA---TTATTG---TCTGGTATAGTGCAACAGCAGAAC---
AATTTG---CTGAGA---GCTATTGAGGCACAACAGCATCTGTTGCAACTCACAGTCTGGGGCATC
>KC595168
TTTTACTGTAATTCAACACAACTGTTTAATAGTACTTGGGAGTTTAATAGTACTTGGAATAGTACTTGGAATG
AT--

ACTGGAGAAAAGATCACACTCCCATGCAGAATAAAACAAATTATAAACATGTGGCAGGAAGTAGGAAAAGCAA
TGTATGCCCCTCCCATCAGAGGACAAATTAGATGCTCATCAAAT---
ATTACAGGACTGCTATTAACAAGAGATGGTGGCAATAACAAGAGC----------------------------
----

GACACTGAAACCTTCAGACCTGGAGGAGGAGACATGAGGGACAATTGGAGAAGTGAATTATATAAATATAAAG
TAGTAAAAATTAAACCATTA---GGAGTAGCACCCACCAAGGCAAAAAGAAGAGTGGTG--------------
-------------------------CAGAGAGAAAAAAGAGCA---GTGGGA---ATAGGAGCT---------
ATATTCCTTGGGTTCTTGGGAGCA---GCCGGAAGCACTATG---GGCGCAGCGTCA---
ATGACGTTGACGGTACCG---GCCAGACAA---TTATTG---TCTGGTATAGTGCAACAGCAGAAC---
AATTTG---CTGAGG---GCTATTGAGGCGCAACAACATCTGTTGCAACTCACAGTCTGGGGCATT
>KC595190
TTTTACTGTAATACAACGGGACTGTTTAATAGTACTTGGAGTTGGAATGATACTAACACAAATGACACAAGAG
AA--

AATAACACACATATCATACTCCCATGCAGAATAAAACAAATTATAAACATGTGGCAGGGAGTAGGAAAAGCAA
TGTATGCCCCGCCCATCAGAGGACAAATTAGATGTTCATCAAAT---
ATTACCGGGTTGCTATTAACAAGAGATGGTGGTGGTAACGGGAAC----------------------------
----

AATACCACCGAGGTCTTCAGACCTGGAGGAGGAGATATGAAGGACAATTGGAGAAGTGAATTATATAAATATA
AAGTAGTAAAAATTGAACCATTA---GGAGTAGCACCCACCAAGGCAAAGAGAAGAGTGGTG----------
----------------------------CAGAGAGAAAAAAGAGCA---ATAGGA---ATAGGAGCT-----
---ATGTTCCTTGGGTTCTTGGGAGCA---GCAGGAAGCACTATG---GGCGCAGCATCA---
ATAACGCTGACGGTACAG---GCCAGACAA---TTGTTG---TCTGGTATAGTGCAACAGCAGAAC---
AATTTG---CTGAGG---GCTATTGAGGCGCAACAACATCTGTTGCAACTCACAGTCTGGGGCATC
>KT200351
TTCTACTGTGATACATCAAAATTGTTTAATAGTACTTGGTCTAATAATGATAGTGTTATTAATAGCACTGAAG
TA--

AATAACACAGTCATCACACTCCCATGTAGAATAAAACAAATTATAAACATGTGGCAGGAAGTAGGAAAAGCAA
TGTATGCCCCGCCCATCAGAGGACAAATTAACTGTTCATCAAAT---
ATTACAGGGCTGTTATTAACAAGAGATGGTGGTAACAATGCCACC---------------------------
----

AATGAGACTTTCAGACCGGCAGGAGGAGATATGAGGGACAATTGGAGAAGTGAATTATATAAATACAAAGTAG
TAAGAATTGAACCACTA---GGAATAGCACCCACCAAGGCAAAGAGAAGAGTGGTG----------------
----------------------CAAAGAGAAAAAAGAGCA---GTGGGA---ATAGGAGCT--------
GTGTTCCTTGGGTTTCTGGGAGCA---GCAGGAAGCACGATG---GGCGCAGCGTCA---
TTGACGCTGACGGTACAG---GCCAGACTA---TTATTG---TCTGGTATAGTGCAGCAGCAGAAC---
AATCTG---CTGAGG---GCTATTGAGGCGCAACAGCATCTGTTGCAACTCACAGTCTGGGGCATC
>EU786676
TTTTACTGTAATACATCACTACTGTTTAATAGTACCTGGAATGGTACTGGAGGGACAGATACTGAA------
----

GGAAATGAAACAATCACACTCCCATGCAGAATAAAACAAATTATAAACATGTGGCAAGAAGTAGGAAAAGCAA
TGTATGCCCCTCCCATCAGAGGACTAATTAGATGCTCATCAAAC---
ATTACAGGGTTGCTATTAACAAGAGATGGTGGGAAAGAGAGTAAT---------------------------
----

AGCACTACAGAAACCTTCAGACCTGGAGGAGGAGATATGAGGGACAATTGGAGAAATGAATTATACAAATATA
AAGTAGTAAAAATTGAACCATTA---GGAGTAGCACCCACCAAGGCAAAGAGAAGAGTGGTG----------
----------------------------CAGAGAGAAAAAAGAGCA---GTGGGA---ATAGGAGCT-----
---TTGTTCCTTGGGTTCTTGGGAGCA---GCAGGAAGCACTATG---GGCGCAGCGTCG---
ATGACGTTGACGGTACAA---GCCAGACAA---TTATTG---TCTGGTATAGTGCAACAGCAGAAC---
AACTTG---CTGAGG---GCTATTGAGGCGCAACAGCATCTGTTGCAACTCACAGTCTGGGGCATC
>KC595196
TTCTACTGTAATTCAACACAACTGTTTAATAGTACTTGGAACGATACTGGAGGATCAAATAATACTGGAGAAT
CAAAT---

AGCACTGGAACAATCACACTCCCATGCAGAATAAGACAAGTTGTAAACATGTGGCAGGAAGTAGGAAAAGCAA
TGTACGCCCCTCCCATCAAAGGACAAATTAGATGTTCATCAAAT---
ATTACGGGGCTGCTCTTAACAAGAGATGGTGGTGATAACGGGACC---------------------------
----

ACCGAGATTTTCAGACCTGGGGGAGGAGATATGAGGGACAATTGGAGAAGTGAATTATATAAATATAAGGTAG
TAAAAATTGAGCCATTG---GGAGTGGCACCCACCAAGGCAAAGAGAAGGGTGGTG----------------
----------------------CAGAGAGAAAAAAGAGCA---ATGGGA---ATAGGAGCT--------
CTGTTCCTTGGGTTCTTGGGAGCA---GCAGGAAGCACTATG---GGCGCAGCGTCA---
ATGACGTTGACGGTACAG---GCCAGACTA---TTGTTG---TCTGGTATAGTGCAACAGCAGAAC---
AATTTG---CTGAGG---GCTATTGAGGCGCAACAGCATCTATTGCAACTCACAGTCTGGGGCATC
>EU786678
TTCTACTGTAATACAACAGCACTGTTTAATAGTACCTGGAATACTAATGGTACTTGGAAGGGTACAACAGGA-
----

GAAAATGACACCATCACACTCCAATGCAGAATAAAACAAATCATAAACATGTGGCAGGAAGTAGGAAAAGCAA
TGTATGCTCCTCCCATCAGAGGACAAATTAGATGCACATCAAAT---
ATTACAGGAATTCTATTAACAAGAGATGGTGGTAAAAGCAATAACAAT------------------------
----

AGCAATGGGAGCGAGATCTTCAGACCTGGAGGAGGAGATATGAGGGACAATTGGAGAAGTGAACTATATAAAT
ATAAAGTAGTAAAAATTGAACCATTA---GGAATAGCACCCTCCAAGGCAAGGAGAAGAGTGGTG--------
-------------------------------CAGAGAGAAAAAAGAGCA---GTGGGA---ATAGGAGCT---
------CTGTTCCTTGGGTTCTTGGGAGCA---GCAGGAAGCACTATG---GGCGCAGCGTCA---
ATGGCGTTGACGGTACAG---GCCAGACAA---TTATTG---TCTGGTATAGTGCAACAGCAGAAC---
AATCTG---CTGAGG---GCTATTGAGGTGCAACAGCATATGTTGCAACTCACAGTCTGGGGCATT
>EU786680
TTCTACTGTGATTCAACAAAACTGTTTAATAGTACCTGGTATTTTAATGGTACTTGGAATAATACAAATAATA
ATAAT--

GATACCCATGATCCCATCACACTCCCATGCAGAATAAAACAAATTGTAAACATGTGGCAGGAAGTAGGAAAAG
CAATGTATGCCCCTCCCATCAAGGGACAAATTAGATGCTCATCAAAT---
ATTACAGGGCTGATATTAACAAGAGATGGTGGTGTTGATAACAGC----------------------------
----

AGCACCAATGAGACCTTCAGACCTGGAGGAGGAGATATGAGGGACAATTGGAGAAGTGAATTATATAAATATA
AAGTAGTAGAACTTAAACCATTA---GGAATAGCACCCACCAGGGCAAAGAGAAGAGTGGTG-----------
----------------------------CAAAGAGAGAAAAGAGCA---GTGGGA---ATAGGAGCT------
---ATGTTCCTTGGGTTCTTGGGAGCA---GCAGGAAGCACTATG---GGCGCAGCGTCA---
ATGACGCTGACGGTACAG---GCCAGACTA---TTATTG---TCTGGTATAGTGCAACAGCAGAGC---
AATTTG---CTGAGA---GCTATTGAGGCGCAACAGCATCTGTTGCAACTCACAGTCTGGGGCATC
>HQ236609
TTCTACTGTGATTCAACACAACTGTTTAATAGTACTTGGTCGTATAATAGTACTAAGAATAGTTGGAGTAGTA
CTGAA--
-------------------GGG---
ACAAATGGCACCACCATCACACTTCCATGCAGTATAAAACAATTTGTGAGAATGTGGCAAAGAGTTGGACAAG
CAATGTATGCCCCTCCCATCGCAGGAAACATATCATGCAGGTCAAAC---
ATTACAGGGTTGATATTAACAAGAGATGGTGGCACCCCAGATGAT----------------------------
----

GGCAATAACGAAACCTTCAGACCTGGAGGTGGAGACATGAAAGACAATTGGAGAAGTGAATTATATAAATATA
AAGTAGTAAAGCTTGAACCATTA---GAAATAGCACCCACCAGGGCACAGAGAAGAGTGGTG-----------
----------------------------CAGAGAGAAAAAGAG------CGGGGA---CTAGGAGCT------
---CTGTTCCTTGGGTTCTTGGGAGCA---GCAGGAAGCACTATG---GGCGCAGCGTCA---
ATGACGTTGACGGTACAG---GCCAGACAA---CTACTG---TCTGGTATAGTGCAACAGCAGAAC---
AATCTG---CTGAGG---GCTATTGAGGCGCAACAGCATATGTTGCAACTCACAGTCTGGGGCATA
>FJ817366
TTCTACTGTAATTCATCACAACTGTTTAATAGTACTTGGTATAGTAATGGGACTAGTAATGGAGGGTTAAAT-
----

AACAGTGCCAACATTACACTTCCATGCAGAATAAAACAAATTATAAATCTGTGGCAGGAAGTAGGAAAAGCAA
TGTATGCCCCTCCCATCAAAGGAGTAATTAACTGTTTATCAAAT---
ATTACAGGGATAATATTAACAAGAGATGGTGGTGAGAACAATGGC----------------------------
----

ACCACCGAGACCTTCAGACCTGGAGGGGGAGATATGAGGGACAATTGGAGAAGTGAATTATATAAATATAAAG
TAGTAAAAATTGAACCATTA---GGAATAGCACCCACCAAGGCAAAAAGAAGAGTGGTG--------------
-------------------------CAAAGAGAGAAAAGAGCA---GTGGGA---TTAGGAGCT---------
GTGTTCCTTGGGTTCTTGGGACTA---GCCGGAAGCACTATG---GGCGCAGCGTCA---
GTGACGTTGACGGTACAG---GCCAGGCTA---TTATTG---TCTGGTATAGTGCAACAGCAGAAC---
AATTTG---CTGAGG---GCTATTGAGGCGCAGCAGCAGCTGCTGCAACTCACAGTCTGGGGCATC
>GQ372988
TTCTACTGTAATACATCAAAGCTGTTTAATAGTACGTTGTTTAGTAATAATACTTGGTTCAATAGTACTATGA
ATGAA--
-------------------ACT---
AATACAAATGACACTATCATGATTCCATGCAGGATAAAACAAATTATAAACAGGTGGCAAGAAGTAGGAAAAG
CAATGTATGCCCCTCCCATCAGTGGACTAGTTAGATGTTCATCAAAT---
ATTACAGGGCTGTTATTAACAAGAGATGGTGGTGGAGGCCAGAACCAGACT----------------------
----

GAAAACCAGACTGAGATCTTCAGACCTGGAGGAGGAGATATGAGGGACAATTGGAGAAGTGAATTATATAAGT
ATAAAGTAGTAAGAATTGAACCATTA---GGAATAGCACCCACCAAGGCAAAGAGAAGAGTGGTG--------
-------------------------------CAGAGAGAAAAAAGAGCA---GTGGGAACAATAGGAGCT---
------ATGTTCCTTGGGTTCTTGGGAGCA---GCAGGAAGCACTATG---GGCGCAGCGTCA---
GTCACGCTGACGGTACAG---GCCAGACTA---TTGTTG---TCTGGTATAGTGCAACAGCAGAAC---
AACTTG---CTGAAG---GCTATTGAGGCGCAACAACATCTGTTGCAACTCACAGTCTGGGGCATC
>FJ670531
TTCTACTGTGATTCAAAAGGACTGTTTAATAGTACTTGGAAGCTGGTTAATAGTACTTGGGAGCTGGTTAATG
GTACTTTGGAT--
----------TCAATAACA------
AATGGTACTGTCAATATCACACTCCCATGCAAAATAAAACAAATTGTAAACCGGTGGCAGGAAGTAGGGAAAG
CAATGTATGCCCCTCCCATCAAAGGGCCAATTAACTGCACATCAAAT---
ATTACAGGGCTGCTATTAACAAGAGATGGTGGTAATGACAAAAAC----------------------------
----

CAGTCCATTGAGATCTTCAGACCGGGAGGAGGAGATATGAGGGACAATTGGAGAAGTGAATTATATAAATATA
AAGTAGTAAAAATTGAACCATTA---GGAGTAGCACCCACCAAGGCAAAGAGAAGAGTGGTG-----------
----------------------------CAGAGAGAAAAAAGAGCA---GTGGGA---TTAGGAGCT------
---ATGTTCCTTGGGTTCTTGGGAGCA---GCAGGAAGCACTATG---GGCGCAGCGTCA---
GTGACGTTGACGGTACAG---GCCAGACTA---TTATTG---TCTGGTATAGTGCAACAGCAGAAC---
AATTTG---CTGAGG---GCTATTGAGGCGCAACAGCATCTGTTGCAACTCACAGTCTGGGGCATC
>FJ853620
TTCTACTGTAATACAACACAACTGTTTAATAGTACTTGGAATAGTACTATTAGGAATGACACTGAA-------
----

GCTGGAGAAAATATTACACTCACATGCAGAATAAAACAAATTATAAATCTGTGGCAGGAAGTAGGAAAAGCAA
TGTATGCCCCTCCCATCAGCGGACAAATTAGTTGTTCATCAAAT---
ATTACAGGGATACTATTAACAAGGGATGGGGGTAAAAACAGTAAT----------------------------
----

GATACTGAGATCTTCAGACCTGGAGGAGGAGATATGAGGGACAATTGGAGAAGTGAATTATATAAATATAAAG
TAGTAAAAATTGAACCATTA---GGAATAGCACCCACCAAGGCAAAGAGAAGAGTGGTG--------------
-------------------------CAAAGAGAAAAAAGAGCA---GTGGGA---ATAGGAGCT---------
GTGATCCTTGGGTTCTTGGGAGCA---GCAGGAAGCACTATG---GGCGCAGCATCA---
ATAACGCTGACGGTACAG---GCCAGGCTA---TTACTG---TCTGGTATAGTGCAACAGCAAAGC---
AATTTG---CTGAGA---GCTATTGAGGTGCAACAGCATCTGTTGCAACTCACAGTCTGGGGCATC
>KT200349
TTCTACTGTAATACATCAGAACTATTTAATAGTACTTGGCAGTTTAATAATAATAATACTGGAAATAATAGT-
----

GCTGAAAGAAATATCACAATCCCATGTAGAGTAAGACAAATTATAAACATGTGGCAGAGAGTAGGAAAAGCAA
TGTATGCCCCTCCCATCGCAGGACAAATTAGATGTGTATCAAAT---
ATTACGGGGATGCTATTAACAAGAGATGGTGGTAATGATGGTGGT----------------------------
----
AATAACAGCACGAAC---
GTGAGCGAGATCTTCAGACCGGCAGGAGGAGAGATGAGGGACAATTGGAGAAGTGAATTATATAAATACAAAG
TAGTAAAAATTGAACCATTA---GGGATAGCGCCCACCAGGGCAAAGAGAAGAGTGGTG--------------
-------------------------CAGAGAGAAAAAAGAGCA---GTGGGA---ATAGGAGCT---------
GTGTTCCTTGGGTTCTTGGGAGCA---GCAGGAAGCACTATG---GGCGCAGCATCA---
ATGATGCTGACGGTACAG---GCTAGACAA---CTATTG---TCTGGTATAGTGCAACAGCAGAAC---
AATCTG---TTGAGA---GCTATTGAGGCGCAACAACATCTGTTGCAACTCACAGTCTGGGGCATC
>KT200354
TTCTACTGTAATACAACAAAACTATTTAATAGTACTTGGCAGTTTAATAGTACTGAGAGTGGTACTGGGAAAC
TA---

AATACCACTGACATCATACTTCCATGCAGGATAAAACAAATTGTAAACATGTGGCAGGAAGTAGGAAAAGCAA
TGTATGCCCCTCCAGTCACAGGAATAATTAGTTGTTTATCAAAT---
ATTACAGGACTGCTAATAACAAGAGATGGTGGTAATGATAATAAGACC-------------------------
----

AACAACACTGAGATCTTTAGACCTGGAGGAGGAAATATGAAGGACAATTGGAGAAGCGAATTATATAAATATA
AAGTAGTAAAAATTGAACCAATA---GGAATAGCACCCACCAAGGCAAAAAGAAGAGTGGTG----------
---------------------------CAGAGAGAAAAAAGAGCAATAGTGGGA---ATAGGAGCT------
---GTGTTCCTTGGGTTCTTGGGAGCA---GCAGGAAGCACTATG---GGCGCAGCATCA---
ATAACGCTGACGGTACAA---GCCAGACAG---CTATTG---TCTGGTATAGTGCAACAGCAGAAC---
AATCTG---CTGAGG---GCTGTTGAGGCGCAACAGCATCTGTTGCAACTCACAGTCTGGGGCATC
>KT200358
TTCTACTGTAATACAACAAAGCTGTTTAATAGTACCTGGGAAACAAACAGGAGTGAAAGTGCAAAT-------
----

ACCAGTGAAACAATTATACTCCCATGTAGAATAAGACAAATTATAAACTTGTGGCAGAGAGTAGGAAAAGCAA
TGTATGCCCCTCCTATCAGAGGACAAATTAACTGCTCATCAAAT---
ATTACAGGGCTAATATTAACAAGAGATGGTGGGAATGATAATGATACT------------------------
----

AATAATACTGAGACCTTTAGACCTGGAGGAGGAGATATGAGGGACAATTGGAGAAATGAATTATATAAATATA
AAGTGGTACAAATTGAACCAATA---GGACTGGCACCCACCAAGGCAAAGAGAAGAGTGGTG----------
---------------------------CAGAGAGAAAAAAGAGCA---GTAACC---ATAGGAGCT------
---TTGTTCCTTGGGTTCTTAAGAGCA---GCAGGCAGCACTATA---GGCGCAGCGTCA---
ATGGCGCTGACGGTACAG---GCCAGACAA---TTATTG---TCTGGTATAGTGCAACAGCAGAGC---
AATCTG---CTAAGA---GCTATTGAGGCGCAACAGCATCTGTTGCAACTCACAGTCTGGGGCATC
>FJ853622
TTCTACTGTAATACAACAACACTGTTTAACAGTACTTGGTATAATAATGGAAGTTCAAATGACTCTGAAAAA-
----

AATGGCACCATCATCACACTCCCATGTAAAATAAAGCAAATTATAAACATGTGGCAGGAAGTAGGAAAAGCAA
TATATGCCCCTCCTATCGCAGGACAAATTAACTGTTCATCAAAT---
ATTACAGGGCTGCTATTAACAAGAGATGGTGGTAACAGAACTGATAGT------------------------
----

AACCAGACCGAGACATTCAGACCTGAAGGGGGAAATATGAGGGACAATTGGAGAAGTGAATTATATAAATATA
AGGTAGTAAAAATTGAACCATTA---GGAGTAGCACCCACCAAGGCAAAGAGAAGAGTGGTG----------
---------------------------CAGAGAGAAAGAAGAGCA---GTGGGAACGATGGGAGCT------
---ATGTTCCTTGGGTTCTTGGGAGCA---GCAGGAAGCACTATG---GGCGCAGCGTCA---
ATGACGCTGACGGTACAG---GCCAGGCTA---TTATTG---TCTGGTATAGTGCAACAGCAAAAC---
AACTTG---CTGAAG---GCTATTGAGGCGCAACAACATCTGTTGCAACTCACAGTCTGGGGCATC
>GU362883
TTCTACTGTAATACTACACAACTGTTTAATAGTACTTGGCTGCCTAATAGTACTTGGAATGATACTGCAAAC-
----

GACACAATCACAATCACACTCCCCTGCAGAATAAAACAAATTGTAAACATGTGGCAGGAAGTAGGAAAAGCAA
TGTATGCCCCTCCCATCAGAGGACCAATTATCTGTAAATCAAAT---
ATTACAGGGCTACTATTAACAAGAGATGGGGGTAACAAGACCAAT---------------------------
----

GACACATACGAGACCTTCAGACCTGGAGGAGGAGATATGAAGGACAATTGGAGAAGTGAATTATATAAATACA
AAGTAGTAAAAATTGAACCATTA---GGAGTAGCACCCACCAAGGCAAAGAGAAGAGTGGTG----------
---------------------------CAGAGAGAAAAAAGAGCT---GTGGGA---ATAGGAGCT------
---GTATTCTTTGGGTTCTTGGGAGCA---GCAGGAAGCACTATG---GGCGCAGCGTCA---
CTGACGCTGACGGTACAG---GCCAGACAA---TTATTG---TCTGGTATAGTGCAACAGCAGAGC---
AATCTG---CTGAGG---GCTATTGAGGCGCAACAGCATCTGTTGCAACTCACAGTCTGGGGCATC
>GU362886
TTCTACTGTAATACAACACAACTATTTAATAGTACTTGGAATGAAACTAAGAATGAAACTGAGACAGGGAAC-
----

ACAAGTGACAGTATCACACTCCCATGCAGAATAAAACAAATTATAAACCTATGGCAGGAAGTAGGAAAAGCAA
TGTATGCCCCTCCCATCAAAGGACCAATTAGCTGTTCATCAAAT---
ATTACAGGGTTACTATTAACAAGAGATGGTGGCATAGAAAGCAAC---------------------------
----

GAGACAGAGATCTTCAGACCTGGAGGAGGAGATATGAGGGACAATTGGAGAAATGAATTATATAAATATAAAG
TAGTAAAAATTGAACCATTA---GGAATAGCACCCACCAAGGCAAGGAGAAGAGTGGTG--------------
-------------------------CAGAGAGAAAAGAGAGCA---GTAACA---TTGGGAGCT---------
CTGTTCCTTGGGTTCTTGGGAGCA---GCAGGAAGCACTATG---GGCGCAGCGTCA---
ATGACGCTGACGGTACAG---GCCAGACTA---TTATTA---ACTGGTATAGTGCAACAGCAGAAC---
AATCTG---CTGAGA---GCTATTGAGGCGCAACAGCACCTGTTGCAACTCACAGTCTGGGGCATT
>JX140656
TTCTACTGTAATACAACGAGACTATTTAATAGGACTTGGAATAGTAATGATAATATTACAAATAATACAAATT
CA---

AATGACTCAGATATCACACTCCCATGCAGAATAAAACAAATTATAAACAGATGGCAGGAAGTAGGGAAAGCAA
TGTATGCCCCTCCCATCCAAGGACAAATTAGATGTTCATCAAAT---
ATTACCGGGATGCTATTAACAAGAGATGGTGGACATGGGAATGAAACT-------------------------
----

AATAATACTGAGGTATTCAGACCTGGAGGAGGAGATATGAGGGACAATTGGAGAAGTGAATTATATAAATATA
AAGTAGTAAGAATTGAACCGTTA---GGAGTAGCACCCACCAAGGCAAAGAGAAGAGTGGTG-----------
----------------------------CAGAGAGAAAAAAGAGCA---ATAGGA---ATAGGAGCT------
---GTGTTCCTTGGGTTCTTGGGAGCA---GCAGGAAGCACTATG---GGCGCAGCATCA---
ATGACGCTGACGGTACAG---GCCAGACAA---TTATTG---TCTGGTATAGTGCAGCAGCAAAAC---
AATTTG---CTGAGG---GCTATTGAGGCGCAACAACATCTGTTGCAACTCACAGTCTGGGGCATC
>GU362881
TTCTACTGTAATACATCAAGTCTATTTAACAGTACTTGGGGGGTTAACGATACTTGGAATAATACTTGGAATA
GG---

AATGAAAATGACAATATCACACTCCAATGCAGGATAAAACAAATTATAAACATGTGGCAGGCAGTAGGGAAAG
CAATATATGCCCCTCCCATTGCAGGATCAATTAGATGTTCATCAAAT---
ATTACAGGGCTACTATTAACAAGAGATGGTGGAAATGGTAATAATACT-------------------------
----

AATAATACCATCGAGATTTTCAGACCAGGAGGAGGAGACATGAGGGACAATTGGAGAAGTGAATTATATAAAT
ATAAAGTAGTAAGAATTGAACCATTA---GGAATAGCACCCACCAAGGCAAAGAGAAGAGTGGTG--------
-------------------------------CAGAGAGAAAAAAGAGCA---GTGGGA---CTAGGAGCT---
------CTGTTCCTTGGGTTCTTGGGAGCA---GCAGGAAGCACTATG---GGCGCAGCGTCA---
GTGACGCTGACGGTACAG---GCCAGACAA---TTATTG---TCTGGTATAGTGCAGCAGCAGAAC---
AATCTG---CTGAGG---GCTATTGAGGCGCAACAACATCTGTTGCAACTCACAGTCTGGGGCATC
>GU362885
TTCTACTGTAATACAACAAAGCTGTTTAACAGTACTTGGTCGACTAATGATACTTTGAATAATACTACAAGGG
GT---

AATGACACTATCATAACACTCCCCTGTAGAATAAAACAAATTATAAACATGTGGCAGGAAGTGGGAAAAGCAA
TGTATGCCCCTCCTATCAAAGGACAAATTAGATGTTCATCAAAT---
ATTACAGGACTGCTATTAACAAGAGATGGTGGTAATAAAACAGAA----------------------------
----

AACGAGACCGAGACCTTTAGACCTGGAGGAGGAAATATGAAGGACAATTGGAGAAGTGAATTATATAAATATA
AAGTAGTGCAAATTGAACCATTA---GGAGTAGCACCTACCAAGGCAAAGAGAAGAGTGGTG-----------
----------------------------CAAAGAGAAAAAAGAGCA---GTGGGAGCATTAGGAGCT------
---ATGGTCCTTGGGTTCTTGGGAGCA---GCAGGAAGCACTATG---GGCGCAGCATCA---
ATAACGCTGACGGTACAG---GCCAGACGA---TTAATG---TCTGGAATAGTGCAACAGCAGAAC---
AATCTG---CTGAAG---GCTATTGATGCGCAACAACAGATGTTGCAACTCACAGTCTGGGGCATC
>KC473842
TTCTACTGTAATACAACACAACTGTTTAATAATACTTGGAATGAAACTGCTTGGAATGAGACTAACAATGTAA
AT---

AACAGTGAAACTATCACACTCAAATGCAGAATAAAACAAATTATAAACAGGTGGCAGGGAGTAGGAAAAGCAA
TGTATGCCCCTCCCATCAGAGGATCAATTAGATGTTCATCAGAT---
ATTACAGGGCTGCTATTAACAAGAGATGGTGGGAACACGAATGAG----------------------------
----

ACCACTGAGATCTTCAGACCGGGAGGAGGAGATATGAGGGATAATTGGAGAAGTGAATTATATAAATATAAAG
TAGTACAAATTGAACCAATA---GGAATAGCACCCACCAAGGCAAAGAGAAGAGTGGTG--------------
-------------------------CAGAGAGAAAAAAGGGCA---GCG------TTCGGAGCA---------
TTGTTCCTTGGGTTCTTGGGAGCA---GCAGGAAGCACTATG---GGCGCAGCGTCA---
CTAACGCTGACGGTACAG---GCCAGACAA---TTATTG---TCTGGTATAGTGCAACAGCAGAGC---
AATCTG---CTGAGG---GCTATTGAGGCGCAACAGCATATGTTGCAACTCACAGTCTGGGGCATC
>KT200353
TTCTACTGTGATACATCAAAACTGTTTAATAGTACTTGGCCGCCTAATAGTACTGTTAATGGTACTGATAATG
GA---

AGTAATGAAAATATCACACTCCAATGTAGAATAAAACAAATTATAAACATGTGGCAGGAAGTAGGAAAAGCAA
TGTATGCCCCTCCCATTAAAGGACTAATTAAATGCTCATCAAAT---
ATCACAGGGCTGCTGATAGCAAGAGATGGTGGTAATAGTGGTAAT----------------------------
----

AACACTACTGAGATCTTCAGACCTTGGGGAGGAGATATGAGGGACAATTGGAGAAGTGAGTTATACAAATATA
AAGTAGTGAAAATTGAACCATTA---GGGTTAGCACCCACCAAGGCACAGAGAAGAGTGGTG-----------
----------------------------CAGAGAGAGAAAAGAGCA---GTGGGA---ATAGGAGCC------
---GTGTTCCTTGGGTTCTTGGGAGCA---GCAGGAAGCACTATG---GGCGCAGCGTCA---
TTGACGCTGACGGTACAG---GCCAGATTA---TTATTA---TCTGGTATAGTGCAACAGCAGAAC---
AATCTG---CTGAGA---GCTATTGAGGCGCAACAACATCTGTTGCAACTCACAGTCTGGGGCATC
>KT200355
TTCTATTGTAATACATCAACACTGTTTAACAGTACTTGGAATGCTAACAGTACTTGGAATGATACTGAAGGAA
CAGATGACCAC--
-------------------AAC---
ACAAATAATCTTAACATCACACTCCAGTGCAGAATAAAACAAATTGTAAACATGTGGCAGGAAGTAGGAAAAG
CAATGTATGCCCCTCCCATTAGAGGACAAATTAGATGTACATCAAAC---
ATTACAGGGGTGTTATTAACAAGAGATGGGGGGAATAATAATAACACAAGA----------------------
----

AACGGGAATGAGACTTTCAGACCTGGAGGAGGAGATATGAGGGACAATTGGAGAAGTGAATTATATAAATATA
AGGTAGTACAAATTGAACCATTA---GGAATAGCACCCACCAAGGCAAAGAGAAGAGTGGTG-----------
----------------------------CAGAGAGAAAAAAGAGCA---GTGGGA---CTAGGAGCT------
---GTGTTCCTTGGGTTCTTGGGAGCA---GCCGGAAGCACTATG---GGCGCAGCGTCA---
CTGACGCTGACGGTACAG---GCCAGACTA---TTATTG---TCTGGTATAGTGCAACAGCAAAAC---
AATTTG---CTGAGA---GCTATTGAGGCGCAACAGCATCTGTTGCAACTCACAGTCTGGGGCATC
>KT200356
TTCTACTGTAATACAACAGAACTGTTTAATAGTACATGGAATGGTAATGTAAGCAATAATACTGAA-------
----

GGAGATAAAATTATCACAATCCCATGCAGAATAAAACAAATTATAAACAGGTGGCAGGAAGTAGGAAAATCAA
TGTATGCCCCTCCTATCAGCGGAATAATTAGTTGTTCATCAAAT---
ATTACAGGGTTGCTATTAACAAGAGATGGTGGTAATACAGGGAAC----------------------------
----

AACGCCACCGAGATCTTCAGACCTGGAGGAGGAGATATGAGGGACAATTGGAGAAGTGAATTATATAAATATA
AAGTAGTAAAAATTGAACCAATA---GGAGTAGCACCCACCAAGGCAAAGAGAAGAGTGGTG-----------
----------------------------CAGAGAGAAAAAAGAGCA---ATAGGA---CTAGGAGCT------
---TTGTTCCTTGGGTTCTTGGGAGCA---GCAGGAAGCACTATG---GGCGCAGCGTCA---
GTGACGCTGACGGTACAG---GCCAGACAA---TTATTG---TCTGGTATAGTGCAACAGCAGAAC---
AATTTG---CTGAGG---GCTATTGAGGCGCAGCAGCATCTGTTGCAACTCACAGTCTGGGGCATC
>KY465967
TTCTACTGTAATTCAACACCACTGTTTAACTGGACTTTGAATGATACTAAGAAGGAGAATGACACTGAA----
----

GAAGATAAAAATATCACACTCCAATGCAGAATAAAACAATTTATAAACATGTGGCAGGGAGTAGGAAGAGCAA
TGTATGCCCCGCCCATCCAAGGACAAATTAAATGTTCATCAAAT---
ATTACAGGGCTAATATTAACAAGAGATGGTGGTAAGAGTGATAAC----------------------------
----

CCAAGTGAGATCTTCAGACCTGGAGGAGGAAATATGAAGGACAATTGGAGAAGTGAATTATATAAATATAAAG
TAGTAAAAATTGAACCATTA---GGAGTAGCACCCACCAAGGCAAGGAGAAGAGTGGTG--------------
-------------------------CAGAGAGAAAAAAGAGCA---GCA------CTAGGAGCT---------
TTGTTCCTTGGGTTCTTGGGAGCA---GCAGGAAGCACTATG---GGCGCAGCGTCG---
CTAACGCTGACGGTACAG---GCCAGACAA---TTATTG---TCTGGTATAGTGCAACAGCAAAGC---
AATTTG---CTGAGG---GCTATTGAGGCGCAACAGCATATGTTGCAACTCACAGTCTGGGGCATC
>KT276263
TTCTACTGTAATACAACACAACTGTTTAACAGTACTTGGGATAATAATACTACACAATCAAATAAT-------
----

ACCAATGGAAACATCACACTCCACTGCAGAATAAAACAAATTATAAACATGTGGCAGGAAGTAGGAAAAGCAA
TGTATGCCCCGCCCATCGCAGGACAAATTAGTTGTTCATCAAAT---
ATTACAGGGCTGCTATTAACAAGAGATGGTGGTAATAGCAGTAGT----------------------------
----

GGCAATGAGACCTTTAGACCTGCAGGAGGAGATATGAAAGACAATTGGAGAAGTGAATTATATAAATACAAAG
TAGTAAAAATTGAGCCATTA---GGAGTAGCACCCACCAGGGCAAAAAGGAGAGTGGTG--------------
-------------------------CAGAGAGAAAAAAGAGCA---GTGGGAGCGATAGGAGCA---------
ATGTTCCTTGGGTTCTTGGGGGCA---GCAGGAAGCACTATG---GGCGCAGCGTCG---
ATGGCGCTGACGGTACAG---GCCAGACAA---TTATTG---TCTGGTATAGTGCAGCAGCAAAAT---
AACTTG---CTGAGA---GCTATTGAGGCGCAACAGCATATGTTGCAACTCACAGTCTGGGGCATC
>KT276266
TTCTACTGTAATACATCAGACCTGTTTAATGAGACTAAGTTTAAT----------------------------
----

GAGACTGAGTCTATCATACTTCCATGCAGGATAAAGCAAATTATAAACATGTGGCAAGAAGCAGGAAAAGCAA
TGTATGCCCCTCCCATTAGCGGAATTATTAGCTGTTCATCAAAT---
ATTACAGGGCTGCTATTAACAAGAGATGGTGGTAATGGCACAAAC----------------------------
----

AACACGGAGATCTTCAGGCCTGCAGGAGGAAATATGAAAGATAATTGGAGAAGTGAATTATATAAATACAAAG
TAGTAAAAATTGAGCCATTA---GGAGTAGCACCCACCAAGGCAAGGAGAAGAGTGGTG--------------
-------------------------CAGAGAGAAAAAAGAGCA---ATAGGA---ATGGGAGCT---------
ATGTTCCTTGGGTTCTTGGGAGCA---GCAGGAAGCACTATG---GGCGCAGCGTCA---
ATGACGCTGACGGTACAG---GCCAGACAA---TTATTG---TCTGGTATAGTGCAACAGCAGAAC---
AACCTG---CTGAAG---GCTATTGATGCGCAACAGCATATGTTGCAGCTCACAGTCTGGGGCATC
>KT276267
TTCTACTGCAATACATCAAAACTGTTTGATAGTACTTGGCTTAATGGTACTAGGTCTAATAGTACTGGAAATA
ACAAC--

ACTGGAGAAACTATCACACTCCCATGTAGAATAAAACAAATCATAAACATGTGGCAGCAGGTAGGAAAAGCAA
TGTATGCCCCTCCTATCGAAGGACATATTAGGTGTTCATCAAAT---
ATTACAGGGCTGATCTTAACAAGAGATGGCGGTGATGACAACAAT----------------------------
----

AATAGCACAGAGATCTTCAGACCGGGAGGAGGAGATATGAGGGACAATTGGAGAAGTGAATTATATAAATACA
AAGTAGTAAAAATTGAACCATTA---GGGATAGCACCCACCAAGGCAAAGAGAAGAGTGGTG-----------
----------------------------CAGAGAGAAAAAAGGGCA---GTGGGA---ATAGGAGCT------
---GTGTTCCTTGGGTTCTTGGGAGCA---GCAGGAAGCACTATG---GGCGCAGCGTCA---
CTGACGCTGACGGTACAG---GCTAGACAA---TTACTG---TCTGGTATAGTGCAACAGCAGAAC---
AATTTG---CTGAGG---GCTATTGAGGCGCAACAACATTTGCTGCAACTCACAGTCTGGGGCATC
>KU685585
TTCTACTGCAATACATCACAACTGTTTAATAGTACTTGGAATAATAATGGTACCCGGATTAATGATACCTGGT
CTAATAGT---
-------------ACTAGC------
AACACTAACGAAACTATCACACTCCCATGTAGAATAAAACAAATTATAAACATGTGGCAGAGGGTAGGAAAAG
CAATGTATGCCCCTCCCATCAGAGGATACATTAATTGTTCATCAAAT---
ATTACAGGGCTGATCTTAACAAGAGATGGCGGTGTTAACAGCAGT----------------------------
----

GGGAGCGAGATCTTCAGACCGGGAGGAGGAGATATGAGGGACAATTGGAGAAGTGAATTGTATAAATACAAAG
TAGTACAAATTGAACCATTA---GGGATAGCACCCACCAAGGCAAAGAGAAGAGTGGTG-------------
-------------------------CAAAGAGAAAAAAGAGCA---GTGGGA---TTAGGAGCT---------
ATGTTCCTTGGGTTCTTGGGAGCA---GCAGGAAGCACTATG---GGCGCAGCGTCA---
ATAACGCTGACGGCACAG---GCTAGACAA---TTATTG---TCTGGTATAGTGCAACAGCAGAAC---
AATTTG---CTGAGG---GCTATTGAGGCGCAACAACATTTGTTGCAACTCACAGTCTGGGGCATC
>KU685589
TTCTACTGTAATACATCACAACTGTTTAACAGTACTTGGGATAACAGTACTTGGAATAATAATGGAAGCTCAA
GTAACAGCACA---TCC---

TCAAATGACATCATCACACTCAGGTGTAGAATAAAACAAATTATAAACATGTGGCAGAAAGTAGGAAGAGCAA
TGTATGCCCCTCCCATCAGCGGAAACATTAGCTGTTCATCCAAT---
ATCACAGGGCTGCTCTTAACAAGAGATGGTGGCAGTGGCAACAATACC-------------------------
----

AACAACACTGAGGTCTTTAGACCTGAAGGAGGAGATATGAGGGACAATTGGAGAAGTGAATTATACAAATACA
AAGTAGTAAAAATTGAACCATTA---GGAATAGCACCCACCAAGGCAAAAAGAAGAGTGGTG-----------
----------------------------CAGCGAGAAAAAAGAGCA---ATAACA---TTTGGAGCT------
---ATGATCCTTGGTTCCTTGGGAGCA---GCCGGAAGCACTATG---GGCGCAGCGTCA---
ATAGCGCTGACGGCACAG---GCCAGACAA---TTATTG---TCTGGTATAGTGCAACAGCAGAAC---
AATCTG---CTGAGG---GCTATTGAGGCGCAACAGCATCTGTTGCAACTCACAGTCTGGGGCATC
>KY989949
TTCTATTGTAATACAACACAACTGTTTAACAGTACATGGGTTAGTAATGAAAACTTAACTAATAGTACCAATG
AA---

GACAATAGCACAATCACACTCCCATGTAGAATAAAACAAATCATAAACATGTGGCAGGAAATAGGAAAAGCAA
TGTATGCCCCTCCCATCAGTGGACAAATTAGATGTTCATCAAAT---
ATCACAGGACTGCTATTAACAAGAGATGGTGGCAACACAAGCAAC----------------------------
----

CCCAACACAGAGACCTTCAGACCTGGAGGAGGAGATATGAGGGACAATTGGAGAAGTGAATTATATAAATATA
AAGTAGTAAAAATTGAACCATTA---GGAGTAGCACCCACCAGGGCAAGGAGAAGAGTGGTG-----------
----------------------------CAGAGAGAAAAAAGAGCA---GTGGAA---CTAGGAGCT------
---TTGTTTCTTGGGTTCTTGGGAGCA---GCAGGAAGCACTATG---GGCGCAGCGTCA---
GTGACGCTGACGGTACAG---GCCAGACAA---TTATTG---TCTGGTATAGTGCAACAGCAGAAC---
AATTTG---CTGATG---GCGATTGAGGCGCAACAACGCATGTTGCAACTCACAGTCTGGGGCATC
>KY989954
TTCTACTGTAATACAACACCACTGTTTAATAGTACTTGGAGTAATGGTACTTGGAATGGG-------------
----

AGTGCTGAGAATATCACACTCCCATGCAGAATAAAACAAATCATAAACATGTGGCAAGAAGTAGGAAGAGCAA
TGTATGCCCCTCCCATCAGCGGACTAATTAGGTGCTCATCAAAC---
ATTACAGGACTGATATTAACAAGAGATGGTGGCAATAGCAATGATACC-------------------------
----

AATGGCACCGAGATCTTCAGACCTGGAGGAGGAGATATGAGGGATAATTGGAGAAGTGAATTATATAAATACA
AAGTAATAGAACTTGAACCATTA---GGAATAGCACCCACCAAGGCAAAGAGACGAGTGGTG-----------
----------------------------CAGAGAGAAAAAAGAGCA---GTGGGA---CTAGGAGCC------
---ATGTTCTTTGGGTTCTTGGGTGCA---GCAGGAAGCACTATG---GGCGCAGCGTCA---
GTGACGCTGACGGTACAG---GCCAGACGC---TTGTTG---TCTGGTATAGTGCAACAGCAAAAC---
AATTTG---CTGAAG---GCTATTGAGGCGCAACAGCATCTGTTGCAACTCACAGTCTGGGGCATT
>KY989956
TTCTACTGTAATACAACAAAACTGTTTAATAGTAGTTGGGATATGAGCAACCTAAATAGCACAGAAGCAAATA
GCAGC--

AGCGAAGCAAAGATCACACTCCCATGCAGAATAAAACAAATTATAAATATGTGGCAGGGAGTAGGCAAAGCAA
TGTATGCCCCTCCCATCAGAGGACAAATTAAATGTGTATCAGAT---
ATTACAGGGCTGCTATTAACAAGAGATGGT---ACTGGAAACGAT----------------------------
----

ACCCTTGAAATCTTTAGACCTGGAGGAGGAAATATGAAGGACAATTGGAGAAGTGAATTATATAAATATAAAG
TAGTAAGAATTGAACCATTA---GGAGTAGCACCCACCAAGGCAAAGAGAAGAGTGGTG--------------
-------------------------CAGAGAGAAAAAAGAGCA---TTGGGAACACTAGGAGCT---------
GTGTTTCTTGGGTTCTTGGGAACA---GCAGGGAGCACTATG---GGCGCAGCGTCA---
CTGACGCTGACGGTACAG---GCCAGACAA---TTATTA---TCTGGTATAGTGCAACAGCAGAAC---
AATCTG---CTGAAA---GCTATTGATGCACAACAGCATCTGTTGCAACTCACGGTCTGGGGCATC
>KC595171
TTCTACTGTAATACAACACAACTGTTTAATAGTACTTGGAATGGTACTGAAGGGTCAAATAACACTGAA----
----

GGAAATGACACAATCACACTCCCATGCAGAATAAAACAAATTGTAAACAGGTGGCAGGAAGTAGGAAAAGCAA
TGTATGCCCCTCCCATCAGAGGACAGATTAGATGTTCATCCAAT---
ATTACAGGGCTGCTCTTAACAAGAGATGGGGGTGAGAACAATACT----------------------------
----

ACTGGGACCGAGACCTTCAGACCTGGAGGAGGAGATATGAGGGACAATTGGAGAAGTGAATTATATAAATATA
AAGTAGTAAAAATTGAGCCATTA---GGAATAGCACCCACCAAGGCAAAGAGAAGAGTGGTG-----------
----------------------------CAGAGAGAAAAAAGAGCA---GTGGGA---ATAGGAGCT------
---GTGTTCCTTGGGTTCTTGGGAGCA---GCAGGAAGCACTATG---GGCGCAGCGTCA---
ATGACGCTGACGGTACAG---GCCAGACTA---TTATTG---TCTGGTATAGTGCAACAGCAGAAC---
AATTTG---CTGAGG---GCTATTGAGGCGCAACAGCATCTGTTGCAACTCACAGTCTGGGGCATC
>KC595172
TTCTACTGTAATACAACAAAACTGTTTAATAGTACCTGGAGTGATACTGAAGGGTTAAATACCACTGAAGGAA
ATAAA--

ACAAATGAAATAATCACACTCCCATGCAGAATAAAACAAATTGTAAACATGTGGCAGGAAGTAGGAAAAGCAA
TGTATGCCCCTCCCATCAATGGAATAATTAGATGTTCATCAAAT---
ATTACAGGGCTGCTATTAACAAGAGATGGTGGTAATAACACGAAC----------------------------
----

GGGTCCGAGATTTTCAGACCTGGAGGAGGAAATATGAGGGACAATTGGAGAAGTGAATTATATAAATATAAAG
TAGTAAAAATTGAACCATTA---GGAGTAGCACCCAACAAGGCAAAGAGAAGAGTGGTG--------------
-------------------------CAGAGAGAAAAAAGAGCA---CTGGGAATAGGAGCTGCT---------
GTGTTCCTTGGGTTCTTGGGAGCA---GCAGGAAGCACTATG---GGCGCAGCGGCA---
CTGACGCTGACGGTACAG---GCCAGACTA---TTATTG---TCTGGTATAGTGCAGCAGCAGAAT---
AAATTG---CTGAGA---GCTATTGAGGCGCAACAGCATCTGTTGCAACTCACAGTCTGGGGCATC
>KC595174
TTCTACTGTAATACAACACAACTGTTTAATAGTACTTGGAATATTACTGAAGGGTTTAATGGTACTTGGAATG
AT---

ACTGCAAATAATATCACACTCCCATGTAGAATAAAACAAATTGTAAACATGTGGCAGGAAGTAGGAAAAGCAA
TGTATGCCCCTCCCATCAAAGGACAAATTAGATGTTCATCAAAT---
ATTACAGGGCTGATATTAACAAGAGATGGTGGTAATACTACCAAC----------------------------
----

AACGAGACCGAGACTTTCAGACCTGGAGGAGGAGATATGAGGGACAATTGGAGAAGTGAATTATATAAATATA
AAGTAGTAAAACTTGAACCATTA---GGAATAGCACCCACCAAGGCAAAGAGAAGAGTGGTG-----------
----------------------------CAAAGAGAAAAAAGAGCA---GTA------ATAGGAGCT------
---TTGTTCCTTGGGTTCTTGGGAGCA---GCAGGAAGCACTATG---GGCGCAGCGTCA---
GTGACGTTGACGGTACAG---GCCAGACAA---TTATTG---TCTGGTATAGTGCAACAGCAGAAT---
AATTTG---CTGAGG---GCTATTGAGGCGCAACAGCATCTGTTGCAACTCACAGTCTGGGGCATC
>KC595177
TTCTACTGTAATTCAACACAACTGTTTAATAGTACTTGGAATAGTACTGAAGGGTCAAATAATATTACA----
----

GCAAATGGAACAATCACACTCCCATGCAGAATAAAACAAATTATAAACATGTGGCAGGAAGTAGGAAAAGCAA
TGTATGCTCCTCCCATCAGAGGACAAATTAGATGCTCATCAAAT---
ATTACAGGGCTGCTATTAACAAGAGATGGTGGTAATGGTAACGAG----------------------------
----

ACCACCGAGATTTTCAGACCTGGGGGAGGAGATATGAGGGACAATTGGAGAAGTGAATTATATAAATATAAAG
TAGTAAAAATTGAACCATTA---GGAGTAGCACCCACCAGGGCAAAGAGAAGAGTGGTG-------------
-------------------------CAGAGAGAAAAAAGAGCA---GTGGGA---ATAGGAGCT---------
TTGTTCCTTGGGTTCTTGGGAGCA---GCAGGAAGCACTATG---GGCGCAGCGTCA---
CTGACGTTGACGGTACAG---GCCAGACAA---TTATTG---TCTGGTATAGTGCAACAACAGAAC---
AATTTG---CTGAGG---GCTATTGAGGCGCAACAGCATCTGTTGCAACTCACAGTCTGGGGCATC
>KC595178
TTCTACTGTAATTCAACACAACTGTTTAATAGTACTTGGAAGGATACTGAAGGGTCAAATGACACTACA----
----

GGAAATGACACAATCACACTCCCATGCAGAATAAAACAAATTGTAAACATGTGGCAGGAAGTAGGAAAAGCAA
TGTATGCCCCTCCCATCAGAGGACAAATTAGATGTTCATCAAAT---
ATTACAGGGCTGATATTAACAAGAGACGGTGGTACAGACAATAAC-----------------------------
----

AAGACCGAGACCTTCAGACCTGGAGGAGGAGATATGAGGGACAATTGGAGAAGTGAATTATATAAATATAAAG
TAGTAAAAGTTGAACCATTA---GGAGTAGCACCCACCAGGGCAAAGAGAAGAGTGGTG-------------
-------------------------CAAAGAGAAAAAAGAGCA---GTGGGA---ATAGGAGCT---------
GTGTTCCTTGGGTTCTTGGGAGCA---GCAGGAAGCACTATG---GGCGCAGCGCCA---
ATGACGCTGACGGTACAG---GCCAGACTA---TTATTG---TCTGGTATAGTGCAACAGCAGAAC---
AATTTG---CTGAGG---GCTATTGAGGCGCAACAGCATCTGTTGCAACTCACAGTCTGGGGCATC
>KC595195
TTCTACTGTAATACAACAGAACTGTTTAATAGTACTTGGAAAGGTACTGAAAGGTTAAATAGCTCTTCTACAA
ATTCA---

AATCCTACAGAAATCATGCTCCCATGCAGAATAAAGCAAATTATAAACATGTGGCAGGAAGTAGGAAAAGCAA
TGTATGCCCCTCCCATCAAAGGACAAATTAAATGTTCATCAAAC---
ATTACAGGGTTGCTACTAACAAGGGATGGTGGTAATGATAATAACACT-------------------------
----

AACGGGACCGAGATCTTCAGACCTGGAGGAGGAGATATGAGGGATAATTGGAGAAGTGAATTATATAAATATA
AAGTAGTAAGACTTGAACCATTA---GGAGTAGCACCCACCAAGGCAAAAAGGAGAGTGGTG-----------
----------------------------CAAAGAGAAAAAAGAGCA---ATAGGA---ATAGGAGCG------
---ATGTTCCTTGGGTTCTTGGGAACA---GCAGGAAGCACTATG---GGCGCAGCGTCA---
CTGACGTTGACGGTACAG---GCCAGACTA---TTGTTG---TCTGGTATAGTACAACAGCAGAAC---
AATTTG---CTGAGA---GCTATAGAGGCGCAACAACATCTGTTGCAACTCACTGTCTGGGGCATT
>DQ854716
TTCTACTGTAATTCAACACAACTGTTTAACAGTACTTGGAATGGTACTGCATGGTCAAATAACACTGAAGGAA
AT--

GAAAATGACACAATCACACTCCCATGCAGAATAAAACAAATTATAAACATGTGGCAGGAAGTAGGAAAAGCAA
TGTATGCCCCTCCCATCAGAGGACAAATTAGATGTTCATCAAAT---
ATTACAGGGCTGATATTAACAAGAGATGGTGGTATTAACCAGACC-----------------------------
----

AACACCACCGAAATCTTCAGGCCTGGAGGAGGAGATATGAGGGACAATTGGAGAAGTGAATTATATAAATATA
AAGTAGTAAAAATTGAACCATTA---GGAATAGCACCCACCAAGGCAAAGAGAAGAGTGGTG-----------
----------------------------CAGAGAGAAAAAAGAGCA---GTGGGAATAATAGGAGCT------
---ATGCTCCTTGGGTTCTTGGGAGCA---GCAGGAAGCACTATG---GGCGCAGCGTCA---
ATGACGCTGACGGTACAG---GCCAGACAA---TTATTG---TCTGGTATAGTGCAACAGCAGAAC---
AATTTG---CTGAGG---GCTATTGAGGCGCAACAGCATCTGTTGCAACTCACAGTCTGGGGCATC
>KC595182
TTCTACTGTAATTCATCACAACTGTTTAATAGTACTTGGGAGTTTAATAGTACTTGGGAGTTTAATAGTACTG
GGAATAGTACT---

GAAGAAGACCCTATCATACTCCCATGCAGAATAAAACAAATTATAAACATGTGGCAGGAAGTAGGAAAAGCAA
TGTATGCCCCTCCCATCAGAGGACAAATTAGATGCTTATCAAAT---
ATTACAGGACTGCTATTAACAAGAGATGGTGGTAAGGACGATAAC-----------------------------
----

ATGACCGAGACCTTCAGACCTGGAGGAGGAGATATGAGGGACAATTGGAGAAGTGAATTATATAAATATAAAG
TAGTAAAAGTTGAACCATTA---GGAGTAGCACCCACCAAGGCAAAGAGAAGAGTGGTG--------------
-------------------------CAGAGAGAAAAAAGAGCA---GTGGGA---ATAGGAGCT---------
GTGTTCCTTGGGTTCTTGGGAGCA---GCAGGAAGCACTATG---GGCGCAGCGTCG---
ATGACGTTGACGGTACAA---GCCAGACAA---TTATTG---TCTGGTATAGTGCAACAGCAAAAC---
AATTTG---CTGAGG---GCTATTGAGGCGCAACAGCATCTGTTGCAACTCACAGTCTGGGGCATC
>KC595159
TTCTACTGTAATTCAACACAACTGTTTAATAGTACGTGGAATGGTACTGAAAGGTCAAATAACACT-------
----

GAAGGAAATATAATCACACTCCCATGCAGAATAAAACAAATTATAAATATGTGGCAGGAAGTAGGAAAAGCAA
TGTATGCCCCTCCTATCAGAGGACAAATTAGATGTTCATCAAAT---
ATTACAGGGCTGATATTAACAAGAGATGGTGGTAAGAACGAGAAC----------------------------
----

GAGACCGAGATCTTCAGACCTGGAGGAGGAGATATGAGGGATAATTGGAGAAGTGAATTATATAAATATAAAG
TAGTAAAAATTGAACCATTA---GGAGTAGCACCCACCAAGGCAAAGAGGAGAGTGGTG--------------
-------------------------CAGAGAGAGAAAAGAGCA---GTGGGA---ATAGGAGCT---------
GTGTTTCTTGGGTTCTTGGGAGCA---GCAGGAAGCACTATG---GGCGCAGCGTCA---
GTGACGCTGACGGTACAG---GCCAGACTA---TTATTG---TCTGGTATAGTGCAACAGCAGAGC---
AATTTG---CTGAGG---GCTATTGAAGCGCAACAGCATCTGTTGCAACTCACAGTCTGGGGCATT
>KC595225
TTTTATTGTAATTCAACACAACTGTTTAATAGTACTTGGTATACAAATGATACAAATGTTACTGCTGAA----
----

GGAAGTGACACAATTGTACTCCCATGCAAAATAAAACAAATTATAAACATGTGGCAGGAAGTAGGAAAAGCAA
TGTATGCCCCTCCCATCAGAGGACAAATTAACTGCTCATCAAAC---
ATTACAGGGTTGCTATTAACAAGAGATGGTGGTCCAAATAATACC----------------------------
----

AACGAGACCTTTAGACCTGGAGGAGGAAATATGAAGGACAATTGGAGAAGTGAATTATATAAATATAAAGTAG
TAAAAATTGAACCATTA---GGAGTAGCACCCACCGAGGCAAAAAGAAGAGTGGTG-----------------
----------------------CAGAGAGAAAAAAGAGCA---GTGGGA---ATGGGAGCT---------
ATGTTCCTTGGGTTCTTGGCCACA---GCAGGAGGCACTATG---GGCGCAGCGTCA---
GTGACGCTGACGGTACAG---ACCAGACAA---TTATTG---TCTGGTATAGTGCAACAGCAGAGC---
AATTTG---CTGAGG---GCTATTGAGGCGCAACAGCATATGTTGCAACTCACAGTCTGGGGCATC
>KC595222
TTCTACTGTAATTCAACACAACTGTTTAACAGTACTTGGTATGCGAATGGTACTAGAGAGCCAAATGACACTG
CA---

AGAAATGGCACAATCACACTCCCATGCAGAATAAAACAAATTATAAACATGTGGCAGAAAGTAGGAAAAGCAA
TGTATGCCCCTCCCATCAAAGGACCAATTAGATGTTCATCAAAT---
ATCACAGGGGTGATATTAACAAGGGATGGTGGCGGTAATGACAGTAGTGAG----------------------
----

GAGACCTTCAGACCTGGAGGAGGAGACATGAGGGACAATTGGAGAAGTGAATTATATAAATATAAAGTAGTAG
AACTTGAGCCATTA---GGAGTAGCACCCACCAGGGCAAAGAGAAGAGTGGTG--------------------
-------------------CAGAGGGAAAAAAGAGCA---GTGGGAATATTAGGAGCT---------
ATGTTCCTTGGGTTCTTGGGAGCA---GCAGGAAGCACTATG---GGCGCAGCGGCA---
GTGACGCTGACGGTACAG---GCCAGACAA---CTAATG---TCTGGTATAGTGCAACAGCAGAAC---
AACTTG---CTGAGG---GCTATTGAGGCGCAACAGCATCTGTTGCAACTCACAGTCTGGGGCATC
>KC595206
TTCTACTGTGACTCAACACAACTGTTCAATAGTACTTGGAATGGTACTAAAAGGATAATAGGAACTGAA----
----

GAGAATGGCACGATCACGCTCCCATGCAGAATAAAACAAATTGTAAACATGTGGCAGGAAGTAGGAAAAGCAA
TGTATGCCCCTCCCATCAGAGGAAACATTAACTGCTCATCAAAT---
ATTACAGGACTGTTATTAACAAGAGATGGTGGTAAAGACGAAAAT----------------------------
----

GAGACCGATACTGAGACCTTCAGACCTGGAGGAGGAGATATGAGGGACAATTGGAGAAGTGAATTATATAAAT
ATAAAGTAGTAAAAATTGAACCATTA---GGAGTAGCACCCACCAGGGCAAAGAGAAGAGTGGTG--------
-------------------------------CAGAGAGAAAAAAGAGCA---ATTGGAGCA---GGAGTT---
------ATGCTCTTTGGGTTCTTGGGAGCA---GCAGGAAGCACTATG---GGCGCAGCGTCA---
ATGGCGTTGACGGTACAG---GCCAGACAA---TTATTG---TCTGGTATAGTGCAACAGCAGAGA---
AATTTG---CTGAGG---GCTATTGAGGCGCAACAACATCTGTTGCAACTCACAGTCTGGGGCATC
>DQ141341
TTCTACTGTAATTCATCACAACTGTTTAATAGTTCTTGGAATGTTAATGGAGAATCAAATAACACTGAA----
----

GGAAATGACACAATCACACTCCCATGCAGAATAAAACAAATTATAAACATGTGGCAGGAAGTAGGAAAAGCAA
TGTATGCCCCTCCCATTAGAGGACAAATTAGCTGTTCATCAAAT---
ATTACAGGGCTGCTATTAACAAGAGATGGTGGTAACAATAACGAGACC-------------------------
----

ACCACCACCGAGACCTTCAGACCTGGAGGAGGAGATATGAGGGACAATTGGAGAAGTGAATTATATAAATATA
AAGTAGTAAAAATTGAACCATTA---GGAATAGCACCCACCAGGGCAAAGAGAAGAGTGGTG-----------
----------------------------CAGAGAGAAAAAAGAGCA---GTGGGA---ATAGGAGCT------
---ATGTTCCTTGGGTTCTTGGGAGCA---GCAGGAAGCACTATG---GGCGCAGCGTCA---
GTGACGTTGACGGTACAG---GCCAGACTA---TTATTG---TCTGGTATAGTGCAACAGCAGAAC---
AATTTG---CTGAGG---GCTATTGAGGCGCAACAGCGTATGTTGCAACTCACAGTCTGGGGCATC
>DQ141345
TTCTACTGTAATACAACACAACTGTTTAATAGTACTTGGGAGGCTAATGGTACTTGGAATGGTGCTAAC----
----

AAGTCAGGTGACATCATACTCCCATGCAGAATAAAACAAATTATAAACATGTGGCAGGGAGTAGGAAAAGCAA
TGTATGCCCCTCCCATCAGTGGACAAATTAGATGTTCATCAAAC---
ATTACAGGGCTGCTATTAACAAGAGATGGTGGTAGTAACGAGACC----------------------------
----

AACGGGACTGAGATCTTCAGACCTGGAGGAGGAGATATGAGGGATAATTGGAGAAGTGAATTATATAAATATA
AAGTAGTAAAAATTGAACCATTA---GGAATAGCACCCACCAAGGCAAAGAGAAGAGTGGTG-----------
----------------------------CAGAGAGAAAAAAGAGCA---GCATTT------GGAGCT------
---ATGTTCCTTGGGTTCTTGGGAGCA---GCAGGAAGCACTATG---GGCGCAGCGTCA---
CTGACGCTGACGGTACAG---GCCAGGCAA---TTATTG---TCTGGTATAGTGCAACAGCAGAAC---
AATCTG---CTGAGG---GCTATTGAGGCGCAACAGCATCTGTTGCAACTCACAGTCTGGGGCATC
>EF531330
TTCTACTGTAATACATCACAGCTATTTAATAATACTTTTAATAGTACTGGGCCTAATAATAGTACTAAGAAG-
----

TTAAATGACACTATCACACTCCCATGCAGAATAAAACAAATTATAAACATGTGGCAGGAAGTAGGAAAAGCAA
TGTATGCCCCTCCCATTAGTGGACAAATTAACTGCTCATCAATT---
ATTACAGGGTTGCTATTAACAAGAGATGGTGGTGAGGGCAATGAGACC-------------------------
----

AATGGGAACGAGACCTTCAGACCTGGAGGAGGAGATATGAGGGATAATTGGAGGAGTGAATTATATAAATATA
AAGTAGTACAAATTGAACCAGTA---GGAGTAGCACCCACCAAGGCAAAGAGAAGAGTGGTG-----------
----------------------------CAGAGAGAAAAAAGAGCA---GTATTA------GGAGCT------
---ATGTTCCTTGGGTTCTTGGGAGCA---ACAGGAAGCACTATG---GGCGCAGCGTCA---
ATGGCGTTGACGGTACAG---GCCAGACAA---TTATTG---TCTGGTATAGTGCAACAGCAGAAC---
AATTTG---CTGAAG---GCTATTGAGGCGCAACAGCATCTGTTGCAACTCACAGTCTGGGGCATC
>EF531333
TTCTACTGTAATACAACACAACTGTTTACTTGGATTGTTGATGGTAATGGGAATATTAGTAGTATTGGGAATG
GTACTAAT---
-------------ACTACT------
GATGCTAACAAGAATATCACACTCCCATGTAGAATAAAACAAATTGTAAACATGTGGCAGGAAGTAGGAAAAG
CAATGTATGCTCCTCCCATAAGGGGACAAATTAGATGCTTATCAAAT---
ATTACCGGGTTGCTAATAACAAGAGATGGAGGTAGTAATAACAGT----------------------------
----

```
-------------
GCTAACGAGACTTTCAGACCTGGAGGAGGAGATATGAGGGACAATTGGAGAAGTGAATTATATAAATATAAAG
TGGTAAAAATTGAACCATTA---GGAGTAGCACCCACCAAGGCAAAGAGAAGAGTGGTG--------------
-------------------------CAGAGAGAAAAAAGAGCA---GTGGGA---ATAGGAGCT---------
GTGTTCCTTGGGTTCCTGGGAGCA---GCAGGAAGCACTATG---GGCGCAGCGTCA---
ATGACGTTGACGGTACAG---GCCAGACAA---TTATTG---TCTGGTATAGTGCAACAGCAGGAC---
AATCTG---CTGAGG---GCTATTGAGGCGCAACAGCATCTGTTGCAACTCACAGTCTGGGGCATC
>JX422195
TTCTACTGTAATACAACACAACTGTTTAATACAGCACTGCTTAATAGTTCT----------------------
-------------------------------------------------------------------------
----------------------------
AACGAGACAAATCTCACACTCCCATGCAGAATAAAACAAATTGTAAATATGTGGCAGGAAGTAGGAAGAGCAA
TGTATGCCCCTCCCAGACAAGGAATAATTAGATGTTCATCAAAT---
ATCACAGGATTGATATTAACAAGAGATGGTGGGGTCACGAACGGGTCC-------------------------
-------------------------------------------------------------------------
-------------
GAGACCGAGACCTTCAGACCAGGAGGAGGAGATATGAGGGACAATTGGAGAAGTGAATTATACAAATATAAAG
TAGTAAAAATAGAACCATTG---GGAATAGCACCCACCAAGGCAAGGAGAAGAGTGGTG--------------
-------------------------CAGAGAGAAAAAAGAGCA---GCA------CTAGGAGCT---------
ATGTTCCTTGGGTTCTTGGGAGCA---GCAGGAAGCACTATG---GGCGCAGTGTCA---
CTAACACTGACGGTACAA---GCCAGAATG---TTATTG---TCTGGTATAGTGCAACAGCAGAAT---
AATCTG---CTGAGG---GCTATTGAGGCGCAACAGCATATGTTGCAACTCACAGTCTGGGGCATC
>AY535455
TTCTACTGTAATACAACACAACTGTTTAATAGTACTTGGAAAAATAATACTACAAGTCCGAATAAA-------
-------------------------------------------------------------------------
----------------------------
GGAAATGGCACAATCACACTCCCATGCAGAATAAAACAAATTATAAACATGTGGCAGGAAGTAGGAAAAGCAA
TGTATGCCCCTCCCATCGCAGGACAAATTAGATGTGTATCAAAT---
ATTACAGGGCTGCTATTAACAAGAGATGGTGGTGGTGGTGGTAATATG-------------------------
-------------------------------------------------------------------------
-------
ACTAATGGGACCGAGATCTTCAGACCGGGAGGAGGAGATATGAGGGACAATTGGAGAAGTGAATTATATAAAT
ATAAAGTAGTAAAAATTGAGCCATTG---GGAGTAGCACCCACCAAGGCAAAAAGAAGAGTGGTG--------
-------------------------------CAGAGAGAAAAAAGAGCA---GTGGGA---ATAGGAGCT---
------TTGCTCTTTGGGTTCTTGGGAGCA---GCAGGAAGCACTATG---GGCGCAGCATCA---
ATGACGCTGACGGTACAG---GCCAGACTA---TTATTG---TCTGGTATAGTGCAACAGCAGAAT---
AATCTG---CTGAGG---GCTATTGAGGCGCAACAACATCTGTTGCAACTCACAGTCTGGGGCATC
>KC699018
TTCTACTGTAATACAACACAACTGTTTAATAGTACTTGGGATGCTACGGATGCTAATAACACTAGAGTAGAT-
-------------------------------------------------------------------------
----------------------------
AACACCACTGTCATCACACTCCCATGCAGAATAAAACAAATTATAAACATGTGGCAGGAGGTAGGAAAGGCAA
TGTATGCCCCTCCCATCAGCGGACAAATTAACTGCTCATCAAAT---
ATTACAGGGCTGCTATTAACAAGCGATGGGGGTAATAGTAATAAT----------------------------
-------------------------------------------------------------------------
-------------
GAGAGCGAGACCTTCAGACCTGGAGGGGGAGATATGAGGGACAATTGGAGAAGTGAATTATATAAATATAAAG
TAGTAGAAATTGAACCACTA---GGAGTAGCACCCACCAGGGCAAAAAGAAGAGTGGTG--------------
-------------------------CAGAGAGAAAAAAGAGCA---GTGACA---CTAGGAGCT---------
TTTTTCCTCGGGTTCTTGGGAGCA---GCAGGAAGCACTATG---GGCGCAGCGTCA---
ATGACGCTGACGGTACAG---GCCAGACAA---TTATTG---TCTGGTATAGTGCAACAGCAGAAC---
AATCTG---CTGAGG---GCTATTGAGGCGCAACAGCATCTGTTGCAACTCACAGTCTGGGGCATC
>KU168261
TTCTACTGTGATTCATCACCACTGTTTAACAGTACTTGGAGGAGAAATAGCACGGAAGGAAATGCCACCATTG
TAGGAAAT-----------------------------------------------------------------
----------GCCACCATT------
GGAAAAAATAACACACTTACACTCCAATGCAGAATAAAACAAATTATAAACATGTGGCAGGGAGTAGGAAGAG
CCATGTATGCCCCTCCCATTAGCGGAATCATTAGCTGCTCATCAAAT---
ATTACGGGGCTGATACTAACAAGAGATGGTGGTGACGAGAATAGGACC-------------------------
--------------------------------------------------------------------
```

ACTGACGAGAATAAT---
ACCACTGAGACCTTCAGACCTGGAGGGGGAGATATGAGGGATAATTGGAGAAGTGAATTATATAAATACAAAG
TAGTAAAGATTGAACCATTA---GGAGTAGCACCCACCGAGGCCAAGAGAAGAGTGGTG--------------
-------------------------CAGAGAGAAAAAAGAGCG---ATAGGA---CTAGGAGCT---------
ATGTTCCTCGGGTTCTTAGGAGCA---GCAGGAAGCACTATG---GGCGCAGCGTCA---
TTGACGTTGACGGTACAG---GCCAGACAA---TTATTG---TCTGGTATAGTGCAACAGCAAAAC---
AACTTG---CTGAGG---GCTATTGAGGCGCAACAGCATCTGTTGCAACTCACAGTCTGGGGCATC
>JX140652
TTCTACTGTAATTCAACGCAACTGTTTAATAGTACTTGGCTGTTTAATAGTACTGGGAATGATACAACA----
----

GAAAATGGTACCATCACACTCCAATGCAGAATAAAACAAATTATAAACATGTGGCAGGAAGTAGGAAAAGCAA
TGTATGCCCCTCCCATCAGAGGAAACATTAGGTGTTCCTCAAAT---
ATTACCGGGTTGCTATTAACAAGAGATGGTGGTAGCCATGAGAACGGGACCAAC-------------------
----

ACCACCAACACTGAGGTCTTCAGACCTGGAGGAGGAAATATGAGGGACAATTGGAGAAGTGAATTATATAAAT
ATAAAGTAATAGAAATTGAACCATTA---GGAATAGCACCCACCAAGGCAAAGAGAAGAGTGGTG--------
-------------------------------CAGAGAGAAAAAAGAGCA---GCG------CTAGGAGCT---
------GTATTCCTTGGGTTCTTGGGAGCA---GCAGGAAGCACTATG---GGCGCAGCATCA---
ATGACGCTGACGGTACAG---GCCAGACAA---TTATTG---TCTGGTATAGTGCAGCAGCAAAAC---
AACTTG---CTGAGG---GCTATTGAGGCGCAACAACATCTGTTGCAACTCACAGTCTGGGGCATC
>KC699029
TTCTACTGTAATACATCACAACTGTTTAACAGTACTTGGTCAAATGATACTAATAAG----------------
----

TCAAATGCAATAATCACACTCCCATGCAGAATAAAACAAATTATAAACATGTGGCAGGAAGTAGGAAAAGCAA
TGTATGCCCCTCCTATCAGAGGAGAAATTAAATGTTCATCAAAT---
ATTACAGGGCTGCTATTAACAAGAGATGGTGGTAATGGGAATAAGACA-------------------------
----

AACATCAGCATTGAAATCTTCAGACCTGGAGGAGGAAACATGAAGGACAATTGGAGAAGTGAATTATATAAAT
ATAAAGTAGTAAAAATTGAACCATTA---GGAGTAGCACCCACCAAGGCAAAGAGAAGAGTGGTG--------
-------------------------------CAGAGAGAAAAAAGAGCA---GTAGGA---ATAGGAGCT---
------TTGTTCCTTGGGTTCTTGGGAGCA---GCAGGGAGCACTATG---GGCGCAGCGTCA---
CTGACGCTGACGGTACAG---GCCAGACAA---TTATTG---TCTGGTATAGTGCAGCAGCAGAAC---
AATCTG---CTGAAG---GCTATTGAGGCGCAACAGCATCTGTTGCAACTCACAGTCTGGGGCATC
>KC699030
TTCTATTGTAATACAACAAGACTGTTTAGTAGTACTTGGTATAATAATGGTACTTGGTATACTAATGGTACTG
AGAATGGTATT--
-------------------AAA---
GAGTCAAATGCAACAATCATGCTGCCATGCAGGATAAAACAAATTATAAACATGTGGCAGAGTGTAGGAAAAG
CAATGTATGCCCCTCCCATCAGGGGACCACTTAGGTGTTCATCAAAT---
ATTACAGGGCTGCTATTAATGAGAGATGGTGGAGGTAATGAGAGC----------------------------
----

GAGACCACCCACAATGAGACCTTCAGACCCGGAGGGGGAGACATGAGGGACAATTGGAGAAGTGAACTATATA
AATATAAAGTAGTAGCAGTTGAACCATTA---GGAATAGCGCCCACCAAGGCAAAGAGAAGAGTGGTG-----
----------------------------------CAGAGAGAAAAAAGAGCA---GTAACA---
ATGGGAGCT---------ATGTTCCTTGGGTTCTTGGGAGCA---GCAGGAAGCACTATG---
GGCGCAGCGTCA---ATGACGCTGACGGTACAG---GCCAGACAA---TTATTG---
TCTGGTATAGTGCAACAGCAAAGC---AATTTG---CTGAAG---
GCTATTGAGGCGCAGCAACATTTGTTGCAACTCACGGTCTGGGGCATT
>KC699028
TTCTACTGTAACACAACACAGCTGTTTAATAGTACTTGGACATTCAATGATACTAGGAGTAATGATACAGAGT
CA---

GATAACGCTACGATTATACTCCCATGCAGAATAAAACAAATTATAAACAGGTGGCAGGAAGTAGGAAAAGCAA
TGTATGCCCCTCCCATCAGTGGAATAATTAGATGCTCATCAAAT---
ATTACAGGGCTGCTATTAACAAGAGATGGTGGTAATGAGAAAAAT----------------------------

----

AAGAGTACCAATGAGACCTTCAGACCTGGAGGAGGAGACATGAGAGACAATTGGAGAAGTGAATTATATAAAT
ATAAAGTAGTAAGAATTGAGCCAATA---GGAATAGCACCCACCAGGGCAAGGAGAAGAGTGGTG--------
------------------------------CAGAGAGAAAAAAGAGCA---GTGGGAACGCTGGGAGCT---
------ATGTTCCTTGGGTTCTTGGGAGCA---GCAGGAAGCACTATG---GGCGCAGCGTCA---
ATGACGCTGACGGTACAG---GCCAGACAA---TTATTG---TCTGGTATAGTGCAACAGCAAAAC---
AATTTG---CTGAGA---GCTATTGAGGCGCAACAGCATCTGTTGCAACTCACAGTCTGGGGCATT
>KC699039
TTCTACTGTAATACAACACAACTGTTTAATGGTACTTGGAATGGTACCTGGGATGGTACTTGGAATGGTACTT
GGACTCGGATA---
----------GAGCCAAAT------
ATCAATGATACAAATATCACACTCCCATGCAGAATAAAACAAATTATAAACATGTGGCAGGGAATAGGAAGAG
CAATGTATGCCCCTCCCATCAGGGGACAAATTAAATGTATATCAAAT---
ATTACCGGGTTGCTAATAACAAGAGATGGTGGTGATGAGGAGAAC---------------------------
----

AAGACTGAGGAGGTCTTCAGACCTGGAGGAGGAGATATGAGAGACAATTGGAGAAGTGAATTATATAAATATA
AAGTAGTACAAATTGAACCAATA---GGAATAGCACCCACCAAGGCAAAGAGAAGAGTGGTG-----------
---------------------------CAGAGAGAGAAAAGAGCG------GGA---ATAGGAGCT------
---TTGTTCCTTGGGTTCTTGGGAGCA---GCAGGAAGCACTATG---GGCGCAGCATCA---
ATAACGCTGACGGTACAG---GCCAGACAA---TTGTTG---TCTGGTATAGTGCAACAGCAGAAC---
AATTTG---CTGAGA---GCTATTGAGGCGCAACACCATCTGTTGCAACTCACAGTCTGGGGCATC
>JX140654
TTCTACTGTAATACAACACCACTGTTTAATAGTGCTTGGAATAGGAATAAGAGTAAGGATACTTGGAGTATCA
AAGGGTCAAAT---
----------AATAGCACT------
GGAGGAAATAACACTATCATACTCCAATGTAAAATAAAACAAATTATAAACATGTGGCAAAGAGTAGGAAAAG
CAATGTATGCCCCTCCCATCAGAGGACAAATTAACTGTTTATCAAAT---
ATTACAGGGCTACTATTAACAAGAGATGGTGGTAATATCAGTAGC---------------------------
----

ACTAATGAAACCTTCAGACCTGGAGGAGGAGATATGAGGGACAATTGGAGAAGTGAATTGTATAAATATAAGG
TAGTACAAATTGAACCATTA---GGATTAGCACCCACCAAGGCAAAGAGAAGAGTGGTA-------------
------------------------CAAAGAGAAAAAAGAGCA---GTGGGGACACTAGGAGCT---------
ATGTTCCTTGGGTTCTTGGGAGCA---GCAGGAAGCACTATG---GGCGCAGCGTCA---
ATAACGCTGACGGCACAG---GCCAGACAA---TTATTG---TCTGGTATAGTGCAACAGCAGAAC---
AACTTG---CTGAAG---GCTATTGATGCGCAACAGCATATGTTGCAACTCACAGTCTGGGGCATC
>KC699035
TTCTACTGTGATACAACAAAACTGTTTAATAGCACTTGGAATAGCACTTGGTATAGTAATAGTACTGGGAGTG
ATATTAAA--

GGAAATGACACAATCACACTCCCGTGCAGAATAAAACAAATTATAAACATGTGGCAGGAAGTAGGAAAAGCAA
TGTACGCCCCTCCCATCAGAGGATCAATTAAATGTTCATCAAAT---
ATTACAGGGTTGCTACTAACAAGAGATGGGGGTACAGACAATAGT---------------------------
----

AGTACTACAGAGACCTTCAGACCTGGAGGAGGAAATATGAAGGACAATTGGAGAAGTGAATTATATAAATATA
AAGTAGTACAAATTGAACCATTA---GGAGTAGCACCCACCAAGGCAAAGAGAAGAGTGGTG-----------
---------------------------CAGAGAGAAAAAAGAGCA---GTGGGA---CTAGGAGCT------
---ATGTTCCTTGGGTTCCTGGGAGCA---GCAGGAAGCACTATG---GGCGCAGCGTCA---
ATAACGCTGACGGTACAG---GCCAGACAA---TTATTG---TCTGGTATAGTGCAACAGCAAAAC---
AATTTG---CTGAGA---GCTATTGAGGCGCAACAACATCTGTTGCAACTCACAGTCTGGGGCATC
>KC699036
TTCTACTGTGAAACAGAAAAACTGTTTAATAGTACTTGGTGGAATAGTACTTGGAATAGTACTGAAGGGTCAA
ATAGT---

ACTGGGGTAAATATCACACTCCCATGCAGAATAAGACAAATTATAAACATGTGGCAGAAAGTAGGAAAAGCAA
TGTATGCCCCTCCCATCGCAGGACCAATTAGATGTGTATCAAAT---
ATCACAGGGATATTCTTAACAAGAGATGGTGGTAATGAGACCAGC---------------------------

----

AACGAGACCTTCAGACCTGGAGGAGGAGATATGAGGGACAATTGGAGAAGTGAATTATATAAATATAAAATAG
TAAAAATTGAGCCAATA---GGAGTAGCACCCACCAAGGCAAAGAGAAGAGTGGTG----------------
----------------------CAGAGAGAAAAAAGAGCA---GTGGGA---CTAGGAGCT--------
ATGTTCCTTGGGTTCTTGGGAGCA---GCAGGAAGCACTATG---GGCGCAGCATCA---
ATAACGCTGACGGTACAG---GCCAGACAA---TTACTG---TCTGGTATAGTGCAACAGCAGAAT---
AATCTG---CTGAGG---GCTATTGAAGCGCAACAACATATGTTGCAACTCACAGTCTGGGGCATA
>KF716494
TTCTACTGTAATACAACAGAACTATTTAATAGTACTTGGAAGATTGAGAATAATACTGGAATATTACTGAAC
AGGCAAAT--
-------------AACACT------
GCAAGAAATGACACAATCATACTCCCATGCAGAATAAAGCAAATTATAAACCTGTGGCAGGAAGTAGGAAAAG
CAATGTATGCCCCTCCCATTGAGGGACTAATTAACTGTACATCAAAT---
ATCACAGGGCTAATACTAACAAGGGATGGTGGCAATAACAATAGCACT------------------------
----

AATGAGACCGAGATCTTCAGACCTGGAGGAGGAAATATGAGGGACAATTGGAGAAGTGAATTATATAAATATA
AAGTAGTACAAATTGAACCAATA---GGACTAGCACCCACCAAGGCAAAGAGAAGAGTGGTG----------
----------------------------CAGAGAGAAAAAAGAGCA---CTGGGA---ATAGGAGCT-----
---GTGTTCCTTGGGTTCTTGGGAGCA---GCAGGAAGCACTATG---GGCGCAGCGTCG---
CTGACGCTGACGGTACAG---GCCAGGCAA---TTACTG---TCTGGTATAGTGCAACAGCAGAGC---
AATTTG---CTGAGA---GCTATTGAGGCGCAACAGCATATGTTGCAACTCACAGTCTGGGGCATT
>KF716495
TTCTACTGTAATACAACACAACTGTTTAATAGTACTTGGAATGCTACTAGCGAGTCAAGTGAC---------
----

CTAAATGACACAATCACACTCCCATGCAGAATAAAACAAATTATAAACATGTGGCAGAAAGTAGGAAAAGCAA
TGTATGCCCCTCCCATCAAAGGACTAATTAATTGTACATCAAAT---
ATCACAGGGCTCCTATTAACAAGAGATGGTGGTAACAAGGTTAAT---------------------------
----

GAGAGCAACCATGAGACCTTCAGACCGGGAGGAGGAAATATGAAGGACAATTGGAGAAGTGAATTATATAAAT
ACAAAATAGTAAAAATTGAACCATTA---GGAGTAGCCCCCACCAAGGCAAAGAGAAGAGTGGTG-------
------------------------------CAGAGAGAAAAAAGAGCA---GTGGGA---ATAGGAGCT---
------ATGTTCCTTGGGTTCTTGGGAGCA---GCAGGAAGCACTATG---GGCGCAGCGTCA---
GTGACGCTGACGGTACAG---GCCAGAAAA---TTATTG---TCTGGTATAGTGCAACAGCAGAAC---
AATTTG---CTGAGG---GCTGTTGAGGCGCAACAACGTATGTTGCAACTCACAGTCTGGGGCATT
>KC699031
TTCTACTGTGATACAACACAGCTATTTAATAGTACCTGGAATAGTACTACTTTGAATAATACTTGGAATAATA
CTTGG---
-------------AATAAT------
AGTGAAAATGACACTATCATACTCAGATGCAGAATAAAACAGATCATAAACAGGTGGCAGGAAGTAGGAAAAG
CAACGTATGCCCCTCCCATCAGTGGAGAAATTAGATGTACATCAAAT---
ATTACAGGGCTACTATTAACAAGAGATGGTGGTAAAAACAACAAC---------------------------
----

GACAACGAGACCTTCAGACCTGGAGGAGGAGATATGAGGGACAATTGGAGAAGTGAATTATATAAATATAAAG
TAGTAAAAATTGAGCCATTA---GGACTGGCACCCACCAAGGCAAAGAGAAGAGTGGTG-------------
-------------------------CAGAGAGAAAAAAGAGCA---GTGACA---ATGGGAGCT--------
TTGTTCCTTGGGTTCTTGGGAACA---GCGGGAAGCACTATG---GGCGCAGCGTCA---
GTGACGCTGACGGTACAG---GCCAGACTA---TTGTTG---TCTGGTATAGTGCAGCAGCAAAAC---
AATTTG---CTGAGG---GCTATTGAGGCGCAACAACATTTGTTGCAACTCACAGTCTGGGGCATC
>KC699034
TTCTATTGTAATGTAACACAACTGTTTAATAGTACTTGGCATGGTAATGAGACTGTGAATAGTACTGAA---
----

GAAGGAGAAGTCATCACACTTCCATGCAGAATAAAACAAATTATAAACCTGTGGCAGGAAGTAGGAAAAGCAA
TGTATGCCCCTCCCATCAGTGGAAAAATTAGATGTTCATCAAAT---
ATTACAGGGCTGTTATTAACAAGAGATGGTGGATACAACGGGGAC---------------------------

----

AACAATACTGAGACCTTCAGACCTGGAGGAGGAGATATGAGGGATAATTGGAGAAGTGAATTATACAAATATA
AAGTAGTAAAAGTTGAACCATTA---GGAATAGCACCCACCAAGGCAAAGAGAAGAGTGGTG-----------
-----------------------------CAGAGAGAAAAAAGAGCA---GTGGGA---GTAGGAGCT------
---ATGATCATTGGGTTCTTGGGAGCA---GCAGGAAGCACTATG---GGCGCAGCGTCA---
GTGACGCTGACGGTACAG---GCCAGACTA---TTGTTG---TCTGGTATAGTGCAACAGCAGAGC---
AACCTG---CTGAGG---GCTATTGAGGCGCAACAGCATATGTTGCAGCTCACAGTCTGGGGCATT
>KF716496
TTCTACTGTAATACAACACCACTGTTTGACAGTACCTGGAATGGGGGAGAGCCAGTGTCAAATAACACTGGAG
GA---

AATGACACAATAATCACACTCCAATGTAGAATAAAACAAATTATAAACATGTGGCAGGAAGTTGGAAAAGCAA
TGTATGCCCCTCCCATCAGAGGACAAATTAGATGCTCATCAAAT---
ATTACAGGGCTGATATTAACAAGAGATGGTGATCTTAACAATACC----------------------------
----

CAGAACGAGACCTTCAGACCTGGAGGAGGAGATATGAGGGACAATTGGAGAAGTGAATTATATAAATATAAAG
TAGTAAAAATTGAACCATTA---GGAGTAGCACCCACCAAGGCAAAGAGAAGAGTGGTG--------------
-------------------------CAGAGAGAGAAAAGAGCA---GTGGGA---ATAGGAGCT---------
CTGTTCCTTGGGTTCTTAGGAGCA---GCAGGAAGCACTATG---GGCGCAGCGTCA---
ATGACGCTGACGGTACAG---GCCAGACAA---CTATTG---TCTGGTATAGTGCAACAGCAGAAC---
AATCTG---CTAAGG---GCTATTGAGGCGCAACAACATATGTTGCAACTCACAGTCTGGGGCATC
>MH000302
TTCTACTGTAATACAACACAACTGTTTAATAGTACGTGGGCTTCG----------------------------
----

AATACTAACACTATCACACTCCCATGCAGAATAAAACAAATTATAAACATGTGGCAACAAGTAGGAAAAGCAA
TGTATGCCCCTCCCATCAGAGGAACAATTAAATGTTCATCAAAT---
ATTACAGGGCTACTATTATCAAGAGATGGAAGT---

ATCGAGACCTTCAGACCTGGAGGAGGAGATATGAGAGATAATTGGAGAAGTGAATTATATAAATATAAAGTAG
TAAAAGTTAAGCCATTA---GGAGTAGCACCCACCAGTGCAAAAAGAAGAGTGGTG-----------------
----------------------CAGAGAGAAAAAAGAGCA---ATAGGA---TTCGGAGCT---------
ATGTTCATTGGATTCTTGGGAGCT---GCAGGAAGCACTATG---GGCGCAGCGTCA---
GTGACGCTGACGGTACAG---GCCAGACAA---TTATTG---TCTGGTATAGTGCAACAGCAGAAT---
AATCTG---CTGAGG---GCTATTGAGGCGCAACAGCATCTGTTGCAACTCACAGTCTGGGGCATC
>MH000296
TTCTACTGCAATACAACAGCACTGTTTAATAGTACTTGGGTTATG----------------------------
----

AATGATTCCACTATCACACTCCCATGCAGAATAAAACAAATTATAAACAGATGGCAGGAAGTAGGAAAAGCAA
TGTATGCCCCTCCCATTGCAGGACCAATTAGGTGTTCATCAAAT---
ATTACAGGGCTGCTATTAACAAGAGATGGGGGA---

GAGGAGATCTTCAGACCAGGAGGAGGAGATATGAGGGACAATTGGAGAAGTGAATTATATAAGTATAAAGTAG
TGAAAATTGAACCATTA---GGAGTAGCACCCACCAAGGCAAAAAGAAGAGTGGTG-----------------
----------------------CAGAGAGAAAAAAGAGCA---ATAGGA---ATAGGAGCT---------
ATGTTCATTGGGTTCTTGGGAGCA---GCAGGAAGCACTATG---GGCGCAGCGTCA---
GTGACGCTGACGGTACAG---GCCAGACAA---TTATTG---TCTGGTATAGTGCAACAGCAGAAC---
AATTTG---CTGAGG---GCTATTGAGGCGCAACAACATCTGTTGCAACTCACAGTCTGGGGCATC
>MH000299
TTCTATTGTGATACATCACAACTGTTTAATAGTACTTGGAAT-------------------------------
----

AGTACTGTTAATATCACTCTCCCATGCAGATTAAAGCAATTTATAAACATGTGGCAGCAAGTAGGAAAAGCAA
TGTATGCCCCTCCCATCAGCGGACTAATCCAGTGCTCATCAAAT---
ATTACAGGGCTGCTGTTAGTAAGGATGGTGGT---

ACTGAAATCTTCAGACCTGGAGGGGGAGATATGAGGGACAATTGGAGAAGTGAACTATATAAATATAAAGTAG
TAAAGCTTGCACCATTA---GGAATAGCACCCACCAAGGCAAAGAGAAGAGTGGTG-----------------
----------------------CAGAGAGAAAAAAGAGCA---GTGGGA---CTAGGAGCT---------
ATGTTCCTCGGGTTCTTGGGAGCA---GCAGGAAGCACTATG---GGCGCAGCGTCA---
GTGACGCTGACGGTACAG---GCCAGACAA---TTATTG---TCTGGTATAGTGCAACAGCAGAGC---
AATCTG---CTGAGA---GCTATTGAGGCGCAACAACATATGTTGCAACTCACAGTCTGGGGCATA
>KC699002
TTCTACTGTAATTCAACACAACTGTTTAATAGTATCTGGAATGGTACTTGGAATGATACTTGGAATGGTACTA
GAGGGGTA---
-------------------AAT---
AACACTGAAGACAATATCATACTCCCATGTAGAATAAAACAAATTATAAACATGTGGCAGGAAGTAGGAAAAG
CAATGTATGCCCCTCCCATCAAAGGACGAATTATGTGTTCATCAAAT---
ATTACAGGGCTGCTATTAACAAGAGATGGTGGTAATAGTAACGCT----------------------------
----

ACCACCGAGATCTTCAGACCTGGAGGAGGAGATATGAGGGACAATTGGAGAAGTGAATTATATAAATATAAAG
TAGTAAAAATTAAACCATTA---GGAGTAGCACCTACCAAGGCAAAGAGAAGAGTGGTG--------------
-------------------------CAGAGAGAAAAAAGAGCA---GTGGGA---ATAGGAGCT---------
GTGTTCCTTGGGTTCTTGGGAGCA---GCAGGAAGCACTATG---GGCGCAGCGTCA---
ATGACGCTGACGGTACAG---GCCAGACGA---TTATTG---TCTGGTATAGTGCAACAGCAGAAC---
AATTTG---CTGAGG---GCTATTGAGGCGCAACAGCATCTGTTGCAACTCACAGTCTGGGGCATC
>KC699011
TTCTACTGTAACTCAACACAACTGTTTAATAGTACTTGGGTGTTTAATAATACTTGGAATAGAACTGAAGGGT
TAAATAAC---

ACTGCAGAAAATATCACACTCCCATGCAGAATAAAACAAATTGTAAATATGTGGCAGGAAGTAGGAAAAGCAA
TGTATGCCCCTCCCATCAGAGGACAAATTAGATGTTCATCAAAT---
ATTACAGGGCTGCTATTAACAAGAGATGGTGGTAATAACACGAGA----------------------------
----

AATAGCGAGACCTTCAGACCTGGGGGAGGAGATATGAGGGACAATTGGAGAAGTGAATTATATAAATATAAAG
TAGTACAAATTGAACCATTA---GGAGTAGCACCCACTAAGGCAAGAAGAAGAGTGGTG--------------
-------------------------CAGAGAGAAAAAAGAGCA---ATAACA---CTAGGAGCT---------
GTGTTCCTTGGGTTCTTGGGAGCA---GCAGGAAGCACTATG---GGCGCAGCGTCA---
GTGACGCTGACGGTACAG---GCCAGACAA---TTACTG---TCTGGTATAGTGCAACAGCAGAAC---
AATTTG---CTGAGG---GCTATTGAGGCGCAACAGCATCTGTTGCAACTCACAGTCTGGGGCATC
>KC699010
TTCTACTGTAATACAACACAACTGTTTAATAGCACTTGGAATAGTACTAGGATTGAAAATGATACTGAAGAGT
CAAATAGC---

ACTGAAAGAACGATCACACTCCCATGCAGAATAAGACAAATTATAAACAGGTGGCAGGAAGTAGGGAAAGCAA
TGTATGCCCCTCCCATCAGCGGACAAATTAGATGCTCATCAAAC---
ATTACAGGGCTGCTATTAACAAGAGATGGCGGTAATGACAAGAAAAACGAG----------------------
----

ACTACTGAGGTCTTCAGACCTGGAGGAGGAGATATGAAGGACAATTGGAGAAGTGAATTATATAAATATAAAG
TAGTAAAAATTGAACCATTA---GGAGTAGCACCCACTAAGGCAAAGAGAAGAGTGGTG--------------
-------------------------CAGAGAGAAAAAAGAGCA---GTGGGA---CTAGGAGCT---------
GTGTTCCTTGGGTTCTTGGGAGCA---GCAGGAAGCACTATG---GGCGCAGCGTCA---
ATGACGCTGACGGTACAG---GCCAGACTA---TTATTG---TCTGGTATAGTGCAACAGCAGAAC---
AATTTG---CTGAGG---GCTATTGAGGCGCAACAGCATCTGTTGCAACTCACAGTCTGGGGCATC
>KC699009
TTCTACTGTAATTCAACACAACTGTTTAATAGTACTTGGAATGGTACTAATAGTACTTGGAAT----------
----

GGTACTGAAAGTATCACACTCCCATGCAGAATAAAACAATTTATAAACATGTGGCAGGAAGTAGGAAAAGCAA
TGTATGCCCCTCCCATCAAAGGACAAATTAGATGTTCATCAAAT---
ATTACAGGGCTGCTATTAACAAGAGATGGTGGTAATAACAATAAC----------------------------

----

GAGAGCGAGACCTTCAGACCTGGAGGAGGAGATATGAGGGACAATTGGAGAAGTGAATTATACAAATATAAAG
TAGTAAAAATTGAACCATTA---GGAGTAGCACCCACCAAGGCAAAGAGAAGAGTGGTG--------------
-------------------------CAGAGAGAAAAAAGAGCA---GTGGGA---ATAGGAGCT---------
TTGTTCCTTGGGTTCTTGGGAGCA---GCAGGAAGCACTATG---GGCGCAGCGTCA---
ATAACGCTGACGGTACAG---GCCAGACTA---TTATTG---TCTGGTATAGTGCAACAGCAAAAC---
AATTTG---CTGAGG---GCTATTGAGGCGCAACAGCATCTGTTGCAACTCACAGTCTGGGGCATC
>KC699008
TTCTACTGTAATTCAACACCACTATTTAATAGTACTTGGCATAATAATGACACTGAAGGAAATAACATC----
----

ACTAACGGAAACATCACACTCCCATGCAGAATAAAACAAATTATAAACATGTGGCAGGAAGTAGGAAAAGCAA
TGTATGCCCCTCCCATCAAAGGACAAATTAGATGCTCATCAAAT---
ATTACAGGGCTGCTATTAACAAGAGATGGAGGTAATAATAACAATAGT-------------------------
----

ACCAATGACACTGAGATCTTCAGACCTGGAGGGGGAGATATGAGGGACAATTGGAGAAGTGAATTATATAAAT
ATAAAGTAGTAAAAATTGAACCATTA---GGAGTAGCACCCACCAAGGCAAAGAGAAGAGTGGTG--------
-------------------------------CAGAGAGAAAAAAGAGCA---GTGGGAACGTTGGGAGCC---
------ATGTTCCTTGGGTTCTTGGGAGCA---GCAGGAAGCACTATG---GGCGCAGCGTCA---
ATGACGCTGACGGTACAG---GCCAGACAA---TTATTG---TCTGGTATAGTGCAACAGCAGAAC---
AATCTG---CTGAGG---GCTATTGAGGCGCAACAGCATATGTTGCAACTCACAGTCTGGGGCATC
>KC699003
TTCTACTGTAACACAACAAAACTGTTTAATAGTACTTGGAATGTTGCTGAAGGGTCAAATAAC----------
----

ACTGAAGGAAATATCACACTCCCATGCAGAATAAAACAAATTATAAACATGTGGCAGGAAGTAGGAAAGGCAA
TGTATGCCCCTCCCATCAGAGGACAAATTAGATGTGTATCAAAT---
ATTACAGGGCTGCTATTAACAAGAGATGGTGGTAATAACAGGAATGGG-------------------------
----

ACCAACGGGACCGAGATCTTCAGACCTGGAGGAGGAGATATGAGGGACAATTGGAGAAGTGAATTATATAAAT
ATAAAGTAGTAAAAATTGAACCATTA---GGAATAGCACCCACCAAGGCAAAGAGAAGAGTGGTG--------
-------------------------------CAGAGAGAAAAAAGAGCA---GTCACA---TTAGGAGCT---
------ATGTTCCTTGGGTTCTTGGGAGCA---GCAGGAAGCACTATG---GGCGCAGCGTCA---
ATAACGCTGACGGTACAG---GCCAGACTA---TTATTG---TCTGGTATAGTGCAACAGCAGAGC---
AATTTG---CTGAGA---GCTATTGAGGCGCAACAACATTTGTTGCAACTCACAGTCTGGGGCATC
>KC699004
TTCTACTGTAATACAACAAAACTGTTTAATAGTACTTGGGCTTGGAATAATGGTACTTGGAATAGTACTGAAG
GGTCAAAT---

AACACAGCAAATATCACACTCTCATGCAGAATAAGACAAATTATAAACATGTGGCAAGAAGTAGGAAAAGCAA
TGTATGCCCCTCCCATCAGCGGACAAATTAAATGCTCATCAAAT---
ATTACAGGGCTGCTGTTAACAAGAGATGGTGGTACTAACAATAGC----------------------------
----

GAGACCGAGACTTTCAGACCTGGAGGAGGAGATATGAGGGACAATTGGAGAAGTGAATTATACAAATATAAAG
TAGTAAAAATTGAACCATTA---GGAGTAGCTCCCACCAAGGCAAAGAGAAGAGTGGTG--------------
-------------------------CAGAGAGAAAAAAGAGCA---GTGGGA---ATAGGAGCT---------
TTGTTCCTTGGGTTCTTGGGAGCA---GCAGGAAGCACTATG---GGCGCAGCATCA---
ATGACGCTGACGGTACAG---GCCAGACAA---TTACTG---TCTGGTATAGTGCAACAGCAGAAC---
AATTTG---CTGAGG---GCTATTGAGGCGCAACAGCATATGTTGCAACTCACAGTCTGGGGCATC
>KC699005
TTCTACTGTAATACAACACAACTGTTTAATAGTACTTGGAATGATACTAAAGGGCCAGATAGCACTGACAATA
GTACTTGG---

ATCATACTACCATGCAGAATAAAACAAATTATAAACATGTGGCAGGAAGTAGGAAAAGCAATGTATGCCCCTC
CTATCAGAGGACAAATTAGATGTTCATCAAAT---
ATTACAGGGCTACTATTAACAAGAGATGGTGGGGAAACAAGAAC-----------------------------

----

GAGAGCACCGAGACCTTCAGACCTGGAGGAGGAGATATGAGGGACAATTGGAGAAGTGAATTATATAAATATA
AAGTAGTACAAATTGAACCATTA---GGAGTAGCACCCACCAAGGCAAAGAGAAGAGTGGTG-----------
----------------------------CAGAGAGAAAAAAGAGCA---GTGGGA---ATAGGAGCT------
---GTGTTCCTTGGGTTCTTGAGCGCA---GCAGGAAGCACTATG---GGCGCAGCGTCG---
GTGACGCTGACGGTACAG---GCCAGACAA---TTATTG---TCTGGTATAGTGCAGCAGCAAAAC---
AATTTG---CTGAGG---GCTATTGAGGCGCAACAACATCTGTTGCAACTCACAGTCTGGGGCATC
>KC699001
TTCTACTGTGATTCAACACAACTGTTTAATAGTACTTGGAATAGTACTACTTGGAATAGTACTGAAGGGTTAA
ATAAC---

ACTGAAGGCAATATCACACTCCAATGCAGAATAAAACAAATTATAAACATGTGGCAGGAAGTAGGAAAAGCAA
TGTATGCTCCTCCCATCAGAGGACATATTAAATGTTCATCAAAT---
ATTACAGGGCTGTTATTAACAAGAGATGGTGGTAATAACGAGAGTAGA-------------------------
----

ACCAACGAGACCTTTAGACCTGAAGGAGGAAATATGAAAGACAATTGGAGAAGTGAATTATATAAATATAAAG
TAGTAAAAATTGAACCATTA---GGAATAGCACCCACCAAGGCAAAGAGAAGAGTGGTG--------------
-------------------------CAGAGAGAAAAAAGAGCA---GTGGGA---TTAGGAGCT---------
GTGTTCCTTGGGTTCTTGGGAGCA---GCAGGAAGCACTATG---GGCGCAGCGTCA---
CTGACGCTGACGGTACAG---GCCAGAACA---TTATTG---TCTGGTATAGTGCAACAGCAGAAC---
AATCTG---CTGAGG---GCTATTGAGGCGCAACAGCACCTGTTGCAACTCACAGTCTGGGGCATC
>AY535425
TTCTACTGTAATACAACACAACTGTTTAATAGTACTTGGATGCTTAATAATACTTGGGATGGTACT-------
----

AAAGAGGAAAATATTACACTCCCATGCAGAATAAAACAAATTATAAACATGTGGCAGGAAGTAGGAAAAGCAA
TGTATGCCCCTCCCATCAAAGGACAAATTAGATGTTCATCAAAC---
ATTACAGGGCTGCTATTAACAAGAGATGGTGGTGGTAACACGAGC----------------------------
----

AGCGATAATGAGACCTTCAGACCTGGAGGAGGAGATATGAGGGATAATTGGAGAAGTGAATTATATAAATATA
AAGTAGTAAAAATTGAACCATTA---GGAGTAGCACCCACCAAGGCAAGGAGAAGAGTGGTG-----------
----------------------------CAGAGAGAAAAAAGAGCA---GTGGGAATGCTAGGAGCC------
---ATGTTCCTTGGGTTCTTGGGAGCA---GCAGGAAGCACTATG---GGCGCAGCGTCA---
CTGGCGTTGACGGTACAG---GCCAGACAA---GTATTG---TCTGGTATAGTGCAACAGCGGAAC---
AATTTG---CTGAGG---GCTATTGAGGCGCAACAGCATCTGTTGCAACTCACAGTCTGGGGCATC
>AY713411
TTCTACTGTAATACAACACAACTGTTTAATAGTACTTGGAATGAAACTAACAGTGAAGGAAATATC-------
----

ACCAGTGGAACTATAACACTCCCATGCAGAATAAAACAAATTATAAACATGTGGCAGGAAGTAGGAAAAGCAA
TGTATGCCCCTCCCATCGGAGGACAAATTAAATGTTTGTCAAAC---
ATCACAGGGCTGTTATTAACAAGAGATGGTGGTAGTGATAACAGTAGT-------------------------
----

AGTGGGAAAGAGATCTTCAGACCTGGAGGGGGAGATATGAGGGACAATTGGAGAAGTGAATTATATAAATATA
AGGTAGTAAAAATTGAACCATTA---GGAATAGCACCCACCAAGGCAAAGAGAAGAGTGGTG-----------
----------------------------CAGAGAGAAAAAAGAGCA---GTGGGA---ATAGGAGCC------
---ATGTTCCTTGGGTTCTTGGGAGCA---GCAGGAAGCACTATG---GGCGCAGCGTCA---
CTAACGCTGACGGTACAG---GCCAGACAA---TTATTG---TCTGGTATAGTGCAGCAGCAAAAC---
AATTTG---CTGAGG---GCTATTGAGGCGCAACAGCACCTGTTGCAACTCACAGTCTGGGGCATC
>AF041125
TTCTACTGTAACTCAACAAAACTGTTTAACAGTACTTGGAGTGCTAATGGTACTGTCATGCCAAATGACACTG
AA--

GGGAATGGCACAATCATGCTCACATGCAGAATAAAACAAATTGTAAACAGGTGGCAGGAAGTAGGAAAAGCAA
TGTATGCCCCTCCCATCAGAGGACAAATTAGATGTTCATCAAAT---
ATTACAGGGCTAATATTAACAAGAGGTGGTGGTGATAAAGAGAAC----------------------------

----

GAGACTACTGAGACCTTCAGACCTGGAGGAGGAAATATGAGGGATAATTGGAGAAGTGAATTATATAAATATA
AAGTAGTAAAACTCGAACCATTA---GGGATAGCACCCACCAAGGCAAAGAGAAGAGTGGTG-----------
-----------------------------CAGAGAGAAAAAAAGAGCA---GTGGGA---ATAGGAGCT------
---TTGTTCCTTGGGTTCTTGGGAGCA---GCAGGAAGCACTATG---GGCGCAGCGTCA---
CTGACGCTGACGGTACAG---GCCAGACTA---TTATTG---TCTGGTATAGTGCAACAGCAGAAC---
AATTTG---CTGAGA---GCTATTGAGGCGCAACAGCATCTGTTGCAACTCACAGTCTGGGGCATC
>AY535471
TTTTACTGTAATACAACACAACTGTTTAATAGTACTTGGAATGCTAACCATATTAGGAATGTTACTAGAGGGT
CAAATAGA---
-------------ACAACT------
GGAGGAAATGACACACTCATACTCCCATGTAGAATAAAACAAATTATAAATATGTGGCAGGAAGTAGGAAAAG
CAATGTATGCCCCTCCCGTCAGAGGACAAATTAGATGTTCATCAAAT---
ATTACAGGGTTGCTATTAACAAGAGATGGTGGTAATAACGGGAATGAG---------------------------
----

ACCAACGGGACTGAGATCTTCAGACCTGGAGGGGGAGATATGAGGAACAATTGGAGAAGTGAATTATATAAAT
ATAAAGTAGTAAAAATTGAACCATTA---GGAGTACCACCCACCAAGGCAAGGAGAAGAGTGGTG--------
--------------------------------CAGAGAGAAAAAAAGAGCA---GTGACA---CTAGGAGCT---
------ATGTTCCTTGGGTTCTTAGGAGCA---GCAGGAAGCACTATG---GGCGCAGCGTCA---
ATGACGCTGACGGTACAG---GCCAGACAA---TTATTG---TCTGGTATAGTGCAACAGCAAAGC---
AATTTG---CTGAGG---GCTATTGAGGCGCAACAGCATCTGTTGCAACTCACAGTCTGGGGCATC
>AF041130
TTCTACTGTAATACAACACAGCTGTTTAATAGTACTTGGCTGGTTAATAGTACTGGGGATGATACTAAT----
----

ACTACCACCAATATCACACTCCCATGCAGAATAAAACAAATTATAAACATGTGGCAGAAAGTAGGAAAAGCAA
TGTATGCCCCTCCCATCAGAGGACAAATTAGATGTTTATCAAAT---
ATTACAGGGATACTATTAACAAGGGATGGTGGTAATAACACAAAC------------------------------
----

GGGACCGAGATCTTCAGACCTGGAGGAGGAGATATGAGGGATAATTGGAGAAGTGAATTATATAAATATAAAG
TAGTAAAAATTGAACCATTG---GGAATAGCACCCACCAAGGCAAAGAGAAGAGTGGTG-------------
--------------------------CAAAGAGAAAAAAAGAGCA---GTGGGA---TTAGGAGCT---------
ATGTTCCTTGGGTTCTTGGGAGCA---GCAGGAAGCACTATG---GGCGCAGCGTCA---
CTGACGCTGACGGTACAG---GCCAGACAA---TTATTG---TCTGGTATAGTGCAACAGCAGAAC---
AATTTG---CTGAGA---GCTATTGAGGCGCAACAGCATCTGTTGCAACTCACAGTCTGGGGCATC
>AF041131
TTCTACTGTAATACAACACGACTGTTCAATAGTACTTGGAATGCTAGTAACATTGAAGAAAATAGCACCACT-
----

GAAGGAAATATCACCACTATCCCATGCAGAATAAAACAAATTATAAACATGTGGCAGGAAGTAGGAAAAGCAA
TGTATGCCCCTCCCATCAGAGGACAAATTAGATGTTCATCAAAT---
ATTACAGGGCTGCTATTAATAAGAGATGGTGGTAATGATAACAAT------------------------------
----

AATGACACCGAGATCTTCAGACCTGGAGGGGGAGATATGAGGGACAATTGGAGAAGTGAATTATATAAATATA
AAGTAGTAAAAATTGAACCATTA---GGAATAGCACCCACCAAGGCAAAGAGAAGAGTGGTG-----------
-----------------------------CAGAGAGAAAAAAAGAGCA---GTGGGAACGCTAGGAGCC------
---ATGTTCCTTGGGTTCTTGGGAGCA---GCAGGAAGCACTATG---GGCGCAGCGTCA---
ATGACGCTGACGGTACAG---GCCAGACAA---TTATTG---TCTGGTATAGTGCAACAGCAGAGT---
AATCTG---CTGAGG---GCTATTGAGGCGCAACAGCATCTGTTGCAACTCACAGTCTGGGGCATC
>AF041134
TTCTACTGTAATACAACCCAACTGTTTGATAGTATTTGGAATGATACTGAAGGGTCATATAACACTGAA----
----

GGAAATGACACAATCACTCTCCCATGCAGGATAAAACAAATTATAAACTTGTGGCAGGAAGTAGGAAAAGCAA
TGTATGCCCCTCCCATCAGAGGACAAATTAGATGTTCATCAAAT---
ATTACAGGGCTGCTATTAACAAGAGATGGTGGGAACGAGAGCGAA------------------------------

```
-------------------------------------------------------------------------
-------------
GCCACCGAGATCTTCAGACCTGGAGGAGGAGATATGAGGGACAATTGGAGAAGTGAATTATATAAATATAAAA
TAGTAAAAATTGAACCATTA---GGAGTAGCACCCACCAAGGCAAAGAGAAGAGTGGTG--------------
-------------------------CAAAGAGAGAAAAGAGCA---GTGGGA---TTAGGAGCT---------
TTGTTCCTTGGGTTCTTGGGAGCA---GCAGGAAGCACTATG---GGCGCAGCGTCA---
ATAACGCTGACGGTACAG---GCCAGACAA---TTATTG---TCTGGTATAGTGCAACAACAGAAC---
AATTTG---TTGAGG---GCTATTGAGGCGCAACAGCATCTGTTGCAACTCACAGTCTGGGGCATC
>AF041133
TTCTACTGTGATTCAACACAACTATTTAATAGTACTTGGAGTATTAATAGTACTTGGAATAGTACTGAA----
-------------------------------------------------------------------------
----------------------------
ATAACAGATAATATCACACTCCCATGCAGAATAAAACAAATTATAAACATGTGGCAGGAAGTAGGGAAAGCAA
TGTATGCCCCTCCCATCAGAGGACAAATTAGATGTTCATCAAAT---
ATTACAGGGCTGCTATTAACAAGAGATGGTAGTGGCACGAATGAGACG-------------------------
-------------------------------------------------------------------------
----------
AACGGGACTGAGACCTTCAGACCGGGAGGAGGAGATATGAGGGACAATTGGAGAAGTGAATTATATAAATATA
AAGTAGTAAAAATTGAACCATTA---GGAGTAGCACCCACCAAGGCAAAGAGAAGAGTGGTG-----------
----------------------------CAGAAAGAAAAAAGAGCA---GTGGGG---ATAGGAGCT------
---GTGTTCCTTGGGTTCTTGGGAGCA---GCAGGAAGCACTATG---GGCGCAGCGTCA---
CTGACGCTGACGGTACAG---GCCAGACTA---TTATTG---TCTGGTATAGTGCAACAGCAGAAC---
AATTTG---CTGAGG---GCTATTGAGGCGCAACAGCATCTGTTGCAACTCACAGTCTGGGGCATC
>KC699014
TTCTACTGTAATACAACACCACTGTTTAATAGTACTTGGACTTTTAATGGTACTACTTGGAACACAACTAACA
CTGAA--------------------------------------------------------------------
----------------------------
GGAAATGACACAATCACACTCCAATGTAAAATAAAGCAAATTATAAACATGTGGCAGGAAGTAGGAAAAGCAA
TGTATGCCCCTCCCATCAAAGGACTAATTAAATGTTCATCAAAT---
ATTACTGGGTTGCTACTAACAAGGGATGGTGGTAGTAATACTACC----------------------------
-------------------------------------------------------------------------
----------
AATACCACCGAGACCTTCAGACCTGGAGGAGGAGATATGAAGGACAATTGGAGAAGTGAATTATATAAATATA
AAGTAGTAAAAATTGAACCATTA---GGAGTAGCACCCACCAAGGCAAAGAGAAGAGTGGTG-----------
----------------------------CAGAGAGAAAAAAGAGCA---GTAACA---CTAGGAGCT------
---ATGTTCCTTGGGTTCTTGGGAGCA---GCAGGAAGCACTATG---GGCGCAGCGTCA---
ATAACGCTGACGGTACAG---GCCAGACAA---TTATTG---TCTGGTATAGTGCAACAGCAGAAC---
AATTTG---CTGAGG---GCTATTGAGGCGCAACAACATCTGTTGCAACTCACAGTCTGGGGCATC
>KC699016
TTCTACTGTGATACAACAAAACTGTTTAGTAGTACTTGGAATAGGACTGAGACTACTTGGAATATTGCTGAAG
GGTTA--------------------------------------------------------------------
-------------------AAT---
GAGACTGAAACCACCATCACACTCCAATGCAGAATAAAACAAATTGTAAACAAGTGGCAGGAAGTAGGAAAAG
CAATGTATGCCCCTCCCATCAAAGGACAAATTAAATGCTCATCAAAT---
ATTACAGGGCTGCTCTTAACAAGAGATGGTGGGAGCGGGAACGAGACCGAG----------------------
-------------------------------------------------------------------------
----------
AACGGGACCGAGACTTTCAGACCTGGAGGAGGAGATATGAGGGACAATTGGAGGAGTGAATTATACAAATATA
AAGTAGTACAAATTGAACCATTA---GGAGTAGCACCCACCAAGGCAAAGAGAAGAGTGGTG-----------
----------------------------CAGAGAGAAAAAAGAGCA---GTGGGA---ATAGGAGCT------
---ATGTTCCTTGGGTTCTTGGGAGCA---GCAGGAAGCACTATG---GGCGCAGCGTCA---
ATGATGCTGACGGTACAG---GCCAGACAA---TTATTG---TCTGGTATAGTGCAACAGCAGAAC---
AATTTG---CTGAGG---GCTATTGAGGCGCAACAGCATATGTTGCAACTCACAGTCTGGGGCATT
>KC699017
TTCTACTGCAATACAACACAGCTGTTTAACAGTACTTGGTATAATAATAGTGCTTGGAATGGTACTGCACAGG
AAAAT--------------------------------------------------------------------
----------------------------
AGCACTGAAATTATCACACTCCCATGCAGAATAAAACAGATTATAAACAGGTGGCAGGAAGTAGGAAGAGCAA
TGTATGCCCCTCCCATCGCAGGACAAATTAGATGTTCATCAAAC---
ATTACAGGACTGCTATTAACAAGAGACGGTGGAAACTCCAATAAT----------------------------
```


AGTGAAAATGAAACCTTTAGACCTGGAGGAGGAGATATGAGAGACAATTGGAGAAGTGAGTTATATAAATATA
AAGTAGTACAAATTAAACCATTA---GGAGTAGCACCTACCATGGCAAAGAGAAGAGTGGTA-----------
----------------------------CAGAGAGAAAAAAGAGCA---GTGGGAATGCTAGGAGCT------
---ATGTTCCTTGGGTTCTTGGGAGCA---GCAGGAAGCACTATG---GGCGCAGCGTCA---
ATGACGCTGACGGTACAG---GCCAGACAA---TTATTA---TCTGGTATAGTGCAACAGCAAAAC---
AATTTG---CTGAGG---GCTATTGAGGCGCAGCAGCATCTGTTGCAACTCACAGTCTGGGGCATC
>KC699012
TTCTACTGTAATTCAACAGAACTGTTTAATAGTACTTGGACTTGGAATGATACTAGGGGGTTAAATAACACTG
AAGGA--

AATAGCACAATAATCAAACTCCCATGCAGAATAAAACAAATTATAAACATGTGGCAGGAAGTAGGAAAAGCAA
TGTATGCCCCTCCCATCAGAGGACAAATTAACTGTTTATCCAAT---
ATTACAGGGCTGCTATTAACGAGGGATGGTGGTATTATTACCACAAATGGGTCC-------------------
----

GAGAACGAGACCTTCAGACCTGGAGGAGGAGATATGAGGGACAATTGGAGAAGTGAATTATACAAATATAAAG
TAGTAAAAATTGAACCATTA---GGATTGGCACCCACCAAGGCAAAGAGAAGAGTGGTG--------------
-------------------------CAGAGAGAAAAAAGAGCA---GTGGGA---ATAGGAGCT---------
TTGTTCCTTGGGTTCTTGGGAGCA---GCAGGAAGCACTATG---GGCGCAGCGTCA---
ATAACGCTGACGGTACAG---GCCAGACAA---TTATTT---TCTGGTATAGTGCAGCAGCAGAAT---
AATCTG---CTGAGG---GCTATTGAGGCGCAACAGCATCTGTTGCAACTCACAGTCTGGGGCATC
>KC699020
TTCTACTGTGATACAACACAACTGTTTAATAGTACTTGGAATGATACATGGTCAAATAACACAAATGAT----
----

ATTGCAGAAAATATTACACTCCCATGCAGAATAAAACAAATTATAAACATGTGGCAGGAAGTAGGAAAAGCAA
TGTATGCCCCTCCCATCAGAGGACAAATTAGATGTATATCAAAT---
ATTACCGGGCTGCTATTAACAAGAGATGGTGGCAAT-------------------------------------
----

AATGAGACCGAGGTCTTCAGGCCAGGAGGAGGAGATATGAGGGACAATTGGAGAAGTGAATTATATAAATATA
AAGTGGTACAAATTGAACCATTA---GGAATAGCACCCACCAAGGCAAAGAGAAGAGTGGTG-----------
----------------------------CAGAGAGAGAAAAGAGCA---GTGGGA---ATAGGAGCT------
---GTGTTCCTTGGGTTCTTGGGAGCA---GCAGGAAGCACTATG---GGCGCAGCATCA---
GTGATGCTGACGGTACAG---GCCAGACAA---TTATTG---TCTGGTATAGTGCAACAGCAAAAC---
AATTTG---CTGAGA---GCTATTGAGGCGCAACAACATCTGTTGCAACTCACAGTCTGGGGCATT
>KC699026
TTCTACTGTAATACATCAGGACTGTTTAATAGTACTTGGATTTGGAATGATACTACTTGGAATGATACTATGG
GGTCC--
-------------------AAT---
GACACAAAGGAAAATATCACACTCATATGCAGAATAAAACAAATTGTCAACATGTGGCAGGAAGTAGGAAAAG
CAATGTATGCCCCTCCCATCAGGGGACTAATTAGATGTTCATCAAAT---
ATTACAGGGCTGCTATTAACAAGAGATGGTGGTAATAATAATGAAAGC-------------------------
----

AACAACGACACTGAGACTTTCAGACCTGGAGGAGGAGATATGAGGGACAATTGGAGAAGTGAATTATATAAAT
ATAAAGTAGTAAAGATTGAACCCTTA---GGAGTAGCACCTACCAGGGCAAAGAGAAGAGTGGTG--------
-------------------------------CAGAGAGAAAAAAGAGCA---GTAACG---CTGGGAGCT---
------TTGTTCCTTGGGTTCTTGGGAGCA---GCAGGAAGCACTATG---GGCGCAGCGTCA---
ATGACGCTGACGGTACAG---GCCAGACAA---TTGTTG---TCTGGTATAGTGCAGCAGCAGAGT---
AATCTA---CTGAGA---GCTATTGAGGCGCAACAACATCTGTTGCAACTCACAGTCTGGGGCATC
>KC699015
TTCTACTGTAATACAACAAAACTGTTTAATAGTACTTGGACTTGGAATAATACTGAAGAGGCAAATAACAATA
GTACT--
-------------GACGGG------
GCAAATGACACTATCATCACACTTCCATGCAGAATAAAACAAATTATAAACATGTGGCAGACAGTAGGAAAAG
CAATGTATGCCCCTCCCATCAAAGGAAAAATTGAATGCTCATCAAAT---
ATTACAGGGCTGCTATTAACAAGAGATGGTGGTGAAAACAACACT----------------------------

----

ACCAAAGAGATCTTCAGACCTGGAGGAGGAGATATGAGGGACAATTGGAGAAGTGAATTATATAAATATAAAG
TAGTAAAAATTGAACCATTA---GGAGTAGCACCCACCAAGGCAAAGAGAAGAGTGGTG--------------
-------------------------CAGAGAGAAAAAAGAGCA---GTGGGA---ATAGGAGCT---------
TTGTTCCTTGGGTTCTTGGGAGCA---GCAGGAAGCACTATG---GGCGCAGCGTCA---
ATGACGCTGACGGTACAG---GCCAGACAA---TTATTG---TCTGGTATAGTGCAACAGCAGAAC---
AATCTG---CTGAGA---GCTATTGAGGCGCAACAGCATATGTTGCAACTCACAGTCTGGGGCATC
>KC699013
TTCTACTGTAATACAGCAGAACTGTTTAATAGTACTTGGAATGTAAGTGGTACTGAAGAGTTAAATAAC----
----

ACTGAAAAAATAATCACACTCCCATGCAGAATAAGACAAATTATAAACAAGTGGCAGGAAGTAGGAAAAGCAA
TATATGCCCCTCCCATTAGGGGACAAATTAGATGCTCATCAAAC---
ATTACAGGGCTGCTATTAACAAGAGATGGTGGGAACGAGAACGGGACC-------------------------
----

AACGGGACCACCGAGGTCTTCAGACCTGGAGGAGGAAATATGAAGGACAATTGGAGGAGTGAATTATATAAAT
ATAAAGTAGTAAAAATTGAACCATTA---GGAGTAGCACCCACCAGGGCAAAGAGAAGAGTGGTG--------
-------------------------------CAGAGAGAAAAAAGAGCA---GCGGGA---ATAGGAGCT---
------CTGTTCCTTGGGTTCTTGGGAGCA---GCAGGAAGCACTATG---GGCGCAGCGTCA---
CTGACGCTGACGGTACAG---GCCAGACAA---TTATTG---TCTGGTATAGTGCAACAGCAGAAC---
AATTTG---CTGAGG---GCTATTGAGGCGCAACAGCATATGTTGCAACTCACAGTCTGGGGCATC
>KC699021
TTCTACTGTAATACAACACAACTGTTTAATGGTACTTGGAATTGGACTGATAATAGCTGGCCAAATGACACAA
AT---

ATTAATGACACTATCACACTCCCATGCAGAATAAGACAAATTATAAATATGTGGCAGGGAGTAGGAAAAGCAA
TGTATGCCCCTCCTATCAGCGGATACATTAACTGTTCATCAAAT---
ATTACAGGGCTGATATTAACAAGAGATGGTGGTAACACAAACACATCA-------------------------
----

AATGGGACCGAGACCTTCAGACCTGGAGGAGGAAATATGAAAGACAATTGGAGAAGTGAATTATATAAATATA
AAGTAGTAAAAATTGAACCATTA---GGAATAGCACCCACCAGGGCAAAAAGAAGAGTGGTG-----------
----------------------------CAAAGAGAAAAAAGAGCA---GTGGGA---CTAGGAGCT------
---TTGTTCCTTGGGTTCTTGGGAGCA---GCAGGAAGCACTATG---GGCGCAGCGTCA---
GTGACGCTGACGGTACAG---GCCAGACAA---TTAATG---TCTGGTATAGTGCAACAGCAGAAC---
AATCTG---CTGAAG---GCTATTGAGGCGCAACAGCGTATGTTGCAGCTCACAGTCTGGGGCATC
>KF695111
TTCTACTGTAACTCAACAAAACTGTTTAATAGAACTTGGTCTAATGGTACTTGGGATGATGCTTGGAATGATA
CT---
-------------------ACA---
GACGTTAACGGAACGATCATACTCCCATGCAGAATAAAACAAATTATAAACATGTGGCAGAAAGTAGGAAAAG
CAATGTATGCCCCTCCCATCAGAGGAATAATTAACTGTACATCAAAT---
ATTACAGGGCTGATATTAACAAGAGATGGTGGTGAATAATACA------------------------------
----

AACGAGACCTTCAGACCTGGAGGGGGAGATATGAGGGACAATTGGAGAAGTGAATTATATAGATATAAAGTAG
TAGAAATTGAACCATTA---GGAGTAGCACCCACCAAGGCAAAAAGAAGAGTGGTG-----------------
----------------------CAGAGAGAAAAAAGAGCA---GTGCCA---CTAGGAGCT---------
ATGTTCCTTGGGTTCTTAGGAGCA---GCAGGAAGCACTATG---GGCGCAGCGTCA---
ATAGCGCTGACGGCACAA---GCCAGACAA---TTATTG---TCTGGTATAGTGCAGCAGCAGAAC---
AATCTG---CTGAGG---GCTATTGAGGCGCAACAGCATCTGTTGCAACTCACAGTCTGGGGCATC
>EF033658
TTCTACTGTAATACAACAGAACTGTTTAATAGTACCTGGAATGATACTGAAAGGGTAGATGACACCGAT----
----

AGGGGTGACAATATCAAACTTCCATGCAGAATAAAACAAATTGTAAACAGGTGGCAGGAAGTAGGAATAGCAA
TGTATGCCCCTCCCATCGAAGGAAATATTAGATGTTCATCAAAT---
ACTACAGGACTGCTATTAACAAGAGATGGGGGTAATGATAACACGACA-------------------------

```
-------------------------------------------------------------------------
-------
GCTAACAAGACTGAGACCTTCAGACCTGGAGGAGGAGATATGAGGGACAATTGGAGAAGTGAATTATATAAAT
ATAAAGTAGTAAAAATTGAACCATTA---GGAGTAGCACCCACCCAGGCAAAAAGAAGAGTGGTG--------
-------------------------------CAGAGAGAAAAAAGAGCA---GTGACA---CTAGGAGCT---
------ATGTTCCTTGGGTTCTTGGGAGCA---GCAGGAAGCACTATG---GGCGCAGCGTCA---
ACGGTGCTGACGGTACAG---GCCAGACAA---TTATTG---TCTGGTATAGTGCAACAGCAGAAT---
AATCTG---CTGAGA---GCTATTGAGGCGCAACAGCATCTGTTGCAACTCACAGTCTGGGGCATC
>AF321080
TTCTACTGTGATACAACACAGCTGTTTAATAGTACTTGGAATGTTACTTGGAATGAAACTGGAGAGTCAAAT-
-------------------------------------------------------------------------
----------------------------
AGCACTGGAAATATCACACTCCCATGCAGAATAAGACAAATTATAAACAGGTGGCAGGAAGTAGGAAAAGCAA
TGTATGCTCCTCCCATCAAAGGACAAATTAGATGTTCATCAAAC---
ATTACAGGGCTGCTATTAACAAGAGACGGTGGTGCTGAGAACGCC----------------------------
-------------------------------------------------------------------------
-------------
ACCACCGAGACCTTTAGACCTGGAGGAGGAGATATGAGAGACAATTGGAGAAGTGAGTTATATAAATATAAAG
TAGTGCAAATTAAGCCATTA---GGAGTAGCACCCACCAGGGCAAAGAGAAGAGTGGTG--------------
-------------------------CAGAGAGAAAAAAGAGCA---GTGGGAACGCTAGGAGCT---------
ATGTTCCTTGGGTTCTTGGGAGCA---GCAGGAAGCACTATG---GGCGCAGCGTCA---
ATGACGCTGACGGTACAG---GCCAGACAA---TTATTG---TCTGGTATAGTGCAACAGCAAAAC---
AATTTG---CTGAGA---GCTATTGAGGCGCAACAGCATCTGTTGCAACTCACAGTCTGGGGCATT
>M26727
TTCTACTGTAATACATCACAACTGTTTAATAGTACTTGGAATGATACTACAAGGGCAAATAGC----------
-------------------------------------------------------------------------
----------------------------
ACTGAAGTAACTATCACACTCCCATGTAGAATAAAACAAATTGTAAACATGTGGCAGGAAGTAGGAAAAGCAA
TGTATGCCCCTCCCATCAGTGGACAAATTAGATGTTCATCAAAG---
ATTACAGGGCTGCTATTAACAAGAGATGGTGGTAAGAACACCACG----------------------------
-------------------------------------------------------------------------
----------
AACGGGATCGAAATCTTCAGACCTGCAGGAGGAGACATGAGGGACAATTGGAGAAGTGAATTATATAAATATA
AAGTAGTAAAAATTGAACCATTA---GGAGTAGCACCCACCAAGGCAAGGAGAAGAGTGGTG-----------
----------------------------CAGAGAGAAAAAAGAGCA---GTGGGAATGCTAGGAGCT------
---ATGTTCCTTGGGTTCTTGGGAGCA---GCAGGAAGCACTATG---GGCGCACGGTCG---
ATGACGCTGACGGTACAG---GCCAGACAA---TTATTG---TCTGGTATAGTGCAGCAGCAGAAC---
AATCTG---CTGAGG---GCTATTGAGGCGCAACAGCACCTGTTGCAACTCACAGTCTGGGGCATC
>JN034158
TTCTACTGCAATTCAACACCACTGTTTAATAGTACTTGGAATGGTACTTTGGATAGTGCTGGAGAA-------
-------------------------------------------------------------------------
----------------------------
TCAAGTGACAATATCACACTCATATGCAGAATAAAACAGTTTATAAACCTGTGGCAGGAAGTAGGAAAAGCAA
TGTATGCCCCTCCCATCAGAGGACAAATTAGCTGTTCATCAAAG---
ATTACAGGGCTGCTATTAACAAGAGATGGTGGTATTAAGAATGACACC-------------------------
-------------------------------------------------------------------------
----------
AACAAGACTGAGATTTTTAGACCTGCAGGAGGAGATATGAGGGACAATTGGAGAAGTGAATTATATAAATATA
AAGTAGTAAAAATTGAACCATTA---GGAGTAGCACCCACCACGGCAAAGAGAAGAGTGGTG-----------
----------------------------CAGAGAGAAAAAAGAGCA---GTGGGAACGATAGGAGCT------
---ATGTTCCTTGGGCTCTTGGGAGCA---GCAGGAAGCACTATG---GGCGCAGCGTCA---
GTGACGCTGACGGTACAG---GCCAGACTA---TTATTG---TCTGGTATAGTGCAACAGCAGAAC---
AATTTG---CTGAAG---GCTATTGAGGCGCAACAGCATATGTTGCAACTCACAGTCTGGGGCATT
>JN034139
TTCTACTGTAATTCAACACAGCTGTTTAATAGTACTTGGATAAATGGTATTATACAGTCAAATATCACTGCT-
-------------------------------------------------------------------------
----------------------------
GAAAATGACAACATCACACTCCCATGCAGAATAAAACAAATTATAAACAGGTGGCAGGAAGTAGGAAAAGCAA
TGTATGCCCCTCCCATCAGAGGAATAATTAGATGCACATCAAAC---
ATTACAGGGCTGCTACTAACAAGAGATGGTGGTAATAAGAATGGGAGC-------------------------
```


AACAACAATGAGACCTTTAGACCTGGAGGAGGAGATATGAGAGACAATTGGAGAAGTGAATTATATAAATATA
AAGTAGTAAAAATTGAGCCATTA---GGAGTAGCACCCACCAAGGCAAAAAGAAGAGTGGTG-----------
----------------------------CAGAGAGAAAAAAGAGCA---GTAACG---CTAGGAGCT------
---ATGTTCCTTGGGTTCTTGGGAGCA---GCAGGAAGCACTATG---GGCGCAGCGTCC---
ATGACGCTGACGGTACAG---GCCAGACAA---TTATTG---TCTGGTATAGTGCAACAGCAGAAT---
AATTTG---CTGAGG---GCTATTGAGGCGCAACAGCATCTGTTGCAACTCACAGTCTGGGGCATC
>MG902199
TTTTACTGTAATACAACACAGCTGTTTAATAGTACTTGGAATAGTACTTGGAATTGGAATAATACTGAAGAGT
CAGCTAAC---

ACTACAGCAAACATAACACTCCCATGCAGAATAAAACAAATTATAAACATGTGGCAGGAAGTAGGAAAAGCAA
TGTATGCCCCCCCCATCTCAGGACAGATTAGATGCTCATCAAAT---
ATTACAGGGCTGATCTTAACAAGAGATGGTGGTAATGATACTAGCAAT-------------------------
----

AACGGGACCGAGATCTTCAGACCTGGAGGAGGAAATATGAAGGACAATTGGAGAAGTGAATTATATAAATATA
AAGTAGTAAAAATTGAACCATTA---GGAGTAGCCCCCACCAAGGCAAAGAGAAGAGTGGTG-----------
----------------------------CAAAGAGAAAAAAGAGAC---ATGGGA---CTAGGAGCT------
---TTGTTCCTTGGGTTCTTGGGAGCA---GCAGGAAGCACTATG---GGCGCAGCGTCA---
TTGACGTTGACGGTACAG---GCCAGACAA---TTATTG---TCTGGTATAGTGCAACAGCAGAAC---
AATTTG---CTGAGA---GCTATTGAGGCGCAACAACATTTGTTGCAGCTCACAGTCTGGGGCATC
>MG902157
TTCTACTGTAATACAACACAACTGTTTAATAGCACTTGGAATACTACTGATGGTAGTACTTGGAATAATACTG
ATAATAGTACT--
GCA---AATGACATTAGT---------
ACTGCAAATGACACTATCATACTCCCATGTAGAATAAAACAAATTATAAACATGTGGCAGGAAGTAGGAAAAG
CAATGTATGCCCCTCCCATTAAAGGACTAATTAGATGTTCATCAAAC---
ATTACAGGACTGATATTAACAAGAGATGGTGGTAATAATAGTAGC----------------------------
----

AACGAGACCTTCAGACCTGCAGGGGGAAATATGAAGGACAATTGGAGAAGTGAATTATATAAATACAAAGTAG
TAAAAATTGAACCATTA---GGAATAGCACCCACCAAGGCAAAGAGAAGAGTGGTG-----------------
----------------------CAAAGAGAAAAAAGAGCA---GTGACA---CTGGGAACC---------
ATGCTCCTTGGGTTCTTGGGAGCA---GCAGGAAGCACTATG---GGCGCAGCGTCC---
TTGACGCTGACGGTACAG---GCCAGACAA---TTATTG---TCTGGTATAGTGCAACAGCAGAAC---
AATCTG---CTGAGG---GCTATTGAGGCGCAGCAGCATCTGTTGCAGCTCACAGTCTGGGGCATC
>HM586193
TTCTACTGTAGTACAACACAACTGTTTAATAGTACTTGGAATTGGAATGAGACTGACAATGATCCTGGGAATA
CA---

ACTGGTAATGGCACACTCACACTCCCATGCAGAATAAAACAATTTGTAAACATGTGGCAAGAAGTAGGAAAAG
CAATGTATGCCCCTCCTATCAGCGGACCGATTAACTGTACATCAAAT---
ATTACAGGGCTGCTATTAACCAGAGATGGTGGTAATAGCTCGAAT----------------------------
----

GATTCAGAGACCTTTAGACCTGGAGGAGGAGATATGAGGGACAATTGGAGAAGTGAATTATATAAATACAAAG
TAGTAAAAATTGAACCATTA---GGAGTAGCACCCACCAAGGCAAAGAGAAGAGTGGTG--------------
-------------------------CAAAGAGAAAAAAGAGCA---GTGGGA---CTAGGAGCT---------
ATGTTCCTTGGGTTCTTGGGAGCA---GCAGGAAGCACTATG---GGCGCAGCGTCA---
GTGACGCTGACGGTACAG---GCCAGACTG---TTATTG---TCTGGTATAGTGCAACAGCAGAAT---
AATCTG---CTGAGA---GCTATTGAGGCGCAACAACATCTGTTGCAACTCACAGTCTGGGGCATC
>HM586198
TTCTACTGCAATACAACACAACTATTTAATAGTACTTGGAATAAGAACACAACC-------------------
----

ATGAATGACACAATCATACTCCCATGTAGAATAAAACAGATTATAAACATGTGGCAGGAAGTAGGAAAAGCAA
TGTATGCCCCTCCCATCAGAGGACAAATTAATTGTTTATCAAAT---
ATTACAGGGCTGCTATTAACAAGAGACGGGGGTGATACCAATGGCACC-------------------------

----

AATGGCACAGAAATATTCAGACCTGGAGGAGGAGATATGAGGGACAATTGGAGAAGTGAATTATACAAATATA
AAGTAGTAAAAATTGAACCTTTA---GGAGTAGCACCCACTAGGGCAAAGAGAAGAGTGGTG-----------
----------------------------CAAAGAGAAAAAAGGGCA---GTGGGA---CTGGGAGCT------
---CTGTTCCTTGGGTTCTTAGGAGCA---GCAGGAAGCACTATG---GGCGCAGCGTCA---
ATGACGCTGACGGTACAG---GCCAGACAA---CTGCTG---TCTGGTATAGTGCAACAGCAGAAC---
AATCTG---CTGAGA---GCTATTGAGGCGCAACAGCATCTGTTGCAACTCACAGTCTGGGGCATC
>HM586210
TTCTACTGCAATTCAACACAGCTGTTTAATAGTACTTGGAATAGTACTGAAGGCAGTATTGAAGGGTCAAATA
GCACT--
-------------------GAA---
AAGAACATCACACTCATCACACTCCCATGCAGAATAAAGCAAATTGTAAACCTGTGGCAGGGAGTAGGAAAAG
CAATATATGCCCCTCCCATCAGGGGGCTAATTAGCTGTTCATCAAAT---
ATTACAGGGTTGATATTAACAAGAGATGTTGGTAATGGTGAGAACATA-------------------------
----

AGCAACAATACTGAAACCTTCAGACCAGGAGGAGGAGATATGAGGGACAATTGGAGAAGTGAATTATATAAAT
ATAAAGTAGTAAAAATTGAACCATTA---GGAGTAGCACCCACTAAGGCAAAGAGAAGAGTGGTG--------
-------------------------------CAGAGAGAAAAAAGAGCA---ATAGGA---CTAGGAGCT---
------GTGTTCCTTGGGTTCTTGGGAGCA---GCAGGAAGCACTATG---GGCGCAGCATCA---
GTGACGCTGACGGTACAG---GCCAGACAA---TTATTG---TCTGGTATAGTGCAACAGCAGAAC---
AATCTG---CTGAAG---GCTATTGAGGCGCAACAGCATCTGTTGCAACTCACAGTCTGGGGCATC
>FJ653437
TTCTACTGTAATTCAACGAAACTGTTTAGTAGTACTTGGATGGCTAATAGTACTTGGACTAATAGTACTTTGG
AGAAG--
-------------------AAT---
AGCACTGAAAAAAATATCACACTCCCATGTAAAATAAAACAAATTATAAACATGTGGCAGAAAGTAGGAAAAG
CAATGTATGCCCCTCCTATCAGAGGACAGATTAGATGTTCAACAAAT---
ATCACAGGTCTGTTATTAACAAGAGATGGTGGTGACAACACGACC----------------------------
----

GACAGCGAGGAGGTCTTTAGACCTGGAGGAGGAAATATGAAGGACAATTGGAGAAGTGAATTATATAAATATA
AAGTAGTAAGAATTGAACCATTA---GGAGTAGCACCCACCAAGGCAAAGAGAAGAGTGGTG-----------
----------------------------CAAAGAGAAAAAAGAGCA---GTGGGAGTTGTAGGAGCT------
---ATGTTCCTTGGGTTCTTAGGAGCA---GCAGGAAGCACTATG---GGCGCAGCGGCA---
GTGACGCTGACGGTACAG---GCCAGACAA---TTATTG---TCTGGTATAGTGCAGCAGCAGAAT---
AATCTG---CTGAAG---GCTATTGAGGCGCAACAACATCTGTTGCAACTCACAGTCTGGGGCATC
>HQ595768
TTCTACTGTAATACAACACAACTGTTTAATAGTACCTGGTATGGAAATGAGACTTGGAATAATACTGAAGGTG
CAGAC--

AACACTAATGACACCATCATACTCCCATGCAGAATAAAACAAATTATAAACATGTGGCAGGAAGTAGGAAAAG
CAATGTATGCCCCTCCCATCAGTGGACTAATTAGATGCTCATCAAAC---
ATTACAGGGCTACTATTAACAAGAGATGGGGGTACTACCAATGTCACG-------------------------
----

AACGAGACCGAGACCTTCAGACCAGGAGGAGGAGATATGAGGGACAATTGGAGAAGTGAATTATACAAATATA
AAGTAGTAAAAATTGAGCCATTA---GGAATAGCACCCACCAAGGCAAAAAGAAGAGTGGTG-----------
----------------------------CAGAGAGAAAGAAGAGCA---GTGGGG---CTAGGAGCT------
---TTGTTCGTCGGGTTCTTGGGAGCA---GCAGGAAGCACTATG---GGCACAGCATCA---
ATAATGCTGACGGCACAG---ACCAGACAA---TTACTG---TCTGGTATAGTGCAACAGCAGAAC---
AATCTG---CTGAGA---GCTATTGAGGCGCAACAACATCTGTTGCAACTCACAGTCTGGGGCATT
>HQ595771
TTTTACTGTAATACAACACAACTGTTCAAAGGGACTTGGAATGGTACTAGTGGTACTTGGGAAAGTACTAAT-
----

AGTACTGAGAATATTACACTCCCATGCAGAATAAAACAAATTGTAAACATGTGGCAGGAAGTAGGAAAAGCAA
TGTATGCCCCTCCCATCAGGGGACTAATTAGATGTGAATCAAAT---
ATTACAGGGCTGCTATTAATAAGAGATGGTGGTAATAATAGTAGC----------------------------

```
-------------------------------------------------------------------------
-------------
GATAATGAGACTTTCAGACCTATAGGAGGAAACATGAGGGACAATTGGAGAAGTGAATTATATAAATACAAAA
TAGTAAAAGTTGAACCAATA---GGAATAGCACCCACCATGGCAAAGAGAAGAGTGGTG--------------
-------------------------CAAAGAGAAAAAAGAGCA---GTGGGA---TTAGGAGCT---------
ATGTTCCTTGGGTTCTTGGGAGCA---GCAGGAAGCACTATG---GGCGCAGCGTCG---
ATGACGCTGACGGTACAA---GCCAGACAA---TTATTG---TCTGGTATAGTGCAGCAGCAGAGC---
AATCTG---CTGAGG---GCTATTGAGGCGCAACAGCATATGTTGCAACTCACAGTCTGGGGCATC
>HQ595772
TTCTATTGCAATACAACACAACTGTTTAATAATATTTGGATTACTGATAATGTTACTGTTAATGAAACTAATA
GT-----------------------------------------------------------------------
----------------------------
GACAATAGCAATATCACACTCCCATGCAGAATAAAACAAATTATAAACATGTGGCAGGAAGTAGGAAAAGCAA
TGTACGCCCCTCCCATCAGAGGACAGATTAGATGTTCATCAAAT---
ATTACAGGGCTACTATTAACAAGAGATGGTGGTAATACGAACAATACG-------------------------
-------------------------------------------------------------------------
----------
AACGGGTCTGAGACCTTCAGACCTGGAGGAGGAAATATGAAGGACAATTGGAGAAGTGAATTATATAAATATA
AAGTAGTAAAAGTTGAACCATTA---GGATTAGCACCCACCAAGGCAAAAAGAAGAGTGGTC-----------
----------------------------CAAAGAGAAAAAAGAGCA---ATAACA---CTAGGAGCT------
---GTTTTCCTTGGGTTCTTGGGAGCA---GCAGGAAGCACTATG---GGCGCAGCGTCA---
ATAACGCTGACGGTACAG---GCCAGACAA---TTATTG---TCTGGTATAGTGCAACAGCAGAAC---
AACTTG---CTGAGG---GCTATTGAGGCGCAACAACATATGTTGCAACTCACAGTCTGGGGCATC
>HQ595774
TTCTACTGTAATACAACACAATTGTTTAATAGTACCTGGAATGATACTAATATTAATAGTACTGAAGAGTCAA
AT-----------------------------------------------------------------------
----------------------------
AACACTGACACTATCACACTCCAATGCAGAATAAAACAAATTATAAACATGTGGCAGGAAGTAGGAAAAGCAA
TGTATGCCCCTCCTATCCGAGGACATATTAATTGTTCATCAAAT---
ATTACAGGGCTGCTATTAACAAGAGATGGTGGTAGTAATACTACC----------------------------
-------------------------------------------------------------------------
-------------
ACCCAGGAGACCTTCAGACCTGGAGGAGGAAATATGAAGGACAATTGGAGAAGTGAATTATATAAATATAAAG
TAGTAAAAATTGAACCATTA---GGAGTAGCACCCTCCAAGGCAAAAAGAAGAGTGGTG--------------
-------------------------CAGAGAGAAAAAAGAGCA---GTAGGA---CTGGGAGCT---------
TTGTTCCTTGGGTTCTTGGGAGCA---GCAGGAAGCACTATG---GGCGCAGCATCA---
CTAACGCTGACGGTACAG---GCCAGACAA---TTATTG---TCTGGTATAGTGCAACAGCAGAAC---
AATTTG---CTGAGA---GCTATTGAGGCGCAACAGCATCTGTTGCAACTCACAGTCTGGGGCATC
>HM204626
TTCTACTGTAATACAACAAAACTGTTTAATAGCACTTGGAATAGTAATAGTACTTGGAATAGTACGCAA----
-------------------------------------------------------------------------
----------------------------
GGAAATGACACAATCACACTCCCGTGCAGAATAAAACAAATTATAAACCTGTGGCAGGAAGTAGGAAAAGCAA
TGTATGCCCCTCCCATCAGAGGATCAATTAAATGTTCATCAAAT---
ATTACAGGGCTGCTACTAACAAGAGATGGGGGTATAAACAATAGTAAT-------------------------
-------------------------------------------------------------------------
----------
AATAATATAGAGACCTTCAGACCTGGAGGAGGAGATATGAGGGACAATTGGAGAAGTGAATTATATAAATATA
AAGTAGTACAAATTGAACCATTA---GGAGTAGCACCCACCAGGGCAAAGAGAAGAGTGGTG-----------
----------------------------CAAAGAGAAAAAAGAGCA---GTGGGA---CTAGGAGCT------
---TTGTTCCTTGGGTTCTTGGGAGCA---GCAGGAAGCACTATG---GGCGCAGCGTCA---
ATAACGCTGACGGTACAG---GCCAGACAA---TTATTG---TCTGGTATAGTGCAACAGCAGAAC---
AATTTG---CTGAGA---GCTATTGAGGCGCAACAACATCTGTTGCAACTCACAGTCTGGGGCATC
>HM204627
TTCTACTGTAATACAACACAACTGTTTAATAGTACTTGGACTTGGAATGATACTACTTGGAATGGTACTAATG
GCGGG--------------------------------------------------------------------
-------------------AAT---
AACACTGAAAAAACCATCACACTCCCATGCAGAATAAAACAAATTATAAACATGTGGCATGAAGTAGGAAAAG
CAATGTATGCCCCTCCCATCAGGGGAATAATTAGCTGTTCATCAAAT---
ATTACAGGTCTGCTATTAACAAGAGATGGTGGTAGTGGTAATATGAGC-------------------------
```


AACACCACCGAGACCTTCAGACCTGAAGGAGGAAATATGAAGGACAATTGGAGAAGTGAATTATATAAATATA
AAGTAGTAAGAATTGAACCATTG---GGAATAGCACCCACCAAGGCAAAGAGAAGAGTGGTG-----------
----------------------------CAAAGAGAAAGAAGGGCA---GTGGGA---TTCGGAGCT------
---ATGTTCCTTGGGTTCTTGGGAGCA---GCAGGAAGCACTATG---GGCGCAGCGTCA---
GTGACGCTGACGGTACAG---GCCAGACAA---TTATTG---TCTGGTATAGTGCAACAGCAGAAC---
AATCTG---CTGAGG---GCTATTGAGGCGCAACAGCATCTGTTGCAACTCACAGTCTGGGGCATC
>HM204628
TTCTACTGTAATACAACACCGATATTTAGTAGTACATGGAATAGTAATAGTACTAAAGATGATATTGAGAGAT
CAAAT--

AGCACTGACAGGAATATCACACTCCAATGCAGAATAAGACAAATTATAAACATGTGGCAGAAAGTAGGAAAAG
CAATGTATGCCCCTCCCATCCAAGGGCTAATTAGATGTTCATCAAAT---
ATTACAGGGCTGCTATTAACAAGGGATGGTGGTAAGGACAACAAT----------------------------
----

AACAACACCGAGACCTTCAGACCTGGGGGAGGAGATATGAGGGACAATTGGAGAAGTGAATTATATAAATATA
AAGTAGTACAAATTGAACCAGTA---GGAGTGGCACCCACTAAGGCAAAGAGAAGAGTGGTG-----------
----------------------------CAGAGAGAAAAAAGAGCA---GTGGGA---ATAGGAGCT------
---TTGTTCCTTGGGTTCTTGGGAGCA---GCAGGAAGCACTATG---GGCGCAGCGTCA---
ATGACACTGACGGTACAG---GCCAGACAA---TTGTTG---TCTGGTATAGTGCAACAGCAAAAC---
AATCTG---CTGAGG---GCTATTGAGGCGCAACAGCATCTGTTGCAACTCACAGTCTGGGGCATC
>HM204629
TTCTACTGTAATACAACACAACTGTTTAATAGTACTTGGCCATTCAATGATACTGGGAATGATAATAGTAGTA
GC---

ACTGACAATACAACAATCTCACTCCCATGTAGAATAAAACAAATTGTAAACATGTGGCAGGAAGTAGGAAAAG
CAATGTATGCCCCTCCCATTAGAGGACAAATTAGATGTTCATCAAAT---
ATTACAGGACTGATATTAACAAGAGATGGTGGCACACCCGACAACCAA-------------------------
----

AGTAGTGAGGAGGTCTTCAGACCTAGTGGAGGAAATATGAAAGACAATTGGAGAAGTGAACTATATAAATATA
AAGTAGTAAAAATTGAACCGTTA---GGAATAGCACCCACCAAGGCAAAAAGAAGAGTGGTG-----------
----------------------------CAGAGAGAGAAAAGAGCA---GCCACA---CTGGGAGCT------
---ATGTTCCTTGGATTCTTGGGAGCA---GCAGGAAGCACTATG---GGCGCAGCATCA---
ATAACGCTGACGGTACAG---GCCAGACTA---TTACTG---TCTGGTATAGTGCAGCAGCAGAGC---
AATCTG---CTGAGG---GCTATTGAGGCGCAACAGCATATGTTGCAACTCACAGTCTGGGGCATC
>HM204632
TTCTACTGTAATTCAACACAACTGTTTAATAGCACTTGGAATAGTAATAGTACTTGGAATGATACTATGGGCA
CTAAT--
-------------------GAT---
ACTATGGACACTAATATCACACTTCCATGTAGAATAAAACAAATTATAAACTTGTGGCAGGAAGTAGGAAAAG
CAATGTATGCCCCTCCCATCGCAGGACAAATTAGATGTTCATCAAAT---
ATTACAGGGTTGCTGTTAACAAGAGATGGTGGTAGTAATAGCACC----------------------------
----

AACAATGAGACCTTCAGACCTGGAGGAGGAAATATGAGGGACAATTGGAGAAATGAATTATATAAATATAAAG
TAGTACAAATTGAACCATTA---GGAATAGCACCCACCAAGGCAAAGAGAAGAGTGGTG--------------
-------------------------CAGAGAGAAAAAAGAGCA---GTGGGAACGCTAGGAGCT---------
ATGTTCCTTGGGTTCTTGGGAGCA---GCAGGAAGCACTATG---GGCGCAGCGTCA---
GTGACGCTGACGGTACAG---GCCAGGCAA---TTGCTG---TCTGGTATAGTGCAGCAGCAGAAC---
AATCTG---CTGAAG---GCTATTGAGGCACAACAGCATATGTTGCAACTCACAGTCTGGGGCATT
>HM204633
TTCTACTGTAGTACAGCAGAACTGTTTAATAGTACCTGGTTTAACAATGGTACATGGATAAGTGACAATGGAA
AA---

AATGACACAAATATCACACTCCCATGCAGAATAAAACAAATCATAAACATGTGGCAGGGAGTAGGAAAAGCAA
TGTATGCCCCTCCCATCAGTGGACTAATTAGCTGTTCATCACAA---
ATTACAGGGTTGCTATTAACAAGAGATGGTGGTAATGCGAGTGTTAAC-------------------------

```
-------------------------------------------------------------------------
----------
AACTGGACCGAGACCTTCAGACCTGTGGGAGGAGATATGAGGGACAATTGGAGAAGTGAATTATATAAATATA
AAGTAGTAAAAATTGAACCATTA---GGAGTAGCACCCACCAAGGCAAGGAGAAGAGTGGTG-----------
----------------------------CAGAGAGAAAAAAGAGCA---GTGGGA---ATAGGAGCT------
---GTGTTTCTTGGGTTCTTGGGAGCA---GCAGGAAGCACTATG---GGCGCAGCATCA---
GTGATGCTGACGGTACAA---GCCAGGCAA---TTATTG---TCTGGTATAGTGCAGCAGCAAAAC---
AATTTG---CTGAGA---GCTATTGAGGCGCAACAGCATCTGTTGCAACTCACTGTCTGGGGCATC
>HM204638
TTCTACTGTAATACAACACAGCTGTTTAATAGTACTTGGCAGCTGTCTAATAGTACTCTGAAGGAAACA----
-------------------------------------------------------------------------
----------------------------
CAAGAGAGTAATATCACACTCCCATGCAGAATAAAACAAATTATAAACAAATGGCAGGAAGTAGGAAAAGCAA
TGTATGCCCCTCCCATCCAAGGAAAGATTAGTTGTACATCAAAT---
ATTACAGGGCTGCTATTAACAAGAGATGGTGGTGTCGAGAACAAGACT-------------------------
-------------------------------------------------------------------------
-------
AACGAGACTAACGAAACCTTCAGACCTGGAGGAGGAGATATGAGGGATAATTGGAGAAGTGAATTATATAAAT
ATAAAGTAGTAAGAATTGAGCCATTA---GGAGTAGCACCTTCCAAGGCAAGGAGAAGAGTGGTG--------
-------------------------------CAGAGAGAAAAAAGAGCA---GTAGGA---CTGGGAGCT---
------TTGTTCCTTGGGTTCTTGGGAGCA---GCAGGAAGCACTATG---GGCGCAGCGTCA---
TTAACGCTGACGGTACAG---GCCAGACAA---CTATTG---TCTGGTATAGTGCAACAGCAGAAC---
AACCTG---ATGAAA---GCTATTGAGGCGCAACAGCATTTGTTGCAACTCACAGTCTGGGGCATC
>HM204640
TTCTACTGCAATTCATCACTGCTGTTCAATAATACTTGGAATGGCAATGATACA-------------------
-------------------------------------------------------------------------
----------------------------
AATAATACTATACTCACACTCCCATGCAAAATAAAACAAATTATAAACCTGTGGCAGGCAGTAGGAAAAGCAA
TGTATGCCCCGCCCATCCAAGGACTAATTAGATGTTCATCAAAT---
ATTACAGGGCTGTTATTAACAAGAGATGGTGGTAATGATAATAGCACA-------------------------
-------------------------------------------------------------------------
----------
AACCAGACCGAGGTCTTCAGACCTGGAGGAGGAAATATGAAGGACAATTGGAGAAGTGAATTATATAGATATA
AAGTAGCAAAAATTGAACCATTA---GGAGTAGCACCCACCAAGGCAAAGAGAAGAGTGGTG-----------
----------------------------CAGAGAGAGAAAAGAGCA---GTAACG---TTAGGGGCA------
---ATGTTCCTTGGGTTCTTGGGAACA---GCAGGAAGCACTATG---GGCGCAGCGTCG---
ATGACGCTGACGGTACAG---GCCAGACTC---TTATTG---TCTGGTATAGTGCAACAGCAGAAC---
AACTTG---CTGAGA---GCTATTGAGGCGCAACAGCATCTGTTGCAACTCACAGTCTGGGGCATT
>HM204641
TTCTATTGTAATACAACAAAGCTGTTTAATTCTACTTGGAACAATACTAGTACTTGGAATAATAGTACTTGGA
ATAATACAATT--------------------------------------------------------------
----------TGGGATACT------
GATGGCAATGACACTATCATACTCCAATGCAGAATAAGACAAATTATAAACAGGTGGCAGGAAGTAGGGAAAG
CCATGTATGCCCCTCCCATCCAAGGACAAATTAAGTGCTCCTCAAAT---
ATTACAGGGCTGCTATTAGCAAGAGATGGAGGAAGTAATGGAAGT----------------------------
-------------------------------------------------------------------------
----------
AACAACATAGAGACCTTCAGACCAGGAGGAGGAAATATGAAGGATAATTGGAGAAGTGAATTATATAAATATA
AAGTAGTACAAATTGAGCCATTA---GGAATAGCACCCACCAAGGCAAAGAGAAGAGTGGTG-----------
----------------------------CAGAGAGAAAAAAGAGCA---GTGGGGGCACTGGGAGCT------
---ATGTTCCTTGGGTTCCTGGGAGCA---GCAGGAAGCACTATG---GGCGCAGCGTCA---
ATAGCGCTGACGGCACAG---GCCAGACAA---TTATTG---TCTGGTATAGTGCAACAGCAAAAC---
AATTTG---CTGAGG---GCTATTGAGGCGCAGCAGCATCTGTTGCAACTCACAGTCTGGGGCATC
>HM204642
TTCTACTGTAATACATCACAACTGTTTAATAGTACCTGGGTTAATGGTACCTGGAATGGTACTGAAGGGTCA-
-------------------------------------------------------------------------
----------------------------
AATAGCCCAAACATCACACTCCAATGTAAAATAAAACAAATTATAAACATGTGGCAAGGAGTGGGAAAAGCAA
TGTATGCCCCTCCCATCGCAGGAAAAATTAGCTGTTCATCACAA---
ATCACAGGGCTGCTATTAGCAAGAGATGGTGGTAATGACAATTCCACT-------------------------
```


GACAATAACACTGAGATCTTCAGACCTTCAGGAGGAAATATGAAAGACAATTGGAGAAGTGAATTATATAAAT
ATAAAGTAGTAAAAATTGAACCAATA---GGAATAGCACCCACCAAGGCAAAAAGAAGAGTGGTG--------
-------------------------------CAGAGAGAAAAAAGAGCA---GTAGGAATGATGGGAGCT---
------ATGTTCCTTGGGTTCTTGGGAGCA---GCAGGAAGCACTATG---GGCGCAGCGTCA---
GTGACGCTGACGGTACAG---GCCAGGCAA---TTATTG---GCTGGTATAGTGCAACAGCAGAAC---
AATTTG---CTGAGA---GCTATTGAGGCGCAACAGCATCTGTTGCAACTCACAGTCTGGGGCATT
>HM204643
TTCTACTGTAATACAACAAAACTGTTTAATAGTACTTGGAATGTTAATAGTACTTGGAATACTAATAGTACTT
GGAAT--

ACTACTGAAGAGATAGTAAATATCCCATGCAGAATAAGACAAATTGTAAATAGGTGGCAAGAAGTAGGAAAAG
CAATGTATGCCCCTCCCATCAGAGGACAAATTAGATGTTCATCAAAC---
ATTACAGGGTTACTATTAACAAGAGATGGTGGTAATGGCAACCAG----------------------------
----

TCCAATAACGAGACCTTCAGACCTGGAGGAGGGGATATGAGGGACAAATGGAGAAGTGAATTATATAAATATA
AAGTAGTAAGAGTTGAACCATTA---GGAGTAGCACCCACCAAGGCAAAGAGAAGAGTGGTG-----------
----------------------------CAGAGAGAAAAAAGAGCA---GTGGGA---ATAGGAGCT------
---GTGTTCCTTGGGTTCTTGGGAGCA---GCAGGAAGCACTATG---GGCGCAGCCTCA---
ATAACGCTGACGGTACAA---GCCAGACTA---TTATTG---TCTGGTATAGTGCAACAGCAGAAC---
AATCTG---CTGAGG---GCTATTGAGGCGCAACAACATCTGTTGCAACTCACAGTCTGGGGCATC
>HM204646
TTTTACTGTAATTCATCACAACTGTTTACTTGGAATGAGACTAGTATTCGGAATAAGACAAATAAT-------
----

ACAAATGATACTATCACACTCCCATGTAAAATAAAACAAATTATAAACAGGTGGCAGGAAGTAGGAAGAGCAA
TGTATAACCCTCCCATCAAAGGAATAATTAGATGTGAATCAAAT---
ATTACAGGGTTACTGTTAACAAGAGATGGTGGTACTAATAATAAC----------------------------
----

ACCAACGAGACCTTCAGACCTGCAGGAGGAGACATGAGGGACAATTGGAGAAGTGAACTATATAAATATAAAG
TAGTACAAATTGAACCCTTA---GGAGTAGCACCCACCAAGGCAAAAAGAAGAGTGGTG--------------
-------------------------CAGAGAGAAAGAAGAGCA---GTGGGA---ATAGGAGCT---------
ATGTTCCTTGGGTTCTTGGGGGCA---GCAGGAAGCACTATG---GGCGCAGCGTCG---
GTGACGCTGACGGTACAG---ACCAGACAA---TTATTG---TCTGGTATAGTGCAGCAGCAGAAC---
AATCTG---CTGAGG---GCTATTGAGGCGCAACAGCACATGTTGCAACTCACAGTCTGGGGCATT
>KY658697
TTCTACTGTAATACATCACACCTGTTTAATAGTACTTGGGAACGTAATAGTAGTACTTGGAACCTTACTAAAG
GGTTAAAT---

GACACTGGAGGAAATATCACACTCCAATGCAAAATAAGACAATTCGTAAGACTGTGGCAGGAAGTAGGGAAAG
CAATGTATGCCCCTCCCATCAAAGGACTAATTAGATGTTCATCAAAT---
ATTACAGGACTGTTATTAACAAGAGATGGTGGTAATAATAATAATAATGAAACA-------------------
----

AACACGACTGAAACCTTCAGACCTGCAGGAGGAGATATGAGGGATAATTGGAGAAGTGAATTATATAGATATA
AAGTAGTGAGAATTGAACCAATA---GGAGTAGCACCCACCAGGGCAAAGAGAAGAGTGGTG-----------
----------------------------CAGAGAGAAAAAAGAGCG---GCA------CTAGGAGTT------
---TGGTTCCTTGGGTTCTTGGGAGCA---GCAGGAAGCACTATG---GGCGCAGCGTCA---
GTGACGCTGACGGCACAG---GCCAGACAA---TTATTG---TCTGGTATAGTGCAACAGCAGAGC---
AATTTG---TTGAAG---GCTATTGATGCGCAACAGCATCTGCTGCAACTCACAGTCTGGGGCATC
>HQ595776
TTCTACTGTAATACATCAAAACTGTTTGATAGTAGTTGGCTGCCCAACAATACTGGTGGGCTGCCTACCAAT-
----

TCTGCAGACCCAATCATACTCCCATGCAGAATAAGACAAATTATAAACATGTGGCAAAGGGTAGGAAAAGCAA
TGTATGCCCCTCCCATCAGTGGACAAATTAACTGTACATCCAAT---
ATTACAGGGCTACTATTAACAAGAGATGGTGGTAAGGGAAATGACACT-------------------------

----

AACCAGACCGAGATCTTTAGACCTGCAGGAGGAGATATGAAGGACAATTGGAGAAGTGAACTATATAAATATA
AAGTAGTAAGAATTGAACCATTA---GGAGTAGCACCCACCAAGGCAAAGAGAAGAGTGGTG-----------
----------------------------CAGAGAGAAAAAAGAGCA---GTGGGA---TTAGGAGCT------
---GTGTTCCTTGGGTTTTTGGGAGCA---GCAGGAAGCACTATG---GGCGCAGCGTCA---
GTGACGCTGACGGTACAG---GCCAGACAA---TTATTG---TCTGGTATAGTGCAACAGCAGAGC---
AATCTG---CTGAGA---GCTATAGATGCACAACAGCATCTGTTGCAGCTCACAGTCTGGGGCATA
>HQ595778
TTCTACTGTAATTCAACACTACTGTTTAATGGTACTTGGCATGATTCTAATAGGACTTGGCATAACCCTAAAG
GA---

AATAACAGTGACACAATCACACTCCCATGCAGAATAAAACAAATTATAAACATGTGGCAGGAAGTAGGAAAAG
CAATGTATGCCCCTCCCATCCAAGGACTAATTAGATGTAATTCAAGT---
ATTACAGGGCTGATGATAACAAGAGATGGTGGTAATAAAACTAACGAGACC----------------------
----

ATCGAGAACGTCGAGACTTTTAGACCTGGAGGAGGAAATATGAAGGACAATTGGAGAAGTGAATTATATAAAT
ATAAAGTAGTAAAAATTAAACCATTA---GGAATAGCACCCACTAAGGCAAAGAGAAGAGTGGTG--------
-------------------------------CAGAGAGAAAAAAGAGCA---GTGATA------GGAGCT---
------ATGTTCCTTGGGTTCTTGGGAGCA---GCAGGAAGCACTATG---GGCGCAGCGTCA---
CTGACGCTGACGGTACAG---GCCAGACAA---TTATTG---TCTGGTATAGTGCAACAGCAGAAT---
AATTTG---CTGAGA---GCTATTGAGGCGCAACAGCATCTGTTGCAACTCACAGTCTGGGGCATC
>HQ595779
TTCTACTGTAATTCCACACAGCTGTTTAATAGTACTTGGGGAATAGGGGCAAATGTCACAAATACC-------
----

ACTGAATACAATATCACACTCCCATGTAGAATAAAACAAATTATAAACAGGTGGCAGGAAGTAGGAAGAGCAA
TGTATGCCCCTCCCATCAGTGGACAAATTAGATGTTCATCAAAT---
ATTACAGGGCTGCTATTAACAAGAGATGGTGGTGGTGGTAATATT----------------------------
----

AGTAATACAACTGAGATCTTCAGACCTGGGGGAGGAGATATGAGGGACAATTGGAGAAGTGAATTATATAAAT
ATAAAGTAGTAAAAATTGAGCCATTA---GGAGTAGCACCCACCACGGCAAAGAGAAGGGTGGTG--------
-------------------------------CAAAGAGAAAAAAGAGCA---CTGACG---CTAGGAGCT---
------ATGTTCCTTGGGTTTTTGGGAGCA---GCAGGAAGCACTATG---GGCGCAGCGTCA---
ATAACACTGACGGTACAG---GCCAGACAA---TTATTG---TCTGGTATAGTGCAACAGCAGAAC---
AATATG---CTGAAA---GCTATTGAGGCGCAACAGCACTTGTTGCAACTCACAGTCTGGGGCATT
>HQ595788
TTCTACTGTGATACAGCAAACCTGTTTAATAGTACTTGGTATGCTAATGGGAGTGGGAATATTAATGAAAGT-
----

TCAAGTAGCAATATCACAATCACATGTAGAATAAAACAAATCATAAACATGTGGCAGAAAGTAGGAAAAGCAA
TGTATGCCCCTCCCATCAGAGGACCAATTAGCTGTTCATCAAAT---
ATTACAGGGCTGCTATTAGTAAGAGATGGAGGTAACGTGACCAGT----------------------------
----

GAGAACGAGATCTTCAGACCTGCAGGGGGAGATATGAGGGACAATTGGAGAAGTGAATTATACAAATATAAGG
TAGTAAAAATTGAACCATTA---GGAATAGCACCCACCAAGGCAAAGAGAAGAGTGGTG--------------
-------------------------CAAAGAGAAAAAAGAGCA---GTGGGAACAATAGGAGCT---------
ATGATCCTTGGGTTCTTGGGAACA---GCAGGAAGCACTATG---GGCGCAGCGTCA---
CTGACGCTGACGGTACAA---GCCAGACAA---TTATTG---TCTGGTATAGTGCAACAGCAGAAC---
AATTTG---CTGAAG---GCTATTGAGGCGCAACAACATCTGTTGCAACTCACAGTCTGGGGCATT
>HQ595790
TTCTACTGTGATACAGCAAAACTGTTTAATAGTACTTGGCTGCTGTCTAATGGTACTTGGAATAATTCAGCAG
TTAAT--
-------------------AGT---
ACTGGGAATGGAAATATCACGCTCCAATGCAGAATAAAACAAATTATAAACATGTGGCAGAGAGTAGGAAGAG
CAATGTATGCCCCTCCCATTAAAGGACAAATTAGATGTTCATCAAAT---
ATTACAGGGCTGCTATTGACGAGAGATGGTGGTAACACCAATAACACT-------------------------

----

AATGGCACCGAGACTTTCAGACCTGGAGGGGGAAATATGAAGGACAATTGGAGAAGTGAATTATATAAATATA
AGGTAGTACAAATTGAGCCCTTA---GGAATAGCACCCACCAAGGCAAAGAGAAGAGTGGTG-----------
----------------------------CAAAGAGAAAAAAGAGGA---GTGGGA---TTAGGAGCT------
---CTGTTCCTTGGGTTCTTGGGAGCA---GCAGGAAGCACTATG---GGCGCAGCGTCA---
ATAACGCTGACGGTACAG---GCCAGACAG---TTATTG---TCTGGTATAGTGCAACAGCAGAAC---
AATCTG---CTGAAG---GCTATTGAGGCGCAACAGCATCTGTTGCAACTCACAGTCTGGGGCATT
>HQ595792
TTCTACTGTAATACATCACAACTGTTTAATAGTACTTGGAATAGTACTTTGGAGGTGAATAATGGTACT----
----

GACAATGACAATATCACACTCCCATGTAAAATAAAACAAATTATAAACATGTGGCAGGAAGTAGGAAAAGCAA
TGTATGCCCCTCCCATCGAAGGACTAATTAGTTGTTCATCAAAT---
ATTACAGGGCTGATATTAACAAGAGATGGTGGGAGTAACAGCACG----------------------------
----

AATGAGACTTTCAGACCTGGAGGAGGAGATATGAGGGACAATTGGAGAAGTGAATTATATAAATATAAAGTAG
TAAAAATTGAACCATTA---GGAATAGCACCCACCCAGGCAAAGAGAAGAGTGGTG-----------------
----------------------CAAAGAGAAAAAAGAACA---GTGGGG---ATAGGAGCT---------
GTGTTCCTTGGGTTCTTGGGAGCA---GCAGGAAGCACTATG---GGCGCAGCGTCA---
GTGACGCTGACGGTACAG---GCCAGACTA---TTATTG---TCTGGTATAGTGCAACAGCAGAAC---
AATCTG---CTGAAG---GCTATTGAGGCGCAACAACATCTGTTGCAACTCACAGTCTGGGGCATT
>HQ595794
TTCTACTGTAATACATCAAAACTGTTTAATAGTACTTGGAATAGTACTGAAGGGTTAAATGACACTGAAGAA-
----

GGAAATGGCACAATTGTACTCCCATGCAGAATAAAACAGATTATAAACATGTGGCAGGAAGTAGGAAAAGCGA
TGTATGCCCCTCCCATCAGTGGACAAATTAACTGTGTATCAAAT---
ATTACAGGGATGATATTAACAAGAGATGGTGGACAGGATAATAGG----------------------------
----

AGCAGCGAGACCTTCAGACCTGGAGGGGGAGATATGAGGGACAATTGGAGGAGTGAACTATATAAATATAAAG
TAGTACAAATTGAACCACTA---GGAGTAGCACCCACCAAGGCAAAGAGAAGAGTGGTG--------------
-------------------------CAGAGGGAAAAAAGAGCA---ATAGGGGTACTAGGAGCT---------
ATGTTCATTGGGTTCTTGGGAGCA---GCAGGAAGCACTATG---GGCGCAGCGTCA---
ATGACGCTGACGGTACAA---GCCAGACTA---TTATTG---TCTGGTATAGTGCAACAGCAGAAC---
AACTTG---CTGAGG---GCTATTGAGGCGCAACAGCATCTGTTGCAACTCACAGTCTGGGGCATT
>HQ595803
TTCTACTGTAATACAACACAGCTATTTAATAGTACTTGGAGTTGGAATGGTACTGACATTAGAGATAATGGCA
CTGAC--
-------------------ATT---
AGAGATAATGGCACAATCATACTCCCATGCAGGATAAAACAAATTATAAACAGGTGGCAGGAAGTGGGAAAAG
CAATGTATGCCCCTCCCATCAAGGGACAAATTATGTGTAACTCAACC---
ATTACAGGGTTGCTATTAACAAGAGATGGTGGTGATAACAATAAT----------------------------
----

GAGACTGAGATCTTCAGACCTGGAGGAGGAAATATGAAGGACAATTGGAGAAGTGAATTATTTAAATATAAAG
TAGTAAAAATTGAGCCATTA---GGAATAGCACCCACCAAGGCAAAGAGAAGAGTGGTG--------------
-------------------------CAGAGAGAAAAAAGAGCA---GTGGAA---ATAGGAGCT---------
GTGTTCCTTGGGTTCTTGGGAGCA---GCAGGAAGCACTATG---GGCGCAGCGTCA---
GTAACGCTGACGGTACAA---GCCAGACAA---TTATTA---TCTGGTATAGTGCAACAGCAGAAT---
AATTTG---CTGAAG---GCTATTGAAGCGCAACAGCATCTGTTGCAACTCACAGTCTGGGGCATC
>JF680906
TTCTACTGTGATACAGCAAAGCTGTTTAATAGTACTTGGAAAAATGGTAATATAAGCATCGCA----------
----

AAGGACAACCCTATCATACTCCCATGCAGAATAAAACAAATTATAAACAGGTGGCAGGAGGTAGGAAAAGCAA
TGTATGCCCCTCCCATCAGAGGAAAGATTAGCTGTTCATCAAAT---
ATTACAGGGTTGCTTTTAACAAGAGATGGTGGCAGTAAAAATGTGACA-------------------------

----

GATAATAAGACAGAGATCTTCAGACCTGGAGGAGGAGATATGAGGGACAATTGGAGAAGTGAATTATATAAAT
ACAAAGTAGTAAGAATTGAGCCATTA---GGAGTAGCACCCACCAAGGCAAAGAGAAGAGTGGTG--------
-------------------------------CAGAGAGAAAAAAGAGCA---GTGGGA---ATAGGAGCT---
------TTGTTCCTTGGGTTCTTGGGAGCA---GCAGGAAGCACTATG---GGCGCAGCGTCA---
ATGACGCTGACGGTACAG---GCCAGACAA---TTATTG---TCTGGTATAGTGCAACAGCAGAAC---
AATCTG---CTGAGG---GCTATTGAGGCGCAACAGCATCTGTTGCAACTCACAGTCTGGGGCATC
>HQ595769
TTCTACTGTAATACATCACAACTGTTTAATAGTACTTGGAATTCGAGTGATACTACTTGGAATTCAAATGATA
CTAAT--
-------------------ATT---
AATGGAACGGGTACAATCACACTCCCATGTAAAATAAGACAAATTATAAATATGTGGCAGGAAGTAGGAAAAG
CAATGTATGCCCCTCCCATCAGAGGATTCATTAACTGTTCATCAAAT---
ATTACAGGGCTGCTAATAACAAGAGATGGTGGTAGTGAGAATGGC----------------------------
----

ACAAACACTGAGACCTTCAGACCTGGAGGAGGAAATATGAGGGACAATTGGAGAAGTGAATTATATAAATATA
AAACAGTAAAAGTTGAACCATTA---GGAGTAGCACCAACCAGGGCAAGGAGAAGAGTGGTG-----------
----------------------------CAGAGAGAGAAAAGAGGG---CTAACT---CTAGGAGCT------
---TTGTTCCTTGGGTTCTTGGGAGCA---GCAGGAAGCACTATG---GGCGCAGCGTCA---
GTGACGCTGACGGTACAG---GCCAGACAA---TTATTG---TCTGGTATAGTGCAACAGCAGAGC---
AATCTG---CTGAGG---GCTATTGAGGCGCAACAGCATATGTTGCAACTCACAGTCTGGGGCATC
>HQ595770
TTCTACTGTAATACAACACAACTGTTTAACAGTACTTGGAACAAGGATGGTAGTATTGGGAATACTACTAAGG
GGTCA--

ATTAACACAGACACACTCACACTCCCATGCAGAATAAAACAAATTATAAACCTGTGGCAGGAAGTAGGAAAAG
CAATGTATGCCCCTCCCATCAGAGGACAAATTAGATGTACATCAAAT---
ATTACAGGGCTGATGTTAACAAGGGATGGTGGTAATAATGGGAGC----------------------------
----

GAGACCGAGATCTTCAGACCTGCAGGAGGAAATATGAGGGACAATTGGAGAAGTGAATTATATAAATATAAAG
TAGTAAAAATTGAACCATTA---GGAGTAGCACCCACCAAGGCAAAAAGAAGAGTGGTG--------------
-------------------------CAGAGAGAAAAAAGAGCA---GTAGTGGGAATAGGAGCT---------
GTGTTCCTTGGGTTCTTGGGAGCA---GCAGGAAGCACTATG---GGCGCAGCGTCA---
ATGACGCTGACGGTACAG---GCCAGACAA---TTATTG---TCTGGTATAGTGCAACAGCAGAAC---
AATCTG---CTGAGA---GCTATTGAGGCGCAACAGCATCTGTTGCAACTCACAGTCTGGGGCATC
>JF680910
TTCTACTGTGATTCAACGCGATTGTTTAATAGTACTTGGACAAATGGAAGTTGGACAAATGGTACTGAAGAG-
----

AAAAATGACACTATCACGCTCCCATGCAGAATAAAACAAATTATAAACAGGTGGCAGGAAGTAGGAAAAGCAA
TGTATGCCCCTCCCATCAGAGGACTAATTAAATGTTCATCAAAC---
ATTACAGGGCTGTTGTTAACAAGAGATGGTGGTGAGAAGAAGAACAAC-------------------------
----

AGTAATAGTACAGAAACCTTCAGACCTGGAGGAGGGGATATGAGGGATAATTGGAGAAGTGAATTATATAAAT
ATAAAGTAGTAAAAATTGAACCATTA---GGAGTAGCACCTACCAAGGCAAAGAGAAGAGTGGTA--------
-------------------------------CAGAGAGAAAAAAGAGCA---ATAGGAACGCTAGGAGCT---
------GTGTTCCTTGGATTCTTGGGAGCA---GCAGGAAGCACTATG---GGCGCAGCGTCG---
CTGACGCTGACGGTACAG---GCCAGACAA---TTATTG---TCTGGTATAGTGCAACAGCAGAAC---
AATCTG---CTAAGG---GCTATTGAGGCGCAACAACATCTGTTGCAACTCACAGTCTGGGGCATC
>JF680911
TTCTACTGTGAAACAGCACAGCTGTTTAATAGTACTTGGTATGGGAATGAAACTGAAGAGTCACAAAAT----
----

AAGACAGACCCTATCATACTCCCATGCAGAATAAAACAAATCATAAATAGGTGGCAAGAAGTAGGAAAAGCAA
TGTATGCCCCTCCCATCAGTGGATACATTAACTGTTCATCAAAT---
ATTACAGGGCTATTATTAACAAGAGATGGTGGTAAGGATGATAAC----------------------------

```
-------------------------------------------------------------------------
-------------
ACCACCGAGACCTTCAGACCTGGAGGAGGAGATATGAGGGATAATTGGCGAAGTGAATTATATAAATATAAAG
TAGTAAGAATTGAACCAGTA---GGAATAGCACCCACCAGGGCAAAAAGACGAGTGGTG--------------
-------------------------CAGAGAGAAAAAAGAGCA---GTGGGAACAATAGGAGCT---------
ATGTTCCTTGGGTTCTTGGGAGCA---GCAGGAAGCACTATG---GGCGCAGCGTCA---
GTGACGCTGACGGTACAG---GCCAGACAA---TTATTG---TCTGGTATAGTGCAACAGCAAAAC---
AATTTG---CTGAAA---GCTATTGAGGCGCAACAACATCTGTTGCAACTCACAGTCTGGGGCATC
>JF680913
TTCTATTGTAATACAACAAATCTGTTTAATGCAACATTTAATATAACAAACAATACAACAAAGCTACCTAAC-
-------------------------------------------------------------------------
----------------------------
ACAGGTGACACTATCATACTCCCATGCAGAATAAAACAGATTATAAACAGGTGGCAGGAAGTAGGAAAAGCAA
TGTATGCCCCTCCCATCAGGGGACGAATTAGCTGTTCATCAAAT---
ATTACAGGGCTGCTATTAACAAGAGATGGTGGCACTAACATGACTGAT-------------------------
-------------------------------------------------------------------------
----------
AATGGTACAGAGACCTTCAGACCTGCAGGAGGAGATATGAGGGACAATTGGAGAAGTGAATTATATAAATACA
AAGTAGTAAGAATTGAGCCATTA---GGAATAGCACCCACCAAGGCAAAGAGAAGAGTGGTG-----------
----------------------------CAAAGAGAAAAAAGAGCA---GTGGGA---ATAGGAGCT------
---TTGTTCATTGGGTTCTTGGGAGCA---GCAGGAAGCGCTATG---GGCGCAGCGTCA---
ATGACGCTGACGGTACAG---GCCAGACAA---TTATTG---TCTGGTATAGTGCAACAGCAGAAC---
AATTTG---CTGATG---GCTATTGAGGCGCAACAGCATATGTTGCAACTCACAGTTTGGGGCATC
>HQ595783
TTCTACTGTAATTCAACACCACTATTTAATGAGAGCTTTAGAAGATCAAATGAAAGG----------------
-------------------------------------------------------------------------
----------------------------
AGTGAAGGGAATATCACTCTCTCATGTAGAATAAAACAAATTATAAACTTGTGGCAGGAAGTAGGAAAAGCAA
TGTATGCCCCTCCCATCAGAGGACAAATTAGATGTTCATCAAAT---
ATTACAGGGCTGCTATTAATAAGAGATGGTGGGGAAAATGGGACAGAG-------------------------
-------------------------------------------------------------------------
----------
GGAAATAATGAGACCTTCAGACCTGGAGGGGGAGATATGAAGGACAATTGGAGAAGTGAATTATATAAATATA
AAGTAGTAAAAATTGAACCATTG---GGAGTAGCACCCACCAAGGCAAAGAGAAGAGTGGTG-----------
----------------------------CAGAGAGAAAAAAGAGCA---GTGGGAACGCTAGGAGCT------
---ATGTTCCTTGGGTTCTTGGGAGCA---GCAGGAAGCACTATG---GGCGCAGCGTCA---
ATAACGCTGACGGTACAG---GCCAGACAA---TTATTG---TCTGGTATAGTGCAACAGCAGAAC---
AATTTG---CTGAGG---GCTATTGAGGCGCAACAACATATGTTGCAACTCACAGTCTGGGGCATC
>JF680919
TTCTACTGTAATACAACACAGCTGTTTAATAGTACTTGGACCACTGATACTTGGAATAGTACTAATGGGTCAA
GC-----------------------------------------------------------------------
-------------------------
AATAGCAATGACACTATTATACTCCCATGTAGAATAAAACAAATTATAAACAGGTGGCAGGAAGTAGGAAAAG
CAATGTATGCCCCTCCCATCAGCGGACAAATTATATGTCCATCAAAT---
ATTACAGGGCTACTATTAACAAGAGATGGTGGTAATGATAATAGCACC-------------------------
-------------------------------------------------------------------------
-------
AAAAACGGGACTGAAACCTTCAGACCTGGAGGAGGAGATATGAAGGACAATTGGAGAAGTGAATTATATAAAT
ATAAAGTAGTAAGAATTGAGCCATTA---GGAGTAGCACCCACCAGGGCAAAGAGAAGAGTGGTG--------
-------------------------------CAGAGAGAAAAAAGAGCA---GCG------TTCGGAGCT---
------TTGTTCCTTGGGTTCTTGGGAGCA---GCAGGAAGCACTATG---GGCGCAGCGTCC---
CTGACGCTGACGGTACAG---GCCAGAACA---TTATTG---TCTGGTATAGTGCAACAGCAGAAC---
AATTTG---CTGAGA---GCTATTGAGGCGCAACAGCATCTGTTGCAACTCACAGTCTGGGGCATC
>JF680920
TTCTACTGTAATACAACAAAACTGTTTAATAGTACTTGGCCTGTTAATGGTAATGATACTGGGTCAAATATC-
-------------------------------------------------------------------------
----------------------------
ACTGAAGAAAATATTACACTCCCATGTAGAATAAAACAAATTATAAACATGTGGCAGGAAGTAGGAAAAGCAA
TGTATGCCCCTCCCATCAAAGGACTAATTAGATGTTCATCAAAC---
ATTACAGGGATACTACTAACAAGAGATGGTGGGGACGGGAGCAAT----------------------------
```


GCCAACGAGACCTTCAGACCTGGAGGAGGGAATATGAGGGACAATTGGAGAAGTGAATTATATAAATATAAAG
TAGTACAAATTGAACCATTA---GGAGTAGCACCCACCAAGGCAAAGAGAAGAGTGGTG--------------
-------------------------CAGAGAGAAAAAAGAGCA---GTGGGA---ATAGGAGCT---------
TTGTTCCTTGGGTTCTTGGGAGCA---GCAGGAAGCACTATG---GGCGCAGCATCA---
ATGACGCTGACGGTACAG---GCCAGACAA---TTATTG---TCTGGTATAGTGCAACAGCAGAAC---
AATTTG---CTGAGA---GCTATTGAGGCGCAACAGCATCTGTTGCAACTCACAGTCTGGGGCATC
>HQ595786
TTCTACTGTGATACAACACAGCTATTTAATAGTACTTGGAATATAACACAGCTGTCTAATAATAATAATACAG
GAAAT--
-------------------GAC---
ATAAATAACACAACAATCGTGCTTCCATGTAGAATAAAACAAATTATAAACAGGTGGCAGGAAGTAGGAAAAG
CAATGTATGCCCCTCCCATCAGAGGAGTAATTAAATGTTCATCAAAT---
ATTACGGGGCTGATTTTAGCAAGAGATGGTGGAATTAATGAGACT----------------------------
----

AATGACACCGAGATCTTTCGACCTGCAGGAGGAGATATGAGGGACAATTGGAGAAGTGAATTATATAAATATA
AAGTAGTAAAAATTGAACCATTA---GGAGTAGCACCCACCAAGGCAAAGAGAAGAGTGGTG-----------
----------------------------CAGAGAGAAAAAAGAGCA---GTGGGAACATTAGGAGCT------
---ATGTTCCTTGGGTTCTTGGGAGCA---GCAGGAAGCACTATG---GGCGCAGCGTCA---
ATAACGCTGACGGTACAG---GCCAGACAA---TTATTG---TCTGGTATAGTGCAACAGCAGAGC---
AATTTG---CTGAGA---GCTATTGAGGCGCAACAGCACCTGTTGCAACTCACAGTCTGGGGCATC
>JF680928
TTCTACTGTAATACAACAAAACTGTTTAATAGTACTTGGGAGAATGGTACTTGGCAGAATACTACAGAG----
----

TTGAATGGCACTATCACTCTTCCTTGTAGAATAAAACAAATTATAAACATGTGGCAGGAAGTAGGGAAAGCAA
TGTATGCCCCTCCCATCAGAGGACAAATTAAGTGTTCATCAAAT---
ATTACAGGGATACTATTAACAAGAGATGGGGGTAATAGCACCAAC----------------------------
----

AACACTGAGACCTTCAGACCTGCAGGAGGAAATATGAAGGACAATTGGAGAAGTGAATTATATAAATATAAAG
TAGTAAAAATTGAACCATTA---GGAGTAGCACCCACCAAGGCAAAGAGAAGAGTGGTG--------------
-------------------------CAGAGAGAAAAAAGAGCA---GCGGGA---CTAGGAGCT---------
GTGCTCATTGGGTTCTTGGGAGCA---GCAGGAAGCACTATG---GGCGCAGCGTCA---
CTAACGCTGACGGTACAG---GCCAGACAA---TTATTG---TCTGGTATAGTGCAACAGCAGAAT---
AATCTG---CTGAGA---GCTATTGAGGCGCAACAACATCTGTTGCAACTCACAGTCTGGGGCATT
>JF680929
TTCTACTGTAATACATCACAGCTGTTTAATAGTACTTGGGAGAATAGTGCTGGGGAGAATAATACAGAGTCAA
AC---

GCTACAGAGGTAAATATCATACTCCCATGCAGAATAAAACAAATCATAAACAGGTGGCAGGAGGTAGGAAAAG
CAATGTATGCCCCTCCCATCAGAGGAAATATCAGCTGTTCATCAAAT---
ATTACAGGGCTGATATTGACAAGAGATGGTGGTAACGAAAATCGC----------------------------
----

ACAGGCAACGAGACCTTCAGGCCGGGAGGGGGAGATATGAAAGACAATTGGAGGAGTGAATTATATAAATATA
AAGTAGTAAAAATTGAGCCATTA---GGAATAGCACCCACAAAGGCAAGGAGAAGAGTGGTG-----------
----------------------------CAGAGAGAAAAAAGAGCA---GTGACA---ATAGGAGCT------
---GTACTCCTTGGGTTCTTGGGAACA---GCAGGAAGCACTATG---GGCGCAGCGGCA---
GTGACGCTGACGGTACAG---GCCAGACAA---TTACTG---TCTGGTATAGTGCAACAGCAAAAC---
AATTTG---CTGAGA---GCTATTGAGGCGCAACAACATCTGTTGCAACTCACAGTCTGGGGCATC
>JF680934
TTCTACTGTAATTCAACACAGCTGTTTAATAGTACTTGGACGTTTAATGGTACTTGGATGTTTAATGGTACTA
CTTGGAATAAT--
----------ACTGATAAC------
AGTAACCATACCAATATTACACTCCCATGCAGAATAAAACAAATTATAAACATGTGGCAGAGAGTAGGAAAGG
CAATGTATGCCCCTCCCATCAGAGGACTAATTAGATGTAAATCGAAT---
ATTACAGGGATACTATTAACAAGGGATGGTGGTGATGGGAACAAT----------------------------

----

AGTGAAGAGATCTTCAGACCTGGAGGAGGAGATATGAAGGACAATTGGAGAAGTGAATTATATAAGTACAAAG
TAGTAAAAATTGAGCCATTA---GGAGTAGCACCCACTAAGGCAAGGAGAAGAGTGGTG--------------
-------------------------CAGAGAGAAAAAAGAGCA---GTGGGAACACTAGGAGCT---------
ATGTTCCTTGGGTTCTTGGGAGCA---GCAGGAAGCACTATG---GGCGCAGCGTCG---
GTGACGCTGACGGTACAG---GCCAGACTA---TTATTG---TCTGGTATAGTGCAACAGCAGAGC---
AATTTG---CTGAGA---GCTATTGAGGCGCAACAGCATATGTTGCAACTCACAGTCTGGGGCATC
>HQ595801
TTCTACTGTAATACAACACAACTGTTTAATAGCACTTGGAATAGTACTGAGAATGTTGATAAT----------
----

AGGACAGATAACATCACGCTCCCATGCAAAATAAAACAATTTATAAATATGTGGCAGGAAGTAGGAAAAGCAA
TGTATGCCCCTCCCATTAGAGGACAAATTAGCTGTTCATCAAAT---
ATTACAGGGCTTCTATTAACAAGAGATGGTGGTAAAGATAACAATAGCAT-----------------------
----

GACAACAATATTGAGGTCTTTAGACCTGGAGGAGGAAATATGAAAGACAATTGGAGAAGTGAATTATATAAAT
ATAAAGTAGTAAAAATTGAACCATTA---GGAATAGCACCCACCAAGGCAAAGAGAAGAGTGGTA--------
-------------------------------CAGAGAGAAAAAAGAGCA---GTGGGC---CTAGGAGCT---
------TTGTTCCTTGGGTTCTTGGGAACA---GCAGGAAGCACTATG---GGCGCAGCGTCA---
ATAACGCTGACGGTACAG---GCCAGACAA---TTATTG---TCTGGTATAGTGCAACAGCAGAAC---
AATCTG---CTGAGA---GCTATTGAGGCGCAGCAGCGTATGTTGCAACTCACAGTCTGGGGCATC
>HQ595802
TTCTACTGTAATACAACACAACTGTTTAATAGCACTTGGAAACTGGTTAATAACACTTGGAGTAAGATTGATA
ATAAGAATGAG--
ACTGGTAATAGTAATATC---------
ACACTCCCTAGTAATATCACACTCCCATGTAGAATAAAACAAATTATAAACATGTGGCAGAAAGTAGGAAAAG
CAATGTATGCCCCTCCTATTGGAGGACAAATTAAATGTTCATCAAAC---
ATTACAGGGCTAATATTAGTAAGAGATGGTGGTAATGACACAGGA----------------------------
----

GGCAACGAGACCTTCAGACCTGCAGGGGGAGATATGAGGGACAATTGGAGAAGTGAATTATATAAATACAAAG
TAGTAAAAATTGAACCATTA---GGAATAGCACCCACCAGGGCAAAGAGAAGAGTGGTG--------------
-------------------------CAAAGAGAAAGAAGAGCA---GTGACA---CTGGGAACC---------
ATGCTCCTTGGGTTCTTGGGAGCA---GCAGGAAGCACTATG---GGCGCAGCGTCC---
TTGACGCTGACGGTACAG---GCCAGACAA---TTATTG---TCTGGTATAGTGCAACAGCAGAAC---
AATCTG---CTGAGG---GCTATTGAGGCGCAACAGCATCTGTTGCAGCTCACAGTCTGGGGCATC
>HQ595804
TTCTACTGTAATACATCAGAACTGTTTAATAGTACTTGGAATGATACTGCAGGGTTAAATAACACTAACACTA
CA---

GACCAAGGCACAATCACACTCCCATGCAGAATAAAACAAATTATAAACATGTGGCAAAAAGTAGGAAAAGCAA
TGTATGCCCCTCCCATTGCAGGACAAATTAACTGCTCAACAAAT---
ATTACAGGAATACTACTAACAAGAGATGGTGGTGGTAATAGCAGT----------------------------
----

AACACCACCGAGACCTTCAGACCAGCAGGAGGAGATATGAGGGATAATTGGAGAAGTGAATTATATAAATATA
AAGTAGTAAAAATTGAACCATTA---GGAATAGCACCCACCAAGGCAAAAAGGAGAGTGGTG-----------
----------------------------CAAAGAGAGAAAAGAGCA---ATAGGGGGATTGGGAGCT------
---TTGTTCCTTGGGTTCTTGGGAGCA---GCAGGAAGCACTATG---GGCGCAGCGTCA---
CTAACGCTGACGGTACAG---GCCAGACAA---TTATTG---TCTGGTATAGTGCAGCAGCAGAAC---
AATCTG---CTGAGA---GCTATTGAGGCGCAACAACATTTGTTGCAACTCACAGTCTGGGGCATC
>HQ595806
TTCTACTGTAATACAACACAATTGTTTAATAGTACTTGGAATATAAAAGAGAATACAACAAAACGGGATAATA
GTACT--
-------------------GAA---
AGTAATAGTACTACTATCGTACTCCCATGCAGAATAAAACAAATTATAAACAGATGGCAGGAAGTAGGAAAAG
CAATGTATGCCCCTCCCATCAGCGGAACAATTAAATGCTCATCAAAT---
ATTACAGGGTTACTATTAACAAGAGATGGGGGTAATAATAACGAGACC-------------------------

----

AATGGAACTGAGGTCTTCAGACCTGGAGGAGGAGATATGAGGGACAATTGGAGAAGTGAATTATATAAATATA
AAGTAGTAAAAATTGAACCATTA---GGAGTAGCACCCACCCAGGCAAGGAGAAGAGTGGTG-----------
---------------------------CAGAGAGAAAAAAGAGGA---GTAGGA---CTAGGAGCT------
---TTGTTCCTTGGGTTCTTGGGAGCA---GCAGGGAGCACTATG---GGCGCAGCGTCA---
ATGACGCTGACGGTACAG---GCCAGACAA---CTATTG---TCTGGTATAGTGCAACAGCAAAAC---
AATTTG---CTGAGA---GCTATTGAGGCGCAACAGCGTCTGTTGCAACTCACAGTCTGGGGCATC
>JF680937
TTCTACTGTAATACAACACAACTGTTTAATAGCGATTGGAATATTACTGAAAGGGCAAATGACACTGAA----
----

GGAAATGGCACAATTACACTCCCATGCAGAATAAAACAAATTATAAACATGTGGCAGGAAGTAGGAAAAGCAA
TGTATGCCCCTCCCATCAAAGGAGAAATTGGATGTTCATCAACC---
ATCACAGGGATACTCTTAACAAGAGATGGTGGAACGAACAGTACC---------------------------
----

AATGAGACCTTCAGACCTGGAGGAGGGGATATGAGAGACAATTGGAGAAGTGAATTATTTAAATATAAAGTAG
TACAAATTGAACCATTA---GGAATAGCACCCACCAGGGCAAAGAGAAGAGTGGTG-----------------
----------------------CAGAGAGAAAAAAGAGCA---GTGGGA---CTAGGAGCC---------
TTGTTCCTTGGGTTCTTGGGAGCA---GCAGGAAGCACTATG---GGCGCAGTGTCA---
ATGACGCTGACGGTACAG---GCCAGACAA---TTATTG---TCTGGTATAGTGCAACAGCAGAAC---
AATTTG---CTGAGA---GCTATTGAGGCGCAACAGCATCTGTTGCAACTCACAGTCTGGGGCATC
>D10112
TTCTACTGTAATACAACACAGCTGTTTAATACTACTTGGCTGTTTAATGGTACTTGGAATGATACTGAAGGGT
TAAAT---

AACACTGAAAGAAATATTACACTTCCATGCAGAATAAAACAAATTATAAACAGGTGGCAGGAAGTAGGAAAAG
CAATGTATGCCCCTCCCATCACAGGAACAATTAGCTGTTCATCAAAT---
ATTACAGGGCTGCTATTAACAAGAGATGGTGGTAGAGGCGAAAAT---------------------------
----

GAGACCGAGACCTTCAGACCTGGAGGAGGAGATATGAGGGACAATTGGAGAAGTGAATTATATAAATATAAAG
TAGTAAAAATTGAACCATTA---GGAGTAGCACCCACCAAGGCAAAGAGAAGAGTGGTG--------------
------------------------CAGAGAGAAAAAAGAGCA---GTGGGAGCGATAGGAGCT---------
CTGTTCCTTGGGTTCTTGGGAGCA---GCAGGAAGCACTATG---GGCGCAGTGGCA---
CTGACGCTGACGGTACAG---ACCAGACAA---TTATTG---TCTGGTATAGTGCAACAGCAGAAC---
AATCTG---CTGAGG---GCTATTGAGGCGCAACAGCATCTGTTGCAACTCACAGTCTGGGGCATC
>Y13716
TTCTACTGTAAGACAGCACAACTGTTTAATAGTACTTGGAATAGTACTGGAAATGGTACTATAAAGTCAAAT-
----

ACCACTGAAATTATCACACTCCCATGTAGAATAAAACAAATTGTAAACATGTGGCAGGAAGTAGGAAAAGCAA
TGTATGCCCCTCCCATCACAGGACAAATTAGATGTGCATCACAT---
ATTACAGGGCTACTATTAACAAGAGATGGAGGTAGGGAGAACAATACC----------------------
----

AACGAGACCGAGACCTTCAGACCTGGGGGAGGAGATATGAGGGACAATTGGAGAAGTGAATTATATAAATATA
AAGTAGTAAAAATTGAACCATTA---GGAGTAGCACCCACCAAGGCAAAGAGAAGAGTGGTG-----------
---------------------------CAGAGAGAAAAAAGAGCA---GTGGGAATGATAGGAGCT------
---ATGTTCCTTGGGTTCTTGGGAGCA---GCAGGAAGCACTATG---GGCGCAGCCTCA---
CTGACGCTGACGGTACAG---GCCAGACTA---TTATTG---TCTGGTATAGTGCAGCAGCAAAAT---
AATTTG---CTGAGG---GCTATTGAGGCGCAACAGCATCTGTTGCGACTCACAGTCTGGGGCATC
>JF706393
TTCTACTGTAATACATCAAAACTGTTTAATAGTACTTGGACTAATATTACTGAAGGGTTAAATAAAACTGAA-
----

AGAAATGGCACATTCATACTCCCATGCAGAATAAAACAAATTATAAACAGGTGGCAGGAAGTAGGAAAAGCAA
TGTATGCCCCTCCCATCAGAGGACAAATTAGATGTTCATCAAAT---
ATTACAGGGCTGCTATTAATGAGAGATGGTGGTAATACTAACGAGAGC------------------------

----

AAGACCACCGAGGTCTTCAGACCTGGAGGAGGAGACATGAGGGACAATTGGAGAAGTGAATTATATAAATATA
AAGTAGTAAGAATTGAACCATTA---GGAGTAGCACCCACCAAGGCAAAGAGAAGAGTGGTG-----------
----------------------------CAGAGGGAAAAAAGAGCA---GTGGGA---ATAGGAGCT------
---TTGTTCCTTGGGTTCTTGGGAGCA---GCAGGAAGCACTATG---GGCGCAGCGTCA---
ATGACGCTGACGGTACAG---GCCAGACAA---TTATTG---TCTGGTATAGTGCAGCAGCAGAAC---
AATCTG---CTGAGG---GCTATTGAGGCGCAACAGCATCTGTTGCAACTCACAGTCTGGGGCATC
>JN786861
TTCTACTGCAATTCAACACAACTGTTTAATAGTACTTGGAATGTTAAAGGTACTTGGAATGGTACTGAAGAGT
CAAATAAC---
-------------------AAT---
GAAGGAAATGACACAATCACACTCCAATGCAGAATAAAACAAATTATAAACATGTGGCAGGAAGTAGGAAAAG
CAATGTATGCCCCTCCCATCAGAGGACAAATTAGATGTTCATCAAAT---
ATTACAGGGCTGATATTAACAAGAGATGGTGGTAATGAGAATACC----------------------------
----

ACCACAGAGATCTTCAGACCTGGAGGAGGAAATATGAGGGACAATTGGAGAAGTGAATTATATAAATATAAAG
TAGTAAAAATTGAACCATTA---GGAGTAGCACCCACCAGGGCAAAGAGAAGAGTGGTG--------------
-------------------------CAGAGAGAAAAAAGAGCA---GTGGGA---CTAGGAGCT---------
TTGTTCCTTGGGTTCTTGGGAGCA---GCAGGAAGCACTATG---GGCGCAGCGTCA---
CTGACGCTGACGGTACAA---GCCGGACAA---TTATTG---TCTGGTATAGTGCAACAGCAGAAC---
AACTTG---CTGAGG---GCTATTGAGGCGCAACAGCACCTGTTGCAACTCACAGTCTGGGGCATC
>AJ535594
TTCTACTGTGATACAACACAACTGTTTAATGGGAGTGTTCCTAAAGGAAATAACACTGAAGGAAGTAGT----
----
----------------------------AATGACCTATCACACTCCCATGTT---
AGAATAAAACAAATTATAAACAGGTGGCAGGAAGTAGGAAAAGCAATGTATGCCCCTCCCATCAGTGGAATAG
TTACATGTTCATCAAAT---ATTACAGGGCTGCTATTAACAAGAGATGGGGGTGTGACTAACGATACT-----
----

AACGGCACTGAGATTTTCAGACCTGGAGGAGGAAATATGAGGGACAATTGGAGAAGTGAATTATATAAATATA
AAGTAGTAAGAATTGAACCATTA---GGAGTAGCACCCACTACGGCAAAGAGAAGAGTGGTG-----------
----------------------------CAAAGAGAAAAAAGAGCA---GTGGGA---CTAGGAGCT------
---ATGTTCCTTGGGTTCTTGGGAGCA---GCAGGAAGCACTATG---GGCGCAGCGTCA---
ATAACGCTGACGGTACAG---GCCAGACTA---TTATTG---TCTGGTATAGTGCAACAGCAAAAC---
AATTTG---CTGAGG---GCTATTGAGGCACAACAGCATCTGTTGCAGCTCACAGTCTGGGGCATC
>AJ535607
TTCTACTGTAATACATCACAACTGTTTAATAGTACTTGGAATGAGAATAATACTTGGACAGAGTCAAATAGC-
----

ACTGGAGAAAATATCACACTCCCATGTAGGATAAAACAAATTATAAACATGTGGCAGGATGTAGGAAGAGCAA
TGTATGCCCCTCCCATCAGTGGACAAATTAGATGTTCATCAAAT---
ATTACAGGGCTGCTATTAGTAAGAGATGGTGGTATTAACGTGACCAAC-------------------------
----

GAGGCCGGCACCGAGGTCTTCAGACCTGGGGGAGGAGATATGAAGGACAATTGGAGAAGTGAATTATATAAAT
ATAAAGTAGTAAAAATTGAACCATTA---GGAATAGCACCCACCAAGGCAAAAAGAAGAGTGGTG--------
-------------------------------CAGAGAGAAAAAAGAGCA---GCA------ATAGGAGCT---
------ATGTTCCTTGGGTTCTTGGGAGCA---GCAGGAAGCACTATG---GGCGCAGCGTCA---
CTGACGCTGACGGTACAG---GCCAGACAA---TTATTG---TCTGGTATAGTGCAACAGCAGAAC---
AATCTG---CTGAGG---GCTATTGAGGCGCAACAGCATCTGTTGCAACTCACTGTCTGGGGCATC
>AJ535612
TTCTACTGTAATTCAACAAAACTGTTTAGTAGTACTTGGAATAGTAATAGTACTTGGAATAGTGCTGAAGAGT
CA---

AATAGCACTGAAGTTATCACACTCCCATGCAGAATAAAACAAATTATAAACATGTGGCAGGAAGTAGGAAAAG
CAATGTATGCCCCTCCCATCAGAGGACAAATTAGATGCTCATCAAAT---
ATTACAGGGCTGCTATTAACAAGAGATGGTGGTAAGAATCCGAACGAG-------------------------
----

AACACCACCGAGACCTTCAGACCTGGAGGAGGAAATATGAGGGACAATTGGAGAAGTGAATTATATAAATATA
AAGTAGTAAAAATTGAACCATTA---GGAGTAGCACCCACCAAGGCAAGGAGAAGAGTGGTG-----------
----------------------------CAGAGAGAAAAAAGAGCA---ATAGGA---ATGGGAGCT------
---TTGTTCCTTGGGTTCTTGGGAGCA---GCAGGAAGCACTATG---GGCGCAGCGTCA---
CTGACGCTGACGGTACAG---GCCAGGCTA---TTATTG---TCTGGTATAGTGCAACAGCAGAAT---
AATTTG---CTGAGG---GCTATTGAGGCGCAACAGCATCTGTTGCAACTCACAGTCTGGGGCATC
>AJ418531
TTCTACTGTAATACTACTAAGCTGTTTAATAGTACTTGGAATAGTACTTGGACTGGGGATACTACT-------
----

GGAGATGACAATATCACACTCCCATGTAGAATAAAACAATTTATAAACAGGTGGCAGGAAGTAGGAAAAGCAA
TGTATGCCCCTCCCATCAGTGGACAAATTAGATGTTTATCAAAT---
ATTACAGGGCTGCTATTAACAAGAGATGGTGGTAGTACCAAGAACGAGACC----------------------
----

AGTAACCAGACCGAGATTTTCAGACCTGGAGGAGGAGATATGAGGGACAATTGGAGAAGTGAATTATATAAAT
ATAAAGTAGTAAAAATTGAGCCATTA---GGAGTAGCACCTACCAGGGCAAAGAGAAGAGTGGTG--------
-------------------------------CAAAGAGAAAAAAGAGCA---GTGGGAACAATAGGAGCT---
------ATGTTCCTTGGGTTCTTGGGAGCA---GCAGGAAGCACTATG---GGCGCAGCGTCA---
TTGACGCTGACGGTACAG---GCCAGACTA---TTATTG---TCTGGTATAGTGCAGCAGCAAAGC---
AATTTG---CTGAGG---GCTATTGAGGCGCAACAGCATCTGTTGCAACTCACAGTCTGGGACATC
>AJ418494
TTCTACTGTAATACAACACAACTGTTTAATAGTACCTGGAATGTTAGTAATACTGAAGGATTAAATATCAATA
TCACT--

GAAAGAAATGACACATTCACACTCCCATGCAGAATAAAACAAATTATAAACATGTGGCAGGAAGTAGGAAAAG
CAATGTATGCCCCTCCCATCAAAGGAAAAATTAGATGTTCATCAAAT---
ATTACAGGGCTGCTATTAACAAGAGATGGTGGTGGTAACGAGAGC----------------------------
----

AGCAGCAGCGAGACCTTCAGACCTGGAGGAGGAGATATGAGGGACAATTGGAGAAGTGAATTATATAAATATA
AAGTAGTAAGAATTGAACCATTA---GGAGTAGCACCCACTAAGGCAAAAAGAAGAGTGGTG-----------
----------------------------CAGAGAGAAAAAAGAGCA---GCA------CTAGGAGCT------
---ATGTTCCTTGGATTCTTGGGAGCA---GCAGGAAGCACTATG---GGCGCAGCGTCA---
ATGGCGCTGACGGTACAG---GCCAGACAA---TTATTG---TCTGGTATAGTGCAACAGCAGAAT---
AATCTG---CTGAGA---GCTATTGAGGCGCAACAGCACCTGTTGCAACTCACAGTCTGGGGCATT
>AJ418495
TTCTACTGTAATACAACAAAACTGTTTAATAGTACTTGGCAAAATGAGACTAGCGGGTCCATTAACATAACTG
AC---

ATTGGAGAAAATATCACACTCCCATGCAGAATAAAACAAATTATAAACCTGTGGCAGGGAGTAGGAAAAGCTA
TGTATGCCCCTCCCATCCAAGGACAAATTAGTTGTTCATCAAAT---
ATTACAGGGCTGCTATTAACAAGAGATGGTGGTAACAACAACGAGACA-------------------------
----

AACAGGACCGAGACCTTCAGACCTGGAGGAGGAAATATGAGGGACAATTGGAGAAGTGAATTATATAAATATA
AAGTAGTAAAAATTGAGCCATTA---GGAGTAGCACCCACCAAGGCAAAGAGAAGAGTGGTG-----------
----------------------------CAGAGAGAAAAAAGAGCA---GTAACG---CTAGGAGCT------
---ATGTTCCTTGGGTTCTTGGGAGCA---GCAGGAAGCACAATG---GGCGCAGCGTCA---
ATGACGCTGACGGTACAG---GCCAGACTA---TTATTG---TCTGGTATAGTGCAACAGCAGAAC---
AACCTG---CTGAGG---GCTATTGAGGCGCAACAGCATCTGTTGCAACTCACAGTCTGGGGCATC
>AJ418519
TTCTACTGTGATTCATCACAGCTGTTTAATGGTACTTGGAATGATACTAATACTAATGAA-------------
----

GGAAATGGCACAATCACACTCCAATGCAAAATAAAACAAATTATAAACCTGTGGCAGGAAGTAGGAAAAGCAA
TGTATGCCCCTCCCATCAGAGGACGAATTAGATGTTCATCAAAT---
ATTACAGGGCTGTTATTAACAAGAGATGGTGGTAATACTAATGAC----------------------------
----

```
-------------
ACAGACGAGATATTCAGACCTGGAGGAGGAGATATGAGGGACAATTGGAGAAGTGAATTATATAAATATAAAG
TAGTTAAAATTGAACCATTA---GGAGTGGCACCCACCAAGGCAAAGAGAAGAGTGGTG--------------
-------------------------CAGAGAGAAAAAAGAGCA---GTGGGA---ATAGGAGCT---------
GTGTTCCTTGGGTTCTTGGGAGCA---GCAGGAAGCACTATG---GGCGCAGCGTCA---
ATGACGCTGACGGTACAG---GCCAGACTA---TTATTG---TCTGGTATAGTGCAACAGCAGAAC---
AATTTG---CTGAGG---GCTATTGAGGCGCAACAGCATCTGTTGCAGCTCACAGTCTGGGGCATC
>AJ418521
TTCTACTGTAATACATCACAACTATTTAATAGTATTTGGAATAGTACTGAAAAGACAAATATC----------
-------------------------------------------------------------------------
----------------------------
ACTAAAGGAATTATCACACTCCCATGCAGAATAAAACAAATTATAAACATGTGGCAGGAAGTAGGGAAAGCAA
TGTACGCCCCTCCCATCAGAGGAAATATTAGTTGTTCATCAAAT---
ATTACAGGGCTACTATTAACAAGAGATGGTAGTAACGGGAACGGGACC-------------------------
-------------------------------------------------------------------------
-------
GGGAACAGGACCGAGATCTTTAGACCTGGAGGAGGAGATATGAGGGACAATTGGAGAAGTGAATTATATAAAT
ATAAAGTAGTAAAAATCGAACCATTA---GGAGTAGCACCCTCCAAGGCAAAGAGAAGAGTGGTG--------
-------------------------------CAGAGAGAAAAAAGAGCA---GTGACA---TTAGGAGCT---
------TTGTTCCTTGGGTTCTTGGGAGCA---GCAGGAAGCACTATG---GGCGCAGCGACA---
ATGACGCTGACGGTACAG---GCCAGACTA---TTATTG---TCTGGTATAGTGCAACAGCAGAAC---
AATTTG---CTGAGG---GCTATTGAGGCGCAACAGCATCTGTTGCAACTCACAGTCTGGGGCATC
>U36869
TTCTACTGTAACACAACGCAACTGTTTAATAGTACTTGGAATGGTACTGAAGGGTCTTGGAATGATACTAAA-
-------------------------------------------------------------------------
----------------------------
GGGCCAGAAAACATCACACTCCCATGCAGAATAAAACAAATTATAAACATGTGGCAAGAAGTAGGAAAAGCAA
TGTATGCCCCTCCCATTAGTGGACAAATTAGATGCTCATCAAAT---
ATTACAGGACTGCTATTAACAAGAGATGGTGGTAAGGGCAATAATACC-------------------------
-------------------------------------------------------------------------
----------
AATACCACCGAGATCTTTAGACCGGGAGGAGGAGATATGAGGGACAATTGGAGAAGTGAATTATATAAATATA
AAGTAGTAAGAATTGAACCATTA---GGAGTAGCACCCACCAAGGCAAAGAGAAGAGTGGTG-----------
----------------------------CAGAGAGAAAGGAGATCA---ATAACA---CTAGGAGCT------
---ATGTTCCTTGGGTTCTTGGGAGCA---GCAGGAAGCACTATG---GGCGCAGCGTCA---
ATAACGCTGACGGTACAG---GCCAGACAA---TTATTG---TCTGGTATAGTGCAACAGCAGAAC---
AATTTG---CTGAGG---GCTATTGAGGCGCAACAGCATCTGTTGCAGCTCACAGTCTGGGGCATC
>U36872
TTCTACTGTAATACAACAAAATTATTCAATAGTACTTGGACTTGGAATGATACTGAAGGGTCAGATAGC----
-------------------------------------------------------------------------
----------------------------
ACTGAAGGAAGTATCACACTCCCATGCAGAATAAAACAATTTATAAACATGTGGCAGGAAGTAGGAAAAGCAA
TGTATGCCCCTCCCATCAGAGGACAAATTAGATGTTCATCAAAT---
ATTACAGGGCTGCTATTAACAAGAGATGGTGGTAATGATAACAAGACT-------------------------
-------------------------------------------------------------------------
----------
AACACCACCGAGACCTTCAGACCTGGAGGAGGAGATATGAGGGACAATTGGAGAAGTGAATTATACAAATATA
AAGTAGTAAAAATTGAACCATTA---GGAGTAGCACCCACTAAGTCAAGGAGAAGAGTGGTG-----------
----------------------------CAGAGAGAAAAAAGAGCA---GTGGGA---ATAGGAGCT------
---GTGTTCCTTGGGTTCTTGGGAGCA---GCAGGAAGCACTATG---GGCGCAGCGTCA---
CTGACACTGACGGTACAG---GCCAGACTA---TTATTG---TCTGGTATAGTGCAACAGCAGAAC---
AATTTG---CTGAGG---GCTATTGAGGCGCAACAGCATCTGTTGCAACTCACAGTCTGGGGCATC
>AJ286342
TTCTACTGTAATACAAAACAGCTGTTTAATTGGAGTAGTACTTGGAATGATACTATAAGGTCAGAT-------
-------------------------------------------------------------------------
----------------------------
GCTCCAGAAATTATCACACTCCCATGCAGAATAAAACAAATTATAAACAGGTGGCAGGAAGTAGGAAAAGCAA
TGTATGCCCCTCCAATCGAAGGACAAATTAGATGTTCATCAAAC---
ATTACAGGGCTGCTATTAACAAGAGATGGTGGTAGTAGCAACAATACC-------------------------
-------------------------------------------------------------------------
```

AACAATACCACCGAGATCTTCAGACCTGGAGGGGGAAATATGAAAGACAATTGGAGAAGTGAATTATATAAAT
ATAAAGTAGTAAAAATTGAGCCATTA---GGAGTAGCACCCACCAAGGCAAAGAGAAGAGTGGTG-------
------------------------------CAGAGAGAAAAAAGAGCA---ATAGGAACGCTGGGAGCT---
------ATGTTCCTTGGGTTCTTGGGAGCA---GCAGGAAGCACTATG---GGCGCAGCGGCA---
ATGACGCTGACGGTACAA---ACCAGACAA---TTATTG---TCTGGTATAGTGCAACAGCAAAAT---
AATTTG---CTGAGG---GCTATTGAGGCGCAACAGCATCTGTTGCAACTCACAGTCTGGGGCATC
>U36875
TTCTACTGTAATACAACAAAACTGTTTAATAGTATTTGGATGCCTAATGGTACTTGGAATAGTACTGACACGT
CA--

AATAGCACTGAGATCAGGCTCCCATGCAGAATAAAACAAATTGTAAACATGTGGCAGGAAGTAGGAAAAGCAA
TGTATGCCCCTCCCATCAGTGGACGAATTAGATGTGTATCAAAT---
ATTACAGGGCTGCTATTAACAAGAGATGGTGGTAATAACAACGAG---------------------------
----

ACCACCGAGATCTTCAGACCTGGAGGAGGAGATATGAGGGACAATTGGAGAAGTGAATTATACAAATACAAAT
TAATAAAAATTGAACCATTA---GGAATAGCACCCACCAAGGCAAAAAGAAGAGTGGTG-------------
------------------------CAAAGAGAAAAAAGAGCA---GTGGGA---ATAGGAGCT---------
GTGCTCCTTGGGTTCTTGGGAGCA---GCAGGAAGCACTATG---GGCGCAGCGTCA---
CTGACGCTGACGGTACAG---GCCAGACTA---TTACTG---TCTGGTATAGTGCAACAGCAGAAC---
AATTTG---CTGAGG---GCTATTGAGGCGCAACAGCATATGTTGCAACTCACAGTCTGGGGCATC
>U36879
TTCTACTGTAATTCAACACAATTGTTTAATAGTACTTGGAATAATGGTACTAGGAATAATACTGAAGGAGGTA
AC--

ATTACAGACACAATTACACTCCCATGTAGAATAAAACAAATTATAAACATGTGGCAGGAAGTAGGAAAAGCAA
TGTATGCCCCTCCCATCAGAGGACAAATTAGATGTTCATCAAAT---
ATTACAGGGCTGTTATTAACAAGAGATGGTGGCAGGAATGAGACC---------------------------
----

ACCACCACTGAGATCTTCAGACCTGGGGGAGGAAATATGAAGGACAATTGGAGAAGTGAATTATATAAATATA
AAGTAGTAAAAATTGAACCATTA---GGAGTAGCACCCACCAAGGCAAAGAGAAGAGTGGTG----------
---------------------------CAGAGAGAAAAAAAAGCA---ATAGGA---ATAGGAGCT------
---ATGTTCCTTGGGTTCTTGGGAGCA---GCAGGAAGCACTATG---GGCGCAGCGTCA---
ATAACGCTGACGGTACAG---GCCAGACAA---TTATTG---TCTGGTATAGTGCAACAGCAGAAC---
AATTTG---CTGAGG---GCTATTGAGGCGCAACAGCATATGTTGCAACTCACAGTCTGGGGCATT
>U36880
TTCTACTGTAATTCAACACCATTGTTTAGTGGTACTTGGAATACTAGTACTTGGAATGATACTACAGGGTCGA
ATGAC---
-------------------ACT---
GTAGGAAATAACACCATCATACTCCAATGTAGAATAAAACAAATTATAAACCTCTGCCAGAAAGTAGGAAAAG
CAATGTATGCCCCTCCCATTAGAGGACAAATTAGATGTTCATCAAAG---
ATTACAGGGCTGCTATTAACAAGAGATGGTGGGACTGACAATAAG---------------------------
----

AATATCGAGATCTTCAGACCTGGAGGAGGAGATATGAGGGACAATTGGAGAAGTGAATTATATAAATATAAAG
TAGTAAAAATTGAACCATTA---GGACTAGCACCCACTAAGGCAAAGAGAAGAGTGGTG-------------
------------------------CAGAGAGAAAAAAGAGCA---GTGGGA---CTAGGAGCT---------
ATGTTCCTTGGGTTCTTGGGAGCA---GCAGGAAGCACTATG---GGCGCAGCGTCA---
TTAACGCTGACGGTACAG---GCCAGACAA---TTATTG---TCTGGTATAGTGCAACAGCAGAGC---
GATTTG---CTGAGG---GCTATTGAGGCGCAACAGCATATGTTGCAACTCACAGTCTGGGGCATC
>U23487
TTCTACTGTAATTCAACACAATTGTTTAATAGTACTTGGAATACTGGGAATGATACTAGAGAGTCAAATGACA
CA--

AATAATACTGGAAATATCACACTCCCATGCAGAATAAAACAAATATTAAATTTGTGGCAGGAAGTAGGAAAAG
CAATGTATGCCCCTCCCATCAGCGGACAAATTAGCTGTTCATCAAAT---
ATTACAGGGCTATTATTAACAAGAGATGGTGGTAATACTAATACTACT------------------------
----

GGGAACACCACCGAGACCTTCAGACCTGGAGGAGGAAATATGAGGGACAATTGGAGAAGTGAATTATATAAAT
ATAAAGTAGTAAAAGTTGAACCATTA---GGAATAGCACCCACCAAGGCAAAGAGAAGAGTGGTG--------
-------------------------------CAGAGAGAAAAAAGAGCA---GTGGGAATGCTAGGAGCT---
------ATGTTCCTTGGGTTCTTGGGAGCA---GCAGGAAGCACTATG---GGCGCACGGTCA---
ATAACGCTGACGGTACAG---GCCAGACAA---TTATTG---TCTGGTATAGTGCAACAGCAGAAC---
AATCTG---CTGAGG---GCTATTGAGGCGCAACAGCATCTGTTGCAACTCACAGTCTGGGGCATC
>Y13719
TTCTACTGTAATTCAACACAACTGTTTAATAGTACTTGGAATGGTACTGGAGGGTCAAATAACACTGGA----
----

GGAAATGACACAATCACACTCCCATGCAGAATAAAACAAATTATAAACATGTGGCAGGAAGTAGGAAAAGCAA
TGTATGCCCCTCCCATCAGAGGAATTATTAGCTGTTCATCAAAT---
ATTACAGGGCTGATATTAACAAGAGATGGTGGTAACAACAGCACA----------------------------
----

AACGATACCGAGACCTTCAGACCCGGAGGAGGTGATATGAGGGATAATTGGAGAAGTGAATTATATAAATATA
AAGTAGTACAAATTGAACCATTA---GGAATAGCACCCACCAAGGCAAAGAGAAGAGTGGTG-----------
----------------------------CAGAGAGAAAAAAGAGCG---GTGACA---CTAGGAGCT------
---ATGTTCCTTGGGTTCTTGGGAGCA---GCAGGAAGCACTATG---GGCGCAGCGTCA---
ATAACGCTGACGGTACAG---GCCAGACAA---TTATTG---ACTGGTATAGTGCAACAGCAAAGC---
AATTTG---CTGAGG---GCTATTGAGGCGCAACAGCATCTGTTGCAACTCACAGTCTGGGGCATC
>AJ286330
TTCTACTGTAATACAACACAACTGTTTAATAGTACTTGGGTGCGTGATGATGCTAATAGTTCTTGGAGGCTAA
ATAGCTCT---
-------------------GAA---
GGAAATGACACACTTATCATACTCCAGTGCAGAATAAAACAAATTATAAACATGTGGCAGGAAGTAGGAAGAG
CAATGTATGCCCCTCCCATCAGAGGAAACATTAGCTGTTTATCAAAT---
ATTACAGGGCTGATATTAACAAGAGATGGTGGGATCCAGAACGAGTCCCAGTCC------------CAG----
----
-
AACGGGTCCGAGAGCGAGGAGACCTTCAGACCTGTAGGAGGAGATATGAGGGACAATTGGAGAAGTGAATTAT
ACAAATATAAAGTAGTAAAAATTGAACCATTA---GGAGTAGCACCCACCAGGGCAAAGAGAAGAGTGGTG--
-------------------------------------GCAAGAGAAAAAAGAGCG---GTGGCC---
CTAGGAGCT---------GTATTCATTGGGTTCTTGGGAGCA---GCAGGAAGCACTATG---
GGCGCAGCGTCA---ATGACGCTGACGGTACAG---GCCAGACAA---TTATTG---
TCTGGTATAGTGCAACAGCAGAAC---AATTTG---CTGAGG---
GCTATTGAGGCGCAACAACATCTGTTGCAACTCACAGTCTGGGGCATT
>AJ286332
TTCTACTGTAATACAACAAAACTGTTTAACAGTACTTGGCTTAATAATGGTACCTGGATTAATGGTACTTGGA
ATAAT--
-------------------ATT---
GATGGAAATGACACAATCACACTCCCATGCAGAATAAAACAAATTGTACACATGTGGCAGGAAGTAGGAAAAG
CAATGTATGCCCCTCCCATCAGTGGACAAATTAGATGTTCATCAAAT---
ATTACAGGGCTATTATTAACAAGAGATGGTGGTACTGACAACAGTACT-------------------------
----

GACAATAACACTGAGACCTTCAGACCTGGAGGAGGAAATATGAAGGACAATTGGAGAAGTGAATTATATAAAT
ATAAAGTAGTAAAAAATTGAACCATTA---GGAATAGCACCCACGAAGGCAAAGAGAAGAGTGGTG-------
-------------------------------CAGAGAGAAAAAAGAGCA---ATACCA---CTAGGAGCT---
------TTGTTCCTTGGGTTCTTGGGAGCA---GCAGGAAGCACTATG---GGCGCAGCGTCA---
ATAGCGCTGACGGCACAG---GCCAGACTA---TTATTG---ACTGGTATAGTGCAACAGCAAAAC---
AATTTG---CTGAGG---GCTATTGAGGCGCAACAGCATCTGTTGCAACTCACAGTCTGGGGCATT
>AJ286334
TTCTACTGTAATACATCACAACTGTTTAATAGTACTTGGTATGGGAATGAGACTGAAGGGTCAAATAAC----
----

CCTGATAGAAATATCACACTCCCATGCAGAATAAGACAAATTGTAAACAGATGGCAGGAAGTAGGAAAAGCAA
TGTATGCCCCTCCCATCAAAGGACTAATTAGATGTACATCAAAT---
ATTACAGGGCTGTTATTAACAAGGGATGGTGGTAATAATAATGTCACA-------------------------

----

AACCACACAGAGATCTTCAGACCTATAGGAGGAAATATGAGGGATAATTGGAGAAGTGAATTATATAAATATA
AAGTAGTAAAAATTGAACCATTA---GGAATAGCACCCACCAGGGCAAAGAGGAGAGTGGTG----------
---------------------------CAGAGAGAAAAAAGAGCA---GTGGGA---ATAGGAGCT------
---GTGTTCCTTGGGTTCTTGGGAGCA---GCAGGAAGCACTATG---GGCGCAGCGTCA---
ATAACGCTGACGGTACAG---GCCAGACAA---TTATTG---TCTGGTATAGTGCAGCAGCAGAGC---
AACTTG---CTGAGG---GCTATTGATGCGCAACAGCATCTGTTGCAACTCACAGTCTGGGGCATC
>AJ286336
TTCTACTGTAATACAACAAAGCTGTTTAATAGTACTTGGACTTGGAATGATACTAAAGGGTCAAATAACACTG
GG--

TCAAATGACACTATCATACTCCCATGCAGAATAAAACAAATTATAAACAGGTGGCAAGGAGTAGGAAAAGCAA
TGTATGCCCCTCCCATCAAAGGACCAATTAGATGTTCATCAAAC---
ATTACAGGGCTACTATTAACAAGAGATGGTGGTAATGATAATAGTAGT------------------------
----

GACAATTCCTCCGAGACCTTCAGACCTGGAGGAGGAGATATGAAGGACAATTGGAGAAGTGAATTATATAAAT
ATAAAGTAGTGAGAATTGAACCATTA---GGAGTAGCACCTACCAAGGCAAAGAGAAGAGTGGTG--------
------------------------------CAGAGAGAAAAAAGAGCA---GTGGGAATGCTAGGAGCT---
------CTGTTCCTTGGGTTCTTGGGAGCA---GCAGGAAGCACTATG---GGCGCAGCGGCA---
ATGACGCTGACGGTACAA---ACCAGACAA---TTATTG---TCTGGTATAGTGCAACAGCAAAAC---
AATCTG---CTGAGG---GCTATTGAGGCGCAACAGCATCTGTTGCAACTCACAGTCTGGGGCATC
>AJ286337
TTCTACTGTAATACAACACCACTGTTTAATAGTACTTGGAATAGTAATGGTACTAGGGTGTCAAATGACACTG
AA--

GGAAATAATACCACTATCACACTCAAATGCAGAATAAAACAATTTATAAACATGTGGCAGGAAGTAGGAAAAG
CAATGTATGCCCCTCCCATCAGAGGACAAATTAGATGTTCATCAAAT---
ATCACAGGGCTGCTATTAACAAGAGATGGTGGCCATGGTATTAATGGG------------------------
----

ACTAACGACACTGAGACCTTCAGACCTGGAGGAGGAGATATGAGGGACAATTGGAGAAGTGAATTATATAAAT
ATAAAGTAGTAAAAATTGAGCCATTA---GGAGTAGCACCCACCAGGGCAAAGAGAAGAGTGGTG--------
------------------------------CAGAGAGAAAAAAGAGCA---GCGGGA---ATAGGAGCT---
------GTGTTCCTTGGGTTCTTGGGAGCA---GCTGGAAGCACTATG---GGCGCAGCATCA---
ATAACGCTGACGGTACAG---GCCAGACAA---TTATTG---TCTGGTATAGTGCAACAGCAGAAC---
AATCTG---CTGAGG---GCTATTGACGCGCAGCAGCATATGTTGCAACTCACAGTCTGGGGCATT
>AF491740
TTCTACTGTGATTCATCACAACTGTTTAATAGTACTTGGATGTTTAATAGTACTTGGAATGATACTGACGGGG
CAAATAGC--
-------------------ACT---
AGGAAAAATGATACAATCACACTCCAGTGCAGAATAAAACAAATTATAAACATGTGGCAGCAAGTAGGAAAAG
CAATGTATGCCCCTCCCATCAGAGGACAGATTAGATGTACATCAAAC---
ATTACAGGGCTGATATTAACAAGAGATGGTGGTGATACCCAAAACAAG------------------------
----

AGCGACCCCGAGATCTTTAGACCTGGAGGAGGAGATATGAGGGACAATTGGAGAAGTGAATTATATAAATATA
AAGTAGTAAAAATTGAACCACTA---GGAATAGCACCCACCAGGGCAAAGAGAAGAGTGGTG----------
---------------------------CAGAGAGAAAAAAGAGCA---GTGGGG---ATGGGAGCT------
---ATGTTCCTTGGGTTCTTGGGAGCA---GCAGGAAGCACTATG---GGCGCAGCGTCA---
GTGACGCTGACGGTACAG---GCCAGACAA---TTATTG---TCTGGTATAGTGCAACAGCAGAGC---
AATTTG---CTGAGG---GCTATTGAGGCGCAACAGCATCTGTTGCAACGCACAGTCTGGGGCATC
>HQ122397
TTCTACTGTAATTTAACAAAACTGTTTAATAGTACTTGGAATGTTAATAGTACTTGGAATGGTACTGAAAGGT
CAAATAGC--
-------------------AAT---
GCAGAAAATGACACAATCACACTCCCATGCAGAATAAAACAAATTATAAACATGTGGCAGGAAGTAGGAAAAG
CAATGTATGCCCCTCCCATTAGAGGACAAATTAGCTGTTCATCAAAT---
ATTACAGGGCTGATATTAACAAGAGATGGTGGTAATAATGGAAGC---------------------------

----

GAGACCACCGAAATCTTCAGACCTGGAGGAGGAGACATGAGGGACAATTGGAGAAGTGAATTATATAAATATA
AAGTAGTAAAAATTGAACCATTA---GGAGTAGCACCCACCAGGGCAAAGAGAAGAGTGGTG-----------
----------------------------CAGAGAGAAAAAAGAGCA---GTGGGA---ATAGGAGCT------
---ATGTTCCTTGGGTTCTTGGGAGCA---GCAGGAAGCACTATG---GGCGCAGCGTCA---
CTGACGCTGACGGTACAG---GCCAGACAA---TTATTG---TCTGGTATAGTGCAACAGCAGAGC---
AATCTG---CTGAGG---GCTATTGAGGCGCAACAGCATCTGTTGCAACTCACAGTCTGGGGCATC
>JN002029
TTCTACTGTAATACAACACAACTGTTTAATAGTACTTGGAATGATACTGAAGAGTCAAAGGGCATTGAA----
----

GGAAATGACACAATCACACTCCCATGCAGAATAAAACAAATTGTAAACATGTGGCAGGAAGTAGGAAAAGCAA
TGTATGCCCCTCCCATCAGAGGACAAATTAGATGTTCATCAAGT---
ATTACAGGGTTAATATTAACAAGGGATGGTGGTGATACCGAGAAC----------------------------
----

GGGACAGAGATCTTCAGACCTGGAGGAGGAGATATGAGGGACAATTGGAGAAGTGAATTATATAAATATAAAG
TAGTAAAAATTGAACCATTA---GGAATAGCACCCACCAAGGCAAAGAGAAGAGTGGTG--------------
-------------------------CAGAGAGAAAAAAGAGCA---GTGGGA---ATAGGAGCT---------
GTGTTCCTTGGGTTCTTGGGAGCA---GCAGGAAGCACTATG---GGCGCAGCGTCA---
GTGACGCTGACGGTACAG---GCCAGACTA---TTATTG---CCTGGTATAGTGCAGCAACAGAAC---
AATTTG---CTGAGG---GCTATTGAGGCGCAACAGCATCTGTTGCAACTCACAGTCTGGGGCATC
>U36882
TTCTACTGTAATACAACACAGCTGTTTAATAGTACTTGGCTGTCTAATGGTGCTAGGAAGGAAGAGTCAAATA
AC---

AATGAAACAACCATCATACTCCCATGCAGAATAAAACAAATTATAAACAGGTGGCAGGAAGTAGGAAAAGCAA
TGTATGCCCCTCCCATCAGCGGATATATTAACTGTTCATCAAAT---
ATTACAGGGCTAATATTAACAAGAGATGGTGGTACTACTAACAATACC-------------------------
----

AATGCCACCGAGATTTTCAGACCTGGAGGAGGAAATATGAAGGACAATTGGAGAAGTGAATTATATAGATATA
AAGTAGTAAAAATTGAGCCATTA---GGAGTAGCACCAACCAAAGCAAAGAGAAGAGTGGTG-----------
----------------------------CAGAGAGAGAAAAGAGCA---GTGGGAACGCTAGGAGCT------
---ATGTTCCTTGGTTTCTTGGGAGCA---GCAGGAAGCACTATG---GGCGCAGCGTCA---
CTGACGCTGACGGTACAG---GCCAGACAA---TTATTG---TCTGGTATAGTGCAACAACAGAAC---
AATCTG---CTGAGA---GCTATTGAGGCGCAACAGCATCTGTTGCAACTCACAGTCTGGGGCATC
>DQ207940
TTCTACTGTGATACAAAACAATTGTTTAATAGTACTTGGAATTCTAATGATACTTGGAATTCTAATGGTACGA
GG---

AATGATTCAGAAATTATCACACTCCCATGTAGAATAAGACAAATTGTAAACATGTGGCAAGAAGTAGGAAAAG
CAATGTATGCCCCTCCAATCAAAGGACAAATTAAATGTGTATCAAAT---
ATTACAGGAATAATACTAACAAGAGATGGTGGTGATAATGAGACT----------------------------
----

AGAGGCAATGAGACTTTCAGACCTGGAGGAGGAGATATGAGGAACAATTGGAGAAGTGAATTATATAAATATA
AAGTAGTAAGAATTGAACCATTA---GGAATAGCACCCACCCAGGCAAGGAGAAGAGTGGTG-----------
----------------------------CAAAGAGAAAAAAGAGCA---GTGGGAACGCTAGGAGCT------
---ATGTTCCTTGGGTTCTTAGGAGCA---GCAGGAAGCACTATG---GGCGCAGCGTCA---
ATGACGCTGACGGTACAG---GCCAGACTA---TTATTG---TCTGGTATAGTGCAGCAGCAGAAC---
AATCTA---CTGAGG---GCTATTGAGGCGCAACAGCATCTGTTGCAACTCACAGTCTGGGGCATC
>HQ385456
TTCTACTGCAATTCATCACAATTGTTTAATAGTACTTGGAATGCTACTGATACTGGGAATACTAGTATTAGGA
AT---

GAGGCTGAGAATATCACACTCCCTTGCAGAATAAAACAAATTATAAACATGTGGCAGGAAGTAGGAAAAGCAA
TGTATGCCCCTCCCATCAGAGGACAAATTAGATGTGTATCAAAT---
ATCACAGGACTGCTATTAACAAGAGATGGTGGTGACAAGAACACT----------------------------

```
--------------------------------------------------------------------------
-------------
ACCACCGAGACCTTCAGACCTGGAGGAGGAAATATGAAGGATAATTGGAGAAGTGAATTATATAAATATAAAG
TAGTAAGAATTGAACCATTA---GGAGTAGCACCCACCAAGGCAAAGAGAAGAGTGGTG---------------
-------------------------CAGAGAGAAAAAAGAGCA---GTGGGA---ATAGGAGCT----------
ATGTTCCTTGGGTTCTTGGGAGCA---GCAGGAAGCACTATG---GGCGCAGCGTCA---
GTGACGCTGACGGTACAG---GCCAGACAA---TTATTG---TCTGGTATAGTGCAACAGCAAAAC---
AATTTG---CTGAGG---GCTATTGAAGCGCAACAGCATATGTTGCAGCTCACAGTCTGGGGCATC
>EU839603
TTCTACTGTAATACAACAAAACTGTTTAATAGTACCTGGAACAGTACTATGAATGGAACTACAGAAGGAGAT-
--------------------------------------------------------------------------
----------------------------
GACACGGAAGTTATCATACTCCCATGCAGAATAAAACAAATTATAAACATGTGGCAGAAAGTAGGCAAAGCAA
TGTATGCCCCTCCCATTGCAGGAGAAATTAGATGTTCATCAAAT---
ATTACAGGGCTGCTATTAACAAGAGATGGTGGTAATAATGCCACC-----------------------------
--------------------------------------------------------------------------
----------------
ACCGAGACCTTCAGACCTGGAGGAGGAAATATGAAGGACAATTGGAGAAGTGAATTATATAAATATAAAGTAG
TAAAAATTAAACCATTA---GGAGTAGCACCCACCAAGGCAAAGAGAAGAGTGGTG------------------
----------------------CAGAGAGAAAAAAGAGCA---GTGGGAACACTAGGAGCT---------
GTGTTCCTTGGGTTCTTGGGAGCA---GCAGGAAGCACTATG---GGCGCAGCGTCA---
ATGACGCTGACGGTACAG---GCCAGACAA---TTATTG---TCTGGTATAGTGCAACAGCAGAAC---
AATTTG---CTGAGG---GCTATTGAGGCGCAACAGCATTTGTTGCAACTCACAGTCTGGGGCATC
>EU839601
TTCTACTGTAATACAACAAGGCTGTTTAATAGTACTTGGAATGATGCTACACTAGACAGTGCTGGGAATAGTA
CTGAA---------------------------------------------------------------------
-------------------------
GGGCCAGATAAAAATATCACACTCCCATGCAGAATAAAACAAATTATAAACATGTGGCAGGGAGTAGGCAAAG
CAATGTATGCCCCTCCTATCAGCGGACAAATTGCATGTTCATCAAAA---
ATTACAGGGATGATATTAACAAGAGATGGTGGGCCTGAGAATGAA-----------------------------
--------------------------------------------------------------------------
----------------
ACTGAGATCTTCAGACCTGGAGGAGGAAATATGATGGACAATTGGAGAAGTGAATTATATAAATATAAAGTAG
TAAAAATTGAACCATTA---GGAGTAGCACCCACCAAGGCAAAGAGAAGAGTGGTG------------------
----------------------CAGAGAGAAAAAAGAGCA---GTGGGAACACTAGGAGCT---------
GTGTTCCTTGGGTTCTTGGGAGCA---GCAGGAAGCACTATG---GGCGCAGCGTCA---
ATGACGCTGACGGTACAG---GCCAGACAA---TTATTG---TCTGGTATAGTGCAACAGCAGAAT---
AATCTG---CTGAGG---GCTGTTGAGGCGCAACAGCATCTGTTGCAACTCACAGTCTGGGGCATC
>MH078551
TTCTACTGTAACACAACAAAGCTGTTTAATAGTACTTGGACTGGAAATGAGACAGAGAATAGTCTAAGG----
--------------------------------------------------------------------------
----------------------------
TCAAATGGCACAATCACACTCCCATGCAGGATAAAACAAATTGTAAACATGTGGCAGGAAGTAGGCAAAGCAA
TGTATGCCCCTCCCAGAAGAGGAATGATCAACTGTACATCAAAC---
ATTACAGGGCTACTATTGACAAGAGATGGTGGTACTAACAGTACT-----------------------------
--------------------------------------------------------------------------
----------------
AATGAGACCTTCAGACCTGGAGGAGGAGATATGAGGGACAATTGGAGAAGTGAACTATATAAATATAAAGTAG
TAAAAATTGAACCATTA---GGAGTAGCACCCACCAGGGCGAAAAGAAGAGTGGTG------------------
----------------------CAGAGAGAGAAAAGAGCA---GTGGGA---CTAGGAGCT---------
CTGTTCCTTGGGTTCTTGGGAACA---GCAGGAAGCACTATG---GGCGCAGCGTCA---
ATAACGCTGACGGTACAG---GCCAGACAA---TTATTG---TCTGGTATAGTGCAACAGCAGAGC---
AATTTG---CTGAGG---GCTATTGAGGCGCAGCAGCATATGTTGCAACTCACGGTCTGGGGCATC
>MK303352
TTCTACTGTAATACAACAAAACTGTTTAACAATGATTCAACACAACTGCCTAATGAG----------------
--------------------------------------------------------------------------
----------------------------
ACTAATGAGAATATCATACTTCCATGCAGAGTAAAACAAATTATAAACATGTGGCAGAGAGTAGGAAAAGCAA
TGTACGCCCCTCCCATCAGAGGACAAATTAGATGTTCATCAAAT---
ATTACAGGGCTGCTATTAACAAGAGATGGGGGTAATGAGAACAAC-----------------------------
```


AACAGTAACCCTGAGATCTTCAGACCTGCAGGAGGAGATATGAGGGATAATTGGAGAAGTGAATTATATAAAT
ATAAAGTAGTGCAAATTGAACCATTA---GGAGTAGCACCCACCAAAGCAAAGAGAAGAGTGGTG--------
-------------------------------CAGAGAGAAAAAAGAGCG---GTGGGA---CTAGGAGCT---
------TTGTTCCTTGGGTTCTTGGGAGCA---GCAGGAAGCACTATG---GGCGCAGCATCA---
ATAACGCTGACGGTACAG---GCCAGGCAA---TTATTG---TCTGGGATAGTGCAACAGCAGAAT---
AATCTG---CTGAGG---GCTATTGAGGCGCAGCAGCATCTGTTGCAACTCACAGTCTGGGGCATC
>MK303348
TTCTACTGTAATTCATCACAATTGTTTAATAGTACTTGGAATAGTACTAGGAATAATGATACTAGTACTAGGA
ATAATGATACA--

GAGTCAGGAGTTATCACACTCCAATGCAGAATAAAACAAATTATAAACATGTGGCAGAGAGTAGGAAAAGCAA
TGTATGCCCCTCCCATCAAAGGAAAGATTAGCTGCTCATCAAAT---
ATTACAGGGCTGCTATTAACAAGAGATGGTGGTAATGAGACCAAA----------------------------
----

AACAACACTGAGATCTTCAGACCTGCAGGAGGAAATATGAAGGATAATTGGAGAAGTGAATTATATAAATATA
AAGTAGTAAAAATTGAACCATTA---GGAATAGCACCCACCAAGGCAAAGAGACGAGTGGTG-----------
----------------------------CAGAGAGAAAAAAGAGCA---GTGGGA---CTAGGAGCT------
---TTGTTCCTTGGGTTCTTGGGAGCA---GCAGGAAGCACTATG---GGCGCAGCGTCA---
ATAACGCTGACGGTACAG---GCCAGACAA---TTATTG---TCTGGTATAGTGCAACAGCAGAAC---
AATCTG---CTGAGG---GCTATTGAGGCGCAGCAGCATCTGTTGCAACTCACAGTCTGGGGCATC
>MK303350
TTCTACTGTGATACAACAAAACTGTTTAATAGTACTTGGAATGGTACTGCTTGGAATGGTACGGAGGAT----
----

GATAACGGTACTATCACACTCCCCTGTAGAATAAAACAAGTTATAAAAATGTGGCAGAGAGTAGGAAAAGCAA
TATATGCCCCTCCCATTGCAGGACAAATTAACTGTACATCAAAT---
ATTACAGGGCTGCTATTACTAAGAGATGGTGGAAGTGGTAGTAACAATAGCATT-------------------
----
-
GTTACCAACAACAACACTGAGATCTTCAGACCTGGAGGAGGAGATATGAGAGACAATTGGAGAAGTGAATTAT
ACAAATATAAAGTAGTAGAAATTAAACCATTA---GGAGTAGCACCCACCAAGGCAAAGAGAAGAGTGGTG--
-------------------------------------CAGAGAGAAAAAAGAGCA---GTGGGG---
ATAGGAGCA---------TTGTTCCTTGGGTTCTTGGGAGCA---GCAGGAAGCACTATG---
GGCGCAGCGTCA---ATAACGCTGACGGCACAG---GCCAGACAA---TTATTG---
TCTGGTATAGTGCAACAGCAGAAC---AATCTG---CTGCAG---
GCTATTGAGGCGCAACAGCATCTGTTGCAACTCACAGTCTGGGGCATC
>MK303351
TTCTACTGTAACACAACAGGACTGTTTAATAGTAGTTCAATACAACTGCTTAATGAAACTTGGAATAGTACTG
GGAAT--

GAAAATGAAAATATCACACTCCCATGCAGAATAAAACAAATTGTAAACATGTGGCAAAGAGTAGGAAAAGCAA
TGTATGCCCCTCCCATCAGTGGACAAATTAGATGTTCGTCAAAT---
ATTACAGGGCTGCTATTAACAAGAGATGGGGGTAACAACACCAAC----------------------------
----

AACAACAACACAGAGATCTTCAGACCTGTAGGAGGAAATATGAAGGATAATTGGAGAAGTGAATTATATAAAT
ATAAAGTAGTACAAATTGAACCATTA---GGAGTAGCACCCACCAAGGCAAAGAGAAGAGTGGTG--------
-------------------------------CAGAGAGAAAAAAGAGCA---GTGGGA---CTAGGAGCT---
------TTGTTCCTTGGGTTCTTGGGAGCA---GCAGGAAGCACTATG---GGCGCAGCGTCA---
ATGACGCTGACGGTACAG---GCCAGGCAA---TTATTG---TCTGGGATAGTGCAACAGCAGAAC---
AATCTG---CTGAGG---GCTATTGAGGCGCAGCAGCATCTGTTGCAACTCACAGTCTGGGGCATC
>MK303353
TTCTACTGTAATACATCACAGCTGTTTAATAGTATTTGGTTTAATGGTACCTGGAATGGTACTAGATCAAAT-
----
-AGGACTGGGGATGATACT------
ATATCAAATAACACTATCACTCTTCCATGTAGGATAAAACAAATTATAAATAGATGGCAGGAGGTAGGGAAAG
CAATGTATGCCCCTCCCATCAGGGGACTAATTCGATGTTCATCAGAC---

ATTACAGGGCTGCTATTAACAAGAGATGGAGGTGAAGGTACAGAG----------------------------
----

AAGGACAACACGACTGAGGTCTTCAGACCAGGAGGAGGAGATATGAGGGACAATTGGAGAAGTGAATTATATA
AATATAAAGTAGTAAAAGTTGAACCAATA---GGAATAGCACCCACCAAGGCACAGAGAAGAGTGGTG-----
----------------------------------CAGAGAGAAAAAAGAGCA---GTAACG---
CTGGGAGCT---------GTGTTCCTTGGGTTCTTGGGAGCA---GCAGGAAGCACTATG---
GGCGCAGCGTCG---ATAACGCTGACGGCACAG---GCCAGACAA---TTATTG---
TCTGGTATAGTGCAACAGCAAAAC---AATTTG---CTGAGA---
GCTATTGAGGCGCAACAGCACATGTTGCAACTCACAGTCTGGGGCATC
>MK303354
TTCTACTGTAATACATCAGCACTGTTTAATAGTACCTGGAATAATACTTTGACTAATACTACAGGGTCAAAT-
----

GACACTGACACTATCACACTCCCATGCAGAATAAAACAAATTATAAACATGTGGCAGGAAGTAGGAAAAGCAA
TGTATGCCCCTCCCATCAGCGGACACATTAACTGTGTATCAAAT---
ATTACAGGGCTGATATTAACAAGAGATGGTGGTAATTCTAGCAAC----------------------------
----

GAGACCTTCAGACCCGCAGGAGGAGATATGAGGGACAATTGGAGAAGTGAATTATATAAATATAAAGTAGTAA
AAATTAAACCATTA---GGAATAGCACCCACCGAGGCAAAGAGAAGAGTGGTG--------------------
-------------------CAGAGAGAAAAAAGAGCA---GTGGGA---ATGGGAGCT---------
TTGTTCCTTGGGTTCTTGGGAGCA---GCAGGAAGCACTATG---GGCGCAGCGTCA---
ATGACGCTGACGGTACAG---GCCAGACAG---CTATTG---TCTGGTATAGTGCAACAGCAAAAC---
AATTTG---CTGAGA---GCTATTGAGGCGCAACAACATATGTTGCAACTCACAGTCTGGGGCATC
>MK303355
TTCTACTGTAATACATCAAAACTGTTTAATAATAATATTACTGCTGAT-------------------------
----

GAGAATGGGACTATCATACTCCCATGCAGAATAAGACAAATTGTAAACATGTGGCAGAGAGTAGGAAAAGCAA
TGTATGCCCCTCCCATCAGTGGACTAATTAGATGCTCATCAAAT---
ATTACGGGGCTGCTATTAACAAGAGATGGTGGTAACAGGATCAACAGCACTGAT-------------------
----
AACAGCACTATTGGGTACAGCAATGACAAC---
AACACTGAGACCTTCAGACCTGGAGGAGGAAATATGAAAGATAATTGGAGAAGTGAATTATATAAATATAAAG
TAGTAAAAATTGAACCATTA---GGAGTAGCACCCACCAAGGCAAAGAGAAGAGTGGTG--------------
-------------------------CAGAGAGAAAAAAGAGCA---GTGGGA---CTAGGAGCT---------
TTGTTCCTTGGGTTCTTGGGAGCA---GCAGGAAGCACTATG---GGCGCAGCGTCA---
ATAACGCTGACGGTACAG---GCCAGACAA---TTATTG---TCTGGTATAGTGCAACAGCAGAAC---
AATCTG---CTGAGG---GCTATTGAGGCGCAGCAACATCTGTTGCAACTCACAGTCTGGGGCATC
>GU191372
TTCTACTGTAATACAACCCCACTGTTTAATAGTACATGGAATGACACTAAACAATTCACTGTAAGTAAT----
----

AGCACCGAAGTTATCACACTCCCATGCAGAATAAAACAAATTATAAACATGTGGCAGGAAGTAGGAAAAGCAA
TGTATGCCCCTCCTATCAGAGGACAACTTAGCTGCTCATCAAAG---
ATTACAGGGCTGATACTAACAAGGGATGGTGGTGGTACCGAGTCT----------------------------
----

AAGGAAAACGAGACATTCAGACCTGCAGGAGGAGATATGAGGGACAATTGGAGAAGTGAATTATATAAATATA
AAGTAGTAAAAGTTGAACCATTA---GGAATAGCACCCACCAAGGCAAAGCGAAGAGTGGTG-----------
----------------------------CAGAGAGAAAAAAGAGCA---GTAACA---ATAGGAGCT------
---ATGTTCCTTGGGTTCTTGGGAGCA---GCAGGAAGCACTATG---GGCGCAGCGTCA---
ATGACGCTGACGGTACAG---GCCAGGCAA---TTATTG---TCTGGTATAGTGCAACAGCAGAGC---
AATTTG---CTGAGA---GCTATTGAGGCGCAACAGCATATGTTGCAACTCACAGTCTGGGGCATC
>JF680936
TTCTACTGTAATACAACAAAACTGTTTAATAGTACTTGGAATGGTACTGAATCATCAAACACCACC-------
----

ACCACTGGAAGCATCACGCTCCCATGCAAAATAAGACAAATTGTAAACATGTGGCAGGAAGTAGGAAAAGCAA

TGTATGCCCCTCCTATCAGAGGAAAGATTAGTTGTTCATCAAAT---
ATTACAGGGATACTATTAACAAGAGATGGTGGTAATGATAATGGC----------------------------
----

AGTAACCCTGAAATCTTCAGACCTGGAGGAGGAGATATGAGGGACAATTGGAGAAGTGAATTATTTAAATATA
AAGTAGTAAAAATTGAACCATTA---GGAATAGCACCATCTAAGGCAAGAAGAAGAGTGGTG-----------
----------------------------CAGAGAGAAAAAAGAGCA---GTGGGA---TTTGGAGCC------
---TTGTTCCTTGGGTTCTTGGGAGCA---GCAGGAAGCACTATG---GGCGCAGCGTCA---
ATGGCGCTGACGGTACAG---GCCAGACAA---TTATTG---TCTGGTATAGTGCAACAGCAAAAC---
AATTTG---CTGAGA---GCTATTGAGGCGCAACAGCATATGTTGCAACTCACAGTCTGGGGCATT
>KF061032
TTCTACTGTGATTCAACACAACTGTTTAATAGTACTTGGAATAGTACTTGGAATAGTACTGGAGGGTCAGAA-
----

GAAAATGACACAATCACACTCCCATGCAGAATAAAACAAATTATAAACATGTGGCAGGAAGTAGGAAAAGCAA
TGTATGCCCCTCCCATCAGAGGACAAATTAGATGCTCATCAGAT---
ATTACAGGGCTGATATTAACAAGAGATGGTGGTAATAACGAGAAC----------------------------
----

GGGTCTGAGATCTTCAGACCTGGAGGAGGAGATATGAGGGACAATTGGAGAAGTGAATTATATAAATATAAAG
TAGTAAAAATTGAACCATTA---GGAATAGCACCCACCAAGGCAAAGAGAAGAGTGGTG--------------
-------------------------CAGAGAGAAAAAAGAGCA---GCA------CTAGGAGCT---------
ATGTTCCTTGGGTTCTTGGGAGCA---GCAGGAAGCACTATG---GGCGCAGCGTCA---
GTAACGCTGACGGTACAG---GCCAGACTA---TTATTG---TCTGGTATAGTGCAACAGCAGAAC---
AATTTG---CTGAGG---GCTATTGAGGCGCAACAGCACCTGTTGCAACTCACAGTCTGGGGCATC
>KF061031
TTCTACTGCAATTCAACACAACTGTTTAATAGTACATGGAATGTTACTGAAGGGTCAAATAACACT-------
----

GCAGGAGAAAATATCACACTCCCATGCAGAATAAAACAAATTATAAACATGTGGCAGGAAGTAGGAAAAGCAA
TGTATGCCCCTCCCATCAGAGGACAAATTAAATGCTCATCAAAT---
ATTACAGGGCTGATATTAACAAGAGATGGTGGTAATAGCAGTAAC----------------------------
----

ACGACCGAGATCTTCAGACCTATAGGAGGAGATATGAGAGACAATTGGAGAAGCGAATTATATAAATATAAAG
TAGTAGAACTTGAACCATTA---GGAGTAGCACCCACCAAGGCAAAGAGAAGAGTGGTG--------------
-------------------------CAGAGAGAAAAAAGAGCA---GTGGGA---CTAGGAGCT---------
TTGTTCCTTGGGTTCTTGGGAGCA---GCAGGAAGCACTATG---GGCGCAGCGTCA---
ATAACGCTGACGGTACAG---GCCAGACTA---TTATTG---TCTGGTATAGTGCAACAGCAAAAT---
AATTTG---CTGAGA---GCTATTGAGGCGCAACAGCATATGTTGCAACTCACAGTCTGGGGCATT
>AY835445
TTCTACTGTAATACAACAAAACTGTTTAATAGTACCTGGAATGGGAATAATACTACAGAATCAGATAGC----
----

ACAGGAGAAAATATCACACTCCCATGCAGAATAAAACAGATTATAAACCTGTGGCAAGAAGTAGGAAAAGCAA
TGTATGCCCCTCCTATCAAAGGACAAATTAGTTGTTCATCAAAT---
ATTACAGGGCTACTATTAACAAGAGATGGTGGAAATAACAATAGC----------------------------
----

AGCGGGCCCGAGACCTTTAGACCTGGAGGAGGAAATATGAAGGACAATTGGAGAAGTGAATTATATAAATATA
AAGTAATAAAAATTGAACCATTA---GGAGTAGCACCCACCAGGGCAAAGAGAAGAGTGGTG-----------
----------------------------CAAAGAGAAAAAAGAGCA---GTGGGAACACTAGGAGCT------
---ATGTTCCTTGGTTTCTTGGGAGCA---GCAGGAAGCACTATG---GGCGCAGCGTCA---
GTGACGCTGACGGTACAA---GCCAGACTA---CTGTTG---TCTGGTATAGTGCAGCAGCAGAAC---
AATTTG---CTGAGA---GCTATTGAGGCGCAACAGCATATGTTGCAACTCACAGTCTGGGGCATT
>AY835444
TTCTACTGTAATACAACAAAACTGTTTAATAGTACTTGGGATGCTAATGGTAATTGCACCGGTTGTGATGAAA
GT---

GATGGAAATAACACTATCACACTTCCATGCAGAATAAAACAAATTGTAAACATGTGGCAGGAAGTAGGCAAAG

CAATGTATGCCCCTCCCATCAAGGGACTAATTAAATGTACATCAAAC---
ATTACAGGGCTGCTATTAACAAGAGATGGTGGTGCTAATAATACA----------------------------
----

AACGAGACCTTCAGACCTGGAGGAGGAGATATGAGGGACAATTGGAGAAGTGAATTATATAAATATAAAGTAG
TACAAATTGAACCATTA---GGAATAGCACCCACCAGGGCAAGGAGAAGAGTGGTG-----------------
----------------------CAGAGAGAAAAAAGAGCA---GTGGGAACACTAGGAGCT---------
ATGTTCCTTGGGTTCTTGGGGGCA---GCAGGAAGCACTATG---GGCGCAGCGTCA---
GTGACGCTGACGGTACAG---GCCAGACAA---TTGTTG---TCTGGTATAGTGCAACAGCAGAAC---
AATCTG---CTGAAA---GCTATTGAGGCGCAACAGCATATGTTGCAACTCACGGTCTGGGGCATC
>EU839605
TTCTACTGTAATACAGCACAACTGTTTAATAGTACTTTTAATGGTACTCTTAGTCCAGGGGAG----------
----

AATACTACCACTATCACACTCCCATGCAGAATAAAACAAATTATAAACAGGTGGCAGGAAGTAGGCAAAGCAA
TGTATGCCCCTCCCATCAAAGGACTAATTAGATGTTTATCAAAT---
ATTACAGGGCTGCTATTAACAAGAGATGGTGGTAATAATAGTGAT----------------------------
----

GCTAATGAGACCTTCAGACCACAAGGAGGAAATATGAAAGACAATTGGAGAAGTGAATTGTATAAATATAAAG
TAGTAAGAATTGAACCATTA---GGAATAGCACCCACCAGAGCAAGGAGAAGAGTGGTG--------------
-------------------------CAGAGAGAAAAAAGAGCA---GTGGGAGTAATAGGAGCT---------
ATGTTCCTTGGGTTCTTGGGAGCA---GCAGGAAGCACTATG---GGCGCAGCGTCG---
CTGACGCTGACGGTACAG---GCCAGACAA---TTATTG---TCTGGTATAGTGCAACAGCAGAAC---
AATCTG---CTGAAG---GCTATTGAGGCGCAACAACATCTGTTGCAACTCACGGTCTGGGGCATC
>JF680925
TTCTACTGTAACACAACAGAGCTGTTTAATAGTACTTGGAATGCTAATGGTTCCTGGACGGGTACTGAAAAGA
ATGATACT---
-------------AATACT------
AACAAAACAGGCATAATCACACTCCCATGCAAAATAAAGCAGATTATAAACATGTGGCAGGAAGTAGGAAAAG
CAATGTATGCCCCTCCCATCCGAGGACCGATTTCCTGTACATCAAAT---
ATTACAGGACTACTGTTAACAAGAGATGGTGGTAGTAATGAGAGC----------------------------
----

ACAGAGATCTTCAGACCTGGAGGAGGAGATATGAGGGACAATTGGAGAAGTGAATTATATAAGTATAAAGTAG
TAAAAGTTGAACCATTA---GGAGTAGCACCTTCCAAGGCAAGGAGGAGAGTGGTG-----------------
----------------------CAGAGAGAAAAAAGAGCA---GTGGGA---ATAGGAGCT---------
GTGTTCCTTGGGTTCTTGGGGGCA---GCAGGAAGCACTATG---GGCGCAGCGTCA---
ATGACGCTGACGGTACAA---GCCAGACTA---CTACTG---TCTGGTATAGTGCAACAGCAGAAC---
AATCTG---CTGAGA---GCTATTGAGGCGCAACAACATTTGTTGCAACTCACAGTCTGGGGCATC
>HM030564
TTCTACTGTGATACAAAAGGGCTATTCAATAGCACATTCAATAGCACGTGGAATGCTACAGAAGATACTGGAC
TGCCA--
-------------------AAT---
AGCACTAGCACTAATATTACACTCCCATGCAGAATAAAACAAATTATAAACACGTGGCAGGGAGTAGGCAAGG
CAATGTATGCCCCTCCCATCCAAGGACTAATTAGATGTAGATCAAAT---
ATTACAGGGATACTATTGACAAGAGATGGTGGGAGTGGAAATAGTAGT-------------------------
----

GATAATACAGAGATCTTCAGACCGGGAGGAGGAGATATGAGGGACAATTGGAGAAGTGAATTATATAAATATA
AAGTAGTACAAATTGAACCATTA---GGAGTAGCACCCACCAAGGCAAAGAGAAGAGTGGTG-----------
----------------------------CAGAGAGAAAAAAGAGCA---GTGACA---CTGGGAGCT------
---CTGTTCCTTGGGTTCTTGGGAGCA---GCAGGAAGCACTATG---GGCGCAGCGTCA---
ATGACGCTGACGGTACAG---GCCAGACTA---CTATTG---TCTGGTATAGTGCAACAGCAGAAC---
AATTTG---CTGAGA---GCTATTGAGGCGCAACAGCATCTGTTGCAACTCACAGTCTGGGGCATT
>HM030559
TTCTACTGCAATACAACACAACTATTTAATAGTACTTGGAATATTGGGTTAAATAACACAAATGACACTGTAG
AA---

AATGATACTAATATCATACTACCGTGCAAAATAAAACAAATAGTGAACATGTGGCAGGAAGTAGGCAAAGCAA

TGTATGCCCCTCCCATCAATGGAGTAATTAAATGTTCATCAAAT---
ATTACAGGACTGTTATTAACCAGAGATGGTGGTAACACCACCGGGATTGAGAAC-------------------
----

AATATTAGCAATACTGAGGTCTTCAGACCTGGAGGAGGAAATATGAGGGACAATTGGAGAAGTGAATTATATA
AATATAAAGTAATAAAAATTGAACCATTA---GGAATAGCACCTACCAGGGCAAAAAGGAGAGTGGTG-----
----------------------------------CAGAGAGAAAAAAGAGCA---GTAGGG---
CTAGGAGCT---------ATGTTCCTTGGGTTCTTGGGAGCA---GCAGGAAGCACTATG---
GGCGCAGCGTCA---ATAACGCTGACGGTACAG---GCCAGACAA---TTGCTG---
TCTGGTATAGTGCAACAGCAGAAC---AATCTG---CTGAGA---
GCTATTGAGGCGCAACAACATATGTTGCAACTCACGGTCTGGGGCATC
>HM030562
TTCTATTGTGACTCAACACAACTGTTTAATAGCACTTGGACTAAGAATGATAGTACTTGGACTTTGAATGACA
AAGAG--
-------------------TCA---
AATAGAACTGAGAATATCACACTCCCATGCAGAATAAAACAAATTGTAAACCTGTGGCAGGAGGTAGGAAAAG
CAATGTATGCCCCTCCCATCAAAGGACCCATTAAATGCGAATCAAAT---
ATTACAGGGATGCTCTTAACAAGAGATGGTGGTAATAACGAGAGC----------------------------
----

ACAAATGAAACCTTCAGACCTGGAGGAGGAGATATGAGGGACAATTGGAGAAGTGAATTATATAAATATAAAG
TAGTACAGATTGAGCCATTA---GGATTAGCACCCACCAAGGCAAAGAGAAGAGTGGTG--------------
-------------------------CAGAGAGAAAAAAGAGCA---GTGGGA---ATAGGAGCT---------
TTGTTCCTTGGGTTCCTGGGAGCA---GCAGGAAGCACTATG---GGCGCAGCGTCA---
CTAACGCTGACGGTACAG---GCCAGATCA---TTATTG---TCTGGTATAGTGCAGCAGCAGAAC---
AATCTG---CTGAGG---GCTATTGAGGCGCAACAGCATCTGTTGAGACTCACAGTCTGGGGCATC
>HM030565
TTCTATTGTAACACAACACCGCTGTTTAATAGTATACACAAGGAAAATGACACACTCACTAACGAGACTGAA-
----

GGAAATGACACACTCACACTCCAATGCAGAATAAAACAAATCATAAACATGTGGCAGAGAGTAGGAAAAGCAA
TGTATGCCCCTCCCATCAGCGGATGGATTAGATGTTCATCAAAT---
ATTACAGGACTGCTATTAACAAGAGATGGGGGTAATAATAATAGCACT-------------------------
----

AATGAGACCGAGACCTTCAGACCGGGAGGAGGAGATATGAGGGACAATTGGAGAAGTGAATTATATAAATATA
AAGTAGTAAAAATTGAACCATTA---GGAGTGGCACCCACCAAGGCAAAGAGAAGAGTGGTG-----------
----------------------------CAGAGAGAAAAAAGAGCA---GTGGGA---GTAGGAGCT------
---ATGGTCCTTGGGTTCTTGGGAGCA---GCGGGAAGCACTATG---GGCGCAGCGTCA---
TTGACGCTGACGGTACAG---GCCAGGCTA---TTACTG---TCTGGTATAGTGCAACAGCAAAAC---
AATTTG---CTGAGG---GCTATTGAGGCGCAACAACATCTGTTGCAACTCACAGTCTGGGGCATC
>AB428551
TTCTACTGTAATACATCATCACTGTTTAATTGGAGTACTAATGGGTCAAATATTACTAATGGG----------
----

TCAAATATTACCAAAACCATCCCATGCAGAATAAAACAAATTATAAACATGTGGCAGAAAGTAGGAAAAGCAA
TGTATGCCCCTCCCATCAGAGGACAAATTAATTGTTTATCAAAT---
ATTACAGGGCTGCTATTAACAAGAGATGGTGGTAACCAGACTGAGTTT-------------------------
----

AATAATACTGAGATCTTCAGACCTGGAGGAGGAGATATGAGGGACAATTGGAGAAGTGAATTATATAAATATA
AAGTAGTAAAAATTGAACCATTA---GGAGTAGCACCCACCAAGGCAAGGAGAAGAGTGGTG-----------
----------------------------CAAAGAGAAAAAAGAGCA---GTGGGAACAATAGGAGCT------
---ATGTTCCTTGGGTTCTTGGGAGCA---GCAGGAAGCACTATG---GGCGCAGCGTCA---
TTGACGCTGACGGTACAG---GCCAGACTA---TTATTG---TCTGGTATAGTGCAACAGCAGAAC---
AATCTG---CTGAGG---GCTATTGAGGCGCAACAACATCTGTTGCAACTCACAGTCTGGGGCATC
>AB289588
TTCTACTGTAATTCAACACCACTGTTTAATAGTACTTGGAATTTTACTAATACTTGGAATATGACTGAAGGCA
TAACT--

AACACTGAAGGAAATATCACACTCAAATGCAGAATAAAACAAATTATAAACATGTGGCAGGAAGTAGGAAAAG
CAATGTATGCCCCTCCCATCGAGGGACTAATTAGATGTACATCAAAT---
ATTACAGGGCTGCTATTAACAAGAGATGGGGGAATAATAGGACA-----------------------------
----

AATGAGACTGAGACCTTCAGACCTGGAGGAGGAGACATGAGGAACAATTGGAGAAGTGAATTATATAAATATA
AAGTAGTAAAAATTGAACCATTA---GGAGTAGCACCCACCAGGGCAAGGAGAAGAGTGGTG-----------
-----------------------------CAGAGAGAGAAAAGAGCA---GTGGGAGTACTAGGAGCT------
---ATGTTCCTTGGGTTCTTGGGAGCA---GCAGGAAGCACTATG---GGCGCAGCGTCA---
TTAACGCTGACGGTACAG---GCCAGACAA---TTATTG---TCTGGTATAGTGCAACAGCAAAAC---
AATTTG---CTGAGA---GCTATTGAGGCGCAACAGCAGTTGTTGCAACTCACTGTCTGGGGCATC
>AB289590
TTTTACTGTAATACAACACAATTGTTTAATAGTACTTGGAATGAAACAAACCTGTTTAATAATACTGGGAATA
AT---

ACTGAGGGTAATACTATCACACTCCCATGCAGAATAAAACAGATTATAAACATGTGGCAGGAAGTAGGAAAAG
CAATGTATGCCCCTCCTATCAGAGGACAAATTAAATGTTCATCAAAC---
ATTACAGGGCTGCTATTAACAAGAGATGGTAGTACTAATGACACA-----------------------------
----

AACAATACTGAGATATTCAGACCTACAGGAGGGAATATGAGGGACAATTGGAGAAGTGAATTATATAAATATA
AAATAGTACAAATTGAACCATTA---GGAGTAGCACCTACCAAGGCAAAGAGAAGAGTGGTG-----------
-----------------------------CAGAGAGAAAAAAGAGCA---GTGGGA---CTAGGAGCT------
---ATGTTCCTTGGGTTCTTGGGAGCA---GCAGGAAGCACTATG---GGCGCAGCGTCA---
ATAACGCTGACGGTACAG---GCCAGACAA---TTATTG---TCTGGTATAGTGCAACAGCAAAAC---
AATTTG---CTGAGA---GCTATTGAGGCGCAACAGAATCTGTTGCAACTCACAGTCTGGGGCATT
>AB428558
TTCTACTGCAATACAACAGAACTGTTTAATAGTACTTGGATTAATGGTACTAGGACAACCAACACTACG----
----

TCAAATGGCACTATCACACTCCGATGTAGAATAAAACAAATTATAAACATGTGGCAGAGAGTAGGAAAAGCAA
TGTATAACCCTCCCATCGCAGGACAAATTAGATGTTCATCAAAT---
ATTACAGGGATATTATTAGTGAGAGATGGTGGTGATAACAGTACG----------------------------
----

ACCAACGAAACCTTCAGACCTGGAGGAGGGAATATAAGAGACAATTGGAGAAGTGAATTATATAAATATAAAG
TAGTAAAAATTGAACCATTA---GGAGTAGCACCCACCAAGGCAAAGAGAAGAGTGGTG--------------
--------------------------CAGAGAGAAAAAAGAGGA---GTGGGA---ATAGGAGCT---------
GTGTTCCTTGGGTTCTTGGGAGCA---GCAGGAAGCACTATG---GGCGCAGCGTCA---
ATAACGCTGACGGTACAG---GCCAGACAA---TTATTG---TCTGGTATAGTGCAACAGCAGAAC---
AATTTG---CTGAGG---GCCATTGAGGCGCAACAGCATATGTTGCAACTCACAGTCTGGGGCATC
>AB221126
TTCTACTGTAATACAACACAGCTGTTTAATAGCACTTGGACTTGGAATAGTACTACGGGCACTTGGAATAATA
CTGGGAGC---
--------------------AGG---
AGTGAGAATGACACTATCACACTCCCATGCAGAATAAAACAAATTATAAACAGGTGGCAGGAAGTAGGAAAAG
CAATGTATGCCCCTCCCATCCAAGGACAAATTAACTGTTCATCAAAT---
ATTACAGGACTGCTATTAACAAGAGATGGTGGTAACGAAACTGAG----------------------------
----

AACGGGTATGATACCTTCAGACCTGGAGGAGGAGATATGAGGGATAATTGGAGAAGTGAATTATATAAATATA
AAGTGGTACAAATTGAGCCATTA---GGAATAGCACCCACCAGGGCAAAGAGAAGAGTGGTG-----------
-----------------------------CAGAGAGAAAAAAGAGCA---GTGGGAACGCTAGGAGCT------
---GTGTTCCTTGGGTTCTTGGGAGCA---GCAGGAAGCACTATG---GGCGCAGCGTCA---
CTAACGCTGACGGTACAG---GCCAGACTA---TTACTG---TCTGGTATAGTGCAACAGCAGAAC---
AATCTG---CTGAAA---GCTATTGAGGCGCAACAGCATCTGTTGCAACTCACAGTCTGGGGCATC
>AB480697
TTCTACTGTAACACATCAGCACTGTTTAATAGTACTTGGAATAATAACACTTGGTATGTAAATGGCACTGAAG
GGTCA--
--------------------AAT---

GACACTGATAAAAATATCACCCTCCCATGCAGAATAAAACAATTTATAAACATGTGGCAGGCAGTAGGAAAAG
CAATATATGCCCCTCCTATTAAAGGACAAATTAGCTGTTCATCAAAT---
ATTACAGGGCTGCTATTAACAAGAGATGGTGGTGAAGGTAACGAAACT-------------------------
----

GACAACCAGACTGAAGTCTTCAGACCTGGAGGAGGGGATATGAGGGACAATTGGAGAAGTGAATTATACAAAT
ATAAAGTAGTAAAAATTGAACCATTA---GGAGTAGCACCCACCAAGGCAAAGAGAAGAGTGGTG--------
-------------------------------GAAAGAGAAAAAAGAGCA---ATAGGGGGAATAGGAGCT---
------TTGTTCCTTGGGTTCTTGGGAGCA---GCAGGAAGCACTATG---GGCGCAGCGTCA---
CTGACGCTGACGGTACAG---GCCAGACTA---TTATTG---ACTGGTATAGTGCAACAGCAGAAC---
AATTTG---CTGAGG---GCTATTGAGGCGCAACAGCATCTGTTGCAACTCACAGTCTGGGGCATC
>AB480695
TTCTATTGTAATACATCAGGACTGTTTAATAGTACTTGGGAGTTTAATAGTACTTGGAATGATAAT-------
----

AGTACTGAAAAAATCGTACTCCCATGCAGAATAAGACAAATTGTAAGCATGTGGCAGGGAGTAGGAAAAGCAA
TGTATGCCCCTCCCATTAAAGGACAAATTAGCTGTTCATCAAAT---
ATTACAGGGCTGCTATTAACAAGAGATGGTGGTGGTAATGGTACT----------------------------
----

GAGACCTTCAGACCTGAAGGAGGAAATATGAAGGACAATTGGAGAAGTGAATTATATAAATATAAAGTAGTAA
AACTTGAGCCTTTA---GGAATAGCACCTACTCCAGCAAGGAGAAGAGTGGTG--------------------
-------------------CAGAGAGAAAAAAGAGCA---GTGGGA---TTCGGAGTT---------
TTGTTCCTAGGGTTCTTGGGAGCA---GCCGGAAGCACTATG---GGCGCAGCGTCA---
ATGGCGCTGACGGTACAG---ACCAGACAA---TTGTTG---TCTGGTATAGTGCAACAGCAGAAC---
AATCTG---CTGAGG---GCTATTGAGGCGCAACAACATCTGTTGCAACTCACAGTCTGGGGCATT
>AB287363
TTCTACTGTAATTCAACACAGCTGTTTAATAGTACTTGGCATGGAAACATATCAAATGTGTCAAATAACATTG
GA---

AAAAATAGCAACATCACACTCCCGTGTAGAATAAGACAAATTGTAAACATGTGGCAGACAGTAGGAAAAGCAA
TGTATGCCCCTCCCATCAGCGGATACATTAAATGTACATCAAAT---
ATTACAGGACTGCTATTGACAAGAGATGGTGGAAAGGGAAATAAC----------------------------
----

ACGGACACGGAGGTCTTCAGACCTGGAGGAGGAGATATGAGGGACAATTGGAGAAGTGAATTATACAAATATA
AAGTAGTAAAAATTGAACCATTA---GGAATAGCACCCACCAAGGCAAGGAGAAGAGTGGTG-----------
----------------------------CAGAGAGAAAAAAGAGCA---GTAATA------GGAGCT------
---ATGTTCCTTGGGTTCTTGGGAGCA---GCAGGAAGCACTATG---GGCGCAGCGTCA---
GTGACGCTGACGGTACAG---GCCAGGCAA---TTATTG---TCTGGTATAGTGCAACAGCAGAAC---
AATCTG---CTGAAA---GCTATTGAGGCGCAACAGCATCTGTTGCAACTCACAGTCTGGGGCATC
>AB287364
TTCTACTGCAATACAACAAAATTGTTTAATAGTACATGGATCTGGAATAAAACACTAATGGTTAATGACACAA
GA---

GAGTCAAATGACATTATCAGGTTCCAATGCAGAATAAAACAGATTATAAACATGTGGCAGGAAGTAGGAAGAG
CAATGTATGCCCCTCCCATCGAAGGACTAATTAGATGCTCATCAAAT---
ATTACAGGGCTGATATTAACAAGAGATGGTGGTAATAGCAAAAAC----------------------------
----

GATACAGAAACCTTCAGACCTGCAGGAGGAAATATGAAGGACAATTGGAGAAGTGAATTATATAAATATAAAG
TAGTACAAGTTGAACCATTA---GGAATAGCACCCACCAGGGCAAAGAGAAGAGTGGTG--------------
-------------------------CAAAGAGAGAAAAGAGCC---GTGGGA---TTAGGAGCT---------
TTGTTCCTTGGGTTCTTGGGAGCA---GCAGGAAGCACTATG---GGCGCAGCGTCA---
CTAACGCTGACGGTACAG---GCCAGACAA---TTATTG---TCTGGTATAGTGCAACAGCAAAAC---
AACTTG---CTGAGG---GCTATTGAGGCGCAACAACATCTGTTGCAACTCACAGTCTGGGGCATC
>AB588283
TTCTACTGTAATACAACAAAGCTGTTTAATAGTACTTGGCATCCTAATGGGAGTATGATTGCAGAACCAAATA
AC---

ACTAACAACATTATTACACTCCCATGCAGAATAAAACAAATTATAAACAGGTGGCAGGAGGTAGGAAAAGCAA
TATATGCCCCTCCCATCCAAGGACAAATTAACTGTTCATCAAAT---
ATTACAGGACTGCTATTAACAAGAGATGGTGGTAAGGAGGATAAGGAG-------------------------
----

AATAGGTCACAGGAGACCTTCAGACCTGGAGGAGGAGATATGAGGGACAATTGGAGAAGTGAATTATATAAGT
ATAAAGTAGTACAAATTGAGCCATTA---GGAGTAGCACCCACCAGGGCAAAGAGAAGAGTGGTG--------
-------------------------------CAAAGAGAAAGAAGAGCA---GTGGGG---ATAGGAGCT---
------ATGTTCCTTGGGTTCTTGGGAGCA---GCAGGAAGCACTATG---GGCGCAGCGTCA---
CTAACGCTGACGGTACAG---GCCAGACTA---TTATTG---TCTGGTATAGTGCAACAGCAAAAT---
AATCTG---CTGAGA---GCTATTGAGGCGCAACAGCATATGTTGCAACTCACAGTCTGGGGCATT
>AB588296
TTCTACTGTAACACAACACAACTGTTTGATAGTACTTGGAATAGTACTTTAAATATTACTGAGAGGTCAAAT-
----

GACTCTGGTATAATCACCCTCCCATGCAGAATAAAACAATATATAAATTTGTGGCAGGAAGTAGGAAGAGCAA
TGTATGCCCCTCCCATCAGTGGACAAATTAAATGTTCATCAAAT---
ATTACAGGGCTGTTATTAACAAGAGATGGTGGTAAGGGGAACGAAAAC-------------------------
----

AATATCACTGAGACCTTCAGACCTGGAGGAGGAAACATGAGGGACAATTGGAGAAGTGAATTATACAAATATA
AAGTAGTAAAAATTGAACCATTA---GGAATAGCACCCACCAGGGCAAAGAGAAGAGTGGTG-----------
----------------------------CAGAGAGAAAAAAGAGCA---GTGGGA---CTGGGAGCT------
---ATGTTCCTTGGGTTCTTGGGAGCA---GCAGGAAGCACTATG---GGCGCAGCGTCA---
GTGACGCTGACGGTACAG---GCCAGACAG---TTATTG---TCTGGTATAGTGCAACAGCAGAAC---
AATTTG---CTGAGG---GCTATTGAGGCGCAACAACATCTGTTGCAACTCACAGTCTGGGGCATC
>AB287367
TTCTACTGTAATACAACAGGGCTATTTAATGATAGTATGCTGAATGATACTACT-------------------
----

GAAGGTAACAACCTCACACTCCCGTGCAGAATAAAACAAATTATAAACAGATGGCAGGAAGTAGGAAAAGCAA
TGTATGCCCCTCCCATCCAAGGACAAATTAGATGTTCATCAAAT---
ATTACAGGGTTGCTATTAACAAGAGATGGAGGAAATAGTGGCAATAAA-------------------------
----

ACTAATAATACTGAGACTTTCAGACCTGGAGGAGGAAATATGAAGGATAATTGGAGAAGTGAATTATATAAAT
ATAAAGTAGTAAAAATTGAACCATTA---GGAGTAGCACCCACCAAGGCAAGAAGAAGAGTGGTG--------
-------------------------------CAAAGAGAAAAAAGAGCA---GTGGGAACGCTGGGAGCT---
------ATGTTCCTTGGGTTCTTGGGAGCA---GCAGGAAGCACTATG---GGCGCAGCGTCA---
ATGGCGCTGACGGTACAG---GCCAGACAA---TTATTG---TCTGGTATAGTGCAACAGCAGAAC---
AATCTG---CTGAAA---GCTATTGAGGCGCAACAGCATATGTTGCAACTCACAGTCTGGGGCATC
>AB287368
TTCTACTGTAATACAACACCACTGTTTAATAGTAGCTGGTATGAAAATGGAAGTAGTATCTGGTATAGCATAA
AT---

AACACAACTGAAAACATCACACTCCCATGCAGAATAAAACAAATTATAAACATGTGGCAGGGAGTAGGAAAAG
CAATGTATGCCCCTCCCATCGCGGGACAAATTAAATGTTCATCAAAT---
ATTACAGGACTGCTATTAACAAGAGATGGGGGTGGTGATGGTAAT----------------------------
----

AATGATAGTGAGATCTTCAGACCTGAAGGAGGAAATATGAAGGACAATTGGAGAAGTGAGTTATATAAATATA
AAGTAGTAAAAATTGAACCATTA---GGAATAGCACCCACCAAGGCAAGGAGAAGAGTGGTG-----------
----------------------------CAGAGAGAAAAAAGAGCA---GTG---ACGATGGGAGCT------
---ATGTTCCTTGGGTTCTTGGGAGCA---GCAGGAAGCACTATG---GGCGCAGCATCA---
ATGACGCTGACGGTACAG---GCCAGACTA---TTATTG---TCTGGTATAGTGCAACAGCAAAAC---
AACTTG---CTGAGA---GCTATTGAGGCGCAACAGCATCTGTTGCAGCTCACGGTCTGGGGCATT
>AB588311
TTCTACTGTAATACATCAGACTTGTTTAATAATGACACTTGGAATAACCCTAACAGTACA-------------
----

AAAGGAGAGAATATCACCCTCCAATGCAGAATAAAACAAATTATAAACATGTGGCAGGAAGTAGGAAGAGCAA
TGTATGCTCCTCCCATCAGCGGACCAATTAGATGTTCATCAAAT---
ATTACAGGGCTGATACTAACAAGAGATGGTGGTACTAACCAGACC-------------------------------
----

ACCGAGATCTTCAGACCTGGAGGAGGAGATATGAGGGACAATTGGAGAAGTGAATTATACAAGTATAAAGTAG
TAAAAATTGAACCATTA---GGACTAGCACCCACCAAGGCAAAGAGAAGAGTGGTG--------------------
-----------------------CAGAGAGAAAAAAGAGCA---GTGGGA---ATAGGAGCT-----------
TTGTTCCTTGGGTTCTTGGGAGCA---GCAGGAAGCACTATG---GGCGCAGCGTCA---
TTGACGCTGACGGTACAA---GCCAGACTA---TTATTG---TCTGGTATAGTGCAACAGCAAAAC---
AATTTG---CTGAGG---GCTATTGAGGCGCAACAGCACATGTTGCAACTCACAGTCTGGGGCATC
>AB731663
TTCTACTGTAATTCAACACAACTGTTTAATAGTACTTGGCTGCTTAATAGTACTTGGAATAGTAATGATACAA
AT--
-------------AGCATT------
GGAGAAAATGACACTATCATACTCCCATGCAGAATAAAACAAATTATAAACATGTGGCAGGAAGTAGGAAAAG
CAATGTATGCCCCTCCTAGAGAAGGACCAATTAGATGTTCATCAAAC---
ATTACAGGGCTGATATTAACAAGAGATGGTGGTAGTAATGGGTAT-------------------------------
----

AACAATGAGACCTTCAGACCTGGAGGAGGAGATATGAGGGACAATTGGAGAAGTGAATTATATAAATATAAAG
TAATAAAAATTGAACCATTA---GGAGTAGCACCAACCAAGGCAAAAAGGCGAGTGGTG-----------------
--------------------------CAGAGAGAAAAAAGAGCA---GTGGGA---CTAGGAGCC-----------
TTGCTCGTTGGGTTCTTGGGAGCA---GCAGGAAGCACTATG---GGCGCAGCGTCA---
CTGACGCTGACGGTACAG---GCCAGACAA---TTACTG---TCTGGTATAGTGCAACAGCAAAAC---
AACTTG---CTGAGG---GCTATTGAGGCGCAACAACATCTGTTGCAACTCACAGTCTGGGGCATC
>AB731667
TTTTACTGTAATACAACACCATTGTTTAACAGTACTTGGAATGGAACAAATCTGACTACTTGGAAT----------
----

ACTGGAGCAAATATCACACTCCAATGCAAAATAAAACAGATTATAAACATGTGGCAGCAAGTAGGAAAAGCAA
TGTATGCCCCTCCCATCAGAGGACAAATTAGATGTTCATCAAAT---
ATTACAGGGCTGCTATTAACAAGAGATGGT---AATACTAATAGCACA----------------------------
----

AACAATACTGAGATCTTCAGACCTACAGGAGGGAATATGAAGGACAATTGGAGAAGTGAATTATATAAATATA
AAATAGTACAAATTGAACCATTA---GGAGTAGCACCTACCAAGGCAAAGAGAAGAGTGGTG--------------
-----------------------------CAGAGAGAAAAAAGAGCA---GTGGGA---CTAGGAGCT--------
---ATGTTCCTTGGATTCTTGGGAGCA---GCAGGAAGCACTATG---GGCGCAGCGTCA---
ATAACGCTGACGGTACAG---GCCAGACAA---TTATTG---TCTGGTATAGTGCAACAGCAAAAC---
AACTTG---CTGAGA---GCTATTGAGGCGCAACAGAATATGTTGCAACTCACAGTCTGGGGCATC
>KF716497
TTCTACTGCGATACAACACAACTATTTAATAGTACCTGGGATAATAGTACTATGAATAGTACTAAGAAT-------
----

TCAAATGACACTATCACGCTCCCATGTAGAATAAAACAAATTATAAACATGTGGCAGACAGTAGGAAAAGCAA
TGTATGCCCCTCCCATCGCAGGACCAATTAGATGTTCATCAAAT---
ATTACAGGGTTGTTATTAGAGAGAGATGGTGGTATTAACAAGACG-------------------------------
----

ACCACAGAAACCTTCAGACCTGGGGGAGGAAATATGAAGGACAATTGGAGAAGTGAACTATATAAATATAAAG
TAGTAAAAATTGAACCATTA---GGAGTAGCACCCACCAAGGCAAAGAGAAGAGTGGTG-----------------
--------------------------CAGAGAGAAAAAAGAGCA---GTGGGA---ATAGGAGCT-----------
GTGTTCCTTGGGTTCTTGGGAGCA---GCAGGAAGCACTATG---GGCGCAGCGTCA---
ATGGCGCTGACGGTACAG---GCCAGACAA---TTATTG---TCTGGTATAGTGCAACAGCAGAAC---
AATTTG---CTGAGA---GCCATTGAGGCGCAACAGCATATGTTGCAACTCACAGTCTGGGGCATC
>KF716498
TTCTACTGCAATACAACAAAACTGTTTAATAGTACTTGGAATAATAGTAATTGGCCAAATGACACTATGCCA-
----

AATAATAACACTATCACACTCCCATGTAGAATAAAACAAATTGTAAACATGTGGCAGAAAGTAGGAAAAGCAA
TGTATAACCCTCCCATCGCAGGACTAATTAGATGTTCATCAAAT---
ATTACAGGGCTATTATTAGTGAGAGATGGTGGGAACACAACGAATACA-------------------------
----

ACCACCGAAACCTTCAGACCTGGAGGAGGAGATATGAGGGACAATTGGAGAAGTGAATTATATAAATACAAAG
TAGTAAAAATTGAACCATTA---GGAGTAGCACCCACTAAGGCAAAGAGAAGAGTGGTG--------------
-------------------------CAGAGAGAAAAAAGAGCA---GTGGGG---ATAGGAGCT---------
GTGTTCCTTGGGTTCTTGGGAGCA---GCAGGAAGCACTATG---GGCGCAGCGTCA---
CTAATGCTGACGGTACAG---GCCAGACAA---TTATTG---TCTGGTATAGTGCAACAGCAGAAC---
AATCTG---CTGAGG---GCCATTGAGGCGCAACAGCATATGTTGCAACTCACAGTCTGGGGCATC
>AB588201
TTCTACTGTAACTTATCACAACTGTTTAATAGTTCTTGGAATGTAAATGGTACTGAAGGGTCAAT-------
----

AACACTGGAATTATCACACTCCCATGCAGAATAAAACAAATTATAAACATGTGGCAGGAAGTAGGAAAGGCAA
TGTATGCCCCTCCCATCAGAGGACAAATTAGATGTTCATCAAAT---
ATTACAGGGCTGCTATTAACAAGAGATGGTGGTATTAACAAGAGCGAG-------------------------
----

AGCACCAACACCGAGGTCTTCAGACCTGCAGGAGGAAATATGAAGGACAATTGGAGAAGTGAATTATATAAAT
ATAAAGTAGTAAAAATTGAACCATTA---GGAGTAGCACCCACCAAGGCAAAGAGAAGAGTGGTG--------
-------------------------------CAGAGAGAAAAAAGAGCA---GTGGGA---CTAGGAGCT---
------ATGTTCCTTGGGTTCTTGGGAGCA---GCAGGAAGCACTATG---GGCGCAGCGTCG---
TTGACGCTGACGGTACAG---GCCAGACTA---TTATTG---TCTGGTATAGTGCAACAGCAGAGC---
AATTTG---CTGAGG---GCTATTGAGGCGCAACAGCATCTGTTGCAACTCACAGTCTGGGGCATC
>AB588207
TTCTACTGTGATACATCACGACTGTTTAATAGTACTTGGAATGAGACTACTAAAGGGTTAAATATTACTGGG-
----

GAAAATGACACAATCACAATCCCATGCAGAATAAAACAAATTATAAACATGTGGCAGGAAGTAGGGAAAGCAA
TGTATGCCCCTCCCATCAGTGGAGTAATTAGATGTTCATCAAAT---
ATTACAGGGCTGCTATTAACAAGAGATGGTGGTAACGGTAACGGGAGC-------------------------
----

AATACCACTGAGATCTTCAGACCTATAGGAGGAGATATGAGGGACAATTGGAGAAGTGAATTATATAAATATA
AAGTAGTAAAACTTGAACCATTA---GGAATAGCACCCACCAAGGCAAAGAGAAGAGTGGTG-----------
----------------------------CAGAGAGAAAAAAGAGCA---GTGGGA---ATGGGAGCT------
---ATGTTCCTTGGGTTCTTGGGAGCA---GCAGGAAGCACTATG---GGCGCAGCGTCA---
ATAACGCTGACGGTACAG---GCCAGACAA---TTATTG---TCTGGTATAGTGCAACAGCAGAGC---
AATTTG---CTGAGG---GCTATTGAGGCGCAACAGCATATGTTGCAACTCACAGTCTGGGGCATC
>AB588209
TTCTACTGTAATACAACACAGCTGTTTAATAGTACTTGGACTTGGACTGGGAATAATACTGAAGGAAATAAC-
----

ACTGAATACAATATCACACTCCCATGCAGAATAAAACAAATTATAAACAGGTGGCAGGAAGTAGGAAAAGCAA
TGTATGCCCCTCCCATCAAAGGACAAATTAGTTGTTCATCAAAT---
ATTACAGGGCTGCTATTAACAAGAGATGGTGGTGGTGGCAATAAC----------------------------
----

GAGTCCGAGACCTTCAGACCTGGAGGAGGAGATATGAGGGACAATTGGAGAAGTGAATTATATAAATATAAAG
TAGTACAAATTGAGCCATTA---GGAGTAGCACCCACCAAGGCAAAGAGAAGAGTGGTG--------------
-------------------------CAAAGAGAAAAAAGAGCA---GTGGGA---ATAGGAGCT---------
ATGTTCATTGGGTTCTTGGGAGCA---GCAGGAAGCACTATG---GGCGCAGCGTCA---
GTAACGCTGACGGTACAG---GCCAGACTA---TTATTG---TCTGGTATAGTGCAACAGCAGAAC---
AATCTG---CTGAAA---GCTATTGAGGCGCAACAGCATCTGTTGCAACTCACAGTCTGGGGCATC
>AB588211
TTCTACTGTAATACAACAAAGCTATTTAATAATAGTATGCTGTCTTCTAATAATACTGGAGGA----------
----

TCAAATAAGACTATCACAATCCCATGCAGAATAAAACAAATTATAAACAGGTGGCAGGAAGTAGGAAAAGCAA
TGTATGCCCCTCCCATCAAAGGACAAATTAGCTGTTCATCAAAT---
ATTACAGGACTGCTATTAACAAGAGATGGTGGTGGCGGGAACGGT----------------------------
----

TCCGAGACCGAGACCTTCAGACCTGCAGGAGGAGATATGAGGGACAATTGGAGAAGTGAATTATATAAATATA
AAGTAGTAAAAATTGAGCCATTA---GGAGTAGCACCCACCAAGGCAAAGAGAAGAGTGGTG-----------
----------------------------CAGAGAGAAAAAAGAGCA---GTGGGAACACTGGGAGCT------
---ATGTTCCTTGGGTTCTTGGGAGCA---GCAGGAAGCACTATG---GGCGCAGCGTCA---
CTAACGCTGACGGTACAG---GCCAGACTA---TTATTG---TCTGGTATAGTGCAACAGCAGAAC---
AACATG---CTGAGA---GCTATTGAGGCGCAACAGCACCTGTTGCAACTCACAGTCTGGGGCATC
>AB588233
TTCTACTGTAATTCAACACCACTGTTTAATAGTGCTTGGAATTTTACTAGTACTTGGAATATTATTGAAGGAT
CA---

GATAACACTGAAAATATCACACTCCAATGCAGAATAAAACAAATTATAAACATGTGGCAGGAAGTAGGAAAAG
CAATGTATGCCCCTCCCATCCAGGGACAAATTAACTGCACATCAAAT---
ATTACAGGACTGTTATTAACAAGAGATGGGGGTAAGGATGAGAAT----------------------------
----

AAGACGGAGGAGACCTTCAGACCTGGAGGAGGAGATATGAGGGACAATTGGAGAAGTGAATTATATAAATATA
AAGTAGTAAAAATTGAACCATTA---GGAATAGCACCCACCAAGGCGAGGAGAAGAGTGGTG-----------
----------------------------CAGAGAGAAAAAAGAGCA---GTGGGAGCAATAGGAGCT------
---ATGTTCCTTGGGTTCTTGGGAGCA---GCAGGAAGCACTATG---GGCGCAGCGTCA---
ATAACGCTGACGGTACAG---GCCAGACAA---TTATTG---TCTGGTATAGTGCAACAGCAGAAC---
AATTTG---CTGAGA---GCTATTGAGGCGCAACAGCATCTGTTGCAACTCACTGTCTGGGGCATC
>AB480698
TTCTACTGTAATACAACACAGCTGTTTGATAGTACTTGGCTGTCTAATGGTACTAGGAATGATACTACAGAGT
CA---

GATAGCACTGAGATCATACTCCAGTGCAGAATAAAACAAATTATAAATAGATGGCAGGAAGTAGGAAAAGCAA
TGTATGCCCCTCCCATCAGTGGACAAATTAGATGTACATCAAAT---
ATTACAGGGATGATAATAACAAGAGATGGTGGTACCAATGACACG----------------------------
----

CACGGAGACGAGATCTTCAGACCAGCAGGAGGAGACATGAGGGACAATTGGAGAAGTGAATTATATAAATATA
AAGTAGTAAAAATTGAACCATTA---GGAATAGCACCCACCAAGGCAAAGAGAAGAGTGGTG-----------
----------------------------CAGAGAGGAAAAAGAGCA---GTGGGAACATTAGGAGCT------
---ATGTTCCTTGGGTTCTTGGGAGCA---GCAGGAAGCACTATG---GGCGCAGCGTCG---
CTGACGCTGACGGTACAG---GCCAGACAA---TTATTG---TCTGGTATAGTGCAACAGCAAAAC---
AACATG---CTGAAA---GCTATTGAGGCGCAACAACATATGTTGCAACTCACAGTCTGGGGCATC
>AB564745
TTCTACTGTAATACAACAAAACTGTTTAATAGTACTTGGAATAATACTGAAAGGGTAAATAGCACTATG----
----

GAAAATGGCACAATCACACTCCCATGCAGAATAAAACAAATTATACACATGTGGCAAGAGGTAGGAAAAGCAA
TGTATGCCCCTCCCATCGAAGGAAACATTAGCTGCTCATCAAAT---
ATTACAGGGCTGCTATTAACAAGAGATGGGGGTATTAAGAACGAG----------------------------
----

AACAAGCCCGAGATCTTCAGACCTATAGGAGGAAATATGAAGGACAATTGGAGAAGTGAATTATATAAATATA
AAGTAGTAAAAATTGAACCATTA---GGAATAGCACCCACTAGGGCAAAGAGGAGAGTGGTG-----------
----------------------------CAGAGAGAAAAAAGAGCA---GTGGGA---CTAGGAGCT------
---TTGTTCCTTGGGTTCTTGGGAGCA---GCAGGAAGCACTATG---GGCGCAGCGTCA---
ATGACACTGACGGTACAG---GCCAGACAA---TTATTG---TCTGGTATAGTGCAACAGCAAAAT---
AATTTG---CTGAGG---GCTATTGAGGCGCAACAGCATCTGTTGCAACTCACAGTCTGGGGCATC
>AB604946
TTCTACTGTAACACAACACAACTGTTTAACAGTACTTGGCTGTCTAATGGTACTTTGAATAGTACTGAAACGT
CA---

AATGACACCATTATCACACTCCCATGCAGAATAAAACAAGTTATAAACATGTGGCAGGAAGTAGGAAAAGCCA
TGTATGCCCCTCCCATCAGTGGACAAATTAACTGTACATCAAAT---
ATTACAGGGTTGCTATTGACAAGAGATGGGGGTGAGAATAATACT----------------------------
----

GAAGAGATCTTCAGACCTGGAGGAGGAAATATGAAAGACAATTGGAGAAGTGAATTATACAAATATAAAGTAG
TAAGAATTAAACCATTA---GGAGTAGCACCCACCAAGGCAAAGAGAAGAGTGGTG-----------------
----------------------CAGAGAGAAAAAAGAGAA---CTGACA---CTAGGAGCT---------
TTGTTCCTTGGGTTCTTGGGAGCA---GCAGGAAGCACTATG---GGCGCAGCGTCA---
TTGACGCTGACGGTACAG---GCCAGACAA---TTGCTG---TCTGGTATAGTGCAACAGCAAAAC---
AATTTG---CTGAGG---GCTATTGAGGCGCAGCAGCATCTGTTGCAACTCACAGTCTGGGGCATT
>D12582
TTCTACTGTGATTCATCACAACTGTTTAATAGTACACACCTGTCTAATGGTACTTGGTGGAATGGTACT----
----

GGGCCAGAAAATATCACACTCCCCTGCAGAATAAAACAAATTGTAAACATGTGGCAGGAAGTAGGAAAAGCAA
TGTATGCCCCTCCCATCAGAGGACAAATTAGATGTTCATCAAAT---
ATTACAGGGCTGCTATTAACAAGAGATGGTGGTAACACCCAGAACAATAAC----------------------
----

ACCAATAGCAGTATCGAGATCTTCAGACCTGGAGGAGGAGATATGAGGGACAATTGGAGAAGTGAATTATATA
AATATAAAGTAGTAAAAATTGAACCATTA---GGAGTAGCACCCACCAGGGCAAAGAGAAGAGTGGTG-----
----------------------------------CAGAGAGAAAAAAGAGCA---GTGGGA---
ATAGGAGCT---------GTGTTCCTTGGGTTCTTGGGAGCA---GCAGGAAGCACTATG---
GGCGCAGCGGCA---GTGACGCTGACGGTACAG---GCCAGACAA---TTATTG---
CCTGGTATAGTGCAACAGCAGAAC---AATTTG---CTGAGG---
GCTATTGATGCGCAACAACATCTGTTGCAACTCACAGTCTGGGGCATC
>AB565497
TTCTACTGTAATACAACACAACTATTTAATAGTACTTGGCATTGGAATTGGAATAATTCTACTTGGGAGCGGA
ATGATACTGAA--
----------GGGTCAAAT------
AATACTGAGGACAAAATTACACTCCCATGCAGAATAAAACAAATTATAAACATGTGGCAGGAAGTAGGAAAAG
CAATGTATGCCCCTCCCATCAGAGGACTAATTAGATGTGCATCAAAT---
ATTACAGGGCTGATATTAACAAGAGATGGTGGTGGTGTGAACAGA----------------------------
----

ACCACCGAGACATTCAGACCTGGAGGAGGGAATATGAGGGACAATTGGAGAAGTGAATTATATAAATATAAAG
TAGTAAAAATTGAACCATTA---GGAGTAGCACCCACTAAGGCAAAGAGAAGAGTGGTG--------------
-------------------------CAGAGAGAAAAAAGAGCA---GTGGGA---ATTGGAGCT---------
ATATTCCTTGGGTTCTTGGGAGCA---GCAGGAAGCACTATG---GGCGCAGCGTCA---
GTGACGCTGACGGTACAA---GCCAGACAA---TTACTG---TCTGGTATAGTGCAACAGCAGAGC---
AATCTG---CTGCAG---GCTATTGAGGCGCAACAACAACTGTTGAAACTCACAGTCTGGGGCATC
>AB565502
TTCTACTGTAATACATCAAAACTGTTTAATAGTTCTTGGAATATTAATGGTACTTGGAATGGTACTACA----
----

GGAAATGGCACTCTCACGCTCCCTTGCAGAATAAGACAAATTGTAAACATGTGGCAGCAAGTAGGAAAAGCAA
TGTATGCCCCTCCCGTTGCAGGAATAATTAGATGTTCATCAAAT---
ATTACAGGGATGATATTAACAAGAGATGGTGGTACTAATGAGACC----------------------------
----

ATCGAGGTGGAGACCTTCAGACCTGCAGGAGGAGATATGAGGGACAATTGGCGAAGTGAATTGTATAAATATA
AAGTAGTAAGAATTGAACCATTA---GGAGTAGCACCCACCAAGGCAAGGAGAAGAGTGGTG-----------
----------------------------CAGAGAGAAAAAAGAGCA---GTGGGA---CTAGGAGCT------
---GTGTTCCTTGGGTTCTTGGGAGCA---GCAGGAAGCACTATG---GGCGCAGCATCA---
ATAACGCTGACGGTACAG---GCCAGACAA---TTGCTG---TCTGGTATAGTGCAACAGCAGAGA---
AATTTG---CTGAGA---GCTATTGAGGTGCAGCAGCATCTGTTGCAACTCACAGTCTGGGGCATC
>KT961002
TTCTATTGTAATACAACAAAACTGTTTAATAGTAGTTGGAATGAAACAACACTGTTTAATAATACTTGGAATA
GTACTGAA---

GAGTCAAATAGCACAATCACACTCCCATGCAGAATAAAACAAACTATAAACATGTGGCAGGAAGTAGGAAAAG
CAATGTATGCCCCTCCCATCCGCGGACAAATTAATTGTTCATCAAAT---
ATTACAGGGCTGCTATTAACTAGAGATGGTGGTAATAATAACACG----------------------------
----

AATAAAAACGAGACCTTCAGACCTGGAGGAGGAAATATGAAGGACAATTGGAGAAGTGAATTATATAAATATA
AAGTAGTAAAAATTGAACCATTA---GGAATAGCACCCACCAAGGCAAAAAGAAGAGTGGTG-----------
----------------------------CAGAGAGAAAAAAGAGCA---GCA------ATAGGAGCT------
---ATGTTCCTTGGGTTCTTGGGAGCA---GCAGGAAGCACTATG---GGCGCAGCGGCA---
GTGACGCTGACGGTACAG---GCCAGACAA---TTATTG---TCTGGTATAGTGCAACAGCAAAAC---
AATTTG---CTGAGA---GCTATTGAGGCGCAACAACATATGTTGCAACTCACAGTCTGGGGCATC
>AB742153
TTCTACTGTAGTACAACTAAATTGTTTAATAGTACTTGGAATAGTACTGAGAATTTGAATATTACTACTGAAA
GGTCAAAT---
-------------AGCACT------
GGAGAAAACGACACAATCATACTCCACTGTAGAATAAAACAAATTATAAATATGTGGCAAGAAGTAGGAAAAG
CAATGTATGCCCCTCCCATCAGTGGGCCAATTAACTGCTCATCAAAA---
ATTACAGGGCTGCTGTTAACAAGAGATGGTGGTAGTGGTAATGAGACT-------------------------
----

ACCAATAACACTGAAACCTTCAGACCTGGAGGAGGAGATATGAGGGACAATTGGAGAAGTGAATTATATAAAT
ATAAAGTAATAAAAATTGAACCATTA---GGAATAGCACCCACCAAGGCACAGAGAAGAGTGGTG--------
-------------------------------CAGAGAGAGAAAAGAGCA---GTGGGA---ATAGGAGCT---
------GTGTTCCTTGGGTTCTTGGGAGCA---GCAGGAAGCACTATG---GGCGCAGCGTCA---
GTGACGCTGACGGTACAG---GCCAGACTA---TTATTG---TCTGGTATAGTGCAACAGCAGAAT---
AATCTG---CTGAGA---GCTATTGAGGCGCAACAGCATCTGTTGCAACTCACAGTCTGGGGCATC
>AB641836
TTCTACTGCAATACAACAGAACTATTTAATAGTAGTTGGAATGAGAGTACCTGGAATGAGAGTACTCGGAATG
ATACT--
-------------------GAC---
GGGTCAGGAAATATCATCACACTCCCATGTAGAATAAAACAAATTATAAACATGTGGCAGGAAGTAGGAAAAG
CAATGTATGCCCCTCCCATCAGAGGACAAATTAATTGTACATCAAAC---
ATCACAGGGTTGCTATTAACAAGAGATGGTGGTAATAATAATAGCACC-------------------------
----

AATGAGACGGAGACCTTCAGACCTGGAGGAGGAGACATGAAGGACAATTGGAGAAGTGAATTATATAAATATA
AAGTAGTAAAACTTGAACCATTA---GGAATAGCACCCACCAAGGCGAAGAGAAGAGTGGTG-----------
----------------------------CAGAGAGAAAAAAGAGCA---GTGGGAACAATAGGAGCT------
---ATGTTCCTTGGGTTCTTGGGAGCA---GCAGGAAGCACTATG---GGCGCAGCGTCA---
ATGACGCTGACGGTACAG---GCCAGGCTA---TTATTG---TCTGGTATAGTGCAGCAGCAGAAT---
AATCTG---CTGAAG---GCTATTGAGGCGCAACAGCATCTGTTGCAACTCACAGTCTGGGGCATC
>KU869602
TTCTACTGTAATACAACACAACTGTTTAATAGTACTTGGGATTTTAATAGTACTTGGAATAGTACTGAAGGG-
----

TCAAATATCACACTCACACTCCCATGCAGAATAAAACAAATTATAAACAGGTGGCAGGAAGTAGGAAAAGCAA
TGTATGCCCCTCCCATCAGTGGACAAATTAGATGTTCATCAAAT---
ATTACAGGGCTGCTATTAACAAGAGATGGGGGGAAGACTGGGGAC----------------------------
----

AACGGGAGTGAGATCTTCAGACCTGGGGGAGGAGATATGAGGGACAATTGGAGAAGTGAATTATATAAATATA
AAGTAGTAAAAATTGAACCATTA---GGGATAGCACCCACCGAGGCAAGGAGAAGAGTGGTG-----------
----------------------------CAGAGAGAAAAAAGAGCA---GTGGGAACAATAGGAGCT------
---ATGTTCCTTGGGTTCTTGGGAGCA---GCAGGAAGCACTATG---GGCGCAGCGTCA---
ATGACGTTGACGGTACAG---GCCAGACTA---TTGTTG---TCTGGTATAGTGCAACAGCAGAAT---
AACTTG---CTGAGA---GCTGTTGAGGCGCAACAACATCTGTTGCAACTCACAGTCTGGGGCATC
>MN043590
TTCTACTGTAATACAACACAACTGTTTAATAGTACTTGGGATTTTAATAGTACTTGGAATAGTACTGAAGGG-
----

TCAAATATCACACTCACACTCCCATGCAGAATAAAACAAATTATAAACAGGTGGCAGGAAGTAGGAAAAGCAA
TGTATGCCCCTCCCATCAGTGGACAAATTAGATGTTCATCAAAT---
ATTACAGGGCTGCTATTAACAAGAGATGGGGGGAAGACTGGGGAC----------------------------
----

AACGGGAGTGAGATCTTCAGACCTGGGGGAGGAGATATGAGGGACAATTGGAGAAGTGAATTATATAAATATA
AAGTAGTAAAAATTGAACCATTA---GGGATAGCACCCACCGAGGCAAGGAGAAGAGTGGTG-----------
----------------------------CAGAGAGAAAAAAGAGCA---GTGGGAACAATAGGAGCT------
---ATGTTCCTTGGGTTCTTGGGAGCA---GCAGGAAGCACTATG---GGCGCAGCGTCA---
ATGACGTTGACGGTACAG---GCCAGACTA---TTGTTG---TCTGGTATAGTGCAACAGCAGAAT---
AACTTG---CTGAGA---GCTGTTGAGGCGCAACAACATCTGTTGCAACTCACAGTCTGGGGCATC
>MN043603
TTCTACTGTAATACAACAAAACTGTTCAATAGTACTTGGAATAGTACTGGAGAGTTGCTTAATAGTACTGGGA
ATAGTACT--

AACAATGGAACTATCATACTCCAATGCAGAATAAAACAAATTATAAACAGGTGGCAGGAAGTAGGAAAAGCAA
TGTATGCCCCTCCCATTGAAGGGATAATTAGATGTTCATCAAAT---
ATTACAGGGCTGCTATTAACAAGAGATGGGGGGAATGAAAACGGGACT-------------------------
----

AACAGTACTGAGACCTTCAGACCTGGGGGAGGAAATATGAAGGACAATTGGAGAAGTGAATTATATAAATATA
AAGTAGTAAGAATTGAGCCATTA---GGGATAGCACCCACCGAGGCAAGGAGAAGAGTGGTG-----------
----------------------------CAGAGAGAAAAAAGAGCA---GTGGGAACAATAGGAGCT------
---ATGTTCCTTGGGTTCTTGGGAGCA---GCAGGAAGCACTATG---GGCGCAGCGTCG---
ATGACGCTGACGGTACAG---GCCAGACTA---TTATTG---TCTGGTATAGTGCAACAGCAGAAT---
AATTTG---CTGAGA---GCTATTGAGGCGCAACAGCATCTGTTGCAACTCACAGTCTGGGGCATC
>JQ316128
TTCTACTGTAATACAACAAAACTGTTTAGTAGTACTTGGAATGATACTGAAGGCCCAAATAATGGA-------
----

ACCAATAGAAATATCACACTCCAATGCAGAATAAAACAAATTATCAACAGGTGGCAGGAAGTAGGAAAGGCAA
TGTATGCCCCTCCCATCGCAGGAATAATTAGATGTTCATCAAAT---
ATTACAGGACTGCTATTAACAAGAGATGGGGGGAATAATGAGAATGGG-------------------------
----

ACCAGTGGGAATGAGACCTTCAGACCTGGGGGAGGAAATATGAAGGACAATTGGAGAAGTGAATTATATAAAT
ATAAAGTAGTAAAAATTGAGCCATTG---GGGATAGCACCCACCGAGGCAAGGAGAAGAGTGGTG--------
-------------------------------CAGAGAGAAAAAAGAGCA---GTGGGAGCGCTAGGAGCT---
------ATGTTCCTTGGGTTCTTGGGAGCA---GCAGGAAGCACTATG---GGCGCAGCGTCA---
CTGACGCTGACGGTACAG---GCCAGACAA---TTATTG---TCTGGTATAGTGCAACAGCAGAAT---
AATTTG---CTGAGA---GCTATTGAGGCGCAACAGCATCTGTTGCAACTCACAGTCTGGGGCATC
>JQ316132
TTTTACTGTAATACAACACAACTGTTTAATAGTACTTGGAGGGTGAATGGTACTGAAGGGTTAAATGTAACTG
AA--

GGAAATAGACACTTTACCCTCCCATGCAGAATAAAACAAATTATAAACAGGTGGCAGGAAGTAGGAAGAGCAA
TGTATGCCCCTCCCATCGCGGGACAAATTAGATGTTCCTCAAAT---
ATTACAGGACTGCTATTAACAAGAGATGGGGGAACTAAGAACAACAGCAACAAC-------------------
----

GAGACTGATACTGAGATCTTCAGACCTGGGGGAGGAGATATGAGGGACAATTGGAGAAGTGAATTATATAAAT
ATAAAGTAGTAAAAATTGAGCCATTA---GGGATAGCACCCACCGAGGCAAGGAGAAGAGTGGTG--------
-------------------------------CAGAGAGAAAAAAGAGCA---GTGGGAGTGATAGGAGCT---
------ATGTTCCTTGGGTTCTTGGGAGCA---GCAGGAAGCGCTATG---GGCGCAGCGGCA---
ATAACGCTGACGGTACAG---GCCAGACAA---TTATTG---TCTGGTATAGTGCAACAGCAAAAT---
AATTTG---CTGAGA---GCTATTGAGGCGCAACAGCATCTGTTGCAACTCACAGTCTGGGGCATC
>JQ316127
TTCTACTGTAATACAACACAACTGTTTAATAGTACTTGGGAGTTTAATAGTACTGAAGGGTCAAATACAACTG
AAGGA---

AATAACACACTTATCACACTCCCATGCAGAATAAAACAAATTATAAACAGGTGGCAGGAAGTAGGAAAAGCAA
TGTATGCCCCTCCCATCAGAGGACAAATTAAATGTTCATCAAAT---
ATTACAGGGTTGCTATTAACAAGAGATGGGGGGACTGGGAACAACAAG-------------------------
----

ACTGACAACGGGACTGAGATCTTCAGACCTGGGGGAGGAGATATGAGGGACAATTGGAGAAGTGAATTATATA
AATATAAAGTAGTAAAAATTGAGCCATTA---GGGATAGCACCCTCCAAGGCAAGGAGAAGAGTGGTG-----
----------------------------------CAGAGAGAAAAAAGAGCA---
GTGGGAACGATAGGAGCT---------ATGTTCCTTGGGTTCTTGGGAGCA---GCAGGAAGCACTATG---
GGCGCAGCGTCA---CTGACGCTGACGGTACAG---GCCAGACTC---TTATTG---
TCTGGTATAGTGCAACAGCAGAAT---AATTTG---CTGAGA---
GCTATTGAAGCGCAACAGCATCTGTTGCAACTCACAGTCTGGGGCATC
>JQ316135
TTTTATTGTAATACAACACAACTGTTTAATAGTACTTGGAATGGTATTACTTTTAATAGAACTTGGGGTGAC-
----

CTTAATAAAAAGATTATATTCCCTTGCAAATTAAAACAAATTATAAACAGGTGCCAGGAAGTAGGAAAAGCAA
TGTATGCCCCTCCCTTCAGAGGACAAATTAGATGTTCATCAAAT---
ATTACAGGGCTGTTATTACCAAGAGATGGGGGGACCAAGAACAAAACTGAC----------------------
----

AAGGCCATTATTAAATTTTTCAGCCTTGGGGGAGGAGATATGAGGGACAATTGGAGAAGTAAATTATATAAAT
ATAAAGTAGTAAAAATTGAGCCATTA---GGGATAGCACCCACCACGGCAAGGAAAAGAGTGGTG--------
-------------------------------CAGAGAGAAAAAAGAGCA---GTGGGAACGATAGGAGCT---
------ATGTTCCTTGGGTTCTTGGGAGCA---GCAGGAAGCACTATG---GGCGCAGCGTCA---
ATGACGCTGACGGTACAG---GCCAGACTA---TTATTG---TCTGGTATAGTGCAACAGCAGAAT---
AATTTG---CTGAAA---GCTATTGAGGCGCAACAGCATCTGTTGCAACTCACAGTCTGGGGCATC
>JQ316130
TTCTACTGTAATACCACACCACTGTTTAATAGTACTTGGCGGTATAATAGTACTGGG----------------
----

GAAGAGGAAACTATCATACTCCCATCCAGGATAAAACAAATTATTATCAGCTGGCAGGAAGTAGGAACAGCAA
CGTATGCCCCTCCCATCAGCGGAATAATTAGCTGTTCATCAAAT---
ATTACAGGGGGGCTATTAACAACAGATGGGGGGAGTGAGAAAAACGGG-------------------------
----

ACTAACGATACTGAGACCTTCAGACCTGGGGGAGGAGATATGAGGGACAATTGGAGAAGTGAATTATATAAAT
ATAAAGTAGTAAAAATTGAGCCATTA---GGGATAGCACCCACCGAGGCAAGGAGAAGAGTGGTG--------
-------------------------------CAGAGAGAAAAAAGAGCA---GTAACA---ATAGGAGCT---
------CTGTTCCTTGGGTTCTTGGGAGCA---GCAGGAAGCACTATG---GGCGCAGCGTCA---
ATGACGTTGACGGTACAG---GCCAGACTA---TTGTTG---TCTGGTATAGTGCAACAGCAGAAT---
AACTTG---CTGAGA---GCTATTGAGGCGCAACAGCATATGTTGCAACTCACAGTCTGGGGCATC
>DQ295195
TTTTACTGTAATACAACACAGCTGTTTAATAGCACTTGGCCTAATAGTAGTGAGAATTCCAATAATACTGCT-
----

GATAATGGCACATTTATCCTCCCCTGCAGAATAAAACAAATTATAATCAGCTGGCAGGAAGTAGGAAAAGCAA
TATATGCCCCTCCCATCAGTGGACTAATTAAATGTTCATCAAAT---
ATTACAGGGCTAATATTAACAAGAGATGGGGGAACTAAAAACAATTGCATT----------------------
----

AACATCACCGGGGAGATCTTCAGACCTGGGGGAGGAAATATGAAGGACAATTGGAGAAGTGAATTATATAAAT
ATAAAGTAGTAAGAATTGAGCCATTA---GGGGTAGCACCCACTGGGGCAAAGAGAAGAGTGGTG--------
-------------------------------CAGAGAGAAAAAAGAGCA---GTGGGAACGATAGGAGCT---
------ATGTTCCTTGGGTTCTTGGGAGCA---GCAGGAAGCACTATG---GGCGTCGCGTCA---
ATGACGCTGACGGTACAG---GCCAGACTA---TTATTGTCTGGCTGTATAGTGCAACAGCAGAAT---
AATCTG---CTGAGA---GCTATTGAGGCGCAACAGCATCTGTTGCAACTCACAGTTTGGGGCATC
>DQ295193

TTCTACTGTAATACAACACAACTGTTTAATAGTACTTGGCTGCTTAATGATACTGGGAATGAGAGTAAACAG-
----

CCAAATGAAACTATCACACTCCCATGCAGAATAAAACAAATTATAAACAGGTGGCAGGAAGTAGGAAAAGCAA
TGTATGCCCCTCCCATCAGCGGACAAATTAGATGTTCATCAAAT---
ATTACAGGGCTGTTGTTAACAAGAGATGGGGGGAATACTAATGAGACT-------------------------
----

GGGAATGGGACTGAGACCTTCAGACCTGGGGGAGGAGATATGAGGGACAATTGGAGAAGTGAATTATATAAAT
ATAAAGTAGTACAAATTGAGCCATTA---GGGATAGCACCCACTAAGGCAAGGAGAAGAGTGGTG--------
-------------------------------CAGAGAGAAAAAAGAGCA---GTGGGAGTAATAGGAGCT---
------ATGTTCCTTGGGTTCTTGGGAGCA---GCAGGAAGCACTATG---GGCGCAGCGTCA---
GTGACGTTGACGGTACAG---GCCAGACTA---TTATTG---TCTGGTATAGTGCAACAGCAAAAT---
AATTTG---CTGAGA---GCTATTGAGGCGCAACAGCATCTGTTGCAACTCACAGTCTGGGGCATC
>JQ316126
TTTTACTGTAATACAACAAAACTGTTTAATAGTACTTGGTATGGTAATGGTACTCGGAAGGAAGGGTCAAAT-
----

GAAAATGGAACTATCATACTCCCATGCAGGATAAGACAAATTGTAAACAGGTGGCAGGAAGTAGGAAAAGCAA
TGTATGCCCCTCCCATTACAGGACAAATTAGATGTTCATCAAAT---
ATTACAGGGCTGCTATTAACAAGAGATGGGGGGACTGGGAACGGGACC-------------------------
----

AACACGAATGAGACTTTCAGACCTGGGGGAGGAAATATGAAGGACAATTGGAGAAGTGAATTATATAAATATA
AAGTAGTAAGAATTGAGCCATTA---GGGATAGCACCCACCCAGGCAAGGAGAAGAGTGGTG-----------
----------------------------CAGAGAGAAAAAAGAGCA---GTAACG---CTAGGAGCT------
---ATGTTCCTTGGGTTCTTGGGAGCA---GCAGGAAGCACTATG---GGCGCAGCGTCA---
ATAACACTGACGGTACAG---GCCAGACTA---TTATTG---TCTGGTATAGTGCAACAGCAAAAT---
AATTTG---CTGAGA---GCTATTGAGGCGCAACAGCATCTGTTGCAACTCACAGTCTGGGGCATC
>DQ295196
TTCTACTGTAATACATCACAACTGTTTAATAGAGCTTGGAATGGTTCTGAAAGGTCCAATAACACTGAA----
----

GGAAATACAACTATCACACTCCCATGCAGAATAAAACAAATTATAAACAGGTGGCAAGAAGTAGGAAAAGCAA
TGTATGCCCCTCCCATCAGCGGACAAATTAGATGTTCATCAAAT---
ATTACAGGGCTGTTATTAACAAGAGATGGGGGGAATGGGAACGAGACT-------------------------
----

GAGAACGGGACTGAAATCTTCAGACCTGGGGGAGGAAATATGAGGGACAATTGGAGAAGTGAATTATATAAAT
ATAAAGTAGTAAAAATTGAGCCATTA---GGGATAGCACCCACCAGGGCAAAGAGAAGAGTGGTG--------
-------------------------------CAGAGAGAAAAAAGAGCA---GTGGGAACGATAGGAGCT---
------ATGTTCCTTGGGTTCTTGGGAGCA---GCAGGAAGCACTATG---GGCGCAGCGTCA---
GTAACGCTGACGGTACAG---GCCAGACTA---TTATTG---TCTGGTATAGTGCAACAGCAGAAT---
AATTTA---CTGAAA---GCTATTGAGGCGCAACAGCGTCTGTTGCAACTCACAGTCTGGGGCATC
>AY839827
TTCTACTGTAATACAACACAACTGTTTAATAGTACTTGGTACAGTAATAATACTGAAGGGTCAAATAATACTA
AA---

GGAAATGACACAATTACCCTCCCATGCAGAATAAGACAAATTATAAACAGATGGCAGGAAGTAGGAAAAGCAA
TGTATGCTCCTCCCATCAGTGGACAAATTAGATGTTCATCAAAT---
ATTACAGGGCTGCTGTTAACAAGAGATGGGGGAATCAACAATACA----------------------------
----

ATTAATGAGACCTTCAGACCTGAGGGAGGAGATATGAGGGACAATTGGAGAAGTGAATTATATAAATATAAAG
TAGTAAGAATTGAGCCATTA---GGGATAGCACCCACCGAGTCAAAAAGAAGAGTGGTG--------------
-------------------------CAGAGAGAAAAAAGAACA---GTGGGAACATTAGGAGCT---------
ATGTTCCTTGGGTTCTTGGGAGCA---GCAGGAAGCACTATG---GGCGCAGCGTCG---
ATGACGTTGACGGTACAG---GCCAGACTA---TTATTG---TCTGGTATAGTGCAACAGCAAAAT---
AACTTG---CTGAGA---GCTATTGAGGCGCAACAGCATCTGTTGCAACTCACAGTCTGGGGCATC
>DQ837381

TTCTACTGTAATACAACACAACTGTTTAATGGTAATTGGAGTAATGGTACCTGGAATGATCTAAACAACTCT-
----

AGTTCTATAAATATCACACTTCCATGCAGAATAAAACAAATTGTAAACATGTGGCAGGAAGTAGGAAAAGCAA
TGTATGCCCCTCCCATCAAAGGACAAATTAGCTGTTCATCAAAT---
ATTACAGGGCTGATATTAACAAGAGATGGTGGTAAGGACAATACTACCGGCAGC-------------------
----

AATGAGACAAAAGGGGAGATCTTCAGACCTGCAGGAGGAGATATGAGGGACAATTGGAGAAGTGAATTATATA
AATATAAAGTAGTAAGAATTGAACCATTA---GGAGTAGCACCCACCAAGGCAAGGAGAAGAGTGGTG-----
----------------------------------CAGAGAGAAAAAAGAGCA---GTGACA---
CTGGGAGCG---------ATGTTCCTTGGTTTCTTGGGAGCA---GCAGGAAGCACTATG---
GGCGCAGCGTCA---ATGACGCTGACGGTACAG---GCCAGACAA---TTATTA---
TCTGGTATAGTGCAACAGCAAAAC---AACTTG---CTGAGG---
GCTATTGAGGCGCAACAGCATCTGTTGCAACTCACAGTCTGGGGCATC
>JQ316134
TTCTACTGTAACACAACAAAACTGTTCAATAGTACTTGGAATAGTACTGAGAATGGTACTGGTATTGAACAT-
----

TTAAATGGGACTATCACACTCCCATGCAGAATAAAACAATTTATAAACAGGTGGCAGGAAGTAGGAAAAGCAA
TGTATGCCCCTCCCATCAGAGGAATAATTAGATGTTCATCAAAT---
ATTACAGGGCTGCTATTAACAAGAGATGGGGGGACTGAGAACAACAAAACTGAC-------------------
----

AGCACTGGGACTGACATCTTCGGACCTGGGGGAGGAGATATGAGGGACAATTGGAGAAGTGAATTATATAAAT
ATAAAGTAGTAAGAATTGAGCCATTA---GGGATAGCACCCACCAAGGCAAGGAGAAGAGTGGTG--------
-------------------------------CAGAGAGAAAAAAGAGCA---GTAACG---ATAGGAGCT---
------ATGTTCCTTGGGTTCTTGGGAGCA---GCAGGAAGCACTACG---GCCGCGACGTCG---
ATGACACTGACCGTACAA---GCCATACTA---TTATTG---TCTGGTATAGTGCAACAGCAGAAT---
AATTTG---CTGAGA---GCTATTGAGGCGCAACAACATCTGTTGCAACTCACAGTCTGGGGCATC
>MN043605
TTCTACTGTAGTACAACAAATCTGTTTAATAGTTCTTGGAATGAAACACTGCTTAATAGTACTTGGAATGAGA
AT---

GACAATAAAAATATCACACTCCGATGCAGAATAAAACAAATTATAAACAGGTGGCAGGAAGTAGGAAAAGCAA
TGTATGCCCCTCCCATCAGAGGACAAACTAGATGTTCATCAAAT---
ATTACAGGGCTGCTATTAACAAGAGATGGGGGGCCTGAGAACAAT----------------------------
----

AGCAATGAAATCTTCAGACCTGGGGGAGGAAATATGAAGGACAATTGGAGAAGTGAACTATATAAATACAAAG
TAGTAAAGATAGAGCCATTA---GGGATAGCACCCACCAAGGCAAGGAGAAGAGTGGTG--------------
-------------------------CAGAGAGAAAAAAGAGAA---GTAACA---TTAGGAGCC---------
TTGTTCCTTGGGTTCTTGGGAGCA---GCAGGAAGCACTATG---GGCGCAGCGTCA---
ATGACGCTGACGGTACAG---GCCAGACTA---TTATTG---TCTGGTATAGTGCAACAGCAAAAT---
AATTTG---CTGAGA---GCTATTGAGGCGCAACAGCATATGTTGCAACTCACAGTCTGGGGCATC
>KJ140263
TTCTACTGTGAGACAACAAAACTGTTTAATAGTACTTGGAGGTTTAATAGTACT-------------------
----
-TGGAATGGTACTGAA---------
GAGATAAATGACCCAATTACACTCCCATGCAGAATAAAACAAATTATAAACAGGTGGCAGGAAGTAGGAAAAG
CAATGTATGCCCCTCCCATCAGAGGACAAATTAAATGTTCATCAAAT---
ATTACAGGGCTGCTATTAACAAGAGATGGGGGGACT-------------------------------------
----
GAGAACAACAAGACTGGGAGCAACAGGACTGAGATCTTCAGACCAGGGGGAGGAGATATGAGGGACAATTGGA
GAAGTGAATTATATAAATATAAAGTAGTAAGAATTGAGCCATTA---
GGGATAGCACCCACCAAGGCAAGGAGGAGAGTGGTG-------------------------------------
--CAGAGAGAAAAAAGAGCA---GTGGGAACGATAGGAGCT---------
ATGTTCCTTGGGTTCTTGGGAGCA---GCAGGAAGCACTATG---GGCGCAGCGTCA---
ATGACGCTGACGGTACAG---GCCAGACTA---TTATTG---TCTGGTATAGTGCAACAGCAGAAT---
AATTTG---CTGAGA---GCTATTGAGGCGCAACAGCATCTGTTGCAACTCACAGTCTGGGGCATC

>KT878030
TTCTACTGTAATACATCACAACTGTTTAATAGTACTTGGAATGATACTTGGAGGGGTAGTAAAAGA-------
----

TCCAATGGTACTATCACACTCCCATGCAGAATAAAACAAATTATAAATAGGTGGCAGGAAGTAGGGAAAGCAA
TGTATGCCCCTCCCATCAGTGGACAAATTAGATGTACATCAAAT---
ATTACAGGGCTGATATTAACAAGAGATGGGGGGAATAACAGCGAG----------------------------
----

ACTACAGAGATCTTCAGACCTGGGGGAGGAAATATGAAGGACAATTGGAGAAGTGAATTATATAAATATAAAG
TAGTAAGAATTGAGCCATTA---GGGATAGCACCCACCAAGTCAAAGAGAATAGTGATG--------------
-------------------------CAGAGAGAAAAAAGAGCA---GTGGGAACAATAGGAGCT---------
ATGTTCCTTGGGTTCTTGGGAGCA---GCAGGAAGCACTATG---GGCGCAGCGTCA---
TTGACGCTGACGGTACAG---GCCAGACAA---TTATTG---TCTGGTATAGTGCAACAGCAGAAT---
AATTTG---CTGAGA---GCTATTGAGGCGCAACAACATATGTTGCAGCTCACAGTCTGGGGCATC
>KT878031
TTCTACTGTAATACATCACAATTGTTTAATAGTACCTGGAATGAAACACTGTTGAATAGTACCTGGAAT----
--GAA---
ACACTGTCGAATAGAACCTGG---------------
AATAGTAATGAAACTATCACACTTCCATGCAGAATAAAACAAATTGTAAACAGGTGGCAGGAAGTAGGAACAG
CAATGTATGCCCCTCCCATCAAAGGACAAATTAAATGCTCATCAAAT---
ATTACAGGGCTGTTATTAACAAGAGATGGGGGGAACAACAGCAAC----------------------------
----

AGCAATGAGACCTTCAGACCTGGGGGAGGAGATATGAGGGACAATTGGAGAAGTGAATTATATAAATATAAAG
TAGTAAAAATTGAGCCATTA---GGGATAGCACCCACCAAGGCAAGGAGAAGAGTGGTG--------------
-------------------------CAGAGAGAAAAAAGAGCA---GTGGGAACAATAGGAGCT---------
ATGTTCCTTGGGTTCTTGGGAGCA---GCAGGAAGCACTATG---GGCGCAGCGTCA---
ATGACGCTGACGGTACAG---GCCAGACAA---TTATTG---TCTGGTATAGTGCAACAGCAGAAT---
AACCTG---CTGAGA---GCTGTTGAGGCGCAACAGCATCTGTTGAAACTCACAGTCTGGGGCATC
>KT878032
TTCTACTGTAATACAACACGACTGTTTAATAGTACTTGGAAGAATGGTACACTGATAAATGGAACTGAAGACA
ATAGC--

AATAGCACCTTCATCACACTCCCATGCAGAATAAAACAAATTATAAACAGGTGGCAGGAAGTAGGAAAGGCAA
TGTATGCCCCTCCCATCAGTGGACTAATTAGATGTACATCAAAT---
ATTACAGGGCTGCTATTAACAAGAGATGGGGGGAACGACAACAGT----------------------------
----

AGTAGCAACGAGACCTTCAGACCTGGGGGAGGAAATATGATGGACAATTGGAGAAGTGAATTATATAAATATA
AAGTAGTAAGAATTGAGCCATTA---GGGATAGCACCCACCGATGCAAAGAGAAGAGTGGTG-----------
----------------------------CAGAGAGAAAAAAGAGCA---GTGGGAACACTGGGAGCT------
---ATGTTCCTTGGGTTCTTGGGAGCA---GCAGGAAGCACTATG---GGCGCAGCGTCA---
ATGACGCTGACGGTACAG---GCCAGACTA---TTATTG---GCTGGTATAGTGCAACAGCAAAAT---
AATTTG---ATGAGA---GCTATTGAGGCGCAACAGCATCTGTTGCAACTCACAGTCTGGGGCATC
>KT878033
TTCTACTGTAACACATCAAAATTGTTTAATACTACTTGGAGTAAGGATAATAATACTGAACCAAATGAAAATG
GA---

ACTGAGAAAGTTATCACAATCCCATGCAGAATAAAACAAATTATAAACAGGTGGCAGGAAGTAGGAAGAGCAA
CATATGCCCCTCCCATCAAAGGAATAATTAGATGTTCATCAAAT---
ATTACAGGGCTGCTACTAACAAGAGATGGGGGAGGAGGAAACAAG----------------------------
----

ACAGAAGAAACCTTCAGACCTGGGGGAGGAGATATGAAGGACAATTGGAGAAGTGAATTATACAAATATAAAG
TAGTAAAAATTGAGCCATTA---GGGATAGCACCCACCGAGGCAAGGAGAAGAGTGGTG--------------
-------------------------CAGAGAGAAAAAAGAGCA---GTGGGAACAATAGGAGCT---------
ATGTTCCTTGGGTTCTTGGGAGCA---GCAGGAAGCACTATG---GGCGCAGCGTCA---
CTGACGCTGACGGTACAG---GCCAGACTA---TTATTG---TCTGGTATAGTGCAACAGCAGAGT---
AATTTG---CTGAGA---GCTATTGAGGCGCAACAGCATATGTTGCAACTCACAGTCTGGGGCATC

>KT878036
TTCTACTGTAACACAACACAACTGTTTAATAGTACCTGGCTTGGGAATGATACTAATAAC------------
----

AATGAAACACCTATCACGCTCCCATGCAGAATAAAACAGTTTATAAACAGGTGGCAGGGAGTAGGAAGAGCAA
TGTATGCCCCTCCCATCGCGGGAAACATTAGCTGTTCATCAAAT---
ATTACAGGGCTACTATTAGCAAGAGATGGGGGGAAGAATGAGACC---------------------------
----

AAGTCTGAGACCTTCAGGCCTTGGGGAGGAAATATGAAGGACAATTGGAGAAGTGAATTATATAAATATAAAG
TAGTAAAAATTGAGCCATTA---GGGATAGCACCCACCGAGGCAAGAAGAAGAGTGGTG-------------
------------------------CAGAGAGAAAGAAGAGCA---GTGGGAACGATAGGAGCT---------
ATGTTCCTTGGGTTCTTGGGAACA---GCAGGAAGCACTATG---GGCGCAGCGTCA---
CTGACGCTGACGGTACAG---GCCAGACAA---TTGTTG---TCTGGTATAGTGCAACAGCAAAGT---
AATTTG---CTAAGA---GCTATTGAGGCGCAACAGCATATGTTGCAACTCACAGTCTGGGGCATT
>KT878037
TTCTACTGTAATACATCAAAACTGTTTAATAGTACTTGGGATGATAATAATACTAGTACTTGGGAAAATAGTA
CT--

GAAAATGACATTATCGAACTCCCATGTAGAATAAAACAAATTATAAACAGGTGGCAGGAAGTAGGAAAAGCAA
TGTTTGCCCCTCCCATAGCAGGATACATTAATTGCTCATCAAAT---
ATTACAGGGCTGTTATTAGTGAGAGATGGTGGTATTGTTAACAGT---------------------------
----

ACCAACGACACCTTCAGACCTGGAGGAGGAAATATGAAGGACAATTGGAGAAGTGAATTATATAAATATAAAG
TAGTAAAAATTGAACCATTA---GGAATAGCACCCACCAAGGCAAGGAGAAGAGTGGTG-------------
------------------------CAGAGAGAAAAAAGAGCA---GCGGGA---ATAGGAGCT---------
TTGCTCCTTGGGTTCTTGGGAGCA---GCAGGAAGCACTATG---GGCGCAGCGTCA---
ATGACGCTGACGGTACAG---GCCAGACAA---TTATTG---TCTGGTATAGTGCAACAGCAGAAC---
AATCTG---CTGAAG---GCCATTGAGGCGCAACAGCGTGTGTTGCAACTCACAGTCTGGGGCATC
>KT878038
TTCTACTGTAATACAACAAAACTGTTTAGTGGTACCTGGAATGGTACTTGGTATGGTACCTGGAATGGTACTT
GGGATGGT--
GCTACAGAAAATAGTACTAAT------------
AAAAATATTACAGATATCATAATCCCATGCAGAATAAAACAAATTATAAACAGATGGCAAGAAGTAGGAAAAG
CAATGTATGCCCCTCCTATCGCAGGACGAATCAGATGTTCATCAAAT---
ATTACAGGACTGTTACTAACAAGAGATGGGGGCACAAACACAAGCACC------------------------
----

AGCAATGAGACGGAGACCTTCAGACCTGGGGGAGGAGATATGAGAGACAATTGGAGAAGTGAATTATATAAAT
ATAAAGTAGTAAGAATTGAGCCACTG---GGGATAGCACCCACCAAGGCAAAGAGAATAGTGGTG--------
------------------------------CAGAGAGAAAAAAGAGCT---GTGGGAACATTAGGAGCT---
------ATGTTCCTTGGGTTCTTGGGAGCA---GCAGGAAGCACTATG---GGCGCAGCGTCA---
GTGACGCTGACGGTACAG---GCCAGACAA---TTATTG---TCTGGTATAGTGCAACAGCAGAAT---
AATTTG---CTGAGA---GCTATTGAGGCGCAACAGCATATGTTGCAACTCACAATCTGGGGCATC
>KT878022
TTCTACTGTAATACAACAAAACTGTTTAATAGTATCTGGAATGGTACTTCGAATGGTTTTACA---------
----

GGAAATGATACAATCGTACTCCCATGCAGAATAAAACAAATTATAAACAGATGGCAAGAAGTAGGAAAAGCAA
TGTATGCCCCTCCTATCGCAGGAAATATTAGCTGTTCATCAAAT---
ATTACAGGGCTGTTATTAACAAGAGATGGGGGGCAGGAGAATGGCACC------------------------
----

AATGGGACGGAGACCTTCAGACCTGGGGGAGGAGATATGAGGGACAATTGGAGAAGTGAATTATATAAATATA
AAGTAGTAAGAATTGAGCCATTA---GGGATAGCACCCACCAAGGCAAAGAGAAGAGTGGTG----------
---------------------------CAGAGAGAAAAAAGAGCA---GTGGGAACAATAGGAGCT------
---ATGTTCCTTGGGTTCTTGGGAGCA---GCAGGAAGCACTATG---GGCGCAGCGTCA---
GTGACGCTGACGGTACAG---GCCAGGCAA---TTATTG---TCTGGTATAGTGCAACAGCAGCAT---
AATTTG---CTAAGA---GCTATTGAGGCGCAACAGCATATGTTGCAACTCACAGTCTGGGGCATC

>KT878023
TTCTACTGTAATACAACATCATTGTTTAATAGTACTTGGACTACTAATGATACTAGGAATGATACTGAGAAT-
----

AATAATGGAACAATCACACTCCAATGCAAAATAAAACAGATTATAAACTTGTGGCAGGAAGTAGGAAAAGCAA
TGTATGCCCCTCCCATCAGTGGACAAATTAACTGTTCATCAAAC---
ATTACAGGGCTGATATTAACAAGAGATGGTGGTAATGACACAAAA----------------------------
----

GATAATGAGACCTTCAGACCTATAGGAGGGAATATGAAGGACAATTGGAGAAGTGAATTATATAAATATAAAA
TAGTACAACTTGAACCATTA---GGAGTAGCACCCACCAAGGCAAAGAGAAGAGTGGTG--------------
-------------------------CAGAGAGAAAAAAGAGCA---GTGGGA---CTAGGAGCT---------
ATGTTCCTTGGGTTCTTGGGAGCA---GCAGGAAGCACTATG---GGCGCAGCGTCA---
ATAACGCTGACGGTACAG---GCCAGACAA---TTATTG---TCTGGTATAGTGCAACAGCAAAAC---
AATTTG---CTGAGA---GCTATTGAGGCGCAACAGAATCTGTTGCAACTCACAGTCTGGGGCATT
>KT878024
TTCTACTGTGATACATCAAAATTGTTTAATAGTACTTGGAATAATACTGACACTTGGAATAATACTGACACTG
GGAATAGT---

ACTGAAACAAGAAATATCACACTCCCATGCCAAATAAAACAATTTATAAACAGGTGGCAGGAAGTAGGAAGAG
CAATGTATGCCCCTCCCATCGCAGGACTAATTAGATGTTCATCAAAC---
ATTACAGGGCTGCTATTAACAAGAGATGGGGGGAATAAAAATAATACA-------------------------
----

AGTAACGAGACCTTCAGGCCTGGGGGAGGAAATATGAAGGACAATTGGAGGAGTGAATTATATAAATATAAAG
TAGTAAAAATTGAGCCATTA---GGGATAGCACCCACCAAGGCAAGGAGAAGAGTGGTG--------------
-------------------------CAGAGAGAAAAAAGAGCA---ATAGGA---CTAGGAGCT---------
GTGTTCCTTGGGTTCTTGGGAGCA---GCAGGAAGCACTATG---GGCGCAGCGTCA---
CTGACGCTGACGGCACAG---GCCAGACAA---TTATTG---TCCGGTATAGTGCAACAGCAGAAT---
AATTTG---CTGAGA---GCTATTGAGGTGCAACAGCATATGTTGCAGCTCACAGTCTGGGGCATC
>KT878025
TTCTACTGTAATACAACAAAATTGTTTAAGAGTACTTGGAATAGTACTTGGAGTGATTGGAATAGTACTTGGA
GTGATAAT---
-------------------GAA---
GGAAAAGAACATAATATCATACTCCCATGCAGAATAAGACAAATTATAAACAGGTGGCAGGAAGTAGGAAAAG
CAATGTATGCCCCTCCCATCGAAGGACAAATTAGATGCTCATCAAAT---
ATTACAGGGCTGCTATTAACAAGAGATGGGGGGACAGAAGACAATAGG-------------------------
----

ACTAATGGGACTGAGACCTTCAGACCTGGGGGAGGAGATATGAGGGACAATTGGAGAAGTGAATTATATAAAT
ATAAAGTAGTAAAAATTGAGCCATTA---GGGATAGCACCCACCAAGGCAAAGAGAAGAGTGGTG--------
-------------------------------CAGAGAGAAAAAAGAGCA---GTAGGAACACTAGGAGCT---
------ATGTTCCTTGGGTTCTTGGGAGCA---GCAGGAAGCACTATG---GGCGCAGCGTCA---
ATGACGCTGACGGTACAG---GCCAGACTA---TTATTG---TCTGGTATAGTGCAACAGCAGAAT---
AATTTG---CTGAGA---GCTGTTGAGGCGCAACAGCATCTGTTGCAACTCACAGTCTGGGGCATT
>KT878027
TTCTACTGTAATACAACACAACTGTTTAACAGTACTTGGAATGACACAAACCTGTATAATGCAACAAAA----
----

CAGAATGGCACAATTACACTCCCATGCAGAATAAAACAAATTATAAACAGATGGCAGGAAGTAGGAAAAGCAA
TGTATGCCCCTCCCATCGCAGGACAAATTAGATGCTCATCAAAT---
ATTACAGGGCTGCTATTAACAAGAGATGGGGGGATTAATACCTCT----------------------------
----

ACGGAAACCTTCAGACCTGGGGGAGGAGATATGAGGGACAATTGGAGAAGTGAATTGTACAAATATAAGGTAG
TAAGAATAGAACCATTA---GGGATAGCACCCACCAAGGCAAGGAGAAGAGTGGTG-----------------
----------------------CAGAGAGAAAAAAGAGCA---GTGGGAACAATAGGAGCT---------
ATGTTCCTTGGGCTCTTGGGAGCA---GCAGGAAGCACTATG---GGCGCAGCGTCA---
ATGACGCTGACGGTACAG---GCCAGACTG---TTATTG---TCTGGTATAGTGCAACAGCAAAAT---
AATTTG---CTGAGA---GCTATTGAGGCGCAACAGCGTCTGTTGCAACTCACAGTCTGGGGCATC

>MK871374
TTCTACTGTGATACAACACCACTGTTTAATAGTACTTGGAATATTAATGGGACTAAAAGGTCAAATGAAACTG
CA---

GAAAATGGCACAATTACCCTCCACTGCAGAATAAGACAAATTGTAAACAGGTGGCAGGAAGTAGGAAAAGCAA
TGTATGCCCCTCCCATCAGCGGACAAATTAACTGTACATCAAAT---
ATTACAGGGCTGTTATTAACAAGAGATGGGGGCGGTAATCCCGCC----------------------------
----

AACAAAACTAATGACACCTTCAGGCCTGGGGGAGGAGATATGAGGGACAATTGGAGAAGTGAATTGTATAAAT
ATAAAGTAGTAGAAATTGAACCATTA---GGGATAGCACCCACCAGGGCAAGGAGAATAGTGGTG--------
-------------------------------CAGAGAGAAAAAAGACCA---GTGGGAACATTAGGAGCC---
------ATGTTCCTTGGGTTCTTGGGAGCA---GCAGGAAGCACTATG---GGCGCAGCGTCA---
CTGACGCTGACGGTACAG---GCCAAGAAA---GTATTG---TCTGGTATAGTGCGACAGCAGCAT---
AATTTG---CTGAGA---GCTATTGAGGCGCAACAGCGTCTGTTGCAACTCACAGTCTGGGGCATC
>MN043576
TTCTACTGTGATACAACACAACTGTTTAATAGTACTTGGAATAGTACTTGGAATAGTACTGAAGGGTCAAAAA
CTGAA--

AGAAATGACACACTTACCCTCCCATGCAGAATAAAACAAATTATAAACAGGTGGCAGGAAGTAGGAAAAGCAA
TGTATGCCCCTCCCATCAAAGGACAAATTAGATGTTCATCAAAT---
ATTACAGGGCTGCTATTAACAAGAGATGGGGGGACTGAGAACAAC----------------------------
----

AGCGAGACTGAGATCTTCAGACCTGGGGGAGGAAATTTGAGGGACAATTGGAGAAGTGAATTATATAAATATA
AAATAGTAAAAATTGAGCCATTA---GGGATAGCACCCACCAGGGCAAGGAGAAGAGTGGTG-----------
----------------------------CAGAGAGAAAAAAGAGCA---GTGGGAACGATAGGAGCT------
---ATGTTCCTTGGGTTCTTGGGAGCA---GCAGGAAGCACTATG---GGCGCAGCGTCA---
ATGACGCTGACGGTACAG---GCCAGACTA---TTATTG---TCTGGTATAGTGCAACAGCAGAAT---
AATTTG---CTGAGA---GCTATTGAGGCGCAACAGCATCTGTTGCAACTCACAGTCTGGGGCATC
>KU896118
TTCTACTGTGATACAGCACAACTGTTTAATAGTACTTGGAATGATACACAACTGTTTAATAGTACTGGGAATG
ATACTGAA---

GGGTTAAGTGAAAATATCACACTCCCATGCAGAATAAAACAAATTATAAACAGGTGGCAGGAAGTAGGAAAAG
CAATGTATGCCCCTCCCATCGCAGGACAAATTAGATGTTCATCAAAT---
ATTACAGGGCTGCTATTAACAAGAGATGGGGGGATTGAGAACAAC----------------------------
----
GGGACTAACAGGACT---
GAGATTGAGATCTTCAGACCTGGGGGAGGAGATATGAGGGACAATTGGAGAAGTGAATTATATAAATATAAAG
TAGTAAAAATTGATCCATTA---GGGATAGCACCCACCAGGGCAAGGAGAAGAGTGGTG--------------
-------------------------CAGAGAGAAAAAAGAGCA---GTG---ACGATAGGAGCT---------
ATGTTCCTTGGGTTCTTGGGAGCA---GCAGGAAGCACTATG---GGCGCAGCGTCA---
CTGACGCTGACGGTACAG---GCCAGACAA---TTATTG---TCTGGTATAGTGCAACAGCAGAAT---
AATTTG---CTGAGA---GCTATTGAGGCGCAACAGCATCTGTTGCAACTCACAGTCTGGGGCATC
>MN043598
TTCTACTGTGATACAGCACAACTGTTTAATAGTACTTGGAATGATACACAACTGTTTAATAGTACTGGGAATG
ATACTGAA---

GGGTTAAGTGAAAATATCACACTCCCATGCAGAATAAAACAAATTATAAACAGGTGGCAGGAAGTAGGAAAAG
CAATGTATGCCCCTCCCATCGCAGGACAAATTAGATGTTCATCAAAT---
ATTACAGGGCTGCTATTAACAAGAGATGGGGGGATTGAGAACAACGGGACTAAC-------------------
----

AGGACTGAGATTGAGATCTTCAGACCTGGGGGAGGAGATATGAGGGACAATTGGAGAAGTGAATTATATAAAT
ATAAAGTAGTAAAAATTGATCCATTA---GGGATAGCACCCACCAGGGCAAGGAGAAGAGTGGTG--------
-------------------------------CAGAGAGAAAAAAGAGCA---GTG---ACGATAGGAGCT---
------ATGTTCCTTGGGTTCTTGGGAGCA---GCAGGAAGCACTATG---GGCGCAGCGTCA---
CTGACGCTGACGGTACAG---GCCAGACAA---TTATTG---TCTGGTATAGTGCAACAGCAGAAT---
AATTTG---CTGAGA---GCTATTGAGGCGCAACAGCATCTGTTGCAACTCACAGTCTGGGGCATC

>MN043588
TTCTACTGTAATACAACACAATTGTTTAATAGTACTTGGAATGGGAATGATACTGTAGGGTCAAATAAA----
----

ACTGAAGGAAATATCACACTCCCATGCAGAATAAAACAAATTATAAACAGGTGGCAGGAAGTAGGAAAAGCAA
TGTATGCCCCTCCCATCAGAGGACAAATTAGATGTTCATCAAAT---
ATTACAGGGCTGCTATTAACAAGAGATGGGGGGAATGAAGGCAAC----------------------------
----

GAGACTGAGATCTTCAGACCTGGGGGAGGAGACATGAGGGACAATTGGAGAAGTGAATTATATAAATATAAAG
TAGTAAGAATTGAGCCATTA---GGGATAGCACCCACCAAGGCAAGGAGAAGAGTGGTG--------------
-------------------------CAGAGAGAAAAAAGAGCA---GTGGGAACGATAGGAGCT---------
ATGTTCCTTGGGTTCTTGGGAGCA---GCAGGAAGCACTATG---GGCGCAGCGTCA---
CTGACGCTGACGGTACAG---GCCAGACTA---TTATTG---TCTGGTATAGTGCAACAGCAGAAT---
AATTTG---CTGAGA---GCTATTGAGGCGCAACAGCGTCTGTTGCAACTCACAGTCTGGGGCATC
>KU869580
TTCTACTGTAATACAACACAACTGTTTAATAGTACTTGGAATGAGAATAGTACTGAATGGTCAAATACAACTG
AA---

GGAAATGACACAATTATCACACTCCCATGTAGAATAAAACAAATTATAAACAGGTGGCAGGAAGTAGGAAAAG
CAATGTATGCCCCTCCCATCAGAGGACAAATTAGATGTTCATCAAAT---
ATTACAGGGCTGCTATTAACAAGAGATGGAGGGACTGAGAAAAACAGG-------------------------
----
ACTGAGAACAACGGGACGACTGAGATCTTCAGGCCAACGGGAGGGAATATGAGGGACAATTGGAGAAGTGAAT
TATATAAATATAAAGTAGTAAAAATTGAGCCATTA---
GGGATAGCACCCACCAAGGCAAGGAGAAGAGTGGTG-------------------------------------
--CAGAGAGAAAAAAGAGCA---GTGGGAACGATAGGAGCT---------
ATGTTCCTTGGGTTCTTGGGAGCA---GCAGGAAGCACTATG---GGCGCAGCGTCA---
CTGACGCTGACGGTACAG---GCCAGACTA---CTATTG---TCTGGTATAGTGCACCAGCAGAAT---
AATTTG---CTGAGA---GCTATTGAGGCGCAACAGCATCTGTTGCAACTCACAGTCTGGGGCATC
>MN043589
TTCTACTGTAATACAACACAACTGTTTAATAGCACTTGGAATAGTACTGAAAGGTTCAATAACACTGAAGGA-
----

AATATCACAACTATCAGACTCCCATGCCGAATTATACACATTAGAAACGGGTGGCAAGAAGTAAGGAAAGCAA
TGTATGCCCCTCCCATCATAGGACGCACTACATGTTCTTCAGGC---
ATTACAGGGCTGCTATTAGCAAGAGATGGGGGGACTGAGAACAAC----------------------------
----

GGGACTGAAATCTTCGGACCTGGGGGAGGAGATATGAGGGATAATTGGAGAAGTGAATTATATAAATATAAAG
TAGTAAAAATTGAGCCATTA---GGGATACCACCCACCCAGGCAAAGAGAAGAGTGGTG--------------
-------------------------CAAAGAGAAAAAAGAGCA---GTGGGAACGATAGGGGCT---------
ATGTTCCTTGGGTTCTTGGGACCA---CCAGGAACCATTATG---GGCGCAGCTTCA---
ATGACGCTGACGGTACAG---GCCAGATTA---TTATTG---TCTGGTATAGTGCAACAGCAGAAT---
AATTTG---CTGAGA---GCTATTGAGGCGCAACAGCATCTGTTGCAACTCACAGTTTGGGGCATC
>KU869596
TTCTACTGTAATACAACACAACTGTTTAATAGTACTTGGACCGGGAATGGTACTGAAGGGTTAAATGAAACTG
GA---

GGAAATAACACACTTATCACACTCCCATGCAGAATAAAACAAATTATAAACAGGTGGCAGGAAGTAGGAAAAG
CAATGTATGCCCCTCCCATCAGCGGAGTAATTAGATGTTCATCAAAT---
ATTACAGGGCTACTATTAACAAGAGATGGGGGGATTGAGAACAAC----------------------------
----
GAGACTAATAGCAACAGGACTGAGATCTTCAGACCTGGGGGAGGAGATATGAGGGACAATTGGAGAAGTGAAT
TATATAAATATAAAGTAGTAAAAATTGAGCCATTA---
GGGATAGCACCCACCATGGCAAGGAGAAGAGTGGTG-------------------------------------
--CAGAGAGAAAAAAGAGCA---GTGGGACTGATAGGAGCT---------
ATGTTCCTTGGGTTCTTGGGAGCA---GCAGGAAGCACTATG---GGCGCAGCGTCA---
TTGACGCTGACGGTACAG---GCCAGACAA---TTATTG---TCTGGTATAGTGCAACAGCAGAAT---
AATTTG---CTGAGA---GCTATTGAGGCGCAACAGCATCTGTTGCAACTCACCGTCTGGGGCATC

>KX960971
TTCTACTGTGATACAACAAAACTGTTTAACAGTACTTGGAATAATACTAAAAGGGTAGAGGGAACTGAAGGA-
----

GAAAATGGCACACTTACACTCCCATGCAGAATAAAACAAATTATAAACAGATGGCAGGAAGTAGGAAAAGCAA
TGTATGCCCCTCCCATCAGAGGACAAATTAGATGTTCATCAAAT---
ATTACAGGGCTGTTATTAACAAGAGATGGGGGTGAGAGGAATGACACC-------------------------
----

AACGAGACTGAAACCTTCAGACCTGGGGGAGGAGATATGAGGGACAATTGGAGAAGTGAATTATATAAATATA
AAGTAGTAAAAATTGAGCCATTA---GGGATAGCACCCACCAAGGCAAGGAGAAGAGTGGTG-----------
----------------------------CAGAGAGAAAAAAGAGCA---GTGGGAACAATAGGAGCT------
---ATGTTCCTTGGGTTCTTGGGAGCA---GCAGGAAGCACTATG---GGCGCAGCGTCA---
CTGACGTTGACGGTACAG---GCCAGACTA---TTATTG---TCTGGTATAGTGCAACAGCAGAAT---
AATTTG---CTGAGA---GCTATTGAGGCGCAACAGCATCTGTTGCAACTCACAGTCTGGGGCATC
>KJ140267
TTCTACTGCAATACAACACAACTGTTTAATAGTACTTGGTTTAATAGTACTTGGAATGATACT----------
----

GAAGGGTCAAATATCACTGGTACTGTAGGGTCAAATATCACACTCCCATGCAGAATAAAACAAATTATAAACA
GGTGGCAGGAAGTAGGAAAAGCAATGTATGCCCCTCCCATCAGAGGACAAATTAGATGTTCATCAAAT---
ATTACAGGACTGCTATTAACAAGAGATGGGGGG--

AGTGGGGACAACGAGACTAACGGGACGACTGAGACCTTCAGACCTGGGGGAGGAGATATGAGGGACAATTGGA
GAAGTGAATTATATAAATATAAAGTAGTAAAAATTGAGCCATTA---
GGGATAGCACCCACCAAGGCAAAGAGAAGAGTGGTG-------------------------------------
--CAGAGAGAAAAAAGAGCA---GTGGGAACGATAGGAGCT---------
ATGTTCCTTGGGTTCTTGGGAGCA---GCAGGAAGCACTATG---GGCGCAGCGTCA---
CTGACGCTGACGGTACAG---GCCAGACTA---CTATTG---TCTGGTATAGTGCAACAGCAGAAT---
AATTTG---CTGAGA---GCTATTGAGGCGCAACAGCATCTGTTGCAACTCACAGTCTGGGGCATC
>KJ140248
TTCTACTGCAATACAACACGACTGTTTAATAGTACTTGGAATATTAATAGTACTTGGAATGATACTGAA----
----

AGGTCAAATAAAACTGCAAATGACACACTTACACTCCCATGCAGAATAAAACAAATTATAAACATGTGGCAGG
AAGTAGGAAAAGCAATGTATGCCCCTCCCATCAGTGGACAAATTAGATGTTCATCAAAT---
ATTACAGGGCTGCTATTAACAAGAGATGGGGGGGCTAAGAACAACGGGACT----------------------
----

GAGAACAACGGGACTGAGATCTTCAGACCTGGGGGAGGAGATATGAAGGACAATTGGAGAAGTGAATTATATA
AGTATAAAGTAGTAAAAATTGAGCCATTA---GGGATAGCACCCACCAAGGCAAGGAGAAGAGTGGTG-----
----------------------------------CAGAGAGAAAAAAGAGCA---
GTGGGAACGATAGGAGCT---------ATGTTCCTTGGGTTCTTGGGAGCA---GCAGGAAGCACTATG---
GGCGCAACGTCA---ATGACGCTAACAGTACAG---GCCGGACTA---TTATTG---
TCTGGTATAGTGCAACAGCAGAAT---AATTTG---CTGAGA---
GCTATTGAGGCGCAACAGCATCTGTTGCAACTCACAGTCTGGGGCATC
>KJ140252
TTCTACTGTAATACAACACAACTGTTTAATAGCACTTGGAATAGTACTGAAGGGTTTAATAGTAGTACTGAAG
GG---
----------------TTA------------
AATAAAACTATCACACTCCCATGCAGAATAAAACAAATTATAAACAGATGGCAGGAAGTAGGAAAAGCAATGT
ATGCCCCTCCCATCAGAGGACAAATTAGATGTTCATCAAAT---
ATTACAGGGCTGCTATTAACAAGAGATGGGGGG--

-
AATGAGAGCAACGAGGCTGAGATCTTCAGACCTGGGGGAGGAAATATGAGGGACAATTGGAGAAGTGAATTAT
ATAAATATAAAGTAGTAAAAATTGAGCCATTA---GGGATAGCACCCACCCAGGCAAGGAGAAGAGTGGTG--
-------------------------------------CAGAGAGAAAAAAGAGCA---
GTGGGAACGATAGGAGCT---------ATGTTCCTTGGGTTCTTGGGAGCA---GCAGGAAGCACTATG---
GGCGCAGCGTCG---ATGACGCTGACGGTACAG---GCCAGACTA---TTATTG---

TCTGGTATAGTGCAACAGCAGAAT---AATCTG---CTAAGA---
GCTATTGAGGCGCAACAGCATCTGTTGCAACTCACAGTCTGGGGCATC
>AJ417425
TTCTACTGTAATACAACACAACTGTTTAATAGTACTTGGAATGGGAACAGCACTTGGAATAAGACTGATAAG-
----

GCAAATGACACCATCACACTCCCATGCAGAATGAAGCAAGTCGTAAACATGTGGCAGGAGGTAGGAAAAGCAA
TGTATGCCCCTCCCATCAGAGGGCAAATTAGCTGTTCATCAAAT---
ATTACAGGGCTGCTACTAACAAGAGATGGTGGTAATACCACGAAT----------------------------
----

GATACCGAGACCTTCAGACCTGGAGGAGGGGATATGAGGGACAATTGGAGAAGTGAATTATATAAATATAAAG
TAGTAAAAATTGAACCATTA---GGAGTAGCACCCACCAAGGCAAAAAGGAGAGTGGTG--------------
-------------------------CAAAGAGAAAAAAGAGCA---GTGGGA---ATAGGAGCT---------
TTGTTCCTTGGGTTCTTGGGAGCA---GCAGGAAGCACTATG---GGCGCAGCGTCA---
GTGACGCTGACGGTACAG---GCCAGACAA---TTATTG---TCTGGTATAGTGCAACAGCGGAGT---
AATTTG---CTGATG---GCTATTGAGGCGCAACAACATCTATTGCAACTCACGGTCTGGGGCATC
>KY820529
TTCTACTGTAATACAACACAACTGTTTAATAGTACTTGGAAATGGGATAATAGTACTTGGAATAGAACTGAAG
GGTTGAAT---

ATAACTGGAAGAAATATCACACTCCCATGCAGCATAAAACAAATTATAAACAGGTGGCAGGAAGTAGGAAAAG
CAATGTATGCCCCTCCCATCAGTGGACAAATTAGATGTTCATCAAAT---
ATTACAGGGCTGCTACTAACAAGAGATGGGGGGGCTGGGAACAAC----------------------------
----

GGGACTAACGGGACTGAGATCTTCAGACCTGGGGGAGGAGATATGAGGGACAATTGGAGAAGTGAATTATATA
AATATAAAGTAGTAAAAATTGAGCCATTA---GGGATAGCACCCACCAGGGCAAAGAGAAGAGTGGTG-----
----------------------------------CAGAGAGAAAAAAGAGCA---
GTGGGAACGATAGGAGCT---------ATGTTCCTTGGGTTCTTGGGAGCA---GCAGGAAGCACTATG---
GGCGCAGCGTCA---CTGACGCTGACGGTACAG---GCCAGACTA---TTATTG---
TCTGGTATAGTGCAACAGCAGAAT---AATTTG---CTGAGA---
GCTATTGAGGCGCAACAGCATCTGTTGCAACTCACAGTCTGGGGCATC
>MK577479
TTCTACTGTAATACAACACAACTGTTTAATAGTACTTGGAATGAGAATAGTACTGAATGGTCAAATACAACTG
AAGGA--

AATGACACAATTATCACACTCCCATGTAGAATAAAACAAATTATAAACAGGTGGCAGGAAGTAGGAAAAGCAA
TGTATGCCCCTCCCATCAGAGGACAAATTAGATGTTCATCAAAT---
ATTACAGGGCTGCTATTAACAAGAGATGGAGGGACTGAGAAAAACAGGACTGAG-------------------
----

AACAACGGGACGACTGAGATCTTCAGGCCAACGGGAGGGAATATGAGGGACAATTGGAGAAGTGAATTATATA
AATATAAAGTAGTAAAAATTGAGCCATTA---GGGATAGCACCCACCAAGGCAAGGAGAAGAGTGGTG-----
----------------------------------CAGAGAGAAAAAAGAGCA---
GTGGGAACGATAGGAGCT---------ATGTTCCTTGGGTTCTTGGGAGCA---GCAGGAAGCACTATG---
GGCGCAGCGTCA---ATGACGCTGACGGTACAG---GCCAGACGA---TTATTG---
TCTGGTATAGTGCAACAGCAGAAT---AATTTG---CTGAGA---
GCTATTGAGGCGCAACAGCATCTGTTGCAACTCACAGTCTGGGGCATC
>KU869586
TTCTACTGTAATACAACACAACTGTTTAATAGCACTTGGAATAGTACTGAAAGGTTCAATAACACTGAAGGA-
----

AATATCACAACTATCAGACTCCCATGCCGAATTATACACATTAGAAACGGGTGGCAAGAAGTAAGGAAAGCAA
TGTATGCCCCTCCCATCATAGGACGCACTACATGTTCTTCAGGC---
ATTACAGGGCTGCTATTAGCAAGAGATGGGGGGACTGAGAACAAC----------------------------
----

GGGACTGAAATCTTCGGACCTGGGGGAGGAGATATGAGGGATAATTGGAGAAGTGAATTATATAAATATAAAG
TAGTAAAAATTGAGCCATTA---GGGATACCACCCACCCAGGCAAAGAGAAGAGTGGTG--------------

-------------------------CAAAGAGAAAAAAGAGCA---GTGGGAACGATAGGGGCT---------
ATGTTCCTTGGGTTCTTGGGACCA---CCAGGAACCATTATG---GGCGCAGCTTCA---
ATGACGCTGACGGTACAG---GCCAGATTA---TTATTG---TCTGGTATAGTGCAACAGCAGAAT---
AATTTG---CTGAGA---GCTATTGAGGCGCAACAGCATCTGTTGCAACTCACAGTTTGGGGCATC
>MN043595
TTCTACTGTAATACAACACAACTGTTTAATAGTACTTGGACCGGGAATGGTACTGAAGGGTTAAATGAAACTG
GAGGA--

AATAACACACTTATCACACTCCCATGCAGAATAAAACAAATTATAAACAGGTGGCAGGAAGTAGGAAAAGCAA
TGTATGCCCCTCCCATCAGCGGAGTAATTAGATGTTCATCAAAT---
ATTACAGGGCTACTATTAACAAGAGATGGGGGGATTGAGAACAACGAGACT----------------------
----

AATAGCAACAGGACTGAGATCTTCAGACCTGGGGGAGGAGATATGAGGGACAATTGGAGAAGTGAATTATATA
AATATAAAGTAGTAAAAATTGAGCCATTA---GGGATAGCACCCACCATGGCAAGGAGAAGAGTGGTG-----
----------------------------------CAGAGAGAAAAAAGAGCA---
GTGGGACTGATAGGAGCT---------ATGTTCCTTGGGTTCTTGGGAGCA---GCAGGAAGCACTATG---
GGCGCAGCGTCA---TTGACGCTGACGGTACAG---GCCAGACAA---TTATTG---
TCTGGTATAGTGCAACAGCAGAAT---AATTTG---CTGAGA---
GCTATTGAGGCGCAACAGCATCTGTTGCAACTCACCGTCTGGGGCATC
>KX960978
TTCTACTGTAATACAACACAACTGTTTAATAGTACTTGGAATAGTAATAGTACTGAAGGGCCAAATAGAACTG
AA---

GGAAATAGCACACTTACCCTCCCATGCAGAATAAAACAAATTATAAACAGGTGGCAGGAAGTAGGAAAAGCAA
TGTATGCCCCTCCCATCAGTGGACAAATTAGATGCTCATCAAAT---
ATTACAGGGCTGCTATTAACAAGAGATGGGGGGAATGGGACCAAC----------------------------
----

GAGACTGAAACCTTCAGACCTGGGGGAGGAGATATGAGGGACAATTGGAGAAGTGAATTATATAAATATAAAG
TAGTAAAAATTGAGCCATTA---GGGATAGCACCCACCGGGGCAAGTAGAAGGGTGGTG--------------
-------------------------CAGAGAGAAAAAAGAGCA---GTGGGAACCATAGGAGCT---------
GTGTTCCTTGGGTTCTTGGGAGCA---GCAGGAAGCACTATG---GGCGCAGCGGCA---
GTGACGCTGACGGTACAG---GCCAGACTA---TTATTG---TCTGGTATAGTGCAACAGCAGAAT---
AATTTG---CTGAGA---GCTATTGAGGCGCAACAGCATCTGTTGCAACTCACAGTCTGGGGCATC
>AJ417415
TTCTACTGTAATACAACAAACCTGTTTAATGGTACTTGGAATGGTACTGCTTGGAATGGTACTTGGAATGTTA
GT---

AAAACTGATAAAAATATCACACTCTCATGCAGAATAAAACAAATTATAAACAGGTGGCAGGAAGTAGGAAAAG
CAATGTATGCCCCTCCCATCGCAGGAAAAATTAGCTGTTCATCTAAT---
ATTACAGGGCTGTTATTAACAAGAGATGGGGGGAATGAGACCAAC----------------------------
----

GGGACCCTGGAGATCTTCAGACCTGGGGGAGGAGATATGAGGGACAATTGGAGAAGTGAATTATATAAATATA
AAGTAGTAAAAATTGAGCCATTA---GGGATAGCACCCACCAAGGCAAGGAGAAGAGTGGTG-----------
----------------------------CAGAGAGGAAAAAGAGCA---GTGGGAACGATAGGAGCT------
---ATGTTCCTTGGGTTCTTGGGAGCA---GCAGGAAGCACTATG---GGCGCAGCGTCA---
TTGACGCTGACGGTACAG---GCCAGACTA---TTATCG---TCTGGTATAGTGCAACAGCAGAGT---
AATTTG---CTGAGA---GCTATTGAGGCGCAACAGCATCTGTTGCAACTCACAGTCTGGGGCATT
>AJ417431
TTCTACTGTAATACATCAAAACTGTTTAATAGTACTTGGGGTCTTAACCAGACAGCTAACCATGAAGGA----
----

AATGATACAACAATCACACTCCCATGCAGAATAAAACAAATTATAAACATGTGGCAGAAAGTAGGAAAAGCCA
TGTATGCCCCTCCCATTGCGGGACGGATTGCATGTTCATCACAT---
ATTACAGGGCTACTATTAACAAGAGATGGGGGTAATGACACTAAC----------------------------
----

AATGAGACCTTCAGACCTGGAGGAGGAAATATGAGGGACAATTGGAGGAGTGAATTATATAAATATAAAGTAG

TAAAAATTAAACCATTA---GGAATAGCACCCACCAAGGCAAAGAGAAGAGTGGTG-----------------
----------------------CAGAGAGAAAAAAGAGCA---GTGGGAACACTGGGAGCT---------
ATGTTCCTTGGGTTCTTGGGAGCA---GCAGGAAGCACTATG---GGCGCAGCGTCA---
GTGACGCTGACGGTACAG---GCCAGACAA---TTATTG---TCTGGTATAGTGCAACAGCAGAAC---
AACTTG---CTGAGG---GCTATTGAGGCGCAACAGCATCTGTTGCAACTCACGGTCTGGGGCATC
>AJ417410
TTCTACTGTAATACAACACAACTGTTTAATAGTACTTGGAATATTACTGAAGGGTCAAATACAACTGAAAGAA
AT---

AATAATACACATATCACACTCCAATGCAGAATAAGACAAATTATAAACAGATGGCAGGAAGTAGGAAAAGCAA
TGTATGCCCCTCCCATCAGAGGACAAATTAGATGTTCATCAAAT---
ATTACAGGGCTGCTATTAACAAGAGATGGGGGGATTGAGAACAACGAG-------------------------
----

ACCAAGACTATTGAGATCTTCAGACCTGGGGGAGGAAACATGAAGGACAATTGGAGAAGTGAATTATATAAAT
ATAAAGTAGTAAAAATTGAGCCATTA---GGGATAGCACCCACCAAGGCAAGGAGAAGAGTGGTG--------
-------------------------------CAGAGAGAAAAAAGAGCA---GTGGGAACGATAGGAGCT---
------ATGTTCCTTGGATTCTTGGGAGCA---GCAGGAAGCACTATG---GGCGCAGCGTCA---
ATGACGCTGACGGTACAG---GCCAGACTA---TTATTG---TCTGGTATAGTGCAACAGCAGAAT---
AATTTG---CTGAGA---GCTATTGAGGCGCAACAGCATATGTTGCAACTCACAGTCTGGGGCATC
>AJ417413
TTCTACTGTAATACAACACAACTGTTTAATAGTACTTGGAATAGTAATAGTACTTGGAATAGTACTGAA----
----

AGAAATGACACAATTACCCTCCCATGCAGAATAAAACAAATTATAAACAGGTGGCAGGAAGTAGGAAAAGCAA
TGTATGCCCCTCCCATCAGAGGACAAATTAGATGCTCATCAAAT---
ATTACAGGGTTGTTATTAACAAGAGATGGGGGAACTGGGAACAACGAG-------------------------
----

ACCAACGAGACTGAGATCTTCAGACCTGGGGGAGGAGACATGAGGGACAATTGGAGAAGTGAATTATATAAAT
ATAAAGTAGTAAAAATTGAACCATTA---GGGATAGCACCCACCAAGGCAAGGAGAATAGTGGTG--------
-------------------------------CAGAGAGAAAAAAGAGCA---GTGGGAACGCTAGGAGCC---
------ATGTTCCTTGGGTTCTTGGGAGCA---GCAGGAAGCACTATG---GGCGCAGCGTCA---
CTGACGCTGACGGTACAG---GCCAGACTA---TTATTG---TCTGGTATAGTGCAACAGCAGAAT---
AATTTG---CTGAGA---GCTATTGAGGCGCAACAGCATCTGTTGCAACTCACAGTCTGGGGCATC
>AJ417419
TTCTACTGTAATACAACAAAACTGTTTAATAGTACTTGGAATGAAACAAGACTTAGTAATTGGAATAGTACTG
AA---

AAGTTAAATGAAAATATCACACTCCAATGCAGAATAAAACAAATTATAAACAGGTGGCAGGAAGTAGGAAAAG
CAATGTATGCCCCTCCCATCGAAGGAATAATTAGATGTTCATCAAAT---
ATTACAGGGCTGCTATTAACAAGAGATGGGGGAACTGGGAACAACGAGACT----------------------
----

GGCAACGAGACTGAGATCTTCAGACCTGGGGGAGGAGACATGAGGGACAATTGGAGAAGTGAATTATATAAAT
ATAAAGTAGTAAGAATTGAGCCATTA---GGGCTAGCACCCACCAAGGCAAGGAGAAGAGTGGTG--------
-------------------------------CAGAGAGAAAAAAGAGCA---GTGGGAGCAATAGGAGCT---
------ATGTTCCTTGGGCTCTTGGGAGCA---GCAGGAAGCACTATG---GGCGCAGCGTCA---
CTGACGCTGACGGTACAG---GCCAGACTA---TTATTG---TCTGGTATAGTGCAACAGCAGAAT---
AATTTG---CTGAGA---GCTATTGAGGCGCAACAGCGTTTGTTGCAACTCACAGTCTGGGGCATC
>AF224507
TTCTACTGTAATACAACACAACTGTCTAATAGTACTTGGCAGAGGTCTGATGGTACTTGGAATAGAACTGGAG
GGTTA--
-------------------AAT---
GAAACTAAAGAAAATATCACACTCCCATGCAGAATAAAACAAATTATAAACAGGTGGCAGGAAGTAGGAAAAG
CAATGTATGCCCCTCCCATCAGTGGACTAATTAGATGTTCATCAAAT---
ATTACAGGGCTGCTATTAACAAGAGATGGGGGGAATGAGAACAATGGG-------------------------
----

ACCAATGGGACTGAGACCTTCAGACCTGAGGGAGGAAATATGAAGGACAATTGGAGAAGTAAATTATATAAAT

```
ATAAAGTAGTAAGAATTGAGCCATTA---GGGATAGCACCCACCAGGGCAAGGAGAAGAGTGGTG--------
-------------------------------CAGAGAGAAAAAAGAGCA---GTAACA---TTCGGAGCT---
------CTGTTCCTTGGGTTCTTGGGAGCA---GCAGGAAGCACTATG---GGCTGCACGTCA---
ATGACGCTGACGGTACAG---GCCAGACTA---TTATTG---TCTGGTATAGTGCAACAGCAGAAT---
AATTTG---CTGAGA---GCTATTGAGGCGCAACAGCATCTGTTGCAACTCACAGTCTGGGGCATC
>AB097870
TTCTACTGTAATACATCACAACTGTTTAATAGTACTTGGTATGGGAATAATACTAGTACTTGGAATAAT----
-------------------------------------------------------------------------
----------------------------
ACTGCAGAAAATATCACGCTCCCCTGCAGAATAAAACAAATTGTAAACATGTGGCAGGAAGTAGGAAGAGCAA
TGTATGCCCCTCCCATCGCAGGACAAATTAAGTGTTCATCAAAT---
ATTACAGGGTTGCTGTTAACAAGAGATGGTGGTAATAAGACCAAC----------------------------
-------------------------------------------------------------------------
-------------
CCCACCGAGATCTTCAGACCTGCAGGAGGAGATATGAGGGACAATTGGAGAAGTGAATTATATAAATATAAAG
TAGTAAGAATTGAACCATTA---GGAGTAGCACCCACCAGGGCAAAGAGAAGAGTGGTG--------------
-------------------------CAAAGAGAAAAAAGAGCA---GTGGGAACGCTAGGAGCT---------
ATGTTCCTTGGGTTCTTGGGAGCA---GCAGGAAGCACTATG---GGCGCAGCGTCA---
ATAACGCTGACGGTACAG---GCCAGACAA---TTATTG---TCTGGTATAGTGCAACAGCAAAGA---
AATTTG---CTGAGA---GCTATTGAGGCGCAACAGCATTTGTTGCAACTCACAGTCTGGGGCATC
>AY423386
TTCTACTGTAATACATCACAGCTGTTTAATAGTACTTGGGATGTGAATGCTACTGGGAATGGTACTACAGAA-
-------------------------------------------------------------------------
----------------------------
CCAAATAGTACTATAACACTCCCATGCAGAATAAAACAAATTATAAACAGATGGCAGGAAGTAGGAAAAGCAA
TGTATGCCCCTCCCATCGAAGGACAAATTAGCTGTTCATCAAAC---
ATCACAGGGCTGCTATTAACAAGGGATGGTGGTAGGAATAGGAACGAG-------------------------
-------------------------------------------------------------------------
-------
AGCACCGAGATCGAGATCTTTAGACCTGGAGGAGGAGATATGAGAGACAATTGGAGAAGTGAATTATATAAAT
ATAAAGTAGTAAAAATTGAGCCATTA---GGAGTAGCACCCACCAAGGCAAAGAGAAGAGTGGTG--------
-------------------------------CAGAGAGAAAAAAGAGCA---ATAGCG---CTAGGAGCT---
------ATGTTCCTTGGGTTCTTGGGAGCA---GCAGGAAGCACTATG---GGCGCAGCGTCA---
ATGGCGCTGACGGTACAG---GCCAGACAA---TTATTG---TCTGGTATAGTGCAACAGCAAAAC---
AATTTG---CTGAGG---GCTATTGAGGCGCAACAGCATCTGTTGCAACTCACAGTCTGGGGCATT
>HQ386146
TTCTACTGTAATACAACACAACTGTTTAATAGTACTTGGGAAAAGGATACTAAAGGGTCAAATGAC-------
-------------------------------------------------------------------------
----------------------------
ACTGAACGCATAATCCAACTTCCATGCAGAATAAAACAAATTATAAATATGTGGCAGGAAGTAGGAAAAGCAA
TGTATGCCCCTCCCATCAAAGGACAAATTAGATGTTCATCAAAT---
ATTACAGGGCTGATATTAACAAGAGATGGTGGTAATAGTAGTAATACCACGAGC-------------------
-------------------------------------------------------------------------
----
AATTCTAACAAAAATGAGACCTTCAGACCTGGAGGAGGAAACATGAAGGATAATTGGAGAAGTGAACTATATA
AATATAAAGTAGTACAAATTGAACCATTA---GGAGTAGCACCCACCAAGGCAAAGAGAAGAGTGGTG-----
----------------------------------CAAAGAGAAAAAAGAGCA---GTGGGA---
TTCGGAGCT---------CTGTTCCTTGGGTTCTTGGGAGCA---GCAGGAAGCACTATG---
GGCGCAGCATCA---ATAACGCTGACGGTACAG---GCCAGACAA---TTACTG---
TCTGAAATAGTGCAACAGCAGAAC---AATCTG---CTGAAG---
GCTATTGAGGCGCAACAGCATCTGTTGCAACTCACAGTCTGGGGCATT
>HQ386218
TTCTACTGTAATACAACAAAACTGTTCAATAGTACTTGGTGGTCTAATGGTACTTCGATTGGTACAAATAAC-
-------------------------------------------------------------------------
----------------------------
TCTAACGAAAATATCACTCTCCCATGCAGAATAAGACAAATTATAAACAGGTGGCAGGAAGTAGGAAAAGCAA
TGTATGCCCCTCCCATTGGAGGACTAATTAGATGTTCATCAAAT---
ATTACAGGGATGATATTAACAAGAGATGGGGGTAATAATAATACAACA-------------------------
-------------------------------------------------------------------------
----------
```

AATGAGACAGAGACCTTCAGACCTGGAGGAGGAGATATGAGGGATAATTGGAGAAGTGAATTATATAAATATA
AAGTAGTAAAAATTGAACCATTA---GGAGTAGCACCCACCAAGGCAAAGAGAAGAGTGGTG-----------
-----------------------------CAGAGAGAAAAAAGAGCA---GTGGGA---ATAGGAGCT------
---TTGTTCCTTGGGTTCTTGGGAGCA---GCAGGAAGCACTATG---GGCGCAGCGTCA---
TTGACGCTGACGGTACAG---GCCAGACAA---TTATTG---TCTGGTATAGTGCAACAGCAGAAC---
GATCTG---CTGAGG---GCTATTGAGGCGCAACAGCATATATTGCAACTCACAGTCTGGGGCATC
>U34603
TTCTACTGTGATACAACACAACTGTTTAATAGTACTTGGAATGGTACTGAAGGAAATAACACTGAA-------
----

GGAAATAGCACAATCACACTCCCATGCAGAATAAAACAAATTATAAACATGTGGCAGGGAGTAGGAAAAGCAA
TGTATGCCCCTCCCATCAGTGGACAAATTAGATGTTCATCAAAT---
ATTACAGGGCTGCTATTAACAAGAGATGGTGGTAAGGACGAGAACAAG-------------------------
----

ACTGGGACTGAGATCTTCAGACCTGGAGGAGGAGATATGAAGGATAATTGGAGAAGTGAATTATATAAATATA
AAGTAGTAAAAATTGAACCATTA---GGAGTAGCACCCACCAGGGCAAAGAGAAGAGTGGTG-----------
-----------------------------CAGAGAGAAAAAAGAGCA---GTGGGA---ATAGGAGCG------
---TTGTTCCTTGGGTTCTTGGCAGCA---GCAGGAAGCACTATG---GGCGCACGGTCA---
ATAACGCTGACGGTACAG---GCCAGACAA---TTATTG---TCTGGTATAGTGCAACAGCAAAAC---
AATTTG---CTGAGG---GCTATTGAGGCGCAACAGCATCTGTTGCAACTCACAGTCTGGGGCATC
>AY970946
TTCTACTGTAATTCAACACAACTGTTTAATAGCACTTGGAAAGATACTGGAAAGTTAAATAACCCTGAA----
----

GGAAATGGCAACATCACACTCCCATGTAAAATAAAACAAATTATAAACAGGTGGCAGGGAGTAGGAAAAGCAA
TGTATGCCCCTCCCATCAGAGGACAAATTAGATGTTCATCAAAT---
ATTACAGGGCTGCTATTAACAAGAGATGGTGGTAACAACGTGAAT----------------------------
----
----------AATACCACCGAGGTCTTCAGACCTGGAGGAGGAGATATGAGG---------------
GAATTATATAAATATAAAGTAGTAAAAATTGAACCATTA---
GGAGTAGCACCTACCAAGGCAAAGAGAAGAGTGGTG-------------------------------------
--CAGAGAGAAAAAAGAGCA---GTGGGAACGATAGGAGCT---------
ATGTTCCTTGGATTCTTGGGAGCA---GCAGGAAGCACTATG---GGCGCAGCGTCA---
GTGGCGCTGACGGTACAG---GCCAGACAA---TTATTG---TCCGGTATAGTGCAACAGCAGAAT---
AATTTG---CTGAAA---GCTATTGAGGCGCAACAACATCTGTTGCAACTCACAGTCTGGGGCATC
>GU455480
TTCTACTGTAATTCAACAAAACTGTTTAATAGTACTTGGAATTTGAATGGTACTTGGCATGGTACTGAAAGGC
CAAATAGC--
-------------------ACT---
GAAAGAAATGACACACTCACAATCCCATGCAGAATAAAACAAATTATAAACATGTGGCAGGAAGTAGGAAAAG
CAATGTATGCCCCTCCCATCAAAGGACAAATTAGCTGTGTATCAAAT---
ATTACAGGGTTGCTATTAACAAGAGATGGTGGTACCAGCAAGAACGGGACC----------------------
----

ACCAACGACACCGAGGTCTTCAGACCTGGAGGAGGAAATATGAAGGACAATTGGAGAAGTGAATTATACAAAT
ATAAAGTAGTAAAAATTGAACCATTA---GGGGTAGCACCCACCAGGGCAAAGAGAAGAGTGGTG--------
---------------------------------CAGAGAGAAAAAAGAGGA---GTGGGA---
TTAGGAGTGCTGTATGGATTAGGAGCTTTGTTCCTTGGGTTCTTGGGAGCAGCAGGAAGCACTATGGGCGCAG
CGTCAATGACGCTGACGGTACAG---GCCAGACTA---TTATTG---TCTGGTATAGTGCAACAGCAGAAC--
-AATCTG---CTGAGG---GCTATTGAGACGCAACAGCATCTGTTGCAACTCACAGTCTGGGGCATC
>GU455465
TTCTACTGCAATACAACACAGCTGTTTAATAGTACTTGGCTGCTTAATAGTACTTGGAATAGTACTGACGAGT
CATCT---

AACAAGGAAATTAATATCACACTCCCATGCAGAATAAAACAAATTATAAACAGGTGGCAGGAAGTAGGAAAAG
CAATGTATGCCCCTCCCATCCAAGGACTAATTAAATGTTCATCAAAT---
ATTACAGGGCTGTTATTAACAAGAGATGGTGGTAATGGGACCGAG----------------------------
----

AATGAGACCGAGACCTTCAGACCTGGGGGAGGAGATATGAGGGACAATTGGAGAAGTGAATTATATAAATATA
AAGTAGTTAAAATTGAGCCATTA---GGAGTAGCACCCACCAAGGCAAAGAGAAGAGTGGTG----------
----------------------------CGAAGAGAAAAAAGAGCA---GTGGGAGTGATAGGAGCT------
---ATGTTCCTTGGGTTCTTGGGAGCA---GCAGGAAGCGCTATG---GGCGCAGCGTCA---
GTGACGCTGACGGTACAG---GCCAGACAA---TTATTG---TCTGGTATAGTGCAACAGCAGAAC---
AATCTG---CTGAGA---GCTATTGAGGCGCAACAGCATCTGTTGCAACTCACAGTCTGGGGCATC
>EU744159
TTCTACTGTAATTCAGCACAACTGTTTAATAGTACTTGGGAGGTTAATAGAACGAAAGAAAAT---------
----

GACAATGGCACAATCACACTCCCATGCAGAATAAAACAGATTATAAACATGTGGCAGGAAGTAGGAAAAGCAA
TGTATGCCCCTCCCATCAGCGGACAAATTAGATGTTCATCAAAT---
ATTACAGGGCTGCTATTAACAAGAGATGGTGGTAACGAGACTAATGGG------------------------
----

ACTGAGACTGAGACCTTCAGACCTGGAGGAGGAAATATGAGGGACAATTGGAGAAGTGAATTATATAAATATA
AAGTAGTAAAAATTGAACCATTA---GGAGTAGCACCCACCAAGGCAAAGAGAAGAGTGGTG----------
----------------------------CAAAGAGAAAAAAGAGCA---GTGGGA---ATAGGAGCT------
---GTGTTCCTTGGGTTCTTAGGAGCA---GCAGGAAGTACTATG---GGCGCAGCGTCA---
ATAACGCTGACGGTACAG---GCCAGACTA---TTATTG---TCTGGTATAGTACAACAGCAGAAC---
AATCTG---CTGAGG---GCTATTGAGGCGCAACAACATCTGTTGCAACTCACAGTCTGGGGCATC
>GU455505
TTCTACTGTAATACAACAAAACTGTTTAGTAGTACTTGGAATTTTACTAACGGGTCATGGTCATCTAACAATG
CGGAAAAT--
-------------------ATC---
ACACTCTCAGAAAATACCACACTCCCATGCAGAATAAAACAAATTATAAACATGTGGCAGGAAGTAGGAAAGG
CAATGTATGCCCCTCCCATCAGAGGACGAATTAGATGTTCATCAAAT---
ATTACAGGGCTGCTATTAACAAGAGATGGTGGTAGGGACAATGAAACA------------------------
----

AACGGGACCGAGATATTCAGACCTGGAGGAGGAAATATGAAGGACAATTGGAGAAGTGAATTATATAAATATA
AAGTAGTAAAAATTGAACCATTA---GGAGTAGCACCCACCAAGGCAAAGAGAAGAGTGGTG----------
----------------------------CAGAGAGAAAAAAGAGCA---GTGGGA---CTAGGAGCT------
---TTGTTCCTTGGGTTCTTGGGAGCA---GCAGGAAGCACTATG---GGCGCAGCGTCA---
ATGACGCTGACGGTACAG---GCCAGACTA---TTATTG---TCTGGTATAGTGCAACAGCAGAAC---
AATTTG---CTGAGG---GCCATTGAGGCGCAACAGCATTTGTTGCAACTCACAGTCTGGGGCATC
>GU455448
TTCTACTGTAATACTTCAAAACTGTTTAATAGTACTTGGAATGATACTACTTTGACTTGGAATGACACTATTT
TAAAT---
-------------------GAG---
ACAATGGACACAAATATCACACTTCCATGCAGAATAAAACAAATTATAAACATGTGGCAGGAAGTAGGAAAAG
CAATGTATGCCCCTCCCATCAAAGGACAAATTAGATGTTCATCAAAC---
ATTACAGGGCTGCTATTAACAAGAGATGGTGGTACTACTGACAATAAA------------------------
----

ACAAACATCGAGACCTTCAGACCTGGAGGAGGAGATATGAGGGACAATTGGAGAAGTGAATTATATAAATATA
AAGTAGTAAGAATTGAACCATTA---GGAGTAGCACCCACCAAGGCAAAGAGAAGAGTGGTG----------
----------------------------CAGAGAGGAAAAAGAGCAATAGGGGGA---CTAGGAGCT------
---TTGTTCCTTGGGTTCTTGGGAACA---GCAGGAAGCACTATG---GGCGCAGCGTCA---
ATAACGCTGACGGTACAG---GCCAGACAA---TTATTG---TCTGGTATAGTGCAACAGCAAAAC---
AATTTG---CTGAGG---GCTATTGAGGCGCAACAACATCTGTTGCAACTCACAGTCTGGGGCATC
>DQ178989
TTTTACTGTAATACATCACAGCTGTTTGATAGTATTTGGAATAATAGTACTAGGAATGACACTAGAGAGTCAA
AT--

AGCACTGATCCTATCATACTCCCATGCAGAATAAAGCAAATTATAAACAGGTGGCAGGAAGTAGGAAAAGCAA
TGTATGCCCCTCCCATCAGTGGACTGATTAGGTGTTCATCAAAT---
ATTACAGGGCTACTATTAACAAGAGATGGTGGTAATAGTGTTAACACCACC---------------------
----

AAGAACACCACCGAGACCTTCAGACCTGGAGGAGGAGATATGAGGGACAATTGGAGAAGTAAATTATATAAAT
ATAAAGTAGTAAAAATTGAACCATTA---GGAGTAGCACCCACCAAGGCAAAAAGAAGAGTGGTG--------
-------------------------------CAGAGAGAAAAAAGAGCA---GTAACG---CTAGGAGCT---
------ATGTTCCTTGGGTTCTTGGGAGCA---GCAGGAAGCACTATG---GGCGCAGCGTCG---
CTGACGCTGACGGTACAG---GCCAGACTA---TTATTG---TCTGGTATAGTGCAACAGCAAAAC---
AACTTG---CTGAGA---GCTATTGAGGCGCAACAGCATCTGTTGCAACTCACAGTCTGGGGCATC
>EU744007
TTCTACTGTAATACAACAGAACTGTTTAAAGGTATTTGGAATTTAAATAATACTTGGACTCCAAATAGTACTT
GGGATTCACTT---------------------------------AATAATACTTTGGAT---
ACATCATGGAATAGTACTATA---------------
GAACCAAATAAGACTATCATACTCCCATGCAGAATAAAACAAATTATAAACATGTGGCAGGAAGTAGGAAAAG
TAATGTATGCCCCTCCCATCAAAGGAATAATTAAATGCTCATCAAAC---
ATTACAGGGCTACTATTAACAAGAGATGGTGGTAATAAAGAGAATGAGACC----------------------
----

CAGAATGGGACCGAGATCTTTAGACCTGGAGGAGGAGATATGAGGGACAATTGGAGAAGTGAATTATATAAAT
ATAAAGTAGTAAAAATTGAACCATTA---GGAGTAGCACCCACCAGGGCAAAGAGAAGAGTGGTG--------
-------------------------------CAGAGAGAAAAAAGAGCA---ATGGGA---ATAGGAGCT---
------ATGTTCCTTGGGTTCTTGGGAGCA---GCAGGAAGCACTATG---GGCGCAGCGTCG---
ATAACGCTGACGGTACAG---GCCAGACAA---TTATTG---TCTGGTATAGTGCAACAGCAGAGC---
AATTTG---CTGAGG---GCTATTGAGGCGCAACAGCATCTGTTGCAACTCACAGTCTGGGGCATT
>GU455525
TTCTACTGTAATACAACAAAACTGTTTGATTGGAAGGATACAACACAACAGTTTAATTGGAATATTACTAACG
GGTCA--

TATAACAATGGCACAATTACACTCCCATGCAGAATAAAACAAATTATAAACATGTGGCAGGAAGTAGGAAAAG
CAATGTATGCCCCTCCCATCGAAGGACGAATTAGATGTTTATCAAAT---
ATTACAGGGCTGCTATTAACAAGAGATGGTGGTAATAATAAGACG----------------------------
----

AGCGACCCCGAGATCTTCAGACCTGGAGGAGGAGATATGAGGGACAATTGGAGAAGTGAATTATATAAATATA
AAGTAGTAAAAATTGAACCATTA---GGAGTAGCACCCACCAAGGCAAAGAGAAGAGTGGTG-----------
----------------------------CAGAGAGAAAAAAGAGCA---GTGGGA---ATAGGAGCT------
---CTGTTCCTTGGGTTCTTGGGAGCA---GCAGGAAGCACTATG---GGCGCAGCGTCA---
CTAACGCTGACGGTACAG---GCCAGACAA---GTATTG---TCTGGTATAGTGCAACAGCAGAAC---
AATTTG---CTGAGG---GCTATTGAGGCGCAACAGCATCTGTTGCAACTCACAGTCTGGGGCATC
>EU744052
TTCTACTGTAATTCAACACCACTGTTTAATAGTACTTGGTATATTAATAGTACTGAGAATGTTACTGAAAAG-
----

GCAAATGAAACAATTACACTCCAATGCAGAATAAAACAAATTATAAACATGTGGCAGGAAGTAGGAAAAGCAA
TGTATGCCCCTCCCATCAGAGGACAAATTAAATGTTCATCAAAT---
ATTACAGGGCTGCTATTAACAAGAGATGGTGGTGAGCACGAGAAC----------------------------
----

AGCACCACCGAGACCTTCAGACCTGGAGGGGGAGATATGAGGGACAATTGGAGAAGTGAATTATATAAATATA
AAGTAGTAAGAATTGAACCATTA---GGAGTAGCACCCACCAAGGCAAAGAGAAGAGTGGTG-----------
----------------------------CAGAGAGAAAAAAGAGCA---GTGGGA---ATAGGAGCT------
---TTATTCCTTGGGTTCTTGGGAGCA---GCAGGAAGCACTATG---GGCGCAGCATCA---
ATAACGCTGACGGTACAG---GCCAGACAA---TTATTG---TCTGGTATAGTGCAACAGCAGAAC---
AATCTG---CTGAGG---GCTATTGAGGCGCAACAGCACCTGTTGCAACTCACAGTCTGGGGCATC
>U15030
TTCTACTGTAATACAACAGGACTGTTTAATAGTACTTGGAATGGTACTGAAGAGATTGTTACTGAAAGGTCAA
ATAGA--
-------------------ACA---
GGAGAAAATGACACACTCATACTCCAATGCAAAATAAGACAAATTGTAAACCTGTGGCAGCAAGTAGGAAAAG
CAATGTATGCCCCTCCCATCCAAGGACAAATTAGCTGTTCATCAAAT---
ATTACAGGGCTGCTATTAACAAGAGATGGTGGTAATAATAATAGTAGT-------------------------
----

AATAACAACACTGAAATCTTCAGACCTGGAGGAGGAGATATGAGGGACAATTGGAGAAGTGAATTATATAAAT
ATAAAGTAGTAAAAATTGAACCATTA---GGACTAGCACCCACCAAGGCAAAGAGAAGAGTGGTG--------
-------------------------------CAGAGAGAAAAAAGAGCA---GTGGGA---ATAGGAGTT---
------TTGTTCCTTGGGTTCTTAGGAGCA---GCAGGAAGCACTATG---GGCGCAGCGGCA---
GTGACGCTGACGGTACAG---GCCAGACAA---TTGTTG---TCTGGTATAGTGCAACAGCAGAAC---
AACCTG---CTGAGA---GCTATTGAGGCGCAACAGCATCTGTTGCAACTCACAGTCTGGGGCATC
>L08655
TTCTACTGTAATACAACACAACTGTTTAATAGTACTTGGTATGCTAATGATACTGAAGGGTCAAATGAC----
----

ACTAAAGCAAATATCACACTCCCATGTAGAATAAAACAAATTATAAACATGTGGCAGGAAGTAGGGAAAGCAA
TGTATGCCCCTCCCATCAAAGGACAAATTAGATGTTCATCAAAT---
ATTACAGGGCTACTATTAACAAGAGATGGTGGTAATAATGAGAACGGGACCGAT-------------------
----

GAGAACAGGACCGAGGAGATCTTCAGACCTGGAGGAGGAGATATGAGGGACAATTGGAGAAGTGAATTATATA
AATATAAAGTAGTAAAAATTGAGCCATTA---GGAGTAGCACCCACCAAGGCAAAGAGAAGAGTGGTG-----
----------------------------------CAGAGAGAAAAAAGAGCA---
GTGGGAATGATAGGAGCT---------ATGTTCCTTGGGTTCTTGGGAGCA---GCAGGAAGCACTATG---
GGCGCAGCGTCG---CTGACGCTGACGGTACAG---GCCAGACAA---TTATTG---
TCTGGTATAGTGCAACAGCAGAAC---AATTTG---CTGAGG---
GCTATTGAGGCGCAACAGCATCTGTTGCAACTCACAGTCTGGGGCATC
>JF320008
TTTTACTGTAATACAACATCATTGTTTAATGGGACTTGGAATGGGACTTGGGATAATACCTGGCAAGGG----
----

TCAAATGGCACTATCACACTCCAATGTAGGATAAAACAAATTATAAACATGTGGCAGGAAGTAGGAAAAGCAA
TGTATGCTCCTCCCAGAGCAGGAATAATTAATTGTGTATCAAAT---
ATTACAGGGCTGATGTTAATGAGAGATGGTGGTAAGGAGGACAATAGTACC----------------------
----

GAGAATAGTACCGAGATCTTCAGACCTGGAGGAGGGAATATGAAGGACAATTGGAGAAGTGAATTATATAAAT
ATAAAGTAGTAAAAATTGAACCATTA---GGAGTAGCACCCACCAAGGCAAAGAGAAGAGTGGTG--------
-------------------------------CAGAGAGAAAAAAGAGCA---GTAACG---ATAGGAGCT---
------ATGTTCCTTGGGTTCTTGGGAGCA---GCAGGAAGCACTATG---GGCGCAGCGTCA---
ATGACGCTGACGGTACAG---GCCAGGCAA---TTATTG---TCTGGTATAGTGCAACAGCAGAAC---
AATCTG---CTGAGA---GCTATTGAGGCGCAACAGCATCTGTTGCAACTCACAGTCTGGGGCATC
>JF320215
TTCTACTGTGATACAACACAACTGTTTAATAGTACTTGGAATACTACTACAGGGTCAAATGCTACTACAGGGT
CAAATGGC---
-------------------ATT---
AGTACAAATGACACAATCACGCTCCCATGTAGAATAAAGCAAATTATAAACATGTGGCAGGAAGTAGGAAAAG
CAATGTATGCTCCTCCCATCAAAGGACTAATTAGATGTTCATCAAAC---
ATTACAGGGCTACTATTAACAAGGGATGGTGGTGATAGCCCCAAC----------------------------
----

AAGAGCCAAGAGACCTTTAGACCTGGCGGAGGAGATATGAGGGACAATTGGAGAAGTGAACTATATAAATATA
AAGTAGTAAAAATTGAACCATTA---GGAGTAGCACCTACCATGGCAAAGAGAAGAGTGGTG-----------
----------------------------CAGAGAGAAAAGAGAGCA---GTGGGA---CTAGGAGCT------
---CTGTTCCTTGGGTTCCTGGGAGCA---GCAGGAAGCACTATG---GGCGCAGCGTCA---
ATAACGCTGACGGTACAG---GCCAGACAA---TTATTA---TCTGGTATAGTGCAACAGCAGAGC---
AATTTG---CTGAGG---GCTATTGAGGCGCAACAGCATATGTTGCAACTCACAGTCTGGGGCATC
>JF320208
TTCTACTGTAATACAACAAAACTGTTTAATAGTACTTGGAATTGGAATAATACAATAGAGGGCAATAGCACTG
AA---

GATAATGAGACTATCACACTCCCATGTAGAATAAAACAAATTATAAACATGTGGCAGACAGTAGGAAAAGCAA
TGTATGCCCCTCCCATCAGAGGACCAATTAGATGTTCATCAAAC---
ATTACAGGGATACTACTGACAAGGGATGGTGGTAATGAGAACAACAAC-------------------------
----

TCCATCCCCGAGATCTTCAGACCTGGAGGAGGAGATATGAGGGACAATTGGAGAAGTGAACTATATAAATATA
AAGTAGTACAAATTGAACCATTA---GGGATAGCACCCACCAAGGCAAAGAGAAGAGTGGTG-----------
----------------------------CAAAGAGAAAAAAGAGCA---GTGGGA---CTAGGAGCG------
---TTGTTCATTGGGTTCTTGGGAGCA---GCAGGAAGCACTATG---GGCGCAGCGTCA---
GTGACGCTGACGGTACAG---GCCAGACAA---TTACTG---TCTGGTATAGTGCAACAGCAGAAC---
AATTTG---CTGAGG---GCTATTGAGGCGCAACAGCATATGTTGCAACTCACAGTCTGGGGCATC
>JF320230
TTCTACTGTAATGCATCACAACTGTTTAATAGTACTTGGAGTGTTTTTGCAAATAATGGTACTTGGACTGCAA
CTAATGGC---
-------------------ACT---
GAAGGAAATGATACTATCACTCTCCCATGTAGAATAAAACAATTTGTAAACATGTGGCAGGAAGTAGGAAAAG
CAATGTATGCCCCTCCTATCAGAGGACTAATTAGATGTTCATCAAAT---
ATTACAGGGCTGCTATTAACAAGAGATGGTGGTAAGGATAATAGCAGC-------------------------
----

AATAGCATGGAGGTCTTCAGACCTGGAGGAGGAAATATGAAGGACAATTGGAGAAGTGAATTATATAAATATA
AAGTAGTAAAAATTGAGCCATTA---GGAATAGCACCCACCAAGGCAAAGAGAAGAGTGGTG-----------
----------------------------CAGAGAGAAAAAAGAGCA---GTGGGAGTACTGGGAGCT------
---ATGTTCCTTGGGTTCTTGGGAACA---GCAGGAAGCACTATG---GGCGCAGCGTCA---
GTGACGCTGACGGTACAG---GCCAGACAA---TTATTG---TCTGGTATAGTGCAACAGCAGAAC---
AATCTG---CTGAAG---GCTATTGAGGCGCAACAGCATCTGTTGCAACTCACAGTCTGGGGCATC
>JF320013
TTCTACTGTAATACAACAAAACTGTTTAACAGTACTTGGTATAGTAATGGTACTTGGACTGGTACTAATGAAA
CA---

AATAACAATGATACTATCACACTCCCATGCAGAATAAGACAAATTGTAAACATGTGGCAGGAAGTAGGAAAAG
CAATGTATGCCCCTCCCATCAAAGGAGTAATCAGATGTACATCAAAT---
ATTACAGGGCTGCTATTAGTAAGAGATGGAGGCCAACAGAACAATACT-------------------------
----

GGGAACGAGACTGAGGTCTTCAGACCTTCAGGAGGAAATATGAAGGACAATTGGAGGAGTGAATTATATAAAT
ATAAAGTAGTAAGAATTGAACCATTA---GGAATAGCGCCCACTAGGGCAAAGAGAAGAGTGGTG--------
-------------------------------CAAAGAGAAAAGAGAGCA---GTAGGA---CTAGGAGCT---
------GTGTTCCTTGGGTTCTTGGGAGCA---GCAGGAAGCACTATG---GGCGCAGCGTCA---
ATTACGCTGACGGTACAG---GCCAGACAA---TTATTG---TCTGGTATAGTGCAACAGCAGAAC---
AATTTG---CTGAGA---GCTATTGAGGCGCAACAGTATATGTTGCAACTCACAGTCTGGGGCATT
>JF320018
TTCTACTGTGACTCTACACAGCTGTTTAATAGTACTTGGAATTGGAGTAATACTATAGGGATAAATGACACGG
AA---

GAAAATTTCAATATCACACTCCCATGCAGAATAAAACAAATTGTAAACATGTGGCAGACAGTAGGAAAAGCAA
TGTATGCCCCTCCCATCAGAGGACAAATTAGATGTTCATCAAAT---
ATTACAGGACTAATATTAACAAGAGATGGTGGAAAAAATAATGATACT-------------------------
----

AATAACACAGAAACCTTCAGACCAGGAGGAGGAAATATGAAGGATAATTGGAGAAGTGAATTATATAAATATA
AAGTAGTAAGAATTGAACCATTA---GGAGTAGCACCCACCAAGGCAAAGAGAAGAGTGGTG-----------
----------------------------CAAAGAGAAAAAAGAGCA---GTGGGA---ATGGGAGCT------
---TTGTTCATTGGGTTCTTGGGAGCA---GCAGGAAGCACTATG---GGCGCAGCGTCA---
GTGACGCTGACGGTACAG---GCCAGACAA---TTATTG---TCTGGTATAGTGCAACAGCAGAAT---
AATCTG---CTGCAA---GCTATTGAGGCGCAACAGCATCTGTTGCAACTCACAGTCTGGGGCATC
>JF320028
TTCTACTGTAATACAACAAAATTATTTAATAGTACGTGGAATGAGAATGGGAGTTGGAATGATACTGACTGG-
----

TCAAATGACACTATCATACTCCCATGCAAAATAAAACAAATTATACGCATGTGGCAGGGGGTAGGACAAGCAA
TATATGCCCCTCCCATCAGCGGACAAATCAGATGTTCATCAAAC---
ATTACAGGGCTATTACTAACAAGAGATGGTGGTATCGAGAACAAT----------------------------
----

GACACTGAAACCTTCAGACCTGTAGGAGGAGATATGAGGGACAATTGGAGAAGTGAATTATATAAATATAAAG
TAGTAAGAATTGAACCATTA---GGAGTAGCACCCACCAAGGCAAAAAGAAGAGTGGTG--------------
-------------------------CAGAGAGAAAAAAGAGCA---GTGGGA---TTCGGAGCT---------
GTGTTCCTTGGGTTCTTGGGAGCA---GCAGGAAGCACTATG---GGCGCAGCGTCA---
GTAACGCTGACGGTACAG---GCCAGACAA---TTATTG---TCTGGTATAGTGCAACAGCAGAAC---
AATTTG---CTGAGG---GCTATTGAGGCGCAACAGCATCTGTTGCAACTCACAGTCTGGGGCATC
>JF320019
TTCTACTGCAATACAACATCACTGTTTAATGGTACTTGGTTTAATAGTACTGATAATAATACTGCAGAAACCA
TT---

ACTGGGAATAATACAATCACACTTCCATGCAGAATAAAACAAATTATAAACTTGTGGCAGGAAGTAGGAAAAG
CAATGTATGCCCCTCCCATCGCAGGACAAATTAATTGTTTATCGAAT---
ATTACGGGGCTGCTATTAACAAGAGATGGTGGTAATAACAACAGCACA-------------------------
----

GACAATAATAATGAAACCTTCAGACCTGCAGGAGGAGATATGAGGGACAATTGGAGAAGTGAATTATATAAAT
ATAAAGTAGTAAGAATTGAACCATTG---GGAGTAGCACCCACCAAGGCAAAGAGAAGAGTGGTG--------
-------------------------------CAGAGAGAAAAAAGAGCA---GTAGGA---CTAGGAGCT---
------TTGTTCCTTGGGTTCTTGGGAGCA---GCAGGAAGCACTATG---GGCGCAGCATCA---
CTAACGCTGACGGTACAA---GCCAGACAA---TTATTG---TCTGGTATAGTGCAACAGCAGAAC---
AATTTG---CTGAGG---GCTATTGAGGCGCAACAGCATCTGTTGCAACTCACAGTCTGGGGCATC
>MH078552
TTCTACTGTAATACAACACAACTATTTAATAGTACTTGGAATAGTACTGCAGTGACCACTCGGAATACTAATA
ATACT--

GATGATGATAAGATTATACTCCCATGCAGAATAAAACAAATTATAAACATGTGGCAGGAAGTAGGAAAAGCAA
TGTATGCTCCTCCCAGAGCAGGAAATATTAGATGTAATTCAAAT---
ATTACAGGGCTGCTATTAATAAGAGATGGGGGTCTGAATGAGACC----------------------------
----

AACAATACCGAGATCTTCAGACCTACAGGAGGGAACATGAAGGACAATTGGAGAAGTGAATTATATAAATATA
AAGTAGTAGAAGTGGAACCAGTA---GGAGTAGCACCCACCAAGGCAAAGAGGAGAGTGGTG-----------
----------------------------CAAAGAGAAAAAAGAGCA---GTAGTGACGGCAGGAGCT------
---ATGTTCCTTGGGTTCTTGAGTGCA---GCAGGAAGCACTATG---GGCGCAGCGTCA---
CTAACGCTGACGGTACAG---GCCAGGCAA---TTATTG---TCTGGTATAGTGCAACAGCAGAAC---
AATCTG---CTGAGA---GCTATTGAGGCGCAACAGCATATGTTGCAACTCACAGTCTGGGGCATC
>MH078553
TTCTACTGTAACACAACACCACTATTTAATAGTACCTGGCATCCGAATAATACCAACATAACTAGC-------
----

ACTAGAGAAAACATCACACTCACATGTAAAATAAGACAAATTATAAACATGTGGCAGGAAGTAGGAAAAGCAA
TGTATGCCCCTCCCATCAGAGGACTAATTAGGTGTTCATCAAAT---
ATTACAGGACTATTACTAACAAGAGATGGGGGTAGTGATAAGAACAGTAAC----------------------
----

CTTAATGGGACTGAAACCTTCAGACCTGGAGGAGGAGATATGAGGGACAATTGGAGAAGTGAATTATATAAAT
ATAAAGTAGTAGAAATTGAACCACTA---GGAATAGCACCCACCAAGGCAAAGAGAAGAGTGGTG--------
-------------------------------CAAAGAGAAAAAAGAGCA---GTGGGA---CTAGGAGCT---
------CTGTTCCTTGGGTTCTTGGGAGCA---GCAGGAAGCACTATG---GGCGCAGCGTCA---
ATAACGCTGACGGTACAG---GCCAGACAA---TTATTG---TCTGGTATAGTGCAGCAGCAAAAC---
AATTTG---CTGAGA---GCTATTGAGGCGCAACAGCATATGTTGCAACTCACAGTCTGGGGCATC
>MH078555
TTCTACTGTAATACAGCACAACTATTTAATGGTACTTGGGAGGCATATAAAAATAGCTCC-------------
----

ATGAATGATACTATAACACTCCCATGTAGAATAAAGCAAATTATAAACATGTGGCAGAAAGTAGGAAAAGCAA
TGTATGCCCCTCCCATCAGTGGAATAATTAACTGCACATCAAAT---
ATAACAGGGCTGTTCTTAACAAGAGATGGAGGCATTAACAATAGT----------------------------
----

AATAATGAAACCTTCAGACCTGGAGGAGGAGACATGAAGGACAATTGGAGAAGTGAATTATATAAATATAAGG
TAATAAAAGTTGAACCTATA---GGACTGGCACCCACCAAGGCAAGAAGAAGAGTGGTA--------------
-------------------------CAGAGGGAAAAAAGAGCA---ATAACA---CTGGGAGCT---------
ATGTTCCTTGGGTTCTTGGGAACA---GCAGGAAGCACTATG---GGCGCAGCGTCA---
GTGGCGCTGACGGTACAG---GCCAGACAG---TTACTA---TCTGGTATAGTGCAACAGCAGAAC---
AATTTG---CTGAGG---GCTATTGAGGCGCAACAGCATATGTTGCAACTCACAGTCTGGGGCATC
>EF210726
TTCTACTGTAATACAACACCACTATTTAATACTACTTGGGTTAATGAGACTGGGCCTAATAATACCCAG----
----

GGGAATGATACCATCACACTCCAATGCAGAATAAAACAAATTATAAACCTGTGGCAGAAAGTAGGAAAAGCAA
TGTATGCCCCTCCTATCAGAGGACGAATTAGATGTTCATCAAAT---
ATTACAGGACTACTATTAACAAGAGATGGTGGGAATAACAATACT----------------------------
----

AATGAGACCTTCAGACCGGGAGGAGGAGATATGAGGGACAATTGGAGAAGTGAATTATATAAATATAAAGTAG
TACAAGTTGAACCATTA---GGATTAGCACCCACCAAGGCAAGGAGAAGAGTGGTG-----------------
----------------------CAGAGAGAAAAAAGAGCA---GTGGGA---CTGGGAGCT---------
GTGTTCCTTGGGTTCTTGGGAGCA---GCAGGAAGCACTATG---GGCGCAGCGTCA---
ATGGCGCTGACGGTACAG---GCCAGACAA---TTATTG---TCTGGTATAGTGCAGCAGCAGAAC---
AACATG---CTGCGG---GCTATTGAGGCGCAACAGCACCTGTTGCAACTCACAGTCTGGGGCATT
>EF210730
TTCTACTGTAATACATCACAACTGTTTAATAGTACTTGGAATGGAACAGAGCCAACTAATAGCAATAAA----
----

GAAAATGGAACTATCACACTCCCATGTAGAATAAAACAAATTGTAAACATGTGGCAGGAAGTAGGAAAAGCAA
TGTATGCCCCTCCCATCAGAGGACAAATTAGATGTTCATCAAAT---
ATTACAGGGCTACTCTTAACAAGAGATGGTGGTAATAGCAGTACC----------------------------
----

GAGACCTTCAGACCTGGAGGAGGAGATATGAGGGACAATTGGAGAAGTGAATTATATAAATATAAAGTAGTAA
AAATTGAACCATTA---GGAGTAGCCCCCACCAAGGCAAAAAGAAGAGTGGTG--------------------
-------------------CAGAGAGAAAAAAGAGCA---GTGACA---CTAGGAGCT---------
ATGTTCCTTGGGTTCTTGGGAGCA---GCAGGAAGCACTATG---GGCGCAGCGTCA---
ATGACGCTGACGGTACAG---GCCAGACAA---TTATTG---TCTGGTATAGTGCAACAGCAGAAC---
AATTTG---CTGAAG---GCTATTGAGGCGCAACAGCATCTGTTGCAACTCACAGTCTGGGGCATC
>EF210732
TTCTACTGTAATTCATCACAACTGTTTAACAGTACTTCAAAACAACTGTTTAACAGTACTTGGAATAAT----
----

ACAAATGCAAATATCGAACTCCCGTGCAGAATAAGACAAATTGTAAATATGTGGCAGAGAATAGGAAAAGCAA
TGTACGCCCCTCCCATCAGTGGACAAATTAACTGTACATCAAAT---
ATTACAGGACTGCTATTAACAAGAGATGGTGATAAGAACAACAAG----------------------------
----

TCCACCGAGGTCTTCAGACCTATAGGAGGAGAAATGAGGGACAATTGGAGAAGTGAATTATATAAATATAAGG
TAGTACAAATTGAACCATTA---GGAGTAGCACCCACCAGGGCAAAAAGAAGAGTGGTG--------------
-------------------------CAGAGAGAAAAGAGAGCA---GTGGGA---ATAGGAGCT---------
GTGTTCCTTGGGTTCTTGGGAGCA---GCAGGAAGCACTATG---GGCGCAGCGGCA---
GTGACGCTGACGGTACAG---GCCAGACTA---TTATTG---TCTGGTATAGTGCAACAGCAAAGC---
AATTTG---CTGAGG---GCTATTGAGGCGCAACAGCATCTGTTGCAACTCACAGTCTGGGGCATC
>KC596069
CTCTACTGTAATACAACACAACTGTTCAATGGGACTTGGAATGGTACTGCCTGGTCAAATGGTACTGACTGGT
CAAATAGCACT--
-------------------GAA---
GGAGATAATACCACACTCACACTCCCATGCAGAATAAAACAAATTATAAACATGTGGCAGAAGGTAGGAAAAG
CAATGTATGCCCCTCCCATCAGAGGAGAAATTAGATGTTCATCAAAT---
ATTACAGGTCTGCTATTAACAAGAGATGGTGGTGAGCAAAATAGC----------------------------
----

TCAGAAGAGACCTTCAGACCTGGAGGAGGAGATATGAGGGACAATTGGAGAAGTGAATTATACAAATATAAAG
TAGTAAAAATTGAACCATTA---GGAATAGCACCCACCAAGGCAAAGAGAAGAGTGGTG--------------
-------------------------CAAAGAGAAAAAAGAGCA---GTGGGA---TTCGGAGCT---------
CTGTTCCTTGGGTTCTTGGGAGCA---GCAGGAAGCACTATG---GGCGCAGCGTCA---
GTGACGCTGACGGTACAG---GCCAGACTG---TTACTG---TCTGGTATAGTGCAACAGCAAAAC---
AACTTG---CTGAGG---GCTATTGAGGCGCAACAGCATCTGTTGCAACTCACAGTCTGGGGCATC
>JN251896
TTCTACTGTAATACAACACCGCTGTTTAATAAGACTTGGAATACAACAGAGCTGTCACAGTATGATAGTAGTG
ATACT--

AATAGTAATGCTACTATCATACTCCCATGCAGAATAAAACAAATTATAAACAGATGGCAGGAAGTAGGAAAAG
CAATGTATGCCCCTCCCATCAGAGGACAAATTAGATGTAAATCAAAT---
ATTACAGGGCTGCTATTAACAAGAGATGGTAGTACCAATGATACA----------------------------
----

ACCGAGACCTTCAGACCTGGAGGAGGAAATATGAAGGACAATTGGAGAAGTGAATTATATAAATATAAAGTAG
TAAAAATTGAGCCATTA---GGAATAGCACCCACCAAGGCAAAGAGAAGAGTGGTG-----------------
----------------------CAGAGAGAAAAAAGAGCA---GTAACG---ATGGGAGCT---------
ATGTTCCTTGGGTTCTTGGGAGCA---GCAGGAAGCACTATG---GGCGCAGCGTCA---
GTGACGCTGACGGTACAG---GCCAGACTA---TTATTG---TCTGGTATAGTGCAACAGCAAAAC---
AATTTG---CTGAGA---GCTATTGAGGCGCAACAGCATCTGTTGCAACTCACAGTCTGGGGCATC
>JN251901
TTCTACTGTAATACAACAAAATTGTTTAATAGCACTTTTAATATTAATAGTACTTTAAATGGGACTGAGAGC-
----

AGTAATGAAACTATCACACTCCCATGCAGAATAAAACAAATTATAAACAGGTGGCAGGAAGTAGGAAAGGCAA
TGTATGCCCCTCCCATCAGTGGACAAATTAAGTGTGAATCAAAT---
ATTACGGGGATGCTATTAACAAGAGATGGTGACGGGAACAACAGC----------------------------
----

AGCTCTGAGACCTTCAGACCTGGAGGAGGAGATATGAGGGATAATTGGAAAAGTGAATTATATAAATATAAAG
TAGTAAAAATTGAACCACTA---GGAGTAGCACCCACCAAGGCAAAGAGAAGAGTGGTG--------------
-------------------------CAGAGAGAAAAAAGAGCA---ATAGGA---ATAGGAGCT---------
GTGTTCCTTGGGTTCTTGGGAGCA---GCAGGAAGCACTATG---GGCGCAGCGTCG---
GTGACGCTGACGGTACAG---GCCAGACAA---TTACTG---TCTGGTATAGTGCAACAGCAGAAC---
AATCTG---CTGAGG---GCTATTGAGGCGCAACAGCATATGTTGCAACTCACAGTCTGGGGCATC
>JN251906
TTCTATTGTAATACAACACAACTGTTTAATAGTACATGGAATGGTACTGAAGGGTCAAATAGCACA-------
----

AAAAATGGCACTATCATACTCCCATGCAGAATAAAACAAATTATAAACATCTGGCAGAGAGTAGGAAAAGCAA
TGTATGCCCCTCCCATCCAAGGACAAATTAGATGTTCATCACAT---
ATTACAGGGCTGCTATTAACAAGAGATGGAGGTATGCAAAATAACAAGAAA----------------------
----

ACTAGCATAACTGAGATTTTCAGACCTGGAGGAGGAGACATGAGGGACAATTGGAGAAGTGAATTATATAAAT
ATAAAGTAGTAAAAATTGAACCATTA---GGAATAGCACCCACTAAGGCAAAGAGAAGAGTGGTG--------
-------------------------------CAGAGAGAAAAAAGAGCA---GTGGGA---ATAGGAGCT---
------TTGTTCCTTGGGTTCTTGGGAGCA---GCAGGAAGCACTATG---GGCGCAGCGTCA---
TTGACGCTGACGGTACAG---GCCAGACAA---TTATTG---TCTGGTATAGTGCAACAGCAGAAC---
AATTTG---CTGAGG---GCTATTGAGGCGCAACAGCATATGTTGCAACTCACAGTCTGGGGCATC
>AY819715
TTCTACTGTAATACAGCACAACTGTTTAATAGTACTTGGAATACTAATGATACTAGGGATAGTACTAATGAC-
----

ACAAATAAAAATATCACACTCCCATGCAGAATAAAACAAATTATAAACATGTGGCAGGAAGTAGGAAAAGCAA
TATATGCCCCTCCCATCGAGGGAGCAATTAACTGTTCATCAAAT---
ATTACAGGGTTGCTATTAACAAGAGATGGTGGTGAGGGTAATGTGAGC-------------------------
----

GGGAGCAATGAAACCTTCAGACCTGGAGGAGGAAATATGAGGGACAATTGGAGAAGTGAATTATATAAATACA
AAGTAGTAAAAATCCAACCATTA---GGAGTAGCACCCACCAAGGCAAGGAGAAGAGTGGTG-----------
----------------------------CAGAGAGAAAAAAGAGCA---GTAGGA---CTGGGAGCT------
---GTGTTCCTTGGGTTCTTGGGAGCA---GCAGGAAGCACTATG---GGCGCAGCGTCA---
GTGACGCTGACGGTACAG---GCCAGACAA---TTATTG---TCTGGTATAGTGCAACAGCAGAAC---
AATTTG---CTGAGA---GCTATTGAGGCGCAACAGCACCTGTTGCAACTCACAGTCTGGGGCATC
>HQ896488
TTCTACTGTAATACATCACAATTGTTTAATAGTACTTGGAAGAATGGTACTTTTGCCAATGGCACTGAA----
----

GGAAATGGCACAATCACACTCCCATGCAGAATAAAGCAATTTATAAACATGTGGCAACAAGTAGGAAAAGCAA
TGTATGCCCCTCCCATCAGTGGACAAATTAACTGCTCATCAAAT---
ATTACAGGGCTACTATTAACAAGAGATGGTGGTAGTGGTAACAGCACAGAG----------------------
----

AACGAGACTGAGATCTTCAGACCTGCAGGGCGAGATATGAGGGACAATTGGAGAAGTGAATTATATAAATATA
AAGTAGTACAAGTTGAGCCATTA---GGATTACCACCCACCAGGGCAAAGAGAAGAGTGGTG-----------
----------------------------CAGAGAGAAAAAAGAGCA---ATAGGA---CTGGGAGCT------
---GCCTTCCTTGGGTTCTTGGGAGCA---GCAGGAAGCACTATG---GGCGCAGCGTCA---
GTGACGCTGACGGTACAG---GCCAGACAA---TTATTG---TCTGGTATAGTGCAACAGCAAAAC---
AATTTG---CTGAGG---GCTATTGAGGCGCAACAGCATATGTTGCAACTCACAGTCTGGGGCATC
>JX500709
TTCTACTGCAAGACAACAGAACTGTTTAATAGTGCTTGGCATAATAATGGTACTAGTACTGTGAATAATACTG
GGAAA--

AATACTGAAAATATTACACTCCCATGTAGAATAAAACAAATTATAAACATGTGGCAGGAAGTAGGAAAAGCAA
TGTATGCCCCTCCCATCAAAGGACTAATTAGATGTACATCAAAT---
ATTACAGGCCTGATATTAACAAGAGATGGCGGTAATAATACTAGT----------------------------
----

AGCAATGAGACCTTCAGACCTGTAGGGGGAGATATGAGGGACAATTGGAGAAGTGAATTATATAAATATAAAG
TAGTAAAAATTGAACCATTA---GGAATAGCACCCACCAAGGCAAAGAGGAGAGTGGTG--------------
-------------------------CAAAGAGAGAAAAGAGCA---ATAGGAACAATGGGAGCT---------
GTGTTCCTTGGGTTCTTGGGAACA---GCAGGAAGCACTATG---GGCGCAGCGTCA---
CTGACGCTGACGGTACAG---GCCAGACAA---TTATTG---TCTGGTATAGTGCAGCAGCAGAAC---
AATCTG---CTGAGG---GCTATTGATGCGCAACAGCATATGTTGCAACTCACAGTCTGGGGCATT
>JX500707
TTCTATTGTAATACAACAAAACTGTTTAATAGTACTTGGGAGAACAATAATACTACTAGTACT----------
----

GGGAATGGGACAATCACACTCCCATGTAGAATAAAACAAATTATAAACAGATGGCAGGAAGTAGGAAAAGCAA
TGTATGCCCCTCCCATCAAAGGAAAAATTCTATGCTCATCAAAT---
ATTACAGGGCTAATATTATCAAGAGATGGTGGTAACAGCAGCACC----------------------------
----

ACCGAGACCTTCAGACCTGCAGGAGGAGATATGAGGGACAATTGGAGAAGTGAATTATACAAATATAAAGTAG
TAAAAATTGAACCATTG---GGAGTAGCACCCACCAAGGCAAAGAGGAGAGTGGTG-----------------
----------------------CAGAGAGAAAAAAGAGCA---GTGGGA---CTAGGAGCG---------
TTATTCCTTGGGTTCTTGGGAGCA---GCAGGAAGCACTATG---GGCGCAGCGTCA---
CTGACGCTGACGGTACAG---GCCAGACAA---TTATTG---TCTGGTATAGTGCAACAGCAAAAC---
AATTTG---CTGAGG---GCTATTGAGGCGCAACAACATCTGTTGCAACTCACAGTCTGGGGCATC
>JX500708
TTCTACTGTAATACAACACAATTGTTTAATAGCACTTGGGATAAGAATATTACAGAGTTTAATAAGACTAATG
AGTTAAAT---

AACACTGAAATTATCACACTCCCATGTAGAATAAAACAAATTATAAACATGTGGCAGGCAGTAGGAAAAGCAA
TGTATGCCCCTCCCATCTCAGGACAAATTAAATGTTCATCAAAT---
ATTACAGGACTGCTATTAACAAGAGATGGTGGTAACAATGACACC----------------------------
----

GAGACCTTCAGACCTGGAGGAGGAGATATGAGGGACAATTGGAGAAGTGAATTATATAAATATAAAGTAGTAA
GAATTGAACCATTA---GGAATAGCACCCACCAAGGCAAAGAGAAGAGTGGTG--------------------
-------------------CAGAGAGAGAAAAGAGCA---GTGGGA---ATAGGAGCT---------
GTGTTCCTTGGGTTCTTGGGAGCA---GCAGGAAGCACTATG---GGCGCGGCGTCA---
ATAACGCTGACGGTACAG---GCCAGGCAA---TTATTG---GCTGGTATAGTGCAACAGCAGAAC---
AATCTG---CTGAGA---GCTATTGAGGCGCAACAACATCTGTTGCAACTCACAGTCTGGGGCATC
>MF373130
TTCTACTGTAATACAACACAACTATTTAATAGTTCTTGGGATATGAATAGAATTGTAAATAGTTCCGAAAATA
AC---

ACTGACAAGAATATCACACTCCCATGCAGAATAAAACAAATTGTAAACATGTGGCAGGAAGTAGGAAAAGCAA
TGTATGCCCCTCCCATCAGTGGACAAATTAGATGTTCATCAAAT---
ATTACAGGGCTGCTATTAACAAGAGATGGTGGTAAAAACAGCAGTAACGAG----------------------
----

ACCGAGACCGAAATCTTCAGACCTGTGGGAGGAAATATGAGGGACAATTGGAGAAGTGAATTATATAAATATA
AAGTAGTACAAATTGAACCATTA---GGAGTAGCACCCACCAAGGCAAAGAGAAGAGTGGTG-----------
----------------------------CAAAGAGAAAAAAGAGCA---GTGGGA---TTAGGAGCA------
---ATGTTCCTTGGGTTCTTGGGAGCA---GCAGGAAGCACTATG---GGCGCAGCGTCA---
ATAACGCTGACGGTACAG---GCCAGACAA---TTATTG---TCTGGTATAGTGCAACAGCAGAAT---
AATTTG---CTGAGA---GCTATTGAGGCGCAACAGCATCTGTTGCAACTCACAGTCTGGGGCATC
>MF373142
TTCTACTGTAATACTACACAACTATTTAATAGTACTTGGAATGGTACTTTGGAAAATACTAATAGTACTGGGA
AT---

GGTGATAATAAAACTATCACACTCCCATGCAGAATAAGACAAATTATAAACATGTGGCAGGAAGTAGGAAAAG
CAATGTATGCCCCTCCCATCAAAGGACAAATTAAATGCTCATCAAAG---
ATTACAGGGCTGTTATTAACAAGAGATGGGGGCAATGTACCAAATGATGGGGGC-------------------
----

AATAATAGCACAGAAATCTTCAGACCTGGAGGAGGAGATATGAGGGACAATTGGAGAAGTGAATTATATAAAT
ACAAAATAGTAAAAGTTAAACCAATA---GGAATAGCACCCACCAAGGCAAAGAGAAGAGTGGTG--------
-------------------------------CAGAGAGAAAAAAGAGCA---GTAACG---CTGGGAGCT---
------GTGTTCCTTGGGTTCTTGGGAGCA---GCAGGAAGCACTATG---GGCGCAGCGTCG---
ATGACGCTGACGGTACAG---GCCAGACAA---TTATTG---TCTGGTATAGTGCAACAGCAGAAC---
AATCTG---CTGAAG---GCTATTGAGGCGCAGCAGCATATACTGCAACTCACAGTCTGGGGCATC
>MF373144
TTCTACTGCAATTCAACACCACTGTTTAATAGCACCTGGTATACTAATGGGACTAGCACCTGGTATACTAATG
GGACTTGG---
AATGTCACTGAAGGGCAT---------
AATAGCACTACAAACATCACACTCCAATGCAGAATAAAACAAATTATAAACATGTGGCAGGAAGTAGGAAAAG
CAATGTATGCCCCTCCCATTGCAGGAATAATTAAATGCTCATCAAAT---
ATCACAGGGCTGCTATTAACAAGAGATGGTGGTAAGGGCAACGGGACTGAC----------------------
----

AACAATACTGAGATCTTCAGACCTGGAGGAGGAGATATGAGGGACAATTGGAGAAATGAATTATATAAATATA
AAGTAGTACAAATTGAACCATTA---GGAGTAGCACCCACCATGGCAAAGAGAAGAGTGGTG-----------
----------------------------CAAAGAGAGAAAAGAGAT---TTGGGA---CTAGGAGCT------
---CTGTTCCTTGGGTTCTTGGGAGCA---GCAGGAAGCACTATG---GGCGCAGCGTCA---
ATAACGCTGACGGTACAG---GCCAGACAA---TTATTG---TCTGGTATAGTGCAACAGCAGAAC---
AACTTG---CTGAGG---GCTATTGAGGCGCAACAGCATCTGTTGCAACTCACAGTCTGGGGCATT
>MF373161
TTCTACTGTAACACAACAAAACTATTTGATAGTACTTGGAATACGAATGCTACTTGGGATAGGAATTGGAATG
ATACCAGT---

GGAGAAAATGACACAATCAGACTCCCATGTAGAATAAAGCAAATTATAAACATGTGGCAGGAAGTAGGAAAAG
CAATGTATGCCCCTCCCATTAGTGGACTAATTAGCTGCTCATCAAAC---
ATTACCGGGTTGTTATTAGTAAGAGATGGTGGTGAAAACACTACC----------------------------
----

GAAGAAGAGATCTTCAGACCTGGAGGAGGAAATATGAAAGATAACTGGAGAAGTGAATTATATAAATATAAAG
TAGTAAAAATTGAACCATTA---GGAGTAGCACCCACCAAGGCAAAGAGGAGAGTGGTG-------------
-------------------------CAGAGAGAAAAAAGAGCA---GTAACG---TTCGGAGCT---------
ATGTTCCTTGGGTTCTTGGGAGCA---GCAGGAAGCACTATG---GGCGCAGCGTCA---
ATAACGCTGACGGTACAG---GCCAGACAA---TTATTG---TCTGGGATAGTGCAACAGCAGAGC---
AATTTG---CTGAAA---GCTATTGAGGCGCAACAACATCTGTTGCAACTCACAGTCTGGGGCATT
>KP411828
TTCTACTGTAATACAACACAACTGTTTAATAGCACTTGGAATATGACTAATAGGTCTAAAAATAACACTGCAG
AA---

AATAATACTACATTCATACTCCCATGCAGAATAAAACAAATTGTAAACTTGTGGCAAGAAGTAGGAAAAGCAA
TGTATGCCCCTCCCATCGCAGGACAAATTAACTGTTCATCAATT---
ATTACAGGACTGCTATTAACAAGAGATGGTGGTAGTAATAATGACACA-------------------------
----

AATGAGACCGAGATCTTTAGACCTGGAGGAGGAGATATGAGTGACAATTGTAGAAGTGAATTATATAAATATA
AAGTAGTAAAAATTGAACCATTA---GGAATAGCACCCACCAGGGCAAAGAGAAGAGTGGTG-----------
----------------------------CAGAGAGAGAAAAGAGCA---GTGACG---CTAGGAGCT------
---GTGTTCCTTGGGTTCTTGGGAGCA---GCAGGAAGCACTATG---GGCGCAGCGTCA---
GTAGCGCTGACGGCACAG---GCCAGACAA---TTGTTG---TTTGGTATAGTGCAACAGCAGAAC---
AATCTG---CTGCGA---GCTATTGAGGCGCAACAACATATGTTGCAACTCACAGTCTGGGGCATC
>MF373204
TTCTACTGTAACACAACAAGACTATTCAACAGTACTTGGAGGATCAATGGTACTCTGAATAGTACTGAAGGGC
CA---

AATAATGCAACTATCATACTCCCATGCAGAATAAAACAAGTTATAAACCTGTGGCAAGAAGTAGGAAAAGCAA
TGTATGCCCCTCCCATCAGGGGAAACATTAGTTGTTCATCAAAC---
ATTACAGGACTGCTATTAACAAGGGATGGTGGTGAT-------------------------------------
----

AATGAAACTGAGACCTTCAGACCTGGAGGAGGAAATATGAAGGACAATTGGAGAAGTGAATTATATAGATATA
AAGTAGTAAAAATTGAACCATTA---GGAATAGCACCCACCACGGCAAGGAGACGAGTGGTG-----------
----------------------------CAGAGAGAAAAAAGAGCA---GTAACA---CTAGGAGCT------
---ATGTTCCTTGGGTTCTTGGGGGCA---GCAGGAAGCACTATG---GGCGCAGCGTCA---
CTGACGCTGACGGTACAA---GCCAGACAA---TTATTG---TCTGGTATAGTGCAGCAGCAAAAC---
AATTTG---CTGAGG---GCTATTGAAGCGCAACAGCATCTGTTGCAACTCACAGTCTGGGGCATT
>GU204920
TTCTACTGTAATACAACACAACTGTTTAATAGTACTTGGAATGGTACTACTGGTACTTGGAGTAGAACTGATA
CTGAA--
-------------------GTG---
TCAAATAGCACTAATATCATACTCCCCTGCAGAATAAAACAAATTATAAACATGTGGCAGGAAGTAGGAAAAG
CAATGTATGCCCCTCCCATCAGTGGACCAATTAACTGTTTATCAAAT---
ATTACAGGGCTGTTATTAACAAGAGATGGTGGTACTAACGAGACTAAC-------------------------
----

GAAAGCAATGAGACCGTCAGACCTGGAGGAGGAGATATGAGGGACAATTGGAGAAGTGAATTATATAAATATA
AAGTAGTAAAAATTGAACCATTA---GGAGTAGCACCCACCAAGGCAAAAAGAAGAGTGGTG-----------
----------------------------CAGAGAGAAAAAAGAGCA---ATAGGA---ATAGGAGCT------
---GTGTTCCTTGGGTTCTTGGGAGCA---GCAGGAAGCACTATG---GGCGCAGCGTCA---
GTGACGCTGACGGTACAG---GCCAGACAA---TTATTG---TCTGGTATAGTGCAACAGCAGAAC---
AATTTG---CTGAGG---GCTATTGAGGCGCAACAGCATATGTTGCAACTCACAGTCTGGGGCATC
>JN251856
TTCTACTGTGATTCATCACCACTGTTTAATAGTACTTGGCCGGTTAATGGTACTAGTACTAATAGC-------
----

ATTAACGAAACTATCACACTCCCATGTAGAATAAAACAAATTATAAACATGTGGCAGGAGGTAGGAAAAGCAA
TGTATGCCCCTCCCATCAGAGGACCAATTAAGTGTTCATCAAAT---
ATTACAGGGCTGATATTAACAAGAGATGGTGGTGATAACATGAAC----------------------------
----

CAAAGCACAGAGACCTTCAGACCGGGAGGAGGAGATATGAGGGACAATTGGAGAAGTGAATTATATAAATACA
AAGTAGTAAAAATTGAACCATTA---GGGGTAGCACCCACCAAGGCAAAGAGACGAGTGGTG----------
-----------------------------CAGAGAGAAAAAAGATCA---GTGACA---CTAGGAGCT------
---ATGTTCCTTGGATTCTTGGGAGCA---GCAGGAAGCACTATG---GGCGCAGCGTCA---
ATGGCGCTGACGGTACAG---GCTAGACAA---TTATTG---TCTGGTATAGTGCAACAGCAGAAC---
AATTTG---CTGAAG---GCTATTGAGGCGCAACAACATCTGTTGCAACTCACAGTCTGGGGCATC
>KY213741
TTCTACTGTAATACATCACAACTATTTAATAGTACTTGGGATGGAAATAGTACTAGGGAGAATAGC-------
----

ACTGGACAGTTATTCACACTCCCATGCAGAATAAAACAAATTATAAACATGTGGCAGAAAGTAGGAAAAGCAA
TGTATGCCCCTCCCATCGCGGGCCCAATTAACTGTTTATCAAAC---
ATTACAGGGCTGTTATTAACAAGAGATGGTGGTGGTAATAATACA----------------------------
----

AATAATGAGACCTTCAGACCAGGAGGAGGAGATATGAGGGACAATTGGAGAAGTGAACTATACAAATATAAAG
TAGTAAGAGTTGAACCATTA---GGACTGGCACCCACCAGGGCAAAGAGAAGAGTGGTG-------------
--------------------------CAGAGAGAAAAAAGAGCA---GTGGGA---CTAGGAGCA---------
TTGTTCATTGGGTTCTTGGGAGCA---GCAGGAAGCACTATG---GGCGCAGCGTCA---
GTGACGCTGACGGTACAG---GCCAGACTA---TTATTG---TCTGGTATAGTGCAACAGCAGAAC---
AATCTG---CTGAGG---GCTATTGAGGCGCAACAGCATCTGTTGCAACTCACAGTCTGGGGCATC
>KY213740
TTTTACTGTAATACAAAACCACTGTTTAATAGTACTTGGACTGGGAAGAATAGTACCTGGAATAGTAATGATA
CTACA--

GGAAATGAAACAATCACACTCCAGTGTAGAATAAAACAAATTATAAACATGTGGCAGAAAGTAGGAAGAGCAA
TGTATGCTCCTCCCATCAGTGGACAAATTAACTGTTCATCAAAT---
ATTACAGGGCTAATATTAACAAGAGATTGGGAGAGAAATAACACG----------------------------
----

AGGGAGAACGAGACCCTCAGACCGGGAGGAGGAGATATGAGGGACATTTGGAGAAATGAATTATACAAATATA
AAGTAGTAAAAATTGAACCATTA---GGAATAGCACCCACCAAGGCAAAGAGAAGAGTGGTG----------
-----------------------------CAGAGAGAAAAAAGAGCA---GTGGGA---ATGGGAGCC------
---TTGTTCCTTGGGTTCTTGGGAGCA---GCAGGAAGCACTATG---GGCGCAGCATCA---
ATAACGCTGACGGTACAG---GCTAGACAA---TTATTG---TCTGGTATAGTGCAACAGCAAAAC---
AATTTG---CTGAGG---GCTATTGAGGCGCAACAACACATGTTGCAACTCACAGTCTGGGGCATC
>KY213747
TTCTACTGTAATACAACACAACTGTTTAATTCAACACACCTGTTTGATACTACTAATAGCACTGGGAAT----
----

GCTACAGGAAATATCACACTTCCTTGCAGAATAAAACAAATTGTAAACATGTGGCAGGGAGTAGGAAAAGCAA
TGTATGCCCCTCCCATTAGAGGACCAATTAGCTGTTCATCAAAG---
ATTACAGGATTGCTATTAACAAGAGATGGTGGTAGTAATAATGAGACA-------------------------
----

AACACCACCGAGACCTTCAGACCTGGGGGAGGAGATATGAAGGACAATTGGAGAAGTGAATTATATAAATATA
AAGTAGTAAAAATTGAACCATTA---GGAGTAGCACCTACCAAGGCAAAGAGAAGAGTGGTG----------
-----------------------------CAGAGAGAAAAAAGAGCA---GTGGGAACAATAGGAGCG------
---ATGTTCCTTGGGTTCTTGGGAGCA---GCAGGAAGCACTATG---GGCGCAGCGTCA---
GTGACGCTGACGGTACAG---GCCAGACAA---TTGTTG---TCTGGTATAGTGCAACAGCAGAGA---
AATTTG---CTGAGA---GCTATTGAGGCGCAACAGCATATGTTGCAACTCACAGTCTGGGGCATC
>KY213751
TTCTACTGCAATACAACACAACTGTTTAACTCTACTTGGAATAGTACTGGGAATATTGCAGAAAATAAT----
----

ACTGCAGAAAATATCACGCTTCCTTGCAGAATAAAACAAATTATAAACATGTGGCAGGGAGTAGGAAAAGCAA
TGTATGCCCCTCCCATTAGCGGACCAATTAGCTGTTCATCAAAT---
ATTACAGGGTTGCTATTAACAAGAGATGGTGGTAATAATGAGACG----------------------------
----

AACACCACCGAGACCTTCAGACCTGGGGGAGGAGATATGAGGGACAATTGGAGAAGTGAATTATATAAATATA
AAATAGTAAAAATAGAACCACTA---GGAATAGCACCTACCAAGGCAAAGAGAAGAGTGGTG-----------
----------------------------CAGAGAGAAAAAAGAGCA---GTGGGAATGATGGGAGCG------
---ATGTTCCTTGGGTTCTTGGGAGCA---GCAGGAAGCACTATG---GGCGCAGCGTCA---
ATGGCGCTGACGGTACAG---GCCAGACAA---TTGTTG---TCTGGTATAGTGCAACAGCAGAGA---
AATCTG---CTGAGG---GCTATTGAGGCGCAACAGCATCTGTTGCAACTCACAGTCTGGGGCATC
>KY213748
TTCTACTGTAATACAACACAACTGTTTAATTCAACACACCCGCCTAATACTACTTGGAATGAGAAT-------
----

AATACTGACATTATCACGCTTCCTTGCAGAATAAAACAAATTGTAAACATGTGGCAGACAGTAGGAAAAGCAA
TGTATGCCCCTCCCATTAGCGGACCAATTAGCTGTTCATCAAAA---
ATTACAGGGTTGCTATTAACAAGAGATGGTGGTAATAGTAACAAT----------------------------
----

GAGACAAACACCACCGAGACCTTCAGACCTGGGGGAGGAGATATGAGGGACAATTGGAGAAGCGAATTATATA
AATATAAAGTAGTAAAAATTGAACCATTA---GGAGTAGCACCTACCAAGGCAAAGAGAAGAGTGGTG-----
----------------------------------CAGAGAGAAAAAAGAGCA---
GTGGGAACAATAGGAGCG---------ATGTTCCTTGGGTTCTTGGGAGCA---GCAGGAAGCACTATG---
GGCGCAGCGTCA---GTGACGCTGACGGTACAG---GCCAGACAA---TTGTTG---
TCTGGTATAGTGCAACAGCAGAGA---AATTTG---CTGAGA---
GCTATTGAGGCGCAACAGCATCTGTTGCAACTCACAGTCTGGGGCATC
>AY945710
TTCTACTGTAATACATCAAAACTGTTTAATAGTACTTGGAATACTAATGGTACTTGGAATGATACTACA----
----

GGAGATGACACTATCACGCTCCCTTGCAGAATAAAGCAAATTGTAAACATGTGGCAGGAAGTAGGAAAAGCAA
TGTATGCCCCTCCCATCGCAGGAAACATCAGCTGTTCATCAAAT---
ATTACAGGGTTAATATTAACATGGGATGGTGGTAATAAGAACGGGAACAAG----------------------
----

ACCACCAATGATACAGAGACCTTTAGACCTGGAGGAGGAGATATGAGGGACAATTGGAGAAGTGAACTATATA
AATATAAAGTAGTGAAAATTGAACCATTA---GGAGTAGCACCCACCAAGGCAAAGAGAAGAGTGGTG-----
----------------------------------CAGAGAGAAAAAAGAGCA---GTGGGA---
ATAGGAGCT---------TTGTTCCTTGGGTTCTTGGGAGCA---GCAGGAAGCACTATG---
GGCGCAGCGTCA---ATGACGCTGACGGTACAG---GCCAGACAA---TTGTTG---
TCTGGTATAGTGCAACAGCAAAGA---AATTTG---CTGAGT---
GCTATTGAGGCGCAACAGCGTCTGTTGCAACTCACAGTCTGGGGCATC
>KJ952535
TTCTACTGTAATACATCAAAACTGTTTAATAGTACTTGGTATAATAATAGTACTTGGAATGGTGCTTGGAATA
ATACTAGA---

GAAGATGACAATATCACGCTCCCTTGCAGAATAAAACAAATTGTAAACATGTGGCAGGAAGTAGGAAAAGCAA
TGTATGCCCCTCCCATCAGAGGACTAATTAGATGTTCATCAAGT---
ATTACAGGGTTGCTATTATTAAGAGATGGTGGTATTAAGAACCAAACC-------------------------
----

AACGAGACCGAGATCTTCAGACCTGCAGGAGGAGATATGAGGGATAATTGGAGAAGTGAATTATATAAATATA
AAGTAGTACAAATTGAACCATTA---GGAGTAGCACCCACCAGGGCAAAGAGAAGAGTGGTG-----------
----------------------------CAGAGAGAAAAAAGAGCA---GTGGGAACGATAGGAGCT------
---ATGTTCCTTGGGTTCTTGGGAGCA---GCAGGAAGCACTATG---GGCGCAGCGTCA---
ATAACGCTGACGGTACAG---GCCAGACAA---TTGCTG---TCTGGTATAGTGCAACAGCAGAGA---
AATTTG---CTGAGG---GCTATTGAGGCGCAACAGCATCTGTTGCAACTCACAGTCTGGGGCATC
>JN248321
TTCTACTGCAATACATCACAGCTGTTTAATAGTACTTGGCTGTTTAATAGTACTTGGAACAGTACTTGGGGTG
ATACTAGTACT--
----------GAAGGGTCA------
AATGGAACAAAAGACATCGTACTCCCATGCAAAATAAAACAAATTATAAACATGTGGCAGGAAGTAGGAAAAG
CAATGTATGCCCCTCCCATCAGAGGACTAATTAAATGTTCATCAAAC---

ATTACAGGGCTGTTATTAACAAGGGATGGAGGTGAAAATAATGACACA-------------------------
----

AACCAAACTGAGGTCTTCAGACCTGGAGGAGGAGATATGAGGGACAATTGGAGAAGTGAATTATATAAATATA
AAGTAGTAAAAATTGAACCATTA---GGAGTGGCACCCACCAAGGCAAGGAGAAGAGTGGTG-----------
----------------------------CAGAGAGAAAAAAGAGCA---GTGGGA---GTAGGAGCT------
---TTGCTTCTTGGGTTCTTAAGTGCA---GCAGGAAGCACTATG---GGCGCAGCGTCA---
GTGACGTTGACGGTACAG---GCCAGACAA---TTATTG---TCTGGTATAGTGCAACAGCAGAAC---
AATTTG---CTGAAG---GCTATTGATGCGCAACAGCATCTGTTGCAACTCACAGTCTGGGGCATC
>JN248329
TTCTACTGTAATACAGCACAACTGTTTAATAGCACTTGGAATAATAATAGTACTGAGAATGATACTATA----
----

GGAGATGGAACTATCACGCTCCCTTGCAGAATAAGACAAATTGTAAACATGTGGCAGGAAGTAGGAAAAGCAA
TGTATGCCCCTCCCATTGAAGGACAAATTAGATGTTCATCAAAT---
ATTACAGGGTTGCTATTAACAAGAGATGGTGGTAATAATGGGAGC----------------------------
----

AATACCAACGAGACCTTCAGACCTGGAGGAGGAGATATGAGGGACAATTGGAGAAGTGAATTATATAAGTATA
AAGTAGTAAAAATTGAACCATTA---GGAGTAGCACCCACCGAGGCAAGGAGAAGAGTGGTG-----------
----------------------------CAGAGAGAAAAAAGAGCA---GTGGGAGCGATAGGAGCT------
---ATGTTCCTTGGGTTCTTGGGAGCA---GCAGGAAGCACTATG---GGCGCAGCGTCA---
ATAACGCTGACGGTACAG---ACCAGACAA---TTGTTG---TCTGGTATAGTGCAACAGCAGAGA---
AATCTG---CTGAGG---GCTATTGAGGCACAACAACATCTGTTGCAACTCACAGTCTGGGGCATC
>JN248333
TTCTACTGTAATACAACACAACTGTTTAATAGTACTTGGAAGGTTAATAGTACTTGGAATGAGAATAGTACTG
GGGACAAT---
-------------AATGAT------
GAAAATAACAGCACTATCACACTCCCATGCAGAATAAAACAAATTATAAACAGGTGGCAGGAAGTAGGAAAAG
CAATGTATGCCCCTCCTATCAGCGGACTAATTAGCTGTTCATCAAAT---
ATTACAGGGCTGCTACTAACAAGAGATGGTGGGGATGATAGTAAT----------------------------
----

AATACTGAGACCTTCAGACCTGCAGGAGGAGATATGAAAGACAATTGGAGAAGTGAATTATATAAATATAAAG
TAGTAAGAATTGAACCATTA---GGAGTAGCACCCACCAAGGCAAAGAGAAGAGTGGTG--------------
-------------------------CAGAGAGAGAAAAGAGCA---GTGGGAACACTAGGAGCT---------
ATGTTCCTTGGGTTCTTGGGAGCA---GCAGGAAGCACTATG---GGCGCAGCGTCA---
ATAACGCTGACGGTACAG---GCCAGACAA---TTACTG---TCTGGTATAGTGCAACAGCAGAAC---
AATTTG---CTGAGA---GCTATTGAGGCGCAACAGCACATGTTGCAACTCACAGTCTGGGGCATC
>JN248335
TTCTACTGTAATACATCACAACTGTTTAATAGTACTTGGTATGCTAATGGTACTAGTACTGGGAATAATACTA
TA---

AAAAATGGCACTATCACGCTCCCTTGCAGAATAAGACAAATTATAAACATGTGGCAGGAAGTAGGAAAAGCAA
TGTATGCCCCTCCCATTAAAGGACTAATTAGATGTTCATCAAAT---
ATTACAGGGATACTATTAACAAGAGATGGTGGTAGTGGTAATGAGACC-------------------------
----

ACCAATACCACTGAGACCTTCAGACCTACAGGAGGAGATATGAGGGATAATTGGAGAAGTGAATTATATAAAT
ATAAAGTAGTAAAAATTGAACCATTA---GGAGTAGCACCCACCAAGGCAAAGAGAAGAGTGGTG--------
-------------------------------CAGAGAGAAAAAAGAGCA---GTGACA---ATAGGAGCT---
------ATGTTCCTTGGGTTCTTGGGAGCA---GCAGGAAGCACTATG---GGCGCAGCGTCA---
ATAACGCTGACGGTACAG---GCCAGACAA---TTGCTG---TCTGGTATAGTGCAACAGCAGAGA---
AATTTG---CTGAGG---GCGATTGAGGCGCAACAGCATCTGTTGCAGCTCACAGTCTGGGGCATC
>JN248337
TTCTACTGTAATACAACACGACTGTTTAACGGTACCTGGAGTAATGGTACCTGGGTTAATAGCACCTGGAATG
AT---

ACCACTAACGAGATCACCCTCCCATGCAGAATAAAACAAATTGTGAACATGTGGCAGGAAGTAGGAAAAGCCA
TATATGCCCCTCCCATCCAGGGACAAATTAACTGTTCATCAAAT---

ATTACAGGGCTGCTATTAACAAGAGATGGTGGTAATAATAATGATACAAAC----------------------
----

AAGACCCAGGGCGAGATCTTCAGACCTGGAGGAGGAAATATGAAGGACAATTGGAGAAGTGAATTATATAAAT
ATAAAGTAGTAAGAATTGAACCATTA---GGAATAGCACCCACCAAGGCAAGGAGAAGAGTGGTG--------
-------------------------------CAAAGAGAAAAAAGAGCA---GTGGGAACAATAGGAGCT---
------ATGTTCCTTGGGTTCTTGGGAGCA---GCAGGAAGCACTATG---GGCGCAGCGTCA---
ATAACGCTGACGGTACAG---GCCAGACTA---TTGTTG---TCTGGTATAGTGCAACAGCAGAAT---
AATTTG---CTGAGA---GCTATTGAGGCGCAGCAGCACATGTTGCAACTCACAGTCTGGGGCATC
>JN248343
TTCTATTGTAATACAACACAGCTGTTTGAGAGTACTTGGCAGGGTGATAATATTGAAGAGTCAAACAGT----
----

ACTGAACAAAATATCACACTCCCATGCAGAATAAAACAAATTATAAACATGTGGCAGGAAGTAGGAAAAGCAA
TGTATGCCCCTCCCATCGCAGGACCAATTAGATGTTCATCAAAT---
ATTACAGGGTTGCTATTAACAAGAGATGGGGGTAATGTCACTAAC----------------------------
----

GGTACTGAGATCTTCAGACCTGGAGGAGGAGACATGAAGGACAATTGGAGAAGTGAATTATATAAATATAAAG
TAGTAAAAATTGAACCATTA---GGAATAGCACCCACTAAGGCAAAGAGAAGAGTGGTG--------------
-------------------------CAAAGAGAAAAGAGAGCA---GTGGGTATAATAGGAGCT---------
ATGTTCCTTGGGTTCTTGGGAGCA---GCAGGAAGCACTATG---GGCGCAGCGTCA---
ATAACGCTGACGGTACAG---GCCAGACAA---TTATTG---TCAGGTATAGTGCAACAGCAGAAC---
AATTTG---CTGAGG---GCTATTGAGGCGCAACAGCATTTGTTGCAACTCACAGTCTGGGGCATT
>JN248344
TTCTACTGCAATACAACACAGCTGTTTAATACTACTTGGATGCCTAATGGTACTTTGAATGGTACTTGGGAA-
----

CATAACAGCACAATCATACTCCCATGCAAAATAAAACAAATTATAAACAGGTGGCAGGAAGTAGGAAAAGCAA
TGTATGCCCCTCCCATCCAAGGACTAATTAACTGTGCATCAAAT---
ATTACAGGACTGTTATTAACAAGGGATGGTGGTGAGGGTAATGGCACA-------------------------
----

AACCAGACTGCTGAGGTCTTCAGACCTGTAGGAGGAAATATGAAGGACAATTGGAGAAGTGAATTATATAAAT
ATAAAGTAGTAAAAATTGAACCACTA---GGAGTGGCACCCACCAAGGCAAGGAGAAGAGTGGTG--------
-------------------------------CAGAGAGAAAGAAGAGCA---GTGGGA---GTAGGAGCT---
------TTGCTCCTTGGGTTCTTGAGTGCA---GCAGGAAGCACTATG---GGCGCAGCGTCA---
GTGACGCTGACGGTACAG---GCCAGACAA---TTATTG---TCTGGTATAGTGCAACAGCAAAAC---
AATTTG---CTGAGG---GCTATTGATGCGCAACAGCATATGTTGCAGCTCACAGTCTGGGGCATC
>JN248347
TTCTACTGTAATACATCACAACTGTTTAATAGTACTTGGCTGAATAGTACTTGGACTAACACCAGTACTTGGA
AT---
-------------GATACT------
GCAGGAAATGACACTATCACGCTCCCCTGCAGAATAAAACAAATTGTAAACATGTGGCAGGGAGTAGGAAAAG
CAATGTATGCCCCTCCCATCGCAGGAAAAATTAGATGTTCATCAAAT---
ATTACAGGGTTGTTATTAACAAGAGATGGTGGTAATAGCACGAAC----------------------------
----

GAGACCTTCAGACCTGGAGGAGGAGAGATGAGGGACAATTGGAGAAGTGAATTATATAAATATAAAGTAGTAA
AAATTGAACCATTA---GGAGTAGCACCCACCAGGGCAAAGAGAAGAGTGGTG--------------------
-------------------CAGAGAGAAAAGAGAGCG------GGA---ATAGGAGCT---------
GTGTTCCTTGGGTTTTTGGGAGCA---GCAGGAAGCACTATG---GGCGCAGCGTCA---
ATAACGCTGACGGTACAG---GCCAGACAA---TTGTTG---TCTGGTGTAGTGCAGCAGCAGAGA---
AATTTG---CTGAAG---GCTATTGAGGCGCAACAGCATCTGTTGCAACTCACAGTCTGGGGCATC
>JN248353
TTTTACTGTAATACAACAACACTGTTTAATAGTACTTGGACTAATAGTACTTGGACTAGCAACTCAACTGACA
CTGAA--

GGCAATATCACACTCACACTCCCATGCAGAATAAAACAAATTGTAAACATGTGGCAGGAAGTAGGAAAAGCAA
TGTATGCCCCTCCCATCCAAGGAAAGATTACATGTTTATCAAAT---

ATTACAGGGCTGCTATTGACAAGAGATGGTGACAAAAACGAAACAGGG-------------------------
----

GAGACCTTCAGACCTATAGGAGGAAATATTAAGGACAATTGGAGAAGTGAATTATATAAATATAAAGTAGTAC
AGATTGAACCATTA---GGAGTAGCACCCACCCAGGCAAAGAGAAGAGTGGTG--------------------
-------------------CAGAGAGAAAAAAGAGCA---ATAGGGGGAATGGGAGCT---------
ATGTTCCTTGGGTTCTTGGCAGCA---GCAGGAAGCACTATG---GGCGCAGCGTCA---
GTGACGCTGACGGTACAG---GCCAGGCAA---TTATTG---TCTGGTATAGTGCAACAGCAGAAC---
AACCTG---CTGAGG---GCTATTGAGGCGCAACAGCACCTGTTGCAACTCACAGTCTGGGGCATC
>JN248354
TTCTACTGTAATACATCACAACTGTTTAATAGTACTTGGTTTGTCAATGGTACTAATGGTACTACAGTGCCAA
ATAATGAC---

ACTGAAAAAATTATCACACTCCCATGTAAAATAAAACAAATTATAAACATGTGGCAAGAAGTAGGAAAAGCAA
TATATGCCCCTCCCATTGCAGGACCAATTAACTGTTCATCAAAT---
ATTACAGGGCTGCTATTAACAAGAGATGGTGGTAATAATAACGAGACC-------------------------
----

ACCACCAATGAAACCTTCAGACCTGGAGGAGGAGATATGAGGGACAATTGGAGAAGTGAATTATATAAATATA
AAGTAGTAAAAATTGAACCTTTA---GGAATAGCACCCACCAGGGCAAAGAGAAGAGTGGTG-----------
----------------------------CAAAGAGAAAAAAGAGCA---GTAACG---CTAGGAGCT------
---ATGTTCCTTGGGTTCTTGGGAGCA---GCAGGAAGCACTATG---GGCGCAGCGTCA---
ATGACGCTGACGGTACAG---GCCAGACTA---TTATTG---TCTGGTATAGTGCAGCAGCAAAAC---
AATTTG---CTGAGA---GCTATTGAGGCGCAACAGCATATGTTGCAACTCACAGTCTGGGGCATC
>JF297222
TTCTACTGTAATACATCAAAACTGTTTAATAGTGCTTGGCCGCCTAATAGTAATTGGAATAACAGGAATAAC-
----

ACTGGAGGAAATATCACACTCCCATGCAGAATAAAACAAATTATAAACATGTGGCAGAGAGTAGGACAAGCAA
TGTATGCCCCTCCCATCAGTGGAATCATTAACTGTGTATCAAAT---
ATTACAGGGCTAATATTAACAAGAGATGGGGGTAATACTAGTGATACTAAG----------------------
----

AATGACACAGAGATCTTCAGGCCTGCAGGAGGAGATATGAGAGACAATTGGAGAAGTGAGTTATACAGATATA
AAGTAGTAAGAATTGAACCATTA---GGAGTAGCCCCCACCAAGGCAAAAAGAAGAGTGGTG-----------
----------------------------CAGAGAGAAAAAAGAGCA---GTGGGA---ATAGGAGCT------
---GTGTTCATTGGGTTCTTGGGAGCA---GCAGGAAGCGCTATG---GGCGCAGCGTCA---
ATGACGCTGACGGTACAG---GCCAGACAA---TTATTG---TCTGGTATAGTGCAACAGCAAAGT---
AATTTG---CTGAGA---GCTATTGAGGCGCAACAACATATGTTGCAACTCACAGTCTGGGGCATT
>JX446800
TTCTACTGTAATACAACACCATTGTTTAATAGTACCTGGTTTAGTACTGAGAATAGTACCTGGAATAGTAATA
ATAGTATT---

GGGAATGATACTATTATACTCACATGCAGAATAAAACAAATTATAAACATGTGGCAGCAAGTAGGAAAAGCGA
TGTATGCCCCTCCCATCAAGGGACGAATTAACTGTACCTCAAAT---
ATTACAGGACTGATATTAACAAGAGATGGTGGCCATAATAATTCT----------------------------
----

AGCAAGAATGAGACCTTCAGACCTGGAGGAGGAGATATGAGGGACAATTGGAGAAATGAATTATATAAATATA
AAGTAGTAAAAATTGAACCATTA---GGAATAGCACCCACCAAGGCAAAGAGGAGAGTGGTG-----------
----------------------------CAGAGAGAAAAAAGAGCA---ATAGGAACACTAGGAGCT------
---GTGTTCCTTGGGTTCTTGGGAACA---GCAGGAAGCACTATG---GGCGCAGCAGCA---
GTGACGCTGACGGTACAG---GCCAGACAA---TTATTG---TCTGGTATAGTGCAACAGCAGAAC---
AATCTG---CTGATG---GCTATTGAGGCGCAACAGCGTCTGTTGCAACTCACAGTCTGGGGCATC
>JX446818
TTCTACTGTAATACATCAAAATTATTTAATAGTACTTGGAATAATACAAGTAATGACAGTACA----------
----

AGTAATGGCACAATCACACTCCCATGCAGAATAAAACAAATCATAAACATGTGGCAGCAAGTAGGAAAAGCAA
TGTATGCCCCTCCCATCCAGGGACAAATTAACTGTTCATCAAAT---

ATCACAGGGATACTATTAACAAGAGATGGTGGTAATGGTGGTAATACC-------------------------
----

AACGATACAGAGGTCTTCAGACCTGCAGGAGGGGATATGAGGAACAATTGGAGGAGTGAATTATACAAATATA
AAGTAGTACAAATTGAACCAGTA---GGAATAGCACCCACCAAGGCAAGGAGAAGAGTGGTG-----------
----------------------------CAAAGAGAAAAAAGAGCA---GTAACA---CTAGGAGCT------
---GTGTTCCTTGGGTTCTTGGGAACA---GCAGGAAGCACTATG---GGCGCAGCGTCA---
ATAGCGCTGACGGCACAG---GCCAGACAA---TTGTTG---TCTGGTATAGTGCAACAGCAGAAC---
AATCTG---CTGAGG---GCTATTGAGGCGCAACAGCATCTGTTGCAACTCACGGTCTGGGGCATT
>JX447795
TTCTACTGTAATACCTCACAACTGTTTAATAGTACTTGGCATACTAATGATACTTGGAATGGTACAAAGGGT-
----

AACAATGGCACTATCACGCTCCCTTGCAGAATAAAACAAATTATAAACATGTGGCAGGAAGTAGGAAAAGCAA
TGTATGCCCCTCCCATCAAAGGACCGATTGGATGTTTATCAAAC---
ATTACAGGGTTGCTATTAACAAGAGATGGTGGTGGGGGGAACGAGACC-------------------------
----

AGGAACAACGAGACCTTCAGACCTGCAGGAGGAGATATGAGGGACAATTGGAGAAGTGAATTATATAAATATA
AAGTAGTAAGAATTGAACCATTA---GGAGTAGCACCTACCAAGGCAAAGAGAAGAGTGGTG-----------
----------------------------CAGAGAGAAAAAAGAGCA---GTGGGAATGCTAGGAGCT------
---ATGTTCCTTGGGTTCTTGGGAACA---GCAGGAAGCACTATG---GGCGCAGCGTCA---
ATAACGCTGACGGTACAG---GCCAGGCAA---TTGTTG---TCTGGTATAGTGCAACAGCAGAGA---
AATCTG---CTGAGG---GCTATTGAGGCGCAGCAGCACCTGATGCAACTCACAGTCTGGGGCATC
>JX447156
TTCTACTGTGATACATCACAACTGTTTAATAGTACTTGGGATGTTAATAGTACTTGGAATGGTACTGGGAATG
ATACCCCA---

GAAAATAGCACTATCATGCTCCCTTGCAGAATAAAACAAATTGTAAACATGTGGCAGGAAGTAGGAAAAGCAA
TGTATGCCCCTCCTATTGCAGGACAAATTAACTGTTCATCAAAT---
ATTACCGGGTTGCTATTAACAAGAGATGGTGGTGATAATAGCAAT----------------------------
----

ACCAGCGAGACCTTCAGACCTGGAGGAGGAAATATGAAGGACAATTGGAGAAGTGAATTATATAAATACAAAG
TAGTAAAAATTGAACCATTA---GGAGTAGCACCTACCAGGGCTAAGAGAAGAGTGGTG--------------
-------------------------CAGAGAGAAAAAAGAGCA---GTGGGAACGATAGGAGCT---------
ATGTTCCTTGGGTTCTTGGGAGCA---GCAGGAAGCACTATG---GGCGCAGCGTCA---
ATAACGCTGACGGTACAG---GCCAGACAA---TTGTTG---TCTGGTATAGTGCAACAGCAGAGA---
AATTTG---CTGAGA---GCTATTGAGGCGCAACAACATATGTTGCAACTCACAGTCTGGGGCATC
>JF297229
TTCTACTGTAATACATCACAACTGTTTAATAGTACTTGGAATGTTAATGATACTAAGAATTGGAATAAT----
----

ACTGCAGGAAATATCACGCTCATCTGTAGAATAAGACAAATTGTAAACATGTGGCAGGAAGTAGGAAAAGCAA
TGTATGCCCCTCCCATTGCAGGAATAATTAACTGTGTATCAAAT---
ATTACAGGGTTGATTTTAACAAGAGATGGTGGTAAAAATGGTGGTAATGAT----------------------
----

ACAAATAACTTCACTGAGATCTTCAGACCTGGAGGAGGAGATATGAAGGACAATTGGAGAAGTGAATTATATA
AATATAAAGTAGTAAAAATTGAACCATTA---GGAGTAGCACCTACCAAGGCAAAGAGAAGAGTGGTG-----
------------------------------------CAGAGAGAAAAAAGAGCA---GTG------
CTAGGAGCT---------ATGTTCCTTGGGTTCTTGGGAGCA---GCAGGAAGCACTATG---
GGCGCAGCGTCA---ATAACGCTGACGGTACAG---GCCAGACAA---TTGTTG---
TCGGGTATAGTGCAACAGCAGAGA---AATCTG---CTGAGG---
GCTATTGAGCTGCAACAGCATCTGTTGCAACTCACAGTCTGGGGCATC
>MG989620
TTCTACTGTAACACAACAAAACTGTTTAATAGTACTTGGAATAATACTGGGGAGAACATTACTAGTCCTGCAG
AT---

ACAAACGACACTATCATACTCCCATGCAGAATAAAACAAATTGTAAGAATGTGGCAGGAAGAAGGCAAAGCAA

TGTATGCCCCTCCCATCAGAGGAAATATTAGCTGTTCATCAAAT---
ATTACAGGGTTGATATTAACAAGAGATGGCGGGGACGAAGGCAAC-------------------------
----

CAAACCCAAGTAAATGAAACCTTCAGACCTGGAGGAGGAAATATGATGGACAATTGGAGAAGTGAATTATATA
GATATAAAGTAGTAAAAATTGAACCATTA---GGAATAGCACCCACCAAGGCAAAGAGAAGAGTGGTG-----
---------------------------------CAAAGAGAAAGAAGAGCA---
GTGGGAGTAATAGGAGCT---------ATGTTCCTTGGATTCCTAGGAACA---GCAGGAAGCACTATG---
GGCGCAGCGTCA---ATAACGCTGACGGTACAA---GCCAGCAAA---TTATTG---
TCTGGTATAGTGCAACAGCAGAGC---AATCTG---CTGAGA---
GCTATTGAGGCGCAACAGCATCTGTTGCAACTCACGGTCTGGGGCATT
>MG989578
TTCTACTGTAATACAACACCACTGTTTAATAGTATTTGGCATAGTAATAATACTTGGAATAGTAGGACTGGA-
----
-GAGCCAACTAACACCACT------
GGAGGAAATGGCACCATCACACTCCAATGCAGAATAAAACAAATTATAAACATGTGGCAGCAAGTAGGGAAAG
CAATGTATGCCCCTCCCATCAGTGGACCAATTAACTGCTCATCAAAT---
ATTACAGGGCTACTATTACTAAGAGACGGGGGTAACCACAAAGATAGC------------------------
----

AATGACACCGAGATCTTCAGACCGGGAGGAGGAGATATGAGGGACAATTGGAGAAGTGAATTATATAAATATA
AAGTAGTAAAAATTGAACCATTA---GGAGTAGCACCAACCATGGCAAGAAGAAGAGTGGTG-----------
---------------------------CAGAGAGACAAAAGAGCG---GCA------CTAGGAGCG------
---GTGTTCCTTGCGTTCTTGGGAGCA---GCAGGAAGCACTATG---GGCGCAGCGTCA---
TTGACGCTGACGGTACAG---GCCAGACAA---TTGCTG---TCTGGTATAGTGCAACAGCAGAAC---
AATTTG---CTGAGA---GCTATTGAGGCGCAACAGCATATGTTGCAACTCACAGTCTGGGGCATA
>MG989587
TTCTACTGTAATACAACACCACTGTTTAATAGTACTTGGAATTTTACTAGTGCTGGGGAGCTTAATAACACTG
CTAATGAGACT--

AATGATGATAACATCACACTCCAATGCAGAATAAAACAAATTATAAACATGTGGCAGCAAACAGGAAAAGCAA
TGTATGCCCCTCCCATCAAAGGACTAATTGACTGTACATCAAAT---
ATTACAGGGCTACTATTAATAAGAGATGGTGGTAACGAGAGTAGC---------------------------
----

ACAAATGAGACCTTCAGACCAGAAGGAGGAAATATGAAGGACAATTGGAGAAGTGAACTATATAAATATAAAG
TAGTACAAATTGAACCATTA---GGAATAGCACCCACCAAGGCAAAGAGAAGAGTGGTG-------------
------------------------CAGAGAGAAAAAAGAGCA---GTGGGA---ATGGGAGCT---------
TTGTTCCTTACGTTCTTGGGAACA---GCAGGAAGCACTATG---GGCGCAGCGTCA---
TTAACGCTGACGGTACAG---GCCAGACAA---TTATTG---TCTGGTATAGTGCAGCAGCAGAGC---
AATCTG---CTGAAG---GCTATTGAGGCGCAACAGCATATGTTGCAACTCACCGTCTGGGGCATC
>MG989506
TTTTATTGTAATACCTCACAGCTGTTTAATAGTATTTGGAATAGTAGTACTGGGAATAATAATAGTACTGGG-
----

AATAAGAGCACTATCATACTCCCTTGTAGAATAAAACAAATTATAAACATGTGGCAGAAGGTAGGAAAAGCAA
TGTACGCCCCTCCCATCGAAGGAATGATTAACTGTTTATCAAAT---
ATCACAGGGTTGCTGTTAACAAGAGATGGTGGCAGGGTGAATGGGACT------------------------
----

GAGGAAAATAGGACTGAGATCTTCAGACCTGGAGGAGGAGATATGAGGGACAATTGGAGAAGTGAATTATATA
AATATAAAGTAGTAAAAATTGAACCATTA---GGAGTAGCACCCACCAAAGCAAAGAGAAGAGTGGTG-----
---------------------------------CAGAGAGAAAAAAGAGCA---GTAGGA---
ATAGGAGCT---------GTGTTCCTTGGGTTCCTAGGAGCA---GCAGGAAGCACTATG---
GGCGCAGCGTCA---ATAACGCTGACGGTACAG---GCCAGACAA---TTAATG---
TCTGGCATAGTGCAACAGCAAAGA---AATTTG---CTGAGG---
GCTATTGAGGCGCAACAGCATCTGTTGCAACTCACAGTCTGGGGCATC
>MG989541
TTCTACTGTAATACAACACAGCTATTTAATAGTAAGTGGAATGATAGTAATTGGAATAGTACTTGGAATAATA
CTGAAAAG--

TCAAATGATATAATCACACTCCCCTGCAGAATAAAACAGATTATAAACATGTGGCAGGAAGTAGGAAAAGCAA
TGTATGCCCCTCCCATCCAGGGACTAATTAAATGTTCATCAAAC---
ATTACAGGGATACTATTAACAAGAGATGGTGGTAAT-------------------------------------
----
GTGACTTTAGATAATGGGACTTCCAGTAATGAGACTGAGATCTTCAGACCTGCAGGAGGAGATATGAGAGACA
ATTGGAGAAGTGAATTATATAAATATAAAGTAGTAAGAGTTGAACCAGTA---
GGAATAGCACCCACCAGGGCAAAGAGAAGAGTGGTG-------------------------------------
--CAGAGAGAAAAAAGAGCG---GTAACA---CTGGGAGCT---------
GTGTTCCTTGGGTTCTTGGGAACA---GCAGGAAGCACTATG---GGCGCAGCGTCA---
ATAGCGCTGACGGCACAG---GCCAGACAA---TTATTG---TCTGGTATAGTGCAACAGCAAAAC---
AATTTG---CTGAGA---GCTATTGAGGCGCAACAGCACCTGTTGCAACTCACGGTCTGGGGCATC
>MG989610
TTTTACTGTAATACAACACCACTGTTTAATGGGACACTGATTAATGGGACTTGGAATGGTATTGAATGGCCAA
ATAAT--

ACGAATGGAACTATCACACTCCAGTGCAGAGTAAAACAAATTATAAACATGTGGCAAAAAGTAGGAAAAGCAA
TGTATGCCCCGCCCATCAAAGGACTAATTAAATGCTCATCAAAT---
ATTACAGGGCTGATATTAACAAGAGATGGTGGCGGGAATGGGACA----------------------------
----

AATGAGACCTTCAGACCTGCAGGAGGAGATATGAGGGACAATTGGAGAAGTGAATTATATAAGTATAAAGTAG
TAAAACTTGAACCATTA---GGAGTAGCACCCACCAAGGCAAAGAGAAGAGTGGTG-----------------
----------------------CAGAGAGAAAAAAGAGCA---GTGGGA---ATAGGAGCT---------
TTGTTCATTGGGTTCTTGGGAGCA---GCAGGAAGCACTATG---GGCGCAGCGTCA---
CTAACGCTGACGGTACAG---GCCAGACAA---TTGTTG---TCTGGTATAGTGCAACAGCAGAGC---
AATCTG---CTGAGG---GCTATTGAGGCGCAACAGCATATGTTGCAACTCACAGTCTGGGGCATT
>MG989551
TTCTACTGTAATACAACACCACTGTTTAATATTACTAACACCACAAATGACAAT-------------------
----

GACAATAACACAATCATACTCCAATGCAAAATAAAACAGATTATAAATATGTGGCAGAAAGTAGGGAGAGCAA
TGTATGCCCCTCCCATCAGTGGAAAAATTATGTGTTCATCAAAC---
ATTACAGGGCTACTGTTAACAAGAGATGGTGGGAATAATAGGACT----------------------------
----

AATGAGACCTTCAGACCTGCAGGGGGAGATATGAGGGACAATTGGAGAAGTGAATTATATAAATATAAGGTAG
TAAAAATTGAACCTTTA---GGAATAGCACCCACCAAGTCAAGGAGGCTAGTGGTG-----------------
----------------------CAAAGAGAAAAACGAGCA---GTGGGA---ATGGGAGCT---------
ATGTTCCTTGGGTTCTTGGGAGCA---GCAGGAAGCACTATG---GGCGCGGCGTCA---
ATGACGTTGACGGTACAG---GCCAGACAA---TTATTG---TCTGGTATAGTGCAACAGCAGAAC---
AACTTG---CTGAAG---GCTATTGAGGCGCAACAGCACCTGTTGCGACTCACAGTCTGGGGCATC
>U39258
TTCTACTGTAATACATCACAACTGTTTAATGGTACTTGGAATAATACTATA----------------------
----

GAAAATAGCACTATCACGCTCCCTTGCAGAATAAGACAAATTGTAAACATGTGGCAGGAAGTAGGAAAAGCAA
TGTATGCCCCTCCCATCAGAGGACAAATTAGATGTTCATCAAAT---
ATTACAGGGCTGCTATTAACAAGAGATGGTGGTAATAATGGGAGC----------------------------
----

AATACCACCGAGACCTTCAGACCTGGAGGAGGAAATATGAGGGACAATTGGAGAAGTGAATTATATAGATATA
AAGTAGTAAAAATTGAACCATTA---GGAGTAGCACCCACCAAGGCAAAGAGAAGAGTGGTG-----------
----------------------------CAGAGAGAAAAAAGAGCA---GTGGGAACAATAGGAGCT------
---ATGTTCCTTGGGTTCTTGGGAGCA---GCAGGAAGCACTATG---GGCGCAGCGTCA---
ATAACGCTGACGGTACAG---GCCAGACAA---TTGTTG---TCTGGTATAGTGCAACAGCAGAGA---
AATTTG---CTGAGG---GCTATTGAGGCGCAACAGCATCTGTTGCAACTCACAGTCTGGGGCATC
>DQ354116
TTCTACTGTAATACAACAGGACTGTTTAATAGTACTTGGAATAATACTAGTACTGGGAATAATACTACA----
----

GGAAATGACAATATCACGCTCCCTTGCAGAATAAGACAAATTGTAAACATGTGGCAGGAAGTAGGAAGAGCAA
TGTATGCCCCTCCCATCGAGGGACAGATTAGATGTTCATCAAAT---
ATTACAGGGTTGCTATTAACAAGAGATGGTGGTAATGATAAGAAC----------------------------
----

GCGACCACCGAGACCTTCAGACCTGGAGGAGGAGATATGAGGGACAATTGGAGAAGTGAATTATATAAATATA
AAGTAGTAAAAATTGAACCATTA---GGAGTAGCACCCACCAGGGCAAAGAGAAGAGTGGTG-----------
----------------------------CAAAGAGAAAAAAGAGCA---GTGGGAACGATAGGAGCT------
---ATGTTCCTTGGGTTCTTGGGAGCA---GCAGGAAGCACTATG---GGCGCAGCGTCA---
ATAACGCTGACGGTACAG---GCCAGACAA---TTGTTG---TCTGGTATAGTGCAACAGCAGAGA---
AATTTG---CTGAGA---GCTATTGAGGCGCAACAGCATCTGTTGCAACTCACAGTCTGGGGCATC
>DQ354118
TTCTACTGTAATACATCAAAACTGTTTAATAGTACTTGGCATACTAATAGTACTTGGCATACTAATAGTACT-
----

GGGAATGATACTATCACGCTCCCTTGCAGAATAAAACAAGTTGTAAACATGTGGCAAGAAGTAGGAAAAGCAA
TGTATGCCCCTCCCATCAAAGGACCAATTAGATGCTCATCAAAT---
ATTACAGGGTTGCTATTAACAAGAGATGGTGGTAAAAAAGGGAACGAA-------------------------
----

ACCAACGAGACCGAAACCTTCAGACCTGCAGGAGGAAATATGAAGGACAATTGGAGAAGTGAATTATATAAAT
ATAAAGTAGTAAAAATTGAACCATTA---GGAGTAGCACCCACCAGGGCAAAGAGAAGAGTGGTG--------
-------------------------------CAGAGAGAAAAAAGAGCA---GTGGGGACGATAGGAGCT---
------ATGTTCCTTGGGTTCTTGGGAGCA---GCAGGAAGCACTATG---GGCGCAGCGTCA---
ATAACGCTGACGGTACAG---GCCAGACAA---TTGCTG---TCTGGTATAGTGCAACAGCAGAGA---
AATTTG---CTGAGG---GCTATTGAGGCGCAACAGCATCTGTTGCAACTCACAGTCTGGGGCATT
>DQ354119
TTCTACTGTAATTCAACACCGCTGTTTAATGGGACGTTTAATAGTACTGGGAATGGAACTGGAGAGTCACAG-
----

AGCAATGGAAATATCACACTCCAGTGTAGAATAAGACAAATTATAAACAGGTGGCAGGAAGTAGGAAAAGCAA
TGTATGCCCCTCCCATCAGCGGACTAATTAGATGTTCATCAAAT---
ATTACAGGGCTACTATTAACAAGAGATGGTGGAAACGGGAGTACG----------------------------
----

AATGAGACCTTCAGACCTGGGGGAGGAAACATGAGGGACAATTGGAGAAGTGAATTATATAAATATAAAGTAG
TAAAAATTGAGCCATTA---GGGGTAGCACCCACCAAGGCAAAGAGAAGAGTGGTG-----------------
----------------------CAGAGAGAAAAAAGAGCA---GCGATA------GGAGCT---------
ATGTTCCTTGGGTTCTTGGGAGCA---GCAGGAAGCACTATG---GGCGCAGCGTCG---
GTGACGCTGACGGTACAG---GCCAGACAA---TTATTG---TCTGGTATAGTGCAGCAGCAGAAC---
AATCTG---CTGAGG---GCTATTAACGCGCAACAGCATCTGTTGCAGCTCACAGTCTGGGGCATC
>AY945711
TTCTACTGTGATACATCAAAACTGTTTACTTGGAATAGTACTTGGAATAGTACTTGGAATAATAATACTTGGA
ATGAT--
-------------------ACT---
ATAGGAGATAGCAATATCACGCTCCCTTGCAGAATAAAACAAGTTGTAAACATGTGGCAGGAAGTGGGAAAAG
CAATGTATGCCCCTCCTATTGAAGGACAAATTAGATGTTCATCAAAT---
ATTACAGGGTTGCTATTAACAAGAGATGGTGGTAATAATGGAGAC----------------------------
----

AATGACACCGAGATCTTCAGACCTGGAGGAGGAGATATGAGGGACAATTGGAGAAGTGAATTATATAAATATA
AAGTAGTAAAAATTGAACCATTA---GGAGTAGCACCCACCAGGGCAAAGAGAAGAGTGGTG-----------
----------------------------CAGAGAGAAAAAAGAGCA---GTGGGAACAATAGGAGCT------
---ATGTTCCTTGGGTTCTTGGGAGCA---GCAGGAAGCACTATG---GGCGCAGCGTCA---
ATAACGCTGACGGTACAG---GCCAGACAG---CTGTTG---TCTGGTATAGTGCAACAGCAGAGA---
AATTTG---CTGAGG---GCTATTGAGGCGCAACAGCATCTGTTGCAACTCACAGTCTGGGGCATC
>HM215431
TTCTACTGTGACTCAACACCACTGTTTAATAGTACTTGGAAGAATATTACAGAGTTAGATGCTACAAAGTTAG
ATAAC--

-------------------GCT---
ACAGAAAAAGGAAATATCACACTCAAATGTAGAATAAAACAAATTATAAACATGTGGCAGGGAGTAGGAAAAG
CAATGTATGCCCCTCCTATCAGTGGACCAATTAGCTGTTTATCAGAA---
ATTACTGGGCTACTATTAACAAGAGATGGTGGTGTCACTAAGAATAATGAGACC------
AATGGGACCACTGGGAAT---GAT---

AAGACCGAGGTCTTCAGACCTGGAGGAGGAGACATGAGGGACAATTGGAGAAGTGAATTATATAAATATAAAG
TAGTACAAATTAAGCCATTA---GGAATAGCACCCACCAAGGCAAAGAGGAGAGTGGTG--------------
-------------------------CAGAGAGAAAAAAGGGCA---GTGACA---TTTGGAGCT---------
ATGTTCCTTGGGTTCTTGGGAGCA---GCAGGAAGCACTATG---GGCGCAGCGTCA---
ATAGCGCTGACGGGACAG---GCCAGACAA---TTATTG---TCTGGTATAGTGCAACAGCAGAAT---
AATCTG---CTGAGG---GCTATTGAGGCGCAACAGCATCTGTTGCAACTCACTGTCTGGGGCATC
>HM215435
TTCTACTGTAATACATCACAACTGTTTAATAGCACTTGGCTTAATAACAATTCA-------------------
----

ACTAATGGAACTATCATACTCCCATGCAGAATAAAACAATTTATAAACATGTGGCAGGAAGTAGGAAAAGCAA
TGTATGCCCCTCCCATCAGCGGAAATATTAGCTGTTCATCAAAT---
ATTACAGGGCTGCTATTAACAAGAGATGGTGGTAATGGCAAAAACACC-------------------------
----

AACTCCTCTGAAACCTTCAGACCTGGAGGAGGAGATATGAGGGACAATTGGAGAAGTGAATTATATAAATACA
AAGTAGTAAGAATTGAACCACTA---GGAATAGCACCCACCAAGGCAAGGAGAAGAGTGGTG-----------
----------------------------CAGAGAGAAAAAAGAGCG---GTAATA------GGAGCT------
---ATGTTCCTTGGGTTCTTGGGAGCA---GCAGGAAGCACTATG---GGCGCAGCGTCA---
GTGACGCTGACGGTACAG---GCCAGACTA---TTATTG---TCTGGTATAGTGCAACAGCAGAAC---
AATCTG---CTGAGA---GCTATTGAGGCGCAACAGCATATGTTGCAACTCACAGTCTGGGGCATC
>EU839606
TTCTACTGTAATACAACAAAACTGTTTAATAGTACTTGGAATAGTAATGGTATTTGGAATAATACTATAGAGT
CAAATGAC---
-------------------ACA---
GAGTCAAATGATACTATCACACTCCCATGCAGAATAAAACAAATCATAAACATGTGGCAGGGAGTAGGTAAAG
CAACGTATGCCCCTCCCATTAGAGGACAAATTAGATGTTCATCAAAT---
ATTACAGGATTACTATTAACAAGAGACGGTGGTAATAACACAAGT----------------------------
----

GGCACCGAGACCTTCAGACCTGGAGGAGGAGATATGAGGGACAATTGGAGAAGTGAATTATATAAATATAAAG
TAGTAAAAATTGAACCATTA---GGAGTAGCACCCACCAAGGCAAAAAGAAGAGTGGTG--------------
-------------------------CAGAGAGAAAAAAGAGCA---GTGGGAACAATAGGAGCT---------
ATGTTCCTTGGGTTCTTGGGAGCA---GCAGGAAGCACTATG---GGCGCAGCATCA---
CTAACGCTGACGGTACAG---GCCAGACAA---TTATTG---TCTGGTATAGTGCAACAGCAGAAC---
AATCTG---CTGAGG---GCTATTGAGGCGCAACAACATCTGTTGCAACTCACGGTCTGGGGCATC
>EU839607
TTCTACTGTAACACATCACACCTGTTTAACAATACTTGGAATGGTACTGAGACTGATACTTGGAATATTACAG
GG---

GAAGATAGTAATATCACACTTCCATGTAGAATAAAACAAATTGTAAACATGTGGCAGGAAGTAGGTAAAGCAA
TGTATGCCCCTCCCATCAGAGGACAAATTAGCTGTTCATCAAAT---
ATTACAGGGATGCTATTAACAAGAGATGGTGGTAATGGTAATGACACG-------------------------
----

AACAACGAGACTGAGATCTTCAGACCTGCAGGAGGAGATATGAGGGACAATTGGAGAAGTGAATTATATAAAT
ATAAAGTAGTACAAATTGAACCATTA---GGAGTAGCACCCACCAAGGCAAAGAGAAGAGTGGTG--------
-------------------------------CAAAGAGAGAAAAGAGCA---GTGGGAGCAATAGGAGCT---
------ATGTTCCTTGGGTTCCTGGGAGCA---GCAGGAAGCACTATG---GGCGCAGCGTCG---
ATAACGCTGACGGTACAG---GCCAGACAA---CTACTG---TCTGGTATAGTGCAACAGCAGAAC---
AATCTG---CTGAAG---GCTATTGAGGCGCAACAGCATCTGTTGCAACTCACGGTCTGGGGCATC
>HQ217662
TTCTATTGTAATGCAACACAATTGTTTAATAGTACTTGGAGTATTGGGAATGATACTATAGAAAAT-------
----

AGCAATATCAATATCACACTCCCATGCAGAATAAAACAAATTGTAAACATGTGGCAGGAAGTAGGCAAGGCAA
TATATGCCCCTCCCATTAGAGGACAAATTAGATGTTCATCAAAT---
ATTACAGGAATACTATTAACAAGAGATGGTGGTGATACTAATGAC----------------------------
----

ACGACCGAGATCTTCAGACCTGGAGGAGGAGATATGAGGGAAAATTGGAGAAGTGAATTATATAAATATAAAG
TAGTAAGGATTGAACCAATA---GGAGTAGCACCCACCAAGGCAAAGAGGAGAGTGGTG--------------
-------------------------CAAAGAGAAAAAAGAGCA---GTGGGAGCAATAGGAGCT---------
ATGTTCCTTGGGTTCTTGGGAGCA---GCAGGAAGCACTATG---GGCGCAGCGTCA---
CTGACGCTGACGGTACAG---GCCAGACAA---TTATTG---TCTGGTATAGTGCAACAGCAGAAC---
AATCTG---CTGAGG---GCTATTGAGGCGCAACAACATCTGTTGCAACTCACGGTCTGGGGCATC
>EU839608
TTCTACTGTAATACATCCCAACTATTTAATAGTACTTGGAAGAATGGCACTTGGTATAGTAATAGTACTAATA
CTACAGAG---
-------------TCAAGC------
AGAAATATTACACTCATTACACTCCCATGCAGAATAAAACAAATTATAAACATGTGGCAGGCAGTAGGCAAAG
CAATGTATGCCCCTCCCATTAGAGGACCAATTACATGTGTATCAAAC---
ATCACAGGGCTGCTATTAATAAGAGATGGTGGTAGAAACGAGAATGAC-------------------------
----

AATAACACTGAGATCTTCAGACCTGGAGGAGGAAATATGAAGGACAATTGGAGAAGTGAATTATATAAATATA
AAGTAGTAAAAATTGAACCATTA---GGAATAGCACCCACCAAGGCAAAGAGAAGAGTGGTG-----------
----------------------------CAGAGAGAAAAAAGAGCA---GTGGGAACAATAGGAGCT------
---ATGTTCCTTGGGTTCTTGGGAGCA---GCAGGAAGCACTATG---GGCGCAGCGTCA---
ATGACGCTGACGGTACAG---GCCAGACTA---TTATTG---TCTGGTATAGTGCAACAGCAAAAC---
AATTTG---CTGAGG---GCTATTGAGGCGCAACAGCATCTGTTGCAACTCACGGTCTGGGGCATC
>EF593260
TTCTACTGTAATACAACACAGCTGTTTAATAGTACTTGGAATATTAGTAGTACTTGGAATGATACTGGAGGG-
----

ACTGATAGTAACATCACACTCCCATGCAGAATAAAACAAATTATAAACATGTGGCAGCAAGTAGGCAGAGCAA
TGTATGCCCCTCCTATCAGAGGACAAATTAGATGTACATCAAAT---
ATCACAGGGCTGCTATTAACAAGAGATGGTGGTAATGACACCACTAAT-------------------------
----

AATAATACAGAGATCTTCAGACCTGGAGGAGGAAATATGAAGGATAATTGGAGAAGTGAATTATATAAATATA
AAGTAGTAAAAATTGAACCATTA---GGAGTAGCACCCACCAAGGCAAAGAGAAGAGTGGTG-----------
----------------------------CAAAGAGAAAGAAGAGCA---GTGGGAACACTGGGAGCT------
---ATGTTCCTTGGGTTCTTGGGAGCA---GCAGGAAGCACTATG---GGCGCAGCGTCA---
CTGACGCTGACGGTACAG---GCCAGACTA---TTATTG---TCTGGTATAGTGCAACAGCAAAAC---
AATTTG---CTGAGA---GCTATTGAGGCGCAACAGCATATGTTGCAACTCACTGTCTGGGGCATC
>EU578580
TTCTACTGTAATACAACACAGCTGTTTGATAGTACTTGGCTGTTTAATAGTACTTGGAATGATAGTAGTACTG
AGAATGGTACT--
----------ACAAAGCAA------
AATGGCAATGACACACTCACACTTCCATGCAGAATAAAACAAATTGTAAACATGTGGCAGACAGTAGGCAAAG
CAATGTATGCCCCTCCCATCAAAGGACAAATTAGATGTTCATCAAAA---
ATTACAGGCTTGCTATTAACAAGAGATGGTGGTAATAGAGATGATGGT-------------------------
----

AATAACACCGAGATCTTCAGACCTGGAGGAGGAGATATGAGGGACAATTGGAGAAGTGAATTATATAAATATA
AAATAGTAAAAATTGAACCATTA---GGAGTAGCACCCACCAAGGCAAGGAGAAGAGTGGTG-----------
----------------------------CAAAGAGAAAAAAGAGCA---GTGGGAACAATAGGAGCT------
---ATGATCCTTGGGTTCTTGGGAACA---GCAGGAAGCACTATG---GGCGCAGCGTCA---
ATAACGCTGACGGTACAG---GCCAGACAA---TTATTG---TCTGGTATAGTGCGACAGCAGAAC---
AATCTG---CTGAGA---GCTGTTGAGGCGCAACAACATCTGTTGCAACTCACAGTCTGGGGCATC
>EU578603
TTTTACTGTAATACAACAAAGCTGTTTAGTAGTACTTGGCATACTAACCATACTAACGGCACTTGGAATAATA
ATGGG--

-------------------GAT---
AATACTGGAGAGAATATCACACTCCCATGCAGAATAAAACAAATTATAAACATGTGGCAGAGAGTAGGCAAAG
CAATGTATGCCCCTCCCATCAGAGGACAAATTAGATGTACATCAAAT---
ATCACGGGGCTGCTATTAACAAGAGATGGTGGTAATGACAAGAAC----------------------------
----

AGCACCTCCGAGACCTTCAGACCTGGAGGAGGAAATATGAAGGACAATTGGAGAAGTGAATTATATAAATATA
AAGTAGTAAAAATTGAACCATTA---GGAGTAGCACCCACCAAGGCAAAGAGAAGAGTGGTG-----------
----------------------------CAAAGAGAAAAAAGAGCA---GTGGGAGCAATAGGAGCT------
---ATGTTCCTTGGGTTCTTGGGAGCA---GCAGGAAGCACTATG---GGCGCAGCGTCA---
GTGACGCTGACGGTACAA---GCCAGACAA---TTATTG---TCTGGTATAGTGCAACAGCAGAAC---
AATTTG---CTGAGG---GCTATTGAGGCGCAACAGCATATGTTGCAACTCACGGTCTGGGGCATC
>AF277060
TTCTACTGTAATACAACACAACTGTTTAATAGTACTTGGAATATTAATGATACTCGGAATGGTACTACAGAG-
----

TCAAGTAAAACTATCACACTCCCATGCAGAATAAAACAAATTATAAACATGTGGCAGGAAGTAGGCAAAGCAA
TGTATGCCCCTCCCATTAGAGGACAGATTAGATGTTCATCAAAT---
ATTACAGGATTACTATTAACAAGAGACGGTGGTAACCAAAACACG----------------------------
----

AGCGGCACCGAGATTTTCAGACCTGGAGGAGGAAATATGAGAGACAATTGGAGAAGTGAATTATATAAATATA
AAGTAGTAAAAATTGAACCATTA---GGAATAGCACCCACCAAGGCAAAGAGAAGAGTGGTG-----------
----------------------------CAAAGAGAAAAAAGAGCA---GTGGGAACAATAGGAGCT------
---ATGTTCCTTGGGTTCTTGGGAGCA---GCAGGAAGCACTATG---GGCGCAGCATCA---
ATAACGCTGACGGTACAG---GCCAGACTA---TTATTG---TCTGGTATAGTGCAACAGCAGAAC---
AATCTG---ATGAGG---GCTATTGAGGCGCAACAGCATATGTTGCAACTCACAGTCTGGGGCATC
>AF277055
TTCTACTGTAATGCAACACCACTGTTTAATAGTAGCTGGTATGTTAATGGTACTAACACTACAGGGTCA----
----

GATGGAGAAAATATCACACTCCTATGCAGAATAAAACAAATTGTAAACATGTGGCAGGAAGTAGGCAAAGCAA
TGTATGCCCCTCCCATTAGAGGACAAATTACATGCTCATCAAAG---
ATCACAGGGTTGCTATTAACAAGAGATGGTGGTAACACGAACGGGACC-------------------------
----

AACGACACCGAGACCTTCAGACCTGGAGGAGGAGATATGAGGGACAATTGGAGAAGTGAATTATATAAATATA
AAGTAGTAAAAATTGAACCATTA---GGAGTAGCACCCACCAAGGCAAAGAGAAGAGTGGTG-----------
----------------------------CAGAGAGAAAAAAGAGCA---GTGGGAACAATAGGAGCT------
---ATGTTCCTTGGATTCTTGGGAGCA---GCAGGAAGCACTATG---GGCGCAGCGTCA---
CTGACGCTGACGGTACAG---GCCAGACTA---TTATTG---TCTGGTATAGTGCAACAGCAGAAC---
AATCTG---CTGAGG---GCTATTGAGGCGCAACAACATCTGTTGCAACTCACGGTCTGGGGCATT
>EF593245
TTCTACTGTAATTCAACACAACTGTTTAATAGTACTTGGAATAATAATGGTACTAGGAATGATACTAGGGAGT
CA---

AATGACACTATCATCACACTACCATGCAGAATCAAACAAATTATAAACATGTGGCAGCAAGTAGGCAAAGCAA
TGTATGCCCCTCCCATCAGAGGACAAATTAGATGTTCATCAAAT---
ATCACAGGGCTGCTGTTAACAAGAGATGGTGGTAACGAGAGCGAC----------------------------
----

GCTGAGACCTTCAGACCTGGAGGAGGAGATATGAGGGACAATTGGAGAAGTGAATTATACAAATATAAAGTAG
TAAAAATTGAACCATTA---GGAGTAGCACCCTCCAAGGCAAAGAGAAGAGTGGTG-----------------
----------------------CAGAGAGAAAAAAGAGCA---GTGGGAACAATAGGAGCT---------
ATGTTCCTTGGGTTCTTGGGAGCA---GCAGGAAGCACTATG---GGCGCAGCGTCA---
CTGACGCTGACGGTACAG---GCCAGACTA---TTATTG---TCTGGTATAGTGCAACAGCAGAAC---
AATCTG---CTGAGG---GCTATTGAGGCGCAACAACATATTTTGCAACTCACGGTCTGGGGCATT
>AF277070
TTCTACTGTAATACAACACAGCTGTTTAATAGTACTTGGAATGCTAACAGCACTTGGAATGATACTAGAAAG-
----

TCAAATGACACTATCACACTCCCGTGCAGAATAAAACAAATTATAAACATGTGGCAGAGAGTAGGCAGAGCAA
TGTATGCCCCTCCCATCAAAGGACAAATTAGATGTGCATCAAAT---
ATCACAGGGCTGTTATTAACAAGAGATGGTGGTAATGAGAACAGC----------------------------
----

ACCGAGAACATCTTCAGACCTGGAGGAGGAAATATGAGGGACAATTGGAGAAGTGAATTATATAAATATAAAG
TAGTAAAAATTGAACCATTA---GGAGTAGCACCCACCAAGGCAAAGAGAAGAGTGGTG--------------
-------------------------CAGAGAGAAAAAAGAGCA---GTGGGAACAATAGGAGCT---------
ATGTTCCTTGGGTTCTTGGGAGCA---GCAGGAAGCACTATG---GGCGCAGCGTCA---
CTGACGCTGACGGTACAG---GCCAGACTA---TTATTG---TCTGGTATAGTGCAACAGCAGAAC---
AATTTG---CTGAGA---GCTATTGAGGCGCAACAGCATCTGTTGCAACTCACGGTCTGGGGCATC
>AF277072
TTCTACTGTAATACAACAAAACTGTTTAACACAACACTGCTTAATGATACTGCAGAG----------------
----

TCAAATGACAATATCACAATCCCATGCAGAATAAAACAAATTATAAACATGTGGCAGGAAGTAGGTAAAGCAA
TGTATGCCCCTCCCATTAGAGGACAAATTAGATGTTCATCAAAT---
ATCACAGGATTACTATTAACAAGAGACGGTGGTAATGATAATAACACG-------------------------
----

GATAACACCACCGAGATCTTCAGACCTGGAGGAGGAGATATGAGGGACAATTGGAGAAGTGAATTATATAAAT
ATAAAGTAGTACAAATTGAACCAATA---GGAATAGCACCCACCAAGGCAAAGAGAAGAGTGGTG--------
-------------------------------CAAAGAGAAAAAAGAGCA---GTGGGAACAATAGGAGCT---
------ATGTTCCTTGGGTTCTTGGGAGCA---GCAGGAAGCACTATG---GGCGCAGCATCA---
ATAACGCTGACGGTACAG---GCCAGACAA---TTATTG---TCTGGTATAGTGCAGCAGCAGAAC---
AATCTG---CTGAGG---GCTATTGAGGCGCAACAGCATCTGTTGCAGCTCACGGTCTGGGGCATC
>AF277073
TTCTACTGTAATACAACACAGCTGTTTAATAGTACTTGGAATGTTAATGGTACTTGGAATGGTACT-------
----

GGAAGTGAAAATATCACACTCCCATGCAGAATAAAACAAATTATAAACATGTGGCAAAAAGTAGGCAAAGCAA
TGTATGCCCCCCCCATCAAAGGACAAATTAGATGTGCATCAAAT---
ATCACAGGGCTGCTGTTAACAAGAGATGGTGGAAATAGTAGTAATACA-------------------------
----

AGCAACACCGAGATCTTCAGACCTGGAGGAGGAAATATGAAGGACAATTGGCGAAGTGAATTATATAAATATA
AAGTAGTAAAAATTGAACCATTA---GGAGTAGCACCCACCAAGGCAAAGAGAAGAGTGGTG-----------
----------------------------CAGAGAGAAAAAAGAGCA---GTGGGAACAATAGGAGCT------
---ATGTTCCTTGGGTTCTTGGGAGCA---GCAGGAAGCACTATG---GGCGCAGCGTCA---
ATGACGCTGACGGTACAG---GCCAGACTA---TTATTG---TCTGGTATAGTGCAACAGCAGAAC---
AATCTG---CTGAGA---GCTATTGAGGCGCAACAGCATCTGTTGCAACTCACGGTCTGGGGCATC
>EU578429
TTCTATTGTAATACAACACCACTGTTTAATAGTACTTGGTATGAAAATGGTACTTGGAGTGGTACTCCAAAGT
CA---

GATAATGACACTATCACACTCCCATGCAGGATAAAACAAATTATAAACATGTGGCAGAGAGTAGGCAAAGCAA
TGTATGCCCCTCCCATCAGAGGACAAATTAGATGTTCATCAAAT---
ATCACAGGGCTGTTATTAACAAGAGATGGTGGTAGTAGTAATAAC----------------------------
----

AGCACCGAGACCTTCAGACCTGGAGGAGGAGATATGAGGGACAATTGGAGAAGTGAACTATATAAATATAAAG
TAGTAAAAATTGAACCATTA---GGAATAGCACCCACCAAGGCAAAGAGAAGAGTGGTG--------------
-------------------------CAAAGAGAAAAAAGAGCA---GTGGGAACAATAGGAGCT---------
ATGTTCCTTGGGTTCTTGGGAGCA---GCAGGAAGCACTATG---GGCGCAGCATCA---
CTAACGCTGACGGTACAG---GCCAGACTA---TTATTG---TCTGGTATAGTGCAACAGCAGAAC---
AATCTG---CTGAGG---GCTATTGAGGCGCAACAGCACCTGTTGCAACTCACAGTCTGGGGCATC
>EU578386
TTCTACTGTAATTCAACACAACTGTTCAATAGTACTTGGAATGGGAATAATACTTGGAATGGTACT-------
----

GGGGAGGATACTATCACACTCCCATGCAGAATAAAACAAATTGTAAATATGTGGCAGAGAGTAGGCAAAGCAA
TGTATGCCCCTCCCATCAAAGGACAAATTAGATGTTCATCAAAC---
ATTACAGGGCTGCTATTAACAAGGGATGGTGGTACTAACGGGACC----------------------------
----

ACCGAGACCTTCAGACCTGGAGGAGGAAATATGAAGGACAATTGGAGAAGTGAATTATATAAATATAAAGTAG
TAAAAATTGAACCATTA---GGAATAGCACCCACCAAGGCAAAGAGAAGAGTGGTG-----------------
----------------------CAGAGAGAAAAAAGAGCA---GTGGGAGTAATAGGAGCT---------
CTGTTCCTTGGGTTCTTGGGAGCA---GCAGGAAGCACTATG---GGCGCAGCGTCA---
CTGACGCTGACGGTACAG---GCCAGACAA---TTATTG---TCTGGTATAGTGCAACAGCAGAAC---
AATCTG---CTGAGG---GCTATTGAAGCGCAACAGCATCTGTTGCAACTCACGGTCTGGGGCATT
>EU577148
TTCTACTGTAATTCAACACAACTGTTTAACAGTACTTGGAATGCTACTGATACTTGGAAATGGAATGATAGTA
ATACT--
-------------------TGG---
TATAATTCCAGCAATATCACACTTCCATGTAGAATAAAGCAAATTATAAACATGTGGCAGAGAGTAGGCAAAG
CAATGTATGCCCCTCCCATCAGAGGACAAATTAACTGTACATCCAAT---
ATCACAGGGCTGCTATTAACAAGAGATGGTGGTGATAACGAGAAC----------------------------
----

GACACCGAGACCTTCAGACCTGGAGGAGGAGATATGAGGAACAATTGGAGAAGTGAATTATATAGATATAAAG
TAGTAAAAATTGAACCATTA---GGAGTAGCACCCACCAAGGCAAAGAGAAGAGTGGTG--------------
-------------------------CAGAGAGAAAAACGAGCG---GTGGGAACACTAGGAGCT---------
ATGTTCCTTGGGTTCTTGGGAGCA---GCAGGAAGCACTATG---GGCGCAGCGTCG---
CTGACGCTGACGGTACAG---GCCAGACTA---TTACTG---TCTGGTATAGTGCAGCAGCAAAAT---
AATTTG---CTGAGG---GCTATTGAGGCGCAACAGCATCTGTTGCAACTCACGGTCTGGGGCATC
>EF593264
TTCTACTGTAATACAACACAACTGTTTAATAGTACTTGGAATAATAGTACTTGGAATGGTATTGACGAGTCAG
GT---

GATGATGACAATATCACACTACCATGCAGAATAAAACAAATTATAAACATGTGGCAGCAAGTAGGCAAAGCAA
TGTATGCCCCTCCCATCAGAGGACAAATTAGCTGTTCATCAAAT---
ATCACAGGGCTGCTGTTAACAAGAGATGGTGGTGGTAACAGTAGC----------------------------
----

AACAATGAGACCTTCAGACCTGGAGGAGGAGATATGAGGGACAATTGGAGAAGTGAATTATATAAATATAAAG
TAGTAAAAATTGAACCATTA---GGAGTAGCACCCACCAAGGCAAAGAGAAGAGTGGTG--------------
-------------------------CAGAGGGAAAAAAGAGCA---GTGGGAACAATAGGAGCT---------
ATGTTCCTTGGGTTCTTGGGAGCA---GCAGGAAGCACTATG---GGCGCAGCGTCA---
GTGACGCTGACGGTACAG---GCCAGACTA---CTATTG---TCTGGGATAGTGCAACAGCAGAAC---
AATCTG---CTGAGG---GCTATTGAGGCGCAACAGCACCTGTTGCAACTCACAGTCTGGGGCATC
>EF593268
TTCTACTGTAATTCAACAGCACTGTTTACTGGTACTTGGAATGGTACTACTTGGAATGGTACTACTTGGAATG
GTACTGAGCCT--
-GATACTGAAGAGTCA---------
AATAACACCACCACCATCATACTCCCATGCAGAATAAAACAAATTGTAAACATGTGGCAGGAGGTAGGCAAAG
CAATGTATGCCCCTCCCATCAGAGGACAAATTAACTGTTCATCAAGT---
ATTACAGGGCTGCTATTAACATGGGATGGTGGTAATAACACGGACCAG-------------------------
----

AACATGACCGAGACCTTCAGACCTGGAGGAGGAAATATGAGGGACAATTGGAGAAGTGAACTATATAAATATA
AAGTAGTAAAAATTGAACCATTA---GGAATAGCACCCACCAAGGCAAAGAGAAGAGTGGTG-----------
----------------------------CAAAGAGAAAAAAGAGCA---GTGGGAACAATAGGAGCT------
---ATGTTCCTTGGGTTCTTGGGAGCA---GCAGGAAGCACTATG---GGCGCAGCGTCA---
CTAACGCTGACGGTACAG---GCCAGATTA---TTATTG---TCTGGTATAGTGCAACAGCAGAAC---
AATTTG---CTGAGG---GCTATTGAGGCGCAACAGCATCTGTTGCAGCTCACGGTCTGGGGCATC
>U32396
TTCTACTGTAATTCAACACAACTGTTTAATAGTACTTGGAATGCTAATGGTAATGGGAATGGTACTGGAGGAT
CA---

AATGACAATGACACTATCACACTCCCATGCAGAATAAAACAAATTGTAAACATGTGGCAAGAAGTAGGCAAAG
CAATGTATGCCCCCCCCATCAGAGGATACATTAACTGTTCATCAAAT---
ATTACAGGGCTGTTATTAACAAGAGATGGTGGTATTGGCACGAAC----------------------------
----

GGGACCGAGATATTCAGACCTGGAGGGGGAGATATGAGGGACAATTGGAGAAGTGAATTATATAAATATAAAG
TAGTAGAAATCGAACCATTA---GGAGTAGCACCCACCAAGGCAAAGAGAAGAGTGGTG--------------
-------------------------CAGAGAGAAAAAAGAGCA---GTGGGAACAATAGGAGCT---------
ATGTTCCTTGGGTTCTTGGGAGCA---GCAGGAAGCACTATG---GGCGCAGCGTCA---
CTGACGCTGACGGTACAG---GCCAGACTA---TTATTG---TCTGGTATAGTGCAACAGCAGAAC---
AATCTG---CTGAGG---GCTATTGAGGCGCAACAGCATCTGTTGCAGCTCACGGTCTGGGGCATC
>AF086817
TTCTACTGTAATGCAACACCACTGTTTAATAGTACTTGGAATGCTACCAGTACTTTGAATGCTACTAATGAA-
----

GAAAATGAAAATATCACACTCCTATGTAGAATAAAACAAATTATAAACATGTGGCAGAGAGTAGGAAAAGCAA
TGTATGCTCCTCCCATCGAAGGACTAATTAAATGTTCATCAAAT---
ATTACAGGGCTGATGTTAACAAGAGATGGGGGTACAAATGACAGT----------------------------
----

GAGGTCGAGGTCTTCAGACCTGGAGGAGGAGATATGAAGGACATTTGGAGAAATGAATTATATAAATATAAAG
TAGTAAAAGTTGAACCATTA---GGACTAGCACCCACCAGGGCAAGGAGAAGAGTGGTG--------------
-------------------------CAGAGAGAAAAAAGAGCA---GTGGGA---ATAGGAGCT---------
TTGTTCCTTGGGTTCTTGGGAGCA---GCAGGAAGCACTATG---GGCGCAGCGTCA---
CTAACGCTGACGGTACAG---GCCAGACAA---TTATTG---TCTGGTATAGTGCAACAGCAGAAC---
AATCTG---CTGAGA---GCTATTGAGGCGCAACAGCACATGTTGCAACTCACAGTCTGGGGCATC
>DQ823362
TTCTATTGTAATACATCAAAGCTGTTCAATAGTACTTGGAATAATGGTACTTGGACGAGTAATATTGATGAGG
GTAAT--
-------------------ACT---
ACAGGGCCAGACCACATCACACTCCCATGCAGAATAAAACAAATTATAAACAGATGGCAGGAAGTAGGAAAAG
CAATATATGCCCCTCCCATCAGCGGACTAATTAGATGTTCATCAAAT---
ATTACAGGGATACTATTAGTAAGAGATGGTGGTAATAATAGTAGT----------------------------
----

AACAACGAGACTTTCAGACCTGGAGGAGGGAATATGAAGGACAATTGGAGAAGTGAATTATATAAATATAAAG
TAGTAAAAATTGAGCCATTA---GGAGTAGCACCCACCAAGGCAAAGAGAAGAGTGGTG--------------
-------------------------CAGAGAGAAAAAAGAAGT---CCAACG---CTTGGAGCT---------
CTGTTCCTTGGGTTCTTGGGAGCA---GCAGGAAGCACTATG---GGCGCAGCGTCA---
ATAACGCTGACGGTACAG---GCCAGACAA---TTATTG---TCTGGTATAGTGCAACAGCAGAAC---
AACTTG---CTGAGA---GCTATTGAGGCGCAACAGCATCTGTTGCAACTCACAGTCTGGGGCATT
>DQ823363
TTCTATTGTAATACAACACAGCTGTTCAATAGTACTTGGAATAATACTTGGAGTGGTGCTACA----------
----

GGATCAGACAACATCACACTCCCGTGCAGAATAAAACAAATTATAAACAGGTGGCAGGAAGTAGGAAAAGCAA
TGTATGCCCCTCCCATCGAAGGACAAATTAAATGTTCATCAAAT---
ATTACAGGGCTATTATTAGTAAGAGATGGTGGTATTGGTAATAAT----------------------------
----

GAAACAGAGATCTTCAGACCTGGAGGAGGAGATATGAAGGACAATTGGAGAAGTGAATTATATAAATATAAAG
TAGTAAAAATTGAACCATTA---GGAGTAGCACCCACCAGGGCAAAGAGAAGAGTGGTG--------------
-------------------------CAAAGAGAAAAAAGAGCA---GTAACG---TTTGGAGCT---------
ATGTTCCTTGGGTTCTTGGGAGCA---GCAGGAAGCACTATG---GGCGCAGCGTCA---
ATAACGCTGACGGTACAG---GCCAGACAA---TTATTG---TCTGGTATAGTGCAACAGCAGAGT---
AATTTG---CTGAGA---GCTATTGAAGCGCAACAGCATATGTTGCAACTCACAGTCTGGGGCATT
>DQ823364
TTCTATTGTAATACAACACAGCTGTTCAATAGTACTTGGCAGGCTAATAGTACTAGGAATGTTAGTACAACAT
CAGAC--

-------------------AAC---
TCAGACAACAGCACAATCACACTCCCGTGCAGAATAAAACAAATTATAAACAGGTGGCAAGAAGTAGGAAAAG
CAATGTATGCCCCTCCCATCAGAGGACAAATTAAATGTTCATCAAAT---
ATTACAGGGCTACTATTAGTAAGAGATGGTGGTAATGGTAATAATAGT------------------------
----

AGTAACGAGACAGAGATCTTCAGACCTGGAGGAGGAAATATAAAGGACAATTGGAGAAGTGAATTATATAAAT
ATAAAGTAGTAAAAATTGAGCCGTTA---GGAGTAGCACCCACCAGGGCAAAGAGAAGAGTGGTG-------
------------------------------CAGAGAGAAAAAAGAGCA---ATAACA---TTTGGAGCT---
------ATGTTCCTTGGGTTCTTGGGAGCA---GCAGGAAGCACTATG---GGCGCAGCGTCA---
GTGACGCTGACGGTACAG---GCCAGACAA---TTATTG---TCTGGTATAGTGCAACAGCAGAGC---
AATTTG---CTGAGA---GCTATTGAGGCGCAACAGCATATGTTGCAACTCACAGTCTGGGGCATT
>JN562768
TTCTACTGTAATACATCACAACTGTTTAACAGTACTTCTAATGTTACTTGGACTACTGGAGAGTCA------
----

GATAACAAAACAATCACACTCCCATGCAGAATAAAGCAAATTATAAACATGTGGCAGAAAGTAGGAAAAGCAA
TGTATGCCCCTCCCATCCAAGGACTAATTAAATGTTCATCAAAT---
ATTACAGGGCTGATATTAACAAGAGATGGTGGTGTTAATATGACAAATGGTGAT------GTTAAT------
----
GGGACAAACAACAAC---
AACACAGAGACCTTTAGACCTGCAGGAGGAGATATGAGGGACAATTGGAGAAGTGAATTATATAAGTATAAAG
TAGTAAGAATTGAACCATTA---GGAGTAGCACCCACCAAGGCAAAGAGAAGAGTGGTG-------------
------------------------CAAAAAGAAAAAAGAGCA---GTGGGA---ATAGGAGCT---------
CTGTTCCTTGGGTTCTTGGGAACA---GCAGGAAGCACTATG---GGCGCAGCGTCA---
ATGGCGCTGACGGTACAG---GCCAGACAA---TTATTG---TCTGGTATAGTGCAACAGCAGAAC---
AACTTG---CTGAGG---GCTATTGAGGCGCAACAACATCTGTTGCAACTCACAGTCTGGGGCATC
>DQ869026
TTCTACTGTAATTCAACACAACTGTTTAATAGCACTTGGTATAGCAATAATACTTGGGAAGGGTTAAATAGC-
----

ACTGAAGCAAATATCACACTCCCATGCAGAATAAAACAAATTGTGAATATGTGGCAGCAAGTGGGAAAAGCAA
TGTATGCCCCTCCCATCAGCGGACGAATTAGCTGCACATCAAAT---
ATTACAGGGCTGTTATTAACAAGAGATGGGGGTGGTAATAGCACAAAC------------------------
----

GAGACCACCGAGATCTTCAGACCTGGAGGAGGAGATATGAGGGACAATTGGAGAAGTGAATTATATAAATATA
AAGTAGTAGAAATTGAACCATTA---GGAGTAGCACCCACCAAGGCAAGGAGAAGAGTGGTG----------
---------------------------CAGAGAGAAAAAAGAGGA---GTA------TTAGGAGCT------
---ATGTTCCTTGGGTTCTTGGGAGCA---GCCGGAAGCACTATG---GGCGCAGCGTCA---
CTGACGCTGACGGTACAG---GCCAGACAA---TTATTG---TCTGGTATAGTGCAACAGCAGAAC---
AATCTG---CTGAGG---GCTATTGAGGCGCAACAGCATATGTTGCAACTCACAGTCTGGGGCATT
>EF363123
TTCTACTGTAATACAACAAAACTATTTAATAGTACTTGGAACAATGGTACTTGGACTTGGAACAATGGTACTT
GGAATGGT--
-------------ACTGAA------
TGGTTAAATGACACTATCACACTCCCATGCAGAATAAAACAAATTATAAACATGTGGCAGGAAGTAGGAAAAG
CAATGTATGCCCCTCCCATCAGAGGACAAATTAGATGTTCATCAAAT---
ATTACAGGGCTGCTATTAACAAGAGATGGTGGTAATACTGAGAAC---------------------------
----

AAGACCGAGGTCTTCAGACCTGGAGGAGGAAATATGAAGGACAATTGGAGAAGTGAATTATATAAATATAAAG
TAGTAAAAATTGAACCACTA---GGAGTAGCACCCACCAAGGCAAAGAGAAGAGTGGTG-------------
------------------------CAGAGAGAAAAAAGAGCA---GTGGGA---ATAGGAGCT---------
ATGTTCCTTGGGTTCTTGGGAGCA---GCAGGAAGCACTATG---GGCGCAGCGTCA---
ATGACGCTGACGGTACAG---GCCAGACAA---TTATTG---TCTGGTATAGTGCAACAGCAAAGC---
AATTTG---CTGAGG---GCTATTGAGGCGCAACAAAAACTGTTGCAACTCACAGTCTGGGGCATC
>EU289199
TTCTACTGTAATACAACAGGACTGTTTAATAGTACTTGGTATAATGAGACTGAAAAGATAAATAGCACTACA-
----

AGTGACAATATCACAACACTTCCATGCAGAATAAAACAAATTGTAAACATGTGGCAGGAAGTAGGAAAAGCAA
TGTATGCCCCTCCCATCAGAGGACAAATTAACTGTTCATCAAAT---
ATCACAGGGATGATATTAACAAGAGATGGTGGTAATAATAATAACAGT-------------------------
----

AGCAATGACACGGAGATCTTCAGACCTGGAGGAGGAGATATGAGGGACAATTGGAGAAGTGAATTATATAAAT
ATAAAGTAGTACAAATTGAACCATTA---GGAGTAGCCCCCACCACGGCAAAGAGAAGAGTGGTG--------
-------------------------------CAGAGAGAAAAAAGAGCA---GTGGGA---CTAGGAGCT---
------ATGTTCCTTGGGTTCTTGGGAGCA---GCAGGAAGCACTATG---GGCGCAGCGTCA---
ATAACGCTGACGGTACAA---GCCAGACAA---TTATTG---TCTGGTATAGTGCAGCAGCAGAGC---
AATCTG---CTGAGG---GCTATTGAGGCGCAACAGCATCTGTTGCAACTCACAGTCTGGGGCATT
>JN944917
TTCTACTGTAATACAACAAAATTGTTTACTAGTACTTGGAATAGTACTTGGAATAGTACTTGGAATAATACTG
AAGGGTCA---

AATAGCACTGTTATCACCCTGCCATGCAGAATAAGACAAATCATAAACATGTGGCAGGAAGTAGGAAAAGCGA
TGTATGCCCCTCCCATCCAAGGACAAATTAAATGTTCATCAAAT---
ATTACAGGGCTGTTATTAACAAGAGATGGTGGTAATAACGACACC----------------------------
----

ACAAAAGAGACCTTCAGACCTGGAGGAGGAAATATGAAGGACAATTGGAGAAGTGAATTATACAAATATAAAG
TAGTAAGAATTGAGCCATTA---GGAGTAGCCCCCACCAAGGCAAAAAGAAGAGTGGTG--------------
-------------------------CAGAGAGAAAAAAGAGCA---GTGGGA---ATAGGAGCT---------
ATGTTCCTTGGGTTCTTAGGAGCA---GCAGGAAGCACTATG---GGCGCAGCGTCA---
ATAACGCTGACGGTACAG---GCCAGACTA---CTATTG---TCTGGTATAGTGCAACAGCAGAGC---
AATCTG---CTGAGG---GCTATTGAGGCGCAACAGCATCTGTTGCAACTCACAGTCTGGGGCATC
>JN944930
TTCTACTGTAATACAACAAACCTGTTCAATAGTAGTTCAACAGAACTTAATAGTACTTGGAGTGGTAATAGTA
AT---
-------------GACACA------
GGAAAAAATGATACAATCACACTCCCATGCAGAATAAAACAAATTATAAACATGTGGCAGCAAGTAGGAAAAG
CAATGTATGCCCCTCCCATCAGTGGAAAAATTAACTGTTTATCAAAT---
ATTACAGGGCTGCTATTAACAAGAGATGGGGGTAGTGATGGGGGTAGT-------------------------
----
AAGAACAGTAGTAAGAATGAGACC---------
GGTACTGAGATCTTCAGACCTGGAGGAGGAGATATGAGGGACAATTGGAGAAGTGAATTATATAAATATAAAG
TAGTAAGAATTGAACCATTA---GGAGTAGCACCCACCAAGGCAAAGAGGAGAGCGGTA--------------
-------------------------CAGAGAGAAAAAAGAGAT---CTGGGA---CTAGGAGCT---------
TTGTTCCTTGGGTTCTTGGGAGCA---GCAGGAAGCACTATG---GGCGCAGCGTCA---
GTGACGCTGACGGTACAG---GCCAGACAG---TTATTG---TCTGGTATAGTGCAACAGCAGAAC---
AATCTG---CTGAGA---GCTATTGAGGCGCAACAGCACCTGTTGCAACTCACAGTCTGGGGCATC
>JN944938
TTCTACTGTAATTCAACACAACTGTTTAATAGTACTTGGTTTAATAATGGTACTAGTACTTGGAATAGT----
----

ACTGCAGACAATATCACACTCCCATGTAGAATAAAACAAGTTATAAACATGTGGCAGGAAGTAGGAAAAGCAA
TGTATGCCCCTCCCATCAGAGGACAAATTGATTGTTCATCAAAT---
ATTACAGGGTTGATATTAACAAGAGATGGTGGTAGTAACAGTAGC----------------------------
----

CAGAACGAAACCTTCAGACCTGGAGGAGGAAATATGAAGGACAATTGGAGAAGTGAATTATATAAATATAAAG
TAGTAAAAATTGAACCATTA---GGAATAGCACCCACCAGGGCAAAGAGAAGAGTGGTG--------------
-------------------------CAGAGAGAAAAAAGAGCA---GTGACG---CTGGGAGCT---------
GTGTTCCTTGGGTTCTTGGGAGCT---GCAGGAAGCACTATG---GGCGCAGCGTCA---
CTGACGCTGACGGTACAG---GCCAGACTA---TTATTG---TCTGGTATAGTGCAACAGCAGAGC---
AATCTG---CTGAGA---GCTATTGAGGCGCAACAACATATGTTGCAACTCACAGTCTGGGGCATT
>EF593288
TTCTACTGTAATACAACACAACTGTTTAATAGTACTTGGAATTCTAATGATACTTGGAATAGTACTACAGGGT
CAAAT--

GGCACTGGAGGAAATATCACACTCCCATGCAGAATAAAACAAATTATAAACATGTGGCAGAAAGTAGGAAAAG
CAATGTATGCCCCTCCCATCAAAGGACAAATTAAATGTTTATCAAAT---
ATTACAGGGATACTATTAACAAGAGATGGGGGTAATGAGAACAGC----------------------------
----

ACTATTGAGACCTTCAGACCTGCAGGAGGAGATATGAGGGACAATTGGAGAAGTGAATTATATAAATATAAAG
TAGTACAAGTTGAACCATTA---GGAGTAGCACCCACCAAGGCAAAGAGAAGAGTGGTG--------------
-------------------------CAAAGAGAAAAAAGAGCA---ATAGTGGGAATAGGAGCT---------
GTGTTCCTTGGTTTCTTGGGAGCA---GCAGGAAGCACTATG---GGCGCAGCGTCA---
TTGACGCTGACGGTACAG---GCCAGACAA---TTATTG---TCTGGTATAGTGCAACAGCAGAAT---
AATCTG---CTGAGG---GCTATTGAGGCGCAACAACATATATTGCAACTCACAGTCTGGGGCATC
>EF593289
TTCTACTGTAATACAACACAGCTGTTTAATAGTACTTGGAATAGTACTAATTGGAATAATACTGAAAAGTCAA
AT---

AGCACTGAAGTCATCACACTCCCATGCAGAATAAAACAAATTATAAACATGTGGCAGAAGGTAGGAAAAGCAA
TGTATGCCCCTCCCATTGCAGGACAAATTAACTGTTCATCAAGT---
ATTACAGGACTGATATTAACAAGAGATGGTGGTGACAACAGCACA----------------------------
----

AACAATACAGAGATCTTCAGACCTGTAGGAGGAGATATGAGGGACAATTGGAGAAGTGAATTATATAAATATA
AAGTAGTAAGAATTGAACCATTA---GGAGTAGCGCCCACCAGGGCAAGGAGAAGAGTGGTG-----------
----------------------------CAGAGAGAAAAAAGAGCA---ATAGGA---CTAGGAGCT------
---GTGTTCATTGGGTTCTTGGGAGCA---GCAGGAAGCACTATG---GGCGCAGCGTCG---
GTGACGCTGACGGTACAA---GCCAGACAA---TTACTG---TCTGGTATAGTGCAACAGCAGAAC---
AATCTG---CTGAAG---GCTATTGAGGCGCAACAGCATATGTTGCAACTCACAGTCTGGGGCATT
>HM769944
TTCTACTGTAATACAACAGAACTGTTTAGTAGCACTTGGAATATAACAGGAGATAGCATTGGGAATATAACAG
GA---

GAGTCTTTAAATATCACACTCCCATGCCGAATAAAACAAATTATAAACATGTGGCAGGGAGTAGGAAAAGCAA
TGTATGCCCCTCCCATCAGTGGACAGATTAGATGCATATCAAAT---
ATTACAGGGCTGCTATTAACAAGAGATGGTGGTGATAATAACACCGAGAAT----------------------
----

GACAACAACACCGAAATCTTCAGACCTTGGGGAGGAGATATGAGGGATAATTGGAGAAGTGAATTATATAAAT
ATAAAGTAGTAAAACTTGAACCATTA---GGATTAGCACCCACCAAGGCAAAGAGAAGAGTGGTG--------
-------------------------------CAAAGAGAAAAAAGAGCA---ATAGGA---GTTGGAGCT---
------ATGTTCCTTGGGTTCTTGGGAGCA---GCAGGAAGCACTATG---GGCGCAGCGTCA---
CTGACGCTGACGGTACAG---GCCAGGCAA---TTATTG---TCTGGTATAGTGCAACAGCAGAAC---
AATCTG---CTGAGG---GCTATTGAGGCGCAACAGCATCTGTTGCAACTCACAGTCTGGGGCATC
>JQ609746
TTCTACTGTAATACAACACAACTGTTTAATAGCACTTGGCATGGGAATGAGACTGAAGAATCAAATACCACCA
AAGAC--

AATAGCACAATAATCACTCTCCCATGCAGAATAAGACAAATTGTAAACCGGTGGCAGGAAGTAGGGAAAGCAA
TGTATGCCCCTCCCATCAGCGGACGGATTAACTGTTCATCAAAT---
ATTACTGGGCTGCTATTAACAAGAGATGGTGGTAATATCACGACC----------------------------
----

GAGAATACTACTGAGACCTTCAGACCTGGAGGAGGAGATATGAGGGACAATTGGAGAAGTGAATTATATAAAT
ATAAAGTAGTAAAAATTGAACCATTA---GGAATAGCACCCACCAGGGCAAAGAGAAGAGTGGTG--------
-------------------------------CAAAGAGAAAAGAGAGCA---GTGGGA---ATAGGAGCT---
------GTGTTCCTTGGGTTCTTGGGAGCA---GCAGGAAGCACTATG---GGCGCAGCGTCA---
CTGACGCTGACGGTACAG---GCCAGACTC---TTATTG---TCTGGTATAGTGCAACAGCAGAAT---
AATTTG---CTGAGA---GCTATTGAGGCGCAACAACATCTGTTGCAACTCACAGTCTGGGGCATC
>DQ869017
TTCTACTGTAATACAGCACAATTGTTTAATAGTACTTGGAAGAGGGATGGTACTACTGGGACCTGGAGTGGTA
ATAATACTGAG--

-------AATGATACTGAA------
CATTCAAATGACACTATCACACTCCCATGCAGAATAAGACAAATTGTAAACATGTGGCAAGAAGTAGGAAAAG
CCATGTATGCTCCTCCCATCAGTGGACAAATTAGATGTTCATCAAAT---
ATTACAGGGTTACTATTAACAAGAGATGGTGGTGAGAGCAATGCC----------------------------
----

ACTGAGGTCTTCAGACCCGCAGGAGGAGATATGAGGGACAATTGGAGAAGTGAACTATATAAATATAAAGTAG
TAAAAATTGAACCATTA---GGAATAGCACCTTCCAAGGCAAAGAGAAGAGTGGTG-----------------
----------------------CAGAGAGAAAAAAGAGCA---GTGGGA---ATAGGAGCT---------
GTGTTCCTTGGGTTCTTAGGAGCT---GCAGGAAGCACTATG---GGCGCAGCATCC---
TTAACGCTGACGGTACAG---GCCAGACTA---TTATTG---TCTGGTATAGTGCAACAGCAAAAC---
AATTTG---CTGAGA---GCTATTGAGGCGCAACAGCATCTGTTGCAACTCACAGTCTGGGGCATT
>DQ869019
TTCTACTGTAATACAACACAATTGTTTAATGGTACTTGGAATAGTACTTGGAGTAGTACTGTA----------
----

GAAAATGGCACAATCACACTCCCATGCAAAATAAAACAGATTATAAACATGTGGCAGGAAGTAGGAAAAGCAA
TGTATGCCCCTCCCATCAGAGGACAAATTAACTGTACATCAAAT---
ATTACAGGGCTGCTATTAACAAGAGATGGTGGTAATAATAATAATACT-------------------------
----

GCTAATGAAACCGAGATCTTCAGACCTGGAGGAGGAGATATGAGGGACAATTGGAGAAGCGAATTGTATAAAT
ATAAAGTAGTAAAAATTGAACCATTA---GGACTAGCACCCACTAAGGCAAAGAGAAGAGTGGTG--------
-------------------------------CAAAGAGAAAAAAGAGCA---GTGGGA---CTAGGAGCT---
------TTGTTCCTTGGGTTCTTAGGAGCA---GCAGGAAGCACTATG---GGCGCAGCGTCA---
ATGACGCTGACGGTACAG---GCCAGACTA---TTATTG---TCTGGTATAGTGCAACAGCAAAAC---
AACTTG---CTGAGA---GCTATTGAGGCGCAACAGCAGCTGTTGCAACTCACAGTCTGGGGCATT
>DQ869014
TTCTACTGTAATACAACACAGCTGTTTAATAGTACTTGGAATACTACTCGGGAGAATGTAAAT----------
----

AACACTGAAACTATCACACTCCCATGCAGAATAAAACAGATTGTAAACAGGTGGCAGGAAGTAGGAAAAGCAA
TGTATGCCCCTCCCATCAGTGGACAAATTAGATGTTCATCAGAA---
ATTACAGGGCTGCTATTAATAAGAGATGGTGGTAATAGTAACAATAGT-------------------------
----

AACGGGACTGAGACCTTCAGACCTGGAGGAGGAAATATGAAAGACAATTGGAGAAGTGAATTATATAAATATA
AAGTAGTAAAAATTGAGCCATTA---GGAATAGCACCCACCAAGGCAAAGAGAAGAGTGGTG-----------
----------------------------CAGAGAGAAAAAAGAGCG---GTGGGAACGCTGGGAGCT------
---GTGTTCCTTGGGTTCTTGGGAACA---GCAGGAAGCACTATG---GGCGCAGCGTCA---
ATAACGCTGACGGTACAG---GCCAGACAA---TTATTG---TCTGGTATAGTGCAGCAGCAAAAC---
AATTTG---CTGAGG---GCTATTGATGCGCAACAGCATATGTTGCAACTCACTGTTTGGGGCATC
>DQ869027
TTCTACTGTAATTCAACACAACTGTTTAAAAGTACTTGGCATGTTAATGGTACTCGGAATGGTACTGAA----
----

GGAAATGACACAATCACTCTCCCATGTAAAATAAAACAAATTATAAACATGTGGCAGAAAGTAGGAAAAGCAA
TGTATGCCCCTCCCATCAAAGGAAATATTACATGTTCATCAAAT---
ATTACAGGGCTCATACTAACAAGAGATGGTGGTAGCAATGGCGAGAACACCACT-------------------
----
-
GAGATCGAGAACACCACCGAGATCTTCAGACCTGGGGGAGGAAATATGAGGGACAATTGGAGAAATGAATTAT
ATAAATATAAAATAATAAAACTTGAACCATTA---GGAATAGCACCCACTAAGGCAAAGAGAAGAGCGGTG--
-------------------------------------CAGAGAGAAAAAAGAGCT---GTGGGA---
ATAGGAGCT---------ATGTTCCTTGGGTTCTTGGGGGCA---GCAGGAAGCACTATG---
GGCGCAGCGTCA---CTGACGCTGACGGTACAG---GCCAGGCAA---TTAATG---
TCTGGTATAGTGCAACAGCAGAAC---AATTTG---CTGAGG---
GCTATTGAGGCGCAGCAGCATATGTTGCAACTCACAGTCTGGGGCATC
>EU578061

TTCTACTGTAATACATCTGGGCTGTTTAATAGTACATGGGTGTTTAATAATAGTAATAAGGTGTATGAGAGTG
ACTGGAAT---
-------------AATACT------
GAAGAGCAAAATATCACCACACTCCAGTGCAGAATAAAACAAATTGTAAACTTGTGGCAGGAAGTAGGAAAAG
CAATGTATGCCCCTCCCATCGAGGGAAAAATTAAATGTAAATCAAAT---
ATCACAGGGCTGCTATTAACAAGAGATGGTGGTACAAATAGTACA---------------------------
----

AATAAAAGTGAGACCTTCAGACCTGGAGGAGGAGATATGAGGGACAATTGGAGAAGTGAATTATATAAATATA
AAGTAGTAAGAATTGAACCATTA---GGAATAGCACCCACCAAGGCAAGGAGAAGAGTGGTG-----------
----------------------------CAGAGAGAAAAAAGAGCA---GTGGGAGCAATAGGAGCT------
---TTGTTCCTTGGGTTCTTGGGAGCA---GCAGGAAGCACTATG---GGCGCAGCGTCG---
ATGACGCTGACGGTACAG---ACCAGACAA---TTATTG---TCTGGTATAGTGCAACAGCAGAAC---
AATCTG---CTGAAG---GCTATTGAGGCGCAACAGCATATGTTGCAGCTCACGGTCTGGGGCATC
>FJ469738
TTCTACTGTAATACAACACAACTGTTTAATAGTACTTGGTATAGGAATAGGACTGAAGGGTTAAATAAAACTA
AA---

GGAAATAACACAATCACACTCCCCTGCAGAATAAAACAAATTGTAAACATGTGGCAGGAAGTAGGAAAAGCAA
TGTATGCCCCTCCTATCAGAGGACAGATTAACTGTTCATCCAAT---
ATTACAGGGCTGCTATTAACAAGAGATGGTGGTGATACTAGCGAG---------------------------
----

AACGAGAACGAGACCTTCAGACCTGGAGGAGGAGATATGAGGGACAATTGGAGAAGTGAATTATATAAATATA
AAGTAGTAAAAATAGAACCATTA---GGAGTAGCACCCACCAAGGCAAAGAGAAGAGTGGTG-----------
----------------------------CAGAGAGAAAAAAGAGCA---GTAGGA---ATAGGAGCT------
---TTGTTCCTTGGGTTCTTGGGAGCA---GCAGGAAGCACTATG---GGCGCAGCGTCA---
GTGACGCTGACGGTACAG---GCCAGACAA---TTACTG---TCTGGTATAGTGCAACAGCAGAAC---
AATTTG---CTGAGA---GCTATTGAGGCGCAACAACATCTGTTGCAACTCACAGTCTGGGGCATC
>KT283937
TTCTACTGTAATACAACACAACTGTTTAATAGTGAATGGCCTTGGAATAGTACTAATAGTACTGGAGGGTCAA
AC---
-------------AGCACT------
GAGGGAGATGACACTATCATACTCCAATGCAGAATAAAACAAATTATAAATATGTGGCAGGAAGTAGGGAAAG
CAATGTATGCCCCTCCCATTGGAGGACAAATTAAATGTGTATCAAAT---
ATTACAGGGCTGATATTAACAAGAGATGGAGGTAATGATAATAAG---------------------------
----

ACGGACAATAACACAGAGACCTTCAGACCTGGAGGAGGAGATATGAGAGAAAATTGGAGAAGTGAATTATATA
AATATAAAGTAGTAAAAATTGAACCATTA---GGAGTAGCACCCACCAAGGCAAAGAGAAGAGTGGTG-----
------------------------------------CAGAGAGAGAAAAGAGCA---
GTGGGAACACTAGGAGCT---------ATGTTCCTTGGGTTCTTGGGAGCA---GCAGGAAGCACTATG---
GGCGCAGCGTCA---ATGACGCTGACGGTACAG---GCCAGACAA---TTATTG---
TCTGGTATAGTGCAACAGCAGAAC---AACTTG---CTGAGA---
GCTATTGAGGCGCAACAGCACCTGTTGCAACTCACGGTCTGGGGCATC
>EF593254
TTCTACTGTAATACAACAAAACTGTTTAATAGTAGTTGGAATGGGAATGATAATGATACTGAAGAGGTAAAC-
----

AGCACAATAAGCATCACACTCCCATGTAGAATAAAACAAATTATAAACATGTGGCAGGAAGTAGGAAAAGCAA
TGTATGCCCCTCCCATCAAAGGACAAATTAGATGTTCATCAAAT---
ATTACAGGGCTGCTATTAACAAGAGATGGTGGTATTGGGAACAATGCCACT---------------------
----

GAGAATACCACTGAGATCTTCAGACCGGGAGGAGGAAATATGAGGGACAATTGGAGAAGTGAATTATATAAAT
ACAAAGTAGTAAAAATTGAACCATTA---GGAGTAGCACCCACCAGGGCAAAGAGAAGAACGGTG--------
-------------------------------CAGAGAGAAAAAAGAGCA---GTAACA---CTGGGAGCT---
------TTGTTCCTTGGGTTCTTGGGATCA---GCAGGGAGCACTATG---GGCGCAGCGTCA---
ATAACGCTGACGGTACAG---GCCAGACAA---TTACTG---TCTGGTATAGTGCAGCAGCAGAGC---
AATTTG---CTGCGA---GCTATTGAGGCGCAACAACACCTGTTGCAACTCACAGTCTGGGGCATC

>JN944936
TTCTACTGTAATACATCAAAACTGTTTAATGGAACCTGGAATAGTACTAAAAGAGCAAATAAC----------
----

ACGGAAGGGATTATCATACTCCAATGCAGAATAAAACAAATTATAAATAGGTGGCAAGAAGTAGGAAAAGCAA
TGTATGCCCCTCCCATCGAAGGACAAATTAAATGCTCATCAAAT---
ATTACAGGGTTGCTATTAACAAGAGATGGTGGTAAAACCACCAAC----------------------------
----

AACACCACAGAATTCTTCAGACCTGGAGGAGGAAATATGAAAGATAATTGGAGAAGTGAATTATATAAATATA
AAGTAGTAAGAATTGAACCATTA---GGAGTAGCACCCACCAAGGCAAAGAGAAGAGTGGTG-----------
----------------------------CAGAGAGAAAAAAGAGCA---ATAGGA---ATAGGAGCT------
---GTGTTCCTTGGGTTCTTAGGAGCA---GCAGGAAGCACTATG---GGCGCAGCGTCA---
ATAACGCTGACGGTACAG---GCCAGAAAA---TTATTG---TCTGGTATAGTGCAACAGCAGAAC---
AATCTG---CTGAGG---GCTATTGAGGCGCAACAGCATCTGTTGCAACTCACAGTCTGGGGCATC
>EF593291
TTCTATTGTAATACATCAAAACTGTTTAATAGTACTTGGGGTACTTGGAATAGTACTAAAGGGTCAAATGAC-
----

ACTGGAGGAAATATCACACTCCCATGCAGAATAAAACAAATCATAAACATGTGGCAGGGAGTAGGAAGAGCAA
TGTATGCCCCTCCCATCAGAGGACAAATTAAATGTTCATCAAAT---
ATTACAGGGCTGATATTAACAAGAGATGGTGGTAATGGCACAAAC----------------------------
----

AGCACCACCGAGATCTTCAGACCTGGAGGAGGAGATATGAGGGACAATTGGAGAAGTGAATTATATAAATATA
AAGTAGTGAAAATTGAACCATTA---GGAATAGCACCCACCAAGGCAAAGAGAAGAGTGGTG-----------
----------------------------CAAAGAGAAAAAAGAGCA---GTGGGA---ATAGGAGCT------
---ATGTTCCTCGGGTTCTTGGGAGCA---GCAGGAAGCACTATG---GGCGCAGCGTCA---
GTGACGCTGACGGTACAG---GCCAGACAA---TTATTG---TCTGGTATAGTGCAACAGCAAAGC---
AATTTG---CTGAGA---GCTATTGAGGCGCAGCAGCATATGTTGCAACTCACAGTCTGGGGCATC
>EF593293
TTCTACTGTAATACAGCACAACTGTTTAATAGTACTTGGAGTGGGGGACAGTCAAATGGAACTGAAGAC----
----

AATAGCACCAATATCAGACTCCCATGCAGAATAAAGCAAATTATAAACATGTGGCAGGAAGTAGGAAAAGCAA
TGTATGCCCCTCCCATCAGAGGACAAATTAGATGTTCATCAAAT---
ATTACAGGGCTGCTATTAATAAGAGATGGTGGTAATAGTAACACCACGAAC----------------------
----

AACAGCAACACGGAGACCTTTAGACCTGGAGGAGGAAATATGAGGGATAATTGGAGAAGTGAATTATATAAAT
ATAAAGTAGTAAAAATTGAACCATTA---GGAGTAGCACCCACCAAGGCAAGGAGAAGAGTGGTG--------
-------------------------------CAGAGAGAAAAAAGAGCA---GTGGGA---ATAGGAGCT---
------GTGTTCCTTGGGTTCTTGGGAGCA---GCAGGAAGCACTATG---GGCGCAGCGTCA---
GTGGCGCTGACGGTACAG---GCCAGACGA---TTATTG---TCTGGTATAGTGCAACAGCAGAAC---
AATTTG---CTGAGG---GCTATTGAGGCGCAACAGCATCTGTTGCAACTCACAGTCTGGGGCATC
>EF593294
TTCTACTGTAATACAACAAAGCTGTTTAATAGTACTTGGCCGAGTAATGGTACTGAGCCGTTGAAT-------
----

GATACTGAAAATATCATACTCCCATGCAGAATAAAACAAATTATAAACAGATGGCAGGAAGTGGGAAAAGCAA
TGTATGCCCCTCCCATCAGCGGACTAATTAGATGCTCATCAAAT---
ATTACAGGACTGCTCTTAACAAGAGATGGTGGTAATAACAGCAGC----------------------------
----

AAGAACGAGACTTTCAGACCAGGAGGAGGAGATATGAGGGACAATTGGAGAAGTGAATTATATAAATATAAAG
TAGTAAAAATTGAGCCATTA---GGGATAGCACCCACCAAGGCAAGGAGAAGAGTGGTG--------------
-------------------------CAGAGAGAAAAAAGAGCA---GTGGGA---ATAGGAGCT---------
TTGTTCCTTGGGTTCTTGGGAGCA---GCAGGAAGCACTATG---GGCGCAGCGTCA---
GTGACGCTGACGGTACAG---GCCAGACTA---TTATTG---TCTGGTATAGTGCAACAGCAAAAC---
AATTTG---CTGAGG---GCTATTGAGGCGCAACAGCATATGTTGCAACTCACAGTCTGGGGCATT

>GU330249
TTCTACTGTAATTCAACACCACTGTTTAATAGTAGTTGGACATCTAATGGTACATCTAATGGTACTTGGAAGC
CTGATGATGCT--
----------TGGAATAAT------
AATACTAAAGAAAATATCACACTCCTATGCAGAATAAAACAAATTATAAACATGTGGCAGGAAGTAGGAAAAG
CAATGTATGCCCCTCCTATTAGAGGACAAATTAGTTGTTTATCAAAT---
ATTACAGGGCTGCTATTAACAAGAGATGGTGGGGGTAATAACGGG----------------------------
----

AGCAACAATGAAACCTTCAGGCCTGGAGGAGGAAATATGAAAGACAATTGGAGAAGTGAATTATATAAATATA
AAGTAGTAAAAATTGAACCATTA---GGAGTAGCACCCACCAAGGCAAAAAGAAGAGTGGTG-----------
----------------------------CAAAGAGAAAAAAGAGCA---GTGGGAACACTAGGAGCT------
---ATGTTTCTTGGGTTCTTGGGAGCA---GCAGGAAGCACTATG---GGCGCAGCGTCA---
ATGGCGCTGACGGTACAG---GCCAGACAA---TTATTG---TCTGGTATAGTGCAGCAGCAGAAC---
AATCTG---CTGAGA---GCTATTGAGGCGCAACAGCATATGTTGCAACTCACAGTCTGGGGCATT
>GU330333
TTCTATTGTAATACAACAAAACTGTTTAATAGTACTTGGCATACTAATAGTACTGGGAATGAGACTACGGGGT
CAAATAAC---
-------------------ACG---
GGAGAAAATGACACAATCATGCTCCCATGCAGAATAAAACAAATTATAAACAGGTGGCAGGAAGTAGGAACAG
CAATGTATGCCCCTCCCATCAGAGGACAAATTAGCTGTTCATCAAAT---
ATTACAGGGCTGCTATTGACAAGAGACGGTGGTATAAATATCACT----------------------------
----

AACGAGAATGAAACCTTCAGACCTGAAGGAGGAGATATGAGGAACAATTGGAGAAGTGAATTATATAAATATA
AAGTAGTAAAAATTGAACCATTA---GGAATAGCACCCACCAAGGCAAAGAGAAGAGTGGTG-----------
----------------------------CAGAGAGAAAAAAGAGCA---GTGGGA---ATGGGAGCT------
---TTGTTCCTTGGGTTCTTAGGAGCA---GCAGGAAGCACTATG---GGCGCAGCGTCA---
GTGACGTTGACGGTACAG---GCCAGACAA---TTATTG---TCTGGTATAGTGCAACAGCAGAAC---
AACTTG---CTGAGG---GCTATTGAGGCGCAACAACATATGTTGCAACTCACAGTCTGGGGCATC
>KT124777
TTCTACTGTAACTCATCAAAATTGTTTAATAGTACTTGGTTTAATAGGACTGGGGAGGATAATAATACTACAG
CAGAT--
-------------AGCAGT------
GCAGGAAATGGTACAATCACAATCCCATGCAGAATAAAACAAATTATAAACATGTGGCAGGAAGTAGGAAAAG
CAATGTATGCTCCTCCCATCAGAGGACAGATTAGATGTATATCAAAT---
ATTACAGGGCTGCTATTAACAAGGGATGGTGGCGAGGAAGAGAAC----------------------------
----

GAGACCGAGGTCTTTAGACCCGGGGGAGGTGATATGAGGGACAATTGGAGAAGTGAATTATATAAGTATAAAG
TAGTAAGAATTGAACCATTA---GGAATAGCACCCACCAAGGCAAAGAGAAGAGTGGTG--------------
-------------------------CAGAGAGGAAAAAGAGCA---GTGGGG---ATAGGAGCT---------
CTGTTCCTTGGGTTCTTGGGAGCA---GCAGGAAGCACTATG---GGCGCAGCGTCA---
ATGACGCTGACGGTACAG---GCCAGACTA---TTATTG---TCTGGTATAGTGCAACAGCAGAAC---
AACTTG---CTGAGG---GCTATTGAGGCGCAACAGCATCTGTTGCAACTCACAGTCTGGGGCATC
>KT124795
TTCTACTGCAATTCAACACAACTGTTTAATAGTACTTGGAATTGGAATGATACTGAAAGGATAAGTAATAAC-
----

ACTGGAAAAAAATATCACACTCCCATGCAGAATAAAACAAGTTATAAACCTGTGGCAGGAAGTAGGAAAAGCAA
TGTATGCCCCTCCCATCAGCGGAAACATTAGCTGTTCATCAAAT---
ATTACAGGGCTACTACTAACAAGAGATGGTGGTGGGGACAATAAC----------------------------
----

AGGACGAACGAGACCTTCAGACCTGGAGGAGGAAATATGAGGGACAATTGGAGAAGTGAATTATATAAATATA
AAGTAGTAAAAATTGAACCATTA---GGAGTAGCACCCACCAGGGCAAAGAGAAGAGTGGTG-----------
----------------------------CAAAGAGAAAAAAGAGCA---GTGGGAACAATAGGAGCT------
---ATGTTCCTTGGGTTCTTGGGAGCA---GCAGGAAGCACTATG---GGCGCAGCGTCA---
CTAACGCTGACGGTACAG---GCCAGACTA---TTACTG---TCTGGTATAGTGCAACAGCAGAAC---
AATTTG---CTGAGA---GCTATTGAGGCACAACAGCATCTGTTGCAACTCACAGTCTGGGGCATC

>DQ869020
TTCTACTGTAATACAACACTACTGTTTGATAGTACTTGGAATAATAATAGTACTTGGAATGCAAAT-------
----

GTGTCAGAAACTATCATACTCCCATGCAGAATAAAACAAATTATAAACATGTGGCAGGCAGTAGGAAAGGCAA
TGTATGCTCCTCCCATCAGAGGACTAATTAACTGTTCCTCAAAT---
ATCACAGGGCTGCTATTAACAAGAGATGGTGGTATTGGTAATAGTAAT-------------------------
----

AGTGAAAATGAGACCTTCAGACCTGAAGGAGGAGATATGAGGGACAATTGGAGAAGTGAATTATATAAATATA
AAGTAGTAAAAATTGAACCATTA---GGAATAGCACCCACCAAGGCAAAGAGAAGAGTGGTG-----------
----------------------------CAGAGAGAGAAAAGAGCA---GTGGGA---ATAGGAGCT------
---GTGTTTCTTGGGTTCTTGGGAGCT---GCAGGAAGCACTATG---GGCGCAGCGTCA---
ATAACGCTGACGGTACAG---GCCAGACTA---TTGTTG---TCTGGTATAGTGCAACAGCAAAAT---
AATTTG---CTGAGG---GCTATTGAGGCGCAACAGCATCTGTTGCAACTCACAGTCTGGGGCATC
>DQ869021
TTCTACTGTGATTCAACACAACTGTTTAGTAGTACTTGGAATAATGGTACTGAGAAGGCAGAAGGAAATGAC-
----

ACAAGTGACACAATTACACTCCCATGCAAAATAAAACAAATTATAAACATGTGGCAGGAAGTGGGAAAAGCAA
TGTATGCTCCTCCCATCAGTGGATATATTAACTGTCTATCAAAT---
ATTACAGGGCTGATATTAACAAGAGATAGGGATGGAGGGGTGAACGAG-------------------------
----

ACAAATATGAACGAGACCTTCAGACCTATAGGAGGAAATATGAAGGACAATTGGAGAAGTGAATTATATAAAT
ATAAAGTAGTGAAAATTGAACCATTA---GGAGTAGCACCCACTAAGGCAAAGAGAAGAGTGGTG--------
-------------------------------CAGAGAGGAAAAAGAGCA---GTGGGA---ATAGGAGCT---
------CTGTTCCTTGGGTTCTTGGGAGCA---GCAGGAAGCACTATG---GGCGCAGCGTCA---
ATGACACTGACGGTACAG---GCCAGACAA---TTATTG---TCTGGTATAGTGCAACAGCAAAAC---
AATTTG---CTGAGA---GCTATTGAGGCGCAACAACATATGTTGCAACTCACAGTCTGGGGCATC
>DQ869022
TTCTACTGTAATTCAACACAGCTGTTTAATAGTACTTGGACCAGGAATGAGACCTTGAATGGGACTGAAGGGT
CAAAT--

AGCAATGACACATTCATCACACTCCCATGCAGAATAAAACAAATTATAAACATGTGGCAACAAGTAGGAAAAG
CAATGTATGCCCCTCCCATCAGAGGAAACATTAGTTGCTCATCAAAT---
ATTACAGGGTTGCTATTAACAAGAGATGGTAGTACCGAAAATGAG----------------------------
----

AGTAACACCGAGATTTTCAGACCTGGAGGAGGAGATATGAGGGACAATTGGAGAAGTGAATTATATAAATATA
AAGTAGTAAGAATTGAACCATTA---GGAATAGCACCCACCAAGGCACAGAGAAGAACGGTG-----------
----------------------------CAGAGAGAAAAAAGAGCA---GTGGGA---CTAGGAGCT------
---TTGTTCCTTGGGTTCTTGGGAGCA---GCAGGAAGCACTATG---GGCGCAGCGTCA---
ATAACGCTGACGGTACAG---GCCAGACTA---TTATTG---TCTGGTATAGTGCAACAGCAGAAC---
AATTTG---CTGAGG---GCTATTGAGGCGCAACAGCATCTGTTGCAACTCACAGTCTGGGGCATC
>FJ469687
TTCTACTGTAGTACAACACCACTGTTTAATAGTACTTGGGAGCCTAATGATACTAGAAGGTTCAATGAC----
----

ACTGAAGAAATTATCACACTCATATGCAAAATAAAACAAGTTGTAAACATGTGGCAGAAAGTAGGAAAAGCAA
TGTATGCCCCTCCCATCAGAGGACAAATTAGATGTTCATCAAAT---
ATTACAGGGCTAATATTAACAAGAGATGGTGGTAGTGACGAGAAC----------------------------
----

ACGACCACCGAGATCTTCAGACCTGGAGGAGGAGATATGAGGGACAATTGGAGAAGTGAATTATATAAATATA
AAGTAGTAAAAATTGAACCATTA---GGAGTAGCACCCACCAGGGCAAAGAGAAGAGTGGTG-----------
----------------------------CAGAGAGAAAAAAGAGCA---GTGGGA---CTAGGAGCT------
---ATGTTCCTTGGGTTCTTGGGAGCA---GCAGGAAGCACTATG---GGCGCAGCGTCA---
ATAACGCTGACGGTACAG---GCCAGACAA---TTATTG---TCTGGTATAGTGCAACAGCAGAAC---
AACCTG---CTGAAG---GCTATTGAGGCGCAACAGCGTATGTTGCAACTCACTGTCTGGGGCATC

>FJ469688
TTCTACTGTAATACATCAAAGCTGTTTAATAGTACTTGGAATACTAGCAGTACTGGGAATGTTACTGATGAG-
----

ACAAATGACACAATCACACTCCCATGCAGAATAAAACAGATTGTAAACCTGTGGCAGGAAGTAGGAAAAGCAA
TGTATGCCCCTCCCATCAGAGGACAAATTAGATGTTCATCAAAC---
ATTACAGGGCTACTACTAACAAGAGATGGAGGTAATGGGAATAAT----------------------------
----

AGCACAATCGAGATCTTCAGACCTGGAGGAGGAGATATGAGGGACAATTGGAGAAGTGAATTATATAAATATA
AAGTAGTAAAAATTGAACCATTA---GGAGTAGCACCCACCAAGGCAAAGAGAAGAGTGGTG-----------
----------------------------CAGAGAGAAAAAAGAGCA---GCGGGA---CTAGGAGCT------
---TTGTTCCTTGGGTTCTTGGGAGCA---GCAGGAAGCACTATG---GGCGCAGCGTCA---
ATAACGCTGACGGTACAG---GCCAGACAA---TTATTG---TCTGGTATAGTGCAACAGCAGAAC---
AATTTG---CTGAGG---GCTATTGAGGCGCAACAGCATATGTTGCAACTCACAGTCTGGGGCATC
>FJ469693
TTCTACTGTAATACAACAGGACTGTTTAATAGTACTTGGAATAGTACTGGGAATATTGAAGGGTCAAATGGGA
CT---

GAAGGAAATGACACAATTACACTCCCATGCAGAATAAAACAAATTATAATCATGCGGCAGGAAGTAGAAAAAG
CAAAGTCTCCCCCTCCCATCAGAGGACAAATTAAATCTTCATCAATT---
ACTACAGGGCTATTATCAACAAGAGATGGTGGTAATAAGAATGGGACTAAT----------------------
----

GAGAGTAGCGACACTGAGATCTTCAGACCTGGAGGAGGAGATATGAGGGACAATTGGAGAAGTGAATTATATA
AATATAAAGTAGTAAAAGTTGAACCATTA---GGAGTAGCACCCACCAAGGCAAAGAGAAGAGTGGTG-----
----------------------------------CAGAGAGAAAAAAGAGCA---
GTAGTGGGATTAGGAGCC---------GTGTTCCTTGGGTTCTTGTCAGCA---GCAGGAAGCACTATG---
GGCGCAGCGTCA---TTAACGCTGACGGTACAG---GCCAGACAA---TTATTG---
TCTGGTATAGTGCAACAGCAGAAC---AATTTG---CTGAGA---
GCTATTGAGGCGCAACAGCATATGTTGCAACTCACAGTCTGGGGCATC
>HQ217201
TTCTACTGTAATACAACAAAACTGTTTAAGGGGACTTGGAATGGTACTACTTGGTGGAATGGTGCTAATGGGA
CAAATATCACA--
-------GATAACAATGAA------
GGGTCAAATGACACAATCATACTCCCATGCAGGATAAAACAAATTGTAAACTTGTGGCAGGAAGTAGGAAAAG
CAATGTATGCCCCTCCCATCCAAGGACAAATTAGATGTTCATCAAAT---
ATTACAGGGCTGATATTAACAAGAGATGGGGGTAACCAAAACAAAACATGCAAC-------------------
----
-
GAGACTAATCCATGCACTGAGACCTTCAGACCAGGAGGAGGAAATATGAAAGACAATTGGAGAAGTGAATTAT
ATAAATATAAAGTAGTAAAAATTGAACCATTA---GGAGTAGCACCCACCAAGGCAAAGAGAAGAGTGGTG--
-------------------------------------CAAAGAGAAAAAAGAGCA---GTGGGA---
ATAGGAGCT---------TTGTTCCTTGGGTTCTTGGGAGCA---GCAGGAAGCACTATG---
GGCGCAGCGTCA---TTGACGCTGACGGTACAG---GCCAGACAA---TTATTG---
TCTGGTATAGTGCAACAGCAAAAC---AATTTG---CTGAGA---
GCTATTGAGGCGCAACAGCATATGTTGCAACTCACAGTCTGGGGCATT
>FJ469730
TTCTACTGTAATACAACACAGCTGTTTAATGGTACTTGGAATAATGGTACTTGGAATAATGGTACTTGGAATA
GTACC--
-------------------GAA---
GGGTCAAATGACACTATCACACTTCCATGCAGAATAAAACAAATTATAAACATGTGGCAAAAAGTAGGAAAAG
CAATGTATGCCCCTCCCATTAGAGGACAAATTAGATGTTCATCAAAT---
ATTACAGGGCTGCTATTAACAAGCGATGGTGGTGGTAATGGGAAT----------------------------
----

AGTAGCGAGACGTTCAGACCTGGAGGAGGGAATATGAAGGACAATTGGAGGAGTGAATTATATAAATATAAAG
TAGTGAAAATTGAACCATTA---GGAGTAGCACCCACCAAGGCAAAGAGAAGAGTGGTG--------------
-------------------------CAGAGAGAAAAAAGAGCA---ATGGGA---CTAGGAGCT---------
CTGTTCCTTGGGTTCTTGGGAGCA---GCAGGAAGCACTATG---GGCGCAGCGTCA---

GTGACGCTGACGGTACAG---GCCAGACTA---TTATTG---TCTGGTATAGTGCAACAGCAGAAC---
AATTTG---CTGAGG---GCTATTGAGGCGCAACAGCATCTGTTGCAACTCACAGTCTGGGGCATC
>FJ469737
TTCTACTGTAATACAACAAGGCTGTTTAATAGTACTTGGATAGGTACTAATAGTACTTGGAAT----------
----

GATACTGATATCATCACACTCCCATGCAGAATAAAACAAATTGTAAACATGTGGCAGGAAGTAGGAAAAGCAA
TGTATGCCCCTCCCATCAGTGGACAAATTAGATGTTCATCAAAT---
ATTACAGGGCTACTATTAACAAGAGATGGGGGTAGCCAGAACCAAACG-------------------------
----

GAGAACAACACGGAGACCTTCAGACCTGGAGGAGGAGATATGAGGGACAATTGGAGAAGTGAATTATATAAAT
ACAAAGTAGTAAGAATTGAACCATTA---GGAGTAGCACCCACCACGGCAAAAAGGAGAGTGGTG--------
-------------------------------CAAAGAGAAAAAAGAGCA---GTGGGA---ATAGGAGCT---
------TTGTTCCTTGGGATCTTGGGAGCA---GCAGGAAGCACTATG---GGCGCAGCATCA---
CTGACGCTGACGGTACAG---ACCAGACTA---TTATTG---TCTGGTATAGTGCAACAGCAGAAC---
AATTTG---CTGAGG---GCTATTGAGGCGCAACAGCATATGTTGCAACTCACAGTCTGGGGCATC
>JQ403102
TTCTACTGTAATACAACACAACTATTTAATAGTAGTTGGGTTGTCAATGGGACTATAGAGTCAAATGCCACCG
AA---

GGAGATAGCAAGATCACCCTCCCATGCAGAATAAAACAAATTGTAAACATGTGGCAGGAAGTAGGAAAAGCAA
TGTATGCCCCTCCCATCAGAGGAAAAATTAGCTGTCCATCAAAT---
ATTACAGGGCTGCTATTAACAAGAGATGGTGGGCATGATGGTAATGGGAATAGC-------------------
----

AGCAACAGTGAGACATTCAGACCTGGAGGAGGAGATATGAGGGACAATTGGAGAAGTGAATTATATAAATATA
AAGTAGTAGAAATTAAACCATTA---GGACTAGCACCCAACAAGGCAAAGAGAAGAGTGGTG-----------
----------------------------CAAAGAGAAAAAAGAGCA---GTGGGA---ATAGGAGCT------
---GTGTTCCTTGGGTTCTTGGGAGCA---GCAGGAAGCACTATG---GGCGCAGCGGCA---
GTGACGCTGACGGTACAG---GCCAGACAA---TTATTG---TCTGGTATAGTGCAACAGCAGAAC---
AATTTG---CTGAGA---GCTATTGAGGCGCAACAGCATCTGTTGCAACTCACAGTCTGGGGCATC
>FJ469741
TTCTACTGTAATACAACACGACTGTTTAACAGTACTTGGACTTGGGATAATGATACTGAAGAA----------
----

TCAAATGACACTATCACACTCCCATGCAGAATAAAACAAATTATAAACATGTGGCAGGAAGTAGGAAAAGCAA
TGTATGCCCCTCCCATCAGAGGACAAATTAAATGTTCATCAAAT---
ATTACAGGGCTGCTATTAATAAGAGATGGTGGTGAGGACGGGAAC----------------------------
----

AGTACCACCGAGATCTTCAGACCTGGAGGAGGAAATATGAGGGACAATTGGAGAAGTGAATTGTATAAATATA
AAGTAGTAAAAATTGAACCATTA---GGATTAGCACCCACCAAGGCAAAGAGAAGAGTGGTG-----------
----------------------------CAGAGAGAAAAAAGAGCA---GCA------ATAGGAGCT------
---ATGATCCTTGGGTTCTTGGGAGCA---GCAGGAAGCACTATG---GGCGCAGCGTCA---
CTGACGCTGACGGTACAG---GCCAGACTA---TTATTG---TCTGGTATAGTGCAACAGCAGAAC---
AATTTG---CTGAGA---GCTATTGAGGCGCAACAGCATCTGTTGCAACTCACAGTCTGGGGCATC
>FJ469748
TTCTATTGTAATACTACACAACTGTTTAATAGTACTTGGTATGGAAATGGTACTGTACAGTCAATATCTGAA-
----

GGAAATGAAACAATCACACTCCCATGCAGAATAAAACAGATTGTAAACATGTGGCAACAAGTAGGAAAAGCAA
CGTATGCCCCTCCCATCAGTGGACAAATTAACTGTACATCAAAT---
ATTACAGGGCTGCTAATAACAAGAGATGGTGGGGGTAACGAGTCC----------------------------
----

AGGAATAATGAGACCTTTAGACCTGCAGGAGGAGATATGAGGGACAATTGGAGAAGTGAATTATATAAATATA
AAGTAGTAAAAATTGAACCATTA---GGAGTAGCACCCACCAAGGCAAAAAGAAGAGTGGTG-----------
----------------------------CAGAGAGAAAAAAGAGCA---GTGACA---CTAGGAGCT------
---GTGTTCCTTGGGTTCTTGGGAGCA---GCAGGAAGCACTATG---GGCGCAGCGTCA---

ATGACGCTGACGGTACAG---GCCAGACTA---TTGATG---TCTGGTATAGTGCAACAGCAGAAC---
AACTTG---CTGAAA---GCTATTGAGGCGCAACAGCATCTGTTGCAACTCACTGTCTGGGGCATC
>FJ469760
TTCTACTGTAATTCAACAAAACTGTTTAATAGTACTTGGCCACTTAATAGTACTGTGAATGATACTACCGGGT
CA---

AATAACAATGATACCATAGTTCTCCCATGCAGAATAAAACAAATTATAAACATGTGGCAACAAGTAGGAAGAG
CAATGTATGCCCCTCCTATCAGAGGAAAAATTAGATGTTCATCAAAT---
ATTACAGGGCTGATATTAACAAGGATGGTGGTGGTGGTGCAAACGAG--------------------------
----

ACCACCAGGAACGAGACCTTCAGACCTGCAGGAGGAGATATGAGGGACAATTGGAGAAGTGAATTATATAAAT
ATAAAGTAGTAAGAATTGAACCATTA---GGAGTAGCACCCACTAAGGCAAAGAGAAGAGTGGTG--------
-------------------------------CAGAGAGAAAAAAGAGCC---GTGGGA---CTAGGAGCC---
------ATGTTCCTTGGGTTCTTGGGAGCA---GCAGGAAGCACTATG---GGCACAGCGTCA---
CTGACGCTGACGGTACAG---GCCAGACAA---TTATTG---TCTGGTATAGTGCAACAGCAGAAC---
AATCTG---CTGAGG---GCTATTGAGGCGCAACAGCATCTGTTGCAACTCACAGTCTGGGGCATC
>FJ469762
TTCTACTGTAATACAACACAACTGTTTAATAGTACTTGGACTGATAATAGTACTACCGGGTCAAATAAC----
----

ACAAAAGGAATTATCACACTCCCATGCAGAATAAAACAAATTATAAATATGTGGCAAGAAGTAGGAAAAGCAA
TGTATGCCCCTCCCATCAGTGGATTAATTAGATGTAAATCAAAT---
ATTACAGGGCTGCTATTAACAAGAGATGGTGGTAATAAGAATAACAAC-------------------------
----

GAGACTGAGACCGAAATCTTTAGGCCTGGAGGAGGAGATATGAGGGACAATTGGAGAAGTGAATTATATAAAT
ATAAAGTAGTAAAAATTGAACCATTA---GGAATAGCACCCACCAGGGCAAAGAGAAGAGTGGTG--------
-------------------------------CAGAGAGAAAAAAGAGCA---GTGGGA---TTAGGAGCT---
------TTGTTCCTTGGGTTCTTGGGTACA---GCAGGAAGCACTATG---GGCGCAGCGTCA---
ATAACGCTGACGGTACAG---GCCAGACAA---TTATTG---TCTGGTATAGTGCAACAGCAGAAC---
AACTTG---CTGAGA---GCTATTGAGGCGCAACAACATCTGTTGCAACTCACAGTCTGGGGCATC
>EU578460
TTCTACTGTAATACAACACAGCTGTTTAATAGTACTTGGCCTAATGATACTAGTACTTGGTATGGGAATGAT-
----

ACTGCAGGAGATATCACACTCCCATGCAGAATAAAACAAATTATAAACAGGTGGCAGGAAGTAGGAAAAGCAA
TGTATGCCCCTCCCATCAGCGGATAATTAGATGTTCATCAAAT---
ATTACAGGGATAATTTTAACAAGAGATGGTGGTAATAACATCACGAAC-------------------------
----

AACACCACCGAGACCTTCAGACCTGAAGGAGGAAATATGAAGGATAATTGGAGAAGTGAATTATATAAATACA
AAGTAGTAAAAATTGAACCATTA---GGAATAGCACCCACCAAGGCAAGGAGAAGAGTGGTG-----------
----------------------------CAGAGAGAAAAAAGAGCA---GTGGGAACAATAGGAGCT------
---ATGTTCCTTGGGTTCTTGGGAGCA---GCAGGAAGCACTATG---GGCGCAGCGTCG---
CTGACGCTGACGGTACAA---GCCAGACTA---TTATTG---TCTGGTATAGTGCAACAGCAGAAC---
AATCTG---CTGAGG---GCTATTGAGGCGCAACAGCATATGTTGCAACTCACAGTCTGGGGCATC
>EF593295
TTCTACTGTGATTCAGCACCACTGTTTAATAGTAGTTGGATACGGAATACAAGTACTTGGACACGGAATGATA
GCGCTTGGAAT--
-AAAACTGAAGGACA---------
AATAGCACTGATCCAATCACACTCACATGTAGAATAAAACAATTTATAAACATGTGGCAGAAAGTAGGAAAAG
CAATGTATGCTCCTCCCATCAGTGGACAAATTAAATGTTTATCAAAT---
ATTACAGGGCTGATACTAACAAGAGATGGTGGTAATAACAAAAATACAAGT----------------------
----

GACAACGAGACTAGCGAGATCTTCAGACCTGGAGGAGGAGATATGAGGGACAATTGGAGAAGTGAATTATATA
AATATAAAGTGGTGAAAATTGAACCATTA---GGAGTAGCACCCACCAAGGCAAAGAGAAGAGTGGTG-----
----------------------------------CAGAGAGAAAAAAGAGCA---GTGACG---
CTAGGAGCT---------ATGTTCCTTGGGTTCTTGGGAGCA---GCAGGAAGCACTATG---

GGCGCAGCGTCA---ATGACGCTGACGGTACAG---GCCAGACTA---TTATTG---
TCTGGTATAGTGCAACAGCAGAAC---AATCTG---CTGAGG---
GCTATTGAGGCGCAGCAGCATCTGTTGCAACTCACAGTCTGGGGCATT
>EF593296
TTCTACTGTAATACAACAAAACTGTTTAATAGTACAGGGGGGAATAATAACACTTGGACTGAT----------
----

ACTACAGGAAATATCACACTCCAATGCAGAATAAAACAAATTATAAACATGTGGCAGGAAGTAGGAAAAGCAA
TGTATGCCCCTCCCATCAGAGGACACATTAAATGTTCATCAAAT---
ATTACAGGACTGATATTAACAAGAGATGGTGGTGGTAGTAATAAC----------------------------
----

AATACTACTGAGACCTTTAGGCCGGGAGGAGGAAATATGAAGGACAATTGGAGAAGTGAATTATATAAATATA
AAGTAGTACAAATTGAACCATTA---GGAGTAGCACCCACCAGGGCAAGGAGAAGAGTGGTG-----------
----------------------------CAGAGAGAAAAAAGAGCA---ATAGGA---CTGGGAGCT------
---CTCCTCCTTGGGTTCTTGGGAACA---GCAGGAAGCACTATG---GGCGCAGCGTCA---
CTGACGTTGACGGTACAA---GCCAGACAA---TTATTG---TCTGGTATAGTGCAACAGCAGAAC---
AATCTG---CTGAGG---GCTATTGAGGCGCAGCAGCATATGTTGCAACTCACAGTCTGGGGCATC
>EF593297
TTCTACTGTAATACAGCAGAACTGTTTAATAGTACTTGGGATACTACTACAGGGTCAAATAACACAAAT----
----

GACACTGAAATTATCACACTTCCATGTAGAATAAAACAAATTATAAACATGTGGCAGCAAGTAGGAAAAGCAA
TGTATGCCCCTCCCATCAAAGGACAAATTAGATGTTCATCAAAT---
ATTACAGGACTACTATTAACAAGGGATGGAGGTAATAACACTGAC----------------------------
----

AACGAGACCTTCAGACCTGGAGGAGGAGATATGAGGGACAATTGGAGAAGTGAATTATATAAATATAAAGTAG
TAAAAATTGAACCATTA---GGACTAGCACCCACCAAGGCAAGGAGAAGAGTGGTG-----------------
----------------------CAGAGAGAAAAAAGAGCA---GTGGGA---ATAGGAGCT---------
CTGTTCCTTGGGTTCTTGGGAGCA---GCAGGAAGCACTATG---GGCGCAGCGTCG---
ATGACGCTGACGGTACAA---GCCAGGCAA---TTATTG---TCTGGTATAGTGCAACAGCAGAAC---
AACTTG---CTGAGA---GCTATTGAGGCGCAACAGCATATGTTGCAACTCACAGTCTGGGGCATC
>GU330460
TTCTACTGTAATACAACAGGACTGTTTAATAATACTTGGGGGTTTAATAGTACTTGGGATGCTATTAATGTGC
CA---

GAAAATGACACAATCACACTCCCATGCAGAATAAAACAAATTGTAAACATGTGGCAGGAAGTAGGAAAAGCAA
TGTATGCCCCTCCCATCAGAGGACGACTTAATTGTTCATCAAAT---
ATTACAGGGCTGCTATTAACAAGAGATGGTGGTAATACCACAAAT----------------------------
----

AACACTGAGGTCTTCAGACCTGGAGGAGGAGATATGAGAGACAATTGGAGAAGTGAATTATATAAATATAAAG
TAGTAAAAATTGAACCATTA---GGAATAGCACCCACCAAGGCAAAGAGAAGAGTGGTG--------------
-------------------------CAGAGAGAAAAAAGAGCA---GTGGGA---ATAGGAGCT---------
TTGTTCCTTGGGTTCTTGGGAGCA---GCAGGAAGCACTATG---GGCGCAGCGTCG---
ATGACGCTGACGGTACAG---GCCAGACTA---TTATTG---TCTGGTATAGTGCAACAGCAGAAC---
AATTTG---CTGAGA---GCTATTGAGGCGCAACAGCATCTGTTGCAACTCACAGTCTGGGGCATC
>HM234503
TTCTATTGTAATACAACGCAACTGTTTAATAGTACTTGGAAAATTAATAGTACTTGGAATGATACTGAAGGG-
----

TCAAATGACACTATCGCACTCCCATGTAGAATAAAACAAATCATAAACATGTGGCAGAAAGTAGGAAAAGCAA
TGTATGCCCCTCCCATCCAAGGACAAATTAACTGTTCATCAAAA---
ATCACAGGGCTGCTATTAACAAGAGATGGTGGCAGCACCGATAAC----------------------------
----

AACAACACCGAGACCTTCAGACCTGGAGGAGGAGATATGAGGGACAATTGGAGAAGTGAGTTATATAAATATA
AAGTAGTAAAAGTTGAACCATTA---GGAATAGCACCCGCCAAGGCAAAGAGAAGAGTGGTG-----------
----------------------------CAGAGAGAAAAAAGAGCA---GTGGGA---ATGGGAGCT------

---CTGTTCCTTGGGTTCTTGGGAGCA---GCAGGAAGCACTATG---GGCGCAGCGTCA---
CTAACGCTGACGGTACAG---GCCAGACAA---TTATTG---TCTGGTATAGTGCAACAGCAGAAC---
AATTTG---CTGAGG---GCTATTGAGGCGCAACAGCATATGTTGCAACTCACAGTCTGGGGCATT
>KC312470
TTCTACTGTAATACAACACAACTGTTTAATAGTACTATCAATGGTACTGAAATGTAC----------------
----

AGTGCAGGAAATATCACACTCCCATGCAAAATAAAGCAAATTATAAACATGTGGCAGGAAGTAGGAAAAGCAA
TGTATGCCCCTCCCATAAGAGGACAAATTAGATGTTCATCAAAT---
ATTACAGGGCTGCTATTAGTGAGAGATGGTGGTAATGCTGAGAATGATACCAAC------------------
----

ACTACCAACATCGAGACCTTCAGACCTGGAGGAGGAGATATGAGGGACAATTGGAGAAGTGAATTATATAAAT
ATAAAGTAGTAAAAATTGAACCATTA---GGAATAGCACCCACCAAGGCAAAAAGAAGGGTGGTG--------
-------------------------------CAGAGAGAGAAAAGAGCG---GTGGGA---CTAGGAGCT---
------TTGTTCATTGGGTTCTTAGGAGCA---GCAGGAAGCACTATG---GGCGCAGCGTCA---
ATAACGCTGACGGTACAG---GCCAGGCTA---TTATTG---TCTGGTATAGTGCAACAGCAAAAC---
AATTTG---CTGAGA---GCTATTGAGGCGCAACAGCATCTGTTGCAACTCACAGTCTGGGGCATC
>KT124807
TTTTACTGTAATACAACACAGCTGTTTAGTACTTGGAATAGTACTGGGCAGTCTAATAGTACT----------
----

GGGAATGATACCATCACACTCCCATGCAGAATAAAACAAATTATAAACAAGTGGCAGGGAGTAGGAAAAGCAA
TGTATGCCCCTCCCATCAGCGGAGTAATTAATTGTACATCAAAT---
ATTACAGGGCTGCTATTGACAAGAGATGGTGGTAAGACGGGCGAG---------------------------
----

AATGAGAGTGAGACCTTCAGACCTGGAGGGGGAGATATGAGGGACAATTGGAGAAGTGAATTATATAAATATA
AAGTAGTAAAAATTGAACCAATA---GGAGTAGCACCCACCAAAGCCAAGAGAAGAGTGGTG-----------
----------------------------CAGAGAGAAAAAAGAGCA---GTAACA---CTGGGAGCT------
---ATGTTCCTTGGGTTCTTGGGAGCA---GCAGGAAGCACTATG---GGCGCAGCGTCA---
ATGACGCTGACGGTACAG---GCCAGACAA---TTATTG---TCTGGTATAGTGCAACAGCAGAAC---
AATCTG---CTGAAG---GCTATTGAGGCGCAACAGCATCTGTTGCAACTTACAGTCTGGGGCATC
>KT124813
TTCTACTGTAATACGTCACAACTATTTAATAGTACTTGGAGTAATGGGACTAATGGTAATTGGACTATAAATA
ACACC---
-ACAGGGGGAAATAAC---------
ATTGAGAATGACACTATCACACTCCCATGCAGGATAAAACAAATTATAAACTTGTGGCAGAAAGTAGGAAGGG
CAATGTATGCCCCTCCCATTAGTGGACAGATTAGCTGTTCATCAAAT---
ATTACAGGGCTAATATTAACAAGAGATGGTGGTGCTACTGATAAT---------------------------
----

AGCATGAACGAGACCTTCAGACCTGCAGGAGGAGATATGAAGGACAATTGGAGAAGTGAATTATATAAATATA
AAGTAGTAAGAATTGAACCATTA---GGAGTAGCACCCACCCCGGCAAGGAGAAGAGTGGTG-----------
----------------------------CAGAGAGAAAAAAGAGAA---CTAACA---ATGGGAGCT------
---CTGTTCCTTGGGTTCTTGGGAGCA---GCAGGAAGCACTATG---GGCGCAGCGTCG---
CTGACGCTGACGGTACAA---GCCAGACTA---TTATTG---TCTGGTATAGTGCAACAGCAGAAC---
AATATG---CTGAGG---GCTATTGAGGCGCAACAACATCTGTTGCAACTCACAGTCTGGGGCATC
>DQ869016
TTCTACTGTAATACAACAAAATTGTTCAATAGTACTTGGAACAATACTGATTCAAATACCACTGAG-------
----

AACAATGACACAATCACACTCCCATGCAGGATAAAACAAATCGTAAACTTGTGGCAGCAAGTAGGAAAAGCAA
TGTATGCCCCTCCCATCAGCGGACAGATTAGATGTTCATCAAAT---
ATCACAGGGCTTTTATTAACAAGAGATGGTGGTGATATTAATAGA---------------------------
----

ACTGAGGAGATATTCAGACCTGGAGGAGGAAATATGAAGGACAATTGGAGAAGTGAATTATATAAATATAAGG
TAGTACAAATTGAACCATTA---GGAGTAGCACCCACCAAGGCAAAGAGAAGAGTGGTG--------------
-------------------------CAGAGAGAAAAAAGAGCT---TTGGGA---ATGGGAGCT---------

TTGTTCCTTGGGTTCTTGGGAGCA---GCAGGAAGCACTATG---GGCGCAGCGTCA---
TTGACGCTGACGGTACAG---GCCAGACAA---TTATTG---TCTGGTATAGTGCAACAGCAGAAC---
AATCTG---CTGAGA---GCTATTGAGGCGCAACAGCATCTGTTGCAACTCACAGTCTGGGGCATT
>DQ869024
TTCTACTGTAATACAACACAACTGTTTAATAGTACTTGGAGGAGTAATGATACAACACAACTGTCTAATAGTA
CTTGGAGGAGT--
AATGATACTGCTTCTGAA---------
GGGTCAAATGACACTATCATCCTCCCATGTAGAATAAAACAAATTATAAACTTGTGGCAGGAAGTAGGAAAAG
CAATGTATGCCCCTCCCATTAGTGGACCAATTAGCTGTTCATCAAAT---
ATTACAGGGCTGCTATTAACAAGAGATGGTGGTAACAACAGCACC----------------------------
----

AATAACACTGAGGTCTTCAGACCTGGAGGAGGAAATATGAGGGACAATTGGAGAAGTGAACTATATAAATATA
AAGTAGTAAAAATTGAACCATTA---GGAATAGCACCCACTAGGGCAAAGAGAAGAGTGGTG-----------
----------------------------CAAAGAGAAAAAAGAGCA---GTGGGA---ATCGGAGCT------
---GTGTTCCTTGGGTTCTTGGGAGCA---GCAGGAAGCACTATG---GGCGCAGCGTCA---
ATGGCGCTGACGGTACAG---GCCAGACAA---TTATTG---TCTGGTATAGTGCAACAGCAGAAT---
GATCTG---CTGAGG---GCTATTGAGGCGCAACAACATCTGTTGCAACTCACAGTCTGGGGCATC
>DQ869025
TTCTATTGTAATACAACACAACTGTTTAATAGTATTTGGAACAGTACTTGGAGTGCTAATAGTAGTGGTAATG
TA---

GAAAATAAAACGATCACACTCCCATGCAGAATAAAACAAATTATAAACATGTGGCAGGAAGTAGGAAAAGCAA
TGTATGCCCCTCCCATCGCAGGAATAATTAGCTGTTCATCAAAT---
ATTACAGGGCTGATATTAACAAGAGATGGTGGTAATGATAATGATACG-------------------------
----

AACAAGGAGGAGGAGGTCTTCAGACCTGGAGGAGGAAATATGAAGGACAATTGGAGAAGTGAATTATATAAAT
ATAAAGTAGTAAGAATTGAACCCTTA---GGAGTAGCACCTACTAAGGCAAAGAGAAGAGTGGTG--------
-------------------------------CAGAGAGAAAAAAGAGCA---GTGGGA---ATAGGAGCT---
------GTGTTCCTTGGGTTCTTGGGAGCA---GCAGGAAGCACTATG---GGCGCAGCGTCA---
GTGGCGCTGACGGTACAG---GCCAGACAA---TTATTG---TCTGGTATAGTGCAACAGCAAAAC---
AATTTG---CTGAGG---GCTATTGAGGCGCAACAGCATATCTTGCAACTCACAGTCTGGGGCATC
>DQ869029
TTCTACTGTAATACAACACAACTGTTTACTTGGAATGATACTAGTATTGAAAATTCAAATGACACTGAGGGA-
----

TCAAATAAAATTATCATTCTTCCATGCAAAATAAAACAAATTATAAAAATGTGGCAGAGAGTAGGAAAAGCAA
CGTATGCCCCTCCCATCAGAGGACAAATTAGATGTTCATCGAAT---
ATTACAGGGCTGCTATTAACAAGAGATGGTGGTAGTAACCAGAGT----------------------------
----

AATAGTGAGACCTTCAGACCTGGAGGAGGAGATATGAGGGACAATTGGAGGAGTGAATTATATAAATATAAAG
TAGTAAAAATTGAACCATTA---GGAGTAGCACCCACCAAGGCAAAGAGAAGAGTGGTG--------------
-------------------------CAAAGGGAAAGAAGAGCA---GTGGGA---ATAGGAGCT---------
ATGCTCCTTGGGTTCTTAGGAACA---GCCGGAAGCACTATG---GGCGCAGCGTCA---
GTGGCGCTGACGGTACAG---GCCAGACTA---TTATTG---TCTGGTATAGTGCAGCAGCAGAAC---
AATTTG---CTGAGA---GCTATTGAGGCGCAACAGCACCTGTTGCAACTCACAGTCTGGGGCATC
>DQ869030
TTCTATTGTAATACAACAGAGCTGTTTAATAGTACTTGGCTGGATAATAATACTAGGAATGATACTGAAGGGT
CA---

AATAGCACTGATATCACACTCCAATGCAGAATAAAACAAATTATAAACAGGTGGCAGGAAGTAGGAAAAGCAA
TGTATGCCCCTCCCATCAGAGGACCAATTAATTGTACATCAAAT---
ATTACAGGGCTACTATTAATAAGAGATGGTGGTAATGACAATAGTACT-------------------------
----

GACAATAACACTGAGATCTTCAGACCTACAGGAGGAAACATGAGGGGCAATTGGAGAAGTGAATTATATAAAT
ATAAAGTAGTAAAAATTGAGCCATTA---GGAATAGCACCCACCGAGGCAAAGAGAAGAGTGGTG--------
-------------------------------CAGAGAGAAAAAAGAGCA---GTAGGC---CTGGGAGCT---

------ATGTTCCTTGGGTTCTTGGGAGCA---GCAGGCAGCACTATG---GGCGCAGCGTCA---
CTGACGCTGACGGTACAG---GCCAGACAA---TTACTG---TCTGGGATAGTGCAACAGCAAAAC---
AATTTG---CTGAGA---GCTATTGAGGCGCAACAGCATCTGTTGCAACTCACAGTCTGGGGCATC
>DQ869031
TTCTACTGTGATTCAACACAACTGTTTAATAGTACTTGGAATACTTGGAATGCTACAGAGTCGAATGGCACTA
GA---

GAAAATGACACAATCACACTCCCATGCAGAATAAGACAAATTATAAACATGTGGCAGAAAGTAGGAAAAGCAA
TGTATGCCCCTCCCATCAGAGGACGAATTAACTGTTCATCAATA---
ATTACAGGGCTGCTATTAACAAGAGATGGT---AGTAACAGTAGC----------------------------
----

AATGAGACCTTCAGACCTGGAGGAGGAGATATGAGGGATAATTGGAGAAGTGAATTATATAAATATAAAGTAG
TAAAAGTTGAACCGTTA---GGAATAGCACCCACCACGGCAAGGAGAAGAGTGGTG-----------------
----------------------CAGAGAGAAAAAAGAGCA---CTAGGGGGACTAGGAGCT---------
ATGTTCCTTGGGTTCTTGAGTGCA---GCAGGAAGCACTATG---GGCGCAGCGTCA---
CTGACGCTGACGGTACAG---GCCAGACAA---TTATTG---TCTGGTATAGTGCAACAGCAGAGC---
AATCTG---CTGATG---GCTATTGAGGCGCAACAACATATGTTGAAACTCACAGTCTGGGGCATT
>FJ469701
TTCTACTGTAATACATCAAAACTGTTTAATAGTACTTGGAGTACTAATAGTACTTGGAATGATACTGAAAGG-
----

TTAAATGAGACTATCATACTCCCATGCAGAATAAAACAAGTTATAAACTTGTGGCAGGAAGTAGGAAAAGCAA
TGTATGCCCCTCCCATCAGAGGAGAAATTAACTGTGCATCAAAT---
ATTACAGGACTGCTATTAATAAGAGATGGTGGAAATGAGAAAAACAAG-------------------------
----

ACCGAGGAGATCGAGATCTTTAGACCTGGAGGAGGAGATATGAGGGACAATTGGAGAAGTGAATTATATAAAT
ATAAGGTAGTAAAAATTGAACCATTA---GGAATAGCACCCACCAAGGCAAAGAGAAGAGTGGTG--------
-------------------------------CAGAGAGAAAAAAGAGCA---GCAGCG---CTAGGAACT---
------ATGATCCTTGGGTTCTTGGGAGCA---GCAGGAAGCACTATG---GGCGCAGCGTCA---
CTGACGCTGACGGTACAG---GCCAGACAA---TTATTG---TCTGGTATAGTGCAACAGCAGAGC---
AATTTG---CTGAAG---GCTATTGAGGCGCAACAGCATCTGTTGCAACTCACAGTCTGGGGCATC
>KC473846
TTCTACTGCAATACAACACCACTGTTTAATAGTACTTGGGATAGTAGTACTTGGAATAGTACTAAAAATAATA
CGGGG--

TCAAATGATACTATCACAATCCAATGTAGGATAAAACAAATTATAAACATGTGGCAGACAGTGGGAAAAGCGA
TGTATGCCCCTCCCATCGCAGGACAAATTAGATGTTTATCAAAT---
ATTACAGGGCTATTATTAGAGAGAGATGGTGGTAATAATAAGACA----------------------------
----

GACCACAACGAAACCTTCAGACCTAGCGGAGGAAATATGAAGGACAATTGGAGAAGTGAATTATATAAATATA
AAGTAGTAAAAGTTGAACCAATA---GGAATAGCACCCACCAGGGCAAAGAGAAGAGTGGTG-----------
----------------------------CAGAGAGAAAAAAGAGCG------GGA---ATAGGAGCT------
---GTGTTCCTTGGGTTCTTGGGAGCA---GCAGGAAGCACTATG---GGCGCAGCGTCA---
GTAACGCTGACGGTACAG---GCCAGACAA---TTATTG---TCTGGTATAGTGCAACAGCAAAAT---
AATTTG---CTGAGG---GCCATTGAGGCGCAACAGCATATGTTGCAACTCACTGTCTGGGGCATC
>EF593222
TTCTACTGCAATACAACAAAACTGTTTAATAGTACTTGGAATAGTGGGAATAATACTAATAATACTGAAGGAT
CA---

AATGACACTATTATCACACTCCCATGCAGAATAAAACAAATTATAAACTTATGGCAGGAAGTAGGAAAAGCAA
TGTATGCCCCTCCCATCAGAGGACAAATTAACTGTTCATCAAAT---
ATTACAGGACTGTTATTAATAAGAGATGGGGGTAATACCACAAAC----------------------------
----

AACACAGAGACCTTCAGACCTTTAGGAGGAAATATGAAGGACAATTGGAGAAGTGAATTATATAAATATAAAG
TAGTACAAATTGAACCATTA---GGAGTAGCACCCACTAAGGCAAAGAGAAGAGTGGTG--------------
-------------------------CAGAGAGAAAAAAGAGCA---GTGGGA---ATAGGAGCT---------

TTGTTCCTTGGGTTCTTGGGAGCA---GCAGGAAGCACTATG---GGCGCAGCGTCA---
GTGACGCTGACGGTACAG---GCCAGACAA---TTATTG---TCTGGTATAGTGCAACAGCAGAAC---
AATTTG---CTGAGA---GCTATTGAGGCGCAACAGCATATGTTGCAACTCACAGTCTGGGGCATC
>DQ410465
TTCTACTGTAATACAACACAACTGTTTAATAGTACTTGGAATACAACACAACTGTTTAATAGTACTTGGAATG
ATACTGGG---
-------------CCAAAT------
AACGAAAATGACACACTCACACTCCCATGCAGAATAAAACAAATTATAAACAGGTGGCAGGAAGTAGGAAAAG
CAATGTATGCCCCTCCCATCAGAGGACAAATTAGATGTTCATCAAAT---
ATTACGGGGCTGCTATTAACAAGAGATGGTGGTAACGACAATAACAAGACC----------------------
----

AATAACCAGACCGAGATCTTCAGACCTGGAGGAGGAAATATGAGGGACAATTGGAGAAGTGAATTATATAAAT
ATAAAGTAGTAAGAATTGAACCATTA---GGAGTAGCACCCACCCCGGCAAAGAGAAGAGTGGTG--------
-------------------------------CAAAGAGAAAAAAGAGCA---GTGGGA---ATAGGAGCT---
------ATGTTCCTTGGGTTCTTGGGAGCA---GCAGGAAGCACTATG---GGCGCAGCATCA---
ATGACGCTGACGGTACAG---GCCAGACTA---TTATTG---TCTGGTATAGTGCAACAGCAGAAC---
AATTTG---CTGAGG---GCTATTGAGGCGCAACAGCACCTGTTGCAACTCACAGTCTGGGGCATC
>DQ410422
TTTTACTGTAATACAACACAATTATTTAATAGTACTTGGAATGATACTGATAGTGGAGGGTCAAATAACACAA
AT---

AACAATGATACTATCACACTCCCATGCAGAATAAAACAGATTATAAACATGTGGCAGGAAGTAGGAAAAGCAA
TGTATGCCCCTCCCATCATGGGACAAATTAGCTGTTCATCAAAT---
ATTACAGGGATGATATTAACAAGAGATGGTGGTACTAATAGGAGCAAGACC----------------------
----

GAGACCGAGACCGAGACCTTCAGACCTGGAGGAGGAGATATGAGGGACAATTGGAGAAGTGAATTATATAAAT
ATAAAGTAGTAAAACTTGAACCATTA---GGAGTAGCACCCACCAAGGCAAAGAGAAGAGTGGTG--------
-------------------------------CAGAGAGAAAAAAGAGCG---ATAACA---GTAGGAGCT---
------ATGCTCCTTGGGTTCTTGGGAACA---GCAGGAAGCACTATG---GGCGCAGCGGCG---
ATAACGCTGACGGTACAG---GCCAGACAA---TTATTG---TCTGGTATAGTGCAACAGCAGAAC---
AATCTG---CTGAGG---GCTATTGAGGCGCAACAGCATCTGTTGCAACTCACAGTCTGGGGCATC
>FJ469739
TTTTACTGTAATACAACAAAACTGTTTAATAGTACTTGGACTTGGAATAATACTGCAGAGTTAAATAACACTG
AA---

CAGGATGTCATAATCCCACTCCCCTGTAGAATAAAACAAATTGTAAACCTGTGGCAGGAAGTAGGAAAAGCAA
TGTATGCCCCTCCCATCCAAGGACAAATTAGATGCTCATCAAAT---
ATCACAGGGGTGCTATTAACAAGAGATGGTGGTATAAAAGGGAGCAACGAGAGC-------------------
----
-
TGCAACGAGAGCCAACCCGAGACCTTCAGACCTGGAGGAGGAGATATGAGGGACAATTGGAGAAGTGAATTAT
ATAAATATAAAGTAGTAAAAATTGAACCATTA---GGACTAGCACCCACCAGGGCAAAGAGAAGAGTGGTG--
-------------------------------------CAAAGAGAAAAAAGAGCA---GTGGGA---
CTAGGAGCT---------TTGTTCCTTGGGTTCTTGGGAGCA---GCAGGAAGCACTATG---
GGCGCAGCGTCA---CTGACGCTGACGGTACAG---GCCAGACTA---TTATTG---
TCTGGTATAGTGCAGCAGCAGAAT---AACTTG---CTGATG---
GCTATTGAGGCGCAACAGCGTATGTTGCAACTCACAGTCTGGGGCATC
>FJ469743
TTCTACTGTAATACAACACCACTGTTCAATAGTACTTGGAGGACAAATGGAACTTGGATAAATGGAACAGCAA
ATAAC--

ACTGGAGAAAACACTATCACACTCCAGTGCAAAATAAAGCAAATTGTAAACATGTGGCAGGAAGTAGGAAAAG
CAATGTATGCCCCTCCCATCCGAGGACTAATTAGATGTTTATCAAAT---
ATTACAGGGCTGATATTAACAAGAGATGGTGGTAATCCAAATAAGACTGATAAG-------------------
----

ACTGATACTACTAGTGAGATCTTCAGACCGGGAGGAGGAAATATGAAGGACAATTGGAGAAGTGAATTATATA
AATATAAAGTAGTAAAAATTGAACCATTA---GGAGTAGCACCCACCAAGGCAAAGAGAAGAGTGGTG-----

----------------------------------CAGAGAGAAAAAAGAGCA---
GTAGGAGCGATAGGAGCT---------ATGTTCCTTGGGTTCTTGGGAGCA---GCAGGAAGCACTATG---
GGCGCAGCGTCA---ATGGCGCTGACGGTACAG---GCCAGACAA---TTATTG---
TCTGGTATAGTGCAACAGCAGAAT---AATTTG---CTGAGG---
GCTATTGAGGCGCAACAGCACCTGTTGCAACTCACGGTCTGGGGCATC
>EF593228
TTCTATTGTAATACAACACAACTGTTTAATAGTACTTGGGGGCCTAATAGAACTTTGAATGAGACTGGTGAG-
----

TCAAATGAAACTATCACCCTCCCATGCAGAATAAAACAAATTATAAACTTGTGGCAGGAAGTAGGAAAAGCAA
TGTATGCCCCTCCTATCAGTGGACAAATTAGTTGTTCATCAAAT---
ATTACAGGGCTGCTATTAATAAGAGATGGTGGTGATGATAACAAT----------------------------
----

AACACCATCGAGATCTTCAGACCTGGAGGAGGAAATATGAGGGACAATTGGAGAAGTGAATTATATAAATATA
AAGTAGTAAAAATTGAACCATTA---GGAATAGCACCCACCAAGGCAAAGAGAAGAGTGGTG----------
----------------------------CAGAGAGAAAAAAGAGCA---GTGGGA---ATAGGAGCT------
---GTGTTCCTTGGGTTCTTAGGAGCA---GCAGGAAGCACTATG---GGCGCAGCGTCA---
GTAACGCTGACGGTACAA---GCCAGACTA---TTAATG---TCTGGTATAGTGCAACAGCAAAAC---
AACTTG---CTGAGA---GCTATTGAGGCGCAACAGCATCTGTTGCAACTCACGGTCTGGGGCATC
>FJ469753
TTCTATTGTAATTCAACACAACTGTTTAATAGTACTTGGTATACTAATGGTACTTGGAGTGGTACTAAC----
----

AAAACTGGTGATATCACACTCCCATGCAAAGTAAAACAAATTGTACACATGTGGCAGAGAGTAGGAAAAGCAA
TGTATGCCCCTCCCATCGAAGGAGTAATTAGCTGTTCATCAAAT---
ATTACAGGGCTGCTATTAACAAGAGACGGTGGTAATACCGATAATACCAAT----------------------
----

AATGCCCAGATTGAGACTTTCAGACCTGGAGGAGGAAATATGAGGGACAATTGGAGAAATGAATTATATAAAT
ATAAGGTAGTAAAAATTGAACCAATA---GGAATAGCACCCACCAAGGCAAAGAGAAGAGTGGTG--------
-------------------------------CAGAGAGAAAAAAGAGCA---GTGGGA---CTAGGAGCT---
------ATGTTCCTTGGGTTCTTGGGAGCA---GCAGGAAGCACTATG---GGCGCAGCGTCA---
ATAACGCTGACGGTACAG---GCCAGATCA---TTATTG---ACTGGTATAGTGCAACAACAGAGA---
AATTTG---CTGACG---GCTATTGAGGCGCAACAGCATCTGTTGAAACTCACAGTCTGGGGCATC
>FJ469755
TTCTACTGTAGTACAACACAACTGTTTAATAGTACTTGGACAGGGAATGATACTGCAGAAGGAAAG-------
----

GGAAATGGCACACTTACACTCCCCTGCAGAATAAGACAAATTGTAAACCTGTGGCAGGAAGTAGGAAAAGCAA
TGTATGCCCCTCCCATCTCTGGACAAATTAACTGTTCATCAAAA---
ATTACAGGGATGATCTTAACAAGAGATGGTGGTACAAAACAGAAC----------------------------
----

AACGAGAGTGAGACCTTCAGGCCTGGAGGAGGAGATATGAGGGACAATTGGAGAAGTGAATTATATAAATATA
AAGTAGTAAAAATTGAACCACTA---GGAGTAGCACCCACCAGGGCAAAGAGAAGAGTGGTG----------
----------------------------CAGAGAGAAAAAAGAGCA---GTGGGC---CTAGGAGCC------
---TTGTTCCTTGGGTTCTTGGGAGCA---GCAGGAAGCACTATG---GGCGCAGCGTCA---
GTGACGCTGACGGTACAG---GCCAGACTA---TTACTG---TCTGGTATAGTGCAGCAGCAAAAT---
AACTTG---CTGAAG---GCTATTGAGGCGCAACAGCGTATGTTGCAACTCACAGTCTGGGGCATC
>FJ469765
TTCTACTGTAATTCAACACAACTGTTTAATAGTACTTGGGATGCTAGGGAAGTTAATGAGACTAGGGAAGGGG
CAAAT--

AACACTGATAAAACTATCATACTCCCATGCAGAATAAAACAAATTGTAAATATGTGGCAGCAAGTAGGAAAGG
CAATGTATGCCCCTCCCATCAGAGGACAAATTAGATGCTCATCAAAT---
ATTACAGGACTGCTATTAACCAGAGATGGTGGTAACAACAATAAC----------------------------
----

AGTACTGAGACCTTCAGACCTGGAGGGGGAGATATGAGGGACAATTGGAGAAGTGAATTATACAAATATAAAG

TAGTAAGAATTGAACCATTA---GGAATAGCACCCACCAAGGCAAAAAGAAGAGTGGTG--------------
-------------------------CAGAGAGAAAAAAGAGCA---GTAACT---TTCGGAGCT---------
ATGTTCCTTGGGTTCTTGGGAGCA---GCAGGAAGCACTATG---GGCGCAGCGTCA---
CTAACGCTGACGGTACAG---GCCAGACAA---TTATTG---TCTGGTATAGTGCAACAGCAGAAC---
AATCTG---CTGAGA---GCTATTGAGGCGCAACAGCATCTGTTGCAACTCACAGTCTGGGGCATC
>FJ469770
TTCTACTGTAATTCAACACAACTGTTTAATAGTACTTGGAATGCTACTACAGGGTTACCTAACAAGGAA----
----

GAAAATGGCACGATCACACTCCCATGTAGAATAAAACAAATTATAAATATGTGGCAGGGAGTAGGAAGAGCAA
TATATGCCCCTCCCATCGCAGGACCAATTACCTGTTCATCAAAC---
ATTACAGGGCTAATATTAACAAGAGATGGTGGTAATGTGACGGGC----------------------------
----

CAGGAAGAAACCTTCAGACCAGGAGGAGGAAATATGAAGGACAATTGGAGAAGTGAATTATATAAATATAAAG
TAGTAAAAATTGAACCATTA---GGAGTAGCACCTACCAAGGCAAAGAGAAGAGTGGTG--------------
-------------------------CAAAGAGAAAAAAGAGAT---CTGGGA---CTAGGAGCT---------
TTGTTCCTTGGGTTCTTGGGAGCA---GCAGGAAGCACTATG---GGCGCAGCGTCA---
GTAACGCTGACGGTACAG---GCCAGACTA---TTATTG---TCTGGTATAGTGCAACAGCAGAAC---
AATTTG---CTGAGG---GCTATTGAGGCGCAACAGCATCTGTTGCAACTCACAGTCTGGGGCATC
>EF593256
TTCTACTGCAATACAACACAACTGTTTAATAGTACTTGGGATGATACTACAGCGTCAAATATCACTAAA----
----

GGAAATGACAACATCACACTCCCATGCAGAATAAGACAAGTTGTACACTTGTGGCAGGAAGTGGGAAAAGCAA
TGTATGCCCCTCCCATCCAAGGACAAATTAGATGTTCATCAAAT---
ATTACAGGGCTGCTATTAACAAGAGATGGTGGTAATAGTGAAAACAAC-------------------------
----

ACCAACACCGAGGAGGTCTTCAGACCTGCAGGAGGAGATATGAGGGACAATTGGAGAAGTGAATTATATAAAT
ATAAAGTAGTAAAAATTGAGCCATTA---GGAATAGCACCCACCAAGGCAAAAAGAAGAGTGGTG--------
-------------------------------CAGAGAGAAAAAAGAGCA---GTAGGC---ATAGGAGCT---
------GTGTTCCTCGGGTTCTTGGGAGCA---GCAGGAAGCACTATG---GGCGCAGCGTCA---
ATAACGCTGACGGTACAA---GCCAGACTA---TTATTG---TCTGGTATAGTGCAACAGCAGAAT---
AATTTG---CTGAGA---GCTATTGAGGCGCAACAGCATCTGTTGCAACTCACAGTTTGGGGCATC
>EF593299
TTCTACTGTAATACAACAAAGCTGTTTAATAGTACTTGGTCATTTAATAGTACTGGGAATGGTAGTACTACAG
AG---

TCAAACGCCACTATCACACTCCCATGCAGAATAAGACAAATTATAAACAGATGGCAAGAAGTAGGAAAAGCAA
TGTATGCCCCTCCCATCAGTGGAAAAATTAGATGTAAATCAATT---
ATTACAGGGCTGCTACTAACAAGAGATGGTGGTACAAACACCACC----------------------------
----

AACAAAACTGAGATCTTCAGACCTGGAGGAGGAGATATGAGGGACAATTGGAGAAGTGAATTATATAAATATA
AAGTAGTAAAAATCAAGCCATTA---GGAATAGCACCCACCAAGGCAAAGAGAAGAGTGGTG-----------
----------------------------CAGAGAGAAAAAAGAGCA---GTGGGAACACTAGGAGCT------
---ATGTTCCTTGGGTTCTTGGGAGCA---GCAGGAAGCACTATG---GGCGCAGCGTCA---
GTGACGCTGACGGTACAG---GCCAGACTA---TTATTG---TCTGGTATAGTGCAGCAGCAGAAC---
AATCTG---CTGAGG---GCTATTGAGGCGCAACAGCACCTGTTGCAACTCACAGTATGGGGCATC
>FJ469684
TTCTACTGTGATACAGCAAAATTGTTTAATAGTACTTGGAATAGTACTAAATCAAATAGC-------------
----

ACGGAAGTAAAGATCACACTACCATGCAAAATAAAACAAATTATAAACAGATGGCAGGAAGTAGGAAAAGCAA
TGTATGCCCCTCCCATCAGAGGACAGATTAAATGTTCATCAAAT---
ATTACAGGGCTGCTATTAACAAGAGATGGTGGTAATACCACAAAC----------------------------
----

AGGTCAGAGATCTTCAGACCTGGAGGAGGAGATATGAGGGACAATTGGAGAAGTGAATTATATAAATATAAAG

TAGTACAAATTGAACCATTA---GGAATAGCACCCACCAAGGCAAAGAGAAGAGTGGTG--------------
-------------------------CAGAGAGAAAAAAGAGCA---GTGGGA---TTAGGAGCT---------
TTGTTCCTTGGGTTCTTGGGAGCA---GCAGGAAGCACTATG---GGCGCAGCGTCA---
ATGACGCTGACGGTACAG---GCCAGACAA---TTATTG---TCTGTTATAGTGCAACAGCAGAAC---
AATTTG---CTGATG---GCTATTGAGGCGCAACAGCATCTGTTGCAACTCACAGTCTGGGGCATC
>HQ216370
TTCTACTGTGATACAACACCACTGTTTAGTAGTAGATGGTCACCTAATGGTAGTAGTAGTAGTACGATTAATG
AAACT--
-------------------AGC---
ACTGAAGGAAATATCACCACACTCCAATGCAGAATAAGACAAATTATAAATATGTGGCAGAGAGTAGGCAAAG
CAATATATGCCCCTCCCATCGCGGGACCAATTGGATGTAATTCAAGT---
ATCACAGGGCTGTTATTAACAAGAGATGGTGGGAGAAACAACAACAGC-------------------------
----

AACAACACAGAAGTTTTCAGACCTGGAGGAGGAGATATGAGGGACAACTGGAGAAGTGAATTATACAAATATA
AAATAGTAAGGATTGAACCATTA---GGAATAGCACCCACCAAGGCAAAGAGAAGAGTGGTG-----------
----------------------------CAAAGAGAAAAAAGAGCA---GTGGGA---ATAGGAGCT------
---GTGTTCCTTGGGTTCTTGGGAGCA---GCAGGAAGCACTATG---GGCGCAGCATCA---
ATAACGCTGACGGTACAG---GCCAGACAA---TTATTG---TCTGGTATAGTGCAACAGCAGAAC---
AATTTG---CTGAGA---GCTATTGAGGCGCAACAGCACCTGTTGCAACTCACGGTCTGGGGCATC
>HQ216504
TTCTACTGTAATACAACAGCACTGTTTAATAGCACCTGGAATATTACTGAAGGGTTAAATAGCACATTCTCAA
ATAAC--
-------------------CCT---
GAAGGAAATGACACAATCACGCTTCCATGCAGAATAAAACAAATTATAAACATGTGGCAGGAAGTAGGAAAAG
CAATGTATGCCCCTCCCATCAGAGGACAAATTAGATGTTCATCAAAT---
ATTACAGGGCTGCTATTAACAAGAGATGGTGGTGATAACAATAAC----------------------------
----

AGTGACAACGAGACCTTCAGACCTGGAGGAGGAGATATGAGGGACAATTGGAGAAGTGAATTATATAAATATA
AAGTAGTAAAAATTGAACCATTA---GGAGTAGCACCAACCAAGGCAAAGAGAAGAGTGGTG-----------
----------------------------CAGAGAGAAAAAAGAGCA---ATAGGA---ATAGGAGCT------
---GTGTTCCTTGGGTTCTTGGGAGCA---GCAGGAAGCACTATG---GGCGCAGCGTCA---
ATAACGCTGACGGTACAG---GCCAGACAA---TTATTG---TCTGGTATAGTGCAACAGCAGAAC---
AACCTG---CTGAGA---GCTATTGAGGCGCAACAACATATGTTGCAACTCACAGTCTGGGGCATC
>JX863969
TTCTACTGTAATACAACACAACTGTTTAATAGTACTTGGAATAGTACTGAAGGGATTGAAGGGACAAAT----
----

AACACTGATAATATCACACTCCCATGCAGAATAAAACAAATTATAAACCTGTGGCAGAGAGTAGGAAAAGCAA
TGTATGCCCCTCCCATCAGAGGACAGATTAGATGTTCATCAAAT---
ATCACAGGGCTGCTATTAGTAAGAGATGGTGGTAACGAACAGAACGGG-------------------------
----

ACAAGCGCAACCGAGATCTTCAGACCTGGAGGAGGAGACATGAGGGACAATTGGAGAAGTGAATTATATAAAT
ATAAAGTAGTAGAAATTAAACCATTA---GGAGTAGCACCCACCAGGGCAAAGAGAAGAGTGGTG--------
-------------------------------CAGAGAGAAAAAAGAGCA---GTGACA---CTAGGAGCT---
------TTGTTCCTTGGGTTCTTGGGAGCA---GCAGGAAGCACTATG---GGCGCAGCGTCA---
ATAACGCTGACGGTACAG---GCCAGACAA---TTGTTG---TCTGGTATAGTGCAACAGCAGAAC---
AATCTG---CTGAGG---GCTATTGAGGCGCAACAGCGTCTGTTGCAACTCACAGTCTGGGGCATC
>JN562798
TTCTACTGTACTACTACAGGACTGTTTAATAGTACTTGGCATTGGAATGGTACTAGGGAGAATAATACTACGG
AG---

CCAAATAGCAATATCACACTCCCATGCAGAATAAAACAGATTATAAACATGTGGCAAGAAGTAGGAAAAGCAA
TGTATGCCCCTCCAATCAAAGGACAAATTAGATGTTCATCAAAC---
ATTACAGGGCTGCTAATAACAAGAGATGGTGGTAACAGTGACCATAGC-------------------------
----

AACGAGACCGAGATCTTCAGACCTGGAGGAGGAGATATGAGGGACAATTGGAGAAGTGAATTATATAAATATA

AAGTAGTAAAAATTGAACCATTA---GGAGTAGCACCCACTGAGGCAAGGAGAAGAGTGGTG----------
----------------------------CAGAGAGAAAAAAGAGCG---ATAACA---CTGGGAGCT------
---TTGTTCCTTGGGTTCTTGGGAGCA---GCAGGAAGCACTATG---GGCGCAGCGTCA---
ATGACGCTGACGGTACAG---GCCAGACAA---TTATTG---TCTGGTATAGTGCAACAGCAGAAC---
AATTTG---CTGAGG---GCTATTGAGGCGCAACAGCATCTGTTGCAACTCACAGTCTGGGGCATC
>EU577811
TTCTACTGTAATACAACACAACTGTTCAATAGTACTTGGTATTGGAATAATACTCGGGATGAAGGGTCAAATA
ACACT---

GGAGGCAATGGCACAATCATACTCCCGTGTAGAATAAAACAATTTATAAACATGTGGCAGGAAGTAGGAAAAG
CAATGTATGCCCCTCCCATCAGAGGACAAATTAGCTGTTCATCAAAT---
ATTACAGGGCTGATGCTATTAAGAGATGGTGGTAATAACGTGAGC----------------------------
----

AATACCACTGAGACCTTCAGACCTGGAGGAGGAGATATGAGGGACAATTGGAGAAGTGAATTATATAAATATA
AAGTAGTAAAAATTGAACCATTA---GGAATAGCACCCACCAAGGCAAAGAGAAGAGTGGTG----------
----------------------------CAGAGAGAAAAAAGAGCA---GTGGGA---ATAGGAGCT------
---ATGTTCCTTGGGTTCTTGGGAGCA---GCAGGAAGCACTATG---GGCGCAGCGTCA---
GTGACGCTGACGGTACAA---GCCAGGCTA---TTATTG---TCTGGTATAGTGCAACAGCAGAGC---
AATTTG---CTGAGA---GCTATTGAGGCGCAACAGCATCTGCTGCAACTCACAGTCTGGGGCATC
>EU577862
TTCTATTGTAATACATCACAACTGTATGATAGTACTTGGAATCTTACTAATACTGAAAGG------------
----

TTAAATGACACTATCCAACTCCCATGCAGAATAAAACAAATTATAAACATGTGGCAGGAAGTAGGAAAAGCAA
TGTATGCCCCTCCCATCAGAGGACTAATTAAGTGTTCATCAAAT---
ATTACAGGGCTGTTATTAACAAGAGATGGTGGTAGTGATAATGCCACA------------------------
----

AACGGTACTGAGACCTTCAGACCTGGAGGAGGAGATATGAGGGACAATTGGAGAAGCGAATTATACAAATATA
AAGTAATAGAAATTAAACCAGTA---GGATTAGCACCCACCAAGGCAAAGAGAAGAGTGGTG----------
----------------------------CAGAGAGAAAAAAGAGCA---GCA------CTAGGAGCT------
---ATGCTCCTTGGGTTCTTGGGAGCA---GCAGGAAGCACTATG---GGCGCAGCGTCA---
ATAACGCTGACGGTACAG---GCCAGACAA---CTGTTG---TCTGGTATAGTGCAACAGCAAAAT---
AATTTG---CTGAGA---GCTATTGAGGCGCAACAGCATCTATTGCAACTCACAGTCTGGGGCATT
>EU577947
TTCTACTGCAATACAACACAACTGTTTAATAGTACTTGGCTGTTTAATGGTACTTGGAATGGTACTGCATTAA
ATGGC---
-------------------ACT---
GAAGGAAATGAAACAATTACACTTCCATGCAGAATAAAACAAATTATAAACATGTGGCAGGAAGTAGGAAAAG
CAATGTATGCCCCTCCCATCAAAGGACTAATTGAATGTTCATCAAAT---
ATTACAGGGCTACTGTTAACAAGAGATGGTGGTAATAATGAGAGC----------------------------
----

ACTGAGACCTTTAGGCCTAGAGGAGGAGATATGAGGGACAATTGGAGAAGTGAATTATATAAATATAAAGTAG
TACAAGTTGAACCATTA---GGAATAGCACCCACCAGGGCAAAGAGAAGAGTGGTG----------------
----------------------CAGAGAGAAAAAAGAGCA---GGA------ATAGGAGCT---------
CTGTTCCTTGGGTTCTTGGGAGCA---GCAGGAAGCACTATG---GGCGCAGCTTCG---
ATAACGCTGACGGTACAA---GCCAGACAA---TTATTG---TCTGGTATAGTGCAACAGCAGAAC---
AATCTG---CTGAGG---GCTATTGAGGCGCAACAGCATATGTTGCAACTCACAGTCTGGGGCATC
>EU577989
TTCTACTGTAACACATCAAAACTGTTTAATAGTAGTTGGAATGCTGATGGTAGTGTTAATACTACTGGA----
----

GAATCAGGAAACATAACACTCCAATGCAGAATAAAACAAATTATAAACATGTGGCAGGAAGTAGGAAAAGCAA
TGTATGCCCCTCCCATCCAAGGACAAATTAATTGTTCATCAAAC---
ATTACAGGGATACTATTAACAAGAGATGGTGGTAGTGATAACACAACAAAC---------------------
----

AATACTGAGATCGAGATCTTCAGACCTACAGGAGGAGATATGAGGGACAATTGGAGAAGTGAATTATATAAAT

ATAAAGTAGTAAGAATTGAACCATTA---GGAATAGCACCCACCAAGGCAAAGAGAAGAGTGGTG--------
-------------------------------CAGAGAGATAAAAGAGCA---GTGGGA---CTAGGAGCT---
------TTGTTCCTTGGGTTCTTGGGAACA---GCAGGAAGCGCTATG---GGCGCAGCGTCA---
CTGACGCTGACGGTACAG---GCCAGACAA---TTATTG---TCTGGTATAGTGCAACAGCAAAAC---
AATTTG---CTGAGG---GCTATTGAGGCGCAACACCATATGTTGCAACTCACAGTCTGGGGCATC
>KX156438
TTTTACTGTAATACAACACAAATGTTTAATAGTACTTGGAATGATACTGAGAATAATATT-------------
----

GGAAATGGAACTATCACACTCCCCTGCAGAATAAAACAAATTATAAATAAGTGGCAGGAAGTAGGAAAAGCAA
TGTATGCCCCTCCCATTGAAGGACGAATTAACTGTACATCAAAT---
ATTACAGGGCTGATATTAACAAGAGATGGTGGTAGCGGGAACAAC----------------------------
----

GACACCAACACCACCGAGATCTTCAGGCCTGCAGGGGGAGATATGAGGGACAATTGGAGAAGTGAATTATATA
AATATAAGGTAGTAAAAATTGAACCATTA---GGAGTAGCACCCACCAAGGCAAAGAGAAGAGTGGTA-----
----------------------------------CAGAGAGAAAAAAGAGCA---GTGGGA---
ATAGGAGCT---------TTGTTCCTTGGGTTCTTGGGAGCA---GCAGGAAGCACTATG---
GGCGCAGCGTCA---ATAACGCTGACGGTACAG---GCCAGACAA---TTACTG---
TCTGGTATAGTGCAACAGCAAAAC---AACTTG---CTGAGA---
GCTATTGAGGCGCAACAGCATCTGTTGCAACTCACAGTCTGGGGCATC
>EU578032
TTCTACTGTAACTCAACACAACTGTTTAATAGCTCTTGGAATGGTACTGCTGGGAATGTTACTACAGGGGGGT
CA---

GATAACACCACTATCACACTCCCATGTAGAATAAAACAAATTATAAACATGTGGCAGGGAGTAGGAAAAGCAA
TGTATGCCCCTCCGATCCCAGGAAAAATTAGCTGTAAATCAATT---
ATTACAGGGCTGCTATTAACAAGAGATGGTGGCAACAATAACAGT----------------------------
----

GAGACCTTCGAAACCTTCAGACCTGGGGGAGGAAATATGAAAGACAATTGGAGAAATGAATTATATAAATATA
AAGTAGTAAAAATTGAACCAATA---GGAGTAGCACCCACCAAGGCAAAGAGAAGAGTGGTG-----------
----------------------------CAGAGAGAAAAAAGAGCA---GTGGGA---ATAGGAGCT------
---GTGCTCTTTGGGTTCTTGGGAGCA---GCAGGAAGCACTATG---GGCGCAGCGTCG---
ATAGCGCTGACGGTACAG---GCCAGACAA---TTATTG---TCTGGTATAGTGCAACAGCAGAAC---
AATCTG---CTGAGG---GCTATTGAGGCGCAACAACATATATTGCAACTCACAGTCTGGGGCATC
>KX156451
TTTTACTGTAACACAACACAACTGTTTAATAGTACTTGGAATATTACTGAAGTGTTAAATAGTACTGGAGAGT
CAAAA--

AGCAATGACTTAATCATACTCCCATGCAGGATAAAACAAATCGTAAACATGTGGCAGAGAGTAGGACAAGCAA
CGTATGCCCCTCCCATCGAAGGATTGATTAGATGTTCATCAAAA---
ATTACAGGGATACTATTAACAAGAGATGGTGGTATTAACAATAGG----------------------------
----

ACCAACGAAACCTTCAGACCTGGAGGAGGAGACATGAGAGACAATTGGAGAAGTGAATTATATAGATATAAGG
TAGTACAAATTGAACCAGTA---GGACTGGCACCCACCAAGGCGCAGCGAAGAGTGGTG--------------
-------------------------CAGAGGGAAAAAAGAGCA---GTGGGA---ATAGGAGCG---------
TTGTTCCTTGGGTTCTTGGGAACA---GCAGGAAGCACTATG---GGCGCAGCGTCA---
ATAACGCTGACGGCACAG---GCCAGACAA---TTATTG---TCTAGTATAGTGCAACAGCAGAAC---
AACTTG---CTGAGA---GCTATTGAGGCGCAACAGCATCTGTTGCAACTCACAGTCTGGGGCATC
>JN562778
TTCTACTGTAATTCAACACAACTGTTTAATAGTACTTGGATTAGGAATGATACTAATAATACTTGGCATAGTG
AAGGG--
-------------------TTA---
AATGGCACTGAAACAATCACACTCCCATGTAGAATAAAACAATTTATAAACATGTGGCAGGAAGTAGGAAAAG
CAATGTATGCCCCTCCCATCAGAGGACAAATAAAATGTTCATCAAAT---
ATTACAGGGCTATTACTAGTAAGAGATGGTGGTAATAACAATAAGAGCAAT----------------------
----

AGCACTAACAATGCTGAGATCTTCAGACCTGGAGGAGGAAATATGAAAGACAATTGGAGAAGTGAATTATATA
GATATAAAGTGGTACAAGTTGAACCATTA---GGAATAGCACCCACCAGGGCAAAGAGAAGAGTGGTG-----
----------------------------------CAGAGAGAAAAAAGAGCT---GTGGGA---
GTAGGAGCT---------TTGTTCCTTGGGTTCTTGTCAGCA---GCGGGAAGCACTATG---
GGCGCAGCGTCA---ATGACGCTGACGGTACAG---GCCAGACAA---TTATTG---
TCTGGTATAGTGCAACAGCAGAGC---AATCTG---CTGAGG---
GCTATTGAGGCGCAACAGCATATGTTGCAACTCACAGTCTGGGGCATC
>JN562811
TTCTACTGTAATTCATCAGGACTGTTTAATAGTACTTGGCATAGTAATAGTACCCAGAATAGTACTGAAGGG-
----

TCAAAGGACACTATTACACTCCCATGCAGAATAAGACAAATCATAAACATGTGGCAGGAAGTAGGAAAAGCAA
TGTATGCCCCTCCCATCCAAGGACAACTTAGATGTTCATCAAAT---
ATTACAGGGCTGCTCTTAACAAGAGATGGAGGTAATAACGGGGGC----------------------------
----

GACAACACCGAGATCTTCAGACCTGGAGGAGGAAATATGAAAGACAATTGGAGAAGTGAATTATATAAATATA
AAGTAGTAAAAATTGAACCATTA---GGATTAGCACCCACCAGGGCAAGGAGAAGAGTGGTG-----------
----------------------------CAGAGAGAAAAAAGAGCA---GTGGGA---ATGGGAGCT------
---TTGTTCCTTGGGTTCTTGGGAGCA---GCAGGAAGCACTATG---GGCGCAGCGTCA---
ATGACGCTGACGGTACAG---GCCAGAACA---CTATTG---TCTGGTATAGTGCAACAGCAAAGC---
AACTTG---CTGAGG---GCTATTGAGGCGCAACAGCATCTGTTGCAACTCACAGTCTGGGGCATC
>DQ869015
TTCTACTGTGATACAACACAACTCTTTAATAGTACTTGGAATGATACTGTTGGGAATGATACTGAAGAG----
----

TCAAATAATACTATCACACTCCCATGCAGAATAAAACAAATTGTAAACCTGTGGCAGGAAGTAGGAAAAGCAA
TGTATGCCCCTCCCATTAGAGGACAAATTAGATGTTCATCAAAT---
ATTACAGGGCTGCTATTAACAAGAGATGGTGGTAATATCAGCACC----------------------------
----

ACCACCACCGAGACCTTCAGACCTGGAGGAGGAAATATGAGGAACAATTGGAGAAGTGAATTATATAAATATA
AAGTAGTAAAAATTAAACCATTA---GGAGTAGCACCCACCAGCGCAAGGAGAAGAGTGGTG-----------
----------------------------CAGAGAGAAAAAAGAGCA---ATAGGA---ATAGGAGCT------
---ATGTTCCTCGGGTTCTTGGGAGCA---GCAGGAAGCACTATG---GGCGCAGCGTCA---
ATAACGCTGACGGTACAG---GCCAGACAA---TTATTG---TCTGGTATAGTGCAACAGCAAAGC---
AATTTG---CTGAGG---GCTATAGAAGCGCAACAGCATCTGTTGCAGCTTACAGTCTGGGGCATT
>DQ869033
TTCTACTGTAATACAACAAAACTGTTTAATAGTACTTGGAATGAGACTAGTATTGAAAATAGCACTAGAGGAA
AT---

AGCACTGGAGGAGATATCACAATTCTATGCAGAATAAAACAAGTTATAAACCTGTGGCAGGAAGTAGGAAAAG
CAATGTATGCCCCTCCCATCGAAGGACTAATCAATTGTACATCAAAT---
GTTACAGGGCTATTATTAATAAGAGATGGTGGTAACATTACCAAC----------------------------
----

AACGACACAGAGATCTTTAGACCTGGAGGAGGAGATATGAGGGACAATTGGAGAAGTGAACTGTACAAATATA
AAGTAGTAAGAATTGAACCATTA---GGAATAGCACCCACCAAGGCAAAGAGAAGAGTGGTG-----------
----------------------------CAGAGAGAAAAAAGAGCA---GTGGGA---CTAGGAGCT------
---ATGTTCCTTGGGTTCTTGGGAGCA---GCAGGAAGCACTATG---GGCGCAGCATCA---
GTGACGCTGACGGTACAG---GCCAGACAA---TTATTG---TCTGGTATAGTGCAACAGCAGAAC---
AATCTG---CTGAGG---GCTATTGAGGCGCAACAGCATCTGTTGCAACTCACAGTCTGGGGCATC
>KM259097
TTCTACTGTAATACAACAAAACTGTTTAATAGTACTTGGAATGATACTGCAGGG-------------------
----
AATGATACTGCAGGGAAT---------
GTTACTGAAGAAAATATAACACTCCCATGCAGAATAAAACAAATTATAAACATGTGGCAGGAAGTAGGAAAAG
CAATGTATGCCCCTCCCATCAGAGGACAAATTAGCTGTTCATCAAAT---
ATTACAGGGCTGCTATTAACAAGAGATGGTGGA--

AAAACCAACGAAACAAAC---
GAGACCGAGATCTTTAGACCTGGAGGAGGGAATATGAAGGACAATTGGAGAAGTGAATTATATAAATATAAAG
TAGTAAAAATTGAACCATTA---GGAGTAGCACCCACCAAGGCAAAGAGAAGAGTGGTG--------------
-------------------------CAGAGAGAAAAAAGAGCA---GTGGGAGTAATAGGAGCT---------
ATGTTCCTTGGGTTCTTGGGAACA---GCAGGAAGCACTATG---GGCGCAGCGTCA---
GTGACGCTGACGGTACAG---GCCAGACAA---TTATTG---TCTGGTATAGTGCAACAGCAGAAC---
AATTTG---CTGAGA---GCTATTGAGGCGCAACAGCATCTGTTGCAACTCACAGTCTGGGGCATC
>DQ410535
TTCTACTGTAATTCAACACCACTGTTTAATAGTAGTTGGAATGATACTACCATAGAGCAAAATGACACA----
----

ACGGAAGAGAATATCACACTCACATGCAGAATAAAACAAATTATAAATATGTGGCAGGAAGTAGGAAAAGCAA
TGTATGCCCCTCCCATCAGAGGACAAATTAGATGTATATCAAAC---
ATTACAGGGCTGCTATTAACAAGAGATGGTGGTATAAATGAGACC----------------------------
----

AACGGGACTGAGATCTTCAGACCTGGAGGAGGAGATATGAGGGACAATTGGAGAAGTGAATTATACAAATATA
AAGTAGTAAAAATTGAGCCATTA---GGAGTAGCACCCACCAAGGCAAAGAGAAGAGTGGTG-----------
----------------------------CAGAGGGAAAAAAGAGCA---GTGGGG---ATAGGAGCT------
---CTGTTCCTTGGGTTCTTGGGAGCA---GCAGGAAGCACTATG---GGCGCAGCGTCA---
GTGACGCTGACGGTACAG---GCCAGGCAA---TTATTG---TCTGGTATAGTGCAACAGCAGAAC---
AATTTG---CTGAGG---GCTATTGAGGCGCAACAGCATCTGTTGCAACTCACAGTCTGGGGCATC
>AY835452
TTTTACTGTAACTCAACACAGCTGTTTAATAGTACTTGGTGGCCTAATGGTACTACGACTGAATGGTCAAATG
AA---

ACGTCAAATGGGACTATCACACTCCCATGCAGAATAAAACAAATTATAAACATGTGGCAGGAAGTAGGAAAAG
CAATGTATGCCCCTCCTATCAGCGGACCAATTAGCTGTTCATCGAAT---
ATTACAGGGCTGCTATTAGTAAGAGATGGTGGTAATGACAATGAGACT-------------------------
----

AACGGGACTGAGACCTTCAGACCTGGAGGAGGAGATATGAGAGACAATTGGAGAAGTGAATTATATAAATATA
AAGTAGTAAAAATTGAACCATTA---GGAGTAGCACCCACCAAGGCAAAGAGAAGAGTGGTG-----------
----------------------------CAAAGAGAAAAAAGAGCA---GTGGGA---CTAGGAGCT------
---ATGTTCCTTGGGTTCTTGGGAGCA---GCAGGAAGCACTATG---GGCGCAGCGTCA---
ATAACGCTGACGGTACAG---GCCAGACTA---TTATTG---TCTGGTATAGTGCAACAGCAAAAC---
AACTTG---CTGAGA---GCTATTGAGGCGCAACAGCATCTGTTGCAACTCACAGTCTGGGGCATC
>KX156493
TTCTACTGTAATTCAACACCACTGTTTAATAGTATTTGGAATGTTAGTAGTGGTTGGAATGGTACTAAAGGG-
----

TCAAATGGCACTATCACACTCTCATGCAGAATAAAACAAATTATAAACATGTGGCAGGAGGTAGGAAAAGCAA
TGTATAACCCTCCCATCAGTGGAATAATTAGATGTAACTCAACT---
ATTACAGGGCTGCTATTAACAAGAGATGGTGGTGAGAACAGGTCTGGGAGT----------------------
----

GATAACTGGACCGAGACCTTCAGACCTGGGGGAGGAGATATGAGGGACAATTGGAGAAGTGAATTATATAAAT
ATAAAGTAGTAAAAATTGAACCATTA---GGAATAGCACCCACCAGGGCAAAGAGAAGAGTGGTG--------
-------------------------------CAAAGAGAAAAAAGAGCA---GTGGGA---TTTGGAGCT---
------ATGTTCCTTGGGTTCTTGGGAGCA---GCAGGAAGCACTATG---GGCGCAGCGTCA---
GTGACGCTGACGGTACAG---GCCAGACAA---TTATTG---TCTGGTATAGTGCAACAGCAGAGC---
AACTTG---CTGAGG---GCTATTGAGGCGCAACAGCATCTGTTGCAACTCACAGTCTGGGGCATC
>KX156542
TTCTACTGTGATTCAACACAGCTATTTAATAGTACTTGGAATAATACTGAACAGCCTACCTGGAATGAT----
----

ACTGGAGAAATTATCACACTCCCGTGCAGAATAAAACAAATTATAAACAGGTGGCAAGAAGTAGGAAAAGCAA
TGTATGCCCCTCCCATCAGAGGACAAATTAGCTGCTCATCACTT---
ATTACAGGGATACTATTAACAAGAGATGGTGGTAAAAAGGAGAGC----------------------------
----

AATGAAACCGAAACCTTTAGACCTGGAGGAGGAGATATGAGGGACAATTGGAGAAATGAATTATATAAATATA
AAGTAGTAAAAATTGAGCCATTA---GGAGTAGCACCCACCAAGGCAAAGAGAAGAGTGGTG-----------
----------------------------CAAAGAGAAAAAAGAGCA---GTGGGAACAATAGGAGCT------
---ATGTTCCTTGGGTTCTTGGGAGCA---GCAGGAAGCACTATG---GGCGCAGCGGCA---
GTAACGCTGACGGTACAG---GCCAGACAA---TTATTG---TCTGGTATAGTGCAACAGCAAAAC---
AACTTG---CTGAGG---GCTATTGAGGCGCAACAGCATATGTTGCAACTCACAGTCTGGGGCATC
>KX156380
TTTTATTGTAATTCAACACCACTGTTTAATAGTACTTGGGCTTGGAATGGTACTTTCAATAAGGAAAATAATA
CTGAAAAT---

TCAAATAGCACTATCACCCTCCAATGCAGAATAAGGCAAATTGTAAACATGTGGCAGGAAGTAGGAAAAGCAA
TGTATGCCCCTCCCATCAGAGGACAAATTAATTGTTCATCAAAC---
ATTACAGGGCTGCTATTACTAAGAGATGGTGGTGTTAATAATAACACA-------------------------
----

AATGATACAGAGATCTTCAGACCTGGAGGAGGAAACATGAAGGACAATTGGAGAAGTGAATTATATAAGTATA
AAGTAGTACAAATTGAACCATTA---GGAGTAGCACCCACCAAGGCAAAGAGAAGAGTGGTG-----------
----------------------------CAGAGAGAAAAAAGAGCA---GTAACA---CTGGGAGCT------
---ATGTTCCTTGGGTTCTTGGGAGCA---GCAGGAAGCACTATG---GGCGCAGCGTCA---
ATGGCGCTGACGGTACAA---GCCAGACAA---TTGTTG---TCTGGTATAGTGCAACAGCAAAAC---
AACTTG---CTGAGA---GCTATTGAGGCGCAACAGCACTTGTTGCAACTCACAGTCTGGGGCATT
>FJ469697
TTCTACTGTGATACAACACAACTGTTTAATAGTACTTGGAATGATACTGAAGAGAATAGTGATAAAGGGAACA
CT---

GAAGGAAATGAACCAATCATACTCCCATGCAGAATAAAACAATTTATAAACATGTGGCAAGCAGTAGGAAAAG
CAATGTATGCCCCTCCCATCAGAGGACTAATTAGATGTTCATCAAAC---
ATTACAGGGCTGTTATTAACAAGAGATGGTGGTAACGAGACTAAT----------------------------
----

ACTACTGAGACCTTCAGACCGCAAGGAGGAAATATGAGGGACAATTGGAGAAGTGAATTATATAGATATAAAA
TAGTGAAAATTGAACCATTA---GGGGTAGCACCCACCAAGGCAAAAAGAAGAGTGGTG--------------
-------------------------CAGAGAGAAAAAAGAGCA---GTGGGAACATTAGGAGCT---------
ATGTTCCTTGGGTTCTTGGGAGCA---GCAGGAAGCACTATG---GGCGCAGCGTCA---
TTGACGCTGACGGTACAG---GCCAGACTA---TTACTG---TCTGGTATAGTGCAACAGCAGAAT---
AATATG---CTGAGG---GCTATTGAGGCGCAACAGCATCTGTTGCAACTCACAGTCTGGGGCATC
>FJ469698
TTCTACTGTAATTCAACACCACTGTTTAATAGTACTTGGCATAGTAATGGTACTATAGAGTCAGCT-------
----

AACAGAACAAATATCAGACTCATGTGCAGAATAAAACAAATTATAAACATGTGGCAGGGAGTAGGAAAAGCAA
TGTATGCCCCTCCCATCACAGGATTAATTAGGTGTTCATCAAAT---
ATTACAGGGCTGCTATTAACAAGAGATGGTGGTCTTAATAATAGT----------------------------
----

GACAATGAGACCTTCAGACCTGGAGGAGGAAATATGAAGGACAATTGGAGAAGTGAATTATATAAATATAAAG
TAGTAAGAGTTGAACCACTA---GGAGTAGCACCCACCAAGGCAAAGAGAAGAGTGGTG--------------
-------------------------CAGAGAGAAAAAAGAGCA---TTAGGGGTACTAGGAGCT---------
GTGTTCCTTGGGTTCTTAGGAGCA---GCAGGAAGCACTATG---GGCGCAGCGTCA---
ATAGCGCTGACGGCACAG---GCCAGACTA---TTATTG---TCTGGTATAGTGCAACAGCAGAAC---
AACCTG---CTGAGG---GCTATTGAGGCGCAACAACATCTGTTGCAACTCACAGTCTGGGGCATT
>HQ217185
TTCTACTGTAATACAACACAATTGTTTAATAGTACTTGGAAGAGTACCGAGTCTAATGATACTGGG-------
----

GGAAATGAAACTATCATACTCCCATGCAGAATAAAACAAATTGTAAACATGTGGCAGGAAGTAGGAAAAGCAA
TGTATGCCCCTCCCATCAGTGGACTAATTAACTGCTCATCAAAT---
ATTACAGGGTTATTATTAACAAGAGATGGTGATAATAGTACTAATGAC-------------------------
----

ACTGACACTGAGATCTTCAGACCTATAGGAGGAAACATGAAAGACAATTGGAGAAGTGAATTATATAAATATA
AAGTAGTAAGAATTGAACCATTA---GGACTAGCACCCACCAAGGCAAAGAGAAGAGTGGTG-----------
----------------------------CAGAGAGAAAAAAGAGCA---GTGGGA---TTAGGAGCT------
---GTGTTCTTTGGGTTCTTGGGAGCA---GCAGGAAGCACTATG---GGCGCAGCGTCA---
ATGACGCTGACGGTACAG---GCCAGGCAA---TTACTG---TCTGGTATAGTGCAGCAGCAGAAC---
AATCTG---CTGAGG---GCTATTGAGGCGCAACAACATCTGTTGCAACTCACAGTCTGGGGCATC
>KC935957
TTCTACTGTAATTCAACACAACTATTTAATAGTACTTGGAATGGAACTGAGTCAAATGACACTAGA-------
----

GGAAATGACACAATCACACTCCCATGCAGAATAAAACAAATTATAAACATGTGGCAGGAAGTAGGAAAAGCAA
TGTATGCCCCTCCCATCAGAGGACAAATTAGATGTTCATCAAAT---
ATTACAGGGCTGCTATTAACAAGAGATGGTGGTGAAAACAAGAAC----------------------------
----

AAGACCGAGGTCTTCAGACCTGGAGGGGGAGATATGAGGGACAATTGGAGAAGTGAATTATATAAATATAAAG
TAGTAAAAATTGAACCATTA---GGAATAGCACCCACCAGGGCGAAGAGAAGAGTGGTG--------------
-------------------------CAGAGAGAAAAAAGAGCA---GTGGGA---ATAGGAGCT---------
GTGTTCCTTGGGTTCTTGGGAGCA---GCAGGAAGCACTATG---GGCGCAGCGTCA---
CTAGCGCTGACGGGACAG---GCCAGACTA---TTATTG---TCTGGTGTAGTGCAACAGCAGAAC---
AATTTG---CTGAGG---GCTATTGAGGCGCAACAGCATCTGTTGCAACTCACAGTCTGGGGCATC
>DQ410068
TTCTACTGTGATTCAACACAACTGTTTAACAGTACTTGGACTGAAGGGTCAAATAACACTGAA----------
----

GGAAATGACCCAATCACACTCCCATGCAGAATAAAACAAATTATAAACATGTGGCAAGAAGTAGGAAAAGCAA
TGTATGCCCCTCCCGTCGGAGGACAAATTAGATGTTCATCAAAT---
ATTACAGGACTGATCTTGATAAGAGATGGTGGTAATAGTAGTTCC----------------------------
----

CACAATGAGACTTTCAGACCTGGAGGAGGAGATATGAGAGACAATTGGAGGAGTGAATTATATAAATATAAAG
TAGTAAAAATTGAACCATTA---GGAGTAGCACCCACCAAGGCAAAGAGAAGAGTGGTG--------------
-------------------------CAGAGAGAAAAAAGAACA---GTGGGA---ATAGGAGCT---------
GTGTTCCTTGGGTTCTTGGGAGCA---GCAGGAAGCACTATG---GGCGCAGCGTCA---
GTGACGTTGACGGTACAG---GCCAGACTA---TTGTTA---TCTGGTATAGTGCAACAGCAGAAC---
AATCTA---CTGAGG---GCTATTGATGCGCAACAGCATCTGTTGCAACTCACAGTCTGGGGCATC
>EF363124
TTCTACTGCGATTCAACACCACTGTTTAATAGTACTTGGAATGTTACTAGTACTTGGAATGTTACTAGTACTG
GGAAT--
-------------------GAT---
ACTGAGTCAAATAACATCACACTCCCATGCAGAATAAAGCAAATTATAAACATGTGGCAGGAAGTAGGAAAAG
CAATATATGCCCCTCCCATCAGAGGACAAATTAGATGTTCATCAAAT---
ATTACAGGGCTGCTATTAACAAGAGATGGTGGTAATAACAAGAAC----------------------------
----

GAGACCGAGATCTTCAGACCTGGAGGAGGAGATATGAGGGACAATTGGAGAAGTGAATTATATAAATATAAAG
TAGTAAAAATTGAACCATTA---GGAATAGCACCCACCAAGGCAAAGAGAAGAGTGGTG--------------
-------------------------CAGAGAGAAAAAAGAGCA---GTGGGA---ATAGGAGCT---------
GTGTTCCTTGGGTTCTTGGGAGCA---GCAGGAAGCACTATG---GGCGCAGCGTCA---
ATGACGCTGGCGGTACAG---GCCAGACTA---TTATTG---TCCGGTATAGTGCAACAGCAGAAC---
AATTTG---CTGAGG---GCTATTGAGGCGCAACAGCATCTGTTGCAACTCACCGTCTGGGGCATC
>EF363126
TTCTACTGTAACACATCACAACTGTTTAATAACACTTGGAATGGTACTGAGTTAAGTAATAGTAAA-------
----

GAAAATGGTAATATCACACTCCAATGCAGAATAAAACAAATTATAAACATGTGGCAGGAAGTAGGGAAAGCAA
TGTATGCCCCTCCCATCAGAGGACAAATTAGATGTGTGTCAAAT---
ATTACAGGGCTGCTATTAACAAGAGATGGTGGTACAAACAGCACC----------------------------
----

ACCGAGACCTTCAGGCCTGCAGGAGGAAATATGAGGGACAATTGGAGAAGTGAATTATATAAATATAAAGTAG
TAAAAATTGAACCATTA---GGAATAGCACCCACCAAGGCAAAGAGAAGAGTGGTG-----------------
----------------------CAGAGAGAAAAAAGAGCA---GCGGGA---ATAGGAGCT---------
TTTTTCCTTGGGTTCTTGGGACTA---GCAGGAAGCACTATG---GGCGCAGCGTCA---
GTGACGCTGACGGTACAG---GCCAGACAA---TTATTG---TCTGGTATAGTGCAACAGCAGAAC---
AATCTG---CTGAGG---GCTATTGAGGCGCAACAGCACATGTTGCAACTCACAGTCTGGGGCATC
>DQ410498
TTCTACTGTAACTCAACAAAACTGTTTAATAGTACTTGGAATGATACTAATAGTACTTGGAATGGTACTGAGT
CACATAAC---
-------------------ACA---
AAAGAAAATGACACAATCATACTCCAATGCAGAATAAAACAAATTATAAACATGTGGCAGAAAGTAGGAAAAG
CAATGTATGCCCCTCCCATCATAGGACAAATTAGATGTTCATCAAAT---
ATTACAGGGCTGCTATTAACAAGAGATGGTGGTAATAGCGGGAACAAT-------------------------
----

ACCACCACCGAGACCTTCAGACCTGGAGGAGGAGAGATGAGGGACAATTGGAGAAATGAATTATATAAATATA
AAGTAGTAAAAATTGAACCATTA---GGAGTAGCACCCACCAAGGCAAAGAGAAGAGTGGTG-----------
----------------------------CAGAGAGAAAAAAGAGCA---GTGGGA---ATAGGAGCT------
---CTGTTCCTTGGGTTCTTGGGAGCA---GCAGGAAGCGCTATG---GGCGCAGCGTCA---
ATGGCGCTGACGGTACAG---GCCAGACCA---TTATTG---TCTGGTATAGTGCAACAGCAGAAC---
AATTTG---CTGAGG---GCTATTGAGGCGCAACAGCATCTGTTGCAACTCACAGTCTGGGGCATC
>JQ251132
TTCTACTGTAATACAACACCACTGTTTAATAGTAAGTGGACCAATGGTAATGGGAGTGGTTCTGAA-------
----

GGGAATGAGACTATCACACTCCAATGTAGAATAAAACAATTTATAAACATGTGGCAGGAAGTAGGAAAAGCAA
TGTATGCCCCTCCTATCAAAGGACAAATTAGATGTTTATCAAAT---
ATTACAGGGCTACTATTAACAAGAGATGGGGGTAATAACCAGAACAATACC----------------------
----

CCTAATGCCACTGAGACCTTCAGACCTGGAGGAGGAGATATGAGGGACAATTGGAGAAGTGAATTATACAAAT
ATAAAGTAGTAAAAATTGAACCATTA---GGAGTAGCACCCACCAAGGCAAAGAGAAGAGTGGTG--------
-------------------------------CAGAGAGAAAAAAGAGCAGCAATAGGA---ATGGGAGCC---
------TTGTTCCTTGGGTTCTTGTCAGCA---GCAGGAAGCACTATG---GGCGCAGCGTCA---
ATAACGCTGACGGTACAG---GCCAGGCAA---TTATTG---TCTGGAATAGTGCAACAGCAGAAC---
AATTTG---CTGAGG---GCTATTGAGGCGCAACAGCATCTGTTGCAACTCACAGTCTGGGGCATC
>DQ410260
TTCTACTGTGATACAGCACGACTGTTTAATAGTACTTGGAGTGTTAATGTTACTGAAGGAGCAAATAACACTG
CA---

GGAAATGACACAATCACACTCCCATGCAAAATAAAACAAATTATAAACATGTGGCAGGAAGTAGGAAAAGCAA
TGTATGCCCCTCCCATCAGAGGACTAATTAATTGTTCATCAAAT---
ATTACAGGGCTGCTATTAACAAGAGATGGTGGTCCTAACACAAAC----------------------------
----

GAGACTGAGGTCTTTAGACCTGGAGGAGGAGATATGAGGGACAATTGGAGAAGTGAATTATATAAATATAAAG
TAGTAAAAATTGAACCATTA---GGAGTAGCACCCACCAAGGCAAAGAGAAGAGTGGTG--------------
-------------------------CAAAGAGAAAAAAGGGCA---GTAGTGGGAATAGGAGCT---------
TTGTTCCTTGGGTTCTTGGGAGCA---GCAGGAAGCACTATG---GGCGCAGCGTCA---
GTGACGCTGACGGTACAG---GCCAGACAC---TTATTG---TCTGGTATAGTGCAACAGCAAAAC---
AATTTG---CTGAGA---GCTATTGAGGCGCAACAACATCTGTTGCAACTCACAGTCTGGGGCATC
>DQ410292
TTCTACTGTAATTCAACACAACTGTTTAATAGTACTTGGAATAAAACTAGGGATGGTACTTGGAACAGTACTG
GGTCA--
-------------------ACT---
ACAGGAAATGAAACAATCACACTCCCATGCAGAATAAAACAAATTATAAACATGTGGCAGGAAGTAGGAAAAG
CAATGTATGCCCCTCCCATCAGAGGACAAATTAGATGCTCATCAAAT---
ATTACAGGGCTGATATTAACAAGAGATGGTGGTAAAGACAACAATACC-------------------------
----

AATACCATCGAGATCTTCAGACCTGGAGGAGGAGAGATGAGGGACAATTGGAGAAATGAATTATATAAATATA
AAGTAGTAAGAATTGAACCATTA---GGAATAGCACCCAACAGGGCAAAGAGAAGAGTGGTG-----------
----------------------------CAGAGAGAAAAAAGAGCA---ATA------GCGGGAGCT------
---TTGTTCCTTGGGTTCTTGGGAACA---GCAGGAAGCACTATG---GGCGCAGCGTCA---
CTGACGCTGACGGTACAG---GCCAGACAA---TTATTG---TCTGGTATAGTGCAACAGCAGAAC---
AATTTG---CTAAGG---GCTATTGAGGCGCAACAGCATCTGTTGCAACTCACAGTCTGGGGCATC
>DQ410356
TTCTACTGTAATACAACAAAACTGTTTACCTGGAATAGTACTGAAGAGTCAAATAACACTGAG----------
----

GGGAATAGAACAATCACACTCCCATGCAGAATAAAACAAATTATAAACATGTGGCAGGAAGTAGGAAAAGCAA
TGTATGCCCCTCCCATCAAGGGACTAATTAACTGTACATCAAAG---
ATTACAGGACTACTATTAACAAGAGATGGTGGTGGTAATAGTACA----------------------------
----

AACAGGACTGAGATCTTCAGACCTGGAGGAGGAGATATGAGGGACAATTGGAGAAGTGAATTATATAAATATA
AAGTAGTAAGAATTGAACCAGTA---GGAATAGCACCCGGCAAGGCAAAGAGAAGAGTGGTG-----------
----------------------------CAGAGAGAAAAAAGAGCG---GTAACA---CTGGGAGCT------
---ATGTTCCTTGGGTTCTTGGGAGCA---GCAGGAAGCACTATG---GGCGTAGCGTCA---
ATGACGCTGACGGTACAG---GCCAGACAA---TTACTG---TCTGGTATAGTGCAACAGCAGAAC---
AACCTG---CTGAAG---GCGATTGAGGCGCAACAGCACCTGTTGCAACTCACGGTCTGGGGCATT
>JQ403103
TTCTACTGTAATACAACAAAACTGTTTAACAGTACTTGGAGGATTAGTAATGGTACGGAGATTAATGAT----
----

ACTAATAGTACTATCACCCTCCCATGCAGAATAAAACAAATTATAAACAGGTGGCAGGAAGTAGGAAAAGCAA
TGTTTGCCCCTCCTATCAGAGGACAAATTAGTTGTTCATCAAAT---
ATTACAGGGCTGCTATTAACAAGAGATGGTGGGAACGTCAGCAAC----------------------------
----

ACGGCAGAAACCTTTAGGCCTGGAGGAGGAGATATGAGGGACAATTGGAGAAGTGAATTATATAAATATAAAG
TAGTAAGGGTTGAACCATTA---GGAGTAGCACCCACCAAGGCGAAGAGAAGAGTGGTG--------------
-------------------------CAGAGAGAAAAAAGAGCA---GCA------ATAGGAGCT---------
GTGTTCCTTGGGTTCTTGGGAGCA---GCAGGAAGCACTATG---GGCGCAGCGTCA---
GTGGCGCTGACGGTACAG---GCCAGGCAA---TTATTG---TCTGGTATAGTGCAGCAGCAGAAC---
AATCTG---CTGAGA---GCTATTGAGGCGCAACAGCATCTGTTGCAACTCACAGTCTGGGGCATC
>JQ403104
TTCTACTGTAATACATCACAACTGTTTAATAGCACTTTCAGTGGTAATGAGAGTATAAATACTACT-------
----

GAGAACGCTACAATCACACTCCCATGTAGAATAAAACAAATTATAAATATGTGGCAGGAAGTAGGAAAAGCAA
TGTATGCCCCTCCCATCAGTGGACAAATTAACTGTACATCAAAC---
ATTACAGGCCTGTTATTAACAAGAGATGGTGGAGATAACAATACC----------------------------
----

ACCAACACTGAGACCTTCAGACCTACAGGGGGAAATATGAAGGACAATTGGAGAAGTGAATTATATAAATATA
AAGTAGTAAAAATTGAACCATTA---GGACTAGCACCCTCCAAGGCAAAGAGAAGAGTGGTG-----------
----------------------------CAGAGAGAAAAAAGAGCA---GTGGGA---CTAGGAGCT------
---TTGTTCATTGGGTTCTTGGGAGCA---GCAGGAAGCACTATG---GGCGCAGCGTCG---
ATGACGCTGACGGTACAG---GCCAGACAA---TTATTG---TCTGGTATAGTGCAACAGCAGAAC---
AATCTG---CTGAGA---GCTATTGAGGCGCAACAACATCTGTTGCAACTCACAGTCTGGGGCATC
>HQ217329
TTCTACTGTAATTCAACACAACTGTTTGATAGTACTTGGAATGAGACTACAACAAGGTCAAAT----------
----

AGAAGTGGTGACATCACACTCCCATGCAGAATAAAACAATTTATAAACATGTGGCAGGGAGTAGGAAAAGCAA
TATATGCCCCTCCCATCAGTGGACAAATTAACTGCTCATCAAAT---
ATTACTGGGCTACTATTAACAAGAGATGGTGGTAACGGAAACAAT----------------------------
----

ACCACCGAGATCTTCAGACCTGGAGGAGGAGATATGAGGGACAATTGGAGAAGTGAATTATATAAATATAAGG
TAGTAAAAATTGAGCCATTA---GGAATAGCACCCACCAAGGCAAAAAGAAGAGTGGTG--------------
-------------------------CAGAGAGAAAAAAGAGCA---GTGGGA---CTAGGAGTT---------
TTGTTCCTTGGGTTCTTAGGAGCA---GCAGGAAGCACTATG---GGCGCAGCGTCA---
GTGGCGCTGACGGTACAG---GCCAGACAA---TTATTG---TCTGGTATGGTGCAGCAGCAGAAC---
AATCTG---CTGAGA---GCTATTGAGGCGCAACAGCATATGTTGCAACTCACAGTCTGGGGCATC
>FJ469745
TTCTACTGTAGTACAACACAACTGTTTAGTAGTACTTGGAATAGTACTAACAGGACCGATACA----------
----

GGAAATGAAACTATCACACTCCCATGCAAAATAAAACAAATTATAAACTTGTGGCAAGAAGTAGGAAAAGCAA
TGTATGCCCCTCCCATCAGAGGACAAATTAAATGTTCATCAAAT---
ATTACAGGGCTACTATTAACAAGAGATGGTGGTAATGACAATAAT----------------------------
----

ACCACCGAGGAAATTTTCAGACCTGCAGGAGGAGACATGAGGGACAATTGGAGAAGTGAGCTATATAAATATA
AAGTAGTAAAAATTGAACCATTA---GGAGTAGCACCCACCAAGGCAAAGAGAAGAGTGGTG-----------
----------------------------CAGAGAGAAAAAAGAGCA---ATAGGA---ATAGGAGCT------
---ATGTTCCTTGGGTTCTTGGGAGCA---GCAGGAAGCACTATG---GGCGCAGCATCA---
GTGACGCTGACGGTACAG---GCCAGACAA---TTATTG---TCTGGTATAGTGCAACAGCAGAGC---
AATCTG---CTGAGG---GCTATTGAGGCGCAACAGCATCTGTTGCAACTCACAGTCTGGGGCATC
>MK147530
TTCTACTGTAATTCAACACAACTGTTTAATAGTACTTGGACATTTAATAATACTTGGAAGCCTAATATTACTG
GGAATGATACT--

GGAGGGGACAATATCATACTCCAATGCAGAATAAAACAAATTATAAACATGTGGCAGGAAGTAGGAAAAGCAA
TGTATGCCCCTCCCATCAGGGGAGAAATTAGATGTACATCAAAT---
ATTACAGGGCTGCTATTAACAAGAGATGGTGGTAAGGATAATACCACT-------------------------
----

AACGGGACTGAGACCTTCAGACCTGGAGGAGGAGATATGAGAGACAATTGGAGAAGTGAATTATATAAATATA
AAGTAGTAAGAATTGAACCATTA---GGAGTAGCACCCACCAAGGCAAAGAGAAGAGTGGTG-----------
----------------------------CAGAGAGAAAAAAGAGCA---GTGGGA---ATAGGAGCT------
---ATGTTCTTTGGGCTCTTGGGAGCA---GCAGGAAGCACTATG---GGCGCAGCGTCA---
GTGACGCTGACGGTACAA---GCCAGACTA---TTATTG---TCTGGTATAGTGCAACAGCAAAAC---
AATTTG---CTGAGG---GCTATTGAGGCGCAACAGCATTTGTTGCAACTCACAGTCTGGGGCATC
>FJ469772
TTCTACTGTAATACAACACAACTGTTTAATAGTACTTGGCTTAACAGTAATAGTACTGAAAATGGGACT----
----

ACTAACGGCACTATCACACTCCCCTGCAGAATAAAACAAATTATAAACATGTGGCAAGGAATAGGAAGAGCAA
TGTATGCCCCTCCCATCAGTGGACAAATTAGGTGCTTATCAAAT---
ATTACAGGGCTACTATTAACAAGAGATGGTGGTGGTGGGAACGAGACC-------------------------
----

GAGACCAATACCGAGATTTTCAGACCTGCAGGAGGAGATATGAAGGACAATTGGAGAAGTGAATTATATAAAT
ATAAAGTAGTAAGAATTGAACCCTTA---GGAATAGCACCCACCAAGGCAAGGAGAAGAGTGGTG--------
-------------------------------CAGAGGGAAAAAAGAGCA---GTGGGA---ATAGGAGCT---
------ATGTTCCTTGGGATCTTGGGAGCA---GCAGGAAGCACTATG---GGCGCAGTGTCA---
ATGACGCTGACGGTACAG---GCCAGACTA---TTATTG---TCTGGTATAGTGCAACAGCAGAAC---
AATCTG---CTGAGG---GCTATTGAGGCGCAACAGCATCTGTTGAAACTCACAGTCTGGGGCATC
>EF593269
TTCTACTGTAATACAACACAACTGTTTAATAGTACTTGGGGGACTAATAGTACTTGGGAGATTAATACTACT-
----

GGGAATGATACTATCACACTCCCATGCAGAATAAAACAAATTATAAACATGTGGCAGGAAGTAGGAAAAGCAA
TATATGCCCCTCCCATCAGAGGACAAATTAGATGTTCGTCAAAT---
ATTACAGGGCTGCTATTAACAAGAGATGGTGGTGAAAATAGCACA----------------------------
----

AACGGCACCGAGATCTTCAGACCTGGAGGAGGGGATATGAGGGACAATTGGAGAAGTGAATTATATAAATATA
AAGTAGTACAAGTTGAACCATTA---GGAGTAGCACCCACCAAGGCAAAGAGAAGAGTGGTG-----------
----------------------------CAGAGAGAAAAAAGAGCA---GTGGGT---ATAGGAGCT------
---CTATTCCTTGGGTTCTTAGGAGCA---GCAGGAAGCACTATG---GGCGCAGCGTCA---
ATAACGCTGACGGCACAG---GCCAGACAA---TTATTG---TCTGGTATAGTGCAGCAGCAGAGC---
AATTTG---CTGAGG---GCTATTGAGGCGCAACAGCATCTGTTGCAACTCACAGTCTGGGGCATC
>EF593271
TTCTACTGTGATACAAAAATACTGTTTAATAGTACTTGGAATTTTACTAACGGGAAAATTACTGCAGGGCCAA
ATAGC--
-------------------ACT---
GAAGGAAATGACACAATCACAATCCCATGCAGAATAAAACAAATTATAAATATGTGGCAAGAAGTAGGAAAAG
CAATGTATGCTCCTCCCATCAGAGGAATTATTAGCTGTTTATCAAAT---
ATTACAGGGCTGCTATTAACAAGAGATGGTGGTAGGAGTAATGATAGCGCG----------------------
----

ACCAATAAAACTGAAGTCTTCAGACCTGGAGGAGGAGATATGAGGGACAATTGGAGAAGTGAATTATATAAAT
ATAAGGTAGTAAAAATTGAACCATTA---GGGGTAGCACCCACCAAGGCAAAGAGAAGAGTGGTG--------
-------------------------------CAGAGAGAAAAAAGAGCA---GTGGGA---TTTGGAGCT---
------TTGTTCCTTGGGTTCTTGGGAGCA---GCAGGAAGCACTATG---GGCGCAGCGTCG---
GTGACGCTGACGGTACAG---GCCAGACAA---CTATTG---TCTGGTATAGTGCAACAGCAAAAC---
AACTTG---CTGAGA---GCTATTGAGGCGCAACAACATCTGTTGCAACTCACAGTCTGGGGCATC
>EF593273
TTCTATTGTGATTCAACAAAATTGTTTAATAGTACTTGGGGAATTAATGGTACTTGGAATACCACT-------
----

GAAGGGGCAAATATCACACTCCCATGCAGAATAAAACAGATTATAAATCTGTGGCAGCAAGTAGGAAAAGCAA
TGTATGCCCCTCCCATCAAAGGAAAAATTAGCTGCTCATCAAAT---
ATTACAGGGCTGATATTGACAAGAGATGGTGGTGATAATGGAAAT----------------------------
----

GGGACAGAGGAGATCTTCAGACCTGGAGGAGGAGATATGAGGGACAATTGGAGAAGTGAATTATATAAATATA
AGGTAGTAAAAATTGAACCATTA---GGAATAGCACCCACCAAGGCAAAGAGAAGAGTGGTG-----------
----------------------------CAGAGAGAGAAAAGAGCA---GTGGGA---ATGGGAGCT------
---CTGTTCCTTGGGTTCTTGGGAGCA---GCAGGAAGCACTATG---GGCGCAGCGTCA---
GTGACGCTGACGGTACAA---GCCAGACGA---TTATTG---TCTGGTATAGTGCAACAGCAGAAC---
AACTTG---CTGAGG---GCTATTGAGGCGCAACAGCATCTGTTGCAACTCACAGTCTGGGGCATT
>EF593274
TTCTACTGTAATACAACACAACTGTTTAATAGTACTTGGTTTCCTAATGGTACTTGGTTTCCTAATGATACTG
TGAAT--
-------------------AAT---
ACTGGAGATGTGAATATCACCCTACCATGCAGAATAAAACAAATTGTAAACATGTGGCAGGAAGTAGGAAAAG
CAATGTATGCCCCTCCCATTGCAGGACAAATTAGATGTTCATCAAAT---
ATTACAGGGTTACTATTAACAAGAGATGGTGGTATTGGTAATAGCACA-------------------------
----

AATAATGAGACCGAGACCTTCAGACCTGGAGGAGGAGATATGAGGGATAATTGGAGAAGTGAATTATATAATT
CCAAAGTAGTAAAAATTGAACCATTA---GGAATAGCACCCACCAAGGCCAAGAGAAGAGTGGTG--------
-------------------------------CAGAGAGAAAAAAGAGCA---GTGGGA---ATAGGAGCT---
------ATGTTCCTTGGATTCTTGGGAGCA---GCAGGAAGCACTATG---GGCGCAGCGTCA---
CTGACGCTGACGGTACAG---GCCAGACAA---TTATTG---TCTGGTATAGTGCAACAGCAAAAC---
AACTTG---CTGAGA---GCTATTGAGGCGCAACAACATCTGTTGCAACTCACAGTCTGGGGCATC
>EF593276
TTCTACTGTAATACAACGAAGTTGTTTGATAGTGCTTGGCCTAATGCTACTTGGAAGAATGCC----------
----

AATGATACAATTATCACACTCCCATGCAGAATAAAACAAATTATAAACATGTGGCAAGAAGTAGGAAAAGCCA
TGTATGCCCCTCCCATCAGTGGACAAATTAACTGTTCATCAAAT---
ATTACAGGGCTGCTATTAACAAGAGATGGTGGTACCGGGAACAATGAGACA----------------------
----

```
-------
GACAATAATACTGAAATTTTCAGACCTGGGGGAGGAGACATGAGGGACAATTGGAGAAGTGAATTATATAAAT
ATAAAGTAGTACAAATTGAACCATTA---GGAATAGCACCCACCAAGGCAAGGAGGAGAACGGTG--------
-------------------------------CAGAGAGAAAGAAGAGCA---GCA------ATAGGAGCT---
------ATGATCCTTGGGTTCTTGGGAGTA---GCCGGAAGCACTATG---GGCGCAGCGTCA---
GTGACGCTGACGGTACAG---GCCAGACTA---TTATTG---TCTGGTATAGTGCAACAGCAGAAC---
AATCTG---CTGAGG---GCTATTGAGGCGCAACAGCATCTGTTGCAACTCACCGTCTGGGGCATT
>JN024210
TTCTACTGTAATACAACACAACTGTTTAATAGTACTTGGAATGTGACTGGGTCAAATAACACTGAA-------
-------------------------------------------------------------------------
----------------------------
GGAAATGACACAATCACGCTCCCATGCAGAATAAAACAAATTATAAACATGTGGCAGGAAGTAGGAAAAGCAA
TGTATGCCCCTCCCACCAGAGGACAAATTAGATGTTCATCAAAT---
ATTACAGGGCTGCTATTAACAAGAGATGGTGGTGTGAACACCACCACC-------------------------
-------------------------------------------------------------------------
-------
ACCAACGAGACAGAGGTCTTCAGACCTGGAGGAGGAAATATGAAGGACAATTGGAGAAGTGAATTATATAAAT
ATAAAGTAGTAAAAATTGAACCATTA---GGAGTAGCACCCACCAAGGCAAAGAGAAGAGTGGTG--------
-------------------------------CAGAGAGAAAAAAGAGCA---ATAGGA---ATAGGAGCT---
------GTGTTCCTTGGGTTCTTGGGAGCA---GCAGGAAGCACTATG---GGCGCAGCGTCA---
ATAACGCTGACGGTACAG---GCCAGACAA---TTATTG---TCTGGTATAGTGCAACAGCAGAAC---
AACTTG---CTGAGA---GCTATTGAGGCGCAACAACATATGTTGCAACTCACAGTCTGGGGCATC
>FJ469683
TTCTACTGTAATACAACACAACTGTTTAATAGTACTTGGTTTAATGGTACTTGGAATGGTACTATAGAGTCAA
GT-----------------------------------------------------------------------
----------------------------
AACACTACTAATATCACACTCCCATGCAGAATAAAACAAATTGTAAACATGTGGCAGGAAGTAGGAAAAGCAA
TGTATGCCCCTCCCATCAGAGGACAAATTAGATGTTCATCAAAT---
ATTACAGGGCTGCTATTGACAAGAGATGGTGGTAAGAACAACAGC----------------------------
-------------------------------------------------------------------------
-------------
ACCAATGAGACCTTCAGACCTGAAGGAGGAAATATGAGGGACAATTGGAGAAGTGAATTATATAAATATAAAG
TAGTAAGAATTGAACCATTA---GGAGTAGCACCCACCAAGGCAAAGAGAAGAGTGGTG--------------
-------------------------CAAAGAGAAAAAAGAGCA---GTGGGAACGATAGGAGCT---------
ATGTTCCTTGGGTTCTTGGGAGCA---GCAGGAAGCACTATG---GGCGCAGCGTCA---
ATGACGCTGACGGTACAA---GCCAGACAA---TTATTG---TCTGGTATAGTGCAACAGCAAAAC---
AATTTG---CTGAGG---GCTATTGAGGCGCAACAACATCTGTTGCAACTCACAGTCTGGGGCATT
>HQ238279
TTCTACTGTAATACAACACAACTGTTTAATAGTACTTGGAATAATAATAGTACTGAGAATAAGAATGATACTA
AC-----------------------------------------------------------------------
----------------------------
AGCAATGAAACTATCATGCTCCCATGCAGAATAAAACAAATTATAAACAGATGGCAAGAAGTAGGAAAAGCAA
TGTATGCCCCTCCCATCGCTGGACAAATTAGATGTTCATCAAAT---
ATTACAGGGCTGCTATTGACAAGAGATGGTGGTAATAGCAGCAGCGAC-------------------------
-------------------------------------------------------------------------
-------
AATACAAGTACAGAGATCTTCAGACCTGGAGGAGGAGATATGAGAGACAATTGGAGAAGTGAATTATATAAAT
ATAAAGTAGTAAAAATTGAACCATTA---GGAGTAGCACCCACTAAGGCAAAGAGAAGAGTGGTG--------
-------------------------------CAGAGAGAAAAAAGAGCA---GTAGTGGGAATAGGAGCT---
------GTGTTCCTTGGGTTCTTGGGAGCA---GCAGGAAGCACTATG---GGCGCAGCATCA---
TTAACGCTGACGGTACAG---GCCAGACAA---TTATTG---TCTGGTATAGTGCAGCAGCAGAAC---
AATCTG---CTGAGG---GCTATTGAGGCGCAACAGCATCTGTTGCAACTCACAGTCTGGGGCATC
>FJ496085
TTCTACTGTAATTTAACACAACTGTTTAATAGTACTTGGAATAGTACTGAAAGCTTTAATAGTACTGAAAGCT
CTAATAGT-----------------------------------------------------------------
-------------------ACT---
GAAGGAAATGACACAATCAGCCTCCCATGCAGAATAAAACAAATTATAAACATGTGGCAGGAAATAGGAAAAG
CAATGTATGCCCCTCCCATTGCAGGAATAATTAGATGTTCATCAAAT---
ATTACAGGAATACTATTAACAAGAGATGGTGGTATAAATAGTACAGAT-------------------------
-------------------------------------------------------------------------
```

AATACCACTGAGATCTTCAGACCTGCAGGAGGAGATATGAGAGACAATTGGAGAAGTGAATTATATAAATACA
AAGTAGTAAAAATTGAACCATTA---GGAATAGCACCCACCAAGGCAAAGAGAAGAGTGGTG-----------
----------------------------CAGAGAGAAAAAAGAGCA---GTGGGA---ATAGGAGCT------
---GTGTTCCTTGGGTTCTTGGGCGCA---GCAGGAAGCACTATG---GGCGCAGCGTCA---
GTGGCGCTGACGGTACAG---GCCAGACAA---TTATTG---TCTGGTATAGTGCAACAGCAGAAC---
AATTTG---CTGAGG---GCTATTGAGGCGCAACAGCATCTGTTGCAACTCACAGTCTGGGGCATC
>JF689854
TTCTACTGTAATACAACACAACTGTTTAATAGTACTTGGAATGATACTACTGAAGGGTCAAATAAC-------
----

ACTGGAAGAACTATCACACTCCCATGCAGAATAAAACAAATTATAAATATGTGGCAGGAAGTAGGAAAAGCAA
TGTATGCCCCTCCCATCAGCGGACAAATTAGATGTTCATCAAAT---
ATTACAGGGCTTCTATTAACAAGGGATGGTGGTAATAATAACGAGACC-------------------------
----

AATAACGGGACCGAGACCTTCAGACCTGGAGGGGGAAATATAAGGGACAATTGGAGAAGTGAATTATATAAAT
ATAAAGTAGTAAAAATTGAACCATTA---GGAGTAGCACCCACCAGGGCAAAGAGAAGAGTGGTG--------
-------------------------------CAGAGAGAAAAAAGAGCA---GTGGGA---ATAGGAGCT---
------GTGTTCCTTGGGTTCTTGGGAGCA---GCAGGAAGCACTATG---GGCGCAGCGTCA---
ATAACGCTGACGGTACAG---GCCAGACAA---TTATTG---TCTGGTATAGTGCAACAGCAAAGC---
AATTTG---CTGAGG---GCTATTGAGGCGCAACAGCATATGTTGCAGCTCACAGTCTGGGGCATC
>JF689856
TTCTACTGTAATACAACAAGACTGTTTAATAGTACGTGGTTTTGGAATAATGCTACTGCTAATTGGACTTTGA
AAAATGATACT--
-AAGGGAGAATTCAAT---------
ATCTCTGGAGGGAATATCACACTCCCATGCAGAATAAAACAAATTATAAACATGTGGCAGGAAGTAGGGAAAG
CAATGTATGCCCCTCCCATCAGAGGACAAATTAGATGTTCATCAAAT---
ATTACAGGGCTGCTATTAACAAGAGATGGTGGAGAGGAGCCTAAC----------------------------
----

GATACTGAGACTTTCAGACCTGCAGGAGGAAATATGAAGGACAATTGGAGAAGTGAATTATATAAATATAAAG
TAGTAAAAATTGAACCATTA---GGAATAGCACCCACCAGGGCAAAGAGAAGAGTGGTG--------------
-------------------------CAGAGAGAAAAAAGAGCA---GTGGGA---ATAGGAGCT---------
TTGTTCCTTGGGTTCTTGGGAGCA---GCAGGAAGCACTATG---GGCGCAGCGTCA---
GTAACGCTGACGGTACAG---GCCAGAATG---CTATTG---TCTGGTATAGTGCAACAGCAGAAC---
AATCTG---CTGAGG---GCTATTGAGGCGCAACAGCATCTGTTGCAACTCACAGTCTGGGGCATC
>JF689857
TTCTACTGTAACACATCACAACTGTTTAATAATACTTGGAATGGAACTGCATGGTCAACTAACAGTTCA----
----

AATAGCACTGATATCACACTCCCATGCAGAATAAAACAAATTATAAACATGTGGCAGGAAGTAGGAAAAGCAA
TGTATGCCCCTCCCATTAGAGGACTAATTAGATGTTCATCAAAT---
ATTACAGGGCTACTATTAACAAGAGATGGTGGTAATAATAATAAT----------------------------
----

ACTAATGAGACCTTCAGGCCTGGAGGAGGAGATATGAGGGACAATTGGAGAAGTGAATTATATAAATATAAAG
TAGTAAAAATTGAACCATTA---GGAATAGCACCCACTAGGGCAAAGAGAAGAGTGGTG--------------
-------------------------CAGAGAGAAAAAAGAGCA---GTAGGA---ATAGGAGCT---------
ATGTTCCTTGGGTTCTTGGGAGCA---GCAGGAAGCACTATG---GGCGCAGCGTCA---
ATGACGCTGACGGTACAA---GCCAGACAA---TTATTG---TCTGGTATAGTGCAACAGCAGAAC---
AATCTG---CTGAGG---GCTATTGAGGCGCAACAGCATCTGTTGCAACTCACAGTCTGGGGCATC
>JF689859
TTCTACTGTGATTCAACACAACTATTTAATAGTACTTGGAATGGTACTGAAGGGTCAAATAACACTGAA----
----

GGAAATAGCACACTCACACTCCCATGCAGAATAAAACAGATTATAAACATGTGGCAGGAAGTAGGAAAAGCAA
TGTATGCCCCTCCCATCAGAGGACAAATTAGATGTTCATCAAAT---
ATTACAGGGCTGCTATTAACAAGAGATGGTGGTACTAACAATAGC----------------------------
----

GAGAACACCGAGATCTTCAGACCTGGAGGAGGAGATATGAGGGATAATTGGAGAAGTGAATTATATAAATATA
AAGTAGTAAAAATTGAACCATTA---GGAATAGCACCCACCAAGGCAAAGAGAAGAGTGGTG-----------
----------------------------CAGAGAGAAAAAAGAGCA---GTGGGA---ATAGGAGCT------
---GTGTTCCTTGGGTTCTTGGGAGCA---GCAGGAAGCACTATG---GGCGCAGCGTCA---
CTGACGCTGACGGTACAG---GCCAGACTA---TTATTG---TCTGGTATAGTGCAACAGCAGAAC---
AATTTG---CTGAGG---GCTATTGAGGCGCAACAGCATCTGTTGCAACTCACAGTCTGGGGCATC
>JF689860
TTCTACTGTAATACATCAGAGCTATTTAATTGGACTGAGGGAAAACCTGCTAAC-------------------
----

ACTGAAGACAAAATTACAATCCAATGCAGAATAAAACAAATTATAAACATGTGGCAGGAAGTAGGAAAAGCAA
TGTATGCCCCTCCCATCAGTGGACAAATTAGATGTTCCTCAACT---
ATTACAGGGTTGATATTAACAAGAGATGGTGGTAATGACACAAGG----------------------------
----

GAGAACGAGACCTTCAGACCTGGAGGAGGAGATATGAGGGACAATTGGAGAAGTGAATTATATAAATATAAAG
TAGTAAAAATTGAACCATTA---GGAATAGCACCCACCAAGGCAAAGAGAAGAGTGGTG--------------
-------------------------CAGAGAGAAAAAAGAGCA---GTGGGA---ATAGGAGCT---------
TTATTCCTTGGGTTCTTGGGAGCA---GCAGGAAGCACTATG---GGCGCAGCGTCA---
CTGACGCTGACGGTACAG---GCCAGACAA---TTATTG---TCTGGTATAGTGCAACAGCAGAGC---
AATTTG---CTGAGG---GCTATTGAGGTGCAACAGCATCTGTTGCAACTCACAGTCTGGGGCATC
>MK383403
TTCTATTGTAATACAACACAACTGTTTAATAGTACTTGGAATTTAACACAACCGTTTAATAGTACTTGGAATG
GTACTGAA---

GGGTCAAGTAATATAATCACACTCCCATGCAGAATAAAACAAGTTATAAACATGTGGCAGGAAGTAGGAAAAG
CAATGTATGCCCCTCCCATCAGAGGATACATTAGATGTTCATCAAAT---
ATTACAGGGCTACTATTAACAAGAGATGGTGGTAATGACAAAAACGGGACA----------------------
----

ACAACCGAGATCTTCAGACCTGGAGGAGGAGATATGAGGGATAATTGGAGAAGTGAATTATATAAATATAAAG
TAGTAAAAATTGAACCATTA---GGAGTAGCACCCACCAAGGCAAAGAGAAGAGTGGTG--------------
-------------------------CAGAGAGAAAAAAGAGCA---GTGGGA---ATAGGAGCC---------
TTGTTCCTTGGGTTCTTGGGAGCA---GCAGGAAGCACTATG---GGCGCAGCGTCA---
ATGACGCTGACGGTACAG---GCCAGACAA---TTATTG---TCTGGTATAGTGCAACAGCAGAAC---
AACTTG---CTGAGA---GCTATTGAGGCGCAACAACATCTGTTGCGACTCACAGTCTGGGGCATC
>HQ216575
TTCTACTGTAATACAACAAAACTGTTTAATAGTACTTGGAGTGATAATAGTACTTGGAATAATAGTAGTAAAT
GGTTC--
-------------------AAT---
GGCACTGGAAAAAATATCACACTCCTATGCAGAATAAAACAAATTGTAAATATGTGGCAGGAAGTAGGAAGAG
CAATGTACGCCCCTCCCATCAGTGGAATAATTGACTGTACATCAAAT---
ATTACAGGGATAATACTAGTAAGAGATGGTGGTAATATAAATGGGAGT-------------------------
----

AGGGAGAAAGAGACATTCAGACCTATAGGTGGAAATATGAAGGACAATTGGAGAAGTGAACTATATAAATATA
AAGTAGTAAAACTTGAACCATTA---GGAATAGCACCCACCAAGGCAAAGAGAAGAGTGGTG-----------
----------------------------CAGAGAGAAAAAAGAGCA---GTGGGA---CTAGGAGCT------
---CTGTTCTTTGGGTTCTTGGGAGCA---GCAGGAAGCACTATG---GGCGCAGCGTCA---
TTGACGCTGACGGTACAG---GCCAGACTA---TTATTG---TCTGGTATAGTGCAACAGCAAAAC---
AATTTG---CTAAAA---GCTATTGAGGCGCAACAGCATCTGTTGAAGCTCACAGTCTGGGGCATT
>HQ216584
TTCTACTGTAATACAACAAAACTGTTTAATAGTACTTGGGTTAATGGAACTAAGAATGACACAAAGGGG----
----

GGTAATGGAACTATCACACTCCAATGCAGAATAAAACAAATTATAAACATGTGGCAGCAAGTAGGAAAAGCAA
TGTATGCCCCTCCCATCAGCGGACCAATTAGTTGTTCATCAAAT---
ATTACAGGGCTGATATTAACAAGAGATGGTGGTACAAACACCACC----------------------------
----

AATGAGACCTTCAGACCTGGAGGAGGAGATATGAGGGACAATTGGAGAAGTGAATTATATAAATATAAAGTAG
TAAAAATTGAACCAATA---GGAGTAGCACCCACCAAGGCAAGGAGAAGAGTGGTG-----------------
----------------------CAGAGAGAAAAAAGGGCA---GTGGGA---ATAGGAGCT---------
TTTTTCCTTGGGTTCTTGGGAGCA---GCAGGAAGCACTATG---GGCGCAGCATCA---
ATAACGCTGACGGTACAA---GCCAGACTA---TTATTG---TCTGGTATAGTGCAACAGCAAAGC---
AACTTG---CTGAGA---GCTATTGAGGCGCAACAGCACCTGTTGCAACTCACAGTCTGGGGCATC
>HQ216686
TTCTACTGTGATTCAACACAGCTGTTTAATAGTACTTGGCCGTCTAATAGTACTTGGTCGTTTAATGATACTA
CAAAT--
-------------------AAT---
ACTGTAGGCACAAATATCACACTCCCATGCAGAATAAAACAAATTATAAACATGTGGCAGGAAGTAGGAAAAG
CAATGTATGCCCCTCCCATCAGAGGACAAATTAGATGTTCATCAAAT---
ATTACAGGCCTGCTATTAACAAGGGATGGTGGGGTTGGTAATAATAGCAGC----------------------
----

AATACAACGAACCCCGAGATCTTCAGACCTGGAGGAGGAGATATGAGGGACAATTGGAGAAGTGAATTATATA
AATATAAAGTAGTAAAAATTGAACCATTA---GGAATAGCGCCCACTAAGGCAAAGAGAAGAGTGGTG-----
----------------------------------CAAAGAGAAAAAAGAGCA---GTGGGG---
CTAGGAGCT---------ATGTTCCTTGGGTTCTTGGGAGCA---GCAGGAAGCACTATG---
GGCGCAGCGTCA---ATAACGCTGACGGTACAG---GCCAGACAA---TTATTG---
TCTGGTATAGTGCAACAGCAGAAC---AATTTG---CTGAAG---
GCTATTGAGGCGCAACAGCATCTGTTGCAACTCACAGTCTGGGGCATC
>HQ216727
TTCTACTGTAATTCAACAAAGCTGTTTAATAGTACTTGGCATTGGAATGGTACGATGTTTAATAGTACTGGGA
TTGAA--
-------------------AGG---
TCAAATGAGACTAATATCACACTCCCATGCAGAATAAAACAAATTGTAAACATGTGGCAGGAAGTAGGAAAAG
CAATGTATGCCCCTCCCATCAGAGGACAAATTAGATGTACATCAAAT---
ATTACAGGGCTGCTGTTATTTAGAGATGGTGGTAATACCACGAAGAAC-------------------------
----

AATACCGACGAGACTTTCAGACCTGGAGGAGGAGAGATGAGGGACAATTGGAGAAGTGAATTATATAAATATA
AAGTAGTAAAAATTGAACCATTA---GGAGTAGCACCCACTAAGGCAAAGAGAAGAGTGGTG-----------
----------------------------CAGAGAGAAAAAAGAGCA---GTGGGA---ATAGGAGCT------
---GTGTTCCTTGGGTTCTTGGGAGCA---GCAGGAAGCACTATG---GGCGCAGCGTCA---
ATGACGCTGACGGTACAG---GCCAGACTA---TTATTG---TCTGGTATAGTGCAACAGCAGAAC---
AATTTG---CTGAGG---GCTATTGAGGCGCAACAGCATCTGTTGCAACTCACAGTCTGGAGCATT
>HQ216762
TTCTACTGTAATACAACACAACTGTTTAATAGTACTTGGCCTACTGATAAAAATATTACTGGGAAGGATAGT-
----

GGCAATAGAAACATCACCCTCCCATGCAGAATAAAACAATTTATAAACATGTGGCAGGAAGTAGGAAAAGCAA
TGTATGCCCCTCCCATTAAAGGACAAATTAACTGCTTATCAAAT---
ATTACAGGGTTGCTATTAACAAGAGATGGTGGTAGTAACAACAAC----------------------------
----

AACAGCGAGATCTTCAGACCTGGAGGAGGAGATATGAGGGACAATTGGAGAAGTGAATTATATAAATATAAAG
TAATAGAAATTGAACCATTA---GGAATAGCACCCACCAAGGCAAAAAGAAGAGTGGTG--------------
-------------------------CAAAGAGAAAAAAGAGCA---GTGGGAGTAGTAGGAGCT---------
ATGATCCTTGGGTTCTTGGGAACA---GCAGGAAGCACTATG---GGCGCAGCGTCA---
CTGACGCTGACGGTACAG---GCCAAACAA---TTATTG---TCTGGTATAGTGCAGCAGCAGAAC---
AATCTG---CTGAGA---GCTATTGAGGCGCAACAGCACCTGTTGCAACTCACCGTCTGGGGCATC
>HQ216816
TTCTACTGTAATACAACACAGCTGTTTAATAGTACTTGGAATCTTAATGGTACTTGGAGTGGTACTACA----
----

GGAGAGGGGAACATTACACTCCCATGCAGAATAAAACAAATTGTAAATAGGTGGCAGGAAGTAGGAAAAGCAA
TGTATGCCCCTCCCATCAGCGGAAGAATTAGCTGTTCATCAAAT---
ATTACAGGGTTATTATTAACAATAGATGGTGGTAATAACAGGACA----------------------------

----

AACGAGACCTTCAGACCTGGAGGAGGAAATATGAGGGATAATTGGAGAAGTGAATTATATAAATATAAAGTAG
TACAAATTGAGCCATTA---GGAGTAGCACCCACCAAGGCAAAGAGAAGAGTGGTG-----------------
----------------------CAGAGAGAAAAAAGAGCA---GTGGGAATACTAGGAGCT---------
ATGTTCCTTGGGTTCTTGGGAGCA---GCAGGAAGCACTATG---GGCGCAGCATCG---
ATGACGCTGACGGTACAG---GCCAGACAA---CTATTG---TCTGGTATAGTGCAGCAGCAAAAC---
AATTTG---CTGAGG---GCTATTGAGGCGCAACAACATATGTTGCAACTCACAGTCTGGGGCATC
>HQ216841
TTCTACTGTAATACAACGAATCTGTTTAATAGTACTTGGAATAGTACTAGTACTGTAAATAAGACTACAGGG-
----

CCAAATGGGACTATCACACTCCCATGTAGGATAAAACAAATTATAAACATGTGGCAGGAAGTAGGAAAAGCAA
TGTATGCCCCTCCCATCCAAGGAAAAATTAGATGTACATCAAAT---
ATTACAGGGCTGTTATTAGTAAGAGATGGTGGCAACAACAATGAT----------------------------
----

ACTAATACAGAGATATTCAGACCTGGAGGAGGAAATATGAGGGACAATTGGAGAAGTGAATTATATAAATATA
AAGTAGTAAAAGTTGAACCATTA---GGAATAGCGCCCACCGAGGCAAAAAGAAGAGTGGTG-----------
----------------------------CAGAGAGAGAAAAGAGCA---GTGGGA---CTAGGAGCT------
---TTGTTCCTTGGGTTCTTGGGAGCA---GCAGGAAGCACTATG---GGCGCAGCGTCA---
ATGACGCTGACGGTACAG---GCCAGACAA---TTACTG---TCTGGTATAGTGCAACAGCAAAAC---
AACTTG---CTGAAG---GCTATTGAGGCGCAACAGCATATGTTGCAACTCACAGTCTGGGGCATC
>JX863966
TTCTACTGTAATCTAACACAACTGTTTAATAGTACTTGGAATGTAAGTGAAGGGTTAAATATCACAGAAAGTG
TC---

AATGACACAGATATCATACTCCCATGCAGGCTAAAACAAATTATAAACATGTGGCAGAAAGTAGGAAGAGCAA
TGTATGCCCCTCCCATCCCAGGACTAATTAACTGTACCTCAAAT---
ATTACAGGGTTGTTAATAACAAGAGATGGTGGTAACGAGAGCAAG----------------------------
----

AACGGGACTGAGATCTTCAGACCTTGGGGAGGAGATATGAGGGACAATTGGAGAAGTGAATTATATAAATATA
AAGTAGTAAAAATTGAACCATTA---GGAGTAGCACCCACCAAGGCAAAGAGAAGAGTGGTG-----------
----------------------------CAGAGAGAAAAAAGAGCA---TTAGGAGGATTAGGAGCT------
---ATGTTCCTTGGGTTCTTGGGAGCA---GCAGGAAGCACTATG---GGCGCAGCGTCA---
GTGACGCTGACGGTACAG---GCCAGACTA---TTATTG---TCTGGTATAGTGCAGCAGCAAAAC---
AATTTG---CTGAAG---GCTATTGAGGCGCAACAACATCTGTTGCAACTCACAGTCTGGGGCATC
>JX863971
TTCTACTGTAACACAACGCCACTGTTTACTAATTCTTGGAATGGTACCCATTGGGCTGATGAGTGGAATTGGG
AGAATAGT---

ACTAATGGAACAATCACACTCCAATGCAGAATAAAACAAATTATAAATATGTGGCAGGAAGTAGGAAAAGCAA
TGTATGCCCCTCCCATCAGGGGACAAATTAACTGTACATCAAAT---
ATTACAGGGCTGCTATTAGTAAGAGATGGTGGTAATAATGATAGC----------------------------
----

AGCACCAACGAGACCTTCAGACCTGGAGGAGGAAATATGAAGGACAATTGGAGAAGTGAATTATATAAATATA
AAGTAGTAAGAATTGAACCATTA---GGAGTAGCACCCACCAAGGCAAAGAGAAGAGTGGTG-----------
----------------------------CAAAGAGAAAAAAGAGCA---GTGGGA---ATAGGAGCT------
---CTGTTCCTTGGGTTCTTGGGAGCA---GCAGGAAGCACTATG---GGCGCAGCGTCA---
GTGACGCTGACGGTACAG---GCCAGACTA---TTATTG---TCTGGTATAGTGCAACAGCAAAAC---
AATTTG---CTGAGG---GCTATTGAGGCGCAACAGCACATGTTGCAGCTCACAGTCTGGGGCATC
>JX863972
TTCTACTGTAATACAACACCACTGTTTAATAGTAGTTGGAGGAATTCAAATAATACTTGGGATAGTACTGAAG
GATCAAAT---
-------------AACACT------
GCAGGAAATGACACAATCATACTCCAATGCAGAATAAAACAAATTATAAACATGTGGCAAGAAGTAGGAAAAG
CAATATATGCCCCTCCCATCAGAGGAAACATTACATGTTTATCAAAT---
ATTACAGGACTGCTATTAACAAGAGATGGAGGCAAAAGAGATAATAGCAGTAAC-------------------

----

AATAATGATACTGAAATCTTCAGACCTGGAGGAGGGGATATGAGGGACAATTGGAGAAGTGAATTATATAAAT
ATAAAGTAGTAAAAATTGAGCCATTA---GGAGTAGCACCCACTAAGGCAAAGAGAAGAGTGGTG--------
-------------------------------CAAAGAGAAAAAAGAGCA---GTGGGA---TTAGGAGCT---
------ATGTTCCTTGGGTTCTTGGGAGCA---GCAGGAAGCACTATG---GGCGCAGCGTCA---
CTGACGCTGACGGTACAG---GCCAGACAA---TTATTG---TCTGGTATAGTGCAACAGCAGAAC---
AATTTG---CTGAGA---GCTATTGAGGCGCAACAGCATCTGTTGCAACTCACAGTCTGGGGCATC
>JX863919
TTCTACTGTAATTCATCACGACTGTTTGATAGTACTTGGTCACTGGAGGACACT-------------------
----

GGAAATGACACAATCATGCTACCATGCAGAATAAGACAAATTGTAAACATGTGGCAGGGAGTAGGAAAAGCAA
TGTATGCCCCTCCCATCAGAGGACAAATTAACTGTACATCAAAT---
ATTACAGGGCTGATATTAACAAGAGATGGTGGTGCTGATATGAAT----------------------------
----

GAGACTTTCAGACCTGGAGGAGGAAATATGAAGGACAATTGGAGAAGTGAATTATATAAATATAAAGTAGTAA
GAATTGAACCAATA---GGATTAGCACCCACCAAGGCACAGAGAAGAGTGGTG--------------------
-------------------CAGAGAGAAAAAAGAGCA---ATAGGA---CTAGGAGCT---------
GTGTTCCTTGGGTTTCTAGGAGCA---GCAGGAAGCACTATG---GGCGCAGCGTCA---
CTGACGCTGACGGTACAG---GCCAGACAA---TTATTG---TCTGGTATAGTGCAACAGCAGAAC---
AATTTG---CTGAGG---GCTATTGAGGCGCAACAACATATGTTGCAACTCACAGTCTGGGGCATC
>JX863983
TTCTACTGTAATACAACACCACTATTTAACAGTAGTTGGCATAGTAATGGGACTAGAAATGATACTGGAGGAT
CAAAC--

AGCACAGACCCAATCACGCTCCAATGCAGAATAAAACAAATTATAAACATGTGGCAGGAAGTAGGAAAAGCAA
TGTATGCCCCTCCCATCAGTGGGCAAATTAACTGTTCATCAAAT---
ATTACAGGGCTGATATTAACAAGAGATGGTGGTACTAACAATAAC----------------------------
----

ACTACCACCGAGACCTTCAGACCTGGAGGAGGAGATATGAGGGACAATTGGAGAAGTGAACTATATAAATATA
AAGTAGTAAAAATTGAACCATTA---GGAATAGCACCCACCAGGGCAAAGAGAAGAGTGGTG-----------
----------------------------CAGAGAGAAAAAAGAGCA---GCA------CTAGGAGCT------
---CTGTTCCTTGGGTTCTTGGGAGCA---GCAGGAAGCACTATG---GGCGCAGCATCA---
ATGACGCTGACGGTACAG---GCCAGACTA---CTATTG---TCTGGTATAGTGCAACAGCAGAAC---
AATTTG---CTGAGA---GCTATTGAGGCGCAACAGCATCTGTTGCAACTCACAGTCTGGGGCATC
>JX863984
TTCTACTGTAATACAACACTACTGTTTAATTGGACTGAAGAGGAAAATAACACAAATAAA-------------
----

TATAATAAAACAATCAAGCTCCCATGCAGAATAAAACAAGTTATAAACATGTGGCAGGAAGTAGGAAAAGCAA
TGTATGCCCCTCCCATCAAAGGAGTAATTAACTGCTTATCAAAT---
ATTACAGGACTGTTATTAGTAAGAGATGGGGGTAATGGCAATAACACC-------------------------
----

AACCGCACCGAGACCTTCAGACCTGGAGGAGGAGATATGAGGGACAATTGGAGAAGTGAGCTATATAAATATA
AAGTAGTAAAAATTGAACCAATA---GGAATAGCACCCACTAAGGCAAAGAGAAGAGTGGTG-----------
----------------------------CAAAGAGAAAAAAGAGCAGTAGTGGGA---ATAGGAGCT------
---ATGTTCCTTGGGTTCTTGGGAGCT---GCAGGAAGCACTATG---GGCGCAGCGTCA---
TTGACGCTGACGGTACAG---GCCAGACTA---TTATTG---TCTGGTATAGTGCAACAGCAGAAC---
AATTTG---CTGAGG---GCTATTGAGGCGCAACAACGTATGTTGCAACTCACAGTCTGGGGCATC
>JX863985
TTCTACTGTAATTCAACACCACTGTTTACAACTGCAAATAACTCT----------------------------
----

GGAGGAGTTAATATCACACTCCAATGCAAAATAAAACAAGTTATAAACAGGTGGCAGGAAGTAGGAAAAGCAA
TGTATGCCCCTCCCATCAGAGGACAAATTAGATGTTCATCAAAT---
ATTACAGGGCTGCTATTAATAAGGATGGTGGTAATAATAGCAAG-----------------------------

----

AGTGATCCCGAGATCTTCAGACCTGGAGGAGGAGATATGAGGGACAATTGGAGAAGTGAATTATATAAATATA
AAGTAGTGAAAATTGAACCATTA---GGAATAGCACCCACCAAGGCAAAGAGAAGAGTGGTG-----------
-----------------------------CAGAGAGAAAAAAGAGCA---GTGGGA---CTAGGAGCT------
---ATGTTCCTTGGGTTCTTGGGAGCA---GCAGGAAGCACTATG---GGCGCAGCATCA---
ATAACGCTGACGGTACAG---GCCAGACAA---TTATTG---TCTGGTATAGTGCAACAGCAGAGC---
AATCTG---CTGAGA---GCTATTGAGGCGCAACAGCATCTGTTGCAACTCACAGTCTGGGGCATC
>JX863986
TTCTACTGTAATTCAACACCACTGTTTAATAGTACTTGGTATGGGAATGGTACTAGTGCTGAGAATGATAGTA
CAAAT--

CCAAGTGAAAATATCACACTCCAATGTAGAATAAAACAAATTATAAACATGTGGCAGGAAGTAGGAAAAGCAA
TGTATGCCCCTCCCATCAGTGGACAAATTAGCTGTACATCAAAT---
ATTACAGGGCTGTTATTAACAAGAGATGGTGGTAAGAACAATAGCACC-------------------------
----

AACAACACCGATGAGATCTTTAGACCTGGAGGAGGAAATATGAAGGACAATTGGAGAAGTGAATTATATAAAT
ATAAAGTAGTACAAATTGAACCAATA---GGAATAGCACCCACCAAGGCAAAAAGAAGAGTGGTG--------
--------------------------------CAAAGAGAAAAAAGAGGA---GTGGGA---ATAGGAGCT---
------TTGTTCCTTGGGTTCTTGGGAGCA---GCAGGAAGCACTATG---GGCGCAGCATCA---
ATAACGCTGACGGTACAG---GCCAGACAA---TTATTG---TCTGGTATAGTGCAACAGCAAAAT---
AATTTG---CTGAGA---GCTATTGAGGCGCAACAGCATCTGTTGCAACTCACAGTCTGGGGCATC
>JF320059
TTCTATTGCAATACAACACAATTGTTTAATAGTATTTGGCCTACTAATGATACTAGCAAAGGGACTAAT----
----

ATCAAGGGAAATATCACACTCCCATGCAGAATAAGACAAATTGTAAACAGGTGGCAGGAAGTAGGAAAAGCAA
TGTATGCCCCTCCCATTAAAGGACCAATTACATGTATATCAAAT---
ATTACAGGGTTGCTATTAACAAGAGATGGTGGAGGTGCCAGTAAT----------------------------
----

GGGACAAATGAGACCTTCAGACCTGGAGGAGGAGATATGAGGGACAATTGGAGAAGTGAATTATATAAATACA
AAGTGGTGAAAATTGAACCATTA---GGAATAGCACCCACCAGGGCAAGGAGAAGAGTGGTG-----------
-----------------------------CAGAGAGAAAAAAGAGCA---GTGGGA---TTTGGAGCT------
---TTGTTCCTTGGGTTCTTGGGAGCA---GCAGGAAGCACTATG---GGCGCAGCTTCA---
ATGACGCTGACGGTACAG---GCCAGACAA---TTACTG---TCTGGTATAGTGCAACAGCAGAAC---
AATCTG---CTGAGG---GCTATTGAGGCGCAACAGCATCTGTTGCAACTCACAGTCTGGGGCATA
>JF320361
TTCTACTGTAACACAACACCACTATTTAATAGTACTTGGAATAATACTGAAAGGTGGAATGGTACTGAAGGG-
----

TTAAATGGTACCATCACACTCCAATGCAGAATAAAACAAATTATAAACCTGTGGCAGGAAGTAGGAAAAGCAA
TGTATGCCCCTCCCATCAGCGGACAAATTAGATGTTCATCAAAT---
ATTACAGGGCTGCTGATAGTAAGAGATGGTGGTATTAATACTACTAAC-------------------------
----

GAGACGACCGAGATCTTCAGACCTGGAGGAGGAGACATGAGGGACAATTGGAGAAGTGAATTATATAAATATA
AAGTAGTAAAAATTGAACCATTA---GGAGTAGCACCCACCAAGGCAAAGAGAAGAGTGGTG-----------
-----------------------------CAAAGAGAAAAAAGAGCA---GTGGGAACTATAGGAGCT------
---ATGTTCCTTGGGTTCTTGGGAGCA---GCAGGAAGCACTATG---GGCGCAGCGTCA---
ACAGCGCTGACGGTACAG---GCCAGACAA---TTATTG---TCTGGTATAGTGCAACAGCAGAGC---
AATCTG---CTGAGG---GCTATTGAGGCACAACAGCATGTGTTACAACTCACAGTCTGGAGCATC
>JF320054
TTCTACTGTAATACAACACAACTGTTTAATAGTTCTTGGAGGGTTAATGAGACTTGGAATAATACT-------
----

GAAGAGGGCAATATCATACTCCCCTGCAGAATAAAACAATTTATAAACATGTGGCAGGGAGTAGGAAAAGCTA
TGTATGCCTCTCCCATTGCAGGACGAATTAAATGTAGATCAGAT---
ATTACAGGGCTGCTATTACTAAGAGATGGTGGACAAGAAAATAACACAGAG----------------------

----

ACCAATAACACAGAGACCTTCAGACCTGGAGGAGGAGATATGAAAGACAATTGGAGAAGTGAATTATATAAAT
ATAAAGTAGTAAAAATTGAACCATTA---GGGATAGCACCCACTAAGGCAAAGAGAAGAGTGGTG--------
------------------------------CAGAGAGAGAAAAGAGGA---GCGGGA---ATAGGAGCT---
------CTGTTCCTTGGGTTTCTGGGAGCA---GCAGGAAGCACTATG---GGCGCAGCGTCA---
ATGACGCTGACGGTACAG---GCCAGACAA---TTATTG---TCTGGTATAGTGCAACAGCAGAAT---
AATCTG---CTGAAG---GCTATTGAGGCGCAACAGCATATGTTGCAACTCACAGTCTGGGGCATC
>EU577917
TTCTACTGTGACACAACAAAACTGTTTGATAGTATTTGGGAGTTTACTAAAGGGAATATTACGAATATTACTG
CAAGATCAAAT---
-------------AGCACT------
GAAAGAAATAACACAATCACACTCCAATGTAGAATAAAACAAATTATAAATATGTGGCAGGAAGTAGGAAAAG
CAATGTATGCCCCTCCCATTAGAGGAGATATTAGCTGTTCATCAAAA---
ATTACAGGGCTGCTATTAACAAGAGATGGTGGTAGTAACAACGATAGCGAG---------------------
----

ACCAACACCACTGAGGTCTTCAGACCTGGAGGAGGAGATATGAGGGACAATTGGAGAAGTGAATTATATAAAT
ATAAGGTAGTAAGAATTGAACCATTA---GGAGTAGCACCCACCAAGGCAAAGAGAAGAGTGGTG--------
------------------------------CAGAGAGAAAAAAGAGCA---TTGGGA---TTCGGAGCG---
------TTGTTCCTTGGGTTCTTGGGAGCA---GCAGGAAGCACTATG---GGCGCAGCGTCG---
GTGACGCTGACGGTACAG---GCCAGACAA---TTATTG---TCTGGTATAGTGCAACAGCAAAAC---
AACTTG---CTGAGA---GCTATTGAGGCGCAACAGCATCTGTTGCAACTCACCGTCTGGGGCATC
>EU578004
TTCTACTGTAATACATCAGGACTGTTTAATAGTACTTGGAATGTTAATAAAAATGAAACTAATAGCACA----
----

CACAATACCAATATCACACTCCCATGTAGAATAAAACAAATTATAAACATGTGGCAGGAAGTAGGAAAAGCAA
TGTATGCCCCTCCCATCAGAGGACAAATTAGATGTGTATCAAAT---
ATTACAGGATTGCTATTAACAAGAGATGGTGGTAATAGTAATGGCAATAGT---------------------
----

ACTGAAAATGAGACTGAGATCTTCAGACCTGGAGGAGGAGATATGAGGGACAATTGGAGAAGTGAATTATATA
AATATAAAGTAGTAAAAATTGAACCATTA---GGAATAGCACCCACCAAGGCAAGGAGAAGAGTGGTG-----
-----------------------------------CAAAGAGAAAAAAGAGTA---GTGGGA---
CTAGGAGCT---------CTGTTCCTTGGGTTCTTGGGAGCA---GCAGGAAGCACTATG---
GGCGCAGCGTCA---ATAACGCTGACGGTACAG---GCCAGAATA---TTACTG---
TCTGGTATAGTGCAACAGCAGAAC---AACCTG---CTGAAG---
GCTATTGAGGCGCAACAGCGTATGTTGCAACTCACAGTCTGGGGCATT
>KM258984
TTCTATTGTAATACAACAAAACTGTTCAAAAATAGTACTTGG------------------------------
----
-------------------AAT---
GAGACTGAAGGGAATATCACAATCCCATGCAGAATAAAGCAAATTATAAACATGTGGCAGGAAGTAGGAAAAG
CAATGTATGCCCCTCCCATTAGAGGACAAATCAGATGTTCATCAAAT---
ATTACAGGGCTGATATTAACAAGAGATGGTGGG---------------------------------------
----
ACTAATGGGACTAAT---
GAGACAGAAACCTTCAGACCGGGAGGAGGAAATATGAAGGACAATTGGAGAAGTGAATTATATAAATATAAAG
TAGTAAAAATTGAACCATTA---GGAGTAGCACCCACCAGGGCAAAGAGAAGAGTGGTG--------------
-------------------------CAAAGAGAAAAAAGAGCA---GTGGGA---CTAGGAGCC---------
TTGTTCCTTGGGTTCTTGGGAGCA---GCAGGAAGCACTATG---GGCGCAGCGTCG---
ATAACGCTGACGGTACAG---GCCAGAATA---TTATTG---TCTGGTATAGTGCAACAGCAGAAC---
AACTTG---CTGAAA---GCTATTGAGGCGCAACAGCATATGTTGAAACTCACAGTCTGGGGCATC
>KM259000
TTCTACTGTGATACAACAAATCTGTTTAATAGTACTTGGTTTCGGAATGATACTGGAAAGTCACAGAATAATA
CTATGGAGCCA---TGGACTTGGAATAATGAGACAGGGACATGGGATGATAAAAAGTTA---
TGGACATGGACTAATATT------------------
AATGATACAGAACACATCACACTCCAATGTAGAATAAAACAAATTATAAACAGATGGCAGGAAGTAGGAAAAG
CAATGTATGCCCCTCCCATCAGCGGACTAATTAATTGTTCATCAAAT---

ATTACAGGGCTAATATTAGCAAGAGATGGT---

ATTGACAATAAAACTGAGACCTTCAGACCTATAGGAGGAAATATGAAGGACAATTGGAGAAGTGAATTATATA
AATATAAAGTAGTAAAAATCGAACCATTA---GGAATAGCACCCACCAGGGCAAAGAGAAGAGTGGTG-----
----------------------------------CAGAGAGAAAAAAGATCA---GTGACG---
CTGGGAGCT---------ATGTTCCTTGGGTTCTTGGGAGCA---GCAGGAAGCACTATG---
GGCGCAGCGTCG---CTGACGCTGACGGTACAG---GCCAGACTA---TTATTG---
TCTGGTATAGTGCAACAGCAGAAC---AATCTG---CTGAGG---
GCTATTGAGGCGCAACAGCATATGTTGCAACTCACAGTCTGGGGCATC
>KM259100
TTCTACTGTAATTCAACACAACTGTTTAATAATACTTGGGATGGTACTAAGTGGAATGAT-------------
--ACT---
GCTTGGAATGTTACTGCAGTG---------------
TCAAAAAACGAAAGCATCACACTCCAATGCAAAATAAAACAAATTATAAACATGTGGCAGAAAGTAGGAAAAG
CAATGTATGCCCCTCCCATTAGGGGAACAATTAGATGTTCATCAAAC---
ATTACAGGACTGATATTAACAAGAGATGGTGGTATTAAG----------------------------------
----
AACGAATCCGAGAATAACGGGTCC---------
AAGACCGAGATCTTCAGACCTGGAGGAGGAGATATGAGGGACAATTGGAGAAGTGAATTATATAAATATAAAG
TAGTAAAAATTGAACCATTA---GGAGTAGCACACACCAAGGCAAAGAGAAGAGTGGTG--------------
-------------------------CAGAGAGAAAAAAGAGCA---GTGGGA---ATAGGAGCT---------
GTGTTCCTTGGGTTCTTGGGAGCA---GCAGGAAGCACTATG---GGCGCAGCATCA---
ATGACGCTGACGGTACAG---GCCAGGCTA---TTATTG---TCTGGTATAGTGCAACAGCAGAAC---
AATTTG---CTGAGG---GCTATTGAGGCGCAACAGCATCTGTTGCAACTCACAGTCTGGGGCATC
>DQ410616
TTCTACTGCAGTACAACAAGACTGTTCAATAGTACTTGGTATGGGAATGGTACTGGCACAATCGAGAAT----
----

GGTACTGACACAATCATACTCCCATGCCGAATAAAACAAATTATAAACATGTGGCAGGAAGTAGGAAAAGCAA
TGTATGCCCCTCCCATCAGGGGAAAAATTATGTGTTCATCAAAT---
ATTACAGGGCTGCTATTAACAAGAGATGGCGGGAATAACAATAACGAAACG----------------------
----

AACAACACTGAGATCTTCAGACCTGGAGGAGGAGATATGAGGGACAATTGGGGGGAAAGTGAATTATATAAAT
ATAAAGTAGTAAAAATTGAACCATTA---GGAATAGCACCCACCAAGGCAAAGAGAAGAGTGGTG--------
-------------------------------CAGAGAGAAAAAAGAGCA---GTGGGA---ATAGGAGCT---
------GTGTTCCTTGGGTTCTTGGGAGCA---GCAGGAAGCACTATG---GGCGCAGCGTCA---
GTGACGCTGACGGTACAG---GCCAGACTG---TTATTG---TCTGGTATAGTGCAACAGCAAAAC---
AATTTG---CTGAGG---GCTATTGAGGCGCAACAGCATCTGTTGCAACTCACAGTCTGGGGCATC
>DQ410553
TTCTACTGTAATACATCCCAACTGTTTAATAGTACTTGGAATCCTAATGGTACTTGGGAAGGGACGAAT----
----

GTTACAGAAAATACCACACTCCCATGTAGAATAAAACAAATTATAAACATGTGGCAGGAAGTAGGAAAAGCAA
TGTACGCCCCTCCCATCAGTGGAATAATTAAATGTACATCAAAT---
ATTACAGGGCTACTGTTAACAAGAGATGGTAGTGGTGGTGCTACTAAC-------------------------
----

AACACCACCGAGATCTTCAGACCATTAGGCGGAGATATGAGGGACAATTGGAGAAGTGAATTATATAAATATA
AAGTAGTAAAAATTGAACCATTA---GGAGTAGCACCCACCAGGGCAAAGAGAAGAGTGGTG-----------
----------------------------CAGAGAGAAAAAAGAGCA---GTGGGA---CTAGGAGCT------
---GTGTTCCTTGGGTTCTTGGGAGCA---GCAGGAAGCACTATG---GGCGCAGCGTCA---
GTGGCGCTGACGGTACAG---GCCAGACAA---TTGTTG---TCTGGTATAGTGCAACAGCAGAAC---
AATCTG---CTGAGG---GCTATTGAGGCGCAACAACATCTGTTGCAACTCACAGTCTGGGGCATC
>DQ410585
TTCTACTGCAATACGACATCACTGTTTAATAGTACTTGGGATAATAGTACTTGGGGTAATACTATTGAAGGGT
CAAAT--

AACACAAACACAAATATCACACTCCTATGTAGAATAAAACAGATTATAAACATGTGGCAGGGAGTAGGAAAAG

CAATGTATGCCCCTCCCGTCAGGGGACCAATTAAGTGTACATCAATT---
ATTACAGGGCTGTTGTTAACAAGGGATGGTGGTAGTAACAACCCT----------------------------
----

AATGAGACTTTCAGACCTGGAGGAGGAGAGATGAGGGACAATTGGAGAAGTGAATTTAATAAATATAAAGTAG
TAAAATTGAACCCAATGGGA---GGGGTAGCACCCCCCCCGGCAAAGAGGAGAGTGGTG--------------
-------------------------CAGAGAGAGAAAAGAGCA---GTGGGAACGCTGGGAGCT---------
ATGTTCCTTGGGTTCTTGGGAGCA---GCAGGAAGCACTATG---GGCGCAGCGTCA---
GTGACGTTGACGGTACAG---GCCAGACAG---TTATTG---TCTGGTATAGTGCAACAGCAGAAC---
AATCTG---CTGAGG---GCTATTGAGGCGCAACAACATATATTGCAACTCACAGTCTGGGGCATC
>DQ410596
TTCTACTGTAATACAACACAGCTGTTTAACAGTACTTGGCCGAGTAATAGTACTGATAATAGTACTGGTGAAG
GG---

TCAAATAGCACTATCACACTTCCATGCAGAATAAAACAAATTATAAACATGTGGCAACAAGTAGGAAAAGCAA
TATATAACCCTCCCATTCGAGGACGAATTAACTGTTTATCAAAT---
ATTACAGGGTTGCTACCCGTAAGGGATGGGGGGAGTGCCAATAGC----------------------------
----

AGTAGTGAGACCTTCAAACCTGGGGGAGGAGATATGAGGGACAATGGGAGAAGTGAATTATATAAATATAAAG
TAGTAAAAATTGAACCATTAGGG---GTAGCCCCCCCCCCCAAGGCAAAGAGAAGAGTGGTG-----------
----------------------------CAGAGAGAAAAAAGAGCAGGGGGAACAATAGGAGTTTTT------
---ATGTTCCTTGGGTTCTTGGGAGCA---GCAGGAAGCACTATG---GGCGCAGCGTCA---
GTGACGCTGACGGTACAG---GCCAGACAA---TTATTT---TTTGGTATAGTGCAACAGCAGAAC---
AATTTG---CTGAAA---GCCATTGAGGCACAACAACATATGTTGCAACTCACAGTCTGGGGCATC
>KX156472
TTCTACTGTAATACAACAGTACTGTTCAATAGTACTTGGAATGGTACTGAAGGATCAATTGCTGGA-------
----

GAAAATGACACAATCACACTCCCATGCAGAATAAAACAAATTATAAACTTGTGGCAGGAAGTAGGAAAAGCAA
TGTATGCCCCTCCCATCAAAGGACTAATTAGATGTTCATCAAAT---
ATTACAGGACTGTTATTAACAAGAGATGGTGGTGACCCAAATAGC----------------------------
----

ACTACTGAGACATTCAGACCTGGAGGAGGAGATATGAGGGACAATTGGAGAAGTGAATTATATAAATATAAAG
TAGTAAAAATTGAACCATTA---GGAATAGCACCCACCAAGGCAAAGAGAAGAGTGGTG--------------
-------------------------CAGAGAGAAAAAAGAGCA---GTGGGA---ATAGGAGCT---------
GTGTTCCTCGGGTTCTTGGGAGCA---GCAGGAAGCACTATG---GGCGCAGCGGCA---
GTGACGCTGACGGTACAG---GCCAGACAA---TTATTG---TCTGGTATAGTGCAACAGCAGAAC---
AATTTG---CTGAGA---GCTATTGAGGCGCAACAGCATATGTTGCAACTCACAGTCTGGGGCATC
>KX156483
TTCTACTGTAATTCAGCACAACTGTTTAATAGTACTTGGAATGGTACTGAAGGGATAAATACCACT-------
----

GGCAATGAAACTATCATACTCCCATGCAGAATAAAACAAATTATAAATATGTGGCAAGAAGTAGGAAAAGCAA
TGTATGCCCCTCCCATCAGAGGACAAATTAGATGTTCATCAAAT---
ATTACAGGGCTGCTATTAACAAGAGATGGTGGTAATAGCAATAGC----------------------------
----

ACAATCGAGACCTTCAGACCTGGGGGAGGAAATATGAAGGACAATTGGAGAAGTGAATTATATAAATATAAAG
TAGTAAAAATTGAGCCATTA---GGAATAGCACCCACCAAGGCAAAGAGAAGAGTGGTG--------------
-------------------------CAGAGAGAAAAAAGAGCA---ATAGCA------GGAGCG---------
TTGTTCCTTGGGTTCTTGGGAGCA---GCAGGAAGCACTATG---GGCGCAGCATCA---
GTGACGCTGACGGTACAG---GCCAGACAA---TTATTG---TCTGGTATAGTGCAACAGCAGAAC---
AATCTG---CTAAGG---GCTATTGAGGCGCAACAGCATATGTTGCAACTCACAGTCTGGGGCATC
>KX156523
TTCTACTGTAATTCAACACAACTGTTTGATAGCACTTGGAATATTACTTGGGATGGTAATAGTACTTGGAATA
GTACTGAG---

GAAGATAGAAATATCACACTCCCATGCAGAATAAAACAAATCATAAACATGTGGCAGGAAGTAGGAAAAGCGA

TGTATGCCCCTCCCATCAGTGGACAAATTAGATGTTCATCAAAT---
ATTACAGGGCTGCTAATAACAAGAGATGGTGGTAAGAGTAATGACACA----------------------
----

AACAACGAGACTGAGATCTTTAGACCTGGAGGAGGAAATATGAAGGACAATTGGAGAAGTGAATTATATAAAT
ATAAAGTAGTAAGAATTGAACCATTA---GGAGTAGCACCCACCAAGGCAAAGAGAAGAGTGGTG--------
------------------------------CAGAGAGAAAAAAGAGCA---GTGGGA---TTAGGAGCT---
------TTGTTCCTTGGGTTCTTGGGAGCA---GCAGGAAGCACTATG---GGCGCAGCATCA---
CTAACGCTGACGGTACAG---GCCAGACAA---TTATTG---TCTGGTATAGTGCAACAGCAGAAC---
AATTTG---CTGAGG---GCTATTGAGGCGCAACAGCATCTGTTGCAACTCACAGTCTGGGGCATC
>KX156374
TTCTACTGTAATACAACGCCATTGTTTAATAGTACTTCAACGCAGCTATTTGGGAATACTACTGAAGGG----
----

TCAAATGCCATTATCAAACTCCAATGCAGAATAAAACAAATTATAAACAGGTGGCAGGAAGTAGGAAAAGCAA
TGTATGCCCCTCCCATCAAAGGACCAATTAGCTGTTTGTCAAAT---
ATTACAGGGCTGCTACTACTAAGGGACGGTGGTACAAAGAATGAGTCCAAT-------------------
----

GGTACAGGGTCCGAAATCTTTAGACCTGGAGGAGGAGATATGAGAGACAATTGGAGAAGTGAATTATATAAAT
ACAAAGTAGTAAAAATTGAGCCATTA---GGAGTAGCACCTACCAAGGCAAAAAGAAGAGTGGTG--------
------------------------------CAAAGAGAAAAAAGAGCA---GTGGGAACGATAGGAGCT---
------ATGTTCCTTGGATTCTTGGGAGCA---GCAGGAAGCACTATG---GGCGCAGCGTCA---
GTGACGCTGACGGTACAG---GCCAGACAA---TTATTG---TCTGGTATAGTGCAACAGCAGAAC---
AATTTG---CTGAGG---GCTATTGATGCGCAACAGCATATGTTGCAACTCACAGTCTGGGGCATC
>FJ469703
TTCTACTGTAATACAACACCACTGTTTAATAGTACTTGGACTTGGAATAGTACTAGAATGTCTAGTACTGAAA
AT--

ATTACTGAGGGAAATATCACACTCCAATGCAGGATAAAACAAATTGTAAACATGTGGCAGGAAGTAGGAAAAG
CAATGTATGCCCCTCCCATCAAAGGACAGATTAGATGTTCATCAAAT---
ATTACAGGTCTAATATTAACAAGAGATGGTGGTAACATCAGCACT-------------------------
----

AACAACGAGACCTTCAGACCTGCAGGAGGAGATATGAGGGACAATTGGAGAAGTGAATTATATAAATATAAAG
TAGTAAAAATTGAACCATTA---GGGGTAGCACCCACCAAGGCAAAGAGAAGAGTGGTG-------------
------------------------CAAAGAGAAAAAAGAGCA---ATAGGA---ATAGGAGCT---------
GTGTTCCTTGGGTTCTTGGGAGCA---GCAGGAAGCACTATG---GGCGCAGCGTCA---
GTAACGCTGACGGTACAG---GCCAGGCAA---TTATTG---TCTGGTATAGTGCAACAGCAGAAC---
AATCTG---CTGAGA---GCTATTGAGGCGCAACAGCATCTGTTGCAACTCACAGTCTGGGGCATC
>FJ469706
TTCTACTGTAATACATCAAAACTGTTTAATAGCACCTGGGAATGGAATAATAGTACCAAAGATTGGAATAATA
CTGAAATGTCA---
---------AATAACACT------
GAAGGAAATAGAACTATCACTCTCCAATGCAGAATAAAACAAATTGTAAATATGTGGCAGGAAGTAGGAAAAG
CAATGTATGCCCCTCCCATCAGTGGACAAATTAACTGTTCATCAAAT---
ATTACCGGGTTGCTCTTAACAAGAGATGGTGGTATTAGCAACAGC-------------------------
----

AGCACCACCGAGATCTTCAGACTTGGAGGAGGAGATATGAGGGATAATTGGAGAAGTGAATTATACAGATATA
AAGTAGTAAAAATTGAACCATTA---GGAATAGCACCCACCAAGGCAAGGAGAAGAGTGGTG----------
---------------------------CAAAGAGAAAAAAGAGCA---GTGGGA---TTAGGAGCT------
---ATGTTCCTTGGGTTCTTGGGAGCA---GCAGGAAGCACTATG---GGCGCAGCATCA---
ATGATGCTGACGGTACAG---GCCAGACAA---TTATTG---TCTGGTATAGTGCAACAGCAGAGC---
AATCTG---CTGCAG---GCTATTGAGGCACAACAGCATCTGTTGAAACTCACAGTCTGGGGCATT
>FJ469718
TTCTACTGTAATTCAACACAACTGTTTAATAGTACCTGGACTAATAGTACTTGGTATAATAGTACTTGGAATA
AAACT---
------------------AAA---
GAGTCAAATGACACAATCATACTCCCATGCAGAATAAAACAAATTATAAACATGTGGCAGGAAGTAGGAAGAG

CAATGTATGCCAATCCCATCGCAGGACAAATTAGATGTTCATCAAAT---
ATCACAGGGTTGCTACTAACAAGAGATGGTGGTAATAACACAAAT----------------------------
----

GGGACCACCGAGATCTTCAGACCGGGAGGAGGAAATATGAAGGACAATTGGAGAAGTGAGCTATATAAATATA
AGGTAGTAGAAATTAAACCATTA---GGAGTAGCACCCACCAAGGCAAAAAGAAGAGTGGTG-----------
----------------------------CAGAGAGAAAAAAGAGCA---GTAACA---CTAGGAGCT------
---TTGTTCCTTGGGTTCTTGAGTGCA---GCAGGAAGCACTATG---GGCGCAGCGTCA---
ATAACGCTGACGGTACAG---GCCAGACAA---ATGTTG---TCTGGTATAGTGCAACAGCAAACC---
AACTTG---CTGAGG---GCTATTGAGGCGCAGCAGCATCTGCTGCAACTCACAGTCTGGGGCATC
>DQ410115
TTCTACTGTAATTCAACACAACTGTTTAATAGTACTTGGAATAGTAGTACTTGGAATAGTACTAGGTCAGATA
ATACT--

ACAGAAAATGGCACAATCACACTCCCATGCAGAATAAAACAAATTATAAACATGTGGCAGAAAGTAGGAAAAG
CAATGTATGCCCCTCCCATCAGAGGACAAATTAGATGTTCATCAAAT---
ATCACAGGGCTGCTATTAACAAGAGATGGTGGTAACGAGAACGAG----------------------------
----

ACTGAGAGCTTCAGACCTGGAGGAGGAGATATGAGGGACAATTGGAGAAGTGAATTATATAAATATAAAGTAG
TAAAAATTGAACCATTA---GGAGTAGCACCCACCAGAGCAAAGAGAAGAGTGGTG-----------------
----------------------CAGAGAGAAAAAAGAGCA---GTGGGA---ATAGGAGCT---------
GTGTTCCTTGGGTTCTTGGGAGCA---GCAGGAAGCACTATG---GGCGCAGCGTCA---
ATGACGCTGACGGTACAG---GCCAGACTA---TTATTG---TCTGGTATAGTGCAACAGCAGAAC---
AATTTG---CTGAGG---GCTATTGAAGCGCAACAGCATCTGTTGCAACTCACAGTCTGGGGCATC
>DQ886037
TTCTACTGTAATTCATCACAACTGTTTAATAGCACTTGGCATACGAATAAAACTAAGGGGTTAAATGACACTG
GA---

ATTAATGGCACAATCATACTCCCATGCAGAATAAAACAAATTGCTATCTTGCGGTTGGAAGTAGGTAAAGCAA
TGTATGCCCCCCCCCCAATTAAACAACAAAGATGTGAGTCATAT---
ATTACAGGGCTGCTGTTAACAAGAGATGGTGGTAATAACAATAAT----------------------------
----

ACCGAAAACGAGATATTCAGACCTGCAGGAGGAAATATGAAGGACAATTGGAGAAGTGAATTATATAAATACA
AAGTAGTACAAATTGAACCATTA---GGAATAGCACCCACCAAGGCAAAGAGAAGAGTGGTG-----------
----------------------------CAGAGAGAAAAAAGAGCA---GTGGGA---CTAGGAGCA------
---CTGTTCCTTGGGTTCTTGGGAGCA---GCAGGAAGCACTATG---GGCGCAGCGTCG---
GTGACGCTGACGGTACAG---GCCAGACAA---TTATTG---TCTGGTATAGTGCAACAGCAGAGC---
AATTTG---CTGAGA---GCTATTGAGGCGCAACAGCATCTGTTGCAACTCACAGTCTGGGGCATC
>FJ653236
TTCTACTGTAGTACAACAAACCTGTTTAATAGTACTTGGAATAGTACTAGGAATGATAGAGGG----------
----

TTCAATGGCACTATCACACTCCCATGCAGAATAAAACAAATTATAAACATGTGGCAGGAAGTAGGAAAAGCGA
TGTATGCCCCTCCCATCAAAGGACAAATTAGATGTTCATCAAAT---
ATTACAGGGCTGCTATTAACAAGAGATGGGGGTACTAATAATAATAATGGT----------------------
----

ACTGAGAACGGGACAGAGATCTTTAGACCTGGAGGAGGAAATATGAAGGACAATTGGAGAAGTGAATTATATA
AATATAAAGTAGTAAAAATTGAACCAGTA---GGAATAGCACCCACCAAGGCAAAGAGAAGAGTGGTG-----
----------------------------------CAGAGAGAAAAAAGAGCA---GTGGGA---
ATAGGAGCT---------GTGTTCCTTGGGTTCTTGGGAGCA---GCAGGAAGCACTATG---
GGCGCAGCGTCA---GTGACGCTGACGGTACAA---GCCAGACAA---TTATTG---
TCTGGTATAGTGCAACAGCAAAGC---AACTTG---CTGAGG---
GCTATTGAGGCGCAACAGCATCTGTTGCAACTCACAGTCTGGGGCATC
>FJ653196
TTCTACTGTAATACAACAAAACTGTTTAACAGTACTTGGAATAGTACTGAAGGGTCAGTGGATGACACAATTG
AAGGG--

ACAGGGAATGACACAATTACACTCCCATGCAGAATAAAACAAATTATAAACATGTGGCAGGAAGTAGGAAAAG
CAATGTATGCCCCTCCCATCGAAGGACCAATTAAATGCACATCAAAT---
ATTACAGGGCTGCTACTAACAAGAGATGGTGGTAATGACACCACT----------------------------
----

AATAATGAGACTTTTAGACCTGGAGGAGGAGATATGAGGGACAATTGGAGAAGTGAACTATATAAATATAAAG
TAGTAAAACTTGAACCATTA---GGAATAGCACCCACCAAGGCAAAGAGAAGAGTGGTG--------------
-------------------------CAGAGAGAAAAAAGAGCA---GTAACA---ATAGGAGCC---------
ATGTTCCTTGGGTTCTTGGGAGCA---GCAGGAAGCACTATG---GGCGCAGCGTCA---
GTGACGCTGACGGTACAG---GCCAGACTA---TTATTG---TCTGGTATAGTGCAGCAGCAGAAC---
AATCTG---CTGAGG---GCTATTGAGGCGCAACAGCATCTGTTGCAACTCACAGTCTGGGGCATC
>FJ653597
TTCTACTGTGATACAACACAGCTGTTTAACAGCACTTGGCAGTCTAATGGTACTAGGAATGATACTGAGGAGT
CA---

GGGTCAAATGACGCTATCACACTCCAATGCAGAATAAAACAAATTATAAACAGGTGGCAGGAAGTAGGAAAAG
CAATGTATGCCCCTCCCATCAGTGGACACATCAACTGCTCATCAAAT---
ATTACAGGGATACTATTAACAAGAGATGGTGGTAACGAGAGCAGT----------------------------
----

AGCACCGAGACCTTCAGACCTGCAGGAGGAGATATGAGGGACAATTGGAGAAATGAATTATATAAATATAAAG
TAGTAAAAATAGAGCCATTA---GGAATAGCACCCACCAAGGCAAAGAGAAGAGTGGTG--------------
-------------------------CAGAGAGAAAAAAGAGCA---GTGACG---CTGGGAGCT---------
ATGTTCCTTGGGTTCTTGGGAGCA---GCAGGAAGCACTATG---GGCGCAGCGTCG---
ATGACGCTGACGGTACAG---GCCAGACAA---TTATTG---TCTGGTATAGTGCAACAGCAGAGT---
AATTTG---CTGAAG---GCTATTGAGGCGCAACAACATCTGTTGCAACTCACAGTCTGGGGCATC
>FJ653474
TTCTACTGTAATACAACACCACTGTTTAATAGTACTTGGAATGGTAATGAAACTTGGAATAGCACTGAAAAGT
CA---

GATAACACCACCATCACACTCCAATGCAGAATAAAACAAATTATAAACATGTGGCAGGAAGTAGGAAAAGCAA
TGTATGCCCCTCCCATCGCGGGACCAATTAGATGTTCATCAAAT---
ATTACAGGACTGCTATTAATAAGAGATGGTGGTAGTAATGGTAATGAG-------------------------
----

ACCAATAAAACTGAGATTTTTAGACCTGGAGGAGGAAATATGAAGGATAATTGGAGAAGTGAGTTATATAAAT
ATAAAGTAGTAAAAATTGAACCATTA---GGAATAGCACCCACCAAGGCAAAGAGAAGAGTGGTG--------
-------------------------------CAGAGAGAAAAAAGAGCA---GTGGGA---CTAGGAGCT---
------TTGTTCCTTGGGTTCTTGGGAGCA---GCAGGAAGCACTATG---GGCGCAGCGTCA---
GTGACGCTGACGGTACAG---GCCAGGCGA---TTATTG---TCTGGTATAGTGCAACAGCAGAAC---
AACTTG---CTGAGG---GCTATTGAGGCGCAACAGCATCTGTTGCAACTCACGGTCTGGGGCATC
>FJ653390
TTCTACTGTAATACAACAAAGCTGTTTAGCAGTACTTGGAGTGGTAATAGTACTTTGAATGATACTGAAAGGT
CAGATACT---
-------------------GAA---
AGGTCAGATATCAATATCACACTCCCATGTAGAATAAGGCAAATTGTAAACAGATGGCAGGAAGTAGGGAAAG
CAATGTATGCCCCTCCCATCAGAGGACAAATTAGCTGTTCATCAAAT---
ATTACAGGGCTGCTATTAACAAGAGATGGTGGTGATATTAGTGACAGT-------------------------
----

AATGGGACCGAGACCTTCAGACCTGGAGGAGGAGATATGAGAGACAATTGGAGAAGTGAATTATATAAATATA
AAGTAATAAAAATTGAACCATTA---GGAGTAGCACCCACCAAGGCAAAGAGAAGAGTGGTG-----------
----------------------------CAGAGAGAAAAAAGAGCA---GTGGGA---TTGGGAGCT------
---GTGTTCATTGGGTTCTTGGGAGCA---GCAGGAAGCACTATG---GGCGCAGCGTCA---
GTGACGCTGACGGTACAG---GCTAGACAA---TTATTG---TCTGGTATAGTGCAACAGCAGAAC---
AATCTG---CTGAGA---GCTATTGAGGCGCAACAACATTTGTTGCAACTCACAGTCTGGGGCATT
>FJ653478
TTCTACTGTAATACAACAGCACTGTTTAATGGCACTTGGTATAATAGTACTTGGAATACTTGGAATACTACTG
CAAAT--
-------------------GGC---

ACCGCAGAGTCAAATATCACACTCCCCTGCAGAATAAAACAAATTATAAACATGTGGCAGGGAGTAGGAAGAG
CAATGTATGCTCCTCCTATCAGAGGACAAATTAAATGCTCATCAAAT---
ATTACAGGGCTACTATTAACAAGAGATGGTGGGGATGGTAATGATACC-------------------------
----

AATAGACCCGAGACCTTCAGACCTGGAGGAGGAGATATGAGGGACAATTGGAGAAGTGAATTATATAAATATA
AAGTAGTAAGCATTGAACCATTA---GGAGTAGCACCCACCAAGGCAAAGAGAAGAGTGGTG-----------
----------------------------CAGAGAGAAAAAAGAGCA---GTGGGA---ATGGGAGCT------
---ATGATCCTTGGGTTCTTGGGAGCA---GCAGGAAGCACTATG---GGCGCAGCGTCA---
ATAACGCTGACGGTACAG---GCCAGACAA---TTACTG---TCTGGTATAGTGCAACAGCAGAAT---
AATTTG---CTGAGG---GCTATTGATGCGCAACAGCATATGTTGCAACTCACCGTCTGGGGCATC
>FJ653547
TTCTACTGTAATACATCAGGACTGTTTAATAACAGGACCGGGAATAGTACTGAAGGA----------------
----

GTAAATACCACCATCACACTCCCATGCAAAATAAAACAAATTGTAAACATGTGGCAGGAAGTAGGAAAGGCAA
TGTATGCCCCTCCCATCAGAGGACAAATTAACTGTACATCAAAT---
ATTACAGGGATACTATTAACAAGAGATGGTGGTAACACAAATATCACC-------------------------
----

ACCACCACCGAGACCTTCAGACTTGGAGGAGGAGATATGAGGGACAATTGGAGAAATGAGTTATATAAATATA
AAGTAGTAAAAATTGAACCATTA---GGAGTAGCACCCACCAGGGCAAAGAGAAGAGTGGTG-----------
----------------------------CAAAGAGAAAAAAGAGCA---GTGGGA---CTAGGAGCT------
---GTGCTCATTGGGTTCTTGGGAGCA---GCAGGAAGCACTATG---GGCGCAGCGTCA---
ATAACGCTGACGGTACAG---GCCAGACAA---TTATTG---TCTGGTATAGTGCAACAGCAGAAC---
AATCTG---CTGAGA---GCTATTGAGGCGCAACAGCATATGTTGCAACTCACAGTCTGGGGCATC
>HQ217476
TTCTACTGTGATACAACACAGCTGTTTAATAGTACTTGGGTAAATGGTAATTGGGTTAATGGTACTAGGCTGT
CGAAT--
-------------------GAA---
ACTGGGAATGATACTATCACACTCCCATGCAAAATAAAACAAATCATAAACAGATGGCAGGAAGTAGGAAAAG
CAATGTATGCCCCTCCCATCAGAGGACAAATTAGATGTTCATCAAAT---
ATTACAGGGCTGCTCTTGACAAGAGATGGTGGTCTACACAACAATAGC-------------------------
----

AATAGCAACGAGACTTTCAGACCTGGAGGAGGAGATATGAGGGACAATTGGAGAAGTGAATTATATAGATATA
AAGTAGTAAAAATTGAACCATTA---GGAATAGCACCCACCAAGGCGAGGAGAAGAGTGGTG-----------
----------------------------CAGAGAGAAAAAAGAGCA---GCGGGA---ATAGCAGCT------
---ATGCTCATTGGGTTCTTGGGAGCA---GCAGGAAGCACTATG---GGCGCAGCGTCA---
GTGACGCTGACGGTACAA---GCCAGACAA---TTATTG---TCTGGTATAGTGCAACAGCAAAAC---
AATTTG---CTGAGA---GCTATTGAGGCGCAACAGCATATGTTGCAACTCACAGTCTGGGGCATT
>HQ217546
TTCTACTGTAATTCCACAGAACTGTTTAGTAGTACTTGGGATGGCACTAACTTGACTAATGATACTGAAAGGT
CA---

AATGACACTGTTATCACACTCCCATGCAGAATAAAACAAATAATAAACATGCGGCAGGAAGTAGGAAAAGCAA
TGTATGCCCCTCCCATCACAGGACAAATTAGATGTTCATCAAAT---
ATTACAGGGCTGCTATTAACAAGAGATGGTGGTAAAGGCACTAACGGAACT----------------------
----

GATAACCAGACTGAGATCTTTAGACCTGCAGGAGGAGATATGAAGGACAATTGGAGAAGTGAATTATACAAAT
ATAAAGTAGTAAAAGTTGAACCATTA---GGAGTAGCACCCACCAAGGCAAGGAGAAGAGTGGTG--------
-------------------------------CAAAGAGAAAAAAGAGCA---GTGGGA---ATAGGAGTG---
------TTACTCCTTGGGTTCTTGGGAGCA---GCAGGAAGCACTATG---GGCGCAGCGGCG---
ATGACGCTGACGGTACAG---ACCAGACAA---TTATTG---TCTGGTATAGTGCAACAGCAGAAC---
AATCTG---CTGAGG---GCTATTGAGGCGCAACAGCATCTGTTGCAACTCACAGTCTGGGGCATC
>HQ217818
TTCTACTGTAATACAACACCACTGTTTACTGGTACTTGGGATGGTAGTACTTGGAATAGCAGTAACAATGAG-
----

GAAAATGAAACACTCACACTCCAATGCAGAATAAAACAAATTATACGCCGGTGGCAGGAAGTAGGACAAGCAA
TGTATGCCCCTCCCATCAGAGGACAGATTAGATGTTCATCAAAT---
ATTACAGGGTTACTAATAACAAGGGATGGTGGTGACGACAACGGG----------------------------
----

ACGACCGAGGTCTTTAGACCTGGAGGAGGGGATATGAGAGACAATTGGAGAAGTGAATTATATAAATATAAAG
TAGTAAAAATTGAACCATTA---GGAGTAGCACCCACCAAGGCAAAAAGAAGAGTGGTG--------------
-------------------------CAGAGAGAAAAAAGAGCA---GTGGGA---TTCGGAGCT---------
ATGTTCCTTGGGTTCTTGGGAGCA---GCAGGAAGCACTATG---GGCGCAGCATCA---
CTAACGCTGACGGTACAG---GCCAGACAA---TTGTTG---TCTGGTATAGTGCAACAGCAAAAC---
AATCTG---CTGATG---GCTATTGAGGCGCAACAACATCTGTTGCAACTCACAGTCTGGGGCATC
>HQ217889
TTCTACTGTAATACAACAGATCTGTTTAAGAGCAATTGGACCTTTAATGAGAATGCTACTTGGCATGATAAAA
GC---

AATGGAACAAATATCATACTCCCATGCAAGATAAAACAAATTATAAACATGTGGCAGGAGGTAGGAAAAGCAA
TGTATGCCCCTCCCATCAGAGGACTAATTAGCTGTTCATCAAAT---
ATTACAGGGCTGCTATTAACAAGAGATGGGGGTAACAAGAATGAGACT-------------------------
----

AACGAGACCGAAACCTTCAGACCTATAGGAGGAAATGTGAAGGACAATTGGAGAAGTGAATTATATAAATATA
AAGTAGTAAGAATTGAACCATTA---GGAGTAGCACCCACCAAGGCAAGGAGAAGAGTGGTG-----------
----------------------------CAGAGAGAAAAAAGAGCA---GTGGGAATAGTAGGAGCT------
---ATGTTCCTTGGGTTCTTGGGAGCA---GCAGGAAGCACTATG---GGCGCAGCGTCA---
TTGGCGCTGACGGTACAG---ACCAGACAA---TTATTG---TCTGGTATAGTGCAGCAGCAGAAC---
AATTTG---CTGAGG---GCTATTGAGGCGCAACAGCATCTGTTGCAACTCACAGTCTGGGGCATC
>GU330736
TTCTACTGTAACTCATCACAACTGTTTAATAGTACTTGGAATAGTACTGAGGATAATGAGACTAATAGC----
----

ACTGAAAGAAATATCACAATCCCATGCAGAATAAAACAAATTATAAACATGTGGCAGGAAGTAGGAAGAGCAA
TGTATGCCCCTCCCATCCAAGGGCTAATTAGATGTTCATCAAAT---
ATTACAGGGCTACTCTTAACAAGAGATGGTGGTAATATAACTGAGACT-------------------------
----

AATAATACAGAGATCTTCAGACCTGAAGGAGGAAATATGAAGGACAATTGGAGAAGTGAATTATATAAATATA
AAGTAGTACAAATTGAACCATTA---GGAATAGCACCCACCAAGGCAAAGAGAAGAGTGGTG-----------
----------------------------CAGAGAGAAAAAAGAGCA---GTGGGAATGCTAGGAGCT------
---ATGTTCCTTGGGTTCTTAGGAGCA---GCAGGAAGCACTATG---GGCGCAGCGTCA---
TTGACGCTGACGGTACAG---GCCAGACAA---TTATTG---TCTGGTATAGTGCAGCAGCAAAAC---
AACTTG---CTGAGA---GCTATTGAGGCGCAACAACATCTGTTGCAACTCACAGTCTGGGGCATT
>GU330809
TTCTACTGTAATTCAACACAACTGTTTAATAGTACTTGGTTTAATGGTACTTGGGATAATACTGCAGAGTCAA
ATAGC--
-------------ACTGAA------
GGAAATGGCACAATGATCACACTCCCATGCAGAATAAAACAAATTATAAACATGTGGCAGGCAGTAGGAAAAG
CAATGTATGCCCCTCCCATCAGAGGACAAATTAGATGTTCATCAAAT---
ATTACAGGACTACTGTTAACAAGAGATGGTGGTATCAATACTCCCGAT-------------------------
----

AACAATGAGACTGAGATCTTCAGACCTGCAGGGGGAAATATGAAGGACAATTGGAGAAGTGAATTATATAAAT
ATAAAGTAGTAAAAATTGAACCATTA---GGGATAGCACCTACCAAGGCAAAGAGAAGAGTGGTG--------
-------------------------------CAGAGAGAAAAGAGAGCA---ATAGGA---ATAGGAGCT---
------GTGTTCCTTGGGTTCTTGGGAGCA---GCAGGAAGCACTATG---GGCGCAGCGTCA---
ATAACGCTGACGGTACAG---GCCAGACAA---TTATTG---TCTGGTATAGTGCAGCAGCAGAGC---
AATTTG---CTGAGG---GCTATTGATGCGCAACAACATCTGTTGCAACTCACAGTCTGGGGCATC
>GU330839
TTCTACTGTAATACAACACAACTGTTTAATAGTACTTGGAATATAACACAACCGCTTACTTTGAATAGGACTA
AA---

```
GGGCCAAATGATACAATTACACTCCCATGCAGAATAAAACAAATTATAAATATGTGGCAGAAAGTAGGAAAAG
CAATGTATGCCCCTCCCATCAAAGGACAAATTAATTGTACATCGAAT---
ATTACAGGACTAATATTAACAAGAGATGGTGGAAATGAGACTAGG----------------------------
-------------------------------------------------------------------------
-------------
AACAACGAGACCTTCAGACCTACAGGAGGAGATATGAAGGACAATTGGAGAAGTGAATTATATAAATATAAAG
TAGTAAAAATTGAACCATTA---GGAGTAGCACCCACCAAGGCAAAGAGAAGAGTGGTG--------------
-------------------------CAGAGAGAAAAAAGAGCA---GTGGGA---CTAGGAGCT---------
CTGTTCCTTGGGTTCTTGGGAGCA---GCAGGAAGCACTATG---GGCGCAGCGTCA---
GTGACGTTGACGGTACAG---GCCAGACTA---TTATTG---TCTGGTATAGTGCAGCAGCAGAAC---
AACCTG---CTGAGA---GCTATTGAGGCGCAACAACATCTGTTGCAACTCACAGTCTGGGGCATC
>JF689862
TTCTACTGTAGTACAACACACCTGTTTAATAGTTCTTGGAATGGGACTGCATGGGTAAATGCCACAAATAAC-
-------------------------------------------------------------------------
----------------------------
ACTGGAGAAACTATCACACTCCCATGTAGAATTAAACAAATTATAAATATGTGGCAGGAAGTAGGAAAAGCAA
TGTATGCTCCTCCCATCAGTGGACCAATTAGATGTTTATCAAAC---
ATTACAGGCCTAATATTAACAAGAGATGGTGGTATTAACAATGAC----------------------------
-------------------------------------------------------------------------
----------
ACCGGGGTCGAGGTCTTCAGACCTGGAGGAGGAAATATGAAGGACAATTGGAGAAGTGAATTATATAAATATA
AAGTAGTACAAATTGAACCATTA---GGAGTAGCACCTACCAAGGCAAAGAGAAGAGTGGTG-----------
----------------------------CAGAGAGAAAAAAGAGCA---GTGGGAACGATAGGAGCT------
---ATGTTCCTTGGGTTCTTGGGAGCA---GCAGGAAGCACTATG---GGCGCAGCGTCA---
GTGACGCTGACGGTACAG---GCCAGACTA---TTATTG---TCTGGTATAGTGCAACAGCAGAAC---
AATCTG---CTGAGA---GCTATTGAGGCGCAACAGCATCTGTTGCAACTCACAGTCTGGGGCATC
>JF689863
TTCTATTGTAATACAACACAACTGTTTAATAGTACATGGTATAGTAATAGTACTTGGAATGAGACTGAAGAG-
-------------------------------------------------------------------------
----------------------------
ACAAATGACACTATCATACTCCCATGCAGAATAAAACAAATTATAAACCTGTGGCAGGAAGTAGGAAAAGCAA
TGTATGCCCCTCCCATCAAAGGAGTAATTAACTGTTTATCAAAC---
ATTACAGGGCTGTTATTGACAAGAGATGGTGGTAATGGTAATAGCAGC-------------------------
-------------------------------------------------------------------------
----------
AATGGGACTGAGGTCTTCAGACCTGGAGGAGGAGATATGAGGGACAATTGGAGAAGTGAATTATATAAATATA
AAGTAGTAAAAATTGAACCATTA---GGAATAGCACCCACCAAGGCAAAAAGAAGGGTAGTG-----------
----------------------------CAAAGAGAAAAAAGAGCA---GTGGGAACATTAGGAGCT------
---ATGTTCCTTGGGTTCTTGGGAGCA---GCAGGAAGCACTATG---GGCGCAGCGTCA---
ATGACGCTGACGGTACAG---GCCAGACTA---TTATTG---TCTGGTATAGTGCAGCAGCAGAGC---
AATCTG---CTGAGA---GCTATTGAGGCGCAACAGCGACTGTTGCAACTCACAGTCTGGGGCATC
>JF689866
TTCTACTGTAACACAACAAAACTGTTTAATAGTATTTGGTGGAGGAATGGTACTAATAATTGGAATGATACTA
AG-----------------------------------------------------------------------
-------------------------
AGTGACAATGACACAATCACACTCAAATGCAGAATAAAGCAAATTATAAACATGTGGCAGGAGGTAGGAAAAG
CAATGTATGCCCCTCCCATCAGAGGACAAATTAGATGTTCATCAAAT---
ATTACAGGGCTGATATTAACAAGGGACGGTGGGCGTACCAATGAAACA-------------------------
-------------------------------------------------------------------------
----------
AACAATACTGAGATCTTTAGACCTGCAGGAGGAAATATGAAGGACAATTGGAGAAGTGAATTATATAAATATA
AAGTAGTACAAATTGAACCATTA---GGAATAGCACCCACCAAGGCAAAGAGAAGAGTGGTG-----------
----------------------------CAGAGAGAAAAAAGAGCA---GTGGGA---ATAGGAGCT------
---CTGTTCCTTGGGTTCTTGGGAGCA---GCAGGAAGCACTATG---GGCGTAGCGTCA---
ATAACGCTAACGGTACAG---GCCAGACAA---TTATTG---TCTGGTATAGTGCAGCAGCAGAAC---
AATCTG---CTGAGG---GCTATTGAGGCGCAACAACATATGTTGCAACTCACAGTCTGGGGCATA
>JF689867
TTCTACTGTAACACAACAGGACTGTTTAACTCAACATTTAACTCAACAAAACTGCCTGATAGTAATAAT----
-------------------------------------------------------------------------
----------------------------
```

ACTGATTCAAATTTCACGATCCCATGCAGAATAAAACAAATTATAAACATGTGGCAGAAAGTAGGAAAAGCAA
TGTATGCCCCTCCCATCAGAGGACCAATTAGATGTTCATCAAAC---
ATTACAGGGCTGATATTAACAAGAGATGGTGGTGGTAATGAGAGC----------------------------
----

GGTAGAGAGACTTTCAGACCTGGAGGAGGAAATATGAAGGACAATTGGAGAAGTGAATTATATAAATATAAAG
TAGTAAAAATTGAACCATTA---GGAGTAGCACCCAACAAGGCAAAGAGAAGAGTGGTG--------------
-------------------------CAGAGAGAAAAAAGAGCA---GTGGGA---ATAGGAGCT---------
CTGTTCCTTGGGTTCTTGGGAGCA---GCAGGAAGCACTATG---GGCGCAGCGTCC---
GTGACGCTGACGGTACAG---GCCAGAAAA---TTACTG---TCTGGTATAGTGCAACAGCAGAAC---
AATTTG---CTGAAG---GCTATTGAGGCGCAACAGCATCTGTTGCAACTCACCGTCTGGGGCATC
>JF689870
TTCTATTGTAATTCAACACAACTGTTTAATAGTACTTGGAATGATACTTGGTCAAATAGCACTAGA-------
----

GGAAATGATACAATCACACTCCCATGCAGAATAAAACAAATTATAAACATGTGGCAGAAAGTAGGAAAAGCAA
TGTATGCCCCTCCCATCAGAGGACAAATTAGATGCTCATCAAAT---
ATTACAGGGCTGCTATTAACAAGAGATGGTGGCAATACAACCGGG----------------------------
----

AGCAGGTCAGAGACCTTTAGACCTGGAGGAGGAGACATGAGGGACAATTGGAGAAGTGAATTATATAAATATA
AAGTAGTAAAAATTGAACCATTA---GGAGTAGCACCTACCAGGGCAAAGAGAAGAGTGGTG----------
----------------------------CAGAGAGAAAAAAGAGCA---GTGGGA---ATAGGAGCT------
---ATGTTCCTTGGGTTATTGGGAGCA---GCAGGAAGCACTATG---GGCGCAGCGTCA---
ATGACGCTGACGGTACAG---GCCAGACTA---TTATTG---TCTGGTATAGTGCAACAGCAGAAC---
AACTTG---CTGAAG---GCTATTGAGGCGCAACAGCATCTGTTGCAACTCACAGTCTGGGGCATC
>JF689871
TTCTATTGTAATTCATCCCAATTGTTTAATAGTACTTGGAATAGTACTGAAGGGTCAAATAATGGTACTGAAG
TAACA--
-------------------AAT---
AATGGTACTGAAGTAATTACACTCCCATGCAGAATAAAACAAATTATAAACATGTGGCAGGAAGTAGGAAAAG
CAATGTATGCCCCTCCCATCAGAGGACAAATTAACTGCTCATCAAAT---
ATTACAGGGCTGTTATTAACAAGAGATGGTGGTCGTAACAGCACTGAG-------------------------
----

GACGATACTGAGACCTTCAGGCCTGGAGGAGGAGACATGAGGGACAATTGGAGAAGTGAATTATATAAATATA
AAGTAGTAAAAATTGAACCATTA---GGAGTAGCACCCACCAGGGCAAAGAGAAGAGTGGTG----------
----------------------------CAGAGAGAAAGAAGAGCA---GTGGGAACAATAGGAGCT------
---ATGTTCCTTGGGTTCTTGGGAGCA---GCAGGAAGCACTATG---GGCGCAGCGTCA---
GTGACGCTGACGGTACAG---GCCAGACAA---TTATTA---TCTGGTATAGTGCAACAGCAGAAC---
AATTTG---CTGAGA---GCTATTGAGGCGCAACAGCATCTGTTGCAACTCACAGTCTGGGGCATC
>JF689872
TTCTACTGTAATTCAACACAACTGTTTAATAGTACTTGGACTTGGAATGTTACTGGAGGAAATGCCACACTCA
CATCATCAAAC--
-------------AACACT------
GAAGGGAATGACACAATCAGACTCCCATGCAGAATAAAACAAATTATAAACATGTGGCAGGAAGTAGGAAAAG
CAATGTATGCCCCTCCCATCAGAGGGCAAATTAACTGTTCATCAAAT---
ATTACAGGGCTACTATTAATAAGAGATGGTGGTAACAAGAGAGAGAGCGGGAAC------------AAG----
----
AACGAGAGCGGGAAC---
AAGACTGAGATTTTCAGACCTGGAGGAGGAGATATGAGGGACAATTGGAGAAGTGAATTATATAAATATAAAG
TAGTAAAAATTGAACCATTA---GGAGTAGCACCCACCAAGGCAAAAAGAAGAGTGGTG--------------
-------------------------CAGAGAGAAAAAAGAGCA---GTGGGA---TTAGGAGCT---------
ATGTTCCTTGGGTTCTTGGGAGCA---GCAGGAAGCACTATG---GGCGCAGCGTCA---
GTGACGCTGACGGTACAG---GCCAGACAA---TTATTG---TCTGGTATAGTGCAACAGCAGAAC---
AATCTG---CTGAGG---GCTATTGAGGCGCAACAGCATCTGTTGCAACTCACAGTCTGGGGCATC
>JF689873
TTCTACTGTAATTCAACACAACTGTTTAATAGTACTTGGAATGTTACTAGTCCTTGGAATGTTACTGAAGGGT
CAAAT--

```
AACACTGAAAGAACTATCACACTCCCATGCAGAATAAAACAAATTATAAACATGTGGCAGAAAGTAGGAAAAG
CAATGTATGCCCCTCCCATCAAAGGACAAATTAGATGTTCATCAAAT---
ATTACAGGGCTGCTATTAACAAGAGATGGTGGTAATAGCGAGAAC----------------------------
-------------------------------------------------------------------------
----------
GAGACCACCGAGATCTTCAGACCTGCAGGAGGAGATATGAGGGACAATTGGAGAAGTGAATTATATAAATATA
AAGTAGTAAAAATTGAGCCATTA---GGAGTAGCACCCACCAAGGCAAAGAGAAGAGTGGTG-----------
----------------------------CAGAGAGAAAAAAGAGCA---GTGGGA---ATAGGAGCT------
---TTGTTCCTTGGGTTCTTGGGAGCA---GCAGGAAGCACTATG---GGCGCAGCGGCA---
GTGACGCTGACGGTACAG---GCCAGACTA---TTATTG---TCTGGTATAGTGCAACAGCAGAAC---
AATTTG---CTGAGG---GCTATTGAGGCGCAACAGCATCTGTTGCAACTCACAGTCTGGGGCATC
>JF689876
TTCTACTGCAATACATCACAACTGTTTAATAGTACTTGGAATGGTACTAAGTCAAATAACAATGGA-------
-------------------------------------------------------------------------
----------------------------
ATAAATGACACAATCATACTCCCATGTAGAATAAGACAAATTATAAACATGTGGCAGAAAGTAGGAAAAGCAA
TGTATGCCCCTCCCATCAGAGGAAAAATTGAGTGTAAGTCAAAT---
ATTACAGGGCTGCTATTAACAAGAGATGGTGGTACAAACGAGAACAATACC----------------------
-------------------------------------------------------------------------
-------
AATACCAACACCGAGACCTTCAGGCCTGGAGGAGGGAATATGAAGGACAATTGGAGAAGTGAATTATATAAAT
ATAAAGTAGTAAAAATTGAACCATTA---GGAGTAGCACCCACCAGGGCAAAGAGAAGAGTGGTG--------
-------------------------------CAGAGAGAGAAAAGAGCA---GTGGGA---CTAGGGGCT---
------GTGTTCCTTGGGTTCTTGGGAGCA---GCAGGAAGCACTATG---GGCGCAGCGTCA---
ATAACGCTGACGGTACAG---GCCAGACTG---TTATTG---TCTGGTATAGTGCAACAGCAGAAC---
AATTTG---CTGAGG---GCTATTGAGGCGCAACAGCATCTGTTGCAACTCACAGTCTGGGGCATT
>HQ216908
TTCTACTGTAATTCAACACAACTGTTTAATAGTACTTGGGGGAATGATAATAGTACTTGGGAAAATGATACT-
-------------------------------------------------------------------------
----------------------------
GAAGCAACTAACATCACTCTCCCATGCAGAATAAAACAAATTGTAAACATGTGGCAGCAAGTAGGAAAAGCAA
TGTATGCCCCTCCCATCAGAGGACAAATTAGATGTTCATCAAAT---
ATTACAGGGCTGATATTAACAAGAGATGGTGGTAACAACACGAAT----------------------------
-------------------------------------------------------------------------
-------------
GGGTCCGAGACCTTCAGACCTGGAGGAGGAGATATGAGGGATAATTGGAGAAGTGAATTATATAAATATAAAG
TAGTAAAGATTGAGCCATTA---GGAGTAGCACCCACCAAGGCAAAGAGAAGAGTGGTG--------------
-------------------------CAGAGAGAAAAAAGAGCA---GTGGGA---ATGGGAGCT---------
GTGTTCCTTGGGTTCTTGGGAGCA---GCAGGGAGCACTATG---GGCGCAGCGTCA---
ATGACGCTGACGGTACAG---GCCAGACTA---TTATTG---TCTGGTATAGTGCAACAGCAAAAC---
AATTTG---CTGAGG---GCTATTGAGGCGCAACAGCATCTGTTGCAACTCACAGTCTGGGGCATC
>HQ216925
TTTTACTGTAATACAACAAAACTGTTTAATAGTACAGAACTATTTAATAGTACTAAAGTATCAAATACA----
-------------------------------------------------------------------------
----------------------------
ACTGGAGACACTATCACACTCCAATGCAGAATAAAACAAATTATAAACATGTGGCAGAAAGTAGGAAAAGCAA
TGTATGCCCCTCCCATCAAAGGGAGAATTAGTTGTTCATCAAAC---
ATTACAGGACTGCTACTAACAAGAGATGGTGGTAATAACACTGGTAAC-------------------------
-------------------------------------------------------------------------
----------
AATGACAATGAGACCTTCAGACCTATAGGAGGAAATATGAAGGACAATTGGAGAAGTGAATTATATAAATATA
AAGTAGTGAAAATTGAACCATTA---GGAATAGCACCCACCAAGGCAAAGAGAAGAGTGGTG-----------
----------------------------CAGAGAGAAAAAAGAGCA---GTGGGA---TTTGGAGCG------
---GTGATCTTTGGGTTCTTGGGAGCA---GCAGGAAGCACTATG---GGCGCAGCGGCG---
GTAACGCTGACGGCACAG---GCCAGACTG---TTATTG---TCTGGTATAGTGCAACAACAGAAC---
AATCTG---TTGAGG---GCTATTGAGGCGCAACAGCATCTGTTGCAACTCACAGTCTGGGGCATC
>FJ152546
TTCTACTGTGATACAACAAAACTATTTAATAGTACTTGGAATAATAGTACTGAACTGGTAAATGGCACA----
-------------------------------------------------------------------------
----------------------------
```

GTTGACACAAATATCACACTCCCATGTAGAATAAAACAAATTATAAACATGTGGCAGGAAGTAGGAAAAGCAA
TGTATGCCCCTCCCATCAGAGGAAACATTAACTGTACATCAAAT---
ATTACAGGGCTGATATTAACTAGAGATGGTGGTAATCCAGATAACACCACA----------------------
----

GATAACACCACTGAGACCTTCAGACCTGGAGGAGGAGACATGAGGGACAATTGGAGAAGTGAATTATATAAAT
ATAAAGTAGTACAAATTGAGCCATTA---GGAATAGCACCCACCAAGGCAAAGAGAAGAGTGGTG--------
-------------------------------CAAAGAGAAAAAAGAGCA---GTGGGAACGCTGGGAGCT---
------ATGTTCCTTGGGTTCTTGGGAGCA---GCAGGAAGCACTATG---GGCGCAGCGTCA---
CTAACGCTGACGGTACAA---GCCAGACTA---TTGTTG---TCTGGTATAGTGCAACAGCAGAAC---
AATCTG---CTGAGA---GCTATTGAGGCGCAACAGCATCTGTTGCAACTCACAGTCTGGGGCATC
>FJ152547
TTCTACTGTGATACAACAAAACTGTTTAATAATTCTTGGAATGGTACATGGTCAAATAACAGTACA-------
----

GCAAATATCACAATCACACTCCCATGCAGAATAAGACAGATTGTAAACATGTGGCAGGAAGTAGGAAAAGCAA
TGTATGCCCCTCCTATCAGAGGAATAATTAACTGTACATCAAAT---
ATTACAGGGCTGCTATTAACAAGAGATGGTGGTAGTACCACGAGTAATAAT----------------------
----

ACCATAAACAAAACTGAAACCTTCAGACCTGGAGGAGGAGATATGAGAGACAATTGGAGAAGTGAATTATATA
AATATAAAGTAGTACAAATTAAACCATTA---GGAATAGCACCCACCCAGGCAAAGAGAAGAGTGGTG-----
----------------------------------CAAAGAGAA---AAAAGG---GGA------
ATAGGAGCT---------CTTTTCCTTGGGTTCTTGGGAGCA---GCAGGAAGCACTATG---
GGCGCAGCATCA---GTAACGCTGACGGTACAG---GCCAGACTA---TTATTG---
TCTGGTATAGTGCAACAGCAGAAC---AATCTG---CTGAGA---
GCTATTGAGGCGCAACAGCATCTGTTGCAACTCACAGTCTGGGGCATC
>JX863987
TTCTACTGTAATTCAACACAACTGTTCAATAGTTCTTGGAAGTGGAATAATGGTACTTGGAGTAGCAATGATT
CTAAAGGG---
-------------TTAAAT------
GGCACTGAGGACATAATCATACTCCCATGCAGAATAAAACAAATTATAAACATGTGGCAGAGAGTGGGAAAAG
CAATGTATGCCCCTCCCATCAGAGGAATAATTAACTGTACATCAAAC---
ATTACAGGGATACTGCTAACAAGAGATGGTGGTAGTAACACAAAC----------------------------
----

GATAATGAGACCTTCAGACCTGGAGGAGGAGATATGAGGGACAATTGGAGAAGTGAATTATATAAATATAAAG
TAGTAAAAATTGAACCATTA---GGAATAGCACCTACCAGGGCAAAGAGAAGAGTGGTG--------------
-------------------------CAGAGAGAAAAAAGAGCA---GTGGGA---ATAGGAGCT---------
ATGTTCCTTGGGTTCTTGGGAGCA---GCAGGGAGCACTATG---GGCGCAGCGTCA---
CTGACGCTGACGGTACAG---GCCAGACAA---TTATTG---TCTGGTATAGTGCAACAGCAGAAC---
AACCTG---CTGAGG---GCTATTGAGGCGCAACAGCATATGTTGCAACTCACAGTCTGGGGCATC
>JX863988
TTCTACTGTAGTACAACACAATTATTTAATGGTACTTGGAATGGTACTTGGGTTAATAATACTTGGAATGATA
CTGGAAAC---

AGCAATATAACTATCTCACTCCCATGCAAAATAAAACAAATTATAAACATGTGGCAGGGAGTAGGGAAGGCAA
TATATGCCCCTCCAATCAGGGGAGTAATTAAATGTACATCAAAT---
ATCACAGGGCTGATATTAACAAGAGATGGTGGTATTAATAATAGCGCC-------------------------
----

GAGGGCGAGACCTTCAGACCCCAAGGAGGAAATATGAAGGACAATTGGAGAAGTGAATTATATAAATATAAAG
TAGTAAAAATTGAACCATTA---GGAGTAGCACCCACCAAGGCAAAGAGAAGAGTGGTG--------------
-------------------------CAGAGAGAAAAAAGAGCA---GTGGGA---ATGGGAGCT---------
TTGTTCCTTGGGTTCTTGGGAGCA---GCAGGAAGCACTATG---GGCGCAGCGTCA---
ATAACGCTGACGGTACAA---GCCAGACAA---TTGTTG---TCTGGTATAGTGCAACAGCAAAAC---
AATTTG---CTGAGA---GCTATTGAGGCGCAGCAGCATCTGTTGCAACTCACAGTCTGGGGCATC
>JX863991
TTCTACTGTAATACAACAGAATTGTTTAATGGTACTTTGGAGGCTAATGATACTTGGAAGAATGGACTATAA
AGCCA--

CAGAACGCCAACATCACACTTCCATGCAGAATAAAACAAATTATAAACCTGTGGCAAGAAGTAGGAAAAGCAA
TGTATGCCCCGCCCATCAGTGGACAAATTAGATGTTCATCAAAT---
ATTACAGAGCTACTATTAACAAGAGATGGTGGTAATGGGAACGAGACC-------------------------
----

GACAATGAGACCGAGATCTTCAGACCTGGAGGAGGAGATATGAGGGAAAATTGGAGAAGTGAATTATATAAAT
ATAAAGTAGTAAGAGTTGAACCATTA---GGATTAGCACCCACCAAGGCAAAGAGGAGAGTGGTG--------
-------------------------------CAGAGAGAAAAAAGAGCA---GTGGGA---CTAGGAGCT---
------GTGTTCCTGGGGTTCTTGGGAGCA---GCAGGAAGCACTATG---GGCGCAGCGTCG---
TTGACGCTGACGGTACAG---GCCAGAATG---TTATTG---TCTGGTATAGTGCAACAGCAGAAC---
AATCTG---CTGAGA---GCTATTGAGGCGCAACAGCGTATGTTGCAACTCACAGTCTGGGGCATC
>JX863992
TTCTACTGTAATTCAACACAACTGTTTAATAGTAATAGCACTAAAGTAAATGAC-------------------
----

ACTAGAGATAATATCACACTCCCATGCAGAATAAAACAAATTGTAAACATGTGGCAGGAAGTAGGAAAAGCAA
TGTATGCCCCTCCCATCAGAGGACAAATTAAATGCTCATCAAAT---
ATTACAGGGCTGATATTAACAAGAGATGGTGGTAATGCGACAGAC----------------------------
----

GGGAACGAGACTTTTAGACCTGGAGGAGGAAATATGAAGGACAATTGGAGAAGTGAATTATATAAATATAAAG
TAGTAAAAATTGAACCAATA---GGAATAGCACCCACCAAGGCAAAGAGAAGAGTGGTG--------------
-------------------------CAAAGAGAAAGAAGAGCA---GTGGGA---ATAGGAGCT---------
GTGTTCCTTGGGTTCTTGGGAGCA---GCAGGAAGCACTATG---GGCGCAGCGTCA---
CTAACGCTGACGGTACAG---GCCAGACTA---TTATTG---TCTGGTATAGTGCAACAGCAGAGC---
AATCTG---CTGAGG---GCTATTGAGGCGCAACAACATCTGTTGCAACTCACAGTCTGGGGCATC
>JN562781
TTCTACTGTAATACATCAAAACTGTTTAATAGTACTTGGAATAGTACTAGTACTTGGAATAATACTGAA----
----

GAAAATAGCACACTCACACTCCCATGTAAAATAAGACAAATTATAAACATGTGGCAGAAAGTAGGAAAAGCAA
TGTATGCCCCTCCCATCAGTGGACGAATTAACTGCTTATCAAAT---
ATTACTGGGCTGCTATTAATAAGAGATGGTGGTGGTGGTGGTAAT----------------------------
----

GACACGACCGAGACCTTCAGACCTGGAGGAGGAGATATGAGGGACAATTGGAGAAGTGAGTTATATAAATACA
AAGTAATCAAAATTGAACCATTA---GGAATAGCACCCACCAAGGCAAAGAGAAGAGTGGTG-----------
----------------------------CAGAGAGAAAAAAGAGCA---GTGGGA---TTAGGAGCT------
---GTGTTCCTTGGGTTCTTGGGAGCA---GCAGGAAGCACTATG---GGCGCAGCGTCA---
CTAACGCTGACGGTACAG---GCCAGACAA---TTATTG---TCTGGTATAGTGCAACAGCAAAGC---
AATTTG---CTGAGA---GCTATTGAGGCGCAACAGCATCTGTTGCAACTCACAGTCTGGGGCATA
>JF320615
TTCTATTGTAATACAGCACAGCTGTTTAATAGCACTTGGCCTAAGAATGATACTAACAGTACAGATACTGAAG
AA---

AATGGAAATGGCACAATCACACTCCCATGTAGAATAAAACAAATTATAAATATGTGGCAGGAAGTAGGAAAAG
CAATATATGCCCCTCCCATCAGAGGACAAATTAGCTGTACATCAAAT---
ATTACAGGGCTGCTATTAACAAGAGATGGTGGTAATGAGAGCAGT----------------------------
----

GCTGACGAGATCTTCAGACCTGGAGGAGGAGATATGAGGGACAATTGGAGAAGTGAATTATATAAATATAAAG
TAGTAAAAATTGAACCATTA---GGAGTAGCACCCACTAAGGCAAAGAGAAGAGTGGTG--------------
-------------------------CAGAGAGAAAAAAGAGCA---GTGGGA---ATAGGAGCT---------
GTGTTCCTTGGGTTCTTGGGAGCA---GCAGGAAGCACTATG---GGCGCAGCGTCA---
ATAACGCTGACGGTACAG---GCCAGACAA---TTATTG---TCTGGTATAGTGCAGCAGCAAAAT---
AATTTG---CTAAGG---GCTATTGATGCACAGCAGAGTCTGTTGCAACTCACAGTCTGGGGCATC
>JF320613
TTCTACTGTGATACAACAAAACTGTTTAACAGTACTTGGCAGGATAACAGTACTGTGAATGCTAGTAGT----
----

GATGAGGATCCCATCATACTCCCCTGTAAAATAAGACAAATTATAAACATGTGGCAGGAAGTAGGAAAAGCAA
TGTATACCCCTCCCATCAGAGGACTAATTAAATGTTCATCAAAT---
ATTACAGGGCTACTATTAACAAGAGATGGGGGGGAAGATAACGAC----------------------------
----

ACTGAGATATTCAGACCTGGAGGAGGAAATATGAGGGACAATTGGAGAAGTGAATTATATAAATATAAAGTAG
TAAGAATTGAACCATTA---GGAGTAGCACCCACCAGGGCAAAGAGAAGAGTGGTG-----------------
----------------------CAGAGAGAAAAAAGAGCA---GTGGGG---CTGGGAGCG---------
TTGTTCCTTGGGTTCTTGGGAGCA---GCAGGAAGCACTATG---GGCGCAGCGTCA---
GTGACGCTGACGGTACAG---GCCAGACTA---TTATTG---TCTGGTATAGTGCAACAGCAGAAC---
AACTTG---CTGAGA---GCTATTGAGGCGCAACAGCATCTGTTGCAACTCACAGTCTGGGGCATT
>JF320048
TTCTACTGTAATACAACACAACTGTTTAACAGTACTTGGAATAGTACTGGAGAGTCTTGGAATAGGACTGGAG
GGTCAAAT---
-------------AACACG------
GGAAGAAATGGCACAATCACACTCCAATGCAGAATAAAACAAATTATAAACATGTGGCAGAAAGTAGGAAAAG
CAATATATGCCCCTCCCATCAAAGGACTAATTAGATGTTCATCAAAC---
ATTACAGGGCTGATATTAACAAGAGATGGTGGTAATAATAATAGC----------------------------
----

ACCATCTCCGAGATCTTCAGACCTGGAGGAGGAGATATAAGGGACAATTGGAGAAGTGAATTATATAAATACA
AAGTAGTAAAAATTGTACCAATA---GGGATAGCACCCACTAAGGCAAAGAGAAGAGTGGTG-----------
----------------------------CAGAGAGAAAAAAGGGCA---GTGGGA---ATAGGAGCT------
---ATGTTCCTTGGGTTCTTGGGAGCA---GCAGGAAGCACTATG---GGCGCAGCGTCA---
GTGACGCTGACGGTACAG---GCCAGACAA---TTATTG---TCTGGTATAGTGCAACAGCAAAAC---
AACTTG---CTGAGG---GCTGTGGAGGCGCAACAGCATCTGTTGCAACTCACAGTCTGGGGCATC
>JF320308
TTCTACTGTAATACCACAGGACTGTTTAATAGTACTTGGAACAATGAAACTAAAGAGTCAAAT----------
----

AACACTGAAACTATCATACTCCCATGCAGAATAAAACAAATTATAAACATGTGGCAGAAAGTAGGAAAAGCAA
TGTATGCCCCTCCCATCAGTGGACTAATTAGCTGTTCATCAAAT---
ATTACAGGGCTACTATTGACAAGAGATGGTGGTAATAACAATGGTAGTGAT----------------------
----

AACAACAGAAGTGAGATCTTCAGACCTGGAGGAGGAGATATGAGGGACAATTGGAGAAGTGAATTATATAAAT
ATAAAGTAGTAAAAATTGAACCATTA---GGAATAGCACCCACCAAGGCAAAGAGAAGAGTGGTG--------
-------------------------------CAGAGAGAAAAAAGAGCA---GTGGGA---TTAGGAGCT---
------TTGTTCCTTGGGTTCTTGTCAGCA---GCAGGAAGCACTATG---GGCGCAGCGTCA---
ATGACGCTGACGGTACAG---GCCAGACAA---TTATTG---TCTGGTATAGTGCAACAGCAGAAC---
AATTTG---CTGAGA---GCTATTGAGGCGCAACAGCATCTGTTGCAACTCACAGTCTGGGGCATC
>JF320003
TTCTACTGTGATACAACACAACTGTTTAATAGTACTTGGAATACTAGTAGTGCTTGGAATAGTACTGAAGGG-
----

TCAAATGACACTATCACACTCCCATGCAGAATAAAACAAATTATAAACATGTGGCAGGAAGTAGGAAAAGCAA
TGTATGCCCCTCCCATCAGAGGAGAAATTAAATGTTCATCAAAT---
ATTACAGGGCTGCTATTAACAAGGGATGGTGGCAGTAGTAACGAGACT-------------------------
----

AACGGGACTGAGATATTCAGGCCTGGAGGAGGAGATATGAGGGACAATTGGAGAAGTGAGTTATATAAATATA
AAGTAGTAAAAATTGAACCATTA---GGAGTAGCACCCACCAAGGCAAAGAGAAGAGTGGTG-----------
----------------------------CAAAGAGAAAAAAGAGCA---GTGGGA---CTAGGAGCT------
---TTGTTCCTTGGGTTCTTGGGAACA---GCAGGAAGCACTATG---GGCGCAGCGTCA---
CTGACGCTGACGGTACAG---GCCAGACAA---TTATTG---TCTGGTATAGTGCAACAGCAGAGC---
AACTTG---CTGAGA---GCTATTGAGGCGCAACAGCATATGTTGAAACTCACAGTCTGGGGCATC
>JF320097
TTCTACTGTAACTCAACACCACTGTTTAATAGTACTTGGAATGCTAATGGTACTTGGAATGGTACCGGAAATT
TA---

ACTACTGGAAACATCACACTCACATGTAGAATAAAACAAATTATAAACATGTGGCAGGAAGTAGGAAAAGCAA
TGTACGCCCCTCCCATAAGAGGACTAATTAGTTGTTCATCAAAT---
ATTACAGGACTGTTATTAACAAGAGATGGTGGTGAAGGTAATGACACC-------------------------
----

AACAATATCACCGAGGTCTTTAGACCTGGAGGAGGAGATATGAGGGACAATTGGAGAAGTGAATTATATAAAT
ATAAAGTAGTAAAAATTGAGCCATTA---GGAGTAGCACCCACCAAGGCAAAGAGAAGAGTGGTG--------
-------------------------------CAAAGAGAAAAAAGAGCA---GTGGGA---ATAGGAGCT---
------CTGTTCCTTGGGTTCTTGGGAGCA---GCAGGAAGCACTATG---GGCGCAGCGTCG---
ATGACGCTGACGGTACAA---GCCAGACAA---CTATTG---TCTGGTATAGTGCAACAGCAGAAC---
AATCTG---CTGAGA---GCTATTGAAGCGCAACAACATCTGTTGCAACTCACAGTCTGGGGCATC
>JF320038
TTCTACTGTAATACAACACAACTGTTTAATAGCACTTGGAATAATGGTACTAATAATGCTACTACAGACTCA-
----

AATGCCACTGTCATTACACTCCCATGTAGAATAAAACAAATTGTAAACATGTGGCAAGAAGTAGGAAAAGCAA
TGTATGCCCCTCCCATCAAAGGAAAAATCAGATGTTCATCAAAT---
ATTACAGGGCTGCTATTAACAAGAGATGGTGGCAGCGATGGGAGCTCC-------------------------
----

TCGAATGAGACTGAGACCTTCAGACCTGGAGGAGGAAATATGAGGGACAATTGGAGGAGTGAATTATATAAAT
ATAAAGTAGTAAGAGTTGAACCATTA---GGAGTAGCACCCACCAAGGCAAAGAGAAGAGTGGTG--------
-------------------------------CAGAGAGAAAAAAGAGCA---GTGGGA---ATAGGAGCT---
------GTGTTCCTTGGGTTCTTGGGAGCA---GCAGGAAGCACTATG---GGCGCAGCGTCA---
ATAACGCTGACGGTACAG---GCTAGACAA---TTATTG---TCTGGTATAGTGCAACAACAGAAC---
AATCTG---TTGAGG---GCTATTGAGGCGCAACAGCATCTGTTGCAACTCACAGTCTGGGGCATC
>JF320526
TTCTACTGTGATACAACAGAACTGTTTAATAGTACTTGGAATAATACAAGGTCAAATGACACTGCA-------
----

AGCAATAAGACTATCATACTCCAATGTAGAATAAGACAAATTATAAACAGATGGCAGGAAGTAGGAAAAGCAA
TGTATGCCCCTCCCATCAGCGGACAAATTAACTGTTCATCAAAT---
ATTACAGGGCTGATATTAACAAGAGATGGTGGAAACAATAGTCCAGGA-------------------------
----

AACGGGAGTGAGATCTTCAGACCTGGAGGGGGAGATATGAGGGACAATTGGAGAAGTGAATTATATAAATATA
AAGTAGTAAAAGTTGAGCCATTA---GGAGTAGCACCCACCAAGGCAAAGAGAAGAGTGGTG-----------
----------------------------CAGAGAGAAAAAAGAGCA---GTAGGAACAATGGGAGCT------
---TTGTTCCTTGGGTTCTTGGGAACA---GCAGGAAGCACTATG---GGCGCAGCGGCA---
ATGACGCTGACGGTACAG---GCCAGGCTA---TTATTG---TCTGGTATAGTGCAACAGCAGAAC---
AATTTG---CTGAGG---GCTATTGAGGCGCAACAGCATCTGTTGCAACTCACAGTCTGGGGCATC
>JF320160
TTCTACTGTAATACATCACAACTGTTTAATAGTACTTGGCCTTGGAATGCTACTGAAGGAGTAAATAGC----
----

ACTAATGATAATATCACACTCCCATGCAGAATAAAACAAATTGTAAACATGTGGCAGGAAGTAGGAAAAGCAA
TGTATGCCCCTCCTATCAGCGGAATAATTAGGTGTTCATCAAAT---
ATTACAGGAATGATATTAACAAGAGATGGGGGAGCGGGGAACGGGAGC-------------------------
----

AATAACGAGACTGAGATCTTCAGACCTGGAGGAGGAAATATGAAGGACAATTGGAGAAGTGAATTATATAAAT
ATAAAGTAGTAAAAATTGAACCATTA---GGAATAGCACCCACCAGGGCAAAGAGAAGAGTGGTG--------
-------------------------------CAGAGAGAAAAAAGAGCA---ATAACA---CTAGGAGCT---
------TTTTTCCTTGGGTTCTTGGGAGCA---GCAGGAAGCACTATG---GGCGCAGCGTCA---
ATAACGCTGACGGTACAG---GCCAGACTA---TTACTG---TCTGGTATAGTGCAGCAGCAGAGC---
AATCTG---CTGAGG---GCTATTGAGGCGCAACAGCATATGTTGCAACTCACAGTCTGGGGCATC
>JF320169
TTCTATTGTAACACAACAGCACTGTTTAATAGTACTTGGGGGGCTAACAGTACTTGGGAGATTACA-------
----

AATGACACTGAAATCATACTCCCATGCAGAATAAGACAAATCATAAACATGTGGCAGGAAGTAGGAAAAGCAA
TGTATGCCCCTCCCATTAGTGGAAACATTAGTTGCTCATCAAAT---
ATTACAGGGTTACTATTAACAAGAGATGGGGGTAATGGAAATGGT----------------------------
----

ACCAAAAACGAGACCTTCAGACCTACAGGAGGGGATATGAGGGACAATTGGAGAAGTGAATTGTATAAATATA
AAGTAGTAAGAATTGAACCATTA---GGAGTAGCACCCACCAAGGCAAAGAGAAGAGTGGTG-----------
----------------------------CAGAGAGAAAAAAGAGCA---GTGGGA---CTAGGAGCT------
---ATGTTCCTTGGGTTCTTGGGAGCA---GCAGGAAGCACTATG---GGCGCAGCGTCG---
GTAACGCTGACGGTACAG---GCCAGACAA---TTATTG---TCTGGTATAGTGCAGCAGCAGAGC---
AATCTG---CTGAGA---GCTATTGAGGCGCAACAGCATATGTTGCAACTCACAGTCTGGGGCATC
>JF320053
TTCTACTGTAATACAGCACAACTGTTCAATAGTACCTGGATCAATGGTACTGAAAGGTCAAATACCACTGAA-
----

GGAAATACCACAATCACACTCCCATGCAGAATAAAACAAATAGTAAATATGTGGCAGGAAGTAGGAAAAGCCA
TGTATGCCCCTCCCATCAGAGGAAAAATTAACTGCTTCTCAAAT---
ATCACAGGGCTGCTATTAACAAGAGATGGTGGCTCTGATAACAACACCACT----------------------
----

GGCAACAACACCGAGATCTTCAGACCTGGAGGAGGAGATATGAGGGACAATTGGAGAAGTGAATTATATAAAT
ATAAAGTAGTAAAAATCGAACCATTA---GGAGTAGCACCTACCAGGGCAAAGAGAAGAGTGGTG--------
-------------------------------CAGAGAGAAAAAAGAGCA---GCAGGA---ATAGGAGCT---
------TTGTTCCTTGGGTTCTTGGGAGCA---GCAGGAAGCACTATG---GGCGCAGCGTCA---
GTGACGCTGACGGTACAG---GCCAGACAA---TTATTG---TCTGGTATAGTGCAGCAGCAAAAC---
AATTTG---TTGAAG---GCTATTGAGGCGCAACAGCATCTGTTGCAACTCACAGTCTGGGGCATC
>JF320356
TTCTACTGTAATACAACAATACTGTTTAATAGCACTTGGAATGATACTAAAGGGACAAATAGC----------
----

ACAAATGACACTATCACACTCCCATGCAGAATAAAACAAATTATAAACATGTGGCAGAGAGTAGGAAAAGCAA
TATATGCCCCTCCCATCAGCGGACCAATTAGGTGTTCATCAAAT---
ATTACAGGGCTGCTATTAACAAGAGATGGTAGTACTAACAACAACAGC-------------------------
----

ACTAAGAATGAGACCTTCAGACCTGGAGGAGGAGACATGAGGGACAATTGGAGAAGTGAATTATATAAATATA
AAGTAGTAAAAGTTGAACCAGTA---GGAATAGCACCCACCAAGGCAAAGAGAAGAGTGGTG-----------
----------------------------CAGAGAGAAAAAAGAGCA---GTGGGAACACTAGGAGCT------
---GTGTTCCTTGGGTTCTTGGGAGCA---GCAGGAAGCACTATG---GGCGCAGCGTCA---
ATGACGCTGACGGTACAG---GCCAGACAA---CTATTG---TCTGGTATAGTGCAACAGCAGAAC---
AATCTG---CTGAAG---GCTATTGAGGCGCAACAGCACATGTTGCAACTCACAGTCTGGGGCATT
>JF320592
TTCTACTGTAATACAACAAAGCTGTTTAATAGTACTTGGGTTAATGGTACTAGGAATGATACAGGGTCAAAT-
----

GACACTGAAACCATAATACTCCCATGCAGAATAAAACAAATTATAAACATGTGGCAGAAAGTAGGAAAAGCAA
TGTATGCCCCTCCCATCAAAGGACAAATTAGCTGTTCATCAAAC---
ATTACAGGGCTGCTATTAACAAGAGATGGATCTCTAAAGAATGAG----------------------------
----

ACCACCTTGACCTTCAGACCTGGAGGAGGAAATATGAGGGACAATTGGAGAAGTGAATTATATAAATATAAAG
TAGTAAAAATTGAACCATTA---GGGGTAGCACCCTCCAAGGCAAAGAGACGGGTGGTG--------------
-------------------------CAGAGAGAAAAAAGAGCA---GCGGGA---CTAGGAGCT---------
ATGTTCCTTGGGTTCTTGGGAGCA---GCAGGAAGCACTATG---GGCGCAGCGTCA---
GTGACGCTGACGGTACAG---GCCAGACTA---TTATTG---TCTGGTATAGTGCAACAGCAGAAC---
AATCTG---CTGAGA---GCTATTGAGGCGCAACAGCATCTGTTGCAGCTCACGGTCTGGGGCATC
>KT124791
TTCTACTGTAATACAACACAACTGTTTAATAGTACTTGGAATAGTACTAGAGAGAAAAATAGCACA-------
----

AGAAATGACACTATCACACTCCCATGCAGAATAAAACAAATTATAAATATGTGGCAAAAAGTAGGAAAAGCAA
TGTATGCCCCTCCCATCAGGGGACAAATTAGATGTTCATCAAAT---
ATTACAGGGTTGCTATTAACAAGAGATGGTGGTAGTAACCAAAAT----------------------------
----

AGGACTGAGATCTTTAGACCTGCAGGAGGAAATATGAAGGACAATTGGAGAAGTGAATTATATAAATATAAAG
TAGTAAGAATTGAACCATTA---GGAGTAGCACCCACTGGGGCAAAGAGAAGAGTGGTG--------------
--------------------------CAGAGAGAAAAAGAGCA---GTGGGA---ATAGGAGCT---------
GTGTTCCTTGGGTTCTTGGGAGCA---GCAGGAAGCACTATG---GGCGCAGCGTCA---
CTGACGCTGACGGTACAG---GCCAGACTC---TTATTG---TCTGGTATAGTGCAACAGCAGAAC---
AATCTG---CTGAGG---GCTATTGAGGCGCAACAGCATCTGTTGCAACTCACAGTCTGGGGCATC
>KR423026
TTCTATTGTAATACAGCAAGATTGTTTAATAGCTCTTGGAATGATACCGAAGGGTCAATCAATGAGACAAAC-
----

TCAGAAAACATCATCACACTCCCATGCAGAATAAAACAAATTGTAAACATGTGGCAGGAAGTAGGAAAAGCAA
TGTATGCCCCTCCCATCAGGGGAATAATTAACTGTTCATCAACT---
ATTACAGGGCTGTTATTAACAAGAGATGGTGGTAATGGTAAGAATGGGAGC----------------------
----

AATGAGACCAATGAGATTTTCAGACCTGGAGGAGGAGATATGAGGGACAATTGGAGAAGTGAATTATATAAAT
ATAAAATAGTAAAAATTGAACCATTA---GGACTAGCACCCACCCCGGCAAAGAGAAGAGTGGTG--------
--------------------------------CAGAGAGAAAAAGAGCA---GTGGGA---CTAGGAGCA---
------TTGTTCCTTGGGTTCTTGGGAGCA---GCAGGAAGCACTATG---GGCGCAGCGTCA---
ATGACGCTGACGGTACAG---GCCAGACAA---TTATTG---TCTGGTATAGTGCAACAGCAGAAC---
AATTTG---CTGAGA---GCTATTGAGGCGCAACAGCATATGTTGCAACTCACAGTCTGGGGCATA
>GU331039
TTCTACTGTAATACAACAGAACTGTTTAATAGTACTTGGAATAATGGTAGCAGTACTTGGACTAGCAATGATA
CTAAA--

GGGTTAAATAACACTATCACACTCCGATGCAGAATAAAACAAATTATAAACATGTGGCAGGAAGTGGGAAAAG
CAATGTATGCCCCTCCCATCAGAGGACAAATTAGATGTTCATCAAAT---
ATTACAGGGCTACTATTAACAAGAGATGGTGGTAATAATAAGACCGAA-------------------------
----

AACAAAACGGAGATCTTCAGACCTGGAGGAGGAGATATGAGGGACAATTGGAGAAGTGAATTATATAAATATA
AAGTGGTACAAATTGAACCAATA---GGAGTAGCACCCACCAAGGCAAAGAGAAGAGTGGTG-----------
-----------------------------CAAAGAGAAAAAGAGCA---GTAACA---CTGGGAGCT------
---ATGTTCCTTGGGTTCTTGGGAGCA---GCAGGAAGCACTATG---GGCGCAGCGTCA---
ATGGCGCTGACGGTACAG---GCCAGACAA---TTATTG---TCTGGTATAGTGCAACAGCAAAAC---
AATTTG---CTGAGA---GCTATTGAGGCGCAACAGCACCTGTTGCAACTCACAGTCTGGGGCATC
>GU331147
TTCTACTGTAATTCAACACAACTGTTTAATAGTATCTGGACTAAAAATGATAATGGTATCTGGACTAAAAATG
AGACTAATGGT---AAT---
TGGACTAGAAGCGGGAGCACA---------------
AATGACAATGACACAATCATACTCCCTTGCAGAATAAGACAAATTATAAACATGTGGCAGGAAGTAGGAAAAG
CAATGTACGCTCCTCCCATTAGAGGAAACATTAGCTGTTCATCAAAT---
ATTACAGGGCTGTTACTAACAAGAGATGGAGGTAACAATAATGAT----------------------------
----

AGCGCAAATGAGACGTTCAGACCTGGAGGAGGAAATATGAAGGACAATTGGAGAAGTGAATTATATAAATATA
AAGTAGTAGAAATTGAGCCATTA---GGACTAGCACCCACCAGGGCAAAAAGAAGAGTGGTG-----------
-----------------------------CAGAGAGAAAAAGAGCA---GCC------CTAGGAGCT------
---TTGTTCCTTGGGTTCTTAGGAGCT---GCAGGAAGCACTATG---GGCGCAGCGTCA---
GTGACGCTGACGGTACAA---GCCAGACTA---TTATTG---ACTGGTATAGTGCAGCAGCAGAAC---
AATCTG---CTGAAG---GCTATTGAGGCGCAACAACATCTGTTGCAACTCACAGTCTGGGGCATC
>KM353701
TTCTACTGCAATACAACAAAACTATTTAGCAGTACCTGGAGTATAAATGAAACTATCTGG-------------
----

CATCTAAATGAAACTACAAAT------------
AACACTAATGACACTATCACACTCCCATGCAAAATAAAACAATTTATAAACATGTGGCAGCAAGTAGGAAAAG
CAATGTATGCCCCTCCTATCAGAGGACAAATTAGATGTAAATCAGAT---
ATTACAGGGCTGATATTAATGAGAGATGGTGGTGAA-------------------------------------
----
ACTAACAGGGGCAACGAGACCTTC---------
AGCAATGAGACCTTCAGACCTGGGGGAGGAAATATGAGGGACAATTGGAGAAGTGAATTATATAAATATAAAG
TAGTAAAGATTGAGCCATTA---GGACTGGCACCCACCGAGGCAAAGAGAAGAGTGGTG--------------
-------------------------CAGAGAGAAAAAAGAGCA---GTGGAA---ATAGGAGCT---------
ATGTTCCTTGGGTTCTTGGGAGCA---GCAGGAAGCACTATG---GGCGCAGCATCA---
CTAACGCTGACGGTACAG---GCCAGAATG---TTATTG---TCTGGTATAGTGCAACAGCAGAGC---
AATTTG---CTGAAG---GCTATTGAGGCGCAACAACATCTGTTGCAACTCACAGTCTGGGGCATC
>JN687749
TTCTACTGTGATACAACACCACTGTTTAATAGTACGTGGCCTTGGAATAGTACTTGGAATGATACAAATACA-
----

AATAATACCAATATCACACTCCAGTGCAGAATAAAACAAATTATAAACATGTGGCAGGAAGTAGGAAAAGCAA
TGTATGCCCCTCCCATCAAAGGACAAATTAGGTGCTCATCAATT---
ATTACAGGGCTGCTGTTAACAAGAGATGGTGGTAATAGCAGCGAA----------------------------
----

AATAATACTGAGATCTTCAGACCTGGAGGGAGAGATATGAGGGACAATTGGAGAAGTGAATTATATAAATATA
AAGTTGTAAAACTTGAACCATTA---GGAGTAGCACCCACCAAGGCAAAGAGAAGAGTGGTG-----------
----------------------------CAAAGAGAAAAAAGAGCA---GTGAAA---ATAGGAGCT------
---CTGTTCCTTGGATTCTTGGGAGCA---GCAGGAAGCACTATG---GGCGCAGCGTCA---
GTGACGCTGACGGTACAG---GCCAGACTA---TTATTG---TCTGGTATAGTGCAACAGCAGAAC---
AATCTG---CTGAGG---GCTATTGAGGCGCAACAACATATGTTGCAACTCACAGTCTGGGGCATC
>JN687758
TTCTACTGTAATACAACACAACTATTTGATAGTAATTGGACTCTATCTGATATTACTAAAGGGTCAAATACCA
CTGAA--

GGCAACAGCACACACATCACACTCCCATGCAGAATAAAACAAATTATAAACATGTGGCAAGAAGTAGGAAAAG
CAATGTATGCCCCTCCTATCAGAGGAAACATTACTTGCATATCAAAT---
ATTACAGGGTTGTTACTAGTAAGAGATGGTGGTAATAATAATGAG----------------------------
----

ACAGAGATCTTTAGACCTGGAGGAGGAGATATGAGGGACAATTGGAGAAGTGAATTGTATAAATATAAAGTAA
TAAAAATTGAACCATTA---GGAATAGCACCCACCAAGGCAAAAAGAAGAGTGGTG-----------------
----------------------CAGAGAGAAAAAAGAGCA---GCA------TTAGGAGCT---------
TTGTTCCTTGGGTTCTTGGGAGCA---GCAGGAAGCACTATG---GGCGCAGCGTCA---
CTGACGCTGACGGTACAG---GCCAGGCTA---TTATTG---TCTGGTATAGTGCAACAGCAGAAC---
AATCTG---CTGAGG---GCTATTGAGGCGCAACAGCATCTGTTGCAACTCACAGTCTGGGGCATC
>HQ908116
TTCTACTGTAATACAACAGAACTGTTTAATAGTACTTGGAATGCTAATGAAGGATCAAATAAAACAGGATCA-
----

AATGACACTACAATCATACTCCCATGTAGACTAAGACAAATTGTAAACATGTGGCAGGAAGTAGGAAAAGCAA
TGTATGCTCCTCCCATTAGAGGACAGATTAGATGTTCATCAAAT---
ATTACAGGGCTGCTATTAACAAGAGATGGTGGTATTACTAATACTACCAAC----------------------
----

AAGACTAAGACTGAGACCTTCAGACCTGCAGGAGGAGATATGAGGGACAATTGGAGAAGTGAATTATATAAAT
ATAAAGTAGTAAAAATTGAACCATTA---GGAATAGCACCTTCCAGGGCAAAGAGAAGAGTGGTG--------
--------------------------------CAAAGAGAAAAAAGAGCA---GTGGGA---ATAGGAGCT---
------CTGTTCCTTGGGTTCTTGGGAGCA---GCAGGAAGCACTATG---GGCGCAGCGTCA---
ATGACGCTGACGGTACAG---GCCAGACTA---TTATTG---TCTGGTATAGTGCAACAGCAGAAC---
AACTTG---CTGAGA---GCTATTGAGGCGCAACAGCATTTGTTGCAACTCACAGTCTGGGGCATC
>JQ251004
TTCTACTGTAATACAACACAACTATTTAGTAGTACTTGGCTGCCTAATAGTACTCGGAATGATACT-------
----

GATAATAGCCCTATCACACTCCCATGCAGAATAAAACAAATTGTAAACATGTGGCAGGAAGTAGGAAAAGCAA
TGTATGCCCCTCCCATCAGAGGAAACATTAGTTGCTCATCAAAT---
ATTACAGGGCTACTATTAACAAGAGATGGGGGTAATGATAATAAT----------------------------
----

AATACCGAGATCTTCAGACCTGGAGGAGGAGATATGAGGGACAATTGGAGAAGTGAATTATACAAATATAAAG
TAGTAAAAATTGAACCATTA---GGAATAGCACCCACCAAGGCAAAGAGAAGAGTGGTG--------------
-------------------------CAGAGAGAAAAAAGAGCA---GTGGGA---ATAGGAGCT---------
GTGTTCCTTGGGTTCTTGGGAGCA---GCAGGAAGCACTATG---GGCGCAGCGTCA---
ATAACGCTGACGGTACAA---GCCAGACAA---TTATTG---TCTGGTATAGTGCAACAGCAGAAC---
AATTTG---CTGAGG---GCTATTGAGGCGCAACAGCATCTGTTGCAACTCACAGTCTGGGGCATC
>JN944897
TTCTACTGCAATACAACACAACTGTTTAATAGTACCTGGAATCTTAATAGTACCTGGAATGGAACTAAAAATT
TAAATAAC--
--------------------ACT---
ACAAGAAATGACACAATCATACTCCCATGCAGAATAAGACAAATTATAAACTTGTGGCAAAAAGTAGGAAAAG
CAATGTATGCCCCTCCCATCAGAGGACAAATTAATTGTACATCAAAT---
ATCACAGGGCTGCTATTAACAAGAGATGGTGGAAATACAAGTGAGACC-------------------------
----

AACACCACTGAGGTCTTCAGACCTGGAGGAGGAAACATGAGGGACAATTGGAGAAGTGAATTATATAAATATA
AAGTAGTAGAAATTGAACCAATA---GGGATAGCACCCACCAAGGCAAAGAGAAGAGTGGTG-----------
----------------------------CAGAGAGAAAAAAGAGCA---GTGGGA---ATAGGAGCT------
---GTGTTCCTTGGGTTCTTGGGAGCA---GCAGGAAGCACTATG---GGCGCAGCGTCG---
GTAGCGCTGACGGTACAG---GCCAGACAG---TTATTG---TCTGGTATAGTGCAACAGCAGAAC---
AACTTG---CTGAGG---GCTATTGAGGCGCAACAACATATGTTGAAACTCACGGTCTGGGGCATT
>JN944907
TTCTACTGTGATACAAAACAACTGTTTAATAGTACTTGGAATGCTACTAAGGCAAATGGCACTACA-------
----

GGAAATGACACAATCATACTCCCATGCAGAATAAAGCAAATTATAAACATGTGGCAGAAAGTAGGAAAAGCAA
TGTATGCCCCTCCCATCAAAGGAAAAATTAGTTGCTCATCAAAT---
ATCACAGGGCTGCTATTAACAAGAGATGGAGGAGGTGGGGGACAG----------------------------
----

AATGAGACCTTCAGACCTGCAGGGGGAGATATGAGGGACAATTGGAGAAGTGAATTATATAAATATAAGGTAG
TAAAAATTGAACCATTA---GGAGTAGCACCCACCAGGGCAAAGAGAAGAGTGGTG-----------------
----------------------CAGAGAGAAAAAAGAGGA---GTAGGA---ATAGGAGCT---------
ATGGTCCTTGGGTTCTTGGGAGCA---GCAGGAAGCACTATG---GGCGCAGCATCA---
ATGATGCTGACGGTACAA---GCCAGACAA---TTGTTG---TCTGGTATAGTGCAACAGCAGAAC---
AATCTG---CTGAGA---GCTATTGAGGCGCAACAACATATGTTGCAACTCACAGTCTGGGGCATC
>JN944909
TTCTACTGCGATTCAACAGCGCTGTTTAATAGTACATGGAGGAGAAATAACACTTGGACTGGT----------
----

ACTACAGGAAATATCACACTCCAATGCAGAATAAAACAAATTATAAACATGTGGCAGAAAGTAGGAAAAGCAA
TGTATGCCCCTCCCATCAGAGGATACATTAACTGTTCATCAAAT---
ATTACAGGACTGATATTAACAAGAGATGGTGGTAATAATGATAGT----------------------------
----

GAAACCGAGATCTTCAGGCCTGGAGGAGGGAATATGAAGGACAATTGGAGAAGTGAATTATATAAATATAAAG
TAATAAAAATTGAACCATTA---GGAATAGCACCCACCAAGGCAAAGAGAAGAGTGGTG--------------
-------------------------CAAAGAGAAAAAAGAGCA---ATAGGA---ATAGGAGCT---------
CTTCTCCTTGGGTTCTTAGGAGCA---GCAGGAAGCACTATG---GGCGCAGCGTCA---
GTGACGCTGACGGTACAA---GCCAGACAA---TTATTG---TCTGGTATAGTGCAACAGCAGAAC---
AATCTG---CTGAGG---GCTATTGAGGCGCAACAGCATCTGTTGCAACTCACAGTCTGGGGCATC
>FJ469691
TTCTACTGTAATACAACACAACTGTTTAATAGTACTTGGACTGGGAATAATACTACAAATAAC----------
----

ATTTCAGGATATATCACACTCCCATGCAGAATAAAACAAATTATAAACATGTGGCAAGAAGTAGGAAAAGCAA
TGTATGCCCCTCCCATCAGTGGACAAATCAGGTGTTCATCAAAT---
ATTACAGGGCTACTATTAACAAGAGATGGTGGTAATAGTAATAACACC-------------------------
----

AACGGGACGACCGAGGTTTTCAGACCTATAGGAGGAAATATGAGGGACAATTGGAGAAGTGAATTATATAAAT
ATAAAGTAGTAAAAATTGAACCATTA---GGAGTAGCACCCACCACGGCAAAGAGAAGAGTGGTG--------
-------------------------------CAAAGAGAAAAAAGAGCA---GTGGGA---ATAGGAGCT---
------TTGTTCCTTGGGTTCTTGGGAGCA---GCAGGAAGCACTATG---GGCGCAGCGTCA---
TTGACGCTGACGGTACAG---GCCAGGCTA---TTATTG---TCTGGTATAGTGCAACAGCAGAAC---
AATCTG---CTGAGA---GCTATTGAGGCGCAACAACATCTGTTGCAACTCACAGTCTGGGGCATC
>FJ469696
TTCTACTGTAATACAACACAATTGTTTAATAGTACTTGGAATAGTACTGAAGGGTCAAATAAGACTGGAGGA-
----

AATGACACAACAATCACACTCCCATGCAGAATAAAACAAATTATAAACATGTGGCAGGAAGTAGGAAAAGCAA
TATATGCCCCTCCCATCAGAGGACTAATTAGATGTTCATCAAAT---
ATTACAGGGTTGCTAATAACAAGAGATGGTGGTAATAACAATAAC----------------------------
----

AACAAGACAGAGACCTTCAGACCTGGAGGAGGAGATATGAGGGACAATTGGAGAAGTGAATTATATAAATATA
AAGTAGTAAAAATTGAACCATTA---GGAGTAGCACCAACCAAGGCAAAGAGGAGAGTGGTG-----------
----------------------------CAGAGAGAAAAAAGAGCA---ATGGGA---ATGGGAGCT------
---TTGTTCCTTGGGTTCTTGGCATCA---GCAGGAAGCACTATG---GGCGCAGCGTCA---
ATAACGCTGACGGTACAG---GCCAGACAA---TTATTG---TCTGGTATAGTGCAACAGCAGAAC---
AATTTG---CTGAGA---GCTATTGAGGCGCAACAGCATCTGTTGCAACTCACAGTCTGGGGCATC
>FJ469700
TTCTACTGTGACTCAACACAACTGTTTAATAGTTCCTGGAATGGTACTGGAGGGTCAAGTAAC----------
----

ACTGCAGAGAATATCACACTCCATTGTAGAATAAGACAAATTGTAAACTTGTGGCAAGAAGTGGGAAGAGCAA
TGTATGCCCCTCCTATCAGAGGACAAATTAGCTGCTCATCAAAT---
ATTACAGGACTGATATTAACAAGAGATGGAGGTAATAACACTGAAAGT-------------------------
----

AATAACACCGAGACCTTCAGACCTGGAGGAGGAAATATGAGGGATAATTGGAGAAGTGAATTATATAAATATA
AAGTAGTAAAAATTGAACCAATA---GGAGTAGCACCCACCAAGGCAAAGAGAAGAGTGGTG-----------
----------------------------CAGAGAGAAAAAAGAGCG---GTGGGA---TTCGGAGCT------
---ATGTTCCTTGGGTTCTTGGGAGCA---GCAGGAAGCACTATG---GGCGCAGCGTCG---
ATAACGCTGACGGTACAG---GCCAGACAA---TTATTG---TCTGGTATAGTGCAACAGCAGAAC---
AATTTG---CTGAGG---GCTATTGAGGCGCAACAGCATCTGTTGCAACTCACGGTCTGGGGCATC
>FJ469708
TTCTATTGTAATACAACACAACTGTTTAATAGTACTTGGAACAGTAATGAAGAGACAAAGAATGTCACA----
----

GGAAATGGAACTATCACACTCCCATGCAGAATAAGACAAATTATAAACATGTGGCAACAAGTAGGAAAAGCAA
TGTATGCCCCTCCCATTAGCGGAAAAATTAGCTGTTCATCAAAC---
ATTACAGGGCTACTATTAACAAGAGATGGGGGTGAAAACACGAGC----------------------------
----

GAGACCGAGATTTTTAGACCTGCAGGAGGAAATATGAAGGACAATTGGAGAAGTGAATTATACAAATATAAAG
TAGTAAAAATTGAACCATTA---GGAATAGCACCCACCAAGGCAAAGCGAAGAGTGGTG--------------
-------------------------CAAAGAGAAAAAAGAGCA---ATAGGA---ATAGGAGCT---------
ATGTTCCTTGGGTTCTTGGGAGCA---GCAGGAAGCACTATG---GGCGCAGCGTCA---
CTGACGCTGACGGTACAG---GCCAGACTA---TTATTG---TCTGGTATAGTGCAACAGCAGAAC---
AATCTG---CTGAGG---GCTATTGAGGCGCAGCAGCATCTGTTGCAACTCACAGTCTGGGGCATC
>FJ469709
TTCTACTGTGTTACAACAAAACTGTTTAATAGTACTTGGACGTTTAATAGTACTAATGGTACTTGGACGTTGA
ATAGTACTTGG--

AATAGCACTGAAGGGCTA---------
AACAACAATGACACTATCATACTCCCATGCAGAATAAAACAAATTATAAACATGTGGCAAACAGTAGGAAAAG
CAATGTATGCCCCTCCAATCAGAGGACCAATTAACTGTACATCAAAT---
ATTACAGGGTTGCTATTAACAAGAGATGGTGGTAAGGAGACCAAC---------------------------
----

ACCACCACCGAGACCTTCAGACCTGGAGGAGGAGACATGAGGGACAATTGGAGAAGTGAATTATATAAATATA
AAGTAGTAAAAATTGAGCCATTA---GGAATAGCACCCACCAAGGCAAGGAGAAGAGTGGTG----------
---------------------------CAAAGAGAAAAAAGAGCA---GTGGGA---ATAGGAGCT------
---GTGTTCCTTGGGTTCTTGGGAGCA---GCAGGAAGCACTATG---GGCGCAGCGTCA---
CTGACGCTGACGGTACAG---GCCAGACTA---TTGTTG---TCTGGTATAGTGCAGCAGCAGAAC---
AATCTG---CTGAGG---GCTCTTGAGGCGCAACAACATCTGTTGCAACTCACAGTCTGGGGCATC
>JQ250880
TTCTACTGTAATACAACACCATTGTTTAATGGTAGTAGTTGGAATCTAAATAAGACTAAAGAAAATACAACTA
ATCTA---

GAAAATGGCACAATCACCCTCCAATGCAGAATAAAACAATTTGTAAACATGTGGCAGAAAGTAGGAAAAGCAA
TGTATGCTCCTCCCATCAGAGGACGGATTGAATGTTCATCAAAT---
ATTACAGGGCTGATATTAACAAGAGATGGGGACATGAATAGTACA---------------------------
----

GGGAACGAGACCTTCAGACCTGGAGGAGGAGATATGAGGGACAATTGGAGAAGTGAATTATACAAATACAAAG
TAGTAAAAATTGAGCCATTA---GGAGTAGCACCCACCAAGGCAAAGAGAAGAGTGGTG-------------
------------------------CAGAGAGAAAAAAGAGCA---GTGGGAGCACTAGGAGCT---------
ATGTTCCTTGGGTTCTTGGGAGCA---GCAGGAAGCACTATG---GGCGCAGCGTCA---
GTGACGCTGACGGTACAG---GCCAGACTA---TTATTG---TCTGGTATAGTGCAACAGCAAAAT---
AATTTG---CTGAAG---GCTATTGAGGCGCAACAGCGTCTGTTGCAACTCACAGTCTGGGGCATC
>HQ217266
TTCTACTGTAACACAACACAATTGTTTAATAGTACTTGGAATAAAAATAGTACTATTGAGGATAGA------
----

AATGATGCTAATATCACACTCCCATGCAGGATAAGGCAAATTGTAAACATGTGGCAGCGAGTAGGAAAAGCAA
TGTATGCTCCTCCCATCAGCGGAATAATTAGATGTTCATCAAAT---
ATTACTGGGCTGCTATTAACAAGAGATGGAGGTAGTAATGCTACT---------------------------
----

ACCGAGACCTTCAGACCTGGAGGAGGAGATATGAGGGATAATTGGAGAAGTGAATTATATAAATACAAAGTAG
TAAAAATTGAACCATTA---GGAATAGCACCCACCAAGGCAAAGAGAAGAGTGGTG----------------
---------------------CAGAGAGAAAAAAGAGCG---GCAATAGGACTAGGAGCT---------
TTGTTCCTTGGGTTCTTGGGAGCA---GCAGGAAGCACTATG---GGCGCAGCGTCA---
ATAACGCTGACGGTACAG---GCCAGACTA---TTATTG---TCTGGTATAGTGCAGCAGCAGAAC---
AATTTG---CTGAGG---GCTATTGAGGCGCAACACCATCTGTTGCAACTCACAGTCTGGGGCATC
>JQ403068
TTCTACTGTGATTCATCAAAACTGTTTAATAGTACTTGGAATGTAACTAGTTGGAATGTAACTGGTTGGAATG
GTACTGAA--

GAGTCAAATGGCACTATCACACTCCCATGCAGAATAAAACAGATTATAAACATGTGGCAAGAAGTAGGAAAAG
CAATGTATGCCCCTCCCATCAGAGGACTAATTAGATGTTCAACAAAT---
ATTACAGGGCTGATATTAACAAGAGATGGTGGTAACGAGAACAAT---------------------------
----

AACAATAGTGAGGTCTTTAGACCTGGAGGAGGAGATATGAGGGACAATTGGAGAAGTGAATTATATAAATATA
AAGTAGTAAAAATTGAACCATTA---GGAGTAGCACCCACCAGGGCAAAAAGAAGAGTGGTG----------
---------------------------CAGAGAGAAAAAAGAGCA---GTGGGA---CTAGGAGCA------
---CTGTTCCTTGGGTTCTTGGGAGCA---GCAGGAAGCACTATG---GGCGCAGCGTCA---
ATGACGCTGACGGTACAA---GCCAGACAA---TTATTG---TCTGGTATAGTGCAACAGCAGAAC---
AATTTG---CTGAGG---GCTATTGAGGCGCAACAACATTTGTTGCAACTCACAGTCTGGGGCATC
>JQ403095
TTCTACTGTAATTCAACGCAACTGTTTAATAGTACTTGGGATATTAATAATACTAGTAGGGCAAAT------
----

AGCACTGGAAATATCACACTCCCATGCAGAATAAGACAAATTATAAACATGTGGCAGGAAGTAGGAAAAGCAA
TGTATGCCCCTCCCATCGCGGGACAAATTAGATGTACATCAAAT---
ATTACAGGACTGTTATTAACAAGAGATGGTGGTAATGACACTACC----------------------------
----

ACCGAGATCTTCAGACCTGGAGGAGGAAATATGAAGGACAATTGGAGAAGTGAATTATATAAATACAAAGTAG
TAAAAATTGAACCATTA---GGAATAGCACCCACCAAGGCAAAGAGAAGAGTGGTG-----------------
----------------------CAGAGAGAAAAAAGAGCA---ATAGGA---CTGGGAGCT---------
ATGTTCCTTGGGTTCTTGGGAGCA---GCAGGAAGCACCATG---GGCGCAGCGTCA---
GTGACGCTGACGGTACAG---GCCAGACAA---TTATTG---TTTGGTATAGTGCAACAGCAGAAC---
AATCTG---CTGAGG---GCTATTGAGGCGCAACAGCATATGTTGCAACTCACAGTCTGGGGCATC
>KM259364
TTCTACTGTGATACAACACCACTATTTAATAGTATTTGGAATACAACACAAATATTTAATGGT----------
--ACT---
TGGAATGATACTGTAGGGTCT---------------
GGAGAAAATGACACAATCATACTCCAATGCAGAATAAAACAAATTATAAACATGTGGCAAGAAGTAGGAAAAG
CAATGTATGCCCCTCCCATCAGAGGACAAATTAGATGTGAATCAAAT---
ATTACAGGGCTGCTATTAACAAGAGATGGTGGTACT-------------------------------------
----
GGTGAGAACAACACTGGTGAGAAC---------
AACACTGAGATCTTCAGACCTGGAGGAGGAGATATGAGGGACAATTGGAGAAGTGAATTATATAAATATAAAG
TAGTAAAAATTGAACCATTA---GGAGTAGCACCTACTAAGGCAAAGAGGAGAGTGGTG--------------
-------------------------CAGAGAGAAAAAAGAGCA---GTGGGA---TTAGGAGCT---------
GTGCTCTTTGGGTTCTTGGGAGCA---GCAGGAAGCACTATG---GGCGCAGCGTCA---
ATAACGCTGACGGTACAG---GCCAGACAA---TTATTG---TCTGGTATAGTGCAACAGCAAAAC---
AATTTG---CTGAAG---GCTATTGAGGCGCAACAGCATCTGTTGCAGCTCACAGTCTGGGGCATC
>KM259479
TTCTACTGTAATACAACACGGCTGTTTAATAGTGAGTGGCTGTCCAATGGTACT-------------------
----
-TGGAAAGGGTCAAAT---------
GACACTGGAGAAAATATTACACTCCCATGCAGAATAAAACAAATTATAAACATGTGGCAGGAAGTAGGAAAAG
CAATGTATGCTCCTCCCATCAGAGGACAAATCAATTGTGTATCAAAT---
ATTACAGGAATATTATTAACAAGAGATGGGGGT--

AATGGCAATAACAGT---
ACCAATGAGACTTTCAGACCTGGAGGAGGAGATATGAGGGACAATTGGAGAAGTGAATTATATAAATATAAAG
TAGTAAAAATTGAACCATTA---GGAGTAGCACCCACCAAGGCAAAGAGAAGAGTGGTG--------------
-------------------------CAGAGAGAAAAAAGAGCA---GCA------CTAGGAGCT---------
ATATTCCTTGGGTTCTTGGGAGCA---GCAGGAAGCACTATG---GGCGCAGCGTCA---
TTGACGCTGACGGTACAA---GCCAGACTT---TTATTG---TCTGGTATAGTGCAACAGCAAAAC---
AATTTG---CTGAGG---GCTATTGAGGCGCAACAGCATCTGTTGCAACTCACAGTCTGGGGCATC
>EU578667
TTCTACTGCAATTCAACACAACTGTTTAATAGTACTTGGAATGATAGTACTTGGAATGATACTACAGAGCCAA
AT---

AACACTGACGACACAATCACACTCCCATGTAGAATAAGACAAATTGTAAACAGGTGGCAGGAAGTAGGAAGAG
CAATGTATGCCCCTCCCATCAGTGGACAAATTAACTGTTCATCACAG---
ATTACAGGGCTGATATTAACAAGAGATGGTGGTATTAACGAGAGT----------------------------
----

AATACCGAGACCTTCAGACCTGGAGGAGGAGATATGAGGGACAATTGGAGAAGTGAATTATATAAATATAAAG
TAGTAAAAATTGAACCATTA---GGATTAGCACCCACCAAGGCAAGGAGAAGAGTGGTG--------------
-------------------------CAGAGAGAAAAAAGAGCA---GCA------CTAGGAGCT---------
GTGTTCCTTGGGTTCTTGGGAGCA---GCAGGAAGCACTATG---GGCGCAGCGTCA---
ATAGCGCTGACGGTACAG---GCCAGACTA---CTATTG---TCTGGTATAGTGCAACAGCAGAGC---
AATCTG---CTGAGA---GCTATTGAGGCGCAACAACATATGTTGCAACTCACAGTCTGGGGCATC
>HQ217967
TTCTACTGTAATACAACACAATTGTTTAATAGTACATGGAAGAATGGTACTGCTGGGGTCAATGATACTACA-
----

GGAAATGACACCCATATCACCCTCCCATGCAGAATAAAACAATTATAAACATGTGGCAGCAAGTAGGAAAAG
CAATGTATGCCCCGCCCATCAGAGGACAAATTAGTTGTTTATCAAAT---
ATTACAGGGCTACTATTAACAAGAGATGGTGGTAATAACGACAAAAACGGG---------------------
----

ACCAACATCACCGAGATCTTCAGACCTGGAGGAGGAGATATGAGGGATAATTGGAGAAATGAATTATATAGAT
ATAAAGTAGTAAAAATTGAACCATTA---GGAGTAGCACCCACCAAGGCAAAGAGAAGAGTGGTG-------
------------------------------CAAAGAGAAAAAAGAGCA---GCGGGG---TTAGGAGCT---
------TTGTTCCTCGGGTTCTTGGGAGCA---GCAGGAAGCACTATG---GGCGCAGCATCA---
GTGACGCTGACGGTACAG---GCCAGACAA---TTATTG---TCTGGTATAGTGCAACAGCAGAAC---
AATTTG---CTGAGG---GCTATTGAGGCGCAACAGCATCTGTTGCAACTCACAGTCTGGGGCATC
>HQ218020
TTCTACTGTAATACAGATAAACTGTTTAATAGTAATTGGACAACAAATACTAGCTCTGAGAATGTAACAAGTG
GCACT---

GAAGGAAATGATATAATCACACTCCCATGCAGAATAAGACAAATTATAAACCTGTGGCAGGAAGTAGGAAAAG
CAATGTATGCCCCTCCCATCCAAGGACAAATTAATTGTTCATCAAAT---
ATTACAGGGCTGTTATTAACAAGAGATGGTGGTGCTAACCAAACA---------------------------
----

AACAACACTGAGGTCTTCAGACCTGGAGGAGGAGATATGAGGGACAATTGGAGAAGTGAATTGTATAAATATA
AAGTAATAAAAATTGAGCCAGTA---GGAATAGCACCCACCAAGGCAAAGAGAAGAGTGGTG----------
---------------------------CAGAGAGAAAAAAGAGCA---GTGACG---ATAGGAGCT------
---GTGTTCCTTGGGTTCTTGGGAACA---GCAGGAAGCACTATG---GGCGTAGCGTCA---
CTGACGCTGACGGTACAG---GCCAGACAA---TTATTG---TCTGGTATAGTGCAGCAGCAGAAC---
AATTTG---CTGAGA---GCTATTGAGGCGCAACAGCATCTATTGCAACTCACAGTCTGGGGCATC
>HQ218034
TTCTACTGTAATACATCAGGGCTGTTTAATAGTACTTGGAATGTTTCTGCAGATTTGAATACCACTGAAGAGT
CA--

AATAGTAATGAAACCATCACACTCCAATGCAGAATAAAACAATTTATAAACATGTGGCAGGAAGTAGGAAGAG
CAATGTATGCTCCTCCCATCAGAGGACAAATTAAATGTTCATCAAAT---
ATTACAGGGCTACTATTAACAAGAGATGGTGGTAACGACAGCGCC---------------------------
----

CCCACAGAGACCTTCAGACCAGGAGGAGGAGATATGAGGGACAATTGGAGAAGTGAATTATATAAATATAAAG
TAGTACAGATTGAACCAATA---GGAGTAGCACCCACCAAGGCAAAGAGAAGAGTGGTG-------------
------------------------CAAAGAGAAAAAAGAGCA---GTGGGA---TTAGGAGCT---------
ATGTTCCTTGGGTTCTTGGGAGCA---GCAGGAAGCACTATG---GGCGCAGCGTCA---
GTGACGCTGACGGTACAG---GCCAGACAA---TTATTG---TCTGGTATAGTGCAACAGCAGAAC---
AATTTG---CTGATG---GCTATTGAGGCGCAACAAAAACTGTTGCAACTCACAGTCTGGGGCATC
>GU330865
TTCTATTGTAATACAACAAAACTGTTTAACAGTACTTGGATGGCTAATAGTACTTGGAATGATACTACACAGT
CA--

GATAACAATGACACTATCATACTCCAATGCAGAATAAAACAAATTATAAACAGGTGGCAGGAAGTAGGAAGAG
CTATGTATGCCCCTCCCATTAAGGGAGAAATTAAATGTTCATCAAAT---
ATTACAGGGTTGCTATTAACAAGAGATGGTGGTAATATTGATAAC---------------------------
----

GAGACTGAGACCTTTAGACCTGGGGGAGGAGACATGAGGGACAATTGGAGAAGTGAATTATATAAATATAAAG
TAGTAAAAATTGAGCCATTA---GGAGTAGCACCCACCAAGGCAAAGAGAAGAGTGGTG-------------
------------------------CAGAGAGAAAAAAGAGCA---GTAACA---CTGGGAGCT---------
ATGTTCCTTGGGTTCTTGGGAGCA---GCAGGAAGCACTATG---GGCGCAGCGTCG---
ATGACGCTGACGGTACAG---GCCAGACAA---TTATTG---TCTGGTATAGTGCAACAGCAGAGC---
AACATG---CTGAGA---GCTATTGAGGCGCAACAACATCTGTTGCAACTCACAGTCTGGGGCATC
>JF689877
TTCTACTGTAATACATCACAATTGTTTAATATGAGTTCAACACAACTGTTTAATAAGACTTGGACTAATGAC-
----

ACTAGAGAAAACATCACACTCCCATGTAGGATAAGACAAATTGTAAACCTGTGGCAGGAAGTAGGAAAAGCAA
TGTATGCCCCTCCCATCAGTGGACCAATTAGATGTGAATCAAAT---
ATTACAGGGCTGATATTAACAAGAGATGGTGGTAATGAGACCAATGGG-------------------------
----

AATGAGACGATTGAGACTTTCAGACCTGGAGGAGGTAATATGAAGGACAATTGGAGAAGTGAATTATATAAAT
ATAAAGTAGTAAGAATTGAACCAATA---GGAGTAGCACCCACCAAGGCAAAGAGAAGAGTGGTG--------
-------------------------------CAGAGAGAAAAAAGAGCA---GTGGGA---ATAGGAGCT---
------GTGTTCCTTGGGTTCTTGGGAGCA---GCAGGAAGCACTATG---GGCGCAGCGTCA---
ATGACGCTGACGGTACAG---GCCAGACAA---TTATTG---TCTGGTATAGTGCAACAGCAGAAC---
AATCTG---CTGAGG---GCTATTGAGGCGCAACAACATCTGTTGCAACTCACAGTCTGGGGCATC
>JF689879
TTCTACTGTGATACAACAAAGCTGTTTAATAGCACTTGGCAGGTTAATAGTACTTGGAATGATACTGAAAAG-
----

TCAAATGACACTCTCACAATCCCATGTAGAATAAGGCAAATTGTAAATAGGTGGCAGGAAGTAGGAAAAGCAA
TGTATGCCCCTCCCATCAGTGGACTAATTAGTTGTTCATCAAAT---
ATTACAGGAATGATATTAACAAGAGATGGTGGGTATAATAATGGGAGC-------------------------
----

AATAATACGGAGGTCTTCAGACCTGGAGGAGGAAATATGAAGGATAATTGGAGAAGTGAATTATATAAATATA
AAGTAGTAGAAATTGAGCCATTA---GGAGTAGCACCTACCAGGGCAAGGAGAAGAGTGGTG-----------
----------------------------CAAAGAGAAAAAAGAGCA---GTGGGAACATTAGGAGTT------
---ATGTTCCTTGGGTTCTTGGGAGCA---GCAGGAAGCACTATG---GGCGCAGCGTCG---
CTGACGCTGACGGTACAA---GCCAGGCAA---TTATTG---TCTGGTATAGTGCAACAGCAGAGC---
AATCTG---CTAAGG---GCTATTGAGGCGCAACAGCATATGTTGCAACTCACAGTCTGGGGCATC
>JF689883
TTCTACTGTAATTCAACACAACTGTTTAATAGCACTTGGCATGCTAATGGGAGTAATACAAAT----------
----

CTCGCTACTAACATCACACTCCCATGTAAAATAAAGCAAGTTATAAACATGTGGCAGGAAGTAGGAAAAGCAA
TGTATGCCCCTCCCATCGAAGGACTAATTAGATGCTCATCAAAT---
ATTACAGGAATACTATTAACAAGAGATGGTGGTAACAGGAACGGGACC-------------------------
----

AGTGATACTGAGATCTTCAGGCCTGCAGGAGGAGATATGAGGGACAATTGGAGAAGTGAATTATATAAATATA
AAGTAGTAAAAATTGAACCATTA---GGAGTAGCACCCACCAAGGCTAAGAGAAGAGTGGTG-----------
----------------------------CAGAGAGAAAAAAGAGCA---GTGGGA---ATCGGAGCG------
---TTGTTCCTTGGGTTCTTGGGAGCA---GCAGGAAGCACTATG---GGCGCAGCGTCA---
ATGGCGCTGACGGTACAG---GCCAGACAA---TTATTG---TCTGGTATAGTGCAACAGCAGAAC---
AATCTG---CTGAGG---GCTATTGAGGCGCAACAGCATCTGTTGCAACTCACAGTCTGGGGCATC
>JF689885
TTCTACTGTGATACAAAAGAACTGTTTAATAGTACTTGGACTAACAATGGGACTGAGAATAAGAATGAGACTG
AG---

AATAAGAATGGGACTATCACACTCCCATGTAGAATAAAACAAATTGTAAACATGTGGCAGGAAGTAGGAAAAG
CAATGTATGCCCCTCCCATCAGGGGACAAATTAGATGTGAATCAAAT---
ATTACAGGGCTGCTATTAACAAGAGATGGGGGTACGGATAATGATAATAGC----------------------
----

ACAAACACCACCGAGATCTTTAGACCTGTGGGAGGAGACATGAAGGATAATTGGAGAAGTGAATTATATAAAT
ACAAAGTAGTAAAAGTTGAACCATTA---GGAATAGCACCCACCAAGGCAAAGAGAAGAGTGGTG--------
-------------------------------CAGAGAGAAAAAAGAGCA---TTAACA---CTAGGAGCG---
------ATGTTCCTTGGGTTCTTAGGAGCA---GCAGGAAGCACTATG---GGCGCAGCGTCA---
ATAACGCTGACGGTACAT---GCCAGGCAA---TTATTG---ACTGGTATAGTGCAACAGCAGAGC---
AATCTG---CTGAAG---GCTATTGAGGCGCAACACCACCTGTTGCAACTCACAGTCTGGGGCATC
>JF689890
TTCTACTGTAATACAACAAAACTGTTTAATAGTACTTGGGAGGTTAATGGAACTTGGCCTGAAGGGTCAAATA
ACACT--

GAAGGAAATGACACAATCACACTCCCATGCAGAATAAAACAAATTATAAACATGTGGCAGGAAGTAGGAAAAG
CAATGTATGCCCCTCCCATCAGAGGACAAATTAGATGTGTATCAAAT---
ATTACAGGGCTGCTATTAACAAGAGATGGTGGTAGTGACAACCAGACT-------------------------
----

AACAACAATACTGAGACCTTCAGACCTGGAGGAGGAGATATGAAGGACAATTGGAGAAGTGAATTATATAAAT
ATAAAGTAGTACAAATTGAACCATTA---GGAATAGCACCCAACAAGGCAAAGAGAAGAGTGGTG--------
-------------------------------CAGAGAGAAAAAAGAGCA---GTGGGAGTAGTAGGAGCT---
------ATGTTCCTTGGGTTCTTGGGAGCA---GCAGGAAGCACTATG---GGCGCAGCGTCA---
GTGACGCTGACGGTACAG---GCCAGACTA---TTATTG---TCTGGTATAGTGCAACAGCAAAAC---
AATTTG---CTGAGA---GCTATTGAGGCGCAACAGCATCTGTTGCAACTCACAGTCTGGGGCATC
>JF689893
TTCTACTGTAATTCAACACCACTGTTTAATAGTACTTGGAAGGTTAATGATACTCGGAATGATACTGGAGGA-
----

GAAAATATCACACTCACACTCACATGCAGAATAAAACAAATTATAAACATGTGGCAGACAGTAGGAAAAGCAA
TGTATGCCCCTCCTATCAAAGGACAAATTAGATGTTCATCAAAT---
ATTACAGGGCTGTTATTAACCAGAGATGGTGGTAATGAGAACGAG----------------------------
----

AACAACACCGAGATCTTCAGACCTGCAGGAGGAGATATGAGGGACAATTGGAGAAGTGAATTATATAAATATA
AAGTAGTACAAATTGAACCATTA---GGAGTAGCACCCAACAGGGCAAAGAGAAGAGTGGTG-----------
----------------------------CAGAGAGAAAAAAGAACA---GTGGGA---ATAGGAGCT------
---TTGTTCCTTGGGTTCTTGGGAGCA---GCAGGAAGCACTATG---GGCGCAGCGTCA---
ATGACGCTGACGGTACAG---GCCAGACAA---TTATTG---TCTGGTATAGTGCAACAGCAGAGC---
AATCTG---CTGAGG---GCTATTGAGGCGCAACAGCATCTGTTGCAACTCACAGTCTGGGGCATT
>MK383385
TTCTACTGTAATACAACAAAACTATTTAATAGTACTTGGAATAGTACTGAAGAATTAAATATTACTGAAGGAT
CAAATACC---

ACGGGAGACCCTATCATACTCCCATGCAGGATAAAACAAGTTATAAACCTGTGGCAGGAAGTAGGAACAGCGA
TGTACGCCCCTCCCATCAGTGGACAAATTAACTGTTCATCAAAT---
ATTACAGGGCTGCTATTAACAAGAGATGGTGGTGTTGACAGTAAC----------------------------
----

ACGACCGAGATTTTCAGACCTGGAGGAGGAGATATGAGGGACAATTGGAGAAGTGAATTATATAAATATAAAG
TAGTAAAAATTGAACCATTA---GGAGTAGCACCCACCAGGGCAAAGAGAAGAGTGGTG--------------
-------------------------CAAAGAGAAAAAAGAGCA---GTGGGA---ATAGGAGTT---------
TTGTTCCTTGGGTTCTTGGGAGCA---GCAGGAAGCACTATG---GGCGCAGCATCA---
CTGACGCTGACGGTACAG---GCCAGACAG---TTATTG---TCTGGTATAGTGCAACAGCAGAAC---
AATCTG---CTGAGG---GCTATTGAGGCGCAACAGCACCTGTTGCAACTCACAGTCTGGGGCATC
>JX863994
TTCTACTGTAATACAACACAACTGTTTAATAGCACTTGGTTTGGGAATGATACTCGGAATGGTAGTGAAGAG-
----

GCAAATGACACAATTACACTCCCATGCAGAATAAAACAGATTATAAACATGTGGCAGGAAGTAGGAAAAGCAA
TGTATGCCCCTCCCATCAGAGGACAAATTAGCTGTTCTTCAAAC---
ATTACAGGGCTGTTATTAACAAGAGATGGTGGTATAAGAAATAAC----------------------------
----

AATAGCGAGACCTTCAGACCTGGGGGAGGAGACATGAGGGATAATTGGAGAAGTGAATTATATAAATATAAAG
TAGTAAAAATTGAACCATTA---GGAATAGCACCCACCAAGGCAAAGAGAAGAGTGGTG--------------
-------------------------CAGAGAGAAAAAAGAGCA---GTGGGA---TTGGGAGCT---------
TTGTTCCTTGGTGTCTTGGGAGCA---GCAGGAAGCACTATG---GGCGCAGCGTCG---
ATAACGCTGACGGTACAA---GCCAGAACT---TTATTG---TCTGGTATAGTGCAACAGCAGAAC---
AATCTG---CTGAGG---GCTATTGAGGCGCAACAGCATCTGTTGCAACTCACAGTCTGGGGCATC
>JX863995
TTCTACTGTAGTACATCACAACTCTTTAATAGTACTTGGTATGAGAATGGAACTAGGAATATTAGTGGA----
----

GGGACAGAAATTATCACACTCCCATGCAGAATAAAACAAATTATAAACATGTGGCAGCAGGTAGGAAAAGCAA
TGTATGCCCCTCCCATCAGGGGATTAATTAACTGTTCATCAAAT---
ATTACAGGGCTGCTATTAACAAGAGACGGTGGTGGGGAGAATAGA----------------------------
----

ACTAATGAAACATTCAGACCTGGAGGAGGAGACATGAGGGACAATTGGAGAAGTGAATTATATAAATATAAAG
TAGTAAAAATTGAACCCTTA---GGATTAGCACCCACCAAGGCAAAGAGAAGAGTGGTG--------------
-------------------------CAGAGAGAAAAAAGAGCA---GTGGGA---ATAGGAGCT---------
CTGTTCCTTGGGTTCTTGGGAGCA---GCAGGAAGCACTATG---GGCGCAGCGTCA---
ATAACGCTGACGGTACAG---GCCAGGCTA---TTATTG---TCTGGTATAGTGCAGCAGCAGAAC---
AATCTG---CTGAGG---GCTATTGAGGCGCAACAGCACCTGTTGCAACTCACAGTCTGGGGCATC
>JX863921
TTCTACTGTAATACAGCAGAACTGTTTAATAGTACTTGGGATAATGACAGGTCAAATAGCACAGAA-------
----

ATCACACTCCAATGTAGAATAAAACAAATTATAAACATGTGGCAGGAAGTAGGAAGAGCAATGTATGCCCCTC
CCATTGCAGGACAAATTAACTGCACATCAAAT---
ATTACAGGGCTGCTATTGACAAGAGATGGTGGTACAGACAGTAAT----------------------------
----

AATACTGAGATCTTCAGACCTGGAGGAGGAGATATGAGGGATAATTGGAGAAGTGAATTATATAAATATAAGG
TAGTAAAAATTGAACCATTA---GGAATAGCACCCACCAGGGCAAGGAGAAGAGTGGTG--------------
-------------------------CAGAGAGAGAAAAGAGCA---GTGGGA---CTGGGAGCT---------
TTGTTCCTTGGGTTCTTGGGAACA---GCAGGAAGCACTATG---GGCGCAGCGTCA---
ATAGCGCTGACGGTACAG---GCCAGACAA---TTATTG---TCTGGTATAGTGCAACAGCAGAAC---
AATTTG---CTGAGG---GCTATTGAGGCGCAACAGCGTATGTTGCAACTCACAGTCTGGGGCATC
>JX864007
TTCTACTGTAATACAGCACAGCTGTTTAATAGTACTTGGAGTACTTGGAATGGTACTGAAGGAGCAAATAACA
CTGAA--

GGAAATGATACAATCACACTCCCATGCAGAATAAAACAAATTATAAACATGTGGCAGGAAGTAGGAAAAGCAA
TGTATGCCCCTCCCATCAGTGGAATAATTAACTGTTCATCAAAT---
ATTACAGGGTTGCTATTAACAAGAGATGGTAGTAATAACACAAAG----------------------------
----

AACGAGACCTTCAGACCTGAAGGAGGGAATATGAGGGACAATTGGAGAAGTGAATTATATAAATACAAAGTAG
TAAAAATTGAACCATTA---GGAGTAGCACCTACCAAGGCAAGGAGAAGAGTGGTG-----------------
----------------------CAAAGAGAAAAAAGAGCA---GTGGGA---TTAGGAGCT---------
ATGTTCCTTGGGTTCTTGTCAGCA---GCAGGAAGCACTATG---GGCGCAGCGTCA---
GTGACGCTGACGGTACAG---GCCAGACAA---TTATTG---TCTGGTATAGTGCAACAGCAAAAC---
AACTTG---CTGAGG---GCTATTGAGGCGCAACAGCATCTGTTGCAACTCACAGTCTGGGGCATC
>JX864008
TTCTACTGTAATACAACAAGGCTGTTTAATAGTACCTGGCATCCGAATGGTGCTAAAATATCAAATGGC----
----

ACTGAAGAAACTATCACACTCCCATGTAGAATAAAACAAATTATAAACAGATGGCAGGAAGTGGGAAAAGCAA
TGTATGCCCCTCCCATCCAAGGAAAAATTAGCTGTGTATCAAAT---
ATTACAGGGCTACTATTAACAAGAGACGGTGGTAACAAGAGCCAT----------------------------
----

AACAGTACCGAGACCTTCAGACCTGGAGGAGGAGATATGAGGGACAATTGGAGAAGTGAATTATATAAATATA
AAGTAGTAAAAATTGAGCCATTA---GGAGTAGCACCGACCAAGGCAAAGAGAAGAGTGGTG-----------
----------------------------CAGAGAGAAAAAAGAGCA---GTAACG---CTGGGAGCT------
---ATGTTCCTTGGTTTCTTGGGAGCA---GCAGGAAGCACTATG---GGCGCAGCGTCG---
ATGACGCTGACGGTACAG---GCCAGACTA---TTATTG---TCTGGTATAGTGCAACAGCAGAAC---
AACTTG---CTGAGA---GCTATTGAGGCGCAACAACATCTGTTGCAGCTCACAGTCTGGGGCATC
>JX864020
TTCTACTGTAATACAACAATGCTGTTTAATAGTACTTGGTGGGCTAATGATACTTGGAATAGCACTTGGAATA
ATAATATC---

-------------------AGT---
AAAGAAAATGACACAATCACACTCCCATGCAGAATAAGACAAATTGTAAACATGTGGCAACAAGTAGGAAAAG
CAATGTATGCACCTCCCATCAGAGGAGAAATTAGATGTTCATCAAAT---
ATTACAGGGCTGCTAATAACAAGAGATGGGGGAATAACAACACTGGGAGAGGG------------------
----

GAGAATAAGACTGAGATCTTCAGACCTGTAGGAGGAGATATGAAGGACAATTGGAGAAGTGAATTATATAAAT
ATAAAGTAGTAAGAATTGAACCATTA---GGAATAGCACCCACCAAGGCAAAGAGAAGAGTGGTG--------
-------------------------------CAGAGAGAAAGAAGAGCA---GTGGGA---CTCGGAGTT---
------TTTTTTCTTGGGTTCTTGGCAACA---GCAGGAAGCACTATG---GGCGCAGCATCA---
ATAACGCTGACGGTACAG---GCCAGACAA---TTATTG---TCTGGTATAGTGCAGCAGCAGAAC---
AATCTG---CTGAGA---GCTATTGAGGCGCAACAGCATCTGTTGCAACTCACAGTCTGGGGCATT
>JF320375
TTCTACTGTAATACAACAAAGCTGTTTAATAGTACTTGGAATGGTACTGATACTTGGGATACTACTACAGAGG
CA--

GATAACAATGACACTATCACACTCCCATGCAGAATAAAACAAATTATAAACAGGTGGCAGGAAGTAGGAAAAG
CAATGTATGCCCCTCCCATTGCAGGACAGATCAGATGCTCATCAAAT---
ATTACAGGGATACTCTTAACAAGAGATGGTGGTAGTAATGACACA----------------------------
----

GACAGTGAGACCTTCAGACCAGGAGGAGGAGATATGAGGGACAATTGGAGAAGTGAATTATATAAGTATAAAG
TAGTAAGAATTGAGCCATTA---GGAATAGCACCCACCCAGGCAAAAAGAAGAGTGGTG--------------
-------------------------CAGAGAGAAAAAAGAGCA---GTGGGAACAATAGGAGCT---------
ATGTTCCTTGGGTTCTTGGGAGCA---GCAGGAAGCACTATG---GGCGCAGCGTCA---
TTGACGCTGACGGTACAG---GCCAGACAA---TTATTG---TCTGGTATAGTGCAGCAGCAGAAC---
AATCTG---CTGAGA---GCTATTGAGGCGCAACAGCATATGTTGCAACTCACAGTCTGGGGCATC
>JF320563
TTCTACTGTAATTCAACAAAACTGTTTAATAGTACTTGGAATGCTAATGGTACTATTAATGATACTAACGGGT
CA--

AATACAACAGAGGTGATCACGCTCCCATGCAGAATAAAACAATTTATAAACATGTGGCAGGAAGTAGGAAAAG
CAATGTATGCCCCTCCCATCAGAGGAGCAATTAACTGCTCATCAAAT---
ATTACAGGAATACTGTTAACAAGAGATGGTGGAAGTAACCAAAGT----------------------------
----

AATAACATCGAGGTCTTCAGACCTGGAGGAGGAGATATGAGGGACAATTGGAGAAGTGAATTATATAAATATA
AAGTAGTAAGAATTGAACCATTA---GGACTAGCACCTACTAAGGCAAGGAGAAGAGTGGTG-----------
----------------------------CAGAGAGAAAAAAGAGCA---GCGGGC---CTGGGAGCT------
---GTGTTCCTTGGGTTCTTGGCAGCA---GCAGGAAGCACTATG---GGCGCAGCGTCA---
ATAACGCTGACGGTACAG---GCCAGACAA---TTATTG---TCTGGTATAGTGCAACAGCAGAAC---
AATTTG---CTGAGG---GCTATTGAGGCGCAACAACGCATGTTGCAACTCGCAGTCTGGGGCATC
>JF320530
TTCTACTGTGATACAACACCGCTGTTTAATTCTACTTGGAACAATACTACCAATGAAAATAACATTACT----
----

AGAGGAGAAAACATCACACTCCCATGCAGAATAAAACAAATTATAAATAGGTGGCAAGAAGTAGGAAAAGCAA
TGTACGCCCCTCCCATCGCAGGACAAATTAGATGCTCATCAAAC---
ATTACAGGGCTGTTATTAACAAGAGATGGTGGTAATAATAATAACACC-------------------------
----

ACCACCACCGAGACCTTCAGACCAGGAGGAGGGGATATGAGAGATAATTGGAGAAGTGAATTATATAAATATA
AAGTAGTAAAAATTGAGCCATTA---GGAATAGCACCCACCAAGGCAAGGAGAAGAGTGGTG-----------
----------------------------CAAAGAGAAAAGAGAGCA---GTGGGAACAATAGGAGCT------
---ATGTTCCTTGGGTTCTTGGGAGCA---GCAGGAAGCACTATG---GGCGCAGCGTCA---
ATAACGCTGACGGTACAA---GCCAGACTA---TTATTG---TCTGGTATAGTGCAGCAGCAGAAC---
AATCTG---CTGCAA---GCTATTGAGGCGCAACAGCATCTGTTGCAGCTCACAGTCTGGGGCATC
>JF320631
TTCTACTGTGACTCAACACAACTGTTTAATAGTACTTGGACATTTACTGACGGGAGGTTAAATGAAACTGGGT
TAAAT---

-------------------GAA---
ACTGAAGCTGGAAATATCACACTCCCATGCAGAATAAAACAATTTATAAATATGTGGCAGGGAGTAGGAAGAG
CAATGTATGCCCCTCCCATCAGAGGACACATTAACTGTACATCAAAT---
ATTACAGGGTTGCTATTAACAAGAGATAGTGGTAGTAATGAGACTAGT-------------------------
----

AAGAACGGGTCAGAGACCTTCAGACCTGGAGGAGAAAATATGAAGGACAATTGGAGAAGTGAATTATATAAAT
ACAAAGTAGTAAGAATTGAACCATTA---GGAATAGCACCCACCAGGGCAAGGAGAAGAGTGGTA--------
--------------------------------CAAAGAGAAAAAAGAGCA---GTGGGA---ATAGGAGCT---
------ATGTTCCTTGGGTTCTTGGGAGCA---GCAGGAAGCACTATG---GGCGCAGCGTCA---
CTGACGCTGACGGTACAG---GCCAGACAA---TTATTG---ACTGGTATAGTGCAACAGCAAAGA---
AATTTG---CTGAGA---GCTATTGAGGCGCAACAGCATATGTTGCAACTCACAGTCTGGGGCATT
>JF320045
TTCTACTGTAATACATCAAAACTGTTTAACAGTACTTGGAGTATGAATAGTACTGAAGGGAGTATGAATGGTA
CTGAAGGG---
--------------GCAAAT------
AACACTGATGAGAAAATCGTACTCCAATGCAGAATAAAACAAATTATAAACATGTGGCAGGAAGTAGGAAAAG
CAATGTATGCCCCTCCCATCAGCGGACTAATTAGATGTTCATCAAAT---
ATTACAGGGTTGTTATTAACAAGAGATGGCGGTAAAAAGGATAACATGACA----------------------
----

GAGAAGGATGAGACAGAGACTTTCAGACCTTGGGGAGGAGATATGAGGGACAATTGGAGAAGTGAATTATATA
AATATAAAGTAGTACAAATTGAACCATTA---GGAATAGCACCCACCAGGGCAAAGAGAAGAGTGGTG-----
-----------------------------------CAGAGAGAAAAAAGAGCA---GTGGGA---
ATAGGAGCT---------CTATTCCTTGGGTTCTTGGGAGCA---GCAGGAAGCACTATG---
GGCGCAGCGGCA---GTGACGCTGACGGTACAG---GCCAGACAA---TTATTG---
TCTGGTATAGTGCAACAGCAGAAC---AATCTG---CTGAGA---
GCTATTGAGGCGCAACAGCATCTGTTGCAACTCACAGTCTGGGGCATC
>JF320173
TTCTATTGTAATACCACACAACTGTTTAATAATACTTGGTCTAACGGTACTTGGAATAGTACTTGGAATAGT-
----

AGTATCACACATATCACACTCCCATGCAAAATAAGACAAATTATAAACATGTGGCAGGAGGTAGGAAAAGCAA
TATATGCCCCTCCCATCAGAGGGCTAATTAGCTGTTCATCAAAT---
ATTACAGGGCTGCTGTTAGTAAGAGATGGTGGTACTAATACTACTGAA-------------------------
----

GACAATACTGAGATCTTCAGACCTGGAGGAGGAGATATGAGGGACAATTGGAGAAGTGAATTATATAAATATA
AAGTAGTAAAAATTGAACCATTA---GGAGTAGCACCCACCAGGGCAAAGAGAAGAGTGGTG-----------
-----------------------------CAAAGAGAAAAAAGAGCA---GTGGGAACAATAGGAGCT------
---ATGTTCCTTGGGTTCTTGGGAGCA---GCAGGAAGCACTATG---GGCGCAGCGTCA---
ATGGCGCTGACGGTACAG---GCCAGACAA---TTATTG---TCTGGTATAGTGCAACAGCAAAAT---
AATTTG---CTGAGG---GCTATTGAGGCGCAACAACATTTGTTGCAACTCACAGTCTGGGGCATT
>JF320387
TTCTACTGTAATTTATCACAGCTGTTTAATAGTACTTGGAATTTTACTGAAGGGTCAAATAGCACAGGAAAT-
----

TTGTCAGACACTATCACAATCCCATGCAGAATAAAGCAAATTATAAACATGTGGCAGGAAGTAGGAAAAGCAA
TATATGCTCCTCCTATCAGGGGACTAATTAGATGTTCATCAAAT---
ATTACAGGGCTGTTAATAACAAGAGATGGTGGTATTAATAATAGTGATAGT----------------------
----

GATGATAACACCGAGATCTTTAGACCTGCAGGGGGAAATATGAAGAACAATTGGAGAAGTGAATTATATAAAT
ATAAAGTAGTAAAACTTGAACCATTA---GGAGTGGCACCCACCAAGGCAAAGAGAAGAGTGGTG--------
--------------------------------CAAAGAGACAAAAGAGCA---GTGGGAATGATAGGAGCT---
------ATGTTCCTTGGGTTCTTGGGAGCA---GCAGGAAGCACTATG---GGCGCAGCGTCA---
CTAACGCTGACGGTACAG---GCCAGACAA---TTATTG---ACTGGTATAGTGCAACAGCAGAGC---
AATTTG---CTGAGA---GCTATTGAGGCGCAACAGCACCTGTTGCAACTCACAGTCTGGGGCATC
>JF320117

```
TTCTACTGTAATTCAACACAATTATTTAACAGTACTTGGAATGATACTACAGGGACAGGGAACATT-------
-------------------------------------------------------------------------
----------------------------
ACAGGAAATATTATTACACTCCCATGCAGAATAAGACAAATTATAAATATGTGGCAGGAAGTAGGAAAAGCAA
TGTATGCCCCTCCCATCAGAGGAATAATTAGCTGTTCATCAAAT---
ATTACAGGGCTGATATTAACAAGAGATGGGGGTAATAATGAGACC----------------------------
-------------------------------------------------------------------------
----------
AATGGACCTGAGATCTTCAGACCTCTAGGAGGAAATATGAGGGATAATTGGAGAAGTGAACTATATAAATATA
AAGTAGTAAAGATAGAACCATTA---GGAGTAGCACCCACCAAGGCAAAGAGAAGAGTGGTG-----------
----------------------------CAGAGAGAAAAAAGAGCA---ATAGGA---CTAGGAGCT------
---GTGTTCATTGGGTTCTTGGGAGCA---GCAGGAAGCACTATG---GGCGCAGCGTCA---
GTGACGCTGACGGTACAG---GCCAGACAA---TTGTTG---TCTGGTATAGTGCAACAGCAGAAC---
AACTTG---CTGAAG---GCTATTGAGGCGCAACAGCATCTGTTGCAACTCACAGTCTGGGGCATC
>JF320182
TTCTACTGTAATACATCACAGCTGTTTAATAGCACGTGGCCAATTAATGGTGCATGGAATGGAACT-------
-------------------------------------------------------------------------
----------------------------
GAAGGGTCAGATATCACACTTCCATGTAGAATAAGACAAATTGTAAACAGATGGCAGGAAGTAGGAAAAGCAA
TGTATGCCCCTCCCATCAGTGGACTAATTAGATGTTCATCAGAT---
ATTACAGGGATACTATTAACAAGAGATGGTGGGGGCAATGAGACA----------------------------
-------------------------------------------------------------------------
-------------
GAGACAGAGACCTTCAGACCTGGAGGAGGAAATATGAGGGACAATTGGAGAAGTGAATTATATAAATATAAAG
TAGTAAAAATTGAGCCATTA---GGGATAGCACCCACCAAGGCAAGGAGAAGAGTGGTG--------------
-------------------------CAGAGAGAAAAAAGAGCA---GTGGGAACAATAGGAGCT---------
ATGTTCCTTGGGTTCTTGGGAGCA---GCAGGAAGCACTATG---GGCGCAGCGTCA---
GTGACGCTGACGGTACAG---GCCAGACTA---TTATTG---TCTGGTATAGTGCAACAGCAGAAT---
AATCTG---CTGAGG---GCTATTGAGGCGCAGCAGCATCTGTTGCAGCTCACAGTCTGGGGCATC
>JF320577
TTCTACTGTAATACAACATCACTGTTTAATAGTACTTGGAATAGTACTGGGAGGCTTAATGATACTGAAGGG-
-------------------------------------------------------------------------
----------------------------
TCAAATGACACCATTACACTCCAATGCAGAATAAAACAAGTTATAAACTTGTGGCAGGAAGTAGGAAAAGCAA
TGTATGCTCCTCCCATCAAAGGACCAATTAGGTGTTCATCAAAT---
ATTACAGGACTGATATTAACAAGAGATGGTGGTAACAGTAACGAG----------------------------
-------------------------------------------------------------------------
----------
AGCGAAAGCGAGACCTTCAGACCTGGAGGAGGAGATATGAGGGATAATTGGAGAAGTGAATTATATAAATACA
AAGTAGTAAAAATTGAACCATTA---GGAGTAGCACCCACCAAGGCGAAGAGAAGAGTGGTG-----------
----------------------------CAGAGAGAAAAAAGAGCA---ATTGGA---CTAGGAGCT------
---ATGTTCCTTGGGTTCTTGGGAGCA---GCAGGAAGCACTATG---GGCGCAGCGTCA---
ATAACGCTGACGGTACAG---GCCAGACAA---TTATTG---TCTGGTATAGTGCAACAGCAGAAC---
AATTTG---CTGAGA---GCTATTGAGGCGCAACAACATCTGTTGCAACTCACAGTCTGGGGCATC
>KX156431
TTCTACTGTAATACAACACGACTGTTTAATAGTACTTGGGATGTTAATAATACTTGGGTTGATGGTAATTGGA
GTGATACC-----------------------------------------------------------------
----------AACAGCGGA------
GTAAATATCACAAATATCACCCTCCCATGCAGAATAAAACAAATTGTAAACATGTGGCAGAAAGTAGGAAAAG
CAATGTATGCCCCTCCCATTAGAGGACAAATTAGATGCTCATCAAAT---
ATTACAGGGCTGCTATTAACAAGAGATGGTGGTGATATCAATAGC----------------------------
-------------------------------------------------------------------------
-------------
ACGACCGAGACCTTCAGACCTGGAGGAGGAGATATGAAGGACAATTGGAGAAGTGAATTATATAAATATAAAG
TAGTAAGAATTGAACCATTA---GGAATAGCACCCACCAAGGCAAAGAGAAGAGTGGTG--------------
-------------------------CAGAGAGAAAAAAGAGCA---GTGGGA---ATAGGAGCT---------
CTGTTCCTTGGGTTCTTGGGAGCA---GCAGGAAGCACTATG---GGCGCAGTGTCA---
ATGACGCTGACGGTACAG---GCCAGACTA---TTATTG---TCTGGTATAGTGCAACAGCAGAAC---
AATTTG---CTGAGG---GCTATTGAGGCGCAACAGCATCTGTTGCAACTCACAGTCTGGGGCATC
>HQ216971
```

TTCTACTGTAATACAACAAGGCTGTTTAATAGTACTTGGAATGGTACTGAGGTAACTAACAATGGAGAA----
----

AATAGCACAATTATCACACTCCAATGCAAAATAAAACAATTTATAAACATGTGGCAGAGAGTAGGAATAGCAA
TGTATGCCCCTCCCATCACAGGACAAATCAGTTGTTCATCAAAT---
ATTACAGGGCTGCTATTAACAAGAGACGGTGGTAATAATAACAGT----------------------------
----

ACAGAGACCTTCAGACCTCTAGGAGGAAATATGAGGGACAATTGGAGAAGTGAATTATATAAATATAAAGTAG
TAAAGGTTGAACCATTA---GGAATAGCACCCTCCAGGGCAAGAAGAAGAGTGGTG-----------------
----------------------CAGAGAGAAAAAAGAGCG---GTGGGAGCACTAGGAGCT---------
TTGTTCCTTGGGTTCTTGGGAGCA---GCAGGAAGCACTATG---GGCGCAGCGTCT---
CTGACACTGACGGTACAG---GCCAGACAA---TTATTG---TCTGGTATAGTGCAACAGCAGAAC---
AATCTG---CTGAGA---GCTGTTGAGGCGCAACAACATCTGTTGCAACTCACAGTCTGGGGCATC
>HQ908150
TTCTACTGTAATACAACAGGACTATTTAATAGTACTTGGAATAGTACAGAAGGGTCAAATAAT----------
----

GATGGCAAAAACATAACACTCCAGTGCAGAATAAAACAAATTATAAACCTGTGGCAGGAAGTAGGAAAAGCAA
TGTATGCCCCTCCCATCCGAGGAGAAATTAGATGTTCATCAAAC---
ATTACAGGGTTGCTACTAACAAGAGATGGTGGTAATGACACAGAA----------------------------
----

AACATCGAAACCTTCAGACCTGCAGGAGGGGATATGAGGGACAATTGGAGAAGTGAATTATATAAATATAAAG
TAGTAAAAATTGAACCATTA---GGAGTAGCACCCACCAAGGCAAAGAGAAGAGTGGTG--------------
-------------------------CAGAGAGAAAAAAGAGCA---GTGGGA---ATAGGAGCT---------
ATATTCCTTGGGTTCTTGGGAGCA---GCAGGAAGCACTATG---GGCGCAGCGTCA---
GTGACGTTGACGGTACAG---GCCAGACAA---TTATTG---TCTGGTATAGTGCAACAGCAAAAC---
AATTTG---CTGAGG---GCTATTGAGGCGCAACAAAGTCTGTTGCAACTCACAGTCTGGGGCATC
>HQ217045
TTCTACTGTGATACAGCAAAACTGTTTAATGGTACTTTAAACAATACTATAAATAATACTACATGGAAGTTTG
AA---

GGGTCAAATGGCACTATCATACTCCCATGCAGAATAAGACAAATTGTAAACATGTGGCAGGAAGTAGGAAAAG
CAATGTATGCCCCTCCCATCAAAGGAAACATTAGTTGTACATCAAAT---
ATTACAGGGCTGCTATTAGTAAGAGATGGTGGTGTTAAAAACAGC----------------------------
----

ACGAACGAGACCTTCAGACCTGGAGGAGGAAATATGAAAGACAATTGGAGGAGTGAATTATACAAATATAAAG
TAGTAAAAATTGAGCCATTA---GGAATAGCACCCACCAAGGCAAAGAGAAGAGTGGTG--------------
-------------------------CAGAGAGAAAAAAGAGCA---GTGGGA---ATAGGAGCT---------
TTGTTCCTTGGGTTCTTGGGAGCA---GCAGGAAGCACTATG---GGCGCAGCGTCA---
ATGACGCTGACGGTACAG---GCCAGACTA---TTATTG---TCTGGTATAGTGCAACAGCAGAAC---
AATCTG---CTGAGG---GCTATTGAGGCGCAACAGCATCTGTTGCAACTCACAGTCTGGGGCATT
>HQ908184
TTCTACTGTAATACAACAAATCTGTTTACTAACGAGCTAAATGAC----------------------------
----

ACTGATGGAAATATCATACTCCCATGTAGAATAAAACAAATTATAAACATGTGGCAGGAAGTAGGAAAAGCAA
TGTATGCTCCGCCCATTAGTGGACCAATTAATTGTTTATCAAAT---
ATCACAGGGCTGCTATTAACAAGAGATGGTGGTATAGGCAACAAC----------------------------
----

ACCATCAACGAAACCTTCAGACCTGGAGGAGGAGATATGAGGGACAATTGGAGAAGTGAACTATATAAATACA
AAGTAGTAAAAATTAAACCATTA---GGATTAGCACCCACTAAGGCAAAGAGAAGAGTGGTG-----------
----------------------------CAAAGAGAAAAAAGAGCA---GTGGGA---ATAGGAGCT------
---TTGTTCCTTGGGTTCTTGGGAGCA---GCAGGAAGCACTATG---GGCGCAGCGTCA---
TTGACGTTGACGGTACAG---GCCAGATCC---TTATTG---TCTGGTATAGTGCAACAGCAGAAC---
AATCTG---CTGAGG---GCTATTGAGGCGCAACAGCATCTGTTGCAACTCACAGTCTGGGGCATC
>KM353733

TTCTACTGTAATACAACACAACTGTTTAATAGTACTTGGCCGCTTAATAGTACCTGG----------------
----
GAGGAGACTGAAAGGTCA---------
AATGGCACTGGTGTATACACACTCCAATGCAAAATAAAACAAATTATAAACAGGTGGCAGGAAGTAGGAAAAG
CAATGTATGCTCCTCCCATCAAGGGAATCATCAGTTGTGCATCAAAT---
ATTACAGGTCTGCTATTAACAAGAGATGGTGGTAAT-------------------------------------
----
AAGGCAAACACAAACGGAACTGAT---------
GAGACTGAGATCTTCAGACCTGGAGGAGGAGATATGAGGGACAATTGGAGAAGTGAATTATATAAATATAAAG
TAGTAAGGATTGAGCCATTG---GGAGTAGCACCCACTAAGGCAAAAAGAAGAGTGGTG--------------
-------------------------CAGAGAGAAAAAAGAGCA---GTAGGA---CTAGGAGCT---------
ATGTTCCTTGGGTTCTTGAGCACA---GCAGGAAGCACTATG---GGCGCAGCGTCA---
GTGACGCTGACGGTACAA---GCCAGACAA---TTATTG---TCTGGTATAGTGCAACAGCAGAAC---
AATTTG---CTGAGG---GCTATTGAGGCGCAACAGCATCTGTTGCAACTCACAGTCTGGGGCATC
>KM354114
TTCTACTGTAATACAACAAAACTGTTTAATGGTACTTGGTTTAATAGT-------------------------
----
----------ACTGTGAAT------
GATACTGAAGAAGAAATCATACTCCCATGCAGGATAAAACAAATTATAAATAGATGGCAGGAAGTAGGAAAGG
CAATGTATGCCCCTCCCATCAGAGGATACATTAATTGTTCATCAAAT---
ATTACAGGAATGATATTGACAAGAGATGGTGGT--

AATAACACCAGCAATGAGACCTTCAGACCTGGAGGAGGAGATATGAGGGACAATTGGAGAAGTGAATTATATA
AATATAAAGTAGTAAGAATTGAACCATTA---GGAATAGCACCCACTAAGGCAAGAAGAAGAGTGGTG-----
----------------------------------CAGAGAGAAAAAAGAGCA---GTGGGA---
ATAGGAGCT---------ATATTCCTTGGGTTCTTGGGAGCA---GCAGGAAGCACTATG---
GGCGCAGCATCA---ATGACGCTGACGGTACAG---GCCAGACAA---TTATTG---
TCTGGTATAGTGCAACAGCAGAAC---AACCTG---CTGAGG---
GCTATTGAGGCGCAACAGCATCTGTTGCAACTCACAGTCTGGGGCATT
>HQ217096
TTCTACTGTAATTCAACACAATTGTTTAATAGTACTTGGGATAAGGCTAATGACACTTTTTCTAGGAATGGGA
CT---

AGGTTAAATGGAACTATCACACTCCCATGTAGAATAAAACAAATTATAAACATGTGGCAGGGAGTAGGAAAAG
CAATGTATGCCCCTCCTATCAAAGGACAAATTAGCTGTTCATCAAAT---
ATTACAGGGCTGCTATTAACAAGAGATGGTGATAAGAATAAGAAC----------------------------
----

GAGACCGAGATCTTCAGACCGATAGGAGGAGATATGAGGGGCAATTGGAGAAATGAATTATATAAATATAAAG
TAGTAAAAATTGAACCATTA---GGAGTAGCACCCACCAAGGCAAAAAGAAGAGTGGTG--------------
-------------------------CAGAGAGAAAAAAGAGCA---GTGGGA---ATAGGAGCA---------
TTGTTCCTTGGGTTCTTGGGAGCA---GCAGGAAGCACTATG---GGCGCAGCGTCA---
CTAACGCTGACGGTACAG---GCCAGACAA---TTATTG---TCTGGTATAGTGCAGCAGCAAAGC---
AATTTG---CTGAGG---GCTATTGAGGCGCAGCAGCAGCTGTTGCAACTCACAGTCTGGGGCATC
>HQ217102
TTCTACTGTAATACAACACCACTGTTTAGTAGTACTTGGAATGAGACTACTTGGTCTAATAGTACTGCAAATA
CCACT--
-------------------GAA---
AAGGAAAGTGAAAATATTACACTCCAATGTAGACTAAAACAAATTGTAAACCTGTGGCAGGAGGTAGGAAAAG
CAATGTATGCCTCTCCCATCAGTGGACCAATTAGCTGTAAATCAAAT---
ATTACAGGGCTGATATTAACAAGAGATGGTGGTACAAATAATAATACG-------------------------
----

AACGGGACCACCGAGACCTTCAGACCTGGAGGAGGAGATATGAGGGACAATTGGAGAAGTGAATTATATAAAT
ACAAAGTAGTAAAAATTGAACCATTA---GGAATAGCACCCACCAGGGCAAAGAGAAGAGTGGTG--------
-------------------------------CAGAGAGAAAAAAGAGCA---GTGGGA---ATAGGAGCT---
------TTGTTCCTTGGGTTCTTGGGAGCA---GCAGGAAGCACTATG---GGCGCAGCGTCA---
ATGACGCTGACGGTACAG---GCCAGACTA---TTATTG---TCTGGTATAGTGCAACAGCAGAAC---
AATTTG---CTGAGG---GCTATTGAGGCGCAACAGCATCTGCTGCAACTTACAGTCTGGGGCATC

>JN687759
TTCTACTGTAATACATCATCACTGTTTAATAGTACTTGGAATGAAAATACAACTACAGATCTAAAT-------
----

AATAATGGAAATATCACACTCAAATGCAGGATAAAACAAATTATAAGAATGTGGCAGGAAGTAGGAAAAGCAA
TGTATGCTCCTCCCATCGAAGGACAAATTAGCTGTTCATCAAAT---
ATTACAGGGCTGCTATTAACAAGAGATGGTGGAAATGAGAACGAGACC-------------------------
----

AACGCGACCACCGAGACCTTCAGACCTGGGGGAGGAGATATGAGGGACAACTGGAGAAGTGAATTATATAAAT
ATAAAGTAGTACAACTTGAACCATTA---GGAGTGGCACCCACCAAGGCAAAGAGAAGAGTGGTG--------
-------------------------------CAGAGAGAAAAAAGAGCA---GTGGGA---ATAGGAGCT---
------GTGTTTCTTGGGTTCTTGGGAGCA---GCAGGAAGCACTATG---GGCGCAGCGTCA---
CTGACGCTGACGGTACAG---GCCAGACAA---TTATTG---TCTGGTATAGTGCAACAGCAGAAC---
AATCTG---CTGAGG---GCTATTGAGGCGCAACAGAGTCTGTTGCAACTCACAGTCTGGGGCATT
>JN687761
TTCTACTGTAATTCAACACAACTGTTTAATAGCACTTGGGATTCAAATAGCACTTGGGATGATTCAAATAATA
CTATT--

GAGGAGAATGGGACTATCACACTCCCATGTAGAATAAAACAAATTGTAAACATGTGGCAAAAAGTAGGGAGAG
CAATGTATGCCCCTCCCATCAGGGGACAAATTAGATGCTCATCAAAT---
ATTACAGGACTGCTATTAGCAAGAGATGGGAGTGACAACAGCATT----------------------------
----

AACGAGACCTTCCGACCTGGAGGAGGAGATATGAGGGACAATTGGAGAAGTGAATTATACAAATATAAAGTAG
TACAAATTGAACCAGTA---GGAATAGCACCCACCAAGGCAAAGAGAAGAGTGGTG-----------------
----------------------CAGAGAGAAAAAAGAGCA---GTGGGA---CTAGGAGCT---------
ATGTTCCTTGGGTTCTTGGGAGCA---GCAGGAAGCACTATG---GGCGCAGCGTCA---
ATAACGCTGACGGTACAA---GCCAGACAA---TTATTG---TCTGGTATAGTGCAACAGCAGAAC---
AACCTG---CTGAGA---GCTATTGAGGCGCAACAGCATCTGTTGCAACTCACAGTCTGGGGCATC
>JN687762
TTCTACTGCAATTCAACACAGCTGTTTAATAGTAGTTGGAATAGTACTGCTAATAGTACTGAAGGGAATGATG
AAACT--

ATCAATAATGAAATTATCACACTCCCATGCAGAATAAAACAAATTATAAACATGTGGCAGGAAGTAGGAAAAG
CAATGTATGCCCCTCCCATCGCAGGACCAATTAGATGTTCATCAAAT---
ATTACAGGGCTGTTATTAACAAGAGATGGTGGGAATAATGGGACT----------------------------
----

AATGAGACCGAGACCTTCAGACCTGCAGGAGGAAATATGAAGGACAATTGGAGAAGTGAATTATATAAATATA
AAGTAGTAAAAATTGAACCATTA---GGAGTAGCACCCACCAAGGCAAGGAGAAGGGTGGTG-----------
----------------------------CAGAGAGAAAAAAGGGCA---GCA------CTAGGAGCT------
---GTGTTCCTTGGGTTCTTGGGAGCA---GCAGGAAGCACTATG---GGCGCAGCATCA---
GTAATGCTGACGGTACAG---GCCAGACAA---TTATTG---TCTGGTATAGTGCAACAGCAGAAC---
AATCTG---CTGAGA---GCTATTGAGGCGCAACAGCACCTGTTGCAACTCACAGTCTGGGGCATC
>HQ217115
TTCTACTGTAATTCAACAAAACTGTTTAATAGTACTTGGGAGACTAATGGTACTTCGAATTTCAATGAGACTA
GA---

GGGTCAAATGACCATATCACACTCCCATGCAGAATAAAACAAATTATAAACATGTGGCAGGAAGTAGGAAAAG
CAATGTATGCCCCTCCTATCAGTGGACCAATTAGATGTTCATCAAAT---
ATTACAGGGCTGCTATTAACAAGAGATGGTGGTAATGAGAGCAGC----------------------------
----

ACCAACGAGACCTTCAGACCTGGGGGAGGAAACATGAAGGACAATTGGAGAAGTGAATTATATAAATATAAAG
TAGTAAAAGTTGAACCATTA---GGATTAGCACCCACCAAGGCAAAAAGAAGAGTGGTG--------------
-------------------------CAGAGAGAAAAAAGAGCA---ATAATGGGAATGGGAGCT---------
TTGTTCCTTGGGTTCTTGGGAGCA---GCAGGAAGCACTATG---GGCGCAGCGTCA---
ATAACGCTGACGGCACAG---GCCAGACAA---TTATTG---TCTGGTATAGTGCAGCAGCAAAAC---
AACTTG---CTGAGA---GCTATTGAGGCGCAACAGCATATGTTGCAACTCACAGTCTGGGGCATC

>KM259082
TTCTACTGTAATACAACAAAACTGTTTAATAGTACTTGGACTTGGAATAGTACTATT----------------
----
AATAGTACCGAGAATAGT---------
AGAAATGACACTAATATCACACTCCCATGCAGAATAAAACAAATTATAAACATGTGGCAGAAAGTAGGAAGAG
CAATGTATGCCCCTCCCATCGCAGGAATAATTAAATGCTCATCAAAT---
ATTACAGGGCTGCTATTAACAAGAGATGGTGGTAAAAAG----------------------------------
----
ACTAAGGATAACGGGACTGAGAGC---------
GACACTGAGATCTTCAGGCCTGGAGGAGGAGATATGAGGGACAATTGGAGAAGTGAATTATATAAATATAAAG
TAGTAAAAATTGAACCATTA---GGAGTAGCACCCACCAAGGCAAAAAGAAGAGTGGTG--------------
-------------------------CAAAGAGAAAAAAGAGCA---GTGGGA---ATAGGAGCT---------
GTGTTCCTTGGGTTCTTGGGAGCA---GCAGGAAGCACTATG---GGCGCAGCGGCA---
TTAACGCTGACGGTACAG---GCCAGACAA---TTATTG---TCTGGTATAGTGCGACAGCAGAAC---
AATCTG---CTGAGA---GCTATTGAGGCGCAGCAACATCTGCTGCAACTCACAGTCTGGGGCATC
>HQ908139
TTCTACTGTAATACAACAAAACTGTTTAATAGTACTTGGTATAGTAATACAACTGATACAGATAAT-------
----

ACTACAGGGAATATCACACTCCCATGCAGAATAAAACAAGTTATAAACATGTGGCAGGAAGTAGGAAAAGCAA
TGTATGCCCCTCCCATCAGAGGACAAATCAGATGTTCATCAAAT---
ATTACAGGGCTGTTATTAACAAGAGACGGTGGTAAGAACAATACC----------------------------
----

AACGGTACCGAGACTTTCAGACCTGGAGGAGGAGATATGAGGGACAATTGGAGAAGTGAATTATATAAATATA
AAGTAGTAAAAATTGAACCATTA---GGAGTAGCACCTACCAAGGCAAGGAGAAGAGTGGTG-----------
----------------------------CAGAGAGAAAAAAGAGCA---GTGGGA---ATAGGAGCT------
---GTACTCCTTGGGTTCTTGGGAGCA---GCAGGAAGCACTATG---GGCGCAGCATCA---
ATAACGCTGACGGTACAG---GCCAGACAA---TTATTG---TCTGGTATAGTGCAACAGCAGACC---
AATTTG---CTGAGG---GCTATTGAGGCGCAACAGCACCTGTTGCAACTCACAGTCTGGGGCATC
>HQ217160
TTCTACTGTAACACAACACAATTGTTTAATAGTACTTGGAATAATACTGAACTTAGTAGTACTTGGGAGACAG
AGCAA--

AATGACAATGACAATATCACACTCCCATGCAGAATAAAACAAATTATAAACACATGGCAGGAAGTAGGAAAAG
CAATGTATGCCCCTCCCATCAAAGGAGTAATTAGATGTTCATCAAAT---
ATTACAGGGTTGTTATTAACAAGAGATGGTGGTAAGAACACGAAC----------------------------
----

ACCACCGAGACCTTCAGGCCTGGAGGAGGAGATATGAGGGACAATTGGAGGAGTGAATTATATAAATATAAAG
TAATACAAATTGAACCATTA---GGGGTAGCACCCACCAAGGCAAAGAGAAGAGCGGTG--------------
-------------------------CAGAGAGAAAAAAGAGCA---ATAACA---ATAGGAGCT---------
ATGTTCCTTGGGTTCTTGGGAGCA---GCAGGAAGCACTATG---GGCGCAGCGTCA---
TTGACGCTGACGGTACAG---GCCAGACAG---TTGTTG---TCCGGTATAGTGCAACAGCAGAAC---
AATCTG---CTGAGA---GCTATTGAGGCGCAACAGCACCTGTTGCAACTCACAGTCTGGGGCATC
>FJ469689
TTCTACTGTAATACAACGAAATTGTTTAATAGTACTTGGAATAAGACTGATAGTGTTAATAGTACTTGGAATT
TTATTGAAGGG--
-------TTAAATAACACT------
GGAGAAAATGATATAATCACACTCCCATGCAGAATAAAACAAATTATAAACATGTGGCAGGAAGTAGGAAAAG
CAATGTATGCCCCTCCCATCAAAGGAATAATTAGCTGTAGATCAAAT---
ATTACAGGGCTGATATTAACAAGAGATGGTGGTAGTAATAACACA----------------------------
----

AACGAGACCTTCAGACCTGGAGGAGGAGATATGAGGGACAATTGGAGAAGTGAATTATATAAATATAAAGTAG
TAAAAATTGAACCATTA---GGAGTAGCACCCACCAGGGCAAAGAGAAGAGTGGTG-----------------
----------------------CAGAGAGAAAAAAGAGCA---GTGGGA---CTAGGAGCT---------
CTGTTCCTTGGGTTCTTGGGAGCA---GCAGGAAGCACTATG---GGCGCAGCGTCA---
GTGACGCTGACGGTACAG---GCCAGACAA---TTACTG---TCTGGTATAGTGCAACAGCAGAAC---
AATTTG---CTGAGG---GCTATTGAGGCGCAACAGCATCTGTTGCAACTCACAGTCTGGGGCATC

>FJ469707
TTCTACTGTAACACAACACAACTGTTTAATAGTACTTGGAATGTTACTGAAGGAGAAGGGTCAAATGACACTG
AA---

ACAAATGACACAATCACACTCCCGTGCAGAATAAGACAAATTATAAATATGTGGCAGGAAGTAGGAAAAGCAA
TGTATGCCCCTCCCATCAGAGGACCAATTAGCTGTTCATCAAAT---
ATTACAGGGTTACTGTTAACAAGAGATGGTGGTAATACCACGGGG----------------------------
----

GAAAGTGAGACCTTCAGACCTGGAGGAGGAAATATGAAGGACAATTGGAGAAGTGAATTATATAAATATAAAG
TAGTAAAAATTGAACCATTA---GGAATAGCACCCACCACGGCAAAGAGAAGAGTGGTG--------------
-------------------------CAGAGAGAGAAAAGAGCA---GTGGGA---ATAGGAGCT---------
TTGTTCCTTGGGTTCTTGGGAGCA---GCAGGAAGCACTATG---GGCGCAGCGTCA---
ATGACGCTGACGGTACAA---GCCAGACTA---CTATTG---TCTGGTATAGTGCAACAGCAGAAC---
AATTTG---CTGAGG---GCTATTGAGGCGCAACAGCATCTGTTGCAACTCACAGTCTGGGGCATC
>FJ469716
TTCTACTGTGATACAACAAAACTGTTTAATAGTATCTGGCATAGTAATGATACCTGGACTGGGGCAAATAAC-
----

ACTGAAAAGAATATCACACTCCCATGCAGAATAAAACAAATTGTAAACATGTGGCAGGAAGTAGGAAAAGCAA
TGTATGCCCCTCCTATCGAGGGACAAATTAAATGTACATCAAAT---
ATTACAGGGCTGCTATTAACAAGAGATGGTGGTAATGAGACGAAG----------------------------
----

GAAACTTTCAGACCTGGAGGAGGAGATATGAGGGACAATTGGAGAAGTGAATTATATAAATATAAAGTAGTAA
AAATTGAACCATTA---GGAATAGCACCCACCGAGGCAAAGAGAAGAGTGGTG--------------------
-------------------CAGAGAGAAAAAAGAGCA---GTGGGAGCACTAGGAGTT---------
ATGTTCCTTGGGTTCTTGGGAGCA---GCAGGAAGCACTATG---GGCGCAGCGTCA---
ATGGCGCTGACGGTACAG---ACCAGACAA---TTATTG---TCTGGTATAGTGCAACAGCAAAAC---
AATCTG---CTGAAG---GCTATTGAGGCGCAACAGCATCTGTTGCAACTCACGGTCTGGGGCATC
>FJ469721
TTCTACTGTAATTCAACACAACTGTTTAATAGTACTTGGAATAGTACTACTTGGAATAATACTAATGTGAATG
ATACTATT---
-------------------GGG---
AATGAGACTGACCCAATCATACTCCCATGCAGAATAAAACAAATTATAAACATGTGGCAGGGAGTAGGAAGAG
CAATGTATGCCCCTCCCATCAGTGGACTAATTAGATGTTCATCAAAT---
ATTACAGGACTGCTATTAGTAAGGGATGGTGGTGTTAACAACAGC----------------------------
----

CGCACAAATGAGACCTTTAGACCTTTAGGAGGAAATATGAAGGATAATTGGAGAAGTGAATTATACAAATATA
AAGTAGTAAGAATTGAACCATTA---GGAGTAGCACCCACCAAGGCAAGGAGAAGAGTGGTG-----------
----------------------------CAGAGAGAAAAAAGAGCA---CTGGGA---ATAGGAGCT------
---GTGTTCATTGGGTTCTTGGGAGCA---GCAGGAAGCACTATG---GGCGCAGCGTCA---
GTGACGCTGACGGTACAG---GCCAGACTA---TTGCTG---TCTGGTATAGTGCAACAGCAGAAC---
AACTTG---CTGAGA---GCTATTGAGGCGCAACAACATCTGTTGCAACTCACAGTCTGGGGCATT
>MN516423
TTCTACTGTAATACAACACAACTATTTAATAGTACATGGACATGGAATACTACCGGGGATGGTACCTGGAAG-
----
-GTTACTGAAGGGTCAAAT------
AACACTGCAGAAAACATCACACTCCCATGTAGAATAAAACAAATTATAAATATGTGGCAGGAAGTAGGAAAAG
CAATGTATGCCCCTCCCATCAGAGGAAAAATTAGATGCTCATCAAAT---
ATTACAGGGCTACTATTAACAAGAGATGGGGGTGATACAAACGAA----------------------------
----

GGCAACAATGAGACCTTCAGACCTGCAGGAGGGGATATGAAGGACAATTGGAGAAGTGAACTATATAAATATA
AAGTAGTACAAATTGAACCATTA---GGAGTAGCACCCACCAAGGCAAAGAGAAGAGTGGTG-----------
----------------------------CAGAGAGAAAAAAGAGCT---GTGGGA---CTAGGAGCG------
---TTGTTCCTTGGGTTCCTGGGAGCA---GCAGGAAGCACTATG---GGCGCAGCGTCG---
CTGACGCTGACGGTACAG---GCCAGACAA---TTATTG---TCTGGTATAGTGCAACAGCAAAAC---
AATTTG---CTGCAG---GCTATTGAGGCGCAACAGCGTCTGTTGCAACTCACAGTCTGGGGCATC

>HQ217308
TTCTACTGTAATACAACAAAACTATTTAGTAGTACTTGGAATAATACTTGGAATAGTACTTGGAATAGTACTA
AT---

GAATCAAATGACACAATTACACTCCCATGCAGAATAAAACAAATTATAAACAGATGGCAAGAAGTAGGAAGAG
CAATGTATGCCCCTCCCATCGAGGGACAAATCAGATGTTCATCAAAT---
ATTACAGGGTTGCTATTAACAAGAGATGGTGGCAATGGAGAGAATGAG-------------------------
----

TCTGATACTGAGATCTTCAGACCTGGGGGAGGAGATATGAGGGACAATTGGAGAAGTGAATTGTACAAATATA
AAGTAGTAAAAATTGAGCCATTA---GGAGTAGCACCCACCAAGGCAAAGAGAAGAGTGGTG-----------
----------------------------CAAAGAGAAAAAAGAGCA---ATAGGAACGATAGGAGCT------
---ATGTTCCTTGGGTTCTTGGGAGCA---GCAGGAAGCACTATG---GGCGCAGCGTCA---
ATAACGCTGACGGTACAA---GCCAGACAA---TTATTG---TCTGGTATAGTGCAACAGCAGAAC---
AATCTG---CTGAGG---GCTATTGATGCGCAACAGCATATGTTGCAGCTCACAGTCTGGGGCATC
>JQ403058
TTCTACTGTGATTCAACACAACTGTTTAATAGTACTTGGTCGTCCAATGGTACTTGGACTAATGATACTACAG
GGTTAAAT---
-------------ACAACT------
GATAAATATGATAACTTCACACTCCCATGCAAAATAAAACAAATTATAAACATGTGGCAGAAAGTAGGAAAAG
CAATGTATGCCCCTCCCATTAGCGGACTAATTAGCTGTGAATCAAAT---
ATCACAGGACTGATATTAGTAAGAGATGGTGGTAATAATGCAAACGAGACC----------------------
----

GACAACACCACCGAGATCTTCAGACCTGGAGGAGGAAATATGAAGGACAATTGGAGAAGTGAATTATATAAAT
ATAAAGTAGTAAGAATTGAACCATTA---GGAGTAGCACCCACCAGGGCAAAGAGAAGAGTGGTG--------
-------------------------------CAAAGAGAAAAAAGAGCA---GTGGGAACATTAGGAGCT---
------ATGTTCCTTGGGTTCTTGGGAGCA---GCAGGAAGCACTATG---GGCGCAGCATCA---
ATGACGCTGACGGTACAG---GCCAGACAA---TTATTG---TCTGGTATAGTGCAACAGCAGAAC---
AATTTG---CTGAGG---GCTATTGAGGCGCAACAGCATTTGTTGCAACTCACAGTCTGGGGCATC
>JQ403059
TTCTACTGTAATACAACACAGCTGTTTAATAGTACTTGGAATGTTACTGAAGAGACAAATAGCACTAGA----
----

GGAAATGACACAATCACACTCCAATGCAGAATAAAACAAATTATAAACATGTGGCAGGAAGTAGGAAAAGCAA
TGTATGCCCCTCCCATCAGGGGACAGATTAGATGTTCATCAAAT---
ATTACAGGGTTGCTACTAACAAGAGATGGTGGTGGTAACAATAAC----------------------------
----

AACAGCGAGGAGGTCTTCAGACCTGGAGGAGGAGATATGAGGGACAATTGGAGAAGTGAATTATATAAATATA
AAGTAGTAAAAGTTGAACCATTA---GGAGTAGCACCCACCAAGGCAAAGAGAAGAGCGGTG-----------
----------------------------CAGAGAGAAAAAAGAGCA---GTGGGG---ATAGGAGCT------
---ATGTTCCTTGGGTTCTTGGGAGCA---GCAGGAAGCACTATG---GGCGCAGCGTCA---
CTAACGCTGACGGTACAG---GCCAGACTA---TTATTG---TCTGGTATAGTGCAACAGCAGAAC---
AATTTG---CTGAAG---GCTATTGAGGCGCAACAGCATATGTTGCAACTCACAGTCTGGGGCATC
>JQ403060
TTCTACTGTAATTCAACACCACTGTTTAATAGTAATTGGTCAGCTAGTAGTCCTTGGAATAGTAGTACT----
----

GAACGAGGAAATATCACACTCCAATGCAGAATAAAACAAATTGTAAACATGTGGCAGGAAGTAGGAAGAGCAA
TGTATGCACCTCCCATCAAAGGAGTAATTAAATGTACATCAAAT---
ATTACAGGACTGATATTAACAAGAGATGGTAGTAATAACACTAGT----------------------------
----

GAAACTGAAACCTTCAGACCTGGAGGAGGAAATATGAAGGACAATTGGAGAAGTGAATTATATAAATATAAAG
TAGTAAAAATTGAACCATTA---GGAGTAGCACCCACTACAGCCAAGAGAAGAGTGGTG--------------
-------------------------CAGAGAGAAAAAAGAGCA---GTGGGA---ATAGGAGCT---------
GTGTTCCTTGGGTTCTTGGGAGCA---GCAGGAAGCACTATG---GGCGCAGCGTCA---
ATAACGCTGACGGTACAG---GCCAGACTA---TTATTG---TCTGGTATAGTGCAACAGCAGAAC---
AATCTG---CTGAGG---GCTATTGAGGCGCAACAGCACCTGTTGCAACTCACAGTCTGGGGCATT

>JQ403064
TTCTACTGTAATACAACACAACTGTTTAACACTAATTGGACTAGGACTAATGCTAATTGGACTGTTACTAATT
GGACTGAT---

GCTGGAGTAAATATTACACTCCCATGCAGAATAAAACAAATTATAAATATGTGGCAGGAAGTAGGAAAAGCAA
TGTATGCCCCTCCCATCAGAGGACAAATTAGATGCTCATCAAAT---
ATTACAGGGTTGCTATTAACAAGAGATGGTGGTAATATCACGAAC----------------------------
----

GAGACCACCGAGATCTTCAGGCCTGGAGGAGGAAATATGAGGGACAATTGGAGAAGTGAATTATATAAATATA
AAGTAGTAAAAATTGAACCATTA---GGAATAGCACCCACCAAGGCAAGGAGAAGAGTGGTG-----------
----------------------------CAAAGAGAAAAACGAACA---GTGGGA---CTAGGAGCT------
---TTGTTCATTGGGTTCTTGGGAGCA---GCAGGAAGCACTATG---GGCGCAGCGTCA---
CTGACGCTGACGGTACAA---GCCAGACTA---TTATTG---TCTGGTATAGTGCAACAGCAGAAC---
AACCTG---CTGAAG---GCTATTGAGGCGCAACAGCATCTGTTGCAACTCACAGTCTGGGGCATC
>JQ403077
TTCTACTGTGATACAACAGGACTGTTTAACAGCACTTGGAACTCAACAGAATGGGATAATAGCACAGATAAAA
AAGTCACA---
-------------ATCAAT------
AGCACAGATAAAAAAGTCACAATCAAATGTAGAATAAAACAAATTATAAACTTGTGGCAGGAAGTAGGAAAAG
CAATGTATGCCCCTCCCATCAAAGGACTAATTAGATGTAGTTCAAAT---
ATTACAGGGCTGCTGTTAACAAGAGATGGTGGTAACAGTAACAATAACAGTAAC-------------------
----

AACGGGACCGAGGTCTTTAGACCTGGAGGAGGAAATATGAAAGACAATTGGAGAAGTGAATTATATAAATATA
AAGTAGTGAAAATTGAGCCATTA---GGAATAGCACCCACCAAGGCAAAGAGACGAGTGGTG-----------
----------------------------CAGAGAGAAAAAAGAGCA---GTAACG---TTAGGAGCT------
---ATGTTCCTTGGGTTCTTGGGAACA---GCAGGAAGCACTATG---GGCGCAGCGTCA---
TTGACACTGACGGTACAG---GCCAGACTA---TTATTG---TCTGGTATAGTGCAACAGCAGAAT---
AATCTG---CTGAGA---GCTATTGAGGCGCAACAGCATATGTTGCAACTCACAGTCTGGGGCATT
>JQ403092
TTCTACTGTAATACAACACAGTTATTTAATAGTACTTGGGGTAATGGTACTTGGCAAGGGTCAAAT-------
----

GGGAATGATACTATCACACTCCCGTGCAGAATAAAACAGATTATAAACAGGTGGCAAGAAGTAGGAAAAGCAA
TGTATGCCCCTCCCATCAAAGGAATAATTAGATGTTCATCAAAA---
ATTACAGGGTTAATATTAACAAGAGATGGTGGTAACGTTAACAGT----------------------------
----

AGTGACCCCGAGATCTTCAGACCTGGAGGAGGAGATATGAGGGATAATTGGAGAAGTGAATTATATAAATATA
AAGTAGTAAGAATTGAGCCATTA---GGAATAGCACCCACCAAGGCAAAGAGAAGAGTGGTG-----------
----------------------------CGGAGAGAAAAAAGAGCA---GTAACA---CTGGGAGCT------
---ATGTTCCTTGGGTTCTTGGGAGCA---GCAGGAAGCACTATG---GGCGCAGCGTCA---
ATAACGCTGACGGTACAG---GCCAGACAA---TTATTG---TCTGGTATAGTGCAACAGCAGAAC---
AATTTG---CTGAGG---GCTATTGAGGCGCAACAACATATATTGCAACTCACAGTCTGGGGCATC
>JQ403093
TTCTACTGTAATACAACAGAATTGTTTAATGGTACTTTGAATAACACTGGGTGGAGTAATAGTAGCACTGGG-
----

GTAAATGGAACTATCATACTCCCATGCAGAATAAAACAAATTATAAACCTGTGGCAGGAAGTAGGAAAAGCAA
TGTATGCCCCTCCCATCAGAGGACAAATTAACTGTTCATCAAAG---
ATTACAGGGCTGTTATTAACAAGAGATGGTGGTACTAATAACGAGACC-------------------------
----

ACGAACGCCACCACCGAGATCTTTAGACCTGGAGGAGGAGATATGAGGGACAATTGGAGAAGCGAATTATATA
AATATAAAGTAGTAAAAATTGAACCATTA---GGAGTAGCACCCACCAAGGCAAAGAGAAGAGTGGTG-----
----------------------------------CAGAGAGAAAAAAGAGAA---
GTGGGAACACTGGGAGCT---------ATGTTCCTTGGGTTCTTGGGAGCA---GCAGGAAGCACTATG---
GGCGCAGCGTCA---CTGACGCTGACGGTACAG---GCCAGACTA---TTATTG---

TCTGGTATAGTGCAACAGCAGAAT---AATTTG---CTGAGG---
GCTATTGAGGCGCAACAGCATCTGTTGCAACTCACGGTCTGGGGCATC
>JQ403096
TTCTACTGTGATACAACACGGCTGTTTAATACTACCTGGAATGATACAAATAACAGTGGACTT----------
----

AACAATGAAACTATCACCCTCCCATGCAGAATAAGACAAATTATAAACAGGTGGCAGGAAGTAGGAAAAGCAA
TGTATGCCCCTCCCATCAGCGGACAAATTAATTGTTCATCAAAA---
ATTACAGGGCTGCTGTTAACAAGAGATGGTGGTACTAATGACACAAATAAC----------------------
----

ACAAATAAGACCGAGACCTTCAGACCAGGGGGAGGAGATATGAGGGACAATTGGAGAAGTGAATTATATAAAT
ATAAAGTAGTAAAAATTGAGCCATTA---GGAGTAGCACCCACCAAGGCAAAGAGAAGAGTGGTG--------
-------------------------------CAAAGAGAAAAAAGAGCA---GTGGGAGTGATAGGAGCT---
------ATGTTCCTTGGGTTCTTGGGAGCA---GCAGGAAGCACTATG---GGCGCAGCGTCA---
ATGACGCTGACGGTACAG---GCCAGACAA---TTATTG---TCTGGTATAGTGCAACAGCAGAAT---
AACTTG---CTGAGG---GCTATTGAGGCGCAACAGCACCTGTTGCAGCTCACAGTCTGGGGCATC
>HQ217347
TTCTACTGTAATTCAACAGTACTATTTAATAGTACTTGGAATTCAACAACTTGGAATTCAACACTGTTTAATA
GTACTGGG---
-------------AATGAG------
ACTAGTGGAGAAAATATCACCCTCAAATGCAGAATAAAACAGATTATAAACATGTGGCAAGAAGTAGGAAAAG
CAATGTATGCCCCTCCCATCAAAGGAAAAATTAAATGTAAATCAAAT---
ATTACAGGGCTGCTTTTAACAAGAGATGGGGGTAATGGGGGTAATGAT-------------------------
----

ACGAACAAGGAGATCTTCAGACCTGGAGGAGGAGATATGAGGGACAATTGGAGAAGTGAATTATATAAATATA
AAGTAGTAAGAATTGAACCATTA---GGAATAGCGCCCACCAAGGCAAAGAGAAGAGTGGTG-----------
----------------------------CAAAGAGAAAAAAGAGAT---TTGGGA---ATAGGAGCA------
---TTGTTCCTTGGGTTCTTGGGAGCA---GCAGGAAGCACTATG---GGCGCAGCGTCA---
ATGACGCTGACGGTACAG---GCCAGACAA---TTATTG---TCTGGTATAGTGCAACAGCAAAAC---
AATCTG---CTGAGG---GCTATTGAGGCGCAACAACATCTGTTGCAACTCACAGTCTGGGGCATC
>HQ217386
TTCTACTGTAACTCATCAAAACTATTTAATAGTACTTGGAATGTTAATAATACTTGGAATAATACTGGAGAAA
AA---

GAAAATGACACAATAATACTCCCATGCAGAATAAAACAGATTATAAACATGTGGCAGGAAGTAGGAAAAGCAA
TGTATGCCCCTCCCATCAGAGGACAAATTAAATGTTCATCAAAT---
ATTACAGGGTTGCTGTTAACAAGAGATGGAAGAAATAGTGAGGAC----------------------------
----

AATGACACTGAGACCTTCAGACCTGGAGGAGGGGATATGAGGGACAATTGGAGAAGTGAATTATATAAATATA
AAGTAGTAAAACTTGAACCATTA---GGAATAGCACCCACCAGGGCAAAGAGAAGAGTGGTG-----------
----------------------------CAGAGAGAAAAAAGAGCA---ATAGGA---ATAGGGGCT------
---ATGTTCCTCGGGTTCTTGGGAGCA---GCCGGAAGCACTATG---GGCGCAGCGTCA---
ATGACACTGACGGTACAG---GCCAGACAA---TTATTG---TCTGGTATAGTGCAACAGCAGAAC---
AATCTG---CTGAAG---GCTATTGAGGCGCAACAACATCTGTTGCAACTCACTGTCTGGGGCATT
>KC312583
TTCTACTGTAATACAACAAAGCTGTTTAATAGCACTTGGTTGTCTAATGATACTACTACTTGGAATGAGACTA
AT---

GACAACAGCACAATCATACTCCCGTGCAGAATAAGACAAATTATAAATAGATGGCAGGAAGTAGGAAAAGCAA
TGTATGCCCCTCCCATCAGCGGTCTAATTAAATGTACATCAAAT---
ATTACAGGGCTACTATTAACAAGAGATGGAGGTAATAATAATAATAATAGT----------------------
----

AATACCTCAGAAGAGATCTTCAGACCTGGAGGAGGGGATATGAGGGACAATTGGAGAAGTGAATTATATAAAT
ACAAAGTAGTCAAAATTGAGCCATTA---GGATTAGCACCCACCAAGGCAAAGAGAAGAGTGGTG--------
-------------------------------CAGAGAGAAAAAAGAGCA---ATAACG---ATGGGAGCT---
------TTGTTCCTTGGGTTCCTGGGAGCA---GCAGGAAGCACTATG---GGCGCAGCGTCA---

TTGACGCTGACGGTACAG---GCCAGACTA---TTATTG---TCTGGTATAGTGCAACAGCAGAAC---
AATCTG---CTGAGA---GCTATTGAGGCGCAACAGCATCTGTTGCAACTCACAGTCTGGGGCATC
>HQ217430
TTCTACTGTAATTCAACACAGCTGTTTAATAGTACTTGGACTTTTAATAGTAATAATACTACCTCTAGCAATG
GA---

AATAGCAATGGAAATATCATACTCCCGTGCAGAATAAAACAAATTATAAATAGGTGGCAGGAAGTAGGAAAAG
CAATGTATGCCCCTCCCATCAGTGGATTAATTAATTGCTCATCAAAT---
ATTACAGGGTTACTATTAACAAGAGATGGTGGTGATAATGAGACCAAC-------------------------
----

AACACCACCGAGACCTTCAGACCTGGGGGAGGAGATATGAGGGACAATTGGAGAAGTGAATTATATAAATATA
AAGTAGTAAAAATTGAGCCATTA---GGGATAGCACCCTCCAGGGCAAAGAGAAGAGTGGTG-----------
----------------------------CAGAGAGAAAAAAGAGCA---GTGGGAGTAATAGGAGCT------
---ATGTTCCTTGGGTTCTTGGGAGCA---GCAGGAAGCACTATG---GGCGCAGCGTCG---
ATGACGCTGACGGTACAG---GCCAGACAA---TTATTG---TCTGGTATAGTGCAACAGCAGAAC---
AATTTG---CTGAAA---GCTATTGAGGCGCAACAACATTTGTTGCAACTCACAGTCTGGGGCATC
>KM259264
TTCTACTGTAATACAACAGAATTGTTTAATAGTACTTGGAATGGTACTGTG----------------------
----
-------AACGAGTCAAAC------
AACACTGGAGAAACTATCACACTCCCATGCAGAATAAAACAAATTATAAACATGTGGCAGGAAGTAGGAAAAG
CAATGTACGCCCCTCCCATCAAAGGACAAATTAAATGTTCATCAAAT---
ATTACAGGGCTGCTGTTAACACGAGATGGTGGT--

ACTAATAAGACTAAT---
GATACTGAGACCTTTAGACCTGGAGGGGGAAATATGAAGGACAATTGGAGAAGTGAATTATATAAATATAAAA
TAGTAAAAATTGAACCATTA---GGAATAGCACCCACCAAGGCAAAGAGAAGAGTGGTG--------------
-------------------------CAGAGAGAAAAAAGAGCA---GTGGGAACATTAGGAGCT---------
ATGTTCCTTGGGTTCTTGGGAGCA---GCAGGAAGCACTATG---GGCGCAGCGTCA---
CTGACGCTGACGGTACAG---GCCAGACTA---TTATTG---TCTGGTATAGTGCAACAGCAAAAC---
AATTTG---CTGAGA---GCTATTGAGGCGCAACAACATATGTTGCAACTCACAGTCTGGGGCATC
>KM259566
TTCTACTGTAACTCAACACCACTGTTTAACAGTACTTGGTTGTCTAATGGTACT-------------------
----
-TTGAATGAAACTGAA---------
GGCAACGGGGGCAACATCACCCTCCTATGCAGAATAAAACAAATTATAAATGTATGGCAGGAAGTAGGAAAAG
CAATGTATGCCCCTCCTATCAGAGGACAAATTAAATGTACATCAAAT---
ATTACAGGACTGCTATTAACAAGAGATGGTGGG--

AACAATGGGTCTGAA---
CCCACCGAGACCTTCAGACCTGGAGGGGGAAATATGAAGGACAATTGGAGAAGTGAATTATATAAATATAAGG
TAGTAAAAGTTGAACCATTA---GGATTGGCACCCACCAAGGCAAAGAGAAGAGTGGTG--------------
-------------------------CAGAGAGAAAAAAGAGCAATAGGGGGA---CTAGGAGCT---------
TTGTTCCTTGGGCTCTTGGGAGCA---GCAGGAAGCACTATG---GGCGCAGCGTCA---
CTAACGCTGACGGTACAG---GCTAGACAA---TTATTG---TCTGGTATAGTGCAACAGCAAAAC---
AACTTG---CTGAGG---GCTATTGAGGCGCAACAGCATCTGTTGCAACTCACAGTCTGGGGCATC
>HQ217509
TTCTACTGTAATACAACACAACTGTTTAATAGTACTTGGGGGTCAAATAACACTGAAGAGCCAAATAACACTG
GA---

GGAAATGACACAATCACACTCCCATGTAGACTAAAACAAATTATAAACATGTGGCAGGAAGTAGGAAAAGCAA
TGTATGCCCCTCCCATCAGAGGACAAATTAGATGTTCATCAAAT---
ATTACAGGACTGTTATTAACAAGAGATGGGGGGAACAGGAATGAGACTAAC----------------------
----

GGGAACGGGACTGAGATCTTCAGGCCTGGAGGAGGAGATATGAGGGACAATTGGAGAAGTGAATTATACAAAT
ATAAAGTAGTAAAAATTGAACCATTA---GGAGTAGCACCCACTAAGGCAAAAAGAAGAGTGGTG--------
-------------------------------CAAAGAGAAAAAAGAGCA---GTGGGA---TTAGGAGCT---
------ATGTTCCTTGGGTTCTTGGGAGCA---GCAGGAAGCACTATG---GGCGCAGCGTCA---

ATGACGTTGACGGTACAG---GCCAGACTG---TTATTG---TCTGGTATAGTGCAACAGCAGAAC---
AATCTG---CTGAGG---GCTATTGAGGCGCAACAACATCTGTTGCAGCTCACAGTCTGGGGCATC
>HQ217601
TTCTACTGTAATACAACAAAACTGTTTAATAGTACTTGGAATAGGACTACTAGGGAAAGCAATGTTACTGAAG
GGTCA--

GATAACAATGAAACTATCACACTCCAATGCAGGATAAAACAGATTATAAACATGTGGCAGGAAGTAGGAAAAG
CCATATATGCCCCTCCTATCAGCGGACCGATTAGATGTTCATCAAAT---
ATCACAGGGCTGCTATTAACAAGAGATGGAGGTAATACAAATAAGAGCAAT----------------------
----

GAGAACGAGACTGAGACCTTCAGACCTGGAGGAGGAGATATGAGGGACAATTGGAGAAGTGAATTATATAAAT
ATAAAGTAGTACAAATTGAACCATTA---GGAGTAGCACCCACCAAGGCAAAGAGAAGAGTGGTG--------
-------------------------------CAGAGAGAAAAAAGAGCA---GTGGGA---ATAGGAGCT---
------CTGTTCCTTGGGTTCTTGGGAGCA---GCAGGAAGCACTATG---GGCGCAGCGTCA---
ATAACGCTGACGGTACAG---GCCAGACAA---TTATTG---TCTGGTATAGTGCAACAGCAGAAC---
AACTTG---CTGAGG---GCTATTGAGGCGCAACAGCATATGTTGCAACTCACAGTCTGGGGCATC
>GQ256646
TTCTATTGTAATACAGCACAACTGTTTAATAGTACTTGGCCTGAGAATAGTACTTGGCCTGGGAATAATGGTA
CTACAGAGTCA--
-------AATAACAACACT------
GGAGAAAATGACACAATCACACTCCCATGCAAAATAAAACAAATTATAAACATGTGGCAGAAAGTAGGAAAAG
CAATGTATGCCCCTCCCATCAAAGGGATAATTAAATGTTCATCAAAT---
ATTACAGGATTGCTATTAGTAAGAGATGGTGGTAACACCAGCATG----------------------------
----

ACCAACGAGACTTTCAGACCTGGAGGAGGAGATATGAGAGACAATTGGAGAAGTGAATTATATAAATATAAAG
TAGTAAAAATTGAACCGTTA---GGAGTAGCACCCACCAAGGCAAAGAGAAGAGTGGTG--------------
-------------------------CAGAGAGAAAAAAGAGCA---GTGGGA---TTAGGAGCT---------
ATGTTCCTTGGGTTCTTGGGAGCA---GCAGGAAGCACTATG---GGCGCAGCGTCA---
CTATCGCTGACGGTACAG---GCCAGAAAC---TTATTG---TCTGGTATAGTGCAACAGCAAAAC---
AATTTG---CTGAGG---GCTATTGAGGCGCAACAGCATATGTTGCAACTCACAGTCTGGGGCATC
>JF689895
TTCTACTGTAATACAACACAACTGTTTAATAGTACCTGGAATAATAATAATACCTGGACTGGTAACACTGAA-
----

GGAAATGACACACTCATACTCCCATGCAGAATAAAACAAATTATAAACAGGTGGCAAGAAGTAGGAAAAGCAA
TGTATGCCCCTCCCATCAGAGGACCAATTAACTGTTTATCAAAT---
ATTACAGGGTTGCTATTAACAAGAGATGGAGGTGATAATAACAAC----------------------------
----

ATGAGTGAGATTTTCAGACCTGCAGGAGGAGATATGAGGGACAATTGGAGAAGTGAATTATATAAGTATAAAG
TAGTAAAAATTGAACCACTA---GGAATAGCACCTACCAAGGCAAAGAGAAGAGTGGTG--------------
-------------------------CAGAGAGAAAAAAGAGCA---GTGGGA---ATAGGAGCT---------
GTGCTCCTTGGGTTCTTAGGAGCA---GCAGGAAGCACTATG---GGCGCAGCGTCA---
ATGACGCTGACGGTACAA---GCCAGGCTA---TTATTG---TCTGGTATAGTGCAACAGCAGAGC---
AATTTG---CTGAGG---GCTATTGAGGCGCAACAGCATCTGTTGCAACTCACAGTCTGGGGCATT
>JF689896
TTCTACTGTAATTCAACACAACTGTTTAATAGTACTTGGAGGTTAAATAACACTGAAGGAAATAGCACTGGGT
CAGGTAAC---
-------------------ACT---
GAAGGAAATAACACAATCACACTCCCATGCAAAATAAAACAAATTGTAAACATGTGGCAGAAAGTAGGAAAAG
CAATGTATGCCCCTCCCATCAAAGGACAAATTAGATGTTCATCAAAT---
ATTACAGGGCTGCTATTAACAAGAGATGGGGGTAAAAACAACAGT----------------------------
----

GGGACCGAGACCTTCAGACCTGGAGGAGGAGATATGAGGGACAATTGGAGAAGTGAATTATATAAATATAAAG
TAGTAAAGCTTGAACCATTA---GGAGTAGCACCCACCAGGGCAAAGAGAAGAGTGGTG--------------
-------------------------CAGAGAGAAAAGAGAGCA---ATAGGA---ATAGGAGCT---------
ATGTTCCTTGGGTTCTTGGGAGCA---GCAGGAAGCACTATG---GGCGCAGCGTCG---

ATGACGCTGGCGGTACAG---GCCAGACAA---TTATTG---TCTGGTATAGTGCAACAGCAGAAC---
AATTTG---CTGAAG---GCTATTGAGGCGCAACAGCATCTGTTGCAACTCACAGTCTGGGGCATC
>JX864023
TTCTATTGTAATACAACAAAGCTGTTTAATAGTACTTGGGTGTTTAATGGTACTTGGACTATTAATGGGAGTG
TT---

ACTGAAGGAAATATCACACTCCCGTGCAGAATAAAACAAATTATAAACAGATGGCAGGAAGTAGGAAAAGCAA
TGTATGCCCCTCCCATCAGAGGACAAATTAGATGTATATCAAAT---
ATTACAGGGTTACTATTAACAAGAGATGGGGGTACTAAAAATAATAGTAATAAC-------------------
----

ACCAGTGAACCCGAGATCTTCAGACCAGGAGGAGGGGATATGAGAGACAATTGGAGAAGTGAATTATATAAAT
ATAAAGTAGTAAGAATTGAGCCATTA---GGAGTAGCACCCACCAAGGCAAAGAGAAGAGTGGTG--------
-------------------------------CAAAGAGAAAAAAGAGCAGCAGTT------ATGGGAGCT---
------ATGTTCCTTGGGTTCTTGGGAGCA---GCAGGAAGCACTATG---GGCGCAGCGGCA---
ATAACGCTGACGGTACAG---GCCAGACAA---TTATTG---TCTGGTATAGTGCAACAGCAAAAT---
AATTTG---CTGAGA---GCTATTGAGGCGCAGCAGCATATGCTGCAACTCACAGTCTGGGGCATT
>JX864024
TTCTACTGCAATACAACACAACTGTTTAATAGTACTTGGAACTCTAATAGTACTTTGAAC-------------
----

AGTACTGGGAATATCACACTCCCATGTAGAATAAAACAAATTGTAAACATGTGGCAGGAAGTAGGAAAAGCAA
TGTATGCCCCTCCCATCCAAGGACTAATTAGATGCTCATCAAAT---
ATTACAGGGCTGTTATTAGTAAGAGATGGTGGTAACAGTAACAGA----------------------------
----

ACCAATGAAACCTTCAGACCTGGAGGAGGAAATATGAAGGACAATTGGAGAAATGAATTATATAAATATAAAG
TAGTAAAAATTGAACCATTA---GGAGTAGCACCCACCAGGGCAAAGAGAAGAGTGGTG--------------
-------------------------CAGAGAGAAAAAAGAGCA---GTGGGA---ATAGGAGCT---------
GTGTTCCTTGGGTTCTTAGGAGCA---GCAGGAAGCACTATG---GGCGCAGCGTCA---
GTGACGCTGACGGTACAG---GCCAGACAA---TTATTG---TCTGGTATAGTGCAGCAGCAGAAC---
AATCTG---CTGAGG---GCCATTGAGGCGCAACAGCATCTGTTGCAACTCACAGTCTGGGGCATT
>JX864025
TTCTACTGCAATACAACAAAGCTGTTTAACAGTACGTGGACTAATCGGACTACAACTAACGAGACAACTAGT-
----

AGTAATGAAACTATTACAATCCCATGCAGAATAAAACAAATTATAAACATGTGGCAGGAAGTAGGAAAAGCAA
TGTATGCCCCTCCTATCAGGGGACCAATTAGATGTTCATCAAAT---
ATTACAGGACTAATATTACTAAGAGATGGTGGTAATAGCAATAACACAAACAAT------------AAC----
----

ACAAACAAGACTGAGATCTTCAGACCTGGAGGAGGAGACATGAGGGACAATTGGAGAAGTGAATTATATAAAT
ATAAAGTAGTACAACTTGAACCATTA---GGAATAGCACCCACCAGGGCAAAGAGAAGAGTGGTG--------
-------------------------------CAGAGAGAAAAAAGAGCA---ATAGGA---ATAGGAGCC---
------TTGTTCCTTGGGTTCTTGGGAACA---GCAGGAAGCACTATG---GGCGCAGCGTCA---
ATAACGCTGACGGTACAG---GCCAGACAA---TTATTG---TCTGGTATAGTGCAACAGCAGAAC---
AATCTG---CTGAGG---GCTATTGAGGCGCAACAACATCTGTTGCAACTCACAGTCTGGGGCATC
>HM638517
TTCTACTGTAATTCAACAAAACTGTTTAATAGTACCTGGAATGGGACTAGTACTGAAGAGAGCAAGGGA----
----

GAAAATGACACAATCACACTCCCATGCAGAATAAAACAATTTATAAACATGTGGCAGAGAGTAGGAAAAGCAA
TGTATGCCCCTCCTATCAGCGGAAAAATTAGATGTTCATCAAAT---
ATTACAGGGCTGTTATTAACAAGAGATGGTGGCATTAACACCACCAACGAGACCTTCAGACCTGGAGGAGGAA
ATATGAGGGACAATTGGAGAAGTGAATTATATAAATATAAAGTAGTAAGAGTTGAACCATTAGGAGTAGCACC
TACCAAGGACACCACCAACGAGACCTTCAGACCTGGAGGAGGAAATATGAGGGACAATTGGAGAAGTGAATTA
TATAAATATAAAGTAGTAAGAGTTGAACCATTA---GGAGTAGCACCTACCAAGGCAAAGAGAAGAGTGGTG-
--------------------------------------CAGAGAGAAAAAAGAGCA---GTGACA---
TTTGGAGCT---------GTGTTCCTTGGGTTCTTGGGAGCA---GCAGGAAGCACTATG---
GGCGCAGCGTCA---GTGACGCTGACGGTACAG---GCCAGACAA---TTATTG---

TCTGGTATAGTGCAACAGCAAAGT---AATTTG---CTGAGG---
GCTATTGAGGCGCAACAGCATCTGTTGCAACTCACAGTCTGGGGCATC
>MF499166
TTCTACTGTAATACAACAAAACTGTTCAACACGAATTTG----------------------------------
----
----ACACAACTGTCTAAT------
AACACTAAAGAAAACATCACACTCCCATGTAGGATAAAACAAATTATAAACATGTGGCAGGAAGTAGGAAAAG
CAATGTATGCCCCTCCTATCAGTGGACTAATTAGATGCTCATCAAAT---
ATTACAGGGCTGCTGTTAACAAGAGATGGTGGTAATAACAACAAC----------------------------
----

AGCCAAAATGAGACCTTCAGACCTATAGGGGGAAATATGAGGGACAATTGGAGAAGTGAATTATATAAATATA
AAGTAGTAAGAATTGAACCAGTA---GGAATAGCACCCACCAAGGCAAAGAGAAGAGTGGTG-----------
----------------------------CAGAGAGAAAAAAGAGCA---GTGGGA---ATAGGAGCT------
---ATGTTCCTTGGGTTCTTGGGAGCA---GCAGGAAGCACTATG---GGCGCAGCGTCA---
GTGACGCTGACGGTACAG---GCCAGACAA---TTATTG---TCTGGTATAGTGCAACAGCAGAAC---
AATCTG---CTGAGG---GCGATTGAGGTGCAACAGCATATGTTGCAACTCACAGTCTGGGGCATC
>KR423340
TTCTACTGTAACTTAACAAAACTATTTAATAGTACTTGGCCTAATAATGATACTTCAAAGTTTAATAGTACTG
GG---

GGTAATGATACTATCAGACTCCCATGTAGAATAAAACAAATTATAAACATGTGGCAGGAAGTAGGAAAAGCAA
TGTATGCCCCTCCCATCGAAGGAGAAATTACATGTAATTCAAGT---
ATTACAGGGCTGCTATTAACAAGAGATGGTGGTAATACTAATGACACT-------------------------
----

AAGGTGAATGAGACCTTCAGGCCTGGAGGAGGAGACATGAGGGACAATTGGAGAAGTGAATTATATAAATATA
AAGTAGTAAGAATTGAACCATTA---GGAATAGCACCCACCAAGGCAAGGAGAAGAGTGGTG-----------
----------------------------CAGAGAGAAAAAAGAGCA---GTGGGA---ATAGGAGCT------
---GTGTTCATTGGGTTCTTGGGAGCA---GCAGGAAGCACTATG---GGCGCAGCGTCA---
GTGGCGCTGACGGTACAG---ACCAAACAG---TTGCTG---TTTGGGATAGTGCAACAGCAAAAC---
AACTTG---CTGAGG---GCTATTGAGGCGCAACAGCATATGTTGCAACTCACAGTCTGGGGCATC
>HM638584
TTCTACTGCAATACAACACAGCTGTTTAATAGTACTTGGTATAGTAATGAGACTGAAGAATTAAATAACACAG
GAGTG--

AATAGCACAGAAACTATCACACTCCCATGCAAGATAAAGCAAATTATAAACATGTGGCAAGGAGTAGGAAGAG
CAATGTATGCCCCTCCCATCAGTGGACAAATTAATTGTTCATCAAAT---
ATTACAGGGCTGCTACTACTAAGAGATGGTGGTAATATTAATGACACG-------------------------
----

AGAGACAATGAAACCTTCAGACCTGGAGGAGGAAATATGAGGGACAATTGGAGAAGTGAATTATATAAATATA
AAGTAGTACAAATTGAACCATTA---GGAGTAGCACCCACCAAAGCAAGGAGAAGAGTGGTG-----------
----------------------------CAGAGAGAAAAAAGAGCA---GCAGTGGGAATAGGAGCT------
---TTGCTCATTGGGTTCTTGGGAACA---GCAGGAAGCACTATG---GGCGCAGCGTCA---
ATAACGCTGACGGTACAA---GCCAGACAA---TTATTG---TCTGGTATAGTGCAGCAGCAGAAC---
AATCTG---CTGAGA---GCTATTGATGCGCAACAACATCTGTTGCAGCTCACGGTCTGGGGCATC
>KM354155
TTCTACTGTAATTTAACACAACTGTTTAATAAAACATTTAATAACACCGGGGAT-------------------
----
-GATACTGACAACAGT---------
GAAGGTAATGACACAATAATACTCCCGTGCAGAATAAAACAAATTATAAACATGTGGCAGGAAGTAGGAAAAG
CAATGTATGCCCCTCCCATCAGAGGACAAATTAAATGTTCATCAAAT---
ATTACAGGGCTACTATTAACAAGAGATGGTGGT--

GTTGTGGAGGACAAT---
AACACTGAGACCTTCAGACCTGCAGGAGGAGATATGAGGGACAATTGGAGAAGTGAATTATATAAATATAAAG
TAGTAAAAATTGAACCATTA---GGAGTAGCACCCACCAGGGGAAAGAGACGGGTGGTG--------------
-------------------------CAAAGAGAAAAAAGAGCAGTAGTGGGA---ATAGGAGCT---------
GTGCTCTTTGGGTTCTTGGGAGCA---GCAGGAAGCACTATG---GGCGCAGCGGCA---

GTAACGCTGACGGTACAA---GCCAGACAA---TTATTG---TCTGGTATAGTGCAACAGCAGAAC---
AATCTG---CTGAGG---GCTATTGAGGCGCAACAGCATATGTTGCAACTCACAGTCTGGGGCATT
>KM354232
TTCTACTGTGATACAACAGCATTGTTTAATAATACTTGGAATAATAATGATACT-------------------
----
-TGGAAGGGTACTAGA---------
GCACCAAATGACACCATCACACTCCCATGTAGAATAAAACAAATTATAAACATGTGGCAGGAAGTAGGAAAAG
CAATGTATGCCCCTCCCATTAGAGGACGAATTAGATGTGTATCAAAT---
ATTACAGGACTACTATTAATAAGGGATGGTGGT--

AATACTAACACAACAAAC---
AAGTCTGAAACCTTCAGACCTGGAGGAGGAAATATGAAAGACAATTGGAGAAGTGAATTATATAAATATAAAG
TAGTAAAAATTGAACCATTA---GGAGTAGCACCCACCAAGGCAAAGAGAAGAGTGGTG--------------
-------------------------CAAAGAGAAAAAAGAGCA---GTGGGAACAATAGGAGCT---------
ATGTTCCTTGGGTTCTTGGGAGCA---GCAGGAAGCACTATG---GGCGCAGCGTCA---
CTGACGCTGACGGTACAG---GCCAGACTA---TTGTTG---TCTGGTATAGTGCAACAGCAGAAC---
AATCTG---CTGAGA---GCTATTGAGGCGCAACAGCACATGTTGCAACTCACAGTCTGGGGCATC
>KM354355
TTCTATTGTAATACAACACAACTGTTTAATAGTACTTGGAATGAGACT-------------------------
----
-------GGAGAGCCAGAT------
AACATTGTAAAAGAGATCATACTCCCATGCAGAATAAAACAAATTATAAACATGTGGCAGGGAGTAGGAAAGG
CAATGTATGCCCCTCCCATCAGAGGACGAATTAGCTGTGTATCAAAT---
ATTACAGGGCTGCTACTAGTAAGAGACGGAGGT--

-
AACGAGACTAACGGGAATGAGACTTTCAGACCTGGAGGAGGAAATATGAAGGACAATTGGAGAAGTGAATTAT
ATAAATATAAAGTAGTAGAAGTTGAACCATTA---GGAATAGCACCCACTAGGGCAAGGAGAAGAGTGGTG--
-------------------------------------CAGAGAGAAAAAAGAGCA---GTGACA---
TTAGGAGCT---------CTGTTCCTTGGGTTCTTGGGAGCA---GCAGGAAGCACTATG---
GGCGCAGCGTCA---GTGACGCTGACGGTACAG---GCCAGACAA---TTGTTG---
TCTGGTATAGTGCAACAGCAAAAC---AATTTG---CTGAGA---
GCTATTGATGCGCAACAACATATGTTGCAACTCACAGTCTGGGGCATC
>JN687773
TTCTACTGTAATACATCACAGCTGTTTAATACTACTTGGAATGATACTAATAGCACCAGGTATGAG-------
----

AATAACAGCAATATCACACTCCCATGCAGAATAAAACAAATTATAAACAGATGGCAGGAAGTAGGAAAAGCAA
TGTACGCCCCTCCCATCGCAGGACAAATTAGCTGTACATCAAAT---
ATTACAGGGGTGCTATTAACAAGAGATGGTGGTATCAATGGGAGC----------------------------
----

AACGAGACCGAGGTCTTCAGACCTGGAGGGGGAGATATGAGGGACAATTGGAGAAGTGAGTTATATAAATATA
AAGTAGTAAAAATTGAACCATTA---GGAATAGCACCCACCAAGGCAAAGAGAAGAGCGGTG-----------
----------------------------CAGAGAGAAAAAAGAGCA---GTGACA---TTCGGAGCT------
---ATGTTCCTTGGGTTCTTGGGAGCA---GCAGGAAGCACTATG---GGCGCAGCATCA---
ATGACGCTGACGGTACAG---GCCAGACTA---TTATTG---TCTGGTATAGTGCAACAGCAGAAC---
AATCTG---CTGAGA---GCTATTGAGGCGCAACAACATCTGTTGCAACTCACAGTCTGGGGCATC
>HQ908219
TTCTATTGTAACACAACACGGCTGTTTAATAATTGGACTTCTAATAATACTTGGAATGATACTACAGGGTCA-
----

AATAACAGCACAATCACACTCCCATGCAGAATAAAACAAATTATAAATAGGTGGCAGGAAATAGGAAAAGCAA
TGTATGCCCCTCCCATCGCAGGACAAATTAACTGTTCATCAAAT---
ATTACAGGGTTGCTATTAACAAGAGATGGTGGTAAGACCAACAACAGC-------------------------
----

AACAGCAGTGAGACCTTCAGACCAGGGGGAGGAAATATGAAGGACAATTGGAGAAGTGAATTATATAAATATA
AAGTAGTAAAAATTGAACCATTA---GGAATAGCACCCACCGAGGCAAGGAGAAGAGTGGTG-----------
----------------------------CAGAGAGAAAAAAGAACA---GTAACA---TTAGGAGCT------

---ATGTTCCTTGGGTTCTTGGGAGCA---GCAGGAAGCACTATG---GGCGCAGCATCA---
ATGACGCTGACGGTACAG---GCCAGACAA---TTATTG---TCTGGTATAGTGCAACAGCAGAGC---
AATCTG---CTGAGG---GCTATTGAGGCGCAACAGCATATGTTGCAACTCACAGTCTGGGGCATC
>HM204596
TTCTACTGTAATACATCACAATTGTTCAATAGTACTTGGGAGTTTAATAGTACTTGGAATGGGAATGAAACTG
AA---

GGAAATGACACCATCATTCTCCCATGTAGAATAAAACAGATTATAAACCTGTGGCAGAGAGTAGGAAAAGCAA
TGTATGCCCCTCCCATCAAAGGAATAATTAGATGTTCGTCAAAT---
ATTACAGGGCTGCTATTAACAAGAGATGGTGGTAATAGTAATACCTCCAAC----------------------
----

TCCAGCAATATTGAAACCTTTAGACCTGGAGGAGGAAATATGAAGGACAATTGGAGAAGTGAATTATATAAAT
ATAAAGTAGTAAGACTTGAACCATTA---GGAGTAGCACCCACCAAGGCAAGGAGAAGAGTGGTG--------
-------------------------------CAGAGAGAAAAAAGAGCA---GTGGGA---CTAGGAGCT---
------TTGTTCCTTGGGTTCTTGGGAGCA---GCAGGAAGCACTATG---GGCGCAGCGTCA---
GTGACGCTGACGGTACAG---GCCAGACAA---TTGTTG---TCTGGTATAGTGCAACAGCAGAAC---
AACCTG---CTGAGA---GCTATTGAGGCGCAACAGCATCTGTTGCAACTCACAGTCTGGGGCATC
>KY112149
TTCTACTGTAATACATCACGACTGTTTAATAGTACTTGGAATACTACACAATTGTTTAATAGTACTGAGAGTG
CTAATGATACT--
-------------GGAGGG------
TTGAATAACACAGTTATTACCCTCCCATGCAGAATAAAACAAATTATAAACATGTGGCAGGAAGTAGGAAAAG
CAATGTATGCCCCTCCCATCGAAGGATTACTTAATTGTTCATCAAAC---
ATTACAGGGCTGCTATTAACAAGAGATGGTGGTAGTGATCTTAACAGTACTGAC-------------------
----

AACAATACCAATATAGAGGTCTTCAGACCGCAAGGAGGAGATATGAGGGATAATTGGAGAAGTGAATTATATA
AATATAAAGTAGTAAAAATTGAACCATTA---GGAGTAGCACCCACCAAGGCAAAGAGAAGAGTGGTG-----
----------------------------------CAGAGAGAAAAAAGAGCA---GTGGGA---
ATAGGAGCT---------CTGTTCCTTGGGTTCTTGGGAGCA---GCAGGAAGCACTATG---
GGCGCAGCGTCC---ATGACGCTGACGGTACAG---GCCAGGCTA---TTATTG---
TCTGGTATAGTGCAGCAGCAAAAC---AATTTG---CTGAAG---
GCTATTGAGGCGCAACAGCGTCTGTTGCAACTCACAGTCTGGGGCATC
>KY112490
TTCTACTGTGATTCATCACAACTGTTTAATAGCACTTGGAATAATGAGACTTGGAGTGATAATAGTACTAAAG
GGTCA--

AATAGCACAGAAATTATTACACTCCCATGCAGAATAAAACAATTTATAAACATGTGGCAGGAAGTAGGGAAAG
CAATGTATGCCCCTCCTATCAAAGGACAACTTAATTGTTCATCAAAC---
ATTACAGGGCTGCTGTTAACAAGAGATGGCGGTAGTGGTCTTAACAGGACTGAC-------------------
----
AACAGGACTGAGAAC---
AATACTGAGGTCTTCAGACCGCAAGGAGGAGATATGAGGGATAATTGGAGAAGTGAATTATATAAATATAAAG
TAGTAAGAATTGAACCATTA---GGAATAGCACCCACCAAGGCAAAGAGAAGAGTGGTG--------------
-------------------------CAGAGAGAAAAAAGAGCA---GTGGGA---GTAGGAGCT---------
CTGTTCCTTGGGTTCTTGGGAGCA---GCAGGAAGCACTATG---GGCGCAGCGTCA---
ATGACGCTGACGGTACAG---GCCAGGCTA---TTATTG---TCTGGTATAGTGCAACAGCAGAAC---
AATTTG---CTGAAG---GCTATTGAGGCGCAACAGCGTCTGTTGCAACTCACAGTCTGGGGCATC
>JQ403067
TTCTACTGTAATACAACAAAGCTGTTTAATAGTACTTGGAATGGTACTGAGAATGGTACTCTTACAGGGTCA-
----

AATGACACTAGCATCACACTCCCATGTAGAATAAAACAAATTATAAACAGGTGGCAAGAAGTAGGAAAAGCAA
TGTATGCCCCTCCCATCAGTGGGCAAATTACATGTTCATCAAAT---
ATTACAGGGCTACTATTAACAAGAGATGGAGGTCCGAATGAGAAT----------------------------
----

GAGACTGAGACCTTCAGACCTGGAGGAGGAAATATGAGGGATAATTGGAGAAGTGAATTATATAGATATAAAG
TAGTAAGAATTGAGCCATTA---GGAGTAGCACCCACCAAGGCAAAGAGAAGAGTGGTG--------------

-------------------------CAGAGAGAAAAAAGAGCA---GTGACG---CTAGGAGCT---------
GTGTTCCTTGGGTTCTTGGGAACA---GCAGGAAGCACTATG---GGCGCAGCGTCA---
GTGGCGCTGACGGCACAG---GCCAGACAA---TTATTG---TCTGGTATAGTGCAACAGCAGAAC---
AATTTG---CTGAGG---GCTATTGAGGCGCAACAGCATCTGTTGCAACTCACAGTCTGGGGCATC
>JQ403069
TTCTACTGCAATACAACACCACTATTTAATAGTATTTGGAATGATACACAACTGTCTAATAGTACTTGGAATG
AGACTGACTGG--
----------TCAAATAAA------
ACTACAAGAGAAAACATCACACTCCAATGCAGAATAAAACAAATTGTAAACATGTGGCAGGAAGTAGGAAGAG
CAATGTACGCCCCTCCCATTAGGGGAATAATTAAATGTTCATCAAAT---
ATCACAGGGCTACTATTAACAAGAGATGGAGGGGATTCTAATGAC----------------------------
----

AATGAAACCTTCAGACCTGGAGGGGGAGATATGAGGGACAATTGGAGAAGTGAATTATATAAATATAAAGTTG
TAAAAATTGAGCCATTA---GGAGTAGCACCCACCAAGGCAAAGAGAAGAGTGGTG-----------------
----------------------CAGAGAGAAAAAAGAGCA---GTGGGA---TTAGGAGCT---------
ATGTTCCTGGGGTTTCTGGGAGCA---GCAGGAAGCACTATG---GGCGCAGCGTCA---
ATAACGCTGACGGTACAG---GCCAGACAA---CTATTG---TCTGGTATAGTGCAACAGCAGAGC---
AATTTG---CTGAGA---GCTATTGAGGCGCAACAGCATCTGTTGCAACTCACAGTCTGGGGCATC
>JQ403082
TTCTACTGTAATACATCACAGCTATTTAATACATCACTTCTATTTGATAAGAGTCCTGAAAAGCCA-------
----

AATAATGGCACTATCACACTCCCATGTAGAATAAAACAAATTATAAACCTGTGGCAGGAAGTAGGGAAAGCAA
TGTATGCCCCTCCCATCAGTGGACCAATTAACTGTTCATCAGAA---
ATTACAGGGCTGCTATTAACAAGAGATGGTGGTAATAATAATGACACCAAT----------------------
----

AGCACCCACGAGGTCTTCAGACCTGGAGGAGGAGATATGAGGGACAATTGGAGAAGTGAATTATATAAATATA
AAGTAGTGAAAATTGAACCATTA---GGAGTAGCACCCACCAAGGCAAAGAGAAGAGTGGTG-----------
----------------------------CAGAGAGAAAGAAGAGCA---GTGGGA---ATAGGAGCT------
---ATATTCCTTGGGTTCTTGGGAGCA---GCAGGAAGCACTATG---GGCGCAGCGTCA---
ATAACGCTGACGGTACAG---GCCAGGCAA---TTATTG---TCTGGTATAGTGCAACAGCAGAGC---
AATTTG---CTGAGA---GCTATCGAGGCGCAACAGCATCTGTTGCAGCTCACAGTCTGGGGCATT
>JQ403035
TTCTACTGTAATACATCAAGACTGTTTAATAGTACTTGGCATAGTAATGGTACTGTAGAGTCAAGTAACAAT-
----

AACAGTACAAATATCACACTCCAATGCAGAATAAAACAAATTGTAAACATGTGGCAGGAAGTAGGAAGAGCAA
TATATGCCCCTCCCATCAGAGGACTAATTAGATGTTCATCAAAC---
ATTACTGGGCTGCTATTAACAAGAGATGGTGGTAGTAACACCACC----------------------------
----

AATGGGACCGAGATCTTCAGGCCTGGAGGAGGAGATATGAGGGACAACTGGAGAAGTGAATTATACAAATATA
AAGTAGTAAAAATTGAACCATTA---GGAATAGCACCCACCAAGGCAAAGAGAAGAGTGGTG-----------
----------------------------CAGAGAGAAAAAAGAGCA---GTGGGA---CTAGGAGCT------
---ATGTTCCTTGGGTTCTTGGGAGCA---GCAGGAAGCACTATG---GGCGCAGCGTCA---
GTGACGCTGACGGTACAA---GCCAGACAA---TTATTG---TCTGGTATAGTGCAACAGCAAAAC---
AATTTG---CTGAGG---GCCATTGAGGCGCAACAACATCTGTTGCAACTCACAGTCTGGGGCATT
>JQ403083
TTCTATTGTGATTCAACAAAACTGTTTAATAGTACTTGGTATACTAATGGGAATACTACTGGAGAGCCAATTA
ACACTGAC---

GGAAATGGTACAATCACACTCCCATGCAGAATAAAACAAGTGATAAACATGTGGCAGGAAGTAGGAAAAGCAA
TGTATGCCCCTCCCATCAGCGGAATAATTAACTGTTCATCAAGT---
ATTACAGGGCTAATATTAACAAGGGATGGTGGTAACGAGAACGGGACC-------------------------
----

AACGAGACCGAGATCTTCAGACCTGGAGGAGGAGATATGAGGGATAATTGGAGAAGTGAATTATATAAATATA
AAGTAGTGAAACTTGAACCATTA---GGAATAGCACCCACCAAGGCAAGGAGAAGAGTGGTG-----------

---------------------------CAAAGAGAAAAAAGAACA---GTGGGA---CTAGGAGCT------
---ATGTTCCTTGGGTTCTTGGGAGCA---GCAGGAAGCACTATG---GGCGCAGCGTCA---
GTGACGCTGACGGTACAG---GCCAGACTA---CTATTG---TCTGGTATAGTGCAACAGCAGAGC---
AATTTG---CTGAGG---GCTATTGAGGCGCAACAGCATATGTTGCAACTCACAGTCTGGGGCATC
>JQ403084
TTCTACTGTAATACAACACAGCTGTTTAATGATAGTATTTGGAATGGGACTAGGACTAAAGAGCAAAAT----
----

GAAAATGAAACTATCACACTCCCATGCAGAATAAAACAAATTATAAACATGTGGCAGAAAGTGGCAAAGCAA
TGTATGCCCCTCCCATCAGAGGACAGATTAAATGTTCATCAAAT---
ATTACAGGGCTGCTATTAACAAGAGATGGTGGTACCCAGAGCAACGGT------------------------
----
-
ACCCAGGGCAATGAGACCGAGATCTTCAGACCTGCAGGAGGAGATATGAGGGACAATTGGAGAAGTGAATTAT
ATAAATATAAAGTAGTACAAATTGAGCCAGTA---GGAGTGGCACCCACCAAGGCAAAGAGAAGAGTGGTG--
------------------------------------CAAAGAGAAAAAAGAGCA---
GTGGGAACAATAGGAGCT---------ATGTTCCTTGGGTTCTTGGGAGCA---GCAGGAAGCACTATG---
GGCGCAGCGTCA---ATAACGCTGACGGCACAG---GCCAGACAA---TTATTG---
TCTGGTATAGTGCAACAGCAGAAC---AACTTG---CTGAGA---
GCTATTGAGGCGCAACAGCATTTGTTGCAACTCACAGTCTGGGGCATT
>JQ403086
TTCTACTGTAATACAACACAACTGTTTAATAGTGCTTGGAATAGTACTAGGGATAATAGTACTGCAGAGTCAA
GT--

AACAGTACACATATCACACTCCCATGCAGAATAAAACAAATTATAAACATGTGGCAGGAAGTAGGAAGAGCAA
TATATGCCCCTCCCATCAGAGGACTGATTAGATGTTCATCAAAT---
ATTACAGGGCTACTATTAACAAGAGATGGTGGTAGTAACAACACC---------------------------
----

AATGAGATCGAGACCTTCAGGCCAGCAGGAGGAGATATGAGGGACAATTGGAGAAGTGAATTATATAAATACA
AAGTAGTAAAAATTGAACCATTA---GGAATAGCACCCACCAAGGCAAAGAGAAGAGTGGTG-----------
-----------------------------CAAAGAGAAAAAAGAGCA---GTGGGAGTAATAGGAGCT------
---ATGTTCCTTGGGTTCTTGGGAACA---GCAGGAAGCACTATG---GGCGCAGCGTCA---
GTGACGCTGACGGTACAA---GCCAGACAA---TTATTG---TCTGGTATAGTGCAACAGCAAAAC---
AACTTG---CTGAGG---GCTATTGAGGCGCAACAACATCTGTTGCAACTCACAGTCTGGGGCATC
>JQ403087
TTCTATTGTAATACAACACAACTGTTTAATAGTACTTGGATAAATAATGGTACTTGGAAGGGTACTAACGTGT
CAGAT---

TCAAATGACACTGTCACACTCCCATGCAGAATAAAACAAATCATAAACAGGTGGCAAGAAGTAGGAAAAGCAA
TATATGCCCCCCCCATCGCAGGACGACTTGAGTGCATATCAAAT---
ATTACAGGGCTGCTATTAACAAGAGATGGTGGTATTATTAACACCACC------------------------
----

AAGGACAATGAGACCTTCAGACCTGGAGGAGGAGACATGAGGGACAATTGGAGAAATGAGTTATATAAATATA
AAGTAGTAAAAATTGAACCATTA---GGAATAGCGCCCTCCAAGGCAAGGAGAAGAGTGGTG-----------
-----------------------------CAAAGAGAAAAAAGAGCA---GTGGGG---TTCGGAGCC------
---TTGTTCCTTGGGTTCTTGGGAGCA---GCAGGAAGCACTATG---GGCGCAGCATCA---
TTGATGCTGACGGTACAG---GCCAGACAA---TTATTG---TCTGGTATAGTGCAACAGCAGAAC---
AATCTG---CTGAGA---GCTATTGAGGCGCAACAACATATGTTGCAACTCACAGTCTGGGGCATC
>JQ403088
TTCTACTGTAATACAACACAACTGTTTAATAGTAATTGGACTAATACAACTAGAGAGGGA-------------
----

GAGAATGGCACAATCACACTCCCATGCAGAATAAAACAAATTATAAACATGTGGCAGGAAGTAGGAAAGGCAA
TGTATGCCCCTCCCATCAGAGGACATATTAACTGTTCATCAAAT---
ATTACAGGGTTGCTATTAACAAGAGATGGTGGTGAAAACAATGGGAGT------------------------
----

AATGACAGCGAGACCTTCAGACCTGGAGGAGGAGATATGAGAGACAATTGGAGAAGTGAATTATATAAATATA

AAGTAGTACAAATTGAACCATTA---GGAGTAGCACCCACCAAGGCAAAGAGAAGAGTGGTG-----------
----------------------------CAGAGAGAAAAAAGAGCA---GTGACA---ATAGGAGCT------
---ATGTTCCTTGGGTTCTTGGGAGCA---GCAGGAAGCACTATG---GGCGCAGCGTCA---
ATAACGCTGACGGTACAG---GCCAGACAA---TTATTG---TCTGGTATAGTGCAACAGCAGAGC---
AATTTG---CTGAGG---GCTATTGAGGCGCAACAGCATCTGTTGCAACTCACAGTCTGGGGCATC
>JQ403089
TTCTACTGCAATACAACAAAACTGTTTAATAGTACTTGGACTGAGAATAGAACTGTAGGGACTGAA-------
----

GGAAATGACACAATCACAATCCAATGCAGAATAAAACAAATTATAAACATGTGGCAGCAAGTAGGAAAAGCAA
TGTATGCCCCTCCCATCAGCGGACCCATTAGATGCTCATCAAAT---
ATTACAGGACTGCTATTAATGAGAGATGGTGGTACAAACCAGACC----------------------------
----

AACAATACCGAGATCTTCAGACCTGGAGGAGGAGATATGAGGGACAATTGGAGAAGTGAACTATATAAATATA
AAGTAGTGCAAATTGAACCATTA---GGAATAGCACCCACCAAGGCAAGGAGAAGAGTGGTG-----------
----------------------------CAGAGAGAAAAAAGAGCA---GTGGGA---ATAGGAGCT------
---CTGTTCCTTGGGTTCTTGGGAGCA---GCAGGAAGCACTATG---GGCGCAGCGTCA---
ATAACGCTGACGGTACAG---GCCAGACAA---TTATTG---TCTGGTATAGTGCAACAGCAGAAC---
AATTTG---CTGAAA---GCTATTGAGGCGCAACAGCATCTGTTGCAACTCACAGTCTGGGGCATC
>JQ403091
TTCTACTGTAATACAACACAACTGTTTAATAGTAGTTGGGTTAATAGTAGTTGGGTTAGTGGGAGTAATGAAT
CTAGCGAT---

GCCAATGGCACAATAACACTCCCATGCAGAATAAAACAAATTATAAACATGTGGCAGGAGGTAGGAAAAGCAA
TGTATGCCCCTCCCATCAAGGGACAAATTAGATGCACATCAAAT---
ATCACAGGGCTGATATTAACAAGAGATGGTGGTAAAAATACAAGT----------------------------
----

AACAACAGCGAGACCTTCAGACCTGGAGGAGGAGATATGAGGGACAATTGGAGAAGTGAGCTATATAAATATA
AGGTAGTAAAAATTGAACCTTTA---GGAATAGCACCCACCAAGGCAAAGAGAAGGGTGGTG-----------
----------------------------CAGAGAGAAAAAAGAGCA---GTGGGA---GTAGGAGCT------
---TTGTTCCTCGGGTTCTTGGGAGCA---GCAGGAAGCACTATG---GGCGCAGCGTCA---
ATAACGCTGACGGTACAG---GCCAGACTA---TTGTTG---TCTGGTATAGTGCAACAGCAGAAC---
AATTTG---CTGAGA---GCTATTGAGGCGCAACAGCATATGTTGCAACTCACAGTCTGGGGCATT
>JQ610123
TTCTATTGTAACACAACACAACTATTTGATAGTACTTGGGATAGTAATAGTACTTGGAATAGTACTGGGAATA
CAAATAAA---
-------------------ACT---
GGAGGAAAGGACACAATCACACTCCCATGCAGAATAAAACAAATTATAAACAGGTGGCAGGAAGTAGGAAAAG
CAATGTATGCCCCTCCCATCAGCGGACAAATTAGATGTTCATCAAAT---
ATTACAGGACTGCTATTAACAAGAGATGGTGGTAAGAATAATAGTACG-------------------------
----

AACAACACTGAGACTTTCAGACCTGCAGGAGGAGATATGAAAGACAATTGGAGAAGTGAATTATATAAATACA
AAGTGGTAAGAATTGAGCCATTA---GGAGTAGCACCCACCAAGGCAAAAAGAAGAGTGGTG-----------
----------------------------CAGAGAGAAAAAAGAGCA---GCA------CTAGGAGCT------
---ATGTTCCTTGGGTTCTTGGGAGCA---GCAGGAAGCACTATG---GGCGCAGCGTCA---
GTGACGCTGACGGTACAG---GCCAGACTA---TTATTG---TCTGGTATAGTGCAACAGCAGAAT---
AATTTG---CTGAGG---GCTATTGAGGCGCAACAGCATATGTTGCAACTCACAGTCTGGGGCATC
>KM081848
TTCTACTGTAATTCAACACCACTGTTTGGTAGTACTTGGCTGTTAAATAATACTGGGAATGGTACTTGGAATA
GGAATGATACT--
----------GAAAAGCCA------
AATAACACTGACCCAATCACACTCCAATGCAGAATAAAACAATTTATAAACATGTGGCAGGAAGTAGGAAAAG
CAATGTATGCCCCTCCCATCCAAGGACATATTAGATGTTCATCAAAT---
ATTACAGGGCTGCTATTGACAAGAGATGGTGGTAGTAATGACACCACG-------------------------
----

AACGGCACAGAGACCTTCAGACCTGGAGGAGGAGATATGAGGGACAATTGGAGAAGTGAATTATATAAATATA

AAGTAGTAAAAATTGAACCATTA---GGAGTAGCACCCACTAGGGCAAAGAGGAGAGTGGTG-----------
----------------------------CAGAGAGAAAAAAGAGCA---GTGACA---TTAGGAGCT------
---ATGTTCCTTGGGTTCTTGGGAGCA---GCAGGAAGCACTATG---GGCGCAGCGTCA---
ATAACGCTGACGGTACAG---GCCAGACAA---TTATTG---TCTGGTATAGTGCAACAGCAGAAC---
AATTTG---CTGAGA---GCCATTGAGGCGCAACAACATATGTTGCAACTCACAGTCTGGGGCATC
>KM081861
TTCTACTGTGACTCAACAAAACTGTTTAATAGTACTTGGAATAATAACACTGGGAATGTAAGTGATACTGAAG
GG---

TCAAATAACACTATCACGCTCCCATGCAGGATAAAACAAATTATAAACATGTGGCAGACAGTGGGAAAAGCAA
TGTACGCCCCTCCTATCCGAGGACAAATTAACTGTTTATCAAAT---
ATTACAGGGCTACTCTTAACAAGAGATGGTGGTAACGGGAACAACACT-------------------------
----

AATACTAATGAGACCTTCAGACCTGGAGGAGGAGATATGAGGGACAATTGGAGAAGTGAATTATATAAATATA
AAGTAGTAAAAATTGAACCATTA---GGAGTAGCACCCACCAAGGCAAAGAGAAGAGTGGTG-----------
----------------------------CAAAGAGAAAAAAGAGCA---GTGGGA---ATAGGAGCT------
---GTGTTCCTTGGGTTCTTGGGAGCA---GCAGGAAGCACTATG---GGCGCAGCGTCA---
CTGACGCTGACGGTACAG---GCCAGACAA---TTATTG---TCTGGTATAGTGCAACAGCAGAAC---
AATTTG---CTGAGG---GCTATCGAGATGCAACAGCATCTGTTGCAACTCACAGTCTGGGGCATC
>KM081893
TTCTACTGTAATACAACACAACTATTTAATAGTACTTGGACTTGGAATAGTACTGGGGAGGACAATAAC----
----

ACTGCAGAAAATATCACACTCCCCTGTAGAATAAAACAAATTATAAACATGTGGCAGGAAGTAGGAAAAGCAA
TGTATGCCCCTCCCATCAGAGGACAAATTAGATGTTCATCTAAT---
ATTACAGGGTTGCTATTAACAAGAGATGGTGGTAGAGGTAGCGGGAAC-------------------------
----
AATTCCAAGACCAAC---
GATTCCGAGATCTTCAGACCTGGAGGAGGAGATATGAGGGACAATTGGAGGAGTGAATTATATAAATATAAAG
TAGTAAAAATTGAACCATTA---GGAGTAGCACCCACCAAGGCAAAGAGAAGAGTGGTG--------------
-------------------------CAGAGAGAAAAAAGAGCA---GTGGGAACACTGGGAGCT----------
ATGTTCCTTGGGTTCTTGGGAGCA---GCAGGAAGCACTATG---GGCGCAGCGGCA---
GTGACGCTGACGGCACAG---GCCAGACAA---TTATTG---TCTGGTATAGTGCAGCAGCAGAAC---
AATCTG---CTGAGG---GCTATTGAGGCGCAACAGCATCTGTTGCAACTCACAGTCTGGGGCATC
>KM081913
TTCTACTGTAACTCAACACCACTGTTTAATAGCACTTGGTATCCTAATAATAGCACTTGGTATCCTAATGAGA
CTGTGGCAGAT--

ACTGAAGGAAATATCACACTCCAATGCCGAATAAAACAAGTCATAAACCTGTGGCAGGAAGTAGGAAAGGCAA
TGTATGCCCCTCCCATCAGCGGACCAATTAAATGTTCATCAAAT---
ATTACAGGGCTGCTATTAACAAGAGATGGTGGTAATGGCAATACCACA-------------------------
----

AACACCATCGAGGTCTTTAGACCTGGAGGAGGAGATATGAGGGATAATTGGAGAAGTGAGTTATACAAATACA
AAGTAGTAAGAATTGAACCATTA---GGAGTAGCACCCACCAGGGCAAAGAGAAGAGTGGTG-----------
----------------------------CAGAGAGAAAAAAGAGCA---ATAGGA---ATAGGAGCT------
---GTGTTCCTTGGGTTCTTGGGAGCA---GCAGGAAGCACTATG---GGCGCAGCGTCA---
ATAACGCTGACGGTACAA---GCCAGACAA---TTATTG---TCTGGTATAGTGCAACAGCAGAAC---
AACTTG---CTGATG---GCTATTGAGGCGCAACAGCATCTGTTGCAACTCACAGTCTGGGGCATC
>KM081963
TTCTACTGTAATACATCACAGCTGTTTAATGGTACTTTACAGCTTAATGGTACTTGGATTTGGAATGGTACTT
GGAATGGTACT--
-------------TGGAAT------
GGTACTATAGGTACCATCACACTTCCATGCAGAATAAAACAAATTATAAACAGGTGGCAGGAAGTAGGAAAAG
CAATGTATGCCCCTCCCATCAGTGGACAAATTAGATGCTCATCCAAT---
ATTACAGGGCTACTATTAACAAGAGATGGAGGAGATAAAAGTAGTAACAGC----------------------
----

ACAAACAATACTGAGACCTTCAGACCTGGAGGAGGAAATATGAGGGACAATTGGAGAAGTGAATTATATAAAT

ATAAAGTAGTAAGAATTGAGCCATTA---GGAATAGCACCCACCAAGGCAAAGAGAAGAGTGGTG--------
-------------------------------CAGAGAGAAAAAAGAGCA---GTGGGAACACTAGGAGCT---
------ATGTTCCTCGGGTTCTTGGGAGCA---GCAGGAAGCACTATG---GGCGCAGCGTCA---
ATGACGCTGACGGTACAA---GCCAGACAA---TTATTG---TCTGGTATAGTGCAACAGCAGAGC---
AATTTG---CTGAGA---GCTATTGAGGCGCAACAGCATATGTTGCAACTCACAGTCTGGGGCATC
>JX864027
TTCTACTGCAATACAACACCACTATTTAATAGCACTTGGAAAAGGAATGGTAATTGGAGTAGAAATGATACTG
AAGAA--
-------------------TCA---
AACAGCACAGAACCAATCACACTCCAATGCAGAATAAAACAAATTGTAAACATGTGGCAGGAAGTAGGAAGAG
CAATGTATGCCCCTCCCATCAGTGGACGAATTAACTGTTCATCAAAT---
ATTACAGGGCTGATACTAACAAGAGATGGTGGTAATAACAGTGCC----------------------------
----

GCCAACGAGACCTTCAGACCAGCAGGAGGAGATATGAGGGACAATTGGAGAAGTGAACTATATAAATATAAAG
TAGTAAAAATTGAACCATTA---GGAATAGCACCCACCAAGGCAAAGAGAAGAGTGGTG--------------
-------------------------CAGAGAGAAAAAAGAGCA---GCA------GTAGGAGCT---------
CTGTTCCTTGGGTTCTTGGGAGCA---GCAGGAAGCACTATG---GGCGCAGCGTCA---
GTGACGCTGACGGTACAG---GCCAGACTA---TTATTG---TCTGGTATAGTGCAACAGCAGAAC---
AATTTG---CTGAAG---GCTATTGAGGCGCAACAGCATCTGTTGCAACTCACAGTCTGGGGCATC
>MH666175
TTCTACTGTAATACGACAATACTGTTTAATAGTACTTGGAATTCAACACAACTGCAGAATAATGACACTGAAC
AG---

TCAAATGACACTATCACACTCCCATGCAGAATAAAACAGTTTATAAACATGTGGCAGGAAGTAGGAAAAGCAA
TGTATGCCCCTCCCATCAGAGGACAAATTAAATGTTCATCAAAT---
ATTACAGGGCTGATATTAACAAGAGATGGTAATAAGGAAGAAAACAATACCGAA-------------------
----

GGGACCGAGATCTTCAGACCTGGAGGAGGAGATATGAGAGACAATTGGAGAAGTGAATTATATAAATATAAAG
TAGTAAGAATTGAACCATTA---GGAGTAGCACCCACCAAGGCAAAGAAAAGAGTGGTG--------------
-------------------------CAGAGAGAAAAAAGAGCA---GTGGGA---ATAGGAGCT---------
TTGTTCCTTGGTTTCTTGGGAGCA---GCAGGAAGCACTATG---GGCGCAGCGTCA---
GTGACGCTGACGGTACAA---GCCAGACAA---TTATTG---TCTGGTATAGTGCAGCAGCAAAAT---
AATTTG---CTGAGG---GCTATTGAGGCGCAACAACATATGTTGCAACTCACAGTCTGGGGCATC
>MH666186
TTCTACTGTAATACAACAAAACTATTTAATAGCATCTGGAATGGCACCAGGAGTGAGAATAATAATACTGGAG
GG---

TCAAATGAAAACATCACACTCCCATGCAGAATAAAACAAATTATAAACATGTGGCAGAGAGTAGGAAAAGCAA
TGTATGCCCCTCCCATCAGAGGACAAATTAGATGTACATCAAAT---
ATTACAGGGCTGATATTAACAAGAGATGGAGGCCTAAACAACAAC----------------------------
----

ACCAACGAGACCTTCAGACCGGGAGGAGGAAATATGAAGGATAAGTGGAGAAGTGAATTATATAAATATAAAG
TAGTAAGAATTGAACCATTA---GGAGTAGCACCCACCAGGGCAAAAAGGAGAGTGGTG--------------
-------------------------CAGAGAGAAAAAAGAGCA---GTAGGA---ATAGGAGCT---------
TTGTTCCTTGGATTCTTGGGAGCA---GCAGGAAGCACTATG---GGCGCAGCGTCA---
CTGACGCTGACGGTACAG---GCCAGACAA---TTATTG---TCTGGTATAGTGCAACAGCAGAAC---
AACTTG---CTGAGA---GCTATAGAGGCGCAACAGCATATGTTGCAACTCACAGTCTGGGGCATC
>MH666216
TTCTACTGTAATACATCAAAGCTGTTTAGTAGTACTTGGAATAGTACTACTAATTGGAATAGTACTAATTGGA
ATAGTACTACT--

GATGGGAATAATACTATCATACTCCCATGCAGAATAAAACAAATTATAAACATGTGGCAGGCAGTAGGAAAAG
CAATGTATGCCCCTCCCATTAAAGGAGTAATTAAGTGTTTATCAAAT---
ATTACAGGGTTGCTATTAGTAAGAGATGGTGGTATTAACCAGACCAACACCACC-------------------
----

GAGATCGAGACCTTCAGACCTGGAGGAGGAGATATGAGGGACAATTGGAGAAGTGAATTATATAAATATAAAG

TAGTAAGAATTGAACCATTA---GGAGTAGCACCCACCAAGGCAAAAAGGAGAGTGGTG--------------
-------------------------CAGAGAGAAAGAAGAGCG---GTGGGA---ATAGGAGCT---------
GTGTTCCTTGGGTTCTTGGGAGCA---GCAGGAAGCACTATG---GGCGCAGCGTCA---
ATAACGCTGACGGTACAG---GCCAGACAA---TTATTG---TCTGGTATAGTGCAACAGCAAAAC---
AATTTG---CTGAGG---GCTATTGATGCGCAACAGCATCTGTTGCAACTCACAGTCTGGGGCATA
>MH666227
TTCTACTGTAATACAACAAAATTATTTAGTAGTACTTGGCATAATGAAACCAATAGCACTTTGAATGATCCTG
AAAATAGC---

AATAACGACACAATCACACTCCCATGTAGAATAAAACAATTTATAAACATGTGGCAGGAAGTAGGAAAAGCAA
TGTATGCCCCTCCCATCCAAGGACTAATTAACTGTTCATCAAAC---
ATTACAGGGCTATTATTAGTAAGAGATGGTGGTGGTAATGATACA----------------------------
----

AGGGACAATGAGACCTTCAGACCTGGAGGAGGAAATATGAAAGACAATTGGAGAAGTGAATTATATAAATATA
AAGTAGTAAAAATTGAACCATTA---GGGGTAGCACCCACTAAGGCAAAGAGAAGAGTGGTG-----------
----------------------------CAGAGAGAAAAAAGAGCA---GTGGGA---ATAGGAGCT------
---ATGTTCCTTGGGTTCTTGGGAGCA---GCAGGAAGCACTATG---GGCGCAGCGTCA---
ATAACGCTGACGGTACAG---GCCAGACAA---CTATTG---TCTGGTATAGTGCAACAGCAGAAC---
AATTTG---CTGAGG---GCTATTGAGGCGCAACAGCATCTGTTGCAACTCACAGTCTGGGGCATT
>MH666240
TTCTACTGTAATACAACACAGCTGTTTAATAGCACTTGGAAGGGGGGAGAGTTAAATAGCACTGAAGAAAATA
ATAGCACT---

AAAGAAAGTAATATCACACTCCCATGCAGGATAAAACAAATTATAAATATGTGGCAGGAAGTAGGAAAAGCAA
TGTATGCCCCTCCCATCAAAGGAGAAATTAGATGTTCATCAAAT---
ATTACAGGACTGCTAATAACAAGAGATGGTGGTAACAAGAATGATACT-------------------------
----

AATGATACTGATACTGAGATCTTCAGACCTGGAGGAGGAAATATGAAGGACAATTGGAGAAGTGAATTATATA
AATATAAAGTAGTAAAAATTGAGCCATTA---GGAGTAGCACCCACCAAGGCAAAGAGAAGAGTGGTG-----
----------------------------------CAGAGAGAAAAAAGAGCA---GTGGGA---
ATAGGAGCT---------TTGTTCCTTGGGTTCTTGTCAGCA---GCAGGAAGCACTATG---
GGCGCAGCGTCA---ATAACGCTGACGGTACAG---GCCAGACAA---TTACTG---
TCTGGTATAGTGCAACAGCAGAAC---AATTTG---CTGAGG---
GCTATTGAGGCGCAACAACATCTGTTGCAACTCACAGTCTGGGGCATC
>MH666253
TTCTATTGTAATACAACACCACTGTTTAATAGTACCTGGAATAAGAATGATAGTTCCGGGAACTGGACTGGG-
----
-AATGATACTGGAGTGGCA------
AATAACAATGGCACAATCACCCTCCAATGCAGACTAAAACAAATTATAAACATGTGGCAGGAAGTAGGAAAGG
CAATGTATGCCCCTCCCATCCGGGGAAACATTAGCTGTTCATCAAAC---
ATTACAGGGCTGCTATTAACAAGAGATGGTGGTGTTAATGCCACA----------------------------
----

AGCAACGAAACCTTTAGACCTGCAGGAGGAGATATGAGGGACAATTGGAGAAGTGAATTATATAAATATAAAG
TAGTACAAATTGAACCATTA---GGAATAGCACCCACCAAGGCAAAGAGAAGAGTGGTG--------------
-------------------------CAAAGAGAAAAAAGAGCA---GTGGGA---TTTGGAGCT---------
ATGTTCCTTGGGTTCTTGGGAGCA---GCAGGAAGCACTATG---GGCGCAGCGTCA---
GTAACGCTGACGGTACAG---GCCAGACAA---TTATTG---TCTGGTATAGTGCAACAGCAGAGC---
AACCTG---CTGAGA---GCTATTGAGGCGCAACAACATATGTTGCAACTCACGGTCTGGGGCATT
>MH666262
TTTTACTGTGATACAACACTACTGTTTAACAGTACTTGGAACAGTAACAGTACTGGGAACAGTACTGGGAATG
GTACTGAA---

GAGGGTGATCCAATCACACTCCAATGCAGAATAAAACAAATTATAAACATGTGGCAGGAAGTAGGAAAAGCAA
TGTATGCCCCTCCCATCAGAGGACAAATTAGATGTTCATCAAAC---
ATTACAGGGTTGCTATTAACAAGAGATGGTGGCAACAGTGATAGCACT-------------------------
----

AACGGAACTGAGATCTTCAGACCTGCAGGAGGAGATATGAAGGACAATTGGAGAAGTGAATTATACAAATATA
AAGTAGTAAAAGTTGAACCATTA---GGAGTAGCACCCACCATGGCAAGGAGAAGAGTGGTG-----------
----------------------------CAGAGAGAGAAAAGAGCA---GTGGGA---CTAGGAGCT------
---TTGTTCCTTGGGTTCTTGGGAGCA---GCAGGAAGCACTATG---GGCGCAGCGTCA---
ATGACACTGACGGTACAG---GCCAGACAA---CTATTG---TCTGGTATAGTGCAGCAGCAGAAC---
AATCTG---CTGAGG---GCTATTGAGGCGCAACAGCATATGTTGCAACTCACAGTCTGGGGCATC
>KM354431
TTCTACTGTAATACATCACAACTGTTTAATAATACTTGGAATAGTACTGATAAA-------------------
----
-ACGTTAAATAACACT---------
GAAGGAAATGACACAATTACCCTCCCATGCAGAATAAGACAGATTATAAACAGGTGGCAGGAAGTAGGAAAAG
CAATGTATGCCCCTCCAATCGCAGGAATAATTACATGTTCATCAAAT---
ATTACAGGGTTAATATTAACAAGAGATGGGGGG--

ACAGAGAATGGGACTAACACC---------
ACCACTGAGACCTTCAGACCTGGGGGAGGAAACATGAAAGACAATTGGAGAAGTGAATTATATAAATATAAAG
TAGTAAGAATTGAACCATTA---GGGATAGCACCCACCAGGGCAAGGAGAATAGTGGTG--------------
-------------------------CAGAGAGAAAAAAGAGCA---GTGGGAACGATAGGAGCC---------
ATGTTCCTTGGGTTCTTGGGAGCA---GCAGGAAGCACTATG---GGCGCAGCGTCA---
GTGACGCTGACGGTACAG---GCCAGACTA---TTATTG---TCTGGTATAGTGCAACAGCAAAAT---
AATTTG---CTGAGA---GCTATTGAGGCGCAACAGCGTATGTTGCAACTCACAGTCTGGGGCATC
>KM354585
TTCTACTGTAATACATCAGGACTGTTTAATAGCACTTGGACTCGGAACAATACTACTGGCACT----------
--TGG---
AACAGTATCAATACCACCACC---------------
ATAGGAAATGACACCATCACACTCCAATGCAGAATAAGACAAATTATAAACAGGTGGCAGGAAGTAGGAAAAG
CAATGTATGCCCCTCCCATCGCAGGAATAATTAGATGTTCATCAAAT---
ATTACAGGGCTGCTAATAACAAGAGATGGTGGG--

-
AACGAAACAGTGCACAATGAGACCTTCAGACCTGCAGGAGGGGATATGAGGGACAATTGGAGAAGTGAATTAT
ATAAATATAAAGTAGTAAAAGTTGAACCATTA---GGAATAGCACCCACCAAGGCAAGGAGAAGAGTGGTG--
-------------------------------------CAGAGAGAAAAAAGAGCA---GTGGGA---
ATAGGAGCT---------GTATTCCTTGGGTTCTTGGGAGCA---GCAGGAAGCACTATG---
GGCGCAGCGTCA---CTGACGCTGACGGTACAG---GCCAGACTA---TTATTG---
TCTGGTATAGTGCAACAGCAAAAC---AATTTG---CTGAAG---
GCTATTGAGGCGCAGCAGCATATGTTGCAACTCACAGTCTGGGGCATT
>KM354670
TTCTACTGTAATACAACACGACTATTTAATAGTATTTGGTATGCTAATGGTAGTAGTAAAGAG----------
--ACA---
GAGCCTGTTAATAGTACTGAA---------------
GGGACAAATGCCACTATCAGACTCCCATGCAGAATAAAACAAATTATAAACCTGTGGCAGGAAGTAGGAAAGG
CAATGTATGCCCCTCCCATCAAAGGACTAATTAGATGTTCATCAAAT---
ATTACAGGGATACTATTAACAAGAGATGGTGGTAGT-------------------------------------
----
ATAAGGAATGACACAGATAAC---------
ACCACAGAGGTCTTCAGACCTGGAGGTGGAGATATGAGGGACAATTGGAGAAGTGAATTATATAAATATAAAG
TAGTAAAAATTGAACCATTA---GGAGTAGCACCCACCAAGGCAAAGAGAAGAGTGGTG--------------
-------------------------CAGAGAGAAAAAAGAGCA---GTGGGA---ATAGGAGCT---------
GTGTTCCTTGGGTTCTTGGGAGCA---GCCGGAAGCACTATG---GGCGCAGCGTCA---
ATAACGCTGACGGTACAG---GCCAGACAA---TTATTG---TCTGGTATAGTGCAACAGCAAAGC---
AATTTG---CTGAGG---GCTATTGAGGCGCAACAACATCTGTTGCAACTCACAGTCTGGGGCATC
>KX587204
TTCTATTGTAATACATCAAAACTGTTTAATAGTACTTGGAATAGTACTAGGAATGATAGTGTTACTGGAGAG-
----

TCAAATAACACTATCACACTCCAATGCAGGATAAAACAAATTATAAACATGTGGCAGGAAGTTGGAAAAGCAA
TGTATGCCCCTCCCATCAGCGGACCAATTAGATGCTCATCAAAT---
ATTACAGGGTTGCTATTAACAAGAGATGGTGGGAAGGAGTCCAAT----------------------------
----

ACGACTGAGACCTTCAGACCAGGGGGAGGAAATATGAAGGATAATTGGAGAAATGAATTATATAAATATAAGG
TAGTAAAAATTGAACCATTA---GGAGTAGCACCCACCAAGGCAAGGAGAAGAGTGGTG--------------
-------------------------CAGAGAGAAAAAAGAGCA---GTGGGA---CTAGGAGCT---------
ATGTTCCTTGGGTTCTTGGGAGCA---GCAGGAAGCACTATG---GGCGCAGCGTCA---
GTGACGCTGACGGTACAG---GCCAGACAA---TTATTG---TCTGGTATAGTGCAACAGCAAAAC---
AATTTG---CTGAGA---GCTATTGAGGCGCAACAGCATCTGTTGCAGCTCACAGTCTGGGGCATC
>KC473824
TTCTACTGTGATACAACACAACTGTTTAATAGTACTTGGAATAATACTAATATTAATTCAAATGACACTGAGA
GA---

TCAAATAAAACTATCATTCTTCCATGCAAAATAAAACAAATTGTAAAAATGTGGCAGAGAGTAGGAAAAGCAA
CGTATGCCCCTCCCATCAGAGGACAAATTAGATGCTCATCAAAT---
ATTACAGGGCTGTTATTAACAAGAGATGGTGGTAGTAACCATAAT----------------------------
----

AATAGTGAGACCTTCAGACCTGGAGGAGGAGATATGAGGGACAATTGGAGGAGTGAATTATATAAATATAAAG
TAGTAAAAATTGAACCATTA---GGAGTAGCACCCACCAGGGCAAAGAGAAGAGTGGTG--------------
-------------------------CAAAGGGAGAGAAGAGCA---GTGGGA---ATAGGAGCT---------
ATGCTCCTTGGGTTCTTAGGAACA---GCCGGAAGCACTATG---GGCGCAGCGTCA---
GTGGCGCTGACGGTACAG---GCCAGACTA---TTATTG---TCTGGTATAGTGCAGCAGCAGAAC---
AATTTG---CTGAGA---GCTATTGAGGCGCAACAGCACCTGTTGCAACTCACAGTCTGGGGCATC
>MK164663
TTCTACTGTAATACAACAGAACTGTTTGAAAGAAGTTGGAGTAATAGTACTTGGAATGTTACTACCAATAATA
CTGAAGGG---

GAAGATAAAAATATCACTCTCCCATGCAGAATCAAACAGATTATAAACATGTGGCAGGAAGTAGGAAAAGCAA
TGTATGCCCCTCCCATCAAAGGACAAATTAGCTGTTCATCAAAT---
ATTACAGGGCTAATATTAACAAGAGATGGTGGTAACAACAACGGCAGTAATAAC-------------------
----
AACAACGGCAGTAATAACAACAAC------
AGTAGTGAGATCTTCAGACCTACAGGAGGAAATATGAGGGACAATTGGAGAAGTGAATTATATAAATATAAAG
TAGTAAAAATTGAACCATTA---GGAATAGCCCCCACCAAGGCAAAGAGAAGAGTGGTG--------------
-------------------------CAGAGAGAAAAAAGAGCA---GTGGGAGTACTAGGAGCT---------
ATGTTCCTTGGGTTCTTGGGAGCA---GCAGGAAGCACTATG---GGCGCAGCGTCA---
CTGACGCTGACGGTACAG---GCCAGACAA---CTATTG---TCTGGTATAGTGCAACAGCAGAAC---
AACCTG---CTGAGG---GCTATTGAGGCGCAACAGCATCTGTTGCAACTCACAGTATGGGGCATC
>MK164670
TTCTACTGTAATACAACAAAACTGTTTAATAATACTTGGGTTAATGGTACTTGGAGTAATATGCCTAACAATG
ATACTGAA---

GGGGCAGATGGAAAGATCACTCTCCCATGCAGAATCAAACAGATTATAAACATGTGGCAGGAAGTAGGAAAAG
CAATGTATGCCCCTCCCATCAAAGGACAAATTAGCTGTTCATCAAAT---
ATTACAGGGCTAATATTAACAAGAGATGGTGGCGACAACAACAGTCGGACCAAC-------------------
----

GACAGTAGTGAGATCTTCAGACCTAATGGAGGAAATATGAGGGACAATTGGAGAAGTGAATTATATAAATATA
AAGTAGTAAAAATTGAACCATTA---GGAATAGCCCCCACCAAGGCAAAGAGAAGAGTGGTG-----------
-----------------------------CAGAGAGAAAAAAGAGCA---GTGGGAGTACTAGGAGCT------
---ATGTTCCTTGGGTTCTTGGGAGCA---GCAGGAAGCACTATG---GGCGCAGCGTCA---
TTGACGCTGACGGTACAG---GCCAGACAA---CTATTG---TCTGGTATAGTGCAACAGCAGAAC---
AACCTG---CTGAGG---GCTATTGAGGCGCAACAGCATCTGTTGCAACTCACAGTCTGGGGCATC
>KC935958
TTCTACTGTAATACAACAAAACTGTTTAATAGTACTTGGAATGGTACTGAGTCAGCTAACAATACA-------
----

GAAAATGGGACAATCACACTCCCATGCAGAATAAAACAAATCATAAATATGTGGCAGACAGTAGGAAAAGCAA
TGTATGCCCCTCCCATTAGGGGACAAATTACATGTATATCAAAT---
ATTACAGGGCTGCTATTAACAAGAGATGGTGGTGAGGAAAATAATACAACC----------------------
----

AATGCAACCATCGAGACCTTTAGACCGGGAGGAGGAGATATGAGGGACAATTGGAGAAGTGAATTATATAAAT
ATAAAGTAGTAGAAATTGAACCATTA---GGAATAGCACCCACCAAGGCAAAGAGAAGAGTGGTG--------
-------------------------------CAGAGAGAAAAAAGAGCA---GTGACA---CTAGGAGCT---
------CTGTTCCTTGGGTTCTTGGGAGCA---GCAGGAAGCACTATG---GGCGCAGCATCA---
ATGACGCTGACGGTACAA---GCCAGACAA---TTATTG---TCTGGTATAGTGCAACAGCAGAAT---
AATCTG---CTGAGG---GCTATTGAGGCGCAACAGCACCTGTTGCAGCTCACAGTCTGGGGCATC
>JF680914
TTCTACTGTAATACAACAAAACTGTTTAATAGTACTTGGTATTCGAATGATACTAGCAAGGGAGATAACAATG
AA---

GGAAATGATACAATCACACTCCCATGCAGAATAAAACAAATTGTAAACATGTGGCAGGAAGTAGGAAAAGCAA
TGTATGCCCCTCCCATCAGAGGACAAATTAGATGTTCATCAAAT---
ATTACAGGGCTGCTATTAACAAGAGATGGTGGTAACCATACTGAA----------------------------
----

AATAACGAGACTTTCAGACCTGGAGGAGGAGATATGAGGGACAATTGGAGAAGTGAATTATATAGGTATAAGG
TAGTAAAAATTGAACCATTA---GGAGTAGCACCCACCAAGGCAAAGAGAAGAGTGGTG--------------
-------------------------CAGAGAGAAAAAAGAGCA---GTAGTGGGAATGGGAGCT---------
CTGTTCCTTGGGTTCTTGGGAGCA---GCAGGAAGCACTATG---GGCGCAGCGTCA---
GTGACGCTGACGGTACAG---GCCAGACTA---TTATTG---TCTGGTATAGTGCAACAGCAGAAC---
AACTTG---CTGAGG---GCTATTGAGGCGCAACAACATCTGTTGCAACTCACAGTCTGGGGCATC
>JF680916
TTTTACTGTAATACAACAAGGCTGTTTAATAGTAATAGTACTATCTTGGATGCTTGGAATGAAACTACTGCTG
AT---

GAGAACAAGACTATCACACTCCCATGTAGGATAAAACAAATTATAAACATGTGGCAGGAAGTAGGAAAAGCAA
TGTATGCCCCTCCCATCAGTGGACAAATTAAGTGTTCATCAAAT---
ATTACAGGACTAATATTAACAAGAGATGGGGGTAATAATGATACA----------------------------
----

AGCGGGACTGAGACCTTCAGACCTGGAGGAGGAGATATGAGAGACAATTGGAGAAGTGAATTATATAAATACA
AAGTAGTAAAAGTTGAACCATTA---GGAGTAGCACCCAATAAGGCAAAGAGAAGAGTGGTG-----------
----------------------------CAGAGAGAAAAAAGAGCA---GTGGGA---ATAGGAGCT------
---ATGTTCCTTGGGTTCTTGGGAGCA---GCAGGAAGCACTATG---GGCGCAGCGTCA---
CTGACGCTGACGGTACAG---GCCAGACAA---TTATTG---TCTGGTATAGTGCAACAGCAGAAC---
AATATG---CTGAGA---GCTATTGAGGCGCAACAACATCTGTTGCAACTCACAGTCTGGGGCATC
>MK383384
TTCTACTGTAATACAACAAAACTGTTTAATAGTACCTGGATCTGGGATAATGGTGCTGAAAACTGGAATGGTA
CTGAAAGGCCA--

GAGAATGACACTATCACACTCAAATGCAGAATAAAACAAATTATAAACATGTGGCAGGAAGTGGGAAAAGCAA
TGTATGCCCCTCCCATCAGTGGACAACTTAATTGTTCATCAAAT---
ATTACAGGTCTGCTTTTAACAAGAGATGGTGGTAAAAACAATAGT----------------------------
----

ACTAATGAGACCTTCAGACCTGGAGGAGGGGATATGAGGGACAATTGGAGAAGTGAATTATATAAATATAAAG
TAGTAAAAATTGAACCATTA---GGAATCGCACCCACCAAGGCAAAGAGAAGAGTGGTG--------------
-------------------------CAAAGAGAAAAAAGAGCA---GTGACA---TTTGGAGCT---------
ATGTTCCTTGGGTTCTTGGGAGCA---GCAGGAAGCACTATG---GGCGCAGCGTCA---
ATGACGCTGACGGTACAG---GCCAGACAA---TTATTG---TCTGGTATAGTGCAACAGCAGAGC---
AATTTG---CTGAGA---GCTATTGAGGCGCAACAGCATCTGTTGCAACTTACGGTCTGGGGCATC
>KF526141
TTCTATTGTAATTCAACACCACTATTTAATAGTACTTGGAATGTTAATGATACTAGTACTGGGAATGATACT-
----

GAAGAAGGAGATATCACACTCCAATGCAGAATAAAACAAATTGTAAACATGTGGCAAGAAGTAGGAAAAGCAA
TGTATGCCCCTCCCATCAGAGGACCAATTAGTTGTTCATCAAAT---
ATTACAGGGCTGCTATTAACAAGAGATGGCGGTGTTAATAGGAGC----------------------------
----

AATGGAACCGAGGTCTTCAGACCTTCAGGGGGAGATATGAGGGACAATTGGAGAAGTGAATTATATAAATATA
AAGTAGTACAAATTGAACCATTA---GGAGTAGCACCCAGCAAGGCAAAAAGAAGAGTGGTG-----------
----------------------------CAGAGAGAAAAAAGAGCA---GCGGGA---ATGGGAGCT------
---CTGTTCCTTGGGTTCTTGGCAGCA---GCAGGAAGCACTATG---GGCGCAGCATCA---
ATGACGCTGACGGTACAG---GCCAGACAG---TTACTG---TCTGGTATAGTGCAACAGCAGAGC---
AATTTG---CTGAGG---GCTATTGAGGCGCAACAGCATCTGTTGCAACTCACAGTCTGGGGCATT
>KM081852
TTCTACTGTAATACAACACAGCTGTTTAATAGTACTTGGCTTAGCAATGGAACTTGGACTAATGCTAGA----
----

AGCAATGAGACTATCATACTCCAATGCAGAATAAAACAAATTATAAACAAGTGGCAGGGAGTAGGAAGAGTAA
TGTATGCCCCTCCCATCAGTGGAAATATTAGCTGTTTATCAAAT---
ATTACAGGGCTACTATTAACAAGAGATGGGGGTACAAAAAATGACATA-------------------------
----

AATGAGGAGATCTTCAGACCTGGAGGAGGAGATATGAGGGACAATTGGAGAAGTGAATTATATAAATATAAAG
TAGTAAAAATTGAACCAATA---GGAGTAGCACCCACCAAGGCAAAGAGAAGAGTGGTG--------------
-------------------------CAGAGAGAAAGAAGAGCA---GCA------TTAGGAGCT---------
ATGTTCCTTGGGTTCTTGGGAGCA---GCAGGAAGCACTATG---GGCGCAGCGTCG---
GTGACGCTGACGGTACAG---GCCAGACAA---TTATTG---TCTGGTATAGTGCAGCAGCAGAGC---
AATCTG---CTGAGG---GCTATTGAGGCGCAACAACATCTGTTGCAACTCACAGTCTGGGGCATT
>KM082074
TTCTACTGTAATACATCACAACTGTTTAATAGTACTTGGACTTGGAATGGTACTGAAGGGTCCAATAACACAA
AT---

AGCACTGATAATATCACACTCCAATGTAGAATAAAACAAATTGTAAATTTGTGGCAAGAAGTAGGGAAAGCAA
TGTATGCCCCTCCCATTAGAGGACAGATTAGATGTTCATCAAAT---
ATTACAGGGCTAATATTAACAAGAGATGGTGGACATGGAAATGAT----------------------------
----

AACACCACCGAGATCTTCAGACCTGGAGGGGGAGATATGAGGGACAATTGGAGAAGTGAATTATATAAGTATA
AAGTAGTACAAATTGAACCATTG---GGAATAGCACCCACCAAGGCAAAGAGAAGAGTGGTG-----------
----------------------------CAGAGAGAAAAAAGAGCA---GCA------ATAGGAGCT------
---GTGTTCCTTGGGTTCTTGGGAGCA---GCAGGAAGCACTATG---GGCGCAGCGTCG---
GTAGCGCTGACGGTACAG---GCCAGACAA---TTATTG---TCTGGTATAGTGCAACAGCAGAAC---
AATCTG---CTGAGA---GCTATTGATGCGCAACAGCATATGTTGCAGCTCACAGTCTGGGGCATC
>MG196787
TTCTACTGTAATACAACACAACTGTTTAATAGTACTTGGCATTGGAATAGCACTGAAGAATCAAATACT----
----

ACTGAAAGAAATATCACACTTCCATGCAGAATAAAACAAATCATAAACATGTGGCAGGAAGTAGGAAAAGCAA
TGTATGCCCCTCCCATCAGAGGACTAATTAAATGTTCATCAAAT---
ATTACAGGGCTGCTATTGACAAAAGATGGGGGTACTGGAAATAAG----------------------------
----

ACTGAGGAGATCTTCAGACCTGGGGGAGGAAATATGAAGGACAATTGGAGAAGTGAATTGTATAAATATAAAG
TAGTAAAAATTGAACCATTA---GGAATAGCACCCACCAGGGCAAAGAGAAGAGTGGTG--------------
-------------------------CAGAGAGAAAAAAGAGCA---GTGGGA---CTAGGAGCT---------
GTGTTCCTTGGGTTCTTGGGAGCA---GCAGGAAGCACTATG---GGCGCAGCGTCA---
GTGACGCTGACGGTACAG---GCCAGACAA---TTATTG---TCTGGCATAGTGCAACAGCAAAAC---
AATTTG---CTGAGG---GCTATTGATGCGCAACAGCATCTGTTGCAACTCACAGTCTGGGGCATC
>MG196997
TTCTATTGTAATTCAACACAACTGTTCAACAGTACTTGGAATAGTACTTTGACTAATAACTGGAATGATACTT
GGAATGCTAAT--
-------------AGCACT------
GATGTCAATGACACTATCACAATCCCATGCAGAATAAAACAAATTATAAACATGTGGCAGGAAGTAGGAAAAG
CCATGTATGCCCCTCCCATCAGAGGACAAATTAGATGTGAATCAAAT---
ATTACAGGGCTGCTATTGACAAGAGATGGTGGTGATAGCGATGGC----------------------------
----

ACAGACGAGATCTTCAGACCTGGAGGAGGAGATATGAGGGACAATTGGAGAAGTGAATTATATAAATATAAAG
TAGTAAAAATTGAGCCATTA---GGAATAGCACCCACCAGGGCAAGGAGAAGAGTGGTG--------------
-------------------------CAGAGAGAAAAAAGAGCA---GTGGGA---CTAGGAGCT---------
TTGTTCCTTGGGTTCTTGGGAGCA---GCAGGAAGCACTATG---GGCGCAGCGTCA---
ATAACGCTGACGGTACAG---GCCAGGCAA---TTATTG---TCTGGTATAGTGCAGCAGCAAAGC---
AACTTG---CTGAGG---GCTATTGAGGCGCAACAACATCTGTTGCAACTCACAGTCTGGGGCATC
>KM354703
TTCTACTGTAATTCAACGCCACTATTTAACAGTAATTGGACTCGGAACAATGGGACT----------------
----
TGGAAGCTAAATAACACTTCA------------
AATAGCACTGAGCCAATCACACTCCAATGCAGAATAAAACAAATTGTAAACAGGTGGCAAGAAGTAGGAAAAG
CAATGTATGCCCCTCCCATCGGAGGACAAATTAACTGTACATCAAAT---
ATTACAGGGCTACTAATAACAAGGGATGGTGGT--

-
GACCCGAACAGCACGACTGAGACCTTCAGACCAGGAGGAGGAGATATGAGGGACAATTGGAGAAGTGAATTAT
ATAAATATAAAGTAGTAAAAATTGAACCATTG---GGAATAGCACCCACCAAGGCAAAGAGAAGAGTGGTG--
-------------------------------------CAGAGAGAAAAAAGAGCA---GTGACA---
CTGGGAGCT---------ATGTTCCTTGGGTTCTTGGGAGCA---GCAGGAAGCACTATG---
GGCGCAGCGTCA---ATAACGCTGACGGTACAG---GCCAGACAA---TTACTG---
TCTGGTATAGTGCAACAGCAGAAC---AATCTG---CTGAAG---
GCTATTGATGCGCAACAGCATATGTTGCAACTCACCGTCTGGGGCATC
>KM354781
TTCTACTGTAATACAACAAAACTGTTTAATAGTACTTGGAATAGTACTTGGAATAGTGAG-------------
----
GAACAGTCAGGTGTTGATAAC------------
ACATCAAATAAGATTATCACAATCCAATGCAGAATAAAACAAATTGTACACATGTGGCAGAGAGTAGGAAGGG
CAATGTATGCCCCTCCCATCAGTGGACCAATTAGATGTTCATCAAAT---
ATTACAGGGTTGCTATTAGTAAGAGATGGTGCC--

AACGATACCAAGGAGACTTTCAGACCAGGAGGAGGAAATATGAAGGACAATTGGAGAAGTGAATTATATAAAT
ACAAAGTAGTAAAAATTGAACCATTA---GGAATAGCACCCACTAGGGCAAGGAGAAGAGTGGTG--------
-------------------------------CAGAGAGAAAAAAGAGCA---GTGGGA---CTAGGAGCT---
------ATGTTCCTTGGGTTCTTAGGAGCA---GCAGGAAGCACTATG---GGCGCAGCGTCG---
ATGGCGCTGACGGTACAA---GCCAGACAA---TTATTG---TCTGGCATAGTGCAGCAGCAGAAC---
AATCTG---CTGAGG---GCTATTGAGGCGCAACAGCACATGTTGCAACTCACAGTCTGGGGCATC
>JN397365
TTCTACTGTAACACATCAGGACTGTTTAATAGTACTTGGACTGTTATCGGTGAGTGGGAGGATCCTACACGG-
----

AATGATACCAACATCACACTCCCATGTAGAATAAAACAAATTATAAACATGTGGCAGGAAGTAGGGAAAGCAA
TGTATGCCCCTCCCATCAAAGGACTAATCAACTGTACATCAAAT---
ATCACAGGGCTGCTATTAACAAGAGATGGTGGTATTAATAGAACT----------------------------
----

AATAACGAGACCTTCAGACCTGCAGGAGGAAATATGAAGGACAATTGGAGAAGTGAATTATATAAATATAAAG
TAGTACAAATTGAACCATTA---GGATTAGCACCCACCAAGGCAAAGAGGAGAGTGGTG--------------
-------------------------CAGAGAGAAAGAAGAGCA---GTGACA---CTCGGAGCT---------
ATGCTCCTTGGGTTCTTGAGCGCA---GCAGGAAGCACTATG---GGCGCAGCGTCG---
CTGACGCTGACGGTACAG---GCCAGACTA---TTATTG---TCTGGTATAGTGCAACAGCAGAAC---
AATCTG---CTGAGA---GCTATTGAGGTGCAACAGCATCTGTTGCAACTCACAGTCTGGGGCATT
>HQ846901
TTCTACTGTAATTCAACACCACTGTTTAATAGTACTTGGAATGTTACTGGAGAGTCTTGGAATGATACAAAA-
----

GGGGGAGTAAACATCACACTTCCATGCAGAATAAGACAAATTATAAACATGTGGCAGAAAGTAGGAAAAGCAA
TGTATGCCCCTCCCATCAGAGGACAAATTAGATGTACATCAAAT---
ATTACAGGGTTGATATTAACAAGAGATGGTGGTAATAACAAGAGTGAG-------------------------

----

AACGAGACTGAGACCTTCAGACCTGGAGGAGGAGATATGAGGGACAATTGGAGAAGTGAACTATATAAATATA
AAGTAGTAAAAATTGAACCATTA---GGAATAGCACCCACCAAGGCAAAGAGAAGAGTGGTG-----------
----------------------------CAGAGAGAAAAAAGAGCA---GTGGGA---ATAGGAGCT------
---GTGTTCCTTGGGTTCTTGGGAGCA---GCAGGAAGCACTATG---GGCGCAGCGTCA---
CTGACGCTGACGGTACAG---GCCAGACTA---TTATTG---TCTGGTATAGTGCAGCAGCAGAAC---
AATCTG---CTGAGG---GCTATTGAGGCGCAACAGCATCTGTTGCAGCTCACAGTCTGGGGCATC
>KC473825
TTCTACTGTAATACAACAAGACTGTTTGCTTGGAATAGTGCTTGGAATAGTACTGGGAATGAAACT-------
----

GCAGATAGAAATATTACACTCCCATGCAGAATAAAACAAATTATAAACATGTGGCAGGCAGTAGGAAAAGCAA
TGTATGCCCCTCCCATCAGAGGAGCAATTAATTGTGCATCAAAT---
ATTACAGGGCTGTTATTATTAAGAGATGGTGGTAATAGCAGCGAA----------------------------
----

GAGATCTTCAGACCTGGAGGAGGAGATATGAGGGACAATTGGAGAAGTGAATTATATAAATATAAAGTAGTAA
AAATTGAACCAATA---GGAGTAGCACCCACCAAGGCAAAGAGAAGAGTGGTG--------------------
-------------------CAGAGAGAAAAAAGAGCA---GTAGGA---ATAGGAGCT---------
CTGTTCCTTGGGTTCTTGGGAGCA---GCAGGAAGCACTATG---GGCGCAGCGTCA---
ATAACGCTGACGGTACAA---GCCAGACAA---TTATTG---ACTGGTATAGTGCAACAGCAGAAC---
AATCTG---CTAAGG---GCTATTGAGGCGCAACAGCACCTGTTGCAACTCACAGTCTGGGGCATT
>KC473827
TTCTACTGTAATACAACAGCACTGTTTAATAGCACTTGGAATAGGAATGAGACTTGGAATTCTACTGGAGAG-
----

TCAAATGAAACTATCACACTCCAATGTAGAATAAAACAAATTATAAACATGTGGCAGGAAGTAGGAAAAGCAA
TGTATGCCCCTCCCATCAGAGGACAAATTAGATGTTCATCCAAT---
ATTACAGGGCTGTTATTAACAAGAGATGGGGGTAACACAAACGAG----------------------------
----

ACTACCTCTGAGACTTTTAGACCCGGAGGAGGAAATATGAGGGACAATTGGAGAAGTGAATTATATAAATATA
AAGTAGTAAAGATTGAACCATTA---GGAATAGCACCCACCAAGGCAAAGAGAAGAGTGGTG-----------
----------------------------CAGAGAGAAAAAAGAGCA---GTGGGA---ATAGGAGCT------
---ATGTTCCTTGGGTTCTTGGGAGCA---GCAGGAAGCACTATG---GGCGCAGCGTCA---
ATGACGCTGACGGTACAG---GCCAGACTA---TTATTG---TCTGGTATAGTGCAACAGCAGAAC---
AATCTG---CTGAGG---GCTATTGAGGCGCAACAACATCTGTTGCAACTCACAGTCTGGGGCATC
>KU678074
TTCTACTGTAATACAACACAACTGTTTAATAGTACTTGGTATCCTAATGATACAAGTACTTGGAATGATACTA
AT---

GGAATTAATGACACACTCACACTACCATGTAAAATAAAACAATTTGTAAACTTGTGGCAGGAGGTAGGAAAGG
CAATGTATGCCCCTCCCATCCAGGGACAAATTAATTGTGCATCAAAT---
ATTACAGGGCTGCTATTAACAAGAGATGGAGGTGTTAACACTACC----------------------------
----

AACACCACTGAGGTCTTCAGACCTGCAGGAGGAGATATGAGGGACAATTGGAGAAGTGAACTATATAAATATA
AAGTAGTAAAAATTGAACCATTA---GGAATAGCACCCACTAAGGCAAAGAGAAGAGTGGTA-----------
----------------------------CAAAGAGAAAAAAGAGCA---GTGGGA---ATAGGAGCT------
---ATGTTCCTTGGGTTCTTGGGAGCA---GCAGGAAGCACTATG---GGCGCAGCGTCA---
GTGACGCTGACGGTACAG---GCCAGACAA---TTAATG---TCTGGTATAGTGCAACAGCAGAGA---
AATTTG---CTGAGG---GCTATTGAGGCGCAGCAGCATCTGTTGCAACTCACAGTCTGGGGCATT
>KM082156
TTCTACTGCAATACAACACAACTGTTTAATAGTACTTGGGCTAATAGTACTTGGAATGACACTGAAGGGTTTA
GTGACACT---

GGAAATGATACAATCACACTCCCATGCAGAATAAAACAAATTATAAATCTGTGGCAGGAAGTAGGAAAAGCAA
TGTACGCCCCTCCCATCAGAGGACAAATTAGATGTTCATCAAAT---
ATTACAGGGCTGCTATTAACAAGAGATGGTGGTAATATTACCAAT----------------------------

----
-
AATAATACAAACAATAATGAGACCTTCAGACCTGGAGGAGGAGATATGAGGGACAATTGGAGAAGTGAATTAT
ATAAATATAAAGTAGTAAAAATTGAACCATTA---GGAATAGCCCCCACCAAGGCAAAGAGGAGAGTGGTG--
-------------------------------------CAGAGAGAAAAAAGAGCA---GTGGGA---
ATAGGAGCT---------GTGTTCCTTGGGTTCTTGGGAGCA---GCAGGAAGCACTATG---
GGCGCAGCGTCA---ATAACGCTGACGGTACAG---GCCAGACTG---TTATTG---
TCTGGTATAGTGCAACAGCAGAAC---AATTTG---CTGAGA---
GCTATTGAGGCGCAACAACATCTGTTGCAACTCACAGTCTGGGGCATC
>KF526228
TTCTACTGTAATACAACACAACTGTTTAACAGTACTTGGAATTCACCACAACCTAATAGGAATTGGACA----
----

GGAAATGACACAATCACACTCCCATGCAGAATAAAACAATTTATAAACATGTGGCAGGAAGTAGGAAAAGCAA
TGTATGCCCCTCCCATCAAAGGAATAATTAGATGTGTATCAAAT---
ATTACAGGAATGATATTAACAAGAGATGGTGGTATTGGTAACACCACCGGG----------------------
----

GAGAACGGAACAGAGATCTTTAGACCAGGAGGAGGAGATATGAGGGACAATTGGAGAAGTGAACTATATAAAT
ATAAAGTAGTAAGAATTGAACCATTA---GGAATAGCACCCACCAAGGCAAAGAGAAGAGCGGTG--------
-------------------------------CAGAGAGAAAAAAGAGCA---GTGGGA---ATAGGAGCT---
------GTGTTCCTTGGGTTCTTGGGAGCA---GCAGGAAGCACTATG---GGCGCAGCGTCA---
CTAACGCTGACGGTACAA---GCCAGACTA---TTATTG---TCTGGTATAGTGCAACAGCAGAAC---
AATCTG---CTGAGG---GCTATTGAGGCGCAACAGCATCTGTTGCAACTCACTGTCTGGGGCATC
>KF526265
TTCTACTGCAATTCATCACAATTGTTTAATAGTACTTGGAATGGTACTGAGTCTTGGAATAGTACTGGG----
----

CATGACACTAACATCACACTCCAATGCAGAATTAAACAAATTATAAACATGTGGCAGGAAGTAGGAAAAGCAA
TGTATGCCCCTCCCATCCAAGGACCAATTAGCTGTTCATCAAAT---
ATTACAGGGCTGCTATTAACAAGAGATGGTGGTGGGAACAAGAGC----------------------------
----

GAGACTGAGACTGAGACTTTCAGACCTGTAGGAGGAAATATGAGGGACAATTGGAGAAGTGAATTATATAAAT
ATAAAGTAGTAAAAATTGAACCATTA---GGAATAGCACCCACCAAGGCAAAGAGAAGAGTGGTG--------
-------------------------------CAGAGAGAAAAAAGAGCA---ATGGGA---CTAGGAGCT---
------TTGTTCATTGGGTTCTTGGGAGCA---GCAGGAAGCACTATG---GGCGCAGCGTCA---
ATAACGCTGACGGCACAG---GCCAGACTA---CTATTG---TCTGGTATAGTGCAACAGCAGAAC---
AATTTG---CTGAGA---GCTATTGAGGTGCACCAACATATGTTGCAACTCACAGTCTGGGGCATC
>KF526312
TTCTACTGTAATGCAACAAAACTGTTTAATTCTACGTGGAATAGTAGTACTTGGAATGATAATAATCCTGGG-
----

GAAAATGTTACCATCACACTCCCATGCAGGATAAAACAAATTATAAACATGTGGCAGAAAACAGGAAAAGCAA
TGTATGCCCCTCCCATCAGTGGACGAATTAGCTGTTTATCAAAT---
ATTACAGGGCTGATATTAACAAGAGATGGGGGTATTAATAACACG----------------------------
----

AACGAGACCTTCAGACCTGCAGGAGGAGATATGAGGGACAATTGGAGAAGTGAATTATATAAATATAAAGTAG
TAAAAATTGAACCATTA---GGAATAGCACCCAACAAGGCAAGGAGAAGAGTGGTG-----------------
----------------------CAGAGAGAAAAAAGAGCA---GTGGGA---ATAGGAGCT---------
GTGTTCCTTGGGTTCTTGGGAGCA---GCAGGAAGCACTATG---GGCGCAGCGTCA---
GTAACGCTGACGGTACAG---GCCAGACTA---TTGCTG---TCTGGTATAGTGCAACAGCAGAAC---
AATCTG---CTGAGG---GCTATTGAGGCGCAACAGCATCTGTTGCAACTCACAGTCTGGGGCATC
>KF526323
TTCTATTGTAATACATCAAAATTATTTAATAGTACCTGGAATGAGACTAGTACTTGGAATGAGATGGGA----
----

AAAGATGATAATATCATACTCCCATGTAGAATAAAACAAATTATAAACAGATGGCAGGAACCAGGAAAAGCAA
TGTATGCCCCTCCCATCGCAGGACTAATTAGATGTTCATCAAAT---

ATTACAGGGCTGCTATTAATAAGAGATGGTGGTAACAAGGGCAGTAATGGT----------------------
----

AGCAACACCGAGATCTTCAGACCTGGAGGAGGAGACATGAGGGACAATTGGAGAAGTGAATTATATAAATATA
AAGTAGTAAAAATTGAACCATTA---GGAATAGCACCCACCAAGGCAAAGAGAAGAGTGGTG-----------
----------------------------CAGAGAGAAAAAAGAGCA---GTGGGAGTACTAGGAGCT------
---ATGTTCCTTGGGTTCTTGGGAACA---GCAGGAAGCACTATG---GGCGCAGCGTCA---
GTGACGCTGACGGTACAG---GCCAGGCAA---TTAATG---GCTGGTATAGTGCAACAGCAGAAC---
AATCTG---CTGAGA---GCTATTGATGCGCAACAGCAACTGTTGCAACTCACGGTCTGGGGCATC
>KT124766
TTCTACTGTAACACAACAGAACTGTTTAACAGTACTTGGAATGTTACTAGTGATGAGTCAAATAACACA----
----

AATAACACTACTATCACGCTCCAATGCAGAATAAAACAAATTGTAAACATGTGGCAGACAGTAGGAAAAGCAA
TGTATGCCCCTCCCATCCAAGGAATAATTAGATGTTCATCAAAT---
ATTACAGGGCTGCTATTAACAAGAGATGGTGGGTATAATAATGGCACCAAT----------------------
----

AATGATACCGAGATCTTCAGACCTGGAGGAGGAGATATGAGGGACAATTGGAGAAGTGAATTATATAAATATA
AAATAGTAAGAATTGAACCATTA---GGAATAGCACCCAACAAGGCAAAGAGAAGAGTGGTG-----------
----------------------------CAAAGAGAAAAAAGAGCA---GTGGGGATGATAGGAGCA------
---ATGTTCCTTGGGTTCTTGGGAGCA---GCAGGAAGCACTATG---GGCGCAGCGTCA---
CTAACGCTGACGGTACAT---GCCAGACAA---GTATTG---GCTGGTATAGTGCAACAGCAGAAC---
AATCTG---CTGAGG---GCTATTGAGGCGCAACAACATATATTGCAACTCACAGTCTGGGGCATC
>KT124773
TTCTATTGTAATACAACACAACTGTTTAATAGTACCTGGGTTAATAGTACATGGAATAGTACTGAAGGAATG-
----

AACAACACCAACATCACACTCCCATGCAGAATAAAACAGATTGTAAACATGTGGCAGGAAGTAGGAAAAGCAA
TGTATGCCCCTCCCATCAGAGGAAATATTACATGTGCATCAAAT---
ATTACAGGGGTGCTATTAACAAGAGATGGTGGACATAATGGATCA----------------------------
----

AATGAGACCTTCAGACCTGCAGGAGGAGATATGAGGGATAATTGGAGAAGTGAATTATATAAATATAAAATAG
TAAAAATTGAACCATTA---GGAATAGCACCCACCAAGGCAAAAAGGAGAGTGGTG-----------------
----------------------CAGAGAGAAAAAAGATCA---CCAACA---CTGGGAGCT---------
TTGTTCATTGGGTTCTTGGGAGCA---GCAGGAAGCACTATG---GGCGCAGCGTCA---
CTGACGCTGACGGTACAA---GCCAGGCAA---TTATTG---TCTGGTATAGTGCAACAGCAGAGC---
AATCTG---CTGAGG---GCTATTGAGGCGCAACAACACATATTGCAACTCACGGTCTGGGGCATC
>KT124775
TTCTACTGTAACACCACACAGCTGTTTAATGAAACCTGGATTAACAGTAACAGTACCGGG-------------
----

AGTAATAGCAGTATCACACTCCCATGCAGAATAAAACAAATTATAAACAGATGGCAGGAAGTAGGAAAAGCAA
TGTATGCCCCTCCCATGCAAGGACAAATTAGTTGCTCATCAAAT---
ATTACAGGACTACTATTAACAAGAGATGGAGGAGGAGGTAATGGCACA-------------------------
----

AACAATACTGAGACCTTCAGACCTGGAGGAGGAGATATGAGAGATAATTGGAGAAGTGAATTATATAAATATA
AGGTAGTAAGAATTGAGCCATTA---GGAGTAGCACCCACCAAGGCAAAGAGAAGAGTGGTG-----------
----------------------------CAGAGAGAAAAAAGAGCA---GTGGGAACAATAGGAGCT------
---ATGTTCCTTGGGTTCTTGGGAGCA---GCTGGAAGCACTATG---GGCGCAGCATCA---
GTGATGCTGACGGTACAG---GCCAGACAA---TTATTG---TCTGGTATAGTGCAACAGCAGAAT---
AATCTG---CTGAGA---GCTATTGAGGCGCAACAGCATATGTTGCAACTCACGGTCTGGGGCATC
>MG196689
TTCTATTGTAACTCAACAAAACTGTTCAACAGTATTTGGAATAGTAGTTTGATTAATGAGAATATTACTTGGA
ATGCTAAT---
-------------AGCACT------
GATGCCAATGACACTATCACACTCACATGCAGAATAAAACAAATTGTAAACATGTGGCAGGAAGTAGGAAAAG
CCATGTATGCCCCTCCCATCAGAGGACAAATTAGATGTGAATCAAAT---

ATTACAGGGCTGCTATTAACAAGAGATGGTGGTGATACCAGTAGC----------------------------
----

ACAAATGAGACCTTCAGACCTGGAGGAGGAGATATGAGGGACAATTGGAGAAGTGAATTATATAAATACAAGG
TAGTAAAAATTGAGCCATTA---GGAATAGCACCCACCAAGGCAAGGAGAAGAGTGGTG--------------
-------------------------CAGAGAGAAAAAAGAGCA---GTGGGA---CTAGGAGCT---------
TTTTTCCTTGGGTTCTTGGGAGCA---GCAGGAAGCACTATG---GGCGCAGCGTCA---
ATAACGCTGACGGTACAG---GCCAGGCAA---TTATTG---TCTGGTATAGTGCAGCAGCAAAAC---
AACTTG---CTGAGG---GCTATTGAGGCGCAACAACATCTGTTGCAACTCACAGTCTGGGGCATC
>MG196815
TTCTACTGTAATTCAACACAATTGTTTAATAGTACTTGGCATAGTAATAGTACTTTGAATAGTACTGAGAAT-
----

GTAAATAAAACTATCACACTCCCATGCAAAATAAAACAAATTGTAAACATGTGGCAGGAAGTAGGAAGAGCAA
TATATGCCCCTCCCATCAGAGGAGTAATCAGATGTTCATCAAAT---
ATTACAGGGCTGCTACTAACAAGAGATGGTGGTAAGAGTAGCAACACCAGCAAC-------------------
----

ACCACCGAGGAGGTCTTCAGACCTGAGGGAGGAAATATGAGGGACAATTGGAGAAGTGAATTATATAAATATA
AAGTAGTAAGAATTGAACCATTA---GGAGTAGCACCCACCAAGGCAAAGAGAAGAGTGGTG-----------
----------------------------CAGAGAGAAAAGAGAGCA---GTGGGAACACTAGGAGCT------
---ATGTTCCTTGGATTCTTGGGAACA---GCAGGAAGCACTATG---GGCGCAGCGTCA---
CTGGCGCTGACGGTACAG---GCCAGACAA---TTATTG---TCTGGAATAGTGCAACAGCAAAAC---
AATTTG---CTGAGA---GCTATTGAGGCGCAACAGCATCTGTTGCAACTCACAGTCTGGGGCATT
>MG196840
TTCTACTGTAATTCAACACCGTTGTTTAATAGTACTTGGTCTAATAATACTTGGAACGATACTGAAGAATTAA
GTGCC--
-------------------ACT---
GAAGGAAATGAAACAATCACACTCCCATGCAGAATAAAACAAATTATAAACATGTGGCAAGAAGTAGGAAAAG
CAATGTATGCCCCTCCCATCAGAGGACTGATTAGATGCTCATCAAAC---
ATTACAGGGCTGCTATTAACAAGGGATGGTGGTCCTAATAACACC----------------------------
----

AACGAAACCTTCAGACCTACAGGAGGAGATATGAGGGATAATTGGAGAAGTGAATTGTATAAATATAAAGTAG
TAGAACTTGAACCATTA---GGAGTAGCACCCACCAAGGCAAAAAGAAGAGTGGTG-----------------
----------------------CAGAGAGAAAAAAGAGCA---GTGGGA---CTAGGAACT---------
TTGTTCCTTGGGTTCTTGGGAGCA---GCAGGAAGCACTATG---GGCGCAGCATCA---
ATGGTGCTGACGGTACAG---GCCAGGCAA---TTATTG---TCTGGTATAGTGCAACAGCAGAAC---
AATTTG---CTGAGA---GCTATTGAGGCGCAACAGCATCTGTTGCAACTCACAGTCTGGGGCATC
>MG196846
TTCTATTGTGATACAGCAAGCCTGTTTAATAGTACTTGGCAGGCAAAGGAGACTGAAAGAAATGACACTGGA-
----

GGAAATGACACAATCACACTCCCCTGTAGAATAAAACAAATTATAAACATGTGGCAGGCAGTAGGGAAAGCAA
TATATGCCCCTCCTATTGCAGGGTTAATTAGATGTTCATCAAAT---
ATAACAGGGCTGCTGTTAACAAGAGATGGTGGTAATGAGACCATGACC-------------------------
----

AAGGATAACGAGACCTTCAGACCTGGAGAAGGAAATATGAGGGATAATTGGAGGAGTGAATTATATAAATATA
AAGTAGTAAAAGTTGAACCATTA---GGAATAGCACCCACCAAGGCAAGGAGAAGAGTGGTG-----------
----------------------------CAAAGAGAAAAAAGAGCA---GTGACA---ATAGGAGCT------
---GTGTTCCTTGGGTTCTTGGGAGCA---GCAGGAAGCACTATG---GGCGCAGCATCA---
GTGACGCTGACGGTACAG---GCCAGACAA---TTATTG---TCTGGTATAGTGCAGCAGCAAAAC---
AACTTG---CTGAAA---GCTATTGAGGCGCAACAGCATCTGTTGCAACTCACAGTCTGGGGCATC
>MG196942
TTCTACTGTAACACAACAGCACTGTTTAATAGCAGTTGGAATGAGAATAATACTTGGGTTAATAATACTGAAA
CA---

GGAGAGAGAAACATCACACTCCAATGTAGAATAAAACAAGTTATAAACTTGTGGCAGCAAGTAGGAAAAGCAA
TGTATGCCCCTCCCATCAGAGGGCAAATTAGATGTTCATCAAAT---

ATTACAGGGATACTATTAACAAGAGATGGTGGTGAGCCTAACAATACC-------------------------
----

CAAAATGAGACCTTTAGACCTGGGGGAGGAAATATGAAGGACAATTGGAGAAGTGAATTATATAAATATAAAG
TAGTAAGGATTGAACCATTA---GGAGTAGCACCCACCAAGGCAAAAAGAAGAGTGGTG--------------
-------------------------CAGAGAGAAAAAAGAGCA---GTGGGA---ATAGGAGCT---------
GTGTTCCTTGGATTCTTGGGAGCA---GCAGGAAGCACTATG---GGCGCAGCGTCA---
CTGACGCTGACGGTACAG---GCCAGACAA---TTATTG---TCTGGTATAGTGCAACAGCAGAGC---
AATTTG---CTGAGA---GCTATTGAGGCGCAACAGCATATGTTGCAACTCACAGTCTGGGGCATC
>MG197084
TTCTATTGTAATACAACAAAACTATTTGATAGTACTTGGAATGTTACTTGGAAAGAGGCAAATAACACTGGA-
----

GAGAATGACACAATCATCCTCCAGTGCAGGATAAAACAAATTATAAACATGTGGCAGGAAGTAGGAAAAGCAA
TGTATGCCCCTCCCATCAGAGGAAATATTAGTTGCTCATCAAAT---
ATTACTGGATTGCTATTAACAAGAGATGGTGGTGACGATAGGAAC----------------------------
----

ACCACCGAGATCTTTCGACCTGGAGGAGGAGATATGAGGGACAATTGGAGAAGTGAATTATATAAATATAAAG
TAGTACAAGTTGAACCATTA---GGAATAGCACCTACCAAGGCAAAAAGAAGAGTGGTG--------------
-------------------------CAGAGAGAAAAAAGAGCA---GTGGGA---ATAGGAGCT---------
CTGTTCCTTGGGTTCTTGGGAGCA---GCAGGAAGCACTATG---GGCGCAGCATCA---
ATGACGCTGACGGTACAG---GCCAGACAA---TTATTG---TCTGGTATAGTGCAACAGCAGAGC---
AATCTG---CTGAGA---GCTATTGAGGCGCAACAGCATCTGTTGCAACTCACAGTCTGGGGCATC
>MG197146
TTCTACTGTAATTCAATACCACTGTTTAATAGTACTTGGGATAGTAATAGTACTTGGGGTAATGATACCATAG
GG---

TTAAATGAAACTATTACACTCCAATGCAGAATAAAACAAATTATAAACATGTGGCAGGAAGTAGGAAAAGCAA
TGTATGCCCCTCCCATCAAAGGACAGATTGGATGTTCCTCAAAT---
ATTACAGGGCTATTGTTAACAAGAGATGGTGGTAATGCTAAGAGG----------------------------
----

GACAAAAACGACACAGAGATCTTCAGACCTGCAGGAGGAGATATGAGGGACAATTGGAGAAGTGAATTATATA
GATATAAAGTAGTAAGAATTGAACCATTA---GGAGTAGCACCCACCAAGGCAAGGAGAAGAGTGGTG-----
----------------------------------CAAAAAGAAAAAAGAGCA---GTGGGA---
ATAGGAGCT---------TTGTTCATTGGGTTCCTGGGAGCA---GCTGGGAGCACTATG---
GGCGCAGCGTCA---CTGACGCTGACGGTACAG---GCCAGACAA---TTGTTG---
TCTGGTATAGTGCAGCAGCAAAAC---AATTTG---CTGATG---
GCTATTGAGGCGCAACAGCATCTGTTGCAACTCACAGTCTGGGGCATT
>KM354470
TTCTACTGTAATACAACACAACTGTTTAATAGTACTTGGAATGATACAGAGTTA-------------------
----
AATAGTACTACAGGGAAT---------
AGTACTACAGACACAATCACACTCCCATGCAGAATAAAACAAATTATAAACATGTGGCAGGAGGTAGGAAAAG
CAATGTATGCCCCTCCCATAAGGGGATACATTAACTGTTCATCAAAT---
ATTACAGGGCTGCTATTAACAAGAGATGGAGGA--

-
GAGGGCAACAACAGGAGTGAGACCTTTAGACCTGGAGGAGGAAATATGAGGGACAATTGGAGAAGTGAATTAT
ATAAATATAAAGTAGTAAAAATTGAACCATTA---GGAATAGCACCCACCAAGGCAAAGAGAAGAGTGGTG--
-------------------------------------CAAAGAGAAAAAAGAGCA---
GTGGGAACAATAGGAGCT---------ATGTTCCTTGGGTTCTTGGGAGCA---GCAGGAAGCACTATG---
GGCGCAGCGTCA---ATAACGCTGACGGTACAG---GCCAGACAA---TTATTG---
TCTGGTATAGTGCAACAGCAGAAC---AATCTG---CTGAGA---
GCTATTGAGGCGCAACAACATCTGTTGCAACTCACAGTCTGGGGCATC
>KM354904
TTCTACTGTAACACAACACAACTGTTTAATAGTACTTGGACTAATAGTGGTGATGGT----------------
----
AATTGGACTAGTAGTGAT---------

AAGTTAAATGGCACTATCACACTCCCATGTAGAATAAGACAAATTATAAACATGTGGCAGGAAGTAGGAAAAG
CAATGTATGCCCCTCCCATCAGAGGATATATTAACTGCTCATCAAAT---
ATTACAGGAATACTATTAACAAGAGATGGTGGT--

ATTGAGAACAATACCAAC---
AGTACTGAGATCTTCAGACCTGGAGGAGGAGATATGAGGGACAATTGGAGAAGTGAGCTATATAAATATAAAG
TAGTACAAATTAAACCATTA---GGAGTAGCACCCACCGAGGCAAAAAGAAGAGTGGTA--------------
-------------------------CAGAGAGAAAAAAGAGCA---GCA------CTAGGAGCT---------
CTGTTCCTTGGGTTCTTGGGAGCA---GCCGGAAGCACTATG---GGCGCAGCGTCA---
ATGACGCTGACGGTACAG---GCCAGACAA---TTATTG---TCTGGTATAGTGCAACAGCAGAGC---
AATCTG---CTGAGG---GCTATTGAGGCGCAACAGCATCTGTTGCAACTCACAGTCTGGGGCATA
>KM355003
TTCTACTGTAATACATCACGACTGTTTAATAGTACTTGGAATAGTACTGGA----------------------
----
-TCAAATAACACTGAG---------
GGAAATGACACAACAATCACAATCCCATGTAAAATAAAACAAGTTATAAACCTGTGGCAGGAAGTAGGAAAAG
CAATGTATGCCCCTCCCATCAGAGGACAGATTAAATGCTCATCAAAT---
ATTACAGGGTTATTACTAACAAGAGATGGGGGT--

ATTAATGCCACAAACGGG---
ACCATGGAGATCTTCAGACCTGGAGGAGGAGATATGAGGGACAATTGGAGAAGTGAATTATACAAATATAAAG
TAGTACAAATTGAACCATTA---GGAATAGCACCCACCAGGGCAAAGAGAAGAGTGGTG--------------
-------------------------CAGAGAGAAAAAAGAGCA---GTAGGA---TTAGGAGCT---------
CTGTTCCTTGGGTTCTTGGGAGCA---GCAGGAAGCACTATG---GGCGCAGCGTCA---
GTGACGCTGACGGTACAG---GCCAGACAA---TTATTG---TCTGGTATAGTGCAGCAGCAGAAC---
AATCTG---CTGAGG---GCTATTGAGGCGCAACAGCATCTGTTGCAACTCACAGTCTGGGGCATC
>KM355044
TTCTACTGTAATTCAACAAAATTGTTTAATAGTACTTGGACGAATAGTAGTTTG-------------------
----
-GAAAATAATACTACA---------
GAGTCAAATATCAATATCACACTCCCATGCAGAATAAAACAGATTATAAACATGTGGCAGGGAGTAGGAAAAG
CGATGTATGCCCCTCCCATCAGTGGACCAATTAGATGTTCAACAAAT---
ATCACAGGGCTACTATTAACAAGAGATGGTGGG--

-
AATATCACTAATAACACTGAGGTCTTCAGACCTGGAGGAGGAGATATGAGAGACAATTGGAGAAGTGAATTAT
ATAAATATAAAGTAGTGAAAATTGAACCATTA---GGAGTAGCACCCACTAAGGCAAAGAGAAGAGTGGTG--
-------------------------------------CAGAGAGAAAAAAGAGCA---GTGGGA---
TTAGGAGCT---------ATGTTCCTTGGGTTCTTGGGAGCA---GCCGGAAGCACTATG---
GGCGCAGCGTCA---ATAACGCTGACGGTACAG---GCCAGACAA---TTATTG---
TCTGGTATAGTGCAACAGCAGAAC---AATCTG---CTGAGG---
GCTATTGAGGCGCAACAGCATCTGTTGCAACTCACAGTCTGGGGCATC
>KU901727
TTCTACTGCGATACAACACAACTGTTTAATAGTACTTGGAATTGGAATGATACTCGGACAGGGTCAAAT----
----

GGCAATGACAAGATCATACTCCCATGTAGAATAAGACAAATTGTAAATAGGTGGCAGGAGGTAGGAAAGGCAA
TGTATGCTCCTCCCATTAGAGGACAAATTAAATGCTCATCAAAC---
ATTACAGGGCTACTATTAACAAGAGATGGGGGCAATAACAATACA----------------------------
----

AATGAAAATGAGACCTTCAGACCTGGAGGAGGAGATATGAGGGACAATTGGAGAAGTGAATTATATAAATATA
AGGTGGTACAGGTTGAACCAATA---GGAATAGCACCCACCACGGCAAGGAGAAGAGTGGTG-----------
----------------------------CGAAGAGAAAAAAGAGCG---GTAACA---ATGGGAGCT------
---GTGTTCCTTGGGTTCTTGGGAACA---GCAGGAAGCACTATG---GGCGCAGCGTCA---
CTGACGCTGACGGTACAG---GTCAGACAA---TTATTG---TCTGGCATAGTGCAGCAGCAAAGC---
AATTTG---CTGAGG---GCTATTGAGGCGCAACAGCATCTGTTGCAACTCACAGTCTGGGGCATC
>KF384799
TTCTACTGTAATACAGCACAACTGTTTAATAGTATTTGGAATGGTACTGAGCCTAATGCTGAATGGCAACTA
AC---

AATAAAGCAAATATCACACTCCCATGCAGAATAAAACAAATTATAAACATGTGGCAGGAAGTAGGAAAAGCAA
TGTATGCCCCTCCCATCGAAGGACTAATTAGATGTACATCAAAT---
ATTACAGGACTATTACTAACAAGAGATGGTGGTAATAGCGGGAAAAATGATAGT-------------------
----

AATAATAACACTGAGATCTTCAGACCTGGAGGGGGAGATATGAGAGACAATTGGAGAAGTGAATTATATAAAT
ATAAAGTAGTAAAAGTTGAACCATTA---GGAGTAGCACCCACCAGGGCAAAGAGAAGAGTGGTG--------
-------------------------------CAGAGAGAAAAAAGAGCA---ATAGGC---CTAGGAGCT---
------GTGTTCATTGGGTTCTTGGGAGCA---GCAGGAAGCACTATG---GGCGCAGCGTCA---
GTGACGCTGACGGTACAA---GCCAGACAA---TTATTG---TCTGGTATAGTGCAACAGCAGAAT---
AATCTG---CTGAGG---GCTATTGAGGCGCAGCAGCACCTGTTACAACTCACAGTCTGGGGCATC
>KF384805
TTCTACTGTAATACAACACAACTGTTTAATAGTACTTGGTTGTCAAATAGTACTTGGATGTCAAATAACACTG
GAGGA--

AATGACACAACAATCATACTACCATGCAGAATAAAACAAATTATAAACATGTGGCAGGAAGTAGGAAAAGCAA
TGTATGCCCCTCCCATTCCAGGACAAATTAGCTGTTTATCAAAC---
ATTACAGGGCTACTATTAACAAGAGATGGTGGGAATGTGAGCAACAGC-------------------------
----

AACACCACCGAGATCTTCAGACCTGTAGGAGGAGATATGAGGGACAATTGGAGAAGTGAATTATATAAATATA
AAGTAGTAAAAATTGAACCATTA---GGAGTAGCACCCTCCAAGGCAAAAAGAAGAGTGGTG-----------
----------------------------CAGAGAGAAAAAAGAGGA---GCG---------GGACTG------
---GTGTTCCTTGGGTTCTTGGCAACA---GCAGGAAGCACTATG---GGCGCGGCGTCA---
CTGACGCTGACGGTACAG---GCCAGACAA---TTATTG---TCTGGTATAGTGCAACAGCAGAAC---
AATCTG---CTGAGA---GCTATTGAGGCGCAACAACATCTGTTGCAACTCACAGTCTGGGGCATT
>KF384806
TTCTACTGTAATACATCACAATTGTTTAATAGTACTTGGTTTGGGAATACTACTAAACAGTCAAATGAC----
----

ACTACAGAATATATCAGACTCCCATGCAGAATAAAACAAATTGTAAACCTGTGGCAGGAAGTAGGAAAAGCAA
TGTATGCCCCTCCCATCAGCGGAGAAATTAACTGTACATCAAAT---
ATTACAGGGCTGCTACTAACAAGAGATGGTGGTAATGATAATAACACA-------------------------
----

AATGGGACTGAGACCTTTAGACCTGGAGGAGGAGATATGAGGGACAATTGGAGAAGTGAATTATACAAGTATA
AGGTAGTACAAATTGAACCCTTA---GGGGTAGCGCCCACCAAGGCAAAGAGAAGAGTGGTG-----------
----------------------------CAGAGAGAAAAAAGAGCA---ATAGGA---CTAGGAGCT------
---TTATTCCTTGGGTTCTTGGGAGCA---GCAGGAAGCACAATG---GGCGCAGCGTCA---
ATGACGCTGACGGTACAG---GCCAGACTA---TTATTG---ACTGGTATAGTGCAACAGCAAAAC---
AACTTG---CTGAGG---GCTATTGAGGCGCAACAGCATCTGTTGCAACTCACAGTCTGGGGCATC
>KC473831
TTCTACTGTAATTCAACACAACTGTTTAATAGTAAATGGGAGGGTAATAGTACTTGGAATAGTACTGGAGAGT
CAAATAAC---

ACTGCAGAAAACATCATACTCCCATGCAGAATAAAGCAAATTGTAAACATGTGGCAGGAAGTAGGAAAAGCCA
TATATGCCCCTCCCATCAGCGGACCAATTAGATGCTCAACAAAT---
ATTACAGGGCTGCTATTAACAAGAGATGGGGGAAAGGATGAGAAC----------------------------
----

CAAACAGAGATCTTTAGACCTGGAGGAGGAGATATGAGGGACAATTGGAGAAGTGAATTATACAAATATAAAG
TAGTAAAAATTGAACCATTA---GGAGTAGCACCCACCAAGGCAAAGAGAAGAGTGGTG--------------
-------------------------CAGAGAGAAAAAAGAGCA---GTGGGA---ATGGGAGCT---------
CTGTTCCTTGGTTTCTTGGGAGCA---GCAGGAAGCACTATG---GGCGCAGCGTCA---
ATAACGCTGACGGCACAG---GCTAGACAA---TTATTG---TCTGGTATAGTGCAACAGCAGAGC---
AATTTG---CTGAGG---GCTATTGAGGCGCAGCAACATCTGCTGCAACTCACAGTCTGGGGCATC
>KC473833
TTCTACTGCAATACATCAGCACTGTTTAATAGTACTTGGAATAGTACTATAAATACAAGT-------------
----

GAAAATGACACAATCATACTCCCATGCAGAATAAAACAAATTATAAATCTGTGGCAGGAAGTAGGAAGAGCAA
TGTATGCTCCTCCCATCAGGGGAAACATTAGCTGTACATCAAAT---
ATTACGGGGGTGCTATTAACAAGAGATGGTGGCGATGACCCTAACGGG-------------------------
----

ACCAACGACACCGAGACCTTCAGACCTGGAGGAGGAGATATGAGGGACAATTGGAGAAATGAATTGTATAAAT
ACAAAGTAGTAAAAATTGAACCATTG---GGAATAGCACCCACCAGGGCAAAGAGAAGAGTGGTG--------
-------------------------------CAAAGAGAAAAAAGAGCA---GTGGGA---ATAGGAGCA---
------TTGTTCCTTGGGTTCTTGGGAGCA---GCAGGAAGCACTATG---GGCGCAGCGTCA---
GTGACGCTGACGGTACAA---GCCAGGCTA---TTATTG---TCTGGTATAGTGCAACAGCAGAAC---
AATTTG---CTGAGA---GCTATTGAGGCGCAACAGCATCTGTTGCAACTCACAGTCTGGGGCATC
>KC473834
TTCTACTGTAATACAACAAAACTGTTTAATGGTAATTTGACTAATACTTGGAATGGAAATTGGTCTGAT----
----

ACTAATGGTACTATCATACTCCAGTGCAGAATAAAACAGATTGTAAACATGTGGCAGGAAGTAGGAAAAGCAA
TGTATGCTCCTCCCATCAAAGGACAGATTAACTGCACATCAAAT---
ATTACAGGATTGCTATTAACAAGAGATGGTGGCACAGGCAATGACAGT-------------------------
----

GACAGCAAGGAGACCTTCAGACCTGGAGGAGGAGATATGAGAGACAATTGGAGAAGTGAATTATATAAATATA
AAGTAGTAAAAGTTGAACCATTA---GGAATAGCACCCACCAAGGCAAAGAGAAGAGTGGTG-----------
----------------------------CAGAGAGAAAAAAGAGCA---GTGGGA---ATAGGAGCT------
---GTGTTCCTTGGGTTCTTGGGAGCA---GCAGGAAGCACTATG---GGCGCAGCGTCA---
ATAACGCTGACGGTACAG---GCCAGGCTA---TTATTG---TCTGGTATAGTGCAGCAGCAGAAC---
AATCTG---CTGAGG---GCTATTGAGGCGCAACAGCATCTGTTGCAACTCACAGTCTGGGGCATT
>JN397362
TTCTACTGTAATACAACAAAACTGTTTAACAGTACTTGGGAAAAAACAAATGATACTTGGAGTGGTAATGAAA
CGTTA--
-------------------AAT---
GGCACAATCACACCAATCACACTCCCATGCAGAATAAAACAAATAATAAACATGTGGCAGAAAATAGGAAAAG
CAATGTATGCCCCTCCTATTCCAGGACCAATTAACTGTTCATCAAAT---
ATTACAGGGCTGATATTAACAAGAGATGGTGGAGGTGATAGGAGTAAT-------------------------
----

GCCACCAACGAGACCTTCAGACCTGGAGGAGGAGATATGAGGGACAATTGGAGAAGTGAATTATATAAATATA
AAGTAGTAAAAATTGAACCATTA---GGGTTAGCACCCACCAAGGCACAGAGAAGAGTGGTG-----------
----------------------------CAGAGAGAAAAAAGAGCT---GTGGGA---TTTGGAGCG------
---CTGTTCCTTGGGTTCTTGGGAGCA---GCAGGAAGCACTATG---GGCGCAGCGTCA---
GTGACGCTGACGGTACAG---GCCAGACAA---TTATTG---TCTGGTATAGTGCAACAGCAGAAC---
AATTTG---CTGAGA---GCTATTGAGGCGCAACAGCACCTGTTGCAACTCACAGTCTGGGGCATC
>KF384810
TTCTACTGTAATACAACACAGCTGTTTAATAGTACTTGGGAATGGAATAGTACCAATTGGAGTAATAACACT-
----

AGCAATGAAAATATCATACTCCCGTGCAGAATAAGACAAATTGTAAACAGGTGGCAGGAAGTAGGAAAAGCAA
TGTATGCCCCTCCCATCAGTGGACGGATTAGTTGTTCCTCAAAT---
ATTACAGGACTGCTACTAACAAGAGATGGTGGTGATAGTAATGATACA-------------------------
----

AATAATACTGAGATCTTCAGACCAGGAGGAGGAGATATGAGGGACAATTGGAGAAGTGAATTATATAAATATA
AAGTAGTAGAGATTGAACCATTA---GGAATAGCACCCACCAAGGCAAAAAGGAGAGTGGTG-----------
----------------------------CAGAGAGAAAAAAGAGCA---ATAACG---CTGGGAGCT------
---TTATTCCTCGGGTTCTTGGGAACA---GCAGGAAGCACTATG---GGCGCAGCGTCA---
ATGACGCTGACGGTACAG---GCCAGGCAA---TTATTG---TCTGGTATAGTGCAACAGCAGAAT---
AATCTG---CTGAGA---GCTGTTGAGGCGCAACAGCATATGTTGCAACTCACAGTCTGGGGCATC
>KM516891
TTCTACTGTAATACAACACAACTGTTTGATAATACTTGGAACAGCACTAAG----------------------
----

```
-GATGCGACTGGTACT---------
GAAAGAAATGATACTATTACACTTCCATGTAGAATAAAACAAATTATCAATATGTGGCAAAGAGTAGGAAAAG
CAATATATGCCCCTCCCATCAGAGGACAAATTAATTGTACATCAAAT---
ATTACAGGGCTGTTATTAACAAGAGATGGTGGT----------------------------------------
-----------------------------------------------------------------
GTGAACGTGACAACCAAC---
AACACTGAGACCTTCAGACCAGGAGGAGGAGATATGAGGGATAATTGGAGAAGTGAATTATATAAATATAAAG
TAGTAAGAATTGAACCATTA---GGAATAGCGCCCACCAAGGCAAAGAGAAGAGTGGTG--------------
-------------------------CAAAGAGAGAGAAGAGCA---GTA------CTGGGAGCT---------
ATGTTCCTTGGGTTCTTGGGAGCA---GCAGGAAGCACTATG---GGCGCAGCGGCA---
GTGACGCTGACGGTACAG---GCCAGACAA---TTATTG---TCTGGTATAGTGCAACAGCAGAGC---
AATTTG---CTGAGG---GCTATTGAGGCGCAACAGCATCTGTTGCAACTCACAGTCTGGGGCATT
>KU901848
TTCTACTGTAATACAACAAAACTGTTTAATAGTACTTGGAATAGTACTGAAGAGTCAAATAACACTACTGCA-
-------------------------------------------------------------------------
----------------------------
GGAAATGATATAATCAAACTCCCATGCAGAATAAAACAAATTGTAAACATGTGGCAGGAGGTAGGAAAAGCAA
TGTATGCCAATCCCAACAGAGAACTAATTAGATGTTCATCAAAT---
ATTACGGGGCTGCTATTAACAAGAGATGGTGGTGTCACCAACACCACCAATGAG-------------------
-------------------------------------------------------------------------
----------
GCCAAAATTGAGACCTTCAGACCAGGAGGAGGAGATATGAGGGACAATTGGAGAAGTGAATTATATAAATATA
AAGTAGTACAAATTGAACCATTA---GGAGTAGCACCCACCAGGGCAAAGAGAAGAGTGGTG-----------
----------------------------CAAAGAGAAAAAAGAGCA---GTGGGA---ATAGGAGCT------
---TTGTTCCTTGGGTTCTTGGGAGCA---GCAGGAAGCACTATG---GGCGCAGCGGCA---
GTGACGCTGACGGTACAG---GCCAGACAA---TTATTG---TCTGGTATAGTGCAACAGCAAAAC---
AACTTG---CTGAGG---GCTATTGAGGCGCAACAGCATCTGTTGCAACTCACAGTCTGGGGCATC
>KF384812
TTCTATTGTAATACAACAGAACTGTTTAATAGTACTTGGAATGATACTGAAGGGTCAAATAACACTGGGGGGT
CAGAAAAC-----------------------------------------------------------------
----------------------------
ACTGACAACACAATCACACTCCCATGTAGAATAAAACAATTTATAAACATGTGGCAGGAAGTAGGAAAAGCAA
TGTATGCCCCTCCCATCAGAGGACAGATTAGTTGTGTATCAAAC---
ATCACAGGGCTGCTATTACTAAGAGATGGTGGTAAAAAGAATGAGACTGAC----------------------
-------------------------------------------------------------------------
-------
GGGAACGAGACTGAGACCTTCAGACCTGGAGGAGGAGATATGAGGGACAATTGGAGAAGTGAGTTATATAAAT
ATAAAGTAGTGAAAATTGAACCATTA---GGAGTAGCACCCACCAAGGCAAAGAGAAGAGTGGTG--------
-------------------------------CAGAGAGAAAAAAGAGCA---GTGGGA---ATAGGAGCT---
------GTGTTCCTTGGATTCTTGGGAGCA---GCAGGAAGCACTATG---GGCGCAGCGTCA---
ATGACGCTGACGGTACAG---GCCAGACTA---TTATTG---TCTGGTATAGTGCAACAGCAGAAC---
AATCTG---CTGAGA---GCTATTGAGGCGCAACAGCATCTGTTGCAACTCACAGTCTGGGGCATT
>KF384813
TTCTACTGTAATTCAACACAACTGTTTAATAGTACTTGGACTGGTAATGAGTCAGATAGTACTAAC-------
-------------------------------------------------------------------------
----------------------------
AAAAGTGTCACAATCACACTCCCATGCAGAATAAAACAAATTATAAACATGTGGCAGGAAGTAGGAAAAGCAA
TGTATGCCCCTCCCATTAGAGGACAAATTAGATGTTCATCAAAC---
ATTACAGGGCTGCTCTTAACAAGAGATGGTGGTATAGACAACAAGACAGAC----------------------
-------------------------------------------------------------------------
-------
AAGAACGAGACCGAGATCTTTAGGCCTGCAGGAGGAGATATGAGGGACAATTGGAGAAGTGAATTATATAAAT
ATAAAGTAGTAAAAATTGAACCGTTA---GGAGTAGCACCCACCAGGGCAAAGAGAAGAGTGGTG--------
-------------------------------CAGAGAGAAAAAAGAGCA---GTGGGA---ATAGGAGCT---
------ATGCTCCTTGGGTTCTTGGGAGCA---GCAGGAAGCACTATG---GGCGCAGCGTCA---
ATGACGCTGACGGTACAG---GCCAGACTA---TTATTG---TCTGGTATAGTGCAACAGCAGAAC---
AATTTG---CTGAGG---GCTATTGAGGCGCAACAGCATCTGTTGCAACTCACAGTCTGGGGCATC
>MK383400
TTCTACTGTAATTCAACACCACTGTTTAATAGTACCTGGAATTGGAATGATACTAAAGGGTCAAATAGCACTG
GAGAA--------------------------------------------------------------------
```

AATAGCACACTCATCACACTCCAATGCAGAATAAAACAAATTATAAACATGTGGCAGGAAGTAGGAAGAGCAA
TGTATGCCCCTCCCATTAGAGGAAACATTAGCTGTTCATCAAAT---
ATTACAGGGCTGCTATTAACAAGAGATGGTGGTAACCAGACTAAC---------------------------
----

AACACCGAGATCTTCAGGCCTGAAGGAGGAAATATGAAGGATAATTGGAGAAGTGAATTATATAAATATAAAG
TAGTAAGAATTGAACCATTA---GGAGTAGCACCCACCAAGGCAAGGAGAAGAGTGGTG-------------
------------------------CAGAGAGAAAAGAGAGCA---GTGGGA---ATAGGAGCT---------
TTGTTCCTTGGGTTCTTGGGAGCA---GCAGGAAGCACTATG---GGCGCAGCGTCG---
ATGACGCTGACGGTACAG---GCCAGACTA---TTACTG---TCTGGTATAGTGCAACAGCAGAAC---
AATTTG---CTGAGG---GCTATTGAGGCGCAACAGCGTCTGTTGCAACTCACAGTCTGGGGCATC
>KY778473
TTCTACTGTAATTTAACACAGTTGTTTAATAGTACTTGGGGGCATAATAATTCAATACAATTGGATAATAGTA
CTTGGGAT--

ACTAATAATACTATCACACTCCCATGTAAGATAAAACAAATTATAAACATGTGGCAAGAAGTAGGAAAAGCAA
TGTATGCCCCTCCCATCAGAGGACAAATTAAATGTATATCAAAT---
ATTACAGGGCTGTTATTAACAAGAGATGGTGGTAGCAATACCACC---------------------------
----

AACGAAACCTTCAGACCTGGAGGAGGAGATATGAGGGACAATTGGAGAAGTGAATTATATAAATATAAAGTAG
TAAAAATTGAGCCATTA---GGAATAGCACCCACCAAGGCAAAGAGAAGAGTGGTG----------------
---------------------CAGAGAGAAAAAAGAGCA---GTGGGA---GTGGGAGCT---------
CTGCTCATTGGGTTCTTGGGAGCA---GCAGGAAGCACTATG---GGCGCAGCGTCA---
ATAACGCTGACGGTACAA---GCCAGACAA---TTGTTG---TCTGGTATAGTGCAACAGCAGAGC---
AATCTG---CTGAAA---GCTATTGAGGCGCAACAGCATCTGTTGCAACTCACAGTCTGGGGCATT
>KT124756
TTCTACTGCAATACAACACAACTGTTTAATAGCAGTTGGACAGATGGGCAAGAGCTAAATAACACTGGGCAAG
AG--

CTAAATAGCACTATCACGCTCCCGTGCAGAATAAAACAAATTATAAACAGGTGGCAGGAAGTAGGAAAAGCAA
TGTATGCTCCTCCCATTAGTGGACCAATTAAATGTACATCCAAT---
ATTACAGGGCTGTTATTAACAAGAGATGGTGGTAACTCTAACTCTAGTGGGAAG------------------
----

AACGACACTGAGACTTTCAGACCTGGAGGAGGAGATATGAGGGACAATTGGAGAAGTGAATTATATAAATATA
AGGTAGTAAAAATTGAACCATTA---GGAGTAGCACCCACCAAGGCAAGGAGAAGAGTGGTG----------
---------------------------CAGAGAGAGAAAAGAGCA---GTGGGA---ATAGGAGCT------
---GTGTTCCTTGGATTCTTGGGAGCA---GCAGGAAGCACTATG---GGCGCAGCGTCA---
TTAACGCTGACGGTACAG---GCCAGACAA---TTATTG---TCTGGTATAGTGCAACAGCAGAAC---
AATTTG---CTGAGG---GCTATTGAGGCGCAACAGCATCTGTTGCAACTCACAGTCTGGGGCATC
>MH897911
TTCTACTGTAATACATCAAAACTATTTAATAGTACTTGGCAGACTGATAATAGTACTTGGCAGCCTGAAGCT-
----

GAGAATAACACTATCACACTCCAATGCAGAATAAGACAAATTATAAACATGTGGCAAGAAGTAGGAAAAGCAA
TGTATGCCCCTCCCATCAGTGGACAAATTAGATGTTCATCAAAT---
ATTACAGGACTGCTACTAACAAGAGATGGTGACAATGAGAACAAC---------------------------
----

AGCACTGAGGTCTTCAGACCTGCAGGAGGAGATATGAGGGACAATTGGAGAAGTGAATTATACAAATATAAAG
TAGTAAAAATTGAACCATTA---GGAATAGCACCCACCAAGGCAAAGAGAAGAGTGGTG-------------
------------------------CAAAGAGAGAAAAGGGCT---GTGACG---CTGGGAGCC---------
TTGTTCCTTGGGTTCTTGGGAGCA---GCAGGAAGCACTATG---GGCGCAACGTCA---
ATGACGTTGACGGTACAG---GCCAGACAG---TTATTG---TCTGGTATAGTGCAACAGCAGAGC---
AATCTG---CTGAGA---GCTATTGAGGCGCAACAGCATCTGTTGCAACTCACAGTCTGGGGCATC
>MH897913
TTCTACTGTAATACAACAAAACTATTTAATAGTACTTGGACAAATGGTACTGAAGAGGCAAATAACACTGGAG
GA--

AATGACACACATATCACACTCCCATGCAGAATAAAACAAATTATAAACATGTGGCAGGGAGTAGGAAAAGCAA
TGTATGCCCCTCCCATCAAAGGACAAATCAGATGTTCATCAAAT---
ATTACAGGGCTGCTATTAACAAGAGATGGTGGTAATAACAATAACGGG-------------------------
----

ACGACCGAGATCTTCAGACCTGGGGGAGGAGATATGAGGGACAATTGGAGAAGTGAATTATATAAATATAAAG
TAGTAAAAATAGAACCATTA---GGAGTAGCTCCCACCAAGGCAAAGAGAAGAGTGGTG--------------
-------------------------CAAAGAGAAAAAAGAGCA---GTGGGAACAATAGGAGCT---------
ATGTTCCTTGGGTTCTTAGGAGCA---GCAGGAAGCACTATG---GGCGCAGCGTCA---
ATGACACTGACGGTACAG---GCCAGACAA---TTATTG---TCTGGTATAGTGCAACAGCAAAAC---
AATTTG---CTGAGG---GCTGTTGAGGCGCAACAGCATCTGTTGCAACTCACAGTCTGGGGCATC
>MH897916
TTCTACTGTAATACAACACAACTATTTAATAGTACTTTTAATAGTCCTTGGAATGATACTAAAATGTCAAATG
GCACTGAA---

GGATATAACACAACCATCACACTCCAATGCAGAATAAAACAAATTATAAACAGGTGGCAGGAAGTGGGAAAAG
CAATATATGCCCCTCCCATCCAAGGACAAATTAATTGTTCATCAAAT---
ATTACAGGGCTGCTCTTAGTGAGAGATGGTGGTATTGATAATGCCACA-------------------------
----

AACAATATTGAAACCTTCAGACCTGGAGGAGGAGATATGAGGGACAATTGGAGAAGTGAATTATATAGATATA
AAGTAGTAAAAATTGAACCATTA---GGAGTAGCACCCACTAAGGCAAGGAGAAGAGTGGTG-----------
----------------------------CAGAGAGAAAAAAGAGCA---ATTGGA---CTAGGAGCT------
---ATGTTCCTTGGGTTCTTGGGAGCA---GCAGGAAGCACTATG---GGCGCAGCATCA---
ATAACGCTGACGGTACAG---GCCAGACAA---TTATTG---TCTGGTATAGTGCAACAGCAAAAC---
AACTTG---CTGAGG---GCTATTGAGGCGCAACAACATCTGTTGCAACTCACAGTCTGGGGCATC
>MH897917
TTCTACTGCAATACAACACCACTGTTTGATAATGTTTGGAATGGCACTAAGTTTACAAAGAATTTTACAAAGA
ATAAA--

GGAGAGAATATCACTACTCTCTCATGCAGAATAAAACAATTTATAAACCTGTGGCAGGAAGTAGGGAAAGCAA
TGTATGCCCCTCCCATCAGAGGAGAAATTAACTGTTCATCCAAT---
ATTACAGGACTGATACTAACAAGGGAGGGTGGTGTTAACAGGACT----------------------------
----

GACAATGACACCTTCAGACCTATAGGAGGAAATATGAAGGACAATTGGAGAAGTGAATTGTACAAATATAAAG
TAGTAAGAATTGAACCATTA---GGAGTAGCACCCGACAAGGCAAAGAGAAGAGTGGTG--------------
-------------------------CAGAGAGAAAAAAGAGCA---GTGGGA---CTAGGAGCG---------
TTGATCCTTGGGTTCTTAGGAACA---GCAGGAAGCACTATG---GGCGCAGCGTCA---
CTGACGCTGACGGTACAA---GCCAGACAA---TTATTG---TCTGGTATAGTGCAACAGCAGAGC---
AATTTG---CTGAGG---GCTATTGAGGCGCAACAACATCTGTTGCAACTCACAGTCTGGGGCATC
>MG196702
TTCTACTGTAATACAACACAACTGTTTAATAGTACTTGGCCTTTGAATGATACTGAAGGGACAAAT-------
----

AGAACTGAAAATATCACGCTCCCATGCAGAATAAGACAAATTGTAAACATGTGGCAGGAAGTAGGAAAAGCAA
TGTATGCCCCTCCCATCAAAGGACAAATTAATTGTGTATCAAAT---
ATTACAGGGCTGTTATTAACAAGAGATGGTGGTATTAACTCTACA----------------------------
----

AAGAATGAGACCTTTAGACCTGGAGGAGGAGATATGAGGGACAATTGGAGAAGTGAATTATATAAATATAAAG
TAGTAAGAATTGAACCATTA---GGAGTAGCACCCACCAAAGCAAGGAGAAGAGTGGTG--------------
-------------------------CAGAGAGAAAAAAGAGCA---GTGGGA---CTAGGAGCT---------
TTGTTCCTTGGGTTCTTGGGAGCA---GCAGGAAGCACTATG---GGCGCAGCGTCA---
ATAACGCTGACGGCACAA---GCCAGGCAA---TTATTG---TCTGGTATAGTGCAACAGCAGAAC---
AATCTG---CTGAGG---GCTATTGAGGCGCAACAGCATCTGTTGCAACTCACAGTCTGGGGCATC
>MG196732
TTCTACTGTAATACAACACAATTGTTTGAAGAGTCAAATAACACTGAAAACAACACTAAAAGAGAAAATATCA
CACTT--

GAAAGAGAAAATATCACACTCCCATGCAGAATAAGACAAATTGTAAACCTGTGGCAGAAAGTAGGCAAAGCAA
TGTATGCCCCTCCCATCAGAGGACAAATTAGCTGTTCATCAAAC---
ATTACAGGACTGTTATTAACAAGAGATGGTGGCAATAATAATAATACT------------------------
----

GATGATCCGGAGATCTTCAGACCTGGAGGAGGAGATATGAGGGACAATTGGAGAAGTGAATTATACAAATATA
AAGTAGTAAAAATTGAACCATTA---GGAATAGCACCCACCAAGGCAAAGAGAAGAGTGGTG-----------
----------------------------CAGAGAGAAAAAAGAGCA---ATAGGGGGACTAGGAGCT------
---TTGTTCCTTGGATTCTTGGGGGCA---GCAGGAAGCACTATG---GGCGCAGCGTCA---
GTGACGCTGACGGTACAG---GCCAGACAA---TTACTG---TCTGGTATAGTGCAACAGCAAAAC---
AATTTG---CTGAGA---GCTATTGAGGCGCAGCAACATATGTTGCAACTCACAGTCTGGGGCATC
>MG196767
TTCTATTGTAATATAACACAGTTATTTAATAGTACTTGGCTGGGTAATGGTACTGGGAATAGT----------
----

ACTACAGGCAATATCACACTCCCATGCAGAATAAAACAAATTATAAATAGGTGGCAGGAAGTAGGAAAAGCAA
TGTATGCCCCTCCCATCAGCGGAGAGATCAGCTGTAGTTCAAAT---
ATTACAGGGCTATTATTAACAAGAGATGGTGGTAAGCCCGGAAACGCCGCC----------------------
----

GAGAGCATCAATGAGACCTTCAGACCTGGAGGAGGAAATATGAAGGACAATTGGAGAAGTGAATTATATAAAT
ATAAAGTAGTAAAGATTGAACCATTA---GGAGTAGCACCCACCAAGGCAAAGAGAAGAGTGGTG--------
-------------------------------CAAGGAGAAAAAAGAGCA---GTGGGAACATTTGGAGCT---
------CTGTTCCTTGGGTTCTTGAGTGCA---GCGGGAAGCACTATG---GGCGCAGCGTCA---
ATGACGCTGACGGTACAG---GCCAGACAA---TTATTG---TCCGGTATAGTGCAACAGCAGAAC---
AACTTG---CTGAGA---GCTATTGAGGCGCAACAGCATATGTTGCAACTCACAGTCTGGGGCATC
>MG196819
TTCTACTGTGATTCAACAAAACTGTTTAATAGTACTTGGAATGCTAATAGTACTTGGAATAATACTGAA----
----

GAGCCAGAGAACATCACCCTCCCATGTAGAATAAAACAATTTATCAACATGTGGCAGGAAGTAGGAAAAGCAA
TGTATGCCCCTCCCATCAGAGGACCAATTAAATGTTCATCAAAT---
ATTACAGGGATACTATTGACTAGAGATGGTGGTAATGGTAGCAGCACCGAG----------------------
----

AACAATACCGAGACCTTCAGACCTGGAGGAGGAGACATGAGGGATAATTGGAGAAGTGAATTATATAAATACA
AAGTGGTAAAAATTGAACCATTA---GGAGTAGCACCCACCAGGGCAAAGAGAAGAGTGGTG-----------
----------------------------CAGAGAGAAAAGAGAGCA---GTGACA---CTGGGAGCT------
---TTGTTCCTTGGGTTCTTGGGAGCA---GCAGGAAGCACTATG---GGCGCAGCGTCA---
ATGACGCTGACGGTACAA---GCCAGACAA---TTATTG---TCTGGTATAGTGCAACAGCAAAAC---
AATTTG---CTGAGG---GCTATTGAGGCGCAGCAGCATCTGTTGCAACTCACAGTCTGGGGCATC
>MG196866
TTCTACTGTAATTCAACACCACTGTTTAATAGTAGTTGGCATAGTACTACTAAGTATAGTACTAACAGGACAA
CTGAA--

GGAAATGACACAATCACACTCCAATGTAGAATAAAACAAATTATAAACATGTGGCAGGAAGTAGGAAAAGCAA
TGTACGCCCCTCCCATCAGCGGACCAATTGGATGTTTATCAAAT---
ATTACAGGGCTGCTATTAACAAGAGATGGTGGTACTAACATGAGC----------------------------
----

GATAACGAAACTTTCAGACCTGGAGGAGGAGATATGAGGGACAATTGGAGAAGTGAATTATATAAATATAAAG
TAGTGAAAATTGAACCATTA---GGAGTAGCACCCACTCGGGCAAGGAGAAGAGTGGTG--------------
-------------------------CAGAGAGAAAAAAGAGCA------GGA---CTAGGAGCT---------
CTGCTCCTTGGGTTCTTGGGAGCA---GCAGGAAGCACTATG---GGCGCAGCGTCA---
ATAACGCTGACGGCACAG---GCCAGAAAA---TTATTG---TCTGGTATAGTGCAACAGCAGAAC---
AATCTG---CTGAGG---GCTATTGAGGCGCAACAGCATCTGTTGCAACTCACAGTCTGGGGCATC
>MG196902
TTCTACTGTAATACAACACAGCTGTTTAATAGTACTTGGCTGTTTAATGATACTATGAATAATGCTACAGGAT
CA---

AACAGCAGTGATATCATACTCCCATGCAGAATAAAACAAATTATAAACAGGTGGCAGGAAGTAGGAAAAGCAA
TGTATGCCCCTCCCATAGCAGGAATAATTAAATGTTCATCAAAT---
ATTACAGGGCTACTATTAACAAGAGATGGTGGTAATAGTAACAAC----------------------------
----

ACCAAGGAGACCTTCAGACCTGGAGGAGGAGATATGAGGGACAATTGGAGAAGTGAATTATATAAATATAAAG
TAGTAAAAATTAAGCCATTA---GGAGTAGCACCCACCAAGGCCAAGAGAAGAGTGGTG--------------
-------------------------CAGAGAGAAAAAAGAGCA---GTAACA---ATAGGAGCC---------
TTTTTCCTTGGGTTCTTGGGAGCA---GCAGGAAGCACTATG---GGCGCAGCGTCG---
GTGACGCTGACGGTACAG---GCCAGACAA---CTATTG---TCTGGTATAGTGCAACAGCAGAGC---
AATCTG---CTGAGA---GCTATTGAGGCGCAACAGCATATGTTGCAACTCACAGTCTGGGGCATA
>MG196921
TTCTACTGTAACTCAACACCACTGTTTGATAGTACTTGGAATAGTAATAGTACAGGGTTAAATAGCACCAAA-
----

ACCAATAACACAATCACACTCCAATGCAGAATAAAACAAATTATAAACATGTGGCAGGAAGTAGGAAGAGCAA
TGTACGCCCCTCCCATCAGTGGACACATTAACTGTTCATCAAAT---
ATCACAGGGCTGCTGTTAACAAGAGATGGTGGTGTTGACAAGAAT----------------------------
----

GGCAGTGAGACCTTTAGACCTGGAGGAGGAGATATGAGGGACAATTGGAGAAGTGAATTGTATAAATATAAAG
TAGTAAAAATTGAACCATTA---GGAGTAGCACCCACCAAGGCAAAGAGAAGAGTGGTG--------------
-------------------------CAGAGAGAAAAAAGAGCA---GTGGGA---TTAGGAGCT---------
ATGTTCCTTGGGTTCTTGGGAGCA---GCAGGAAGCACTATG---GGCGCAGCGTCA---
CTGACGCTGACGGTACAG---GCCAGACAA---TTACTG---TCTGGTATAGTGCAACAGCAGAAC---
AATTTG---CTGAGG---GCCATTGAGGCGCAACAGCATCTGTTGCAACTCACAGTCTGGGGCATC
>MG196922
TTCTACTGTAATACAACAAAACTGTTTAATGGGACTGCAGAACAGATAGAAAATAACACTAAAGCTGAA----
----

AATATCACAACAATCATACTCCCATGCAGAATAAAACAAATTGTAAACAGGTGGCAGGAAGTAGGAAAAGCAA
TGTATGCCCCTCCCATCCAAGGACGAATTAGCTGTTCATCAAAT---
ATTACAGGGCTGCTATTAACAAGAGATGGTGGGAACAATACTAGT----------------------------
----

AACAACGAGACCTTCAGACCTGCAGGAGGAGATATGAGGGACAATTGGAGAAGTGAATTATATAAATATAAAG
TAGTGAGAATTGAGCCATTA---GGAGTAGCACCCACCAAGGCAAAAAGAAGAGTGGTG--------------
-------------------------CAGAGAGAAAAAAGAGCA---GTGGGAGCACTAGGAGCT---------
ATGTTCCTTGGGTTTCTGGGAACA---GCAGGAAGCACTATG---GGCGCAGCGGCA---
GTGACGCTGACGGTACAG---GCCAGGCAA---TTATTG---TCTGGTATAGTGCAACAGCAGAAC---
AACCTG---CTGAGA---GCTATTGAGGCGCAACAACACCTGTTGCAACTCACAGTCTGGGGCATT
>MG196952
TTCTACTGCAATACAACACCACTGTTTAATACATTACTGTTCAATGAAACAAGTGGT----------------
----

GATAATAAGACTATTACACTCCAATGCAGAATAAAACAAATTATAAACATGTGGCAGCAAGTAGGCAAAGCAA
TGTATGCCCCTCCCATCAGGGGACAAATTAACTGTTCATCAACT---
ATCACAGGGCTGCTATTAACAAGAGATGGTGGTAACCCAAACGCCAGC-------------------------
----

AACAGCAACGAGACCTTCAGACCTGCAGGAGGAAATATGAGGGACAATTGGAGAAGTGAACTATATAAATATA
AAGTAGTAAAAATTGAACCATTA---GGGATAGCACCCACCAAGGCAAAGAGAAGAGTGGTG-----------
----------------------------CAAAGAGAAAAAAGAGCA---GTGGGAGTAATCGGAGCT------
---ATGTTCCTTGGGTTCTTGGGAACA---GCAGGAAGCACTATG---GGCGCAGCGTCA---
ATAGCGCTGACGGTACAG---ACCAGACAA---TTATTG---TCTGGTATAGTGCAACAGCAGAAC---
AATTTG---CTGAGA---GCTATTGAGGCGCAACAACATCTGTTGCAACTCACGGTCTGGGGCATC
>MG196979
TTCTACTGTAATACAACACCATTGTTTAATAGTAATTGGACTTGGAATAATAATACAGGGTTAAAT-------
----

AACAGTGGTACTATCACACTCCAATGTAGAATAAAACAAATTATAAACCTGTGGCAGGAAGTAGGAAAAGCAA
TGTATGCCCCTCCTATCGCAGGACAAATTAACTGTTTATCAAAT---
ATTACAGGGCTAATATTAACAAGAGATGGTGGTAACAGTAACCCA----------------------------
----

AATAATACTGAGATCTTCAGACCTGGAGGAGGAGATATGAGGGACAATTGGAAAAGTGAATTATATAAATATA
AAGTAGTAAAAATTGAACCATTA---GGAGTAGCACCCACTAAGGCAAAGAGAAGAGTGGTG-----------
-----------------------------CAGAGAGAAAAAAGAGCA---GTGGGA---ATAGGAGCT------
---GTGTTCCTTGGGTTCTTGGGAGCA---GCAGGAAGCACTATG---GGCGCAGCGTCA---
ATAACGCTGACGGCACAG---GCTAGACAA---TTATTG---TCTGGTATAGTGCAACAGCAGAGT---
AATCTG---CTAAGG---GCTATCGAGGCGCAACAACATCTGTTGCAACTCACAGTCTGGGGCATC
>MG197083
TTCTACTGTAATACAACACCCCTGTTTAATAGTAGCTGGAGTAATAGTAATAGTACCAATGCAACAAGTGGTC
CTGAA---

AAAGATGACACAATCATACTCCACTGCAGAATAAAGCAAATTATAAACATGTGGCAAAAAGTAGGGAAAGCAA
TGTATGCCCCTCCCATTAGAGGACAAATTAGATGCTCATCAAAC---
ATCACAGGTCTGCTATTAACAAGAGACGGTGGTAACACAACTGGG----------------------------
----

ACCGAGACCTTCAGACCTGGAGGAGGAGATATGAGGAACAATTGGAGAAGTGAATTATATAAATATAAAGTAG
TAAAAATTGAACCATTA---GGAGTAGCACCCACCAAGGCAAAGAGAAGAGTGGTG-----------------
-----------------------CAGAGAGAAAAAAGAGCA---CTAGGA---TTCGGAGCA---------
ATGTTCCTTGGGTTCTTGGCAGCA---GCAGGAAGCACTATG---GGCGCAGCGTCA---
ATGACGCTGACGGTACAG---GCCAGGCAA---TTGTTG---TCTGGTATAGTGCAACAGCAGAAC---
AATCTG---CTGAGG---GCTATTGAGGCGCAACAACATCTGTTGCAACTCACAGTCTGGGGCATC
>MG197164
TTTTACTGTAATACAACACAACTGTTTAATAGTACTTGGCCTTTAAATAGTAGTACTTGGTGGAATAATGATA
CTGTG---

AATAATGGAAGCATCACACTCCCATGCAGAATAAAACAAATTATAAACAGGTGGCAGGAAGTAGGAAAAGCAA
TGTATGCCCCTCCCATCAGGGGACAAATTAAATGTACATCAAAT---
ATTACAGGGATACTACTAACAAGAGATGGTGGTGCTAACAATACA----------------------------
----

CAGAACGAGACCTTCAGACCTACAGGAGGAAATATGAAGGACAATTGGAGAAGTGAATTATATAAATATAAAG
TAGTAGAAATTGAGCCATTA---GGAATAGCACCCACCAGGGCAAAAAGAAGAGTGGTG--------------
--------------------------CAGAGAGAAAAAAGAGCA---GTGGGA---CTGGGAGCT---------
GTCTTCCTTGGGTTCTTGGGAGCA---GCAGGCAGCACTATG---GGCGCAGCGTCA---
ATAACGCTGACGGCACAA---GCCAGACAA---TTAATG---TCTGGTATAGTGCAACAGCAGAAC---
AATTTG---CTGAGG---GCTATTGAGGCGCAACAGCATCTGTTGCAACTCACAGTCTGGGGCATC
>MG197187
TTCTACTGTAATACATCACAACTGTTTAATAGTACTTGGGGTTCAACACAGTTGTCTAATAGTAGTTGGGATA
AGACT---
--------------------AAA---
GGACCAAATGACACACTCACACTCCTATGCAGAATAAAACAAGTCGTCAACATGTGGCAGGGAGTAGGAAAAG
CAATGTATGCTAATCCCATCAAAGGACAAATTCTATGTAGATCAAAT---
ATTACAGGGATAATATTAACAAGAGATGGTGGTGAGAGTAATAACGGAAGTGAG-------------------
----

AACAACGAGACCTTCAGACCTGGAGGAGGAGATATGAGGGATAATTGGAGAAGTGAATTATATAAATATAAAG
TAGTAAAAATTGAGCCATTA---GGAGTAGCACCCACCAAGGCAAAGAGAAGAGTGGTG--------------
--------------------------CAGAGAGAAAAAAGAGCA---GTGGGAACACTAGGAGCT---------
ATGTTCCTTGGGTTCTTGGGAGCA---GCAGGAAGCACTATG---GGCGCAGCGTCA---
ATGACGCTGACGGTACAG---GCCAGACTA---TTATTG---TCTGGTATAGTGCAGCAGCAAAAC---
AATTTG---CTGAGG---GCTATTGAGGCGCAACAGCATATGTTGCAACTCACAGTCTGGGGCATC
>KT124785
TTCTATTGTAATACAACAAAACTGTTTAATAGTACTTGGAATGAAACTGAAATAGTGAATGGCACTGGA----
----

ACAAATGAAACAATCACACTCCCATGTAGAATAAAACAAATTATAAACAGATGGCAGGAAGTAGGAAAAGCAA
TATATGCCCCTCCCATTGCGGGGCCAATTAACTGTTCATCAAAT---
ATTACAGGACTGTTATTAACAAGGGATGGTGGTAATAATAAAACC----------------------------
----

ACTGAGACCTTCAGACCTGGAGGAGGAAACATGAAGGACAATTGGAGAAGTGAATTATATAAATACAAAGTAG
TAAAAATTGAACCATTA---GGAATAGCACCCACCAGGGCAAAGAGAAGGGTGGTG-----------------
----------------------CAGAAAGAAAAAAGAGCA---GTGGGA---CTAGGAGCT---------
ATGTTCCTTGGGTTCTTGGGAGCA---GCAGGAAGCACTATG---GGCGCAGCGTCG---
ATAACGCTGACGGTACAG---GCCAGACAA---TTATTG---TCTGGTATTGTGCAGCAGCAGAAC---
AATCTG---CTGAGG---GCTATTGAGGCGCAACAGCATCTGTTGCAACTCACGGTCTGGGGCATC
>KT124790
TTCTATTGTAATACAACACAACTGTTTAATAGTATTTGGAATGGGACAAATGTTACTGGAGAG----------
----

TTAAATGGTACAATTACACTCCCATGTAGAATAAAACAAATTATAAACATGTGGCAGGAAGTAGGAAAAGCAA
TGTATGCCCCTCCCATCAAAGGACCAATTACGTGCTTATCAAAT---
ATTACAGGGATACTGTTAGTAAGAGATGGAGGTAATAATAATAAT----------------------------
----

ACTGCGAACGAGACCTTCAGACCTGGAGGGGGAGATATGAGGGACAATTGGAGAAGTGAATTATATAAATATA
AGGTAGTAAAAATTGAACCATTA---GGAATAGCACCCACCAAGGCAAAGCGAAGGGTGGTG-----------
----------------------------CAGAGAGAAAAAAGAGCA---GTGGGA---ATAGGAGCT------
---GTGTTCCTTGGGTTCTTATCAGCA---GCAGGAAGCACTATG---GGCGCAGCGTCA---
GTGACGCTGACGGTACAG---GCCAGGCTA---TTATTG---TCTGGTATAGTGCAACAGCAAAAC---
AATTTG---CTGAGG---GCTATTGAGGCGCAACAGCATCTGTTGCAACTCACAGTCTGGGGCATC
>KM355085
TTCTACTGTAATACAACACAACTGTTTAATAGTACTTGGGAAAATGGTACT----------------------
----
-------TCAACTAATACT------
ACAGGGAATGAAACTATCACACTCCCATGCAGAATAAAGCAAATTATAAACATGTGGCAGGAAGTAGGGAAAG
CAATGTATGCCCCTCCCATCAGAGGACAAATTAGATGTTCATCAAAT---
ATTACAGGGTTGCTATTAACAAGAGATGGGGGG--

-
AACAACTCCAGCAATAATGACACCTTCAGACCTGGAGGAGGGAATATGAAGGACAATTGGAGAAGTGAATTAT
ACAAATATAAAGTAGTAAGCATAGAACCAATA---GGAGTGGCACCCACCAAGGCAAGGAGAAGAGTGGTG--
-------------------------------------CAGAGAGAAAAAAGAGCA---
GTGGGAACAATAGGAGCT---------ATGTTCCTTGGGTTCTTGGGAGCA---GCAGGAAGCACTATG---
GGCGCAGCGTCA---GTGACGCTGACGGTACAG---GCCAGACAA---TTATTG---
TCTGGTATAGTGCAACAGCAGAAC---AATCTG---CTGAGA---
GCTATTGATGTGCAACAGCATATGTTGCAGCTCACAGTCTGGGGCATC
>KX587079
TTTTACTGTAATTCATCAATATTATTTAATAGTACTTGGTATACTAATAGCACTTGGAATAGTACTTGGAATG
AAACTGGA---

AATAAAAATGACACACTCACACTCCAGTGCAGAATAAAACAAATTATAAACATGTGGCAGGGAGTAGGAAAAG
CAATATATGCCCCTCCCATCCAAGGACAAATTAATTGCTCATCAAAT---
ATTACAGGACTACTATTAGTAAGAGATGGTGGTAATAGTAATGACACA-------------------------
----

AAAAACAATGAGACTTTCAGACCTCTAGGAGGAAATATGATGGACAATTGGAGAAGTGAATTATATAAATACA
AAGTAGTACAAATTGAACCATTA---GGAGTAGCACCCACCAAGGCAAAAAGGAGAGTGGTG-----------
----------------------------CAGAGAGAAAAAAGAGCA---CTAGGAGGAATGGGAGCT------
---ATGTTCCTTGGGTTCTTAAGTGCA---GCAGGAAGCACTATG---GGCGCAGCGTCA---
CTAACGCTGACGGTACAG---GCCAGACAA---TTGTTG---TCTGGTATAGTGCAACAGCAGAGC---
AATCTG---CTGAGG---GCTATTGAGGTGCAACAGCATCTGCTGCAACTCACAGTCTGGGGCATC
>KU901792

TTCTACTGTAATACAACAAAGCTGTTTAATAGTACCTGGGATAGTAATAGTACCTGGAATAGTAATAGTACCA
CAGGGGTA---

AATAGCACTGTTATCACACTGCCATGCAGAATAAAACAAATTATAAACATGTGGCAGGAAGTAGGAAAAGCAA
TGTATGCCCCTCCCATCAGAGGAGAAATTAAATGTACATCAAAT---
ATTACAGGGCTGTTACTGGTAAGAGATGGTGGTAATAACAGCAGC----------------------------
----

CAAAATGAGACCTTCAGACCTGAAGGAGGAGATATGAGGGACAATTGGAGAAGTGAATTATATAAATATAAAG
TAGTAAAAATTGAACCATTA---GGAGTAGCACCCACCAAGGCAAAGAGAAGGGTGGTG--------------
-------------------------CAGAGAGAAAAAAGAGCA---GCA------TTCGGAGCT---------
ATGTTCCTTGGGTTCTTGGGAGCA---GCAGGAAGCACTATG---GGCGCAGCGTCA---
ATAACGCTGACGGTACAA---GCCAGACAA---TTATTG---TCTGGTATAGTGCAACAGCAGAAC---
AATTTG---CTGAAA---GCTATTGATGCGCAACAACATATGTTGCAACTCACAGTCTGGGGCATC
>KU901816
TTCTACTGTAATACAACAAAGCTGTTTAATAGTACTTGGTATACTAATAGTACTGGAAATGGTACTGAAGGG-
----

TCAAATGACACAATCACACTCCCATGCCGAATAAGACAAATTATAAACAAATGGCAGAGAGTAGGAAAAGCAA
TGTATGCCCCTCCCATTAAAGGACACATTAACTGTACATCAAAT---
ATTACAGGGCTGCTATTAACAAGAGATGGTGGTGATAACAGCAGT----------------------------
----

ACGAACGAGACCTTCAGACCTGGAGGAGGAGATATGAGGGACAATTGGAGAAGTGAATTATATAAATATAAAG
TAGTAAGAATTGAGCCAGTA---GGAATAGCTCCCACCAAGGCAAGAAGAAGAGTGGTA--------------
-------------------------CAGAGAGAAAAAAGAGCA---GCC------TTAGGAGCT---------
GTGTTCCTTGGGTTCTTGGGAGCA---GCAGGAAGCACTATG---GGCGCAGCGTCA---
CTAACGCTGACGGTACAA---GCCAGACTA---TTATTG---TCTGGTATAGTGCAACAGCAAAGC---
AATTTG---CTGAAG---GCTATTGAGGCGCAACAGCGTCTGTTGCAACTCACAGTCTGGGGCATC
>KM986883
TTCTACTGTAACTCATCAAAACTGTTTAATAGTACTTGGAATAGTACTGGGAGTGAAAATAATAACACTGAAG
GG---

TCAACTGGCACTATCACACTCCAATGCAGGATAAAACAAATTATAAATATGTGGCAGGAAGTAGGAAAAGCAA
TGTATGCCCCTCCCATCAGAGGACAAATTAGCTGTAATTCAACT---
ATTACAGGGCTGCTACTAACAAGAGATGGGGGTGATACGGACAAT----------------------------
----

ACCTCCGAGGTCTTCAGACCTGGAGGAGGAGATATGAGGGACAATTGGAGAAATGAATTATACAAATATAAAG
TAGTAAAAGTAGAACCATTA---GGAATAGCACCCACTAAAGCAAAAAGAAGAGTGGTG--------------
-------------------------CAGAGAGAAAAAAGAGCA---GTAGGA---CTAGGAGCT---------
GTGTTCCTTGGGTTTTTGTCAGCA---GCAGGAAGCACTATG---GGCGCAGCGTCG---
GTGACGCTGACGGTACAG---GCCAGACAA---TTACTG---TCTGGTATAGTGCAACAGCAGAAC---
AATCTG---CTGAGG---GCTGTTGAGGCGCAACAACACCTGTTGCAACTCACAGTCTGGGGCATC
>KU901976
TTCTACTGTAGTACAACACAACTGTTTGATAGTACTTGGGCAACTAATAGTACTGGGAATATT----------
----

ACTAACGGAACTATCACACTCCCATGTAGAATAAAACAAATTATAAACATGTGGCAGGGAGTAGGAAAAGCAA
TGTATGCCCCTCCCATTAAAGGACAAATTAGATGTTCATCAAAT---
ATTACAGGGCTGCTATTAACAAGGGATGGTGGTAATAATGACACG----------------------------
----

AACGAGAACGAGACCTTCAGACCTGGAGGAGGAGATATGAGGGACAACTGGAGAAGTGAATTATATAAATATA
AAGTAGTAAAAATTGAACCATTA---GGAGTAGCACCCACCAAGGCAAAGAGAAGAGTGGTG-----------
----------------------------CAGAGAGAAAAAAGAGCA---GTGGGA---ATAGGAGCT------
---TTGTTCCTTGGGTTCTTGGGAGCA---GCAGGAAGCACTATG---GGCGCAGCGTCA---
ATGACGCTGACGGTACAA---GCCAGGCAA---TTATTG---TCTGGTATAGTGCAACAGCAGAAC---
AACTTG---CTGAGA---GCTATTGAGGCGCAACAGCATCTGTTGCAACTCACAGTCTGGGGCATC
>KY766175

TTCTACTGTAATACAATACAGCTGTTTAATAGTACTTGGGATAATAATACTTGGAATAGGTATAATAGTACTA
CTGGG--

TCAAATGAAATTATCAGGCTCCCATGCAGAATAAGACAAATTGTAAACAGGTGGCAGGAAGTAGGAAAAGCAA
TGTATGCCCCTCCCATCGAAGGACAAATTAGATGTTCATCAAAT---
ATTACAGGGCTGATATTAACAAGAGATGGTGGTGGTAATGGCACG----------------------------
----

AAAGGAAACGAGACCTTCAGACCTGGAGGAGGAGATATGAAGGACAATTGGAGAAGTGAATTATATAAATATA
AAGTAGTAAAAATTGAGCCATTA---GGAATAGCACCCACCGCGGCAAAGAGAAGAGTGGTG-----------
----------------------------CAGAGAGAAAAAAGAGCA---ATAGGAATGCTAGGAGCT------
---ATGTTCCTTGGGTTCTTGGGAGCA---GCAGGAAGCACTATG---GGCGCAGCGTCA---
ATGACGCTGACGGTACAG---GCCAGACAA---TTATTG---TCTGGTATAGTGCAACAGCAGAAC---
AATTTG---CTGAGG---GCTATTGAGGCGCAACAGCATCTGTTGCAACTCACAGTCTGGGGCATT
>KY778395
TTCTACTGTAATACAACACAACTGTTTAATGGTACTTGGGAGAATAGTACTAGGAATGATAATAACACTGCA-
----

AAAAATGGCACAATCACACTCCCATGCAGAATAAAACAAATTATAAACATGTGGCAAAAAGTAGGAAAAGCAA
TATATGCCCCTCCCATCAGAGGAGAAATTAGGTGTTCATCTAAC---
ATTACAGGGCTGCTACTAACTAGAGATGGTGGTAATGGGAACAGCACC-------------------------
----

AAGGACAACGAGACCTTCAGACCTGGGGGAGGAGATATGAGGGACAATTGGAGAAGTGAATTATATAAATACA
AAGTAGTAAAAATTGAACCATTA---GGAATAGCACCCACTAAGGCAAAGAGAAGAGTGGTG-----------
----------------------------CAGAGAGAAAAAAGAGCA---GTGGGA---CTAGGAGCT------
---GTGTTCCTTGGGTTCTTGGGAGCA---GCAGGAAGCACTATG---GGCGCAGCGTCA---
ATGACGCTGACGGTACAG---GCCAGACTA---TTATTG---TCTGGTATAGTGCAACAGCAAAAC---
AATCTG---CTGAGA---GCTATTGAGGCGCAACAGCATCTGTTGCAACTCACAGTCTGGGGCATC
>MH897918
TTCTACTGTGATTCATCACAACTGTTTAATAGTACTTGGAGTAATGATACTAATACTGGGAAT----------
----

GATAATGGGAATATCACACTCCTATGCAGAATAAAACAATTTATAAACATGTGGCAGCAAGTAGGAAAAGCGA
TGTATGCCCCTCCCATCAGAGGACAAATTAAATGTTCATCAAAT---
ATTACAGGGCTACTGTTAACAAGAGATGGTGGTAATAATAACAATACTGGA----------------------
----

AACCGTACTGAGACCTTCAGACCTGGAGGAGGAGATATGAGGGACAATTGGAGAAGTGAATTATATAAATATA
AAGTAGTAAGAATTGAACCATTA---GGATTAGCACCCACCAAGGCAAAGAGAAGAGTGGTG-----------
----------------------------CAGAGAGAAAAAAGAGCA---GTGGGA---ATAGGAGCT------
---ATGTTCCTTGGGTTCTTGGGAGCA---GCAGGAAGCACTATG---GGCGCAGCGTCA---
CTGACGCTGACGGTACAG---GCCAGACAA---TTATTG---TCTGGTATAGTGCAACAGCAGAAC---
AATTTG---CTGAGG---GCTATTGAGGCGCAACAGCATTTGTTGCAACTCACAGTCTGGGGCATT
>MG196711
TTCTACTGTAATACAACACAGTTGTTTAATAGTACTTGGCAGCCGGTTAATAGTACTACTTGGAGTAATTTCG
GTCCGGGG---

CCAAATGAAAATATCACACTCCCATGTAGAATAAAACAAATTATAAACAGATGGCAGGAAGTAGGAAAATCAA
TGTATGCCCCTCCCATTAGAGGAGTCATTAGCTGTTCATCAAAT---
ATTACAGGGTTGATATTAACAAGAGATGGTGGTGGGACCAATAAT----------------------------
----

ACTAACGAAACCTTCAGACCTGGGGGAGGAGATATGAGGGACAATTGGAGAAGTGAATTGTATAAATACAAAG
TAGTAAAAATTGAGCCATTA---GGAATAGCACCCACCAGGGCAAAGAGAAGAGTGGTG--------------
-------------------------CAGAGAGAAAAAAGAGCA---GTAACA---ATAGGAGCT---------
ATGTTCCTTGGGTTCTTGGGAGCA---GCAGGAAGCACTATG---GGCGCAGCATCA---
CTGACGCTGACGGTACAG---GCCAGACTA---TTATTG---TCTGGTATAGTGCAACAGCAAAAC---
AATTTG---CTGAGA---GCTATTGAGGCGCAACAGCATCTGTTGCAGCTCACAGTCTGGGGCATC
>MG197127

TTCTACTGTAATACAACACCACTGTTTAATAGTACTTGGAATAAAAACAATAGTACTTGGACTTGGAATAGCA
CTAGAAAA--
-------------AACTTT------
ACAGAAAATGGCAATATCACACTACAGTGTAGAATCAGACAAATTGTAAACAGGTGGCAAGAAGTAGGAAAAG
CAATGTATGCTCCTCCCATCAGGGGACTAATTAGATGTTCATCAAAT---
ATTACTGGGCTATTGTTAACAAGAGATGGTGGTAATAATAAAACT-----------------------------
----

AATGGGAGTGAGATCTTCAGACCTGGAGGAGGAAATATGAGGGACAATTGGAGAAGTGAATTATATAAATATA
AAGTGGTAAAAATTGAACCATTA---GGAGTAGCACCCACCAGGGCAAAGAGAAGAGTGGTG-----------
----------------------------CAGAGAGAAAAAAGAGCA---GTAGGA---TTGGGAGCT------
---TTGTTCCTCGGGTTCTTGGGAGCA---GCAGGAAGCACTATG---GGCGCAGCATCA---
CTAACGCTGACGGTACAG---GCCAGACAA---TTATTG---TCTGGTATAGTGCAACAGCAAAAC---
AATTTG---CTGAGG---GCTATTGAGGCGCAACAACATATGTTGCAACTCACAGTCTGGGGCATC
>KT124796
TTCTACTGTAATTTAACAAAACTGTTTAATAGTACTTGGAATGAAACAGGGTCAAATAACACT----------
----

TTAAATGGCACAGTCATACTCCCATGCAGAATAAAACAAATTGTAAATATGTGGCAGGAAGTAGGAAAAGCAA
TGTATGCCCCTCCCATCGCAGGGCTAATTAACTGTGTATCAAAT---
ATTACAGGGCTGATATTAACAAGAGATGGTGGT---

ACTAATGACACTGAGACCTTCAGACCTGGAGGAGGGGATATGAGGGACAATTGGAGAAGTGAATTATATAAAT
ATAAAGTAGTAAAAGTTGAACCATTA---GGAGTAGCACCCACCAGGGCAAAGAGGAGAACGGTG--------
--------------------------------CAAAGAGAAAAAAGAGCA---GTGGGA---CTAGGAGCT---
------GTGTTCCTTGGGTTCTTGGGAGCA---GCAGGAAGCACTATG---GGCGCAGCATCA---
ATGACGCTGACGGTACAG---GCCAGACAA---TTATTG---TCTGGTATAGTGCAACAGCAGAAC---
AATCTG---CTGAGG---GCTATTGAGGCGCAACAGCACCTGTTGCAACTCACAGTCTGGGGCATC
>KX505648
TTCTACTGTAATACAGCACAACTGTTTAATAGTACTTGGAATGGTACTGAAGGGTCAAATAACATTGAAGGAA
ATAACAAT--

GAAGAAAATGACACAATCACACTCCAATGCAGAATAAAACAAATTATAAACATGTGGCAGGAAGTAGGAAAAG
CAATGTATGCCCCTCCCATCAATGGGCAAATTAGATGTACATCAAAT---
ATTACAGGGCTGCTATTAGTAAGAGATGGAGGGGTAAAAAATGGTACTGAGGAT-------------------
----

GAAAACAAGACCGAGATTTTCAGACCTGGAGGAGGAGATATGAGGGACAATTGGAGAAGTGAATTATATAAAT
ATAAAGTAGTAAAAATTGAACCGTTA---GGAATAGCACCCACCAGGGCAAAGAGAAGAGTGGTG--------
--------------------------------CAAAGAGAGAAAAGAGCA---GTGGGATTAGTAGGAGCT---
------ATGTTCCTTGGGTTCTTGGGAGCA---GCAGGAAGCACTATG---GGCGCAGCGGCA---
ATAACGCTGACGGTACAG---GTCAGACAA---ATGTTG---TCTGGTATAGTGCAACAGCAGAAC---
AATCTG---CTGAGG---GCTATTGAGGCGCAACAGCATCTGTTGCAACTCACAGTCTGGGGCATC
>KX505652
TTCTACTGTAATACAACACAACTGTTTAATAGTACTTGGAATGATACCGAAGGGTTAAGTAACACTGAA----
----

GGAGATGACACACTCACACTCCCATGCAGAATAAAACAAATTGTAAACATGTGGCAGGAAGTAGGAAAAGCAA
TGTATGCCCCTCCCATCAAAGGACAAATTAATTGTACATCAAAT---
ATTACAGGGCTGCTATTAACAAGAGATGGTGGGAATAATGAGAGC-----------------------------
----

AGCACCACCGAGACCTTCAGACCTGGAGGAGGAGATATGAGGGACAATTGGAGAAATGAATTATATAAATATA
AAGTAGTAAAGGTTGAACCATTA---GGAGTAGCACCCACCAAGGCAAGGAGAAGAGTGGTG-----------
----------------------------CAGAGAGAAAAAAGAGCA---GTGGGA---ATAGGAGCT------
---ATGTTCCTTGGGTTCTTGGGAGCA---GCCGGAAGCACTATG---GGCGCAGCGTCA---
ATGACGCTGACGGTACAG---GCCAGACAA---TTATTG---TCTGGTATAGTGCAACAGCAAAAC---
AATTTG---CTGAGG---GCTATTGAGGCGCAACAGCATATGTTGCAACTCACAGTCTGGGGCATC
>KX505686

TTCTACTGTAATTCAACACAACTGTTTAATAAGACATGGAATGATACTGAACTATCTGATAAAACTTGGAAAG
TTGATAAA---
-------------------AAT---
AATGCTAGTGACATCATCACACTCCCATGTAAAATAAAACAAATCATAAACCTGTGGCAGGAAGTAGGAAAAG
CAATGTATGCCCCTCCTATCAGAGGATCAATTACATGTTCATCAAAT---
ATTACAGGGCTGGTATTAACAAGAGATGGTGGTACGAACAATACC----------------------------
----

ATGAATGAAACCTTCAGACCTGGAGGAGGAGATATGAGGGATAATTGGAGAAGTGAATTATATAAATATAAAG
TAGTAAGAATTGAACCATTA---GGAGTAGCACCCACCAAGGCAAGGAGAAGAGTGGTG--------------
-------------------------CAGAGAGAAAAAAGAGCA---GTGGGA---CTAGGAGCT---------
CTGTTCTTTGGGTTCTTATCAGCA---GCAGGAACCACTATG---GGCGCAGCGTCA---
ATGACGTTGACGGTACAG---GCCAGACAG---TTACTG---TCTGGTATAGTGCAACAGCAGAAC---
AATATG---CTGAGA---GCTATTGAGGCGCAACAGCATCTGTTGCAACTCACAGTCTGGGGCATT
>KX505707
TTTTACTGTAATACATCAAAACTGTTTAATAGTATTTGGAATGATAATAGTACTTGGAATAATACTAGAGAGT
TTAATAAC---

ACTGACAGAAATATCACACTCCCATGCAGAATAAAACAAATTGTAAACAGGTGGCAGGAAGTAGGAAAAGCAA
TGTATGCCCCTCCCATCAGTGGAAAAATTAGTTGTTCATCAAAT---
ATTACAGGACTGCTATTAACAAGAGATGGTGGTAAGGGTAACAAGACAAAT----------------------
----

AACACCACCGAGATCTTCAGACCAGGGGGAGGAAATATGAGGGACAATTGGAGAAGTGAATTATACAAATATA
AAGTAGTAAAAATTGAACCATTA---GGAATAGCACCCACCAAGGCAAAGAGAAGAGTGGTG-----------
----------------------------CAGAGAGAAAAAAGAGCA---GTGGGA---ATAGGAGCT------
---ATGTTCCTTGGGTTCTTGGGAGCA---GCAGGAAGCACTATG---GGCGCAGCGTCA---
GTGACGCTGACGGTACAG---GCCAGACAA---TTATTG---TCTGGTATAGTGCAACAGCAGAAC---
AATCTG---CTGAAG---GCTATTGAGGCGCAACAGCGTATGTTGCAACTCACAGTCTGGGGCATT
>KY057587
TTCTACTGTAATACAACACCACTGTTTAATAGTATTTGGAATACAAGTGATACTTGGAATAGTACTAATGAAG
AATCAAAT---
-------------AACACT------
GCAGGAAATGACACAATCATACTTCGATGCAGAATAAAACAAATTATAAACATGTGGCAAGAAGTAGGAAAAG
CAATGTATGCCCCTCCCATCAGAGGAAATATTACATGTTTATCAAAT---
ATTACAGGACTGCTGTTAACAAGAGATGGAGGCAATAGCAGTAAT----------------------------
----

AATGGGACTGAGATCTTCAGACCTGGAGGAGGGGATATGAGGGACAATTGGAGAAGTGAATTATATAAATATA
AAGTAGTAAAAATTGAACCATTA---GGAGTAGCACCCACTAAGGCAAAGAGAAGAGTGGTG-----------
----------------------------CAGAGAGAAAAAAGAGCA---GTGGGG---TTAGGAGCT------
---ATGTTCCTTGGGTTCTTGGGAGCA---GCAGGAAGCACTATG---GGCGCAGCGTCA---
CTGACGCTGACGGTACAG---GCCAGACAA---TTATTG---TCTGGTATAGTGCAACAGCAGAAT---
AATTTG---CTGAGA---GCTATTGAGGCGCAACAGCATCTGTTGCAACTCACAGTCTGGGGCATC
>KY748513
TTCTACTGTAACACAACAAGGCTGTTTAATAGTACTTGGAATGGTACGGATACAATAAATAACACTGCA----
----

GAAAACAGTACACTTACACTCCCATGCAGAATAAAACAAATTATAAATAGGTGGCAGGAAGTAGGAAAAGCAA
TATATGCCCCTCCCATCAGGGGACAAATTAGATGTAAATCAAAT---
ATTACAGGGTTGCTATTAACAAGAGATGGTGGTCCCGAAGGGAATAAGACC----------------------
----

ACCTTCAGACCTGGAGGAGGAGATATGAGGGACAATTGGAGAAGTGAATTATATAAATATAAAGTAGTAAAAA
TTGAGCCATTA---GGGATAGCACCCACCAAGGCAAAGAGAAGAGTGGTG-----------------------
----------------CAGAGAGAAAAAAGAGCA---GTGGGC---ATAGGAGCT---------
ATGTTCCTTGGGTTCTTGGGAGCA---GCAGGAAGCACTATG---GGCGCAGCGTCA---
ATGACGCTGACGGTACAG---GCCAGACAA---TTATTG---TCTGGTATAGTGCAACAGCAGAAC---
AATTTG---CTGAGG---GCTATTGAGGCGCAACAGCATCTGTTGCAACTCACAGTCTGGGGCATC
>KY748576

TTCTACTGTGATTCAACAAAACTGTTTAATAGCACTTGGAATGATACTTGGGATGTGAATATCACTGAAAGG-
----

ACAAATGACACTATCATACTCCCATGCAGAATAAAACAAATTGTAAACATGTGGCAGGAAGTAGGAAAAGCAA
TGTATGCCCCTCCCATCAAAGGACCAATTAGCTGTTCATCAAAC---
ATTACAGGACTACTATTAACAAGAGATGGTGGTAGTGGCAACGAGACCACT----------------------
----

AACACCACCGAGATCTTCAGACCTGGAGGAGGAGATATGAGGGACAAGTGGAGAAGTGAATTATACAAATATA
AAGTAGTAAAAATTGAACCATTA---GGAGTAGCACCCACCAAGGCAAAGAGAAGAGTGGTG----------
----------------------------CAGAGAGAAAAAAGAGCA---GTGGGA---ATAGGAGCT------
---TTGTTCCTTGGGTTCTTGGGAACA---GCAGGAAGCACTATG---GGCGCAGCGGCA---
GTGACGCTGACGGTACAG---GCCAGACAA---TTATTG---TCTGGTATAGTGCAACAGCAGAAC---
AATCTG---CTGAGG---GCTATTGAGGCGCAACAACATCTGTTGCAACTCACAGTCTGGGGCATC
>KT284371
TTCTACTGTAATACAACAAAACTGTTTAATAGTACATGGTATGTAAATAATACTGAAGAGTTAAATAAG----
----

GAAGGAGACACGATCACACTCCCATGCAGAATAAAACAAATTGTAAACATGTGGCAGGAAGTAGGAAAAGCAA
TGTATGCCCCTCCCATCAGAGGACAAATTAGCTGCTCATCAAAT---
ATTACAGGGCTACTATTAACGAGAGATGGTGGTACCGACAACAGCACG-------------------------
----

AACAATACCGAGACCTTCAGACCTGGAGGGGGAGATATGAGGGACAATTGGAGAAGTGAATTATATAAATATA
AAGTAGTAAAAATTGAACCATTA---GGAGTAGCACCCACCAAGGCAAAGAGAGTAGTGGTG----------
----------------------------CGAAGAGAAAAAAGAGCA---GCGGGA---ATAGGAGCT------
---TTGTTCCTTGGGTTCTTGGGAGCA---GCAGGAGGCACTATG---GGCGCAGCGTCA---
CTGACGCTGACGGTACAG---GCCAGACAA---TTATTG---TCTAGTATAGTGCAACAGCAGAAT---
AACTTG---CTGAGA---GCTATAGAGGCGCAACAGCATCTGTTGCAGCTCACAGTCTGGGGCATC
>KR182173
TTCTACTGTAATACAACAAAACTGTTTAATAGTACTTGGAATAAGACGGGTGAGATTTGGAATAGTACTATGG
AGAATAATAAT--

GACACTGAACCAATCACACTCCCATGCAGAATAAAACAAATTGTAAACATGTGGCAGGAAGTAGGAAAAGCAA
TGTATGCCCCTCCCATCAGTGGACCAATTAGATGTGTATCAAAT---
ATCACAGGGCTGCTATTAACAAGAGATGGTGGAAATGATGGTAATACC-------------------------
----

AACACCAGGAATGAGACTTTCAGACCTGGAGGAGGAGATATGAGGGACAATTGGAGAAGTGAATTATATAAAT
ATAAAGTAGTAAGAATTGAACCAATA---GGACTAGCACCTACCAGGGCAAAGAGAAGAGTGGTG--------
-------------------------------CAGAGAGAAAAAAGAGCA---ATAGGA---ATAGGAGCT---
------GTGTTCCTTGGGTTCTTGGGAGCA---GCAGGAAGCACTATG---GGCGCAGCGTCA---
CTGACGCTGACGGTACAA---GCCAGACAA---TTATTG---TCTGGTATAGTGCAACAGCAGAGC---
AACTTG---CTGAGG---GCTATTGAGGCGCAACAACATCTGTTGCAACTCACAGTCTGGGGCATC
>KR182183
TTCTACTGTAATACAACACAGCTGTTTGATAGTATTTGGGAGCGTAATAGAACTTTGAATGATACAGAAAATA
CA---

AATGACCGTAACATCACACTCCCATGCAGAATAAAACAAATTATAAACAGATGGCAGGGAGTAGGAAAAGCAA
TGTATGCCCCTCCCATAAAAGGAGAAATTAGATGCACATCAAAT---
ATTACAGGGCTAATATTAACAAGAGATGGTGGTAGTGATACTAACAGG-------------------------
----
GATAATAACACGACA---
GGGAATGAGACCTTCAGACCTGGAGGAGGAGATATGAGGGACAATTGGAGAAGTGAATTATATAAATATAAAG
TAGTAAAAATTGAGCCATTA---GGAGTAGCACCCACCAAGGCAAAGAGAAGAGTGGTG--------------
-------------------------CAGAGAGAAAAAAGAGCA---GTGACA---ATGGGAGCT---------
ATGCTCCTTGGGTTCTTGGGAGCA---GCAGGAAGCACTATG---GGCGCAGCATCG---
ATAACGCTGACGGTACAA---GCCAGACAA---TTATTG---TCTGGTATAGTGCAACAGCAGAGC---
AATTTG---CTGAAG---GCTATTGACGCGCAACAGCACCTGTTGCAACTCACAGTCTGGGGCATT
>KR182187

TTCTACTGTAATACAACACAGCTGTTTAATAGTACTTGGTCGCTGTTTAATAGTACTTGGTCGCATAATGAAA
CTAGG--
-------------AATGCT------
ACAAATGACCGTAACATCACACTTCCATGCAGAATAAAACAAATTATAAACAGATGGCAGGGAGTAGGAAAAG
CAATGTATGCCCCTCCCATCAAGGGAGAAATTAGATGCACATCAAAT---
ATTACAGGGCTAATATTAACAAGAGATGGTGGTAGTGATACTAACAAT-------------------------
----
GATACTAACAAGACA---
GGGAATGAGACCTTCAGACCTGGAGGAGGAGATATGAGGGACAATTGGAGAAGTGAATTATATAAATATAAAG
TAGTAAAAATTGAGCCATTA---GGGGTAGCACCCACCAAGGCAAAGAGAAGAGTGGTG--------------
-------------------------CAGAGAGAAAAAAGAGCA---GCGGGAACGATGGGAGCT---------
ATGTTCCTTGGGTTCTTGGGAGCA---GCAGGAAGCACTATG---GGCGCAGCATCG---
ATAACGCTGACGGTACAA---GCCAGACAA---TTATTG---TCTGGTATAGTGCAACAGCAGAGC---
AATTTG---CTGAAG---GCTATTGACGCGCAACAGCACCTGTTGCAACTCACAGTCTGGGGCATT
>KR182191
TTCTACTGTAATACAACACAGCTGTTTAATAGTACTTGGAATAGTACTTGGCCGCTTAATGAAACTTGGGATG
GTATCGAAGGA--

AATAACACTAACATCACACTTCCATGCAGAATAAAACAAATTATAAACAGATGGCAGGGAGTAGGAAAAGCAA
TGTATGCCCCTCCCATCAAGGGACAAATTAGCTGTTCATCAAAT---
ATTACAGGGCTAATATTAACAAGAGATGGTGGTAATAACACGAGT----------------------------
----

GGGAGTGAGACCTTCAGACCGGGAGGAGGAGATATGAGGGACAATTGGAGAAGTGAATTATATAAATATAAAG
TAGTAAAAATTGAGCCATTA---GGGGTAGCACCCACCAGGGCAAAGAGAAGAGTGGTG--------------
-------------------------CAGAGAGAAAAAAGAGCA---GTGACG---ATGGGAGCT---------
ATGCTCCTTGGGTTCTTGGGAGCA---GCAGGAAGCACTATG---GGCGCAGCGTCG---
ATAACGCTGACGGTACAG---GCCAGACTA---TTGTTG---TCTGGTATAGTGCAACAGCAGAGC---
AATTTG---CTGAAG---GCTATTGAGGCGCAACAGCACCTGTTGCAACTCACAGTCTGGGGCATC
>KR182196
TTCTACTGTAACACAACACAACTGTTTAATAGTACTTGGAATGCTAATAGTACTTGGAATGATACTAGTTCTA
ATATTGGA---

GAGTCAAATGACACAATCACACTCCCATGCAAAATAAGACAGATTGTAAATATGTGGCAGGAAGTAGGAAAAG
CAATGTATGCTCCTCCCATTGCAGGACAACTTAATTGCTCATCAAAT---
ATTACAGGAATATTACTAACAAGAGATGGTGGTGAGAGTAATACCACG-------------------------
----

AATAGTAACGAGACTTTCAGACCTGGAGGAGGAAATATGAGGGACAATTGGAGGAGTAAATTATATAAATATA
AAGTAATAAAAATTGAACCATTA---GGACTGGCACCCACCAAGGCAAAGAGAAGAGTGGTG-----------
----------------------------CAGAGAAAAGCAAGAGCA---GCAGGA---TTAGGAGCT------
---GTGATCTTTGGGTTCTTGGGAGCA---GCAGGAAGCACTATG---GGCGCAGCGTCA---
GTAGCGCTGACGGTACAG---GCCAGACAA---TTATTG---TCTGGTATAGTGCAACAGCAAAAC---
AATTTG---CTGAGG---GCTATTGAGGCGCAACAGCATATGTTGCAACTCACAGTCTGGGGCATC
>KR182341
TTCTATTGTAATACAACACAACTGTTTAATAGTACTTGGTTTAATGGTACTTGGAATAGTACTAATGACAATA
GT---

ACAAATGGCACTATCACGCTCCCATGCAAAATAAAACAAATTATAAACATGTGGCAGAAAGTAGGAAAAGCAA
TGTATGCCCCTCCCATCAGAGGAAACATTAGCTGTTCATCAAAT---
ATAACGGGGTTGCTATTAATAAGAGATGGTGGTATTAATAGTACA----------------------------
----

GAGAATAGCACAGAAACCTTCAGACCGGGAGGAGGAGATATGAGGGACAATTGGAGAAGTGAATTATATAAAT
ATAAAGTAGTACAAATTGAACCAATA---GGACTAGCACCCACAAAGGCAAAGAGAAGAGTGGTG--------
-------------------------------CAGAGAGAAAAAAGAGCA---GTGGGAACATTAGGAGCT---
------GTGTTCCTTGGGTTCTTGGGAGCA---GCAGGAAGCACTATG---GGCGCAGCGTCA---
ATAACGCTGACGGTACAG---GCCAGACAA---TTATTG---TCTGGTATAGTGCAACAGCAGAAT---
AATCTG---CTGAAG---GCTATTGATGCGCAACAACATCTGTTGCAGCTCACAGTCTGGGGCATC
>KR182409

TTCTACTGTGAGTCAACACCACTGTTTAATAGCACTTGGAATAGTAATGACTTGACTCCTACTATAGAGTCAA
ATAGC--

AATGGAGAAAACATTACACTAACATGCAGAATAAAACAAATTATAAACATGTGGCAGGAAGTAGGAAAAGCAA
TGTATGCCCCTCCCATCAGTGGACCAATTGAATGTACATCAAAT---
ATTACAGGGCTACTATTAACAAGAGATGGTGGTGATAACAATAATATG-------------------------
----

AGCAGCGAGACTTTCAGACCTGGAGGAGGAAATATGAAAGATAATTGGAGAAATGAATTATATAAATATAAAG
TAGTAAAAATTAAACCATTA---GGAATAGCACCCACCAAGGCGAGAAGAAGAGTGGTG--------------
-------------------------CAAAGAGAAAAAAGAGCA---GTGGGA---ATAGGAGCA---------
TTATTCCTTGGGTTCTTGGGAGCA---GCAGGAAGCACTATG---GGCGCAGCGTCA---
TTGACGCTGACGGTACAG---GCCAGACAA---TTATTG---ACTGGTATAGTGCAACAGCAGAAC---
AATCTG---CTGAGG---GCTATTGAAGCGCAACAGCATCTGTTGCAACTCACAGTCTGGGGCATC
>KR182465
TTCTACTGTAATACAACACAACTGTTTAATAGTACCTGGATCAATGGTACTAAAAAGTCAAATACC-------
----

ACTGAAGACACAATCATACTCCCATGCAGAATAAGACAAATTATAAACATGTGGCAGGAAACAGGAAAAGCAA
TGTATGCCCCTCCCATAAGAGGACAACTTAGATGTTCATCAAAT---
ATTACAGGATTACTATTAACAAGAGATGGTGGGAATGATAATAAT----------------------------
----

GAGACTGAGACCTTCAGACCTGGGGGAGGAAATATGAAAGACAATTGGAGAAGTGAACTATATAAATACAAAG
TAGTAAAAGTTAAACCATTA---GGAATAGCACCCACCAGGGCAAAGAGAAGAGTGGTG--------------
-------------------------CAGAGAGAAAAAAGAGCA---GTGGGA---ATAGGAGCT---------
GTGTTCCTTGGGTTCTTGGGAGCA---GCAGGAAGCACTATG---GGCGCAGCGTCA---
CTGACGCTGACGGTACAG---GCCAGACAA---TTATTG---TCTGGTATAGTGCAACAGCAGAAC---
AATCTG---CTGAGA---GCTATTGAGGCGCAACAGCATATGTTGCAACTCACAGTCTGGGGCATC
>MG171201
TTCTACTGTAATTCAACAAAACTGTTTAATAGTACTTGGAGTGTTAATAGTACTCGGAGTGGTAATAGTACAG
GGTCAAAT---
-------------------AAC---
ACTGGAAACTCAACAATCACACTCCCATGCAGAATAAAACAAGTTATAAACATGTGGCAGGCAGTAGGAAAAG
CAATGTATGCCCCTCCCATCAGCGGACAAATTAGATGTTCATCAAAT---
ATTACAGGGCTGATATTAACAAGAGATGGTGGTGATAACGCTAGC----------------------------
----

ACTACTGAGACTTTCAGACCTGGAGGAGGGGATATGAGGGACAATTGGAGAAGTGAATTATATAAATATAAAA
TAGTAAAAATTGAACCATTA---GGAGTAGCACCCACCAAGGCAAAGAGAAGAGTGGTG--------------
-------------------------CAGAGAGAAAAAAGAGCA---GTGGGA---ATAGGAGCT---------
GTGTTCCTTGGGTTCTTGGGAGCA---GCAGGAAGCACTATG---GGCGCAGCGTCA---
ATGACGCTGACGGTACAG---GCTAGACTA---TTATTG---TCTGGTATAGTGCAACAGCAGAAC---
AATTTG---CTGAGG---GCTATTGAGGCGCAACAGCATCTGTTGCAACTCACAGTCTGGGGCATC
>KY778299
TTCTACTGTAATACAACACAACTGTTTAATAGTACTTGGCAGGGTAATACTTGGAATGGT-------------
----

ACTAAAGAAAATATCACACTCCCATGCAAAATAAGACAAATTGTAAACATGTGGCAAGAAGTAGGAAAAGCAA
TGTATGCCCCTCCTATCAGAGGACAAATTACATGCTCATCAAAT---
ATTACAGGGCTGTTATTAACAAGAGATGGTGGTAATACCACAAAC----------------------------
----

AATACCGAGGTCTTCAGACCTGGAGGAGGAGATATGAAGGACAATTGGAGAAGTGAATTATACAAATATAAAG
TAATAAAAATTGAACCATTG---GGAGTAGCACCCACTAAGGCAAAAAGAAGAGTGGTG--------------
-------------------------CAGAGAGAAAAAAGAGCA---GTGGGA---TTAGGAGCT---------
GTGTTCCTTGGGTTCTTGGGAGCA---GCAGGAAGCACTATG---GGCGCAGCGTCA---
ATCACGCTGACGGTACAG---GCCAGACAA---TTATTG---TCTGGTATAGTGCAACAGCAGAAC---
AACTTG---CTGAGA---GCTATTGAGGCGCAACAGCATCTGTTGCAACTCACAGTCTGGGGCATC
>MK384347

TTCTACTGTAATACAACAAAACTGTTTAATAGTACCTGGACCTGGAATAGTACTGAAGGGTCAGGGAATGACA
CTGAGAGGAAT--

ATCACTGAGAGGAATATCACACTCCCATGCAGAATAAAACAAATTGTAAACATGTGGCAGGAAGTGGGAAAAG
CAATGTATGCCCCTCCCATCAGTGGACAAATTAAATGTTCATCAAAT---
ATTACAGGTCTGCTTTTAACAAGAGATGGTGGTGGAAAAATAAC-----------------------------
----

AGTGACAACGAGACCTTCAGACCTGGAGGAGGAGATATGAGGGACAATTGGAGAAGCGAATTATATAAATATA
AAGTAGTAAAAATTGAACCATTA---GGAGTCGCACCCACCAAGGCAAAGAGAAGAGTGGTG-----------
----------------------------CAGAGAGAGAAAAGAGCA---GTGACA---TTTGGAGCT------
---ATGTTCCTTGGGTTCTTGGGAGCA---GCAGGAAGCACTATG---GGCGCAGCGTCA---
ATAACGCTGACGGTACAG---GCCAGACAA---TTATTG---TCTGGTATAGTGCAACAGCAGAGC---
AATTTG---CTGAGA---GCTATTGAGGCGCAACAGCATCTGTTGCAACTCACGGTCTGGGGCATC
>KX505396
TTCTACTGTGATACAACAAAACTATTTAATAGTACTTGGAATGAAACTAACACTGTAGTAAACCAA-------
----

ACTACAGAAAATATCACACTCTCATGCAGAATAAAACAAATTATAAACAGGTGGCAGGAAGTAGGAAAGGCAA
TGTATGCCCCTCCCATCGAAGGACAAATTAGGTGTTCATCAAAC---
ATTACAGGGCTGTTATTAACAAGAGATGGTGGTATTAATAATGAGACT-------------------------
----

AACACGACCGAGATCTTCAGACCTGGAGGAGGAGATATGAGGGACAATTGGAGAAGTGAATTATATAAATATA
AAGTAGTAAGAATTGAACCATTA---GGAGTAGCACCCACCAAGGCAAAGAGAAGAGTGGTG-----------
----------------------------CAGAGAGAAAAAAGAGCA---GTGGGA---TTAGGAGCT------
---GTGTTCCTTGGGTTCTTAGGAGCA---GCAGGAAGCACTATG---GGCGCAGCGTCA---
ATAACGCTGACGGTACAG---GCCAGGCAA---TTATTG---TCTGGTATAGTGCAACAGCAGAAC---
AACTTG---CTGAAA---GCTATTGAGGCGCAACAGCATCTGTTGCAGCTCACAGTCTGGGGCATC
>KX505419
TTCTACTGTAATACAACAAACCTGTTTAATAGTACTTGGAATGTTAGTGATACTACTGGTAGGGGA-------
----

GTGTCAGACAACATCACACTCCCATGCAGAATAAAACAAGTTATAAACATGTGGCAGGAAGTAGGAAAAGCAA
TGTATGCTCCTCCCATCCCAGGACAAATTAGATGCTCATCAAAT---
ATTACAGGGTTACTATTAACAAGAGATGGTGGTAACACCTCTGAG----------------------------
----

TCTGAGACCTTCAGACCTGGAGGAGGAGATATGAGAGACAATTGGAGAAGTGAACTATATAAATATAAAGTAG
TAAAGATTGAACCATTA---GGAGTAGCACCCACTAAGGCAAAGAGAAGAGTGGTG-----------------
----------------------CAGAGAGAAAAAAGAGCA---GTGGGA---ATAGGAGCT---------
GTGTTCCTTGGGTTCTTGGGAGCA---GCAGGAAGCACTATG---GGCGCAGCGTCA---
ATAACGCTGACGGTACAG---GCCAGACAA---TTATTG---TCTGGTATAGTGCAACAGCAGAAC---
AATCTG---CTGAGA---GCTATTGAGACGCAACAGCATCTGTTGCAGCTCACAGTCTGGGGCATC
>KX505435
TTCTACTGTAATACATCAAAGCTGTTTAACAGTACTTGGAATGATACAAGTACTGGGGAGTGGGAGTCAAAT-
----

GATAATGAAACAATCATACTCCCGTGCAAAATAAGACAAATTATAAACAGATGGCAGAAAGTAGGAAGAGCCA
TGTATGCCCCTCCCATCAGAGGACAAATTAGATGTTCATCAAAT---
ATTACAGGGTTACTATTAACAAGAGATGGTGGTATAAATAGGACC----------------------------
----

GAGAATGAGACCGAGATCTTCAGACCTGGAGGAGGAGATATGAGGGACAATTGGAGAAGTGAATTGTATAAAT
ATAAAGTAGTAAAAATTGAGCCATTA---GGAATAGCACCCACCAGGGCAAGGAGAAGAGTGGTG--------
-------------------------------CAGAGAGAAAAAAGAGCA---GTGGGAACGATAGGAGCT---
------ATGTTCCTTGGGTTCTTGGGAGCA---GCAGGAAGCACTATG---GGCGCAGCGTCA---
GTGACGCTGACGGTACAG---GCCAGACTA---TTATTG---TCTGGTATAGTGCAACAGCAGAAC---
AATCTG---CTAAGA---GCTATTGAGGCGCAACAGCATCTGTTGCAACTCACAGTCTGGGGCATC
>KX505475

TTCTACTGTAATACAACAAAACTGTTTAATAGTACTTGGAATAGTACTTGGAATAGTACTTGGAATAGTGAGG
AACAGTCA--

GGTGTTGATAACATTATCACAATCCAATGCAGAATAAAACAAATTGTACACATGTGGCAGAGAGTAGGAAGGG
CAATGTATGCCCCTCCCATCAGTGGACCAATTAGATGTTCATCAAAT---
ATTACAGGGTTGCTATTAGTAAGAGATGGTGGTAGTTCCAACGATACC----------------------------
----

AAGGAGACTTTCAAGGAGACTTTCAGACCAGGAGGAGGAAATATGAAGGACAATTGGAGAAGTGAATTATATA
AATACAAAGTAGTAAAAATTGAACCATTA---GGAATAGCACCCACTAAGGCAAAGAGAAGAGTGGTG-----
-----------------------------------CAGAGAGAAAAAAGAGCA---GTGGGA---
CTAGGAGCT---------ATGTTCCTTGGGTTCTTAGGAGCA---GCAGGAAGCACTATG---
GGCGCAGCGTCG---ATGGCGCTGACGGTACAA---GCCAGACAA---TTATTG---
TCTGGCATAGTGCAGCAGCAGAAC---AATCTG---CTGAGG---
GCTATTGAGGCGCAACAGCACATGTTGCAACTCACAGTCTGGGGCATC
>KX028091
TTCTACTGTAATACAACAAAACTATTTAATAGTTCTTGGAATAGTACAGAAAGGTCACAATACAAAAACATAA
CA--

CAAAACGGAACCATAACACTCCAGTGCAGAATAAAACAAATTATAAACATGTGGCAGGAAGTAGGAAAAGCAA
TGTATGCCCCTCCCATCCGAGGAGAAATTAGATGTTCATCAAAC---
ATTACAGGGTTGCTACTAACAAGAGATGGTGGTGGTAATAGCACAGGGGAC--------------------------
----

AACGAAACCGAAACCTTCAGACCTGCAGGAGGAGATATGAGGGACAATTGGAGAAGTGAATTATATAAATATA
AAGTAGTAAAAATTGAACCATTA---GGAGTAGCACCCACCCCGGCAAGGAGAAGAGTGGTG-----------
-----------------------------CAGAGAGAAAAAAGAGCA---GTGGGA---ATAGGAGCT------
---CTGTTCCTTGGGTTCTTGGGAGCA---GCAGGAAGCACTATG---GGCGCAGCGTCA---
GTGACGTTGACGGTACAG---GCCAGACAA---TTATTG---TCTGGTATAGTGCAACAGCAAAAC---
AACTTG---CTGAGG---GCTATTGAGATGCAACAAAATCTGTTGCAACTCACAGTCTGGGGCATC
>KX028321
TTCTACTGTAATACAACAGCACTGTTTAATAGTACTTGGAATAGTACTTGGAATGGTACTACAGAGACAAGTG
GCACTGAA--

GAAAATGGAAACATCACACTCCCATGCAGAATAAAACAAATCATAAACATGTGGCAGACAGTAGGAAAAGCAA
TGTATGCCCCTCCCATCAGAGGACAAATTAGATGTTCATCAAAT---
ATTACAGGGCTACTATTAACAAGAGATGGTGGGAGTAGCACTAAT-----------------------------
----

GGGACGGAAATCTTCAGACCTGGAGGAGGAGATATGAGGGATAATTGGAGAAGTGAATTGTATAAATATAAAG
TAGTGAAGATTGAACCATTA---GGAGTAGCACCCACTAAGGCAAAGAGAAGAGTGGTG--------------
--------------------------CAGAGAGAAAAAAGAGCA---GTGGGA---TTAGGAGCT---------
TTGTTCCTTGGGTTCTTGGGAGCA---GCAGGAAGCACTATG---GGCGCAGCGTCA---
CTGACGCTGACGGTACAG---GCCAGACAA---TTGTTG---TCTGGTATAGTGCAACAGCAGAAC---
AACTTG---CTGAGA---GCTATTGAGGCGCAACAACATCTGTTGCAACTCACAGTCTGGGGCATC
>KX028508
TTCTACTGTAATACAACACAACTGTTCAATAGTACTTGGAATAATACTGGAAATAATTGGACTGATACTGGAG
AGTCA---

GACAATATCACACTTATACTCCCATGCAGAATAAAACAAATTGTAAACCTGTGGCAGAGAGTAGGAAAAGCAA
TGTATGCCCCTCCCAGCAGGGGACCAATTAGATGCTCATCAAAT---
ATTACAGGGATATTATTAACAAGAGATGGTGGTAGTGGTGTGAAC-----------------------------
----

GTGAGCAGCACAACTGAAACTTTTAGACCCGGAGGAGGAAATATGAGGGATAATTGGAGAAGTGAATTATATA
AATATAAAATAGTACAAATTGAACCAATA---GGAATAGCACCCACCAGGGCAAAGAGAAGAGTGGTG-----
-----------------------------------CAGAGAGAAAAAAGAGCA---ATAGGA---
CTAGGAGCT---------ATGTTCCTTGGGTTCTTGGGAGCA---GCAGGAAGCACTATG---
GGCGCAGCGTCA---ATGACGCTGACGGTACAG---GCCAGGCAA---CTATTG---

TCTGGTATAGTGCAACAGCAGAAC---AATTTG---CTGAGG---
GCTATTGAGGCGCAACAGCATATGTTGCAACTCACAGTCTGGGGCATC
>KX505739
TTCTACTGTAATTCAACAAAACTGTTTAATAGTACTTGGGAGGCAGGTATTACTGAAAAGTCAGAA-------
----

TCAAATGACACTATTACACTCCCATGCAGGATAAAACAAATTATAAACATGTGGCAGAAAGTAGGAAAAGCAA
TGTATGCTCCTCCCATCAGTGGAATAATTAAATGTAAATCAAAT---
ATTACAGGGCTACTATTAACAAGAGATGGTGGTAATAGTAGCGAG----------------------------
----

AATAAGAGTGAGATCTTCAGACCTGCAGGAGGAGATATGAGGGACAATTGGAGAAGTGAATTATATAAATATA
AAGTAGTAAAAATTGAACCATTA---GGAGTAGCACCCACCAAGGCAAAGAGAAGAGTGGTG-----------
----------------------------CAGAGAGAAAAAAGAGCA---GTGGGA---ATAGGAGCT------
---GTGTTCCTTGGGTTCTTGGGAGCA---GCAGGAAGCACTATG---GGCGCAGCGTCA---
ATGACGCTGACGGTACAG---GCCAGGCAA---TTATTG---TCTGGTATAGTGCAACAGCAGAAC---
AATCTG---CTGAGA---GCCATTGAGGCGCAACAGCATCTGTTGCAACTCACAGTCTGGGGCATC
>KU678060
TTCTACTGTAATACATCACAACTGTTTAACAGTACTTGGTATGGTAATTCAACACAGGATAATACTACTGGG-
----

GATAGTACTGTTATCACACTCCCATGTAGAATAAAACAAATTATAAACATGTGGCAGGGAGTAGGAAAAGCAA
TGTATGCCCCTCCCATCAGTGGATACATTAATTGTTCATCAAAC---
ATTACAGGGCTACTATTAACAAGAGATGGTATTGTGGCTAACAATACCGAT----------------------
----

AACGATACCGAGACATTCAGACCTGAAGGAGGAAACATGAAGGACAATTGGAGAAGTGAATTATATAAATATA
AAGTAGTACAAATTGAACCATTA---GGAATAGCACCCACCAAGGCAAGGAGAAGAGTGGTG-----------
----------------------------CAGAGAGAAAAAAGAGCA---GTGGGAGTGCTGGGAGCT------
---ATGTTCCTTGGGTTCTTGGGAGCA---GCAGGAAGCACTATG---GGCGCAGCGTCA---
ATAACGCTGACGGTACAG---GCCAGACTA---TTATTG---TCTGGTATAGTGCAGCAGCAGAAC---
AATCTG---CTGCGG---GCTATTGAGGCGCAACAGCATCTGCTGCAACTCACAGTCTGGGGCATC
>KX595124
TTCTACTGTAATACAACACCACTGTTTAATAGTACTTGGCCTTGGAATGGTAACAAGACACATGATACA----
----

AATAGAAACAATATCACACTCTCATGCAGAATAAAACAAATTATAAACAGATGGCAGGAAGTAGGAAAAGCAA
TGTATGCTCCTCCCATTGCAGGACGAATTAGCTGTTTATCAAAT---
ATTACAGGGATGATATTAACAAGAGATGGTGGTAACAATATCGGA----------------------------
----

GGGGAAAATAACACTGAGATCTTCAGACCTGCAGGAGGAGATATGAGGGACAATTGGAGAAGTGAATTATATA
AATATAAAGTAGTAAAAGTTGAACCATTA---GGAGTAGCACCCACCAGGGCAAGGAGAAGAGTGGTG-----
----------------------------------CAAAGAGAGAAAAGAGCA---GCGGGA---
ATAGGAGCG---------TTACTCCTTGGGTTCTTGGGAGCG---GCAGGAAGCACTATG---
GGCGCAGCGTCA---CTGACGCTGACGGTACAA---GCCAGACAA---TTATTG---
TCTGGTATAGTGCAACAGCAGAAC---AACCTG---CTGAGG---
GCTATTGAGGCGCAACAGCATATGTTGCAACTCACAGTCTGGGGCATC
>KY323732
TTCTATTGTAATTCAACACGACTGTTTAATAGTACCTGGGAGAGCCCTGATGGCCCTAGGAATGATAGTGGGG
AG---

CTCAATGACAATATCACACTCCCATGCAGAATAAAACAAATTATAAACATGTGGCAGGAAGTAGGAAAAGCAA
TGTATGCCCCTCCCATCAAAGGTATAATTAAATGCTCATCAAAC---
ATTACAGGGCTGCTATTAACAAGAGATGGGGGGAATGAGAATGTCACAGGCAAC-------------------
----

AATAAGACTGAGACCTTCAGACCTGGGGGAGGAGATATGAGGGACAATTGGAGAAGTGAATTATACAAATATA
AAGTAGTGCAAGTTGAACCACTA---GGAGTAGCGCCCACCAGGGCAAAAAGAAGAGTGGTG-----------
----------------------------CAGAGAGAAAAAAGAGCA---GTGACA---ATGGGAGCT------

---ATGTTCCTTGGGTTCTTGGGAGCA---GCAGGAAGCACTATG---GGCGCAGCATCA---
ATGATGCTGACGGTACAA---GCCAGACAA---TTGTTG---TCTGGTATAGTGCAGCAGCAGAGC---
AATTTG---CTGAAG---GCTATTGAGGCGCAACAACATCTGTTGCAACTAACAGTCTGGGGCATC
>KY323909
TTCTACTGTAATACAACACGACTGTTCAATAGTACTTGGAATAATAATAATGGTACTTGGAGTGATAATAGTA
CTGGGAAT---
-------------AATACT------
GGAGAAAATGACACACTCACACTCCCATGCAAAATAAAACAAATTATAAACATGTGGCAGAAAGTAGGAAAAG
CAATGTATGCCCCTCCTATCAGAGGGCAAATTAGATGTTCATCAAAC---
ATTACAGGGTTATTATTGACAAGAGACGGGGGAGTTAATAATACAACC-------------------------
----

AACAACACTGAGACCTTCAGACCTGGAGGGGGAGATATGAGGGACAATTGGAGAAGTGAATTATATAAATATA
AGGTAGTAAAAATTGAACCATTA---GGAGTAGCACCCACCAAGGCAAAGAGAAGAGTGGTG-----------
----------------------------CAGAGAGGAAAAAGAGCA---GTAGGC---ATAGGAGCT------
---GTGTTCCTTGGTTTCTTGGGAGCA---GCAGGAAGCACGATG---GGCGCAGCGTCA---
CTGACGCTGACGGTACAG---GCCAGACTA---TTATTG---TCTGGTATAGTGCAACAGCAGAAC---
AATTTG---CTGAGA---GCTATTGAGGCGCAACAGCATCTGTTGCAACTCACAGTCTGGGGCATC
>KY324122
TTCTACTGTAATACATCCCAACTGTTTAATAGTACTTGGAATAGTACTTGGGATAATACTGGAGAGTCAAATA
GCACTGAA---

GGAAATGACACAATCACACTCCCATGTAGAATAAAACAATTTATAAATTTATGGCAGCAAGTAGGAAAAGCAA
TGTATGCCCCTCCCATCAGGGGAAACATTACCTGTTCATCAAAT---
ATTACAGGGCTGATATTAACAAGAGATGGTGGTAATGATAACGGG----------------------------
----

AGTAACGAGACCTTCAGACCTGCAGGGGGAGATATGAGGGACAATTGGAGAAGTGAATTATATAAATATAAAG
TAGTAAAAATTGAACCATTA---GGAGTAGCACCCACCAAGGCAAAGAGAAGAGTGGTG--------------
-------------------------CAGAGAGAAAAAAGAGCA---GTGGGC---ATAGGAGCT---------
TTGTTCCTTGGGTTCTTGGGAGCA---GCAGGAAGCACTATG---GGCGCAGCGTCA---
ATGACGCTGACGGTACAG---GCCAGACAA---TTGTTG---TCTGGTATAGTGCAACAGCAAAAC---
AACCTG---CTGAGA---GCTATTGAGGCGCAACAGCATATGTTGCAACTCACAGTCTGGGGCATC
>KX028240
TTCTATTGTAATACAACACAATTGTTTAATAGTACTTGGGATGCTAATACTACTGTACAAAATACTGGAGAAA
ATGACACT---

GGAGAAAATGACACTATTATACTCCAATGCAAAATAAAACAAATTATAAACATGTGGCAGCGAACAGGAAAAG
CAATGTATGCCCCTCCCATCAGAGGACAAATTAGATGTTCATCAAAT---
ATTACAGGGCTGCTTTTAGCAAGAGATGGTGGCAACATGACCGAG----------------------------
----

AGCACGGAGGTCTTCAGACCTACAGGAGGAAATATGAAGGACAATTGGAGAAGTGAATTATATAAATACAAAG
TAGTTGAACTTGAACCATTA---GGAATAGCACCCACCAAGGCAAAGAGAAGAGTGGTG--------------
-------------------------CAGAGAGAGAAAAGAGGA------------ATAGGAGCT---------
GTGTTCCTTGGGTTCTTGGGAGCA---GCAGGAAGCACTATG---GGCGCAGCGTCA---
GTGACGCTGACGGTACAG---GCCAGACAA---TTATTG---TCTGGTATAGTGCAACAGCAGAGC---
AATTTG---CTGAAG---GCTATTGAGGCGCAACAACATTTGTTGCAACTCACAGTCTGGGGCATC
>KX505555
TTCTACTGTAACACTGACAAACTGTTTAATAGTACTTGGAATAGTAATGGTAGTGGTACCTGGAATAGGGATG
ATACTGAA---

GAGTCAAATGACACAATCACACTCCAATGCAGAATAAAACAAATCATAAACATGTGGCAGGAAGTAGGAAAAG
CAATGTATGCCCCTCCCATCGAGGGACAAATTAAATGTTCATCAAAT---
ATTACAGGGCTGCTATTAACAAGAGATGGTGGTAATTCTAGCAGCACG-------------------------
----

AACGACGAGATATTTAGACCTGGAGGAGGAGATATGAAGGACAATTGGAGAAGTGAATTATACAAATATAAAG
TAGTAAAAATTGAACCAATA---GGGATAGCACCTGAAAAGGCACAGAGAAGAGTGGTG--------------
-------------------------CAGAGAGAAAAAAGAGCA---GTGGGAGCATTAGGAGCT---------

TTGTTCCTTGGGTTCTTAGGAGCA---GCAGGAAGCACTATG---GGCGCAGCGTCA---
GTGACGCTGACGGTACAG---GCCAGACAA---TTATTG---TCTGGTATAGTGCAACAGCAAAAC---
AATTTG---CTGAGA---GCTATTGAGGCGCAACAGCATCTGTTGCAACTCACGGTCTGGGGCATC
>MH897920
TTCTACTGTAATACATCAGAACTGTTTAAAGGTACTTGGAATGGTACTGAAAGGCCAAATGACACTGAAAAG-
----

TTAAATGATACAATTACACTCCAATGCAGAATAAAACAATTTATAAACATGTGGCAGGAAGTAGGAAAAGCAA
TGTATGCCCCTCCAATCAGAGGACAAATTAACTGTTCATCAAAT---
ATTACAGGGCTGCTACTAACAAGAGATGGTGGAAATGGAAATGAGACG-------------------------
----

AAAGAGACTGAGGTCTTCAGACCTGGAGGAGGAGATATGAGGGACAATTGGAGAAGTGAATTATATAAATATA
AAGTAGTAAAAATTGAACCATTA---GGAATAGCACCCACCAAGGCAAGGAGAAGAGTGGTG-----------
----------------------------CAGAGAGAAAAAAGAGCA---GTGGGA---ATAGGAGCT------
---CTGTTCCTTGGGTTCTTGGGAGCA---GCAGGAAGCACTATG---GGCGCAGCGTCA---
GTGACGCTGACGGTACAG---GCCAGACAA---TTATTG---TCTGGTATAGTGCAACAGCAGAGC---
AATTTG---CTGAGA---GCTATTGAGGCGCAGCAGCATATGTTGCAACTCACAGTCTGGGGCATC
>KX587137
TTCTACTGTAATACATCAAAACTGTTTAATAGTACTTGGTTTAATAATGGGACTAATAGTGAAGAGTCG----
----

TCAAATGACACTATCACACTTCCATGCAGGATAAAACAAATTGTAAATATGTGGCAGGAGGTAGGAAAAGCAA
TGTATGCCCCTCCCATTAGAGGACAAATTAGATGTAACTCAAGT---
ATTACAGGGATACTATTAGTAAGAGATGGTGGTAAGACCAACAGTACCAATGAG------------AGC----
----
AGTACCAATGAGAGC---
GAGATCGAAATCTTCAGACCTGGAGGAGGAGACATGAGGGACAATTGGAGAAGTGAATTATATAAATATAAAG
TAGTACAAATTGAACCATTA---GGAGTAGCACCCACCAAGGCAAAAAGAAGAGTGGTG--------------
-------------------------CAGAGAGAAAAAAGAGCA---ATAGGA---ATGGGAGCT---------
TTGTTCCTTGGGTTCTTAAGCGCA---GCAGGAAGCACTATG---GGCGCAGCGTCA---
GTGACGCTGACGGTACAA---GCCAGACAA---TTACTG---TCTGGTATAGTGCAACAGCAGAAC---
AACCTG---CTGAGG---GCTATTGAGGCGCAGCAGCATCTGCTGCAACTCACAGTCTGGGGCATC
>KX587284
TTCTACTGCAATACAACAAAACTGTTTAATAGCACTTGGAATTCAACACATAGTTGGAACGATACAGGG----
----

CCAAATGACAATGTCACACTTCCATGCAGGATAAAACAAATCATAAACATGTGGCAGGAAGTAGGAAAAGCAA
TGTATGCCCCTCCTATTAAGGGAGAAATTAAATGTTCATCAAAT---
ATTACAGGGCTGATATTAACTAGAGATGGTGGGACAGGTAATGAGACT-------------------------
----

GACAACAACACTGAGACCTTCAGACCAGGAGGAGGAGATATGAGAAACAATTGGAGAAGTGAATTATATAAAT
ATAAAGTAATAAGAATTGAACCAATA---GGATTAGCACCCACCAAGGCAAAGAGAAGAGTGGTG--------
-------------------------------CAGAGAGAAAAAAGAGCA---ATACCA---ATAGGAGCT---
------GTGTTCTTTGGGTTCTTGGGAGCA---GCAGGAAGCACTATG---GGCGCAGCGTCA---
ATAGCGCTGACGGCACAG---GCCAGACAA---TTACTG---TCTGGTATAGTGCAACAGCAAAAC---
AATTTG---CTGAGG---GCTATTGAGGCGCAACAACATCTGTTGCAACTCACAGTCTGGGGCATC
>KY113738
TTCTACTGTAATTCAACACAACTGTTTAATAGTACTTGGAATAGTAATAGTACTCGGAATGGTACTGAAGGGT
CAAATATCACT--

GTAGAAAATGACACAATCATACTTACTTGCAGAATAAAACAAATTATAAACATGTGGCAGGAAGTAGGAAAAG
CAATGTATGCCCCTCCCATCAGTGGAATAATTAGATGTTCATCAAAT---
ATTACAGGGCTGCTATTAACAAGAGATGGTGGTAACAGTAGCAAC----------------------------
----

GGGACAGAGGTCTTCAGACCTGGAGGAGGAAATATGAAAGACAATTGGAGGAGTGAATTATATAAATATAAAG
TAGTAAAAATTGAACCATTA---GGAATAGCACCCACCAAGGCGAAGAGAAGAGTGGTG--------------
-------------------------CAGAAAGAAAAAAGAGCA---GCA------CTAGGAGCC---------

TTGTTCCTTGGGTTCTTGGGAGCA---GCAGGAAGCACTATG---GGCGCAGCGTCA---
ATGACGCTGACGGTACAG---GCCAGACTA---TTATTG---TCTGGTATAGTGCAACAGCAAAAT---
AATTTG---CTGAGG---GCTATTGAGGCGCAACAGCATCTGTTGCAACTCACAGTCTGGGGCATT
>KY113566
TTCTACTGTAATACATCAAAACTGTTTAATAGTACTTGGAATAATAATAGTACTTGGAATAGTACTACTACTG
AAAGAAAT---

GAGACAGAAGGAAATATCACACTCCCATGTAGAATAAAACAAATTATAAACATGTGGCAGGAAGTAGGAAAAG
CAATGTATGCCCCTCCCATTAGGGGAATAATTAAATGTTCATCAAAT---
ATTACAGGATTGCTATTGACAAGAGATGGTGGTAAAGACAATACC----------------------------
----
ACAAATAATAATGGG---
ACTGAGGAGGTCTTCAGACCAGGAGGAGGAGATATGAGGGACAATTGGAGAAGTGAATTGTATAAATATAAAG
TAGTAAGAATTGAACCATTA---GGAGTAGCACCCACCAAGGCAAAGAGAAGAGTGGTG--------------
-------------------------CAGAGAGAAAAAAGAGCA---GTGACA---CTAGGAGCT---------
ATGTTCCTTGGGTTCTTGGGAGCA---GCAGGAAGCACTATG---GGCGCAGCGTCA---
ATAACGCTGACGGCACAG---GCCAGACAA---TTATTG---TCTGGTATAGTGCAACAGCAGAAT---
AATCTG---CTGAGA---GCTATTGAGGCGCAACAGCATCTGTTGCAACTCACAGTCTGGGGCATC
>KY113379
TTCTATTGTAATTCAACACAACTGTTTAATAGTACTTGGAATAATACTGATTGGAATAATACTGAAGAGGCAA
ATAAA--
-------------ACTAGA------
GGAGGAAATGACACAATTATACTCCCATGTAGAATAAAACAAATTATAAACATGTGGCAGGAAGTAGGAAAAG
CAATGTATGCCCCTCCCATCAGAGGAAACATTAGTTGTTTTTCAAAG---
ATTACAGGGCTGTTACTCACAAGAGATGGTGGTAATAGTAATATTACT-------------------------
----

AGTGAGAGCGAGACCTTCAGACCTGCAGGAGGAGATATGAGGGACAATTGGAGAAGTGAGTTATATAAATATA
AAGTAGTAAAAATTGAACCCTTA---GGAGTAGCACCCACCAAGGCAAAGAGAAGAGTGGTG-----------
----------------------------CAGAGGGAAAAAAGAGCA---GTTGGA---ATTGGAGCT------
---TTGTTCCTTGGGTTCTTGGGAGCA---GCAGGAAGCACTATG---GGCGCAGCATCA---
TTAACGCTGACGGTACAG---GCCAGACAA---TTATTG---TCTGGTATAGTGCAACAGCAGAAC---
AATTTG---CTGAGA---GCTATTGAGGCGCAACAGCATCTGTTGCAACTCACAGTCTGGGGCATC
>KY057390
TTCTACTGTAACACATCAAAACTATTTAATGGAACTTGGAATGGTACTGATGGAACTTGGAATGATATT----
----

ACTGATGAAATTATCACAATCCCATGCAGAATAAAACAATTTATAAACCTGTGGCAAGAAGTAGGAAAAGCAA
TGTATGCCCCTCCCATCGCAGGACCAATTAGCTGTTCATCAAAC---
ATTACAGGACTGATATTAACAAGAGATGGTGGTAATACTAATAGCACT-------------------------
----

AAGGGGAAGGAGACCTTCAGACCGGGAGGAGGAGATATGAGGGACAATTGGAGAAGTGAATTATATAAATATA
AAGTAGTAAAAATTGAACCATTA---GGAATAGCACCCACCAGGGCAAGGAGAAGAGTGGTG-----------
----------------------------CAGAGAGAAAAAAGAGCA---GCA------ATAGGAGCT------
---CTGTTCCTTGGGTTCTTGGGAGCA---GCAGGAAGCACGATG---GGCGCAGCATCA---
GTGACGCTGACGGTACAG---GCCAGACTA---CTATTG---TCTGGTATAGTGCAACAGCAGAAC---
AACCTG---CTGAGA---GCTATTGAGGCGCAACAGCATATGTTGCAACTCACAGTCTGGGGCATC
>KY748402
TTCTACTGTAATTCAACACAACTGTTTAATAGTACTTGGAATAGTACTTTGAATACTACTTGGGAGGTTAAT-
----

GAGACTGACATTATCACACTCCCATGCAGAATAAAACAAATTATAAACATGTGGCAGGAAGTAGGAAAAGCAA
TGTATGCCCCTCCCATCCAAGGAAGAATTAAATGCTCATCGAAT---
ATTACAGGGCTGTTACTAATAAGAGATGGTGGTAATAACAATGGTAGCGAA----------------------
----

ACAACAGAAATCTTCAGACCTGGAGGAGGAGACATGAGGGACAATTGGAGAAGTGAATTATATAAATATAAGG
TAGTAAAAATTGAGCCATTA---GGAATAGCACCCACCAAGGCAAAGAGAAGAGTGGTG--------------
-------------------------CAAAGAGAAAAAAGAGCA---GTGGGAGTATTTGGAGCT---------

ATGTTCCTCGGGTTCTTGGGAACA---GCAGGAAGCACTATG---GGCGCAGCGTCA---
ATAACGCTGACGGTACAG---GCCAGACAA---TTATTA---TCTGGTATAGTGCAACAGCAAAAC---
AATTTG---CTGAGA---GCTATTGAGGCGCAACAGCATCTGTTGCAACTCACAGTCTGGGGCATT
>KY057518
TTCTACTGTAATACAACACAACTGTTTAATAGTGCTTGGAATAATACAGGGTCAAATGGAGCT----------
----

ACCAGTGACAACATCACGCTCCCATGCAGAATAAAGCAAATTATAAACATGTGGCAGGAAGTAGGAAAAGCAA
TGTATGCCCCGCCCATCAGTGGACTAATTAACTGTACATCAAAT---
ATTACAGGGCTACTATTAACAAGAGATGGGGGAAATGGAAATGACACAAAT----------------------
----

AAAACCGATGAGATCTTCAGACCTGGAGGAGGAAATATGAGGGACAATTGGAGAAATAAATTATATAAATATA
AAGTAGTGAAAATTGAACCATTA---GGAGTAGCACCCACCAAGGCAAAGAGAAGAGTAGTG-----------
----------------------------CAGAGAAAAAAAAGAGCA---TTGGGA---ATAGGAGCT------
---GTGTTCCTTGGGTTCTTGGGAGCA---GCAGGAAGCACTATG---GGCGCAGCGTCA---
ATGACGCTGACGGTACAG---GCCAGACAA---TTATTG---TCTGGTATAGTGCAGCAGCAGAAC---
AATTTG---CTGAGA---GCTATTGAGGCGCAACAGCATGTGTTGCAACTCACAGTCTGGGGCATC
>KY324010
TTCTACTGTGATACAACAAAATTGTTTAATAGTACTTGGAAGCTTAATAGTACTTGGAATAGTATGGAAGAAG
TGACAAAT---
-------------AGCACT------
AAAGGAGATGCCAACATCACACTCCCCTGCAGAATAAAACAAATTGTAAACATGTGGCAGAGAGTAGGAAGAG
CAATGTATGCCCCTCCCATCCAAGGACGAATTAGCTGTTCATCAGAT---
ATTACAGGGCTGCTATTACTAAGAGATGGTGGTAGTGGTAATAATACCACG----------------------
----

AATGATACTGAGATCTTCAGACCTGGAGGAGGAAATATGAAGGATAATTGGAGAAGTGAATTATATAAATATA
AAGTAGTAAAAATTGAACCATTA---GGAGTAGCACCCACCAAGGCAAAGAGAAGAGTGGTG-----------
----------------------------CAGAGAGAAAAAAGAGCA---GCGGGA---ATAGGAGCT------
---GTGCTCCTTGGGTTCTTGGGAGCA---GCAGGAAGCACTATG---GGCGCAGCGGCA---
GTGACGCTGACGGTACAG---GCCAGACAA---TTATTG---TCTGGTATAGTGCAGCAGCAGAAT---
AATTTG---CTGAAG---GCTATTGAGGCGCAACAACATCTGTTGCAACTCACAGTCTGGGGCATC
>KY324473
TTCTACTGTAATACAACATCATTGTTTAATAGTACTTGGTATGCTAATAATACTACTGAAGGGACAAATAGC-
----

ACTGGAAGAAATATCACACTCCAATGCAGAATAAGACAAATTATAAACATGTGGCAGAAAGTAGGAAAAGCAA
TGTATGCCCCTCCCATCAGTGGAGTAATTAACTGTAAATCAAAT---
ATTACAGGGCTGATATTAACGAGAGATGGTGGTAACAATAATGGTAACAAT----------------------
----
-
AATAATACCAACGAAACCGAGACCTTCAGACCAGGAGGAGGAGATATGAGGGACAATTGGAGAAGTGAATTAT
ATAAATATAAAGTAGTAAAAATTGAACCATTA---GGAATAGCACCCACCAGGGCACAAAGAAGAGTGGTG--
-------------------------------------CAGAGAGAAAAAAGAGCA---GTGGGA---
TTAGGAGCT---------TTGTTCCTTGGGTTCTTGGGAGCA---GCAGGAAGCACTATG---
GGCGCAGCATCA---ATGACGCTGACGGTACAG---GCCAGACAA---TTATTG---
TCTGGTATAGTGCAGCAGCAAAGC---AATTTG---CTGAGA---
GCTATTGAGACGCAACAGCATCTGTTGCAACTCACAGTCTGGGGCATT
>MK148554
TTCTACTGTAATACAACACAACTGTTTAATAGTACTTGGAATGGTACTACTGAAGGGTTAAATAACACTGCA-
----

GGAAATGGCACAATCACACTTCCATGCAGAATAAAACAAATTATAAACATGTGGCAGGAAGTAGGAAAAGCAA
TGTATGCCCCTCCCATCAGAGGACTAATTAGATGTTCATCAAAT---
ATTACAGGGCTGCTATTAACAAGGGATGGTGGTGATGGTAATAGC----------------------------
----

AACGAGACCGAGGTTTTCAGACCTGGAGGAGGAGATATGAGGGACAATTGGAGAAGTGAATTATATAAATATA
AAGTAGTAAAAATTGAACCATTA---GGAGTAGCACCCACCAAGGCAAAGAGAAGAGTGGTG-----------

----------------------------CAAAGAGAAAAAAGAGCA---GTGGGA---ATAGGAGCT------
---ATGTTCCTTGGGTTCTTGGGAGCA---GCAGGAAGCACTATG---GGCGCAGCGTCA---
ATGACGCTGACGGTACAG---GCCAGACTA---TTATTG---TCTGGTATAGTGCAACAGCAGAAC---
AATTTG---CTGAGG---GCTATTGAGGCGCAACAACATCTGTTGCAACTCACAGTCTGGGGCATC
>MK114705
TTCTACTGTAATACAACAAAACTATTTAATAGTACTTGGCCACGTAATAGTACTTGGAATAATACTGAAGGGT
CAAAT---

GACACTGAAATAATCACACTCCCGTGCAGAATAAAACAAATTGTAAACAGGTGGCAGGAAGTAGGCAAAGCAA
TGTATGCCCCTCCCATCCAAGGACAAATTAGTTGTTCATCAAAT---
ATTACAGGGCTGCTACTAGTTAGAGATGGTGGAATTAACACCAGT----------------------------
----

GAGAGCAACGAGACCTTCAGACCTGGAGGAGGAGATATGAGGGACAATTGGAGAAGTGAATTATATAAATATA
AGGTAGTAAAAATTGAACCATTA---GGAGTAGCACCCACCAAGGCAAAGAGAAGAGTGGTG----------
----------------------------CAGAGAGAAAAAAGAGCA---GTGGGAATGCTGGGAGCT------
---ATGTTCCTTGGGCTCTTGGGAGCA---GCAGGAAGCACTATG---GGCGCAGCGTCA---
GTGACGTTGACGGTACAG---ACCAGACTA---TTATTG---TCTGGTATAGTGCAACAGCAGAAC---
AATCTG---CTGAGG---GCTATTGAGGCGCAGCAGCATCTGTTGCAACTCACAGTCTGGGGCATC
>MK114983
TTCTACTGTAACACAACACGACTGTTTAATAGTACTTGGAGTGTTAATGGAACTAGCATAAACGAAACT----
----

AACAATAAAAATATCACACTCCCGTGCAGAATAAAACAAATTATAAACAGGTGGCAGGAAGTAGGAAAAGCAA
TGTATGCCCCTCCTATCAGTGGGATAATTAGGTGCTCATCAAAT---
ATTACAGGACTAATATTAACAAGAGATGGTGGTACAACTGATGGTGGTACA----------------------
----

ACTAATAGTACAAAGGAGACCTTCAGACCTGGAGGGGGAAATATGAAGGACAATTGGAGAAGTGAATTATATA
AATACAAAGTAGTAAAAATTGAGCCATTA---GGAGTAGCACCCACCAAGGCACAGAGAAGAGTGGTG-----
-----------------------------------CAAAGAGAAAAAAGAGCA---
ATAGGAACGTTAGGAGCT---------ATGTTCCTCGGGTTCTTGGGAGCA---GCAGGAAGCACTATG---
GGCGCAGCGTCA---CTGACGCTGACGGTACAA---GCCAGACAA---TTATTG---
TCTGGTATAGTGCAACAGCAGAAC---AATCTG---CTGAGG---
GCTATTGAGGCGCAACAGCATATGTTGCAACTCACAGTCTGGGGCATC
>MK115065
TTCTACTGTAATACAACACAGCTGTTTAATAGTACTTGGAATAATAGTACTTGGAATGGTACTGACAATTGGA
AT--
--------------GGTACT------
GAATCAAATAGCACTATCACACTCCCATGCAGAATAAAACAAATTATAAACTTGTGGCAGGAAGTAGGAAGAG
CAATGTATGCCCCTCCCATCCAAGGGCAAATTAGATGTTCATCAAAT---
ATTACAGGGCTGCTGCTAGTAAGAGATGGTGGGAGCAACAATAGTAGT-------------------------
----

AATGATACAGAGACCTTCAGGCCTGGAGGAGGAGATATGAGGGACAATTGGAGAAGTGAATTATATAAATATA
AAGTAGTCAAAATTGAACCATTA---GGAATAGCACCCACCCATGCAAAGAGAAGAGTGGTG----------
----------------------------CAAAGGGAAAAAAGAGCA---ATAGGA---CTTGGAGCT------
---TTCTTCCTTGGGTTCTTGGGAGCA---GCAGGAAGCACTATG---GGCGCAGCGTCA---
GTGACGCTGACGGTACAG---GCCAGACAA---TTATTG---TCTGGTATAGTGCAGCAGCAAAAC---
AATTTG---CTGAGA---GCTATTGAGGCGCAACAGCATCTGTTGCAACTCACAGTCTGGGGCATC
>MK115195
TTCTACTGTAATACAACACAACTGTTTAATAGTACTTGGTATAGTACTACAGGAGTAAATGGTACAGGAGCAA
ATAACACA--

GAAGTAAATATTATCACACTCCCATGCAGGATAAGGCAAATTGTAAACATGTGGCAGGAAGTAGGAAAAGCAA
TGTATGCCCCTCCCATCAGTGGACAAATTAGTTGTTCATCAAAT---
ATTACAGGGCTACTACTACTAAGAGATGGTGGTAATAGCAATAAC----------------------------
----

ACGAATAACATGACTGAAATCTTCAGACCTGGAGGAGGAGATATGAGAGACAATTGGAGAAGTGAATTATATA

AATATAAAGTAGTACAAATTGAACCATTA---GGAATAGCACCCACCAAGGCAAGGAGAAGAGTGGTG-----
----------------------------------CAGAGAGAAAAAAGAGCA---GTGGGA---
ATAGGAGCT---------CTGTTCCTTGGGTTCTTGGGAGCA---GCAGGAAGCACTATG---
GGCGCAGCATCA---ATAACGCTGACGGTACAA---GCCAGACAC---TTATTG---
TCTGGTATAGTGCAACAGCAGAAC---AATTTG---CTGAGA---
GCTATTGAGGCGCAACAGCATATGTTGCAGCTCACAGTCTGGGGCATC
>MK115360
TTCTACTGTAATACAACACAACTGTTCAATAGTACCTGGCCCAATAATAGTACTGGGAATGATACGGGAGGA-
----

CCAAATGGTACTATCACACTCCCATGCAAAATAAAACAGATTGTAAACTTATGGCAGGAAGTAGGAAAAGCAA
TGTATGCCCCTCCCATCAAAGGACAAATTAGATGTTCATCAAAT---
ATTACAGGGATGATATTAATAAGAGATGGTGGATCAGGTAATGAAAGTAATACC-------------------
----

AATAGTGAGACCTTCAGACCTGGAGGAGGAAATATGAAGGACAATTGGAGAAGTGAATTATACAAATATAAAG
TAGTAAAAATTGAACCATTA---GGAATAGCACCCACCAGGGCAAAGAGAAGAGTGGTG--------------
-------------------------CAGAGAGAAAGAAGAGCA---GTGGGA---ATAGGAGCT---------
TTGTTCCTTGGGTTCTTGGGAGCG---GCAGGAAGCACTATG---GGCGCAGCGTCG---
ATGACGCTGACGGTACAG---GCCAGACAA---TTATTG---TCTGGTATAGTGCAGCAGCAGAAC---
AACCTG---CTGAGG---GCTATTGAGGCACAACAGCATCTGTTGCAACTCACAGTCTGGGGCATT
>MK115488
TTCTATTGTAATACAACACAGCTGTTTAATAGTACTTGGAATAGTACTTGGACTGGGAATGCTACCGAA----
----

TTAGATGGAAACATCACACTCCCATGCAGAATAAAACAGATTATAAACAGGTGGCAAGAAGTAGGAAAAGCAA
TGTATGCCCCTCCCATTAGAGGAAAAATTAGATGTTCATCAAAT---
ATTACAGGGTTACTATTAGTAAGAGATGGTGGTAACACGAGCGGG----------------------------
----

ACTGAGACCTTCAGACCTGGAGGAGGAGATATGAAGGACAATTGGAGGAGTGAATTATATAAATATAAAGTAG
TAAAAATTGAGCCATTA---GGAGTAGCACCCACCAGGGCAAAGAGAAGAGTGGTG-----------------
----------------------CAGAGAGAAAAAAGAGCA---GTGGGA---ATAGGAGCT---------
GTGTTCCTTGGGTTCTTGGGAGCA---GCAGGAAGCACTATG---GGCGCAGCGTCA---
GTGACGCTGACGGTACAA---GCCAGGCTG---TTATTG---TCTGGTATAGTGCAACAGCAAAAC---
AATTTG---CTAAGA---GCTATTGAGGCGCAACAACATCTGTTGCAACTCACAGTCTGGGGCATC
>MK115745
TTCTACTGTAATACAACAAAACTGTTT------ACTTGGAATGCTACAGAT----------------------
----

AGCAATGGAACCATCATACTCCCATGTAGAATAAAACAAATTATAAACATGTGGCAAGAGGTAGGAAAAGCAA
TGTATGCCCCTCCCATTCGTGGACAAATTAGATGTTCATCAAAT---
ATTACAGGACTGCTATTAACAAGAGATGGTGGGACTAACAGGACA----------------------------
----

GAGAACAGGACTGAGATCTTCAGACCTGGAGGGGGAAATATGAAGGACAATTGGAGAAGTGAATTATATAAAT
ACAAAGTAGTAGAAATTAAACCATTA---GGAGTAGCACCCACCAAGGCAAAGAGAAGAGTGGTG--------
-------------------------------CAGAGAGAAAAAAGAGCA---GTGACC---ATAGGAGCT---
------ATGTTCCTTGGGTTCCTGGGAGCA---GCAGGAAGCACTATG---GGCGCAGCATCA---
CTGACGCTGACGGTACAG---GCCAGACAA---TTATTG---TCTGGTATAGTGCAACAGCAGAAC---
AATCTG---CTGAGG---GCGATTGAGGCGCAGCAGCATCTGTTGCAACTCACAGTCTGGGGCATA
>KX587450
TTCTACTGTAATACAACACCACTGTTTAGCAGCTCTTGGAATGTTACTACAGGGGCAAATATCACTGGAGGAA
ATAGC--

ACACAAAATAATATCACACTCCGATGCAGAATAAGACAATTTATAAACATGTGGCAGGAAGTAGGAAAAGCAA
TGTATGCCCCTCCCATCAAAGGACCCATTAAATGCTCATCGAAT---
ATTACAGGGCTGCTATTAGTAAGAGATGGTGGTATAAATGTGACA----------------------------
----
-

GACACAGACAACAACACAGAGACCTTCAGACCTGGAGGAGGAAATATGAAAGACAATTGGAGAAGTGAATTAT
ATAAATATAAAGTAGTAAAAATTGAACCATTA---GGAGTAGCACCTACCAAGGCAAAGAGAAGAGTGGTG--
-------------------------------------CAGAGAGAAAAAAGAGCA---GTGGGA---
CTAGGAGCT---------ATGTTCCTTGGGTTCTTAGGAGCA---GCAGGAAGCACTATG---
GGCGCAGCGTCG---CTGACGCTGACGGTACAG---GCCAGACAG---TTACTG---
TCTGGTATAGTGCAGCAGCAGAAC---AATCTG---CTGAGG---
GCTATTGAGGCGCAACAACATCTGTTGCAACTCACAGTCTGGGGCATC
>MK499378
TTCTACTGTGATACAACACAACTGTTTAATAGTACTTGGTCAAATGATACCTGGTTGAATAGTACTTGGAAT-
----
-GGAAATGAAGGGTCAAAT------
AGCAATGGAGAACCTATCACACTCCAATGCAGAATAAAACAGATTATAAACATGTGGCAGGAAGTAGGAAAAG
CAATGTATGCCCCTCCCATCAGAGGAGAAATTAACTGTTCATCAAAT---
ATTACAGGGCTGCTCTTAACAAGAGATGGTGGTAATAATAATACC----------------------------
----

ATGAACGAGACCTTCAGACCAGGAGGAGGAGATATGAGGGACAATTGGAGAAGTGAATTATATAAATATAAAG
TAGTAAAAGTTGAACCATTA---GGAGTAGCACCCACCAAGGCAAAGAGAAGAGTGGTG--------------
-------------------------CAAAGAGAAAAAAGAGCA---GTGGGA---ATAGGAGCT---------
GTGTTCCTTGGGTTCTTGGGAGCA---GCAGGGAGCACTATG---GGCGCAGCGTCA---
ATGACGCTGACGGTACAA---GCCAGACTA---TTATTG---TCTGGTATAGTGCAACAGCAGAAC---
AATTTG---CTGAGG---GCTATTGAGGCGCAACAGCATATGTTGCAACTCACAGTCTGGGGCATC
>MK169493
TTCTACTGTAACACAACAAAACTGTTTAATAGCTCTTGGCCTGAGAATAATACTAAAAATAATACTAGTAATA
AC---

ACTGGAGAAATAATAACACTCCCATGTAAAATAAAACAAATTATAAACATGTGGCAGGAAGTAGGAAGAGCAA
TGTATGCCTCTCCCATCAGTGGACAAATTAACTGTAACTCAAGT---
ATTACAGGGCTGTTGTTAACAAGAGATGGTGGTAAAAAGACTGAT----------------------------
----

AATGAGACCTTCAGACCTGCAGGGGGAGATATGAGGGACAATTGGAGAAGTGAATTATATAAATATAAAGTAG
TAAAAGTTGAACCATTA---GGAATAGCACCCACCAAGGCAAAGAGAAGAGTGGTG-----------------
----------------------CAGAGAGAAAAAAGAGCA---GTGGGA---ATAGGAGCT---------
GTGTTCCTTGGGTTCTTGGGAGCA---GCAGGAAGCACTATG---GGCGCAGCGTCA---
ATAACGCTGACGGTACAG---GCCAGACAG---TTATTG---TCTGGTATAGTGCAACAGCAGAGC---
AATCTG---CTGAGA---GCTATTGAGGCGCAACAGCATATGTTGCAACTCACAGTCTGGGGCATT
>MK169546
TTCTACTGTAATACAACAGCATTGTTTAATAGTACTTGGTTTAATGGTACTTGGTCTAATAGCACTGTGGAAT
GG---

CCAAATGATACTATCACACTCCCATGCAGAATAAAACAAATTATAAACATGTGGCAGGAAGTAGGAAAAGCAA
TGTACGCCCCTCCCATCAGAGGACAAATTAATTGTTCATCAAAT---
ATTACAGGGCTATTATTACACCGAGATGGTGGTAATAATGTTAGT----------------------------
----

AATAATGAGACCTTTAGACCTATAGGAGGAGATATGAAAAACAATTGGAGAAGTGAATTATACAAATATAAAG
TAGTAAGAATTGAACCAATA---GGAGTAGCACCCACCAAGGCAAAGAGAAGGGTGGTG--------------
-------------------------CAGAGAGAAAAAAGAGCA---ATAACA---CTAGGAGCT---------
ATGTTCCTTGGGTTCTTGGGAGCA---GCAGGAAGCACTATG---GGCGCAGCGTCA---
CTGACGCTGACGGTACAG---GCCAGACAA---TTATTG---TCTGGTATAGTGCAACAGCAAAAC---
AATTTG---CTGAGA---GCTATTGAGGCGCAACAACATCTGTTGCAACTCACAGTCTGGGGCATC
>MK169551
TTCTACTGTAATACAACAGCATTGTTTAATAGTACTTGGTGGTCTAATAGTACTTGGCCTAATAGTACTGTGG
AAGAG--

TCAAATAGAACTATCACACTCCCATGCAGAATAAAACAAATTATAAACATGTGGCAGGAAGTAGGAAAAGCAA
TGTATGCCCCTCCCATCAGCGGACAAATTAATTGTGTATCAAAT---
ATTACAGGGCTGTTATTACACCGAGATGGTGGTGAAAATAGTAATAATAATACT-------------------
----

-
GAGGTTAGTAATAGTACTGAGACCTTTAGACCCATAGGAGGAGATATGAAGAACAATTGGAGAAGTGAATTAT
ACAAATATAAAGTAGTAAGAATTGAACCAATA---GGAGTAGCACCCACCAAGGCAAAGAGAAGGGTGGTG--
-------------------------------------CAGAGAGAAAAAAGAGCA---GTAACA---
CTAGGAGCT---------ATGTTCCTTGGGTTCTTGGGAGCA---GCAGGAAGCACTATG---
GGCGCAGCGTCA---CTGACGCTGACGGTACAG---GCCAGACAA---TTATTG---
TCTGGTATAGTGCAACAGCAAAAC---AATTTG---CTGAGA---
GCTATTGAGGCGCAACAACATCTGTTGCAACTCACAGTCTGGGGCATC
>MK169676
TTCTACTGTAATACAACACTATTGTTTAATAGTACTTGGTCTAATAGTACTGTGGAAGGGTCAAATAACACTG
AA---

GAAAATAGCACACTCACACTCCCATGCAGAATAAAACAAATTATAAACATGTGGCAGGGAGTAGGAAAAGCAA
TGTATGCCCCTCCCATCAGAGGACAAATTAGATGTTCATCAAAT---
ATTACAGGACTGTTATTGACACGAGATGGTGGTAATAATAGTGCT----------------------------
----

AATAATGAGACCTTTAGACCTGGAGGAGGAAATATGAGGGACAATTGGAGAAGTGAATTATATAAATATAAAG
TAGTAAGAATTGAACCATTA---GGAGTAGCACCCACCAAGGCAAAGAGAAGAGTGGTG--------------
-------------------------CAGAGAGAAAAAAGAGCA---GTAACA---CTAGGAGCT---------
ATGTTCCTTGGGTTCTTGGGAGCA---GCAGGAAGCACTATG---GGCGCAGCGTCA---
CTGACGCTGACGGTACAG---GCCAGACAA---TTGTTG---TCTGGTATAGTGCAACAGCAAAAC---
AATTTG---CTGAGA---GCTATTGAGGCGCAACAACATCTGTTGCAACTCACAGTCTGGGGCATC
>MK169581
TTCTACTGTAATACAACAGCATTGTTTAATAGTACTTGGTTTAATGGTACTTGGTCTAATAGTACTGTGAAAG
GG---

TCAAATAATACTATCACACTCCCATGCAGAATAAAACAAATTATAAACATGTGGCAGGAAGTAGGAAAAGCAA
TGTATGCCCCTCCCATCAGAGGACAAATTAATTGTGTATCAAAT---
ATTACAGGGCTGTTATTACACCGAGATGGTGGTAAAAAAGTTAGT----------------------------
----

AATGATACTGAGACCTTTAGACCTATAGGAGGAAATATGAAGGACAATTGGAGAAGTGAATTATACAAATATA
AAGTAGTAAGAATTGAACCAATA---GGAGTAGCACCCACCAAGGCAAAGAGAAGGGTGGTG-----------
----------------------------CAGAGAGAAAAAAGAGCA---GTAACA---CTAGGAGCT------
---ATGTTCCTTGGGTTCTTGGGAGCA---GCAGGAAGCACTATG---GGCGCAGCGTCA---
CTGACGCTGACGGTACAG---GCCAGACAA---TTATTG---TCTGGTATAGTGCAACAGCAAAAC---
AATTTG---CTGAGA---GCTATTGAGGCGCAACAACATCTGTTGCAACTCACAGTCTGGGGCATC
>MK169545
TTCTACTGTAATACAACACTATTGTTTAATAGTACTTGGTCTAATAGTACTGTGGAAGGGTCAAATAAC----
----

ACTGAAGAAAATATCACACTCCCATGCAGAATAAAACAAATTATAAACATGTGGCAGGGAGTAGGAAAAGCAA
TGTATGCCCCTCCCATCAGAGGACAAATTAGATGTTCATCAAAT---
ATTACAGGGCTGTTATTAACACGAGATGGTGGTAATAATAGTGCT----------------------------
----

GATACTGAGATCTTTAGACCTGGAGGAGGAGATATGAGGGACAATTGGAGAAGTGAATTATATAAATATAAAG
TAGTAAAAATTGAACCATTA---GGAGTAGCACCCACCAAGGCAAAGAGAAGAGTGGTG--------------
-------------------------CAGAGAGAAAAAAGAGCA---GTGGGAACACTAGGAGCT---------
ATGTTCCTTGGGTTCTTGGGAGCA---GCAGGAAGCACTATG---GGCGCAGCGTCA---
CTGACGCTGACGGTACAG---GCCAGACTA---TTATTG---TCTGGTATAGTGCAACAGCAAAAC---
AATTTG---CTGAGG---GCTATTGAGGCGCAACAACATCTGTTGCAACTCACAGTCTGGGGCATC
>MK169677
TTCTACTGTAATACAACATCACTGTTTAATAGTACTTGGGATAATAGTACAAATAGTACTGGA----------
----

GAGGGAGAAGATATCACACTCCCATGCAGACTAAAACAGTTTATAAACATGTGGCAGAAAGTAGGAAAAGCAA
TGTATGCCCCTCCCATCAGAGGACAAATTAAATGTTCATCAAAT---
ATTACAGGGCTGCTATTAACAAGAGATGGTGGTGGAGGTAATAGCACT-------------------------

----

ACCACAAACGAGACCTTCAGACCAGGAGGGGGAGATATGAGAGATAATTGGAGAAGTGAATTATATAAATATA
AAGTAGTAAAAATTGAACCATTA---GGAATAGCACCCACCAAGGCAAAGAGGAGAGTGGTG------------
----------------------------CAGAGAGAAAAAAGAGCA---GTGGGA---ATAGGAGCT-------
---TTGTTCCTTGGGTTCTTGGGAGCA---GCAGGAAGCACTATG---GGCGCAGCGTCA---
GTAACGCTGACGGTACAG---GCCAGACTA---TTATTG---TCTGGTATAGTGCAACAGCAGAAC---
AATTTG---CTGAGG---GCTATTGAGGCGCAACAGCATCTGTTGCAACTCACAGTCTGGGGCATC
>MN090376
TTCTACTGTAATACAACACAACTGTTTAATAGTACTTGGAATAGTACTACAGGAGTAAATGGTACAGGAGCAA
ATAACACA--

GAAGTAAATATTATCACACTCCCATGCAAGATAAGGCAAATTGTAAACATGTGGCAGGAAGTAGGAAAAGCAA
TGTATGCCCCTCCCATCAGTGGACAAATTAGTTGTTCATCAAAT---
ATTACAGGGCTACTACTACTAAGAGATGGTGGTAATAGCAATGAC-----------------------------
----

ACGGATAACACGACTGAAATCTTCAGACCTGGAGGAGGAGATATGAGGGACAATTGGAGAAGTGAATTATATA
AATATAAAGTAGTACAAATTGAACCATTA---GGAGTAGCACCCACCAAGGCAAGGAGAAGAGTGGTG------
----------------------------------CAGAGAGAAAAAAGAGCA---GTGGGA---
ATAGGAGCT---------CTGTTCCTTGGGTTCTTGGGAGCA---GCAGGAAGCACTATG---
GGCGCAGCATCA---ATAACGCTGACGGTACAA---GCCAGACAT---TTATTG---
TCTGGTATAGTGCAACAGCAGAAC---AATTTG---CTGAGA---
GCTATTGAGGCGCAACAGCATATGTTGCAGCTCACAGTCTGGGGCATC
>MN090399
TTCTACTGTAATACAACACAACTGTTTAATAGTACTTGGAATAGTACTACAGGAGTAAATGGTACAGGAGCAA
ATAACACA--

GAAGTAAATATTATCACACTCCCATGCAAGATAAGGCAAATTGTAAACATGTGGCAGGAAGTAGGAAAAGCAA
TGTATGCCCCTCCCATCAGTGGACAGATTAGTTGTTCATCAAAT---
ATTACAGGGCTACTACTACTAAGAGATGGTGGTAATAGCAATGAC-----------------------------
----

ACGAATGACACGGCTGAAATCTTCAGACCTGGAGGAGGAGATATGAGGGACAATTGGAGAAGTGAATTATATA
AATATAAAGTAGTACAAATTGAACCATTA---GGAGTAGCACCCACCAAGGCAAGGAGAAGAGTGGTG------
----------------------------------CAGAGAGAAAAAAGAGCA---GTGGGA---
ATAGGAGCT---------CTGTTCCTTGGGTTCTTGGGAGCA---GCAGGAAGCACTATG---
GGCGCAGCATCA---ATAACGCTGACGGTACAA---GCCAGACAC---TTATTG---
TCTGGTATAGTGCAACAGCAGAAC---AATTTG---CTGAGA---
GCTATTGAGGCGCAACAGCATATGTTGCAGCTCACAGTCTGGGGCATC
>AY835781
TTCTACTGTAATTCATCACAACTGTTTAATAGTACTTGGAATGATAGTACTTGGAATGATACTAAAGGGTTAA
AT--

AACACTGAAGGAATTATCACACTCCCATGCAGAATAAAACAATTTATAAACATGTGGCAGGAAGTAGGAAAAG
CAATGTATGCCCCTCCCATCAGAGGACAAATTAGATGTTCATCAAAT---
ATTACAGGGCTGCTCTTAACAAGAGATGGTGGTAATAGCGAGAAC-----------------------------
----

GATACCACCGAGATCTTCAGACCTGGAGGAGGAGATATGAGGGACAATTGGAGAAGTGAATTATATAAATATA
AAGTAATAAAAATTGAACCATTA---GGAGTAGCACCCACCAAGGCAAAGAGAAGAGTGGTG------------
----------------------------CAGAGAGAAAAAAGAGCA---GTGGGAACGCTAGGAGCT-------
---GTGTTCCTTGGGTTCTTGGGAGCA---GCAGGAAGCACCATG---GGCGCAGCGTCA---
ATGACGCTGACGGTACAG---GCCAGACAA---TTATTG---TCTGGTATAGTGCAACAGCAGAAC---
AATTTG---CTGAGG---GCTATTGAGGCGCAACAGCATCTGTTGCAACTCACAGTCTGGGGCATC
>M17451
TTCTACTGTAATACAACACAACTGTTTAATAGTACTTGGAATAGTACTGAAGGGTCAAATAACACTGGA-----
----

GGAAATGACACAATCACACTCCCATGCAGAATAAAACAAATTGTAAACATGTGGCAGGAAGTAGGAAAAGCAA

TGTATGCCCCTCCCATCAGTGGACAAATTAAATGTATATCAAAT---
ATTACAGGGCTACTATTAACAAGAGATGGGGGTGAAGATACAACT----------------------------
----

AATACTACAGAGATCTTCAGACTTGGAGGAGGAAATATGAGGGACAATTGGAGAAGTGAATTATATAAATATA
AAGTGGTAAGAATTGAGCCATTA---GGAGTGGCACCCACTAGGGCAAAGAGAAGAGTGGTG-----------
----------------------------CAAAGAGAAAAAAGAGCA---GTGGGAACAATAGGAGCT------
---ATGTTCCTTGGGTTCTTGGGAGCA---GCAGGAAGCACTATG---GGCGCAGGCTCA---
ATAACGCTAACGGTACAG---GCCAGACAC---TTATTG---TCTGGTATAGTGCAACAGCAAAAC---
AATTTG---CTGAGG---GCTATTGAGGCGCAACAGCATCTGTTGCAACTCACGGTCTGGGGCATC
>AY835779
TTCTACTGTAATTCAACACAACTGTTTAATAGTACTTGGAATGTTACTGGAGGGACAAATGGCACTGAAGGA-
----

AGTAACCCAAATATCACACTCCCATGCAGAATAAAACAAATTATAAACATGTGGCAGGAAGTAGGAAAAGCAA
TGTATGCCCCTCCCATCAGTGGACAAATTAGATGTTCATCAAAT---
ATTACAGGACTGCTATTAACAAGAGATGGTGGTAGTAACAAGAGC----------------------------
----

GATACCGAGATCTTCAGACCTGGAGGAGGAGATATGAGGGACAATTGGAGAAGTGAATTATATAAATATAAAG
TAGTAAAAATTGAACCATTA---GGAGTAGCACCCACCAAGGCAAAGAGAAGAGTGGTG--------------
-------------------------CAGAGAGAAAAAAGAGCA---GCGGGA---CTAGGAGCT---------
TTGTTCCTTGGGTTCTTGGGAGCA---GCAGGAAGCACTATG---GGCGCAGCGGCA---
GTAACGCTGACGGTACAG---GCCAGACTA---TTATTG---TCTGGTATAGTGCAACAGCAGAAC---
AATTTG---CTGAGG---GCTATTGAGGCGCAACAGCATCTGTTGCAACTAACAGTCTGGGGCATC
>M38431
TTCTACTGTAAGACAACACAGCTGTTTAATAGTACTTGGCTGTTTAATAGTACTTGGAATGATACTGAAAGGT
CA---

GATAACAATGAAACTATCATACTCCCATGCAGAATAAAACAAATTATAAACAGGTGGCAGGAAGTAGGAAAAG
CAATGTATGCCCCTCCCATCAGTGGACAAATTAGATGCTCATCAAAT---
ATTACAGGGCTGCTATTAACAAGAGATGGTGGTGATAAAGAGAAC----------------------------
----

AGTACCACCGAGATCTTCAGACCTGGAGGAGGAGATATGAGGGACAATTGGAGAAGTGAATTATATAAATATA
AAGTAGTAAAAATTGAGCCATTA---GGAGTAGCACCCACCAAGGCAAAGAGAAGAGTGGTG-----------
----------------------------CAGAGAGAAAAAAGAGCA---GTGGGAGCGCTAGGAGCT------
---CTGTTCCTTGGGTTCTTGGGAGCA---GCAGGAAGCACTATG---GGCGCAGCGTCA---
ATGGCGCTGACGGTACAG---ACCAGACAA---TTAATG---TCTGGTATAGTGCAACAGCAGAAC---
AATTTG---CTGAAG---GCTATTGAGGCGCAACAGCATCTGTTGCAACTCACAGTCTGGGGCATC
>U90935
TTCTACTGTAATACAACAAAACTGTTTAATAGTACTTGGATGTTTAATAATACTTGGAATGATACTGAAGACA
CT---

GAAGGAAATGGCGCAATCACACTCCCATGCAGAATAAAACAGATTATAAACAGGTGGCAGGAAGTAGAAAAAG
CAATGTATGCCCCTCCCATCAGAGGACAAATTAGATGTTCATCAAAC---
ATTACAGGGATACTATTAACAAGAGATGGTGGTACTAACAATAGT----------------------------
----

ACCAACGAGACCTTCAGACCTGGAGGAGGAGATATGAGGGACAATTGGAGAAGTGAATTATATAAATATAAAG
TAGTAAAAATTGAACCATTA---GGAGTAGCACCCACCAAGGCAAAGAGAAGAGTGGTG--------------
-------------------------CAGAGAGAAAAAAGAGCA---GTGGGA---ATAGGAGCT---------
TTGTTCCTTGGGTTCTTGGGAGCA---GCAGGAAGCACTATG---GGCGCAGCGGCA---
GTGACGCTGACGGTACAG---GCCAGACAA---TTATTG---TCTGGTATAGTGCAACAGCAGAAC---
AACTTG---CTGAGG---GCTATTGAGGCGCAACAGCATCTGTTGCAACTCACAGTCTGGGGCATC
>M17450
TTCTACTGTAATTCAACACAACTGTTTAGTAGTACTTGGAATGGTACTGAAGGGTCAAATAACACTGGA----
----

GGAAATGACACAATCACCCTCCCATGCAGAATAAAAGAAATTATAAACATGTGGCAGGAAGTAGGAAAAGCAA

TGTATGCCCCTCCCATCAAAGGACAAGTTAAATGTTCATCAAAT---
ATTACAGGGCTGCTATTAACAAGAGATGGTGGTAATAGCAAGAATGGTAGC---------------------
----

AAGAATGAGAACACAGAGATCTTTAGACCGGGAGGAGGAGATATGAGGGACAATTGGAGAAGTGAATTATATA
AATATAAAGTAGTAAAAATTGAACCATTA---GGAGTAGCACCCACCAAGGCAAAGAGAAGAGTGGTG-----
-----------------------------------CAGAGAGAAAAAAGAGCA---
GTGGGAACGATAGGAGCT---------ATGTTCCTTGGGTTCTTGGGAGCA---GCAGGAAGCACTATG---
GGCGCTACGTCA---ATGACGCTGACGGTACAG---GCCAGACTA---TTATTG---
TCTGGTATAGTGCAACAGCAGAAC---AATTTG---CTGAGG---
GCTATTGAGGCGCAACAGCATCTGTTGCAACTCACAGTCTGGGGCATC
>AY352275
TTCTACTGTAATACAACAAAACTGTTTAATAGTACTTGGAATGAAAATAGTACTTGGAATGCTACT-------
----

GGAAATGACACTATCACACTCCCATGTAGAATAAAACAAATTATAAACATGTGGCAGGAAGTAGGAAAAGCAA
TGTATGCCCCTCCCATCGAAGGACAAATTAGATGTTCATCAAAT---
ATTACAGGGCTGCTATTAACAAGAGATGGTGGTGGTGACAAGAAC----------------------------
----

AGTACCACCGAGATCTTTAGACCTGCAGGAGGAAATATGAAGGACAATTGGAGAAGTGAATTATATAAATATA
AAGTAGTAAAAATTGAACCATTA---GGAGTAGCACCCACCAAGGCAAAGAGAAGAGTGGTG-----------
-----------------------------CAAAGAGAAAAAAGAGCA---GTGGGAGTGATAGGAGCT------
---ATGTTCCTTGGGTTCTTGGGAGCA---GCAGGAAGCACTATG---GGCGCAGCGTCA---
ATAACGCTGACGGTACAG---GCCAGAAAA---CTATTG---TCTGGTATAGTGCAACAGCAGAAC---
AATCTG---CTGAGA---GCTATTGAGGCGCAACAGCATCTGTTGCAACTCACAGTCTGGGGCATC
>AY835769
TTCTACTGTGATTCAACACAACTGTTTAACAGTACTTGGCATGGTAATGGTACTTGGAATGGTACTGAA----
----

GGAAATGGCACACTCACACTCCCATGCAGAATAAAACAAATTATAAACATGTGGCAGGAAGTAGGAAAAGCAA
TGTATGCCCCTCCCATCAGAGGACAAATTAGATGTTCATCAAAT---
ATTACAGGGCTGCTATTAACAAGAGATGGTGGTAGGAACGAGAGC----------------------------
----

AATATCACCCTCAGACCTGGAGGAGGGGATATGAGGGACAATTGGAGAAGTGAATTATATAAATATAAAGTAG
TAAAAATTGAACCATTA---GGAGTAGCACCCACTAAGGCAAAGAGAAGAGTGGTG-----------------
----------------------CAGAGAGAAAAAAGAGCA---GCGGGA---TTAGGAGCT---------
ATGTTCCTTGGGTTCTTGGGAGCA---GCAGGAAGCACTATG---GGCGCAGCGTCA---
ATAACGCTGACGGTACAG---GCCAGACTA---TTATTG---TCTGGTATAGTGCAACAGCAGAAT---
AATTTG---CTGAGG---GCTATTGAGGCGCAACAGCATATGTTGAAACTCACAGTCTGGGGCATC
>M38430
TTCTACTGTAATTCAACACAACTATTTAATAGTACTTGGAATAGTACTGAAGGGCCTAAAGGGTCAAATAAC-
----

ACTGAAGAAAATATCACACTCCCATGCAGAATAAAACAAATTGTAAACATGTGGCAGGAAGTAGGAAAAGCAA
TGTATGCCCCTCCCATCGAAGGACAAATTAGATGCTCATCAAAT---
ATTACAGGGCTGCTATTAACAAGAGATGGTGGTAATAGCAGCAAC----------------------------
----

AACACCGAGATCTTCAGACCTGGAGGAGGAGATATGAGGGACAATTGGAGAAGTGAATTATATAAATATAAAG
TAGTAAAAATTGAACCATTA---GGAGTAGCACCCACCAGGGCAAAGAGAAGAGTGGTG--------------
--------------------------CAGAGAGAAAAAAGAGCA---GTGGGA---CTAGGAGCT---------
TTGTTCCTTGGGTTCTTGGGAGCA---GCAGGAAGCACTATG---GGCGCACGGTCA---
ATGACGCTGACGGTACAG---GCCAGACAA---TTATTG---TCTGGTATAGTGCAACAGCAGAAC---
AATTTG---CTGAGG---GCTATTGAGGCGCAACAGCATCTGTTGCAACTCACAGTCTGGGGCATC
>AB221005
TTCTACTGTAATTCAACACAACTGTTTAATAGTACTTGGAATGTTACTGAAGAGTCAAATAACACTGTA----
----

GAAAATAACACAATCACACTCCCATGCAGAATAAAACAAATTATAAACATGTGGCAGGAAGTAGGAAGAGCAA
TGTATGCCCCTCCCATCAGAGGACAAATTAGATGTTCATCAAAT---
ATTACAGGGCTGCTATTAACAAGAGATGGTGGTCCTGAGGACAAC----------------------------
----

AAGACCGAGGTCTTCAGACCTGGAGGAGGAGATATGAGGGATAATTGGAGAAGTGAATTATATAAATATAAAG
TAGTAAAAATTGAACCATTA---GGAGTAGCACCCACCAAGGCAAAGAGAAGAGTGGTG--------------
-------------------------CAGAGAGAAAAAAGAGCA---GTGGGA---ATAGGAGCT---------
GTGTTCCTTGGGTTCTTGGGAGCA---GCAGGAAGCACTATG---GGCGCAGCGTCA---
ATGACGCTGACGGTACAG---GCCAGACTA---TTATTG---TCTGGTATAGTGCAACAGCAGAAC---
AATCTG---CTGAGA---GCTATTGAGGCGCAACAGCATCTGTTGCAACTCACAGTCTGGGGCATC
>AY357406
TTCTACTGTAAGTCAACAAAACTGTTTAATAGTACTTGGACTAGGAATAATGGTACTTGGACTCGGAATGATA
CTGAAAGG---
-------------TCAAAT------
AGCACTGAAGAACATATCACACTCCCATGCAGAATAAAACAAATTATAAACATGTGGCAGGAAGTAGGAAAAG
CAATGTATGCCCCTCCCATCAGAGGACAAATTAGATGTTCATCAAAT---
ATTACAGGGCTGCTATTAACAAGAGATGGTGGTAATGACACGAGC----------------------------
----

GGGACTGAGACCTTCAGACCTGGAGGAGGAGATATGAGGGACAATTGGAGAAGTGAATTATATAAATATAAAG
TAGTAAGAATTGTACCATTA---GGAGTAGCACCCACCGAGGCAAAGAGAAGAGTGGTG--------------
-------------------------CAGAGAGAAAAAAGAGCA---GTGGGA---ATAGGAGCT---------
GTGTTCCTTGGGTTCTTGGGAGCA---GCAGGAAGCACTATG---GGCGCAGCGGCA---
GTGACGCTGACGGTACAG---GCCAGACTA---TTATTG---TCTGGTATAGTGCAACAGCAGAAC---
AATTTG---CTGAGG---GCTATTGAGGCGCAACAGCATCTGTTGCAACTCACAGTCTGGGGCATC
>AF025758
CTCTACTGTAATACAACACAACTGTTTAATAGTACTTGGAATGCTAATGGGACTTGGAATGATACAGGAGGGT
CAAAT--

AGCACTGAAGGAATTATCTCACTCCCATGCAGAATAAAACAAATTATAAACATGTGGCAGGAAGTAGGAAAAG
CAATGTATGCCCCTCCCATCGAAGGACAAATTAGATGTTCATCAAAT---
ATTACAGGGCTATTATTAACAAGAGATGGTGGTAGTAGCCAAAACAAG-------------------------
----

AGCGAGACCGAGATCTTCAGACCTGGAGGAGGAGATATGAGGGACAATTGGAGAAGTGCATTATATAAATACA
AAGTAGTAAAAATTGAGCCATTA---GGAGTAGCACCCACTAAGGCAAAGAGAAGAGTGGTG-----------
----------------------------CAGAGAGAAAAAAGAGCA---GTGGGA---ATAGGAGCT------
---GTACTCCTTGGGTTCTTGGGAGCA---GCAGGAAGCACTATG---GGCGCAGCGTCA---
TTGACGCTGACGGTACAG---GCCAGACTA---TTATTG---TCTGGTATAGTGCAACAGCAGAAC---
AATTTG---CTGAGG---GCTATTGAGGCGCAACAGCATCTGTTGCAACTCACAGTCTGGGGCATC
>AF025764
TTCTACTGTAATTCAACACAACTGTTTAATAGTACTTGGCCATTTAATAGTACTTTGAATGATACTAGCACTG
AA---

GGAAATGCCACAATCACACTCCCATGCCGAATAAAACAAGTTGTAAACACGTGGCAGGAAGTAGGAAAAGCAA
TGTATGCCCCTCCCATCAGAGGACAAATTAGATGTTCATCAAAT---
ATTACAGGGCTGCTATTAACAAGAGATGGAGGGACTAGCGAGAACGGG-------------------------
----

ACGAACGAGATTGAGATCTTCAGACCTGGAGGAGGAGATATGAGGGACAATTGGAGAAGTGAATTATATAAAT
ATAAAGTAGTAAAAATTGAACCATTA---GGAGTAGCACCCACCAAGGCAAAGAGAAGAGTGGTC--------
-------------------------------CAAAGAGAAAAAAGAGCA---GTGGGAACAATAGGAGCT---
------ATGTTCCTTGGGTTCTTGGGAGCA---GCAGGAAGCACTATG---GGCGCAGCGTCA---
ATAACGCTGACGGTACAG---GCCAGACTA---TTATTG---TCTGGTATAGTGCAGCAGCAGAAC---
AATCTG---CTGAGG---GCTATTGAGGCGCAACAGCATCTGTTGCAACTCACAGTCTGGGGCATC
>AF025751
TTCTACTGTAATTCAACACAACTGTTTAATAGTACTTGGAATGTTACTAGAGAGTCAAATAACAGTACTGAA-
----

GGAAATGGGACTATCACACTCCCATGCAGAATAAAACAAATTATAAACATGTGGCAGGGAGTAGGAAAAGCAA
TGTACGCCCCTCCCATCAGAGGACAAATTAGATGTTCATCAAAT---
ATTACAGGGCTGCTATTAACAAGGGATGGTGGTAATAACGAGAGC----------------------------
----

AATGAGACCGAGACCTTCAGACCTGGAGGAGGAGATATGAGGGACAATTGGAGAAGTGAATTATATAAATATA
AAGTAGTAAAAATTGAACCATTA---GGAGTAGCACCCACCAAGGCAAAAAGAAGAGTGGTG-----------
----------------------------CAGAGAGAAAAAAGAGCA---GTGGGA---ATAGGAGCT------
---GTGTTCCTTGGGTTCTTGGGAGCA---GCAGGAAGCACTATG---GGCGCAGCGTCA---
ATGACGCTGACGGTACAG---GCCAGACTA---TTATTG---TCTGGTATAGTGCAACAGCAGAAC---
AATTTG---CTGAGG---GCTATTGAGGCGCAACAGCATCTGTTGCAACTCACAGTCTGGGGCATC
>U69584
TTCTACTGTAATACATCACAACTGTTTAATAGTACTTGGAATAGTACTGGGAATAATATTAGCGAGTCAGATA
AC---

ACTGAAAGAAATATCACACTCCCATGCAGAATAAAACAAATTATAAACCTGTGGCAGGAAGTAGGAAAAGCAA
TGTATGCCCCTCCCATCAGAGGACAAATTAGATGTTCATCAAAT---
ATTACAGGGCTGCTATTAACAAGAGATGGTGGTAGTAACACGGACGAG-------------------------
----

AACAGGACCGAGATCTTCAGACCTGGAGGAGGAGATATGAGGGACAATTGGAGAAGTGAATTATATAAATATA
AAGTAGTAAAAATTGAACCATTA---GGAGTAGCACCCACCAAGGCAAAGAGAAGAGTGGTG-----------
----------------------------CAGAGAGAAAAAAGAGCA---GTGGGAACAATAGGAGCT------
---ATGTTCCTTGGGTTCTTGGGAGCA---GCAGGAAGCACTATG---GGCGCAGCGTCG---
ATGACGCTGACGGTACAG---GCCAGACTA---TTATTG---TCTGGTATAGTGCAACAGCAGAAC---
AATTTG---CTGAGG---GCTATTGAGGCGCAACAGCATCTGTTGCAACTCACAGTCTGGGGCATC
>AY835778
TTCTACTGTAATTCAAGAGAGCTGTTTAATAGTACTTGGAATGATACTGAAGGGTGGAATAATACTGACAGG-
----

TCAAATAAAACTATCACACTCCCATGCAAAATAAAACAAATTATAAACAGATGGCAGGAAGTAGGAAAAGCAA
TGTATGCCCCTCCCATCAGAGGACAAATTAGATGTACATCAAAT---
ATTACAGGGCTGCTACTAACAAGAGATGGTGGTAGGAACAATAGC----------------------------
----

GAAAACGAGACCTTCAGACCTGGAGGAGACGATATGAGGGACAATTGGAGAAGTGAATTATATAAATATAAAG
TAGTAAAAATTGGACCATTG---GGAGTAGCACCCACCAAGGCAAAGAGAAGAGTGGTG--------------
-------------------------CAGAGAGAAAAAAGAGCA---GTGGGAGTAATAGGAGCT---------
ATGTTCCTTGGGTTCTTGGGAGCA---GCAGGAAGCACTATG---GGCGCAGCGTCA---
ATGACGCTGACGGTACAA---GCCAGACAA---TTATTG---TCTGGTATAGTGCAACAGCAGAAC---
AATCTG---CTGAGG---GCTATTGAGGCGCAACAGCATCTGTTGCGACTCACAGTCTGGGGCATC
>AY835775
TTCTACTGTAATTCAACACAACTATTTAATAGTACTTGGAATGGTACTGAGAGGTCATATAACACTTCA----
----

GAAAATATCAATATCACACTCCCATGCAGAATAAAACAAATTGTAAACATGTGGCAGGAAGTAGGAAAAGCAA
TGTATGCTCCTCCCATCAGTGGACAAATTAGATGTTCATCAAAT---
ATTACAGGGCTGCTATTAACAAGAGATGGTGGTAAGAAACAGAACGAGACC----------------------
----

AAGAACGAGACCGAGATCTTTAGACCTGGAGGAGGAGATATGAGGGACAATTGGAGAAGTGAATTATATAGAT
ATAAAGTAGTAAAAATTGAACCATTA---GGAGTAGCACCCATCAAGGCAAAGAGAAGAGTGGTG--------
-------------------------------CAGAGAGAAAAAAGAGCA---GCGGGA---ATAGGAGCT---
------TTGTTCCTTGGGTTCTTGGGAGCA---GCAGGAAGCACTATG---GGCGCAGCGTCA---
ATAACGCTGACGGTACAG---GCCAGACTA---TTATTG---TCTGGTATAGTGCAACAGCAGAAC---
AATTTG---CTGAGG---GCTATTGAGGCGCAACAGCATCTGTTGCAACTCACAGTCTGGGGCATC
>AY835749
TTCTACTGTAATACAACACAACTATTTAATAGTACTTGGAATGTTAATAGTACTTGGAGTAGTACTGAAGGGT
CAAATAAC---
-------------------ACT---

GAAAGAAATGACACACTCACACTCCCATGCAGAATAAAACAAATTATAAACATGTGGCAGGAAGTAGGAAAAG
CAATGTATGCCCCTCCCATCAGCGGACAAATTAGTTGTTCATCAAAT---
ATTACAGGGCTGATATTAACAAGAGATGGTGATAAAAACAACAAT----------------------------
----

GAGACCGAGATCTTCAGACCTGTAGGAGGAGATATGAGGGACAATTGGAGAAGTGAATTATATAAATATAAAG
TAGTAAAGATTGAACCATTA---GGAGTAGCACCCACTAGGGCAAAGAGAAGAGTGGTG--------------
-------------------------CAGAGAGAAAAAAGAGTAGCAGTGGGA---CTAGGAGCT---------
GTGTTCCTTGGGTTCTTGGGAGCA---GCAGGAAGCACTATG---GGCGCAGCGGCA---
GTGACGCTGACGGTACAG---GCCAGACTA---TTATTG---TCTGGTATAGTGCAACAGCAGAAC---
AATTTG---CTGAGA---GCTATTGAGGCGCAACAGCATCTGTTGCAACTCACAGTCTGGGGCATC
>AY426119
TTCTACTGTAATTCAACACAACTGTTTAATAGTACTTGGAATTTTAATGGTACTTGGAATTTAACACAATCGA
ATGGT--
-------------------ACT---
GAAGGAAATGACACTATCACACTCCCATGTAGAATAAAACAAATTATAAATATGTGGCAGGAAGTAGGAAAAG
CAATGTATGCCCCTCCCATCAGAGGACAAATTAGATGCTCATCAAAT---
ATTACAGGGCTAATATTAACAAGAGATGGTGGAACTAACAGTAGT----------------------------
----

GGGTCCGAGATCTTCAGACCTGGGGGAGGAGATATGAGGGACAATTGGAGAAGTGAATTATATAAATATAAAG
TAGTAAAAATTGAACCATTA---GGAGTAGCACCCACCAAGGCAAAAAGAAGAGTGGTG--------------
-------------------------CAGAGAGAAAAAAGAGCA---GTGGGAACGATAGGAGCT---------
ATGTTCCTTGGGTTCTTGGGAGCA---GCAGGAAGCACTATG---GGCGCAGCGTCA---
ATAACGCTGACGGTACAG---GCCAGACTA---TTATTG---TCTGGTATAGTGCAACAGCAGAAC---
AATTTG---CTGAGG---GCTATTGAGGCGCAACAGCATCTGTTGCAACTCACAGTCTGGGGCATC
>AY426125
TTCTACTGTAATTCAACACAACTGTTTAATAGTACTTGGAATGATACTGAAAAGTCAAGTGGCACTGAA----
----

GGAAATGACACCATCATACTCCCATGCAGAATAAAACAAATTATAAACATGTGGCAGGAAGTGGGAAAAGCAA
TGTATGCTCCTCCCATTAAAGGACAAATTAGATGTTCATCAAAT---
ATTACAGGGCTGCTATTAACAAGAGATGGTGGTAAAAATGAGAGT----------------------------
----

GAGATCGAGATCTTCAGACCTGGAGGAGGAGACATGAGGGACAATTGGAGAAGTGAATTATATAAATATAAAG
TAGTAAAAATTGAACCATTA---GGAGTAGCACCCACCAAGGCAAAGAGAAGAGTGGTG--------------
-------------------------CAAAGAGAAAAAAGAGCA---GTGGGA---ATAGGAGCT---------
TTGTTCCTTGGGTTCTTGGGAGCA---GCAGGAAGCACTATG---GGCGCAGCGTCA---
ATGACACTGACGGTACAG---GCCAGACAA---TTATTG---TCTGGTATAGTGCAACAGCAAAAC---
AATTTG---CTGAGG---GCTATTGAGGCGCAACAGCATATGTTGCAACTCACAGTCTGGGGCATC
>AF025759
TTCTACTGTAATTCAACACAGCTATTTAATAGTACTTGGCTGTTTAATAGTACTGGGAATGATACTACAGGGT
CA---

AATAACACGGAACCTATCACACTCCCATGCAGAATAAAACAAATTATAAATATGTGGCAAAAAGTAGGAAAAG
CAATGTATGCCCCTCCCATCAGAGGACAAATTAGATGTTCATCAAAT---
ATTACAGGGCTGCTATTAACAAGAGATGGTGGTCTATTAACAAGAAATGACATC-------------------
----

AAAGAGAACGAGACTGAGATCTTCAGACCTGGAGGAGGAGATATGAGGGACAATTGGAGAAGTGAATTATATA
AATATAAAGTAGTAAAAATTGAGCCATTA---GGAGTAGCACCCACCAAGGCAATGAGAAGAGTGGTG-----
----------------------------------CAGAGAGAAAAAAGAGCA---
GTGGGAACGATAGGAGCT---------ATGTTCCTTGGGTTCTTGGGAGCA---GCAGGAAGCACTATG---
GGCGCAGCGTCG---ATGACGCTGACGGTACAG---GCCAGACTA---TTATTG---
TCTGGTATAGTGCAACAGCAGAAC---AATTTG---CTGAGG---
GCTATTGAGGCGCAACAGCATCTGTTGCAACTCACAGTCTGGGGCATC
>AF025761
TTCTACTGTAATACAACACAACTGTTTAATAGTACTTGGAATAGTACTGGGGATGAAGGGTCAAATAACACTG
AAGGA--

AATAACACACTCCCAATCATACTTCCATGCAGAATAAAACAAATTATAAACATGTGGCAGGGAGTAGGAAAAG
CAATGTATGCCCCTCCCATCGGAGGACAAATTAGATGTTCATCAAAT---
ATTACAGGGCTGCTATTAACAAGAGATGGTGGTAAGAGTCCAAAC----------------------------
----

GATACCGAGACCTTCAGACCTGCAGGAGGAGATATGAGGGACAATTGGAGAAGTGAATTATATAAATATAAAG
TAGTAAAAATTGAACCATTA---GAAGTAGCACCCACTAGGGCAAAGAGAAGAGTGGTG--------------
-------------------------CAAAGAGAAAAAAGAGCA---GTAGGA---ATAGGAGCT---------
ATGTTCCTTGGGTTCTTGGGAGCA---GCAGGAAGCACTATG---GGCGCAGCGTCG---
ATAACGCTGACGGTACAG---GCCAGACTA---TTATTG---TCTGGTATAGTGCAACAGCAGAAC---
AATCTG---CTGAGG---GCTATTGAGGCGCAACAGCATCTGTTGCAACTCACAGTCTGGGGCATC
>AF025749
TTCTACTGTAATACAGCAAAACTATTTAATAGTACTTGGAATAGTACTTGGAGTTCAAATAATACTGAAGGAA
AT---

AGCACTGAAGAAATTATCATACTCCCATGTAGAATAAAACAAATTATAAACAGGTGGCAGGAAGTAGGAAAAG
CAATGTATGCCCCTCCCATCAGAGGACAAATCAGATGTTCATCAAAT---
ATTACAGGGCTGCTATTAACAAGAGATGGTGGTACTAATGGGAAGAAC-------------------------
----

GAGACCCCCGAGATCTTCAGACCTGGAGGAGGAGATATGAGGGACAATTGGAGAAGTGAATTATATAAATATA
AAGTAGTAGAAATTGAACCATTA---GGAGTAGCACCCACCAGGGCAAGGAGAAGAGTGGTG-----------
----------------------------CAGAGAGAAAAAAGAGCA---GTGGGAACAATAGGAGCT------
---ATGTTCCTTGGGTTCTTGGGAGCA---GCAGGAAGCACTATG---GGCGCAGCGTCA---
ATGACGCTGACGGTACAG---GCCAGACTA---TTATTG---TCTGGTATAGTGCAACAGCAGAAC---
AATTTG---CTGAGG---GCTATTGAGGCGCAACAGCATCTGTTGCAACTCACAGTCTGGGGCATC
>AF025750
TTCTACTGTAATTCAGCACAACTGTTTAACAGTACTTGGAATGATACTGAAAGGTCAAATAACACTAGAGAA-
----

AATGACACAACAATCACACTCCCATGCAGAATAAAACAAATTATAAACATGTGGCAGGAAGTAGGAAAAGCAA
TGTATGCCCCTCCTATCAGAGGACAAATTAAATGTGCATCAAAT---
ATCACAGGGCTGCTATTAACAAGAGATGGTGGTAATAACAATACT----------------------------
----

GGGACCACCGAGACCTTCAGACCTGGAGGAGGAGATATGAGGGACAATTGGAGAAGTGAATTATATAAATATA
AAGTAGTAAAAATTGAACCATTA---GGAATAGCACCCACTAAGGCAAAGAGAAGAGTGGTG-----------
----------------------------CAGAGAGAAAAAAGAGCA---GTGGGA---ATAGGAGCT------
---GTGTTCCTTGGGTTCTTGGGAGCA---GCAGGAAGCACTATG---GGCGCAGCGTCA---
GTGACGCTGACGGTACAA---GCCAGACTA---TTATTG---TCTGGTATAGTGCAACAGCAAAAC---
AATCTG---CTGAGG---GCTATTGAGGCGCAACAGCATCTGTTGCAACTCACAGTCTGGGGCATC
>AF025752
CTCTACTGTAATTCAACACAATTGTTTAATAGTACTTGGAAGTTTAATAGTACTTGGAATGATACTGAAGGGT
CAAAT--

AACACTGAAGGAAATATCACACTCCCATGCAGAATAAAACAAATTATAAACATGTGGCAGGAAGTAGGAAAAG
CAATGTATGCCCCTCCCATCAGAGGACAAATTAGATGTTCATCAAAG---
ATTACAGGGCTGTTATTAACAAGAGATGGTGGTACTACCAAAAACGGGAAC----------------------
----

AACAACGATACTGAGATCTTTGGACCTGGAGGAGAAGATATGAAGGACAATTGGAGAAGTGAATTATATAAAT
ATAAAGTAGTAAAAATTGAACCATTA---GGAGTAGCACCCACTAAGGCAAAGAGAAGAGTGGTG--------
-------------------------------CAGAGAGAAAAAAGAGCA---GTGGGA---CTAGGAGCT---
------ATGTTCCTTGGGTTCTTGGGAGCA---GCAGGAAGCACTATG---GGCGCAGCGTCA---
ATGACGCTGACGGTACAG---GCCAGACAA---TTATTG---TCTGGTATAGTGCAACAGCAAAAC---
AATTTG---CTGAGG---GCTATTGATGCGCAACAGCATATGTTGCAACTCACAGTCTGGGGCATC
>AF025756
TTCTACTGTAATTCAACAAAACTGTTTAATAGAACTTGGAGTGTTAATAGTACTTGGAATGATACTGAAGGGG
TAAAT--

AACACTGGAGAAAATATCACACTCCCATGCAGAATAAAACAAATTATAAACATGTGGCAGGAAGTAGGAAAAG
CAATGTATGCCCCTCCCATCAAAGGACAAATTAGATGTTCATCAAAT---
ATTACAGGGCTGCTATTAACAAGAGATGGTGGTAATAGCAACAAC----------------------------
----

GAGACCGAGACTTTCAGACCTGGAGGAGGAGATATGAGGGACAATTGGAGAAGTGAATTATATAAATATAAAG
TAGTAAAAATTGAACCATTA---GGAGTAGCACCTACTAAGGCAAAGAGAAGAGTGGTG--------------
-------------------------CAAAGAGAAAAAAGAGCA---GTAGGA---ATAGGAGCT---------
GTGTTCCTTGGGTTCTTGGGAGCA---GCAGGAAGCACTATG---GGCGCAGCGTCA---
ATGACGCTGACGGTACAG---GCCAGACTA---TTATTG---TCTGGTATAGTGCAACAGCAGAAC---
AATTTG---CTGAGG---GCTATTGAGGCGCAACAGCATCTGTTGCAACTCACAGTCTGGGGCATC
>M93258
TTCTACTGTAATTCAACACAACTGTTTACTTGGAATGATACTAGAAAGTTAAATAAC----------------
----

ACTGGAAGAAATATCACACTCCCATGTAGAATAAAACAAATTATAAATATGTGGCAGGAAGTAGGAAAAGCAA
TGTATGCCCCTCCCATCAGAGGACAAATTAGATGTTCATCAAAT---
ATTACAGGGCTGCTATTAACAAGAGATGGTGGTAAGGACACGAAC----------------------------
----

GGGACTGAGATCTTCAGACCTGGAGGAGGAGATATGAGGGACAATTGGAGAAGTGAATTATATAAATATAAAG
TAGTAAAAATTGAACCATTA---GGAGTAGCACCCACCAAGGCAAAGAGAAGAGTGGTG--------------
-------------------------CAGAGAGAAAAAAGAGCA---GTGGGA---CTAGGAGCT---------
TTGTTCCTTGGGTTCTTGGGAGCA---GCAGGAAGCACTATG---GGCGCAGCGTCA---
ATAACGCTGACGGTACAG---GCCAGACAA---TTATTG---TCTGGTATAGTGCAACAGCAGAAC---
AATCTG---CTGAGG---GCTATTGAGGCGCAACAGCACCTGTTGCAACTCACAGTCTGGGGCATC
>AY835758
TTCTACTGTAACACAACAAAACTGTTTAATAGTACTTGGAATAATAGTACTTGGAATGCTACTGCAGGCCAAG
AATTA--
-------------------AAT---
AGCACTGAAGACACCATCACACTCCCATGCAAAATAAAACAAATTATAAACATGTGGCAGGAAGTAGGAAAAG
CAATGTATGCCCCTCCCATCAAAGGACTACTTAGATGTTCATCAAAT---
ATTACAGGGCTGCTATTAACAAGAGATGGTGGTAATAGCAATAACACG-------------------------
----

AACACGACCGAGATCTTCAGACCTGGGGGAGGAGATATGAGGGACAATTGGAGAAGTGAATTATATAAATATA
AAGTAGTAAAAATTGAACCACTA---GGAATAGCACCCACTAAGGCAAAGAGAAGAGTGGTG-----------
----------------------------CAGAGAGAAAAAAGAGCA---GTGGGA---ATAGGAGCT------
---TTGTTCCTTGGGTTCTTGGGAGCA---GCAGGAAGCACTATG---GGCGCAGCGTCA---
ATGACGCTGACGGTACAG---GCCAGACTA---TTATTG---TCTGGTATAGTGCAACAGCAGAAC---
AATTTG---CTGAGG---GCTATTGAGGCGCAACAGCACCTGTTGCAACTCACAGTCTGGGGCATC
>L02317
TTCTACTGTAAGTCAACACAACTGTTTAATAGTACTTGGGCTGGGAATAATACTTGGAATAGTAGTGCTGAAA
GGTCA--
-------------------GAT---
GACACTGGAGGAAATATCACACTCCCATGCAGAATAAAACAAATTATAAACATGTGGCAGGAAGTAGGAAAAG
CAATGTATGCCCCTCCCATCAGCGGACAAATTAGATGTACATCAAAT---
ATTACAGGGCTGCTATTAACAAGAGATGGTGGTACGAGCGATACC----------------------------
----

AATACCACCGAGACCTTCAGACCTGGAGGAGGAGATATGAGGGACAATTGGAGAAGTGAATTATATAAATATA
AAGTAGTAAAAATTGAACCATTA---GGAGTAGCACCCACCACGGCAAAGAGAAGAGTGGTG-----------
----------------------------CAAAGAGAAAAAAGAGCA---GTGGGA---ATAGGAGCT------
---CTGTTCCTTGGGTTCTTGGGAGCA---GCAGGAAGCACTATG---GGCGCAGCGTCA---
ATGACGTTGACGGTACAG---GCCAGACAA---TTATTG---TCTGGTATAGTGCAACAGCAGAAC---
AATTTG---CTGAGG---GCTATTGAGGCGCAACAGCATCTGTTGCAACTCACAGTCTGGGGCATC
>AF025755
TTCTACTGTAATTCAACAAAACTGTTTAATAGTACTTGGAAGTTTAATAATACTTGGAATATTACTGAAAGGT
CA---

AATAACACTGACACAATCACACTCCCATGTAGAATAAAACAAATTATAAACATGTGGCAGGGAGTAGGAAAAG
CAATGTATGCCCCTCCCATCAGTGGACTAATTAGCTGTTCATCAAAT---
ATTACAGGGCTGCTATTAACAAGAGATGGGGGTAATAACAATGGGACCGAGGGT-------------------
----
-
GATAATAACAACGAGACTGAGATTTTCAGACCTGGAGGAGGGAATATGAAGGACAATTGGAGAAGTGAATTAT
ATAAATATAAAGTAGTAAAAGTTGAACCATTA---GGAATAGCACCCACTAAGGCAAAGAGAAGAGTGGTG--
-------------------------------------CAAAGAGAAAAAGGAGCA---GTGGGA---
ATAGGAGCT---------GTGTTCCTTGGGTTCTTGGGAGCA---GCAGGAAGCACTATG---
GGCGCAGCGTCA---ATAACGCTGACGGTACAG---GCCAGACAA---TTATTG---
TCTGGTATAGTGCAACAGCAGAAC---AATTTG---CTGAGG---
GCTATTGAAGCGCAACAGCATCTCTTGCAACTCACAGTCTGGGGCATC
>AF025757
TTCTACTGTAATTCAACACCGCTGTTTAATAGTACTTGGAATAATAATAATTGGAGTGATCCTAAT-------
----

AACGATGTAAATATCACACTCCCATGCAGAATAAAACAAATTATAAACAGGTGGCAGGAAGTAGGAAAAGCAA
TGTATGCCCCTCCCATCAGTGGCCAAATTAGATGTTCATCAAAT---
ATTACAGGGCTGCTATTAACAAGAGATGGTGGTAATACCACGGAG----------------------------
----

GAGATCTTCAGACCTGGAGGAGGAGATATGAGGGACAATTGGAGAAGTGAATTATGTAAATATAAAGTAGTAA
AAATTGAGCCATTA---GGAGTAGCACCCACCAAGGCAAAGAGAAGAGTGGTG--------------------
-------------------CAGAGAGAAAAAAGAGCA---GTGGGAACGATAGGAGCT---------
ATGTTCCTTGGGTTCTTGGGAGCA---GCAGGAAGCACTATG---GGCGCAGCGTCA---
ATAACGCTGACGGTACAG---GCCAGACTA---TTATTG---TCCGGTATAGTGCAACAGCAGAAC---
AATTTG---CTGAGG---GCTATTGAGGCGCAACAGCATCTGTTGCAACTCACAGTCTGGGGCATC
>AY835763
TTCTACTGTAATTCAACAAAACTGTTTAATAGTACTTGGAAGTTTAATGGTACTACTTGGAATTGGACTACAG
GGTTA--
-------------------AAT---
AAAACTGAAGTAAATGTCACACTCCCATGCAGAATAAAACAAATTATAAACATGTGGCAGGAAGTAGGAAAAG
CAATGTATGCCCCTCCCATCAAAGGAGTAATTAGATGTTCATCAAAT---
ATTACAGGGCTGCTATTAACAAGAGATGGTGGTAGTAACAATGGTAGTAACAAT------------GGG----
----
ACCGGGATTAACAAT---
GAGACCGAGATCTTCAGACCTGGAGGAGGAAATATGAAGGACAATTGGAGAAGTGAATTATATAAATATAAAG
TAGTAAGAATTGAACCATTA---GGAATAGCACCCACCAAGGCAAAGAGAAGAGTGGTG--------------
-------------------------CAGAGAGAGAAAAGAACA---GTGGGA---TTAGGAGCC---------
TTGTTCCTTGGGTTCTTGGGAGCA---GCAGGAAGCACTATG---GGCGCAGCGTCA---
CTGACGCTGACGGTACAG---GCCAGACTA---TTATTG---TCTGGTATAGTGCAACAGCAGAAC---
AATTTG---CTGAGG---GCTATTGAGGCGCAACAGCGTATGTTGCAACTTACAGTCTGGGGCATC
>AF025762
TTCTACTGTAATTCAACACAACTGTTTAATAGTACTTGGACTGAATGGTCAAATAACACTGAAGGAAATATC-
----

ACTAAAGAAAATATTACACTCCCATGCAGAATAAAACAAATTATGAACATGTGCCAGGAAGTAGGAAGAGCAA
TGTATGCCCCTCCCATCAAAGGACAAATTAGATGTTCATCAAAA---
ATTACAGGGCTGCTATTAACAAGAGATGGTGGTACTGGCGGGAAC----------------------------
----

GGGTCCGAGTTCTTCAGACCTGGAGGAGGAGATATAAGGGACAATTGGAGAAGTGAATTATATAAATATAAAG
TAGTAAAAATTGAACCATTA---GGAGTAGCACCCACCAGGGCAAAGAGAAGAGTGGTG--------------
-------------------------CAAAGAGAAAAAAGAGCA---GTGGGA---ATAGGAGCT---------
GTGTTCCTTGGGTTCTTGGGAGCA---GCAGGAAGCACTATG---GGCGCAGCGTCA---
CTGACGCTGACGGTACAG---GCCAGACTA---TTATTG---TCTGGTATAGTGCAACAGCAGAAC---
AATTTG---CTGAGG---GCTATTGAGGCGCAACAGCATCTGTTGCAACTCACAGTCTGGGGCATC
>AF025754

TTCTACTGTAATTCAACACAACTGTTTAGTAGTACTTGGAATAATACTGAATCTGAAGGGGTAAATAACACTG
AA---

GGAAATGACACAATCATACTCCTATGCAGAATAAAACAAATTGTAAACATGTGGCAGGAAGTAGGAAAAGCAA
TGTATGCCCCTCCCATCAGAGGACAAATTAGATGCTCATCAAAT---
ATTACAGGGCTGCTATTAACAAGAGATGGTGGTAATAGTAATGGCAGC-------------------------
----

AACAAGGCCGAGGTCTTCAGACCTGGAGGAGGAGATATGAGGGACAATTGGAGAAGTGAATTATATAAATATA
AAATAGTAAAAATTGAACCATTA---GAAGTAGCACCCACTAAGGCAAAGAGAAGAGTGGTG-----------
----------------------------CAGAGAGAAAAAAGAGCA---GCGGGA---CTAGGAGTT------
---ATGTTCCTTGGGTTCTTGGGAGCA---GCAGGAAGCACTATG---GGCGCAGCGTCA---
ATGACGCTGACGGTACAG---GCCAGACAA---TTATTG---TCTGGTATAGTGCAACAGCAGAAC---
AATTTG---CTGAGG---GCTATTGAGGCGCAACAGCATCTGTTGCAACTCACAGTCTGGGGCATC
>AF286365
TTCTACTGTAATTCAACACAACTGTTTAATAGTACTTGGAATAGTACTGAAGGAAATAGTACTTGG-------
----

TCAGATAAAATTATCAGACTCCCATGCAGAATAAAACAAATTATAAACATGTGGCAGGAAGTAGGAAAGGCAA
TGTATGCCCCTCCCATCGATGGACAAATTAGATGTTCATCAAAT---
ATTACAGGGCTGCTATTAACAAGAGATGGTGGGAATAGCAACGAG----------------------------
----

ACCACCGAGATCTTCAGACCTGGGGGAGGAGATATGAGGGACAATTGGAGAAGTGAATTATACAAATATAAAG
TAGTAAAAATTGAACCATTA---GGAGTAGCACCCACCAAGGCAAAGAGAAGAGTGGTG--------------
-------------------------CAAAGAGAAAAAAGAGCA---GTGGGAGTAATAGGAGCT---------
ATGTTCCTTGGGTTCTTGGGAGCA---GCAGGAAGCACTATG---GGCGCAGCGTCA---
ATGGCGCTGACGGTACAA---GCCAGACAA---TTATTG---TCTGGTATAGTGCAGCAGCAGAAC---
AATCTG---CTGAGA---GCTATTGAGGCGCAACAACATATGTTGCAGCTCACAGTCTGGGGCATC
>U39362
TTCTACTGTAATACAGCACAACTGTTTAATAGTACTTGGAATGTTACTGGAGGGACAAATGGCACTGAA----
----

GGAAATGACATAATCACACTCCAATGCAGAATAAAACAAATTATAAATATGTGGCAGAAAGTAGGAAAAGCAA
TGTATGCCCCTCCCATCACAGGACAAATTAGATGTTCATCAAAT---
ATTACAGGGCTGCTACTAACAAGAGATGGAGGTAATAGTACTGAG----------------------------
----

ACTGAGACTGAGATCTTCAGACCTGGAGGAGGAGATATGAGGGACAATTGGAGAAGTGAATTATATAAATATA
AAGTAGTAAGAATTGAACCAATA---GGAGTAGCACCCACCAGGGCAAAGAGAAGAACAGTG-----------
----------------------------CAAAGAGAAAAAAGAGCA---GTGGGA---ATAGGAGCT------
---GTGTTCCTTGGGTTCTTGGGAGCA---GCAGGAAGCACTATG---GGCGCAGCGTCA---
GTGACGCTGACGGTACAG---GCCAGGCTA---TTATTG---TCTGGTATAGTGCAGCAGCAGAAC---
AATCTG---CTGAGG---GCTATTGAGGCGCAACAGCATATGTTGCAACTCACAGTCTGGGGCATC
>AF128126
TTCTACTGTAATACAACACAACTGTTTGATAGTATTTGGAATAGTGAGAATGGTACTTGGAATATTACTAGGG
GGTTAAAT---
-------------AACACT------
GGAAGAAATGACACAATCACACTCCCATGCAGGATAAAACAAATTATAAACAGGTGGCAGGAAGTAGGAAAAG
CAATGTATGCCCCTCCCATCAAAGGAAACATTAGCTGTTCATCAAAT---
ATTACAGGGCTGCTATTAACAAGAGATGGTGGTAAGGATGATAATAGC-------------------------
----

AGGGACGGGAACGAGACCTTCAGACCTGGAGGAGGAGATATGAGGGACAATTGGAGAAGTGAATTATATAAAT
ATAAAGTAGTAAAAATTGAACCATTA---GGAGTAGCACCCACCAAGGCAAAGAGAAGAGTGGTG--------
-------------------------------CAAAGAGAAGAAAGAGCA---GTGGGA---CTAGGAGCT---
------ATGTTCATTGGGTTCTTGGGAGCA---GCAGGAAGCACTATG---GGCGCAGCGTCA---
GTGACGCTGACGGTACAG---GCCAGGCAA---TTATTG---TCTGGTATAGTGCAACAGCAGAGC---
AATTTG---CTGAGA---GCTATTGAGGCGCAACAGCATCTGTTGCAACTCACAGTCTGGGGCATC
>AF025763

TTCTACTGTAATACAACACAACTATTTAATAGTACTTGGAATGTTGCTGAAGGGTCAAATAGCACTGAAGGA-
----

AATAGCACACTAATCGAACTCCCATGCAGAATAAAACAAATTATAAATCTGTGGCAGGAAGTAGGAAAAGCAA
TGTATGCCCCTCCCATCAGAGGACAAATTAGATGTTCGTCAAAT---
ATTACGGGGCTGCTATTAACAAGAGATGGTGGTAATAATAGCGAG----------------------------
----

AGCGCCCCCGAGATCTTCAGACCTGGAGGAGGAGATATGAGGGACAATTGGAGAAGTGAATTATATAAATATA
AAGTAGTACAAATTGAACCATTA---GGAGTAGCACCCACTAGGGCGAAGAGAAGAGTGGTG-----------
----------------------------CAAAGAGAAAAAAGAGCG------GCA---ATAGGAGCT------
---CTGTTCCTTGGGTTCTTGGGAGCA---GCAGGAAGCACTATG---GGCGCAGCGTCA---
ATAACGCTGACGGTACAG---GCCAGACTA---TTATTG---TCTGGTATAGTGCAACAGCAGAAC---
AATTTG---CTGAGA---GCTATTGAGGCGCAACAGCATCTGTTGCAACTCACAGTCTGGGGCATC
>MH012793
TTCTACTGCAATACAACACAACTGTTTAATAGTACTTGGGGGAATGGTACTGAGAGGTCAAATGAA-------
----

ACGGAAGATACTATCACACTCCCATGCAGAATAAAACAAATTGTAAACATGTGGCAGCAAGTAGGAAAAGCAA
TGTATGCCCCTCCCATCAGAGGACAAATTAGATGTTCATCAAAT---
ATTACAGGGCTGCTATTAACAAGGGATGGTGGTGATAAATTAACAAGGGATGGT-------------------
----
-
GGTGATAATGAGACCACCGAGATCTTCAGACCTGGAGGAGGAGATATGAAGGACAATTGGAGAAGTGAATTAT
ATAAATATAAAGTAGTAAAAATTGAACCATTA---GGAGTAGCACCCACCAGGGCAAAAAGAAGAGTGGTG--
-------------------------------------CAGAGAGAAAAAAGAGCA---
GTGGGAACGATAGGAGCT---------ATGTTCCTTGGGTTCTTGGGAGCA---GCAGGAAGCACTATG---
GGCGCAGCGTCA---CTGACGCTGACGGTACAG---GCCAGACTA---TTGTTG---
TCTGGTATAGTGCAACAGCAGAAC---AATTTG---CTGAGA---
GCTATTGAGGCGCAACAGCATCTGTTGCAACTCACAGTCTGGGGCATC
>EU576292
TTCTACTGTAATTCAACACAACTGTTTAATAGTACTTGGAGTTTTAATAGTACTTGGAATAAGAATTTTAATA
GTACTTGGAAT--
-------ATTACTGAAGGG------
ACAAATAACACTGAAATCACACTCCCATGTAGAATAAAACAAATTATAAACATGTGGCAGGAAGTAGGAAAAG
CAATGTATGCCCCTCCCATCAGAGGACAAATTAAATGTTCATCTAAC---
ATTACAGGGCTGCTATTAACAAGAGATGGTGGGGGTAATGGGAGC----------------------------
----

GGGACCACTGAGGTCTTCAGACCTGGAGGGGGAGATATGAGGGACAATTGGAGAAGTGAATTATACAAATATA
AAGTAGTAAAAATTGAACCATTA---GGAGTAGCACCTACCAAGGCAAAGAGAAGAGTGGTG-----------
----------------------------CAAGGAGAAAAAAGAGCA---GTGGGAGCAATAGGAGCT------
---ATGTTCCTTGGGTTCTTGGGAGCA---GCAGGAAGCACTATG---GGCGCAGCGTCA---
GTGGCGCTGACGGTACAG---GCCAGACAA---TTATTG---TCTGGTATAGTGCAACAGCAGAAC---
AATTTG---CTGAGG---GCTATTGAGGCGCAACAGCATCTGTTGCAACTCACAGTCTGGGGCATC
>KT283697
TTCTACTGTGATTCAACACAATTGTTTAATAGTACTTGGAATAGTACTTGGCTGAATGATACCGAAGGGTTAA
ATAAAACT---

GAAGGAGACCCTATTATACTCCAATGCAGAATAAAACAATTTATAAACATGTGGCAGGAAGTAGGAAAAGCAA
TGTATGCCCCTCCCATTGCAGGACCAATTAATTGTTCATCAAAT---
ATTACAGGACTGCTATTAACAAGAGATGGTGGCAATAACAATGACACT-------------------------
----

AGCAACACCGAGATTTTTAGACCTGGAGGAGGAGATATGAGGGACAATTGGAGAAGTGAATTATATAAATATA
AAGTAGTAAAAATTGAACCATTA---GGACTAGCACCTACCAAGGCAAAGAGAAGAGTGGTG-----------
----------------------------CAGAGAGAAAAAAGAGCA---GTGGGA---ATAGGAGCT------
---ATGTTCCTTGGGTTCTTGGGAGCA---GCAGGAAGCACTATG---GGCGCAGCGTCA---
GTGACGCTGACGGTACAG---GCCAGACTA---TTATTG---TCTGGTATAGTGCAACAGCAGAAC---
AATTTG---CTGAGG---GCTATTGAGGCGCAACAGCATCTGTTGCAACTCACAGTCTGGGGCATC

>AY173952
TTCTACTGTAATTCAACACAACTGTTTAATAGTACTTGGGGGGCTAATGATACTTGGACTGAGTCAAAT----
----

AACAATGAAACAATCACACTCCCATGCAGAATAAAACAAATTATAAACATGTGGCAGGAAGTAGGAAAAGCAA
TGTATGCCCCTCCCATCGCAGGACAAATTAGATGTTCATCAAAT---
ATTACAGGGCTGCTACTAACAAGAGATGGTGGTAATAAAAACGATAGC-------------------------
----

AACGGGACCGAGATCTTCAGACCTGGAGGAGGAGATATGAGGGACAATTGGAGAAGTGAATTATATAAATATA
AAGTAGTAAAAATTGAACCATTA---GGAATAGCACCCACCAAGGCAAAGAGAAGAGTGGTG-----------
----------------------------CAGAGAGAAAAAAGAGCA---GTAGGC---CTAGGAGCT------
---ATGTTCCTTGGGTTCTTGGGAGCA---GCAGGAAGCACTATG---GGCGCAGCGTCA---
ATAACGCTGACGGTACAG---GCCAGACAA---TTATTG---TCTGGTATAGTGCAACAGCAGAAC---
AATTTG---CTGAGG---GCTATTGAGGCGCAACAGCATCTGTTGCAACTCACAGTCTGGGGCATC
>AY173953
TTCTACTGTAATACAACAAATCTGTTTAATAGTACTTGGAATGGTACTGAAGAGTTTGATAGTACTTTGAGT-
----

GATGCTGAAAACATCACACTCCAATGCAGAATAAAACAAATTATAAACATGTGGCAGGAAGTAGGAAAAGCAA
TGTATGCCCCTCCCATCAGAGGACACATTAGATGTGTATCAAAC---
ATTACAGGGCTGTTATTAACAAGAGATGGGGGTATGAACAACGGGACCAAG----------------------
----

GACAACGAGACCGAGGTCTTCAGACCTGGAGGAGGAGATATGAGGGACAATTGGAGAAGTGAATTATATAAAT
ATAAAGTAGTAAAAATTGAACCATTA---GGAGTAGCACCCACCAAGGCAAAGAGAAGAGTGGTG--------
-------------------------------CAGAGAGAAAAAAGAGCA---GTGGGA---ATAGGAGCT---
------ATGTTCCTTGGGTTCTTGGGAGCA---GCAGGAAGCACTATG---GGCGCAGCGTCA---
CTGACGCTGACGGTACAG---GCCAGACAA---TTATTG---TCTGGTATAGTGCAACAGCAGAAC---
AATTTG---CTGAGG---GCTATTGAGGCGCAACAGCATCTGTTGCAACTCACAGTCTGGGGCATC
>AY173954
TTCTACTGTAATTCAACACAGCTGTTTAATAGTACTTGGATTAATAATAATGGTACTTGGAATAGTACTTGGA
GTAGTAATAAT--
-------------AATACT------
GATAACAATGGAACTATCACACTCCCATGCAGAATAAGACAAATTATAAACAGGTGGCAGGAAGTAGGAAAAG
CAATGTATGCCCCTCCCATCAGAGGACAAATTAGATGTTCATCAAAT---
ATTACAGGGCTGCTATTAACAAGAGATGGTGGTAATAACAATAACACG-------------------------
----

AACAGGACCGAGACCTTCAGACCTGGAGGAGGAGATATGAGGGACAATTGGAGAAGTGAATTATATAAGTATA
AAGTAGTACAAATTGAGCCATTA---GGAGTAGCACCCACCAGGGCAAAGAGAAGAGTGGTG-----------
----------------------------CAGAGAGAAAAAAGAGCA---GTGGGAACGATAGGAGCT------
---ATGTTCCTTGGGTTCTTGGGAGCA---GCAGGAAGCACTATG---GGCGCAGCGTCA---
ATGACGCTGACGGTACAG---GCCAGGCTA---TTATTG---TCTGGTATAGTGCAGCAGCAGAAC---
AATTTG---CTGAGG---GCTATTGAGGCACAACAGCATATGTTGCAACTCACAGTCTGGGGCATC
>U21135
TTCTACTGTAATTCAACACAGCTGTTTAATAGTACTTGGCATGCTAATGGTACTTGGAAGAATACTGAAGGG-
----

GCAGATAACAATATCACACTCCCATGCAGAATAAAACAAATTATAAACAGGTGGCAGGAAGTAGGAAAAGCAA
TGTATGCCCCACCCATCGAAGGACAAATTAGATGTTTATCAAAT---
ATTACAGGGTTACTATTAACAAGAGATGGTGGTAGTAGTGAAGAG----------------------------
----

AACCAGACCGAGATCTTCAGACCTGGAGGAGGAAATATGAAGGATAATTGGAGAAGTGAATTATATAAATATA
AAGTAGTAAAAATTGAACCATTA---GGAGTAGCACCCACCAAGGCAAAGAGAAGAGTGGTG-----------
----------------------------CAGAGAGAAAAAAGAGCA---GTGGGAATGCTAGGAGCT------
---ATGTTCCTTGGGTTCTTGGGAGCA---GCAGGAAGCACTATG---GGCGCAGCGTCA---
ATGACGCTGACGGTACAG---GCCAGACTA---TTATTG---TCTGGTATAGTGCAACAGCAGAAC---
AATCTG---CTGAGG---GCTATTGAGGCGCAACAGCATCTGTTCGAACTCACAGTCTGGGGCATC

>MH012589
TTCTACTGTAATACATCAAAACTGTTTAATAGTACTTGGAATGGTACTACAGAGTCAAATAAC----------
----

ATTGGAGAAAATATCACACTCCCATGCAGAATAAAACAAATTATAAACATGTGGCAGGAAGTAGGAAAAGCAA
TGTATGCTCCTCCCATCAGTGGACAAATTAGATGTTCATCAAAT---
ATTACAGGGCTGCTATTAACAAGAGATGGTGGTAATAACACAAAT----------------------------
----

AATATCGAGACCTTCAGACCTGGAGGAGGAGATATGAGGGACAATTGGAGAAGTGAATTATATAAATACAAAG
TAGTAAGAGTTGAACCATTA---GGACTAGCACCCACCAAGGCAAGGAGAAGAGTGGTG--------------
-------------------------CAGAGAGAAAAAAGAGCA---GTGGGA---ATAGGAGCT---------
GTGTTCCTGGGGTTCTTGGGAGCA---GCAGGAAGCACTATG---GGCGCAGCGTCA---
GTGACGCTGACGGTACAG---GCCAGACAA---TTATTG---TCTGGTATAGTGCAACAGCAGAAT---
AATCTG---CTGAGG---GCTATTGAGGCGCAACAGCATATGTTGCAACTCACAGTCTGGGGCATC
>MH012650
TTCTACTGTAATACAACACAACTGTTTAATAGTACTTGGAATAGTACTGAAACGTCAAATATCACT-------
----

GGAGAAGATATCATCACACTCCCATGCAGAATAAAACAAATTATAAACATGTGGCAGGAAGTAGGAAAAGCAA
TGTATGCCCCTCCCATCGCAGGACCAATTAGATGTACATCAAAT---
ATTACAGGGATGATACTAACAAGAGATGGAGGTAAAAACAACGGG----------------------------
----

AGCGATGAGGTCCTCAGACCTGGAGGAGGAGATATGAGGGACAATTGGAGAAGTGAATTATATAAATATAAAG
TAGTAAGAATTGAACCATTA---GGAGTAGCACCCTCCAGGGCAAAGAGAAGAGTGGTG--------------
-------------------------CAAAGAGAAAAAAGAGCA---GTGGGAGCGATAGGAGCT---------
ATGTTCCTTGGGTTCTTAGGAGCA---GCAGGAAGCACTATG---GGCGCAGCGGCA---
GTGACGCTGACGGTACAG---GCCAGACAG---CTACTG---TCTGGTATAGTGCAACAGCAGAAC---
AATTTG---CTGAGG---GCTATTGAGGCGCAACAGCATCTGTTGCAACTCACAGTCTGGGGCATC
>MH012866
TTCTACTGTAATTCAACACAACTGTTTAATAGTACTTGGGTTAATGGTACTTTGAATAGTACT----------
----

GGAGGAGAAAATATCACAATCCCATGCAGAATAAAACAAATTATAAACATGTGGCAGACAGTAGGAAAAGCAA
TGTATGCCCCTCCCATCAGAGGACAAATTAAATGTTCATCAAAT---
ATTACAGGGCTACTATTAACAAGAGATGGTGGTGAAAACGGGAGC----------------------------
----

AATACTGAGATCTTCAGACCTGGAGGAGGAGATATGAGGGACAATTGGAGAAGTGAATTATATAAATATAAAG
TAGTAAAAATTGAACCATTA---GGAGTAGCACCCACCAAGGCAAAGAGAAGAGTGGTG--------------
-------------------------CAAAGAGAAAAAAGAGCA---GTGGGA---ATAGGAGCT---------
TTGTTCCTTGGGTTCTTGGGAGCA---GCAGGAAGCACTATG---GGCGCAGCGTCA---
ATGACGCTGACGGTACAG---GCCAGACAA---TTATTG---TCTGGAATAGTGCAACAGCAAAAC---
AATTTG---CTGAGG---GCTATTGAGGCGCAACAGCATATATTGCAACTCACAGTCTGGGGCATC
>MH012925
TTCTACTGTAATACAACAAAACTGTTTAATAGTACTTGGACTTGGAATGGTACTGCTTGGACTTGGAATAGTA
CTGAAGGGTCA--

CCTGACAATGACACAATCATACTCCCATGCAGAATAAAACAAATTATAAACATGTGGCAGGAAGTAGGAAAAG
CAATGTATGCCCCTCCCATCAGGGGACAAATTAAATGTTCATCAAAT---
ATTACAGGGTTGCTATTAACAAGAGATGGTGGTGATAACAGGAAC----------------------------
----

AACACCACTGAGATCTTCAGACCAGGAGGAGGAGATATGAGGGACAATTGGAGAAGTGAATTATATAAATATA
AAGTAGTAAAAATTGAACCATTA---GGAGTAGCACCCACCAAGGCAAAGAGAAGAGTGGTG-----------
----------------------------CAGAGAGAAAAAAGAGCA---GTGGGA---ATAGGAGCT------
---TTGTTCCTTGGGTTCTTGGGAGCA---GCAGGAAGCACTATG---GGCGCAGCGTCA---
ATGACGCTGACGGTACAG---GCCAGACAA---TTATTG---TCTGGTATAGTGCAACAGCAGAAC---
AATCTG---CTGAGG---GCTATTGAGGCGCAACAACATCTGTTGCAACTCACAGTCTGGGGCATC

>AY835761
TTCTACTGTAATACAACAGCACTGTTTAATAGGACATTTAATAGGACTGAGAATGATAATGGTACCTGGAAT-
----

AGTACTGACCCTATCATACTCCAATGCAGGATAAAACAAATTGTAAACATGTGGCAGGAAGTAGGAAAAGCAA
TGTATGCCCCTCCCATCAAAGGACAAATTAGATGCTCATCAAAT---
ATTACAGGGCTGCTATTAACAAGAGATGGTGGTAATGGCAATGATACT-------------------------
----

AAAAATGATACCGAGATCTTCAGACCTGGAGGAGGAGATATGAGGGACAATTGGAGAAGTGAATTATATAAAT
ATAAAGTAGTAAGAATTGAACCATTA---GGAATAGCACCCACCAAGGCAAAGAGAAGAGTGGTG--------
-------------------------------CAGAGAGAAAAAAGAGCA---GTGGGA---ATAGGAGCT---
------GTGTTCCTTGGGTTCTTGGGAGCA---GCAGGAAGCACTATG---GGCGCAGCGTCA---
ATAACGCTGACGGTACAA---GCCAGACTA---TTATTG---TCTGGTATAGTGCAACAGCAGAAC---
AATTTG---CTGAGG---GCTATTGAGGCGCAACAGCATCTGTTGCAACTCACAGTCTGGGGCATC
>AF069140
TTCTACTGTAATACAAAAAAACTGTTTAATAGTACTTGGAATGGTACTGAAGGGTCATATAACATTGAA----
----

GGAAATGACACTATCACACTCCCATGCAGAATAAAACAAATTATAAACATGTGGCAGGAAGTAGGAAAAGCAA
TGTATGCCCCTCCCATCAGTGGACAAATTTGGTGCTCATCAAAT---
ATTACAGGGCTGCTACTAACAAGAGATGGTGGTAAGAACAGCAGC----------------------------
----

ACCGAAATCTTCAGACCTGGAGGAGGAGATATGAGGGACAATTGGAGAAGTGAATTATATAAATATAAAGTAG
TAAGAGTTGAACCATTA---GGAATAGCACCCACCAAGGCAAAGAGAAGAGTGGTG-----------------
----------------------CAGAGAGAAAAAAGAGCA---GTGGGA---ATAGGAGCT---------
GTGTTCCTTGGGTTCTTGGGAGCA---GCAGGAAGCACTATG---GGCGCAGCGTCA---
ATAACGCTGACGGTACAG---GCCAGACAA---TTATTG---TCTGGTATAGTGCAACAGCAGAAC---
AATTTG---CTGAGG---GCTATTGAAGCGCAACAGCATATGTTGCAACTCACAGTCTGGGGCATC
>GU331649
TTCTACTGTAATACAACACAGCTGTTTAATAGTACTTGGCCTTTTAATAGTACTTGGAATGATACTAACACTG
AA---

GGAAATGACACAATCACACTCCCATGTAGAATAAAACAATTTATAAACATGTGGCAGGAAGTAGGAAAAGCAA
TGTATGCCCCTCCCATCAGTGGACAAATTAGATGTTCATCAAAT---
ATTACAGGGCTGTTATTAACAAGAGATGGTGGTATAAACCAGAGC----------------------------
----

AGGACGAACGAGACCTTCAGACCTGGAGGAGGAAATATGAAGGACAATTGGAGAAGTGAATTGTACAAATATA
AAGTAGTAAAAATTAAACCATTA---GGAATAGCACCCACTAAGGCAAAGAGAAGAGTGGTG-----------
----------------------------CAGAGAGAAAAAAGAGCA---GTGGGA---ATAGGAGCT------
---CTGTTCCTTGGGTTCTTGGGAGCA---GCAGGAAGCACTATG---GGCGCAGCGTCA---
GTGACGCTGACGGTACAG---GCCAGACTA---TTATTG---TCTGGTATAGTGCAACAGCAGAAC---
AATTTG---CTGAGA---GCTATTGAGGCGCAACAGCATCTGTTGCAACTCACAGTCTGGGGCATC
>AY608577
TTCTACTGCAATTCAACACAACTGTTTAATGGTACTTGGAATGGTACTTGGTTAGATGTCAAACAA-------
----

GGAAATGGCACAATCACACTCCCATGCAGAATAAAACAAATTATAAACCTGTGGCAGGAAGTAGGAAAAGCAA
TGTATGCCCCTCCCATCAGCGGACAAATTAGATGTTCATCAAAT---
ATTACAGGGCTGTTATTAACAAGAGATGGTGGTACAAGCAATGAGACC-------------------------
----

ACCACCACCGAGACCTTCAGACCTGGAGGAGGAAATATGAAGGACAATTGGAGAAGTGAATTATATAGATATA
AAGTAATAAAAATTGAACCATTA---GGAGTAGCACCCACCAAGGCAAGGAGAAGAGTGGTG-----------
----------------------------CAGAGAGAAAAAAGAGCA---GTGGGA---ATAGGAGCT------
---GTGTTCCTTGGGTTCTTGGGAGCA---GCAGGAAGCACTATG---GGCGCAGCGTCA---
ATGACGCTGACGGTACAG---GCCAGACAA---TTATTG---TCTGGTATAGTGCAACAGCAGAAC---
AATTTG---CTGAGG---GCTATTGAGGCGCAACAGCAACTGTTGAAACTCACAGTCTGGGGCATC

>JN944928
TTCTATTGTAATACAACACAACTGTTTAATAGTACTTGGTATATTAATAATACTGGGAATGGTGCTAAAGGGT
CAGAT--

AACACTGACACTATCAAACTCCCATGCAGAATAAAACAAATTATAAACATGTGGCAGGAGGTAGGAAAAGCAA
TGTATGCCCCTCCCATCAGAGGACAAATTAGATGTTCCTCAAAT---
ATTACAGGACTGATATTAACAAGAGATGGGGGTAATAACAATGATACC-------------------------
----

AACAATGATACCGAGGTCTTCAGACCTGGAGGAGGAGATATGAGAGACAATTGGAGAAGTGAATTATATAAAT
ATAAAGTAGTAAAAATTGAACCATTA---GGAGTAGCACCCACTAAGGCAAAGAGAAGAGTGGTG--------
-------------------------------CAGAGAGAAAAAAGAGCA---GTGGGA---ATAGGAGCC---
------TTGTTCCTTGGGTTCTTGGGAGCA---GCAGGAAGCACTATG---GGCGCAGCGTCA---
GTGACGCTGACGGTACAG---GCCAGACAA---TTGTTG---TCTGGTATAGTGCAACAGCAGAAC---
AATTTG---CTGAAG---GCTATTGAGGCGCAACAGCATCTGTTGCAACTCACAGTCTGGGGCATC
>MH013129
TTCTACTGTAATTCAACACAACTATTTAATAGTACTTGGGAGAGGGAAGAGTCAAATGACACTCGA-------
----

GGGAATGACACAATCACACTCCCATGCAGAATAAAACAAATTATAAACATGTGGCAGGAAGTAGGAAAAGCAA
TGTATGCCCCTCCCATCAGAGGACAAATTAGCTGTTCATCAAAT---
ATTACAGGGCTGCTATTAACAAGAGATGGTGGTAATGTGAGCGAC----------------------------
----

CCCGAGACCTTCAGACCTGGAGGAGGAGATATGAGGGACAATTGGAGAAGTGAATTATATAAATATAAAGTAG
TAAAAATTGAACCATTA---GGAGTAGCACCCACCAAGGCAAAGAGAAGAGTGGTG-----------------
----------------------CAGAGAGAGAGAAGAGCA---GTGGGC---CTAGGAGCT---------
ATGTTCCTTGGGTTCTTGGGAGCA---GCAGGAAGCACTATG---GGCGCAGCGTCA---
ATAACGCTGACGGTACAG---GCCAGACAA---TTATTG---TCTGGTATAGTGCAACAGCAGAAC---
AATCTG---CTGAGG---GCTATTGAGGCGCAACAGCATCTGTTGCAACTCACAGTCTGGGGCATC
>U04908
TTCTACTGTAATACAACACAGTTATTCAATAGTACTTGGTATCGGAATGGTACTTGGTATTGGAATGGTACTA
GGATTGAGAAT--
-------GGTACTGAAGGG------
TTAAATGACACAATTATCACCCTCCCATGCAGAATAAAACAAATTATAAACATGTGGCAGGAAGTAGGAAAAG
CAATGTATGCTCCTCCCATCAAAGGACAAATTAGCTGTTCATCAAAT---
ATTACAGGGCTGCTATTAACAAGAGATGGTGGTAATACTAATAAGACCGGG----------------------
----

ACCGAGAACGAGACCGAGATCTTTAGACCTGGAGGAGGAGATATGAGGGACAATTGGAGAAGTGAATTATATA
AATATAAAGTAGTAAAAATTGAACCATTA---GGAGTAGCACCCACCAGGGCAAAGAGAAGAGTGGTG-----
----------------------------------CAGAGAGAAAAAAGAGCA---GTGGGA---
ATAGGAGCT---------ATGCTCTTTGGGTTCTTGGGAGCA---GCAGGAAGCACTATG---
GGCGCAGCGTCA---ATAACGCTGACGGTACAA---GCCAGACAA---TTGTTG---
TCTGGTATAGTGCAACAGCAGAGT---AATCTG---CTGAGG---
GCTATTGAGGCGCAACAACATCTGTTGCAACTCACAGTCTGGGGCATC
>KT283706
TTCTACTGTAATACAACACAACTGTTTAATAGTACTTGGAATAGTACTGGAGGACTAAATAACACTGGA----
----

GGAAATGACACAATCACACTCCCATGCAGAATAAAACAAATTATAAACATGTGGCAGGAAGTAGGAAAAGCAA
TGTATGCCCCTCCCATCAGAGGACAAATTAGATGTTCATCAAAT---
ATTACAGGGCTGCTATTAACAAGAGATGGTGGTAATAATGGGACC----------------------------
----

AGCGGGGCCGAGGTCTTCAGACCTGGAGGAGGAGATATGAGGGACAATTGGAGAAGTGAATTATATAAATATA
AAGTAGTACAAATTGAACCATTA---GGAGTAGCACCCACCAAGGCAAAGAGAAGAGTGGTG-----------
----------------------------CAGAGAGAAAAAAGAGCA---GTGGGA---ATAGGAGCT------
---GTGTTCCTTGGGTTCTTGGGAGCA---GCAGGAAGCACTATG---GGCGCAGCGTCA---

ATGACGCTGACGGTACAG---GCCAGACTA---TTATTG---TCTGGTATAGTGCAACAGCAGAAC---
AATTTG---CTGAGA---GCTATTGAGGCGCAACAGCATCTGTTGCAACTCACAGTCTGGGGCATC
>MH012332
TTCTACTGTAATTCAACACAACTGTTTAATAGTACTTGGAATGGTACGGAAGGGTCAAATAACCCTGTA----
----

GACAATGGCACAATCACGCTCCCATGCAGAATAAAACAAATTGTAAACATGTGGCAGGAAGTAGGAAAAGCAA
TGTATGCCCCTCCCATTAGAGGACAAATTAACTGTTTATCAAAT---
ATTACAGGGCTGCTATTAACAAGAGATGGTGGTGATAACGGGAGC----------------------------
----

AATACCACCGAGACCTTCAGACCTGGAGGAGGAGATATGAGGGACAATTGGAGAAGTGAATTATATAAATATA
AAGTAGTAAAAATTGAACCATTA---GGAGTAGCACCTACCAAGGCAAAGAGAAGAGTGGTA-----------
----------------------------CAGAGAGAAAAAAGAGCA---GTGGGA---ATAGGAGCT------
---CTGTTCCTTGGGTTCTTGGGAGCA---GCAGGAAGCACTATG---GGCGCAGCGTCA---
TTGACGCTGACGGTACAG---GCCAGACTA---TTATTG---TCTGGTATAGTGCAACAGCAGAAC---
AATTTG---CTGAGG---GCTATTGAGGCGCAACAGCGTGTGTTGCAACTCACAGTCTGGGGCATC
>MH012400
TTCTATTGCAATACAACACAACTGTTT------
ACTTGGAATAGTAGTAGTCCTTGGAATATTACTGAAGGGGTAAATAGCACT----------------------
----
ATGGGAAATGACACACTCACACTCCCCTGCAAAATAAAACAAATTATAAACATGTGGCAGGAAGTAGGAAAAG
CAATGTATGCCCCTCCCATCAGAGGACAAATTAGATGTTCATCAAAT---
ATTACAGGGCTGCTATTAACAAGAGATGGTGGTAATGGAAACGAGAGC-------------------------
----

CAGGTCAATAATGAGACCTTCAGACCTGCAGGAGGAGATATGAGAGACAATTGGAGAAGTGAATTATATAAAT
ATAAAGTAGTAAAAATTGAACCATTA---GGAGTAGCACCCACCAGGGCAAAGAGAAGAGTGGTG--------
-------------------------------CAGAGAGAAAGAAGAGCA---GTGGGA---GTAGGAGCT---
------ATGTTCCTTGGGTTCTTGGGAGCA---GCAGGAAGCACTATG---GGCGCAGCGTCA---
CTGACGCTGACGGTACAA---GCCAGACTA---TTATTG---TCTGGTATAGTGCAACAGCAGAGC---
AATCTG---CTGAGG---GCTATTGAGGCGCAACAGCATCTGTTGCGACTCACAGTCTGGGGCATC
>MH012257
TTCTACTGTAATTCAACACAACTGTTTAATAGTACTTGGAATGGTACTGAAGGGTCAAATAACACTGAA----
----

GGAAATGATACAATCATACTCCCATGCAGAATAAAACAAATTATAAACATGTGGCAGGAAGTAGGAAAAGCAA
TGTATGCCCCTCCCATCAAAGGACAAATTAGATGTTCATCAAAT---
ATCACAGGGCTGCTATTAACAAGAGATGGTGGTAATAGCAGCGGG----------------------------
----

ACCACCGAGTTCTTCAGACCTGGGGGAGGAGATATGAGGGACAATTGGAGAAGTGAATTATATAAATATAAGG
TAGTAAAAATTGAACCATTA---GGAGTAGCACCCACCAGGGCAAAAAGAAGAGTGGTG--------------
-------------------------CAGAGAGAAAGGAGAGCA---GTGGGA---CTAGGAGCT---------
GTGTTCCTTGGGTTCTTAGGAGCA---GCAGGAAGCACTATG---GGCGCAGCGTCA---
GTGACGCTGACGGTACAG---GCCAGACAA---TTATTG---TCTGGTATAGTGCAGCAGCAGAAC---
AATTTG---CTGAAG---GCTATTGAGGCGCAACACCATCTGTTGCAACTCACAGTCTGGGGCATC
>MH012478
TTCTACTGTAATACAACAAAACTGTTTAATAGTACTTGGTATGGGAATGGTACTAAAGTGTCAACTAACACGG
AA---

GGAAATGGCACAATCATACTCCCATGCAGAATAAAACAACTTATAAACATGTGGCAGGAAACAGGAAAAGCAA
TGTATGCCCCTCCCATTAGAGGACAAATTAGATGTTCATCAAAT---
ATTACAGGGGTGCTATTAACAAGAGATGGGGGTAACAATGATGGGACT-------------------------
----

GGGACTGAAACCTTCAGACCTGGAGGAGGAGATATGAGGGATAATTGGAGAAGTGAATTATATAAATATAAAG
TAGTAAAAATTGAACCATTA---GGAGTAGCACCCACCAAGGCAAAGAGGAGAGTGGTG--------------
-------------------------CAGAGAGAAAAAAGAGCA---GTGGGA---ATAGGAGCT---------
GTGTTCCTTGGGTTCTTGGGAGCA---GCAGGAAGCACTATG---GGCGCAGCGTCA---

GTGACGCTGACGGTACAG---GCCAGACTA---TTATTG---TCTGGTATAGTGCAGCAGCAGAAT---
AATTTG---CTGAGG---GCTATTGAGGCGCAACAGCATCTGTTGCAACTCACAGTCTGGGGCATC
>MH013027
TTCTACTGTAATACAACAGACCTGTTTAGTAGTACTTGGAATGACACACAACTGAATAATACTGGGAGTGAAG
GGTCAAAT---
-------------AGTACT------
GGAAAAAATGACACAATCACACTCCGATGCAGAATAAAACAAATTATAAACATGTGGCAAGAAGTAGGAAAAG
CAATGTATGCCCCTCCCATCAAAGGACAAATTAGATGTGTATCAAAT---
ATTACAGGGCTGCTATTAACAAGAGATGGTGGTAGTAGTAACGAGACT-------------------------
----

AACGGGACCGAGATCTTCAGACCTGGAGGAGGAGATATGAGAGACAATTGGAGAAGTGAATTATATAAATATA
AAGTAGTACAAATTGAACCAGTA---GGAATAGCACCCACCAAGGCAAGGAGAAGAGTGGTG-----------
----------------------------CAGAGAGAAAAAAGAGCA---GTGGGA---ATAGGAGCT------
---CTGTTCCTTGGGTTCTTGGGAGCA---GCAGGAAGCACTATG---GGCGCAGCGTCA---
GTGACGCTGACGGTACAA---GCCAGACAA---TTATTG---TCTGGTATAGTGCAACAGCAGAAC---
AATCTG---CTAAGG---GCTATTGAGGCGCAACAGCATCTGTTGCAACTCACAGTCTGGGGCATC
>MH013075
TTCTACTGTAATACAACAAAACTGTTTAATAGTACTTGGAATTCAACACAACTTAGTAGTACTGAGAATAGTA
CCGAA--

GGGTCAGAGAACATCACACTCCCATGCAGCATAAAACAAATTATAAACATGTGGCAGAAAGTAGGAAAAGCAA
TGTATGCCCCGCCCATCGAAGGACAAATTAGATGTTCATCAAAT---
ATTACAGGGCTGCTATTAACAAGAGATGGTGGTGGTGGGAATGCGAGCAATGAG-------------------
----

ACCACCGAGACTTTCAGGCCTGGAGGAGGAGATATGAGAGAAAATTGGAGAAGTGAATTATATAAATATAAAG
TAGTAAGAATTGAACCATTA---GGAGTAGCACCCTCCACGGCAAAGAGAAGAGTGGTG--------------
-------------------------CAGAGAGAAAAAAGAGCA---GTGGGA---ATAGGAGCA------
TTGTTGTTCCTTGGGTTCTTGGGAGCA---GCAGGAAGCACTATG---GGCGCAGCGTCA---
ATGATGCTGACGGTACAG---GCCAGACTA---TTATTG---TCTGGTATAGTGCAACAGCAGAAC---
AATATG---CTGAGG---GCTATTGAGGCGCAACAGCATCTGTTGCAACTCACAGTCTGGGGCATC
>DQ487188
TTCTACTGTGATACAACACGACTGTTTAATAGTACTTGGAATAATACTTGGACTGGGAATGATACTTGGTCAA
ATAAC--
-------------------ACT---
AGAAGAAATGATACAATCACACTCCCATGCAGAATAAAACAAATTATAAACATGTGGCAGGAAGTAGGAAAAG
CAATGTATGCCCCTCCCATCAGAGGAAACATTACCTGTTCATCAAAT---
ATTACAGGGCTAATATTAACAAGAGATGGTGGTGATAACAATAGCACG-------------------------
----

AACGGGACTGAGACCTTCAGACCTGGAGGAGGAGATATGAGGGACAATTGGAGAAGTGAATTATATAAATATA
AAGTAGTACAAATTGAACCATTA---GGAGTAGCACCCACCAAGGCAAAGAGAAGAGTGGTG-----------
----------------------------CAAAGAGAAAAAAGAGCA---GTGGGC---ATAGGAGCT------
---GTGTTCCTTGGGTTCTTGGGAGCA---GCAGGAAGCACTATG---GGCGCAGCGTCA---
ATAACGCTGACGGTACAG---GCCAGACAA---TTATTG---TCTGGTATAGTGCAACAGCAGAAC---
AATTTG---CTGAGG---GCTATTGAGGCGCAACAACATCTGTTGCAACTCACAGTCTGGGGCATC
>DQ487190
TTCTACTGTGATGCAACACAACTGTTTAATAGTACTTGGAATAGTACTGAAGATTGGAATAGTACTGAAGATT
CAACTAAC---
-------------------GCT---
GATGGAAATAACACAATCATACTCCCATGCAGAATAAAACAGATTGTAAACATGTGGCAGGAAGTAGGAAAAG
CAATGTATGCCCCTCCCATCAGAGGACAGATTAGATGTTCATCAAAT---
ATTACAGGGCTGCTATTAACAAGAGATGGTGGTAACGGCACCAAT----------------------------
----

AACGACAATGAGACCTTCAGACCTGGGGGAGGAGATATGAGGGACAATTGGAGAAGTGAATTATATAAATATA
AAGTAGTAAAAATTGAACCATTA---GGAGTAGCACCCACCAAGGCAAAGAGAAGAGTGGTG-----------
----------------------------CAGAGAGAGAAAAGAGCG------GGC---ATAGGAGCT------
---TTGTTCCTTGGGTTCTTGGGAGCA---GCAGGAAGCACTATG---GGCGCAGCGTCC---

ATGACGCTGACGGTACAG---GCCAGACAA---TTATTG---TCTGGTATAGTGCAACAGCAGAAT---
AATTTG---CTGAGG---GCTATTGAGGCGCAACAGCATCTGTTGCAACTCACAGTCTGGGGCATC
>AY612855
TTCTACTGTAATACAACACAGCTGTTTAATAGTACTTGGAATGAGAATGATACTACCTGGAATAATACAGCAG
GGTCA--

AATAACAATGAAACTATCACACTCCCATGTAGAATAAAACAAATTATAAACAGGTGGCAGGAAGTAGGAAAAG
CAATGTATGCCCCTCCCATCAGTGGACCAATTAATTGTTTATCAAAT---
ATCACAGGGCTATTATTAACAAGAGATGGTGGTGACAACAATAAT----------------------------
----

ACAATAGAGACCTTCAGACCTGGAGGAGGAGATATGAGGGACAATTGGAGAAGTGAATTATATAAATATAAAG
TAGTAAGAATTGAGCCATTA---GGAATAGCACCCACCAAGGCAAAGAGAAGAGTGGTG--------------
-------------------------CAAAGAGAAAAAAGAGCA---GTGGGA---ATAGGAGCT---------
ATGTTCCTTGGGTTCTTGGGAGCA---GCAGGAAGCACTATG---GGCGCAGCGTCA---
GTGACGCTGACGGTACAG---GCCAGACTA---TTATTG---TCTGGTATAGTGCAACAGCAAAAC---
AATTTG---CTGAGA---GCTATCGAGGCGCAACAGCATCTGTTGCAACTCACAGTCTGGGGCATC
>AY835437
TTCTACTGTAATTCAACACAGCTGTTTAATAGTACTTGGACGCGGCCGCCTAATGGTACTTGGCGGTCTAATG
GTACTTGGAAT--
----------AGTACTGAA------
GGGACAAATGACACTATCACACTCCCATGTAGAATAAAACAAATTATAAACATGTGGCAGGGAGTAGGAAAAG
CAATGTATGCCCCTCCCATCAGTGGACAAATTAGATGTACATCAAAT---
ATCACAGGGCTGCTATTAACAAGAGATGGTGGTGAGAACAGGACAGAT-------------------------
----

AACGGGACAGAGATATTCAGACCTGGAGGAGGAAATATGAGGGACAACTGGAGAAGTGAATTATATAAATATA
AAGTAGTAAAAATTGAGCCATTA---GGAGTAGCACCCACCAAGGCAAAGAGAAGAGTGGTG-----------
----------------------------CAAAGAGAAAAAAGAGCA---GTGGGAACGATAGGAGCT------
---ATGTTCCTTGGGTTCTTGGGAGCA---GCAGGAAGCACTATG---GGCGCAGCGTCA---
ATAACGCTGACGGTACAG---GCCAGACTA---TTATTG---TCTGGTATAGTGCAACAGCAGAAC---
AATTTG---CTGAGG---GCTATTGAGGCGCAACAGCATATGTTGCAACTCACAGTCTGGGGCATC
>KT283729
TTCTACTGTAATACAACACAACTGTTTAATAGTACTTGGGCTTGGAATAGTACTGAAGGGTCAAATAGCACTG
GA---

GAAAATGGCACTATCACACTCCAATGCAGAATAAAACAAATTATAAATATGTGGCAGGAAGTAGGGAAAGCAA
TGTATGCCCCTCCCATCAGAGGACAAATTAAATGTTCATCAAAT---
ATTACAGGGCTGATATTAACAAGAGATGGAGGTAATAATAATGGT----------------------------
----

ACGAACAATACCATAGAGATCTTCAGACCTGGAGGAGGAGATATGAGAGAAAATTGGAGAAGTGAATTATATA
AATATAAAGTAGTAAAAATTGAACCATTA---GGAGTAGCACCCACCAGGGCAAAGAGAAGAGTGGTG-----
----------------------------------CAGAGAGAAAAAAGAGCA---
GTGGGAACAATAGGAGCT---------ATGTTCCTTGGGTTCTTGGGAGCA---GCAGGAAGCACTATG---
GGCGCAGCGTCA---ATGACGCTGACGGTACAG---GCCAGACAA---TTATTG---
TCTGGTATAGTGCAACAGCAGAAC---AACTTG---CTGAAA---
GCTATTGAGGCGCAACAGCACCTGTTGCAACTCACGGTCTGGGGCATC
>DQ313246
TTCTACTGTAATTCATCACAACTGTTTAATAGTACTTGGAATACTAATAGTACTTGGAGTGATACTAGAGGG-
----

TCAAATGACACTATCACACTCCCATGTAGAATAAAACAAATTATAAACATGTGGCAGGAAGTGGGAAAAGCAA
TGTATGCCCCTCCCATCAGAGGACAAATTAGCTGTTCATCAAAT---
ATTACAGGGCTGCTATTAACAAGAGATGGTGGGAACGAGAGCAAG----------------------------
----

CCCGACACCGAGACCTTCAGACCTGGAGGAGGAGATATGAGGGACAATTGGAGAAGTGAATTATATAAATATA
AAGTAGTAAAGGTTGAACCATTA---GGAGTAGCACCCACCAGGGCAAGGAGAAGAGTGGTG-----------
----------------------------CAAAGAGAAAAAAGAGCA---GTGGGAACGTTAGGAGCT------

---GTGTTCCTTGGGTTCTTGGGAGCA---GCAGGAAGCACTATG---GGCGCAGCGTCA---
ATGACGCTGACGGTACAG---GCCAGACAA---TTGTTG---TCTGGTATAGTGCAACAGCAGAAC---
AATTTG---CTGAGA---GCTATTGAGGCGCAACAGCATATGTTGCAACTCACAGTCTGGGGCATC
>EU289197
TTCTACTGTGATTCATCACCACTGTTTAATAGTACTTGGGAGAATGATACAGAAGTGTTAAATAACACTGTG-
----

GGAAATGAAACAATCACGCTCCCATGCAAAATAAAACAAATTATAAACTTGTGGCAGGAAGTAGGGAAAGCAA
TGTATGCCCCTCCCATCAGAGGACAAATTAAATGTTCATCAAAT---
ATTACAGGGCTACTATTAACAAGAGATGGTGGTACTAACGGGGAC----------------------------
----

AATACCACTGAGACCTTCAGACCTGGAGGAGGAGATATGAGGGACAATTGGAGAAGTGAATTATATAAATATA
AAGTAGTAAAGATTGAACCATTA---GGAGTAGCACCCACCAAGGCAAAGAGAAGAGTGGTG-----------
----------------------------CAAAGAGAAAAAAGAGCA---GTGGGA---TTAGGAGCT------
---CTGTTCCTTGGGTTCTTGGGAGCA---GCAGGAAGCACTATG---GGCGCAGCGTCA---
GTGACGCTGACGGTACAG---GCCAGACAA---TTACTG---TCTGGTATAGTGCAACAGCAGAAC---
AATCTG---CTGAGG---GCTATTGAAGCGCAACAGCATCTGTTGCAACTCACAGTCTGGGGCATC
>JQ609870
TTCTACTGCAATACATCACAACTGTTTAATAGTACTTGGCTTAATAAAACTTGGACTGGGTCAAT-------
----

GACACTGAAAATATCACACTCCCATGCAGAATAAAACAAATTATAAACATGTGGCAGGAAGTAGGAAAAGCAA
TGTATGCCCCTCCCATCAGCGGACAAATTAGATGTTCATCAAAT---
ATTACAGGGCTGCTATTAATAAGAGATGGTGGTAAGACAAACCAGAGT-------------------------
----

AACACGACCGAGACCTTCAGACCTGGAGGAGGAGATATGAGGGACAACTGGAGAAGTGAATTATATAAATATA
AAGTAGTAAGAATTGAACCAATA---GGAGTAGCACCCACCAAGGCAAAGAGAAGAGTGGTG-----------
----------------------------CAGAGAGAAAAAAGA------GTAGCA---CTAGGAGCT------
---ATGTTCCTTGGGTTCTTGGGAGCA---GCAGGAAGCACTATG---GGCGCAGCGTCA---
GTGACGCTGACGGTACAG---GCCAGACTA---TTATTG---TCTGGTATAGTGCAACAGCAGAAC---
AATTTG---CTGAGG---GCTATTGAGGCGCAACAGCGTCTGTTGCAACTCACAGTCTGGGGCATC
>AY835436
TTCTACTGTAATTCAACACAGCTGTTTAATAGTACTTGGAGTGTTAATAGTACTGGGAATGATACTACT----
----

GAAGAGCCGAATATCACATTCCCATGCAGAATAAAACAAATTATAAACATGTGGCAGGAAGTAGGAAAAGCAA
TGTATAACCCTCCCATTAAAGGAATAATTAGTTGTAGATCAAAT---
ATTACAGGGCTGTTATTAACAAGAGATGGTGGTAATAACAACAACACC-------------------------
----

AACACCACCGAAACCTTCAGACCTGAAGGAGGAAATATGAAGGACAATTGGAGAAGTGAATTATATAAATATA
AAGTAGTAAAAATTGAACCATTA---GGAGTAGCACCCACCAAGGCAAAGAGAAGAGTGGTG-----------
----------------------------CAAAGAGAGAAAAGAGCA---GTAGGA---ATAGGAGCT------
---GTGTTCCTTGGGTTCTTGGGAGCA---GCAGGAAGCACTATG---GGCGCAGCGTCA---
GTGACGCTGACGGTACAA---GCCAGACAA---TTATTG---TCTGGTATAGTGCAACAGCAGAAC---
AATCTG---CTGAGG---GCTATTGAGGCGCAACAGCATATGTTGCAACTCACAGTCTGGGGCATC
>AY835768
TTCTACTGTAATACAACAAAACTGTTTAATAGTACTTGGATGAATAATGGTACTTGGATTGGTACTAACGAG-
----

TCAGATAGCAACATCACGCTCCCATGCAGAATAAAACAAATTATAAACATGTGGCAGGAAGTAGGAAAAGCAA
TGTATGCCCCTCCCATCGAAGAACAAATTAGTTGTTTATCAAAC---
ATTACAGGGCTGCTATTAACAAGGGATGGTGGTGGGAACGAGAGCAAG-------------------------
----

AACGGGAGCGAGACCTTCAGACCTGGAGGAGGAGATATGAGGGACAATTGGAGAAGTGAATTATATAAATATA
AAGTAGTAAAAATTGAACCATTA---GGAGTAGCACCCACTGAGGCAAAGAGAAGAGTGGTG-----------
----------------------------CAGAGAGAAAAAAGAGCA---GTGGGA---ATAGGAGCT------

---TTGTTCCTTGGGTTCTTGGGAGCA---GCAGGAAGCACTATG---GGCGCAGCGGCA---
ATGACGCTGACGGTACAG---GCCAGACAA---TTATTG---ACTGGTATAGTGCAACAGCAGAAC---
AATTTG---CTGAGG---GCTATTGAGGCGCAACAGCATCTGTTGCAACTCACAGTCTGGGGCATC
>EU289190
TTCTACTGTGATACAACACAGCTGTTTAATAGTACTTGGTCGTCTAATGATACTTGGAATAGTACTGGAGTG-
----

CAAGATAACAATATCACACTCCCATGCAGAATAAAACAAATTATAAACATGTGGCAGGAAGTAGGAAAAGCAA
TGTATGCCCCTCCCATCCAAGGACTAATTAGCTGTTCATCAAAT---
ATTACAGGGCTGCTATTAACAAGAGATGGTGGTACTAACAATACC----------------------------
----

AATGCCACCGAGATCTTCAGACCAGGAGGAGGAGATATGAGGGACAATTGGAGAAGTGAATTATATAAATATA
AAGTAGTAAAAATTGAACCATTA---GGAATAGCACCCACCAAGGCAAAGAGAAGAGTGGTG-----------
----------------------------CAAAGAGAAAAAAGAGCA---GTGGGA---CTAGGAGCT------
---GTGTTCATTGGGTTCTTGGGAGCA---GCCGGAAGCACTATG---GGCGCAGCGTCC---
GTGACGCTGACGGTACAG---GCCAGACAA---TTACTG---TCTGGTATAGTGCAACAGCAGAAC---
AATCTG---CTGAGA---GCTATTGAGGCGCAACAACATATGTTGCAACTCACAGTCTGGGGCATC
>AY835438
TTTTACTGTGATACATCACAACTGTTTAATAGTACTTGGAATTCTAATAGTACTTGGAATGATACTAACAATA
AT---

AATAGCACTGAAAAGATTATACTCTCATGTAGAATAAGACAAATTATAAACAGGTGGCAGGAAGTAGGAAAAG
CAATGTATGCCCCTCCCATCAGCGGACCAATTAAGTGTTCATCAAAT---
ATTACAGGGCTGCTATTAGCAAGAGATGGTGGAAATGAGACTAAT----------------------------
----

GTGACCGAGACCTTCAGACCTGCAGGAGGAGATATGAGGGACAATTGGAGAAGTGAATTATATAAATATAAAG
TAGTACAAATTGAACCATTA---GGAATAGCACCCACCAAGGCAAAGAGAAGAGTGGTG--------------
-------------------------CAGAGAGAAAAAAGAGCA---GTGGGAATGCTAGGAGCT---------
ATGTTCCTTGGGTTCTTGGGAGCA---GCAGGAAGCACTATA---GGCGCAGCGTCA---
ATGACGCTGACGGTACAG---GCCAGACAA---TTATTG---TCTGGCATAGTGCAACAGCAAAAC---
AATTTG---CTGAGG---GCTATTGAGGCGCAACAACATATGTTGCAACTCACAGTCTGGGGCATC
>KT283753
TTCTACTGTAATACAACACAACTGTTTAACAGTACTTGGAATGTTACTGATACTGAAGTGTCAAATAACACTA
AT---

GGAAATGACACAATCACACTCCCATGCAGAATAAAACAAATTATAAACATGTGGCAGGAAGTAGGAAAAGCAA
TGTATGCCCCTCCCAGAGAAGGGCAAATTAAATGTTCATCAAAT---
ATTACAGGGCTACTATTAACAAGAGATGGTGGTAATAGTAATAATACT-------------------------
----

GACAATGGAACCGAGGTCTTCAGACCTGGAGGAGGAAACATGAAGGACAATTGGAGAAGTGAATTATATAAAT
ATAAAGTAGTAAGAATTGAACCATTA---GGGATAGCACCCACCAAGGCAAAGAGAAGAGTGGTG--------
-------------------------------CAGAGAGAAAAAAGAGCA---GTGGGA---TTCGGAGCT---
------GTGTTCCTTGGGTTCTTGGGAGCA---GCAGGAAGCACTATG---GGCGCAGCGTCA---
ATAACGCTGACGGTACAG---GCCAGACAA---TTATTG---TCTGGTATAGTGCAACAGCAAAAC---
AATTTG---CTGAGG---GCTATTGAGGCGCAACAGCATCTGTTGCAACTCACAGTCTGGGGCATC
>EU289198
TTCTACTGTAATACAACACAACTGTTTAATAGTACTTGGCCTTGGAATGTTACTGAAGAGTCAAAT-------
----

AGCACTGACAATATCACACTCCCATGCAGAATAAAACAAATTGTAAACATGTGGCAGGAAGTAGGAAAAGCAA
TGTATGCCCCTCCCATCAGAGGACCAATTAGATGTTCATCAAAT---
ATTACAGGTCTGCTATTAACAAGGGATGGTGGGAATACCATAAAC----------------------------
----

GGAACTGAGATCTTCAGACCTGGAGGAGGAGATATGAGGGACAATTGGAGAAGTGAATTATATAAATATAAAG
TAGTAAAAATTGAACCATTA---GGAGTAGCACCCACCAAGGCAAAGAGAAGAGTGGTG--------------
-------------------------CAGAGAGAAAAAAGAGCA---GTGGGA---ATAGGAGCT---------

ATGTTCCTTGGGTTCTTGGGAGCA---GCAGGAAGCACTATG---GGCGCAGCGTCA---
ATAACGCTGACGGTACAG---GCCAGACAA---TTATTG---TCTGGTATAGTGCAACAGCAGAAC---
AATTTG---CTGAAG---GCTATTGAGGCGCAACAGCATCTGTTGCAACTCACAGTCTGGGGCATC
>KU678164
TTCTATTGTAATACAACACAACTGTTTAATAGTACTTGGTATGCTAATAGTACTTGGAATAGCACTGAAGGAA
ATAAC--

ACTGACATACCTATCACACTTCCATGCAGAATAAAGCAAATTATAAACATGTGGCAAGAAGTAGGAAAAGCAA
TGTATGCCCCTCCAATCTCAGGAAAGATTAGATGTTCATCAAAT---
ATTACAGGGATGCTATTAACAAGAGATGGTGGTAATAATAGGAACAGT-------------------------
----

AGTAATCCCGAGATCTTCAGACCTGGAGGAGGAGATATGAGGGACAATTGGAGAAGTGAATTATATAAATATA
AAGTAGTAAAAATTGAACCATTA---GGAGTAGCACCCACCAAGGCAAAAAGAAGGGTGGTG-----------
----------------------------CAGAGAGAAAAAAGAGCA---GTGGGA---ATAGGAGCT------
---TTGTTCCTTGGGTTCTTGGGAGCA---GCAGGAAGCACTATG---GGCGCAGCGTCA---
ATGACGCTGACGGTACAG---GCCAGACAA---TTATTG---TCTGGTATAGTGCAACAGCAGAAC---
AATTTG---CTGAGA---GCTATTGAGGTGCAACAGCACCTGTTGCAACTCACAGTCTGGGGCATC
>JN024303
TTCTACTGTAATACAACAAGGCTGTTTAATTGGAGTGATACTGAAAGTGGGAATGGTACTGAAGGG-------
----

AAAAATGACACAATTATACTCCCATGCAAAATAAAACAAATTATAAACCTGTGGCAAGAAGTAGGAAAAGCAA
TGTATGCCCCTCCCATCAGCGGACCAATTAGATGCTCATCAAAT---
ATTACAGGGCTACTATTAACAAGAGATGGTGGTAGTAGTAATACG----------------------------
----

ACCACCGAGATCTTCAGACCTGGAGGAGGAGATATGAGGGACAATTGGAGAAGTGAATTATATAAATATAAAG
TAGTAAAAATTGAACCATTA---GGAGTAGCACCCACCGAGGCAAAGAGAAGAGTGGTG--------------
-------------------------CAGAGAGAAAAAAGAGCA---GTGGGA---TTTGGAGCT---------
TTCTTCCTTGGGTTCTTGGGAGCA---GCAGGAAGCACTATG---GGCGCAGCGTCA---
ATAACGCTGACGGCACAG---GCCAGACTA---TTATTG---ACTGGTATAGTGCAACAGCAGAAC---
AATCTG---CTGAGG---GCTATTGAGGCGCAACAGCATCTGTTGCAACTCACAGTCTGGGGCATC
>AY332237
TTCTATTGTAATACAACAAAACTGTTTAATAGTACTTGGAATCTGACTAGTGCTGAAGGGTTAAATGACACTG
AA---

GGAAATGACACACTCACACTCCCATGCAGAATAAAACAAATTATAAACATGTGGCAGGAAGTAGGAAAAGCAA
TGTATGCCCCTCCCATCAGAGGACAAATTAGATGTTCATCAAAT---
ATTACAGGGCTGCTATTAACAAGAGATGGTGGTAGAGAGAATGGG----------------------------
----

GACACTGAGACCTTCAGACCTGGAGGAGGAGATATGAGGGACAATTGGAGAAGTGAATTATATAAATATAAAG
TAGTAAAAATTAAACCATTA---GGAATAGCACCCACCAGGGCAAAGAGAAGAGTGGTG--------------
-------------------------CAGAGAGAAAAAAGAACA---GTGGGA---CTAGGAGCT---------
TTGTTCCTTGGGTTCTTGGGAGCA---GCAGGAAGCACTATG---GGCGCAGCGTCG---
TTGACGCTGACGGTACAG---GCCAGACAA---TTACTG---TCTGGTATAGTGCAGCAGCAGAAC---
AATTTG---CTGAGA---GCTATTGAGGCGCAACACCAGCTGTTGCAACTCACAGTCTGGGGCATC
>AY308762
TTCTACTGTAATACAACTAAACTGTTTAATAGTACTTGGGGGGTTAATGGTACTTGGAATGGGACTGAAGGGA
AT---

AATGGGAATAACACTATCATACTCCCATGCAGAATAAAACAAATTATAAACATGTGGCAGCAAGTAGGAAAAG
CAATGTATGCCCCTCCCATCAGCGGATTAATTAGATGTTCATCAAAT---
ATTACAGGGTTGATGTTAACAAGAGATGGTGGTGGTGGTAATAGC----------------------------
----

AGCACAAACGAGACCTTCAGACCTGGAGGAGGAGATATGAGGGACAATTGGAGAAGTGAATTATATAAATATA
AAGTAGTACAAATTAAACCATTA---GGAGTAGCACCCACCAAGGCAAAGAGAAGAGTGGTG-----------
----------------------------CAGAGAGAAAAAAGAGCA---GTGGGA---ATAGGAGCT------

---GTGTTCCTTGGGTTCTTGGGAGCA---GCAGGAAGCACTATG---GGCGCAGCGTCA---
ATAACGCTGACGGTACAG---GCCAGACAA---TTATTG---TCTGGTATAGTGCAACAGCAGAAC---
AATTTG---CTGAGG---GCTATTGAGGCGCAACAGCATCTGTTGCAACTCACAGTCTGGGGCATT
>AY835753
TTCTACTGTGAGACAACACAACTGTTTAATAGCACTTGGATTAGGGATAATGGTAATTGGACTAGGGCTGAGA
ATATT--

ACTGAAGGGAATAATATCACACTCCCATGCAGAATAAGACAAATTGTAAACCTGTGGCAGCAAGTAGGAAAAG
CAATGTATGCCCCTCCCATCAGTGGACCAATTAACTGTTTATCAAAT---
ATTACAGGACTGCTATTAACATGGGATGGTGGTAATAATAGCACTAGT-------------------------
----

AATAACAACACCGAGATCTTCAGACCTGGAGGAGGAGATATGAGGGACAACTGGAGAAGTGAATTATATAAAT
ATAAAGTAGTAAAAATTGAACCATTA---GGAGTAGCACCCACCAAGGCAAAGAGAAGAGTGGTG--------
-------------------------------CAGAGGGAAAAAAGAGCA---GTGGGA---CTAGGAGCT---
------TTGTTCCTTGGGTTCTTGGGAACA---GCAGGAAGCACTATG---GGCGCAGCATCA---
TTGACGCTGACGGTACAG---GCCAGACAA---TTATTG---TCTGGTATAGTGCAACAGCAGAAC---
AATTTG---CTGAGG---GCTATTGAGGCGCAACAACATCTGTTGCAGCTTACAGTCTGGGGCATC
>EU575474
TTCTACTGTAGTACAACACAACTGTTTAATAGTACTTGGATTGCAAATAAGACTGGGAATGATACTGGAGGA-
----

TCAAATGGAACTATTACACTTCCATGCAGAATAAAACAAATTGTAAACATGTGGCAGGAAGTAGGAAAAGCAA
TGTATGCCCCTCCCATCGAAGGACAAATTAGATGTTCATCAAAC---
ATTACAGGACTGCTATTATTAAGAGATGGTGGTAAGAATAACGGGACA-------------------------
----

GGAAACATGACAGAAATCTTCAGACCTGGAGGAGGAGATATGAGGGACAATTGGAGAAGTGAATTATATAAAT
ATAAAGTAGTAAAAATTGAACCATTA---GGAGTAGCACCCACCAAGGCAAAGAGAAGAGTGGTG--------
-------------------------------CAGAGAGAAAAAAGAGCG---GTGGGAACACTAGGAGCC---
------TTGTTCCTTGGGTTCTTGGGAACA---GCAGGAAGCACTATG---GGCGCAGCATCA---
CTAACGCTGACGGTACAG---GCCAGACTA---TTATTG---TCTGGTATAGTGCAACAGCAAAAC---
AATTTG---CTGAGA---GCTATTGAGGCGCAACAGCATTTGTTGCAACTCACAGTCTGGGGCATC
>JN024428
TTCTACTGTAATACAACACAGCTGTTTAATAGTATTTGGTATAATACTACAAATAGTTCTTGGAATAATAAGA
CATGG--

TCAAATAACACGATCACACTCCCATGTAGAATAAAACAAATTATAAACATGTGGCAGCAAGTAGGAAAAGCAA
TATATGCCCCTTCCATCAAAGGAAAAATTAAATGTATATCAAAT---
ATTACAGGGCTGCTATTAACAAGAGATGGTGGTGATACCAATAGT----------------------------
----

GGGACAGAGATCTTCAGACCTGGAGGAGGAGATATGAGGGATAATTGGAGAAGTGAATTATATAAATATAAAG
TAGTACAAATTGAACCATTA---GGAGTAGCACCCACCAAGGCAAAGAGAAGAGTGGTG--------------
-------------------------CAAAGAGAAAAAAGAGCA---GTGGGA---TTAGGAGCT---------
ATGTTCCTTGGGTTCTTGGGAGCA---GCAGGAAGCACTGTG---GGCGCAGCGTCA---
ATAACGCTGACGGTACAG---GCCAGACAA---TTGTTG---TCTGGTATAGTGCAACAGCAGAAC---
AATCTG---CTGAGA---GCTATTGAGGCGCAACAGCGTCTGTTGCAACTCACAGTCTGGGGCATC
>AY331284
TTCTACTGTAATACAACACAACTGTTTAATAGTACTTGGAATGCTAATGTTACTGCAAATAGCACGGAA----
----

GGAAATGACACCCTCATACTCCCATGCAAAATAAAACAAATTATAAACATGTGGCAAGAGGTAGGAAAAGCAA
TGTATGCCCCTCCCATCCAAGGGATAATTAGCTGTTCATCAAAT---
ATCACAGGGCTATTATTAACAAGAGATGGTGGAAGTGAAAACAGG----------------------------
----

CCTAACGAGACCTTCAGACCTGGAGGAGGAGATATGAGGGACAATTGGAGAAGTGAATTATATAAATATAAAG
TAGTACAAATTGAACCACTA---GGAGTAGCACCCACCAAGGCAAAAAGAAGAGTGGTG--------------
-------------------------CAGAGAGAAAAAAGAGCA---ATAGGA---ATGGGAGCG---------

TTGTTCCTTGGGTTCTTGGGAGCA---GCAGGAAGCACTATG---GGCGCAGCGTCA---
GTGACGCTGACGGTACAG---GCCAGACAA---TTATTG---TCTGGTATAGTGCAACAGCAAAAC---
AATTTG---CTGAAG---GCTATTGAGGCGCAACAGCATCTGTTGCAACTCACAGTCTGGGGCATC
>AY331285
TTCTACTGTAATACAACAAAACTGTTTAATAGTACTTGGATTAGGGAGGGTAATAATGGTACTTGGAATGGTA
CTATAGGGTTA--
----------AATGACACT------
GCAGGAAATGACACAATCATACTCCCATGTAAAATAAAACAAATTATAAACATGTGGCAGGAAGTGGGAAAAG
CAATGTATGCCCCTCCCATCAGAGGACAAATTAGATGTTCATCAAAT---
ATTACAGGGCTGATATTAACAAGAGATGGCGGTAAGGACGATAGTAATGGG----------------------
----

AGTGAGATCCTCGAGATCTTCAGACCTGGAGGAGGAGATATGAGGGACAATTGGAGAAGTGAATTATATAAAT
ATAAAGTAGTAAGAATTGAACCATTA---GGAGTAGCACCCACCAGGGCAAGGAGGAGAGTGGTG--------
-------------------------------CAAAAAGAAAAAAGAGCA---GTGGGA---TTAGGAGCT---
------ATGTTCCTTGGGTTCTTAGGAGCA---GCAGGAAGCGCTATG---GGCGCAGCGTCA---
ATGACGCTGACGGTACAG---GCCAGACAA---TTACTG---TCTGGTATAGTGCAACAGCAGAAT---
AATTTG---CTGAGG---GCTATTGAGGCGCAACAGCATATGTTGCAACTCACAGTCTGGGGCATC
>KT124745
TTCTACTGTAATACAACACAACTGTTTAATAGTACTTGGGCTTGGAATAATACTGAAGAGTCAAATAACACTG
AA---

GGAAATAGCACAACAATCACACTGCAATGCAGAATAAAACAAATTGTAAACATGTGGCAGGAAGTAGGAAAAG
CAATGTATGCCCCTCCCATTAGAGGACAAATTAAATGTTTATCAAAT---
GTTACAGGGCTGCTATTAACAAGAGATGGGGGTAATAACAATGAC----------------------------
----

ACTGAAACCTTCAGACCTGGAGGAGGAAATATAAAGGACAATTGGAGAAGTGAATTATATAAATATAAAGTAA
TAAAAATTAAACCATTA---GGAATAGCACCCACCAAGGCAAAGAGAAGAGTGGTG-----------------
----------------------CAAAGAGAAAAAAGAGCA---GTGGGAACATTAGGAGCT---------
ATGTTCCTTGGGTTCTTGGGAGCA---GCAGGAAGCACTATG---GGCGCAGCGTCA---
TTGACACTGACGGTACAG---GCCAGACTA---TTGTTG---TCTGGTATAGTGCAACAGCAAAAC---
AATTTG---CTGAGG---GCTATTGAGGCGCAACAACATCTGTTGCAACTCACGGTCTGGGGCATC
>JN562755
TTCTACTGTGATACAACACAATTGTTTAATAGTACTTGGAATGGTACTGAAGAGTCAAATGACACTGAA----
----

AGTAATATCACACTCACACTCCCATGCAGAATAAAACAAATTGTAAACATGTGGCAGGAAGTAGGAAAAGCAA
TGTATGCCCCTCCCATCAGAGGAATAATTAGCTGCTCATCAAAT---
ATTACAGGGCTGCTATTAGTAAGAGATGGTGGTAAGCCCAATAATGAGACT----------------------
----

AATAATGGTACTGAGGTCTTCAGACCTGGAGGAGGAAATATGAAGGACAATTGGAGAAGTGAATTATATAAAT
ATAAAGTAGTAAAAATTGAACCATTA---GGAGTAGCACCCACTAAGGCAAAAAGAAGAGTGGTG--------
-------------------------------CAGAGAGAGAAAAGAGCA---GTGGGA---TTAGGAGCT---
------ATGTTCCTTGGGTTCTTGGGAGCA---GCAGGAAGCACTATG---GGCGCAGCGTCA---
ATAACGCTGACGGTACAG---GCCAGACAC---TTATTG---TCTGGTATAGTGCAACAGCAAAAC---
AATTTG---CTGAAG---GCTATTGAGGCGCAACAGCATCTGTTGCAACTCACAGTCTGGGGCATC
>JN562794
TTCTACTGTAATACAACACTATTGTTTAATAGTAATTGGACTTGGAATGATACTGGAGGATATTTTACTAAAG
GGTCAAATAAC--
ACAAATGATACTGAAGGG---------
TCAAATAGCACACATATCACACTCCCATGCAGAATAAAACAAATTATAAACATGTGGCAGGGAGTAGGAAAAG
CAATGTATGCCCCTCCTATCAGTGGACTAATTAGATGTTCATCAAAT---
ATCACAGGGCTGCTACTAACAAGGGATGGTGGTATGACGAATGATACC-------------------------
----

AATACCACCGAGACCTTCAGACCTCTAGGAGGAAATATGAAGGACAATTGGAGAAGTGAATTATATAAATATA
AAGTAGTAGAAATTGAACCATTA---GGAGTAGCACCCACCAAGGCAAAGAGAAGAGTGGTG-----------
----------------------------CAGAGAGAAAAAAGAGCA---GTGGGA---ATAGGAGCT------

---TTGTTCCTTGGGTTCTTGGGAGCA---GCAGGAAGCACTATG---GGCGCAGCGTCA---
CTAACGCTGACGGTACAG---GCCAGACAA---TTATTG---TCTGGTATAGTGCAACAGCAGAAT---
AATCTG---CTGAGG---GCTATTGAGGCGCAGCAGCATCTGTTGCAACTCACAGTCTGGGGCATC
>JN562786
TTCTACTGTAATACAACAAAATTGTTTAATAGTACTTGGAATATTAATGGTACTTGGAAGGGTACA-------
----

GGAGAGCCAAATATCACACTCCCATGCAGAATAAAACAAATTGTACACATGTGGCAGGAAGTAGGAAAAGCAA
TGTATGCCCCTCCCATCAGAGGACAAATTAACTGCTCATCACAT---
ATTACAGGGCTGCTATTAACAAGAGATGGTGGGCATACTAACAATAATGGG----------------------
----

AGCAATGAGACAGAGATCTTCAGACCTGGAGGAGGAGATATGAGGGACAATTGGAGAAGTGAATTATACAAAT
ATAAAGTAGTAAGAATTGAACCATTA---GGAGTAGCACCCACCAAGGCAAAGAGGAGAGTGGTG--------
-------------------------------CAGAGAGAAAAAAGAGCA---GTGGGA---ATAGGAGCT---
------GTGTTCCTTGGGTTCTTGGGAGCA---GCAGGAAGCACTATG---GGCGCAGCATCA---
ATAACGCTGACGGTACAG---GCCAGACAA---TTATTG---TCTGGCATAGTGCAACAGCAGAAC---
AATTTG---CTGAAG---GCTATTGAGGCGCAACAGCATCTGTTGCAACTCACAGTCTGGGGCATC
>JN562773
TTCTACTGTAATTTAACACAACTGTTTAATAGTACTTGGAATGGTACAGAGTCAAATAACACTGAA-------
----

GAAGATAACACAATCACACTCCCATGCAGAATAAAACAAATCATAAACATGTGGCAGGAAGTAGGGAAAGCAA
TGTATGCCCCTCCCATCAGAGGAAATATTAGCTGTTCATCAAAT---
ATTACAGGGCTACTATTAACAAGAGATGGTGGTATTAACCAGACC----------------------------
----

AAAGACACTGAGATCTTCAGACCTGGAGGAGGAGATATGAGGGACAATTGGAGAAGTGAACTATATAAATATA
AAGTAGTAAAGGTTGAACCATTA---GGAATAGCACCCACCAAGGCAAAGAGAAGAGTGGTG-----------
----------------------------CAGAGAGAAAAAAGAGCA---GTAGGA---ATAGGAGCT------
---GTGTTCCTTGGGTTCTTGGGAGCA---GCAGGAAGCACTATG---GGCGCAGCGTCA---
GTGACGCTGACGGTACAG---GCCAGACTA---TTGTTG---TCTGGTATAGTGCAACAGCAGAAC---
AATCTG---CTGAGG---GCTATTGAGGCGCAGCAGCATCTGTTGCAACTCACAGTCTGGGGCATC
>EU289189
TTCTACTGTAATACAACAAAACTGTTTAATAATACTTGGAATAGTAATGATACTTGGACTAATACTGAAGAGT
CA---

AATGAAACTGAAATCAGGCTCCCATGCAGAATAAAACAAATTATAAACATGTGGCAGGAAGTAGGAAAGGCAA
TGTATGCCCCTCCCATCCAAGGACAAATAAGATGTTCATCAAAT---
ATTACAGGGCTGCTACTAACAAGAGATGGGGGTAACAACACGAAC----------------------------
----

AATACCGAGGTCTTCAGACCTGGAGGAGGAGATATGAGAGATAATTGGAGAAGTGAATTATATAAATATAAGG
TAGTAAAAATTGAACCATTA---GGAGTAGCACCCACTAAGGCTAAGAGAAGAGTGGTG--------------
-------------------------CAGAGAGAAAAAAGAGCA---GTGGGA---CTAGGAGCT---------
TTGTTCCTTGGGTTCTTGGGAGCA---GCAGGAAGCACTATG---GGCGCAGCGTCA---
GTGACGCTGACGGTACAG---GCCAGACAA---TTATTG---TCTGGTATAGTGCAACAGCAGAGC---
AATTTG---CTGAGG---GCTATTGAGGCGCAACAGCATCTGTTGCAACTCACAGTCTGGGGCATC
>EF593200
TTCTACTGTAATACAACACAACTGTTTAATAGTACTTGGGAGAATTCAACACAACTGTTTAATAGTACTGGG-
----

GAGAATGATACTATCACACTCCCATGCAGAATAAAACAAATTATAAACATGTGGCAGGCAGTGGGAAAAGCAA
TGTATGCCCCTCCTATCAGAGGACCAATTACATGTAAATCAAAT---
ATCACAGGGCTGATACTAACAAGAGATGGTGGGAACAGCAGCAGC----------------------------
----

AACAACGAGACCTTCAGACCTGGAGGAGGAGATATGAGGGACAATTGGAGAAGTGAATTATATAAATATAAAG
TAGTAAAAATTGAACCATTA---GGAGTAGCACCCACCAAGGCAAAGAGAAGAGTGGTG--------------
-------------------------CAGAGAGAAAAAAGAGCA---GTGGGAGTGATAGGAGCT---------

ATGTTCCTTGGGTTCTTGGGAGCA---GCAGGAAGCACTATG---GGCGCAGCGTCA---
GTGGCGCTGACGGTACAG---GCCAGACAA---TTATTG---TCTGGTATAGTGCAGCAGCAGAAC---
AATTTG---CTGAGG---GCTATTGAGGCGCAACAACATCTGTTGCAACTCACAGTCTGGGGCATC
>EF593201
TTCTACTGTAATACAACAAGACTGTTTAATAGTACGTGGAATGCCACTAGGGAGGAGAATGGTACTAAAGAGA
CAAGT--

GACAATACCACACTTATCACACTCCCATGCAGAATAAAACAATTTATAAACATGTGGCAGGAAGTAGGAAAAG
CAATGTATGCCCCTCCAATCAGTGGACAAATTAGTTGTTCATCAAAT---
ATTACAGGGCTGCTATTAACAAGAGATGGTGGTAATGACAATGGTTCT-------------------------
----

AACAACACCGAGATCTTCAGACCTGGAGGAGGAAATATGAGGGACAATTGGAGAAGTGAATTATATAGATATA
AAGTAGTAAAAATTGAACCATTA---GGAGTAGCACCCACCAAGGCAAAGAGAAGAGTGGTG-----------
----------------------------CAGAGGGAAAAAAGAGCA---ATAGGA---CTAGGAGCT------
---TTGTTTCTTGGGTTCTTGGGAGCA---GCAGGAAGCACTATG---GGCGCAGCGTCA---
CTGATGCTGACGGTACAG---GCCAGACAC---TTATTG---TCTGGTATAGTGCAACAGCAGAAC---
AATCTG---CTGAGG---GCTATTGAGGCGCAACAGCATATGTTGCAACTCACAGTCTGGGGCATT
>EU289192
TTCTACTGTAATACAACACAATTGTTTAATAGTACTTGGAATACAACACAACTTAATAGTACTTGGGATGTTA
CAGGAGGG---
-------------------TCA---
AATACTACAGAGAATATCACACTCCAATGTAGAATAAAACAAATTATAAACATGTGGCAGGAAGTAGGCAAAG
CAATGTATGCCCCTCCCATCAGTGGAATAATTAAATGTACATCAAAT---
ATTACAGGGCTACTATTAACAAGAGATGGTGGTACAAACAATACA----------------------------
----

AACGGTAGCGAGATTTTCAGACCTGGAGGAGGAGATATGAAGGACAATTGGAGAAGTGAATTATATAAATACA
AAGTAGTAAAAATTGAACCATTA---GGAGTAGCACCCACCAAGGCAAAGAGAAGAGTGGTG-----------
----------------------------CAAAGAGAAAAAAGAGCA---GTAACA---CTAGGAGCT------
---ATGTTCCTTGGGTTCTTGGGAGCA---GCAGGAAGCACGATG---GGCGCAGCGTCA---
ATGACGCTGACGGTACAG---GCCAGGCAA---TTATTG---TCTGGTATAGTGCAACAGCAGAAC---
AATCTG---CTGCGG---GCTATTGAGGCGCAACAACATCTGTTGCAACTCACAGTCTGGGGCATC
>EF593204
TTCTACTGTAATACAACAAAACTGTTTAATAGTACTTGGTCGCTTAATAGTACTGGGCTTAATAGTACTGGG-
----

AATGGTACTGAGATCATACTCCCATGTAGAATAAAACAAATTATAAACATGTGGCAGGAAGTAGGAAAAGCAA
TGTATGCCCCTCCCATCAGAGGACAAATTAGATGCTCATCAAAT---
ATTACAGGACTACTATTAACAAGAGATGGAGGGAGCAATGACACC----------------------------
----

CGCCAGGAGACTTTCAGACCTGGAGGAGGAGATATGAGAGACAATTGGAGAAGTGAATTATATAAATATAAAG
TAGTACAAATTGAACCATTA---GGATTAGCACCCACCAAGGCAAGGAGAAGAGTGGTG--------------
-------------------------CAGAGAGAAAGAAGAGCA---ATAGGA---CTAGGAGCT---------
ATGTTCCTTGGGTTCTTGGGAGCA---GCAGGAAGCACTATG---GGCGCAGCGTCA---
CTGACGCTGACGGTACAG---GCCAAACAA---TTATTG---TCTGGTATAGTGCAACAGCAGAAC---
AATCTG---CTGAGG---GCTATTGAGGCGCAACAGCATCTGTTGCAACTCACAGTCTGGGGCATC
>EU289195
TTCTACTGTAATACAACAAAGCTGTTTAATAGTACTTGGAATAATACTAATGGTGATTGGAATCTTGCTAGTG
AAGGATCAAAT--
-------------AACACT------
GCAGTAAATGGCAGTATCATACTTCCATGCAGAATTAAACAAATTATAAACATGTGGCAGGAAGTAGGAAAAG
CAATGTATGCCCCTCCCATCAAAGGACAAATTAGATGTTCATCAAAT---
ATTACAGGGCTGATATTAACAAGAGATGGTGGTGTTAATGCGACC----------------------------
----

AATGCCACTGAGATCTTCAGACCAGGAGGAGGAGATATGAGGGACAATTGGAGAAGTGAATTATACAAATATA
AAGTAGTACAAATTGAACCATTA---GGAGTAGCACCTACCAAGGCAAAGAGAAGAGTGGTG-----------
----------------------------CAGAGAGAAAAAAGAGCA---GCATTGGGAATAGGAGCT------

---CTGTTCCTTGGGTTCTTGGGAGCA---GCAGGAAGCACTATG---GGCGCAGCGTCG---
ATAACGCTGACGGTACAG---GCCAGACAA---TTATTG---TCTGGTATAGTGCAACAGCAGAAC---
AACTTG---CTGAGA---GCTATTGAGGCGCAACAGCATCTGTTGCAGCTCACAGTCTGGGGCATC
>EU575815
TTCTACTGTAATTCAACACCACTGTTTAATAGTACCTGGGCTATGAATAGAACCTGGAATGATACTGAAGGGT
CA---

AATAACAATAAAACTATCACACTCCAATGCAAAATAAAACAAATTATAAACATGTGGCAGGAAGTAGGAAAAG
CAATGTATGCCCCTCCCATCAGAGGACAAATTAGATGTTCATCAAAT---
ATTACAGGGCTGCTATTAACAAGAGATGGTGGTAATAGTGAAAATGCG-------------------------
----

AACAAGACCGAGATCTTCAGACCTGGAGGAGGAGATATGAGGGATAATTGGAGAAGTGAATTATATAAATATA
AAGTAGTAAAAATAGAACCATTA---GGAGTAGCACCCACCAAGGCAAAGAGAAGAGTGGTG-----------
----------------------------CAGAGAGAAAAAAGAGCA---GTGGGA---CTAGGAGCT------
---TTGTTCCTTGGGTTCTTGGGAACA---GCAGGAAGCACTATG---GGCGCAGCATCA---
ATAACGCTGACGGTACAG---GCCAGGCAA---TTATTG---TCTGGTATAGTGCAACAGCAGAGC---
AATCTG---CTGAGG---GCTATTGAGGCGCAACAGCATCTGTTGCAACTCACAGTCTGGGGCATC
>EU575850
TTCTACTGCAATACAACAGCACTGTTTAATAGTACTTGGAATAATACTGAATGGTCAAATGACACTAGGTCAA
ATGAC--
-------------------ACT---
AGGTCAAATGCCACTATCACACTCCCATGCAGAATAAAACAAATTATAAACATGTGGCAGGAAGTAGGAAAAG
CAATGTATGCCCCTCCCATCAAAGGACAAATTAGCTGTTCATCAAAT---
ATTACAGGGCTGTTATTAACAAGAGATGGTGGCAGTAGCAATGATAGCACG----------------------
----

AACGGGACCACCGAGATCTTCAGACCTGGAGGAGGAGATATGAGGGACAATTGGAGAAGTGAATTATATAAAT
ATAAAGTAGTACAAATTGAACCATTA---GGAGTAGCACCCAGCAGGGCAAAGAGAAGAGTGGTG--------
-------------------------------CAGAGAGAAAAAAGAGCA---ATAGGA---CTAGGAGCT---
------ATGTTCCTTGGGTTCTTGGGAGCA---GCAGGAAGCACTATG---GGCGCAGCGTCA---
CTAACGCTGACGGTACAG---GCCAGACTA---TTATTG---TCTGGTATAGTGCAACAGCAGAGC---
AATCTG---CTGAGG---GCTATTGAGGCGCAACAGCATCTGTTGCAACTCACAGTCTGGGGCATT
>EF593232
TTCTACTGTAAGTCAACAGAACTGTTCAATAGTACTTGGAATACTAATAGTACTTGGAATACTACTAGTACTT
GGAATACTACT--
-------------AGAGGG------
CCTAACAATAGAACAATCACACTCCCATGCAGAATAAAACAAATTATAAACATGTGGCAGGAAGTAGGAAAAG
CAATGTATGCCCCTCCCATCAGAGGACAAATTAGGTGTTCATCAAAT---
ATTACAGGGCTGCTATTAACAAGAGATGGTGGGAACAATGGGACC----------------------------
----

ACCGAGACCTTCAGACCTGGAGGAGGAAATATGAAAGACAATTGGAGAAGTGAATTATATAAATATAAAGTAG
TAAAAATTGAACCATTA---GGAGTAGCACCCACCAAGGCAAGGAGAAGAGTGGTG-----------------
----------------------CAAAGAGAAAAAAGAGCA---GTGGGAGCAATAGGAGCT---------
ATGTTCCTTGGGTTCTTGGGAGCA---GCAGGAAGCACTATG---GGCGCAGCGTCA---
GTGACGCTGACGGTACAG---GCCAGACTA---CTATTG---TCTGGTATAGTGCAACAGCAGAAC---
AATCTA---CTGAGG---GCTATTGAGGCGCAACAGCATATGTTGCAACTCACAGTCTGGGGCATC
>JN400469
TTCTACTGTAATACAACAAAACTGTTTAATAGTACTTGGAACATTACTGAAGGGTCAAATAACACTGAAGGGT
CAAAT--

AACACTGAAGAACTTATCACACTCCCATGCAGAATAAAACAAATTATAAACATGTGGCAGAGAGTAGGAAAAG
CAATGTATGCCCCTCCCATCCGTGGACAAATTAAATGTTCATCAAAT---
ATTACAGGGCTGTTATTAACAAGAGATGGTGGTAATAGTAGCAGC----------------------------
----

AACGGGTCAGAAATCTTCAGACCTGGAGGAGGAGATATGAGGGACAATTGGAGAAGTGAATTATATAAATATA
AAGTAGTAAAAATTGAACCATTA---GGAGTAGCGCCCACCAAGGCAAAAAGAAGAGTGGTG-----------
----------------------------CAAAGAGAAAAAAGAGCA---GTGGGA---TTAGGAGCT------

---TTGTTCCTTGGGTTCTTGGGAGCA---GCAGGAAGCACTATG---GGCGCAGCGTCA---
ATGACGCTGACGGTACAG---GCCAGACAA---TTATTG---TCTGGTATAGTGCAACAGCAGAAC---
AATTTG---CTGAGA---GCTATTGAGGCGCAACAGCATATGTTGCAACTCACAGTCTGGGGCATC
>EF593304
TTCTACTGCAATACAACAATACTGTTTAATAGTACTTGGAATGAAACTAACCTGTTTAATGCCACTGAAGGAA
AT---

AACACAGAAGTTATTACACTCCAATGCAGAATAAAACAAATTATAAACATGTGGCAGGAAGTAGGAAAAGCAA
TGTATGCCCCTCCCATCAGTGGACAAATTAACTGTTCATCAAAC---
ATTACAGGGCTGCTATTAACAAGAGATGGCGGTATTGGGAACAATATC-------------------------
----

ACCAATACCTCCGAGATCTTCAGACCTATAGGAGGAAATATGAGGGACAATTGGAGAAGTGAATTATATAAAT
ATAAAGTAGTCAAAATTGAACCAATA---GGAATAGCACCCACCAAGGCAAAGAGAAGAGTGGTG--------
-------------------------------CAGAGAGAAAAAAGAGCA---GTGGGA---ATAGGAGCT---
------ATGTTCCTTGGGTTCTTGGGAGCA---GCAGGAAGCACTATG---GGCGCAGCGTCA---
GTGACGCTGACGGTACAG---GCCAGACTA---TTATTG---TCTGGTATAGTGCAACAGCAGAAC---
AATCTG---CTGAGA---GCTATTGAGGCGCAACAACATCTGTTGCAACTCACAGTCTGGGGCATC
>AY331294
TTCTATTGCAACTCAACCAAACTATTTAATAGCATTTGGGATAATAATAAAGATAGCACTAAAACAAATGAAC
CA---

AATGATGGAAAAAATATCACACTCCCATGCAGAATAAAACAAATTATAAACATGTGGCAGGGAGTAGGAAAAG
CAATGTATGCCCCTCCCATCAGAGGACAAATTAGATGTACATCAAAT---
ATTACAGGGCTACTATTAACAAGAGATGGTGGCAAGAACAATGGCACT-------------------------
----

AATGGGACCGAGGTCTTCAGACCTGGAGGAGAAAATATGAAGGACAATTGGAAAAGTGAATTATATAAATATA
AAGTAGTAAAAATTGAACCATTA---GGAGTAGCACCCACCACGGCAAAGAGACGAGTGGTG-----------
----------------------------CAGAGAGAAAAAAGAGCA---GTGACA---CTAGGAGCT------
---CTGTTCCTTGGGTTCTTGGGAGCA---GCAGGAAGCACTATG---GGCGCAGCGTCA---
ATGACGCTGACGGTACAG---GCCAGACTA---TTATTG---TCTGGTATAGTGCAACAGCAGAAC---
AACTTG---CTGAGG---GCTATTGAGGCGCAACAACATCTGTTGCAACTCACGGTCTGGGGCATC
>KT124746
TTCTACTGTAATACAACACAACTGTTTAATAGTACTTGGAATGGAAATGATACTGGAGAGTTTAATAACACTG
GAAAA--

AATATCACATATATCACACTCCCATGCAGGATAAAACAAATTATAAACATGTGGCAGGAAGTAGGAAAAGCAA
TGTATGCCCCTCCCATCGCAGGACAAATTAGATGTTCATCAAAT---
ATTACAGGGATACTATTAACAAGAGATGGTGGTAATAGTAGCGAG----------------------------
----

GACAAGGAGATCTTCAGACCTGAAGGAGGAAATATGAGGGATAATTGGAGGAGTGAATTATACAAATATAAAG
TAGTAAAAATTGAACCATTA---GGAGTAGCACCCACCAAGGCAAAGAGAAGAGTGGTG--------------
-------------------------CAGAGAGAAAAAAGAGCA---GTGGGA---ATAGGAGCT---------
GTGTTCCTTGGGTTCTTGGGAGCA---GCAGGAAGCACTATG---GGCGCAGCGTCA---
ATGACGCTGACGGTACAG---GCCAGACTA---TTATTG---TCTGGTATAGTGCAACAGCAGAAC---
AATTTG---CTGAGG---GCTATTGAGGCGCAACAGCATCTGTTGCAACTCACAGTCTGGGGCATC
>KT124747
TTCTACTGTAATACAACACAGCTGTTTAATAGTACCTGGCCATGGCCGGATAATGATACTAATGATAACGGTA
CA---

GGCAATAGCACAATCACACTTCCATGCAAAATAAAACAAATTATAAACAGGTGGCAAGAAGTAGGAAAAGCAA
TGTATGCCCCTCCCATCAGTGGACAAATTAGATGTACATCAAAT---
ATTACAGGACTGATATTAACAAGAGATGGTGGTCCCACAAATGATACC-------------------------
----

AACAACACCGAGGTCTTCAGACCTGGAGGAGGAGATATGAGGGACAATTGGAGAAGTGAATTATATAAATATA
AAGTAGTAAAAATTGACCCATTA---GGAGTAGCACCCACCAAGGCAAAGAGAAGAGTGGTG-----------
----------------------------CAAAGAGGAAAAAGAGCA---GTGGGG---ATAGGAGCT------

```
---ATGTTCCTTGGGTTCTTGGGAGCA---GCAGGAAGCACTATG---GGCGCAGCGTCA---
CTAACGCTGACGGTACAA---GCCAGACTA---CTATTG---TCTGGTATAGTGCAACAGCAGAAC---
AATCTG---CTGAGG---GCTATTGAGGCGCAACAGCATATGTTGCAACTCACAGTCTGGGGCATT
>EF593208
TTCTACTGTAACTTAACAAGGCTGTTTAATAATACTTGGTATAATGAGAGCAGTAGTACTTGGAAT-------
-------------------------------------------------------------------------
----------------------------
GTAAATGGCACCATCACACTCCCATGCAGGATAAAACAAATTATAAACATGTGGCAGGAAGTAGGAAAAGCAA
TGTATGCCCCTCCCATCATGGGATCAATTAACTGTTCATCAAAT---
ATTACAGGGCTACTATTAACAAGAGATGGTGGTAACCATAACAAT----------------------------
-------------------------------------------------------------------------
----------
GACAGCAACGAGACCTTCAGACCTGGAGGAGGAGATATGAGGGACAATTGGAGAAGTGAATTATATAAATATA
AAGTAGTAAAAATTGAACCATTA---GGAGTAGCACCCACCAAGGCAAAGAGAAGAGTGGTG-----------
----------------------------CAAAGAGAAAAAAGAGGA---GTGACA---CTAGGAGCT------
---ATGTTCCTTGGGTTCTTGGGAGCA---GCAGGAAGCACTATG---GGCGCAGCGTCA---
ATGGCGCTGACGGTACAG---GCCAGACAA---TTATTG---TCTGGTATAGTGCAACAGCAGGAC---
AATTTG---CTGAGG---GCTATTGAGGCGCAACAGCATATGTTGCAACTCACAGTCTGGGGCATC
>AY560107
TTCTACTGTAATACAGCAAAACTGTTTAATAGCATTTGGAATAATAGCAGTTGGAGTGGTACTGAAAGGTCAA
GT-----------------------------------------------------------------------
-------------------------
AACGAGACAAGGAACATCATACTCCCATGTAAAATAAGACAAATTGTAAACCTGTGGCAGGAAGTAGGAAAAG
CAATGTATGCCCCTCCCATCAGAGGACAAATTAGATGTTCATCAAAT---
ATTACAGGGATACTATTAACAAGAGATGGTGGTAATAACACAGAA----------------------------
-------------------------------------------------------------------------
-------------
GGGAACGAGACCTTCAGACCTGGAGGAGGAGATATGAGGGACAATTGGAGAAGTGAATTATATAAATATAAAG
TAGTAAAAATTGAACCATTA---GGAGTAGCACCCACCAAGGCAAAGAGAAGAGTGGTG--------------
-------------------------CAGAGAGAAAAAAGAGCA---GTGGGA---TTAGGAGCT---------
TTGTTCCTTGGGTTCTTGGGAGCA---GCAGGAAGCACTATG---GGCGCAGCGTCA---
GTAACGCTGACGGTACAG---GCCAGACAA---TTATTG---TCTGGTATAGTGCAGCAGCAAAAC---
AATTTG---CTGAGG---GCTATTGAGGCGCAACAGCACCTGTTGCAACTCACAGTCTGGGGCATC
>AY560108
TTTTACTGTAATACAACACCACTGTTTAATAGTACTTGGGTTAATGGTACTAGGAATGATACTGCAAGGCCAA
AT-----------------------------------------------------------------------
----------------------------
GACACTGACACAATTAAACTTCCATGCAGAATAAAACAAATTATAAACCTGTGGCAGGAAGTAGGAAAAGCAA
TGTATGCTCCTCCCATCGAAGGACAAATTAGCTGTTCATCAAAT---
ATTACAGGGCTACTATTAACAAGAGATGGTGGTATTAACAGCAGCAAT-------------------------
-------------------------------------------------------------------------
-------
ACCAATACCACCGAGACCTTCAGACCTGGAGGAGGAGATATGAGGGACAATTGGAGAAATGAATTATATAAAT
ATAAAGTAGTAAGAATTGAGCCATTA---GGAGTAGCACCCACCAAGGCAAAGAGAAGAGTGGTG--------
-------------------------------CAGAGAGAAAAAAGAGCA---GTGGGA---ATAGGAGCT---
------ATGTTCCTTGGGATCTTGGGAACA---GCAGGAAGCGCTATG---GGCGCAGCGTCA---
ATGACGCTGACGGTACAG---GCCAGACAA---TTATTG---TCTGGTATAGTGCAACAGCAGAAC---
AATATG---CTGAGG---GCTATTGGGGCGCAACAGCACCTGTTGCAACTCACAGTCTGGGGCATC
>AY560109
TTCTACTGTAATACAACACAACTGTTTAATAGCACTTGGTATAGTAATAGTACTGGGAATAGTACTGGAGGG-
-------------------------------------------------------------------------
----------------------------
TCAAATGACACTATCACACTCCCATGTAGAATAAAACAAATTATAAACATGTGGCAGGAAGTAGGAAGAGCAA
TGTACGCCCCTCCCATCAGTGGAAAAATTAGTTGTTCATCAAAT---
ATTACAGGGCTACTGTTAACAAGAGATGGTGGTATTAACATGACC----------------------------
-------------------------------------------------------------------------
----------
AATACCACCGAGACCTTCAGACCTGGAGGAGGAGATATGAGGGACAACTGGAGAAGTGAATTATATAAATATA
AAGTAGTACAAATTGAACCATTA---GGAATAGCACCCACCAAGGCAAAGAGAAGAGTGGTG-----------
----------------------------CAGAGAGAAAAAAGAGCA---GTGGGA---ATAGGAGCT------
```

---CTGTTCCTTGGGTTCTTAGGAGCA---GCAGGAAGCACTATG---GGCGCAGCGTCA---
GTGACGCTGACGGTACAG---GCCAGGCAC---TTATTG---CCTGGTATAGTGCAGCAGCAGAAC---
AATCTG---CTGAAG---GCTATTGAGGCGCAACAGCATCTGTTGCAACTCACAGTCTGGGGCATT
>AY560110
TTCTACTGTGATTCAACACCACTGTTTAATAGTACTTGGAATAGTACTACTTGGAATGATACTACTGACACTG
AA---

GGAAATGAAACAATCACACTCAAATGTAGAATAAAACAAATTATAAACATGTGGCAGGAAGTAGGAAAAGCAA
TGTATGCCCCTCCTATCGAGGGAGTAATTAAATGTTTATCAAAT---
ATTACAGGGCTGCTATTAGTAAGAGATGGT---ATAAGCAACAGT----------------------------
----

AGTGACGAGACCTTCAGACCTGGAGGAGGAAATATGAAGGACAATTGGAGAAGTGAATTATATAAATATAAAG
TAGTAAAAATTGAACCATTA---GGAGTAGCACCCACCAAGGCAAGGAGAAGAGTGGTG--------------
-------------------------CAAAGAGAAAAAAGAGCA---GTGGGAACACTGGGAGCT---------
ATGTTCCTTGGGTTCTTGGGAGCA---GCAGGAAGCGCTATG---GGCGCAGCGTCA---
GCGACGCTGACGGTACAG---GCCAGACAA---TTATTG---TCTGGTATAGTGCAACAGCAAAAC---
AACTTG---CTGAGA---GCTATTGAGGCGCAACAGCATATGTTGCAACTCACAGTCTGGGGCATC
>EF175212
TTCTACTGTAATACAACAAAACTGTTTAATAGTAATTGGACTTTGGAGGGTAATGTGACATATACCACAAATA
AC---

ACAAATGGCACAAATATCATACTCCCATGCAGAATAAAACAAATTATAAACATGTGGCAGGGAGTAGGAAAAG
CAATGTATGCCAATCCCATCAGTGGACTAATTAACTGTACATCAAAG---
ATTACAGGGCTGCTATTAACAAGAGATGGGGGTACAGAGGGTAATGGCAGCAACGGGACCGGGACAGAGATCG
AGAAC--

AGGACCGAGATCTTCAGACCTGGGGGAGGAAATATGAAGGACAATTGGAGAAGTGAATTATATAAATATAAAG
TAGTAAGAATTGAACCAATA---GGGATAGCACCCACCAAGGCAAAGAGAAGAGTGGTG--------------
-------------------------CAGAGAGAAAAAAGAGCA---GTGGGGGTGCTGGGAGCT---------
ATGTTCCTTGGGTTCTTGGGAACA---GCAGGAAGCACTATG---GGCGCAGCATCA---
GTAACGCTGACGGTACAG---GCCAGACAA---TTATTG---TCTGGTATAGTGCAACAGCAGAAC---
AATCTG---CTGCAG---GCTATTGAGGCGCAACAGCATCTGTTGCAGCTCACAGTCTGGGGCATC
>EU577426
TTCTACTGTAATTCAACAGAACTGTTTAATAGTACTTGGAATAATACTAATACTTGGAATGATACTGGAGAG-
----

TCAAATGAAACTATCATACTTCCATGTAGAATAAAACAATTTATAAACATGTGGCAGGGAGTAGGAAAAGCAA
TGTATGCCCCTCCCATCAGAGGAGAAATTAACTGCTCATCACAA---
ATTACAGGGCTGCTATTAACAAGAGATGGTGGTAATGATAATACCACT-------------------------
----

AACATGATTGAGGTCTTCAGACCTGGAGGAGGAAATATGAAGGACAATTGGAGAAGTGAATTATCTAAATATA
AAGTAGTAAAAATTGAGCCATTA---GGAGTAGCACCCACTAAGGCAAAGAGAAGAGTGGTG-----------
----------------------------CAGAGAGAAAAAAGGGGA------------ATAGGAGCT------
---GTGTTCCTTGGGTTCTTGGGAGCA---GCAGGAAGCACTATG---GGCGCAGCGTCA---
GTGGCGCTGACGGTACAG---GCCAGACAA---TTATTG---TCTGGTATAGTGCAACAGCAGAAC---
AATCTG---CTGAAG---GCTATTGAGGCGCAACAGCATCTGTTGCAACTCACAGTCTGGGGCATC
>EF593284
TTCTACTGTAATTCAACACAATTGTTTAATAGTACTTGGTATGGTAATGAGACTATTGAAGGGTTAAATGAC-
----

ACTACAGAAAATATCACACTCCCATGCAAAATAAAACAAATTATAAACATGTGGCAGGAAGTAGGAAAAGCAA
TGTATGCCCCTCCCATCAGTGGACAAATTAGATGTTCATCAAAT---
ATTACAGGGCTGATATTAACAAGAGATGGTGGTTTTAGCAACAATACCCAG----------------------
----

GAGAAGAACAAGACCGAGATCTTCAGACCTGGGGGAGGAGATATGAGGGACAATTGGAGAAGTGAATTATATA
AATATAAAGTAGTGCAAGTTGAACCATTA---GGACTAGCACCCACCAAGGCAAAGAGAAGAGTGGTG-----
----------------------------------CAGAGAGAAAAAAGAGCA---GTGGGT---

TTAGGAGCT---------TTGTTCCTTGGGTTCTTGGGAGCA---GCAGGAAGCACTATG---
GGCGCAGCGTCA---GTGACGCTGACGGTACAA---GCCAGACAA---TTATTG---
TCTGGTATAGTGCAACAGCAGAAC---AACTTG---CTGAGA---
GCTATTGAGGCGCAACAGCATCTGTTGCAACTCACAGTCTGGGGCATC
>EF593310
TTCTACTGTAATTCAACACCACTGTTTGATAGTATTTGGAATAGTACTGGGAATGATACTGAAGGGTCAAATA
GT---

ACTGAAAAAGATATCATACTCCAATGCAGAATAAAACAAGTTATAAATATGTGGCAGCAAGTAGGAAAAGCAA
TGTATGCCCCTCCCATCAAAGGACAAATTAACTGTACATCAAAT---
ATTACAGGGCTGCTATTAACAAGAGATGGTGGTAACAGGGGCGAG----------------------------
----

TCTGAGACCTTCAGACCTGGAGGAGGAGATATGAAGGACAATTGGAGAAGTGAATTATATAAATATAAAGTAG
TAAAAATTAAACCATTA---GGAATAGCACCCACCAAGGCAAAGAGAAGAGTGGTG-----------------
----------------------CAGAGAGAAAAGAGATCA---GTGACA---TTAGGAGCT---------
ATGTTCCTTGGGTTCTTGGGAGCA---GCAGGAAGCACTATG---GGCGCAGCGTCA---
ATGGCGCTGACGGTACAA---GCCAGACAA---TTATTG---TCTGGTATAGTGCAACAGCAGAAC---
AATTTG---CTGAAA---GCTATTGAGGCGCAACAGCATCTGTTGCAACTCACAGTCTGGGGCATC
>EF593312
TTCTACTGTAATTCAACACAACTGTTTAATAGTACTTGGTATGGTAATGAGACTGAGACTGGAGGGACAAATG
AC---

ACTATAGGAAATATCACACTCCCATGCAGAATAAGACAAATTATAAACATGTGGCAGGAAGTAGGAAAAGCAA
TGTATGCCCCTCCCATCAGAGGACAAATTAGTTGTTCATCAAAA---
ATTACAGGGCTGCTATTAACAAGGGATGGTGGTAACAACAATGAGACC-------------------------
----

AATACCACCGAGACATTCAGACCAGGAGGAGGAGATATGAGGGATAATTGGAGAAGTGAATTATATAAATATA
AAGTAGTCAAAATTGAGCCACTA---GGAATAGCACCCACCAAGGCAAAGAGAAGAGTGGTG-----------
----------------------------CAAAGAGAAAAAAGAGCA---GTGGGAACAATAGGAGCT------
---ATGTTCCTTGGGTTCTTGGGAGCA---GCAGGAAGCACTATG---GGCGCAGCGTCA---
ATGACGCTGACGGTACAG---GCCAGACTA---TTATTG---TCTGGTATAGTGCAACAGCAGAAC---
AATTTG---CTGAGA---GCTATTGAGGCGCAACAGCATATGTTGCAACTCACAGTCTGGGGCATC
>EF593314
TTCTACTGTGATTCAACACAACTATTTAATAGTACTTGGACTGAAGGGGCGACTAACAATACA----------
----

GGAAATGACACAATCATACTCCCATGCAGAATAAGACAAATTATAAACATGTGGCAGGAGGTAGGAAAAGCAA
TGTATGCCCCTCCCATCAGAGGACAAATTAGATGTTCATCAAAT---
ATTACAGGGCTGCTATTAACAAGAGATGGTGGTAATAGCAGCAAA----------------------------
----

ACAACCCCTGAGATCTTCAGACCTGGAGGAGGAGATATGAGGGACAATTGGAGAAGTGAATTATATAAATATA
AAGTAGTAAAGATTGAACCATTA---GGAATAGCACCCACCAAGGCAAAGAGAAGAGTGGTG-----------
----------------------------CAAAGAGAAAAAAGAGCA---GTGGGG---ATAGGAGCT------
---GTGTTCCTTGGGTTCTTGGGAACA---GCAGGAAGCACTATG---GGCGCAGCGGCA---
GTAACGCTGACGGTACAA---GCCAGACTA---TTATTG---TCTGGTATAGTGCAACAGCAAAAC---
AACTTG---CTGAGG---GCTATTGAGGCGCAACAGCATCTGTTGCAACTCACAGTCTGGGGCATC
>EF593316
TTCTATTGTAATTCAACACAACTGTTTAATAGTACTTGGCCTACTAATAGTACTTGGAATGCTACAGAAGGG-
----

TCAAATAGCACTATCACACTCCCATGTAAAATAAAACAAATTATAAACATGTGGCATGAAGTAGGAAAAGCAA
TGTATGCCCCTCCCATCAGAGGACAAATTAGATGTTCATCAAAT---
ATTACAGGGATATTTTTAACTAGAGATGGTGGTAAAGAGAAGAAC----------------------------
----

AACACCGAGATCTTCAGACCTGGAGGAGGAGATATGAGGGACAATTGGAGAAGTGAATTATATAAATATAAAG
TAGTAAAAATTGAACCATTA---GGAATAGCACCCACCAAGGCAAAGAGAAGAGTGGTG--------------

-------------------------CAGAGAGAAAAAAGAGCG---GGA------ATAGGAGCA---------
CTGTTCCTTGGGTTCTTGGGAGCA---GCAGGAAGCACTATG---GGCGCAGCGTCA---
ATGACGCTGACGGTACAG---GCCAGACTC---TTATTG---TCTGGTATAGTGCAACAGCAGAAC---
AATCTG---CTGAGA---GCTATTGATGCGCAACAGCATCTGTTGCAACTCACAGTCTGGGGCATC
>EF593318
TTCTACTGTGATACAACAAAACTGTTTAATAGTACTTGGCCTTGGAATGGTACTGAAGGGCCAAATATCACTG
GAGGAGAT---
-------------------ACT---
AACATTAACATTAACATTACACTCCCATGCAGAATAAGACAAATTGTAAACATGTGGCAGGAAGTAGGAAAAG
CAATGTATGCCCCTCCCATCAGAGGACAAATTAGATGTTCATCAAAT---
ATCACAGGGTTGTTGTTAACAAGAGATGGAGGTAACAATAGTACA----------------------------
----

AACGAGACCTTCAGACCTGGAGGAGGAAATATGAAGGACAATTGGAGAAGTGAATTATATAAATATAAAGTAG
TAAGAATTGAACCATTA---GGAGTAGCACCCACCGAGGCAAGGAGAAGAGTGGTG-----------------
----------------------CAGAGAGAAAAAAGAGCA---GTAACA---CTAGGAGCT---------
ATGTTCCTTGGGTTCTTGGGAGCA---GCAGGAAGCACTATG---GGCGCAGCGTCA---
ATGGCGCTGACGGTACAG---GCCAGACAA---TTATTG---TCTGGTATAGTGCAACAGCAGAAC---
AATTTG---CTGAGA---GCTATTGAGGCGCAACAACATCTGTTGCAACTCACAGTCTGGGGCATC
>JN786786
TTCTACTGTAATACAACAGAACTGTTTAATAGCACTTGGTATACTAATGGGTCAAGTAATAATATTGAAGGA-
----

AATGACACAATAATCACACTCCCATGCAGAATAAAACAAATTGTACGCATGTGGCAGGGAGTAGGAAAAGCAA
TGTATGCCCCTCCCATCGATGGAATAATTAGATGTACCTCAAAT---
ATTACAGGGCTGCTATTAACAAGGGATGGTGGTATTGATAATAACACG-------------------------
----

ACCGATCCCGAGATCTTCAGACCTGAAGGAGGAGATATGAAAAACAATTGGAGAAGTGAATTATATAGATATA
AAGTAATAAAAATTGAACCATTA---GGAGTAGCACCCACCAAGGCAAGGAGAAGAGTGGTG-----------
----------------------------CAGAGAGAAAAAAGAGCA---GCA------CTAGGAGCT------
---GTGTTCCTTGGGTTCTTGGGAGCA---GCAGGAAGCACTATG---GGCGCAGCGTCA---
ATAACGCTGACGGTACAG---GCCAGACTA---TTATTG---TCTGGTATAGTGCAACAGCAGAGT---
AATTTG---CTGAGA---GCTATTGAGGCGCAACAGCATCTGTTGAAACTCCCAGTCTGGGGCATT
>JN599165
TTCTACTGTAATACAGCAGGACTGTTTAATAGTACTTGGAATAGTACTTGGAGTTTAAATGACACTAAAAGAA
AT---

GGAAATGAAAATATCACACTCCCATGCAGAATAAAACAAATTATAAACATGTGGCAGGGAGTAGGAAAAGCAA
TGTATGCCCCTCCCATCAGTGGACAAATTAGATGCTCATCAAAT---
ATTACAGGGTTGCTATTAACAAGAGATGGTGGGAACGGAACATCACC--------------------------
----

AACACCACCGAGACCTTCAGACCTGGAGGAGGAGATATGAGGGACAATTGGAGAAGTGAATTATATAAATATA
AAGTAGTAAAAATTGAACCATTA---GGAATAGCACCCACCAAGGCAAAGAGAAGAGTGGTG-----------
----------------------------CAGAGAGAAAAAAGAGCA---GTGGGA---ATAGGAGCT------
---GTGCTCCTTGGGTTCTTGGGAGCA---GCAGGAAGCACTATG---GGCGCAGCGTCA---
GTGACGCTGACGGTACAG---GCCAGACTA---TTATTG---TCTGGTATAGTGCAACAGCAGAAC---
AATTTG---CTGAGA---GCTATTGAGGCGCAACAGCATTTGTTGCAGCTCACAGTCTGGGGCATC
>KT124759
TTCTACTGTAATACAACAGACCTGTTTAATAGAACTGTGGAAAAAAATATTACTTGGCCTAGGGCAAACGACA
CAGAAGGA---

AATGAAAATGAAACAATCACACTCCAATGCAGAATAAAACAAATTATAAACATGTGGCAGGAAGTAGGAAAAG
CAATGTATGCCCCTCCTATCAGAGGACAAATTAAATGTACATCAAAC---
ATTACAGGACTACTATTAACAAGAGATGGTGGGAATAGTAATGATACT-------------------------
----

AATGATACCGAGACCTTTAGACCTGGAGGAGGAGATATGAGGGACAATTGGAGAAGTGAATTATATAAATATA
AAGTAGTAAAAATTGAACCATTA---GGAATAGCACCCACCAAGGCAAAGAGAAGAGTGGTG-----------

----------------------------CAGAGAGAAAAAAGAGCA---GTGGGA---CTAGGAGCA------
---CTGTTCCTTGGGTTCTTGGGAGCA---GCAGGAAGCACTATG---GGCGCAGCGTCA---
TTGACGTTGACGGTACAG---GCCAGACAA---TTATTG---TCTGGTATAGTGCAACAGCAGAAC---
AATCTG---CTGAGG---GCTATTGAGGCGCAACAGCATATGTTGCAACTCACAGTCTGGGGCATT
>JN562762
TTCTACTGTAATTCAACACAACTGTTTAGTAGCAGTTGGAATAGTACTAGTACTTGGAATAGTACTACAGTGT
CAAAT--

GATACTGACAAAAATATTACACTCCCATGTAGAATAAAACAATTTATAAACATGTGGCAGGGAGTAGGAAAAG
CAATGTATGCCCCTCCCATCAGCGGAATAATCAGCTGTTCATCAAAT---
ATTACAGGGCTGCTATTAACAAGAGATGGGGGTAATAGCAAGAATGGGAGTACT------AACAGTAGC---
AGT--
GATGGGAGTAATAACAGTAAC---------
AAGAGCGAGATCTTCAGACCTGGAGGAGGAGATATGAGGGACAATTGGAGAAGTGAATTATATAAATATAAAG
TAGTACAAATTGAACCATTA---GGAGTAGCACCCACCAGGGCAAAGAGAAGAGTGGTG--------------
-------------------------CAAAGAGAAAAAAGAGCA---GTGGGA---ATAGGAGCT---------
TTGTTCCTTGGGTTCTTGGGAGCA---GCAGGAAGCACTATG---GGCGCAGCATCA---
ATAACGCTGACGGTACAG---GCCAGACAG---TTATTG---TCTGGTATAGTGCAACAGCAGAAC---
AATTTG---CTGAGG---GCTATTGAGGCGCAACAACATATGTTGCAACTCACAGTCTGGGGCATC
>KT124776
TTCTACTGTAATACAACACAACTGTTTAATAGTATCTGGAGTAGTAATGGTACCAGGAATAGTACAAATAGCA
CTAGG--

ACAAATAGTACTATCATCACACTCCCATGCAAAATAAAACAAATTATAAACATGTGGCAGGAAGTAGGAAAAA
CAATGTATGCCCCTCCCATCAGAGGACAAATTAGCTGTGTATCAAAT---
ATTACAGGGCTATTGTTAACAAGAGATGGTGGTACAAATATCACC----------------------------
----

AACGAAACCTTCAGACCTGGAGGAGGAGACATGAGGGACAATTGGAGAAGTGAATTATATAAATATAAAGTAG
TAAAAATTGAACCATTA---GGAGTAGCACCTACCAAGGCAAAGAGAAGGGTGGTG-----------------
----------------------CAAAGAGAAAAAAGAGCA---GCGGGA---CTAGGAGCT---------
CTGTTCCTTGGGTTCTTGGGAGCA---GCAGGAAGCACTATG---GGCGCAGCGTCA---
ATAACGCTGACGGTACAG---GCCAGACAA---TTACTG---TCTGGTATAGTGCAACAGCAAAAT---
AATTTG---CTGAGG---GCTATTGAGGCGCAACAACATATGTTGCAACTCACAGTCTGGGGCATC
>KT124798
TTCTACTGTAATACAACACCACTGTTTAATAGTACTTGGAAGCGTACTTGGAATAATGTTAAAGGGAACAATG
AT---

ACTAATAAAAATATCACACTCCGATGTAGGATAAAACAAATTATAAACATGTGGCAGAAAGTAGGAAAAGCAA
TGTATGCCCCTCCCATCCAAGGAGAAATTAGATGTTCATCAAAT---
ATTACAGGGCTGCTATTAACAAGGGATGGTGGTATTAGCAACAGCACGAAT----------------------
----

GACACCACCGAGATCTTCAGACCTGAAGGAGGAGATATGAGGGATAATTGGAGAAGTGAATTATATAAATATA
AAGTAGTAAGAATTGAACCAATA---GGAGTAGCACCCACCAAGGCAAAGAGAAGAGTGGTG-----------
----------------------------CAGAGAGAAAAAAGAGCA---GTGGGC---TTCGGAGCT------
---ATGTTCCTTGGGTTCTTGGGAGCA---GCAGGAAGCACTATG---GGCGCAGCATCA---
TTAACGCTGACGGTACAG---GCCAGACAA---TTATTG---TCTGGTATAGTGCAACAGCAGAAC---
AATTTG---CTGAGA---GCTATTGAGGCGCAACAGCATCTGTTGCAGCTCACAGTCTGGGGCATC
>GU331247
TTCTACTGTAATACAACAGAACTGTTTAATAGTACTTGGAATGGTACTGAAGTGGTAAATATCAATGAA----
----

GGAAATGACAATATCACTCTTCCATGCAGAATAAAACAAATTATAAACATGTGGCAGGAAGTAGGAAAAGCAA
TGTATGCCCCTCCCATCAGAGGACCAATTAAATGTTCATCGAAT---
ATTACAGGGCTAATATTAACAAGAGATGGTGGTACTAATGACACC----------------------------
----

ACCGAGGTCTTCAGACCTGCAGGAGGAGATATGATGGACAATTGGAGAAGTGAACTATATAAATATAAAGTGG
TGAAAATTGAACCATTA---GGAGTAGCACCCACCAAGGCAAAGAGAAGAGTGGTG-----------------

----------------------CAGAGAGAAAAAAGAGCA---ATAACA---ATAGGAGCG---------
GTGTTCCTTGGGTTCTTGGGAACA---GCAGGAAGCACTATG---GGCGCAGCGTCC---
GTGGCGCTGACGGTACAG---GCCAGACAA---TTATTG---TCTGGTATAGTGCAACAGCAAAGC---
AACTTG---TTGAGA---GCTATTGAGGCGCAACAGCATATGTTGCAACTCACAGTCTGGGGCATC
>JN786831
TTCTACTGCAATACTTCACCACTGTTTAATAGTATTTGGAATAGTAGTACTTGGAATGACACAAATAATACTG
AA---
-------------------GGG---
GCAAATGACACTGTTATCACACTTCGATGCAGGATAAAACAAATTATAAACATGTGGCAGGCAGTGGGAAAAG
CAATGTATGCCCCTCCCATTGAAGGACAAATTAGGTGTTCATCAAAT---
ATTACAGGGCTGATATTAACAAGAGATGGTGGTAAAGAAAACGCTACC-------------------------
----

AGGAACAACGAGACCTTCAGACCTGCAGGAGGAGATATGAGGGACAATTGGAGAAGTGAATTATATAAATATA
AAATAGTACAAATTGAACCATTA---GGAGTAGCACCCACCAAGGCAAAGAGAAGAGTGGTG-----------
----------------------------CAGAGAGAAAAAAGAGCA---GTGGGA---ATGGGAGCT------
---TTGTTCCTTGGGTTCTTGGGAGCA---GCAGGAAGCACTATG---GGCGCAGCGTCA---
CTGACGCTGACGGTACAG---GCCAGACAA---TTATTG---TCTGGTATAGTGCAACAGCAGAAC---
AATTTG---CTGAGG---GCTATTGAGGCGCAACAACATCTGTTGCAACTCACAGTCTGGGGCATC
>JQ403100
TTCTACTGTAAGACAGCACAACTGTTTAATAGTACTTGGAATGTGTTTAATAGTACTTGGAATGGTATTAAAA
GGGTAAAT---
-------------AGCACT------
GAAGGAAATGACACTATCACACTCCAATGCAGAATAAAACAAATTATAAACATGTGGCAGGAAGTAGGAAAAG
CAATGTATGCCCCTCCCATCAGAGGACAGATTAGATGCTCATCAAAT---
ATTACAGGGCTGCTGTTAACAAGAGATGGTGGCAATAACGAGAACGGA-------------------------
----

ACCCCCGAGGTCTTCAGACCTGGAGGAGGAGATATGAGGGACAATTGGAGAAGTGAATTATATAAATATAAAG
TAGTAAAAATTGAACCATTA---GGAATAGCACCCACCAAGGCAAAGAGAAGAGTGGTG--------------
-------------------------CAGAGAGAAAAAAGAGCA---GTGGGA---CTGGGAGCT---------
ATGTTCCTTGGGTTCTTGGGAGCA---GCAGGAAGCACTATG---GGCGCAGCGTCA---
GTAACGCTGACGGTACAG---GCCAGACAA---TTATTG---TCTGGTATAGTGCAACAGCAGAAC---
AATTTG---CTGAGA---GCTATTGAGGCGCAACAGCATATGTTGCAACTCACAGTCTGGGGCATT
>EU576617
TTCTACTGTAATACATCACCACTGTTTAATAGTACTTGGAATAGTACTACTAATGGGAATGAGACTGAAGGGT
CA---

AATAACGGAACTATCACACTCCAATGCAAAATAAAACAAATTATAAACATGTGGCAGGAAGTAGGGAAAGCAA
TGTATGCCCCTCCCATCAGAGGACAAATTAGATGTATATCAAAT---
ATTACAGGGCTGCTAATAACAAGAGATGGTGGTAATAACGAGACC----------------------------
----

GACAGCACAGAGAGCTTCAGACCTGGAGGAGGAGATATGAGGGACAATTGGAGAAGTGAATTATATAAATATA
AAGTAGTAAAAATTGAACCATTA---GGAGTAGCACCCACCAAGGCAAAGAGGAGAGTGGTG-----------
----------------------------CAGAGAGAAAAAAGAGCA---GTGGGA---TTAGGAGCT------
---CTGTTCCTTGGGTTCTTGGGAGCA---GCAGGAAGCACTATG---GGCGCAGCGTCA---
ATGACGCTGACGGTACAG---GCCAGACAA---TTGCTG---TCTGGTATAGTGCAACAGCAGAAC---
AATCTG---CTGAGG---GCTATTGAGGCGCAACAGCACCTGTTGCAACTCACAGTCTGGGGCATC
>AY331296
TTCTACTGTAATACAACACAACTGTTTAATAGTACTTGGAATGGTACCGAAGAGTTAAATAGTACA-------
----

AATGACAAAATTATCACACTCCCATGCAGAATAAAACAAATTATAAACATGTGGCAGGAAGTAGGAAAAGCAA
TGTATGCCCCTCCTATCAGAGGACAAATTAGATGTTCATCAAAT---
ATTACAGGGCTGCTATTAACAAGAGATGGTGGTAATAATAACAACAGC-------------------------
----

GATACTCCTGAGACCTTCAGACCTGGAGGAGGAGATATGAGGGATAATTGGAGAAGTGAATTATATAAATATA
AAGTAGTAAAAGTTGAACCATTA---GGAATAGCACCCACCAAGGCAAAGAGAAGAACGGTG-----------

----------------------------CAGAGAGAAAAAAGAGCA---GTGGGA---ATAGGAGCT------
---GTGTTCCTTGGGTTCTTGGGAGCA---GCAGGAAGCACTATG---GGCGCAGCGTCA---
ATAACGCTGACGGTACAG---GCCAGACTA---TTATTG---TCTGGTATAGTGCAACAGCAGAAC---
AATCTG---CTGAGG---GCTATTGAGGCGCAACAGCACATGTTGCAACTCACAGTCTGGGGCATC
>EF593286
TTCTACTGTAATACAACAGAACTGTTTAATAGTACTTGGTATGGTAATGATACT-------------------
----

AGGAATGATACTATCACACTCTCATGCAGAATAAAACAAATAATAAACATGTGGCAGGAAGTAGGAAAAGCAA
TGTATGCCCCTCCCATCAAAGGACAAATTAGATGCTCATCAAAT---
ATTACAGGGCTACTATTAACAAGGGATGGTGGTAATGACACGGAC----------------------------
----

GGGAAAGAGACCTTCAGACCTGTAGGAGGAAATATGAAGGACAATTGGAGAAGTGAATTATATAAATATAAAG
TAGTAAGAATTGAACCATTA---GGGGTAGCACCCACCAAGGCAAAGAGAAGAGTGGTG--------------
-------------------------CAGAGAGAAAAAAGAGCA---GTGGGA---TTCGGAGCT---------
TTGTTCCTTGGGTTCTTGGGAGCA---GCAGGAAGCACTATG---GGCGCAGCGTCA---
CTAACGCTGACGGTACAA---GCCAGACAG---TTACTG---TCTGGTATAGTGCAACAGCAAAAC---
AATCTG---CTGAGG---GCTATTGAGGCGCAACAGCATCTGTTGCAACTCACAGTCTGGGGCATT
>EU577525
TTTACTGTAATACAACAAACCTGTTTAGTAGTACTTGGTATGGGAATGAAACTGAAGAGCCAAATAATACTA
CA---

GGAAATAGCACAACAATCACACTCCCATGCAGAATAAAACAAATTATAAACATGTGGCAGCAAGTAGGAAAAG
CAATGTATGCCCCTCCCATCAGTGGGCCAATTAGCTGTTCATCAAAT---
ATTACAGGGCTGATATTAACAAGAGATGGTGGTAACACGAACGAG----------------------------
----

ACCACCGAAACCTTCAGACCTGGAGGAGGAAATATGAGAGATAATTGGAGAAGTGAATTATATAAATATAAGG
TGGTAAAAATTGAACCACTA---GGAGTAGCGCCCACCAGAGCAAGGAGAAGAGTGGTG--------------
-------------------------CAAAGAGAAAAAAGAGCA---GTGGGA---ATAGGAGCT---------
GTGCTCCTTGGGTTCTTGGGAGCA---GCAGGAAGCACTATG---GGCGCAGCGTCA---
GTGACGCTGACGGTACAA---GCCAGACAG---CTATTG---TCTGGTATAGTGCAACAGCAGAGC---
AATCTG---CTGAGG---GCTATTGATGCACAACAGCATATGTTGCAGCTCACAGTCTGGGGCATC
>JQ085297
TTCTACTGCGATTCAACAAAACTGTTTAATAGTACTTGGAATGGTACTGAAGATGATTCTGGA----------
----

AAAAATAGAACAATCACACTCCCATGCAGAATAAAGCAATTTATAAACATGTGGCAGGAAGTAGGAAAAGCAA
TGTATGCCCCTCCTATCAAGGGACAAATTAGCTGTTTATCAAAT---
ATTACAGGGCTGCTATTAACAAGAGATGGTGGTAACAATGTGAGC----------------------------
----

AACACCACCGAGGTCTTCAGACCTGGAGGAGGAAATATGAGGGACAATTGGAGAAGTGAATTATATAAATATA
AAGTAGTAGAAATTGAACCATTA---GGATTAGCACCCACCAAGGCAAAGAGGAGAGTGGTG-----------
----------------------------CAGAGAGAAAAAAGAGCA---GTGGGA---ATAGGAGCT------
---TTGTTCCTTGGATTCTTGGGAGCA---GCAGGAAGCACTATG---GGCGCAGCGTCA---
ATGACGCTGACGGTACAG---GCCAGACAA---TTATTG---TCTGGTATAGTGCAACAGCAGAAC---
AATCTG---CTGAGA---GCTATTGAGGCGCAACAGCACCTGTTGCAACTCACAGTCTGGGGCATC
>JQ085295
TTCTACTGTAATACAACACCACTATTCAATAGTATTTGGAAGGATGGACAGTTAAATAACACATACACT----
----

AAAGAGAAAAATATCACACTCCAATGTAAAATAAAACAAATTGTAAACAGGTGGCAGGAAGTAGGAAAAGCAA
TGTATGCCCCTCCCATCAGAGGACAAATTACATGTATATCAAAT---
ATTACAGGGCTACTATTAACAAGAGATGGTGGTATTGGCAATAATGAAACTGGC-------------------
----

AATAACAATACTGAGATCTTCAGACCTGGAGGAGGAGATATAAGGGACAATTGGAGAAGTGAATTATATAAAT
ATAAAGTAGTAAAAATTGAACCATTA---GGAGTAGCACCCACCAAGGCAAAAAGAAGAGTGGTG--------

-------------------------------CAGAGAGACAAAAGAGCA---ATAGGAACACTAGGGGCT---
------ATGTTCCTTGGGTTCTTGGGAGCA---GCAGGAAGCACTATG---GGCGCAGCGTCA---
TTGACGCTGACGGTACAA---GCCAGATTA---CTATTA---TCTGGTATAGTGCAACAGCAGAAC---
AATCTG---CTGAGA---GCTATTGAGGCGCAACAGCATATGTTGCAACTCACAGTCTGGGGCATT
>FJ798397
TTCTATTGTAATACAACAAAACTGTTTAACAGTACTTGGAATGGTACTGAAGGACTAAACAACTCTACAGGAA
AT---

AGCACAGGAAGTATCACACTCCCATGCAAAATAAAACAAATTATAAACATGTGGCAAGAAGTAGGAAAAGCAA
TGTATGCCCCTCCCATCAGAGGACAAATTAGCTGTTCATCAATT---
ATTACAGGGCTGATATTAACAAGAGATGGTGGTATTGACAATAAAAATGCCACT-------------------
----

AACACAAACAACACAGAGACCTTTAGACCAGGAGGAGGAAATATGAAGGATAATTGGAGAAGTGAATTATATA
AATATAAAGTAGTAAAAATTGAACCATTA---GGAATAGCACCCACCAAGGCAAAGAGGAGAGTGGTG-----
----------------------------------CAGAGAGAAAAAAGAGCA---GTGGGA---
CTAGGAGCT---------GTGTTCCTTGGGTTCTTGGCAACA---GCAGGGAGCACTATG---
GGCGCAGCGTCA---GTGACGCTGACGGTACAG---GCCAGACAA---TTATTG---
TCTGGTATAGTGCAACAGCAAAAC---AATCTG---CTGAAG---
GCTATTGAGGCGCAACAACGTATGTTGCAACTCACAGTCTGGGGCATC
>FJ469685
TTCTACTGTAATACAACACAACTGTTTAATAGTACTTGGAACATTACAGGAAATAACACCATCGCACCA----
----

GGAAATGACACAATCACACTCCCATGCAGAATAAAACAAATTATAAACATGTGGCAGGAAGTAGGAAAAGCAA
TGTATGCCCCTCCCATCAGAGGACCAATTAGATGTTCATCAAAT---
ATTACAGGGCTGCTATTAACAAGAGATGGTGGTAGTGACAAAAAT----------------------------
----

GATACTGAGGTCTTCAGACCTGCAGGAGGAAATATGAAGGACAATTGGAGAAGTGAATTATATAAATATAAAG
TAGTAAGAATTGAACCATTA---GGAATAGCACCCACCAAGGCAAAGAGAAGAGTGGTG--------------
-------------------------CAAAGAGAAAAAAGAGCA---GTGGGA---ATAGGAGCT---------
ATGTTCCTTGGGTTCTTGGGAGCA---GCAGGAAGCACTATG---GGCGCAGCGTCA---
GTGGCGCTGACGGTACAG---GCCAGACTA---TTATTG---TCTGGTATAGTGCAACAGCAGAAC---
AATCTG---CTGAGG---GCTATTGAGGCGCAACAGCATATGTTGCAACTCACAGTCTGGGGCATC
>FJ798416
TTCTACTGTAATACAACAGGACTGTTTAAAAGTACTTGGTATATGAATACAACAAGGAATGAT----------
----

AGTGCTGAAAATATAACACTCCCATGCAGGATAAAACAAATTATAAACATGTGGCAGGAAGTAGGAAAAGCAA
TGTATGCCCCTCCCATCAGTGGACCAATTAGTTGTTCATCAAAT---
ATTACAGGGTTGATATTAACAAGGGATGGTGGTGATAGGAACGAT----------------------------
----

ACTGAGACCTTCAGACCTGGAGGAGGAGATATGAGGGACAATTGGAGAAGTGAGTTATATAAATATAAAGTAG
TAACAATTGAACCATTA---GGAATAGCACCCACCAAGGCGAGGAGAAGAGTGGTG-----------------
----------------------CAGAGAGAAAAAAGAGCA---ATAGGC---CTGGGAGCT---------
TTGTTCATTGGGTTCTTGGGAGCA---GCAGGAAGCACTATG---GGCGCAGCGTCA---
ATAACGCTGACGGTACAA---GCCAGACAA---TTATTG---TCTGGTATAGTGCAACAGCAGAAT---
AATCTG---CTGAGG---GCTATTGAGGCGCAACAACATCTGTTGCAACTCACAGTCTGGGGCATC
>JQ085289
TTCTACTGTAATACAACATCACTGTTTAATAGTACCTGGAATGGGAATGAGACCATAGAGTCAAATAAC----
----

AGCACTGAAAATATTATACTCCAATGCAGAATAAAACAAACTATAAACATGTGGCAGGAAGTAGGAAAAGCAA
TGTATGCCCCTCCCATCAGTGGACAAATTAGATGCTCATCAAAT---
ATTACAGGGCTAATATTAACAAGAGATGGTGGTAATAATGCTAGC----------------------------
----

CAGACAGAGATCTTCAGACCTGGAGGAGGAGATATGAGGGACAATTGGAGAAGTGAATTATATAAATATAAAG

TAGTAGAGATTGAACCATTA---GGAGTAGCACCCACCAAGGCAAAGAGAAGAGTGGTG--------------
--------------------------CAGAGAGAAAAAAGAGCA---GTGGGAACACTAGGAGTT---------
ATGTTCCTTGGGTTCTTGGGAGCA---GCAGGAAGCACTATG---GGCGCAGCGTCG---
ATGGCGCTGACGGTACAG---GCCAGACAA---TTATTG---TCTGGTATAGTGCAACAGCAGAAC---
AATCTG---CTGAAG---GCTATTGAGGCGCAACAGCATCTGTTGCAACTCACGGTCTGGGGCATC
>JQ085293
TTCTACTGTGATACATCACTACTGTTTAATAGTACTTGGAATGAAACTACTTGGAACAAAACTACTGGGAATA
GTACTAAC--
--------------------GAT---
GCAAATAACACAGTAATTACACTCCCATGCAGAATAAAACAAATTATAAACATGTGGCAGGGAGTAGGAAGAG
CAATGTATGCCCCTCCCATCAGCGGAAATATTAGCTGTTCATCAAAT---
ATTACAGGGCTGATATTGACAAGAGATGGTGGTAATGAAAGTAAGAAT-------------------------
----

AACAGCGCTGAGATTTTCAGACCTGCAGGAGGAGATATGAGGGACAATTGGAGAAGTGAATTATATAAATATA
AAGTAGTAAAAATTGAACCATTA---GGAATAGCCCCCACCAGGGCAAAAAGAAGAGTGGTG-----------
-----------------------------CAGAGAGAAAAAAGAGCA---GTGGGA---TTCGGAGCT------
---CTGTTCCTTGGGTTCTTGGGAGCA---GCAGGAAGCACTATG---GGCGCAGCGTCA---
CTGACGTTGACGGTACAG---GCCAGACAA---TTATTG---TCTGGTATAGTGCAACAGCAGAGC---
AATATG---CTGAGA---GCTATTGAGGCGCAACAGCATATGTTGCAACTCACAGTCTGGGGCATC
>AF217150
TTCTACTGTAATTCAACACCATTGTTTAATAGTATTTGGAATATTACTGGGAATATTACTGAAGAGTCAAATA
ACACT---

AAAGGAAATGACACAATCATACTCACATGCAGAATAAAACAAATTATAAACATGTGGCAGGAAGTAGGAAAAG
CAATGTATGCCCCTCCCATCAGAGGACAAATTAAATGTGTATCAAAT---
ATTACAGGGCTACTATTAACAAGAGATGGTGGTAAGGATGAAGGTAAA-------------------------
----

GAGAACGATACCGAGACCTTCAGACCACAAGGAGGAAATATGAGGGATAATTGGAGAAGTGAATTGTATAAAT
ATAAAGTAGTAAAAATTGAACCATTA---GGAGTAGCACCCACCAAGGCAAAGAGAAGAGTGGTG--------
--------------------------------CAGAGAGAAAAAAGAGCA---GTGGGA---ATAGGAGCT---
------ATGTTCCTTGGGTTCTTAGGAGCA---GCAGGAAGCACTATG---GGCGCAGCGTCA---
ATAACGCTGACGGTACAG---GCCAGACAA---TTATTG---TCTGGTATAGTGCAACAGCAGAGC---
AATTTG---CTGAGG---GCTATTGAGGCGCAACAGCATCTGTTGCAACTCACAGTCTGGGGCATC
>MH262735
TTCTACTGTAATACAACACAGCTGTTTAACAGTACTTGGAGGGTGGTCAATGGTACTTGGAATGATACTATAG
AA--

TCAAATAAAACTATCACACTCCCATGCAGAATAAAACAAATTATAAATAGGTGGCAGGAAGTAGGAAAAGCAA
TGTATGCCCCTCCCATTGAAGGACAAATTAACTGTTCATCACAG---
ATTACAGGGCTACTATTAACAAGAGATGGTGGTAATGACACAAACGGG-------------------------
----

ACGACAGAGACCTTCAGACCTGGAGGAGGAAATATGAAAGACAATTGGAGAAATGAATTATATAAATATAAAG
TAGTAAAAATTGAGCCATTA---GGAATAGCACCCACCAAGGCAAAGAGAAGAGTGGTG--------------
--------------------------CAGAGAGAAAAAAGAGCA---ATA------ATAGGAGCT---------
GTGTTCCTTGGGTTCTTGGGGGCA---GCAGGAATCACTATG---GGCGCAGCGTCG---
ATGACGCTGACGGTACAG---GCCAGACAA---TTATTG---TCTGGTATAGTGCAACAGCAAAAC---
AATTTG---CTGAGG---GCTATTGAGGCGCAACAGCATCTGTTGCAACTCACAGTCTGGGGCATT
>MH263144
TTCTACTGTAATACAACACCACTGTTTAATAGCTCATGGAATGGTACTGAAGGATTCAAAAAGTTCAATGAGA
CT--

GAAGACAAAAACATTACACTCCAATGTAGAATAAAACAAATTATAAACATGTGGCAGGGAGTAGGAAAAGCAA
TGTATGCCCCTCCCATCAGCGGAATAATTAAATGTTCATCAAAT---
ATTACAGGGCTGATACTAACAAGAGATGGTGGTAGAGATGATGGTACTAATACC-------------------
----

ACAACAAACGATACCGAGATCTTCAGACCTATAGGAGGAGATATGAAGGATAATTGGAGAAGTGAATTATATA

AATATAAAGTAGTAAGAATTGAACCAGTA---GGAATAGCACCCACCAGGGCAAAGAGAAGAGTGGTG-----
----------------------------------CAGAGAGAAAAAAGAGCA---GCA------
CTAGGAGCA---------GTGTTCCTTGGGTTCTTGGGAGCA---GCAGGAAGCACTATG---
GGCGCAGCGTCA---ATAACGCTGACGGTACAG---GCCAGGCTA---TTATTG---
TCTGGTATAGTGCAGCAGCAGAAC---AATTTG---CTGAGG---
GCTATTGACGCGCAACAACACCTGTTGCAACTCACAGTCTGGGGCATC
>MH263362
TTTTACTGTAATTCAACACCACTGTTTAATAGTACTTGGAAGAGGAATGATACTAATAGTACTTGGAATGTAA
CACAATGGAAT--
-------------------GAT---
AGTACTAATGGTACAATCACACTCCAATGCAGAATAAAACAAATTATAAACATGTGGCAAGAAGTAGGAAAAG
CAATGTATGCCCCTCCTATTAGAGGAAACATTAGCTGCTCATCAAAT---
ATTACAGGGCTCATATTAACAAGAGATGGTGGTGAGAAAAATGAG----------------------------
----

ACCGATGAGATCTTCAGACCTGCAGGAGGAGATATGAGGGACAATTGGAGAAGTGAACTATATAAGTATAAAG
TAGTACAACTTGAGCCATTA---GGAGTAGCCCCCACCAAGGCAAAGAGAAGAGTGGTG--------------
-------------------------CAGAGAGAAAAAAGAGCA---GTGGGAATGATAGGAGCT---------
ATGTTCCTTGGGTTCTTGGGAACA---GCAGGAAGCACTATG---GGCGCAGCGTCA---
ATAGCGCTGACGGCACAG---GCCAGACAA---TTATTG---TCTGGTATAGTGCAACAGCAGAAT---
AATCTG---CTGAGG---GCTATTGAGGCGCAACAGCATCTGTTGCAACTCACAGTCTGGGGCATC
>MH263630
TTCTACTGTAATACAACGAAACTGTTT------ACTTGGAATAGTACTCGGGATGGGAATAGCACTAAAGAG-
----

GCAAATGACACACTCATTCTCCCATGTAGAATAAAACAAATTATAAACATGTGGCAGGAAGTAGGAAAAGCAA
TGTATGCCCCTCCCATCAGAGGACCCATTAATTGTGTATCAAAC---
ATTACAGGGTTGATATTAACAAGAGATGGTGGTACTGGAAAAAATACC-------------------------
----

ACAGACAACAACACTGAGGTCTTCAGACCTACAGGAGGAAATATGAAGGACAATTGGAGAAGTGAATTATATA
AATATAAAGTAGTAAGAATTGAACCATTA---GGAGTAGCACCCACCAAGGCAAAGAGAAGAGTGGTG-----
----------------------------------CAGAGAGAAAAAAGAGCA---GTGGGA---
ATAGGAGCT---------GTGTTCCTTGGGTTCTTGGGAGCA---GCAGGAAGCACTATG---
GGCGCAGCGTCA---GTGACGCTGACGGTACAG---GCCAGACAA---TTATTG---
TCTGGTATAGTGCAACAGCAGAAC---AACTTG---CTGAGG---
GCCATTGAGGCGCAACAACATCTGTTGCAACTCACGGTCTGGGGCATC
>KX984368
TTCTACTGTAATACAACACAACTGTTTAATAGTACTTGGGAGAATGAGACTACAGGGATAAATACCACTGAA-
----

GGAAATGGCACATTCACACTCCAATGCAGAATAAAACAAATTATAAACATGTGGCAGGAAGTAGGAAAAGCAA
TGTATGCCCCTCCCATTAGAGGACAGATTAGATGTTCATCAAAT---
ATTACAGGGCTGCTATTAACAAGAGATGGTGGTAATAGAGATGGT----------------------------
----
GGTAATGACACAAAC---
AGGAATGAGACCTTTAGACCTGGAGGAGGAGATATGAGGGACAATTGGAGAAGTGAATTATATAAATATAAAG
TAGTAAAAATTGAACCATTA---GGAGTAGCACCCACCAAGGCAAAGAGAAGAGTGGTG--------------
-------------------------CAGAGAGAAAAAAGAGCA---GTGGGA---ATAGGAGCT---------
CTGTTCCTTGGGTTCTTGGGAGCA---GCAGGAAGCACTATG---GGCGCAGCGTCA---
CTGACGCTGACGGTACAG---GCCAGACTA---TTATTG---TCTGGTATAGTGCAACAGCAGAAC---
AATTTG---CTGAGA---GCTATTGAGGCGCAACAGCATCTGTTGCAACTCACAGTCTGGGGCATT
>KX984537
TTCTACTGTAATACAACACAACTGTTTAATAGTACCTGGGATAATAGTACCTGGGATACTAACTGGACTAGTC
CTGAA--
-------------------AAT---
TCTACCAATACCAATATCACACTCCCATGCAGAATAAAACAAATTATAAACATGTGGCAGGAAGTAGGAAAAG
CAATGTATGCCCCTCCCATCAGAGGACAAATTAAGTGTTCATCAAAT---
ATTACAGGACTGCTGTTAACAAGAGATGGTGGTAATAGCACAGATGGA-------------------------
----

AACAACTCTGAGATCTTCAGACCTGGAGGAGGAGATATGAGGGACAATTGGAGAAGTGAATTATACAAATATA
AAGTAGTGCAAATTGAACCATTA---GGAGTAGCACCTACCAAGGCAAAGAGAAGAGTGGTG-----------
-----------------------------CAGAGAGAAAAAAGAGCA---GTGGGA---ATAGGAGCT------
---TTATTCCTTGGGTTCTTGGGAGCA---GCAGGAAGCACTATG---GGCGCAGCATCA---
ATAACGCTGACGGTACAG---GCCAGACAA---TTATTA---TCTGGTATAGTGCAACAGCAGAAC---
AATCTG---CTGAGG---GCTATTGAGGCGCAACAGCATCTGTTGCAACTCACAGTCTGGGGCATC
>KX984588
TTCTACTGTAACACAACACAACTGTTTAATAGTACTTGGTATGCTAATGGTTCTGGGAATAATACTAAGTGG-
----

ACAAATGACACCATTACACTCCCATGTAGAATAAAACAAATTATAAACATGTGGCAGGAAGTAGGAAAAGCAA
TGTATGCCCCTCCCATTGCAGGAGTGATTAACTGTGTATCAAGT---
ATTACAGGGCTACTATTAACAAGAGATGGTGGTAGTGGAAATAACACC-------------------------
----

AACACCACTGAGGTCTTCAGACCTGGAGGAGGAGATATGAGGGACAAGTGGAGAAGTGAATTATATAAATATA
AAGTAGTACAAATTGAACCATTA---GGAATAGCTCCCACCAAGGCAAAGAGAAGAGTGGTG-----------
-----------------------------CAGAGAGAAAAAAGAGCA---GTAACA---TTAGGAGCT------
---ATGTTCCTTGGGTTCTTGGGAGCA---GCAGGAAGCACTATG---GGCGCAGCGTCA---
GTGACGCTGACGGTACAG---GCCAGACAA---TTATTG---TCTGGTATAGTGCAACAGCAGAAC---
AATCTG---CTGAGA---GCTATTGAGGCGCAACAACATATGTTGCAACTCACAGTCTGGGGCATC
>AY842786
TTCTACTGTAATTCAACACAACTGTTTAATAGTACTTGGGTTAATGGTACCAGGAATGATACTAAAGGGTCAA
AT---

AGCACTGAAATCATCACACTCCCATGCAGAATAAAACAAATTATAAACAGGTGGCAGGGAGTAGGAAAAGCAA
TGTATGCCCCTCCCATCCAAGGACTAATTAAATGTTCATCAAAT---
ATTACAGGGTTGTTATTAACAAGAGATGGGGGTAACGAGGATAAC----------------------------
----

AAGACCGAGACCTTCAGACCTGCAGGAGGAGAGATGAGGGACAATTGGAGAAGTGAATTATATAAATATAAAG
TAGTAAAAATTGAACCATTA---GGAGTAGCACCCACCAAGGCAAAGAGAAGGGTGGTG--------------
--------------------------CAGAGAGAAAAAAGAGCA---GTGGGA---ATAGGAGCT---------
GTGTTCCTTGGGTTCTTGGGAGCA---GCAGGAAGCACTATG---GGCGCAGCATCA---
ATGACGCTGACGGTACAG---GCCAGACGA---TTATTG---TCTGGTATAGTGCAACAGCAGAAC---
AATTTG---CTGAGG---GCTATTGAGGCGCAACAGCATCTGTTGCAACTCACAGTCTGGGGCATC
>AY842808
TTCTACTGTAACACAACACAACTGTTTAATAGTACTTGGAACGATACTACTATTAATAGAACTGAAGGGTCA-
----

AATACAAGAAATATCACACTCCCATGCAGAATAAAACAAATTATAAACAAGTGGCAGGAAGTAGGGAAAGCAA
TGTATGCCCCTCCCATCAGAGGACAAATTAGATGTTCATCAAAT---
ATTACAGGGCTGTTATTAACAAGAGATGGTGGTGCTGATACTAAC----------------------------
----

ACCACCGAAATCTTCAGACCTGGAGGAGGAGATATGAGGGACAATTGGAGAAGTGAATTATATAAATATAAAG
TAGTAAGAATTGAACCAATA---GGAGTAGCACCCACTAAGGCAAAGAGAAGAGTGGTG--------------
--------------------------CAGAGAGAAAAAAGAGCA---GTGGGA---ATAGGAGCT---------
TTGCTCCTTGGGTTCTTGGGAGCA---GCAGGAAGCACTATG---GGCGCAGCGTCA---
TTGACGCTGACGGTACAG---GCCAGACAA---TTATTG---TCTGGTATAGTGCAACAGCAGAAC---
AACCTG---CTGAGG---GCTATTGAGGCGCAACAGCACCTGTTGCAACTCACAGTCTGGGGCATC
>KX984877
TTCTACTGTAATACAACAAAATTGTTTGATAATAATATTGCTAACAGGTCA----------------------
----

GCTAATAGCACAATCATACTCTCATGCAGAATAAAACAAATTATAAACATGTGGCAGGAAGTAGGGAAAGCAA
TGTATGCCCCTCCCATCGAAGGACAGATTAGCTGTTCATCAAAT---
ATTACAGGGCTGTTATTAACAAGAGATGGTGGTAGTAGTAATGAGAAT-------------------------
----

```
----------
AATGGGAGTGAGATTTTCAGACCTGGAGGAGGAGATATGAGGGACAATTGGAGAAGTGAATTATATAAGTACA
AAGTAATAAAAATTGAACCATTA---GGAGTAGCACCCACCAAGGCAAGGAGAAGAGTGGTG----------
---------------------------CAGAGAGAAAAAAGAGCA---GTGGGA---ATGGCAGCA------
---GTGTTCCTTGGGTTCTTGGGAGCA---GCAGGAAGCGCTATG---GGCGCAGCGTCA---
ATGACGCTGACGGTACAG---GCCAGACAA---TTATTG---TCTGGTATAGTGCAACAGCAAAAC---
AATTTG---CTGAGA---GCTATTGAGGCGCAGCAGCATCTGCTGCAACTCACAGTCTGGGGCATC
>AY842824
TTCTACTGTAATACAGACCAGCTGTTTAATAGTACTTGGCAGTTTAATAGTACTTGGCCGCCTAATAGTACTG
AGTATAATACT-----------------------------------------------------------
----------ACAGGGTCA------
GATAACAATGGAACGATCACACTCCAGTGTAGAATAAAACAAATTATAAATAGATGGCAGGAAGTAGGAAAAG
CAATGTATGCCCCTCCCATCGAAGGACCAATTAACTGTTCATCAAAT---
ATTACAGGGCTGCTATTAACAAGAGATGGTGGTGAGAACAGGAACAAT------------------------
----------------------------------------------------------------------
----------
AGCAGTACAGAGATCTTCAGACCTGGAGGAGGAAATATGAGGGACAATTGGAGAAGTGAATTATATAAATATA
AAGTAGTAAAAATTGAACCACTA---GGAGTAGCACCCACCAAGGCAAAGAGAAGAGTGGTG----------
---------------------------CATAAAGAAAAAAGAGCA---GTGGGA---CTTGGAGCT------
---ATGTTCCTTGGGTTCTTGGGAGCA---GCAGGAAGCACTATG---GGCGCAGCGTCA---
ATAACGCTGACGGTACAG---GCCAGACAA---TTATTG---TCTGGTATAGTGCAACAGCAGAAC---
AATCTG---CTGAGA---GCTATTGAGGCGCAACAACATATGTTGCAACTCACAGTCTGGGGCATC
>MH632949
TTCTACTGTAATACAACACAACTGTTTAATAGTAGTAATATTTGGATAAATGGGACGACA------------
----------------------------------------------------------------------
---------------------------
AATAACACTAATATTACACTCCCATGCAGAATAAAACAAATCATAAATAGGTGGCAGGAAGTAGGAAGAGCAA
TGTATGCCCCTCCCATCGAGGGACGAATTAATTGTGTGTCCAAT---
ATAACAGGACTGATATTAACAAGAGATGGTGGTAACAAGACCAACACC------------------------
----------------------------------------------------------------------
-------
CCTGAGACCTTCGAGACCTTCAGACCTATAGGAGGAGATATGAGGGACAATTGGAGAAGTGAATTATATAAAT
ATAAAGTAGTAAAAATTGAGCCGTTA---GGAGTAGCACCCACCAAGGCAAAAAGAAGAGTGGTG--------
------------------------------CAAAGAGACAAAAGAGCA---GTGGGA---ATAGGAGCT---
------GTGTTCCTCGGGTTCTTGGGAGCA---GCAGGAAGCACTATG---GGCGCAGCGTCA---
ATAACGCTGACGGTACAA---GCCAGGCAA---TTACTG---TCTGGGATAGTGCAACAGCAGAAC---
AATCTG---CTGAGA---GCTATTGAGGCGCAACAGCATATATTGCAACTCACAGTCTGGGGCATC
>MH632956
TTCTACTGTAACACAACACAGTTGTTTAATAGTACTTGGCCTCCTGAGAATGGGACAACAAAT----------
----------------------------------------------------------------------
---------------------------
GATACAGGAAATATAATACTCCCATGTAGAATAAAACAAATTATAAACAGATGGCAGGAAGTAGGAAAAGCAA
TGTATGCCCCTCCCATCAGAGGACAAATTAGCTGTGAATCAAAT---
ATTACAGGGTTGCTATTGACAAGAGATGGTGGTACTCCTGTGAAC---------------------------
----------------------------------------------------------------------
-------------
CAGACCGAGACTTTCAGACCTGGAGGAGGAGATATGAGAGACAATTGGAGAAGTGAATTATACAAATATAAAG
TAGTAAAAATTGAACCATTA---GGAATAGCACCCACCAGGGCAAAGAGAAGAGTGGTG-------------
------------------------CAGAGAGAAAAAAGAGCA---GTGGGAACTATGGGAGCT---------
CTGTTCCTTGGGTTCTTGGGAACA---GCAGGAAGCACTATG---GGCGCAGCATCA---
TTAACGCTGACGGTACAG---GCCAGACTA---CTATTG---TCTGGTATAGTGCAACAGCAAAGC---
AATTTG---CTGAAG---GCTATTGAGGCGCAACAACATCTGTTGCAACTCACAGTCTGGGGCATC
>MH632822
TTCTACTGTAATACATCACAACTGTTTAATACTACTTGGAGGACTAATAATACTGATAACAGGTCAAAT----
----------------------------------------------------------------------
---------------------------
AGAACTAATACAATCACCCTCCCATGTAGAATAAAACAAATTGTAAACATGTGGCAGGAAGTAGGAAGAGCAA
TGTACGCCCCTCCCATCAGTGGACCACTTAGTTGCTTATCAAAT---
ATTACAGGGCTGCTATTAACAAGAGATGGTGGCAACGCCAAAAATGACAGCGAC------------------
----------------------------------------------------------------------
```

AACAACACAGAGACCTTCAGACCTGGAGGAGGAGATATGAGGGACAATTGGAGAAGTGAATTATATAAATATA
AAGTAGTAAAAATTGAACCATTA---GGAATAGCACCCACCAAGGCAAAGAGAAGAGTGGTG-----------
----------------------------CAGAGAGAAAAAAGAGCA---GTGGGAGTAATGGGAGCT------
---ATGTTCCTTGGGTTCTTGGGAGCA---GCAGGAAGCACTATG---GGCGCAGCGTCA---
GTGACGCTGACGGTACAG---GCCAGACAA---TTATTG---TCTGGTATAGTGCAACAGCAGAAC---
AATCTG---CTGAAG---GCTATTGAGGCGCAGCAGCATATGTTGCAACTCACAGTCTGGGGCATT
>MH575991
TTCTACTGTAATACAACAAAACTATTTAATAGTACTTGGCTACTTAATAGTACTTGGAATAATACTGAAAGGA
CAAT---

GACACTGAAATAATCACACTCCCATGCAGAATAAAACAAATTGTAAACAGGTGGCAGGAAGTAGGCAAAGCAA
TGTATGCCCCTCCCATCCAAGGACAAATTAGTTGCTCATCAGAT---
ATTACAGGGCTGCTACTAGTTAGAGATGGTGGAATTAATACCAGCAACAACAGC-------------------
----

AGCAACGAGACCGAGACCTTCAGACCTGGAGGAGGAGATATGAGGGACAATTGGAGAAGTGAATTATATAAAT
ATAAGGTGGTAAAAATTGAACCATTA---GGAGTAGCACCCACCAAGGCAAAGAGAAGAGTGGTG--------
-------------------------------CAGAGAGAAAAAAGAGCA---GTGGGAATGCTGGGAGCT---
------ATGTTCCTTGGGCTCTTGGGAGCA---GCAGGAAGCACTATG---GGCGCAGCGTCA---
ATGACGTTGACGGTACAG---ACCAGACAA---TTATTG---TCTGGTATAGTGCAACAGCAGAAC---
AATCTG---CTGAGG---GCTATTGAGGCGCAGCAGCATCTGTTGCAACTCACAGTCTGGGGCATC
>MH576349
TTCTACTGTAACACAACACGACTGTTTAATAGTACTTGGAGTGTTAATGGAACTAGCATAAACGAAACT----
----

AACAATAAAAATATCACACTCCCGTGCAGAATAAAACAAATTATAAACAGGTGGCAGGAAGTAGGAAAAGCAA
TGTATGCCCCTCCTATCAGTGGGATAATTAGGTGCTCATCAAAT---
ATTACAGGACTAATATTAACAAGAGATGGTGGTACAACTGATGGTGGTACAACT-------------------
----

AATAGTACAAAGGAGACCTTCAGACCTGGAGGGGGAAATATGAAGGACAATTGGAGAAGTGAATTATATAAAT
ACAAAGTAGTAAAAATTGAGCCATTA---GGAGTAGCACCCACCAAGGCACAGAGAAGAGTGGTG--------
-------------------------------CAAAGAGAAAAAAGAGCA---ATAGGAACGTTAGGAGCT---
------ATGTTCCTCGGGTTCTTGGGAGCA---GCAGGAAGCACTATG---GGCGCAGCGTCA---
CTGACGCTGACGGTACAA---GCCAGACAA---TTATTG---TCTGGTATAGTGCAACAGCAGAAC---
AATCTG---CTGAGG---GCTATTGAGGCGCAACAGCATATGTTGCAACTCACAGTCTGGGGCATC
>MH576262
TTCTACTGTAATACAACACAGCTGTTTAATAGTACTTGGAATAATAGTACTTGGAATGGTACTGATAATTGGG
AT---
-------------GGTACT------
GAATCAAATAGCACTATCACACTCCCATGCAGAATAAAACAAATTATAAACTTGTGGCAGGAAGTAGGAAGAG
CAATGTATGCCCCTCCCATCCAAGGGCAAATTAGATGTTCATCAAAT---
ATTACAGGGCTGCTGCTAGTAAGAGATGGTGGGAGCAACAATAGTAGT-------------------------
----

AATGATACAGAGACCTTCAGGCCTGGAGGAGGAGATATGAGGGACAATTGGAGAAGTGAATTATATAAATATA
AAGTAGTCAAAATTGAACCATTA---GGAATAGCACCCACCCATGCAAAGAGAAGAGTGGTG-----------
----------------------------CAAAGGGAAAAAAGAGCA---ATAGGA---CTTGGAGCT------
---TTCTTCCTTGGGTTCTTGGGAGCA---GCAGGAAGCACTATG---GGCGCAGCGTCA---
GTGACGCTGACGGTACAG---GCCAGACAA---TTATTG---TCTGGTATAGTGCAGCAGCAAAAC---
AATTTG---CTGAGA---GCTATTGAGGCGCAACAGCATCTGTTGCAACTCACAGTCTGGGGCATC
>MH576152
TTCTACTGTAATACAACACAACTGTTTAATAGTACTTGGAATGGTACTACAGGAGTAAATGGTACAGGAGCAA
ATAACACA---

GAAGTAAATATTATCACACTCCCATGCAAGATAAGGCAAATTGTAAACATGTGGCAGGAAGTAGGAAAAGCAA
TGTATGCCCCTCCCATCAGTGGACAAATTAGTTGTTCATCAAAT---
ATTACAGGGCTACTACTACTAAGAGATGGTGGTAATAGCAATGACACGGAT----------------------
----

AACACGACTGAAATCTTCAGACCTGGAGGAGGAGATATGAGGGACAATTGGAGAAGTGAATTATATAAATATA
AAGTAGTACAAATTGAACCATTA---GGAATAGCACCCACCAAGGCAAGGAGAAGAGTGGTG----------
-----------------------------CAGAGAGAAAAAAGAGCA---GTGGGA---ATAGGAGCT------
---CTGTTCCTTGGGTTCTTGGGAGCA---GCAGGAAGCACTATG---GGCGCAGCATCA---
ATAACGCTGACGGTACAA---GCCAGACAC---TTATTG---TCTGGTATAGTGCAACAGCAGAAC---
AATTTG---CTGAGA---GCTATTGAGGCGCAACAGCATATGTTGCAGCTCACAGTCTGGGGCATC
>MH576039
TTCTATTGTAATACAACACAGCTGTTTAATAGTACTTGGAATAGTACTTGGACTGGGAATGCTACCGAA----
----

TTAGATGGAAACATCACACTCCCATGCAGAATAAAACAGATTATAAACAGGTGGCAAGAAGTAGGAAAAGCAA
TGTATGCCCCTCCCATTAGAGGAAAAATTAGATGTTCATCAAAT---
ATTACAGGGTTACTATTAGTAAGAGATGGTGGTAACACGAGCGGG-----------------------------
----

ACTGAGACCTTCAGACCTGGAGGAGGAGATATGAAGGACAATTGGAGGAGTGAATTATATAAATATAAAGTAG
TAAAAATTGAGCCATTA---GGAGTAGCACCCACCAGGGCAAAGAGAAGAGTGGTG-----------------
----------------------CAAAGAGAAAAAAGAGCA---GTGGGA---ATAGGAGCT---------
GTGTTCCTTGGGTTCTTGGGAGCA---GCAGGAAGCACTATG---GGCGCAGCGTCA---
GTGACGCTGACGGTACAA---GCCAGGCTG---TTATTG---TCTGGTATAGTGCAACAGCAAAAC---
AATTTG---CTGAGA---GCTATTGAGGCGCAACAACATCTATTGCAACTCACAGTCTGGGGCATC
>MH575857
TTCTACTGTAATACAACAAAACTGTTTAATAGTACTTGGAACAAAAATAATACTAATTGGACTATTACTGAAG
GGTCAGAT--
-------------AACACT------
GCAGAGAATGACACACTCATGCTCCCATGCAGAATAAAACAAATTATAAACATGTGGCAGGAAGTAGGAAAAG
CAATGTATGCTCCTCCCATTAGAGGAGTAATCAAATGTTCATCAAAT---
ATTACAGGGTTGCTATTAACAAGGGATGGTGGTAGAAACCAGAGTAATAAC----------------------
----

CAGAATGAGACAGAGATCTTCAGACCTGGAGGAGGAGATATGAGGGACAATTGGAGAAGTGAATTATATAAAT
ATAAAGTAGTAAGAATTGAACCATTA---GGAGTAGCACCCACCAAGGCAAAGAGAAGAGTGGTG--------
-------------------------------CAAAGAGAAAAAAGAGCA---GTGGGA---ATAGGAGCT---
------GTGTTCCTTGGGTTCTTAGGAGCA---GCAGGAAGCACTATG---GGCGCAGCGTCG---
GTGACGCTGACGGTACAG---GCCAGACAA---TTATTG---TCTGGTATAGTGCAGCAGCAGAAC---
AACTTG---CTGAGA---GCTATTGAGGCGCAGCACCATCTGTTGCAACTCACGGTCTGGGGCATC
>MH575796
TTCTACTGTGATTCAACGCAACTGTTTAATAGTACCTGGCTGCCTAATAATGAGAGTATTTGGTATAATAATA
GGAATAATACT---
----ACAGGGTCAAAC---------
AGCAGTGCAGTCAATATCACACTCCCATGTAGAATAAAACAAATTATAAACCTATGGCAAGAAGTAGGAAAAG
CAATGTATGCCCCTCCCATCAGAGGAGCAATCAACTGCACGTCAAAT---
ATTACAGGGTTGATATTAACAAGAGATGGTGGGAATAATCCTAAC-----------------------------
----

GGGACTAACACCTCTGAGACTTTCAGACCTGGAGGAGGAAATATGAGGGACAATTGGAGAAGTGAATTATATA
GATATAAAGTAGTAAAAATTGAACCATTA---GGAGTAGCACCCACCACGGCAAAGAGAAGAGTGGTG-----
-----------------------------------CAGAGAGAAAAAAGAGCA---GTGGGT---
CTCGGAGCC---------TTGTTCCTTGGGTTCTTGGGAGCA---GCAGGAAGCACTATG---
GGCGCAGCGTCA---GTAACGCTGACGGTACAG---GCCAGACAA---CTATTG---
TCTGGTATAGTGCAGCAGCAGAAC---AATCTG---CTGAGG---
GCTATTGAGGCGCAACAGCATCTGTTGCAACTCACAGTCTGGGGCATC
>MH575728
TTCTACTGTAATTCAACAAAACTGTTTAATAGTACTTGGAGTAGTAATGGGAGTAGTACTTGGAATAGTACTG
AG--

ACGAATGAAATTATCACACTCCCATGCAGAATAAAACAAATTATAAACATGTGGCAGGAAGTAGGAAAAGCAA
TGTATGCCCCTCCTATCAGAGGACAAATTAGATGTTCATCAAAC---
ATTACAGGGCTGCTGTTAACAAGAGATGGTGGTAACAATAGCAACGACACTAGC-------------------

----

AACGACACTGAGATCTTTAGACCTGGAGGAGGAAATATGAAGGATAATTGGAGAAGTGAATTATATAAATACA
AAGTAGTAAAAATTGAACCAATA---GGAGTAGCACCCACCAAGGCAAAGAGAAGGGTGGTG-----------
----------------------------CAGAGAGAGAAAAGAGCA---GTAGGA---CTAGGAGCT------
---GTGTTCCTTGGGTTCTTGGGAGCA---GCCGGAAGCACTATG---GGCGCAGCGTCA---
ATGACGCTGACGGTACAG---GCCAGACAA---TTGTTG---TCTGGTATAGTGCAACAGCAGAAC---
AACCTG---CTGAAG---GCTATTGAGGCGCAGCAGCATCTGTTGCAACTCACAGTCTGGGGCATC
>MH575694
TTCTACTGTAACACATCAAATCTGTTTAATAGTACTTGGAATAGTACTACTTGGAATAGTTGGAATAGTAGTA
CTCGGGGTAAT--

ACTACAGGAAATATCACACTCCAATGCAGAATAAAACAAATTGTAAACATGTGGCAGGAAGTAGGAAAAGCAA
TGTATGCCCCTCCTATCAAAGGACCAATTAAATGTTCATCAAAT---
ATCACAGGGCTGCTACTAACAAGAGATGGGGGTGAGGAGAGTGAGAAT-------------------------
----

AACAACGATTCCGAGATCTTCAGACCTGGAGGGGGAGATATGAGGGATAATTGGAGAAGTGAATTATATAAAT
ATAAAGTAGTAAAAATTGAACCATTA---GGTCTAGCACCCACCAAGGCAAAGAGAAGAGTGGTG--------
-------------------------------CAGAGAGAAAAAAGAGCA---GTGGGAACATTAGGAGCT---
------TTGTTTCTTGGGTTCTTGGGAACA---GCGGGAAGCACTATG---GGCGCAGCGTCA---
ATGACGCTGACGGTACAG---GCCAGGCTA---TTATTG---TCTGGTATAGTGCAACAGCAGAAC---
AATTTG---CTGAGA---GCTATTGAGGCGCAACAGCATATGTTGCAACTCACAGTATGGGGCATC
>MH575680
TTCTACTGTAATACAACAAAACTGTTT------
ACTTGGAATGCTACAACAAAACTGTTTACGTGGAATGCTACAAAT----------------------------
----
AGCAATGGAACCATCATACTCCCATGTAGAATAAAACAAATTATAAACATGTGGCAAGAGGTAGGAAAAGCAA
TGTATGCCCCTCCCATTCGTGGACAAATTAGATGTTCATCAAAT---
ATTACAGGACTGCTATTAACAAGAGATGGTGGGACTAACAAGACA----------------------------
----

GGGAACAGGACTGAGATCTTCAGACCTGGAGGGGGAAATATGAAGGACAATTGGAGAAGTGAATTATATAAAT
ACAAAGTAGTAGAAATTAAACCATTA---GGAGTAGCACCCACCAAGGCAAAGAGAAGAGTGGTG--------
-------------------------------CAGAGAGAAAAAAGAGCA---GTGACC---ATAGGAGCT---
------ATGTTCCTTGGGTTCCTGGGAGCA---GCAGGAAGCACTATG---GGCGCAGCATCA---
CTGACGCTGACGGTACAG---GCCAGACAA---TTATTG---TCTGGTATAGTGCAACAGCAGAAC---
AATCTG---CTGAGG---GCGATTGAGGCGCAGCAGCATCTGTTGCAACTCACAGTCTGGGGCATA
>MH575478
TTCTATTGTAATACAACACAATTGTTTAATAGCACTTGGGATAATGGTACTGAAAAGTCAAGTAACCTTGGAG
GA---

AATGATACAACCATCATTCTCCCATGCAGAATAAAACAAATTATAAACATGTGGCAGGAAGTAGGAAAAGCAA
TGTATGCCCCTCCCATCAAAGGAATAATTAGATGTTCATCAAAT---
ATTACAGGGCTGCTATTGACAAGAGATGGAGGTAATAGTACTGACACA-------------------------
----

AATGGGAGCGAGATCTTCAGACCTGGAGGAGGAGATATGAGGGACAATTGGAGAAGTGAATTATATAAGTATA
AAGTAGTAAAAATTGAACCATTA---GGAGTAGCACCTACCAAGGCAAAGAGAAGAGTGGTG-----------
----------------------------CAGAGAGAAAAAAGAGCA---GTGGGA---CTAGGAGCT------
---TTGTTCCTTGGGTTCTTGGGAGCA---GCAGGAAGCACTATG---GGCGCAGCGTCT---
CTGACGCTGACGGTACAG---GCCAGACAA---TTGTTG---TCTGGTATAGTGCAACAGCAGAAC---
AATTTG---CTGAGG---GCAATTGAGGCGCAACAGCATCTGTTGCAACTCACAGTCTGGGGCATC
>MH575379
TTTTACTGTAATTCAACACAACTGTTTAATAGTACTTGGAATAGTGATGGTACTCTGAATAGTACTTGGAAT-
----

AGTCCAGGTAACATCACACTCCCATGTAAAATAAGGCAAATTGTAAACATGTGGCAGAAAGTAGGAAAAGCAA
TGTATGCTCCTCCCATCCGTGGACAAATTAAATGTTCATCAAAT---
ATTACAGGGCTGCTATTAACAAGAGATGGTGGTAACGAGAGTGGG----------------------------

----

AACAGCGAAACCTTCAGACCTGGCGGAGGAGATATGAGGGACAATTGGAGAAGTGAATTATACAAATATAAAG
TAGTAAGAATTGAACCATTA---GGACTAGCACCCACTAAGGCAAAGAGAAGAGTGGTG--------------
-------------------------CAGAGAGAAAAAAGAGCA---ATAGGAACACTGGGAGCT---------
GTGTTCCTTGGGTTCTTGGGAACA---GCAGGAAGCACTATG---GGCGCAGCGTCA---
ATGACGCTGACGGTACAG---GCCAGGCAA---TTATTG---TCTGGTATAGTGCAGCAGCAGAAC---
AATCTG---CTGAAG---GCTATTGAGGCGCAACAGCATCTGTTGCAACTCACAGTCTGGGGCATT
>MH633031
TTCTACTGTAATACAACAGTACTGTTCAATAGTACTTGGAATTTTAATGATAGTGATACTGGTATTGGG----
----

AATAATACTAATATCACACTCCCATGCAGAATAAGACAAGTTATAAACATGTGGCAGGAAGTAGGAAAGGCAA
TGTATGCCCCTCCCATTAGAGGACAGATTAGATGTTCATCAAAT---
ATTACAGGGCTGATATTAACAAGAGATGGTGGTGATACTAATGTTACCAAT----------------------
----

AACAAGACTGAAACCTTCAGACCTGCAGGAGGAAATATGAAGGATAATTGGAGAAGTGAACTATATAAATATA
AAGTAGTACAAATTGAACCATTA---GGAGTAGCACCCACCAAGGCAAAGAGAAGAGTGGTG-----------
----------------------------CAAAGAGAAAAAAGAGCT---GTGGGA---TTTGGAGCT------
---ATGTTCCTTGGGTTCTTGGGAGCA---GCAGGAAGCACTATG---GGCGCAGCGTCA---
GTGGCGCTGACGGTACAG---GCCAGACAA---TTATTG---TCTGGTATAGTGCAACAGCAAAAC---
AATTTG---CTGAGG---GCTATTGAGGCGCAACAGCATCTGTTGCAACTCACAGTCTGGGGCATT
>MH633155
TTCTATTGCAATACAACAGAATTATTTGACAGTACTTGGATTAATGGTACTGACATTACTACTAATGCTACTG
GAGGGAACAAT--

AGCACAAAAAATATCACCCTCACATGCAGAATAAAACAAATTATAAATATGTGGCAGAAAGTAGGAAAAGCAA
TGTATGCCCCTCCCATCAGAGGACAAATTAGCTGTTCATCAAAT---
ATTACAGGGCTGCTATTAACTAGAGATGGGGGTGGTAATAGCACA----------------------------
----

AACAACAATGAAACCTTCAGACCTACTGGAGGAGATATGAGGGACAATTGGAGGAGTGAATTATATAAATATA
AAGTAGTAAGAATTGAACCATTA---GGGGTAGCACCCAACAAGGCAAAGAGAAGAGTGGTG-----------
----------------------------CAGAGAGAAAAAAGAGCA---GTGGGAGTAATAGGAGCT------
---ATGTTCCTTGGGTTCTTGGGAGCA---GCAGGAAGCACTATG---GGCGCAGCGTCA---
ATAACGCTGACGGTACAG---GCCAGACAA---TTATTG---TCTGGTATAGTGCAACAGCAGAAT---
AACTTG---CTGAGG---GCTATTGAGGCGCAACAGCATCTGTTGCAACTCACAGTCTGGGGCATT
>DQ127534
TTCTACTGTAATACAACAAACCTGTTTAATAGTACCTGGAATGCAACAAACCTGGTTAATGGTACCGGGAATA
GTACT--

GAAGGGAATGACACAATCAAACTCCCATGCAGAATAAGACAATTTATAAACATGTGGCAGACAGTGGGAAAAG
CAATGTATGCCCCTCCCATCGAAGGACAAATTAAATGTACATCAAAT---
ATTACAGGGCTGCTATTAACAAGAGATGGTGGTAATTCGACGGAA----------------------------
----

CAGTCCGAGACCTTCAGACCGGGAGGAGGAGATATGAGGGACAATTGGAGAAGTGAATTATATAAATATAAAG
TAGTAGGAATTGAACCATTA---GGAATAGCACCCACCACGGCAAAGAGAAGAGTGGTG--------------
-------------------------CAGAGAGAAAAAAGAGCA---GTGGGAGCACTAGGAGCT---------
ATGTTCCTTGGGTTCTTGGGAGCA---GCAGGAAGCACTATG---GGCGCAGCGTCA---
CTGACGCTGACGGTACAG---GCCAGACTA---TTATTG---TCTGGTATAGTGCAACAGCAGAAC---
AATTTG---CTGAGG---GCTATTGAGGCGCAGCAGCATATGTTGCAACTCACGGTCTGGGGCATC
>DQ127537
TTCTACTGTGACTCAACACAACTGTTTAATAGTACTTGGAATGAGACTGATTGGAATAATAAAACTGAT----
----

GGAGATGAAACTATCACACTCCCATGCAGAATAAAACAAGTTATAAACAGGTGGCAGGAAGTAGGAAAAGCAA
TGTATGCCCCTCCTATCAGAGGAAACATTAGCTGTTCATCAAAT---
ATTACAGGGCTGATACTAACAAGAGATGGTGGTAATAGTAATAACACT-------------------------

----

AACAACATCGAGATCTTTAGACCTGGGGGAGGAAATATGAAGGACAATTGGAGAAGTGAATTATATAAATATA
AAGTAGTACAAATTGAACCATTA---GGAGTAGCACCCACCAGGGCAAAGAGAAGAGTGGTG-----------
----------------------------CAGAGAGAAAAAAGAGCA---GTGGGAATGATAGGAGCT------
---ATGTTCCTTGGGTTCTTGGGAGCA---GCAGGAAGCACTATG---GGCGCAGCGTCA---
ATAACGCTGACGGTACAG---GCCAGACAA---TTATTG---TCTGGTATAGTGCAGCAGCAGAAC---
AATCTG---CTGAGG---GCTATTGAGGCGCAACAGCATCTGTTGCAACTCACAGTCTGGGGCATT
>DQ127548
TTCTACTGTAATTCAACACAATTGTTTAATAGTATTTGGAAGTATAATAATGGCACTTGGAAGTATAATGATA
GTATTTGGAAT--
-AGTACTGAAAGTTCA---------
AATAACACTGAAAACATCATGCTCCCGTGCAGAATAAGACAAATTATAAACATGTGGCAGGAAGTAGGAAAAG
CAATGTATGCCCCTCCCATCAGAGGACCAATTAGATGTACATCAAAT---
ATTACAGGGCTAATATTAACAAGAGATGGTGGCACAAACAACAAC----------------------------
----

ACGAAGGAGGTCTTCAGACCTGGAGGAGGAGATATGAGGGACAATTGGAGAAGTGAACTATATAAATATAAAG
TAGTACAAATTGAACCATTA---GGAATAGCACCCACCAGGGCAAAAAGAAGAGTGGTG--------------
-------------------------CAGAGAGAAAAAAGAGCA---GTGGGA---ATAGGAGCT---------
TTGTTCCTTGGGTTCTTGGGAGCA---GCAGGAAGCACTATG---GGCGCAGTGTCA---
ATGACGCTGACGGTACAG---GCCAGACAA---TTGTTG---TCTGGTATAGTGCAACAGCAGAAC---
AATCTG---CTGCGG---GCTATTGAGGCGCAACAGCATATGTTGCAACTCACAGTCTGGGGCATC
>AF219627
TTTTACTGTAATACAACACAACTGTTTAATAGTACTTGGATTCGGAATGGTACTGATTGGACTCGAAATGATA
CTGAAGGA---
-------------TCAGAC------
ATCACTAACGAAAATATCACGCTCCCATGTAGAATAAAACAAATTATAAACATGTGGCAGAAAGTAGGGAAAG
CAATGTATGCCCCTCCCATCAGTGGACAAATTAGCTGTTCCTCAAAT---
ATTACAGGGCTGCTATTAACACACGATGGTGTTGTTGGTCTGTACACG-------------------------
----

GACGCGAACAACGTGACCTTCAGACCGGGAGGAGGAAATATGAGGGACAATTGGAGAAGTGAATTATATAAAT
ATAAAGTAATAAAAGTTGAACCAATA---GGAATAGCACCCACCAAGGCAAAGAGAAGAGTGGTG--------
-------------------------------CAGAGAGAAAAAAGAGCA---GTGGGA---ATAGGAGCT---
------ATGTTCCTTGGGTTCTTGGGAACA---GCAGGAAGCGCTATG---GGCGCAGCGTCA---
GTGACGCTGACGGTACAA---GCCAGACAA---TTATTG---TCTGGTATAGTGCAACAGCAGAAC---
AATCTG---CTGAGG---GCTATTGAGGCGCAACAGCATATGTTGCAACTCACAGTCTGGGGCATC
>GU728244
TTCTATTGTAATACAACATCACTGTTTAATAGTACTAATAGTACTTGGAATATTGAAAGGTCAAATAACTCTA
AA---

GTTAATGACACACTCACACTCCCATGCAGAATAAAACAAATTATAAACATGTGGCAGGAAGTAGGAAAAGCAA
TGTATGCTCCTCCCATCAAAGGGAACATTAGCTGTTCATCAAAT---
ATTACAGGGCTACTATTAGTAAGAGATGGTGGTGAAAACCAGAAC----------------------------
----

AGCAGCACCGAGACTTTCAGACCTGGAGGAGGAGATATGAGAGACAATTGGAGAAGTGAATTATATAAATATA
AAGTAGTAAAACTTGAACCGTTA---GGAATAGCACCCACCAAGGCAAAGAGAAGAGTGGTG-----------
----------------------------CAGAGAGAAAAAAGAGCA---TTGGGA---TTCGGAGCT------
---ATGTTCCTTGGGTTCTTGGGAGCA---GCAGGAAGCACTATG---GGCGCAGCGTCA---
ATAGCGCTGACGGTACAG---GCCAGACAA---TTATTG---TCTGGTATAGTGCAACAGCAGAAC---
AATCTG---CTGAGA---GCTATTGAAGCGCAACAGCATCTGTTGCAGCTCACAGTCTGGGGCATC
>GU728216
TTCTACTGTAATACATCAGAGCTGTTTAATAGTGCTTGGCATGCTAATGATACTGGGAATGGCACAAAT----
----

GGGACAGAATATATCACACTCCAATGTAGAATAAAACAAATTATAAACATGTGGCAGAAAGTAGGAAAAGCAA
TGTATGCCCCTCCCATCAGAGGACCAATTAAATGTTCATCAAAT---
ATTACAGGGCTGCTATTAGTAAGAGATGGTGGCAATAATAACGAG----------------------------

----

AGCAACAATGAGACCTTCAGACCTGGAGGAGGAGATATGAGGGACAATTGGAGAAGTGAATTATATAAATATA
AAGTAGTAAAAAATTGAACCATTA---GGACTGGCACCCACCAAGGCAAAGAGAAGAGTGGTG----------
----------------------------CAGAGAGAAAAAAGAGCA---GTGGGA---CTAGGAGCA------
---TTTTTCCTTGGGTTCTTGGGAGCA---GCAGGAAGCACTATG---GGCGCAGCGTCA---
ATGGCGCTGACGGTACAG---GCCAGACAA---TTATTG---TCTGGTATAGTGCAGCAGCAGAAC---
AACCTG---CTGAGG---GCTATTGAGGCGCAACAGCATCTGTTGCAACTCACAGTCTGGGGCATC
>GU728376
TTCTACTGTAACTCAACACCACTGTTTAATAGTACTTGGAATATTCGGAATAGTACTGAAGAG----------
----

TTAAATGATACTATCATACTCAAATGCAGAATAAAACAAATTATAAACATGTGGCAGGAAGTAGGAAAGGCAA
TGTACGCCCCTCCCATCAGAGGACAAATTAGATGTTCATCAAAT---
ATTACAGGGCTGCTATTAACAAGAGATGGTGGTGACACTAAAAAGAATGGG----------------------
----

ACCGATGATACAGAGACCTTCAGACCTGGAGGAGGAAATATGAGGGACAATTGGAGAAGTGAATTATATAAAT
ATAAAGTAGTAAAAATTAAACCATTA---GGAATAGCACCCACCAAGGCAAAGAGAAGAGTGGTG--------
-------------------------------CAGAGAGAAAAAAGAGCA---GTGGGA---TTAGGAGCT---
------ATGTTCCTTGGGTTCTTGGGAGCA---GCAGGAAGCACTATG---GGCGCAGCGTCA---
GTGACGCTGACGGTACAG---GCCAGACAA---TTATTG---TCTGGTATAGTGCAACAGCAAAAC---
AATTTG---CTGAGG---GCTATTGAGGCGCAACAGCATATGTTGCAACTCACAGTCTGGGGCATC
>FJ469690
TTCTACTGTAATTTAACACAACTGTTTAATAGTACTTGGAATGAAACAGGGTCAAATAACACTGAG-------
----

GGAAATGGCACCTTCATACTCCCATGCAGAATAAAACAAATTATAAATATGTGGCAGGAAGTAGGAAAAGCAA
TGTATGCCCCTCCCATCGAAGGGCTAATTAACTGTTTATCAAAT---
ATTACAGGGCTGATATTAACAAGAGATGGTGGTAGTAGTAATAAC----------------------------
----

ACCACCGAGATCTTCAGACCTGGAGGAGGGGATATGAGGGACAATTGGAGAAGTGAATTATATAAATATAAAG
TAGTAAAAGTTGAACCATTA---GGAGTAGCACCCACCAGGGCAAAGAGGAGAACGGTG--------------
-------------------------CAAAGAGAAAAAAGAGCA---GTGGGA---ATAGGAGCT---------
TTGTTCCTTGGGTTCTTGGGAGCA---GCAGGAAGCACTATG---GGCGCAGCGTCA---
ATGACGCTGACGGTACAG---GCCAGACAA---TTATTG---TCTGGTATAGTGCAACAGCAGAAC---
AATCTG---CTGAGG---GCTATTGAGGCGCAACAGCACCTGTTGCAACTCACAGTCTGGGGCATC
>FJ469692
TTCTACTGTACTACAACACCACTGTTTAATAGTACTTGGGATAATAATAGTACTGGGAATATTAACAGT----
----

ACTAACAAAAATATCACACTCCAATGCAGAATAAAACAAATTATAAACATGTGGCAGGAAGTAGGAAAAGCAA
TGTATGCCCCTCCTATCGAGGGAACAATTAGGTGTTCATCAAAT---
ATTACAGGGTTGATATTAACAAGAGATGGCGGTATTAACAATACT----------------------------
----

GACAACAAGGAGGTCTTCAGACCAGGAGGAGGAGACATGAGGGACAATTGGAGAAGTGAATTATACAAATATA
AAGTAGTAAAAAATTGAACCATTA---GGATTAGCACCCACCAAGGCAAAGAGAAGAGTGGTG----------
----------------------------CAAAGAGAAAAAAGAGCA---GTGACA---CTGGGAGCT------
---ATGTTCCTTGGGTTCTTAGGAGCA---GCAGGAAGCACTATG---GGCGCAGCATCA---
ATGACGCTGACGGTACAG---GCCAGACTA---TTATTG---TCTGGTATAGTGCAACAGCAGAAC---
AATCTG---CTGAGG---GCTATTGAGGCGCAACAGCATCTGTTGCAACTCACAGTCTGGGGCATC
>FJ469694
TTCTACTGTAATTCAACACAACTGTTTAATAGTACTTGGAAGTATAATGATACTAATGACACTGAAAGACACG
AA---

AAAGGCAATGACAATATTACACTCCCATGCAGAATAAGACAAATTGTAAACCTGTGGCAAGAAGTAGGGAAAG
CAATGTATGCCCCTCCCATCCAAGGACTAATTAAATGTTCATCAAAT---
ATTACAGGGCTGCTATTAACAAGGGATGGTGGTAATAATGACACC----------------------------

----

TCCCAGACCGAGACCTTCAGACCTGCAGGAGGAGATATGAGGGACAATTGGAGAAGTGAATTATATAAATATA
AAGTAGTACGAATTGAACCATTA---GGAATAGCACCCACCAAGGCAAAGAGAAGAGTGGTGCAG---AGA--
----------------------GAGGTGCAGAGAGAAAAAAGAGCA---ATAGGAATGGGAGCAGTT------
---TTGTTCCTTGGGTTCTTGGGAGCA---GCAGGAAGCACTATG---GGCGCAGCGTCA---
ATGGCGCTGACGGTACAG---GCCAGACAA---TTATTG---TCTGGTATAGTGCAACAGCAAAAC---
AATTTG---CTGAGG---GCTATTGAGGCGCAACAGCATATGTTGCAACTCACAGTCTGGGGCATC
>FJ469699
TTCTACTGTAATACAACACAACTGTTTAAAGGTACTTGGAATGGTACTACTTTGATAAATACAACAGGA----
----

GGAAATGAAACAATCACACTCCCATGCAGAATAAAACAATTTATAAACATGTGGCAGAGAGTAGGAAAAGCAA
TATATGCCCCTCCTATCAGAGGACATATTAACTGTACATCAAAT---
ATTACAGGGCTGCTATTAACAAGAGATGGTGGTAATAATAACGGGACC-------------------------
----

AACACCACCGAAATCTTTAGACCTGGAGGGGGAGATATGAGGGACAATTGGAGAAGTGAATTATATAAATATA
AAGTAGTAAAAATTGAACCATTA---GGAATAGCACCCACCAAGGCAAAGAGAAGAGTGGTG-----------
----------------------------CAGAGAGAAAAAAGAGCA---GTGGGA---ATAGGAGCT------
---CTGTTCCTTGGGTTCTTGGGAGCA---GCAGGAAGCACTATG---GGCGCAGCGTCA---
TTAACGCTGACGGTACAG---GCCAGACAA---TTATTG---TCTGGTATAGTGCAACAGCAGAAC---
AATCTG---TTGAAG---GCTATTGAGGCGCAACAGCATCTGTTGCAACTCACAGTCTGGGGCATC
>FJ469702
TTCTACTGTGACACAACACAACTGTTTAATAGTACCTGGAATGATACTACCTTGAATGATACTAGTAATTGGA
GTAAT--

GATACTACTGAAGTTATCACACTCCCATGCAGAATAAAACAGATTATAAACTTGTGGCAGGAAGTAGGAAAAG
CAATGTATGCCCCTCCCATCAGTGGACAAATTAATTGTTCATCTAAT---
ATTACAGGGCTGCTATTAACAAGAGATGGTGGTAATAATAATAATAGC-------------------------
----

AACAACAGCGAGACCTTCAGACCTGCAGGAGGAAATATGAAGGACAATTGGAGAAGTGAATTATATAAATACA
AAGTAGTAAAAATTGAACCATTA---GGAATAGCACCCACCAAGGCAAGGAGAAGAGTGGTG-----------
----------------------------CAAAGAGAAAAAAGAGCA---GTGGGA---CTAGGAGCT------
---ATGTTCCTTGGGTTCTTGGGAGCA---GCAGGAAGCACTATG---GGCGCAGCGTCA---
ATAACGCTGACGGTACAG---GCCAGACAA---TTATTG---TCTGGTATAGTGCAACAGCAAAAC---
AATTTG---CTGAGA---GCTATTGAGGCGCAACACCATTTGTTGCAACTCACAGTCTGGGGCATC
>FJ469705
TTCTACTGTAATACAACAAAACTGTTTAATAGTACTTGGAATAGTACTTGGAATAATAGTACTTGGAATGGTA
CTGGA--

ACTGAAAATGACACAATCACACTCCCATGCAGAATAAAACAATTTATAAACCTGTGGCAGCAAGTAGGAAAAG
CAATGTATGCCCCTCCTATCCCAGGAAATATTAGCTGTTCATCAAAT---
ATTACAGGGCTGCTATTAACAAGAGATGGTGGTAATAGTAACAAC----------------------------
----

AGCAACAACGAGACCTTCAGACCTGGAGGAGGAGATATGAGGGACAATTGGAGAAGTGAATTATACAAATATA
AAGTAGTAAAAGTTGAACCATTA---GGAGTAGCACCCACTAAGGCAAAGAGAAGAGTGGTG-----------
----------------------------CAGAGAGAAAAAAGAGCA---GCAGGA---ATAGGAGCT------
---CTGCTCCTTGGGTTCTTGTCAGCA---GCAGGAAGCACTATG---GGCGCAGCGTCA---
GTGACGCTGACGGTACAG---GCCAGACAA---TTATTG---TCTGGTATAGTGCAACAGCAAAAC---
AATTTG---CTGAGA---GCTATTGAGGCGCAACAGCATCTGTTGCAACTCACGGTCTGGGGCATT
>FJ469711
TTCTACTGTAATTCAACACAACTGTTTAATAGTACTTGGCATAAGAATGGTACTAATAGTACTGGGGGG----
----

GCAAATGACACTATCACACTCCCATGTAAAATAAAACAAATTATAAACATGTGGCAGGAAGTAGGAAAAGCAA
TGTATGCCCCTCCCATCGAAGGACAAATTAACTGTTCATCAAAA---
ATTACAGGGCTGCTATTAACAAGAGATGGTGGTAATAACAGCACTGCC-------------------------

----

AATGACACCGAGACCTTCAGACCTGGAGGAGGAGATATGAGGGACAATTGGAGAAGTGAATTGTATAAATATA
AAGTAGTAAAAATTGAACCACTA---GGATTAGCACCCACCAGGGCAAGGAGAAGAGTGGTG-----------
----------------------------CAGAGAGAAAAAAGAGCA---GTGGGA---CTAGGAGCT------
---GTGTTCCTTGGGTTCTTGGGAGCA---GCAGGAAGCACTATG---GGCGCAGCGTCA---
ATAACGCTGACGGTACAG---GCCAGGCGA---TTAATG---TCTGGTATAGTGCAACAGCAGAAC---
AATTTG---CTGAGA---GCTATTGAGGCGCAACAGCATCTGTTGCAACTCACAGTCTGGGGCATT
>FJ469712
TTCTATTGTAATTCAACACAACTGTTTAATAGTACCTGGTATGCTAATGATACTAACAGTATTAGGAATAGTA
GCAGG---

GAAGGAAACGATACAATTATACTCCAATGTAGAATAAAACAAATTATAAACATGTGGCATGAAGTAGGAAAAG
CAATGTATGCCCCTCCCATCAAAGGAAACATTAGCTGTTCATCAAAC---
ATTACAGGGATACTATTAACAAGAGATGGTGGTAATAAGAATAACGAG-------------------------
----

ACTAACAACACTGAGATCTTCAGACCTGGAGGAGGAGATATGAGGGACAATTGGAGAAGTGAACTATATAAAT
ATAAAGTAGTAAAAATTGAACCATTA---GGAATAGCACCCACCACGGCAAGGAGAAGAGTGGTG--------
-------------------------------CAGAGAGAAAAAAGAGCA---GTGGGA---CTAGGAGCA---
------CTGTTCCTTGGGTTCTTGGGAGCA---GCAGGAAGCACTATG---GGCGCAGCGTCA---
TTGACGCTGACGGTACAG---GCCAGACTC---TTATTG---TCTGGTATAGTGCAGCAGCAGAAT---
AATCTG---CTGAGA---GCTATTGATGCGCAACAACATCTGTTGCAACTCACAGTCTGGGGCATT
>FJ469719
TTCTACTGTAATACAACACAACTGTTTAATACTACTTGGTATAACAATGAAACTGTGGATAATAAAACTTGGA
CTGAAGATGAT---
-------ACTAGAAACTTA------
ACTGACAAAGACATTATCATACTCCCATGCAGAATAAAACAAATTATAAACATGTGGCAGAAAGTAGGAAAAG
CAATGTATGCCCCTCCTATCCAAGGAATGATTAGCTGTTCATCAAAT---
ATTACAGGGCTACTCTTAACAAGAGATGGTGGTAATGATACAGATACAGAT---------------------
----

CCGAATGGGACAGAGACCTTCAGACCTATAGGAGGAGATATGAGGGACAATTGGAGAAGTGAATTATATAAAT
ATAAAGTAGTAAGAATTGAACCATTA---GGGATAGCACCCTCCAAGGCAAAGAGAAGAGTGGTG--------
-------------------------------CAGAGAGAAAAAAGAGCA---GCA------CTAGGAGCT---
------GTGTTCCTTGGGTTCTTGGGAGCA---GCAGGAAGCACTATG---GGCGCAGCGTCA---
ATAACGCTGACGGTACAG---GCCAGACAA---TTATTG---TCTGGTATAGTGCAACAGCAGAGC---
AGATTG---CTGAGA---GCTATTGAGGCGCAACAACATATGTTGCAACTCACAGTCTGGGGCATC
>FJ469726
TTCTACTGTAATTCAACACCACTATTTAATAGTATTTGGCATGCTAATGGTACCTGGAATGGTACTGAAAAGT
CAAT--

AACACTGAAAGAAATATCACACTCCAATGCAGAATAAAACAAATTATAAATATGTGGCAGGGAGTAGGAAAAG
CAATGTATGCCCCTCCCATCAGAGGACAAATTAGATGTTCATCAAAC---
ATTACAGGGCTGTTATTAACAAGAGATGGGGGTCCAGAGAACGAG----------------------------
----

ACTAAGCCTGAGATCTTCAGACCTGGAGGAGGAGATATGAGGAATAATTGGAGAAGTGAATTATACAAATATA
AAGTAGTAAAAATTGAACCATTA---GGAGTAGCACCCACCAAGGCAAAGAAAAGAGTGGTG-----------
----------------------------CAGAGAGAAAAAAGAGCA---ATAGGGGGATTAGGAGCT------
---TTGTTCCTTGGGTTCTTAAGAGCA---GCAGGAAGCACTATG---GGCGCAGCGTCA---
ATAACGCTGACGGTACAA---GCCAGACAA---TTATTG---ACTGGTATAGTGCAACAGCAGAAC---
AATTTG---CTGAGA---GCTATTGAGGCGCAACAACACCTGTTGCAACTCACAGTCTGGGGCATC
>GU728225
TTCTACTGTAATTCATCACAACTGTTTAATAGCACTTGGAGCAGGAATGGGACGTGGAGTAGTACTTGGAACA
CC--

ACGGGAGAAAATATCACACTCCCATGCAAAATAAAACAAATTATAAACATGTGGCAAGAAGTAGGAAAAGCGA
TGTATGCCCCTCCCATTGAAGGACTAATTAGATGTGTATCAAAT---
ATCACAGGGCTGCTATTAACAAGAGATGGTGGGAACACCACCGAC----------------------------

----

AACGACACCGAGACCTTCAGACCTTTAGGAGGAAATATGAAGGACAATTGGAGAAGTGAATTATATAAATATA
AAGTAGTAAGAATTGAACCATTA---GGGTTAGCACCCACCAAGGCAAAGAGAAGAGTGGTG-----------
----------------------------CAGAGGGAAAAAAGAGCA---GTGGGA---ATGGGAGCT------
---CTGTTCCTTGGGTTCTTGGGAGCA---GCAGGGAGCACTATG---GGCGCAGCGGCA---
ATGACGCTGACGGTACAA---GCCAGACAA---TTATTG---TCTGGTATAGTGCAGCAGCAGAAC---
AATCTG---CTGAGA---GCTATTGAGGCGCAACAGAGTATGTTGCAACTCACAGTCTGGGGCATT
>MN515912
TTCTACTGTAATTCAACACAACTGTTTAATAGTACTTGGAGTGTTAATGAAGGGTCAAATAACACTGAAGGA-
----

AACAATGGCACAATCACACTCCCATGTAGAATAAAACAAATTATAAACATGTGGCAGGAAGTAGGAAAAGCAA
TGTATGCCCCTCCCATCAGAGGACAGATTAAATGTTCATCAAAT---
ATTACAGGGCTGTTGTTAACAAGAGATGGTGGCAATAACAACAGC----------------------------
----

AATACAGAGATCTTCAGACCTACAGGAGGAAATATGAAGGACAATTGGAGGAGTGAATTATATAAATATAAAG
TAGTAAAAATTGAACCATTA---GGAGTAGCACCCACCCCGGCAAAGAGAAGAGTGGTG--------------
-------------------------CAGAGAGAAAAAAGAGCA---GTGGGA---TTAGGAGCT---------
TTGTTCCTTGGGTTCTTGGGAGCA---GCAGGAAGCACTATG---GGCGCAGCGTCA---
GTGACGCTGACGGTACAG---GCCAGACAA---TTATTG---TCTGGTATAGTGCAACAGCAGAAC---
AATCTG---CTGAGG---GCTATTGAGGCGCAACAGCATCTGTTGCAACTCACAGTCTGGGGCATA
>MN515943
TTCTACTGTAATTCAACACAACTGTTTAATAGTACTTGGGCTGTTAATACTACTTGGAATAATACTGAAAACA
ATGAA---

GGAAATGACACAATCACCCTTCCATGCAGAATAAAACAAATTATAAACATGTGGCAGGAAGTAGGAAAAGCAA
TGTATGCCCCTCCCATTAGAGGACAAATAAGATGTTCATCAAAT---
ATTACAGGGCTGCTATTAACAAGAGATGGTGGTAATAGTAATGACACT-------------------------
----

ACCAGGCCCGAGACCTTCAGACCTGGAGGAGGAGATATGAGGGACAATTGGAGAAGTGAATTATATAAATATA
AAGTAGTAAAAATTGAACCATTA---GGAATAGCACCCACCAAGGCAAAGAGAAGAGTGGTG-----------
----------------------------CAGAGAGAAAAAAGAGCA---GTGGGA---ATAGGAGCT------
---ATGTTCCTTGGGTTCTTGGGAGCA---GCAGGAAGCACTATG---GGCGCAGCGTCA---
ATAACGCTGACGGTACAG---GCCAGACTA---TTATTA---TCTGGTATAGTGCAACAGCAGAAC---
AATTTG---CTGAGG---GCTATTGAGGCGCAACAGCATCTGTTGCAACTCACAGTCTGGGGCATC
>MN515956
TTCTACTGTAATACAACAAAACTGTTTAGTAGCACTTGGAATGCTACTGAAAAGATCACTTGGAATAATACTG
AAAGG---

TCAAATGACACTATCATACTCCCATGCAGAATAAAACAAATTATAAACATGTGGCAGGAAGTAGGAAAAGCAA
TGTATGCCCCTCCCATCCAGGGACTAATTAGATGTTCATCAAAT---
ATTACAGGGCTGCTATTAACAAGAGATGGTGGTAATGAAAGTGACACA-------------------------
----

AATAGGTCTGAGACCTTCAGACCTGGAGGAGGAGATATGAAGGACAATTGGAGAAGTGAATTATATAAATATA
AAGTAATAAAACTTGAACCATTA---GGACTAGCACCCACTAAGGCAAAGAGAAGAGTGGTG-----------
----------------------------CAGAGAGAAAAAAGAGCA---CTGGGA---TTCGGAGCT------
---TTGTTCCTTGGGTTCTTGGGAGCA---GCAGGAAGCACTATG---GGCGCAGCGTCA---
ATAACGCTGACGGTACAG---GCCAGACAG---TTACTG---TCTGGTATAGTGCAACAGCAGAAC---
AATCTG---CTGATG---GCTATTGAGGCGCAACAGCATATGTTGCAACTCACAGTCTGGGGCATT
>MN515787
TTCTATTGTAATACAACAAAGCTGTTTAATAGCACCTTTCCCTTTAATAGTACTTCAAATAATACTAATGGGG
CAAAT---

AACACTGAAATAATCACACTCCCATGTAAAATAAAACAAATTATAAACAGGTGGCAGGAAGTAGGAAAAGCAA
TGTATGCCCCTCCCATCAGAGGAGAAATTAAATGTTTATCAAAA---
ATTACAGGGCTGCTATTAACAAGAGATGGTGGTAATAACACAAAT----------------------------

----

AACACATATGAGACCTTCAGACCTGGAGGAGGAAATATGAAGGACAATTGGAGAAGTGAATTATATAAATATA
AAGTAGTACAAATTGAGCCATTA---GGAATAGCACCCACCACGGCAAAGAGAAGAGTGGTG-----------
----------------------------CAGAGAGAAGAAAGAGCA---GTGGGG---TTTGGAGCT------
---ATGTTCCTTGGGTTCTTGGGAGCA---GCAGGAAGCACTATG---GGCGCAGCGTCA---
GTGACGCTGACGGTACAG---GCCAGACAA---TTATTG---TCTGGTATAGTGCAACAGCAGAAC---
AATTTG---CTGAGA---GCTATTGAGGCGCAACAGCATCTGTTGCAGCTCACAGTCTGGGGCATC
>EU850430
TTCTACTGTAATTCTACACAACTGTTTAATAGTACTTGGAATAGTACTGAAGGATCAAATAGCACTGAA----
----

AGAAATACCACACTCACACTCCCATGCAGAATAAAACAAATTGTAAACATGTGGCAGGAAGTAGGAAAAGCAA
TGTATGCCCCTCCCATCAGAGGACAAATTAGATGTTCATCAAAT---
ATTACAGGGCTGATATTAACAAGAGATGGT---AATAGTAACGAG----------------------------
----

ACCGAGATCTTCAGACCTGGAGGAGGAGATATGAGGGACAATTGGAGAAGTGAATTATATAAATATAAAGTAG
TAAAAATTGAACCATTA---GGAGTAGCACCCACCAAGGCAAAGAGAAGAGTGGTG-----------------
----------------------CAGAGAGAAAAAAGAGCA---GTGGGA---ATAGGAGCT---------
TTGTTCCTTGGGTTCTTGGGAGCA---GCAGGAAGCACTATG---GGCGCAGCGTCG---
ATGACGCTGACGGTACAG---GCCAGACTA---TTATTG---TCTGGTATAGTGCAACAGCAGAAC---
AATTTG---CTGAGG---GCTATTGAGGCGCAACAACATATGTTGCAACTCACAGTCTGGGGCATC
>GU728304
TTCTATTGTAATACAACACAACTGTTTAATAGTACTTGGAATGAGACTAACATAAATAGCACTGGA-------
----

GGAAATGGCACAATCACACTCCCATGCAGAATAAAACAGATTATAAATATGTGGCAGCAAGTAGGAAAAGCAA
TGTACGCCCCTCCCATCAGGGGACAAATTAACTGTACATCAAAT---
ATTACAGGGCTGCTATTAACAAGAGATGGTGGGATAAACACGACC----------------------------
----

AACACCACTGAGATCTTTAGACCTGCAGGAGGAGATATGAGGGACAATTGGAGAAGTGAATTATATAAATATA
AAGTAGTAAAAATTGAACCATTA---GGAATAGCACCCACCAAGGCAAGGAGAAGAGTGGTG-----------
----------------------------CAGAGAGAAAAAAGAGCA---GTGGGG---ATAGGAGCT------
---CTGTTCCTTGGGTTCTTGGGAGCA---GCAGGAAGCACTATG---GGCGCAGCATCA---
ATAACGCTGACGGTACAG---GCCAGAAAC---TTATTG---TCTGGTATAGTGCAACAGCAGAAC---
AATATG---CTGAGG---GCTATTGAGGCGCAACAACATATATTGCAACTCACGGTCTGGGGCATC
>GU728410
TTCTACTGTAACACATCACGGCTGTTTAATAGCACATGGTATGTAAATGGAAGTAGTACTGGAGAG-------
----

TCAAATAGCACTATCACCCTCCCATGTAGGATAAAACAAATTATAAACATGTGGCAGAAAGTAGGAAAGGCAA
TGTATGCCCCTCCTATCAAAGGACAAATTAAATGTACATCAAAC---
ATTACAGGGCTGCTATTAACAAGAGATGGTGGTAATAATAGCCAG----------------------------
----

CCCGAAGAAATCTTCAGACCTGCAGGGGGAGATATGAGGGACAATTGGAGAAGTGAATTATATAAATATAAAG
TAGTAAAAATTGAACCATTA---GGAATAGCACCCACCAAGGCACAGAGAAGAGTGGTG--------------
-------------------------CAGAGAGCAAGAAGAGCA---GTGGGA---CTAGGAGCT---------
TTGTTCCTTGGGTTCTTGGGAGCA---GCAGGAAGCACTATG---GGCGCAGCGTCA---
GTGACGCTGACGGTACAA---GCCAGACAA---TTATTG---ACTGGTATAGTGCAACAGCAGAGC---
AATTTG---CTGAGA---GCTATTGAGGCGCAACAGCATCTGTTGCAACTCACAGTCTGGGGCATC
>GU728295
TTCTACTGTAATACAACAAAACTGTTTAATAGTACATGGAATGAGACTGATAGTAACAATACTAAA-------
----

GGAGAAGACACTATCACACTCCCATGCAGAATAAAACAAATTATAAACCTGTGGCAGGAAGTAGGAAAAGCAA
TATATGCCCCTCCCATCAGAGGACAAATTAACTGTTCATCAAAT---
ATTACAGGACTGTTATTAACAAGTGATGGGGGTGGGGGTGGCAAC----------------------------

----

AGGACGGACGAGACCTTCAGACCTATAGGAGGAGATATGAGGGACAATTGGAGAAGTGAATTATATAAATATA
AAGTAGTAAGAATTGAACCATTA---GGAGTAGCACCCACCAAGGCAAAGAGAAGAGCGGTG-----------
----------------------------CAGAGAGAAAAAAGAGCA---ATAGGGGACTAGGAGCT-------
---TTTTTCCTTGGGTTCTTGGGAGCA---GCAGGAAGCACTATG---GGCGCAGCGTCA---
CTGACGCTGACGGTACAG---GCCAGACTA---TTATTG---TCTGGTATAGTGCAACAGCAGAGC---
AACTTG---CTGAGA---GCTATTGAGGCGCAACAGCATCTGTTGCAACTCACTGTCTGGGGCATC
>GU728383
TTCTATTGTGATACAACATCACTGTTTAACAGTACTTGGAATAACAGTACTTGGGAGAATAATACTAAAGGAA
TC---

CTCCCAAACGCAACCATCACACTCCCATGCAGAATAAAGCAAATTATAAACATGTGGCAAGGAGTAGGAAGAG
CAATGTATGCCCCTCCTATTAGAGGACAAATTAGATGTTCATCAAAT---
ATTACAGGGCTACTGTTAACAAGAGATGGTGGTATTGGTAATAATATC-------------------------
----

ACCAATGGGTCCGAGACCTTCAGACCTGGAGGAGGGAATATGAAGGACAATTGGAGAAGTGAATTATATAAAT
ATAAAGTAGTACAACTTGAACCAGTA---GGAATAGCACCCACCAAGGCAAGGAGAAGAGTGGTG--------
-------------------------------CAGAGAGAAAAAAGAGCA---ATAGGA---CTAGGAGCT---
------ATGTTCCTTGGGTTCTTGGGAGCA---GCAGGAAGCACTATG---GGCGCAGCGTCA---
CTAACGCTGACGGTACAG---GCCAGACAA---TTATTG---TCTGGTATAGTGCAACAGCAGAAC---
AATTTG---CTGAGA---GCTATTGAGGCGCAACAGCATATGTTGCAACTCACAGTCTGGGGCATC
>FJ469729
TTCTACTGTGATTCATCAGGACTGTTTAATAGTACTTGGAATGGAACACACCTGGATACTTGGAATGGT----
----

ACTGGAGAAAATATCACACTCCAATGCAGGATAAGACAAATTATAAACATGTGGCAGGAGGTAGGAAAAGCAA
TGTATGCCCCTCCCATCAAGGGAATAATTAAATGTTCATCAAAC---
ATTACAGGATTGCTATTAACAAGAGATGGTGGTCCTGGTAATAGC----------------------------
----

ACCAATGAGACCTTCAGACCTGAAGGAGGAGATATGAGGAACAATTGGAGAAGTGAATTATATAAATACAAAG
TAGTACAAATTGAGCCATTA---GGAGTAGCACCCACCAAGGCAAGGAGAAGAGTGGTG--------------
-------------------------CAGAGAGAAAAAAGAGCA---GTGGGA---ATAGGAGCT---------
GTGCTCCTTGGGTTCTTGGGAGCA---GCAGGAAGCACTATG---GGCGCAGCGTCA---
ATGGCGCTGACGGTACAA---GCCAGACAA---TTATTG---TCTGGTATAGTGCAACAGCAGAAC---
AATTTG---CTGAGA---GCTATTGAGGCGCAACAACATCTGTTGCAACTCACAGTCTGGGGCATT
>DQ886033
TTCTACTGTAATACAACAAAACTTTTTAATAGTACTTGGATGTGGAATACTACTACTAGTAATTGGACTCTAA
ATAGCACTACA--
-------------GGGGCA------
GATGACAATGACATAATCATACTCCCATGCAGAATAAAACAAATTATAAACATGTGGCAGGAAGTAGGAAAAG
CAATGTATGCCCCTCCCATCCAAGGACAAATTAGCTGTTCATCAAAT---
ATCACAGGGTTGCTGTTAACAAGAGATGGTGGTATTAACAATGACACG-------------------------
----

AATGGGACTGAGATCTTCAGACCTGGAGGGGGAGATATGAGGGACAATTGGAGAAGTGAATTATATAAATATA
AAGTAGTAAAAATTAAACCATTA---GGAATAGCACCCACCAGGGCAAAAAGAAGAGTGGTG-----------
----------------------------CAGAGAGAGAAAAGAGCA---GTGGGA---CTAGGAGCT------
---TTGTTCCTTGGGTTCTTGGGAGCA---GCAGGAAGCACTATG---GGCGCAGCGTCA---
GTGACGCTGACGGTACAG---GCCAGACAA---TTATTG---TCTGGTATAGTGCAACAGCAGAAC---
AATTTG---CTGAGA---GCTATTGAGGCGCAACAGCATATGTTGCAACTCACAGTCTGGGGCATC
>GU728280
TTCTACTGTAATACAACAAAACTGTTTACTTGGAATAGTACTTGGGATAATATTGAAAAAGCAAATAAC----
----

ACTGACTCAAATATCACACTCCCATGCAGAATAAAACAAATTATAAACATGTGGCAGGAAGTAGGAAAAGCAA
TGTATGCCCCTCCCATCAAAGGAAGCATTATGTGTTCATCAAAC---
ATTACAGGGTTACTATTAACAAGAGATGGTGGTAACAACGGGACA----------------------------

----

AACGATACTGAAATCTTCAGACCTGGAGGAGGAGATATGAGGGACAATTGGAGAAGTGAATTATATAAATATA
AAATAGTACAAATTGAACCATTA---GGAATAGCACCCACTAGGGCAAGGAGAAGAGTGGTG-----------
----------------------------CAGAGAGAAAAAAGAGCA---ATAGGA---ATAGGAGCT------
---TTGTTCCTTGGGTTCTTGGGAGCA---GCAGGAAGCACTATG---GGCGCAGCGTCA---
ATGACGCTGACGGTACAG---GCCAGACAA---GTATTG---TCTGGTATAGTGCAGCAGCAGAAC---
AATCTG---CTGAAG---GCTATTGAGGCGCAACAGCATCTGTTGCAACTCACAGTTTGGGGCATC
>GU728339
TTTTACTGTAATACAACACAACTGTTTAATAGTACTTGGAATGGTACTGAGAATGGTACTGAGAATGATACTA
CA---

GGAGAAGAAACTATCATACTCCCATGCAGGATAAAACAAATTATAAACAGGTGGCAGGAAGTAGGAAAGGCAA
TGTATGCCCCTCCCATCAGTGGACAAATTAAATGTTCATCAAAT---
ATTACAGGGCTGCTATTAACAAGGGATGGC---AATACCAACGGT----------------------------
----

ACTGAGATCTTCAGACCTGGAGGAGGAGATATGAGGGACAATTGGAGAAGTGAATTATACAAATATAAAGTAG
TAAAAGTTGAACCAGTA---GGAGTAGCACCCACCCAGGCAAAGAGAAGAGTGGTG-----------------
----------------------CAAAGAGAGAAAAGAGCA---GTGGGA---ATAGGAGCT---------
TTGTTCCTTGGGTTCTTGGGAGCA---GCAGGAAGCACTATG---GGCGCAGCGTCA---
ATGACGCTGACGGTACAG---GCCAGACAA---TTATTG---TCTGGTATAGTGCAACAGCAGAAT---
AATCTG---CTGAGG---GCTATTGAGGCGCAGCAGCATATGTTGCAACTCACAGTCTGGGGCATC
>DQ222211
TTCTACTGTAATTCAACACAACTGTTTAATAGTACTTGGAGAGGGTTTAAGAGCTCTAATAGTACTACTTGGA
ATAAAACCGGA--
-------------GGGTCA------
AATAACACCGGACTAATCACACTCCCATGCAGAATAAAACAAATTATAAACATGTGGCAGGAAGTAGGAAAAG
CAATGTATGCCCCTCCCATCAGAGGACAAATTAGATGTTCGTCAAAT---
ATTACAGGGCTGCTATTAACAAGAGATGGTGGTAATGATAATGACCCG-------------------------
----

AACAGGACTGAGATCTTCAGACCTGGAGGAGGAGATATGAGGGACAATTGGAGAAGTGAATTATATAAATATA
AAGTAGTAAAAATTGAGCCATTA---GGAGTAGCACCCACCAAGGCAAAAAGAAGAGTGGTG-----------
----------------------------CAGAGAGAAAAAAGAGCA---GTGGGA---CTAGGAGCT------
---ATGTTCCTTGGGTTCTTGGGGGCA---GCAGGAAGCACTATG---GGCGCAGCGTCA---
GTGACGCTGACGGTACAG---GCCAGACAA---TTATTG---TCTGGTATAGTGCAACAGCAGAAC---
AATTTG---CTGAGG---GCTATTGAGGCGCAACAGCATCTGTTGCAACTCACAGTCTGGGGCATC
>GU367407
TTCTACTGTGATACAACACAACTGTTTAATAGTACTTGGGTTAATAGTACATGGAATAATACTGAAGAG----
----

GTGAATGGCACAATCACACTCCCATGCAAAATAAAACAGATTATAAACATGTGGCAGGGAGTAGGAAAAGCAA
TGTATGCCCCTCCCATCAGTGGAAAAATTAGATGTTCATCAAAT---
ATTACAGGGCTGCTATTAACAAGAGATGGTGGTGACAGTAGTAACAGT-------------------------
----

AATAGCAATGAAACCTTCAGACCTATAGGAGGAGATATGAAGGACAATTGGAGAAGTGAATTATATCCATATA
AAGTAGTAAGAATTGAACCATTA---GGAATAGCACCCACCAAGGCAAAGAGAAGAGTGGTG-----------
----------------------------CAGAAAGAAAAAAGAGCA---TTGGGTGGTTTAGGAGCT------
---TTGTTCCTTGGGTTCTTGGGAACA---GCAGGAAGCACTATG---GGCGCAGCGTCA---
CTGACGCTGACGGTACAG---GCCAGACAA---TTATTG---TCTGGTATAGTGCAACAGCAGAAT---
AATTTG---CTGAGG---GCTATTGAGGCGCAACAGCATCTGTTGCAACTCACAGTCTGGGGCATC
>GU367408
TTCTACTGTAATACAACACAACTGTTTAATAGTACTTGGAATAGTACTACTTGGAATGGAAATAATACAGATA
AC---

ACTGACAAAAACATCACACTCCAATGCAAAATAAAACAAGTTATAAACATGTGGCAGACAGTGGGAAAAGCAA
TGTATGCCCCTCCCATCCCAGGACAAATTAGATGTTCATCAAAT---
ATTACAGGGCTGCTATTAACAAGAGATGGTGGTAACGAGACCTGG----------------------------

----

GAGACCTTCAGACCTGGGGGAGGAAATATGAGGGACAATTGGAGAAGTGAATTATATAAATATAAAGTGGTGA
AAATTGAACCATTA---GGAATAGCACCCACCAAGGCAAAGAGAAGAGTGGTG----------------------
-------------------CAGAGAGAAAAAAGAGCA---GTGGGA---ATAGGAGCT---------
GTGTTCCTTGGGTTCTTGGGAGCA---GCAGGAAGCACTATG---GGCGCAGCGTCA---
TTGACGCTGACGGTACAG---GCCAGACTA---TTATTG---TCTGGTATAGTGCAACAGCAGAAT---
AATCTG---CTGAGG---GCTATTGAGGCGCAACAGCATATGTTGCAGCTCACAGTCTGGGGCATC
>KP754463
TTCTACTGTGATACAACACAATTGTTTAATAGTACTTGGAATGGGACTAATACTGAGAATAAAACTAAAGAGT
CA---

GATGACACTACTATCACACTCCCATGTAGGATAAAACAAATTATAAACATGTGGCAGGAAGTAGGAAGAGCAA
TGTATGCCCCTCCCATCAGCGGCAAATTAACTGTTCATCAAAT---
ATTACAGGGCTGATATTAATAAGAGATGGTGGAGACAAGAATACT------------------------------
----

ACCGAGACCTTCAGACCTGGAGGAGGAGATATGAGGGACAATTGGAGAAGTGAATTATATAAATACAAAGTAG
TGAGCCTTGAGCCAATA---GGAATAGCACCAACAAAGGCAAAGAGAAGAGTGGTG-------------------
----------------------CAGAGAGAAAAAAGAGCA---GTGACC---CTGGGAGCT---------
ATGTTCCTTGGATTCTTGGGAGCA---GCAGGAAGCACTATG---GGCGCAGCATCA---
ATAACGCTGACGGTACAG---GCCAGACAA---TTATTG---TCTGGTATAGTGCAACAGCAGAAT---
AATCTG---CTGAGA---GCTATTGAGGCGCAACAGCATATGTTGCAACTCACAGTCTGGGGCATC
>KP754465
TTCTACTGTAATACAACAAAACTGTTTGAGGGTACTTGGAATGGTAATAATTGGACTACAGAGTCAAATAAAA
CTGAAGAC---

AATAACACAACAATCACAATTCCATGCAGAATAAAACAAGTTATAAACCTGTGGCAGACAGTAGGAAAAGCAA
TGTATGCCCCTCCTATTGCAGGACTAATTAACTGTTCATCAAAT---
ATTACAGGACTGTTATTAATAAGAGATGGGGGTAAAAATAGCACT------------------------------
----

AATGAAACCTTCAGACCTGGAGGAGGAGATATGAGAGACAATTGGAGAAGTGAATTATACAAATATAAAGTAG
TAAAAATTGAACCATTA---GGAATAGCACCCACTAAGGCAAAGAGAAGAGTGGTG-------------------
----------------------CAGAGAGAAAAAAGAGCA---GTGGGA---ATAGGAGCT---------
GTGTTCCTTGGGTTCTTAGGAGCA---GCAGGAAGCACTATG---GGCGCAGCGTCA---
ATGACGCTGACGGTACAG---GCCAGACAA---TTATTG---TCTGGTATAGTGCAGCAGCAGAAC---
AATCTG---CTGAGG---GCTATTGAGGCGCAACAACATCTGTTGCAACTCACAGTCTGGGGCATC
>KP754466
TTCTACTGTAATACATCACAACTGTTTAATAGTACTTGGAATAGTACTACTGACCATACTAACCTTACA----
----

GGAGATGCAATTATCACGCTCCCATGCAGAATAAAACAAATTATAAACATGTGGCAAGGAGTAGGAAGAGCAA
TGTATGCCCCTCCCATCAGAGGACAAATTAACTGTTCATCAAAT---
ATTACAGGGCTGCTATTAACAAGAGATGGTGGTGTTAACAATACCACCGAT------------------------
----

AATAATACCGAGATCTTCAGACCAGGAGGAGGAGATATGAGGGACAATTGGAGAAGTGAATTATATAAATATA
AAGTGGTAAAAATTGAACCATTA---GGAATAGCACCCACCAAGGCAAAGAGAAGAGTGGTG-----------
-----------------------------CATAGAGAAAGAAGAGCG---GCA------CTAGGAGCT------
---ATGTTCCTTGGGTTCTTGGGAGCA---GCAGGAAGCACTATG---GGCGCAGCGTCA---
GTGACGCTGACGGTACAG---GCCAGACTA---TTATTG---TCTGGTATAGTGCAGCAGCAGAAC---
AATTTG---CTGAGG---GCTATTGAGGCGCAACAGCATATGTTGCAACTCACAGTCTGGGGCATC
>KP754468
TTCTACTGTAATACAGCGAAACTGTTTAACAGTACTTGGTTCAATAACACTGAAGGCATAAATACCACTGAAG
GAAATGGCAAT--

ATCACACGCAATATCACACTCCAATGCAGAATAAAACAATTTATAAACATGTGGCAGGAAGTAGGAAAAGCAA
TGTATGCCCCTCCCATCAGTGGACCGATTAGCTGTAAATCAAAT---
ATTACAGGGTTGATATTAACAAGAGATGGTGGTGAGGATAACAAC------------------------------

----

ACCGAGACCGAGATTTTTAGACCTGGAGGAGGAGATATGAGGGACAATTGGAGAAGTGAATTATATAAATATA
AAGTAGTAAGAATTGAACCATTA---GGAGTAGCACCCACCAAGGCAAAGAGAAGAGTGGTG-----------
----------------------------CAGAGAGAAAAAAGAGCA---GTGGGG---CTGGGAGCT------
---TTGTTCCTTGGGTTCTTGGGAGCA---GCAGGAAGCACTATG---GGCGCAGCGTCA---
TTGACGCTGACGGTACAA---GCCAGACAA---TTATTG---TCTGGTATAGTGCAGCAGCAGAAC---
AATCTG---CTGAGG---GCTATTGAGGCGCAACAGCATCTGTTGCAACTCACTGTCTGGGGCATC
>KP754470
TTCTACTGTAACACAGCGAGACTGTTTAATAGTACTTGGGGGGTTAATAGCACGGGAAAATCAAATAAC----
----

AATGAAACACATATCATACTCCCATGCAGAATAAAACAAATTATAAACATGTGGCAAAGAGTAGGAAAAGCAA
TGTATAACCCTCCCATCAGTGGACCAATTAGTTGTACATCAAAT---
ATTACAGGAATAATATTAACAAGAGATGGTGGACAGACCAATGAC----------------------------
----

AGTAGCACTGAGATCTTCAGACCTGGAGGAGGAGACATGAGGGATAATTGGAGAAGTGAATTATACAAATATA
AAGTAGTAAAAATTGAACCATTA---GGAGTAGCACCAACCAAGGCAAGGAGAAGAGTGGTG-----------
----------------------------CAGAGAGAAAAAAGAGCA---ATAGGA---CTAGGAGCT------
---CTGTTCCTTGGGTTCTTGGGAGCA---GCAGGAAGCACTATG---GGCGCAGCGTCA---
TTGACGCTGACGGTACAG---GCCAGACAA---TTATTG---TCTGGTATAGTGCAACAGCAGAAC---
AATTTG---CTGAGA---GCTATTGAGGCGCAACAGCATCTGTTGCAACTCACAGTCTGGGGCATT
>KP754472
TTCTACTGTAATTCAACACAACTGTTTAATAGTACTTGGATAAATGGTACTTGGACAAATGGTACTACAAATA
CCACT--

GGAGAAGATAAGATTATACTCCCATGCAGGATAAAACAAATTATAAACTTGTGGCAGGAAGTAGGAAAGGCAA
TGTACGCCCCTCCCATCAGAGGACAAATTACCTGTTCATCAAAT---
ATTACAGGGTTACTATTAACAAGAGATGGGGGTAATAAGAACGGGAGTAATGAG-------------------
----

GCCGTTGAGACCTTCAGACCTGCAGGAGGAGATATGAGGGACAATTGGAGAAGTGAATTATACAAATATAAAG
TAGTAAAAATTGAACCATTA---GGAGTAGCACCCACCAAGGCAAAGAGAAGAGTGGTG--------------
-------------------------CAGAGAGAAAAAAGAGCA---GTGGGA---TTAGGAGCT---------
TTGTTCATTGGGTTCTTGGGAGCC---GCAGGAAGCACTATG---GGCGCAGCGTCA---
GTGACGCTGACGGTACAG---GCCAGACAA---TTATTG---TCTGGTATAGTGCAACAGCAGAGC---
AATCTG---CTGAGG---GCTATTGAGGCGCAACAGCATCTGTTGCAACTCACAGTCTGGGGCATC
>KP754473
TTCTACTGTAATACTACACCACTGTTTAATAAAATTTGGTTTAATAGGACTGACTGGTCTAATAATACTGCAG
GGTCA--
-------------------AAT---
ACCACTAACGGCATAATTACACTCCAATGCAGGATAAAACAAATTATAAACATGTGGCAGGGAATAGGAAAAG
CAATGTATGCCCCTCCCATCAGAGGACAGATTAAATGTACATCAAAC---
ATTACAGGGCTGCTATTAACAAGAGATGGTGGTAATCCTTCAAACGCA-------------------------
----

ACCGATGAGATCTTCAGACCCATAGGGGGAAATATGAAGGACAATTGGAGAAGTGAATTATATAAATATAAAG
TAGTAAAACTTGAACCAATA---GGACTGGCACCCACCAAGGCGCAGAGAAGAGTGGTG--------------
-------------------------CAGAGAGAAAAAAGAGCA---GTGGGA---CTAGGAGCT---------
GTGTTCCTTGGGTTCTTGGGAGCA---GCAGGAAGCACTATG---GGCGCAGTGTCA---
ATGACGCTGACGGTACAG---GCCAGACAA---TTATTG---TCTGGTATAGTGCAACAGCAAAGC---
AATTTG---CTGAGG---GCTATTGAGGCGCAACAACATATGTTGCAACTCACAGTCTGGGGCATC
>DQ444258
TTCTACTGTAATACAACAAAACTGTTTAACAGTACTTGGAATGATAACAGTACTTGGAATGTTAACGGTACTG
GG---

AATGATAATGACACCATCATACTCCCATGCAGAATTAAACAAATTGTAAACATGTGGCAGGAAGTAGGAAAAG
CAATGTATGCCCCTCCCATCAGAGGACTAATTAAATGTTCATCAAAT---
ATCACAGGGCTGATATTAACAAGAGATGGTGGTAATAACATCACT----------------------------

----

AACGAGACCGAGATCTTCAGACCTGCAGGAGGAGATATGAAGGACAATTGGAGAAGTGAATTATATAAATATA
AAGTAGTACAAATTGAACCATTA---GGAGTAGCACCCACTAAGGCAAAGAGAAGAGTGGTG-------------
-----------------------------CAGAGAGAAAAAAGAGCA---GTGGGA---ATAGGAGCT-------
---TTGTTCCTTGGGTTCTTGGGAGCA---GCAGGAAGCACTATG---GGCGCAGCGTCA---
ATAACGCTGACGGTACAG---GCCAGGCAA---TTATTG---TCTGGTATAGTGCAACAGCAAAAC---
AACTTG---CTGAGG---GCTATTGAGGCGCAACAGCACCTGTTGCAACTCACAGTCTGGGGCATC
>GU728371
TTCTACTGTAACTCAACAAAACTGTTTAATAGTACTTGGTCTAATAATGATACTTGGAATAGTAGTGATGCTA
CA---

AGGTCAAATGAAACTATCACACTCCCATGCAGAATAAAACAAATTATAAACATGTGGCAGAAAGTAGGAAAAG
CGATGTACGCCCCTCCCATCAGAGGACAAATTAAATGTTCATCAAAT---
ATTACAGGGCTGCTGTTAGTAAGAGATGGTGGTACGAATAAGAGC------------------------------
----

GAGTCCGAGACATTCAGACCTGGAGGAGGAGATATGAGGGACAATTGGAGAAGTGAATTATACAAATATAAAG
TAGTAAAAATTGAACCATTA---GGAGTAGCACCCACCAAGGCAAAGAGAAGAGTGGTG----------------
--------------------------CAGAGAGAAAAAAGAGCA---CTGGGA---CTAGGAGCT----------
ATGTTCATTGGGTTCTTGGGAGCA---GCAGGAAGCACTATG---GGCGCAGCGTCA---
GTAACGCTGACGGTACAA---GCCAGACAA---TTATTG---TCTGGCATAGTGCAACAGCAGAAC---
AATCTG---CTGAGA---GCTATTGAGGCGCAACAGCATTTGTTGCAACTCACAGTCTGGGGCATC
>GU728322
TTCTACTGTAATACAACAAAACTGTTTAATAGTACTTGGCTTAATGGTACTTGGAATAGTAATGATACTGGA-
----

TTGTCAGAAAATATCACACTCCCATGCAGAATAAAACAAATTGTAAACATGTGGCAGGAAGTAGGAAAAGCAA
TGTATGCCCCACCCATCAGGGGACAAATTAGGTGTTCATCAAAT---
ATTACAGGGCTACTATTAGAAAGAGATGGTGGTGAGAACAACAGG------------------------------
----

TCCAATGAGACCTTCAGACCTATAGGAGGAAATATGAAGGACAATTGGAGAAGTGAATTATATAAATATAAAG
TAATAAAAATTGAACCATTA---GGAATAGCACCCACCAAGGCAAGGAGAAGAGTGGTG----------------
--------------------------CAAAGAGAAAAAAGAGCA---GTGGGA---ATAGGAGCT----------
ATGCTCTTTGGGTTCTTGGGAGCA---GCAGGAAGCACTATG---GGCGCAGCGTCA---
ATGACGCTGACGGTACAG---GCCAGGCTA---TTATTG---ACTGGTATAGTGCAACAGCAGAAC---
AATCTG---CTGAGG---GCTATTGAGGCGCAACAGCATCTGTTGCAGCTCACAGTCTGGGGCATC
>EF440778
TTCTACTGTAATTCAACAAAACTGTTTAATAGTACTTGGAATAGTGATGGTACTAGAAATAGTAGTGAAACGT
CA---

TCTGAAACAGTTATCACACTCCCATGTAGAATAAAACAAATTGTAAACATGTGGCAGGAAGTAGGAAAAGCAA
TGTATGCCCCTCCCATCAGTGGACAGATTAGGTGCTCATCAAAT---
ATTACAGGGCTAATATTAACAAGAGATGGTGGCAAAGGTGACAACATC---------------------------
----

ACCAACAACACCGAGATTTTCAGACCTGGAGGAGGAAATATGCAGGACAATTGGAGAAGTGAATTATATAAAT
ATAAGGTAGTACAAATTGAACCATTA---GGAATAGCACCCACCAAGGCAAAGAGAAGAGTGGTG---------
--------------------------------CAAAGAGAAAAAAGAGCA---GTGGGG---ATAGGAGCT---
------GTGTTCCTTGGGTTCTTGGGAGCA---GCAGGAAGCACTATG---GGCGCAGCGTCA---
ATAGCGCTGACGGCACAG---GCCAGACAA---TTGTTG---TCTGGTATAGTGCAACAGCAGAAC---
AATCTG---CTGAGG---GCTATTGAGGCGCAACAGCATCTGTTGCAACTCACAGTCTGGGGCATC
>GU728392
TTCTACTGCAACACATCACAACTGTTTAATAGTACTTGGAATGGTACTGAAGGGACAAATAACAATAAA----
----

GGAAATGAAACAATCACACTGCCATGCAGAATAAAACAGATTATAAACATGTGGCAGGAAGTAGGAAAAGCAA
TGTATGCTCCTCCCATCAGAGGACAAATTAGATGTTCATCAAAT---
ATTACAGGGTTGCTATTGACAAGAGATGGTGGTAATAGGACAAAC------------------------------

----

AATACAGAGACTTTCAGACCTGGAGGAGGAAATATGAAGGACAATTGGAGAAGTGAGCTATATAAATATAAAG
TAGTAAAAATTGAACCAATA---GGAATAGCACCCACCAAGGCAAAGAGAAGAGTGGTG--------------
-------------------------CAGAGAGAAAAAAGAGCA---GTGGGA---GTAGGAGCG---------
TTTATCATTGGATTCTTGGGAGCA---GCAGGAAGCACTATG---GGCGCAGCATCA---
ATGACGCTGACGGTACAG---GCCAGACTA---TTGTTG---TCTGGTATAGTGCAACAGCAAAGC---
AATTTG---CTGAAG---GCTATTGAGGCGCAACAACGTCTGTTGCAACTCACAGTCTGGGGCATC
>DQ886034
TTCTACTGTAACACAACAAAACTGTTCAATAGTACTTGGACTTGGAATAGTACTGAAGGGTCAGAA-------
----

GGAAATGAAACAATCACACTCCCATGCAGAATAAAACAAATTATAAACATGTGGCAGGAAGTAGGAAAAGCAA
TGTATGCCCCTCCCATCAGAGGACAAATTAAATGTTCATCAAAT---
ATTACAGGACTGCTATTAACAAGAGATGGTGGTGACCAAAATACC----------------------------
----

ACCACCGAGGTCTTCAGACCTGGAGGAGGAGATATGAGGGACAATTGGAGAAGTGAATTATATAAATATAAAG
TAGTGAAAATTGAGCCATTA---GGCATAGCACCCACCAAGGCAAAGAGAAGAGTGGTG--------------
-------------------------CAGAGAGAAAAAAGAGCA---GTGGGA---CTAGGAGCT---------
ATGTTCCTTGGGTTCTTGGGAGCA---GCAGGAAGCACTATG---GGCGCAGCGTCC---
ATAACGCTGACGGTACAG---GCCAGACTA---TTATTG---TCTGGTATAGTGCAACAGCAGAAC---
AATTTG---CTGAGG---GCTATTGAGGCGCAACAGCATCTGTTGCAACTCACAGTCTGGGGCATC
>DQ886035
TTCTACTGTGATTCAACACAACTGTTCAATAATACTTGGGATGGTACTAATATGTCAAATGCAAGA-------
----

AATGACACAAACATTACACTCCCATGCAGAATAAAACAATTTATAAACATGTGGCAGAGAGTAGGAAAAGCAA
TGTATGCCCCTCCCATCAGAGGAAACATTAGCTGTTCATCAAAT---
ATTACAGGGCTGATATTAACAAGAGATGGTGGTCCCAACGCGACC----------------------------
----

AACGAGACTTTCAGACCTGGAGGAGGAGATATGAGGGACAATTGGAGAAGTGAATTATATAAATATAAAGTAG
TAAGAATTGAACCATTA---GGAGTAGCACCCACCAAGGCAAAGAGAAGAGTGGTG-----------------
----------------------CAGAGAGAGAAAAGAGCA---GTGGGA---ATAGGAGCT---------
ATGTTCCTTGGGATCTTGGGAGCA---GCAGGAAGCACTATG---GGCGCAGCGTCA---
ATAACGCTGACGGTACAG---GCCAGACAA---TTATTG---TCTGGTATAGTGCAACAGCAGAGC---
AATTTG---CTGAGG---GCTATTGAGGCGCAACAGCATCTGTTGCAACTCACAGTCTGGGGCATC
>DQ886036
TTCTACTGTAATACAACACAACTGTTTAATAGTACTTGGTATGAGAATGAGACTATTGAATGGTCAAATAACA
CT---

GTAGGAAATGACATTATCACACTCCCATGCAGAATAAAACAAATTGTAAACATGTGGCAAGAGGTAGGAAAAG
CAATGTATGCCCCTCCCATCAGCGGACAAATTAGATGCTCATCAAAT---
ATTACAGGGTTACTATTAACAAGAGATGGTGGTAATAACACAAAT----------------------------
----

GAGACCGAGATTTTCAGACCTGTAGGAGGAAACATGAGGGACAATTGGAGAAGTGAATTATATAAATATAAAG
TAGTAAGAATTGAACCATTA---GGAGTAGCACCCACCAAGGCAAAGAGAAGAGTGGTG--------------
-------------------------CAGAGAGAAAAAAGAGCA---GTGGGA---CTAGGAGCT---------
ATGTTCCTTGGGTTCTTGGGAGCA---GCAGGAAGCACTATG---GGCGCAGCGTCA---
GTGACGCTGACGGTACAG---GCCAGACAA---TTATTG---TCTGGTATAGTGCAACAGCAGAAC---
AATCTG---CTGAGG---GCTATTGAGGCGCAACAGCATCTGTTGCAACTCACAGTCTGGGGCATC
>FJ469750
TTCTACTGCGATTCAACACCACTGTTTAATAGTACTTGGAATACTACTGAAGGAAATGACACAATCACACGC-
----

GGAAATGAAACAATCACACTCCCATGCAGAATAAAACAAATTATAAACATGTGGCAAGAAGTAGGAAAAGCAA
TGTATGCCCCTCCCATCAAAGGACAAATTAGCTGTTCATCAAAT---
ATTACAGGGCTGATATTAACAAGAGATGGTGGTAATAATAATGGGACC-------------------------

----

AATAACAATACTGAGATCTTCAGACCTGCAGGAGGAGATATGAGGGACAATTGGAGAAGTGAATTATATAAAT
ATAAAGTAGTAAAAATTGAACCATTA---GGAATAGCACCCACCACGGCAAAAAGAAGAGTGGTG--------
-------------------------------CAGAGAGAAAAAAGAGCA---GTAGGA---CTAGGAGCT---
------ATGTTCCTTGGGTTCTTGGGAGCA---GCAGGAAGCACTATG---GGCGCAGCGTCA---
CTGACACTGACGGTACAG---GCCAGACTA---TTATTG---TCTGGTATAGTGCAACAGCAGAAC---
AATTTG---CTGAGG---GCTATTGAGGCGCAACAGCATCTGTTGCAACTCACAGTCTGGGGCATC
>GU728413
TTCTACTGTAATACAACAAAACTATTTAATAGTACTTGGAATGATACTAAGGGGTCAATGACACTGTA----
----

GGAAATGACACAATCATACTCCCATGCAGAATAAAACAAATTATAAACCTGTGGCAGGAAGTAGGAAAAGCAA
TGTACGCCCCTCCCATCAGAGGACAAATTAGGTGTTCATCAAAT---
ATTACAGGGCTGCTATTAACAAGAGATGGTGGTAACGAGAGCAGC----------------------------
----

ACCACCGAGACCTTTAGACCTGGAGGAGGAAATATGAAGGATAATTGGAGAAGTGAATTATATAAATATAAAG
TAGTAAGAGTTGAACCATTA---GGAATAGCACCCACCATGGCAAGGAGAAGAGTGGTG--------------
-------------------------CAGAGAGAAAAAAGAGCA---GTGGGA---ATGGGAGCT---------
CTGTTCCTAGGGTTCTTAGGAGCA---GCAGGAAGCACTATG---GGCGCAGCGTCC---
GTAACGCTGACGGTACAG---GCCAGACAA---TTATTG---TCTGGTATAGTGCAACAGCAGAAC---
AATCTG---CTGAGA---GCTATTGATGCGCAACAGCATCTGTTGCAGCTCACTGTCTGGGGCATC
>GU728313
TTCTACTGTAATACAACACGACTGTTTAATAGTACCTGGAATAATACTACCTGGAGTAATTCTAATGTAGCAA
AT---

GGCACTGAAAGAATTATCACACTTCCATGCAGAATAAAACAAATTATAAACATGTGGCAGGAAGTAGGAAAAG
CAATGTATGCCCCTCCCATTAGAGGACAAATTAGTTGCTCATCAAAT---
ATTACAGGGCTACTGATAACAAGAGATGGTGGGAAGAATGTGACT----------------------------
----

AGGGGGACTGAGATCTTCAGACCTGGAGGAGGAGATATGAGGGACAATTGGAGAAGTGTATTATATAAATATA
AAGTAGTACAAATTGAACCATTA---GGAGTAGCACCCACCAAGGCAAAGAGAAGAGTGGTG-----------
----------------------------CAAAGAGAAAAAAGAGCA---GTGGGA---ATAGGAGCT------
---GTGTTCCTTGGGTTCTTGGGAGCA---GCAGGAAGCACTATG---GGCGCAGCGTCA---
ATGACGCTGACGGTACAG---GCCAGACAA---TTATTG---TCTGGTATAGTGCAACAGCAGAAC---
AATCTG---CTGAGA---GCTATTGAGGCGCAACAGCATCTGTTGCAACTCACAGTCTGGGGCATC
>GU728179
TTCTACTGTAATACAACACAACTGTTTAATAGTACTTGGCTTAACAGTACTTGGAATAATAGTGGAAATGATA
CTGCA--

GAATCAAATGACACTATCACACTCCCATGTAGAATAAGACAAATTATAAACATGTGGCAGCGAGTAGGAAAAG
CAATGTATGCCCCTCCCATCAGCGGAAAGATAAAGTGTATATCAAAT---
ATTACAGGGCTACTATTAACAAGAGATGGTGCTAGGAACAACAGC----------------------------
----

GAGAATGAGACCTTCAGACCCGAAGGAGGGAATATGAGGGACAATTGGAGAAGTGAATTATATAAATATAAAG
TAGTACAAATTGAACCATTA---GGAGTAGCACCCACTAAGGCAAAGAGGAGAGTGGTG--------------
-------------------------CAGAGAGAAAAAAGAGCA---GTGGGA---CTAGGAGCT---------
GTGTTCCTTGGGTTCTTGGGAGCA---GCAGGAAGCACTATG---GGCGCAGCGTCA---
CTGACGCTGACGGTACAG---GCCAGACAA---TTATTG---TCTGGTATAGTGCAACAGCAGAAC---
AATCTG---CTGAGA---GCTATTGAGGCGCAACAACATATGTTGCAACTCACAGTCTGGGGCATC
>GU728409
TTCTATTGCAGTACAACAGAACTATTTAATAGTACTTGGAATGTTACTGAAAAGTTAAATAAAACTAAAGAAA
AT---

GGAAGTGACACAATCACACTCCCATGCAGAATAAAACAAATTATAAACCTGTGGCAGGAAGTAGGAAAAGCAA
TGTATGCCCCTCCCATCAGCGGACAAATTAGATGTTCCTCAAAT---
ATTACAGGGCTGATATTAACAAGAGATGGTGGTAATGAAAACAAG----------------------------

----

ACCACCGAGACCTTCAGACCTGAAGGAGGAAATATGAGGGACAATTGGAGAAGTGAATTATATAAATATAAAG
TAGTACAAATTGAACCATTA---GGAGTAGCACCCACCAGGGCAAAAAGAAGAGTGGTG--------------
-------------------------CAGAGAGAAAAAAGAGCG---GGAATA------GGAGCT---------
CTGCTCCTTGGGTTCTTGGGAGCA---GCAGGAAGCACTATG---GGCGCAGCGTCA---
ATGGCGCTGACGGTACAG---GCCAGGCAA---TTATTG---TCTGGTATAGTGCAACAGCAGAAC---
AATCTG---CTGAGG---GCTATTGAGGCGCAACAGCATCTGTTGCAACTCACAGTCTGGGGCATT
>AF491737
TTCTACTGTGATACAACAAAACTGTTTAATAGTACTTGGAGTAGTAATAGTACTTGGAATGATACTACAGGGT
TAAATAAC--
-------------------ACT---
GGGGAGGATGATACAATCACACTCCCATGCAGAATAAAACAAATTATAGATATGTGGCAGGAAGTAGGAAAAG
CAATGTATGCCCCTCCCAGCAGTGGACAAATTAGATGTTCATCAAAT---
ATTACAGGGCTGTTATTAACAAGAGATGGTGGTATTAACGGGAGC---------------------------
----

GAAGGGAATGAGACCTTCAGACCTGGAGGAGGAGATATGAGGGACAATTGGAGAAGTGAATTATACAAATATA
AAGTAGTAAGAATTGAACCATTA---GGAGTAGCACCCACCAAGGCAAAGAGAAGAGTGGTG-----------
----------------------------CGGAGAGAAAAAAGAGCA---GTGGGA---TTAGGAGCT------
---ATGTTCCTTGGGTTCTTGGGAGCA---GCAGGAAGCACTATG---GGCGCAGCGTCA---
ATAGCGCTGACGGTACAG---GCCAGACAA---TTGTTG---TCTGGTATAGTGCAACAGCAGAAC---
AATTTG---CTGAGG---GCTATTGAGGCGCAACAACATTTGTTGCAACTCACAATCTGGGGCATC
>JN002043
TTCTACTGTGATTCATCACAACTGTTTAATAGTACTTGGATGTTTAATAGTACTTGGAATGATACTGACGGGG
CAAATAGC--
-------------------ACT---
AGGAAAAATGATACAATCACACTCCAATGCAGAATAAAACAAATTATAAACATGTGGCAGCAAGTAGGAAAAG
CAATGTATGCCCCTCCCATCAGAGGACAGATTAGATGTACATCAAAC---
ATTACAGGGCTGATATTAACAAGAGATGGTGGTGATACCCAAAACAAG------------------------
----

AGCGACCCCGAGATCTTTAGACCTGGAGGAGGAGATATGAGGGACAATTGGAGAAGTGAATTATATAAATATA
AAGTAGTAAAAATTGAACCACTA---GGAATAGCACCCACCAGGGCAAAGAGAAGAGTGGTG-----------
----------------------------CAGAGAGAAAAAAGAGCA---GTGGGAATGCTAGGAGCT------
---ATGTTCCTTGGGTTCTTGGGAGCA---GCAGGAAGCACTATG---GGCGCAGCGTCA---
CTGACGCTGACGGTACAG---GCCAGACAA---TTATTG---TCTGGTATAGTGCAACAGCAAAAC---
AATTTG---CTGAGA---GCTATTGAGGCGCAACAGCATCTGTTGCAACTCACAGTCTGGGGCATT
>AF049495
TTCTACTGTAATACAACAAAACTGTTTAATAGTACTTGGAATGAGACTACAGAGTCAAATGGC----------
----

AAGGGAGAAAATATCACACTCCCATGCAGAATAAGACAATTTGTAAACATGTGGCAGAAAGTAGGAAAAGCAA
TGTATGCCCCTCCCAGCGATGGACAAATTAGGTGTACATCAAAT---
ATTACTGGGCTACTATTAACAAGAGATGGGGGTGGTCCTAGTGATAAC------------------------
----

AAAACCGACAAGACCTTCAGACCAGGAGGAGGAGATATGAGGGACAATTGGAGAAGTGAATTATATAAATATA
AAGTAATAAAAATTGAACCATTA---GGAGTAGCACCCACCAAGGCAAAGAGAAGAGTGGTG-----------
----------------------------CAAAGAGAAAAAAGAGCA---GTGGGAATGGTAGGAGCT------
---ATGTTCCTTGGGTTCTTGGGAGCA---GCAGGAAGCACTATG---GGCGCAGCGTCA---
TTGACGCTGACGGTACAG---GCCAGACAA---TTATTG---TCTGGTATAGTGCAGCAGCAGAAC---
AATCTG---CTGAGA---GCTATTGAGGCGCAACAACATCTGTTGCAACTCACAGTCTGGGGCATC
>FJ469752
TTCTACTGTAATACAACAAAACTGTTTAATAGCACTTGGAATGACACTTGGGTTGATACTAGAAATACC----
----

ACTGAAGAAAATATCACACTCACATGCAGAATAAAACAAATTATAAACATGTGGCAGGAAGTAGGAAGAGCAA
TGTATGCCCCTCCCATCAGTGGATGGATTAACTGTACATCAAAT---
ATTACAGGGCTGCTATTGACAAGGATGGTGGGAATAAAACAGGC----------------------------

----

GAAAACGAGACCTTCAGACCTGGAGGAGGAAATATGATGGACAATTGGAGAAGTGAACTATACAAATATAAAG
TAGTAAAAATTGAACCATTA---GGAATAGCACCCACCAGGGCAAAGAGAAGAGTGGTG--------------
-------------------------CAGAGAGAAAAAAGAGCA---GTGGGAGCACTAGGAGCT---------
ATGTTCCTTGGGTTCTTGGGAACA---GCAGGAAGCACTATG---GGCGCAGCGTCA---
ATGGCGCTGACGGTACAA---GCCAGACAA---GTATTG---TCTGGTATAGTGCAGCAGCAGAAT---
AATCTG---CTGAGG---GCTATTGAGGCGCAACAGCATATGTTGCAACTCACAGTCTGGGGCATT
>GU728189
TTCTACTGTAACACAACACAGCTGTTTAATAGTACTTGGCTGTTTAATAGTACTTGGATTGATAATACTACA-
----

GGGTCAGAGAGACTCATACTCCCCTGCAAAATAAAACAAATTATAAACAGGTGGCAGGGAGTAGGGAAAGCAA
TGTATGCCCCTCCCATTAGAGGACTAATTAAATGCACATCAAAT---
ATCACAGGGCTAATATTAACAAGAGATGGCGGTCCCAACAACGAG----------------------------
----

AGCGAGACCTTCAGACCTGGAGGAGGAGATATGAGGGACAATTGGAGAAGTGAATTATATAAATATAAAGTAG
TAAAAATTGAGCCATTA---GGAGTAGCACCCACCAGGGCAAAGAGAAGAGTGGTG-----------------
----------------------CAAAGAGAAAAAAGAGCA---GTGGGAACAATAGGAGCT---------
ATGTTCCTTGGGTTCTTGGGAACA---GCAGGAAGCACTATG---GGCGCAGCGTCA---
ATGGCGCTGACGGTACAG---GTCAGACAA---TTATTG---TCTGGTATAGTGCAACAGCAGAAC---
AATTTG---CTGAGG---GCTATTGAGGCGCAACAGCATCTGTTGCAACTCACAGTCTGGGGCATC
>KU252650
TTCTACTGTAATACAACACAACTGTTTAAGAGTACTTGGTATGCTAATGGTTCTAGGAAAGGGTTAGATGAC-
----

CCTGGAGAAAATATCACACTCCCATGCAGGATAAAACAAATTATAAACATGTGGCAGGAAGTAGGAAAAGCAA
TGTATGCCCCTCCCATTAGTGGACCAATTAACTGTTTATCAAAT---
ATTACAGGGCTACTATTAACAAGAGATGGTGGGACAAACAATGAGACC-------------------------
----

AACAATACCGAGGTCTTCAGACCTGTAGGAGGGGATATGAGGGACAATTGGAGAAGTGAATTATATAAATATA
AAGTAGTAAGAATTGAACCATTA---GGAATAGCACCCACCAAGGCAAAAAGAAGAGTGGTG-----------
----------------------------CAGAGAGAAAGAAGAGCA---ATAGGA---ATAGGAGCT------
---GTGTTCCTTGGGTTCTTGGGAGCA---GCAGGAAGCACTATG---GGCGCAGCGTCA---
GTGGCGCTGACGGTACAA---GCCAGACAA---TTATTG---TCTGGTATAGTGCAACAGCAGAAC---
AATTTG---CTGAGA---GCTATTGAGGCGCAACAGCATCTGTTGCAACTCACAGTCTGGGGCATT
>GU728294
TTCTACTGTAACTCAACACAACTATTTAATAGTACTTGGCATAGAAATGGCACTGTAGTAGGGTTAAATACC-
----

ACTGAAGAAAATATCATACTCCCATGCAGGATAAAACAAATTATAAACATGTGGCAGAAAGTAGGAAAAGCAA
TGTATGCCCCTCCCATCAAGGGACCAATTAACTGTACATCAAAT---
ATCACAGGGCTGATATTAACAAGAGATGGTGGTAGTGAGGATATCACA-------------------------
----

AATAACACCGAGACCTTCAGACCTGGAGGAGGAAATATGGAGGACAATTGGAGAAGTGAATTATATAAATATA
AAGTAGTAAAAATTGAACCATTA---GGAGTAGCACCCACCAAGGCAAAGAGGAGAGTGGTG-----------
----------------------------CAAAGAGAAAAAAGAGCG---GGAATA------GGAGCT------
---GTGTTCCTTGGGTTCTTGGGAGCA---GCAGGAAGCACTATG---GGCGCAGCGTCA---
ATGACGCTGACGGTACAG---GCCAGACTA---TTATTG---TCTGGTATAGTGCAACAGCAGAGC---
AATCTG---CTGAGG---GCTATTGAGGCGCAGCAGCATCTGTTGCAACTCACAGTCTGGGGCATC
>FJ469756
TTCTACTGTGAGTCATTAGCATTGTTTAATAGTACCTGGCATGCTAATGGTACTATGGAAAGGACAAAT----
----

AGCAGTGAAAATATCACACTCCCATGCAGAATAAAACAAATTATAAACATGTGGCAGGGAGTAGGAAAAGCAA
TGTATGCCCCTCCCATCAGAGGACCAATTAAATGTTCATCAAAT---
ATTACAGGGCTAATATTAACAAGAGATGGTGGTAATATTAATGCCACAAATGGA-------------------

----

AATGGGAATGAGACCTTCAGACCTGGAGGAGGAAATATGAGGGACAATTGGAGAAGTGAACTATATAAAATAC
AAAGTAGTACAAAATTGAACCAATTA---GGAATAGCACCCACCAAGGCAAGGAGAAGAGTGGTG-------
------------------------------CAGAGAGAAAAAAGAGCA---GTGGGA---CTAGGAGCT---
------TTGTTCCTTGGGTTCTTGGGAGCA---GCAGGAAGCACTATG---GGCGCAGTGTCA---
ATGACGCTGACGGTACAG---GCCAGGCAA---TTATTG---ACTGGTATAGTGCAACAGCAGAAC---
AATTTG---CTGAAG---GCTATTGAGGCGCAACAGCATCTGTTGCAACTCACAGTCTGGGGCATC
>FJ469761
TTCTACTGTAACTCAACAAAACTGTTTAATAGTACTTGGAGTGCTAATGGTACTGGGGAAGAGTCAAATAGCA
CT--

GGAGATAATGATAATATCACACTCCCATGCAGAATAAAACAAATTATAAACATGTGGCAGGAAGTAGGAAGAG
CAATGTATGCCCCTCCCATCGGAGGACAAATTAGATGTTCATCAAAT---
ATTACAGGGCTGATATTAACAAGAGATGGTGGTAATAACACGAATGAG------------------------
----

ACTGAGCCCGAGATCTTCAGACCTGGAGGAGGAAATATGAAGGACAATTGGAGAAGTGAATTATATAAATACA
AAGTAGTAAAGATTGAACCGTTA---GGAGTAGCACCCACCAAGGCAAAGAGAAGAGTGGTG----------
---------------------------CAGAGAGAAAAAAGAGCA---GTGGGATTAGTAGGAGCT------
---ATGTTCCTTGGGTTCTTGGGAGCA---GCAGGAAGCACTATG---GGCGCAGCGGCA---
ATGACGCTGACGGTACAA---GCCAGACAA---TTATTG---TCTGGTATAGTGCAACAGCAGAAC---
AATTTG---CTGAGG---GCTATTGAGGCGCAACAACATTTGTTGCAACTCACAGTCTGGGGCATC
>FJ469763
TTCTACTGTAATACAACATCACTGTTCAATAGTACTTGGGATAGTAATGGTACTAGACAACTGTTCAATGGTA
CTGGG---

GATAATAACATCACAATCACACTCCCATGCAGAATAAAACAAATTATAAACTTGTGGCAGGAAGTAGGAAAAG
CAATGTATGCCCCTCCCATCAGCGGACCAATTAAATGTTCATCAAAT---
ATTACAGGACTTCTATTAACAAGAGATGGTGGTAACACGAACGAGACC------------------------
----

ACCAACAAGACCGAGGTCTTCAGACCTGCAGGAGGAGATATGAGGGATAATTGGAGAAGTGAATTATATAAAT
ATAAAGTAGTAAAAATTGAACCATTA---GGAGTAGCACCCACCAAGGCAAAGAGAAGAGTGGTG-------
------------------------------CAGAGAGAAAAAAGAGCA---ACA------ATAGGAGCT---
------TTGTTCCTTGGGTTCTTGGGAGCA---GCAGGAAGCACTATG---GGCGCAGCGTCA---
ATAACGCTGACGGTACAG---GCCAGACTA---TTATTG---TCTGGTATAGTGCAACAGCAGAAC---
AATCTG---CTGAGG---GCTATTGAGGCGCAACAACATCTGTTGCAACTCACAGTCTGGGGCATC
>FJ469766
TTCTACTGTAATTCAACACAACTGTTCAATAGTACTTGGCTTAGGAATAATACTGCAGAGTCAAATAGCACTG
AA--

AGAAATAACACAATCACACTCCCATGCAGAATAAAACAAATTATAAACATGTGGCAGGAGGTAGGAAAAGCAA
TGTATGCCCCTCCCATCAGAGGACAAATTAGATGTTCATCAAAG---
ATTACAGGGCTGCTATTAACAAGAGATGGTGGCAATAACGAGACT---------------------------
----

ACTGAGGAGGTCTTCAGACCTGGAGGAGGAGATATGAGGGATAATTGGAGAAGTGAATTATATAAATACAAAG
TGGTAAAAATTGAACCATTA---GGAGTAGCACCCACCAAGGCAAAGAGAAGAGTGGTG-------------
------------------------CAGAGAGAAAAAAGAGCA---GCGGGA---CTAGGAGCT---------
ATGTTCCTTGGGTTCTTGGGAGCA---GCAGGAAGCACTATG---GGCGCAGCATCA---
ATGGTGCTGACGGTACAG---GCCAGACAA---TTATTG---TCTGGTATAGTGCAACAGCAGAAC---
AATTTG---CTGAGG---GCTATTGAGGCGCAACAGCATCTGTTGCAACTCACAGTCTGGGGCATC
>GU728073
TTCTACTGCAATTCAACACAACTGTTTAATAGTACTTGGATTACGAATGGTACTGAATGGTCACCTAACACTA
CTTTA---

GGAGATACAGAAAATATCACACTCATATGCAGGATAAAACAAATTATAAACATGTGGCAGGAAGTAGGAAAAG
CCATGTATGCCCCTCCCATCGAAGGACAAATTAAATGCTCATCAAAT---
ATTACAGGGCTACTATTAACAAGAGATGGTGGTAATGGGAATGAGACC------------------------

----

AGGAACAATGAGACCTTCAGACCTACAGGAGGAAACATGAGGGACAATTGGAGAAGTGAATTATATAAATATA
AAGTAGTAAAAATTGAGCCATTA---GGACTAGCACCCACCAGGGCAAAGAGAAGAGTGGTG-------------
-----------------------------CAGAGAGAAAAAAGAGCA---GTGGGA---ATAGGAGCT------
---ATGTTCCTTGGGTTCTTGGGAGCA---GCAGGAAGCACTATG---GGCGCAGCGTCA---
ATAACGCTGACGGCACAA---GCCAGACAA---TTACTG---TCTGGTATAGTGCAGCAGCAGAAC---
AATTTG---CTGAGG---GCTATTGAGGCGCAACAGCATCTGTTGCAACTCACAGTCTGGGGCATC
>GU727875
TTCTACTGTAATACAACACAGCTGTTTAATAGTACTTGGAATTCTGATAGTACTCGGAATTGGACTGTACAGG
CAAAC--

AACAGTGGAGACAATATCACACTCCCATGCAGAATAAAACAAATTGTAAACATGTGGCAGGAAGTGGGAAAAG
CAATGTATGCCCCTCCCATTAAAGGACCAATTGGATGTTTATCAAAT---
ATTACAGGGCTGCTACTAACAAGAGATGGTGGGAATGGGAATGACACA-------------------------
----

AACACCACTGAGATCTTCAGACCTGGAGGAGGAAATATGAGGGACAATTGGAGAAGTGAATTATATAAATATA
AAGTAGTAAAAATTGAGCCATTA---GGAGTAGCACCCACCAAGGCAAAGAGAAGAGTGGTG-------------
-----------------------------CAGAGAGAAAAAAGAGCA---ATAGGAACACTGGGAGCT------
---GTGTTCCTTGGGTTCTTGGGAGCA---GCAGGAAGCACTATG---GGCGCAGCGTCA---
ATAACGCTGACGGTACAA---GCCAGACAA---TTATTG---TCTGGTATAGTGCAACAGCAGAAC---
AATCTG---CTGAAA---GCTATTGATGCGCAACAGCGTATGTTGCAACTCACAGTCTGGGGCATC
>GU728234
TTCTACTGTAATTCAACACAACTGTTTAATAGTACTTGGAATTCAACACAAATATATAATAGTACTTGGGATA
CAAAG--

AACACAACAGAAAGAATCATACTTCCATGCAGAATAAAACAAATTATAAACATGTGGCAGGAAGTAGGAAAAG
CAATGTATGCCCCTCCCATCCAAGGAATAATTAGCTGTGTATCAAAT---
ATTACAGGGCTGATATTAACAAGAGATGGTGGTGTGAACAGTAGC----------------------------
----

AATACCACCGAGACCTTCAGACCTGCAGGAGGAGATATGAGGGACAATTGGAGAAGTGAATTATATAAATATA
AAGTAGTAAAAATTGAGCCATTA---GGAGTAGCACCCACCAAGGCAAGGAGAAGAGTGGTG-------------
-----------------------------CAGAGAGAAAAAAGAACA---ATA------GCGGGAGCT------
---GTGTTCCTTGCGTTCTTGGGAGCA---GCAGGAAGCACTATG---GGCGCAGCGTCA---
ATAACGCTGACGGTACAG---GCCAGACAA---TTATTG---TCTGGTATAGTGCAACAGCAGAAC---
AATTTG---CTGAGA---GCTATTGAGGCGCAACAGCATATGTTGCAACTCACAGTCTGGGGCATC
>M95292
TTCTACTGTAATACAACAAAACTGTTTAATAGTACTTGGAATGATACTAGACTGTTTAATAGTACTTGGAATA
ATACTATAGGG--
-------------TCAAAT------
AACACTGAAGGAAATATCACACTCCCATGTAGAATAAAACAAATTATAAACATGTGGCAGGAAGTAGGAAAAG
CAATGTATGCCCCTCCCATCAGAGGACAAATTAGATGTTCATCAAAT---
ATTACAGGGCTGCTATTAACAAGAGATGGTGGTGAAACTAATGAG----------------------------
----

ACCACCGAGGTCTTCAGACCTGGAGGAGGAGATATGAGGGACAATTGGAGAAGTGAATTATATAAATATAAAG
TAGTAAAAATTGAACCATTA---GGAGTAGCACCCACCAGGGCAAAGAGAAGAGTGGTG----------------
--------------------------CAGAGAGAAAAAAGAGCA---GTGGGA---ATAGGAGCT---------
GTGTTCCTTGGGTTCTTGGGAGCA---GCAGGAAGCACTATG---GGCGCAGCGTCA---
GTGGCGCTGACGGTACAG---GCCAGACAA---TTATTG---TCTGGTATAGTGCAACAGCAGAAC---
AATTTG---CTGAGG---GCTATTGAGGCGCAACAGCACCTGTTGCAACTCACAGTCTGGGGCATC
>FJ469767
TTCTACTGTAATACAACACAGCTGTTTAATAGTACTTGGAATACAACTACTCTGGATAATTGGAATGAAACTA
CTTGG--

AATACTACTAAAATTATCACACTCCCATGCAGAATAAAACAAATTATAAACAGGTGGCAGGAAGTAGGAAAAG
CAATGTATGCCCCTCCCATCAGTGGACCAATTAGATGTGCATCACAT---
ATTACAGGGCTGCTATTAACAAGAGATGGGGGTAATAATGATAATTCT-------------------------

----

ACAAACGGGACTGAGGTCTTCAGACCTGGAGGAGGAGATATGAGGGACAATTGGAGAAGTGAATTATATAAAT
ACAAAGTAGTAAAAATTGAACCATTA---GGAATAGCACCCACCAAGGCAAAGAGAAGAGTGGTG--------
-------------------------------CAGAGAGAAAAAAGAGCA---GTGGGA---ATAGGAGCT---
------GTGTTCCTTGGGTTCTTGGGAGCA---GCAGGAAGCACTATG---GGCGCAGCGTCA---
ATGACGCTGACGGTACAG---GCCAGACTA---TTATTG---TCTGGTATAGTGCAACAGCAGAAC---
AATTTG---CTGAGA---GCTATTGAGGCGCAACAGCATATGTTGCAACTCACAGTCTGGGGCATT
>FJ469768
TTCTACTGTAATTCAACACAACTGTTTAATAGTACTTGGGATTCAGCACAACTATTTAATAGTACTTGGAATG
GCACG--
-------------------AAA---
GAAAATGACACGACAATCGTACTCCCATGCAGAATAAAACAAATTATAAACATGTGGCAGGAAGTAGGAAAAG
CAATGTATGCCCCTCCCATTAGAGGACAAATTAGATGTTTATCAAAT---
ATTACAGGGCTGATATTAACAAGAGATGGTGGTAATAACACTAGC----------------------------
----

AGGATTGAGACCTTCAGACCTGGAGGAGGAGATATGAGGGACAATTGGAGAAGTGAATTATATAAATATAAAG
TAGTTAAAATTGAACCATTA---GGAATAGCACCCACCAAGGCAAAGAGAAGAGTGGTG--------------
-------------------------CAGAGAGAAAAAAGAGCA---GTGGGA---CTAGGAGCT---------
ATGTTCCTTGGGTTCTTGGGAGCA---GCAGGAAGCACTATG---GGCGCAGCGTCA---
ATGACGCTGACGGTACAG---GCCAGACAA---CTGTTG---TCTGGTATAGTGCAACAGCAGAAC---
AATTTG---CTGAAG---GCTATTGAGGCGCAACAGCGTATGTTGCAACTCACAGTCTGGGGCATC
>FJ469769
TTCTACTGTAATACAACACAATTGTTTAACAGTACGTGGAATGCTACTGATATGTGGAACATTACTGAAGGGT
CAGGA--

AATACCACAACCAAAATCACACTCCCATGCAGAATAAAACAAGTTATAAACTTGTGGCAGGAAGTAGGGAAAG
CAATGTATGCCCCTCCCATCCAAGGACGAATTAGATGCTCATCAAAT---
ATTACAGGGCTGTTATTAACAAGAGATGGTGGTAAGGGCATTGAAACT-------------------------
----

AAAAATGACACTGAGACCTTCAGACCAGGAGGAGGAGACATGAGGGACAATTGGAGAAGTGAATTATATAAAT
ATAAAGTAGTAAAAATTGAACCATTA---GGAGTAGCACCCACCAGGGCAAAGAGAAGAGTGGTG--------
-------------------------------CAGAGAGACAAAAGAGCA---GTGGGA---ATAGGAGCT---
------GTACTCCTTGGGTTCTTGGGAGCA---GCAGGAAGCACTATG---GGCGCAGCGTCA---
ATAGCGCTGACGGCACAG---GCCAGACAA---TTACTG---TCTGGTATAGTGCAACAGCAGAAC---
AACTTG---CTGAGG---GCTATTGAGGCGCAACAGCATCTGTTGCAACTCACAGTCTGGGGCATC
>FJ469771
TTCTACTGTAATACAACACAATTGTTTAATAGTACTTGGAATAAAAATAGTACTTGGAATAATACTGAAAAGA
CAAT---

AGCACTGAAAACATTATCACACTCCCATGCAGGATAAAACAAATTATAAACATGTGGCAGAAAGTAGGAAAAG
CAATGTATGCCCCTCCCATCCAAGGACAAATTAAATGTTCATCAAAT---
ATTACAGGGATGATATTAACAAGAGATGGGGGTAATGAAAACAAC----------------------------
----

AACACCGAGATCTTCAGACCTGGAGGAGGAGATATGAGGGACAATTGGAGAAGTGAATTATATAAATATAAAG
TAGTAAAAATTGAACCATTA---GGAGTAGCACCCACCAAGGCAAAAAGGAGAGTGGTG--------------
-------------------------CAAAGAGAAAAAAGAGCA---GTGGGA---CTAGGAGCT---------
GTGTTCCTTGGGTTCTTGGGAACA---GCAGGAAGCACTATG---GGCGCAGCGGCA---
GTGACGCTGACGGTACAG---GCCAGACTA---TTATTG---TCTGGAATAGTGCAGCAGCAGAAC---
AATTTG---CTGAGA---GCTATTGAGGCACAACAGCATCTGTTGCAACTCACAGTCTGGGGCATC
>GU728204
TTCTACTGCAATACAACACAACTGTTTAATAGTACTTGGAATAGTAGTACTTGGAATAGTAATGATACTGAAG
GG---

TCAAATATCACAATCACACTCCCATGCAGAATAAAACAAATTATAAACATGTGGCAGGAAGTAGGAAAAGCAA
TGTATGCCCCTCCCATCAGGGGAAACATTAGCTGTTCATCAAAT---
ATTACAGGGCTGATATTAACAAGAGATGGTGGGAACAATACCACCAATAGTAAT-------------------

----
-
ACCACCAATACGACCACCGAGATCTTCAGACCTGGAGGGGGAGATATGAGGGATAATTGGAGAAGTGAATTAT
ATAAATATAAAGTAGTAAAAATTGAGCCATTA---GGAGTAGCACCCACCAAGGCAAAGAGAAGAGTGGTG--
-------------------------------------CAGAGAGAAAAAAGAGCA---TTGGGA---
ATGGGAGCT---------TTGTTCCTTGGGTTCTTGGGAGCA---GCAGGAAGCACTATG---
GGCGCAGCGTCA---GTGACGCTGACGGTACAG---GCCAGACAA---TTATTG---
TCTGGTATAGTGCAACAGCAGAAC---AATCTG---CTGAGA---
GCTATTGAGGCGCAACAGCATTTGTTGCAACTCACAGTCTGGGGCATC
>GU728223
TTCTACTGTAATTCAACAAATCTATTTAATAGTACTTGGACTTGGAATGGTACTTGGATTAATGGTACTGAAG
GA---

TCAAATGACACAATCACACTCCCATGCAGAATAAAGCAAATTATAAACATGTGGCAGGGAGTAGGACAAGCAA
TGTATGCCCCTCCCATTAAAGGACAAATTAGATGTTCATCGAAT---
ATTACAGGGTTGATATTAACAAGAGATGGTGGTAGTGATACCAATGAT-------------------------
----

ACCAGTAATGAGGTCTTCAGACCTGCAGGAGGAAATATGAAAGACAATTGGAGAAGTGAATTATATAAATATA
AAGTAGTAAGAATTGAACCATTA---GGAGTAGCACCCACCAGGGCAAGGAGAAGAGTGGTG-----------
----------------------------CAAAGAGAAAAAAGAGCA---GTGGGA---TTAGGAACT------
---GTGCTCTTTGGGTTCTTGAGTGCA---GCAGGAAGCACTATG---GGCGCAGCGTCA---
ATGACGCTGACGGTACAG---GCCAGACAG---TTATTG---TCTGGTATAGTGCAACAGCAGAAC---
AACCTG---CTGAGA---GCTATTGAGGCGCAACAGCATATGTTGCAACTCACAGTCTGGGGCATT
>GU728030
TTCTACTGTAATACAACAAAGCTGTTTAATAGTACTTGGCCGTCTAATGATACTAAAGGGTCAGAT-------
----

GGCAATGAAACTATCATACTCCCGTGCAGAATAAAACAAATTATAAACAGATGGCAGGAAGTAGGAAAAGCAA
TGTATGCCCCTCCCATCAGCGGAATAATTAGTTGTTCATCAAAT---
ATTACAGGGCTAATATTAACAAGAGATGGTGGTGACAATAACCAC----------------------------
----

AGCGAGACCTTTAGACCTGGGGGAGGAAATATGAGGGACAATTGGAGAAGTGAATTATATAAATATAAAATAG
TAAGGATTGAACCATTA---GGAGTAGCGCCCACCAGGTCAAGGAGACTAGTGGTG-----------------
----------------------CAGAGAGAAAAAAGAGCA---GTGGGAACGATAGGAGCT---------
ATGTTCCTTGGGTTCTTGGGAGCA---GCAGGAAGCACTATG---GGCGCAGCGTCG---
GTGACGCTGACGGTACAG---GCCAGACAA---TTATTG---TCTGGTATAGTGCAACAGCAGAAC---
AATTTG---CTGAAG---GCTATTGAGGCGCAACAGCATATGTTGCAACTCACAGTCTGGGGCATC
>GU728229
TTCTACTGTAATTCAACACCACTGTTTAATAGTACATGGGAAAGGAAGGGGGATGGTACTTGGAACAAGAGTG
AAACAAATAGC--
ACTAAAGGAAGGTCAAAC---------
AGCACTGAAGAAAAGATCATACTCCGATGCAGAATAAAACAAATTATGAACATGTGGCAGGAAGTAGGAATAG
CAATGTATGCCCCTCCCATTAACGGACTAATTAGATGTTCATCAAAT---
ATTACAGGGTTACTATTAACAAGGGATGGTGGTGAGACTAACGAGACT-------------------------
----

ACCAAAACTGAGACCTTCAGACCGGGAGGAGGAGATATGAGGGACAATTGGAGAAGTGAATTATATAAATATA
AAGTAGTAAAAATTGAACCATTA---GGTGTAGCACCCACCAAGGCAAAGAGAAGAGTGGTA-----------
----------------------------CAGAGAGAAAAAAGAGCA---ATAGGA---ATAGGAGCG------
---TTGTTCCTTGGGTTCTTGGGAGCA---GCAGGAAGCACTATG---GGCGCAGCGTCA---
CTAACGCTGACGGTACAA---GCCAGACAA---TTGTTG---TCTGGTATAGTGCAACAGCAGAAC---
AATTTG---CTGAGA---GCTATTGAGGCGCAACAACATCTGTTGCAACTCACAGTCTGGGGCATC
>AY189526
TTCTACTGTAATACATCACAAATGTTTAATGTTACTGAAGTGTCAAGGAATGACCCTAGA-------------
----

GCAAATGACACTATCACACTCCAATGCAGAATAAAACAAATTGTAAACATGTGGCAGAGAGTAGGAAGAGCAA
TGTATGCCCCTCCCATCAGTGGACTAATTAGATGTTCATCAAAT---

ATTACTGGGATAATGTTAACAAGAGATGGAGGTAAAATTGATAACGGA-------------------------
----

ACCAACAACACTGAGATCTTCAGACCTGGAGGAGGGGATATGAAGGACAATTGGAGAAGTGAATTATATAAAT
ATAAAGTAGTAAAAATTGAACCATTA---GGACTAGCACCCACCAAGGCAAAGAGAAGAGTGGTG--------
-------------------------------CAGAGAGAAAAAAGAGCA---GTGGGA---TTCGGAGCT---
------ATGTTCCTTGGGTTCTTGGGAGCA---GCAGGAAGCACTATG---GGCGCAGCATCA---
ATAACGCTGACGGTACAG---GCCAGAAAA---TTATTG---TCTGGTATAGTGCAACAGCAGAGC---
AATTTG---CTGAGG---GCTATTGAGGCGCAACAACGTATGTTGCAACTCACAGTCTGGGGCATC
>GU728370
TTCTACTGTAATACAACACAACTGTTTAATAGTACTTGGAATTCAACACAAACAGCTAATAGTAATAGTACTG
AC---

AATGGTACTGTAATCACACTCCCATGTAAAATAAAACAAATTATAAACATGTGGCAAGGAGTAGGAAAAGCAA
TGTATGCCCCTCCTATCAGAGGACAAATTAACTGCTCATCAAAT---
ATTACAGGGCTAATATTAACAAGAGATGGTGGTAATAACAATGAGAGC-------------------------
----

ACCGAGATCGAGATCTTCAGACCTGCAGGAGGAAATATGGAGGACAATTGGAGAAGTGAATTATACAAATACA
AAGTAGTAGAGATTAAACCATTA---GGAATAGCACCCACCAAGGCAAAAAGAAGAGTGGTG-----------
----------------------------CAGAGAGAAAAAAGAGCA---GTAGGAACGATAGGAGCT------
---ATGTTCCTTGGGTTCTTGGGAGCA---GCAGGAAGCACTATG---GGCGCAGCGTCA---
GTGACGCTGACGGTACAG---GCCAGACTA---TTATTG---TCTGGTATAGTGCAACAGCAAAAC---
AATTTG---CTGAAG---GCTATTGATGCGCAACAGCATCTGTTGCAACTCACAGTCTGGGGCATC
>GU728321
TTCTACTGCAATACAACAAAACTGTTTAATGGCACTTTGCATACAAATGGCACTTGGTATTTTAATGGCACTT
GGAATTTTACT--
----------GGAGAGTCA------
AATAGCACTGAAAATATCATACTCACATGCAGAATAAAACAAATTATAAACATGTGGCAGGAAGTAGGAAAAG
CAATGTATGCCCCTCCCATCAGTGGACAAATTAGATGTTCATCAAAT---
ATTACAGGGCTGCTATTAACAAGAGATGGTGGTAATAACAGTACCACA-------------------------
----

AACGAAAGTGAGACTTTCAGACCTGAAGGAGGAAATATGAAGGACAATTGGAGAAGTGAATTATATAAATATA
AAGTAGTGCAAATTGAACCATTA---GGAGTAGCACCCACCAAGGCAAAGAGAAGAGTGGTG-----------
----------------------------CAGAGAGAAAAAAGAGCA---ATAGGA---ATAGGAGCT------
---ATGTTCCTTGGGTTCTTGGGAGCA---GCAGGAAGCACTATG---GGCGCAGCGTCA---
ATGACACTGACGGTACAG---GCCAGACAA---TTATTG---TCTGGTATAGTGCAACAGCAAAAC---
AATTTG---CTGAGG---GCTATTGAGGCGCAACAGCATCTGTTGCAACTCACAGTCTGGGGCATC
>GU727980
TTCTACTGTAATTCAACACCACTGTTTAATAATATTGTGCGTACGGATGGGACTATAGAGAAAATTAAAAATG
ACACG--

GAAACAAATGACACAATCACACTCCAATGTAGAATAAAACAAATTATAAACATGTGGCAGGAAGTAGGAAAAG
CAATGTATGCCCCTCCCATCAGTGGACAAATTAGATGTTCATCAAAT---
ATAACAGGACTATTATTAACAAGAGATGGTGGTAATGGTGTTAACTTAACAGGA------
AATGGGGGTAACGTAACA---GGA---
AATAGTAGTACACACGGAAAT---------
GGGACCGAGATCTTCAGACCTGGAGGAGGAGATATGAGGGACAATTGGAGAAGTGAATTATATAAATATAAAG
TAGTAAGAATTGAACCATTA---GGAGTAGCACCCACCAGGGCAAAGAGAAGAGTGGTG--------------
-------------------------CAGAGAGAAAAAAGAGCA---GTAACA---CTAGGAGCT---------
ATGTTCCTTGGGTTCTTGGGAGCA---GCAGGAAGCACTATG---GGCGCAGCGTCA---
ATAACGCTGACGGTACAG---GCCAGACTA---TTATTG---TCTGGTATAGTGCAACAGCAGAAC---
AATTTG---CTGAGG---GCTATTGAGGCGCAACAACATCTGTTGCAACTCACAGTCTGGGGCATC
>GU728054
TTCTACTGTAATACAACACAACTGTTTAATAGTACTTGGACTTGGAATGATACTAACTGGAATGATACTAGAG
GGTCA--

AACAACTCTGAAAATATCACACTGCCATGCAGAATAAAACAAATCATAAACATGTGGCAGAGAGTAGGAAAAG
CAATGTATGCCCCTCCCATTAAAGGACAGATTAGCTGTTTATCAAAT---

ATTACAGGGCTGCTATTAACAAGAGATGGTGGTAATACTACCGAG----------------------------
----

ACCGAGACCTTCAGGCCTGCAGGAGGAGATATGAGGGACAATTGGAGAAGTGAATTATATAAATACAAAGTAG
TAAAAATTGAACCATTA---GGAATAGCGCCCACCAAGGCAAAAAGAAGAGTGGTG-----------------
----------------------CAGAGAGAAAAAAGAGCA---GTGGGA---CTAGGAGCT---------
ATGTTCCTTGGGTTCTTGGGAGCA---GCAGGAAGCACTATG---GGCGCAGCGTCC---
GTGACGCTGACGGTACAG---GCCAGACAA---TTGCTG---TCTGGTATAGTGCAACAGCAGAAC---
AATCTG---CTGCAA---GCTATTGAGGCGCAACAGCATCTGTTGCAACTCACAGTCTGGGGCATC
>GU728181
TTCTACTGTGATTCAACACCACTGTTTAATAGCACTTGGCATACTAATAATACTTTGGAGAGTACTAACGGG-
----

ACAAATGAAAATATCACACTCACATGCAGAATAAAACAAATTATAAACATGTGGCAGGAAGTAGGAAAAGCAA
TGTATGCCCCTCCCATCAGAGGACAAATTAGATGTATATCAGAT---
ATTACAGGGATGCTACTAACAAGAGATGGTGGTATTAACAACACCAATGGG----------------------
----

GAGAACAACACCGAGATATTCAGACCTGGAGGAGGAAATATGAGAGACAATTGGAGAAATGAATTATATAAAT
ATAAAGTAGTAAAACTTGAACCAATA---GGGTTAGCACCTACCAAGGCAAAGAGAAGAGTGGTA--------
-------------------------------CAGAGAGAAAAAAGAGCA---GTAACA---CTGGGAGCT---
------ATGTTCCTTGGGTTCTTGGGAGCA---GCAGGAAGCACTATG---GGCGCAGCGTCA---
CTGACGCTGACGGTACAG---GCCAGACTA---TTGTTG---TCTGGTATAGTGCAACAGCAGAGC---
AATCTG---CTGAGG---GCTATTGAGGCGCAACAGCATATGTTGCAACTCACAGTCTGGGGCATC
>EU604556
TTCTACTGTAATTCAACACAACTGTTTAATAGTACTTGGGATTCAACACAACTGTTTAATAGTACTTGGTATA
ACGAT--

ACAGGAAATGACACAATCATACTCCAATGCAGAATAAAACAATTTATAAACATGTGGCAGGAAGTAGGAAAAG
CAATGTATGCCCCTCCTATCAAAGGACAAATTAGCTGTTTGTCAAAT---
ATTACAGGGATACTATTAACAAGAGATGGTGGTAGTAACACCAGC----------------------------
----

AACACAACTGAGACCTTCAGACCTGGAGGAGGAGATATGAAGGATAATTGGAGAAGTGAATTATATAAATATA
AAGTAGTACAAATTGAACCATTA---GGAATAGCACCCACCAAGGCAAAGAGAAGAGTGGTG-----------
----------------------------CAGAGAGAAAAAAGAGCA---GTGGGA---ATAGGAGCT------
---TTATTCCTTGGGTTCTTGGGAGCA---GCAGGAAGCACTATG---GGCGCAGCGTCA---
ATGACGCTGACGGTACAG---GCCAGACAA---TTATTG---TCTGGTATAGTGCAACAGCAGAAC---
AATTTG---CTGAGA---GCTATTGAGATGCAACAGCATCTGTTGCAACTCACAGTCTGGGGCATC
>JX503075
TTCTATTGTAATACAACACAACTGTTTAATAGTACCTGGGATGTTAATAGTACCTGGAATGGAGGGTCAGAGA
ACACT--
-------------------ACA---
GGAGGAAATGATACAATCACACTCCCATGTAGAATAAAACAAATTATAAATATGTGGCAGGAAGTAGGCAAAG
CAATGTATGCCCCTCCCATCAAAGGACAAATTAGATGTTCATCAAAT---
ATTACAGGGCTACTACTAGTAAGAGATGGTGGAAATGATAATGAAACT-------------------------
----

AACGACACCGAGATCTTCAGACCTGGAGGAGGAGATATGAGGGACAATTGGAGAAGTGAATTGTATAAATATA
AAGTAGTAAGAATTGAACCATTA---GGAGTAGCACCCACCAAGGCAAAGAGAAGAGTGGTG-----------
----------------------------CAGAAGGAAAAAAGAGCA---ATAACA---TTAGGAGCT------
---ATGTTCCTTGGGTTCTTGGGAACA---GCAGGAAGCACGATG---GGCGCAGCGTCA---
ATAACGCTGACGGTACAG---GCCAGACAA---TTATTG---TCTGGTATAGTGCAGCAGCAGAAC---
AATCTG---CTGAGG---GCTATTGAGGCGCAACAACATTTGTTGCAACTCACAGTCTGGGGCATT
>AY781127
TTCTACTGTAATACAACAGAATTGTTTAATAGTACTTGGCCGTTTAATAGTACTGAAAATAAGGCTAACGAGT
CAGGG--

ACAAGTGAAAGAATATCACACTCCCATGCAGAATAAAACAAATTATAAACAGGTGGCAGGAAGTAGGAAAAG
CAATGTATGCCCCTCCCATCCAAGGACGAATTAGCTGTTCATCAAAA---

ATTACAGGGATGCTATTAACAAGAGATGGTGGTAAAGGGAACGAGACA-------------------------
----

AGGAACGATACCGAGACCTTCAGACCTGCAGGAGGAGATATGAAGGACAATTGGAGAAGTGAATTATATAAAT
ATAAAGTAGTAAAAATTGAGCCATTA---GGAATAGCACCCACCAGGGCACAGAGAAGAGTGGTG--------
-------------------------------CAGAGAGAAAAAAGAGCA---GTGGGAATGATAGGAGCT---
------ATGTTCCTTGGGTTCTTGGGAGCA---GCAGGAAGCACTATG---GGCGCAGCGTCA---
GTGACGCTGACGGTACAG---GCCAGACAA---TTATTG---TCTGGTATAGTGCAGCAGCAGAAC---
AATCTG---CTGAGG---GCTATTGAGGCGCAACAGCATCTGTTGCAACTCACAGTCTGGGGCATC
>JN235958
TTCTACTGTAATACAACACAGCTGTTTAATAGTACCTGGAATGATAGTACCTGGAATGGTACTGCAGGA----
----

CGGAATGATACTATTACACTCCCATGCAGAATAAGACAAATTATAAACAGGTGGCAGGAAGTAGGAAGAGCAA
TGTATGCCCCTCCCATCAGCGGAATGATTAGCTGTTCATCAAAT---
ATCACAGGCCTGCTATTAACAAGAGATGGGGGTAACAATAGTAAC----------------------------
----

AGCAACAACGAGACCTTCAGACCAGCAGGAGGAGATATGAGGGACAATTGGAGAAGTGAATTATATAAATATA
AAGTAGTAAAAATTGAGCCATTA---GGAATAGCACCCACCAAGGCAAAGAGAAGAGTGGTG-----------
----------------------------CAGAGAGAAAAAAGAGCA---GTAACG---CTAGGAGCT------
---ATGTTCCTTGGGTTCTTGGGAGCA---GCAGGAAGCACTATG---GGCGCAGCGTCA---
CTGACGCTGACGGTACAG---GCCAGACAA---TTATTG---TCTGGTATAGTGCAACAGCAGAAC---
AATTTG---CTGAGG---GCTATTGAGGCGCAACAGCATATGTTGCAACTCACAGTCTGGGGCATC
>AY795905
TTCTACTGTGAAACAGGAAGACTGTTTAATAGTACTTGGAATTTAACACAACTGACTTGGAATGATACTAAAG
AG---

TTAAATAACACGATCACACTCCCATGCAAAATAAAACAAATTATAAACATGTGGCAGGAAGTAGGAAAAGCAA
TGTATGCCCCTCCCATCGCAGGACAAATTAACTGTTTATCAAAT---
ATTACAGGGCTGATTTTAACAAGGGATGGTGGTAATAAGGGTAATGGGACC----------------------
----

GATGACACCACCGAGATCTTCAGACCTGGAGGAGGAGATATGAGGGACAATTGGAGAAGTGAATTATATAAGT
ATAAAGTAGTAAAAATTGAACCATTA---GGAATAGCACCCACCAAGGCAAAGAGAAGAGTGGTG--------
-------------------------------CAGAGAGAAAGAAGAGCA---GTAGGA---TTGGGAGCT---
------TTGCTCTTTGGGTTCTTGGGAGCA---GCAGGAAGCACTATG---GGCGCAGCGTCA---
ATAACGCTGACGGTACAG---GCCAGACAA---TTATTG---TCTGGTATAGTGCAGCAGCAAAAT---
AATTTG---CTGAGA---GCTGTGGAGGCGCAACAACATCTGTTGCAACTCACAGTCTGGGGCATC
>KJ948657
TTCTACTGTAATACAACACAACTGTTTAATAGTACTTGGCATGTTAATATGACTGGGAAT-------------
--GAT---
ACTGAGGCTGCTAAAAATATT---------------
ACAGGAAATGACACAATCACACTCCCATGCAGAATAAAACAAATTATAAACATGTGGCAGGAAGTAGGAAAAG
CAATGTATGCCCCTCCCATCAGAGGAATAATTAGATGTTCATCAAAT---
ATTACAGGGCTGCTATTAACAAGAGATGGGGGT--

-
AAAGAGAACAATGAGACTGAGATCTTCAGACCTGGAGGAGGAGATATGAGGGATAATTGGAGAAGTGAATTAT
ATAAATATAAAGTAGTACAAATTGAACCATTA---GGAGTAGCACCCACCAAGGCAAAGAGAAGAGTGGTG--
-------------------------------------CAAAGAGAAAAAAGAGCAATAACGGGA---
TTAGGAGTT---------ATGTTCCTTGGGTTCTTGGGAGCA---GCAGGAAGCACTATG---
GGCGCAGCGTCA---ATAACGCTGACGGTACAG---GCCAGACAA---TTATTG---
TCTGGTATAGTGCAACAGCAGAAC---AATCTG---CTGAGG---
GCTATTGAGGCGCAACAGCATCTGTTGCAACTCACAGTCTGGGGCATC
>KJ948660
TTCTACTGTAATACAACACAACTGTTTAATAATACTGGTATGGCGGGTGATAATTGG----------------
----
ACTACTAGTTGGAATAATACT------------
ACACGAAATGTCAATATCACACTCCCATGCAGAATAAAACAAATTATAAACATGTGGCAGGAAGTAGGAAAAG

CAATGTATGCCCCTCCCATCAATGGACACATTATATGTGCATCAAAT---
ATTACAGGGCTGCTATTAACAAGAGATGGTGGTATC------------------------------------
----
GAGGATAATGGGACTACTATT---------
AGGAATGAGACCTTCAGACCTGGAGGAGGAGATATGAAGGATAACTGGAGAAGTGAATTATATAAATATAAAG
TAGTAAAAATTGAACCATTA---GGAATAGCACCCACCAAGGCAAAGAGAAGAGTGGTG-------------
-------------------------CAGAGAGAAAAAAGAGCA---TTGGGA---ATAGGAGCT---------
TTGTTCCTTGGGTTCTTGGGAGCA---GCAGGAAGCACTATG---GGCGCAGCGGCA---
CTGACGCTGACGGTACAG---GCCAGACCA---TTATTG---TCTGGTATAGTGGAACAGCAAAAC---
AATTTG---CTGAGG---GCTATTGAGGCGCAACAGCATCTGTTGCAACTCACAGTCTGGGGCATC
>HQ595755
TTCTACTGTAATACAACACAACTGTTCAATAATACTTGGAATGGTACTACCTGGTCAAATAACAGGGCAACTA
AC--

AGCAGCAAAAATATCATCACACTCCCATGCAAAATAAAACAAATTATAAACATGTGGCAGCAGGTAGGAAAAG
CAATGTATGCCCCTCCCATCAGAGGACAAATCAGATGTTCATCAAAT---
ATTACAGGGCTGCTATTAACAAGAGATGGTGGTAATGATAATAATAAT------------------------
----

ACAAATGGGTCCGAGATCTTCAGACCTGGGGGAGGAGATATGAGGGACAATTGGAGAAGTGAATTATATAAAT
ATAAAGTAGTAACAATTGAACCAGTA---GGAATAGCACCCACCAAGGCAAAGAGAAGAGTGGTG--------
------------------------------CAGAGAGAAAAAAGAGCA---ATAGGA---ATAGGAGCT---
------GTGTTCCTTGGGTTCTTGGGAGCA---GCAGGAAGCACTATG---GGCGCAGCATCA---
ATAACGCTGACGGTACAG---GCCAGACAA---TTATTG---TCTGGTATAGTGCAACAGCAGAAC---
AATTTG---CTGAGG---GCTATTGAGGCGCAACAGCATATGTTGCAACTCACAGTCTGGGGCATC
>KP109515
TTCTACTGTAATACAACACAACTGTTCAATAATACTTGGAATGGTACTACCTGGTCAAATAACAGGGCAACTA
ACAGC---

AGCAAAAATATCATCACACTCCCATGCAAAATAAAACAAATTATAAACATGTGGCAGCAGGTAGGAAAAGCAA
TGTATGCCCCTCCCATCAGAGGACAAATCAGATGTTCATCAAAT---
ATTACAGGGCTGCTATTAACAAGAGATGGTGGTAATGATAATAATAAT------------------------
----

ACAAATGGGTCCGAGATCTTCAGACCTGGGGGAGGAGATATGAGGGACAATTGGAGAAGTGAATTATATAAAT
ATAAAGTAGTAACAATTGAACCAGTA---GGAATAGCACCCACCAAGGCAAAGAGAAGAGTGGTG--------
------------------------------CAGAGAGAAAAAAGAGCA---ATAGGA---ATAGGAGCT---
------GTGTTCCTTGGGTTCTTGGGAGCA---GCAGGAAGCACTATG---GGCGCAGCATCA---
ATAACGCTGACGGTACAG---GCCAGACAA---TTATTG---TCTGGTATAGTGCAACAGCAGAAC---
AATTTG---CTGAGG---GCTATTGAGGCGCAACAGCATATGTTGCAACTCACAGTCTGGGGCATC
>KF725915
TTCTACTGTAATTTAACACAGCTGTTTAATAGTACTTGGCGGAAAAGTAATAGTACTAGGCAGATGAATAACA
CT--
-------------GGAACA------
AGGTTAAATGGAACCATCATACTCCCATGCAGAATAAAACAAATTATAAACAGGTGGCAAGGAGTAGGAAGAG
CAATGTATGCCCCTCCCATCAGAGGACCAATTAATTGTTCATCAAAG---
ATTACAGGGCTACTATTAATAAGAGATGGTGGTAAAACCAATAAT---------------------------
----

TCTACAGAGATCTTCAGGCCTGGAGGGGGAAATATGAAGGACAATTGGAGAAGTGAATTATATAAATATAAAG
TAGTAAAAATTGAGCCATTA---GGAATAGCACCCACCAAGGCAAAGAGAAGAGTGGTG-------------
-------------------------CAGAGAGAAAAAAGAGCA---GTTGGAGTAATAGGAGCT---------
ATGTTCCTTGGGTTCTTGGGAGCA---GCAGGAAGCGCTATG---GGCGCAGCGTCA---
CTGACGCTGACGGTACAG---GCCAGACAA---TTATTG---TCTGGTATAGTGCAACAGCAGAAC---
AATCTG---CTGAGA---GCTATTGAGGCGCAACAGCATATGTTGCAACTCACAGTCTGGGGCATC
>KF725918
TTCTACTGTGATTCATCAAAACTGTTTAATAGTACTTGGCATGTTAATAGCACTGAAGGAAATAGC-------
----

ACTAATGAAGAAATCATACTCCCATGTAAAATAAAGCAAATTATAAACATGTGGCAGAAAGTAGGAAAAGCAA

TGTATGCTCCTCCCATCAAAGGGAAAATTAACTGTTTATCAAAC---
ATTACAGGACTGCTATTAACAAGGGATGGTGGTAATAATAGGACC----------------------------
----

AACGAGACCTTTAGACCTGTGGGAGGAGATATGAGGGACAATTGGAGAAGTGAATTATATAAATATAAAGTAG
TAAAAATTGAACCATTA---GGAGTAGCACCCACCAAGGCAAAGAGAAGAGTGGTG-----------------
----------------------CAGAGAGAAAAAAGAGCA---GTGGGAACGATGGGAGCT---------
ATGTTCCTTGGGTTCTTGGGAGCA---GCAGGAAGCACTATG---GGCGCAGCGTCA---
ATAACGCTGACGGTACAG---GCCAGACAA---TTATTG---ACTGGTATAGTGCAACAGCAGAAC---
AATTTG---CTGAAG---GCTATTGAGGCGCAACAGCATCTGTTGCAACTCACAGTCTGGGGCATC
>KF725946
TTCTACTGTAACACAACAAAACTGTTTAATAGTATGTGGAATAGTAGTACTTGGAGTAACAATAGTATTGAAG
ACTCA--
-------------------AAT---
AACACTGAGAACAATATCACACTCCCATGCAGGATAAAACAAATTATAAACATGTGGCAGAAGGTAGGAAAGG
CAATGTATGCCCCTCCCATCAGTGGACAAATTAGGTGTTCATCAAAT---
ATTACAGGGCTACTATTAACAAGAGATGGTGGTAATGATAGTAACACC-------------------------
----

AATAGCAAGGAGACCTTCAGACCTGGAGGAGGAGATATGAGGGACAATTGGAGAAGTGAGTTATATAAATATA
AAGTAGTAAAAATTGAACCATTA---GGAATAGCACCCACCAAGGCAAAGAGAAGAGTGGTG-----------
----------------------------CAGAGAGAAAAGAGAGCA---GTGGGA---ATAGGAGCG------
---TTGTTCCTTGGGTTCTTGGGAGCA---GCAGGAAGCACTATG---GGCGCAGCGTCA---
ATAACGCTGACGGTACAG---GCCAGACTA---TTATTG---TCTGGTATAGTGCAGCAGCAAAAC---
AATTTG---CTGAGG---GCTATTGAGGCGCAACAACACTTGTTGCAACTCACAGTCTGGGGCATC
>KF725977
TTCTACTGTAATACAACACAACTATTTAAGGGGTCTTGGAATGGTACTGAGTCTTGGAATGATACTGAGTCTT
GGAATGGT---

AATGACACCATTCTCGAAATCCCATGCAGAATAAAACAAATTGTAAACATGTGGCAGGAAGTAGGAAAAGCAA
TGTATGCCCCTCCCATCAGAGGACAAATTAGCTGTTCCTCAAAT---
ATTACAGGATTACTATTAACAAGAGATGGTGGTAACACCAATAGA----------------------------
----

ACCGACGAGACTTTTAGACCTGGAGGAGGAGATATGAGGGACAATTGGAGGAGTGAATTATATAAATATAAAG
TAGTAAAAATTGAACCATTA---GGAATAGCACCCACCAAGGCAAAGAGAAGAGTGGTG--------------
-------------------------CAGAGAGAAAAAAGAGCA---GTGGGA---CTAGGAGCT---------
ATGTTCCTTGGGTTCTTGGGAGCA---GCAGGAAGCACTATG---GGCGCAGCGTCA---
CTGACGCTGACGGTACAG---GCCAGACTA---TTATTG---TCTGGTATAGTGCAACAGCAGAAC---
AATTTG---CTGAGA---GCTATTGAGGCGCAACAACATTTGTTGCAACTCACAGTCTGGGGCATC
>KF726002
TTCTACTGTAATACAGCACAACTGTTTAATAGTACTTGGAATACAACACAACTTAATAATAATAATACTGGAG
GGGGC--

GACAATGAAACTATCACACTCCAATGTAAAATAAAACAAATTATCAACATGTGGCAGGGAGTAGGAAAAGCAA
TGTATGCCCCTCCCATTAAAGGACAAATTAGATGCACATCAAAT---
ATTACAGGGCTACTATTAACAAGAGATGGTGGTGGGAACGAGAGC----------------------------
----

ACGAACGAGACCTTCAGACCTGGAGGAGGAGATATGAGGGATAATTGGAGAAGTGAATTATACAAATATAAAG
TAGTGAAAATTGAACCATTA---GGAGTAGCACCCACCAAGGCAAGGAGAAGAGTGGTG--------------
-------------------------CAGAGAGAGAAAAGAACA---GTGGGA---CTAGGAGCT---------
CTGTTCCTTGGGTTCTTGGGAGCA---GCAGGAAGCACTATG---GGCGCAGCGTCA---
GTAACGCTGACGGTACAG---GCCAGACTA---TTATTG---ACTGGTATAGTGCAACAGCAGAAT---
AATTTG---CTGAGG---GCTATTGAGGCGCAACAACATCTGTTGCAACTCACAGTCTGGGGCATC
>KF726004
TTCTACTGTAATACAACAGGACTGTTTGATAGTACTTGGAATAATACTGAAAAAGTAAATGGCACT-------
----

AACAATGACACCATCACACTCCCATGCAGAATAAGGCAATTTATAAACATGTGGCAGAAAGTAGGAAAAGCAA

TGTATGCCCCTCCCATTAAAGGACAAATTAAATGTTCATCAAAT---
ATTACAGGACTGATATTAACAAGAGATGGTGGTAATAATAACAGTACC-------------------------
----

AACGAGACCGAGACCTTCAGACCTGCAGGAGGAAATATGAGGGACAATTGGAGAAGTGAATTATATAAATACA
AAGTAGTAAAAATTGAACCATTA---GGATTAGCACCCACCAAGGCAAAGAGAAGAGTGGTG-----------
----------------------------CAGAGAGAAAAAAGAGCA---GTGGGA---TTAGGAGCT------
---ATGTTCCTTGGGTTCTTGGGAGCA---GCAGGAAGCACTATG---GGCGCAGCGTCA---
CTGACGCTGACGGTACAA---GCCAGACAA---TTATTG---TCTGGTATAGTGCAGCAGCAAAAC---
AATTTG---CTGAGA---GCTATTGAGGCGCAACAACATATGTTGCAACTCACAGTCTGGGGCATC
>KF726016
TTCTACTGTAATACAACACAACTGTTTAATAGTACTTGGATTAATGATACTAGGAATGATACA----------
----

GGAAATGAAACAATCACACTCCCATGCAGAATAAAACAAATTATAAACATGTGGCAGGAGGTAGGAAAAGCAA
TGTATGCCCCTCCCATCAGTGGACAAATTAGATGTTCATCAAAT---
ATTACAGGGCTGATATTAACAAGAGATGGTGGGCAGAATAACACT----------------------------
----

AATGACACTGAGATCTTCAGACCTGGAGGAGGAAATATGAAAGACAACTGGAGAAGTGAATTATATAAATATA
AAGTAGTAAGAATTGAACCATTA---GGAGTAGCACCCACCAAGGCAAAGAGAAGAGTGGTG-----------
----------------------------CAAAGAGAAAAAAGAGCA---GTGGGA---ATAGGAGCT------
---TTGTTCCTTGGGTTCTTGGGAACA---GCAGGAAGCGCTATG---GGCGCAGCGTCA---
CTGACGCTGACGGTACAG---GCCAGGCAA---TTATTG---TCTGGTATAGTGCAACAGCAAAAC---
AATTTG---CTGAGG---GCTATTGAGGCGCAACAGCATCTGTTGCAACTCACAGTCTGGGGCATC
>KF726018
TTCTACTGTAATACAACACAACTGTTTAATAGTACTTGGAATAATACAAATAACAATGAG-------------
----

GGAAATGGCACAATCACACTCCAATGTAGAATAAAACAAATTATAAACATGTGGCAGGAAGTAGGAAAAGCAA
TGTATGCCCCTCCCATCAGTGGACAAATTAACTGTTCATCAAAT---
ATTACAGGACTTCTATTAACAAGGGATGGTGGTAGTACTAATGAGACG-------------------------
----

ACCACCGAGACCTTCAGACCTGGAGGGGGAAATATGAAGGACAATTGGAGAAGTGAATTATATAAATACAAAG
TAGTAAAAATTGAACCATTA---GGAGTAGCACCCACCAAGGCAAAGAGAAGAGTGGTG--------------
-------------------------CAGAGAGAAAAAAGAGCA---GTGGGAGCACTAGGAGCT---------
ATGTTCCTTGGGTTCTTGGGAGCA---GCAGGAAGCACAATG---GGCGCGGCGTCA---
CTGACGCTGACGGTACAG---GCCAGACAA---TTATTG---TCTGGTATAGTGCAACAGCAGAAC---
AATCTG---CTGCAA---GCTATTGAGGCGCAACAGCATCTGTTGCAACTCACAGTCTGGGGCATT
>KF726021
TTCTACTGTAATTCATCAGCACTGTTTAATAGTACTTGGGATGTTAATAGCACTGGATTAAATAGC-------
----

ACTGGGGGAAATATCACACTCCCATGTAGAATAAAACAAATTATAAACATGTGGCAGACAGTAGGAAAAGCAA
TGTATGCCCCTCCCATCAGCGGATAATTAACTGTACATCAAAC---
ATTACAGGACTGCTATTAACGAGAGATGGTGGTGGTAATAATAGCACC-------------------------
----

AACGAGAACGAGACCTTCAGACCTGCAGGAGGAGATATGAGGGACAATTGGAGAAGTGAATTATATAAATATA
AAGTAGTAAAAATTGAACCATTA---GGAGTAGCACCCACCAAGGCAAAGAGAAGAGTGGTG-----------
----------------------------CAGAGAGAAAAAAGAGCA---GTGGGAACACTAGGAGCT------
---ATGTTCCTTGGGTTCTTGGGAGCA---GCAGGAAGCACTATG---GGCGCAGCGTCA---
ATGACGCTGACGGTACAG---GCCAGACAA---TTATTG---ACTGGTATAGTGCAACAGCAGAAC---
AATTTG---CTGAAG---GCTATTGAGGCGCAACAGCATCTGTTGCAACTCACAGTCTGGGGCATC
>MH234643
TTCTACTGTAATTCAACAAAACTGTTTAATAGTACTTGGAATGGGAATGATACTGAAGGGTCAAATAACACTG
AA---

GGAAATGACACACTCACACTCCCATGCAGAATAAAACAAATTATAAACATGTGGCAGGAAGTAGGAAAAGCAA

TGTATGCCCCTCCCATCCAAGGACAAATTAGATGTTCATCAAAT---
ATTACAGGGCTGCTATTAACAAGAGATGGTGGTAATGGCAGCGAG----------------------------
----

ACCACCGAGATCTTCAGACCTGGAGGAGGAGATATGAGGGATAATTGGAGAAGTGAATTATATAAATATAAAG
TAGTAAAAATTGAACCATTA---GGAGTAGCACCCACCAAGGCAAAAAGAAGAGTGGTG--------------
-------------------------CAGAGAGAGAAAAGAGCA---GTGGGA---ATAGGAGCT---------
TTGTTCCTTGGGTTCTTGGGAGCA---GCAGGAAGCACTATG---GGCGCAGCGTCA---
ATGACGCTGACGGTACAG---GCCAGACAA---TTATTG---TCTGGTATAGTGCAACAGCAGAAC---
AATTTG---CTGAGG---GCTATTGAGGCGCAACAGCATCTGTTGCAACTCACAGTCTGGGGCATT
>FJ647145
TTCTACTGTAACTTAACACAACTGTTTAATAGTACTTGGAATGTAAATGGTACTGAAGGGTCAAATAAC----
----

ACTGACAAAAATATCACACTCCCATGCAGGATAAAACAAATTATAAACATGTGGCAGACAGTAGGAAAAGCAA
TGTATGCCCCTCCCATCAGAGGACAAATTAGATGTTCATCAAAT---
ATTACAGGGCTGCTATTAACAAGAGATGGTGGTAATAACGAGAAC----------------------------
----
-------------GATCCCGAAAT---
TCACCCGGAGGAGGAGATATGAGGGACAATTGGAGAAGTGAATTATATAAATATAAAGTATTAAAAATTAAAG
CATTA---GGAGTAGCACCCCCCAAGGCCAAGAGAAGAGTGGTG-----------------------------
----------CAAAGAGTAAAAAGAGCA---GTGGGA---ATAGGAGCT---------
GTGTTCTTTGGGTTCTTGGGAGCA---GCAGGAAGCACTATG---GGCGCAGCGTCA---
ATGACGCTGACGGTACAG---GCCAGACTA---TTATTG---TCTGGTATAGTGCAACAGCAAAAC---
AATTTG---CTGAAG---GCTATTGAGGCGCAACAGCACCTGTTGCAACTCACAGTCTGGGGCATC
>MH234640
TTCTACTGTAATACAACACAACTGTTTAATAGTACTTGGGATAATAGTACTTTGAGTACTGGGAATGATGATA
AT---

AACACTGGGATTATCACACTCCCATGTAAAATAAAACAAATTATAAACATGTGGCAGGAAGTAGGAAAAGCAA
TGTATGCCCCTCCCATCAGAGGACAAATTAACTGCTTATCAAAC---
ATTACAGGGCTGCTATTAACAAGAGATGGTGGTAATAACACAAACGGG-------------------------
----

ACCACCGAGATCTTCAGACCTGGAGGAGGAAATATGAGGGACAATTGGAGAAGTGAATTATATAAATATAAAG
TAGTAAAAATTGAACCATTA---GGAGTAGCACCCACCAGGGCAAAGAGAAGAGTGGTG--------------
-------------------------CAGAGAGAAAAAAGAGCA---GTGGGA---ATAGGAGCT---------
GTGTTCCTTGGGTTCTTGGGAGCA---GCAGGAAGCACTATG---GGCGCAGCGTCA---
ATAACGCTGACGGTACAG---GCCAGACTA---TTATTG---TCTGGTATAGTGCAACAGCAGAAC---
AATCTG---CTGAGG---GCTATTGAGGCGCAACAGCATCTGTTGCAACTCACAGTCTGGGGCATC
>AY505010
TTCTACTGTAATACAACACAACTGTTTAATAGTACTTGGAATAACACTGAAGAGTCAGATAACACGGAA----
----

GGAAATGGCACAATCACACTCCCATGTAGAATAAAACAAATTATAAACATGTGGCAGGAAGTAGGAAAAGCAA
TGTATGCCCCTCCCATCGCAGGACAAATTAGATGTTCATCAAAT---
ATTACAGGGCTTCTATTAACAAGAGATGGTGGTAATAGTACTAAT----------------------------
----

GGGAGTAGGGAGGTCTTCAGACCTGGAGGAGGAGATATGAGGGACAATTGGAGAAGTGAATTATATAAATACA
AAGTAGTAAAAATTGAGCCATTA---GGAGTAGCACCCACCAAGGCAAAGAGAAGAGTGGTG-----------
----------------------------CAGAGAGAAAAAAGAGCA---GCA------ATAGGAGCT------
---ATGTTCCTTGGGTTCTTGGGAGCA---GCAGGAAGCACTATG---GGCGCAGCGGCA---
GTGACGCTGACGGTACAG---GCCAGGCTA---TTATTG---TCTGGTATAGTGCAACAGCAAAAC---
AATTTG---CTGAGG---GCTATTGAGGCGCAACAGCATATGTTGCAACTCACAGTCTGGGGCATC
>KU168257
TTCTACTGTAATACATCAGGGCTGTTTAATAGTACTTGGTATAAGAATGATACTACTTGGAATGGTACAGAAA
AA---

TCAAATGGCACTATCACACTCCCATGCAGAATAAAACAAATTATAAACAGATGGCAGGAAGTAGGAAAAGCAA

TGTATGCCCCTCCTATCAGTGGATCAATTAATTGTTCATCACAT---
ATTACAGGGCTGCTATTAGTAAGAGATGGCGGCAATACCAGTAGT----------------------------
----

AATAACGAGACTTTCAGACCTGGAGGAGGAGATATGAGGGACAATTGGAGAAGTGAATTATATAAATATAAAG
TAGTAAGAATTGAGCCATTA---GGAGTAGCACCCACCAAGGCAAAGAGAAGAGTGGTG--------------
-------------------------CAGAGAGAAAAAAGAGCA---GTGGGAACGCTAGGAGCT---------
ATGTTCCTTGGGTTCTTGGGAGCA---GCAGGAAGCACTATG---GGCGCAGCATCA---
CTAACGCTGACGGTACAG---GCCAGACTA---TTATTG---TCTGGTATAGTGCAACAGCAGAAC---
AATCTG---CTAAGA---GCTATTGAGGCGCAACAGCATCTGTTGCAACTCACAGTCTGGGGCATC
>KU168258
TTCTACTGTAATACAACACAACTGTTTAATAGTACTTGGGTTAATGGTACTTGGAATGATACTGAAGCA----
----

CGAAAAGAAAATATCACACTCCCATGCAGAATAAAACAAATTATAAACATGTGGCAGGAAGTAGGAAAAGCAA
TGTATGCCCCTCCCATTGCAGGAAAAATTAGCTGTTCATCAAAT---
ATTACAGGGCTGCTATTAACAAGAGATGGTGGTATTGATAATAAC----------------------------
----

AACAAGAATGAGACCTTCAGACCTGGAGGAGGAGATATGAGGGACAATTGGAGAAGTGAATTATATAAATATA
AAGTAGTAAAAATTGAACCATTA---GGAGTAGCACCCACCAAGGCAAAGAGAAGAGTGGTG-----------
----------------------------CAGAGAGAAAAAAGAGCA---CCAGTA---CTAGGAGCT------
---TTGTTCCTTGGGTTCTTAGGAGCA---GCAGGAAGCGCTATG---GGCGCAGCGGCA---
TTAACGCTGACGGTACAG---GCCAGACTA---TTACTG---TCTGGTATAGTGCAACAGCAGAAT---
AATCTG---CTGAGG---GCTATTGAGGCGCAACAGCACCTGTTGCAACTCACAGTCTGGGGCATC
>KU168259
TTCTATTGTAATTCAACACAACTGTTTAGTAGCACTTGGAATACGACTAAAATTTGGAATGATACTGACACTA
AAGGA--
-------------AATAGC------
ACCACCAATGACACCATCATACTCCCATGTAAAATAAGACAAATTATAAACATGTGGCAGGAAGTAGGAAAAG
CAATGTATGCCCCTCCCATCGAAGGACTAATTAGTTGTTCATCAAAT---
ATTACAGGGCTGTTATTAACAAGAGATGGAGGTAATAATAGCACT----------------------------
----

AATGAGTCTGAGATCTTCAGACCTGGAGGGGGAGATATGAGGGACAATTGGAGAAGTGAATTATATAAATATA
AAGTAGTAACAATTGAACCATTA---GGAGTAGCACCCACCAAGGCAAAGAGAAGAGTGGTG-----------
----------------------------CAGAGAGAAAAAAGAGCA---GTGGGAATGTTAGGAGCC------
---ATGTTCCTTGGGTTCTTGGGAGCA---GCAGGAAGCACTATG---GGCGCAGCGTCA---
ATAACGCTGACGGTACAG---GCCAGGCAA---TTATTG---ACTGGTATAGTGCAACAGCAGAAC---
AATCTG---CTGAGG---GCTATTGAGGCGCAACAGCACATGTTGCAACTCACGGTCTGGGGCATT
>JF680931
TTCTACTGTAACACAACAGGATTGTTTAATAGTACTTGGCCAGTTAATAGTAATTGGACAGAAACTAATGGTA
CT---

AACAGGTCAGAGGTCATCACTCTCCCATGCAGGATAAAACAAATTATAAACATGTGGCAGGAAGTAGGAAAAG
CAATGTATGCTCCTCCCATCCAAGGGCCAATTAGCTGTTCCTCAAAT---
ATTACAGGGCTACTATTAACAAGAGATGGTGGTGGGGGTAACGAAACC-------------------------
----

AACACCACCACTGAGATCTTCAGACCTGGAGGAGGAGACATGAGGGACAATTGGAGAAATGAATTATATAAAT
ATAAAGTAGTACAAATTGAACCATTA---GGAGTAGCACCCACCATGGCAAAGAGAAGAGTGGTG--------
-------------------------------CAGAGAGAAAAAAGAGCA---GTGGGA---ATAGGAGCT---
------CTGTTCCTTGGGTTCTTGGGAGCA---GCAGGAAGCACTATG---GGCGCAGCGTCA---
TTGACGCTGACGGTACAG---GCCAGACAA---TTGCTG---TCTGGTATAGTGCAACAGCAGAAC---
AACTTG---CTGAGG---GCTATTGAGGCGCAACAGCACCTGTTGCAACTCACAGTCTGGGGCATT
>KC834604
TTCTACTGTAATACAACACAACTGTTTAACAGTACTTGGAATTTTTCTAATTCAACACACCTGTTTAATTGGA
CTGGGTCAAAT--

AACACTGAGCCTATCACACTCCCATGCAGAATAAAACAAATTATAAACATGTGGCAGGAAGTAGGAAAAGCAA

TGTATGCCCCGCCCATCAGAGGAGAAATTAACTGTTCATCAAAT---
ATTACAGGGGTGCTGCTAGTAAGAGATGGTGGTAAAGAAAACAGC----------------------------
----

ACTAGCGAGATCTTCAGACCTGGAGGAGGAGATATGAGGGACAATTGGAGAAGTGAATTATATAAATATAAAG
TAGTACAAATTGAACCATTA---GGAGTAGCACCCACCAAGGCAAAGCGAAGAGTGGTG--------------
-------------------------CAAAGAGAAAAAAGAGCA---GTGGGA---ATAGGAGCT---------
GTGCTCCTTGGGTTCTTGGGAGCA---GCAGGAAGCACTATG---GGCGCAGCGTCA---
CTGACGCTGACGGTACAG---GCCAGACTA---TTATTG---TCTGGTATAGCGCAACAGCAGAGC---
AATTTG---CTGAGA---GCTATTGAGGCGCAACAACATCTGTTGCAACTCACAGTCTGGGGCATT
>KC834602
TTCTACTGTAATACAACACAGCTGTTTAATAGTACTTGGCTGTCTAATGGCACTAGGAATGATACTGAAAAA-
----

TTAAATGACATTATCACACTCCCATGCAGAATAAAACAAATTGTAAACAGGTGGCAAGAAGTGGGAAAAGCAA
TGTATGCCCCTCCCATCAGCGGACAAATTAGGTGTTCATCAAAT---
ATTACAGGGTTGCTATTAACAAGAGATGGTGGTAGCAATGTCACC----------------------------
----

ACCGAGACCTTCAGACCTGAAGGAGGAAATATGAAGGACAATTGGAGAAGTGAATTATATAAATATAAAGTAG
TAAAACTTGAGCCATTA---GGAATAGCACCCACCAAGGCAAAGAGGCGAGTGGTG-----------------
----------------------CAGAGAGAAAAAAGA------GTGGCA---CTAGGAGCT---------
TTGTTCCTTGGGTTCTTGGGAGCA---GCAGGAAGCACTATG---GGCGCAGCGTCA---
ATAACGCTGACGGTACAA---GCCAGACTA---CTATTG---GCTGGTATAGAGCAACAGCAGAAC---
AATCTG---CTGAGA---GCTATTGAGGCGCAACAACATCTGTTGCAACTCACTGTCTGGGGCATC
>KT452273
TTCTACTGTAATACAACAAAACTGTTTAATAGTACTTGGCCAGTTAATAGTACTGGGAATAGTACTGAA----
----

GAAAAAGAAAATATCACACTCCCATGCAGAATAAAACAAATTATAAACATGTGGCAGGAAGTAGGAAAAGCAA
TGTATGCCCCTCCCATCAAAGGACAAATTAGATGTTCATCAAAC---
ATTACAGGGCTGCTATTAACAAGAGACGGTGGTGCTACCAACAAT----------------------------
----

ACCACCGAGATCTTCAGACCTGGAGGAGGAAATATGAAAGACAATTGGAGAAGTGAATTATATAAATATAAAG
TAGTGAAAATTGAACCATTA---GGGGTAGCACCCACCAAGGCAAAGAGGAGAGTGGTG--------------
-------------------------CAAAGAGAAAAAAGAGCA---GTGGGA---TTTGGAGCT---------
CTGTTCCTTGGGTTCTTGGGAGCA---GCAGGAAGCACTATG---GGCGCAGCGTCA---
GTGACGCTGACGGTACAA---GCCAGACAA---TTATTG---TCTGGTATAGTGCAGCAGCAAAAC---
GATTTG---CTGAGA---GCTATTGAGGCGCAACAACATCTGTTGCAACTCACAGTCTGGGGCATC
>KT452314
TTCTACTGTAATACAACAAAACTGTTTAATAGTACTTGGAGTAATAGTTCTGAACTGATTAATGGTACTGAAC
TG---
-ACGATGAATGGTACT---------
GGGGAACATGAAAATATCACCCTTCCATGCAGGATAAAACAAATTGTAAACATGTGGCAGGAAGTAGGAAAAG
CAATGTATGCCCCTCCCATTAGAGGACAAATTTTATGTACATCAAAC---
ATTACAGGGCTGATATTAACAAGAGATGGTGGAAATGTTAGTGAGAGC-------------------------
----

AATGGCACGGAGATCTTCAGACCTGGAGGAGGAGACATGAGGGACAATTGGAGAAGTGAATTATATAAATATA
AAGTAATAAGAATTGAACCATTA---GGAATAGCACCCACCAAGGCAAAGAGAAGAGTGGTG-----------
----------------------------CAGAGAGAAAAAAGAGCA---GTGGGT---TTTGGAGCT------
---ATGTTCCTTGGGTTCTTGGGAGCA---GCCGGAAGCACTATG---GGCGCAGCGTCA---
GTGACGCTGACGGTACAG---GCCAGACTA---TTATTG---TCTGGTATAGTGCAACAGCAGAAC---
AATTTG---CTGAGG---GCTATTGAGGCGCAACAGCATCTGTTGCAACTCACGGTCTGGGGCATC
>KT452341
TTCTACTGTAATACAACAAGGTTGTTTAATAGTATTTGGAATAAAACTGAGATTGACAGTAATGCTACAGAAT
CAAT---

AGCAATGACACTATCACACTCCCATGCAGAATAAAACAAATTATAAACAGATGGCAAGAAGTAGGAAAAGCAA

TGTATGCCCCTCCCATCAGTGGACAGATTAGGTGTTTATCAAAT---
ATTACAGGGCTGATATTAACAAGAGATGGAGGTAATAAAACGAAC----------------------------
----

GAGACCAACATCGAAGTCTTCAGACCTGAAGGAGGAGATATGAGACACAATTGGAGAAGTGAATTATATAAGT
ATAAAGTAGTAAGAATTGAGCCATTA---GGGATAGCACCCACCAGGGCAAAGAGAAGAGTGGTG--------
-------------------------------GGAAGAGAAAAAAGAGCG---GTCACA---CTAGGAGCT---
------ATGTTCCTTGGGTTCTTAGGAGCA---GCAGGAAGCACTATG---GGCGCAGCGTCA---
ATGACGCTGACGGTACAG---GCCAGACAA---TTATTG---TCTGGTATAGTGCAGCAGCAAAAT---
AATTTG---CTGAGA---GCTATTGAGGCGCAACAGCATATGTTGCAACTCACAGTCTGGGGCATC
>KT452551
TTTTACTGTAATTCAACACCATTGTTTAATAGTATTTGGTATGATAATGGAACAAGCATCGCTACAGGAAATG
GGACA--

GTAAATAATACTATCATACTCCAATGCAGAATAAAACAAATTATAAACATGTGGCAAAAAGTAGGAAAAGCAA
TGTATGCTCCTCCCATTAAAGGACTAATTAAATGTTCATCAAAT---
ATTACGGGACTGCTATTAACAAGAGATGGTGGTAAAAACCAAACC----------------------------
----

AATGACAGTGAGACCTTCAGGCCGGGAGGAGGAGATATGAGGGACAATTGGAGAAGTGAACTATACAAATATA
AAGTAGTAAAAATTGAACCATTA---GGAGTAGCACCCACCAAGGCAAAAAGAAGAGTGGTG-----------
----------------------------CAGAGAGAAAAAAGAGCA---ATAACA---CTGGGAGCT------
---ATGTTCCTTGGGTTCTTGGGAGCA---GCAGGAAGCACTATG---GGCGCAGCGTCA---
ATGACGCTGACGGTACAG---GCCAGACAA---TTATTG---TCTGGTATAGTGCAACAGCAAAAC---
AATTTG---CTGAGA---GCTATTGAGGCGCAACAGCATCTGTTGCAACTCACAGTCTGGGGCATC
>KT452612
TTCTACTGTAATACAACACCGCTGTTTAATAGTACTTGGAAGTGGAATAATGGTACTGGAGGGTCAAGTATCA
CTGAAGGAAAT--

CTCACAGAACCTATCACACTCCAATGCAAAATAAAACAAATTATAAACATGTGGCAGGAAGTAGGAAAAGCAA
TGTATGCCCCCCCCATCCAGGGACAAATTAACTGTTCCTCAAAT---
ATTACAGGGCTGATATTGACAAGAGATGGAGGTGACGATAATACG----------------------------
----

CAAAATGACACCTTCAGACCTGCAGGAGGAGATATGAGGGACAATTGGAGAAGTGAATTATATAAATATAAAG
TAGTAAGAATTGAACCATTA---GGAATAGCACCTTCCAGGGCAAAGAGAAGAGTGGTG--------------
-------------------------CAGAGAGAAAAAAGAGCA---GTGGGA---TTTGGAGCT---------
TTGTTCCTTGGGTTCTTGGGAGCA---GCAGGAAGCACTATG---GGCGCAGCGTCA---
GTGGCGCTGACGGTACAG---GCCAGATCA---TTATTG---TCTGGTATAATGCAGCAGCAAAAC---
AACTTG---CTGAGG---GCTGTTGAGGCGCAACAGCATCTGTTGCAACTCACAGTCTGGGGCATC
>KT452359
TTCTACTGTAATTCAACACAACTGTTTAATAGTACTTGGAATACTACTGAAGGGTTAAATAGCACTGAAGGA-
----

GATGACACAACAATCACACTCCCATGCAGAATAAAACAAATTATAAACATGTGGCACAAAGTAGGCAGAGCAA
TGTATGCCCCTCCCATCCAAGGACAAATTAGATGTTCATCAAAT---
ATTACAGGGCTGCTATTAACAAGAGATGGTGGTAAGAATGTGAGC----------------------------
----

AATATCAGCGAGACCTTCAGACCTGGAGGAGGAAATATGAAGGACAATTGGAGAAGTGAATTATATAAATATA
AAGTAGTAAGAATTGAACCTTTA---GGAATAGCACCCACCAAGGCAAAGAGAAGAGTGGTG-----------
----------------------------CAAAGAGAGAAAAGAGCA---GTGGGA---ATGGGAGCT------
---CTGTTCCTTGGGTTCTTGGGAACA---GCAGGAAGCACTATG---GGCGCAGCGTCA---
TTGACGCTGACGGTACAG---GCCAGACAA---TTATTG---TCTGGTATAGTGCAGCAGCAGAAT---
GATATG---CTGAGA---GCTATTGAGGCGCAACAGCACCTGTTGCAACTCACAGTCTGGGGCATC
>KT452109
TTCTACTGTAATACAACACAACTGTTCACTAGTACTTGGAAAAATTCTAGTGATGGGAACGTTACAAGAAGT-
----

GAAAATGACACAATCACACTCCCATGCAGAATAAAACAAATTATAAACATGTGGCAGGAAGTAGGAAAAGCAA

TGTATGCCCCTCCCATCAGAGGACACATTAACTGTTCATCAAAT---
ATTACAGGGCTGCTATTAACAAGAGATGGTGGGGGCAACGAGAGCAGA-------------------------
----

GAAAATGAAACCTTCAGACCTGGAGGGGGAGATATGAAGGATAATTGGAGAAGTGAACTATATAAATATAAAG
TAGTAAAAATTGAACCATTA---GGAATAGCACCCACCGAGGCACAGAGAAGAGTGGTG--------------
-------------------------CAGAGAGAAAAAAGAGCA---GTGGGA---CTAGGAGCT---------
ATGCTCTTTGGGTTCTTGGGAGCA---GCAGGAAGCACTATG---GGCGCAGCGTCA---
TTGACGTTGACGGTACAA---GCCAAACTA---TTATTG---TCTGGTATAGTGCAACAGCAGAAT---
AATTTG---CTGAGA---GCTATTGAGGCGCAACAGCATATGTTGCAACTCACAGTCTGGGGCATC
>KT452090
TTCTACTGTAACTCATCACAATTGTTTAATAGTACTTGGAATCTTAATGATACTTGGAATGGTACTAGTGAC-
----

ACCAATGAAAATATTACACTCCCATGCAGAATAAAACAAATCATAAACCTGTGGCAGAAAGTAGGAAAAGCAA
TGTATGCCCCTCCCATCAGTGGATGGATTAACTGTTCATCAAAT---
ATTACAGGGCTGCTATTAACAAGAGATGGTGGTGGTAATAATGAG----------------------------
----

AATACTAGTGAGACCTTCAGACCTGGAGGAGGAAATATGAAGGACAATTGGAGAAGTGAATTGTACAAATACA
AAGTAGTAAAAATTGAACCATTA---GGAGTAGCACCCACCAGGGCAAAGAGAAGAGTGGTG-----------
----------------------------CAGAGAGAAAAAAGAGCA---GTGGGAACACTGGGAGCT------
---ATATTCCTTGGGTTCTTGGGAACA---GCAGGAAGCACTATG---GGCGCAGCGTCA---
GTGACGCTGACGGTACAA---GCCAGACAA---TTATTG---TCTGGTATAGTGCAGCAGCAAAAC---
AATTTG---CTGAGG---GCTATTGAGGCGCAGCAGCATCTGTTGCAACTCACAGTCTGGGGCATC
>KT452407
TTCTACTGTGACACAACACAACTGTTTAATAGCACTTGGCATCCTAATGGCACCAATAGTACTTGGCCTACTA
ATAGTACT---
-------------AATGGA------
AGAGAAAATGATACAATCATACTCCCATGCAGAATAAAACAAATTATAAACATGTGGCAGGAAGTAGGAAAAG
CAATGTATGCCCCTCCCATCAGTGGAGTAATTAAATGTTCATCAAAT---
ATTACAGGGCTGTTATTAACAAGAGATGGTGGTAGGGATAATGGCACG-------------------------
----

AATGGTACCGAGACCTTCAGACCTGGAGGGGGAGATATGAGGGACAATTGGAGAAGTGAATTATACAAATATA
AAGTAGTAAGAATTGAACCATTA---GGAATAGCACCCACTAAGGCAAAGAGAAGAGTGGTG-----------
----------------------------CCGAGAGAAAAAAGAGCA---ATCGGA---ATAGGAGCT------
---ATGTTCATTGGGTTCTTGGGAGCA---GCAGGAAGCACTATG---GGCGCAGCGTCA---
GTGACGCTGACGGTACAG---GCCAGACAA---TTAATG---TCTGAGATAGTGCAACAGCAAAAC---
AATTTG---CTGAGA---GCTATTGAGGCGCAACAGCATATGTTGCAACTCACAGTCTGGGGCATC
>KT452194
TTCTACTGTGATACAACAAGTCTGTTTAATAGTAATTGGACGAAAGATAATAATGAGTCTTGGAGTAATACTA
CTGACTGG---

TCAAATGGAACTATCACACTCCAATGCAGAATAAAACAAATTATAAACCTGTGGCAGCAAGTAGGAAAGGCAA
TGTATGCCCCTCCCATCAGCGGAAACATTAGCTGTTCATCAAAT---
ATTACAGGGGTGATATTAACAAGAGATGGTGGGGGGGGTAATAACACGGAA----------------------
----

AACAATACTGAGATCTTCAGACCAGGAGGAGGAGATATGAAGGACAATTGGAGAAGTGAATTATATAAATATA
AAGTAGTACAAATTGAGCCACTA---GGAATAGCACCCACCAGGGCAAAGAGAAGAGTGGTG-----------
----------------------------CAGAGAGAAAAAAGAGCA---GTTGGC---ATAGGAGCG------
---GTGTTCCTTGGGTTCTTGTCTGCA---GCAGGAAGCACTATG---GGCGCAGCGGCA---
GTAACGCTGACGGTACAG---GCCAGACAA---TTATTG---TCTGGTATAGTGCAACAGCAGACC---
GCTCTG---CTGAGG---GCTATTGAGGCGCAACAGCATATGTTGCAACTCACAGTCTGGGGCATC
>KT452218
TTCTACTGTAATTCAACACAACTGTTTAATAGTACTTGGAATATTACTGGAGAACCAAATAGCACTGCA----
----

GGAAGTGACACAATCATACTGCCATGCAGAATAAAACAAATTATAAACATGTGGCAAGAAGTAGGAAAGGCAA

CGTATGCCCCTCCCATTAAAGGACAAATTAGATGCTCATCAAAT---
ATTACAGGGCTGCTATTAACAAGAGACGGTGGTACTAACAGTAGC----------------------------
----

AACAGTAGTGAGATCTTCAGACCTGGAGGAGGAAATATGAAGGACAATTGGAGAAGTGAACTATATAAATATA
AAGTAGTAAAAATTGAACCATTA---GGAATAGCACCCACCAAGGCAAAGAGAAGAGTGGTG-----------
----------------------------CAGAGAGAAAAAAGAGCA---GTGGGAGTAATAGGAGCT------
---ATGTTCCTTGGGTTCTTGGGAGCA---GCAGGAAGCACTATG---GGCGCAGCGTCA---
GTGACGCTGACGGTACAG---GCTAGACAA---TTATTG---TCTAGTATAGTGCAACAGCAGAAC---
GATTTG---CTGAGA---GCTATTGAGGCGCAACAGCATCTGTTGCAGCTCACAGTCTGGGGCATC
>KT452245
TTCTACTGTAATACATCACAACTGTTTAATAGTACTTGGAATGTTACTGAAGGGACAACTAACACTGAAGGGT
CAAATAACACT--

GGAGGAAGTGACCCTATCACACTCCCCTGCAGAATAAAACAAATTATAAACCTATGGCAGGAAGTAGGAAAAG
CAATGTATGCCCCTCCCATCAGAGGACAACTTAGTTGCTCATCAAAT---
ATTACAGGGCTGCTATTAACAAGAGATGGTGGTAATAACAGCGGA----------------------------
----

AGCGAGGTCGAGACCTTTAGACCAGGAGGAGGAGATATGAGGGACAATTGGAGAAATGAATTATATAAATATA
AAGTAGTAAAAGTTGAACCATTA---GGAATAGCACCCACCAGGGCAAAGAGAAGAGTGGTG-----------
----------------------------CAGAGAGAAAAAAGAGCG---GTAACG---CTAGGAGCT------
---GTGTTCCTTGGGTTCTTGGGAGCA---GCAGGAAGCACTATG---GGCGCAGCATCA---
GTAACGCTGACGGTACAG---GCCAGACAA---TTATTG---TCTGGTATAGTGCAACAGCAGAAC---
AATCTG---CTGAGG---GCTATTGAGGCGCAACAACATATGTTGCAACTCACAGTCTGGGGCATT
>MN486011
TTCTATTGCAATACATCACGCTTGTTTAATAGTACACATAATGTTACAGAAAATGCATATAATATTACAGGAA
ATGCT--

TCAGATTCAACCATCATCCTCCCATGCAGAATAAAACAAATTATAAATATGTGGCAGGAGGTAGGACGAGCAA
TGTATGCCCCTCCCATTGCAGGAAACATAACATGCCACTCAAAT---
ATCACAGGACTATTATTGACACGTGATGGAGGTGAAAATAACAGT----------------------------
----

ACATCAGAAACATTTAGACCTGCAGGAGGAGATATGAGGGACAATTGGAGAAGTGAATTATATAAATATAAAG
TGGTAGAAATTAAACCATTA---GGAATAGCACCCACTGGCGCAAAAAGGAGAGTGGTG--------------
-------------------------GAGAGAGAAAAAAGAGCA---GTGGGA---ATAGGAGCT---------
GTGCTCCTTGGGTTCTTGGGAGCA---GCAGGAAGCACTATG---GGCGCAGCGTCA---
ATAACGCTGACGGTACAG---GCCAGACAA---CTGTTG---TCTGGTATAGTGCAACAGCAAAGC---
AATTTG---CTGAGA---GCTATAGAGGCGCAACAGCATATGTTGCAACTCACGGTCTGGGGCATT
>HQ912708
TTCTATTGCAACACATCAAAACTGTTTAATGAGACAGAACTGCTTAAAAGT----------------------
----

ACAGACAGTACTTTAACCATCCCATGCAGAATAAAACAAATTATAAACATGTGGCAGGGGGTAGGACGAGCAA
TATATGCCCCTCCCATTAAAGGAAAAATAACATGTAAATCAAAT---
ATTACAGGACTACTACTAACACGTGATGGAGGAGTAAATAACGAT----------------------------
----

AGCACAGAAACATTCAGACCTGTAGGAGGAAACATGAAGGACAATTGGAGAAGTGAATTATATAAATATAAAG
TGGTAGAAATTAAACCATTA---GGAGTAGCACCCACTAAGGCAAAAAGGAGAGTGGTG--------------
-------------------------GAGAGAGAAAAAAGAGCA---GTGGGA---ATAGGAGCT---------
GTGTTCCTTGGGTTCTTGGGAGCA---GCAGGAAGCACTATG---GGCGCAGCATCA---
ATAACGCTGACGGTACAG---GCCAGACAA---TTATTG---TCGGGCATAGTGCAACAGCAAAGC---
AATTTG---CTGAGG---GCCATAGAAGCGCAACAGCATATGTTGCAACTCACGGTCTGGGGCATT
>EU191613
TTCTATTGCAATACATCAGGATTGTTTAATGGTACATACATGAATAGTACATACATGAATAGT----------
----

ACAGATAAAATCATCAGACTCCCATGCAGAATAAAACAAATTATAAACATGTGGCAGGAAGTAGGACGAGCAA

TGTATGCCTCTCCCATTAAAGGACAAATAAAATGTGAATCAAAT---
ATCACAGGATTATTATTGACACGTGATGGAGGAAATGAAAATAAT----------------------------
----

GTGACAGAGATCTTCAGACCTGGAGGAGGAAATATGAAGGACAATTGGAGAAGTGAATTATATAAATATAAAG
TGGTGGAAATTAAACCATTA---GGAATAGCACCCACTACAGCAAAAAGGAGAGTGGTG--------------
-------------------------GAGAGAGAAAAAAGAGCA---GCGGGA---CTAGGAGCT---------
GTGTTCTTTGGGTTCTTGGGAGCA---GCAGGAAGCACTATG---GGCGCAGCATCA---
ATAACGCTGACGGTACAG---GCCAGACAA---TTATTG---TCTGGTATAGTGCAACAGCAAAGC---
AATTTG---CTGAGA---GCCATAGAGGCGCAACAGCACATGTTGCAACTCACGGTCTGGGGCATT
>MH746230
TTTTATTGTAACACATCAAATCTGTTTAATAGTACACACAGGGCAGATGATACAAATTCAACCATCACAAATG
ATACAGAT---

TCAAATAGGACCATCACACTCCAATGCAGAATAAAACAAATCATAAACATGTGGCAGAGGGTAGGACGAGCAA
TGTATGCCCCTCCCATTAAAGGAAACATAACATGTATATCAAAT---
ATCACAGGACTACTACTAACAAGGGATGGAGGAGTAAAGAATGAAACAGGGAAG------
AATGAAACAGGGAAGGACTCAGAT---GGG---

AACAACACAGAGATATTCAGACCTGGAGGAGGAGATATGAGGGACAATTGGAGAAGTGAATTATATAAATATA
AAGTGGTAGAAATTAAGCCATTA---GGAGTAGCACCCACTGAGGCAAAAAGGAGAGTGGTG-----------
----------------------------GAGAGAGAAAAAAGAGCA---GTGGGA---ATAGGAGCT------
---ATGTTCCTTGGGTTCTTGGGAGCA---GCAGGAAGCACTATG---GGCGCAGCATCA---
ATAACGCTGACGGTACAG---GCCAGACAA---TTGTTG---TCTGGTATAGTGCAACAGCAAAGC---
AATTTG---CTGAAA---GCTATAGAGGCGCAACAGCATATGTTGCAACTCACGGTCTGGGGCATT
>MH746248
TTCTATTGCAATACATCAAGCCTGTTTAATAGTAATAGTACAAGCCTAGGTAATAGTAGTAATAGTACAGAAA
CA---

GAAAATGCAAACATCACACTCCCATGCAGAATAAAACAAATCATAAACATGTGGCAGGGGGTAGGACAAGCAA
TGTATGCTCCTCCCATTAAAGGAATAATAACATGTAGATCAAAT---
ATCACAGGACTACTACTAACACGTGATGGAGGAGACAACAACAAA----------------------------
----

ACAGAGACATTCAGGCCTGGAGGAGGAGATATGAGGGACAATTGGAGAAGTGAATTATATAAATATAAAGTGG
TAGAAATTAAACCATTA---GGAATAGCACCCACTAAGGCAAAAAGGAGAGTGGTG-----------------
----------------------GAGAGAGAAAAAAGAGCA---GTGGGA---ATAGGAGCT---------
GTGTTCCTTGGGTTCTTGGGAGCA---GCAGGAAGCACTATG---GGCGCAGCATCA---
ATAACGCTGACGGTACAG---GCCAGACAA---TTATTG---TCTGGGATAGTGCAACAGCAAAGC---
AATTTG---CTGAGG---GCTATAGAGGCGCAACAGCATATGTTGCAACTCACGGTCTGGGGCATT
>U39233
TTCTATTGTAATACATCAAAACTGTTTAATAGTACAGACAGGTCTAGCAGTACA-------------------
----

GAAAGTGCAAACATAACCATCCCATGCAGAATAAAACAAATTATAAACATGTGGCAGGGGGTAGGACGAGCAA
TGTATGCCCCTCCCATTAAAGGAAAAATAACATGTAAATCAAAT---
ATCACAGGACTACTACTGACACGTGATGGAGGAACAACAAATGAT----------------------------
----

AGCACAGAGGCGTTCAGGCCTGCAGGAGGAGATATGAAGGACAATTGGAGAAGTGAATTATATAAGTATAAAG
TAGTAGAAATTAAACCATTG---GGAGTAGCACCCACCAAGGCAAAGAGGAGAGTGGTG--------------
-------------------------GAGAGAGAAAAAAGAGCA---GTGGGA---ATAGGAGCT---------
GTGTTCCTTAGGTTCTTGGGAGCA---GCAGGAAGCACTATG---GGCGCAGCATCG---
ATAACGCTGACGGTACAA---GCCAGACAA---TTATTG---TCGGGTATAGTGCAACAGCAAAGC---
AATTTG---CTGAGG---GCTATAGAGACGCAACAGCATATGTTGCAACTCACGGTCTGGGGCATT
>U39237
TTCTATTGCAATACATCAGGCCTGTTTAATAGTACATACAATAGTAATAGTACAGACAATGCTAATAGCACA-
----

GACAATTCAACCATCACGCTTCCATGTAGAATAAAACAAATTATAAACATGTGGCAGGGGGTAGGACAAGCAA

TATATGCCCCTCCCATTAGAGGAAACATAACATGTAAATCAAAT---
ATCACAGGAATATTACTGACACGTGATGGAGGATCAGATGCAAAT----------------------------
----

GAAACAGAGACATTCAGGCCTGGAGGAGGAGATATGAGGGACAATTGGAGAAGTGAATTATATAAATATAAAG
TGGTAGAAGTTAAACCATTA---GGATTAGCACCCACTAAGGCAAAAAGAAGAGTGGTG--------------
--------------------------GAGAGAGAAAAAAGAGCA---GTGGGA---CTAGGAGCT---------
GTGTTCCTTGGGTTCTTGGGAGCA---GCAGGAAGCACTATG---GGCGCAGCGTCA---
ATAACGCTGATGGTACAG---GCCAGACAA---TTGTTG---TCCGGTATAGTGCAACAGCAAAGC---
AATTTG---CTGAGG---GCTATAGAGGCGCAACAGCATATGTTGCAACTCACGGTCTGGGGCATT
>U39239
TTCTATTGCAATACATCAGGACTGTTTAATAGTACATACTGGCCTAACAGTACAAGAGAGGAGATTACAACA-
----

GACAATTCAAACATCACACTCCAATGCAGAATAAAACAAATTGTAAACATGTGGCAGGGGGTAGGACGAGCAA
CATATGCCCCTCCCATTGCAGGAAAAATAACATGTAACTCAAAC---
ATCACAGGACTGTTACTGACACGTGATGGAGGGATAAATGGGACA----------------------------
----

AATAGCACAGAGACATTCAGGCCTGGAGGAGGAAATATGAAGGACAATTGGAGAAGTGAATTATATAAATATA
AAGTGGTAGAAGTTAATCCATTA---GGAGTAGCACCCACTGGGGCGAAAAGGAGAGTGGTG-----------
-----------------------------GAGAGAGAAAAAAGAGCA---GTGGGA---ATAGGAGCT------
---GTGTTCCTTGGGTTCTTGGGAGCA---GCAGGAAGCACTATG---GGCGCAGCGTCA---
ATAACGCTGACGGTACAG---GCCAGACAA---TTATTG---TCAGGTATAGTGCAACAGCAAAGC---
AATTTG---CTGAGG---GCTATCGAGGCGCAACAGCATATGTTGCAACTCACGGTCTGGGGCATT
>U39246
TTCTATTGTAATACATCAAAACTGTTTAATGGTGCATACAATAATGGTACATACAATAGTACAGAGAATATA-
----

GAAAATTCAACTATCACACTCCCATGCAGAATAAAACAAATTATAAACATGTGGCAGGGGGTAGGGCAAGCAA
TGTATGCCCCTCCCATCAAAGGAAACATAACATGTAAATCAAAT---
ATCACAGGACTACTACTGACACGTGATGGAGGAAGCAGCGAGGCAAAT-------------------------
----

AAAACAGGGGAGACATTCAGGCCTGGAGGAGGAGATATGAAGGACAATTGGAGAAGTGAATTATATAAATATA
AAGTGGTAGAAATTAAACCATTA---GGAATAGCACCCACTAAGGCAAAAAGGAGAGTGGTG-----------
-----------------------------GAGAGAGAAAAAAGAGCA---GCA------ATAGGAGCT------
---ATGTTCCTTGGGTTCTTGGGAGCA---GCTGGAAGCACTATG---GGCGCAGCATCA---
ATAACGCTGACGGTACAG---GCCAGACAA---TTATTG---TCGGGCATAGTGCAACAGCAAAGC---
AATTTG---CTGAGG---GCTATAGAGGCGCAACAGCATATGTTGCAACTCACGGTCTGGGGCATT
>U39245
TTCTATTGCAATACATCAAAATTGTTTAAGACATCAGTTAAC-------------------------------
----

CCAAACATCACACTCACACTCCCATGCAGAATAAAACAAATTATAAACATGTGGCAGGAGGTAGGGCGAGCAA
TGTATGCCCCTCCCATTTCAGGAATAATAACATGTAAATCAAAT---
ATCACAGGACTACTACTAACACGTGATGGAGGGGGGCAGAATAAT----------------------------
----

GGACCAGAGACATTCAGACCTGGAGGAGGAAATATGAGAGACAATTGGAGAAGTGAATTATATAAATATAAAG
TGGTAGAAATTAAGCCATTA---GGAATAGCACCCTCTAAGGCAAAAAGGAGAGTGGTG--------------
--------------------------GAGAGAGAAAAAAGAGCA---GTGGGA---ATAGGAGCT---------
GTGTTCCTTGGGTTCTTGGGAGCA---GCAGGAAGCACTATG---GGCGCAGCATCA---
ATAACGCTGACGGTACAG---GCCAGACAA---TTATTG---TCTGGTATAGTGCAACAGCAAAGC---
AATTTG---CTGAGA---GCTATAGAGGCGCAACAGCACATGTTGCAACTCACAGTCTGGGGCATT
>U39251
TTCTATTGCAATACATCACAACTGTTTAATAGTACATACTGGTTTAATAGTACACACTGGTTTAACAGTACAG
AAAGCGCT---
-------------AATGGA------
ACAGAAAATGACACTATCACACTCCCATGCAGAATAAAACAAATTATAAACATGTGGCAGGGGGTAGGACAAG

CAATGTATGCCCCTCCCATTAAAGGAAACATAACATGTAAATCAAAT---
ATCACAGGAATACTCCTAACACGTGATGGAGGAAATTTAACTAAT----------------------------
----

GGCACAGAGACATTCAGGCCTGGAGGAGGAGATATGAGGGACAATTGGAGAAATGAATTATATAAATATAAAG
TGGTAGAAATTAAACCATTA---GGAGTAGCACCCACTGCGGCAAAAAGGAGAGTGGTG--------------
-------------------------GAGAGAGAAAAAAGAGCA---GCA------CTAGGAGCT---------
GTGTTCCTTGGGTTCTTGGGAGCA---GCAGGAAGCACTATG---GGCGCAGCGTCA---
ATAACGCTGACGGTACAG---GCCAGACAA---TTATTG---TCCGGCATAGTGCAACAGCAAAGC---
AATTTG---CTGAGG---GCCATAGAGGCGCAACAGCATATGTTGCAACTCACGGTCTGGGGCATT
>JN692434
TTCTATTGCAATACAGCAAGCCTGTTTAATAGTACATACAATAGTACAGGAAACATTACTGAGACAGAAAAGT
CA---

AACAGCACAGTCATCACAATCCCATGCAGAATAAAACAAATTATAAACATGTGGCAGGAGGTAGGACGAGCAA
TGTATGCCTCCCCTATAAAAGGAGAAATAACATGTAACTCAAGT---
ATCACAGGATTACTACTGACACGGGATGGAGGCCTGAATAGAACA----------------------------
----

AATGAGACATTCAGGCCTGGAGGAGGAGATATGAGAGACAATTGGAGAAGTGAATTATATAAATATAAAGTGG
TAGAAATTAAACCATTA---GGAATAGCACCCACTAGAGCAAAAAGGAGAGTGGTG-----------------
----------------------GGGAGAGAAAAAAGAGCA---GTGGGA---ATAGGAGCT---------
GTGTTCCTTGGGTTCTTGGGAGCA---GCAGGAAGCACTATG---GGCGCAGCATCA---
ATAACGCTGACGGTACAG---GCCAGACAA---TTGTTG---TCTGGGATAGTGCAACAGCAAAGC---
AATTTG---CTGAGA---GCTATAGAGGCGCAACAGCATATGTTGCAACTCACGGTCTGGGGCATT
>AY727522
TTCTATTGCAATACATCAGGCTTGTTTAATGGTACATACAATGGTACAGAAAGCAATAATGAAACAGAAAAT-
----

GCAAACGCAAACATCACACTCCCATGCAAAATAAAACAAATTGTACGCATGTGGCAGGAAGTAGGACAAGCAA
TGTATGCCCCTCCCGTTAAGGGAATGATAACATGTAGATCAAAT---
ATCACAGGATTACTATTGACACGGGATGGAGGCTCTAACACCACG----------------------------
----

AATAACACAGAAACATTCAGGCCTGGAGGAGGAGATATGAGGGACAATTGGAGAAGTGAACTATATAAATATA
AAGTGGTAGAAATTAAACCATTA---GGAATAGCACCCACTGAGGCAAAAAGGAGAGTGGTG-----------
----------------------------CAGAGAGAAAAAAGAGCA---GTGGGA---ATAGGAGCT------
---GTGTTCCTTGGGTTCTTGGGAGCA---GCAGGAAGCACTATG---GGCGCAGCATCA---
ATAACGCTGACGGTACAG---GCCAGACAA---TTATTG---TCTGGAATAGTGCAACAGCAAAGC---
AATTTG---CTGAGG---GCTATAGAGGCGCAACAGCATATGTTGCAACTCACGGTCTGGGGCATT
>AY727523
TTCTATTGTAATACAACAGGCCTGTTTAATGGTACATACATAAAAAATTCTAATGCAACAGGAAAC-------
----

ATTACTAGAAACATCACAATCCCATGCAGAATAAAACAAATTATAAACATGTGGCAGAAGGTAGGACGAGCAA
TGTATGCCCCTCCCATTCGAGGAATAATAACCTGTAGGTCAAAT---
ATCACAGGACTACTACTGACACGTGATGGAGGAGAGAATGATACA----------------------------
----

GAGACAGTCAGGCCTGGAGGAGGAGATATGAGGGACAATTGGAGAAGTGAATTATATAAATATAAAGTGGTAG
AAATTAAACCATTA---GGAATAGCACCCACTAAGGCAAAAAGGAGAGTGGTG--------------------
-------------------GAGAGAGAAAAAAGAGCA---GTGGGA---ATAGGAGCT---------
GTGTTCCTTGGGTTCTTGGGAGCA---GCAGGAAGCACTATG---GGCGCAGCATCA---
ATAACGCTGACGGTACAG---GCCAGACAA---TTGTTG---TCTGGGATAGTGCAACAGCAAAGC---
AATTTG---CTGAGG---GCTATAGAGGCGCAACAGCATATGTTGCAACTCACAGTCTGGGGCATT
>AY727524
TTCTATTGCAATACATCAAGCCTGTTCAATGGTACATACAATGGTACATACAATAGTACAGGAAACATTAATG
GA---

ACAGACACAAACATCACAATACCATGCAGAATAAAACAATTTATAAACATGTGGCAGGAGGTAGGACGAGCAA

TGTATGCCCCTCCCATTAGAGGAATAATAATGTGTAGATCAAAT---
ATCACAGGACTACTACTGACACGTGATGGGGGAATTAGCAGAGATAACAAC----------------------
----

ACAGATAACACCACAGAGATATTCAGACCTGGAGGAGGAGATATGAGGGACAATTGGAGAAGTGAATTATATA
AATATAAAGTGGTAGAAATTAAACCATTA---GGACTAGCACCCACTAAGGCAAAAAGGAGAGTGGTG-----
-----------------------------------GAGAGAGAAAAAAGAGCA---GCA------
CTAGGAGCT---------ATGTTCCTTGGGTTCTTGGGAGCA---GCCGGAAGCACTATG---
GGCGCAGCATCA---ATAACGCTGACGGTACAG---GCCAGACAA---TTATTG---
TCTGGGATAGTGCAACAGCAAAGC---AATTTG---CTGAGG---
GCTATAGAGGCGCAACAGCATATGTTGCAACTCACGGTCTGGGGCATT
>AY727525
TTCTATTGCAATACAGCAAACCTGTTTAATGGTACATACAATGATACAGGAAACATTAAT-------------
----

GAAACAGAAACCATTACAATCCCATGCAGAATAAAACAAATTATAAACATGTGGCAGAAGGTAGGACGAGCAA
TGTATACCCCTCCCATTAACGGAATAATAACATGTAGATCAAAT---
ATCACAGGGTTACTACTGACACGGGATGGAGGCAATAATAATGGC----------------------------
----

ACGAATGAGACATTCAGGCCTGAAGGAGGAGATATGAAGGACAATTGGAGAAGTGAATTATATAAATATAAAG
TGGTGGAAATTAAACCATTA---GGAATAGCACCCACTAAGGCAAAAAGGAGAGTGGTG--------------
--------------------------GAGAGAGAAAAAAGAGCA---GCAGGA---ATAGGAGCT---------
GTGTTCCTTGGGTTCTTGGGAGCA---GCCGGAAGCACTATG---GGCGCAGCATCA---
ATAACGCTGACGGTACAG---GCCAGACAA---TTATTG---TCTGGTATAGTGCAACAGCAAAGT---
AATTTG---CTGAGG---GCTATAGAGGCGCAACAGCATATGTTGCAACTCACGGTTTGGGGCATT
>JX140663
TTCTATTGCAATACATCAAGCCTGTTTAATAGTGAATTAGACAGTAATGGTACATTCAAAATTAATGGGACAG
AAAATGGAACT---
-------------------GGA---
ACAGAAAATTCAAACATCACACTCCAATGCAGAATAAAACAAATTATAAACATGTGGCAGGAGGTAGGACGAG
CAATGTATGCCCCTCCCATTGCAGGAGAAATAACATGTAGATCAAAT---
ATCACAGGATTACTACTAACAAGGGATGGAGGAGACACGAGTGAC----------------------------
----

GAGATATTCAGGCCTGGAGGAGGAGATATGAGGGACAATTGGAGAAGTGAATTATATAAATATAAAGTGGTAG
AAGTTAAACCATTA---GGAGTAGCACCCACCGAGGCAAAAAGGAGAGTGGTG--------------------
-------------------GAGAGAGAAAAAAGAGCA---GTGGGA---ATAGGAGCT---------
GTGTTCCTTGGGATCTTGGGAGCA---GCAGGAAGCACTATG---GGCGCAGCATCA---
ATGACGCTGACGGTACAG---GCCAGACAA---TTATTG---TCTGGGATAGTGCAACAGCAAAGT---
AATTTG---CTGAGG---GCTATAGAGGCGCAACAGCATATGTTGCAACTCACGGTCTGGGGCATT
>KX181929
TTCTACTGCAATACATCAGGCCTGTTTAATAGTACATACTGGCCTAATGCTACATATAATGCAACAGAAAATT
CA--

GAAAATTCAACCATCACAATCCCATGCAGAATAAAACAATTTGTAAACATGTGGCAGGAGGTAGGACGAGCAA
TGTATGCCCCTCCCATTAGAGGACCAATAAACTGTACATCAAAT---
ATAACAGGACTATTGATAACACGTGATGGAGGAATCAACTCAAGT----------------------------
----

GACACAGAGATATTCAGACCTGGAGGAGGAGATATGAGGGACAATTGGAGAAGTGAATTATATAAATACAAAG
TGGTAGAAATTAAACCATTA---GGAATAGCACCCACCGAGGCAAAAAGGAGAGTGGTG--------------
--------------------------GAGAGAGAAAAAAGAGCA---GTGGGA---ATAGGAGCT---------
GTGTTCCTTGGGTTCTTGGGAGCC---GCAGGAAGCATTATG---GGCGCAGCGTCA---
CTAACGCTGACGGTACAG---GCCCGGCAA---TTATTG---TTTGGGATAGTGCAACAGCAAACC---
AATTTG---TTGAGG---GCTATAGAGGCGCAACAGCATATGTTGCAACTCACAGTCTGGGGCATT
>KX181930
TTCTATTGTAATACATCAAGCCTGTTTAATAGGAATATTACAGAAAAAATTACTGAT----------------
----

AATAATTCAAGCATCACTATCACATGCAGAATAAAACAAATTGTAAACATGTGGCAGGGGGTAGGACGAGCAA
TATATGCCCCTCCCATTGAAGGAGTAATAACTTGTAGATCAAAT---
ATCACAGGATTGCTACTGACACGGGATGGAGGCAACACAAATAAC----------------------------
----

ACAACAGAGACATTCAGGCCTGGAGGAGGAGATATGAGGGACAATTGGAGAAGTGAATTATATAAATATAAAG
TGGTAGAAATTAAACCATTA---GGAATAGCACCCACTAAGGCAAAAAGGAGAGTGGTG--------------
-------------------------GAGAGAGAAAAAAGAGCA---GTGGGA---ATAGGAGCT----------
GTGTTCCTTGGGTTCTTGGGAGCA---GCAGGAAGCACTATG---GGCGCAGCATCA---
ATAACGCTGACGGTACAG---GCCAGACAA---TTGTTG---TCTGGGATAGTGCAACAGCAAAGC---
AATTTG---CTGAGA---GCTATAGAGGCGCAACAGCATATGTTGCAACTCACGGTCTGGGGCATT
>KX181931
TTTTATTGCAATACATCAAACCTGTTTAATAATATATACCCGAAATGGAACATGTTTGACTGGAGCAATGCAA
ACAAA--
-------------------ACA---
AACACTACAACAAACATCACACTCCGATGCAGAATAAGACAATTTATAAACATGTGGCAGGAGGTAGGACGAG
CAATGTATGCCCCTCCCATTGAAGGAATAATAACATGTAGATCAAAT---
ATCACAGGATTACTACTGACCCGGGATGGAGGCAGGGGAGACACAAATGGCACA-------------------
----

GAGACAAATAAAACAGAGACATTCAGGCCCGGAGGGGGAGATATGAGGGACAATTGGAGAAGTGAATTATATA
AATATAAAGTGGTAGAAATTAAACCATTA---GGAGTAGCACCAACTAAGGCAAAAAGGAGAGTGGTG-----
----------------------------------GAGAGAGAAAAAAGAGCA---GTGGGA---
ATAGGAGCT---------GTGTTCCTTGGGTTCTTGGGAGCA---GCAGGAAGCACTATG---
GGCGCAGCATCA---ATAACGCTGACGGTACAG---GCCAGACAA---TTATTG---
TCTGGAATAGTGCAACAGCAAAGC---AATTTG---CTGAGG---
GCTATAGAGGCGCAACAGCATATGTTGCAACTCACGGTCTGGGGCATT
>KX181932
TTCTATTGCAATACATCAATCCTGTTTAATGATACATACTGGGTTAATGGGACAGGAAGCACTAAT-------
----

GGAACAGAAAACATCACACTCCCATGCAGAATAAAACAAATTATAAACATGTGGCAGGGAGTAGGACAAGCAA
TATATGCCCCTCCCATTAAAGGAAGAATAACATGTAAATCAAAT---
ATCACAGGAATACTACTGACACGTGATGGAGGAGAAACTAATGAAACT-------------------------
----

AATGCCACAGAGGTATTTAGGCCTGGAGGAGGAGACATGAGGGACAACTGGAGAAGTGAACTATATAAATATA
AAGTGGTAGAAATTAAACCATTA---GGAATAGCACCCACTAGGGCAAAAAGGAGAGTGGTG-----------
----------------------------GAGAGAGAAAAAAGAGCA---GCA------CTAGGAGCT------
---ATGTTCCTTGGGTTCTTGGGAGCA---GCAGGAAGCACTATG---GGCGCAGCGTCA---
ATAGCGCTGACGGTACAG---GCCAGACAA---TTATTG---TCTGGGATAGTGCAACAGCAAAGC---
AATTTG---CTGAGG---GCTATAGAGGCGCAACAGCATATGTTGCAACTCACGGTCTGGGGCATT
>KX181933
TTCTATTGCGATACAACAGGACTGTTTACAGAAAACAGTACTGAGATAGACAACGGTACA-------------
----

GAAAATTCAACCATCATAATCCCATGCAGAATAAGACAATTTGTAAACATGTGGCAAGAGGTAGGACGAGCAA
TATATGCCCCTCCCATTGCAGGAAACATAACATGTAGATCAAAT---
ATCACAGGACTACTACTGACACGTGATGGAGGAGAAGGAAATGAGACA-------------------------
----

CAAAATGATACAGAAACATTCAGGCCTGGAGGGGGAAATATGAAGGACAATTGGAGAAGTGAATTATATAAAT
ATAAGGTGGTAGAAATTAAGCCCTTA---GGAATAGCCCCCCCAAGGCAAAAAGGAGAGTGGTG--------
-------------------------------GAGAGAGAAAAAAGAGCG---GCA------TTAGGAGGT---
------GTGTTCCTTGGGTTTTTGGGAGCA---GCAGGAAGCACTATG---GGCGCAGCATCA---
ATGATGCTGACGGCACAG---GCCCGACAA---TTATTG---TTTGGGATAGTGCAACAGCAAAGC---
AATTTG---TTGAGA---GCTATAGGGGCGCAACAGCACATGTTGCAACCCCCGTTTTGGGGCATT
>KX181934
TTCTATTGCAATACATCAAACCTGTTTAATAGTACATACAATAGTAACTGCACATCTAATAGGACA-------
----

GGAACTGAAAACATAACAATCCCATGCAGAATAAAACAAATTATAAACATGTGGCAGGGGGTGGACGAGCAA
TGTATGCCCCTCCCATTAAAGGACAAATAACATGTAGATCAAAT---
ATCACAGGATTAGTACTAGAAAGGGATGGAGGCACAAATAAGACA---------------------------
----

GAAGAGACATTCTGGCCTACAGGAGGAGATATGAGGGACAATTGGAGAAGTGAATTATATAAATATAAAGTGG
TAGAAGTTAGACCATTA---GGAATAGCACCCACTAGAGCAAAAAGGAGAGTGGTG----------------
----------------------GAGAGAGAAAAAAGAGAT---TTGGGA---CTAGGAGCT---------
GTGTTCCTTGGGTTCTTGGGAGCA---GCAGGAAGCACTATG---GGCGCAGCATCA---
ATAACGCTGACGGTACAG---GCCAGACAA---TTGTTG---TCTGGGATAGTGCAACAGCAAAGC---
AATTTG---CTGAGG---GCTATAGAGGCGCAACAGCATATGTTGCAACTCACTGTCTGGGGCATT
>KX181935
TTCTATTGCAATACATCAAAACTGTTTAATAGTACATACTGGCCTAATGGTACAGAAAAC------------
----

ACAAACATCACCATCACAATTCCATGCAGAATAAAACAAATTATAAACATGTGGCAGGAGGTAGGACGAGCAA
TGTATGCCCCTCCCATTAAAGGAATAATAACATGTAGATCAAAT---
ATCACAGGGCTACTACTGACACGTGATGGAGGAAATGGAGATTTCACAGAT---------------------
----

AGCCCAGAAACATTCAGGCCTGGAGGAGGAGATATGAGGGACAATTGGAGAAGTGAATTATATAAATATAAAG
TGGTAGAAATTAAACCATTA---GGAATAGCACCCACTAAGGCAAAAAGGAGAGTGGTG-------------
-------------------------GAGAGAGAAAAAAGAGCA---GTGGGA---ATAGGAGCT--------
GTGTTCCTTGGGTTCTTGGGAGCA---GCAGGAAGCACTATG---GGCGCAGCATCA---
ATAACGCTGACGGTACAG---GCCAGACAA---TTATTG---TCTGGGATAGTGCAACAGCAAAGC---
AATTTG---CTGAGG---GCTATAGAGGCGCAACAGCATATGTTGCAACTCACGGTCTGGGGCATT
>KX181938
TTCTATTGCAATACATCAGGCCTGTTTAATGGTACATATACACCTAATAGTAATAGTACTGAAACA------
----

GGAAATTCAACAATCACCATCCCATGCAGAATAAAACAAATTATAAATATGTGGCAAGGGGTAGGGCGAGCAA
TGTATGCCCCTCCCATTAGTGGAATAATAACATGTAGATCAAAT---
ATCACAGGGATACTACTGACACGGGATGGAGGCGATAGCAACAAT---------------------------
----

GATACAGAGATATTCAGGCCTACAGGAGGAGATATGAGGGACAATTGGAGAAGTGAATTATATAAATATAAAG
TGGTAGAGATTAAACCCGTA---GGGATAGCCCCCCCCAAGGCAAAGAGAAGAGTGGTG-------------
-------------------------GAGAGAGAAAAAAGGGGA---ATAGGA---ATAGGAGCT--------
GTGTTCCTTGGGTTTTTGGGAGCA---GCAGGAAGCACTATG---GGCGCAGCATCA---
ATAACGCTGACGGTACAG---GCCCGACAA---TTATTG---TTTGGAATAGTGCAACAGCAAAGC---
AATTTG---CTGAGA---GCTATAGAGGCGCAACAGCATATGTTGCAACTCACTGTCTGGGGCATC
>KX181939
TTCTATTGCAATACATCAGGACTGTTTAATAGTACATATTTTAATGGTACATATAATAGTACAGGA------
----

AACAGTCCAAACATCACACTCCCATGCAGAATAAAACAAATTGTAAGAATGTGGCAGAGGGTAGGACAAGCAA
TGTATGCCCCTCCCATTCCAGGAATAATAACATGTAGATCAAAT---
ATCACAGGATTGTTACTGACACGGGATGGAGGCCCGGATAATAAT---------------------------
----

CGGACAGAGACATTCAGGCCTGGAGGAGGAGATATGAGGGACAATTGGAGAAGTGAATTATATAAATATAAAG
TGGTAGAAATTAAACCATTA---GGAATAGCACCCACTAAGGCAAAAAGGAGAGTGGTG-------------
-------------------------GAGAGAGAAAAAAGAGCA---GTAGGA---CTAGGAGCC--------
ATGTTCCTTGGGTTCTTGGGAGCA---GCAGGAAGCACTATG---GGCGCAGCATCA---
ATAACGCTGACGGTACAT---GCCAGACAA---TTACTG---TCTGGGATAGTGCAACAGCAAAGC---
AATTTG---CTGAGG---GCTATAGAGGCGCAACAGCATATGTTGCAACTCACGGTCTGGGGCATT
>KX181940
TTCTATTGTAATACATCAAGCCTGTTTAATAGGACATACCCAGCTAATAGTACAGGATACAATAATGAAACA-
----

GGAAATTCAACCATTACAATCCCATGCAGAATAAAACAAATTATAAATAGATGGCAGGAGGTGGGACAAGCAA
TGTATGCCCCTCCCATTGCAGGAATAATAACATGTAGATCAAAT---
ATCACAGGATTACTACTGACACGGGATGGAGGCAACACAACAAAT----------------------------
----

AAAACTGAGATATTCAGGCCTGAAGGAGGGAATATGAGGGACAATTGGAGAAGTGAATTATATAAATATAAAG
TGGTAGAAATTAAACCATTA---GGAATAGCACCCACTAAGGCAAAAAGGAGAGTGGTG--------------
-------------------------GAGAGAGAAAAAAGAGCA---GTGGGA---ATAGGAGCT---------
GTGTTCCTTGGGTTCTTGGGAGCA---GCAGGAAGCACTATG---GGCGCAGCATCA---
ATAACGCTGACGGTACAA---GCCAGACAA---TTATTG---TCTGGGATAGTGCAACAGCAAAGC---
AATTTG---CTGAGG---GCTATAGAGGCGCAACAGCATATGTTGCAACTCACGGTCTGGGGCATT
>KU749391
TTCTATTGCAATACATCAAGCCTGTTTAACCATACATACCACAACCTGAATAGTAGTAGTACAAACACA----
----

ACAGCAAATATCACAAGAATACCATGCAGAATAAAACAAATTATAAATATGTGGCAGGGGGTAGGACGAGCAA
TGTATGCCCCTCCCATTAAAGGAAAAATAACATGTAGATCAAAC---
ATCACAGGATTAGTACTGACACGGGATGGAGGTAATACAGGAGGG----------------------------
----

ACAGAGATATTCAGGCCTGGAGGAGGAGATATGAGGGACAATTGGAGAAGTGAACTATATAAATATAAGGTGG
TAGAAATTAAACCATTA---GGAATAGCACCCACTAAGGCAAAAAGGAGAGTGGTG-----------------
----------------------GAGAGAGAAAAAAGAGCA---GTGGGA---ATAGGAGCT---------
GTGTTCCTTGGGTTCTTGGGAGCA---GCAGGAAGCACTATG---GGCGCAGCATCA---
CTAACGCTGACGGTACAG---GCCAGACAA---TTATTG---TCTGGGATAGTGCAACAGCAAAGC---
AATTTG---CTGAGG---GCTATAGAGGCGCAACAGCATATGTTGCAACTCACGGTCTGGGGCATT
>KT427678
TTCTATTGCAATACGTCAGGGCTGTTTAATAATACATGGCTTAACGTGACTAATAAAACA-------------
----

GAAAATCCAACCATCACAATCCCATGTAGAATAAAACAAATTATAAACATGTGGCAGGAAGTAGGAAAAGCAA
TGTATGCCCCTCCCATTGCAGGAAAGATAACATGTAGATCAAAT---
ATCACAGGACTACTACTGACACGTGATGGAGGTGACAATATCACA----------------------------
----

GAAGAAATATTCAGGCCTGCAGGAGGAGACATGAGGGACAATTGGAGAAGTGAATTATATAAATATAAAGTGG
TAGAAATTAAACCATTA---GGAATAGCACCCACCAAGGCAAAAAGGAGAGTGGTG-----------------
----------------------GAGAGAGAAAAAAGAGCAGTAGTGGGA---ATAGGAGCT---------
GTGTTCCTTGGGTTCTTGGGAGCA---GCAGGAAGCACTATG---GGCGCAGCATCA---
ATAACGCTGACGGTACAG---GCCAGACAA---TTATTG---TCTGGAATAGTGCAACAGCAAAGC---
AATTTG---CTGAGA---GCTATAGAGGCGCAACAGCATATGTTGCAACTCACGGTCTGGGGCATT
>KT427736
TTCTATTGCGATACATCAGGCCTGTTTAATAGTACATTTAATAAGACAGGAAACAGTAATAGTACA-------
----

GTAAACAGCACAATCACAATCCAATGCAGAATAAAACAAATTATAAACATGTGGCAGGAGGTAGGACGAGCAA
TGTATGCCCCTCCCATTAAAGGAAACATAACATGTAGAACAAAT---
ATCACAGGAATACTACTGACACGTGATGGAGGAAACGAAACTAAC----------------------------
----

ACAGAAGAGATATTCAGACCTGGAGGAGGAGATATGAGGGACAATTGGAGAAGTGAATTATATAAATATAAAG
TGGTAGAAATTAAACCATTA---GGAATAGCACCCACTGAGGCAAGAAGGAGAGTGGTG--------------
-------------------------GAGAGAGAAAAAAGAGCA---ATAGGGGGAATAGGAGCT---------
GTGTTCCTTGGGTTCTTGGGAGCA---GCAGGAAGCACTATG---GGCGCAGCATCA---
ATAACGCTGACGGTACAG---GCCAGACAA---TTATTG---TCTGGGATAGTGCAAGAGCAAAGC---
AATTTG---CTGAGG---GCTATAGAGGCGCAACAGCATATGTTGCAACTCACGGTCTGGGGCATT
>KT427800
TTCTATTGCAATACATCAGGGCTGTTTAATGCTACATTTAATGCTACACACTGGTCTAATATGACAGAAAGTA
CTAATGAA---

ACAGACAATACAACCATCATAATCCCATGTAAAATAAAACAAATTGTAAACCTGTGGCAGGAGGTAGGAAAAG
CAATGTATGCCCCTCCCATTGCAGGAATGATAACATGTAGGTCAAAT---
ATCACAGGACTACTACTGACACGTGATGGAGGTAATACAGATAAC----------------------------
----

AGTAGCACAGAAATATTCAGGCCTGGAGGAGGAGACATGAGGGACAATTGGAGAAGTGAATTATATAAATATA
AAGTGGTAGAAATTAAACCATTA---GGAATAGCACCCACCGAGGCAAAAAGGAGAGTGGTG-----------
----------------------------GAGAGAGAAAAAAGAGCA---GTGGGA---ATAGGAGCT------
---TTGTTCCTGGGGTTCTTGGGAGCA---GCAGGAAGCACTATG---GGCGCAGCATCA---
ATAACGCTGACGGTACAG---GCCAGACAA---TTATTG---TCTGGAATAGTGCAACAGCAAAGC---
AATTTG---CTGAGA---GCTATAGAGGCGCAACAGCATATGTTGCAACTCACGGTCTGGGGCATT
>KU749392
TTCTATTGCAATACATCAAGCCTGTTTAATAAGACATACCTGTTTAATAATACATACCTGGTTAAGAATGGCA
CG---
GTTACAGAAAACAATAAT---------
GGAACAAACATCACAGTCACAATCCCATGCAGAATAAAACAATTTATAAACATGTGGCAGGAGGTAGGACGAG
CAATGTATGCCCCTCCCATTGCAGGACCAATAACATGTATATCAAAT---
ATCACAGGGTTACTACTGACACGGGATGGAGGCCTTAACACAACG----------------------------
----

AACGAGACATTCAGGCCTGGAGGAGGAGATATGAGGGACAATTGGAGAAATGAATTATACAAATATAAAGTGG
TAGAAATTAAACCATTA---GGAATAGCGCCCACTAAGGCAAAAAGAAGAGTGGTG-----------------
----------------------GAGAGAGAAAAAAGAGCA---GTGGGA---ATAGGAGCT---------
GTGTTCCTTGGGTTCTTGGGAGCA---GCAGGAAGCACTATG---GGCGCAGCGTCA---
ATAACGCTGACGGTACAG---GCCAGACAA---TTACTG---TTTGGGATAGTGCAACAGCAAAGT---
AATTTG---CTGAGA---GCTATAGAGGCGCAACAACATATGTTGCAACTCACGGTCTGGGGCATT
>KU749393
TTCTATTGCAATACATCAGATCTGTTTAATGATATAGACACATCTAAT-------------------------
----

AACACTGAGAACATCACAATCCCATGCAGGATAAAACAGATTATAAACATGTGGCAGGAAGTAGGACGAGCAA
TGTATGCCTCTCCCATTAAAGGACCAATAACATGTAGATCAAAT---
ATCACAGGATTATTACTGACACGGGATGGAGGCACGGACGGGGGA----------------------------
----
GGCGAAAAGGACAAC---
AACACGGAGACATTCAGGCCTGGAGGAGGAGATATGAGGGACAATTGGAGAAGTGAATTATATAAATATAAAG
TGGTAGAAATTAAACCATTA---GGAATAGCACCAACTAGGGCAAAAAGGAGAGTGGTG--------------
-------------------------GAGAGAGAAAAAAGAGCG---GCA------ATAGGAGCT---------
ATGTTCCTTGGGTTCTTAGGAGCA---GCAGGAAGCACTATG---GGCGCAGCATCA---
ATAACGCTGACGGTACAG---GCCAGACAA---TTGTTG---TCTGGGATAGTGCAACAGCAAAGC---
AATTTG---CTGAGA---GCTATAGAGGCGCAACAACATATGTTGCAACTCACGGTCTGGGGCATC
>MK041570
TTCTATTGCAATACAACAAAGCTATTTAAT---AATTGCACAGGAAACAGTAGTTGCAACACAGAAAACAGT-
----

GATGGAGAAGAGATCACAATCCCATGCAGAATAAGGCAAATTGTAAGAATGTGGCAGGGGGTAGGACAAGCAA
TGTATGCCCCTCCTATAGCGGGATACGTAACATGTAACTCAAAT---
ATCACAGGATTATTACTGACAAGGGATGGAGGGGATACAGGGAAT----------------------------
----

GAGAGTGAGACATTCAGGCCTGGAGGAGGAGATATGAGGGACAATTGGAGAAGTGAATTATATAAATATAAAG
TGGTAGAAATTAAACCATTA---GGAATAGCACCCACTGAGGCAAAAAGGAGAGTGGTG--------------
-------------------------GAGAGAGAAAAAAGAGCA---GTGGGA---ATAGGAGCT---------
ATGTTCCTTGGGTTCTTGGGAATA---GCAGGAAGCACTATG---GGCGCAGCGTCA---
ATAACGCTGGCGGTACAG---GCCAGACAA---TTATTG---TCTGGAATAGTGCAACAGCAAAGT---
AATTTG---CTGAGG---GCTATAGAGGCGCAACAGCATATGTTGCAACTCACGGTCTGGGGCATT
>MK041576
TTCTATTGCAATACATCAAGGCTGTTTAATAGTACATATGCACCTAATGGTACAGAAAACAGTACTGAAACA-
----

AACAATTCAACCATCACAATCCCATGCAGAATAAAACAAATTGTAAGAATGTGGCAGGGGGTAGGACAAGCAA
TGTATGCCCCTCCCATTAAAGGAAACATAACATGTAGATCAAAT---
ATCACAGGACTACTACTAACACGTGATGGAGGTGATGGAGGAAAT----------------------------
----

AATACAAATAATACAGAAACATTCAGGCCTGAAGGAGGAGATATGAGGGACAATTGGAGAAGTGAATTATATA
AATATAAAGTGGTAGAAATCAAACCATTA---GGAATAGCACCCACTGAGGCAAAAGGAGAGTGGTG-----
----------------------------------GGGAGAGAAAAAAGAGCA---GTGGGA---
ATAGGAGCT---------GTGTTCCTTGGGTTCTTGGGAGCA---GCAGGAAGCACTATG---
GGCGCAGCATCA---ATAACGCTGACGGTACAG---GCCAGACAA---TTATTG---
TCTGGGATAGTGCAACAGCAAAGC---AATTTG---CTGAGG---
GCTATAGAGGCGCAACAGCATATGTTGCAACTCACGGTCTGGGGCATT
>MK041550
TTCTATTGCAACACATCAACACTATTTAATAGTACACACAATGGTACATACAATGGTACAGAAAACATTACAA
TCCCATACAAT--

GGTACAGAAAACATTACAATCCCATGCAAAATAAAACAAATTATAAACATGTGGCAGAAAGTGGGAAGCGCAA
TGTATGCCCCTCCCATTAAAGGAGTAATAACATGTCTATCAAAT---
ATCACAGGATTACTACTGACACGTGATGGAGGGGACACAGAGGAA----------------------------
----

AATGAGACAGAGACATTCAGGCCTGGAGGAGGAGATATGAGGGACAATTGGAGAAGTGAGTTATATAAATATA
AAGTGGTAGAAATTAAACCATTG---GGAGTAGCACCCACTAGGGCAAAAAGGAGAGTGGTG-----------
----------------------------GAGAGAGAAAAAAGAGCA---GTGGGA---TTAGGAGCT------
---GTGTTCCTTGGGTTCTTGGGAGCA---GCAGGAAGCACTATG---GGCGCAGCATCA---
ATAACGCTGACGGTACAG---GCCAGACAA---TTATTG---TCTGGGATAGTGCAACAGCAGAGC---
AATTTG---CTGAGG---GCTATAGAGGCGCAACAGCACATGTTGCAACTCACGGTCTGGGGCATT
>MK041553
TTTTATTGCAATACATCAGGCCTGTTTAATGGTACATATCCACCTAATAGTACAGGAAATGGTAATGGAACA-
----

GATGAGGAAAACATTACCCTCCCATGCAGAATAAAACAAATTATAAATATGTGGCAGGGGGTAGGACGAGCAA
TGTATGCCCCTCCCATTAAAGGACCGATAACATGTAGATCAAAC---
ATCACAGGATTACTGTTGACACGGGATGGAGGGGTAAACAGCAAC----------------------------
----

AACAGCACCGAGACATTCAGGCCTGAAGGAGGAGATATGAGGGACAATTGGAGAAGTGAATTATACAAATATA
AAGTGGTAGAAATCAAACCATTA---GGAATAGCACCCACTAAGGCAAAAAGAAGAGTGGTG-----------
----------------------------GAGAGAGAAAAAAGAGCA---GTGGGA---ATAGGAGCT------
---GTGTTCCTTGGGTTCTTGGGAGCA---GCCGGAAGCACTATG---GGCGCAGCATCA---
ATAACGCTGACGGTACAG---GCCAGACAA---TTATTG---TCTGGGATAGTGCAGCAGCAAAGC---
AATTTG---CTGAGG---GCTATAGAGGCGCAACAGCATATGTTGCAACTCACGGTCTGGGGCATT
>MK041561
TTCTATTGCAATACATCAAGATTGTTTAATGGTACATATAATACTACAGGAAACAGTAATGAAACA-------
----

AACAATGCAAACATCACAATCCCATGTAGAATAAAACAAATTGTAAACATGTGGCAGGAGGTAGGACGAGCAA
TGTATGCCCCTCCCATTGCAGGAGCAATAACATGTGAATCAAAT---
ATCACAGGATTGCTACTGACATGGGATGGCGGCAAGGGGAATGACACG-------------------------
----

AACAACACAGAGACATTCAGGCCTGGAGGAGGAGATATGAAGGACAATTGGAGAAGTGAATTATATAAATATA
AAGTGGTAGAGATTAAACCATTA---GGAATAGCACCCACTGGAGCAAAAAGGAGAGTGGTG-----------
----------------------------GAGAGAGAAAAAAGAGCA---GTGGGA---ATAGGAGCT------
---GTATTCCTTGGGTTCTTGGGAGCA---GCCGGAAGCACTATG---GGCGCAGCATCA---
ATAACGCTGACGGTACAG---GCCAGACAA---CTATTG---TCTGGTATAGTGCAACAGCAAAGC---
AATTTG---CTGAGA---GCTATAGAGGCGCAACAGCATATATTGCAACTCACGGTCTGGGGCATT
>MK041563

TTCTATTGCAATACATCAAGACTATTTAATGGTACATACAACTCTACTACTACAGGAAACAGTACT-------
----

GAAAATGAAATCATCACAATCCAATGCAGAATAAAACAAATTATAAACATGTGGCAGGGGGTAGGACGAGCAA
TGTATGCCCCTCCCATTGCAGGAAACATAACATGTAGATCAAAT---
ATCACAGGATTACTACTGACACGGGATGGAGGGAATAGCAGCAGC----------------------------
----

CCAGAGGAGATATTCAGGCTTGGAGGAGGAGATATGAAGGACAATTGGAGAAGTGAATTGTATAAATATAAAG
TGGTAGAAATTAAACCATTA---GGAATAGCACCCACTAAGGCAAAGAGAAGAGTGGTG--------------
-------------------------GAGAGAGAAAAAAGAGCA---GTGGGA---ATAGGAGCT---------
GTGTTCTTTGGGTTCTTGGGAGCC---GCAGGAAGCACTATG---GGCGCAGCATCA---
ATAACGCTGACGGTACAG---GCCAGACAA---TTATTG---TCTGGGATAGTGCAACAGCAAAGC---
AATTTG---CTGAGG---GCTATAGAGGCGCAACAGCATATGTTGCAACTCACAGTCTGGGGCATT
>MK041564
TTCTATTGCAATACAACACAACTGTTTAATAGAACATATTGGTTTAATAGTACAACTAATGACACAGGCACTA
ATGACACA---

GAAGATAAAAACATCACAATCCCATGCAGAATAAAGCAAATTATAAACCTGTGGCAGGAGGTAGGACGAGCAA
TGTATGCCCCTCCCATTGAGGGAGCCATAACATGTAACTCAAAT---
ATCACAGGATTACTACTGACACGTGATGGAGGCACAGGAAATGATACCACA----------------------
----

AATGCCACAGAGACATTCAGGCCTGGAGGAGGAGATATGAGGGACAATTGGAGAAGTGAATTATATAAATATA
AAGTGGTAGAAATTAAGCCATTA---GGAATAGCACCCACTAAGGCAAAGAGAAGAGTGGTG-----------
----------------------------GAGAGAGAAAAAAGAGCA---GCGGGA---CTAGGAGCT------
---GTGTTCCTTGGGTTCTTGGGAATA---GCAGGAAGCACTATG---GGCGCAGCGTCC---
ATAACGCTGACGGCACAG---GCCAGACAA---TTACTG---TCTGGGATAGTGCAACAGCAAAGC---
AATTTG---CTGAGG---GCTATAGAGGCGCAACAGCATATGTTGCAACTCACGGTCTGGGGCATC
>U52953
TTCTATTGCAATACATCAAGCCTGTTTAATAGTACATACACACCTAATAGTACAGAAAACATTACTGGAACA-
----

GAAAATTCAATCATCACAATCCCATGCAGAATAAAACAAATTATAAATATGTGGCAGGGGGTCGGACGAGCAA
TGTATGCCCCTCCCATTGAAGGAATATTAACATGTAGATCAAAT---
ATCACAGGATTACTACTGACACGGGATGGAGGTACAGGTATGCAT----------------------------
----

GACACAGAGATATTCAGGCCTGAAGGAGGAGATATGAGGGACAATTGGAGAAGTGAATTATATAAATATAAAG
TGGTAGAAATTAAACCATTA---GGAATAGCACCCACTAAGGCGAAAAGGAGAGTGGTG--------------
-------------------------GAGAGAGAAAAAAGAGCA---GTGGGA---ATAGGAGCT---------
GTGTTCCTTGGGTTCCTGGGAGCA---GCAGGAAGCACTATG---GGCGCAGCATCA---
ATAACGCTGACGGTACAG---GTCAGACAA---TTATTG---TCTGGGATAGTGCAACAGCAAAGC---
AATTTG---CTGAGA---GCTATAGAGGCGCAACAGCATATGTTGCAACTCACGGTCTGGGGCATT
>AF443088
TTCTATTGCAATACAACAAGACTGTTTAATGGTACATATAATAGTACAGGAAATGATACAAATTCA-------
----

ACAAATTCAACCATCACACTCCAATGCAGAATAAAACAAATTATAAACATGTGGCAGGGGGTAGGAAAAGCAA
TGTATGCCCCGCCCATTGCAGGAAACATAATATGTAAATCAAAC---
ATTACAGGACTACTATTGACACGTGATGGAGGTGAAGGTGAAAAT----------------------------
----

ACAACAGAGACATTCAGGCCTGCAGGAGGAGATATGAGGGACAATTGGAGAAGTGAATTATATAAATATAAAG
TGGTAGAAATTAAGCCATTA---GGAATAGCACCCACTAGCGCAAAAAGGAGAGTGGTG--------------
-------------------------GAGAGAGAAAAAAGAGCA---GTAGTGGGAATAGGAGCT---------
GTGTTCCTTGGATTCTTGGGAGCA---GCAGGAAGCACTATG---GGCGCAGCATCA---
ATAACGCTGATGGTACAG---GCCAGACAA---CTGTTG---TCTGGTATAGTGCAACAGCAAAAC---
AATTTG---CTGAGA---GCTATAGAGGCGCAACAGCATATGTTGCAACTCACGGTCTGGGGCATT
>AF443089

TTCTATTGCGATACATCAAACCTGTTTAATAAGACACGCAGGGATAAT-------------------------
----

GCTAATGAAACTATCACACTCCCATGCAGAATAAAACAAATTATAAACATGTGGCAGGAGGTAGGACGAGCAA
TGTATGCCCCTCCCATTGAAGGAAACATAACATGTAAATCAAAT---
ATCACAGGACTACTCTTGGTACGTGATGGAGGAAAAACAGAGGAT----------------------------
----

AACAAAAGTGAGATATTCAGACCTGGAGGAGGAGATATGAGGGACAATTGGAGAAGTGAATTATATAAATATA
AAGTGGTAGAAATAAAGCCATTG---GGAGTAGCACCCACTGAGGCAAAAAGGAGAGTGGTG-----------
----------------------------GAGAGAGAAAAAAGAGCA---GTGGGA---ATAGGAGCT------
---GTGCTCCTTGGGTTCTTAGGAGCA---GCAGGAAGCACTATG---GGCGCAGCGTCA---
ATAACGCTGACGGTACAG---GCCAGACAA---GTGCTG---TCTGGAATAGTGCAACAGCAAAGT---
AATTTG---CTGAGG---GCTATAGAGGCGCAACAGCATATGTTGCAACTCACGGTCTGGGGCATT
>AF443092
TTCTATTGCAATACATCAAAACTGTTTAATGGTACATATAATAATACAAATTCTAATAAT-------------
----

AATACAGCAGACATCACACTCCAATGCAGAATAAAACAAATTATAAACTTGTGGCAGGGGGTAGGACGAGCAA
TGTATGCCCCTCCCATTGCAGGAAACATAACATGTAAATCAAAT---
ATCACAGGACTACTATTGACACGTGATGGAGGAGGAGGAGAGAAC----------------------------
----

AACTCAACAGAGACATTCAGACCTGCAGGAGGAGAAATGAGGGACAATTGGAGGAGTGAATTATATAAATATA
AAGTGGTAGAAGTTAAGCCATTA---GGAATAGCACCCACTGAGGCAAAAAGAAGGGTGGTG-----------
----------------------------CAGAGAGAAAAAAGAGCA---GTAGGA---ATAGGAGCT------
---GTGTTCCTTGGGTTCTTGGGAGCA---GCAGGAAGCACTATG---GGCGCGGCGTCC---
ATAACGCTGACGGTACAG---GCCAGACAA---TTATTG---TCTGGTATAGTGCAACAGCAGAAC---
AACTTG---CTGAGA---GCTATAGAGGCGCAACAGCATATGTTGCAACTCACGGTCTGGGGCATT
>AF443093
TTCTATTGCAATACATCAAATCTGTCTAATGAGACATACCTGGCAAACCTGACACGGATGTCTAATGTAACA-
----

AAAAACGCCACAATCACACTCCCATGCAGAATAAAACAAATTATAAACATGTGGCAGGAGGTAGGACGGGCAA
TATATGCCCCTCCTATTGCAGGAAAAATAACATGTATATCAAAT---
ATCACAGGAACACTATTGACGCGTGATGGAGGAGTGTCAAATACAACA-------------------------
----

GAGGGCAATGAGACATTCAGACCTGGAGGAGGAGATATGAGGAACAATTGGAGAAGTGAATTATACAAATACA
AAGTGGTAGAAATCAGGCCATTG---GGAGTAGCACCCACTGAGGCAAGAAGGCGAGTGGTG-----------
----------------------------GAGAGAGAAAAAAGAGCA---GTAGGAGCAATAGGAGCT------
---GTGCTCCTTGGGTTCTTGGGAGCA---GCCGGAAGCACTATG---GGCGCAGCATCA---
ATGACGCTGACGGTACAG---GCCAGACAA---TTGCTG---TCTGGTATAGTGCAACAGCAAAGT---
AATTTG---CTGAGG---GCTATAGAGGCGCAACAGCATATGTTGCAACTCACGGTCTGGGGCATT
>AF443094
TTCTATTGCAATACATCAAACTTGTTTAATAATACATACAGGGCTGATAGTACATCCAATAATATAACAAAT-
----

GATAATTCAAATATCACACTCCAATGCAGGATAAAACAAATTATAAACATGTGGCAGGAAGTAGGACGAGCAA
TGTATGCCCCTCCCATTGAAGGAAACATAACATGTAACTCAAGT---
ATCACAGGACTACTATTGACACGTGATGGAGGAAAAGAAGAGAACAATAGC----------------------
----

ACAAACAATGGCACAGAGATATTCAGACCTGGAGGAGGAGATATGAGGGACAATTGGAGAAGTGAATTATATA
AATATAAAGTGGTAAAAATTAAGCCATTA---GGAATAGCACCCACCAAGGCACAAAGGAGAGTGGTG-----
----------------------------------AAAAGAGAAAAAAGAGCA---GTGGGA---
ATAGGAGCT---------GTGTTCCTTGGGTTCTTGGGAGCA---GCCGGAAGCACTATG---
GGCGCAGCATCA---ATAACGCTGACGGTACAG---GCCAGACAA---TTGCTG---
TCTGGTATAGTGCAACAGCAAAGC---AATTTG---CTGAGG---
GCTATAGAGGCGCAACAGCATATGTTGCAACTCACGGTCTGGGGCATT

>AF443095
TTCTATTGCAACACATCAGCACTGTTTAATAGTACATATAATAGTACATACAATACAAGCGGGCATAAT----
----

GATACAAGAATCATCACACTCCCATGCAGAATAAAACAAATTATAAACATGTGGCAGAAGGTAGGACGAGCAA
TGTATGCCCCTCCCATTGCAGGAAACATAACATGTAAATCCAAT---
ATCACAGGACTACTCTTGACACGTGATGGAGGAAATACAAGTAGC----------------------------
----

ACAGAAGAGATATTCAGACCTGAAGGAGGGGATATGAGGGATAACTGGAGGAGTGAATTATATAAATATAAAG
TGGTAGAAATTAAACCATTG---GGAGTAGCACCCACTAAGGCAAAAAGGAGAGTGGTG--------------
-------------------------GAGAGAGAAAAAAGAGCA---GTGGGA---ATAGGAGCT---------
ATGTTCCTTGGGTTCTTGGGAGCA---GCAGGAAGCACTATG---GGCGCGGCGTCA---
ATAACGCTGACGGTACAG---GCCAGACAA---TTGCTG---TCTGGTATAGTGCAGCAGCAAAGC---
AATTTG---CTGAGG---GCTATAGAGGCGCAACAGCATCTGTTGCAACTCACGGTCTGGGGCATT
>AF443096
TTCTATTGTAATACATCAAAACTGTTTAATGGTACATACAATGGTACATCCATCAGTACGGAC----------
----

AGTAACTCAAGCATCACACTCCAATGCAGAATAAAACAAATTATAAACATGTGGCAGGGGGTAGGACAAGCAA
TATATGCCCCTCCCATTGCAGGAAACATAACATGTAAATCAAAT---
ATCACAGGACTACTATTGACACGTGATGGAGGGAATAACACAGAG----------------------------
----

AACACAGAGACATTCAGACCTGGAGGAGGAGACATGAGGGATAATTGGAGAAATGAATTATATAAATATAAAG
TAGTAGAAATTAAGCCATTA---GGAATAGCACCCACTAGTGCAAAAAGGAGAGTGGTG--------------
-------------------------GAGAGAGAAAAAAGAGCA---GTAGGG---ATAGGAGCT---------
GTGTTCCTTGGGTTCTTGGGGGCA---GCAGGAAGCACTATG---GGCGCGGCGTCA---
ATAACGCTGACGGTACAG---GTCAGACAA---TTGTTG---TCTGGTATAGTGCAACAGCAAAAC---
AATTTG---CTGAGG---GCTATAGAGGCGCAACAGCATATGTTGCAACTCACAGTCTGGGGCATT
>AF443098
TTCTACTGCAATACATCACAACTGTTTAATGGTACATACATGCCTAATAGTACATACATGTCTAATAGTTCA-
----

GACAATAGAAACATCACAATCCCATGCAGAATAAAACAAATTATAAACCTATGGCAGGAGGTAGGACGAGCAA
TGTATGCCCCTCCCATTGCAGGAAACATAACATGTAAATCAAAT---
ATCACAGGGCTACTATTGACACGTGATGGAGGAGGAGAAAGTAACACA-------------------------
----

ACAAATGCAACAGAGACATTCAGACCTGGAGGAGGGGACATGAGGGACAATTGGAGAAGTGAATTATACAAAT
ATAAAGTGGTAGAAGTTAAACCATTG---GGATTAGCACCCACTGAGGCAAAAAGGAGAGTGGTG--------
-------------------------------GAGAGAGAAAAAAGAGCA---GTGGGA---ATAGGAGCT---
------GTGTTCCTTGGGTTCTTGGGAGCA---GCAGGAAGCACTATG---GGCGCGGCGTCA---
ATGACGCTGACGGTACAA---GCCAGACAA---CTATTG---TCTGGTATAGTGCAACAGCAAAGC---
AATTTG---CTGAAG---GCTATAGAGGCGCAACAGCATCTGTTGCAACTCACGGTCTGGGGCATT
>AF443101
TTCTATTGCAATACATCACAACTGTTCAATGGTACATACAATGATACATACAATGGTACAGAAAGTAATTCA-
----

GGTAATTCAACTATCACACTCCCATGCAGAATAAAACAAATTATAAACATGTGGCAGGGGGTAGGACGAGCAA
TATATGCCCCTCCCATTGAAGGAAACATAACATGTAAATCAAAT---
ATCACAGGACTACTATTGACGCGGGATGGAGGGAAAGGGAATGAT----------------------------
----

ACCGCAGAGATATTCAGACCTGAAGGAGGAGATATGAAGAACAATTGGAGAAGTGAATTATATAAATATAAAG
TAGTAGAAATTAGGCCATTG---GGAGTAGCACCCACTAAAGCAAAAAGGAGAGTGGTG--------------
-------------------------GAGAGAGAAAAAAGAGCA---GCA------CTAGGAGCT---------
GTGCTCCTTGGGTTCTTGGGAGCA---GCAGGAAGCACTATG---GGCGCAGCATCA---
ATAACGCTGACGGTACAG---GCCAGACAA---TTGCTG---TCTGGAATAGTGCAACAGCAAAGC---
AATTTG---CTGAAG---GCTATAGAGGCGCAACAGCATCTGTTGCAACTCACGGTCTGGGGCATT

>AF443104
TTCTATTGCAATACATCAGGCCTGTTTAATGGAACGTTTAATGGAACACACCTAAACATATCT----------
----

ACTAATACTAACATCACACTCCCATGCAGAATAAAACAAATTATAAACATGTGGCAAGAGGTAGGACGAGCAA
TGTATGCCCCTCCCATTGCAGGAAACATAACATGTAAATCAAAT---
ATCACAGGAATACTATTGACACGAGATGGAGGACCAGAAGATACAAAG-------------------------
----

AATAAGACAGAGACATTCAGACCTGGAGGAGGAGATATGAGGGACAATTGGAGAAGTGAATTATATAAATATA
AAGTGGTAGAAATTAAGCCATTG---GGAGTAGCACCCACTAAAGCAAAAAGGAGAGTGGTG-----------
----------------------------GAGAGAGAAAAAAGAGCA---GTGGGA---ATAGGAGCT------
---GTGTTCCTTGGGTTCTTGGGAGCA---GCAGGAAGCACTATG---GGCGCGGCGTCA---
ATAACGCTGACGGTACAG---ACCAAACAA---TTGTTG---TCTGGTATAGTGCAACAGCAAAGC---
AATTTG---CTGAAG---GCTATAGAGGCGCAACAGCATCTGTTGCAACTCACAGTCTGGGGCATT
>AF443110
TTCTATTGTAATACATCAATTCTGTTTAATGATACATATTGGTTTAATGGGACAGACATGGCTAATGATACA-
----

GGAAGTAATAACATCACAATCCCATGCAGGATAAAACAAATTATAAACATGTGGCAGGAGGTAGGACGAGCAA
TATATGCCCCTCCCATTAGAGGAATCATAACATGTACATCAAAT---
ATCACAGGACTGTTATTGACACGTGATGGAGGAAACACAGGAGGA----------------------------
----

AACACAACAGAGATATTCAGACCTGAAGGAGGAGATATGAGGAACAATTGGAGAAATGAATTATATAAATATA
AAGTGGTAGAAATTAAGCCATTG---GGAATAGCACCCACTGGGGCAAAAAGGAGAGTGGTG-----------
----------------------------GAGAGAGAAAAAAGAGCA---GTGGGA---ATAGGAGCT------
---GTGTTCCTTGGGTTCTTGGGAGCA---GCAGGAAGCACTATG---GGCGCAGCGTCA---
ATAACGCTGACGGCACAG---GCCAGACAA---TTGCTG---TCTGGAATAGTGCAACAGCAAAGC---
AATTTG---CTGAGG---GCTATAGAGGCGCAACAACATATGTTGCAACTCACGGTCTGGGGCATT
>AF443111
TTCTATTGTAATACATCAGGCCTGTTTAATAATAACCTGATTAATAATGGG----------------------
----

GCAGAAGATACTATCAGACTCCCATGCCGAATAAAACAAATTATAAACATGTGGCAGGAGGTAGGACGAGCAA
TGTATGCCCCTCCCATTGCGGGAAACATAACATGCACATCAAAT---
ATCACAGGACTGTTGCTGACACGGGATGGGGGAAACGGAGGAAAT----------------------------
----

AATACAGAGACATTCAGACCTGGAGGAGGAAATATGAAGGACAATTGGAGAAGTGAATTATATAAATATAAAG
TAGTAGAAATTAAGCCATTG---GGAGTAGCACCCACTGAGGCAAAAAGGAGAGTGGTG--------------
-------------------------GAGAGAGAAAAAAGAGCA---GTGGAA---ATAGGAGCT---------
GTGTTCCTTGGGTTCTTGGGAGCA---GCAGGAAGCACTATG---GGCGCGGCGTCA---
ATAACGCTGACGGTACAG---GCCAGACAA---TTGCTG---TCTGGTATAGTGCAACAGCAAAGC---
AATTTG---CTGAGG---GCTATAGAGGCGCAACAACATCTGTTGCAACTCACGGTCTGGGGCATT
>AF443112
TTCTATTGTAATACATCAAAACTGTTTAATAGTACAAATAATAATACAGAAAGTGAG----------------
----

TCAAATGCAACCATCACACTCCCATGCAGAATAAAACAATTTATAAGAATGTGGCAGAGGGTAGGACAAGCAA
TGTATGCCCCTCCCATTGCAGGAAACATAACATGTAGATCAAAT---
ATCACAGGGCTACTATTGACAAGGGATGGA---AGCAAAAATGAT----------------------------
----

ACAGAGACATTCAGACCTGGAGGAGGAAATATGAGAGATAATTGGAGAAATGAATTATATAAATATAAAGTGG
TAGAAATTAAGCCATTG---GGAATAGCACCCACTGAGGCAAAAAGGAGAGCGGTG-----------------
----------------------GAGAGAGAAAAAAGAGCA---GTGGGA---ATAGGAGCT---------
GTGATCCTTGGGTTCTTGGGAGCT---GCAGGAAGCACTATG---GGCGCAGCGTCA---
ATAACGCTGACGGCACAG---GCCAGACAA---CTGCTG---TCTGGAATAGTGCAACAGCAAAGC---
AATTTG---CTGAGA---GCTATAGAGGCGCAACAACACCTGTTGCAACTCACAGTCTGGGGCATT

>AF443113
TTTTATTGCAATATATCAAGATTGTTTAATAGGCCAAACATGACTAAAGAGACAAACATGACTAGTGATATAA
AA---

AATAATTCAACAATCACACTCCCATGCAGAATAAAACAAATTATAAACATGTGGCAGGGGGTAGGACGAGCAA
TGTATGCCCCTCCCATTGCAGGAAGAATAATATGTAAATCAAAT---
ATCACAGGACTATTATTGGTACGTGATGGAGGACAGGGAGATAATGTC-------------------------
----

ATGAATGCCACCGAGACATTCAGACCTGGAGGAGGAGATATGAGAGATAATTGGAGAAGTGAATTATACAAAT
ATAAAGTGGTAGAAATTAAGCCATTG---GGAGTAGCACCCACTAGTGCAAAAAGGAGAGTGGTG--------
-------------------------------GAGAGAGAAAAAAGAGCA---GCA------ATAGGAGCT---
------ATGTTCCTTGGGTTCTTGGGAGCA---GCAGGAAGCACTATG---GGCGCGGCGTCA---
ATAACGCTGACGGTACAG---GCCAGAAAA---TTGTTG---TCTGGTATAGTGCAACAGCAAAGC---
AATTTG---CTGAGG---GCTATAGAGGCGCAACAGCATATGTTGCAACTCACGGTCTGGGGCATT
>AF443115
TTCTATTGCAATACATCACAACTGTTTAATAGTACATACAGGGCCAATAGTACAAATAGTACT----------
----

TCAAATTCGAACATCACACTCCCATGCAGAATAAAACAAATTATAAACATGTGGCAGGGGGTAGGACGAGCAA
TGTATGCCCCCCCCATTGCAGGAAACATAATATGTAAATCAAAT---
ATCACAGGAGTACTATTGACATATGATGGAGGAGAAGGGGTAAAG----------------------------
----

GAAAATGAGACATTCAGACCTGCAGGAGGAAATATGAAGGACAATTGGAGAAGTGAATTATATAAATATAAAG
TGGTAGAAATTAAACCATTA---GGAATAGCACCCACTAAGGCAAAAAGGAGAGTGGTG--------------
-------------------------GAGAGAGAAAAAAGAGCA---GCA------CTAGGAGCT---------
GTGTTCCTTGGGTTCTTGGGAGCA---GCAGGAAGCACTATG---GGCGCAGCATCA---
ATAACGCTGACGGTACAG---GCCAGACAA---TTGCTG---TCTGGTATAGTGCAACAGCAAAGC---
AATTTG---CTGAGA---GCTATCGAGGCGCAACAGCATATGTTACAACTCACAGTCTGGGGCATT
>KY658704
TTTTATTGCAATACAACACAACTGTTTAATGGTACATACAATGGCACATACATTAATGATACAGAA-------
----

AGTAATTCAACCTTCACACTCCCATGCAGAATAAAACAAATTATAAACATGTGGCAGGAAGTAGGACGAGCAA
TGTATGCACCTCCAATTGCAGGAAACATAACATGTAAATCAAGT---
ATCACAGGACTACTATTGACACGGGATGGAGGTGACAAAAACGAC----------------------------
----

AACAACAGTGAAATATTCAGACCTGGAGGAGGAGATATGAGGAACAATTGGAGAAGTGAATTATATAAATATA
AAGTGGTAGAAATTAAGCCATTG---GGACTAGCACCCACTGGGGCAAAAAGGAGAGTGGTG-----------
----------------------------GAGAGAGAAAAAAGAGCA---GTGGGA---ATAGGAGCT------
---GTGTTCCTTGGGTTCTTGGGAGCA---GCTGGAAGCACTATG---GGCGCAGCGTCA---
ATAACGCTGACGGCACAG---GCCAGACAA---GTATTG---TCTGGTATAGTGCAACAGCAAAGC---
AATTTG---CTGAGG---GCTATAGAGGCGCAACAGCATATGTTGCAACTCACAGTCTGGGGCATT
>KY658705
TTCTATTGCAATACATCAGGCCTGTTTAATGGTACATATAATGGTACATACCTTGATGAGACAAATTCA----
----

ACAAATTCAACCATCACACTCCAATGCAGAATAAAACAAATTATAAACATGTGGCAGGGGGTAGGACGAGCAA
TGTATGCCCCCCCTATTGCAGGAAACATAACATGTAAATCAAAT---
ATCACAGGAATACTGTTGACACGTGATGGAGGAAAAGGAGAAAAT----------------------------
----

AATAGTACAGAGATATTCAGACCTGGAGGAGGAGATATGAGGGACAATTGGAGAAGTGAGTTATATAAATATA
AAGTGGTTGAAATTAAACCATTA---GGAATAGCACCCACTAAGGCAAGAAGGAGAGTGGTG-----------
----------------------------GAGAGAGAGAAGAGAGCA---GTGGGA---ATAGGAGCT------
---GTGTTCCTTGGGTTCTTGGGAGCA---GCAGGAAGCACTATG---GGCGCGGCATCG---
ATAACGCTGACGGTACAG---GCCAGACAA---TTATTG---TCTGGTATAGTGCAACAGCAAAGC---
AATTTG---CTGAGA---GCTATAGAGACGCAACAACGTCTGTTGCAACTCACAGTCTGGGGCATT

>KY658706
TTCTATTGCAATACATCAGGCCTGTTTAATGGTACATATAATGGTACACATAATAATGCGACAGACTCA----
----

AAAAATCCAACCATCACCCTCCAATGCAGAATAAAACAAATTATAAACATGTGGCAGAAGGTAGGACAAGCAA
TGTATGCCCCCCCCATTGCAGGAAACATAACATGTACATCAAAT---
ATCACAGGACTACTATTGACACGTGATGGAGGTAATGAAAATGAAAATGAAACC-------------------
----

AACAAAAATGAGACATTCAGACCTGCAGGAGGAGATATGAGGGACAATTGGAGAAGTGAATTATATAAATATA
AAGTGGTGGAGATTAAGCCATTA---GGAATAGCACCCACTGAGGCAAAAAGGAGAGTGGTG-----------
----------------------------GAGAGAGAGAAAAGAGCA---GTAGGA---ATAGGAGCT------
---GTGTTCCTTGGGTTCTTGGGAGCA---GCAGGAAGCACTATG---GGCGCGGCGTCA---
ATAACGCTGACGGTACAG---GCCAGACAA---TTATTG---TCTGGTATAGTGCAACAGCAAAGC---
AATTTG---CTGAGA---GCTATAGAGGCGCAACAACATATGTTGCAACTCACAGTCTGGGGCATT
>KC154013
TTCTATTGCAATACATCAAAACTGTTTAATGAAACATACCTTAATGATACA----------------------
----

AAGAGTGAAACAATCATACTCCCATGCAGAATAAAACAGATTATAAACATGTGGCAGGAGGTAGGACGAGCAA
TGTATGCCCCTCCCATTGCAGGAAACATAACATGTAACTCAAGT---
ATCACAGGACTACTGTTGACACGGGATGGAGGAGAGACAAATGGCACA-------------------------
----

AATGGCACAGAAACGTTCAGACCTGGAGGAGGAGATATGAGGGACAATTGGAGAAGTGAATTATATAAATATA
AAGTGGTAGAAATTAATCCATTG---GGAATAGCACCCACTAAGGCAAAAAGGAGAGTGGTG-----------
----------------------------GAGAGAGAAAAAAGAGCA---GTGGGA---ATGGGAGCT------
---GTGTTCCTTGGGTTCTTGGGAGCA---GCAGGAAGCACTATG---GGCGCAGCGTCA---
CTAACGCTGACGGCACAG---GCCAGACAA---TTGTTG---TCTGGAATAGTGCAACAGCAAAGC---
AATTTG---CTGAGG---GCTATAGAGGCGCAACAGCATATGTTGCAACTCACGGTCTGGGGCATA
>KC154015
TTTTATTGTAATACATCAGGACTGTTTAATAGTACATACTGGGGTAGGACTAATAGTACTGATATTCTT----
----

TCAAATTCAACTATCACACTCCAATGCAGAATAAAACAAATTATAAACATGTGGCAGGAGGTAGGACGAGCAA
TGTATGCCCCTCCCATTGCAGGAAACATAACATGTAATTCAAGT---
ATCACAGGATTACTATTGACGCGTGATGGAGGAACATGGGAAAAC----------------------------
----

AAGACAGAAGAGATATTCAGACCTGGAGGAGGAGATATGAGGGACAATTGGAGAAGTGAATTATACAAATATA
AAGTGGTAGAAATTAAGCCATTA---GGAGTAGCACCCACTAAGGCAAAAAGGAGAGTGGTGGAG--------
-------------------------AAACAAATAGAAAAAAGAGCA---GTGGGA---CTAGGAGCT------
---GTGTTCTTTGGGTTCTTGGGAGCA---GCAGGAAGCACTATG---GGCGCAGCGTCA---
ATAACGCTGACGGTACAG---GCCAGAAAC---TTATTG---TCTGGCATAGTGCAACAGCAAAGC---
AATTTG---CTGAGA---GCTATAGAGGCGCAACAGCATATGTTGCAACTCACAGTCTGGGGCATT
>KF114882
TTCTATTGCAATACATCAAAGCTGTTTAATGAAATAGACACAAATAAAACAGGAAAC----------------
----

GAAAGTGAAAACATCACACTCCCATGCAGAATAAAACAAATTATAAACATGTGGCAGGAGGTAGGACGAGCAA
TGTATGCCCCTCCCATTGCAGGAAGCATAACATGTAAATCAAAT---
ATCACAGGACTACTATTGCTACGTGATGGAGGCACAAATACAAGC----------------------------
----

GACAACAACAGTGAGATATTCAGACCTGGAGGAGGAAATATGAAGGATAATTGGAGAAGTGAATTATATAAAT
ATAAAGTGGTAGAAATTAAGCCATTG---GGAATAGCACCAACTGAGGCAAAAAGGAGAGTGGTG--------
-------------------------------GAAAGAGAAAAAAGAGCA---GTGGGA---ATAGGAGCT---
------GTACTCCTTGGGTTCTTGGGAGCA---GCAGGAAGCACTATG---GGCGCAGCGTCA---
ATAACGCTGACGGTACAG---GCCAGACAG---CTGTTG---TCTGGTATAGTGCAGCAGCAAAGC---
AATTTG---CTGAGA---GCTATAGAGGCGCAACAGCATATGTTGCAACTCACAGTCTGGGGCATT

```
>KR861313
TTCTATTGCGATACATCAAAACTGTTTAATGACTCGTCTAATAGTACA-------------------------
-------------------------------------------------------------------------
----------------------------
AGCAAGGAGCCAATCACACTCCCATGCAGAATAAAACAAATTATAAACATGTGGCAGGAGGTAGGACGAGCAA
TATATGCCCCTCCCATTGCAGGAAACATAACATGCAGATCTAAT---
ATCACAGGACTACTATTGACACGTGATGGAGGGAGGGTCAATGACACA-------------------------
-------------------------------------------------------------------------
-------------
ACAGAGGAGATATTCAGACCTGGAGGAGGAGATATGAGGGACAATTGGAGAAGTGAATTATATAAATATAAAG
TAGTAGAAATTACACCATTG---GGAGTAGCACCCACTGGGGCAAAAAGGAGAGTGGTG--------------
-------------------------GAGAGAGAAAAAAGAGCA---GTTGGA---ATAGGAGCT---------
GTACTCCTTGGGTTCTTGGGAGCA---GCAGGAAGCACTATG---GGCGCAGCGTCG---
ATGACGCTGACGGTACAG---GCCAGACAA---TTGTTG---TCTGGAATAGTGCAACAGCAAAGC---
AATTTG---CTGAGA---GCTATAGAGGCGCAACAGCATATGTTGCAACTCACGGTCTGGGGCATT
>KR861315
TTCTATTGCAATACAACAAAATTGTTTAACAGTACATACAATTCTAATGGTACATACAGTAAT----------
-------------------------------------------------------------------------
----------------------------
AGTACTTCAAATATCACACTCCAATGCAGAATAAAACAAATTATAAACATGCGGCAGGAGGTAGGACGAGCAA
TGTATGCCCCTCCCATTGCAGGAAACATAACATGCGAATCAAAT---
ATCACAGGACTACTATTGACACGTGATGGAGGAAATGGGTCCAGT----------------------------
-------------------------------------------------------------------------
----------
GATGCCCCAGAGATATTCAGACCAGGAGGAGGAAATATGAAGGACAATTGGAGAAGTGAACTATATAAATATA
AAGTGGTAGAAATTAAACCATTA---GGAGTAGCACCCACTACGGCAAAAAGAAGAGTGGTG-----------
----------------------------GAGAGAGAAAAAAGAGCA---GTGGGA---ATAGGAGCT------
---GTGTTCCTTGGGTTCTTGGGAGCA---GCAGGAAGCACTATG---GGCGCAGCATCA---
ATAACGCTGACGGTACAG---GCCAGACAA---TTGTTG---TCTGGGATAGTGCAACAGCAAAGC---
AATTTG---CTGAAG---GCTATAGAGGCGCAACAGCATCTGCTGCAACTCACGGTCTGGGGCATT
>KR861316
TTCTATTGCAATACATCACAACTGTTTAATAAGACAATGAACAATATTAATGGTACAGAAAACAGTACAGACA
CA-----------------------------------------------------------------------
----------------------------
GAAAACAGGACCATCACAATCCCATGCAGAATAAAACAAATTATAAACATGTGGCAGAGGGTGGGACGAGCAA
TGTATGCTCCTCCCATTGCAGGAAACATAACATGTGAATCAAAT---
ATCACAGGAATACTATTGACACGTGATGGAGGAGAATCAAATAAC----------------------------
-------------------------------------------------------------------------
-------------
ACCAATGAGACATTCAGACCTGGAGGAGGAGATATGAGGGATAACTGGAGAAGTGAATTATATAAATATAAAG
TGGTAGAAATTAAGCCATTG---GGAGTAGCACCCACTGAGGCAAGAAGGAGAGTGGTG--------------
-------------------------GAGAGAGAAAAAAGAGCA---GTGGGA---ATGGGAGCT---------
GTGTTACTTGGGTTCTTGGCAACA---GCAGGAAGCACTATG---GGCGCGGCGTCA---
GTGACGCTGACGGTACAG---GCCAGACAA---TTGTTG---TCTGGTATAGTGCAACAGCAAAGC---
AATTTG---CTGAAG---GCTATAGAGGCGCAACAGCATCTGTTGCAACTCACAGTCTGGGGCATT
>KR861325
TTCTATTGCAATACATCAAGTCTGTTTAATGAGACATACCTATATAATGGTACAGCAGAC-------------
-------------------------------------------------------------------------
----------------------------
AATACAGTAAACATTACACTCCCATGCAAAATAAAACAAATTATAAACATGTGGCAGGGGGTAGGACGAGCGA
TGTATGCCCCCCCCATTGCAGGAAACATAACATGTAAATCAAAT---
ATCACAGGATTACTATTGGTACGTGATGGGGGGAAAGGAAATAAT----------------------------
-------------------------------------------------------------------------
-------------
GACACTGAGACATTCAGACCTGGAGGAGGAAATATGAAGGACAATTGGAGAAGTGAATTATATAAATATAAGG
TAGTAGAAATTAAACCATTA---GGAGTAGCACCCACCAAGGCAAAAAGGAGAGTGGTG--------------
-------------------------GAGAGAGAAAAACGAGCA---GCGGGA---CTAGGAGCT---------
GTGTTCCTTGGGTTCTTGGGAGCG---GCAGGAAGCACTATG---GGCGCAGCATCA---
ATAGTGCTGACGGTACAG---GCCAGACAA---TTGTTG---TCTGGTATAGTGCAACAGCAAAAC---
AATTTG---CTGAGA---GCTATAGAGGCGCAACAACATCTGTTGCAACTCACAGTCTGGGGCATT
```

>KR861326
TTCTATTGCGATACATCACAACTGTTTAATGAGTCCCTGTTTAATAGTACA----------------------
----

AACAATGATACAATCATAGTTCCATGCAAAATAAAACAAATTATAAACATGTGGCAGGGGGTAGGACGAGCAA
TGTATGCCCCTCCCATTGCAGGGAACATTACATGTAACTCTAGT---
ATCACAGGACTACTATTGACATGGGATGGAGGGATAAATAATAGG----------------------------
----

ACTACAGAGATATTCAGACCTGGAGGAGGAAATATGAAGGATAATTGGAGAAATGAATTATATAAATATAAAG
TGGTAGAAATTAGGCCATTG---GGAATAGCACCCACTGGAGCAAAAAGGAGAGTGGTG--------------
-------------------------GAGAGAGAAAAAAGAGCAGTAATGGGA---TTAGGAGCT---------
GTGTTCCTTGGGATCTTGGGAGCA---GCAGGAAGCACTATG---GGCGCAGCGTCA---
ATAACGCTGACGGTACAG---GCCAGGCAA---TTGTTG---TCTGGAATAGTGCAGCAGCAAAAC---
AATTTG---CTGAGA---GCTATAGAGGCGCAACAGCATATGTTGCAACTCACAGTCTGGGGCATT
>KR861337
TTCTATTGTAATACATCAGACCTGTTTAATGGTACATACAATGGTACATACAATGATATAGATCTG-------
----

AATAATGATACACTCACACTCACATGCAGAATAAAACAAATTATAAACATGTGGCAGGAGGTAGGACGAGCAA
TGTATGCCCCTCCCATTGAAGGAAACATAACATGCACATCAAAT---
ATCACAGGACTACTGCTACTGCGGGATGGAGGAATTGGAAATGAG----------------------------
----

ACAGAAGAGATATTCAGACCTGGAGGAGGAGATATGAGAGACAATTGGAGAAGTGAATTATATAAATATAAAG
TAGTAGAAATTAAGCCATTG---GGAGTAGCACCCACTAAGGCAAAAAGGAGAGTGGTG--------------
-------------------------GAGAGAGAAAAAAGAGCA---GTGGGA---ATTGGAGCT---------
GTACTCCTTGGGTTCTTGGGAGCA---GCAGGAAGCACTATG---GGCGCGGCGTCA---
ATAACGCTGACGGTACAA---GCCAGACAA---TTGTTG---TCTGGTATAGTGCAACAGCAAAGC---
AATTTG---CTGAGG---GCTATAGAGGCGCAACAGCATCTGTTGCAACTCACGGTCTGGGGCATT
>KR861345
TTCTATTGCAATACAACAAAACTGTTTGACACAGCAACACTGGTTAATGGTACATACATGAACAGTACAGAA-
----

AGTAATTCAACCATCACAATCAAATGCAGAATAAAACAATTTATAAACATGTGGCAGGGGGTAGGACGAGCAA
TGTATGCCCCTCCCATTGCAGGAAATATAACATGTATATCAAAT---
ATCACAGGATTAATATTGACACGTGATGGAGGACCAAATAGTAGTGATAAT----------------------
----
GTGACTACAACTGGAAATAATACTAAT---------
ATTACTGAGATATTCAGACCTGCAGGAGGAGACATGAGGGACAATTGGAGAAGTGAACTATATAAATATAAAG
TAGTAGAAATTAAGCCATTA---GGACTAGCACCCACCAAGGCAAAAAGGAGAGTGGTG--------------
-------------------------GAGAGAGAAAAAAGAGCG---GTGGGA---ATAGGAGCT---------
GTGTTCCTTGGGTTCTTGGGAGCA---GCAGGAAGCACTATG---GGCGCGGCGTCA---
ATAACGCTGACGGTACAG---ACCAGACAA---CTGTTG---TCTGGTATAGTGCAACAGCAAAGT---
AATTTG---CTGAGA---GCTATAGAGGCGCAACAGCATATGTTGCAACTCACGGTCTGGGGCATT
>KR861312
TTCTATTGCAATACATCAAAACTGTTTAATGGTACATACCCGCAGCTTAATAGGACAGAAGAT----------
----

GTAAATCGAACCATCACACTTAAATGCAGAATAAAACAAATTATAAACATGTGGCAGGGGGTAGGACGAGCAA
TGTATGCCCCTCCCATTGCAGGAAATATAACATGTATATCAAAT---
ATCACAGGATTAATATTGACACGTGATGGAAAATATGAAAGTAGT----------------------------
----
AAGAATGAAAGTAGT---
AAGAATGAGACATTCAGACCTGCAGGAGGAGATATGAGGGACAATTGGAGGAGTGAATTATATAAATATAAAG
TAGTAGAAATTAAGCCATTA---GGACTAGCACCCACCAAGGCAAAAAGGAGAGTGGTG--------------
-------------------------GAGAGAGAAAAAAGAGCA---GTGGGA---ATAGGAGCT---------
GTGTTCCTTGGGTTCTTGGGAGCA---GCAGGAAGCACTATG---GGCGCAGCGTCA---
ATAACGCTGACGGTACAG---GCCAGACAA---CTGTTG---TCTGGTATAGTGCAACAGCAAAGC---
AATTTG---CTGAGG---GCTATAGAGGCGCAACAGCATATGTTGCAACTCACAGTCTGGGGCATT

>KR861322
TTTTATTGTAATACATCAAAATTGTTTAATGAGAGCCATGTGTATAATAAT----------------------
----

ACAAATGGAACCATCACACTCCCATGCAGAATAAAACAAATTATAAACATGTGGCAAGGGGTAGGAAGAGCAA
TGTATGCCCCTCCCATTGCAGGAAACATAACATGTATATCAAAT---
ATCACAGGACTGCTATTGACACGTGATGGAGGAGAGAATAATGGG----------------------------
----

TCAGAAGAAGTATTCAGACCTGGAGGAGGAGATATGAGAGACAATTGGAGAAGTGAATTATATAAATATAAAG
TGGTAGAAGTTAAACCATTA---GGAATAGCACCCACTGAGGCAAGAAGGAGAGTGGTG--------------
-------------------------GAGAGAGAAAAAAGAGCA---GTGGGA---ATAGGAGCT---------
GTGTTCCTTGGGTTCTTGGGAGCA---GCAGGAAGCACTATG---GGCGCGGCGTCA---
ATAACGCTGACGGTACAG---GCCAGACAG---TTATTG---TCTGGTATAGTGCAACAGCAAAGC---
AATTTG---CTGAGG---GCTATAGAGGCGCAACAGCATATGTTGCAACTCACGGTCTGGGGCATT
>KR861324
TTCTATTGTAACACATCAGGCCTGTTTAATAGCACCATCCGGCCTAATGGCACTATCCAGTTTAATAGCACTA
ATGCA--

ACAGCCCCAAACATCACACTTCCATGCAGAATAAAACAATTTATAAACATGTGGCAAGGGGTAGGACGAGCAA
TGTATGCCCCCCCCATTGCAGGAAATATAATGTGTAGATCAAAT---
ATCACAGGACTACTATTGACACGTGATGGAGGTGGCAATGAGACT----------------------------
----

GATACTGAGATATTCAGACCTGAGGGAGGAGATATGAGGAACAATTGGAGAAGTGAATTATATAAATATAAAG
TGGTGGAGATCCAGCCATTA---GGAGTAGCACCCACTGAGGCAAAAAGGAGAGTGGTG--------------
-------------------------GAGAGAGAGAAAAGAGCA---GTGGGA---ATAGGAGCT---------
GTGTTCCTTGGGTTCTTAGGAGCA---GCAGGAAGCACTATG---GGCGCAGCATCA---
ATAACGCTGACGGTACAA---GCCAGACAA---TTATTG---TCTGGTATAGTGCAACAGCAAAGC---
AATTTG---CTGAGG---GCTATAGAGGCGCAACAACATATGTTGCAACTCACAGTCTGGGGCATT
>KR861330
TTCTATTGCAATACATCAAATCTGTTTAATAAGACATACTGTCCTAATGATACAAAGAGTAATGAGACAAAG-
----

AGTAATGAAACCATCACACTGCAATGCAGAATAAAACAAATTATAAACAGGTGGCAGGAGGTAGGACGAGCAA
TGTATGCCCCTCCTATTGAAGGAAACATAACATGTAAATCAAAT---
ATCACAGGACTACTGTTGGTACGTGATGGAGTTAACACAACTGAG----------------------------
----

GAGATATTCAGACCTGGAGGAGGAAATATGAAAGACAATTGGAGAAGTGAATTATATAAATACAAAGTAGTAG
AAATTAAGCCATTG---GGAATAGCACCCACTGCAGCAAAAAGGAGAGTGGTG--------------------
-------------------GAGAGAGAAAAAAGAGCA---GCA------CTGGGAGCT---------
ATGTTCCTTGGGTTCTTGGGATTA---GCAGGAAGCACTATG---GGCGCAGCGTCA---
GTGACGCTGACGGTACAG---GCCAGACAA---CTGTTG---TCTGGTATAGTGCAACAGCAAAAC---
AATTTG---CTGAGA---GCTATAGAGGCGCAACAGCATATGTTGCAACTCACGGTCTGGGGCATT
>KR861335
TTCTATTGCAATACAACAAAACTGTTCAATACAACAATACCGCCTAATGAGACATACAGGAGTACAAAC----
----

AATTCAAGTACCATCACACTCAAATGCAGAATAAAACAAATTATAAATATGTGGCAGGGGGTAGGACGAGCAA
TGTATGCCCCTCCCATTGCAGGAAACATAACATGTATATCAAAT---
ATCACAGGATTAATATTGACACGTGATGGAGGAAACAATAATAAT----------------------------
----

GAGACTGAGACATTCAGACCTGCAGGAGGAAATATGAAGGACAATTGGAGAAGTGAATTATATAAATATAAAG
TAGTAGAAATTAAGCCATTA---GGACTAGCACCCAACAAGGCAAAAAGGAGAGTGGTG--------------
-------------------------GAGAGAGAAAAAAGAGCA---GTGGGA---ATAGGAGCT---------
GTGTTCCTTGGGTTCTTGGGAGCA---GCAGGAAGCACTATG---GGCGCGGCGTCA---
ATAACGCTGACGGTACAG---GCCAGACAA---CTGTTG---TCTGGTATAGTGCAACAGCAAAGT---
AATTTG---CTGAGA---GCTATAGAGGCGCAACAGCATATGTTGCAACTCACGGTCTGGGGCATT

>KR861336
TTTTATTGCGATACATCAAGATTGTTTAATGATACACACTGGAATGACACAGAAGATAATATA----------
----

GCTAATAGTAACACCATCATACCATGCAAAATAAGACAAATTATAAACATGTGGCAGGAAGTAGGACGAGCAA
TATATGCCCCTCCCATTGAAGGAAACATAACATGTAAATCACGT---
ATCACAGGATTACTATTGGAACGTGATGGAGGAAAAAAAAATGAC----------------------------
----

CCAAAAAAAATAGAAATATTCAGACCTGGAGGAGGAGATATGAGGAACAATTGGAGAAATGAATTATATAAAT
ATAAAGTGGTAGAAATTAAGCCATTG---GGAGTAGCACCCACTGAGGCAAAAAGGAGAGTGGTG--------
-------------------------------GAGAGAGAAAAAAGAGCA---GTGGGC---ATAGGAGCT---
------GTGCTCCTTGGGTTCTTGGGAGCA---GCAGGAAGCACTATG---GGCGCAGCATCA---
ATAACGCTGACGGTACAG---GCCAGACAA---TTGTTG---TCTGGAATAGTGCAACAGCAAAAC---
AATTTG---CTGAGG---GCTATAGAGGCGCAACAGCATATGTTGCAACTCACAGTCTGGGGCATT
>KR861343
TTCTATTGCAATACATCAAACCTATTTCCAGGAAATGAGACGCAAGGCACAGGG-------------------
----

ACAAACAGCACAATAACAATACAATGCAGAATAAAACAAATTATAAACATGTGGCAGGAGGTAGGACGAGCAA
TATATGCCCCTCCCATTGAAGGAAACATAACATGTATATCAAAG---
ATCACAGGACTAATACTGACACGTGATGGAGGAAACAGTGGAAACAAT-------------------------
----

ACAAAGCAGCCAGAGATATTCAGACCTGGAGGAGGAGATATGAGAGACAATTGGAGAAGTGAACTATATAAAT
ATAAAGTGGTAGAAATTAAGCCATTG---GGAGTAGCACCCACTGGGGCAAAAAGGAGAGTGGTG--------
-------------------------------GAGAGAGAAAAAAGAGCA---GTGGGA---ATAGGAGCT---
------GTGCTCCTTGGGTTCTTGGGAGCA---GCAGGAAGCACTATG---GGCGCAGCATCA---
ATAACGCTGACGGTACAG---GCCAGACAA---TTGTTG---TCTGGAATAGTGCAACAGCAAAGC---
AATTTG---CTGAGG---GCTATAGAGGCGCAACAACATATGTTGCAACTCACGGTCTGGGGCATT
>KR861311
TTCTATTGTAATACATCAAAACTGTTTAATAGGACATATGAGGTTAATGATACAGGTAGGACTAATGATACTA
GT---

GATAATTCAACCATCATACTCCCATGCAGAATAAAACAAATTATAAACTTGTGGCAGGGAGTAGGACGAGCAA
TATATGCCCCGCCCATTGCAGGAAACATAACATGTACTTCAAAT---
ATCACAGGAATGCTATTGACACGGGATGGAGGAAACAAAAACAATACA-------------------------
----

GAGCCAGAGACATTCAGACCTGGAGGAGGAGATATGAGGGACAATTGGAGAAATGAATTATATAAATATAAGG
TGGTAGAAATTAAGCCATTA---GGAATAGCACCCACTAGGGCAAAAAGAAGAGTGGTG--------------
-------------------------GAGAGAGAAAAAAGAGGA------------ATAGGAGCT---------
GTGTTCCTTGGGTTCTTGGGAGCA---GCAGGAAGCACTATG---GGCGCGGCATCA---
GTGATGCTGACGGTACAA---GCCAGACAA---TTATTG---TCTGGTATAGTGCAACAGCAAAGC---
AATTTG---CTGAGG---GCTATAGAGGCGCAACAGCATATGTTGCAACTCACAGTCTGGGGCATT
>KR861327
TTCTATTGCAATACAACAAAGCTGTTTAATAGTACTGATAAAAGT----------------------------
----

GATAATGGTACCATCACACTCCCATGCAAAATAAAACAAATTATAAACATGTGGCAGGGGGTAGGAAGAGCAA
TGTATGCCCCTCCCATTGCAGGAAACATAACATGTAACTCAAGT---
ATCACAGGACTAATATTGACACGGGATGGAGGAAACGATACAAACACT-------------------------
----

AATAACACAGAGATATTCAGACCTGGAGGAGGAAATATGAAGGACAATTGGAGAAATGAATTATATAAATATA
AAGTGGTAGAAATTAAGCCATTG---GGAATAGCACCCACCCAGGCAAAAAGGAGAGTGGTG-----------
----------------------------GAGAGAGAGAAAAGAGCA---GTGGGA---ATAGGAGCT------
---GTGTTCCTTGGGTTCTTGAGTGCA---GCAGGAAGCACTATG---GGCGCGGCGTCA---
ATAACGCTGACGGTACAG---GCCAGACAA---CTGTTG---TCTGGTATAGTGCAACAGCAAAGT---
AATTTG---CTGAGG---GCTATAGAGGCGCAACAGCATATGTTGCAACTCACGGTCTGGGGCATT

>KR861344
TTCTATTGCAATACAACAAAACTATTTAGTGGGGTTAATGATACA----------------------------
----

GACAATAGGACAATAATACTCCCATGCAGAATAAAACAAATTATAAACATGTGGCAGGGGGTAGGACGAGCAA
TATATGCCCCTCCCATTGCAGGAAACATAACATGTAAATCTAAT---
ATCACAGGACTACTATTGGTACGAGATGGAGGTGATACCAATGCAACA-------------------------
----

GAGATAGAGATATTCAGGCCTGGAGGAGGAGATATGAGAGACAATTGGAGAAGTGAATTATATAAATATAAGG
TGGTAGAAATCAAGCCATTG---GGAATAGCACCCACTGAGTCAAAAAGGAGAGTGGTG--------------
-------------------------GAGAGAGAAAAGAGAGCAGTATTGGGAGGAATAGGAGCT---------
GTGTTCCTTGGGTTCTTGGGAGCA---GCAGGAAGCACTATG---GGCGCGGCGTCA---
CTGACGCTGACGGTACAG---GCCAGGCAA---CTGTTG---TCTGGTATAGTGCAGCAGCAAAGC---
AATTTG---CTGAAG---GCTATAGAGGCGCAACAGCATCTGTTGCAACTCACAGTCTGGGGCATT
>KR861279
TTCTATTGCAATACATCAAATCTGTTTAATGGAAAGCTATTTAATGATACATACAACAAGACAGGA-------
----

GATACTTCAAACATCACACTCTCATGCAGAATAAAACAAATTATAAACATGTGGCAGGAGGTAGGACGAGCAA
TGTATGCCCCTCCCATTGCAGGAAACATAACATGTATATCAAAT---
ATCACAGGAATAATATTGACACGTGATGGAGGAATTAATAACACTACTAAC----------------------
----
AACACTGAGAAAAAT---
GGCGCAGAGACATTCAGACCTGGAGGAGGAAATATGAAGGACAATTGGAGAAGTGAATTATATAAATATAAAG
TGGTAGAAATTAAGCCATTA---GGACTAGCACCCACTGCGGCAAAAAGGAGAGTGGTG--------------
-------------------------GAGAGAGAAAAAAGAGCA---GTGGGA---ATAGGAGCT---------
GTTTTCCTTGGGTTCTTGGGAGCA---GCAGGAAGCACTATG---GGCGCGGCGTCA---
ATAACGCTGACGGTACAG---GCCAGACAA---CTGTTG---TCTGGTATAGTGCAACAGCAAAGT---
AATTTG---CTGAGA---GCTATAGAGGCGCAACAGCATATGTTGCAACTCACGGTCTGGGGCATT
>KR861271
TTCTATTGCAATACAACAAACCTGTTTAATGGAACATATGGGGATAAAGATACAAAC----------------
----

AGTACTTCAAACATCACACTCTCATGCAAAATAAAACAAATTATAAACACATGGCAGGAGGTAGGAAAGGCAA
TATACGCCCCTCCCATTGCAGGAAGCATAAACTGCACATCACAG---
ATCACAGGACTACTCTTGGTACGTGATGGAGGGGGAAATAGCACA----------------------------
----

GATAATGACACATTCAGACCTGAAGGGGGAGATATGAGAGACAATTGGAGAAGTGAATTATATAAATATAAAG
TGGTAGAAATTAAACCATTG---GGAATAGCACCCACCAAGGCAAAAAGGAGAGTGGTG--------------
-------------------------GAGAAAGAAAAAAGAGCA---GTCGGA---ATGGGAGCT---------
GTGATTCTTGGGTTCTTGGGAGCA---GCTGGAAGCACTATG---GGCGCGGCGTCA---
ATAACGCTGACGGCACAG---GCCAGACAA---GTATTG---TCTGGTATAGTGCAACAGCAAAGC---
AATTTG---CTGAAG---GCTATAGAGGCGCAACAGCGTATGTTGCAACTCACGGTCTGGGGCATT
>KR861282
TTCTATTGCGACACATCAAAACTGTTTAATAGTACATACAACGATCTATTTAATAAAACATACAATGATACAG
CT---

AATAGATTAAACATCACAATTCCATGCAGAATAAAACAAATTATAAACATGTGGCAGAAGGTAGGACGAGCAA
TGTATGCTCCTCCCATTGCAGGAAACATAACATGTGAATCCAAT---
ATCACAGGACTGCTACTGGTACGTGATGGAGGTAGAAGTAACAAT----------------------------
----

AACAATGGCACAGAGACATTTAGGCCTGGAGGAGGAAATATGAAGGACAATTGGAGAAGTGAATTATATAAAT
ATAAAGTGGTAGAAATTAAACCATTA---GGAATAGCACCCACTGAAGCAAAAAGGAGAGTGGTG--------
-------------------------------GAGAGAGAAAAAAGAGCA---GTGGGA---ATGGGAGCT---
------GTGTTCCTTGGGTTCTTGGGAGCA---GCAGGAAGCACTATG---GGCGCGGCGTCA---
GTGGCGCTGACGGTACAG---GTCAGACAA---TTGTTG---TCTGGTATAGTGCAACAGCAAAAC---
AATTTG---CTGAGG---GCTATAGAGGCGCAACAGCATATGTTGCAACTCACAGTCTGGGGCATT

>KR861304
TTCTATTGCAATACATCATGCCTGTTTAATGATAGTTGTACAAAAAATGAATCAGTC----------------
----

ATCACACTCCCATGCAGAATAAAACAAATTACAAACATGTGGCAGGAAGTAGGACGAGCAATATATGCCCCTC
CCATTGCAGGAAACATAACATGTAATTCAAGT---
ATCACAGGACTACTCTTAACACGTGATGGAGGACGGTCAAGTGAA----------------------------
----
CCAAATAACACAAAT---
AACAATGAGACATTCAGACCTGCAGGAGGTGATATGAGGGACAATTGGAGAAGTGAATTATATAAATATAAAG
TGGTAGAAGTTAAGCCATTG---GGAATAGCACCCACTAACGCAAAAAGGAGAGTGGTG--------------
-------------------------GAGAGAGAAAAAAGAGCA---GTGGGA---ATAGGAGCT---------
GTGTTCCTTGGGTTCTTGGGAGCA---GCAGGAAGCACTATG---GGCGCAGCATCA---
ATAACGCTGACGGTACAA---GCCAGACAA---CTGTTG---TCTGGTATAGTGCAACAGCAAAGC---
AATTTG---CTGAGA---GCTATAGAGGCGCAACAACATATGTTGCAACTCACAGTCTGGGGCATT
>AF110959
TTTTATTGCGATACATCAGAACTGTTTAATAGTACATACATGTCTAATGGTACAGAAGGTAATATAAGC----
----

CCAAGTACAATCATCATGCTCCCATGCAGAATAAAACAAATTATAAACATGTGGCAGGGGGTGGGACGAGCAA
TGTATGCCCCTCCCATTAAAGGAAGTATAACATGTAGATCAAAT---
ATTACAGGGTTACTATTGACACGTGATGGAGGGCTAAATAGGAGCACA-------------------------
----

GAGGAGCCAGAGATATTCAGACCTGGAGGAGGAGATATGAGGAACAATTGGAGAAGTGAATTATATAAATATA
AAGTGGTAGGAATTAAGCCATTA---GGAGTAGCACCCACTGGGGCAAAAAGGAGAGTGGTG-----------
----------------------------GAGAGAGAAAAAAGAGCA---GTGGGA---ATAGGAGCT------
---GTGTTCCTTGGGTCCTTGGGAGCA---GCAGGAAGCACTATG---GGCGCGGCGTCA---
ATCACGCTGACGGTACAG---GCCAGACAA---TTATTG---TCTGGTATAGTGCAACAGCAAAGC---
AATTTG---CTGAGG---GCCATAGAGGCGCAACAGCACATGTTGCAACTCACGGTCTGGGGCATT
>AF290028
TTTTATTGCAATACATCAAGACTGTTTGATGAGACATACCTGTCTAATGGGACAGACGAG-------------
----

GATAATGGTACAATCACACTCCCATGCAAAATAAAACAAATTATAAACATGTGGCAAGAGGTAGGACGAGCAA
TATATGCCCCTCCCATTGCAGGAAACATAACATGTAAATCAAAT---
ATCACAGGACTATTATTGACACGTGATGGAGGGTTAAGCCCTAAT----------------------------
----

AATGACAGTGAGACATTCAGACCTATAGGAGGAGAGATGAGGAACAATTGGAGAAGTGAATTATATAAATATA
AAGTGGTAGAAATTAAGCCATTG---GGAATAGCACCCACTAAGGCAAAAAGGAGAGTGGTG-----------
----------------------------GAGAGAGAAAAAAGAGCA---GTGGGA---ATAGGAGCT------
---GTGTTCCTTGGGTTCTTGGGAGCA---GCAGGAAGCACTATG---GGCGCAGCATCA---
ATAACGCTGACGGTACAG---GCCAAACAA---TTGTTG---TCTGGAATAGTGCAACAGCAAAGC---
AATTTG---CTGAAA---GCTATAGAGGCGCAACAGCATATGTTGCAACTCACGGTCTGGGGCATT
>AF443075
TTCTATTGCAATACATCAGAACTGTTTAATGGAACATATAATGGAACAGACAACAAT----------------
----

AGCAATAAAACCATCACACTCCTATGCAGGATAAAACAAATTATAAACACATGGCAGGAGGTAGGACGGGCAA
TATACGCCCCTCCCATTGCAGGAAACATAATATGTATATCAAAC---
ATCACAGGACTACTCTTGACACGTGATGGAGGAAAAACAAATGACACA-------------------------
----

AATGACACAGAAATATTCAGACCTGGAGGAGGAAATATGAAGGACAATTGGAGAAGTGAATTATATAAATATA
AAGTGGTAGAAATTAAACCATTG---GGAGTAGCACCCACCAAGGCAAAAAGGCGAGTGGTG-----------
----------------------------GAGAGAGAAAAAAGAGCA---GTCGGA---ATAGGAGCT------
---GTGCTCCTTGGGTTCTTGGGAACA---GCAGGAAGCACTATG---GGCGCGGCGTCA---
ATAACGCTGACGGTACAG---GCCAGACAA---GTATTG---TCTGGTATAGTGCAACAGCAAAGC---
AATTTG---CTGAGG---GCTATAGAGGCGCAACAGCATATGTTGCAACTCACGGTCTGGGGCATT

>AF443074
ATCTATTGTAATACATCAAAGCTGTTTAATGGTACATACAACAGTACAGGAGGTACT----------------
----

TCAAATTCAACAATCACACTCTCATGCAGAATAAAACAAATTATAAACATGTGGCAGGGGGTAGGACGAGCAA
TGTATGCCTCTCCCATTGCAGGAAACATAACATGTAAATCAAAT---
ATCACAGGACTACTATTGACACGTGATGGAGGAAATGAGACAAGT----------------------------
----

GGGATTGAGATATTCAGACCTGCAGGAGGAGATATGAGGGACAATTGGAGAAGTGAATTATATAAATATAAAG
TGGTAGAAATTAAGCCATTA---GGATTAGCACCCACTAAGTCAAAACGGAGAGTGGTG--------------
-------------------------GGGAGAGAAAAAAGAGCA---GTGACA---TTCGGAGCT---------
ATGTTCCTTGGGTTCTTGGGAGCA---GCAGGAAGCACTATG---GGCGCGGCGTCC---
ATGACGCTGACGGTACAG---GCCAGACAA---CTGTTG---TCTGGTATAGTGCAACAGCAAAGC---
AATTTG---CTGAGG---GCTATAGAGGCGCAACAGCATATGTTGCAACTCACGATCTGGGGCATT
>KY658709
TTCTATTGCAATACATCAAAACTGTTTAATGGTACATATAACAGTACAGGAAGTACT----------------
----

TCAAATTCAACCATCACACTCCCATGCAGAATAAAACAATTTATAAACTTGTGGCAGGAGGTAGGACGAGCAA
TGTATGCCCCTCCCATTGCAGGAAACATAACATGTAGATCAAAT---
ATCACAGGACTAATATTGACACGTGATGGAGGAGTTGGTAATGAC----------------------------
----

TCAGAGACATTCAGACCTGAAGGAGGAGATATGAGGAACAATTGGAGAAGTGAATTATATAAATACAAAGTGG
TAGAAATTAAGCCATTA---GGACTAGCACCCACTAAGGCAAAAGGAGAGTGGTG-----------------
----------------------GAGAGAGAAAAAAGAGCA---GTGGGA---ATAGGAGCT---------
GTGTTCCTTGGGTTCTTGGGAGCA---GCAGGAAGCACTATG---GGCGCGGCGTCA---
ATAACGCTGACGGTACAG---GCTAGACAA---CTGTTG---TCTGGTATAGTGCAACAGCAAAGC---
AATTTG---CTGAGG---GCTATAGAGGCGCAACAACATATGTTGCAACTCACGGTCTGGGGCATT
>KY658711
TTCTATTGCAATACATCAAAACTGTTTAATAGTACATACAATAGTACAGACAGGTCTAAT-------------
----

AATACAGACAACATCATAATCCAATGCAGAATAAAACAAATTATAAACATGTGGCAGGGGGTAGGACGAGCAA
TGTATGCCCCTCCCATTGAAGGAAACATAACATGTAAATCAAAT---
ATCACAGGACTACTATTGGTACGTGATGGAGGGAAAGAAGAGAATAACACA----------------------
----

GGGACAGAGATATTCAGACCTGAAGGAGGAGATATGAGGGATAACTGGAGAAGTGAATTATATAAATATAAAG
TGGTAGAAATTAAGCCATTG---GGAATAGCACCCACTAAGGCAAAAGGAGAGTGGTG--------------
-------------------------GAGAGAGAAAAAAGAGCA---GTGGGA---ATAGGAGCT---------
GTGTTCCTTGGGTTCTTGGGAGCA---GCAGGAAGCACTATG---GGCGCGGCGTCA---
ATAACGCTGACGGTACAG---GCCAGACAA---TTGTTG---TCTGGTATAGTGCAACAGCAAAGC---
AATTTG---CTGAAA---GCTATAGAGGCGCAACAACATCTGTTGCAACTCACGGTCTGGGGCATT
>AF443076
TTCTATTGCAATACATCAGGCTTGTTTAATAGTACATACAATCCTAATAGTACATACAATCTTAATAGTACAG
AAAGT--

AAGGCAAATTCAAACATCACACTCCACTGCAGAATAAAACAAATTATAAACATGTGGCAGGAGGTAGGACGAG
CAATGTATGCCCCTCCCATTGCAGGAAACATAACATGTAGATCAAAT---
ATCACAGGATTACTATTGACACGTGATGGAGGAAATACAACAGAG----------------------------
----

ACAAAAGAGACATTCAGACCTGGAGGAGGGGATATGAGGGACAATTGGAGAAGTGAATTATATAAATATAAAG
TGGTAGAAATTAAACCATTG---GGAGTAGCACCCACTGAGGCGAAAGGAGAGTGGTG--------------
-------------------------GAGAGAGGAAAAAGAGCA---GCGGGA---CTAGGAGCT---------
GTGCTCTTTGGGTTCTTGGGAGCA---GCAGGAAGCACTATG---GGCGCAGCGTCA---
ATAACGCTGACGGTACAG---GCCAGACAA---CTGTTG---TCTGGTATAGTGCAACAGCAAAGC---
AATTTG---CTGAGG---GCCATAGAGGCGCAACAGCATATGTTGCAACTCACGGTCTGGGGCATT

>AF443078
TTCTATTGCAATACATCACAACTGTTTAATAGTACATACAATGGTACAGAAAGAAATTCAACA----------
----

ACAAATGCAACCATTACACTCCCATGCAGAATAAAACAAATCATAAACATGTGGCAGGAGGTAGGACGAGCAA
TATATGCCCCTCCCATTAAAGGAAACATAACATGTGAATCAAAT---
ATCACAGGACTACTATTGACACGTGATGGAGGGTCGAATGATACA----------------------------
----

ACAGAAACATTTAGACCTGAGGGAGGAGATATGAGGAACAATTGGAGAAGTGAATTGTATAAATATAAAGTAG
TAGAAATTAGGCCATTA---GGGATAGCACCCACTGGGGCAAAAAGGAGAGTGGTG-----------------
----------------------AATAGAGAAAAAAGAGCA---GTGGGA---GTAGCAGCT---------
GTGTTCCTTGGGTTCTTATCAGCA---GCAGGAAGCACTATG---GGCGCAGCATCA---
ATAACGCTGACGGTACAG---GCCAGACAA---TCGTTG---TCTGGTATAGTGCAACAGCAAAGC---
AATTTG---CTGAGA---GCTATAGAGGCGCAACAGCATCTGTTGCAACTCACGGTCTGGGGCATT
>AF443079
TTCTATTGCAATACATCAAAACTGTTTAATAGTACATACAATGCTACATACAATAGTACATACAATGATACA-
----

TCAAATTCAACCATCACAATCCCATGCAGAATAAAACAAATTATAAACATGTGGCAGGGGGTAGGACAAGCAA
TGTATGCCCCTCCCATTGCAGGAAACATAACATGTAAATCAAAT---
ATCACAGGAATACTGTTGACACGTGATGGAGGCATAAATAACACA----------------------------
----

AATGGCACAGAGACATTCAGACCTGGAGGAGGAGATATGAGGGACAATTGGAGAAGTGAATTATATAAATATA
AAGTGGTAGAAGTTAAACCATTA---GGGATAGCACCCACCAAGGCACAAAGGAGAGTGGTG-----------
----------------------------GAGAGAGAAAAAAGAGCA---GTGGGA---ATAGGAGCT------
---GTGTTCCTTGGGTTCTTGGGAGCA---GCAGGAAGCACTATG---GGCGCAGCGTCA---
ATAACGCTGACGGTACAG---GCCAGACAA---TTGTTG---TCTGGTATAGTGCAACAGCAAAGC---
AATTTG---CTGAGA---GCTATAGAGGCGCAACAGCATATGTTGCAACTCACAGTCTGGGGCATA
>AF443080
TTCTATTGTAATACATCAGGCCTGTTTAATAGTGCATTTAATGATAAT-------------------------
----

AGTGGTGGGACCATCACCCTCCAATGCAGAATAGAACAAATTATAAACATGTGGCAGAAGGTAGGACGAGCAA
TATATGCACCTCCCATTGCAGGAAACATAACATGTAGTTCAAGG---
ATCACAGGACTACTATTGACACGGGATGGTGGAAAAAATGATACA----------------------------
----

CATGAGACATTCAGACCTGCAGGAGGAGATATGAGGGACAATTGGAGAAGTGAATTATATAAATATAAAGTGG
TGGAGATTAAGCCATTA---GGAGTAGCACCCAGTGAGGCAAAAAGAAGAGTGGTG-----------------
----------------------GAGAGAGAGAAAAGAGCG---GCA------CTGGGAGCT---------
GTGTTCCTTGGGTTCTTGGGAGCA---GCAGGAAGCACTATG---GGCGCGGCATCA---
ATAACGCTGACGGTACAG---ACCAGAAAA---TTATTG---TCTGGTATAGTGCAACAGCAAAGT---
AATTTG---CTGAAA---GCTATAGAGGCGCAACAACATATGTTGCAACTCACAGTCTGGGGCATT
>AF443082
TTCTATTGCAATACATCAAAGCTGTTTAATACATCATGGCTGGATTCATACATATCTAATAATACAGGA----
----

AATAATTCAATCATCACACTTCCATGCAGAATAAAACAAATTATAAACATGTGGCAGAAGGTAGGACGAGCAA
TGTATGCCAACCCCATTGAAGGAAATATAACATGTAGGTCAAAT---
ATCACAGGATTACTATTAGAAAATGATGGA---AACATG----------------------------------
----

ACATTCAGACCTGGAGGAGGAGATATGAAGGACAATTGGAGAAGTGAATTATATAAATATAAAGTAGTAGAAA
TTAAGCCATTG---GGAATAGCACCCACTGAAGCAAAAAGGAGAGTGGTG-----------------------
----------------GAGAGAGAAAAAAGAGCA---GTGGGA---ATAGGAGCT---------
GTGTTCCTTGGGTTCTTGGGAGCA---GCAGGAAGCACTATG---GGCGCGGCGTCA---
ATAACGCTGATGGTACAG---GCCAGACAA---TTGTTG---TCTGGTATAGTGCAACAGCAAAGC---
AATTTG---CTGAGA---GCTATAGAGGCGCAACAGCATCTGTTGCAACTCACAGTCTGGGGCATT

>AF443083
TTCTATTGCAATACATCACGCCTGTTTAATAGTACATACAATCCTAATAGTACAAAAAGCAAT----------
----

ACAGGCTCATGGATCATACTCCCATGCAGAATAAAACAAATTATAAACATGTGGCAGAAGGTAGGACGAGCAA
TGTATGCTCCTCCCATTGCAGGAAACATAACATGTAAATCAAAT---
ATCACAGGATTACTATTGGTGCGCGATGGAGGAACAACAAATGCC----------------------------
----

ACGGACGAGATATTCAGACCTGGAGGAGGAGATATGAGGGACAATTGGAGAAGTGAACTATATAAATATAAAG
TGGTAGAAATTAAGCCATTG---GGAATAGCACCCACTGAGGCAAAAAGGAGAGTGGTG--------------
-------------------------GAGAGAGAAAAAAGAGCA---GTAGGA---CTAGGAGCT---------
GTGTTCCTTGGGTTCTTGGGAGCA---GCAGGAAGCACTATG---GGCGCGGCGTCA---
ATAACGCTGACGGTACAG---GCCAGACAA---CTGCTG---TCTGGTATAGTGCAACAGCAAAAT---
AATTTG---CTGAGA---GCTATAGAGGCGCAACAACATCTGTTGCAACTCACGGTCTGGGGCATT
>AF443085
TTCTATTGCAATACATCAGAACTGTTTAACAGTACGTACAATGCAAATATGACATATAATACAAGCATGACTA
ATGCTACA---
-------------------GGA---
AATAACTCAACAACAATCATACTCCCATGCAGAATAAAACAAATTATAAACATGTGGCAGGAGGTAGGACGAG
CAATGTATGCCCCGCCCATTGAAGGGAACATAACATGTAAATCAAAT---
ATCACAGGACTACTATTGGTACGTGATGGAGGAGGAGGAAAGAATGCC-------------------------
----

ACAAATGACACAGAGATATTCAGACCTGAAGGAGGAGACATGAGAAACAATTGGAGAAGTGAATTATATAAAT
ATAAAGTGGTAGAAATTAAGCCATTG---GGAGTAGCACCCACTAAGGCAAAGAGAAGAGTGGTG--------
-------------------------------GACAAAGAAAAAAGAGCA---ATA------GCAGGAGCT---
------GTGTTCCTTGGGTTCTTGGGAGTA---GCAGGAAGCACTATG---GGCGCGGCGTCA---
GTGGCGCTGACGGTACAG---GCCAGACAA---TTGTTG---TCTGGTATAGTGCAACAGCAAAGC---
AATTTG---CTGAGA---GCTATAGAGGCGCAACAGCATATGTTGCAACTCACGGTCTGGGGCATT
>AF443086
TTCTATTGCAATACATCAAAACTGTTTAACAGTACGTTTAATAGCAATGGTTCATACATATGGCATGACAGTA
CA---

GGAAATGATCCACTAACCATCCCATGTAGAATAAAACAAATTATAAACATGTGGCAGGAGGTAGGACGAGCAA
TGTATGCCCCTCCCATTGCAGGAAGAATAATATGTAACTCAACT---
ATCACAGGGTTAATATTGACACGTGATGGAGGAAATAATACTAAT----------------------------
----

AATACAGAGATATTCAGACCTATAGGAGGAAATATGAGGGACAATTGGAGAAGTGAATTATATAAATATAAAG
TGGTAGAAATTAAGCCATTA---GGAATAGCACCCACTAAGGCAAAAAGGAGAGTGGTG--------------
-------------------------GAGAGAGAAAAAAGAGCA---GTGGGA---ATAGGAGCT---------
GTGTTCCTTGGGTTCTTGGGAGCA---GCAGGAAGCACTATG---GGCGCAGCATCA---
ATAACGCTGACGGTACAG---GCCAGACAA---CTGTTG---TCTGGTATAGTGCAACAGCAAAGC---
AATTTG---CTGAGG---GCTATAGAGGCGCAACAGCATATGTTGCAACTCACGGTCTGGGGCATT
>HM215421
TTCTATTGTAATACATCAAGCCTGTTTAATAGCACATACATGTCTAATGGCACATACATGTTTAATGACATGT
CTAATGGTACA--
-------------GAAAGG------
AATTCAAGCTCAATCATCGCAATTCCATGCAGAATAAAGCAAGTTATAAACATGTGGCAGGAGGTAGGACGAG
CGATGTATGCCCCTCCCATTGCAGGAAAATTAACATGTAGATCAAAT---
ATCACAGGACTACTATTGGTACGTGATGGAGGAACAAATAATGCT----------------------------
----

ACAACAGAGACATTCAGACCTGGAGGAGGAGATATGAGGAACAATTGGAGAAGTGAATTATACAAATATAAAG
TGGTAGAAATTAAGCCATTG---GGAGTAGCACCCACTGAGGCAAAAAGGAGAGTGGTG--------------
-------------------------GAGAGAGAAAAAAGAGCA---GTGGGA---ATAGGAGCT---------
GTGTTCCTTGGGTTCTTGGGTGCA---GCCGGAAGCACTATG---GGCGCGGCGTCA---
ATAACGCTGACGGTACAG---GCCAGACAA---TTGCTG---TCTGGTATAGTGCAACAGCAAAGC---
AATTTG---CTGAGG---GCTATAGAGGCGCAACAGCATATGTTGCAACTCACGGTCTGGGGCATT

>HQ699992
TTCTATTGTAATACATCAGGCCTGTTTAATAGTACATACATGACTAATGGTACATACATGTTTAATGGTACAT
ACAGGCTTAAT--
-------------AATATA------
ACAAGTAATTCAAACATCACAATTCCATGCAGAATAAAGCAAATTATAAACACGTGGCAGGAGGTAGGGCGAG
CAATGTATGCCAATCCCATTGCAGGAAACATAACATGTAAATCAAAT---
ATCACAGGACTACTATTGGTACGTGATGGAGGAACAAATAATGCT----------------------------
----

ACAACAGAGACATTCAGACCTGGAGGAGGAAATATGAGGGACAATTGGAGAAGTGAATTATATAAATATAAGG
TGGTAGAAATTAAGCCATTG---GGAATAGCACCCACTGAAGCAAAAAGGCGAGTGGTG--------------
-------------------------GAGAGAGAAAAAAGAGCA---GTGGGA---ATAGGAGCT---------
GTGTTCCTTGGGTTCTTGGGTGCA---GCCGGAAGCACTATG---GGCGCAGCGTCA---
ATAACGCTGACGGTACAG---GCCAGACAA---TTGCTG---TCTGGTATAGTGCAACAGCAAAGC---
AATTTG---CTGAGG---GCTATAGAGGCGCAACAGCATATGTTGCAACTCACGGTCTGGGGCATT
>KF835515
TTCTATTGTGATACATCAGGCCTGTTTAATAGTACATACAATAGTACATACGTGCCTAATGGTACAGAA----
----

AGTAAGCCAAACATCACAATCCAATGCAGAATAAAGCAAATTATAAACATGTGGCAGGAGGTAGGACGAGCAA
TGTACGCCCCTCCCATTAAAGGAAGCATAACATGTAAATCAAAT---
ATCACAGGACTACTATTGGTACGTGATGGAGGAGCAAATACTACA----------------------------
----

GAAGAGATATTCAGACCTGGAGGAGGAGATATGAGGGACAATTGGAGAAGTGAATTATATAAATATAAAGTGG
TAGAAATCAAGCCATTG---GGAATAGCACCCACTGAAGCAAAAAGGAGAGTGGTG-----------------
----------------------GAGAGAGAAAAAAGAGCA---GTGGGA---CTAGGAGCT---------
GTGTTCCTTGGGTTCTTGGGCGCA---GCAGGAAGCACTATG---GGCGCGGCGTCA---
ATAACGCTGACGGTACAG---GCCAGACAA---TTGCTG---TCTGGTATAGTGCAACAGCAAAAC---
AATTTG---CTGAAG---GCTATAGAGGCGCAACAGCATATGTTGCAACTCACGGTCTGGGGCATT
>HQ699985
TTCTATTGTAATACATCAAAACTGTTTAATGGTACATACATGCCTAATGGTACAAACAGTAATTCAAGCAGTA
AT---

CCAAACTCAACCATCACAATCCCATGCAGTATAAAGCAAATTATAAATATGTGGCAGGAGGTAGGACGAGCAA
TGTATGCCCCTCCTATTGCAGGAAACATAACATGTAAATCAAAC---
ATCACGGGACTACTATTGGTACGTGATGGAGGAAATGTTACAGGGAGA-------------------------
----

AATGGTACAGAGATATTCAGACCTGAAGGAGGAAATATGAGGGACAATTGGAGAAGTGAATTATATAAATATA
AAGTGGTAGAAATTAAGCCATTG---GGAGTAGCACCCACTGGAGCAAAAAGGAGAGTGGTG-----------
----------------------------GAGAGAGAAAAAAGAGCA---GTGGGA---ATAGGAGCT------
---ATGTTCCTTGGGTTCTTGGGAGCA---GCAGGAAGCACTATG---GGCGCGGCGTCA---
ATAACGCTGACGGTACAG---GCCAGACAA---TTGCTG---TCTGGTATAGTGCAACAGCAAAAC---
AATTTG---CTGAGA---GCTATAGAGGCGCAACAACATCTGTTGCAACTCACGGTCTGGGGCATT
>HM215411
TTCTATTGTAACACATCAGACCTGTTTAATTCTACATACATGTCTAATGGTACATTCAGGTTTAATGGTACAG
TGTTTAATGGT--
----------ACAGGAGGT------
GATTCAAGCATAAACATCACAATCCCATGCAGAATAAAGCAAATTATAAACATGTGGCAGGAAGTAGGACGAG
CAATGTATGCCCCTCCCATTGCAGGAAATATAACATGTAAATCAAAT---
ATCACAGGACTACTATTGGTACGTGATGGAGGAACAACAAATAATACA-------------------------
----

AATAATACAGAGATATTCAGACCTGGAGGAGGAGATATGAGGGACAATTGGAGAAGTGAATTATATAAATATA
AAGTGGTAGAAGTTAAACCATTG---GGAATAGCACCCACTACAGCAAAAAGGAGAGTGGTA-----------
----------------------------GAGAGAGAAAAAAGAGCA---GTGGGA---CTAGGAGCT------
---GTGTTCCTTGGGTTCTTGGGAGCA---GCAGGAAGCACTATG---GGCGCAGCGTCA---
ATAACGCTGACGGTACAG---GCCAGACAA---TTGCTG---TCTGGTATAGTGCAACAGCAAAGC---
AATTTG---CTGAAA---GCTATAGAGGCGCAACAGCATCTGTTGCAACTCACGGTCTGGGGCATT

>KC898995
TTCTATTGTAATACATCAAGACTGTTTAATAGTACATATATGGATAATAGTACAGAAAGTAAT----------
----

TCAAACTCAACCATCACAATCCCATGTAGAATAAAGCAATTTATAAACATGTGGCAGCAGGTAGGACGAGCAA
TGTATGCCCCTCCCATTGAAGGAAATATAACATGTAAATCAAAT---
ATCACAGGACTACTATTGGTACGTGATGGAGGAAAAACAAATGAT----------------------------
----

ACAACAGAGACATTCAGACCTGGAGGAGGAGATATGAGGGACAATTGGAGAAGTGAATTATATAAATATAAAG
TGGTAGAAATTAAACCATTG---GGAATAGCACCCACTGAAGCAAAAAGGAGAGTGGTG--------------
-------------------------GAGAGAGAAAAAAGAGCA---GTGGGA---ATAGGAGCT---------
GTGTTCCTTGGGTTCTTGGGAGCA---GCAGGAAGCACTATG---GGCGCGGCGTCA---
ATAACGCTGACGGTACAG---GCCAGACAA---TTATTG---TCTGGTATAGTGCAACAGCAAAGC---
AATTTG---CTGAGG---GCTATAGAGGCGCAACAGCATATGCTGCAACTCACGGTCTGGGGCATT
>KC898996
TTCTATTGTAATACATCAGGCCTGTTTAATAGTACATACACGTCTAAGGGTACATACAGGTTTAATGGTACAG
AAAGTAATGGT--
----------ACAGAAAGT------
AATTCAAGCTCAATCATCACAATCCCATGCAGAATAAAGCAAATTATAAACATGTGGCAGGAGGTAGGACGAG
CAATGTATGCCCCTCCCATTGAAGGAAACATAACATGTAAATCAAAT---
ATCACAGGACTATTATTGGTACGTGATGGAGGAAAAGGAGACACAGGG-------------------------
----

CTAAATGATACAGAGACATTCAGACCTGGAGGAGGAGATATGAGAGACAATTGGAGAAATGAATTATATAAAT
ATAAAGTGGTAGAAATTAAGCCATTG---GGAATAGCACCCACTGCAGCAAAAAGGAGAGTGGTG--------
-------------------------------GAGAGAGGAAAAAGAGCA---GTGGGA---ATAGGAGCT---
------GTGTTCCTTGGGTTCTTGGGAGCA---GCAGGAAGCACTATG---GGCGCGGCGTCA---
ATAACGCTGACGGTACAG---GCCAGACAA---TTATTG---TCTGGTATAGTGCAACAGCAAAGC---
AATTTG---CTGAAG---GCTATAGAGGCGCAACAGCATCTGTTGCAACTCACGGTCTGGGGCATT
>KF250403
TTCTATTGCAATACATCAGGCCTGTTTAATAGTACATACACGCCTTTTAATAGTACATACATGCCTAATGGTA
CAGAAAGTAAT--

TCAAGCTTAACCATCACACTCCCATGCAGAATAAAGCAAATTATAAACATGTGGCAGGAGGTAGGACGAGCAA
TGTATGCCCCTCCCGTTGCAGGAAACATAACATGTAAATCAAAT---
ATCACAGGACTACTATTGGTACGTGATGGAGGCATAAATAATACA----------------------------
----

AGAGAGGAGACATTCAGACCTGGAGGAGGAGAAATGAGGGACAATTGGAGAAGTGAATTATATAAATATAAAG
TGGTAGAAATTAAGCCATTG---GGAATAGCACCCACTGCAGCAAAAAGGAGAGTAGTG--------------
-------------------------GAGAGAGAAAAAAGAGCA---GTGGGA---ATAGGAGCT---------
GTGTTCCTTGGGTTCTTGGGAGTA---GCCGGAAGCACTATG---GGCGCGGCGTCA---
ATGACGCTGACGGTACAG---GCCAGACAA---TTATTG---TCTGGTATAGTGCAACAGCAAAGC---
AATTTG---CTGAAG---GCTATAGAGGCGCAACAGCATCTGTTGCAACTCACAGTCTGGGGCATT
>KF250404
TTCTATTGTAATACATCAGATCTGTTTAATAGTACATACATGCCTAATAGTACAGAAAGTAATTCAAACTCCT
CC---

TCAAATTTAACCATCACACTCCAATGCAGAATAAAGCAAATTATAAACATGTGGCAGGAGGTAGGACGAGCAA
TGTATGCCCCTCCCATTGCAGGAAACATAACATGTAGATCAAAT---
ATTACAGGACTACTATTGGTACGTGATGGAGGT--

TCGAATGATACAGAGACATTCAGACCTGGAGGAGGAGATATGAGGGACAATTGGAGAAGTGAATTATATAAAT
ATAAAGTGGTAGAAATTAAGCCATTG---GGAATAGCACCAACTGGAGCAAAAAGGAGAGTGGTG--------
-------------------------------GAGAGAGAAAAAAGAGCA---GTGGGA---ATAGGAGCT---
------GTGTTCCTTGGGTTCTTGGGAGTA---GCAGGAAGCACTATG---GGCGCGGCGTCA---
ATAACGCTGACGGCACAG---GCCAGAAAA---TTATTG---TCTGGTATAGTGCAACAGCAAAGC---
AATTTG---CTGAGA---GCTATAGAGGCGCAACAGCATATGTTGCAACTCACGGTCTGGGGCATT

>KC807921
TTCTATTGTAATACATCAAAACTGTTTAATAGTACATACATACATAATGGTACATACATGAGTGGTACAAAAA
AT---

AATTCAAGCTTCATCAGAATACCATGCAAAATAAAGCAAATTATAAACATGTGGCAGGAGGTAGGACGAGCAA
TGTATGCCCCTCCCATTGAAGGAAACATAACATGTAACTCAAGT---
ATCACAGGACTACTATTGGTACGTGATGGAGTATCAGACACAAAT----------------------------
----

GAGACATTCAGACCTGGAGGAGGAGATATGAGAGACAATTGGAGAAATGAATTATATAAATATAAAGTGGTAG
AAATTAAGCCATTA---GGACTAGCACCCACTGCAGCAAAAAGGAGAGTGGTG--------------------
-------------------GAGAGAGAAAAAAGAGCA---GTGGGA---CTAGGAGCT---------
GTGTTCCTTGGGTTCTTGGGAATG---GCAGGAAGCACTATG---GGCGCGGCGTCA---
ATAACGCTGACGGTACAG---GCCAGACAA---TTATTG---TCTGGTATAGTGCAACAGCAAAAC---
AATTTG---CTGAGA---GCTATAGAGGCGCAACAGCATATGTTGCAACTCACGGTCTGGGGCATT
>KC870038
TTCTATTGTAATACATCACAACTGTTTAATGGTACATACAATGGTACATAC----------------------
----
-ATGTCTAATGGTACA---------
GAAAGTAATGCAACCATCACAATTCCATGCAGAATAAAGCAAATTATAAACATGTGGCAGGAGGTAGGACGAG
CAATGTATGCCAATCCCATTGCAGGAAACATAACATGTAAATCAAAT---
ATTACAGGACTACTATTGGTACGTGATGGAGGA--

GAAGGTACAAATGCT---
ACAAATGAGACATTCAGACCTGGAGGAGGAGATATGAGGGACAATTGGAGAAGTGAATTATATAAATATAAAG
TGGTAGAAATTAAGCCATTG---GGGTTAGCACCCACTGAAGCAAAAAGGAGAGTGGTG--------------
-------------------------GAGAGAGAAAAAAGAGCA---GTGGGA---ATAGGAGCT---------
GTGTTCCTTGGGTTCTTGGGAGCA---GCCGGAAGCACTATG---GGCGCGGCGTCA---
ATAACGCTGACGGTACAG---GCCAGACAG---TTATTG---TCTGGTATAGTGCAACAGCAAAGC---
AATTTG---CTGAGG---GCTATAGAGGCGCAACAGCATCTGTTGCAACTCACGGTCTGGGGCATT
>FJ388913
TTCTATTGCAATACATCAAACCTGTTTAATAGTACATACATGACTAATGGTACATTCATACTTAATGGTACAG
GCATG--
-------------------AAT---
ACAGGTAATGAAACCATCATACTCCCATCCAGGATAAGACAATTTGTAATCCTGTGGCAGGAGGTAGGGCGAG
CAACGTATGCTCCTCCCATCGCAGGAAACATCACATGTAAATCAAAT---
ATTACAGGAGTACTAGTGGTACGTGATGGAGGCACAGGCAACGGGACA-------------------------
----

GAAAATAACACAGAGATATTCAGACCTGGAGGAGGGAATATGAAGGACAATTGGAGAAGTGAATTATATAAAT
ATAAAGTGGTAGAAATTAAGCCATTG---GGAATAGCACCCACTGGAGCAAAGAGGAGAGTGGTG--------
-------------------------------GAGAGAGAGAAAAGAGCA---GTGGGA---CTGGGAGCT---
------GTGATCTTTGGGTTCTTGGGAGCA---GCAGGAAGCACTATG---GGCGCGGCGTCA---
ATGGCGCTGACGGTACAG---GCCAGACAA---GTATTG---GCTGGTATAGTGCAACAGCAAAAC---
AATTTG---CTGAGA---GCCATAGAGGCGCAACAGCATATGTTGCAACTCACGGTCTGGGGCATT
>FJ388948
TTCTATTGCAATACATCAAAACTGTTTGACAGTACACTATTGGATAATAGTACAAATTTAAACTTGACTGGCA
ATATC--

AATTTAACTGGCAATATCACGCTTCCATGCAGAATAAAACAAATTATAAACATGTGGCAGGAGGTAGGACGAG
CAATGTATGCCTCTCCCATAGCAGGAAACATAACATGTAAATCAAAT---
ATCACAGGACTACTGTTAACACGTGATGGAGGAAAAGACAACAAT----------------------------
----

GGCACAGAAACATTCAGACCTGGAGGAGGAAATATGAAAGACAATTGGAGAAGTGAATTATATAAATATAAAG
TAGTAGAAATTAAGCCATTA---GGAATAGCACCCACTACAGCAAAAAGGAGAGTGGTG--------------
-------------------------GAGAGAGAAAAAAGAGCA---GTAGGA---ATAGGAGCT---------
GTGTTCCTTGGGTTCTTGGGAGCA---GCAGGAAGCACTATG---GGCGCGGCGTCT---
GTAACGCTGACGGTACAG---GCCAGACAA---CTGTTG---TCTGGTATAGTGCAGCAACAAAGC---
AATTTG---CTGAAG---GCTATAGAGGCGCAACAACATCTGTTGCAACTCACGGTCTGGGGCATA

>FJ388952
TTCTATTGCAATACATCAAAGCTGTTTAATAGTACGTACATGTCTAATGGTACATACATGGTTAATGGTTCTA
ATGGT--
-------------------GCA---
ACTCCAAATTCAACCATCACACTCCAATGTAGAATAAAACAAATTATAAACATGTGGCAGGGGGTAGGACAAG
CAATGTATGCCCCTCCCATTGCAGGAAACATAACCTGTAACTCAAGT---
ATCACAGGAATACTATTGACACGTGATGGAGGAATTAATAATGACACA-------------------------
----

AATAACATAGAGACTTTCAGACCTGGAGGAGGAGACATGAGGGACAATTGGAGAAGTGAATTGTATAAATATA
AAGTGGTAGAAATTAAACCATTA---GGAATAGCACCCACTAAGGCAAAAAGGAGAGTGGTG-----------
----------------------------GAGAGAGAAAAAAGAGCA---GCAACA---TTAGGAGCT------
---GTGTTCCTTGGGTTCTTGGGAGCA---GCTGGAAGCACTATG---GGCGCAGCATCA---
ATAACGCTGACGGTACAG---GCCAGACAA---TTATTG---TCGGGTATAGTGCAACAGCAAAGC---
AATTTG---CTGAGG---GCTATAGAGGCGCAACAGCATATGTTGCAACTCACGGTTTGGGGCATT
>JF683740
TTCTATTGCAATACATCAAAACTGTTTAATAGGAAATACACATTTAATGGCACAAATGGCACAAAC-------
----

ACTAATATCACTATCACATTCCCGAGCAAGATAAAAAAAAACATAAACCTGGGGCAGGGGGGAGCAAGATCAA
CCTCTGCCCCTGCCGGAACCGTAACCATAACA------TCAAGT---
TTAACAGGAACAGGATTGTTACGGGAAGGAGAAGGGAGAAAGAGCAAA-------------------------
----

CACACTAATGAGACATTCAGACCTGGAGGAGGAAATATGAAGGATAATTGGAGGAGTGAATTATATAAATATA
AAGTAGTAGAAATTAAACCATTA---GGAATAGCTCCCACTGAAGCAGAAAGGAGAGCGGTG-----------
----------------------------AAGAGAGAAAAAAGAGCA---GTGGGA---ATAGGAGCT------
---GTGTTCCTTGGGTTCTTGGGAGCA---GCAGGAAGCACTATG---GGCGCGGCGTCA---
ATAACGCTGACGGCACAG---GCCAGACAA---CTGTTG---TCTGGTATAGTGCAACAGCAAAGC---
AATTTG---CTGAGA---GCTATAGAAGCACAACAGCATCTGTTGCAGCTCACGGTCTGGGGCATT
>JF683755
TTCTATTGCAATACATCAGGACTGTTTAATGGCACAAATAGCACAGATAGCACA-------------------
----

AACAGCACAGTCATCACACTCCCATGCAGAATAAAACAAATTATAAGGATGTGGCAGGGAGTAGGACAAGCAA
TGTATGCTCCTCCCATTAGAGCAAACATAACATGTAGATCAAAT---
ATCACAGGACTACTATTAACACGTGATGGAGGACATAATTTTACT----------------------------
----

AATGAGACCTTCAGACCTGGAGGAGGAGATATGAGGGACAATTGGAGAAGTGAATTATATAAATATAAAGTGG
TAGAAATTAAACCATTA---GGAATAGCCCCCACTACAGCAAAAAGGAGAGTGGTGGAT---GGA--------
-------------GAAAAAATACCAAGAGAAAAAAGAGCA---ATAGGGGGACTAGGAGCT---------
GTGTTCCTTGGGTTCTTAGGAGTA---GCAGGAAGCACTATG---GGCGCGGCGTCA---
ATAACGCTGACGGTACAG---GCCAGAGAA---TTATTG---TCTGGTATAGTGCAACAGCAAAGC---
AATTTG---CTGAAG---GCTATAGAGGCGCAACAGCATATGTTGCAACTCACGGTCTGGGGCATT
>JF683768
TTCTATTGCAATACATCGAGCCTGTTTAATGGTACATACAATAGTACAGACAATAGTAGTTCATCTGAAACCA
TC---

AGTTCATCTGAAACCATCACACTTCCATGCAGAATAAAACAAATTATAAACATGTGGCAGGAGGTAGGACGAG
CAATGTATGCCCCTCCCATTGCAGGAAATATAACATGTAGATCAAAT---
ATCACAGGAATACTATTAACACGTGATGGAGGTAATGGAGGAAACAACACAAAC-------------------
----
-
GGAACTGAAGAGACATCAGAGATATTCAGACCTGGAGGAGGAGATATGAGGGACAATTGGAGAAGTGAATTAT
ATAAATATAAAGTGGTAGAAATTAAACCATTA---GGAGTAGCACCCGAGGAAGCAAAAAGAAGAGTGGTA--
-------------------------------------GAGAGAGAAAAAAGGGCA---GTGGGA---
CTAGGAGTT---------TTGTTCTTTGGGTTCTTGGGAGCA---GCAGGAAGCACTATG---
GGCGCGGCGTCA---TTAACGCTGACGGTACAG---GCCAGACAA---TTGTTG---

TCTGGTATAGTGCAGCAGCAAAGC---AATTTG---CTGAGA---
GCTATAGAGGCGCAACAGCATATGTTGCAACTCACAGTCTGGGGCATT
>KT124786
TTCTATTGTAATACATCAAAACTATTTAACAGTACATTTAATAGTACACACTTAGAAGATAATGCTACAAATA
ATAAT---

GATACAACATCCATTACACTTGCATGCAGAATAAAACAATTTGTGAATATGTGGCAGGAAGTAGGAAGAGCAA
TGTATGCCCCTCCCATTGCAGGAAACATAACATGTAAATCAAAT---
ATCACAGGAATACTGTTAGTACGCGATGGAGTC-------------------------------------
----

GGCAATGAGACAGAGATATTCAGACCTGGAGGAGGAAATATGAAGGACAATTGGAGAAGTGAATTATATAAAT
ATAAAGTGGTAGAAATTAAACCATTG---GGAATAGCACCCACTAAAGCAAAAAGGAGAGTGGTG--------
------------------------------GAGAGAGAAAAAAGAGCA---GCA------CTAGGAGCT---
------GTGTTCCTTGGGTTCTTGGGAGCA---GCCGGAAGCACTATG---GGCGCGGCGTCA---
CTAACGCTGACGGTACAG---GTCAGACAA---CTGTTG---TCTGGTATAGTGCAACAGCAAAGC---
AATTTG---CTGAGG---GCTATAGAGGCGCAACAGCATATGTTGCAACTCACGGTCTGGGGCATT
>KC473844
TTCTATTGCAATACATCAAAACTATTTAATAGTACAATTAATAGTACAGTCATGGTTAATGATACA-------
----

GACAACAGCACAATCACACTCCCATGCAGGATAAAACAAATTATAAACATGTGGCAAGGGGTAGGACGAGCAA
TGTATGCCCCTCCCATTGCAGGAAATATAACATGTAACTCAAGT---
ATCACAGGACTAATATTGACACGTGATGGAGGAATAGGAAATGGAACT------------------------
----

GATAAGACAGAGATATTTAGACCAGCAGGAGGAGATATGAGGGACAATTGGAGAAGTGAATTATATAAATATA
AAGTGGTAGAAATTAAACCATTA---GGGATAGCACCCACTGAGGCAAAAAGGAGAGTGGTG-----------
---------------------------GAGAGAGAGAAAAGAGCA---GTGGGA---ATAGGAGCT------
---GTGTTCCTTGGGTTCTTGGGAGTA---GCAGGAAGCACTATG---GGCGCGGCGTCA---
ATGGCGCTGACGGTACAA---GCCAGACAA---TTATTG---TCTGGTATAGTGCAACAGCAAAGC---
AATTTG---CTGAGG---GCTATAGAGGCGCAACAGCATATGTTGCAACTCACAGTCTGGGGCATT
>HQ236594
TTTTATTGCAATACATCACCCCTGTTTAATAGTACATACAATAAGACAGAAAGCATAATTGAGGAA-------
----

GACAATTCAACCATCACCATCCAATGCAGAATAAAACAAATTATAAACATGTGGCAGGAGGTAGGACAAGCAA
TGTATGCCCCCCCCATAAAAGGAATAATAAAATGTAGGTCAAAC---
ATCACAGGATTACTACTAACACGGGATGGAGGTACGGGTAACGAAACA------------------------
----

GATAATGGAACAGAGACATTCAGGCCTGGAGGAGGAGATATGAGAGACAATTGGAGAAGTGAATTATATAAAT
ATAAAGTGGTAGAAATTAAACCATTA---GGAATAGCACCCACTAAGGCAAAAAGGAGAGTGGTG--------
------------------------------GAGAGAGAAAAAAGAGCA---GTGGGA---ATTGGAGCT---
------GTGCTCCTTGGGTTCTTGGGAGCA---GCAGGAAGCACTATG---GGCGCAGCATCA---
ATAATGCTGACGGTACAG---GCCAGACAA---TTATTG---TCTGGGATAGTGCAACAGCAAAGC---
AATTTG---CTGAGA---GCTATAGAGGCGCAACAGCATATGTTGCAACTCACGGTCTGGGGCATT
>HQ236601
TTCTATTGCAATACATCAAAACTGTTTAATAGTACATACAATAGTGCATACAATAGTACATACAACAGAACTG
AAAAA---

GGAGATGCAAACATCACAACCATCCAGTGCAGAATAAGGCAAATTATAAACATGTGGCAAGGGGTAGGACGAG
CAATGTATGCCCCTCCCATTAAAGGAAACATAACATGTAGATCAAAT---
ATCACAGGACTACTATTGACACGTGATGGAGGAGTAAATAATAGT---------------------------
----

GAAACAACAGAGACATTTAGGCCTGGAGGAGGAGATATGAGGGACAATTGGAGAAGTGAATTATATAAATATA
AAGTGGTAGAAATTAAACCATTA---GGAATAGCACCTACTAGAGCAAAAAGGAGAGTGGTG-----------
---------------------------CAGAGAGAAAAAAGAGCA---GTAACT---CTGGGAGCT------
---ATGTTCCTTGGTTTCTTGGGAGCA---GCAGGAAGCACTATG---GGCGCAGCATCA---

ATAACGCTGACGGTACAG---GCCAGACAA---TTATTG---TCTGGGATAGTGCAACAGCAAAGC---
AATTTG---CTGAGG---GCTATAGAGGCGCAACAGCATATGTTGCAACTCACAGTCTGGGGCATT
>KY496624
TTCTATTGCAATACAACAAGCCTGTTTAATGGTACATACAATAATAGTACAGGAGATCTTACA----------
----

GAAGGAGGAACCATCACGATCCCATGCAAAATAAAACAATTTATAAACATGTGGCAGGAGGTAGGGCGAGCAA
TGTATGCCCCTCCCATTGAAGGAATGATAACATGTAACTCAAAT---
ATTACAGGATTACTACTGACACGGGATGGAGGCAGCGAAGGCAAC----------------------------
----

AATACAGAGATATTCAGGCCTGGAGGAGGAGATATGAGGGACAATTGGAGAAGTGAATTATATAAATATAAAG
TAGTAGAAATTAAACCATTA---GGAATAGCACCCACCAAAGCAAAAAGGAGAGTGGTG--------------
-------------------------GAGAGAGAAAAAAGAGCA---GTGGGA---ATAGGAGCT---------
GTGTTCCTTGGGTTCTTGGGAGTA---GCAGGAAGCACTATG---GGCGCGGCGTCG---
CTGACGTTGACGGTACAG---GCCAGACAA---TTATTG---TCTGGTATAGTGCAACAGCAAAGC---
AATTTG---CTGAGA---GCTATAGAGGCGCAACAACATATGTTGCAACTCACAGTCTGGGGCATT
>AY713417
TTCTATTGCAATACATCAAAACTGTTTAATACAACAGGACTGTCT----------------------------
----

GATAATGCAACTTTAACCATCCCATGCAGAATAAAGCAAATTATAAACATGTGGCAGGAGGTAGGACGAGCAA
TGTATGCCCCTCCCATTAAAGGAATAATAACATGTAGATCAAAT---
ATCACAGGATTATTACTGACACGTGATGGGGGGATGAACAACACA----------------------------
----

AACAACACAGAGACATTCAGACCTGGAGGAGGAGATATGAGGGACAATTGGAGAAGTGAATTATATAAGTATA
AAGTGGTAGAAATTAAGCCATTA---GGAGTAGCACCCACTGGGGCAAAAAGGGCAGTGGTG-----------
----------------------------GGGAGAGAAAAAAGAGCA---GCA------CTAGGAGCT------
---TTGTTCCTTGGGTTCTTGGGAGCG---GCAGGAAGCACTATG---GGCGCAGCGTCA---
ATAACGCTGACGGTACAG---GCCAGACAA---TTATTG---TCGGGTATAGTGCAGCAGCAAAGC---
AATTTG---CTGAGA---GCTATAGAGGCGCAACAGCATATGTTGCAACTCACGGTCTGGGGCATT
>KU319531
TTCTACTGCAATCCATCAGGACTGTTTAATAGGACATACATGTTTAATAGTACAGACGTGTTTAATAGTAATA
GTACAGACAGG---TCT------AAT---
-------------------AGT---
ACTAATAGTACTTCCATCACACTCCAATGCAGAATAAAGCAAATTATAAACCTGTGGCAGGGGGTAGGAAAAG
CAATATATGCCCCTCCCATTGCAGGGAAAATAACATGTAAATCAAAC---
ATTACAGGAATACTACTGACACGAGATGGAGGAAGTAATAACAGT----------------------------
----

AGCACAGAGACATTCAGACCTGGAGGAGGAGATATGAGGGACAATTGGAGAAGTGAACTATATAAATATAAAG
TGGTAGAAATTAAACCATTA---GGAATAGTACCCACCGAGGCACAAAGAAGAGTGGTG--------------
-------------------------GGGAGAGAAAAAAGAGCA---ATAGGA---ATAGGAGCT---------
ATGTTCCTTGGGTTCTTGGGAGCA---GCAGGAAGCACTATG---GGCGCAGCGTCA---
ATAGCGCTGACGGTACAG---GCCAGACAA---TTATTG---TCGGGTATAGTGCAACAGCAAAAT---
AACTTG---CTGAGG---GCTATAGAGGCGCAACAGCATATGTTGCAACTCACAGTCTGGGGCATC
>KU319532
TTTTACTGTGATACAACAAACCTGTTTAATAGTACATACAATGGTACTAGTACTAATAGTACT----------
----
-------------TCCAGT------
GGTACCATCACCCTCTCATGCAGTATAAAACAAATTATACAGATGTGGCAAGGATTACGAAAAGTATTGAATG
CCCCTGTCTGCGCTCCCCTTGACGGAAACATAAAATGTACATCAAAT---
TTCACAGGACTACTATTGCCACGTGCTGGAGGAAGTGGACTTAAT----------------------------
----

GATACTGACGCAGAAGTATTCAGACCAGCAGGAGGAGACATGAGGGACAATTGGAGAAGTGAATTATATAAAT
ATAAAGTGGTAGAAATTAAACCATTA---GGAGTAGCACCAACTACAGCAAAAAGGAGAGTGGTG--------
-------------------------------GAGAGAGAAAAAAGAGCA---GTGGGA---ATAGGAGCT---
------GTGTTCCTTGGGTTCTTGGGAGCA---GCAGGAAGCACTATG---GGCGCAGCATCA---

ATAACGCTGACGGTACAG---GCCAGACAA---TTATTG---TCTGGTATAGTGCAACAGCAAAGC---
AATTTG---CTGAGG---GCTATAGAGGCGCAACAGCATCTGTTGCAACTCACAGTCTGGGGCATT
>KU319534
TTCTATTGGGATACAACAAAACTGTTTAATGAGACATATAATAATACAGAT----------------------
----

GCAAATGGCACCATCATAATGCCATGCAGAATAAAACAAATTATAAACATGTGGCAGAAGGTAGGAAGAGCAA
TGTATGCCCCTCCCATTGAAGGACCAATAACATGTATATCAAAC---
ATCACAGGACTACTACTAACACATGATGGAGAGGAA-------------------------------------
----

AATGACACAGAGACATTCAGACCTTGGGGAGGAGATATGAGGGACAATTGGAGAAGTGAATTATATAAATACA
AAGTGGTAGAAATTAAGCCATTA---GGAATAGCACCCACTAAGGCAAAAAGGAGAGTGGTG-----------
----------------------------CAGAGAGAAAAAAGAGCA---GCA------CTAGGAGCT------
---ATGTTCCTTGGGTTCTTGGGAGCA---GCAGGAAGCACTATG---GGCGCAGCATCA---
ATAACGCTGACGGTACAG---GCCAGACAA---TTATTG---TCTGGTATAGTGCAACAGCAAAGC---
AATTTG---CTGAGA---GCTATAGAGGCGCAACAGCATCTGTTGCAACTCACGGTCTGGGGCATT
>KU319537
TTTTATTGCAATACATCACACTTGTTTAAGGATAATATTAGT-------------------------------
----

AGCACGGGAAACATCACAATCCCATGCAGAATAAGACAAATTATAAACTTGTGGCAGGAAGTAGGACGAGCAA
TGTATGCTCCTCCCATTGAAGGACTAATAAAATGTAGATCAAAT---
ATCACAGGACTATTGCTGACACGTGATGGAGGCAATAGACAAAAT----------------------------
----

GGCACAGAGATATTCAGACCAGGAGGAGGAGATATGAGGGACAATTGGAGAAGTGAATTATATAAATATAAAG
TAGTAGAAATTAAACCATTA---GGAATAGCACCCACTAAAGCAAAAAGGAAAGTGGTG--------------
-------------------------AAGAGAGAAAAAAGAGCA---GCA------ATAGGAGCT---------
GTGTTCCTTGGGTTCTTGGGAGCA---GCTGGAAGCACTATG---GGCGCAGCATCA---
ATAACGCTGACGGTACAG---GCCAGACAA---TTGTTG---TCTGGTATAGTGCAACAGCAAAGC---
AATTTG---CTGAGG---GCTATAGAGGCGCAACAGCATATGTTGCAACTCACAGTCTGGGGCATT
>KU319541
TTCTATTGCAATACATCAGGCCTGTTTAATAATAGTATG----------------------------------
----
-ACAAATGGCACCCTC---------
AGACTCCCATGCAGAATAAAACAAATTATAAACATGTGGCAGGGAGTAGGACGAGCAATGTATGCTCCTCCCA
TTAGCGGAGCAATAAGTAGGAAGGCCATAGTATCATGCTTATCAAAT---
ATCACAGGGATACTCTTAACATGTGATGGAGGCATTAATAGGCACAGA-------------------------
----

CATAACACAGAGACATTCAGACCTGTAGGAGGAGACATGAAGGACAATTGTAGAAGTGAATTATATAAATATA
AGGTGGTAGAAGTTAAAGCCATA---GGAATAGCACCCACACAGGCAAAAAGGAGAGTGGTG-----------
----------------------------CAGAGAGAAAAAAGAGCA---GCA------TTAGGAGTT------
---TTATTCTTTGGGTTCTTGGGAGCA---GCCGGAAGCATTATG---GGCGCAGCATCA---
ATATTTTTTTTGGTACAG---GCCAGACAA---TTATTG---TCTGGTATAGTGCAACAGCAGAGC---
AATTTG---CTGAGG---GCTATAGAGGCGCAGCAGCATCTGTTGCAACTCACGGTCTGGGGCATT
>KU319544
TTCTATTGCAATACAACAGGACTGTTTAACAGTACGTACAATAGTACACACATATTTAATAATACT-------
----

TCAAATGCAACCATCACACTCCCCTGCAGAATAAAACAAATTATCAACATGTGGCAGGGGGTAGGACGAGCAA
TGTATGCCCCTCCCACTGCAGGAAACATAACATGTAGATCAAAT---
ATCACAGGGCTACTATTAACACGTGATGGAGGAAGCGACAGCGACACG-------------------------
----

ACTGAGGAGACATTCAGACCTGGAGGAGGAAATATGAGAGACAATTGGAGAAGTGAATTATATAAATATAAAG
TGGTAGAAATTAAACCATTA---GGAGTAGTACCCACTACTGCAAAAAGGAGAGTGGTG--------------
-------------------------GAAAGAGAAAAAAGAGCA---GTGGGA---ATAGGAGCT---------
GTGTTCCTTGGGTTCTTGGGAGCA---GCAGGAAGCACTATG---GGCGCAGCATCA---

ATAACGCTGACGGTACAG---GCCAGACAA---TTGCTG---TCTGGTATAGTGCAACAGCAAAGC---
AATTTG---CTGAGG---GCTATAGAGGCGCAACAGCATATGTTGCAACTCACGGTTTGGGGCATT
>KU319546
TTCTATTGCAATACATCAAAACTGTTTAATGGTACATACAACTCTAATAGTACTAATAGTACT----------
----

TCAAATTCAAGCATCACAATCCAATGCAGAATAAAACAAATTATAAACATGTGGCAGGAGGTAGGACGAGCAA
TGTATGCCCCTCCCATTGCAGGAAACATAACATGTAGATCAAAT---
ATCACAGGAATACTATTGACATGGGATGGAGGAAACCAAAACGGAACC-------------------------
----

AACAATACAGAGACATTCAGACCTGGAGGAGGAGATATGAGGGACAATTGGAGAAGTGAATTATATAAATATA
AAGTGGTAGAAATTAAACCATTA---GGAGTAGCACCCACTAAGGCAAAGAGGACAGTGGTG-----------
----------------------------GAAAGAGAAAAAAGAGCA---GTGGGA---ATAGGAGCT------
---GTGTTCCTTGGGTTCTTGGGAGCA---GCAGGAAGCACTATG---GGCGCAGCATCA---
ATAACGCTGACGGTACAG---GCCAAACTA---TTGTTG---GCTGGTATAGTGCAGCAGCAAAAT---
AATTTG---CTGAGG---GCTATAGAGGCGCAACAACATATGTTGCAACTCACGGTCTGGGGCATT
>KU319551
TTCTACTGTAGTACTACACCCTTGTTTACTAGTACTAGTACTACTGGGGGTACTACTGATTCAAATTCA----
----

TACATCACAGTCATCACTCCCCCATGCAGTATACTACTATGTCGTAACATGTGGCAGGAGGTAGGACGAGCCA
TATATGCTCCTCCTATTAAAGGAGTAATAACATGTAGATCAAAT---
ATCACAGGACTACTGCTGACACGGGATGGCGGCCATAGCACAAAT----------------------------
----

GACACAGAGATATTCAGACCTATAGGAGGAAATATGAAGGACAATTGGAGAAGTGAATTATATAAATATAAAG
TGGTAGAAGTTAAGCCATTA---GGAGTAGCACCCACTGAGGCAAGAAGGAGAGTGGTG--------------
-------------------------GAGAGAGAAAAAAGAGCA---GCA------CTAGGAGCT---------
TTGTTCCTTGGGTTCTTGGGAGCA---GCAGGAAGCACTATG---GGCGCAGCGTCA---
ATAACGCTGACGGTACAG---GCCAGACAA---TTACTG---TCGGGTATAGTGCAACAGCAAAGC---
AATTTG---CTGAGA---GCTATTGAGGCGCAACAACATATGTTGCAGCTCACGGTCTGGGGCATT
>U46016
TTCTATTGCAATACATCAAACCTGTTTAATAGCACAAAACTAGAACTGTTTAATAGCAGT-------------
----

ACAAATTTAAACATCACACTCCAATGCAGAATAAAACAAATTATAAACATGTGGCAGGGAGTAGGACGAGCAA
TGTATGCCCCTCCCATTGAAGGAATAATAATGTGTAGATCAAAT---
ATCACAGGACTACTACTGACACGTGATGGAGCCAAAGAGCCACAT----------------------------
----

AGCACAAAAGAGATATTCAGACCTGAAGGAGGAGATATGAGGGACAATTGGAGAAGTGAATTATATAAATATA
AAGTGGTAGAAATTAAGCCACTA---GGAGTAGCACCCACTAAGCCAAAAAGGAGAGTGGTG-----------
----------------------------GAGAGAGAAAAAAGAGCA---GCA------CTAGGAGCT------
---TTGTTCCTTGGGTTCTTGGGAGCA---GCAGGAAGCACTATG---GGCGCAGCATCA---
ATAACGCTGACGGTACAG---GCCAGACAA---TTATTG---TCGGGTATAGTGCAACAGCAAAGC---
AATTTG---CTGAAA---GCTATAGAGGCGCAACAGCATATGTTGCAGCTCACGGTCTGGGGCATT
>AF219262
TTCTATTGCAATACAACAGACCTATTTAATAGCACAAAACTGTTTAATAGCAGTAGT----------------
----

ACAAATTCAACCATCACACTTCCATGCAGAATAAAACAAATTATAAACATGTGGCAGGGGGTAGGACGAGCAA
TGTATGCCCCTCCCATTAAAGGAATAATAACATGTAGATCAAAT---
ATCACAGGAATACTACTGACACGTGATGGAGGGAATGACAATAGCACA-------------------------
----

AATGGCACAGAGACATTCAGACCTGGAGGAGGAGATATGAGAGACAATTGGAGAAGTGAATTATATAAATATA
AAGTGGTAGAGATTAAGCCATTA---GGAATAGCACCCACTGGGGCAAAAAGGCGAGTGGTG-----------
----------------------------GAGAGAGAAAAAAGAGCA---GCA------CTAGGAGCT------
---ATGTTCCTTGGGTTCTTGGGAGCA---GCTGGAAGCACTATG---GGCGCAGCATCA---

ATAACGCTGACGGTACAG---GCCAGACAA---TTATTG---TCGGGTATAGTGCAACAGCAAAAT---
AATTTG---CTGAGG---GCTATAGAGGCGCAACAGCATATGTTGCAACTCACGGTCTGGGGCATT
>AF219263
TTCTATTGCAATACATCAAAATTGTCTAATAGGACATACAATGCATCAGACCTGGTTAATAGTACA-------
----

AGTAATTCAACCATCACACTCCCATGCAGAATAAAGCAAATTGTAAACATGTGGCAGGAAGTGGGACGAGCAA
TGTATGCCCCTCCCATTGCAGGAAACATAACATGTAACTCAAAT---
ATCACAGGACTACTATTGACACGTGATGGAGGAAATAATGCAAATAAT-------------------------
----

GCAAGTACCAACGAGACATTCAGACCTGGAGGAGGAGATATGAGGGACAATTGGAGAAGTGAATTGTATAAAT
ATAAAGTGGTAGAAATTAAGCCATTG---GGAGTAGCACCCACCAAGGCAAAAAGGAGAGTGGTG--------
-------------------------------GGGAGAGAGAAAAGAGCA---GTGGGA---CTAGGAGCT---
------GCGTTCCTTGGGTTCTTAGGAGCA---GCAGGAAGCACTATG---GGCGCGGCGTCA---
ATAACGCTGACGGCACAG---GCCAGGCAA---GTGTTG---TCTGGTACAGTGCAACAGCAAAGC---
AATTTG---CTGAGG---GCTATAGAGGCGCAACAGCATATGTTGCAACTCACAGTCTGGGGCATT
>AF219269
TTCTATTGCAATACATCAAAACTGTTTAAGGGTACATACATGTATAATGATACAGAT----------------
----

ACAAACATCACACTCACACTCCCATGCAGAATAAAACAGATTATAAACATGTGGCAGGGGGTAGGACGAGCAA
TATATGCCCCTCCCATTGAAGGAAGCATAAAGTGTACATCGAAT---
ATCACAGGACTACTATTGACACGTGATGGA---GACAAAAATGAT----------------------------
----

ACAGAGACCTTCAGACCTGGAGGAGGAGATATGAGGGACAATTGGAGAAGTGAATTATATAAATATAAAGTGG
TAGAAATTAAGCCATTA---GGAATAGCACCCGCTAAGGCAAAAAGGAGAGTGGTG-----------------
----------------------GAGAGAGAAAAAAGAGCA---GTAGGA---ATGGGAGCT---------
GTGCTCCTTGGGTTCTTGGGAGCA---GCAGGAAGCACTATG---GGCGCAGCGTCA---
ATAACGCTGACGGTACAG---GCCAGGCAA---TTGTTG---TCTGGTATAGTGCAACAGCAAAGC---
AATTTG---CTGAGA---GCTATAGAGGCGCAACAGCATATGTTGCAACTCATGGTCTGGGGCATT
>AF219274
TTCTATTGCAATACATCAAAACTGTTTAATAGTACATATATGTTTAATAGTACATATACGTCTAATAATACAG
GAAGT--

AATTCCAACTCAACCATCACACTCCCATGCAGAATAAGACAATTTATAAACATGTGGCAGGAGGTAGGACGAG
CAATGTATGCCCCTCCCATTGAAGGAAACATAACATGTAAATCAAAT---
ATCACAGGACTACTATTGACACGGGATGGAGGCGTGGAGACACAAGATACCACA-------------------
----
-
GAGACACAAAATAACACAGAGACATTCAGACCTGGGGGAGGAGATATGAGGAACAATTGGAGAAGTGAATTAT
ATAAATATAAAGTGGTAGAAATTAAGCCATTG---GGAGTAGCACCTACTACGGCAAAAAGGAGAGTGGTG--
-------------------------------------GAGAGAGAAAAAAGAGCA---GTGGGA---
ATAGGAGCT---------GTGACCCTTGGGTTCTTGGGAGCA---GCAGGAAGCACTATG---
GGCGCGGCGTCA---GCAGCGCTGACGGTACAG---GCCAGACAA---TTGTTG---
TCTGGTATAGTGCAACAGCAAAGC---AATTTG---CTGAGG---
GCTATAGAGGCGCAACAGCATATGTTGCAACTCACAGTCTGGGGCATT
>FJ977094
TTCTATTGCAATACATCAGGCCTGTTTAATGGTACATTCAAGTTTAATGATACAGACATGACTAAT-------
----

GATACAGGAAACATCACACTTCCATGCAGAATAAAACAAATTATAAACATGTGGCAGGAGGTAGGACGAGCAA
TGTATGCCCCTCCCATTGCAGGAAACATAACATGTAAATCAAAT---
ATCACAGGACTACTATTGCTACGGGATGGAGGGACAAATGTAACA----------------------------
----

AATGAAACAGAAATATTCAGACCTGGAGGAGGAGATATGAGGGACAATTGGAGAAGTGAATTATATAAATATA
AAGTGGTTGAAATTAAGCCATTG---GGAATAGCACCCACTAAGGCAAAAAGGAGAGTGGTG-----------
----------------------------GAAAGACAAAAAAGAGCA---GCA------ATAGGAGCT------

```
---ATGATCATTGGGTTCTTGGGAGCA---GCAGGAAGCACTATG---GGCGCAGCGTCA---
ATAGCGCTGACGGCACAG---GCCAGACAA---GTATTG---TCTGGTATAGTGCAACAGCAAAGC---
AATTTG---CTGAGA---GCTATAGAGGCGCAACAGCATATGTTGCAACTCACAGTCTGGGGCATT
>HQ595789
TTCTATTGCAATACATCAGGCCTGTTTAACAGTAAATACATGCATAACATGACAATCAATGGGACAGACATGA
CAAGCAATGGG--------------------------------------------------------------
-------------ACAGAC------
AGTAATGCAACAATCATCACACTTCCATGCAGAATAAAACAAATTATAAACATGTGGCAGGAGGTAGGACGAG
CAATGTATGCCCCTCCCATTGCAGGAAACATAACATGTAACTCAAGC---
ATTACAGGACTACTATTAGTACGTGATGGAGGCACTGGAGACAGCAGC-------------------------
-------------------------------------------------------------------------
-------
AGCAATGACACAGAGACTTTCAGACCTGGAGGAGGAAATATGAAGGACAATTGGAGAAGTGAATTATATAAAT
ATAAAGTGGTAGAAATCAAGCCATTA---GGAATAGCACCCACTGGAGCAAAAAGGAGAGTGGTG--------
-------------------------------GAGAGAGAAAAAAGAGCA---GTGGGA---ATGGGAGCT---
------TTGTTCCTTGGGTTCTTGGGAGCA---GCAGGAAGCACTATG---GGCGCAGCGTCA---
CTAACGCTGACGGTACAG---GCCAGACAA---GTATTG---TCTGGTATAGTGCAACAGCAAAGC---
AATTTG---CTGAAG---GCTATAGAGGCGCAACAGCATATGTTGCAACTCACGGTCTGGGGCATT
>JF680927
TTCTATTGCAATACATCAGGGATATTTAATAGTACACAGCTGTTTAATGATACATTAAATAATAGTACAGGA-
-------------------------------------------------------------------------
----------------------------
AATAATGAGACCATCACACTCCCATGCAGAATAAAACAAATTGTAAACATGTGGCAGCAGGTAGGACGAGCAA
TGTATGCCCCGCCCATTGCAGGAAACCTAACATGTAACTCAAAC---
ATCACAGGACTGCTATTGACACGTGATGGAGGAAAAGATAACACAACA-------------------------
-------------------------------------------------------------------------
-------
GATAACAATACAGAGATATTCAGACCTGCAGGAGGAGATATGAGGGATAATTGGAGAAGTGAATTATATAAAT
ATAAAGTGGTAGAAATTAAGCCATTG---GGAATAGCTCCCACTGGGGCAAAAAGGAGAGTGGTG--------
-------------------------------GAGAGACCAAAAAGAGCA---GTGGGA---ATGGGAGCT---
------GTGTTCCTTGGGTTCTTGTCAGCT---GCAGGAAGCACTATG---GGCGCAGCGTCG---
ATGACGCTGACGGTACAA---GCCAGACAA---TTGCTG---TCTGGTATAGTGCAACAGCAAAGC---
AATTTG---CTGAGA---GCTATAGAGGCGCAACAACATTTGTTGCAACTCACAGTCTGGGGCATA
>HQ385458
TTCTATTGCAATACATCAAAACTGTTTAATAATAGTACAAATAGTACAGAA----------------------
-------------------------------------------------------------------------
----------------------------
GGAAATTCAACCATCACACTCCCATGCAGAATAAAACAAATTATAAACCTGTGGCAGGAGGTAGGAAAAGCAA
TATATGCCCCTCCCATTGCAGGAAACATAACATGTGAATCAAAT---
ATCACAGGACTACTCTTGGTACGAGATGGAGGCAATAGTACAAATAAT-------------------------
-------------------------------------------------------------------------
----------
GATACAACAGAGATATTCAGACCAGGAGGAGGAAATATGAAGGACAATTGGAGAAGTGAACTATATAAATATA
AAGTAGTAGAAATTAGGCCATTG---GGGGTAGCACCCACCAAGGCAAAAAGGAGAGTGGTG-----------
----------------------------GAGAGAGAAAAAAGAGCA---GTGGGA---ATAGGAGCT------
---GTGTTCCTTGGGTTCTTGGGAGCA---GCAGGAAGCACTATG---GGCGCAGCGTCA---
ATAACGCTGACGGTACAG---GCCAGACAA---TTGTTG---TCTGGTATAGTGCAACAGCAAAGC---
AATTTG---CTGAGA---GCTATAGAGGCGCAACAGCATATGTTGCAACTCACGGTCTGGGGCATT
>AF286233
TTCTATTGCAATACATCAAAACTGTTTAATAGTACATACCTGTCTAATAGTACATCATATAATAGTACT----
-------------------------------------------------------------------------
----------------------------
TCAAATTCAACCATCATACTCCAATGCAGAATAAAACAAATTATAAACATGTGGCAGGAGGTAGGACGAGCAA
TGTATGCCCCTCCCATTGAGGGAAACATAACATGTAAATCAGAT---
ATCACAGGACTGCTACTGATACGTGATGGAGGCATAAATAATGACACA-------------------------
-------------------------------------------------------------------------
----------
AACGAGACAGAGACATTCAGACCGGGGGGAGGAGATATGAGGGACAATTGGAGAAGTGAATTATATAAATACA
AAGTGGTAGAGATTAAGCCATTA---GGAATAGCACCCACTAAGGCAAAAAGGAGAGTGGTG-----------
----------------------------GAGAGAGAAAAAAGAGCA---GCA------CTAGGAGCT------
```

---ATGTTCCTTGGGTTCTTGGGAGCA---GCCGGAAGCACTATG---GGCGCAGCGTCA---
ATAACGCTGACGGTACAG---GCCAGGCAA---TTGTTG---TCTGGTATAGTGCAACAGCAAAAC---
AATTTG---CTGAGG---GCTATAGAGGCGCAACAGCATATGTTGCAACTCACGGTCTGGGGCATT
>AY255824
TTCTACTGCAATACATCAAACCTGTTTAATAGTACATACAGGTCTAATAGTACTAATAGTACT----------
----

TCAGATACATCCATCATACTCCCATGCAGAATAAAACAAATTATAAACATGTGGCAGGGGGTAGGACGAGCAA
TGTATGCCCCTCCCATTGAAGGAAACATAACATGTAAATCAAAT---
ATTACAGGACTACTATTGACACGTGATGGAGGAACGGACAATGAA----------------------------
----

ACAATAGAGACATTCAGACCTGGAGGAGGAGATATGAGGGATAATTGGAGAAGTGAATTATACAAATATAAAA
TAGTAGAAATTAAGCCATTA---GGAGTAGCTCCAACTGCGGCAAAAAGGAGAGTGGTG--------------
-------------------------GAGAGAGAAAAAAGAGCA---GCGGGA---ATAGGAGCT---------
TTGTTCCTTGGGCTCTTGGGAGCA---GCAGGAAGCACTATG---GGCGCAGCGTCA---
ATAACGCTGACGGTACAG---GCCAGGCAA---TTGTTG---TCTGGTATAGTGCAACAGCAAAGC---
AATTTG---CTGAGG---GCTATAGAGGCGCAACAGCATATGTTGCAACTCACAGTCTGGGGCATT
>KP109481
TTCTATTGCAATACATCAGGCCTGTTTAATAGTACATATATGCCTAATGGTACATATATGCCTAATGGTACAA
ATAAT--

TCAAACTCAACCATCATCCTCCCATGCAGAATAAAACAAATTATAAACATGTGGCAGGAGGTAGGACGAGCAA
TGTATGCCCCTCCCATTGCAGGAAACATAACATGTAACTCAAAT---
ATCACAGGACTACTATTGGTACGAGATGGAGGAAAGAACAATAAT----------------------------
----

ACAGAGATATTCAGACCTGGAGGAGGAGATATGAGGGACAATTGGAGAAGTGAATTATATAAATATAAAGTAG
TGGAGATTAAGCCATTG---GGAGTAGCACCCACTAGAGCAAAAAGGAGAGTGGTG-----------------
----------------------GAGAGAGAAAAAAGAGCA---GTGGGA---CTGGGAGCT---------
GTGTTCCTTGGGTTCTTGGGAGCA---GCAGGAAGCACTATG---GGCGCGGCGTCA---
ATAACGCTGACGGTACAG---GCCAGACAA---TTGTTG---TCTGGTATAGTGCAACAGCAAAGC---
AATTTG---CTGCAG---GCTATAGAGGCGCAACAGCATCTGTTGCAACTCACGGTCTGGGGCATT
>KP109482
TTCTATTGTAATACATCAGGCCTGTTTAATGGTACATATATGCCTACATACATGCCTAATAGTACAAACAGTA
AT---

TCAAGCTCAAACATCACAATCCCATGCAGAATAAAGCAAGTTATAAACATGTGGCAGGAGGTAGGAAGAGCAA
TGTATGCCCCTCCCATTGAAGGAGAAATAACATGTAAATCAAAT---
ATCACAGGACTACTATTGGTACGTGATGGAGGAAATGGAAATGATACA-------------------------
----

AATAAAACAGAGATATTCAGACCTGAAGGAGGAGATATGAGGGACAATTGGAGAAGTGAATTATATAAATATA
AAGTGGTAGAAATTAAGCCATTG---GGAATAGCACCCACTGAAGCAAAAAGGAGAGTGGTG-----------
----------------------------GAGAGAGAAAAAAGAGCA---GTGGGA---ATAGGAGCT------
---GTGATCCTTGGGTTCTTGGGAGCA---GCAGGAAGCACTATG---GGCGCGGCGTCA---
ATAACGCTGACGGCACAG---GCCAGACAA---TTGTTG---TCTGGTATAGTGCAACAGCAAAGC---
AATTTG---CTGAAG---GCTATAGATGCGCAACAGCATCTGTTGCAACTCACAGTCTGGGGCATT
>KP109484
TTCTATTGCAATACATCAAAACTGTTTAATGGTATATACAATGGTACACAAAGTAATTCAAGTAAT-------
----

TCAAACTCAACCATCATAATCCCATGCAAAATAAAACAAATTGTAAACATGTGGCAGAAGGTAGGACGGGCAA
TGTATGCCCCTCCCATTGCAGGAAACATAACATGTACATCAAAT---
ATCACAGGACTACTACTGGTACGTGATGGAGGACCAGATAATGTT----------------------------
----

ACAGAGATATTCAGACCTGGAGGAGGAGATATGAGGGACAATTGGAGAAGTGAATTATATAAATATAAAGTGG
TAGAAATTAAGCCATTG---GGAATAGCACCCACTGGGGCAAAAAGGAGAGTGGTG-----------------
----------------------GAGAGAGAAAAAAGAGCA---GTGGGA---TTAGGAGCT---------

GTGTTCCTTGGGTTCTTGGGAGCA---GCCGGAAGCACTATG---GGCGCAGCGTCA---
ATAACGCTGACGGTACAG---GCCAGACAA---TTGTTG---TCTGGTATAGTGCAACAGCAAAGC---
AATTTG---CTGAGG---GCTATCGAGGCGCAACAGCATATGTTGCAACTCACGGTCTGGGGCATT
>EU521729
TTCTATTGTAATACATCACGCTTGTTTAAGGGTACATACACGCCTAATGGTACAGGAAGTATATCAGAAAGTA
ATTCAAAT---
-------------------TCA---
ACCTCAAACATCAGCATCACAATCCCATGCAGAATAAAGCAAATTATAAACATGTGGCAGGAGGTAGGACGAG
CAATGTATGCCCCTCCCATTGAAGGAAGCATAAAATGTAACTCAACT---
ATCACAGGACTACTATTGGTACGTGATGGAGGACTAGGTAATGAGACA-------------------------
----

AAGACAGAAGAGAGATTCAGACCTGGAGGAGGAGATATGAGGGACAATTGGAGAAGTGAATTATATAAATATA
AAGTGGTAGAGATTAGGCCATTG---GGATTAGCACCCACTAAAGCAAAAAGGAGAGTGGTG-----------
----------------------------GAGAGAGAAAAAAGAGCA---GTAGTGGGAATAGGAGCT------
---ATGTTCCTTGGGCTCTTGAGTGCA---GCTGGAAGCACTATG---GGCGCGGCGGCA---
ATAACGCTGACGGTACAG---ACCAGACAA---TTGTTG---TCTGGTATAGTGCAACAGCAAAGC---
AATTTG---CTGATG---GCTATAGAGGCGCAACAGCATTTGTTGCAACTCACGGTCTGGGGCATT
>EF469243
TTCTATTGCAATACATCAGTACTGTTTAATAGTACATACAACCATACAACAAAGCAGTTTAGTAGTCCATACA
AT---

GATACAAACTCAACCATCACAATCCACTGCAAAATAAAGCAAATTATAAACATGTGGCAGGAGGTGGGGCGAG
CAATATATGCCCCTCCCATTGCAGGAAACATAACATGTAAATCCAAT---
ATCACAGGATTACTATTGGTACGAGATGGAGGAAATACTGAGTCA----------------------------
----

AATAATACAGAAATATTCAGACCTGGAGGAGGAGATATGAGGGACAATTGGAGAAGTGAATTGTACAAATATA
AAGTGGTAGAAGTTAAGCCATTG---GGAATAGCACCAACTGCAGCAAAAAGGAGAGTGGTG-----------
----------------------------GAAAGAGAAAAAAGAGCAGTATTGGGTGGAATAGGAGCT------
---GTGTTCCTTGGGTTCTTGGGAGCG---GCAGGAAGCACTATG---GGCGCGGCGTCA---
ATAACGCTGACGGTACAG---GCCAGACAA---TTATTG---TCTGGTATAGTGCAACAGCAGAGC---
AATCTG---CTGAGA---GCTATAGAGGCGCAACAACATATATTGCAACTCACGGTCTGGGGCATT
>KF766537
TTCTATTGTAATACATCAGGCCTGTTTAATAGTACATACAATGTGACAGACAATGGTACAGAAAGTACTTCA-
----

AACTCAACCACCATCACAATCACATGCAGAATAAAGCAAATTATAAATATGTGGCAGGGGGTAGGACGAGCAA
TATATGCCCCTCCCATTGCAGGAAACATAACATGTAATTCAAGC---
ATCACAGGATTACTATTGGAACGTGATGGAGGAGACGAGACAAAA----------------------------
----

AACGGTACAGAGATATTCAGACCTGGAGGAGGAGATATGAAGCACAATTGGAGAAGTGAATTATATAAATATA
AAGTAGTAGAAATTAAGCCATTG---GGAATAGCACCCACTGCAGCAAAAAGGAGAGTGGTG-----------
----------------------------GAGAGAGAAAAAAGAGCA---GTGGGA---CTAGGAGCT------
---GTGTTCCTTGGGTTCTTGGGAGCA---GCAGGAAGCACTATG---GGCGCAGCGTCA---
ATAACGCTGACGGTACAG---GCCAGACAA---TTGTTG---TCTGGTATAGTGCAACAGCAAAGC---
AATTTG---CTGAAG---GCTATAGAGGCGCAACAGCATCTGTTGCAACTCACGGTCTGGGGCATT
>KF766540
TTCTATTGCAATACAACACAACTGTTTAATAGTACATACATACCTAACATGTCTGATAATACAACAAGTAAT-
----

GAAAGCTCAGACATCACAATCCCATGTAGCATAAGACAATTTATAAACATGTGGCAGAGGGTAGGACGAGCAA
TGTATGCCCCTCCCATTTCAGGAAACATAACATGTAAATCAAAT---
ATAACAGGGCTACTATTGGAACGTGATGGAGGGGATGATAATAAG----------------------------
----

ACAGAGACATTCAGACCTGGAGGAGGAGATATGAGGGACAATTGGAGAAGTGAATTATATAAATGTAAAGTGG
TAGAAATTAAGCCATTA---GGAGTTGCACCCACTGGAGCAAAGAGGAGAGTGGTG-----------------
----------------------GAGAGAGAAAAAAGAGCA---GTGGGA---ATAGGAGCC---------

ATGTTCCTTGGGTTCTTGGGAGCA---GCAGGAAGCACTATG---GGCGCAGCGTCA---
ATAACGCTGACGGTACAG---GCCAGACAA---TTGTTG---TCTGGTATAGTGCAACAGCAAAGC---
AATTTG---CTGAGG---GCTATAGAGGCGCAACAACATCTGTTGCAACTCACAGTCTGGGGCATT
>EU908218
TTCTATTGTAATACGTCAGGCCTGTTTAATAGTACATACATGACTAATGGTACATTTACATACAAGCCT----
----

AATGGTACAAACATCACAATCCCATGCAGAATAAAGCAAATTATAAACATGTGGCAGGAGGTAGGACGAGCAA
TGTATGCCCCTCCCATTGCAGGAAACATAACATGTAAATCAAAT---
ATCACAGGAGTGATATTGGTACGTGATGGAGGGGAAAATGAAAAT----------------------------
----

ATTACAGAAGAGACATTTAGACCTGGAGGAGGAAACATGAGGGACAATTGGAGAAGTGAATTATATAAATATA
AAGTAGTAGAAATTAAGCCATTG---GGAGTAGCACCCACTAAAGCAAAAAGGAGAGTAGTG-----------
----------------------------GAGAGAGAAAAAAGAGCA---GTAGGA---CTAGGAGCT------
---ATGTTCCTTGGGTTCTTGGGAGCA---GCAGGAAGCACTATG---GGCGCGGCGTCA---
ATAACGCTGACGGTACAA---GCCAGACAA---TTGTTG---TCTGGTATAGTGCAGCAGCAAAGA---
ATTTTG---CTGCAG---GCTATAGAGGCGCAACAGCATCTGTTGCAACTCACGGTCTGGGGCATT
>EU521727
TTCTATTGCAACACATCAATCCTGTTTAATGGTACATACAACCCTAATGGTACAAAAAGTAAT----------
----

TCAAGTAATCCCATCACAGTCCCATGCCGCATAAAACAAATTATAAACATGTGGCAGGAGGTAGGACGAGCAA
TGTATGCTCCTCCCATTGAAGGAAACATAACATGCAAATCAAAT---
ATCACAGGACTGCTATTGGTACGTGATGGAGGAGAAGAGTCAAATAAA-------------------------
----

ACTAATACAGAGACATTCAGACCTGGAGGAGGAGATATGAGGGACAATTGGAGAAGTGAATTATATAAATACA
AAGTAGTAGAAATTAAGCCATTA---GGAATAGCACCCACTGAAGCAAAAAGGAGAGTGGTG-----------
----------------------------GAGAGAGAAAAAAGAGCA---GTGGGA---ATAGGAGCT------
---GTGTTCCTTGGATTCTTGGGAGCA---GCAGGAAGCACTATG---GGCGCGGCGTCA---
ATAACGCTGACGGTACAG---GCCAGACAA---TTGCTG---TCTGGTATAGTGCAACAGCAAAGC---
AATTTG---CTGAGG---GCTATAGAGGCGCAACAGCATATGTTGCAACTCACGGTCTGGGGCATT
>KC156210
TTCTATTGTAATACATCAGGCCTGTTTAATAGGACATATACAACAGACCTGTTTAATAAGACATATAATGATA
ATGATACA---
-------------GAAGAT------
AATTCAGGCCCAGTCATCATAATCCCATGTAGAATAAAGCAAATTATAAACATGTGGCAGGAGGTAGGACGAG
CAATGTATGCCCCTCCCATTGCAGGAAACATAACATGTAACTCAAGC---
ATCACAGGTCTGCTATTGACACGGGATGGAGGCAATGACAATAAAACGTCA----------------------
----

ACGAATGAGACATTCAGACCTCTAGGAGGAAATATGAGGGACAATTGGAGAAATGAATTATATAAGTATAAAG
TGGTAGAAATTAAGCCACTA---GGAATAGCACCCACTACAGCAAAAAGGAGAGTGGTG--------------
-------------------------GAGAGAGAAAAAAGAGCA---GTGGGAATGATGGGAGCT---------
ATGTTCCTTGGGTTCTTGGGAGCT---GCAGGAAGCACTATG---GGCGCGGCGTCA---
ATAACGCTGACGGTACAG---GCCAGGCAA---TTGTTG---TCTGGTATAGTGCAACAGCAAAGC---
AATTTG---CTGAGG---GCTATAGAGGCGCAACAGCATCTGTTGCAACTCACGGTCTGGGGCATT
>KY929361
TTCTATTGCAATACATCAAGTCTATTTAATAGCACATACATTAATGGCACATACATTAATGGCACAGTAAGTA
ATTCAGGC---

TCAGACTCAAACATCACAATCCCATGTAGAATAAAACAAATTGTAAGAATGTGGCAGCAGGTAGGACAAGCAA
TATATGCCCCTCCTATTGAAGGAAACATAACATGTAAATCAAAT---
ATCACAGGACTACTCTTAGTACGTGATGGAACAATTACAGGTGAGGCA-------------------------
----

AATGATACAGAGACATTCAGACCTGGAGGAGGAGAGATGAGGGATAATTGGAGAAGTGAATTATATAAATATA
AAGTGGTAGAAATTAAACCATTA---GGAATAGCACCCACTGGATCAAAAAGGAGAGTGGTG-----------
----------------------------GAGAGAGAGAAAAGAGCA---GCA------GTGTCAGCT------

---ATGCTCTTTGGGTTCTTGGGATTG---GCAGGAAGCACTATG---GGCGCGGCGTCA---
ATATCGCTGACGGTACAG---GCCAGACAA---TTGTTG---TCTGGTATAGTGCAACAGCAAAGC---
AATTTG---CTGAGA---GCTATAGAGGCGCAACAGCATCTGTTGCAACTCACAGTCTGGGGCATT
>KY929362
TTCTATTGCAATACATCAAAACTGTTTAATAGAAAATACCTGCCTGATGGTACAGACAACACTACGGATACAG
ATGCA--

AACAATACAGACATTACAATCCCATGTAGGCTAAAACAATTTATAAACATGTGGCAGGGGGTAGGGAGAGCGA
TGTATGCCCCTCCCATTGCAGGAAACATAACGTGTATATCAAAT---
ATCACAGGGATACTATTGACACGTGATGGAGGAAATAATAATAAT----------------------------
----

AGTAATAATGAGACATTCAGACCGGGAGGAGGAGATATGAGGGACAATTGGAGAAGTGAATTATATAAATATA
AAGTGGTAGAAATTAAACCATTG---GGAATAGCACCCACTACAGCAAAAAGGAGAGTGGTG-----------
----------------------------GAGAGAGAAAAAAGAGCA---GTGGGA---CTAGGAGCT------
---ATGTTCCTTGGGTTCTTGGGAGCA---GCAGGAAGCACTATG---GGCGCGGCGTCA---
ATAACGCTGACGGTACAG---GCCAGACAA---TTGTTG---TCTGGTATAGTGCAACAGCAAAGC---
AATTTG---CTGAGA---GCTATAGAGGCGCAACAGCATCTGTTGCAACTCACAGTCTGGGGCATT
>KY929366
TTCTATTGCAATACATCAAGCCTGTTTAATGCTACATACAAAAATGGTACATTCATTAATAATACCACC----
----

CTCAAATCAAACATGACCATCCCCTGCAGAATAAGACAATTTATAAACCTGTGGCAGGAGGTAGGAAAAGCAA
TGTATGCCCCTCCCATTGCAGGGAACATAACATGTAGATCAAAT---
ATCACAGGAATACTATTGACAAGTGATGGAGGAATAAGAAATGACACA-------------------------
----

AATAATAATGAGACATTCAGACCTGCAGGAGGAGATATGAGGGACAATTGGAGAAGTGAATTATACAAATATA
AAGTGGTAGAAATTCGGCCATTG---GGAGTAGCACCCACTACAGCAAAAAGGAGAGTGGTG-----------
----------------------------GAGAGAGAAAAAAGAGCA---GTGGGA---CTAGGAGCT------
---ATGTTCCTTGGGTTCTTGGGAACA---GCCGGAAGCACTATG---GGCGCAGCGTCA---
ATGGCGCTGACGGTACAG---GCCAGACAA---TTGTTG---TCTGGTATAGTGCAACAGCAAAGC---
AATTTG---CTGAGG---GCTATAGAGGCGCAACAACACATGTTGCAACTCACGGTCTGGGGCATT
>KY929368
TTCTATTGTAATACATCAGGCCTGTTTAATAGTACATACATTACATACATTAATGGTAACATGTCTATATACA
ATGGTAGT---
-------------GGAAAC------
TCCAATAACACAAACATCACACTCCCATGCCGCCTAAAGCAAATTATAAACATGTGGCAGAGGGTAGGACGAG
CAATGTATGCCCCTCCCATTCCAGGAAACATAACATGTAACTCAACT---
ATCACAGGACTACTATTGGTACGTGATGGAGGAAACGATAATAGAACA-------------------------
----

AATGGGACAGAGATATTTAGACCTGGAGGAGGAGATATGAGGGACAATTGGAGAAGTGAACTATATAAATATA
AAGTGGTAGAAATTAAACCATTG---GGACTAGCACCCACTAAAGCAAAAAGGAGAGTGGTG-----------
----------------------------GAGAGAGAAAAAAGAGCA---GTGGGA---ATAGGAGCT------
---TTGCTCTTTGGGTTCTTGGGAATG---GCAGGAAGCACTATG---GGCGCGGCGTCA---
ATAACGCTGACGGTACAG---GCCAGAGAC---ATATTG---TCTGGTATAGTGCAACAGCAAAGT---
AATTTG---CTGAGA---GCTATAGAGGCGCAACAGCATCTGTTGCAACTCACGGTCTGGGGCATT
>KX069227
TTCTATTGCAATACATCAGGTCTATTTAATGGTACATACAATGGTACAGAAAGTGAT----------------
----

TTAAACTCAACCATCACAATCCCATGCAAAATAAAACAAATTATAAACATGTGGCAGGAGGTAGGAAGAGCAA
TGTATGCCCCTCCTATTGAAGGAAACATAACATGTAAATCAAAT---
ATCACAGGACTACTATTAGAACGTGATGGAGGACAGGGAAATGGGGCAGAA----------------------
----

AATGATACAGAGATATTCAGACCTGGAGGAGGAGACATGAGGGATAATTGGAGAAGTGAATTATATAAATATA
AAGTGGTAGAAATTAAACCATTA---GGAGTAGCACCCACTGGATCAAAAAGGAGAGTGGTG-----------
----------------------------GAGAGAGAAAAAAGAGCA---GCA------GTGTCAGCT------

---ATGCTCTTTGGGTTCTTGGGATTG---GCAGGAAGCACTATG---GGCGCGGCGTCA---
ATATCGCTGACGGTACAG---GCCAGACAA---TTGTTG---TCTGGTATAGTGCAACAGCAAAGC---
AATTTG---CTGAGA---GCTATAGAGGCGCAACAGCATCTGTTGCAACTCACAGTCTGGGGCATT
>KX069228
TTCTATTGCAATACAACAAAGTTGTTTAATAGTACATACAGTACATACAATGATACAAAAAATTCA-------
----

ACAAACTCAACCATCACAATCCCATGCAGAATAAAACAAATTATAAACATGTGGCAGGAGGTAGGACGAGCAA
TGTATGCCCCTCCCATTGCAGGATACTTAACATGTAAGTCAAAT---
ATCACAGGACTACTATTGACACGTGATGGAGGAATAGAGGATAAGAATACT----------------------
----
AGTACAGGTAGTAATGACACA--------
AAGACAGAGACATTCAGACCTATAGGAGGAGATATGAGGGACAATTGGAGAAGTGAATTATATAAATACAAAG
TAGTAGAAGTTAAGCCATTG---GGAATAGCACCCACTGAAGCAAAAAGGAGAGTGGTG--------------
-------------------------GAGAGAGGAAAAAGAGCA---GTAACA---ATGGGAGCT---------
ATGTTCCTTGGGTTCTTGGGAGCA---GCAGGAAGCACTATG---GGCGTGGCGTCA---
ATGGCGCTGACGGTACAG---GCCAGACAA---TTGTTG---TCTGGTATAGTGCAACAGCAAAGC---
AATTTG---CTGAGA---GCTATAGAGGCGCAACAGCATCTGTTGCAACTCACAGTCTGGGGCATT
>KX069222
TTCTATTGTAATACATCAAAACTGTTTAATAGTACATACCTGTCTAATGGCACATACATGCCTAATGAGGCAC
TA---

AATAGTACTTCCATCACGATCCCATGCAGAATAAAGCAAATTATAAACATGTGGCAGGAGATAGGACGAGCAA
TGTATGCCCCTCCCATTGCAGGAAACATAACATGTACATCAAAT---
ATCACAGGACTACTATTGGTACGTGATGGGGGAAAAGAGAAAAAT----------------------------
----

GAAACAGAAATATTCAGACCGGGAGGAGGAAACATGAAGGACAATTGGAGAAGTGAATTATATAAATATAAAG
TGGTAGAGATTAAACCATTG---GGAGTAGCACCCACTGGAGCAACAAGGAGAGTGGTG--------------
-------------------------AAGAGAGAGAAAAGAGCA---GCC------ATAGGAGCT---------
GTGTTCCTTGGGTTCTTGGGAGCA---GCAGGAAGCACTATG---GGCGCGGCGTCA---
ATGGCGCTGACGGTACAG---GCCAGACAA---TTGTTG---TCTGGTATAGTGCAACAGCAAAGC---
AATTTG---CTGAGG---GCTATAGAGGCGCAACAGCATCTGTTGCAACTCACGGTCTGGGGCATT
>KX756601
TTCTATTGTAATACATCAAGCCTGTTTAATAGGACATACATATTTAATGAGACAACAAGTAATTCAAGCTCA-
----

AATAGTTCACACATCACAATCCAATGCAGAATAAAGCAAATTATAAACATGTGGCAGGAGGTAGGACGAGCAA
TGTATGCCCCTCCCATTGAAGGAACCATAACATGTAAATCAAAC---
ATCACAGGGATACTATTGACAAGGGATGGTGGAACACATAATGGA----------------------------
----

ACAGAGACATTCAGACCGGGAGGAGGAAATATGAAGGACAATTGGAGAAATGAATTATATAAGTATAAAGTGG
TAGAAATCAAGCCACTG---GGAGTAGCACCCACTGGAGCAAAAAGGAGAGTGGTG-----------------
----------------------GAGAGAGAAAAAAGAGCA---GTGGGA---CTAGGAGCT---------
ATGTTCCTTGGGATCTTAGGAGCA---GCAGGAAGCACTATG---GGCGCGGCGTCA---
ATAACGCTGACGGTACAG---GCCAGACAA---TTGTTG---TCTGGTATAGTGCAACAGCAAAGC---
AATTTG---CTGAGA---GCCATAGAGGCGCAACAGCATATGTTGCAACTCACAGTCTGGGGCATT
>KX069219
TTCTATTGCAATACATCAAGCCTATTTAATAGTACATACATGTCCAATGGTACATACATGTTTAATGGGACAA
TAAGTAAT---
-------------GGGACA------
ATAAGTAATGATAGCATCACAATCCCATGCAGGATAAAACAAATTGTAAACATGTGGCAGCAGGTAGGACGAG
CAATGTATGCCCCTCCCATTGCAGGAAACATAACGTGTAACTCAAAC---
ATTACAGGAATATTATTGGAACGTGATGGAGGGTTAAGTAATAGG----------------------------
----

TCAGAGGAGACATTCAGACCTGTAGGGGGAGATATGAAGAACAATTGGAGAAGTGAATTATATAAATATAAAG
TGGTAGAAATTAAACCATTG---GGAGTAGCACCCACTGCAGCAAAAAGGAGAGTGGTG--------------
-------------------------GAGAGAGAAAAAAGAGCA---GTGGGC---CTAGGAGTT---------

ATGTTCCTTGGGTTCTTGGGAGCA---GCAGGAAGCACTATG---GGCGCGGCGTCA---
ATAACGCTGACGGTACAG---GCCAGACAA---TTGCTG---TCTGGTATAGTGCAGCAGCAAAGC---
AATTTG---CTGAGG---GCTATAGAGGCGCAACAGCATTTGTTGCAACTCACAGTCTGGGGCATT
>KX069221
TTCTATTGTAATACATCAAAACTGTTTAAGGGTAATGGTACAAGCACAGACACAGGA----------------
----

AATAATTCAAGCATCACAATCCCATGCAGAATAAAGCAAATTATAAATATGTGGCAGGGGGTAGGACGAGCAA
TGTATGCCCCTCCCATTGAAGGACACATAACATGTGAATCAAAT---
ATCACAGGACTACTATTGGTACGTGATGGGGGAGCATCAAATGAGACAAGT----------------------
----

AATGATACAGAGACATTCAGACCTGGAGGAGGAAATATGAAGGACAATTGGAGAAGTGAATTATATAAATATA
AAGTGGTAGAAATTAAACCATTG---GGAGTAGCACCCACTGAAGCAAAAAGGAGAGTGGTG-----------
----------------------------GAGAGAGAAAAAAGAGCA---GTG------TTGGGAGCT------
---GCGATCCTTGGGTTCTTGGGAGCA---GCAGGAAGCACTATG---GGCGCGGCGTCA---
ATAACGCTGACGGTACAG---GCCAGACAA---TTGCTG---TCTGGTATAGTGCAACAGCAAAGC---
AATTTG---CTGAGA---GCTATAGAGGCGCAACAGCATCTGTTGCAACTCACAGTCTGGGGCATT
>KX069223
TTCTATTGCAATACATCAAGACTGTTTAATGGAACATATGATGGAACATATAATGGTACAGCAAGTAAC----
----

AATACAGGAAACATCACCCTCCCATGCAGAATAAAACAAATTATAAACATGTGGCAGGGGGTAGGACGAGCAA
TATATGCCCCTCCCATTGCAGGAAACATAACATGTGTATCAAAT---
ATCACAGGACTATTATTGGTACGTGATGGAGGAACAGAGAATGGCACA-------------------------
----

AATGGCACAGAAACATTCAGACCTACAGGAGGAAATATGAGGGACAATTGGAGAAGTGAATTATATAAATATA
AAGTGGTAGAAATTAAGCCATTG---GGAATAGCACCCACTGCAGCAAGAAGGAGAGTGGTG-----------
----------------------------GAGAGAGGAAAAAGAGCA---GTGGGA---ATAGGAGCT------
---GTGTTCCTTGGGTTCTTGGGAGCA---GCAGGAAGCACTATG---GGCGCGGCGTCA---
ATAACGCTGACGGTACAG---GCCAGACAA---TTGTTG---TCTGGTATAGTGCAGCAGCAAAGC---
AATTTG---CTGAGA---GCTATAGAGGCGCAACAGCATCTGTTGCAACTCACGGTCTGGGGCATT
>KX069224
TTCTATTGCAATACATCAAAACTGTTTAATAGTACATATAATGGTACAGACAATGGTAACTCAAGCAGCTCAA
AC---

TCAAGCTCAATCATCACAATCGCATGCAGACTAAAACAAATTGTAAAACTGTGGCAGGAGGTAGGACGAGCAA
TGTATGCCCCCCCCATTGCAGGAAACATAACGTGCAAATCAAAT---
ATCACAGGAATACTATTGGTGCGTGATGGAGGGACAAATGGGACA----------------------------
----

GAGGAGACCGAGACATTTAGACCTGGAGGAGGAAATATGAGGGATAATTGGAGAAGTGAATTGTATAAATATA
AAGTGGTAGAAATTAAGCCATTG---GGAATAGCACCCACTACAGCAAAAAGGAGAGTGGTG-----------
----------------------------GAGAGAGAAAAAAGAGCA---GTGGGA---ATAGGAGCT------
---GTGTTCCTTGGGTTCTTGGGAGCA---GCAGGAAGCACTATG---GGCGCGGCGTCA---
ATGGCGCTGACGGTACAG---GCCAGACAA---TTGTTG---TCTGGTATAGTGCAACAGCAAAGC---
AATTTG---CTGAGG---GCTATAGAGGCGCAACAGCATCTGTTGCAACTCACGGTCTGGGGCATT
>KX069226
TTCTACTGCAACACAACAAACCTGTTTAATAGTACATACCTAGCGAATAGTACATTA----------------
----

AGTAATTCAACCATCACAATCCCATGCAGCATAAAACAAGTGATAAACATGTGGCAGGAGGTAGGACGAGCAA
TGTATGCCCCTCCCATTGCAGGAAACATAACCTGTAAATCAAAT---
ATCACAGGGCTACTATTGGTACGTGATGGGGGAACAAACAATAGC----------------------------
----

ACTACAGAGGTATTCAGACCTGGAGGAGGAGATATGAGGGACAATTGGAGAAGTGAATTATATAAATATAAAG
TGGTAGAAATTAAGCCATTA---GGAATAGCACCCACTGAAGCAAAAAGGAGAGTGGTG--------------
-------------------------GAGAGAGAAAAAAGAGCA---GTGGGA---ATAGGAGCT---------

GTGTTCCTTGGGTTCTTGGGAGCA---GCAGGAAGCACTATG---GGCGCGGCGTCA---
ATAACGCTGACGGTACAG---GCCAGACAA---TTGTTG---TCTGGTATAGTGCAACAGCAAAGC---
AATTTG---CTGAGG---GCTATAGAGGCGCAACAGCATCTGTTGCAACTCACGGTCTGGGGCATT
>KY713230
TTCTATTGCAATACATCAGGCCTGTTTAATGGTACATATAATAGTACATACAATGGTACATCCATG-------
----

TCTAATGATACCATCACACTCCCATGCAGAATAAAACAAATTATAAACATGTGGCAGGAGGTAGGAAGAGCAA
TGTATGCCCCTCCCATTGCAGGAAACATAACATGTAAATCAAAT---
ATCACAGGAATACTATTGGTACGTGATGGAGGACCAAATAGGACA----------------------------
----

AATGAGACATTCAGACCTGGAGGAGGAGATATGAGAGACAATTGGAGAAGTGAATTATATAAATACAAAGTGG
TAGAAGTTAAGCCATTG---GGAATAGCACCCACTACAGCAAAAAGGAGAGTGGTG-----------------
----------------------GAGAGAGAAAAAAGAGCA---GTGGGA---CTAGGAGCT---------
GTGTTCCTTGGGTTCTTGGGCGCA---GCAGGAAGCACTATG---GGCGCGGCGTCA---
ATAACGCTGACGGTACAG---GCCAGACAA---TTGTTG---TCTGGTATAGTGCAACAGCAAAAC---
AATTTG---CTGAAA---GCTATAGAGGCGCAACAACATCTGTTGCAACTCACGGTCTGGGGCATT
>KY713236
TTCTATTGTAATACATCAGACCTGTTTATTGGTACATATAAGTTTATTAATGGTACATATGAGTTTAATGATA
CAACAAGTAAT--

TCAAGCAGATCAGACATCACAATCCAATGCAGAATAAAGCAATTTATAAACATGTGGCAGGAGGTAGGAAAAG
CAATGTATGCCCCTCCCATTGAAGGAAACATAACATGTAAATCAAAC---
ATCACAGGACTAATATTGGTACGTGATGGAGGGCTAAATACCAAT----------------------------
----

GAGACAAATAATACAGAGATATTCAGACCTGGAGGAGGAGATATGAGGGACAATTGGAGAAGTGAATTATATA
AATATAAAGTGGTAGAAATTAAGCCATTA---GGAATAGCACCCACTGCAGCAAAAAGGAGAGTGGTG-----
----------------------------------GAGAGACCAAGAAGAGCA---GTGGGA---
CTAGGAGCT---------GTGTTCCTTGGGTTCTTGGGAGCA---GCCGGAAGCACTATG---
GGCGCGGCGTCA---ATAACGCTGACGGTACAG---GCCAGACAA---TTGTTG---
TCTGGTATAGTGCAACAGCAAAGC---AATTTG---CTGAGG---
GCTATAGAGGCGCAACACCATCTGTTGCAACTCACAGTCTGGGGCATT
>MN703367
TTCTATTGCAATACATCAAAACTGTTTAGTGGTGTATATAATGGTACATATAATGTTACAACAGGTAATTCAA
CAGGA--

GATAATTCAACTATCACAATCCCATGCAGAATAAGGCAAGTTATAAACCTGTGGCAGGAGGTAGGAAGAGCAA
TATATGCCCCTCCCATTGCAGGAAACATAACATGTAAATCAAAT---
ATCACAGGAATACTGTTAACGCGTGATGGAGGGCAAGGGGCAAAC----------------------------
----

ATTACAAATGATACAGAGACATTTAGACCTGCAGGAGGAGATATGAGGGACAATTGGAGAAGTGAACTATATA
AATATAAAGTGGTAGAAATTAGGCCATTA---GGAATAGCACCCACTCCAGCAAAAAGGAGAGTAGTG-----
----------------------------------GAGAGAGAGAAAAGAGCA---GCGGGA---
TTAGGAGCT---------GTGCTCCTTGGGTTCTTGGGAGCA---GCCGGAAGCACTATG---
GGCGCAGCGTCA---ATAACGCTGACGGTACAG---GCCAGACAA---TTGTTG---
TCTGGTATAGTGCAACAGCAAAGC---AATTTG---CTGAAG---
GCTATAGAGGCGCAACAACACATGTTGCAACTCACTGTCTGGGGCATC
>MN703351
TTCTATTGTAATACATCAAAACTGTTTAATGGTACATATAAAAGTGATTCAAACTCAACC-------------
----

TCAGACAAAAACATCACAATCCCATGCAGAATAAAGCAAATTATAAACATGTGGCAGGAGGTAGGACGAGCAA
TGTATGCCCCTCCCATTGCAGGAAGCATAATATGTAAATCACAA---
ATCACAGGACTCCTATTGGTACGTGATGGAGGAGTAACAAATGACACAGAAAAT-------------------
----

AATGATACAGAGATATTCAGACCTGGAGGAGGAGATATGAAGAACAATTGGAGAAGTGAATTATATAAATATA

AAGTAGTAGAGATTAAGCCATTG---GGAGTAGCACCCACTGGAGCAAAAGGGAGAGTAGTG-----------
----------------------------GAGAGAGAAAAAAGAGCA---GTGGGA---CTGGGAGCT------
---GTGTTCCTCGGGTTTCTGGGAGCA---GCAGGAAGCACTATG---GGCGCGGCGTCA---
ATGACGCCGACGGTACAG---GCCAGACAA---TTGTTA---TCTGGTATAGTGCAACAGCAAAAC---
AATTTG---CTAAAA---GCTATAGAGGCGCAACAGCATCTGTTGCAACTCACAGTCTGGGGCATT
>MN703360
TTCTATTGTAATACATCAGGCCTGTTTAATAGTATATATAATACAACAAGTAATTCAAGCTCCAAT-------
----
----------------------------TCAAATGAA---
ACCAGAATCCCATGCAGAATAAAGCAAATTATAAACATGTGGCAGGGGGTAGGACGAGCAATGTATGCCCCTC
CCATTGCAGGAAACATAACATGTAAATCAAAT---
ATCACAGGACTACTATTGGCACGAGATGGAGGAAAAGAGACAAAT----------------------------
----

GAGACAAGCGCGACAGAGATATTCAGACCTGGGGGAGGAAATATGAAGGACAACTGGAGAAGTGAATTATATA
AATATAAAGTGGTAGAAATTAAACCATTA---GGAATAGCACCCACTAAAGCAAAAAGGAGAGTGGTG-----
----------------------------------GAGAAAGGAAAAAGAGCA---GCGGGA---
ATGGCAGCT---------GTGTTCCTTGGGTTCTTGGGAGCA---GCCGGAAGCACTATG---
GGCGCGGCGTCA---ATAGCGCTGACGGTACAG---GCCAGACAA---TTGTTG---
TCTGGTATAGTGCAACAGCAAAGC---AATTTG---CTGAGG---
GCTATAGAGGCGCAACAGCATCTGTTGCAACTCACGGTCTGGGGCATT
>MN703391
TTCTATTGTAATACATCAGGCCTGTTTAATGATACATATGATACAACAGTTAATTCAAGCCCCACT-------
----

TCAAACGAAACCATCACAATCCTATGCAGAATAAAGCAAATTATAAACATGTGGCAGAAGGTAGGACGAGCAA
TGTATGCCCCTCCCATTGCAGGAAACATAACATGTAGATCAAAT---
ATCACAGGGCTACTATTGGTACGTGATGGAGGGATAAATGTGACAAGCAACACA-------------------
----

ACAGACAACATGACAGAGACATTCAGACCTGGAGGAGGAGATATGAGGGACAATTGGAGAAGTGAATTATACA
AATATAAAGTGGTAGAAATTAAGCCATTG---GGAATAGCACCCACTAAAGCAAAAAGGAGAGTGGTG-----
----------------------------------GAGAGAGAAAAAAGAGCA---GTGGGA---
ATGGCAGCT---------GTGTTCCTTGGGTTCTTGGGAGCA---GCCGGAAGCACTATG---
GGCGCGGCGTCA---ATAGCGCTGACGGTACAG---GCCAGACAA---TTGTTG---
TCTGGTATAGTGCAACAGCAAAAC---AATTTG---CTGAGA---
GCTATAGAGGCGCAACAGCATTTGTTGCAACTCACAGTCTGGGGCATC
>AF067154
TTCTATTGCAATACATCAAGCCTGTTTGATAGCCTGTTTAATCCTAATGGTACAAGAAATGAT----------
----

ACAAACTTAACCATTACAATCCCATGCAGAATAAAACAAATTATAAACATGTGGCAGGAGGTAGGACGAGCAA
TGTATGCCCCTCCCATTGCAGGAAACATAACATGTAAATCAAAC---
ATCACAGGACTACTATTGGTGCGTGATGGAGGAAGAGGTAATGATACA-------------------------
----

GAGAATAATACAGAGATATTCAGACCTGGAGGAGGAGATATGAGGAACAATTGGAGAAGTGAATTATATAAAT
ATAAAGTGGTAGAAATTAAGCCATTG---GGAGTAGCACCCACTAAAGCAAAAAGGAGAGTGGTG--------
-------------------------------GAGAGAGAAAAAAGAGCA---GTAGTGGGACTAGGAGCT---
------GTGTTCCTTGGGTTCTTGGGAGCA---GCAGGAAGCACTATG---GGCGCGGCGTCA---
ATAACGCTGACGGTACAG---GCCAGACAA---TTGTTG---TCTGGTATAGTGCAACAGCAAAGC---
AATTTG---CTGAGG---GCTATAGAGGCGCAACAGCATCTGTTGCAACTCACGGTCTGGGGCATT
>JQ715368
TTCTATTGTAATACATCAGGCCTGTTTAATGGTACATACATGTCTAATGGTACAGAAAGTAAT----------
----

TCAAGCTCAATCATCACAATCCCATGCAGAATAAAGCAAATTATAAACATGTGGCAGGAGGTAGGACGAACCA
TGTATGCCCCTCCCATTAAAGGAAACATAGCATGTAAATCAAAT---
ATCACCGGACTACTATTGGTACGTGATGGAGGAACAGAGCCAAAT----------------------------
----

GATACAGAGACATTCAGACCTGGAGGAGGAGATATGAGGAACAATTGGAGAAGTGAATTATATAAATATAAAG
TGGTAGAAATTAAGCCATTG---GGAATAGCACCCACTGCAGCAAAAAGAAGAGTGGTG-------------
-------------------------GAGAGAGAAAAAAGAGCA---GTGGGA---CTGGGAGCT---------
GTGTTCCTTGGGTTCTTGGGAGCA---GCAGGAAGCACTATG---GGCGCGGCGTCA---
ATAACGCTGACGGCACAG---GCCAGACAA---TTGTTG---TCTGGTATAGTGCAACAGCAAAGC---
AATTTG---CTGAGG---GCTATAGAGGCGCAACAGCATCTGTTGCAACTCACGGTCTGGGGCATT
>AF286223
TTTTATTGTAATACATCAAGACTGTTTAACGGTACATACAATGGTACAGACATGCCTACATACAATGGTACAA
AT---

TCCAGTTCAGACATCATCATGCTTCCATGCAGAATAAGGCAATTTATAAACATTTGGCAGAAGGTAGGACGAG
CAATGTATGCCCCTCCCATTGAAGGAAACATAACATGTGAATCAAAT---
ATCACAGGACTACTATTAGTACGTGATGGAGGCGACACAAATAGT----------------------------
----

AGCACAGAGATATTCAGACCTGGAGGAGGAGATATGAGGGACAATTGGAGAAGTGAATTATATAAATATAAAG
TGGTAGAAATTAAGCCATTA---GGAATAGCACCTACTGAAGCAAAAAGGAGAGTGGTG-------------
-------------------------GAGAGAGAAAAAAGAGCA---GTGGGA---ATAGGAGCT---------
GTGTTCCTTGGGTTCTTGGGAGTA---GCAGGAAGCACTATG---GGCGCGGCGTCA---
ATGACGCTGACGGTACAG---GCCAGACAA---TTGTTG---TCTGGTATAGTGCAACAGCAAAGC---
AATTTG---CTGAAG---GCTATAGAGGCGCAACAGCATATGTTGCAACTCACAGTCTGGGGCATT
>AF067155
TTCTATTGCAATACATCAGGCCTGTTTAATAGGACATACATGCCTAATGATACAAAGAGTAATTCAAGCAGTA
AT---

CCAAACGCAAACATCACAATCCCATGCAGAATAAAGCAAATTATAAACATGTGGCAGGAGGTAGGACGAGCAA
TGTATGCCCCTCCCATTGAAGGAAAAATAACATGTAGATCAAAT---
ATCACAGGACTACTATTGGTACGTGATGGAGGAGAAGACAAAAATAATACA----------------------
----

GAGACAAATAAGACGGAGACATTCAGACCTGGAGGAGGAGATATGAGGGACAATTGGAGAAGTGAATTATATA
AATACAAAGTGGTAGAAGTTAAGCCATTG---GGAGTAGCACCCACTACAGCAAAAAGGAGAGTGGTG-----
------------------------------------GAGAGAGAAAAAAGAGCA---GTGGGA---
ATAGGAGCT---------GTGTTCCTTGGGTTCTTGGGAGCA---GCAGGAAGCACTATG---
GGCGCGGCGTCA---ATAACGCTGACGGTACAG---GCCAGACAA---TTGTTG---
TCTGGTATAGTGCAACAGCAAAGC---AATTTG---CTGAGG---
GCTATAGAGGCGCAACAGCATCTGTTGCAACTCACGGTCTGGGGCATT
>AF286231
TTCTATTGCAGCACATCAAGACTGTTTAATAGTACATACATGCCTAATGATACAAAAAGTAAG----------
----

TCAAACAAAACCATCACAATCCCATGCAGCATAAAACAAATTGTAAACATGTGGCAGGAGGTAGGACGAGCAA
TGTATGCCCCTCCCATTGAAGGAAACATAACCTGTAGATCAAAT---
ATCACAGGAATACTATTGGTACGTGATGGAGGAGTAGATTCAGAAGATCCA----------------------
----

GAAAATAATAAGACAGAGACATTCCGACCTGGAGGAGGAGATATGAGGAACAATTGGAGAAGTGAATTATATA
AATATAAAGCGGCAGAAATTAAGCCATTG---GGAGTAGCACCCACTCCAGCAAAAAGGAGAGTGGTG-----
------------------------------------GAGAGAGAAAAAAGAGCA---GTAGGA---
TTAGGAGCT---------GTGTTCCTTGGATTCTTGGGAGCA---GCAGGAAGCACTATG---
GGCGCAGCGTCA---ATAACGCTGACGGTACAG---GCCAGACAA---TTGTTG---
TCTGGTATAGTGCAACAGCAAAGC---AATTTG---CTGAGG---
GCTATCGAGGCGCAACAGCATCTGTTGCAACTCACGGTCTGGGGCATT
>AF286232
TTCTATTGTAACACATCAGGTCTGTTTAATGGTACATACAATCCTAATGTTACAGAAAGTAATTCAAACTCA-
----

ACAAATGCAACCATCACACTCCCATGCAGAATAAAGCAAATTATAAATTTGTGGCAGGAGGTGGGACGAGCAA
TGTATGCCCCTCCCATTGCAGGAAACATAACATGTAAATCAAAT---

ATCACAGGACTATTATTGACACGTGATGGAGGAACAAATGATACA----------------------------
----

AGTAAGACAGAGATATTCAGACCTGGAGGAGGAGATATGAGGGACAATTGGAGAAGTGAATTATACAAATATA
AAGTGGTAGAAGTTAAGCCATTG---GGAGTAGCACCCACTGGAGCAAAAAGGAGAGTGGTG-----------
----------------------------GAGAGAGAAAAAAGAGCA---GTGGGA---ATAGGAGCT------
---GTGTTCCTTGGGATCTTGGGAGCA---GCAGGAAGCACTATG---GGCGCGGCGTCA---
ATAACGCTGACGGTACAG---GCCAGACAA---TTGTTG---TCTGGTATAGTGCAACAGCAAAGC---
AATTTG---CTGAGA---GCTATAGAGGCGCAACAGCATCTGTTGCAACTCACAGTCTGGGGCATT
>KP109486
TTCTATTGCAATACATCAAGCCTGTTTAACAGTACATACAATCCTAATGATACAAACAGTAATTCAAGCTCAA
GTAAT--

TCAAGCTTAGACATCACAATCCCATGCAGAATAAAACAAATTATAAACATGTGGCAGGAGGTAGGACGAGCAA
TGTATGCCCCTCCTATTGAAGGAAACATAACATGTAAATCAAAT---
ATCACAGGACTACTATTGGTACGTAATGGAGGAATAGAGTCAAAT----------------------------
----

GAAACAGAGATATTCAGACCTGGAGGGGGAGATATGAGGAACAATTGGAGAAGTGAATTATATAAATATAAGG
TGGTGGAAATTAAGCCATTG---GGAATAGCACCCACTGCAGCAAAAAGGAGAGTGGTG--------------
-------------------------GAGAGAGAAAAAAGAGCA---GTGGGA---CTGGGAGCT---------
GTGATCTTTGGGTTCTTGGGAGCG---GCAGGAAGCACTATG---GGCGCGGCGTCA---
ATAACGCTGACGGTACAG---GCCAGACAA---TTGTTG---TCTGGTATAGTGCAACAGCAAAGC---
AATTTG---CTGAAG---GCTATAGAAGCGCAACAACATTTGTTGCAGCTCACGGTCTGGGGCATT
>KP109487
TTCTATTGTAATACATCAGGCCTGTTTAATGGTACATTTAATGGTACATACGTGTCACCTAATAGTACAGACA
GTAAT--

TCAAGTTCAATCATCACGATCCCCTGCAGAATAAAGCAAATTATAAACATGTGGCAGGAGGTAGGACGAGCAA
TGTATGCCCCTCCCATTGCAGGAAACATAACATGCAAATCAAAT---
ATCACAGGACTACTATTGGTACGCGATGGGGGAACAGGGTCAGAGTCA-------------------------
----

AATAAGACAGAGATATTCAGACCTGGAGGAGGAGATATGAGGGACAATTGGAGAAGTGAATTATATAAATATA
AAGTGGTAGAAATTAAGCCATTG---GGAGTAGCACCCACTAAAGCAAAAAGGAGAGTGGTG-----------
----------------------------GAGAGAGAAAAAAGAGCA---GTGGGA---ATAGGAGCT------
---GTGTTCCTTGGGTTCTTGGGAGCA---GCAGGAAGCACTATG---GGCGCGGCGTCA---
ATAACGCTGACGGTACAG---GCCAGACAA---TTGTTG---TCTGGTATAGTGCAACAGCAAAGC---
AATTTG---CTGAGG---GCTATAGAAGCGCAACAGCATCTGTTGCAACTCACGGTCTGGGGCATT
>KP109488
TTCTATTGTAATACATCAGGCTTGTTTAATGGTACATACATGAATAATGATACAAAAAGTAATGATACAAAAA
GTAAT--

TCAAGCTCCATCATCACAATCCCATGCAGAATAAAGCAAATTATAAACATGTGGCAAGAGGTAGGACGAGCAA
TGTATGCCCCTCCCATTGCAGGAAACATAACATGTAAATCAAAT---
ATCACAGGAATACTATTGACACGAGATGGAGGACGAGGAGAAGAG----------------------------
----

GTAAAAAATGATACAGAGACATTCAGACCTGGAGGAGGAAATATGAAGGACAATTGGAGAAGTGAATTATATA
AATACAAAGTAGTAGAAATTAAGCCATTA---GGAGTAGCACCAACTGCAGCAAAAAGGAGAGTGGTG-----
----------------------------------GAGAGAGAAAAAAGAGCA---GTGGGA---
CTAGGAGCT---------GTACTCCTTGGGTTCTTGGGAGCA---GCAGGAAGCACTATG---
GGCGCGGCGTCA---ATAACGCTGACGGTACAG---GCCAGACAA---TTGTTG---
TCTGGAATAGTGCAACAGCAAAGC---AATTTG---CTGAGA---
GCTATAGAGGCGCAACAGCATATGTTGCAACTCACAGTCTGGGGCATT
>KP109489
TTCTATTGTAATACATCAAAACTGTTTAATAGTACATACATACCTACATACAGGCCTAATAATACACAAGGTA
AT---

TCAAGCTCAACCATCACAATCCCATGTAGAATAAAGCAAATTATAAACATGTGGCAGGAGGTAGGACGAGCAA

```
TGTATGCCCCTCCCATTGCAGGAAACATAACATGTAAATCACAT---
ATCACAGGACTACTATTGGTACGGGATGGAGGAACAGGGCTAAAT----------------------------
-------------------------------------------------------------------------
----------
AGTAGTACAGAGACATTTAGACCTGGAGGAGGGGATATGAGGGACAATTGGAGAAGTGAATTATATAAATATA
AAGTAGTAGAAATTAAGCCATTG---GGAGTAGCACCAACTGCAGCAAAAAGAAGAGTGGTG-----------
----------------------------CAGAGAGAAAAACGAGCA---GTGGGA---TTAGGAGCT------
---GTGTTCCTTGGGTTCTTGGGAACA---GCAGGAAGCACTATG---GGCGCAGCGTCA---
ATAACGCTGACGGTACAG---GCCAGACAA---TTGTTG---TCTGGTATAGTGCAACAGCAGAGC---
AATCTG---CTGAGG---GCTATAGAGGCGCAACAGCATATGTTGCAACTCACAGTCTGGGGCATT
>AJ276221
TTCTATTGTAATACAACAGGCCTGTTTAATGGTACATTTAATGGTACATACATGAATAATACAGGA-------
-------------------------------------------------------------------------
----------------------------
AGTAATTCAAGCATCACAATCCCATGCAGAATAAAGCAAATTATAAACATGTGGCAGAAGGTAGGACAAGCAA
TGTATGCCCCTCCCATTGCAGGAAACATAACCTGTAGGTCAAAT---
ATTACAGGACTACTATTGGTACGCGATGGAGGATTGGGGACAAAT----------------------------
-------------------------------------------------------------------------
-------------
GACACAGAGACATTTAGACCTGGAGGAGGAGATATGAGGGACGATTGGAGAAGCGAATTATATAAATATAAAG
TGGTAGAAATTAAGCCATTG---GGAGTAGCACCCACAGGAGCAAAAAGGAGAGTGGTG--------------
-------------------------GAGAGAGGAAAAAGAGCA---GTGGGA---CTAGGAGCT---------
GTGTTCCAAGGGTTCTTGGGAGCA---GCAGGAAGCACTATG---GGCGCGGCGTCA---
ATAACGCTGACGGTACAG---GCCAGACAA---TTGTTGTCTGGTATAGTGCAACAACAAAGCAGC---
AATTTG---CTGAGG---GCTATAGAGGCGCAACAGCATCTGTTGCAACTCACGGTCTGGGGCATT
>EU521728
TTCTATTGCAACACATCAACACTGTTTAATAGTACGTACATGTACATGAATAGTACGTACATGTTTAATGGTA
CAGAAAGTAAT--------------------------------------------------------------
-------GGTACAGAAAGT------
AATTCAAACTCAACCATCACAATCCAATGCAGAATAAAACAAATTATAAACATGTGGCAGAAGGTAGGAAGAG
CAATGTATGCCCCTCCCATTGCAGGAAACATAACATGTAAATCAGGT---
ATCACAGGACTATTATTGGTACGTGATGGAGGAAATCCAAATAATACA-------------------------
-------------------------------------------------------------------------
----------
AATAATACAGAGATCTTCAGACCTGGAGGAGGAGATATGAGGGACAATTGGAGAAGTGAATTATATAAATATA
AAGTGGTAGAAATTAAGCCATTG---GGAATAGCACCCACTGCAGCAAAAAGGAGAGTGGTG-----------
----------------------------GAGAGAGAGAAAAGAGCA---GTGGGA---CTAGGAGCT------
---ATGTTCTTTGGGTTCTTAGGAGCA---GCAGGAAGCACTATG---GGCGCGGCGTCA---
GTGACGCTGACGGTACAG---GCCAGACAA---TTGTTG---TCTGGTATAGTGCAACAGCAAAGC---
AATTTG---CTGAGG---GCTATAGAGGCGCAACAGCAGTTGTTGCAACTCACAGTCTGGGGCATT
>EF694033
TTCTATTGCAATACATCACAACTGTTTAATAGTACATATGATCCTGCATACAGGCCTAATAATACAGAA----
-------------------------------------------------------------------------
----------------------------
AACAATTCAACCATCATACTCCCATGCAGAATAAGACAAGTTGTAAACCTGTGGCAGGAGGTAGGACGAGCAA
TGTATGCCCCTCCCATTGAAGGAAACATAACGTGTAAATCAAAT---
ATCACAGGAATACTATTGGTACGTGATGGAGGGACAGAATTAAATAATACA----------------------
-------------------------------------------------------------------------
----
GAATTTAATAATACAGAAATATTCAGACCGGGAGGAGGAGATATGAGAGACAATTGGAGAAGTGAATTATATA
AATATAAAGTGGTAGAAATTAAGCCATTG---GGAGTAGCACCCACTACGGCAAAAAGGAGAGTGGTG-----
----------------------------------GAGAGAGAAAAAAGAGCA---
GTAGTGGGAATAGGAGCT---------GTGTTCCTTGGGATCTTGGGAGCA---GCAGGAAGCACTATG---
GGCGCGGCATCA---ATAACGCTGACGGTACAG---GCCAGACAA---CTGCTG---
TCTGGTGTAGTGCAACAGCAAAGC---AATTTG---CTGAGG---
GCTATAGAGGCGCAACAGCATATGTTGCAACTCACGGTCTGGGGCATT
>AF457054
TTCTATTGTAATACATCAGGCCTGTTTAATGAAACTCTAACTCGGTTGAATGATACATACAGTTCT-------
-------------------------------------------------------------------------
----------------------------
```

AATGGGACAAACATCACACTCCCATGTAGAATAAAACAAATTATAAACATGTGGCAGAAGGTAGGACGAGCAA
TGTATGCCCCTCCCATTGCAGGAAACATAACATGTACATCAAAA---
ATCACAGGACTATTATTGCAACGTGATGGAGGGAACAGTACAAAT----------------------------
----

AAAACTGAGACATTCAGGCCTGGAGGAGGAAATATGAAGGACAACTGGAGAAGTGAATTATACAAATATAAAG
TGGTGGAAATTAAGCCATTG---GGAATAGCACCCACTGAGGCAAAAAGGAGAGTGGTG--------------
-------------------------GAGAGAGAAAAAAGAGCA---GTGGGA---ATAGGAGCT---------
GTACTCCTTGGGTTCTTGGGAGCA---GCAGGAAGCACTATG---GGCGCGGCGTCA---
ATAACGCTGACGGTACAG---GCCAGACAA---CTGTTG---TCTGGTATAGTGCAACAGCAAAGC---
AATTTG---CTGAGG---GCTATAGAAGCGCAACAGCATATGTTGCAGCTCACGGTCTGGGGCATT
>FJ396028
TTCTATTGCAATACATCAGATTTGTTTAACTTTCCTAATGGTAGTAAGGCTATTAAT----------------
----

GGTAATTCAACCATTACACTCCAATGCAGAATAAAACAAATTATAAACATGTGGCAGGGGGTAGGACAAGCAA
TGTATGCTCCTCCCATTAGAGGGAACATAACATGTAGATCAAAT---
ATCACAGGACTATTACTGACACGCGATGGAGGAGAAGGACGAAATGGCACA----------------------
----

GAGAATGTCACAGAGATATTCAGACCTGGAGGAGGAGATATGAGGGACAATTGGAGAAGTGAATTATACAAAT
ATAAAGTGGTAGAAATTAAGCCATTA---GGAGTAGCACCCACTAAGGCAAAAAGGAGAGTGGTG--------
-------------------------------GAGAGAGAAAAAAGAGCA---GTGGGA---ATAGGAGCT---
------GTGTTCCTTGGGTTCTTGGGAGCA---GCAGGAAGCACTATG---GGCGCAGCGTCA---
ATAACGCTGACGGTACAG---GCCAGACAA---TTATTG---TCAGGCATAGTGCAACAGCAAAAC---
AATTTG---CTGAGA---GCTATAGAGGCGCAACAACATCTGTTGCAACTCACGGTCTGGGGCATT
>KT022366
TTCTATTGCAATACAACAATCCTGTTTAATGAGACATACAGGGAATACATGGCTAATAAAACACAA-------
----

GATAATACGACACTCACACTCCAATGCAGAATAAAACAGATTATAAGGATGTGGCAGGGGGTAGGACAAGCGA
TGTATGCCCCTCCCATTGCAGGCAACATAACATGTAGATCAAAT---
ATCACAGGACTACTATTGATACGTGATGGAGGAACAAACAATAGT----------------------------
----

GAGACATTCAGACCTGGAGGAGGAGATATGAGGGACAATTGGAGAAGTGAATTATATAAATATAAAGTGGTAG
AAATTAAACCATTA---GGAATAGCACCCACCAAGGCAAAAAGGAGAGTGGTG--------------------
-------------------GAGAGAGAAAAAAGAGCA---GCA------CTAGGAGCT---------
TTGTTCCTTGGGTTCTTGGGAGTA---GCAGGAAGCACTATG---GGCGCGGCGTCA---
ATGACGCTGACGGTACAA---GCCAGACAA---TTGCTG---TCTGGTATAGTGCTACAGCAAAGC---
AATTTG---CTGAGA---GCCATAGAGGCGCAACAGCATATGTTGCAACTCACGGTCTGGGGCATT
>KT022371
TTCTATTGCAATACATCAAAACTGTTTAAGGATATTGTTATAAATGATACTGAT-------------------
----

ACAAATACAACCATCATGCTCCCATGCAGAATAAAACAAATTATAAACATGTGGCAGCAGGTAGGACGAGCAA
TGTATGCCCCTCCCATCCGAGGGCAAATAACATGTAACTCAACT---
ATCACAGGATTAATACTGACACGTGATGGAGGAAATACTAATAACAATAACACC-------------------
----

AATGACACAGAGATAGAGATATTCAGACCTGCAGGAGGAGATATGAGGGACAATTGGAGAAGTGAATTATATA
AATATAAAGTGGTAGAAGTTAAGCCATTA---GGAATAGCACCCACTGAGGCAAAAAGGAGAGTGGTG-----
----
GAGAGAGAAAAAAGAGCAGTAGTAGGGGGACTAGGAGTT---------GTGTTCCTTGGGTTCTTGGGAGCA-
--GCCGGAAGCACTATG---GGCGCAGCGTCA---ATGGCGCTGACGGTACAG---GCCAGACAA---
TTACTG---TCGGGTATAGTGCAACAGCAAAAC---AATTTG---CTGAGG---
GCTATAGAGGCGCAACAGCATATGTTGCAACTCACGGTCTGGGGCATT
>KU921723
TTCTATTGCAATACAACAATCCTGTTTAATAGTAGTAGGATTTTTAAAAGAAAT-------------------
----

TTTACAGAAAATATCACACTCCCATGCAGAATAAAACAATTTATAAACATGTGGCAGAGAGTAGGACAAGCAA
TGTATGCTCCTCCCATTGCAGGAAACATAATATGTAACTCAAAC---
ATCACAGGACTAATATTGACACGTGATGGAGGTAATGATAATGATACA-------------------------
----

TCAAACAACAATGAGACATTCAGACCTGCAGGAGGAGATATGAGGGACAATTGGAGAAGTGAATTATATAAAT
ATAAAGTGGTAGAAATTAAACCATTA---GGAATAGCACCCACAAAGGCAAAAAGGAGAGTGGTG--------
-------------------------------GAGAGAGAAAAAAGAGCA---GTGGGA---ATAGGAGCT---
------ATGATCCTTGGGTTCTTGGGAACA---GCAGGAAGCACTATG---GGCGCGGCGTCA---
ATAACGCTGACGGTACAA---GCCAGACAA---TTGTTG---TCAGGCATAGTGCAACAGCAAAGT---
AATTTG---CTGAGA---GCTATAGAGGCGCAACAGCACATGTTGCAACTCACAGTCTGGGGCATT
>KU921743
TTCTATTGCAATACATCAAGCCTGTTTAATAGTATATACAATCCTAATATTACATACAATGCTAGTGATGAGT
CAAGCTCA---

AACATCACACGAAACATCACACTCCCATGCAGAATAAGACAAATTATAAACATGTGGCAGACGGTAGGACGAG
CAGTATATGCTCCTCCCATTAGAGGAAACATAACATGTATATCAAAC---
ATCACAGGACTATTATTGGTACGTGATGGAGGGGACAATAATAACACA-------------------------
----

AATAATACAGAGACATTCAGACCTGGAGGAGGAGATATGAGGGACAATTGGAGAAGTGAATTATATAAATATA
AGGTGGTAGAAATTAAGCCATTG---GGAGTAGCACCAACTGGAGCAAAAAGGAGAGTGGTG-----------
----------------------------GACAGAGGAAAAAGAGCA---GTGGGA---CTAGGAGCT------
---ATGTTCCTTGGGTTCTTGGGAGCA---GCAGGAAGCACTATG---GGCGCGGCGTCA---
ATAACGCTGACGGTACAG---GCCAGACAA---CTGTTG---TCTGGTATAGTGCAACAGCAAAAC---
AATTTG---CTGAGG---GCTATAGAAGCGCAACAGCATATGTTGCAACTCACGGTCTGGGGCATT
>KU921790
TTCTATTGCGAGACAGACAACCTGTTTAATAGTACATACATGGAGGCATACAATAGTACAGATAATAGT----
----

ACCAATTCAACCATCACACTCCCATGCAGAATAAAACAAATTATAAACATGTGGCAGGAAGTAGGACGAGCAA
TGTATGCCCCTCCCATTGCAGGAAACATAACATGTATATCAAAT---
ATCACAGGACTATTATTGACACGTGATGGAGGACAGGGAACTGAT----------------------------
----

AATGAAACAGAGATATTCAGACCAGCAGGAGGAGATATGAGGGACAATTGGAGAAGTGAATTATATAAATATA
AGGTGGTAGAAATTAAACCATTA---GGTATAGCACCCACTTCAGCAAAAAGGAACGTGGTG-----------
----------------------------AAGAGAGAAAAAAGAGCA---GTGGGA---CTAGGAGCT------
---GTGTTCCTTGGGTTCTTGGGAGCA---GCAGGAAGCACTATG---GGCGCAGCGTCA---
GTGACGCTGACGGTACAG---GCCAGGCAA---TTGCTG---TCTGGTATAGTGCAACAGCAAAAC---
AATTTG---CTGAGG---GCTATAGAGGCGCAACAGCATCTGTTGCAACTCACAGTCTGGGGCATT
>KU921807
TTCTATTGCAATACATCCAAACTGTTTAATAGTACATACATGGAGAAATACAATAGTACAGGCAATAGT----
----

ACCAATTCAACCATCACACTCCCATGTAGAATAAAACAAATTATAAACATGTGGCAGGGGGTAGGACGAGCAA
TGTATGCCCCTCCCATTGAAGGAAACATAACATGTAAATCAAAT---
ATCACAGGAATAATATTGACACGTGATGGAGGAGGGGTAGATAAT----------------------------
----

ACAACAGAGATATTCAGACCAGGAGGAGGAGATATGAGGGACAATTGGAGAAGTGAATTATATAAATATAAGG
TAGTAGAAATTAAACCATTA---GGAATAGCACCCACTAAGGCAAAAAGGAACGTGGTG--------------
-------------------------CAGAGAGAAAAAAGAGCAGTAATGGGA---ATAGGAGCT---------
GTGTTCCTTGGGTTCTTGGGAGCA---GCAGGAAGCACTATG---GGCGCAGCATCA---
ATAACGCTGACGGTACAG---GCCAGGCAA---TTGCTG---TCTGGTATAGTGCAACAGCAAAGC---
AATTTG---CTGAGG---GCTATAGAGGCGCAACAGCATCTGTTGCAACTCACGGTCTGGGGCATT
>FJ866132
TTCTATTGTAATACATCAGGCCTGTTTAATAGGGTAATTAATGATACATACATGACTAATGATACT-------
----

TCAAATTCAACCATCACACTCCGCTGCAGAATAAAACAAATTATAAACACGTGGCAGGGGGTAGGACGAGCAA
TGTATGCCCCACCCATTGAAGGAAACATAACATGTATATCAAAC---
ATCACAGGACTACTATTGACACGTGATGGAGGACAAGAAAATAGA----------------------------
----

ACAGAGGTATTTAGGCCTGGAGGAGGAGATATGAGGGACAATTGGAGAAGTGAATTATATAAATATAAGGTGG
TAGAAATTAAACCATTA---GGAATAGCACCCACTACTGCAAAAAGGAGAGTGGTG-----------------
----------------------GAGAGAGAAAAAAGAGCA---GTGGGA---CTAGGAGCT---------
GTGTTCCTTGGGTTCTTGGGAGCA---GCGGGAAGCACTATG---GGCGCAGCGTCA---
ATAACGCTGACGGTACAG---GCCAGACAA---TTATTG---TCTGGTATAGTGCAACAGCAAAGC---
AATTTG---CTGAGA---GCTATAGAGGCGCAACAGCATATGTTGCAACTCACGGTCTGGGGCATT
>JN983803
TTCTATTGCAATACATCAGGTCTGTTTAATGGTACATATGGATCTAATAATACAAAAGGTGAT----------
----

TCAAATGCAACTATCACACTCCCATGCAGAATAAAACAAATTATAAACATGTGGCAGGGGGTAGGACGAGCAA
TATATGCCCCTCCCATTGCAGGAAACATAACATGTACATCAAAT---
ATCACAGGATTAATATTGACACGTGATGGAGGGTCAGAGGATAAT----------------------------
----

ACCACAGAGATATTCAGACCTGCAGGAGGAGATATGAGGGACAATTGGAGAAGTGAAATATACAAATATAAAG
TGGTAGAAATTAAACCATTG---GGAATAGCACCCACTGGGGCAAAAAGGAGAGTGGTG--------------
-------------------------GAGAGAGAGAAAAGAGCA---GTGGGA---ATAGGAGCT---------
GTGTTCCTTGGGTTCTTGGGAGCA---GCAGGAAGCACTATG---GGCGCGGCGTCA---
ATAACGCTGACGGTACAG---GCCAGACAA---CTGTTG---TCTGGTATAGTGCAACAGCAAAGC---
AATTTG---CTGAGA---GCTATAGAGGCGCAACAACATTTGTTGCAACTCACAGTCTGGGGCATT
>JN983804
TTCTATTGTGATACATCAAAACTGTTTAATAGAACATACATGGCTAATAGTACAGAA----------------
----

AATAATTCATCCATCACGCTGCCCTGCAAAATAAAACAAATTATAAACCTGTGGCAGGGAGTAGGACGAGCAA
TGTATGCCCCTCCCATTAAAGGAAAAATAACATGTAACTCAACG---
ATCACAGGACTACTATTGACACGTGATGGAGGGAATGAGAGTGAA----------------------------
----

GAGACGTTCAGACCTGGAGGAGGAGATATGAGAGACAATTGGAGAAGTGAATTATATAAATATAAAGTGGTAG
AAATTAAGCCATTA---GGAATAGCACCCACTGAGGCAAAAAGGAGAGTGGTG--------------------
-------------------GAGAGAGAGAAAAGAGCA---GTGGGA---ATAGGAGCT---------
GTGTTCCTTGGGTTCTTGGGAGCA---GCAGGAAGCACTATG---GGCGCGGCGTCA---
ATAACGCTGACGGTACAG---GCCAGACAA---TTGCTG---TCTGGTATAGTGCAACAGCAAAGC---
AATTTG---CTGAGG---GCTATAGAGGCGCAACAGCATATGTTGCAACTCACAGTCTGGGGCATT
>JN983805
TTCTATTGCAATACATCAGAATTGTTTACAGAAAAAAATTTCA------------------------------
----

AATGAGACAAACATCACGCTTTCATGCAGAATAAAACAGATTATAAATATGTGGCAGGGGGTAGGACGGGCAA
TGTATGCCCCTCCCATTGAAGGAAACATAACATGTACATCAAAT---
ATCACAGGACTACTCATGACATATGATGGAGGACAACACACAGAA----------------------------
----

AATAAAACAGAGACCTTCAGACCTGGAGGAGGAGATATGAGGGACAATTGGAGAAGTGAATTATATAAATATA
AAGTGGTAGAAATTAGGCCATTA---GGAATAGCACCCACTAAGGCAAAAAGGAGAGTGGTG-----------
----------------------------GAGAGAGAAAAAAGAGCA---GTGGGA---ATGGGAGCT------
---GTGCTCCTTGGGTTCTTGGGAGCA---GCAGGAAGCACTATG---GGCGCAGCATCA---
ATAACGCTGACGGTACAG---GCCAGACAA---CTGTTG---TCTGGTATAGTGCAACAGCAAAGC---
AATTTG---CTGAGA---GCTATAGATGCGCAACAGCATATGTTGCAACTCACGGTCTGGGGCATT
>HM215360
TTCTATTGCAATACATCAAGATTGTTTACATACACAGAAGAG-------------------------------
----

ACAGGAAATATTACCCTCCCATGCAGAATAAAACAAATTATAAACATGTGGCAGGAGGTAGGACGAGCAATGT
ACGCCCCTCCCATTGCAGGAAACATAATATGTAGATCAAAT---
ATCACAGGATTACTATTGACACGTGATGGAGGAAAAAATAATAGT----------------------------
----

GGACCTGAGACATTCAGACCTGGAGGAGGAGATATGAGGGACAATTGGAGAAGTGAATTATATAAATATAAAG
TAGTAGAAATTAAACCATTA---GGAATAGCACCCACTAAGGCAAAAAGGAGAGTGGTG--------------
-------------------------GAGAGAGAGAAAAGAGCA---GTGGGA---CTAGGAGCT---------
GTGTTCCTTGGGTTCTTGGGAGCA---GCAGGAAGCACTATG---GGCGCGGCGTCA---
ATCACGCTGACGGTACAG---GCCAGACAA---TTATTG---TCTGGTATAGTGCAACAGCAAAGC---
AATTTG---CTGAGA---GCCATAGAGGCGCAACAGCATATGTTGCAACTCACGGTCTGGGGCATT
>KC894075
TTCTATTGCAATACATCAAAACTGTTCAATGGTACATACAATAGTACATACAAGACTAATACTACAGAA----
----

AGTAATGACACCATCACCATCCCATGCAGAATAAAACAAATTATAAACATGTGGCAGGGGGTAGGACGAGCAA
TGTATGCCCCTCCCATTGCAGGAAACATAACATGTAGATCAAAT---
ATCACAGGACTACTATTGACACGTGATGGAGGAAAAGTAAATAGC----------------------------
----

ACAAATGAGACATTCAGACCTGAAGGAGGAAATATGAAAGACAATTGGAGAAGTGAATTGTATAAATATAAAG
TGGTAGAAATTAAGCCATTA---GGAGTAGCACCCACGGAGGCAAAAAGGACAGCGGTG--------------
-------------------------GGGAGAGGAAAAAGAGCA---GTGGGA---ATAGGAGCT---------
GTGCTCCTTGGGTTCTTGGGAGCA---GCAGGAAGCACTATG---GGCGCGGCGTCA---
ATAACGCTGACGGCACAG---GCCAGACAA---GTATTG---TCTGGTATAGTGCAACAGCAAAGC---
AATTTG---CTGAGA---GCCATAGAGGCGCAACAGCATATGTTGCAACTCACGGTCTGGGGCATT
>KC894077
TTCTATTGCGATACATCAAAATTATTTAATAGTACACACAGG-------------------------------
----

GCTGATGATTCACCCATCACACTCCAATGCAGAATAAAACAAATTATAAACATGTGGCAGGGGGTAGGAAGAG
CAATGTATGCCCCTCCCATTGCAGGAAACATAGTATGTAACTCAAGT---
ATCACAGGACTACTATTAACACGTGATGGAGGAGGGAATGGTACA----------------------------
----

GAGACATTCAGACCTGGAGGAGGAGATATGAGGGACAATTGGAGAAGTGAGTTATATAAATATAAAGTGGTAG
AAATTAAGCCATTA---GGAATAGCACCCACTAAGGCAAAAAGGAGAGTGGTG--------------------
-------------------GAGAGAGAAAAAAGAGCA---GCT------CTGGGAGCT---------
ATGTTCCTTGGGTTCTTGGGAATG---GCAGGAAGCACTATG---GGCGCAGCATCA---
ATAACGCTGACGGTACAG---GCCAGGCAA---TTGTTG---TCTGGTATAGTGCAACAGCAGAAC---
AATTTG---CTGAGA---GCTATAGAGGCGCAACAGCATATGTTGCAACTCACAGTCTGGGGCATT
>FJ444184
TTCTATTGCAATACAACAAAACTGTTTAACAGTACACACCAGGTTAATAGTACCCGGGATAATAGTACAGAA-
----

AGTAATTCAACCATCACACTCCAATGCAGAATAAAACAAATTATAAACATGTGGCAGGGGGTAGGACGAGCAA
TGTATGCCCCTCCCATTGCAGGAGATATAAGATGTGAATCAAAT---
ATCACAGGACTACTATTGACACGTGATGGAGGAATAGGTAACAATGAC-------------------------
----

ACAGAGAAAACAGAGATATTCAGACCTGGAGGAGGAAATATGAGGGACAATTGGAGAAGTGAATTATATAAAT
ATAAAGTGATAGAAATTAAACCATTA---GGAATAGCACCCACTAAGGCAAAAAGGAGAGTGGTG--------
-------------------------------GAGAGAGAAAAAAGAGCA---GTGGGA---ATAGGAGCT---
------GTGTTCCTTGGGTTCTTGGGAGCA---GCAGGAAGCACTATG---GGCGCAGCATCA---
ATAACGCTGACGGTACAG---GCCAGACAA---TTGTTG---TCTGGTATAGTGCAACAGCAAAAC---
AATTTG---CTGAGG---GCTATAGAAGCGCAACAGCATATGTTGCAGCTCACGGTCTGGGGCATT
>FJ444251
TTCTATTGCAATACATCAGGTCTGTTTAATGGTACATACCCGTCTAATGATAATAGTACATACATG-------
----

TCTAATGATACAATAACCCTCCCATGCAGAATAAAACAAATTATAAACATGTGGCAGGGGGTAGGACGAGCAA
TGTATGCTCCTCCCATTAAAGGAAATATAACATGTGAATCAAAC---
ATCACAGGATTACTATTGACGCGTGATGGAGGAGAAACTAATACCACT-------------------------
----

AATAACACAGAGATATTCAGACCTGGAGGAGGAGATATGAGGGACAATTGGAGAAATGAACTATATAAGTATA
AAGTGGTAGAAATTAAGCCATTG---GGAATAGCACCCACTAAGGCAAAAAGGAGAGTGGTG-----------
----------------------------GAGAGAGAAAAAAGAGCA---GTGGGA---ATAGGAGCT------
---GTGTTCCTTGGGTTCTTGGGAGCA---GCAGGAAGCACTATG---GGCGCGGCATCA---
ATAACGCTGACGGTACAG---GCCAGACAA---TTGTTG---TCTGGTATAGTGCAACAGCAAAGC---
AATTTG---CTGAGG---GCTATAGAGGCGCAACAGCATATGTTGCAACTCACGGTCTGGGGCATT
>FJ444277
TTCTATTGCAATACATCAGGACTGTTTAATGAGACATACACATACAAATTTAATGAAAGTAATTCGACAAATG
GA---

ACTAACTCAACAATCAAACTCCTATGCAGAATAAAGCAAATTATAAACATGTGGCAGGGGGTAGGACGAGCAA
TGTATGCCCCTCCCATTGAAGGAAACATAACATGCAAATCAAGT---
ATCACAGGATTACTATTGACACGTGATGGAGGAAATACTAATGAG----------------------------
----

ACAGAGATATTCAGACCTGCAGGAGGAGATATGAGGGACAATTGGAGAAGTGAATTATACAAATATAAAGTGG
TAGAAATTAAGCCATTA---GGAATAGCACCCACTGAAGCAAAAAGGAGAGTGGTG-----------------
----------------------GAGAGGGAGAAAAGAGCA---GTGGGA---ATAGGAGCT---------
GTGTTCCTTGGGTTCTTGGGAGCA---GCAGGAAGCACTATG---GGCGCGGCGTCA---
ATAACGCTGACGGTACAG---GCCAGACAA---TTGTTG---TCTGGTATAGTGCAACAGCAAAGC---
AATTTG---CTGAGG---GCTATAGAGGCGCAACAGCATATGTTGCAACTCACGGTCTGGGGCATT
>FJ444476
TTCTATTGCGATACATCAAAACTGTTTAATGGTAATGGTACCAACAGTAATGGTACTGGTACCAACAGTACT-
----

ACAAACTCAACAATCATGATCCCATGCAGAATAAAACAATTTATAAACATGTGGCAGGAGGTAGGACAAGCAA
TGTATGCCCCTCCCATTGAAGGAAAGATAACCTGTAAATCAAAT---
ATCACAGGACTGCTATTGACACGTGATGGAGGAAATAGTACTGGAAGTAAT----------------------
----

AAAAGTGACACAGAAACATTCAGACCTATAGGAGGAGATATGAGGGACAATTGGAGAAGTGAATTATATAAAT
ATAAAGTGGTAGAAATTAAGCCATTA---GGAATAGCACCCACTAAGGCAAAAAGGAGAGTGGTG--------
-------------------------------GAGAGAGAGAAAAGAGCA---GTGGGA---ATAGGAGCT---
------ATGTTCCTTGGGTTCTTGGGGGCA---GCAGGAAGCACTATG---GGCGCAGCGTCA---
ATAACGCTGACGGTACAT---GCCAAACAA---TTATTG---TCTGGTATAGTGCAACAGCAAAGC---
AATTTG---CTGAGG---GCTATAGAGGCGCAACAGCATATGTTGCAACTCACGGTCTGGGGCATT
>KC894083
TTCTATTGCAATACATCAAAACTGTTTAATAGTACATATCCTAATAATAGTACAGACAAC-------------
----

ACTTCAGATACCATCACACTCCAATGCAGAATAAAACAAATTATAAACATGTGGCAGGAGGTAGGACGAGCAA
TGTATGCCCCTCCCATTGCAGGAAACATAACATGTAGATCAAAT---
ATCACAGGACTGCTATTGACACGTGATGGAGGAGGAAAAACTAAC----------------------------
----

AGCACAAATGAGACATTCAGACCTTTGGGAGGAGATATGAGGGACAATTGGAGAAGTGAATTATATAAATACA
AAGTGGTAGAAATTAAGCCATTA---GGATTAGCACCTACTAAGGCAAAAAGGAGAGTAGTG-----------
----------------------------GACAGAGAAAAAAGAGCA---GTAGGA---ATGGGAGCT------
---TTGTTCCTTGGGTTCTTGGGAGTA---GCCGGAAGCACTATG---GGCGCAGCATCA---
ATGACGCTGACGGTACAA---ACCAGACAA---TTGCTG---TCTGGTATAGTGCAACAGCAAAGC---
AATTTG---CTGAGG---GCCATAGAGGCGCAACAACATATGTTGCAACTCACGGTCTGGGGCATT
>KC894085
TTTTATTGCAATACAACAAAACTGTTTAATAGTACAGATAATAGCACAGGTACAAATAATAGCACAGAA----
----

ATCATACTCCCATGCAGAATAAAACAGATTATAAACATGTGGCAAGAGGTAGGGCGAGCAATGTATGCCCCTC
CCATTGCAGGAAACATAACATGTAAATCAAAT---
ATCACAGGGCTAATATTGACACGCGATGGAGGAGATAACAATAAC----------------------------
----

AATGACATAGAGACATTCAGACCTGGAGGAGGAGATATGAGGGATAATTGGAGAAGTGAATTATATAAATATA
AAGTGGTAGAAATTAAGCCATTG---GGAATAGCACCCACTGAGGCAAAAAGGAGAGTGGTG-----------
----------------------------CAGAGAGAAAAAAGAGCA---GTGGGA---ATAGGAGCT------
---ATGTTCCTTGGGTTCTTGGGATTC---GCAGGAAGCACTATG---GGCGCGGCGTCA---
ATAACGCTGACGGTACAG---GCCAGACTA---CTGTTG---TCTGGTATAGTGCAACAGCAAAAC---
AATTTG---CTGAGA---GCTATAGAGGCGCAACAGCATATGTTGCAACTCACAGTCTGGGGCATT
>KC894087
TTCTACTGCAATACATCAAAACTGTTTAATGGTATATATAATAGTACATATAATAGTACAGGCAACATTACA-
----

GAGGGTAAGAACATCACACTCCAATGCAGAATAAAACAAATTATAAATATGTGGCAGGGGGTGGGACAGGCAA
TGTATGCCCCGCCCATTGCAGGAAACATAACATGTAAGTCAAAT---
ATCACAGGAATACTATTGACACGTGATGGAGGAAATAATAGTACAAGA-------------------------
----

ACAGAAGAGATATTCAGACCTGGAGGAGGAAACATGAAGGACAATTGGAGAAGTGAATTATATAAATATAAAG
TAGTAGAAATTAAGCCATTA---GGAGTAGCACCCACTGAGGCAAAAAGGAGAGTGGTG--------------
-------------------------GAGAGAGAAAAAAGAGCA---GTGGGA---CTGGGAGCG---------
TTGTTCCTTGGGTTCTTGGGAGCA---GCAGGAAGCACTATG---GGCGCAGCATCA---
ATAACGCTGACGGTACAG---GCCAGACAA---TTGTTG---TCTGGTATAGTGCAACAGCAAAGC---
AATTTG---CTGAGA---GCTATAGAGGCGCAACAGCATATGTTGCAACTCACAGTCTGGGGCATT
>KC894088
TTCTATTGCAATACATCAAGCCTGTTTAATGATGCATTTAATGATACAAACAGTACAGAA-------------
----

TCAAATTCAACCATCACACTCCCATGCAGAATAAAACAAATTGTAAACATGTGGCAGAGGGTAGGACGAGCAA
TGTATGCCTCTCCCATTAAAGGAAACATTACATGTAAATCAAAT---
ATCACAGGACTAATATTGACACGTGATGGAGGAAACGAAAATAAT----------------------------
----

GGAACAGACATATTCAGACCTATAGGAGGAAATATGAAGGACAATTGGAGAAGTGAATTATATAAATATAAAG
TAGTAGAAATTAAGCCATTA---GGAGTAGCACCCACTACTGCAAAAAGGAGAGTGGTG--------------
-------------------------GAGAGAGAAAAAAGAGCA---GTGACA---CTAGGAGCT---------
GTGTTCGTTGGGTTCTTGGGAGCA---GCAGGAAGCACCATG---GGCGCAGCATCC---
GTAATGCTGACGGTACAG---GCCAGACAA---TTATTG---TCTGGTATAGTGCAACAGCAAAGC---
AATTTG---CTGAGG---GCTATAGAGGCGCAACAGCATATGTTGCAACTCACGGTCTGGGGCATT
>KC894090
TTCTATTGTAACACATCAAGACTGTTTAATAATACATATAAGTTTAATGATACAAACATAGTTAATAGTACAA
ATAGTACA---

ACAAATTCAACCATCACACTCCAATGCAGAATAAAACAAATTATAAATATGTGGCAGGGGGTAGGACGGGCAA
TGTATGCCCCTCCTATTGCAGGAAACATAACATGTAAATCAAAT---
ATCACAGGACTACTATTGACACGTGATGGAGGTGGAAATAATAAAACA-------------------------
----

GAGACAGAGACATTCAGACCTGGAGGAGGAGATATGAGAGACAATTGGAGAAGTGAATTATATAAATATAAAG
TGATAGAAATTAAACCATTA---GGAATAGCACCCACTAGGGCAAAAAGGAGAGTGGTG--------------
-------------------------GAGAGAGAAAAAAGAGCA---GTGGGA---CTAGGAGCT---------
ATGATCCTTGGGTTCTTGGGAGCA---GCTGGAAGCACTATG---GGCGCAGCATCA---
ATAACGCTGACGGTACAG---GCCAGACAA---TTGTTG---TTTGGTATAGTGCAACAGCAAAAC---
AATTTG---CTGAGG---GCTATAGAGGCGCAACAGCATATGTTGCAACTCACAGTCTGGGGCATT
>KC894091
TTCTATTGCAATACAACACAACTGTTTAATGAAACAGATATA-------------------------------
----

GAAAGTAATTCAACTATAACAATCCCATGCAGATTAAAACAAATCATAAACATGTGGCAGGAGGTAGGACGAG
CAATGTATGCCCCTCCCATTGCAGGAAACATAACATGTAAATCAAAT---
ATCACAGGATTACTATTGACACGTGATGGAGGAAGAAATACAACA----------------------------
----

AATGACACAGAGATATTCAGACCTGCAGGAGGAGATATGAGGGACAATTGGAGAAGTGAATTATATAAATATA
AAGTGGTAGAAATTAAACCATTG---GGAATAGCACCCACTAAGGCAAAAAGGAGAGTGGTG-----------
----------------------------GAGAGAGAAAAAAGAGCA---GCA------ATAGGAGCT------
---ATGTTCCTTGGGTTCTTGGGAGCA---GCAGGAAGCACTATG---GGCGCAGCATCA---
ATAACGCTGACGGTACAG---GCCAGACAA---TTGCTG---TCTGGTATAGTGCACCAGCAAAGC---
AATTTG---CTGAGG---GCTATAGAGGCACAACAACATATGTTGCAACTCACAGTCTGGGGCATT
>KC894093
TTCTATTGCAATACATCAAATCTGTTTAATAGTACACCAGAACTGTTTAATAGTGAGGGTACAGAAAGTAATT
CAACC--

AGCAACAAAAACATCACCCTCCCATGCAGAATAAAACAGATTATAAACATGTGGCAGGAGGTAGGACGAGCAA
TGTATGCCCCTCCCATTGAAGGAAACATAACATGTATATCAAAT---
ATCACAGGACTACTATTGACACGTGATGGAGGAATTGACCCCAAT----------------------------
----

AGCACAGATGAGACATTCAGGCCTGGAGGAGGAGAAATGAGAGACAATTGGAGGAGTGAATTATATAAATATA
AGGTGGTAGAAATTAAGCCATTG---GGAATAGCACCCACTGAGGCAAAAAGGAGAGTGGTG-----------
----------------------------GAGAGAGAAAAAAGAGCA---GTGGGA---ATAGGAGCT------
---GTGTTCCTTGGGTTCTTGGGAGCA---GCAGGAAGCACTATG---GGCGCGGCGTCA---
ATAACGCTGACGGTACAG---GCCAGACAA---CTGTTG---TCTGGTATAGTGCAACAGCAAAGC---
AATTTG---CTGAAG---GCTATAGAGGCGCAACAGCATCTGTTGCAACTCACGGTCTGGGGCATA
>KC894094
TTCTATTGCGATACATCAGGCCTGTTTAATAGCACATACAATAGCACATACAATGATACAGATAACAGTACA-
----

GAAAACGACACGATCACGCTCCAATGTAGAATAAAACAAATTATAAGAATGTGGCAGGGGGTAGGACAAGCAA
TGTATGCCCCTCCCATTGCAGGAAACATAACATGTATATCAAAG---
ATCACAGGACTACTATTGACACGTGATGGAGGTATAAATAATGCT----------------------------
----

ACTAATGAGACATTCAGGCCTGCAGGAGGAGATATGAGGGACAATTGGAGAAGTGAATTATATAAATACAAAG
TGATAGAAATTAAGCCATTA---GGAATAGCACCCACTAAGGCAAAAAGGAGAGTGGTG--------------
-------------------------GAGAGAGAAAAAAGAGCA---GCA------CTAGGAGCT---------
ATGTTCCTTGGGTTCTTGGGAGCA---GCAGGAAGCACTATG---GGCGCAGCATCA---
ATAACGCTGACGGTACAG---GCCAGACAA---TTGTTG---GCTGGTATAGTGCAACAGCAAAAC---
AATTTG---CTGAGG---GCTATAGAGGCGCAACAGCATCTGTTGCAACTCACGGTCTGGGGCATT
>KC894095
TTCTACTGCAATACAACAAACTTGTTTAATAAAACATATGATAGTACG-------------------------
----

TCTAACAAGACAATCATACTCACATGCAAAATAAAACAAATTATAAACATGTGGCAGGAGGTAGGACGAGCAA
TGTATGCCCCTCCTATTGCAGGAAACATAACATGTGAATCAGAT---
ATCACAGGAATACTATTGGTACGTGATGGAGGAAGTGAAAATGGCACA-------------------------
----

GAAAGTAACACTGAGATATTCAGACCTGAAGGAGGAAATATGAAGGACAATTGGAGAAGTGAATTATACAAAT
ATAAAGTGGTAGAAATTAAGCCATTG---GGAGTAGCACCCACTGAGGCAAAAAGGAGAGTGGTG--------
-------------------------------GAGAGAGAAAAAAGAGCA---GTGGGA---ATAGGAGCT---
------GTGTTCCTTGGGTTCTTGGGAGCA---GCAGGAAGCACTATG---GGCGCGGCGTCA---
ATAACGCTGACGGTACAG---GCCAGACAA---GTGCTG---TCTGGTATAGTGCAACAGCAAAGC---
AATTTG---CTGAGG---GCTATAGAGGCGCAACAGCACTTGTTGCAACTCACGGTCTGGGGCATT
>KC862818
TTCTATTGCAATACATCAAACCTGTTTAATAAGACATATAACTTTAATGAGACT-------------------
----

AAGAATGGAGCAATCACACTCCCATGCAAAATAAAACAAATTATAAGAATGTGGCAAAAAGTAGGACAAGCAA
TGTATGCTCCTCCCATTGCAGGAAACATAACATGTAAGTCTAAT---
ATCACAGGGTTACTATTGACGCGTGATGGAGGGAACACAACTGGGAATGAGACA------------TTC----
----

GAGACATTCAGACCTGGAGGAGGAGATATGAGGGACAATTGGAGAAATGAATTATATAAATATAAAGTGGTAG
AAATTAAGCCATTA---GGAGTAGCACCCACCAAGGCAAAAAGGAGAGTGGTG--------------------
-------------------GAGAGAGAAAAAAGAGCA---GTGGGA---ATAGGAGCT---------
GTGTTCCTTGGGTTCTTGGGAGCA---GCAGGAAGCACTATG---GGCGCAGCATCA---
ATAACGCTGACGGTACAG---GCCAGACAA---TTGCTG---TCTGGTATAGTGCAACAGCAGAGC---
AATTTG---CTGAGA---GCTATAGAGGCGCAACAGCATATGTTGCAACTCACAGTCTGGGGCATT
>KC894098
TTCTATTGCAATACATCAAAACTGTTTAATAGTACATACAATGGTACACACATGATTAATAGTACT-------
----

TCAAACTCAACCATCACACTCCCATGCAGAATAAAACAAATTATAAACATGTGGCAGGAGGTAGGACGAGCAA
TGTATGCCCCTCCCATTAAGGGAAACATAACATGTACATCAAAT---
ATCACAGGACTACTGTTGACACGGGATGGGGGAGTTAATTTGACAGGGGGTGAG-------------------
----

GGAGTTAATGACACAGAAACATTCAGACCTGAAGGAGGAAATATGAAGGACAATTGGAGAAGTGAATTATATA
AATATAAAGTGGTAGAAATTAAGCCATTA---GGAGTAGCACCCACTAAGGCAAAAAGGAGAGTGGTG-----
----------------------------------GAGAGAGAAAAAAGAGCA---GCA------
ATAGGAGCT---------ATGTTCCTTGGGTTCTTGGGAGCA---GCAGGAAGCACTATG---
GGCGCAGCATCA---ATAACGCTGACGGTACAA---GCCAGACAA---TTGCTG---
TCTGGTATAGTGCAACAGCAAAGC---AATTTG---CTGAGA---
GCTATAGAGGCGCAACAGCATCTGTTGCAACTCACAGTCTGGGGCATT
>KC862876
TTCTATTGCAATACATCAAACCTGTTTAATTTTACATACCTGCCTAATGGAACTAGTATAAACAGCGCTAACA
TT---

TTAAATGGGACCATCACACTCCCATGCAGAATAAAACAAATTATAAACATGTGGCAGGAGGTAGGAAAAGCAA
TGTATGCCCCTCCAGTTGCAGGAAAAATAACATGTAAATCAAAT---
ATCACAGGAATACTATTGACACGTGATGGAGGCAAAGATAACAAT----------------------------
----

GAGACAGAGACATTCAGACCTGGAGGAGGAGATATGAGGGACAATTGGAGAAGTGAATTATATAAATATAAAG
TGGTAGAAATTAAGCCATTA---GGAATAGCACCCACTGATGCAAAAAGGAGAGTGGTG--------------
-------------------------GAGAGAGAAAAAAGAGCA---GTGGGA---ATAGGAGCT---------
GTGTTCCTTGGGTTCTTGGGAGCA---GCAGGAAGCACTATG---GGCGCGGCGTCT---
ATAACGCTGACGGTACAG---GCCAGACAA---TTATTG---TCTGGTATAGTGCAACAGCAAAGC---
AATTTG---CTGAGG---GCTATAGAGGCGCAGCAGCATATGTTGCAACTCACGGTCTGGGGCATT
>KC894100
TTCTATTGCAATACATCAGGCCTGTTTAAAGGCCTGTTTAATGGTAGCAGAGAATGGGTTAATGGTACAGACA
ATAGT--
----------ACAAATAGT------
ACTCTAGATTCAGTCATCACACTCCCATGCAGAATAAAACAAATTATAAGAATGTGGCAGGGGGTAGGACAAG
CAATGTATGCCCCTCCCATTGCAGGAAACATAACATGTAAATCAAAT---
ATCACAGGAATACTATTGACACGTGATGGAGGAATACGTGAGGGA---GGAAAT------
ACAAGTGAGGGAGGAAAT---

GACACAGAGGTATTCAGACCTGGAGGAGGAGACATGAGGGACAATTGGAGAAGTGAATTATATAAATATAAAG
TGGTAGAAATTAAGCCATTA---GGAGTAGCACCCACTCCTGCAAAAAGGAGAGTGGTG--------------
-------------------------GAGAAACAGAAAAGAGCA---GTGGGA---ATGGGAGCT---------
GTGATCTTTGGGTTCTTGGGAGCA---GCAGGAAGCACTATG---GGCGCGGCGTCA---
ATAGCGCTGACGGCACAG---GCCAGACAA---TTATTG---TCTGGTATAGTGCAACAGCAAAGC---
AATTTG---CTGAGG---GCTATAGAGGCGCAACAGCATATGTTGCAACTCACAGTCTGGGGCATT
>KC862949

```
TTCTATTGCAATACATCAAAACTGTTTAATATTACAAACTTGAATAAAACAGAC-------------------
-------------------------------------------------------------------------
----------------------------
AGTAATTCAACAATCACACTCCCATGCAGAATAAAACAAATCATAAACATGTGGCAGGGGGTAGGACGAGCAA
TATATGCTCCTCCCATTGCAGGAAACATAACATGTAACTCTAGC---
ATCACAGGATTACTATTGACACGTGATGGAGGATCCAATGGTAAC----------------------------
-------------------------------------------------------------------------
-------------
GACACTGAGGTATTCAGACCTGGAGGAGGAGATATGAGGGACAATTGGAGAAGTGAATTATATAAATATAAAG
TGGTAGAAATTAAGCCATTG---GGAATAGCACCCACGGCGTCAAAAAGGAGAGTGGTG--------------
-------------------------GAGAGAGAAAAAAGAGCA---GTGGGA---ATAGGAGCT---------
GTGTTCCTTGGGTTCTTGGGAGCA---GCAGGAAGCACTATG---GGCGCAGCGTCA---
ATAACGCTGACGGTACAG---GCCAGACAA---TTGCTG---TCTGGTATAGTGCAGCAGCAAAAC---
AACTTG---CTGAGG---GCTATAGAGGCGCAACAGCATCTGTTGCAACTCACGGTCTGGGGCATT
>KC863009
TTCTATTGCAATACATCACGACTGTTCAATGGTACATACAGTACTAATAATACAAATAGTACT----------
-------------------------------------------------------------------------
----------------------------
TCAAATTCAACCATCACAATTCCATGCAGAATAAAACAAATTATAAACATGTGGCAGGGGGTAGGACAAGCAA
TGTATGCCCCTCCCATTGAAGGAAACATAACATGCAGGTCAAAT---
ATCACAGGACTATTACTGACACGTGATGGAGGAAAAGGAAATGACACA-------------------------
-------------------------------------------------------------------------
----------
AATGACACACAGACATTCAGACCTGGAGGAGGAGATATGAGGGACAATTGGAGAAGTGAATTATATAAATATA
AAGTAGTAGAAATTAAGCCATTA---GGAATAGCACCCACTAAGGCAAAAAGGAGAGTGGTG-----------
----------------------------GAGAGAGAAAAAAGAGCA---GCG------CTAGGAGCT------
---GTGTTCCTTGGGTTCTTGGGAGCG---GCAGGAAGCACTATG---GGCGCAGCGTCA---
ATAACGCTGACGGTACAA---GCCAGACAA---CTGCTG---TCTGGTATAGTGCAACAGCAAAGC---
AATTTG---CTGAGG---GCTATAGAGGCGCAACAGCATATGTTGCAACTCACGGTCTGGGGCATT
>KC894385
TTTTATTGCAATACATCAAACCTGTTTAATAGGATATATAATATTACAGACAATAGTACAGGAAGTGAT----
-------------------------------------------------------------------------
----------------------------
GACAATTCAACCATCACACTCCAATGCAGAATAAAACAAATTATAAACATGTGGCAGAAGGTAGGAAAAGCAA
TATATGCCCCTCCCATTGCAGGGAACATAACATGTGAATCAAAT---
ATCACAGGACTACTATTGACACGTGATGGAGGAAACAATGATAGT----------------------------
-------------------------------------------------------------------------
----------
ATCACCACAGAGACATTCAGACCTGGAGGGGGAGATATGAAGGACAATTGGAGAAGTGAATTATATAAATATA
AAGTGGTAGAAATTAAGCCATTA---GGAATAGCACCCACCAGGGCAAAAAGGAGAGTGGTG-----------
----------------------------GAGAGAGAAAAAAGAGCA---GTGGGA---ATAGGAGCT------
---GTGTTCATTGGGTTCTTGAGTGCA---GCAGGAAGCACTATG---GGCGCAGCGTCA---
ATAACGCTGACGGTACAG---GCCAGACAA---CTGTTG---TCTGGTATAGTGCAACAGCAAAGC---
AATTTG---CTGAGG---GCTATAGAGGCGCAACAGCATATGTTGAAACTCACGGTCTGGGGCATT
>KC156212
TTCTATTGCAATACATCAAGATTGTTTAGTAGTAATAGTACAGAAATTAATAGTACAGAAAGT----------
-------------------------------------------------------------------------
----------------------------
TCCAATTCAACAATCATACTCCCATGCAGAATAAAACAAATTATAAATATGTGGCAGGGGGTGGGACGGGCAA
TATATGCAGCTCCTATTGCAGGAAAAATAACATGTAAATCAAAT---
ATCACAGGACTAATATTGACACGTGATGGAGGAGAGGGCAATAATACA-------------------------
-------------------------------------------------------------------------
-------
GAAAACAGAAGTGAGATATTCAGACCTACAGGAGGAGATATGAGGGACAATTGGAGAAGTGAATTATACAAAT
ACAAAGTAGTAGAAATTAAGCCATTA---GGAGTAGCACCCACTAATGCAAAAAGGAGAGTGGTG--------
-------------------------------GAGAGAGAAAAAAGAGCA---GTGGGA---TTAGGAGCT---
------ATGTTCCTTGGGTTCTTGGGAATG---GCCGGAAGCACTATG---GGCGCAGCATCA---
ATAACGCTGACGGTACAG---GCCAGACAA---GTATTG---TCTGGTATAGTGCAACAGCAAAGC---
AATTTG---CTGAGG---GCTATAGAGGCGCAACAGCATATGTTGCAACTCACGGTCTGGGGCATT
>KC156211
```

TTCTATTGCAATACATCAGGTCTATTTAATGGGACATACAATAGTACAAGAACTAATTCAACTAAT-------
----

TCAACTTCAATCATCACACTCCAATGCAGAATAAAACAAATTGTAAACCTATGGCAGGGGGTAGGAAAAGCAA
TGTATGCCCCTCCCATTGCAGGAAACATAACATGCAGATCAGAT---
ATCACAGGACTACTATTGACACGTGATGGAGGAAACGGAAATAATAGT-------------------------
----

ACAGAGACAGAGACATTCAGGCCTGGAGGAGGAGATATGAGGGACAATTGGAGAAGTGAATTATACAAATACA
AAGTGGTAGAAATTAAGCCATTA---GGATTAGCACCCACTGAGGCAAAAAGGAGAGTGGTG-----------
----------------------------GAGAGAGAAAAAAGAGCA---GTGGGA---CTGGGAGCG------
---ATGTTCCTTGGGTTCTTAGGAGCA---GCAGGAAGCACTATG---GGCGCAGCATCA---
ATAACGCTGACGGTACAG---GCCAGACAA---TTATTG---TCTGGTATAGTGCAACAGCAAAGC---
AATTTG---CTGAGA---GCTATAGAGGCGCAACAGCATATGTTGCAACTCACAGTCTGGGGCATT
>KC156213
TTCTATTGTAATACATCAGGACTGTTCAATAGTACATACAATAGTACATACAATGGTCCATACAAT-------
----

GGTACAGCAATAATCACACTCCCATGCAGGATAAAACAAATTATAAACATGTGGCAGGAGGTAGGCAGAGCAA
TGTACGCCCCTCCCATTGAAGGAAACATAACATGTAACTCAAGC---
ATCACGGGACTACTCTTGGTACGTGATGGAGGCGAAACAAATAGTTCC-------------------------
----

CAAGAGGAGATATTCAGACCTGGAGGAGGAGATATGAGAGACAATTGGAGAAGTGAATTATATAAATATAAAG
TGGTAGAAATTAAACCATTA---GGAATAGCACCTACTGAGGCAAAAAGGAGAGTGGTG--------------
-------------------------GAGAGAGAAAAAAGAGCA---GTGGGA---ATAGGAGCT---------
GTGTTCCTTGGGTTCTTGGGAGCA---GCAGGAAGCACTATG---GGCGCAGCATCA---
ATAACGCTGACGGTACAG---GCCAGACAA---TTGTTG---TCTGGTATAGTGCAGCAGCAAAGT---
AATTTG---CTGAGA---GCCATAGAGGCGCAACAGCATATGTTGCAACTCACAGTCTGGGGCATT
>KC156119
TTCTATTGCAATACATCAAAACTGTTTAATAGTACATATAATTTTAATTTTAATGGTACAGAAGATAAC----
----

TCAAGCTCAGTCATCACTCTCTCATGCAGAATAAAACAAATTATAAACATGTGGCAAGAGGTAGGACGAGCAA
TGTATGCCCCTCCCATTGAAGGAAAAATAACATGTAGGTCAGAT---
ATCACAGGACTACTATTGACACGTGATGGAGGAAACAATAATACA----------------------------
----

GAGGAAATATTTAGACCTGGGGGGGGAAATATGAAAGACAATTGGAGAAGTGAGTTATATAAATACAAGGTGG
TAGAAATAAAGCCATTA---GGAGTAGCACCCACTAAGGCAAAAAGAAGAGTGGTG-----------------
----------------------GAGAGAGAGAAAAGAGCA---GTAGGA---ATAGGAGCT---------
GTGTTCCTTGGGTTCTTGGGAGCA---GCAGGAAGCACTATG---GGCGCGGCGTCA---
ATAACGCTGACGGTACAG---GCCAGACAA---TTATTG---TCTGGTATAGTGCAACAGCAAAGT---
AATTTG---CTGAAG---GCTATAGAGGCGCAACAGCATATGTTGCGACTCACAGTCTGGGGCATT
>KC894109
TTCTATTGCGATACATCAGAACTGTTTAATGGTACATACAATAATAGTACATACAAT----------------
----

AGTAGTAATAATATCACACTCCAATGCAAAATAAAACAAATCATAAACATGTGGCAGGGAGTAGGACGAGCAA
TGTATGCCCCTCCCATTGCAGGAAACATAACATGTGAATCAAAT---
ATCACAGGACTATTATTGACACGGGATGGAGGTAATAATAAGAGC----------------------------
----

ACTCCTGAAACATTCAGACCTGGAGGAGGAGATATGAGGGACAATTGGAGAAGTGAATTATATAAATATAAAG
TGGTAGAAATTAAGCCATTA---GGAATAGCACCCACTAAGGCAAAAAGGAGAGTGGTG--------------
-------------------------GAGAGAGAAAAAAGAGCA---GTGGGA---ATGGGAGCT---------
GTGTTCCTTGGGTTCTTGGGAGCA---GCCGGAAGCACTATG---GGCGCAGCATCA---
TTAACGCTGACGGTACAG---GCCAGACAA---TTGCTG---TCTGGTATAGTGCAACAGCAAAAC---
AATTTG---CTGAGA---GCTATAGAGGCGCAACAGCATATGTTGCAACTCACGGTCTGGGGCATT
>KC148599

TTCTATTGCAATACATCAGGACTGTTTAATGCTACAAGCAATTTTACAAGAGAT-------------------
----

TCAAATTCAACTATCACAATCCCATGCAGAATAAAGCAAATTATAAACATGTGGCAGGAGGTAGGAAAAGCAA
TGTATGCCCCTCCCATAGAAGGAAACATAACATGTGTATCAAAT---
ATCACAGGACTACTATTGGTACGTGATGGAGGTGGGACAAATAACTCAACCTCA-------------------
----

ACAAATGAGACATTCAGACCTGGAGGAGGAGATATGAGGGACAATTGGAGAAGTGAACTATATAAATATAAAG
TGGTAGAAATTAAGCCATTG---GGAATAGCACCCACTAAGGCAAAAAGGAGAGTGGTG--------------
-------------------------GAGAGAGAAAAAAGAGCA---GTAACA---ATAGGAGCT---------
GTATTCCTTGGGTTCTTGGGAGCA---GCAGGAAGCACTATG---GGCGCGGCGTCA---
ATGACGCTGACGGTACAG---GCCAGACAA---CTGTTG---TCTGGTATAGTGCAACAGCAAAGC---
AATTTG---CTGCGG---GCTATAGAGGCGCAACAACATATGTTGCAACTCACAGTCTGGGGCATT
>KC148663
TTCTATTGCAATACAACAAGTCTGTTTAACGGTCTGTTTAACGGTACATATATGTCTAATAGTACAGATAGTA
CT---

GAAAATGGAACCATCACACTCCCATGCAGAATAAAACAAATCATAAACATGTGGCAGAAGGTAGGACGAGCCA
TATATGCCCCTCCCATTGCAGGAAACATAACATGTATATCAGAT---
ATCACAGGATTACTATTGACACGTGATGGAGGAATAAATAACACA----------------------------
----

GAGGATGAGACGTTCAGACCTGGCGGAGGAGATATGAGGGACAATTGGAGAAGTGAATTATACAAATATAAAG
TGATAGAAATTAAGCCACTA---GGAATAGCACCCACTACTGCAAAAAGGAGAGTGGTG--------------
-------------------------GAAAAAGAAAAAAGAGCA---TTGGGA---GTAGGAGCT---------
GTGCTCCTTGGGTTCTTGGGAGCA---GCAGGAAGCACTATG---GGCGCAGCATCA---
ATAACGCTGACGGTACAG---GCCAGACAA---TTGCTG---TCTGGTATAGTGCAACAGCAAAAC---
AATTTG---CTGAAA---GCTATAGAGGCGCAACAGCATTTGTTGCAACTCACGGTCTGGGGCATT
>HM204583
TTCTATTGTAATACATCAGGCCTATTTAATAGAACAGCAAACATGACTAGAACGGCAAACATGACT-------
----

ACAAATTCAACCATTATACTCCGATGCAGAATAAAACAAATTGTAAACATGTGGCAAGGGGTAGGACGAGCAA
TGTATGCTCCTCCCATTGCAGGAAACATAACATGTGCATCTAAT---
ATCACAGGACTACTGTTGACACGTGATGGAGGAGAAAATAACACC----------------------------
----

GAAAATAAGGAGATATTCAGACCAGGAGGAGGAGACATGAGGGACAATTGGAGAAGTGAATTATATAAATATA
AAGTAGTAGAAATTAAGCCATTA---GGAATAGCACCCACTACGGCAAAAAGGAGAGTGGTG-----------
----------------------------GAGAGAGAAAAAAGAGCA---GTGGGA---ATAGGAGCT------
---GTGCTCCTTGGGTTCTTGGGAGCA---GCAGGAAGCACTATG---GGCGCGGCGTCA---
CTGACGCTGACGGTACAG---GCCAGACAA---TTGTTG---TCTGGTATAGTGCAACAGCAAAGC---
AATTTG---CTGAGA---GCTGTAGAGGCGCAACAGCACATGTTGCAACTCACGGTCTGGGGCATT
>HM204591
TTCTATTGCAATACATCAAACCTGTTCGATAAATTAAATAGTACT----------------------------
----

AATGGTACAAACATCACACTCCCATGCAGAATAAAACAAATTATAAACACGTGGCAGGGGGTAGGACGGGCAA
TATATGCAGCTCCTATTGCAGGAAAAATACAATGTATATCAAAT---
ATCACAGGACTACTATTGACACGTGATGGAGGAAAGGACAATAATAATAATACA-------------------
----

AATAACAGTATTACTGAGACAATCAGACCTGGAGGAGGAGATATGAGGGACAATTGGAGAAGTGAACTATATA
AATATAAAGTAGTAGAAATTAAGCCATTA---GGAATAGCACCCACTAATGCAAAAAGGAGAGTGGTG-----
----------------------------------GAGAGAGAAAAAAGAGCA---GTGGGA---
TTAGGAGCT---------ATGTTCCTTGGGTTCTTGGGAATG---GCCGGAAGCACTATG---
GGCGCAGCATCA---ATAACGCTGACGGTACAG---GCCAGACAA---GTATTG---
TCTGGTATAGTGCAACAGCAAAGC---AATTTG---CTGAGG---
GCTATAGAGGCGCAACAGCATATGTTGCAACTCACGGTCTGGGGCATT

>FJ443615
TTCTACTGCAATACATCACCATTGTTTAATAGTACATACGATCTTAATGGT----------------------
----

ACTGCTTCAACCATCACACTCCAATGTAAAATAAAACAAATCATAAACATGTGGCAAGGGGTAGGAAAAGCAA
TGTATGCTCCTCCCATTGAAGGGAACATAACATGTAACTCAAGT---
ATTACAGGATTACTATTGACACGTGATGGAGGAACACGAGATCGA----------------------------
----

AATGACACAGAAACATTTAGACCTGGAGGAGGAGATATGAGGGACAATTGGAGAAGTGAGTTATACAAATATA
AAGTGGTGGAAATTAAGCCATTG---GGAATAGCACCCACTGAGGCAAAAAGGAGAGTGGTG-----------
----------------------------GAAAGAGAAAAAAGAGCA---GTAGGA---ATAGGAGCT------
---GTGTTCCTTGGGATCTTGGGAGCA---GCAGGAAGCACTATG---GGCGCGGCGTCA---
ATAACGCTGACGGTACAG---GCCAGACAA---CTGTTG---TCTGGTATAGTGCAACAGCAAAAC---
AATTTG---CTGAGA---GCTATAGAGGCGCAACAGCATATGTTGCAACTCACGGTCTGGGGCATT
>KP109520
TTCTATTGCAATACATCAAACCTATTTAATAGTACGTTGTCTAATAGTACTAACAAT----------------
----

AGTACTTCAACCATCACACTCCAATGTAAAATAAAACAAATAATAAACATGTGGCAGGGGGTAGGACGGGCCA
TGTATGCCCCTCCCATTGCAGGAAACATAACATGTAAATCAAAT---
ATCACAGGAATACTTTTGTCACGTGATGGAGGCAAGGCTGAAAATGGT-------------------------
----

ACAAAGATAGAGATATTCAGACCTGGAGGAGGAGATATGAGAGACAATTGGAGAAGTGAACTATACAAATATA
AGGTGATAGAAATAAAACCATTA---GGAATAGCACCCACTAAGGCAAAGAGGAGAGTGGTG-----------
----------------------------GAGAGAAAAAAAAGAGCA---GTAGGA---ATAGGAGCT------
---GTGTTCCTTGGGTTCTTGGGAGCA---GCCGGAAGCACTATG---GGCGCAGCATCA---
ATAACGCTGACGGTACAA---GCCAGACAA---TTGTTG---TCTGGTATAGTGCAACAGCAAAGC---
AATTTG---CTGAAG---GCTATAGAAGCGCAACAACATATGTTGCAACTCACAGTCTGGGGCATT
>HM070539
TTCTACTGCAATACCTCAGCACTGTTTAATAGTACATGGAGGTGTGATAATACTTCATGTTCACGTGTGTCTA
ATAAT--
-------------------ACA---
GATAATTCTCCAAACATCACACTCCAATGCAGAATAAAACAAATTATAAACATGTGGCAGGGGGTAGGAAGAG
CAATGTATGCCTCTCCCATTGCAGGAAACATAACATGTAGATCAAAC---
ATTACAGGACTACTATTGACATGGGATGGAGATAATAGTAGTAAT----------------------------
----

ACAACAGAGACATTCAGACCTGGAGGAGGAAATATGAAAGACAATTGGAGAAGTGAATTATATAAATATAAAG
TGGTAGAAATTAAGCCATTA---GGAATAGCACCTACTGGGGCAAAAAGGAGAGTGGTG--------------
-------------------------GAGAGAGGGAAAAGAGCA---GTGACA---CTAGGAGCT---------
GTGTTCCTTGGGTTCTTGGGAGCA---GCAGGAAGCACTATG---GGCGCGGCGTCA---
ATAACGCTGACGGTACAG---GCCAGACAA---TTATTG---TCTGGTATAGTGCAACAGCAGAGC---
AATCTG---CTGAGA---GCTATAGAGGCGCAGCAGCATCTGTTGCAACTCACGGTCTGGGGCATT
>HQ595984
TTCTATTGCGATACATCAACATTGTTTAATAGTACATACATGCCTAATATTACTTCAAATTCAAGTACTTCAA
AT---

TCAAGTTCACTCATCACACTCCAATGCAAAATAAAACAAATTATAAATATGTGGCAGGAGGTAGGACGAGCAA
TGTATGCCCCTCCCATTAAAGGGAACATAACATGCAACTCAACT---
ATCACAGGATTAATATTGACACGTGATGGAGGAACTGGAAATAACACT-------------------------
----

AACGATACAGAGATATTCAGACCTGGAGGAGGAGATATGAGAGACAATTGGAGAAGTGAATTATATAAATATA
AAGTGGTAGAAATTAAGCCATTA---GGAGTGGCACCCACTAAGGCAAAAAGGAGAGTGGTG-----------
----------------------------GAGAGAGAAAAAAGAGCA---GTGGGA---ATAGGAGCT------
---GTGCTCCTTGGGTTCTTGGGAGCA---GCAGGAAGCACTATG---GGCGCAGCATCA---
ATAACGCTGACGGTACAG---GCCAGACAA---TTATTG---TCTGGTATAGTGCAACAGCAAAGC---
AATTTG---CTGAGA---GCTATAGAGGCGCAACAGCACCTGTTGCAACTCACAGTCTGGGGCATT

```
>HQ596030
TTCTATTGCAATACATCAGGCCTGTTTAATGGGACATATGATCCTAGATATAATAGTACATATAATAGTACAG
AAAAT--------------------------------------------------------------------
-------------------AAT---
AGTACAACCCATAACATCACACTCCAATGCAGAATAAAGCAAATTATAAACATGTGGCAGGAGGTAGGAAAAG
CAATATATGCCCCTCCTATTGCAGGAAGTCTAACATGTACATCAAAC---
ATCACAGGACTACTATTGGTACGTGATGGAGGTAAAAGAAACAATACC-------------------------
-------------------------------------------------------------------------
----------
AATGACCCAGAGATATTCAGACCTGGAGGAGGAAATATGAAGGACAATTGGAGAAGTGAATTATATAAATATA
AAGTAGTAGAAATTAAGCCCTTA---GGAGTAGCACCCACTGAGGCAAAAAGGAGAGTGGTG-----------
----------------------------GAGAGAGAAAAAAGAGCA------------ATAGGAGCT------
---GTGTTCCTTGGGTTCTTGGGAGCA---GCAGGAAGCACTATG---GGCGCGGCGTCA---
GTGGCGCTGACGGTACAC---ACCAGAAAA---CTGTTG---TCTGGTATAGTGCAACAGCAAAGC---
AATTTG---CTGAGA---GCTATAGAGGCGCAACAGCATATGTTGCAACTCACGGTCTGGGGCATT
>HM070639
TTCTATTGCAATACATCAAACCTGTTTAATAAGACATATTGGATTAATAGTACAGAAGGTAATACAGAAGATG
ATACA--------------------------------------------------------------------
-------------------------
AGCTCAAACTCAACGATCACACTCCCATGCAGAATAAAACAAATTATAAATATGTGGCAGGGGGTAGGACGAG
CAATATATGCCCCTCCCATTGCAGGAAACATAACATGTAAATCAAAT---
ATCACAGGACTACTATTGACACGTGATGGAGGAAATGATAATAGA----------------------------
-------------------------------------------------------------------------
----------
AGTACAAATGAAACATTCAGACCTGGAGGAGGAGATATGAGGGACAATTGGAGAAGTGAATTATACAAATATA
AAGTGGTAGAAATTAAGCCATTA---GGAATAGCACCAACTGCTGCCAAGAGAAGAGTGGTG-----------
----------------------------GAGAGAGAAAAAAGAGCA---GTGGGA---ATAGGAGCT------
---GTGTTCCTTGGGTTCTTGGGAGCA---GCAGGAAGCACTATG---GGCGCAGCGTCA---
ATAACGCTGACGGTACAG---GCCAGACAA---TTATTG---TTTGGTATAGTGCAACAGCAAAGC---
AATTTG---CTGAGG---GCCATAGAGGCGCAACAGCATATGTTGCAACTCACGGTCTGGGGCATT
>KF527172
TTCTATTGCGATACATCAGGATTGTTTAATGGCACATACAATCCTAATGATACAACAAGTAATTCAAGCTCAC
TT-----------------------------------------------------------------------
----------------------------
AACAACATCACACTCACACTCCCATGCAAAATAAAGCAAGTTATAAACATGTGGCAGGGGGTAGGAAAAGCGA
TATATGCCCCTCCCATTGCAGGAAACATAACATGTAGATCAAAT---
ATCACAGGACTACTATTGCTACGTGATGGAGGAACTACAAATGAC----------------------------
-------------------------------------------------------------------------
-------------
ACAGAAGAGATATTCAGACCTGGAGGAGGAGATATGAGGGACAATTGGAGAAGTGAATTATATAAATACAAAG
TGGTAGAAATTAAGCCATTG---GGAGTAGCACCCACTAAGGCAAAAAGGAGAGTGGTG--------------
-------------------------GAGAGAGAAAAAAGAGCA---GTGGGA---ATAGGAGCT---------
GTACTCCTTGGGTTCTTAGGAGCG---GCAGGAAGCACTATG---GGCGCGGCGTCA---
ATAACGCTGACGGTACAG---GCCAGACAA---CTGTTG---TCTGGTATAGTGCAACAGCAAAGC---
AATTTG---CTGAGG---GCTATAGAGGCGCAACAGCATATGTTGCAACTCACGGTCTGGGGCATT
>HM070807
TTCTATTGCAATACATCACGCCTGTTTAATGGTACATATAATAGTACAAGATATAATACAACAGAA-------
-------------------------------------------------------------------------
----------------------------
GCAAATTCAACCATCACACTCCAATGCAGAATAAAACAAATTATAAACATGTGGCAGGAGGTAGGACGGGCGA
TGTATGCCCCTCCCATTGCAGGAAACATAACATGTAAATCAAAT---
ATCACAGGACTACTATTGACACGTGATGGAGGACACGGAAATGGGACA-------------------------
-------------------------------------------------------------------------
-------
GACAATAAGACAGAAACATTTAGACCAGGAGGAGGAGATATGAGGGACAATTGGAGGAGTGAATTATATAAAT
ATAAAGTGGTAGAAGTTAAGCCATTA---GGAATAGCACCCACTAAGGCAAAAAGAAGAGTGGTG--------
-------------------------------GAGAGAGAGAAAAGAGCA---GTGGGA---GTAGGAGCT---
------ATGTTCCTTGGGTTCTTGGGAGCG---GCCGGAAGCACTATG---GGCGCAGCATCA---
ATAACGCTGACGGTACAG---GCCAGACAA---TTATTG---TCTGGTATAGTGCAACAGCAAAGC---
AATTTG---CTGAGG---GCTATAGAGGCGCAACAGCATATGTTGCAACTCACGGTCTGGGGCATC
```

>HQ596147
TTCTATTGTAATACATCAAAACTGTTTAATAGTACATACATGCCTAATAGTACTTCAAATTCAACACACATG-
----

TCTAATGGTACCATCACACTCCAATGCAGAATAAAACAAATTATAAACATGTGGCAGGAGGTAGGACGAGCGA
TGTATGCCCCTCCTATCGAAGGAAACATAATATGTAAATCAAAT---
ATCACAGGAATACTATTGACACGTGATGGAGGTAAGAATGAGACT----------------------------
----

ACAGAGACATTCAGACCTGGAGGAGGAGATATGAGGGACAATTGGAGAAGTGAGTTATATAAATATAAAGTGG
TAGAAGTTAAGCCATTA---GGGATAGCACCTACTACTGCAAAAAGGAGAGTGGTG-----------------
----------------------GAGAGAGAAAAAAGAGCA---GTGGGA---ATAGGAGCT---------
GTGTTCCTTGGGTTCTTGGGTGCA---GCAGGAAGCACTATG---GGCGCGGCGTCA---
ATAACGCTGACGGTACAG---GCCAGACAA---TTATTG---TCTGGTATAGTGCAACAACAAAGC---
AATTTG---TTGAGG---GCCATAGAGGCGCAACAACACATGTTGCAACTCACGGTCTGGGGCATT
>KC156218
TTCTATTGTGATACATCAAAACTGTTTAATAGGACATACACGGTTAATGACACACACGAGACTAATAGTACTG
AT---

ACAAACAGTACCATCACACTCCCATGCAGAATAAAACAAATTATAAACATGTGGCAGAGAGTAGGACAAGCAA
TGTATGCTTCTCCCATTGCAGGAAACATAACATGTAACTCAAGT---
ATCACAGGATTGCTACTGACGAGTGATGAAGGAGGAAATAACAAC----------------------------
----

GAAAACGAGACATTCAGGCCTGGAGGAGGAGATATGAGGGACAATTGGAGAAGTGAATTATATAAATATAAAG
TGGTAGAAGTTAAGCCATTG---GGAATAGCACCCACCAAGGCAAAAAGGAGAGTGGTG--------------
-------------------------GAGAGAGAGAAAAGAGCA---GTAGGA---ATAGGAGCT---------
TTGTTCCTTGGGTTCTTGGGAGCA---GCAGGAAGCACTATG---GGCGCGGCGTCA---
ATGACGCTGACGGTACAG---GCCAGACAA---TTGTTG---TCTGGTATAGTGCAACAGCAAAGC---
AATTTG---CTGAGG---GCTATAGAGGCGCAACAACATATGTTGCAACTCACAGTCTGGGGCATT
>KC156215
TTCTATTGTAATACATCACGCCTGTTTAATAGTACATACAATGGTACAGACGGGTCTAAT-------------
----

AGCACAACAAACATCACACTCCCATGCAGAATAAAACAAATTATAAACATGTGGCAGGAGGTAGGACGAGCAA
TGTATGCCCCTCCCATTGCAGGAAACATAACATGTAAATCAAAT---
ATCACAGGAATACTATTGACACGTGATGGAGGAGGGAACAGCACA----------------------------
----

GACCCAGAGATATTCAGACCTGGAGGAGGAGATATGAGGGACAATTGGAGAAGTGAATTATACAAATATAAAG
TGGTAGAAATTAAGCCATTA---GGAATAGCACCCACTACTGCAAAAAGGAGAGTGGTG--------------
-------------------------GAGAGAGAGAAAAGAGCA---GTGGGA---ATAGGAGCT---------
GTGTTCCTTGGGTTCTTGGGAGCA---GCAGGAAGCACTATG---GGCGCGGCGTCA---
ATAACGCTGACGGTACAG---GCCAGACAA---TTATTG---TCTGGTATAGTGCAACAGCAAAAC---
AATTTG---CTGAGA---GCTATAGAGGCGCAACAGCATATGTTGCAACTCACAGTCTGGGGCATT
>HQ615962
TTCTATTGCGATACATCAAAACTGTTTAATAGGACATACACCAATGACACACACTTCAAAGAGACAGATCCAA
GT---

ATAAATGGCACAATCACAATCACATGCAAAATAAAACAATTTATAAACATGTGGCAGGAGGTAGGAAAAGCAA
TGTATGCCCCTCCCATTGCAGGAAACATAACATGTAAGTCAAAT---
ATCACAGGAATACTATTGGTACGCGATGGGGGTAAAACCAATAGT----------------------------
----

ACAAATGAGACATTCAGACCTGGAGGGGGAGACATGAGGGACAATTGGAGAAGTGAATTATATAAATATAAAG
TGGTAAGAATTGAGCCATTG---GGAGTAGCACCCACTACGGCAAAAAGGAGAGTGGTG--------------
-------------------------GAGAGAGAAAAAAGAGCA---GTGGGA---CTAGGAGCT---------
GTGTTCCTTGGGTTCTTGGGGGCA---GCCGGAAGCACTATG---GGCGCGGCGTCA---
ATAACGCTGACGGTACAG---GCCAGACAA---CTGTTG---TCTGGCATAGTGCAGCAGCAAAGC---
AATTTG---CTGATG---GCTATAGAGGCGCAACAACATATGTTGCAACTCACAGTCTGGGGCATT

>KY112213
TTCTATTGTAATACATCAGGTCTGTTTAATAGGACATACTCGCTTAATAATGATACAGAACAGAATAATAGT-
----

ACAAACAAAACCATCACACTCCCATGCAGAATAAAACAAATTATAAACATGTGGCAGGAGGTAGGACGAGCCA
TGTATGCCCCTCCCATTGCAGGAAACATAACATGTAACTCAAAT---
ATTACAGGACTACTATTGACACGTGATGGAGGAGAAGGAAATGAT----------------------------
----

AGTGATGAGATTTTCAGACCTGGAGGAGGAGATATGAGAGACAATTGGAGAAGTGAATTATATAAATACAAAG
TAGTAGAAATAAAACCATTA---GGAATAGCACCCACTAAGGCAAGAAGGAGAGTGGTG--------------
-------------------------GAGAGAGAGAAAAGAGCA---GTGGGA---ATAGGAGCT---------
GTGTTCCTTGGGTTCTTGGGAGCA---GCAGGAAGCACTATG---GGCGCAGCGTCA---
ATAACGCTGACGGTACAG---GCCAGACAA---TTATTG---TCTGGAATAGTGCAACAGCAAAGC---
AATTTG---CTGAGG---GCAATCGAGGCGCAACAGCATATGTTGCAACTCACGGTCTGGGGCATT
>KP109521
TTCTATTGCAATACATCAAAATTGTTTAATAGTACATTTAATAGTACACACATGGGTAATGACACACACAGGA
ATAATGAT---

ACAGACAGTACCATCACGATCCCATGCAGAATAAAACAAATTATAAACATGTGGCAGAGGGTAGGACAAGCAA
TGTATGCTTCTCCCATTGCAGGAAACATAACATGTAACTCAAGT---
ATCACAGGACTGCTATTAACACGTGATGGAGGTAACGAGACAGGC----------------------------
----

GAGGAAGAGACATTCAGGCCTGGAGGAGGAGATATGAGGGACAATTGGAGAAGTGAATTATATAAATATAAAG
TGGTAGAAGTTAAGCCATTG---GGAATAGCACCCACCAAGGCAAAAAGGAGAGTGGTG--------------
-------------------------GAGAGAGAGAAAAGAGCA---GTGGGA---ATAGGAGCG---------
TTGTTCCTTGGGTTCTTGGGAGCA---GCAGGAAGCACTATG---GGCGCGGCGTCA---
ATGACGCTGACGGTACAG---GCCAGACAA---TTGTTG---TCTGGTATAGTGCAACAGCAAAGC---
AATTTG---CTGAGG---GCTATAGAGGCGCAACAACATATGTTGCAACTCACGGTCTGGGGCATT
>KP109522
TTCTATTGTAATACATCAAGATTGTTTACATACAATGGTACAGAAAGTAAGAATTCAACAAACAAAAGTAAG-
----

AATTCAACAGACATCATACTCCCATGCAGAATAAAGCAGATTATAAACATGTGGCAAGAGGTAGGACGAGCAA
TGTATGCTCCTCCCATTGCAGGAAACATAACATGTACGTCAAAT---
ATCACAGGACTACTATTGACACGAGATGGAGGAGGTAAAAATAACACA-------------------------
----

AAAGAAGAGATATTCAGACCTGGAGGAGGAGATATGAGGGACAATTGGAGAAGTGAATTATACAAGTATAAAG
TGATAGAAATTAAGCCATTA---GGAATAGCTCCAACTACGGCAAAAAGGAGAGTGGTG--------------
-------------------------GAGAGAGAAAAAAGAGCA---GTGGGA---ATAGGAGCT---------
GTGCTCCTTGGGTTCTTGGGAGCA---GCCGGAAGCACTATG---GGCGCAGCATCA---
ATAACGCTGACGGTACAG---GCCAGACAA---TTGCTG---TCTGGTATAGTGCAACAGCAAAGC---
AATTTG---TTGAGG---GCTATAGAGGCGCAACAGCATATGTTGCAACTCACAGTCTGGGGCATT
>KC634164
TTCTATTGCAATACATCAAACTTGTTTAATAGTAGTATACCA--------------------------------
----

AATTCAACTATCACACTCCAGTGCAGAATAAAACAGATTATAAACATGTGGCAGGAGGTAGGACGAGCAATGT
ATGCCCCTCCCATTGCAGGAAACATAACATGTAAGTCAAAT---
ATCACAGGACTATTATTGACACGTGATGGAGGGACAGATAATAACAATACAAAT-------------------
----

ACTAACAATACAGAGACATTCAGACCTGGAGGAGGAGATATGAGAGACAATTGGAGAAGTGAACTATATAAAT
ATAAAGTAGTAGAAATTAAGCCCTTA---GGAATAGCACCTACTAAGGCAAAAAGGAGAGTGGTG--------
----------------------------------GAGAGAGAAAAAAGAGCA---GTGGGA---ATAGGAGCT---
------GTACTCCTTGGGTTCTTGGGAGCA---GCAGGAAGCACTATG---GGCGCGGCGTCA---
ATAACGCTGACGGTACAG---GCCAGACAA---TTATTG---TCTGGTATAGTGCAACAGCAAAAC---
AATTTG---CTGAGG---GCTATAGAGGCGCAACAGCATATGTTGCAACTCACGGTCTGGGGCATT

>KC187352
TTCTATTGTAACACATCAAACCTGTTTAATAGGACATACTCGTCTAATAGTACAAATAGTGCCTCAAATGCTA
CC---

TCAAATGCAACCATCACACTCCCATGCAGAATAAAGCAAATTATAAATATGTGGCAGGGAGTAGGACAAGCAA
TATATGCCCCTCCCGTTGCAGGAAACATAACATGTAACTCAAGC---
ATCACAGGACTACTATTGACACGTGATGGAGGAAATACTAATGGAGAAAAT----------------------
----

AATGATACAGAGACATTCAGACCTGGAGGAGGAGATATGAGGGACAATTGGAGAAGTGAATTATATAAATATA
AAGTGGTAGAAATTAAACCATTA---GGAATAGCACCCACTAAGGCAAAAAGGAGAGTGGTG-----------
----------------------------GAGAAAGAAAAAAGAGCA---GTGGGA---ATAGGAGCT------
---GTGTTCCTTGGGTTCTTGGGAGCA---GCAGGAAGCACTATG---GGCGCAGCATCG---
ATAACGCTGACGGTACAG---GCCAGACAA---TTGTTG---TCTGGTATAGTGCAACAGCAAAGT---
AATTTG---CTGAGG---GCTATAGAGGCGCAACAGCATATGTTGCAACTCACAGTCTGGGGCATT
>KC186723
TTCTATTGCAATACATCACAATTGTTTAATATTACAAACACAAGTAATATTACAAACACA-------------
----

AGTACTGGGAATATAATTCTCCAATGTAGAATAAAACAAATTATAAACATGTGGCAGGAGGTAGGAAGAGCAA
TGTATGCCCCTCCCATTGAAGGAAACATAACATGTAAATCAAAT---
ATCACAGGACTACTATTGACACGGGATGGAGGAAACACAGATAAT----------------------------
----

ACAACTGAGAAATTTAGGCCTGGAGGAGGAGATATGAGGGACAATTGGAGAAGTGAACTATATAAATACAAAG
TGGTAGAAATTAAGCCATTA---GGAGTAGCACCCACTGAGGCAAAAAGGAGAGTGGTG--------------
-------------------------GAGAGAGAAAAAAGAGGA------------ATAGGAGCT---------
GTGTTCCTTGGGTTCTTGGGAGCA---GCAGGAAGCACTATG---GGCGCAGCGTCA---
ATAACGCTGACGGTACAG---GTCAGACAA---TTATTG---TCTGGCATAGTGCAACAGCAAAGC---
AATTTG---CTGAGG---GCTATAGAGGCGCAACAGCATATGTTGCAACTCACAGTCTGGGGCATT
>KC186899
TTCTATTGCAACACATCAAGGCTGTTCAATAGCACATTTAATAGTACATTTAATAGTACACTGGATAACAGTA
CAAAT--

AGTACAGGAAACATCACACTCCAATGCAGAATAAAACAAATTATAAACATGTGGCAGGGGGTAGGAAAAGCAA
TATATGCCCCTCCCGTTGCAGGAGACATAATGTGTGAATCAAAT---
ATCACAGGATTACTATTAACAAGGGATGGAGGAAATGAAAATAAT----------------------------
----

AAAACAGAGACATTCAGACCTGCAGGAGGAAATATGAGGGACAATTGGAGAAGTGAATTATATAAATATAAAG
TGGTAGAAATTAAGCCATTA---GGAATAGCACCCACTAAGGCAAAAAGGAGAGTGGTG--------------
-------------------------GAGAGAGAAAAAAGAGCA---GTGGGA---ATAGGAGCT---------
GTGTTCCTTGGGTTCTTGGGAGCA---GCAGGAAGCACTATG---GGCGCAGCATCG---
ATAACGCTGACGGTACAG---GCCAGACAA---TTGCTG---TCTGGTATAGTGCAACAGCAAAGT---
AACTTG---CTGAGG---GCTATAGAGGCGCAACAGCAAATGTTGCAACTCACGGTCTGGGGCATT
>KC186242
TTCTATTGCAATACATCAGGTCTGTTTAGAGATACAAACATATCT----------------------------
----

ACTACGCTCCAATGCAGGATAAAACAAATTATAAATATGTGGCAGGGGGTAGGGCGAGCAATGTACGCCCCTC
CCATTGCAGGAATCATAAAGTGTAAATCAAAG---
ATCACAGGAATACTATTGACAAGGGATGGAGGGGCAGATAATACC----------------------------
----

ACAGAGACATTCAGACCTGCAGGAGGAGATATGAGGGACAATTGGAGAAGTGAATTATATAAATATAAAGTAG
TAGAAATTAAGCCATTA---GGAATAGCACCAACTGAGTCAAAAAGGAGAGTGGTG-----------------
----------------------GAGAGAGAAAAAAGAGCA---GTGGGAGTAGTAGGAGCT---------
ATGTTCCTTGGGTTCTTGGGAGCA---GCCGGAAGCACTATG---GGCGCAGCGGCA---
GTGACGCTGACGGCACAG---GCCAGACAA---TTATTG---TCTGGTATAGTGCAACAGCAAAGC---
AATTTG---CTGAGG---GCTATAGAGGCGCAACAGCATATGTTGCAACTCACAGTCTGGGGCATT

>KC187207
TTCTACTGCAATACATCAAAACTGTTTAATAGTACAATGTTTAGTACTACAGAGCATTCAAGTGAT-------
----

TCAAGCGAAAACATCACACTCCCATGCAGATTAAAGCAAATTATAAACATGTGGCAGGAGGTAGGAAGAGCAA
TATATGCCCCTCCCATTGCAGGAAACATAACATGTAAATCAAAT---
ATCACAGGGCTACTATTGCTACGTGATGGAGGTAATGATGGTAAAAAT-------------------------
----

AACACAAATGAAACATTCAGACCTGGAGGAGGAGATATGAGGGACAATTGGAGAAGTGAATTATACAAATACA
AAGTGGTAGAAATCAAGCCATTA---GGAGTGGCACCCACTAATGCAAAAAGGAGAGTGGTG-----------
----------------------------GAGAGAGAAAAAAGAGCA---GTGGGA---CTAGGAGCT------
---GTGTTCCTTGGGTTCTTGGGAGCA---GCAGGAAGCACTATG---GGCGCAGCGTCA---
ATAACGCTGACGGTACAA---GCCAGACAA---CTGTTG---ACTGGTATAGTGCAACAGCAAAGC---
AATTTG---CTGAGG---GCTATAGAGGCGCAACAGCATATGTTGCAACTCACGGTCTGGGGCATT
>KC186306
TTCTATTGCAATACATCAGGGCTGTTTAATAGCACATACATGTCTGGTGACACCCCCATGAATGATACAGACA
GTAATAAT---
-------------------ACA---
GACAGTAATAGTACAATCACACTCCAATGCAGAATAAAACAAATTATAAACATGTGGCAACAGGTAGGACGAG
CAATGTATGCTTCTCCCATTGAAGGAAACATAACATGTAAATCAAAT---
ATCACAGGGTTACTATTGACACGTGATGGAGGAAAAAATAACCAA----------------------------
----

AGTGAAAATGAGACATTCAGACCTGGAGGAGGAGATATGAGGGACAATTGGAGAAGTGAATTATACAAATATA
AAGTGGTAGAAATTAAGCCATTA---GGAATAGCACCCACTAAGGCAAAAAGGAGAGTGGTG-----------
----------------------------GAGAGAGAAAAAAGAGCA---ATAACAGGAATAGGAGCT------
---GTGTTCCTTGGGTTCTTGGGAGCA---GCAGGAAGCACTATG---GGCGCGGCGTCA---
ATAACGCTGACGGTACAG---GCCAGACAA---TTATTG---TCTGGTATAGTGCAACAGCAAAGC---
AATTTG---CTGAGG---GCTATAGAGGCGCAACAGCATATGTTGCAACTCACAGTCTGGGGCATT
>KC187437
TTCTATTGCAATACATCAGGACTGTTTAATGGTACAAACACA-------------------------------
----

AGGAATGGCTCCATCACACTCCCATGCAGAATAAAACAAATTATAAACATGTGGCAGGGGGTAGGACGAGCAA
TATATGCCCCTCCCATTGAAGGAAACATAACATGTAAATCAAAT---
ATCACAGGACTACTATTGTCACGTGATGGAGGAAATGGAAATAACACA-------------------------
----

AGTACCACAGAGACATTCAGACCTGGAGGAGGAGATATGAGGGACAACTGGAGAAGTGAATTATACAAATATA
AAGTGGTAGAAATTAAGCCATTA---GGAGTAGCACCCACTGGGGCAAAAAGGAAAGTGGTG-----------
----------------------------GAGAGAGAAAAAAGAGCA---GTGGGA---ATAGGAGCT------
---GTGTTCCTTGGGTTCTTGGGAGCA---GCAGGAAGCACTATG---GGCGCGGCGTCA---
ATAACGCTGACGGTACAG---GCCAGACAA---CTGTTG---ACTGGTATAGTGCAACAGCAAAGC---
AATTTG---CTGAGG---GCTATAGAGGTGCAACAGCATATGTTGCAACTCACGGTCTGGGGCATT
>KC186647
TTCTATTGCAACACATCAATATTGTTTAATGGTACATACCTGACTAATAATACAAACAGTACTTCAAATACT-
----

TCAAATTCAACCATTACACTCCAATGCAAAATAAAACAAATTATAAACATGTGGCAGGGGGTAGGACAAGCAA
TGTATGCCCCTCCCATTTCAGGAAACATAACCTGTGAATCAAAT---
ATCACAGGACTATTATTGACACGTGATGGAGGAAATAACACAGAG----------------------------
----

AACACTGAGATATTCAGACCTGGAGGAGGGGACATGAGGGACAATTGGAGAAGTGAATTATATAAATATAAAG
TGGTAGAGATCAAGCCATTA---GGAGTAGCACCCAATAAGGCAAAAAGACGAGTGGTG--------------
-------------------------GAGAGAGAAAAAAGAGCA---GTAGGA---ATAGGAGCT---------
GTACTCCTTGGGTTCTTGGGAGCA---GCAGGAAGCACTATG---GGCGCGGCGTCA---
ATGACGCTGACGGTACAG---GCCAGACAA---TTATTG---TCTGGTATAGTGCAACAGCAAAAC---
AATTTG---CTGAGA---GCTATAGAGGCGCAACAGCATATGTTGCAACTCACGGTCTGGGGCATT

```
>KC187550
TTCTATTGCAATACATCAAAACTGTTTAATAGTACAAAACTGTTTAATAGTACTAATAAT-------------
-------------------------------------------------------------------------
----------------------------
AATAGTGCAATAATCACACTCCCATGTAAAATAAAACAAATTATAAATATGTGGCAGAAGGTAGGACGAGCAA
TGTATGCTCCTCCCATTGAAGGAAACATAACATGTAACTCAAGT---
ATCACAGGATTACTATTGACGCGTGATGGAGCAGGAGGGGGGGGC----------------------------
-------------------------------------------------------------------------
----------------
AATGAGACATTCAGACCTGGAGGAGGAGATATGAGGGACAATTGGAGAAGTGAATTATATAAATATAAAGTGG
TAGAAATTAAGCCATTA---GGAATAGCACCAACTGGAGCAAAAAGGAGAGTGGTG-----------------
----------------------GAGAGAGGAAAACGAGCA---GTGGGA---CTAGGAGCT---------
GTGTTCCTTGGGTTCTTGGGAGCA---GCAGGAAGCACTATG---GGCGCGGCGGCA---
GTAACGCTGACGGCACAG---ACCAGACAA---TTGTTG---TCTGGTATAGTGCAACAGCAAAGT---
AATTTG---CTGAAG---GCTATAGAGGCGCAACAACATATGTTGCAACTCACGGTCTGGGGCATT
>KC187376
TTCTATTGCAATACATCAGGCCTGTTTAGTGGAACATACAATAGTACAGGCAATACTAAT-------------
-------------------------------------------------------------------------
----------------------------
AGTACAACAAACATCACACTCCCATGCAGAATAAGACAAATTATAAGAATGTGGCAGGGAGTAGGACAAGCAA
TGTATGCCCCTCCCATTGCAGGAAACATAATATGTATTTCAAAT---
ATCACAGGGCTACTATTGACACGTGATGGAGGACAAAATAATGGGACAAAT----------------------
-------------------------------------------------------------------------
----------
AATAATACAGAGACATTCAGACCTGGAGGGGGAGACATGAGGGACAATTGGAGAAGTGAATTATATAAATATA
AAGTGGTAGAAATTAAGCCATTG---GGAATAGCACCCACTAAGGCAAAAAGGAGAGTGGTG-----------
----------------------------GAGAGAGAAAAAAGAGCA---GTGGGA---ATAGGAGCT------
---GTGTTCCTTGGGTTCTTGGGAGCA---GCAGGAAGCACTATG---GGCGCAGCGTCA---
ATAACGCTGACGGTACAG---GCCAGACAA---TTGTTG---TCTGGTATAGTGCAACAGCAAAGC---
AATTTG---CTGAAG---GCTATAGAGGCGCAACAGCATATGTTGCAACTCACGGTCTGGGGCATT
>KC187723
TTCTATTGCAATACATCAGCATTGTTTAACACAACACTGGGTAACACAACACTGGGTAACATAACCATACAAA
ATGAT--------------------------------------------------------------------
----------------------------
TCAAACGCAACCATCACACTCCCATGCAGAATAAAGCAGATTATAAACATGTGGCAGGAGGTAGGACGAGCAA
TGTATGCTCCTCCCATTAAAGGAAATATAACCTGTAAATCAAAC---
ATCACAGGACTACTATTGTTACGTGATGGAGGAGTGAACACAACT----------------------------
-------------------------------------------------------------------------
----------------
AACGAGACATTCAGACCTGGAGGAGGAGATATGAGGGACAATTGGAGAAGTGAATTATATAAATATAAAGTGG
TAGAAATTAAGCCATTG---GGAGTAGCACCCACTAAAGCAAAAAGGAGAGTGGTG-----------------
----------------------GAGAGAGAAAAAAGAGCA---GTGGGA---ATAGGAGCT---------
GTGTTCCTTGGGTTCTTGGGAGCA---GCAGGAAGCACTATG---GGCGCGGCGTCA---
ATAACGCTGACGGTACAG---GCCAGACAA---CTGTTG---TCTGGTATAGTGCAACAGCAAAGC---
AATTTG---CTGAGG---GCTATTGAGGCGCAACAGCATATGTTGCAACTCACGGTCTGGGGCATT
>KC186458
TTCTATTGCAATACATCAGGACTGTTTAATAACTCACTACTGACAGAAAATGGTACAGAA-------------
-------------------------------------------------------------------------
----------------------------
AGTAACGAAACAATAACAATCCCATGCAAAATAAAACAATTTTTAAACATGTGGCAGCAGGTAGGACGAGCAA
TGTATGCCCCTCCCATTGCAGGAAACATAACATGTAAATCAAAC---
ATCACAGGACTACTATTGACACGGGATGGAGGAACTACTAATGGGACA-------------------------
-------------------------------------------------------------------------
-------------
GGAAATGAGACATTCAGACCTGGAGGAGGAAATATGAGGGACAATTGGAGAAGTGAATTATATAAATATAAAG
TGGTAGAAATTAAGCCATTA---GGAATAGCACCCACTGGAGCAAAAAGGAGAGTGGTG--------------
-------------------------GAGAGAGAAAAAAGAGCA---GTGGGA---ATAGGAGCT---------
TTGTTCCTTGGGTTCTTGGGAGCA---GCAGGAAGCACTATG---GGCGCGGCGTCA---
ATAACGCTGACGGTACAG---GCCAGACAA---TTATTG---TCTGGTATAGTGCAACAGCAAAGC---
AATTTG---CTGAGA---GCTATAGAGGTGCAACAGCACATGTTGCAACTCACAGTCTGGGGCATT
```

>KC187049
TTCTATTGCAATACATCAAAGCTGTTCAATGGTACATATGTGAATAGTACATACGTGAATAGT----------
----

ACAGATTCAAACATCAACATCACATGCAGAATAAAACAAATTATTAACATGTGGCAGGGAGTAGGACGAGCAA
TGTATGCCCCTCCCATTGAAGGAGTCATAACATGTAAATCAAAT---
ATCACGGACTACTATTGACAAGAGATGGAGGAAAAAAATAATACT----------------------------
----

AATACAGAGACATTCAGACCTGGAGGGGGAGATATGAGGGACAATTGGAGAAGTGAATTATATAAATATAAAG
TGGTAGAAATTAAGCCATTA---GGAATAGCACCAACTGCGGCAAAAAGACGAGTGGTG--------------
-------------------------GAGAGAGAAAAAAGAGCA---GTGGGA---CTAGGAGCT----------
GTGTTCCTTGGGTTCTTGGGAGCA---GCAGGAAGCACTATG---GGCGCGGCATCA---
ATAACGCTGACGGTACAA---GCCAGACAA---TTGTTG---TCTGGTATAGTGCAACAGCAAAGC---
AATTTG---CTGAGG---GCTATAGAGGCGCAACAGCATATGTTGCAACTCACAGTCTGGGGCATT
>KC186320
TTCTATTGTAATACATCAGGACTGTTTAATGGTACATATTGGCCTAATGGTACAAATACATGGGGGAATGCAA
GCAAT--
-------------GAAAAC------
ATCACACTCCAAAACATCACACTCCCATGCAAAATAAAACAAATTATAAACATGTGGCAGGGAGTAGGACGAG
CAATATATGCCCCTCCCATTGCAGGAAACATAACATGTACATCAAAT---
ATCACAGGACTACTATTGGTACGTGATGGAGGGACAAATGAAAGTGCT-------------------------
----

ACAAATGAGACATTCAGACCTGGAGGAGGAGATATGAGGGACAACTGGAGAAGTGAATTATATAAATATAAAG
TGGTAGAAATTAAACCATTA---GGAGTAGCACCCACTGCTGCAAAAAGAAGAGTGGTG--------------
-------------------------GAGAGAGAAAAAAGAGCA---GTAACA---ATGGGAGCT----------
TTGTTCCTTGGGTTCTTGGGAGCA---GCAGGAAGCACTATG---GGCGCGGCGTCA---
ATAACGCTGACGGTACAG---GCCAGACAA---CTGTTG---TCTGGTATAGTGCAACAGCAAAGC---
AATTTG---CTGAGA---GCTATAGAGGCGCAACAGCATATGTTGCAACTCACGGTCTGGGGCATT
>KC187482
TTTTATTGCAATACATCAGATCTGTTCAATGATACATTT----------------------------------
----

ACAAACTCAACCATCACGCTTCCATGTAGGATAAAACAAATTATAAACATGTGGCAGGGGGTAGGACGAGCAA
TGTACGCCCCTCCCATTGAAGGAAACATAACATGTAAATCAAAT---
ATTACAGGACTACTATTGACACGTGATGGAGGAAACAGTAATAAT----------------------------
----

GGAACAGAGATATTCAGACCTGGAGGAGGAGATATGAGAGACAATTGGAGAAGTGAATTATATAAATATAAAG
TGGTAGAAATTAAGCCATTG---GGAATAGCACCCACTGAGGCAAAAAGGAGAGTGGTG--------------
-------------------------GAGAGAGAAAAAAGAGCA---GTGGGG---ATAGGAGCT----------
GTGTTCCTTGGGTTCTTGGGAGCA---GCAGGAAGCACTATG---GGCGCGGCGTCA---
ATAACGCTGACGGTACAG---GCCAGACAA---CTGTTG---TCTGGTATAGTGCAACAGCAAAGC---
AATTTG---CTGAGA---GCTATAGAGGCGCAACAACATCTGTTGCAACTCACAGTCTGGGGCATT
>KC186143
TTCTATTGCAATACATCAGGACTGTTTAATACATCAGGACAGTCTACAAAT----------------------
----

TCAAGTTCAAACATCACACTCCCATGCAAAATAAAACAAATTATAAACATGTGGCAGAAAGTAGGACGAGCAA
TATATGCCCCTCCCACTAAAGGAATCATAACATGTAGCTCAAAT---
ATCACAGGACTACTATTAACACGTGATGGAGGAAGCAACAAAAACAAT-------------------------
----

AACACTACAGAGATATTCAGACCTGGAGGAGGAGATATGAGGGACAATTGGAGAAGTGAATTATATAAATATA
AAGTGGTAGAAATTAAACCATTA---GGAATAGCACCCACTGAGGCAAAAAGGAGAGTGGTG-----------
----------------------------GAGAGAGAGAAAAGAGCA---GTGGGA---ATAGGAGCT-------
---GTGTTCCTTGGGTTCTTGGGAGCG---GCAGGAAGCACTATG---GGCGCGGCGTCA---
ATAACGCTGACGGTACAG---GCCAGACAA---TTATTG---TCTGGTATAGTGCAACAGCAAAGC---
AATTTG---CTGAGA---GCTATAGAGGCGCAACAGCATATGTTGCAACTCACGGTCTGGGGCATT

>KC156214
TTCTATTGCAATACATCAAAAATATTTGATAGTGACAATGCAAACAAA-------------------------
----

ACTTCAGGAAATATCACACTCCCATGCAAAATAAAACAAATCATAAACATGTGGCAGGGGGTAGGAAGAGCAA
TGTATGCCCCTCCCATTGCAGGAAACATAACATGTACATCAAAT---
ATCACAGGACTACTATTGACACGTGATGGAGGCAATGTTACAAAT----------------------------
----

GACACAGAGATATTCAGACCTGGAGGAGGAAATATGAGGGACAATTGGAGAAGTGAATTATACAAATATAAAG
TGGTAGAAATTAAGCCATTA---GGGATAGCACCCACCAAGGCAAAAAGGCGAGTGGTG--------------
-------------------------GAGAGAGAAAAAAGAGCA---GTGGGA---ATAGGAGCT---------
GTATTCCTTGGGTTCTTGGGAGCA---GCAGGAAGCACTATG---GGCGCAGCGTCA---
ATAGCGCTGACGGTACAG---GCCAGACAA---TTGTTG---TCTGGTATAGTGCAACAGCAAAAC---
AATTTG---CTGAGA---GCTATAGAGGCGCAACAGCATATGTTGCAACTCACGGTCTGGGGCATT
>KP109523
TTCTACTGCAATACATCAAGTCTGTTTAATAGTAATAGT----------------------------------
----

ACAGAAGATCCCATCACACTCAAATGCAGAATAAAACAAATCATAAACATGTGGCAGGAGGTAGGAAGAGCAA
TGTATGCCCCTCCCATTGCAGGAGAAATCACGTGTAACTCAAGT---
ATCACAGGACTATTACTGCTACGTGATGGAGGTAACTCAAATGAC----------------------------
----

TCCAAAGAGAAAGAGACATTCAGACCTGGAGGAGGAAATATGAAGGACAATTGGAGGAGTGAATTATATAAAT
ATAAGGTGGTAGAAATTAAGCCATTA---GGAGTAGCACCCACTGAGGCAAAAAGGAGAGTGGTG--------
-------------------------------GAGAGAGAAAAAAGAGCA---GTGGGA---ATAGGAGCT---
------GTGATCTTTGGGTTCTTGGGAGCA---GCAGGAAGCACTATG---GGCGCGGCGTCA---
ATAACGCTGACGGTACAA---GCCAGACAA---CTGCTG---TCTGGTATAGTGCAACAGCAAAAC---
AATTTG---CTGAGG---GCTATAGAGGCGCAACAGCATATGTTGCAACTCACAGTCTGGGGCATT
>KP109524
TTTTATTGCAATACAACAAACCTGTTTAATGGTATTAATGAGACAGAAAAT----------------------
----

ACCAATTCAACCATCATACTTCGTTGCAGAATAAAACAAATAATAAACATGTGGCAGGGGGTAGGACGAGCAA
TGTATGCCCCTCCCATTGAAGGAAACATAACATGTAGATCAAAT---
ATCACAGGGCTACTATTGACACGTGATGGAGGAGTAAATGCGACA----------------------------
----

AAAGAGACATTCAGACCTGGAGGAGGAGATATGAGGGACAATTGGAGAAGTGAACTATATAAATATAAAGTGG
TAGAAATTAAGCCATTA---GGAGTAGCACCCACTAGTGCAAAAAGGAGAGTGGTA-----------------
-------------------------AGAGAGAAAAGAGCA---GTGGGA---CTAGGAGCT---------
GTGTTCCTTGGGTTCTTGGGAGCA---GCAGGAAGCACTATG---GGCGCGGCATCA---
GTAATGCTGACGGTACAG---GCCAGACAA---TTATTG---TCTGGTATAGTGCAACAGCAAAGC---
AATTTG---CTGAAG---GCCATCGAGGCGCAACAGCATATGTTGCAACTCACAGTCTGGGGCATT
>KP109527
TTCTATTGCGATACATCAGGTCTGTTTAATGGTACATACAACGCTACAGACATGTCTAATAGT----------
----
----------------------------------ACAAACGAAACCATCATA---------
AAACAGATTATAAACATGTGGCAGGGGGTAGGACGAGCAATGTATGCCCCTCCCATTGCAGGAAACATAACAT
GTAAATCCAAT---ATCACAGGACTATTATTGACACGTGATGGAGGTAATGATAAAAAT--------------
----

AACACAGAAATATTCAGACCTGGAGGAGGAGATATGAGGGACAATTGGAGAAGTGAATTATATAAATACAAAG
TGGTAGAAATTAAGCCATTG---GGAGTAGCACCCACTGAGGCAAAAAGGAGAGTGGTG--------------
-------------------------AAGAAAGAAAAAAGAGCA---GTGGGA---CTAGGAGCT---------
ATGTTCCTTGGGTTCTTGACAGCA---GCAGGAAGCACTATG---GGCGCGGCGTCA---
ATAACGCTGACGGTACAG---GCCAGACAA---TTGTTG---TCTGGTATAGTGCAACAGCAAAAC---
AATTTG---CTGAGA---GCTATAGAGGCGCAACAGCATATGTTGCAACTCACGGTCTGGGGCATT
>KC634109

TTCTATTGTAATACATCAGGGCTGTTTAATGGTACATACCCGGCTAATGATACAGAAAGTAAC----------
----

TCAAGCTCACCAATCACACTCCCATGCAGAATAAAACAAATTATAAACATGTGGCAGGAAGTAGGACGAGCAA
TGTATGCTCCTCCCATTGCAGGAAATATAACATGTACATCAAAT---
ATCACGGGATTACTCTTGACACGTGATGGAGGCACAGGAAATGGCACAGAATCA-------------------
----

AATAGCACAGAAACATTCAGACCTGGAGGAGGAGATATGAGGGACAATTGGAGAAGTGAATTATATAAATACA
AAGTGGTAGAAATTAAGCCATTG---GGAATAGCACCCACTACGGCAAAAAGGAGAGTGGTG-----------
----------------------------GAGAGAGAAAAAAGAGCA---GTAGGA---ATAGGAGCT------
---GTGTTCCTTGGGTTCTTGGGAGCA---GCAGGAAGCACTATG---GGCGCGGCGTCA---
ATCACGCTGACGGTACAT---GCCAGACAA---TTGTTG---TCTGGTATAGTGCAACAGCAAAGC---
AATTTG---CTGAAG---GCTATAGAGGCGCAACAACATTTGTTGCAACTCACAGTCTGGGGCATT
>KC187500
TTCTATTGCAATACATCAGAACTGTTTAATAGTACACTGTTTAATAGTACACACATGTCTAGTACAAATGCAA
CT---

AAAAACAAAACCATCACACTTTCATGCAGAATAAAACAAATCATAAACATGTGGCAGGGGGTAGGACGAGCAA
TGTATGCCCCTCCCATTGCAGGAAACATAACATGTAGGTCAAAC---
ATCACAGGACTAATAATAACACGTGATGGGGGAACCGGAGAGAATACAACAGGG-------------------
----

GATAGAACAGAGGAAACATTCAGACCTGCAGGAGGAGATATGAGGGACAATTGGAGAAGTGAATTATATAAAT
ATAAAGTGGTAGAAATTAAGCCATTA---GGAATAGCACCCACTAGAGCAAAAAGGAGAGTGGTG--------
-------------------------------GAGAGAGAAAAAAGAGCA---GTGGGA---ATAGGAGCT---
------GTGTTCCTTGGGTTCTTGGGAGCA---GCAGGAAGCACTATG---GGCGCGGCGTCA---
ATAACGCTGACGGTACAG---GCCAGACAA---TTGCTG---TCTGGTATAGTGCAACAGCAAAGC---
AATTTG---CTGAGG---GCTATAGAGGCGCAACAGCATATGTTGCAACTCACAGTCTGGGGCATT
>KC187630
TTCTATTGCAATACATCAAACCTGTTTAATATGACATACATATGGAAAAATGGGTCATACGTGCTTAATAATA
CAGACAAT---

AATGATACAACAATCACACTCCAATGCAGAATAAAACAATTTATAAACATGTGGCAGAGGGTAGGAAGAGCCA
TGTATGCTCCTCCCATTGCAGGAAACATAACATGTGTATCAAAT---
ATCACAGGATTACTATTGACACATGATGGGATAAAGACAAATAGATCAAGTGAC-------------------
----

TCAAATTGGACATGGACATTCAGACCTGGGGGAGGAAATATGAAGGACAATTGGAGTAATGAATTATACAAAT
ATAAAGTGGTAGAAATTAAACCATTG---GGAATAGCACCCACTAAGGCAAAAAGGAGAGTGGTG--------
-------------------------------GAGAGAGAAAAAAGAGCA---GTGGGA---ATAGGAGCT---
------GTGATCTTTGGGTTCTTGGGAGCA---GCAGGAAGCACTATG---GGCGCAGCGTCA---
ATAACGCTGACGGTACAG---GCCAGACAA---TTGCTG---TCTGGTATAGTGCAACAGCAAAGC---
AATTTG---CTGAAA---GCTATAGAGGCGCAACAACATATGTTGCAACTCACGGTCTGGGGCATT
>KC186203
TTCTATTGCAATACATCAGGACTATTTAATAGTACATTTAATAAGGCATACCAGCCTAATAGTACAGAAGAT-
----

GACTCAAAAAACATCACACTCCAATGCAGAATAAAGCAAATTATAAACATGTGGCAGGGGGTAGGACAAGCAA
TGTATGCTCCTCCCATAGCAGGAAACATAACATGTAGATCAAAT---
ATAACAGGACTGCTATTAACACGTGATGGAGGAGATAACAATAGCACA-------------------------
----

AATGGCACAGAGATATTCAGACCTGGAGGAGGAGATATGAAGGACAATTGGAGAAGTGAATTATATAAATATA
AAGTGGTAGAAGTTATGCCATTG---GGATTAGCACCCACTAAGGCCAAAAGGAGAGTGGTGGAGAGA-----
-------GAGGCCAAAACGAGAGTGGTGGAGAGAAAGAAAAGAGCA---GTGGGA---ATAGGAGCT------
---GTGTTCCTTGGGTTCTTAGGAGCA---GCAGGAAGCACTATG---GGCGCAGCGTCA---
GTGACGCTGACGGTACAG---GCTAGACAA---TTATTG---TCTGGTATAGTGCAACAGCAAAGC---
AATCTG---CTGAAG---GCTATAGAGGCGCAACAGCATATGTTGCAACTCACAGTCTGGGGCATT
>KC186594

TTCTATTGCAATACATCAGGCCTGTTCAATGGCACATACAATGGCACATACAATGGCACATACAATGGCATAT
ACAAT--

AGTACAGACAATATCACACTCCCATGTAGAATAAAACAAATTATAAACATGTGGCAGAGGGTAGGACGAGCAA
TATATGCCCCTCCCATTGCAGGAAATATAACATGTAAATCAAAT---
ATCACAGGACTATTATTGACACGTGATGGAGGAAAAAATAATACA----------------------------
----

ACAGATGAAGTATTCAGACCTGGAGGAGGAGATATGAGGGACAATTGGAGAAGTGAATTATATAAATATAAAG
TGGTAGAAATTAAGCCATTA---GGAATAGCACCCACTAATGCAAAAAGGAGAGTGGTG--------------
-------------------------GAGAAAGAAAAAAGAGCA---GTGGGA---CTGGGAGCT---------
GTGTTCCTTGGGTTCTTGGGAGCA---GCAGGAAGCACTATG---GGCGCGGCGTCG---
ATAACGCTGACGGTACAG---GCCAGACAA---TTGTTG---TCTGGTATAGTGCAACAGCAAAGC---
AATTTG---CTGAGG---GCTATAGAGGCGCAACAGCATATGTTGCAACTCACGGTCTGGGGCATT
>KC186169
TTCTATTGCAATACATCAAAACTGTTTAATACTACAGGCAATAGTACA-------------------------
----

GCAAATACAACCATCACACTCCCATGCCAAATAAAACAAATTATAAACATGTGGCAGGGGGTAGGACGAGCGA
TGTATGCCCCTCCCATTGCAGGAAACATAACATGTGAATCAAAT---
ATCACAGGGCTACTATTGACACGGGATGGAGGAAAAGGTAAAGAT----------------------------
----

AATGGAACAGAGACATTCAGACCTGAAGGAGGAGATATGAGGGACAATTGGAGGAGTGAATTATATAAATATA
AAGTGGTAGAAATTAGGCCATTG---GGAATAGCACCCACTACGGCAAAAAGGAGAGTGGTG-----------
----------------------------GAGAGAGAAAAAAGAGCA---GTGGGA---CTAGGAGCT------
---GTGTTCCTTGGGTTCTTAGGAGCA---GCAGGAAGCACTATG---GGCGCAGCGTCA---
ATAACGCTGACGGTACAG---GCCAGACAA---CTGTTG---TCTGGTATAGTGCAACAGCAAAGC---
AATTTG---CTGAGA---GCTATAGAGGCGCAACAGCATATGTTGCAACTCACGGTCTGGGGCATT
>KC186446
TTCTATTGCAATACAACAGACCTGTTTAATGGGACAGGAAATGGTACAAAG----------------------
----

GCAAATGACACAATCAAAATCCCATGCAAAATAAAACAAATTATAAACATGTGGCAGGGAGTAGGAAGAGCAA
TATATGCTCCTCCCATTAAAGGAAACATAACATGTAAATCAAAT---
ATCACAGGAATACTATTGACACGTGATGGAGGAAGAGATGGAGGAGAAGAT----------------------
----

AACGAGACAGAGATATTCAGACCTGGAGGAGGAGATATGAGGGACAATTGGAGGAGTGAATTATATAAATATA
AAGTGGTAGAAATCAAGCCATTG---GGAATAGCACCCACTGCGGCAAAACGGAGAGTGGTG-----------
----------------------------GAGAAAGAAAAAAGAGCA---GTGGGA---ATAGGAGCT------
---GTGTTCCTTGGGTTCTTGGGAGCA---GCAGGAAGCACTATG---GGCGCGGCATCA---
ATAACGCTGACGGTACAG---GCCAGACAA---TTATTG---TTTGGTATAGTGCAACAGCAAAGC---
AATTTG---CTGAGA---GCTATAGAGGCGCAGCAGCATATGTTGCAACTCACAGTCTGGGGCATT
>KT896551
TTCTATTGTAATACATCAAAACTGTTTAATGGTGCATATAATAATTCAAATTCAAGTACAGAAGATACT----
----

TCAAACTCAACAATCACACTCCCATGCAAAATAAAACAAATTATAAACATGTGGCAGGAGGTAGGACGAGCAA
TGTATGCTCCTCCCATTAGAGGAAACATAACATGTGAATCGAAT---
ATCACAGGATTACTATTGGTGCGTGATGGAGGAAATACTACGAATAATGAGACA-------------------
----

GAGACAGAGACATTCAGACCTGGAGGAGGAGACATGAGGGACAATTGGAGAAGTGAATTATACAAGTATAAAG
TGGTAGAAATTAAGCCATTG---GGAATAGCACCCACTAAGGCAAGAAGAATAGTGGTG--------------
-------------------------GAGAGAGAAAAAAGAGCA---GTAGGA---CTAGGAGCT---------
GTGATCTTTGGGTTCTTGGGAGCA---GCAGGAAGCACTATG---GGCGCGGCGTCA---
ATAGCGCTGACGGTACAG---GCCAGGCAA---GTGTTG---TCTGGTATAGTGCAACAGCAAAGC---
AATTTG---CTGAAA---GCCATAGAGGCGCAACAGCATCTGTTGCAACTCACAGTCTGGGGCATT
>MG898649

TTCTACTGCAATACATCAGGCCTATTTAATAGGACATACGATGCAAATGAAACAGCAAAGTATGGTAACAGT-
----

ACAAATGGTAACATCACACTCCAATGCAGAATAAAACAATTTATAAACATGTGGCAGAGGGTAGGACGAGCAA
TGTATGCCCCTCCCATTGCAGGAATCATAACATGTAGATCAAAT---
ATCACAGGACTACTATTGACACGAGATGGAGGAGATGGAGGAGAAAATGGAACA-------------------
----

GAGACAGAGACATTCAGACCTGGAGGAGGAGATATGAGGGACAATTGGAGGAGTGAATTATATAAATATAAAG
TGGTAGAAATTAAGCCATTA---GGAATAGCACCCACCGAGGCAAAAAGGAGAGTGGTG--------------
-------------------------GAGAGAGAAAAAAGAGCA---GTGGGG---CTAGGAGCT---------
GTGCTCCTTGGGTTCTTGGGAGCA---GCAGGAAGCACTATG---GGCGCGGCGTCA---
ATAACGCTGACGGTACAG---GCCAGACAA---TTGTTG---TCTGGTATAGTGCAACAGCAAAGC---
AATTTG---CTGAAG---GCTATAGAGGCGCAACACCATCTGTTGCAACTCACGGTCTGGGGCATT
>MG899863
TTTTATTGCAATACATCACAACTGTTTAATAGGACATACAACCCTACAGAAAATAGTACAGAA----------
----

AGTAATAAAACCATCACAATCCCATGCAGAATAAAACAGATTATAAATATGTGGCAGAAAGTAGGAAAAGCAA
TGTATGCCCCTCCCATTGCAGGATACATAACATGTAGATCAAAC---
ATCACAGGACTACTATTGTCACGTGATGGAGGCAACGATAACACAACAGAA----------------------
----

AGAACAGAAACCTTCAGACCTGCAGGAGGAGATATGAGGGACAATTGGAGAAGTGAATTATATAAATATAAAG
TGGTAGAAATTAAGCCATTG---GGAATAGCACCCACCGAGGCAAAAAGGAGAGTGGTG--------------
-------------------------GAGAGACAAAAAAGAGCA---GTGGGA---ATAGGAGCT---------
GTGCTCCTTGGGTTCTTGGTAGCA---GCAGGAAGCACTATG---GGCGCGGCGTCA---
ATAACGCTGACGGTACAG---GCCAGACAA---CTGTTG---TCTGGTATAGTGCAACAGCAAAGC---
AATTTG---CTGAGA---GCTATAGAGGCGCAACAGCATATGTTGCAACTCACGGTCTGGGGCATT
>AY713413
TTCTATTGTAATACATCAAACCTGTTTACATCAAACCTGTTTACTAACAATCTAACA----------------
----

AATACTACAAACATCACACTCCCATGCAGAATAAAACAAATTATAAACATGTGGCAGGGGGTAGGACGAGCAA
TGTATGCCCCTCCCATTGCAGGAAACATAACATGTAAATCGAAT---
ATCACAGGACTACTACTGACACGTGATGGAGGAGAAAATAATAGG----------------------------
----

ACTGAGACATTCAGACCTGGAGGAGGAGATATGAAGGACAATTGGAGAAGTGAATTATATAAATATAAGGTGG
TAGAAATTAAGCCATTA---GGAGTAGCACCCACTGGTGCAAAAAGGAGAGTGGTG-----------------
----------------------GAGAGAGAGAAAAGAGCA---GTGGGA---ATAGGAGCT---------
GTGTTCCTTGGGTTCTTAGGAGCA---GCAGGAAGCACTATG---GGCGCGGCGTCA---
ATAACGCTGACGGTACAG---GCCAGACAA---TTATTG---TCTGGTATAGTGCAACAGCAAAGC---
AATTTG---CTGAGG---GCTATAGAGGCGCAACAGCATATGTTGCAACTCACGGTCTGGGGCATT
>HM638662
TTCTATTGTAATACATCAAAACTGTTTAATAGTACATACATGCATAATGGTACAACAGGTAAC----------
----

TCAAGCGGAGTCATCACACTCCCATGCAAAATAAAACAAATTATAAACATGTGGCAGGAGGTAGGACGAGCAA
TGTATGCTCCTCCCATTGCAGGAAACATAACATGCAAATCAAAT---
ATCACAGGACTACTATTGACACGTGATGGAGGGTCTGACCCAAACACA-------------------------
----

AATAATGACACAGAGACATTCAGACCTGGAGGAGGAGATATGAGGGACAATTGGAGAAGTGAATTATATAAAT
ATAAAGTGGTAGAAATTAAGCCATTA---GGAGTAGCACCCACTGGAGCAAAAAGGAGAGTGGTG--------
-------------------------------GAGAGAGAAAAAAGAGCA---GTTGGA---ATAGGAGCT---
------GTGCTCCTTGGGTTCTTGGGAACA---GCAGGAAGCACTATG---GGCGCGGCGTCA---
ATAACGCTGACGGTACAG---GCCAGACAA---GTATTG---TCTGGTATAGTGCAACAGCAAAGC---
AATTTG---CTGAGG---GCTATAGAGGCGCAACAGCATATGTTGCAACTCACGGTCTGGGGCATT
>HM638713

TTCTATTGCAATACATCAAACCTGTTTAATAGTACATACATGACAAACATGACTAATAGTACTTCAGATTCTA
AT---

AGTACTTCAAACATTACACTCCAATGCAGAATAAAACAAATTGTAAACATGTGGCAGGAGGTAGGACGAGCAA
TGTATGCCCCTCCCATTGCAGGAAACATAACATGTGAATCAAAT---
ATCACAGGACTACTATTGACACGTGATGGAGGAACAACTAATAACACAGGAACA------------AAT----
----
GGAACAGAAAATAAT---
AACACAGAAACATTCAGACCTGGAGGAGGAGATATGAGGGACAATTGGAGAAATGAATTATACAAATATAAAG
TAGTAGAAATTAAGCCATTG---GGAATAGCACCCACTAAGGCAAAAAGGAGAGTGGTG--------------
-------------------------GAGAGAGAAAAAAGAGCA---GTGGGA---CTAGGAGCT---------
GCGTTCCTTGGGTTCTTGGGAGCA---GCCGGAAGCACTATG---GGCGCAGCATCA---
ATAACGCTGACGGTACAG---GCCAGACAA---TTGTTG---TCTGGTATAGTGCAACAGCAAAGC---
AATTTG---CTGAGG---GCTATAGAGGCGCAACAGCATATGTTGCAACTCACGGTCTGGGGCATT
>HM638770
TTCTATTGCAATACATCAAAACTGTTTAATGGGACATACAATGCGACAGACATGCCTAATGGGACATACAATT
CT---

AATAGTACAAAAGTCATCACACTCCCATGCAGAATAAAGCAAATTATAAACATGTGGCAGGAGGTAGGACGAG
CAATGTATGCAAATCCCATTGAAGGAAACATAACATGTAAATCAAAT---
ATCACAGGAATACTATTGGTACGTGATGGAGGCAAAGAGGACAATAAA-------------------------
----

ACAGACCCAAAAGAGACATTCAGACCTGGAGGAGGAGATATGAGGGACAATTGGAGAAGTGAATTATATAAAT
ATAAAGTGGTAGAAATTAAACCATTA---GGAATAGCACCCACTAAGGCAAAAAGGAGAGTGGTG--------
-------------------------------GAGAGAGAAAAAAGAGCA---GTGGGA---ATAGGAGCT---
------GTGTTCCTTGGGTTCTTGGGAGCA---GCAGGAAGCACTATG---GGCGCGGCGTCA---
ATAACGCTGACGGTACAG---GCCAGACAA---CTGTTG---TCTGGTATAGTGCAACAGCAGAGT---
AATTTG---CTGAGG---GCTATAGAGGCGCAACAGCATATGTTGCAACTCACGGTCTGGGGCATT
>HM638848
TTCTATTGCAATACATCAGGACTGTTTAATAGTACATACAATAGTACATACATGCCTAATGGTACAGAG----
----

AGTAATTCAACAATCACACTCCCATGCAGAATAAAACAAATTATAAACATGTGGCAGGAGGTAGGACGAGCAG
TGTATGCTCCTCCCATTGAAGGACACATAACATGTAACTCAACA---
ATCACAGGATTACTATTGACACGCGATGGAGGCAATAACACAAAT----------------------------
----

GGAACTGAGACATTCAGACCTGGAGGAGGAGATATGAGGGACAATTGGAGAAATGAATTATACAAATATAAAG
TAGTAGAAATTAAGCCATTA---GGAATAGCACCCACTGGAGCAAAAAGGAGAGTGGTG--------------
-------------------------GAGAGAGAAAAAAGAGCA---GTGGGA---ATAGGAGCC---------
TTGTTCCTTGGGTTCTTAGGAGCA---GCAGGAAGCACTATG---GGCGCAGCGTCA---
ATAACGCTGACGGTACAG---GCCAGACAA---CTGCTG---TCTGGTATAGTGCAACAGCAAAGC---
AATTTG---CTGAGA---GCTATAGAGGCGCAACAGCATATGTTGCAACTCACGGTCTGGGGCATT
>HM638911
TTCTATTGCAATACATCAGGCCTGTTTAATAGTACATACAATATAACAAATTCTTCAGATTCAGAAAATTCT-
----

TCAAATTCAACCATCACACTCCAATGCAGAATAAAACAAATTATAAACATGTGGCAGGAGGTAGGACGAGCAA
TGTATGCTCCTCCCATTGAAGGAAGCATAACCTGTAACTCAAGT---
ATCACAGGATTACTATTGACCCGTGATGGAGGCACATCTCCAGGAGAT-------------------------
----

GCTAATAACACAGAGACATTCAGACCTGGAGGAGGAGATATGAGGAACAATTGGAGAAGTGAATTATATAAAT
ATAAAGTGGTAGAAATTAAGCCATTA---GGAATAGCACCCACTAAGGCAAAAAGGAGAGTGGTG--------
-------------------------------GAGAGAGAGAAAAGAGCA---GTGGGA---ATAGGAGCT---
------GTACTCCTTGGGTTCTTGGGAGCA---GCAGGAAGCACTATG---GGCGCGGCGTCA---
ATAACGCTGACGGTACAG---GCCAGACAA---TTGTTG---TCTGGTATAGTGCAACAGCAAAGC---
AATTTG---CTGAGA---GCTATAGAGGCGCAACAGCATATGTTGCAACTCACGGTCTGGGGCATT
>KC894126

TTCTATTGCAATACATCAAAACTGTTTAATGGCACATACATGTCTAATAGTACGTCTAATAGTACAGGA----
----

AACATCACAATCATCACAATCCCATGCAGAATAAAACAAATTATAAACATGTGGCAGGAGGTAGGACGAGCAA
TGTATGCCCCTCCCATTGCAGGAAACATAACATGTAAATCAAAT---
ATCACAGGACTACTATTGACTCGTGATGGAGGAGTCGATAATGGAAATGGA----------------------
----

ACAGAGGAGATATTCAGACCTGGAGGAGGAGATATGAGGGACAATTGGAGAAGTGAATTATATAAATATAAAG
TGGTAGAAATTAAGCCATTA---GGAATAGCACCCACTACTGCAAAAAGGAGAGTGGTG--------------
-------------------------GAGAGAGAGAAAAGAGCA---GTGGGA---ATAGGAGCT---------
GTGTTCCTTGGGTTCTTGGGAGCA---GCAGGAAGCACTATG---GGCGCAGCGTCA---
ATAACGCTGACGGTACAG---GCCAGACAA---TTATTG---TCTGGTATAGTGCAACAGCAAAGC---
AATTTG---CTGAGG---GCTATAGAGGCGCAACAGCATATGTTGCAACTCACGGTCTGGGGCATT
>HM638986
TTCTATTGTAATACATCAAACCTATTTAATAGTACATACATGATTAATGGTACATACATGGCTAATGGTACAG
CAAAT--

ATTCCAAATTCAACCATCACACTCCAATGCAGAATAAAACAAATTGTAAACATGTGGCAAGGGGTAGGACGAG
CAATGTATGCCCCTCCCATTGCAGGAAACATAACATGTGTATCAAAT---
ATCACAGGACTACTATTGACACGTGATGGAGGATATGGAAATAACACC-------------------------
----

AATAACACAGAAGAAATATTCAGACCTGGAGGAGGAAACATGAAGGACAATTGGAGAAGTGAATTATATAAAT
ATAAAGTAGTAGAAATCCAGCCATTA---GGAATAGCACCCACTGAGGCAAAAAGGAGAGTGGTG--------
-------------------------------GAGAGAGAAAAAAGAGCA---GTGGGA---CTAGGAGCT---
------GTGTTCCTTGGGTTCTTGGGAGCA---GCAGGAAGCACTATG---GGCGCAGCATCA---
ATAACGCTGACGGTACAG---GCCAGACAA---TTGTTG---TCTGGTATAGTGCAACAGCAAAGC---
AATTTG---CTGAGG---GCTATAGAGGCGCAACAGCATATGTTGCAACTCACGGTCTGGGGCATT
>KC894128
TTCTATTGTAATACATCAAACCTATTTAATAGTACATATAGTACAAACATGACTAATGATACAGAAAATACT-
----

TTAAATTCAACCATCATACTCCAATGCAGAATAAAACAAATTGTAAACATGTGGCAAGGAGTAGGACGAGCAA
TGTATGCCCCTCCCATTGCAGGAAGCATAACATGTATATCAAAT---
ATCACAGGACTACTATTGACACGTGATGGAGGAAATGGAAATAACACCAAT----------------------
----

AACACAGAAGAAATATTCAGGCCTGGAGGAGGAAACATGAAGGACAATTGGAGAAGTGAATTATATAAATATA
AAGTAGTAGAAATTAAGCCATTA---GGAATAGCACCCACTGAGGCAAAAAGGAGAGTGGTG-----------
----------------------------GAGAGAGAAAAAAGAGCA---GTGGGA---CTAGGAGCT------
---GTGTTCCTTGGGTTCTTGGGAGCA---GCAGGAAGCACTATG---GGCGCAGCATCA---
ATAACGCTGACGGTACAG---GCCAGACAA---TTGTTG---TCTGGTATAGTGCAACAGCAAAGC---
AATTTG---CTGAGG---GCTATAGAGGCGCAACAGCACATGTTGCAACTCACGGTCTGGGGCATT
>KC863127
TTCTATTGCAATACATCAAACTTGTTTAATAAAACAAACCTGTTTAATAGTACAGATAATAGTACAGAAGGGG
AAAAAGGAGTT--

TCAAATGCAACCATCACACTCCAATGCAGAATAAAACAAATTATAAATATGTGGCAGGGGGTAGGACGAGCAA
TGTATGCCCCTCCCATTGCAGGAAACATAACATGTAAATCAAAT---
ATTACAGGACTACTGTTAACACGGGATGGAGGAGATGATAATAAA----------------------------
----

ACAGAGACATTCAGACCTGGAGGAGGAGATATGAGGGACAATTGGAGAAGTGAATTATATAAATATAAAGTGG
TAGAAATTAAGCCATTA---GGAGTAGCACCCACTAAGGCAAAAAGGAGAGTGGTG-----------------
----------------------GAGAGAGAAAAAAGAGCA---GTGGGA---ATAGGAGCT---------
GTGTTCCTTGGGTTCTTGGGAGCA---GCAGGAAGCACTATG---GGCGCAGCATCA---
ATAACGCTGACGGTACAG---GCCAGACAA---TTGTTG---TCTGGTATAGTGCAACAGCAAAGC---
AATTTG---CTGAGG---GCTATAGAGGCGCAACAGCATATGTTGCAACTCACGGTCTGGGGCATT
>KC894129

TTCTATTGCAATACATCAGGCCTGTTTAATGGTACCCTTAATGGTACATATATGCCTAATAATACAGGCAGTA
AT---

TCAAGTTCAACCATCACACTCCAATGCAGGATAAAACAAATTATAAACATGTGGCAGGAGGTAGGACGAGCAA
TGTATGCCCCTCCCATTGCAGGAAACATAACATGTAGATCAAAT---
ATCACAGGACTACTATTAACACGCGATGGAGGAAATACTACTAAT----------------------------
----

AATACAGAAATATTCAGGCCTGGAGGAGGAAATATGAAGGACAATTGGAGAAGTGAATTATATAAATATAAAG
TGGTAGAAATTAAGCCATTA---GGAATAGCACCCACTGAGGCAAAAAGGAGAGTGGTG--------------
-------------------------GAGAGAGAGAAAAGAGCA---GTGGGA---ATAGGAGCT---------
GTGTTCCTTGGGTTCTTGGGAACA---GCAGGAAGCACTATG---GGCGCGGCGTCA---
ATAACGCTGACGGTACAG---GCCAGACAA---TTGTTG---TCTGGTATAGTGCAACAGCAAAGC---
AATTTG---CTGAGG---GCTATAGAGGCGCAACAGCATATGTTGCAACTCACGGTCTGGGGCATT
>HM639015
TTCTATTGCAATACATCAGGACTGTTTAATAGTATATACACATTTAATAGTACATACAATAGTAATAGTACAT
ACAATAGTAAT--
TCAAGTTCATACAATAGT---------
AATTCAAGTTCAATTATCACACTCCAATGCAGAATCAAACAAATTATAAACATGTGGCAGGGGGTAGGGCGAG
CAATGTATGCTCCTCCCATTGCAGGAAACATAACATGTATATCAAAT---
ATCACAGGACTACTATTGGTGCGTGATGGAGGCAATGTGACAGAC----------------------------
----

ACAGAGATATTCAGACCTGGAGGAGGAGATATGAAGGACAATTGGAGAAGTGAATTATATAAATATAAAGTGG
TAGAAATTAAGCCATTA---GGAATAGCACCCACTGGGGCAAAAAGGAGAGTGGTG-----------------
----------------------AAGATAGAAAAAAGAGCA---GTGGGA---ATAGGAGCT---------
GTGCTCCTTGGGTTCTTGGGAGCA---GCAGGAAGCACTATG---GGCGCGGCGTCC---
ATAACGCTGACGGTACAG---GCCAGACAA---TTGTTG---TCTGGTATAGTGCAACAGCAAAGC---
AATTTG---CTGAGG---GCTATAGAGGCGCAACAACATATGTTGCAACTCACGGTCTGGGGCATT
>HM639196
TTCTATTGCAATACATCAAGACTGTTTAATAGGACATACAATTTTAATGGTACAGAGAATCCTAATGGTACAG
AG---

AGTAATTCAAGCTTCATCGAACTCCCATGCAGAATAAAACAAATTATAAACATGTGGCAGGAGGTAGGACGAG
CAATGTATGCCCCTCCCATTGAAGGAAACATAACATGTAAATCAAAT---
ATCACAGGACTACTATTGCTACGTGATGGAGGAACAGGAAGTGAGGTA-------------------------
----

CCAAATGACACAGAGATATTCAGACCTGGAGGAGGAAATATGAGGGACAACTGGAGAAGTGAATTATATAAAT
ATAAAGTGGTAGAAATTAAACCATTG---GGAGTAGCACCCACTAAGGCAAAAAGGAGAGTGGTG--------
-------------------------------GAGAGAGAAAAAAGAGCA---GTG------GGAGCTGCT---
------ATGTTCTTTGGGTTCTTGGGAGCA---GCAGGAAGCACTATG---GGCGCGGCGTCA---
ATAACGCTGACGGTACAG---GCCAGACAA---CTGCTG---TCTGGTATAGTGCAACAGCAAAGT---
AATTTG---CTGAGG---GCTATAGAGGCGCAACAGCATATGTTGCAACTCACGGTCTGGGGCATT
>KC894132
TTCTATTGCAATACATCAGGACTGTTTAATAGTACATACAATCCTGATCTTAATAATGCAAAGAATGAT----
----

TCAAATGCAACCATCACGCTCCCATGCAGAATAAAACAAATTATAAACATGTGGCAGGAGGTAGGACGAGCAA
TGTATGCCCCTCCCATTGCAGGAAACATAACATGTAGATCAAAT---
ATCACAGGACTACTGTTGCTACGTGATGGAGGAAAAGATCCAAAC----------------------------
----

AACACAGAGATATTCAGGCCTGGAGGAGGAAATATGAGGGACAACTGGAGAAGTGAATTATATAAATATAAAG
TGGTAGAAATTAAGCCATTG---GGAGTAGCACCCACTAAGGCAAAAAGGAGAGTGGTG--------------
-------------------------GAGAGAGAAAAAAGAGCA---GTAGGA------GCTGCT---------
ATGTTCTTTGGGTTCTTGGGAGCA---GCAGGAAGCACTATG---GGCGCGGCGTCA---
ATAACGCTGACGGTACAG---GCCAGACAA---CTGCTG---TCTGGTATAGTGCAACAGCAAAGT---
AATTTG---CTGAGA---GCTATAGAGGCGCAACAGCATATGTTGCAACTCACAGTCTGGGGCATT
>HM215361

```
TTCTATTGCAATACATCAGGCCTGTTTAATAATAATCTTACAGAAAATAAT----------------------
-------------------------------------------------------------------------
----------------------------
CTTACAGGCATCATCACACTCCCATGCAGAATAAAACAAATTATAAACATGTGGCAGGGGGTAGGAAGAGCAA
TATATGCCCCTCCCATTGCAGGAAACATAACATGTAAATCAAAT---
ATCACAGGACTACTATTGACACGTGATGGAGGCGATAACACAACT----------------------------
-------------------------------------------------------------------------
-------------------
GAGACATTCAGACCTGGAGGAGGAGATATGAGGGACAATTGGAGAAGTGAATTATATAAATATAAGGTGGTAG
AAATTAAGCCATTA---GGAATAGCACCCACTGAGGCAAAAAGAAGAGTGGCG--------------------
-------------------GAGAGAGAAAGAAGAGCA---GTGGGA---ATAGGAGCT---------
GTGTTCCTTGGGTTCTTGGGAGCA---GCAGGAAGCACTATG---GGCGCGGCGTCA---
ATAACGCTGACGGTACAG---GCCAGACAA---CTGTTG---TCTGGTATAGTGCAACAGCAAGAC---
AATTTG---CTGAGG---GCTATAGAGGCGCAACAGCATCTGTTGCAACTCACGGTCTGGGGCATT
>HM215362
TTCTATTGCAATACATCAGGACTGTTTAATAGTACATACAATGATACATACATTCCTAATGGTACAGAAATG
GA-----------------------------------------------------------------------
----------------------------
AATGATTCAATCATCAGACTCCCATGCAGAATAAAACAAATTATAAACATGTGGCAGGGGGTAGGACGAGCAA
TGTATGCCCCTCCCATTGCAGGAAACATAACATGTAGATCAAAT---
ATCACAGGAATACTATTGACACGTGATGGAGGAGAAAATAATGGT----------------------------
-------------------------------------------------------------------------
----------------
ACAGAGATATTCAGACCTGGAGGAGGAAATATGAAGGACAATTGGAGAAGTGAGTTATATAAATATAAAGTGG
TAGAAATCAAGCCATTG---GGAATAGCACCCACTGGGGCAAAAAGGAGAGTGGTG-----------------
----------------------GAGAGAGAAAAAAGAGCA---GTGGGA---ATAGGAGCT---------
GTGTTCCTTGGGTTCTTGGGAGCA---GCAGGAAGCACTATG---GGCGCGGCGTCA---
ATAACGCTGACGGTACAG---GCCAGACAA---CTGTTG---TCTGGTATAGTGCAACAGCAAAGC---
AATTTG---CTGAGA---GCTATAGAGGCGCAACAGCATATGTTGCAACTCACGGTCTGGGGCATT
>KU341731
TTCTATTGTAATACATCAGGACTGTTTAATAGTACATACATGCCTACATACATGCCTAATGGTACAGAAAGTA
ATGAT--------------------------------------------------------------------
----------------------------
TCAAACACAACTATCATAATCCCATGCAGAATAAAGCAAATTGTAAACATGTGGCAGGAGGTAGGACGAGCAA
TGTATGCCCCTCCCATTGCAGGGAACATAACATGTACATCAAAT---
ATCACAGGATTACTATTGGTACGTGATGGAGGAATAAAGGAAAAT----------------------------
-------------------------------------------------------------------------
-
GATACAGAGAATAAGACAGAGACATTCAGACCTGAAGGAGGAAATATGAGGGACAATTGGAGAAGTGAATTAT
ACAAATATAAAGTAGTAGAAATCAAGCCATTG---GGAATAGCACCCACTACAGCAAAAAGGAGAGTGGTG--
-------------------------------------GAGAGAGAAAAAAGAGCA---GTGGGA---
TTAGGAGCT---------GTGTTCCTTGGGTTCTTGGGAGCA---GCCGGAAGCACTATG---
GGCGCAGCGTCA---ATAACGCTGACGGTACAG---GCCAGACAA---TTGTTG---
TCTGGTATAGTGCAACAGCAAAGC---AATTTG---CTGAGA---
GCTATTGAGGCACAACAGCATCTGTTGAAACTCACGGTCTGGGGCATT
>KU341730
TTCTATTGTAATACATCAGGCCTGTTTAATGGTACATACATGCCTACATACATGCCTAATGGTACAGAAAGTA
AT-----------------------------------------------------------------------
----------------------------
TCAAACTCCACCATCACAATCCCATGCCGCATAAAACAAATTATAAACATGTGGCAAGAGGTAGGACGAGCAA
TGTATGCCCCTCCCATTGCAGGAAACATCACATGTACATCAAAT---
ATTACAGGACTATTATTGGTACGTGATGGAGGAATAAAGGAAAAT----------------------------
-------------------------------------------------------------------------
-
GATACAGAGAATAAGACAGAGATATTCAGACCTGAAGGAGGAGATATGAGGGACAATTGGAGAAGTGAATTAT
ATAAATACAAAGTAGTAGAAATTAAGCCATTG---GGAGTAGCACCCACTGAAGCGAAAAGGAGAGTGGTG--
-------------------------------------GAGAGAGAAAAAAGAGCA---GTGGGA---
ATAGGAGCC---------GTGTTCCTTGGGTTCTTGGGAGCA---GCAGGAAGCACTATG---
GGCGCGGCGTCA---ATGACGCTGACGGTACAG---GCCAGACAA---TTATTG---
```

TCTGGTATAGTGCAACAGCAAAGC---AATTTG---CTGAGA---
GCTATAGAGGCGCAACAGCATCTGTTGCAACTCACGGTCTGGGGCATT
>KU341726
TTCTATTGCAACACATCAGGACTGTTTAATGGTACATACATGCCTACATACATGCCTAATGGTACAGAAAGTA
AT---

TCAAACTCAACTATCACAATCCCATGCAGAATAAAACAATTTGTAAACATGTGGCAGAAGGTAGGACGAGCAA
TGTATGCCCCTCCCATTGAAGGAAACATAACATGTAGATCAAAC---
ATCACAGGAATACTCTTGGTACGTGATGGAGGAATAAAGGAAAAT----------------------------
----
-
GATACAGAGAATAAGACAGAAATATTCAGACCTGAAGGAGGAGATATGAGGGATAATTGGAGAAGTGAATTAT
ATAAATATAAAGTGGTAGAAATTAAGCCATTG---GGAGTAGCACCCACTGGAGCAAGAAGGAGAGTGGTG--
-------------------------------------GAGAGAGAAAAAAGAGCA---ATAGGA---
ATGGGAGCT---------GTGTTCCTTGGGTTCTTGGGAGCA---GCAGGAAGCACTATG---
GGCGCGGCGTCA---ATAACGCTGACGGTACAG---GCCAGACAA---CTGTTG---
TCTGGTATAGTGCAACAGCAAAGC---AATTTG---CTGAGG---
GCTATAGAGGCGCAACAACATATGTTGCAGCTCACGGTTTGGGGCATT
>KU341725
TTCTATTGTAATACAGCAGGCATGTTTAATAGGACATACATGCCTACATACATGCCTAATGGTACAGAAAGTA
AT---

TCAAACTCAACTATCACAATCCCATGCAGAATAAAGCAAATTATTAATATGTGGCAGGAGATAGGAAGGGCAA
TGTATGCCCCTCCCATTGCAGGAGAAATAACATGTAAATCAAAC---
ATTACAGGAATACTATTGGTACGTGATGGAGGAATACAGGCAAAT----------------------------
----
-
GATACAGAGAATAAGACAGAGATATTCAGACCTGGAGGAGGAGATATGAGAGATAATTGGAGAAGTGAATTAT
ATAAATATAAAGTGGTAGAAATTAAGCCATTG---GGAATAGCACCCACTGCAGCAAAAAGGAGAGTGGTG--
-------------------------------------GAGAGAGAAAAAAGAGCA---GTGGGA---
CTAGGAGCT---------GTGTTCCTTGGGTTCTTAGGAGCA---GCAGGAAGCACTATG---
GGCGCGGCGTCA---ATAGCGCTGACGGTACAG---GCCAGACAA---TTGTTG---
TCTGGTATAGTGCAACAGCAAAGT---AATTTG---CTGAGG---
GCTATAGAGGCGCAACAGCATATGTTGCAACTCACGGTCTGGGGCATT
>KU341723
TTCTATTGTAATACATCAGGCCTGTTTAATGGTACATACATGCCTACATACATGCCTAATGGTACAGAAAGTA
AT---

TCAAACTCAACTATCACAATTCCATGTAGAATAAAGCAAATTATAAACATGTGGCAGGAGGTAGGACGAGCAA
TGTATGCCCCTCCCATTGCAGGAGAAATAACATGTATATCAAAT---
ATCACAGGATTGCTATTGGTACATGATGGAGGAATAAAGGAAAAT----------------------------
----
-
GATACAGAGAATAAGCCAGAGATATTCAGACCTGGAGGAGGAGATATGAGGGACAATTGGAGAAGTGAATTAT
ACAAATATAAAGTGGTAGAAATTAGGCCATTA---GGAATAGCACCCACTGCTGCAAAAAGAAGAGTGGTG--
-------------------------------------GAAAGACAAAAAAGAGCA---GTGGGA---
ATGGGAGCT---------GTGATCTTTGGGTTCTTGGGAGCA---GCAGGAAGCACTATG---
GGCGCAGCGTCG---ATAGCGCTGACGGCACAG---GCCAGACAA---TTGTTG---
TCTGGTATAGTGCAACAGCAAAAC---AATTTG---CTGAGG---
GCTATAGAGGCGCAACAGCATCTGTTGCAACTCACGGTCTGGGGCATT
>KU341724
TTCTATTGTAATACAACAGGCTTGTTTAATGGTACATACATGCCTACAGAAAGGCCTAATGGTACAGAAAGTA
AT---

ACAAACTCAACTATCACAATCCCATGCAGAATAAAACAAATTGTAAACATGTGGCAGGAGATAGGACGAGCAA
TGTATGCCCCTCCCATTGCAGGAAACATAACATGTAGATCAAAT---
ATCACAGGATTACTATTGGTACGTGATGGAGGAAAAAAGGCAAATAAT-------------------------
----

ACAAATGATACAGAGACATTCAGACCTGGAGGAGGGGATATGAGGGACAATTGGAGAAGTGAATTATATAAAT

ACAAAGTGGTAGAAATTAAGCCATTG---GGAGTAGCACCCACTGCAGCAAAAAGGAGAGTGGTG--------
-------------------------------GAGAGAGAAAAAAGAGCA---GTGGGA---CTGGGAGCT---
------GTGTTCCTTGGGTTCTTGGGAGCA---GCAGGAAGCACTATG---GGCGCGGCGTCA---
ATAACGCTGACGGTACAG---GCCAGACAA---TTGTTG---TCTGGTATAGTGCAACAGCAAAGC---
AATTTG---CTGATG---GCTATTGAGGCGCAACAGCATCTGTTGCAACTCACGGTCTGGGGCATT
>KJ158430
TTCTATTGTGATACAAAAAAACTGTTTAATGCTACATACATGAATGATGATACA-------------------
----
GCAAATAATTCAAGAAGC---------
AACGGCAATGACACCGTCACTCTCCCATGCAAGATAAAGCAAATTATAAACATGTGGCAGGAGGTAGGGCGAG
CAATGTATGCCCCTCCCATTGCAGGAAACATAACATGTAAATCAAAT---
ATCACAGGACTACTATTGGTACGTGATGGAGGAAAA-------------------------------------
----
ATGAACCAAAATATAGAGACAAAT---------
AAGACGGAGACATTCAGACCTGAAGGAGGAAATATGAAGGACAATTGGAGAAGTGAATTGTATAAATATAAAG
TGGTAGAAATTAAACCATTG---GGAATAGCACCCACTAAAGCAAAAAGGAGAGTGGTG--------------
-------------------------GAGAGAGAAAAAAGAGCA---GTGGGA---CTAGGAGCT---------
GTGTTCCTTGGATTCTTGGGAGTA---GCAGGGAGCACTATG---GGCGCGGCGTCA---
ATAACGCTGACGGCACAG---GCCAGACAA---TTGCTG---TCTGGTATAGTGCAACAGCAAAGC---
AATTTG---CTGAAG---GCTATAGAGGCGCAACAGCATCTGTTGCAACTCACGGTCTGGGGCATT
>KJ158433
TTCTATTGTGGGGTCCTTTGCCTGTTTAATAGGACATACATGATTCCGCCCAAAAAG----------------
----
AGTAATTCAAGCAGTAATCCA------------
AAATACAATAAATCAATCACAATGCACTGCAGAATAATACTAATTATCATCATGAGGGGGGAGGTGGAATTAG
CAACGTATTTTCCTCCCATCGCAGGATGCATTACATCAGAATCAAAT---
ATCACAGGAATAATGTTAACCCGAAATGGGGAC--

AAAAAAAGGAATAAGGCAACA---------
GGGACAGAGACATTCAGACCTGGAGGAGGAGATATGAGGAACAATTGGAGAAGTGAATTATATAAATATAAAG
TGGTAGAAATTAAGCCATTG---GGGGTAGCACCCACTACAGCAAAAAGGAGAGTGGTG--------------
-------------------------GAGAGAGAAAAAAGAGCA---ATGGGA---CTAGGAGCT---------
TTGTTCCTTGGGTTCTTGGGAACA---GCAGGAAGCACTATG---GGCGCGGCGTCA---
ATAACGCTGACGGTACAG---GCCAGACAA---TTGTTG---TCTGGTATAGTGCAACAGCAAAGT---
AATTTG---CTGAAA---GCTATAGAGGCGCAACAGCATCTGTTGCAACTCACGGTCTGGGGCATT
>KJ541840
TTCTATTGTAATACATCAGGCCTGTTTATTGATGCTACCAATGGTAATTCCAGG-------------------
----
GAAAATGGTACAAACAGT---------
AATTCAAGTTTAAACATCACAATCCCATGCAGAATAAAGCAAATTATAAATATGTGGCAGGAGGTAGGACGAG
CAATGTATGCTCCTCCCATTAAAGGAAACATAACATGTAAATCAAAT---
ATCACAGGAATACTATTGGCACGAGATGGTGGACAA-------------------------------------
----
GAAGCAAATAGCACCGAGGGGGGG---------
GACACAGATACATTCAGACCTGGAGGAGGAAATATGAAGGACAATTGGAGAAGTGAATTATATAAATATAAAG
TAGTAGAGATTAAGCCATTG---GGAATAGCACCCACTAAAGCAAAAAGGAGAGTGGTG--------------
-------------------------GAGAGAGAAAAAAGAGCA---GTGGGA---ATAGGAGCT---------
GTGTTCCTTGGGTTCTTGGGAGCA---GCAGGAAGCACTATG---GGCGCGGCGTCA---
ATAACGCTGACGGTACAG---GCCAGACAA---TTGTTG---TCTGGTATAGTGCAACAGCAAAGC---
AATTTG---CTGCAG---GCTATAGAGGCGCAACAGCATCTGTTGCAACTCACAGTCTGGGGCATT
>KJ158435
TTCTATTGCAATACATCAAAACTGTTTAATAGCACATACATGAGTAATGGTACA-------------------
----
AATAGGGGTTTAATTATT---------
ACTAATGATAAAAACATCACAATCCCATGCAGAATAAAACAAATTATAAACATGTGGCAGGAGGTAGGAAGAG
CAATGTATGCCCCTCCCATTGCAGGAAACATAACATGCAGATCAAAT---
ATCACAGGAATACTATTGGTACGTGATGGAGGAGAA-------------------------------------
----
GACAAAAATGGAGACACAAATGAT---------
ACGACCGAGACATTTAGACCTGGAGGAGGAAATATGAGGGACAATTGGAGAAGTGAGTTATATAAATATAAAG

TGGTAGAAATTAAGCCATTG---GGAATAGCGCCCACTGGAGCAAAAAGGAGAGTGGTG--------------
-------------------------GAGAGAGAAAAAAGAGCA---GTGGGA---ATAGGAGCT---------
GTGTTCCTTGGGTTCTTGGGAGCA---GCAGGAAGCACTATG---GGCGCGGCGTCA---
ATAACGCTGACGGTACAG---GCCAGACAA---TTGTTG---TCTGGTATAGTGCAACAGCAAAGC---
AATTTG---CTGAGG---GCTATAGAGGCGCAACAGCATATGTTGCAACTCACAGTCTGGGGCATT
>KJ541849
TTCTATTGTAATACATCAAGCCTGTTTAATGGGTCTAATGATACTGCCAATATT-------------------
----
GGTACCGACAATTTTACA---------
AACAGTAATGATAACATCACAATCCCATGCAGAATAAAGCAAATTATAAACATGTGGCAGGAGGTAGGACGAG
CAATGTATGCCCCTCCCATTGAAGGAAGTATAACATGTAACTCAAAT---
ATCACAGGACTACTATTGGTGCGTGATGGAGGAGGA-------------------------------------
----
GACAATAATGATACTAATACACAC---------
AAACCAGAGATATTCAGACCTGGAGGAGGAAATATGAGGGACAATTGGAGAAGTGAATTATATAAATATAAAG
TGGTAGAAATTAAGCCATTA---GGAGTAGCACCCACTGGGGCAAAAAGGAGAGTAGTG--------------
-------------------------GAGAGAGGAAAAAGAGCA---GTGGGA---ATAGGAGCT---------
GTGTTCCTTGGGTTCTTGGGAGCA---GCAGGAAGCACTATG---GGCGCGGCGTCA---
ATAACGCTGACGGCACAG---GCCAGACAA---TTGTTG---TCTGGTATAGTGCAACAGCAAAAT---
AATTTG---CTGAGG---GCTATAGAGGCGCAACAGCATCTGTTGCAACTCACGGTCTGGGGCATT
>MF373126
TTCTACTGCAATACATCAAACCTGATTAATAATAATATAAACATTGCTTCAGCG-------------------
----

AGCACAATACAACGCAGAATAAAACAAATTATAAACATGTGGCAGGGGGTAGGACGAGCAATGTATGCCCCTC
CCATTGCAGGAAACATAACATGTAAATCGAAT---
ATCACAGGACTACTACTGACACGTGATGGAGGAGAAAATAATAGG----------------------------
----

ACTGAGACATTCAGACCTGGAGGAGGAGATATGAAGGACAATTGGAGAAGTGAATTATATAAATATAAGGTGG
TAGAAAGTAAGCCATTA---GGAATAGCACCCACAAAGGCAAAAAGGAGAGTGGTG-----------------
----------------------GAGAGAGAGAAACGAGCA---GTGGGA---CTAGGAGCT---------
GTGTTCCTTGGGTTCTTAGGAGCA---GCAGGAAGCACTATG---GGCGCAGCATCA---
ATAACGCTGACGGTACAG---GCCAGACAA---TTATTG---TCTGGTATAGTGCAACAGCAAAGT---
AATTTG---CTGAAG---GCCATAGAGGCGCAACAGCATATGTTGCAACTCACAGTCTGGGGCATT
>MF373131
TTCTATTGCAATACATCACAACTGTTTAATGCATCAGATATTTTTAATGATACAACT----------------
----

AATAATACTACCAGCACAATTCCCTGCACTATATACAGGATAATGGACATAAGGCAAGAGATGTGGCCAGCAC
CGTATGCACCTATGATTGCCCGCACGATTACAAGAACGTCAACA---
ATCACTTCAATTCTCCCAAGACTTCTTCTAAGGAATGAGACAGGTAATGAGAAT------
GAGACACACCTGTATGAG---ACATATGAACAA--

CCTGGCAGAGACAATAGGAGAAATAATTGGAGGAAAGATTTACAGGCATATGAAGTGGCATCACTTGGAGCAA
TA---ACAATAGCGCCG---AAGAGAGATAAGAGAACAGTG---------------------------------
-------GAGAAAGCAGAAGGAGCA---CTAGGC---ATTCGAGCT---------
GTTCTCCGTGCATTCGTGAGACTT---CCAGGGAGCACTATG---GGCGCAGCATCA---
ATAACGCTGACGGTACAG---GCCAGACAA---TTACTG---TCTGGTATAGTGCAACAGCAAAAC---
AATTTG---GTGAGA---GCTATAGAGGCGCAACAGCACATGTTGCAACTCACAGTCTGGGGCATT
>MF373136
TTCTATTGTAATACATCAGGCCTGTTTAATGGTACATACATGCCTACATACATGCCTAATGGTACAGAAAGTA
AT---

TCAAACTCAACTATCACAATCCCATGCAGAATAAAGCAAATTATAAACATGTGGCAGGAGGTAGGACGAGCAA
TGTATGCCCCTCCCATTGCAGGAAACATAACATGTATATCAAAT---
ATCACAGGACTACTACTAGTACGTGATGGAGGACATGATGGAGAAAAT-------------------------
----

AATGACACGGAGACATTCAGACCTGGAGGAGGAAATATGAAGGACAATTGGAGAAGTGAATTATATAAATATA

AAGTGGTAGAAGTTAAACCATTG---GGGATAGCACCCACTAAAGCAAAAAGGAGAGTGGTG-----------
----------------------------GAGAGAGAAAAAAGAGCA---GTGGGA---ATAGGAGCT------
---GTGTTCCTTGGTTTCTTGGGAGCT---GCAGGAAGCACTATG---GGCGCGGCGTCA---
ATGACGCTGACGGTACAG---GCCAGACAA---TTGTTG---TCTGGTATAGTGCAACAGCAAAGC---
AATTTG---CTGAAG---GCTATAGAGGCGCAACAGCATCTGTTGCAACTCACGGTCTGGGGCATT
>KP411832
TTCTATTGCGACACATCACAACTGTTTAATGGTACATACAAGGCTACTAGTACTACTACTACT----------
----

TCACATTCCACCATCACACTCCAATGCAGAAGAAAACAAATTATAAATCTGTGGCAGGAGGTAGGACGAGCAA
TGTATGCCCCTTCCCTTGCAGGAAATATAACATGCGAATCAGGT---
ATCTCAGGATTACTAGTGACACGTGATGGAGGTAATGGTAATGAAACTGAT----------------------
----

AATAAGACAGAGACATTCAGACCTACAGGAGGAGATATGAGGGACAATTGGAGAAGTGAATTATATAAATATA
AAGTGGTAGAAATTAAACCATTA---GGAGTAGCACCAACTGGGGCAAAAAGGAGAGTGGTG-----------
----------------------------GAGAGAGAAAAAAGAGCA---GTGGGA---ATGGGAGCT------
---GTGATCCTTGGGTTCTTAGGAGCA---GCAGGAAGCACTATG---GGCGCAGCATCA---
ATAACGCTGACGGTACAG---GCCAGACAA---TTGTTG---TCTGGTATAGTGCAACAGCAAAGC---
AATTTG---CTGAGG---GCTATAGAGGCGCAACAGCATATGTTGCAACTCACAGTCTGGGGCATA
>KP411833
TTCTATTGCGATACATCAAAATTGTTTAATATTACATTTAATGGTAGCCTGTCTAATAATGTT----------
----

CCAAATTCAACCATCACACTCCAATGCAAAATAAAACAATTTATAAACATGTGGCAGGAGGTAGGACAAGCAA
TGTATGCCCCTCCTATTGCAGGAAACATAACATGTACATCAAAT---
ATCACAGGACTACTATTGACACGTGATGGAGGAACAGGGAAAAATAGT-------------------------
----

AAAAATGACACAGAGACATTCACACCTGGAGGAGGAGATATGAGGGACAATTGGAGAAGTGAATTATATAAAT
ATAAAGTGGTAGAAATTAAACCATTA---GGAATAGCACCCACTACGGCAAAAAGGAGAGTGGTG--------
-------------------------------GAGAAAGAAAAAAGAGCA---GTAACA---CTAGGAGCC---
------TTGTTCCTTGGGTTCTTGGGAGCA---GCCGGAAGCACTATG---GGCGCAGCATCA---
ATAACGCTGACGGTACAG---GCCAGACAA---TTATTG---TCTGGTATAGTGCAACAGCAGAAC---
AACCTG---CTGAGG---GCTATAGAGGCGCAACAGCATATGTTGCAACTCACGGTCTGGGGCATT
>MF373145
TTTTATTGCAATACAACAAACCTGTTTAATGGCAGTTTAGAAACACCAGTTATTACATGCAGACAAAATGTTC
CAATGCACCAT---CTA------CTT---
----------------------------------AATGCCAAT------------------
AAACAATTTATAAACATGTGGCAGGAGGTAGGACGAGCAATGTATGCCCCTCCTATTAAAGGAAACATAACAT
GTAGATCAAAT---ATCACAGGACTGCTACTGACAAGGATGGGGGCAACAACAGCAAC---------------
----

GATAGCGAGACATTCAGACCTGCAGGAGGAGATATGAGGGACAATTGGAGAAGTGAATTATATAAATATAAAG
TGGTAGAAATTAAGCCATTA---GGAATAGCACCCACTTCTGCAAAAAGGAGAGTGGTG--------------
-------------------------GAGAGAGAAAAAAGAGCA---GCA------CTAGGAGCT---------
ATGTTCCTTGGGTTCTTGGGAGCA---GCTGGAAGCACTATG---GGCGCAGCATCA---
ATAATGCTGACGGTACAG---GCCAGACAA---TTATTG---TCTGGTATAGTGCAACAGCAAAGC---
AATTTG---CTGAAA---GCTATAGAGGCGCAACAGCATATGTTGCAACTCACGGTCTGGGGCATT
>MF373146
TTTTATTGTAATACATCAGCCCTGTTCAATACATCAATCCCGTTCAATAGTACTATTACTAATAGTAGT----
----

ACAGATTCAAACATCACACTCCCATGCAGGATAAAACAAATTATAAACATGTGGCAAGGGGTAGGACAAGCAA
TGTATGCCCCTCCCATTGAAGGAAAAATAACATGTAGCTCAAAT---
ATCACAGGACTACTACTGACACGAGATGGAGGAGGAAATGAAACT----------------------------
----

GAAACTGAGACATTCAGGCCTGTAGGAGGAGATATGAGGAACAATTGGAGGAGTGAATTATATAAATATAAAG
TGGTAGAAATCAAGCCATTA---GGAATAGCACCCACTGGAGCAAAAAGGAGAGTGGTG--------------

-------------------------GAGAGAGAAAAAAGAGCG---GCA------CTAGGAGCT---------
CTGTTCCTTGGGTTCTTGGGAGCA---GCTGGAAGCACTATG---GGCGCAGCATCA---
CTAACGCTGACGGTACAG---GCCAGACAA---TTGTTG---TCGGGTATAGTGCAACAGCAAAAT---
AATTTG---CTGAGG---GCTATAGAGGCGCAACAGCATATGTTGCAACTCACGGTCTGGGGCATT
>MF373154
TTCTATTGCAATCCAACAAAATTGTTTAATGGTAATAGTACTTCA----------------------------
----

ACTTCAGACCCCATCATACTCCCATGCAGAATAAAACAAATTATAAACATGTGGCAGGGGGTAGGCCGAGCAA
TGTATGCCCCTCCCATTGCAGGAAAGATAACATGTAAATCAAAC---
ATCACAGGACTACTATTGACACGTGATGGAGGACACAACAATAAC----------------------------
----

GAGACAGAAACATTCAGACCTGAAGGAGGAAATATGAGGGACAATTGGAGAAGTGAATTATACAAATATAAAG
TGGTGGAAATTAATCCATTA---GGAATAGCACCCACTAAGGCAAAAAGGAGAGTGGTG--------------
-------------------------GAGAGAGAAAAAAGAGCA---GTGGGA---ATAGGAGCT---------
GTGTTCCTTGGGTTCTTGGGAGTA---GCAGGAAGCACTATG---GGCGCAGCGTCA---
TTGACGCTGACGGTACAG---GCCAGACAA---TTGCTG---TCTGGTATAGTGCAACAGCAAAAC---
AATTTG---CTGAGG---GCTATAGAGGCGCAACAGCATATGTTGCAACTCACAGTCTGGGGCATT
>KP411837
TTCTATTGCAACACATCAAACCTATTTAATAGAAATTTTTCAGAACTGGTTAATAACAGTACTAATAGTACTA
AT---

AGTACTTCAATCATCACACTCCGATGTAGAATAAAACAAATCATAAACATGTGGCAGGGGGTAGGACGAGCAA
TGTATGCCCCTCCCATTGAAGGAGACATAACATGTAAGTCAAAT---
ATCACAGGAATACTACTGACACGTGAGGGAGGAAAAAGACAGCAC----------------------------
----

CAGACAGAGATATTCAGACCTGGAGGAGGAGATATGAGGGACAATTGGAGAAGTGAATTATATAAATACAAAG
TGGTAGAGATTAAGCCACTA---GGAATAGCACCCACTAGAGCAAAAAGGAGAGTGGTG--------------
-------------------------GAGAGAGAAAAAAGAGCA---GTGGGA---ATAGGAGCT---------
GTGATCTTTGGGTTCTTGGGAGCA---GCCGGAAGCACTATG---GGCGCAGCATCA---
ATAGTGCTGACGGCACAG---GCCAGACAA---TTATTG---TCTGGTATAGTGCAACAGCAAAAC---
AATTTG---CTGAGG---GCCATAGAGGCGCAACAGCATATGTTGCAACTCACGGTCTGGGGCATT
>MF373156
TTCTATTGTGATACAACAAAATTGTTTAATGGTACATATGAGCATGAGAGTAATGCTACTTCAAATTCAAGT-
----

TCAAATTCAACCATCACACTCCAATGCAGAATAAAACAAATTATAAATATGTGGCAGGGGGTAGGACGAGCAA
TGTATGCCCCTCCCATTGAAGGAATCATACAATGTGCATCAAAT---
ATCACAGGACTATTATTGACACGTGATGGAGGAGAGAATAATAAT----------------------------
----

AATGACACAGAGACATTCAGACCTGGAGGAGGAGATATGAGGGACAATTGGAGAAGTGAATTATATAAATATA
AAGTGGTAGAAATTAAACCATTA---GGAATTGCACCCACTACTGCAAAAAGGAGAGTGGTG-----------
----------------------------GAGAGAGAAAAAAGAGCA---GTGGGA---ATAGGAGCT------
---GTGTTCCTTGGGTTCTTGGGGGCA---GCAGGAAGCACTATG---GGCGCAGCGTCA---
ATAACGCTGACGGTACAG---GCCAGACAA---TTGTTG---TCTGGCATAGTGCAACAGCAAAGC---
AATTTG---CTGAGG---GCTATAGAGGCGCAACAGCATATGTTGCAACTCACGGTCTGGGGCATT
>MF373157
TTCTATTGCAATACATCACAGCTGTTTGATTCAACACAGCTGGTTAATAGTAGTGGT----------------
----

AGTAATGCACACATCACAATCCCATGCAGAATAAAACAAATTATAAACCTGTGGCAAGAGGTAGGACGAGCAA
TGTATGCCCCTCCCATTGCAGGAAACATAACATGTAAATCAAAT---
ATCACAGGAATACTATTGACACGTGATGGAGGAAATACCAATGAAACA-------------------------
----

AATAGCACAGAGACATTCAGACCTGGAGGAGGAGATATGAGGGACAATTGGAGAAGTGAATTATACAAATATA
AAGTGGTAGAAGTTAAACCATTA---GGAGTAGCACCCACTAAGGCAAAAAGGAGAGTGGTG-----------

----------------------------GAGAAACAAAAAAGAGCA---GTGGGA---CTAGGAGCT------
---ATGTTCCTTGGGATCTTGGGAGCA---GCAGGAAGCACTATG---GGCGCAGCATCA---
ATAACGCTGACGGTACAG---GCCAGGCAA---TTGTTG---TCTGGTATAGTGCAACAGCAAAGC---
AATTTG---CTGAGG---GCTATAGAGGCGCAACAGCATATGTTGCAACTCACAGTCTGGGGCATT
>MF373170
TTCTACTGCAATACATCAGGCCTGTTTAATGGTACATACATGGGTAATATGACTAATAGTACTACAAAT----
----

GAAAATGACACCATCACCCTCCAATGCAGAATAAAACAAATGATAAACATGTGGCAGGAGGTAGGACGAGCAA
TGTATGCTCCTCCCATTGCAGGAAACATAGCATGTAGATCAGAA---
ATCACAGGACTACTATTGACACGTGATGGGGGAAATGGAAATGAAACAGAT----------------------
----

AATAACACAGAGATATTTAGACCTGGAGGAGGAGATATGAGAAACAACTGGAGAAGTGAATTATATAAATATA
AAGTAGTAGAAATCAAGCCATTA---GGATTAGCACCCACTAGAGCAAGAAGGCAAGTGGTG-----------
----------------------------CCGAGACAAAAAAGAGCA---GCA------ATAGGAGCT------
---ATATTCCTTGGGTTCTTGGGAGCA---GCTGGAAGCACTATG---GGCGCAGCGTCA---
ATGACGCTGACGGTACAG---GCCAGACAA---TTATTG---TCGGGTATAGTGCAACAGCAGAGC---
AATCTG---CTGAGA---GCTATAGAGGCGCAACAACATATGTTGCAACTCACAGTCTGGGGCATT
>MF373171
TTCTACTGCAATACATCAGGTCTGTTTAATGGTACATACATGGGTAATATGACTAATGGTAATATAAATGGA-
----

ACAAATGAAACCATCATCCTCCCATGCAGAATAAAACAAATGATAAACATGTGGCAGGAGGTAGGACGAGCAA
TGTATGCTCCTCCCATTGCAGGAAACATAGCATGTAACTCAACA---
ATCACAGGATTACTATTGACACGTGATGGAGGATCTGGAAATGAT----------------------------
----

AGTACCACAGAGATATTCAGACCTGGAGGAGGAGATATGAGAAACAACTGGAGAAGTGAATTATATAAATATA
AAGTAGTAGAAATCAAGCCATTA---GGATTAGCACCCACTACAGCAAGAAGGAGAGTGGTG-----------
----------------------------CAGAGAGAAAAAAGAGCA---GCA------ATAGGAGCT------
---ATGCTCCTTGGGTTCTTGGGAGCA---GCTGGAAGCACTATG---GGCGCAGCGTCA---
ATAACGCTGACGGTACAG---GCCAGACAA---TTATTG---TCGGGTATAGTGCAACAGCAGAGC---
AATCTG---CTGAGG---GCTATAGAGGCGCAACAACATATGTTGCAACTCACAGTCTGGGGCATT
>MF373177
TTCTATTGCGATACATCCAACCTGTTTAATGAAACAGCATTTAATAATAGT----------------------
----

GATGGTACAAACATCACACTCCCATGCAGAATAAAACAAATTATAAACATGTGGCAGAAGGTAGGACGGGCAA
TGTATGCCCCTCCCATTAAAGGACCAATAAAGTGTGACTCAAAT---
ATCACAGGATTACTACTAACACGAGATGGAGGCAATAGAGGCAAT----------------------------
----

GGGGGAAATGACACAGAAATATTCAGACCTGCAGGAGGAGATATGAGGGACAATTGGAGAAGTGAATTATATA
AATATAAGGTGGTAGAAATTAAGCCATTA---GGAATAGCACCCACTGCTGCAAAAAGGAGAGTGGTG-----
----------------------------------GGGAGAGAAAAAAGAGCA---GCA------
CTAGGAGCT---------ATGTTCCTTGGGTTCTTGGGAGCA---GCAGGAAGCACTATG---
GGCGCAGCATCA---ATAACGCTGACGGTACAG---GCCAGACAA---TTATTG---
TCTGGTATAGTGCAACAGCAAAGC---AATTTG---CTGAGA---
GCTATAGAGGCGCAACAACATATGTTGCAGCTCACAGTCTGGGGCATT
>MF373187
TTCTATTGCAATACATCAGGCCTGTTCAATGATACATACTGGAACAATAATACGAAAAATAATACAAAA----
----

GGAAATTCAAACTTCACAATCCAATGCAGAATAAAACAAATTATAAACATGTGGCAGGGAGTAGGAAGAGCAA
TGTATGCTCCTCCCATTGCAGGAGAAATAACATGTAGATCAAAT---
ATCACAGGACTACTACTGACACGGGATGGAGGAAATAAGACAGGAAATGGT----------------------
----

AATGAGACAGAGACATTCAGGCCGGGAGGAGGAGATATGAAGGACAATTGGAGAAGTGAATTATATAAATATA

AAGTGGTAGAAATTAGACCATTA---GGAATAGCACCCACCATGGCAAAAAGGAGAGTGGTG----------
---------------------------GAGAGAGAAAAAAGAGCA---GTGGGA---ATAGGAGCT------
---GTGCTCTTTGGGTTCTTGGGAGCA---GCAGGAAGCACTATG---GGCGCAGCGTCA---
ATAGCGCTGACGGTACAG---GCCAGACAA---TTATTG---TCGGGCATAGTGCAACAGCAAAGC---
AATTTG---CTGAGG---GCTATAGAGGCGCAACAGCATATGTTGCAACTCACGGTCTGGGGCATT
>MF373193
TTCTATTGCAATACATCAGGCCTGTTTAATAGTATATACTGGCCTAATGGTACATTCGCATTCAATGGTAGTA
CAAACAGT--
------------------AAC---
TCAACCAACAGCACAATCACAATCCCATGCAGAATAAAACAAATTATAAATATGTGGCAGGAGGTAGGACGAG
CAATGTATGCCCCTCCCATTAGGGGAAACATAACATGTACCTCAAAT---
ATCACAGGACTACTAATAATACGTGATGGAGGAAACAACGAGACA---------------------------
----

AATGAGACAGAGATATTCAGACCAGGGGGAGGAAATATGAAGGACAACTGGAGAAGTGAATTATACAAATATA
AGGTGGTAGAAATTAAGCCATTA---GGAGTAGCACCCACTGAAGCAAAAAGGAGAGTGGTG----------
---------------------------GGGAGAGAAAAAAGAGCA---GTGGGA---ATAGGAGCT------
---GTGTTCCTTGGGTTCTTGGGATTA---GCAGGAAGCACTATG---GGCGCGGCGTCA---
ATGACGCTGACGGTACAA---GCCAGACAA---TTGCTG---TCTGGTATAGTGCAACAGCAAAGC---
AATTTG---CTGAGG---GCTATAGAGGCGCAACAGCATATGTTGCAACTCACGGTCTGGGGCATT
>MF373199
TTCTATTGCAATACGTCTAGACTGATTAATAGTACATACAGGTCTAATAGTACTAATAGTACT----------
----

TCAGATACATCCATCATACTCCCATGCAGAATAAAACAAATTATAAACATGTGGCAGGGGGTAGGACGAGCAA
TGTATGCCCCTCCCATTGAAGGAAACATAACATGTAAATCAAAT---
ATTACAGGACTACTATTGACACGTGATGGAGGAACGGACAATGAA---------------------------
----

ACAATAGAGACATTCAGACCTGGAGGAGGAGATATGAGGGACAATTGGAGAAGTGAATTATATAAATATAAAG
TGGTAGAAATTAAACCATTA---GGAATAGCACCCACTAAGGCAAAAAGGAGAGTGGTG-------------
------------------------GAGAGAGAAAAAAGAGCA---GTGGGA---ATAGGAGCT---------
GTGTTCCTTGGGTTCTTGGGAGCA---GCAGGAAGCACTATG---GGCGCAGCATCA---
CTGACGCTGACGGTACAG---GCCAGGCAA---TTGCTG---TCTGGTATAGTGCAACAGCAAAGC---
AATTTG---CTGAGG---GCTATAGAGGCGCAACAGCATATGTTGCAACTCACGGTCTGGGGCATT
>MF373202
TTCTATTGCAATACATCAAACCTGTTTAATAGATCAATCCTGAATGTAACTGAAACAAATGTA----------
----

AATGAAGCAAACATCACCCTCCCATGCAGAATAAACCAATTTTTAAACATGTGGCAAGGGGTAGGACGAGCAA
TGTATGCCCCTCCCATTGAAGGAGAAATAACATGTAAATCAAAT---
ATCACAGGACTACTCCTGACACGTGATGGGGGTAATGGGAGAGAT---------------------------
----

AAGCAAAATGAAACATTTAGACCTGCAGGAGGAGATATGAGGGACAATTGGAGAAGTGAGTTATACAAATATA
AAGTGGTAGAACTTAAGCCATTA---GGACTAGCACCCACTACGGCAAAAAGGAGAGTGGTG----------
---------------------------GAGAGAGAAAAAAGAGCA---GTGGGA---TTTGGAGCT------
---CTGTTCTTTGGGTTCTTAGGAGCA---GCAGGAAGCACTATG---GGCGCAGCATCA---
ATAACGCTGACGGTACAG---GCCAGACAA---TTACTG---TTTGGTATAGTGCAACAGCAAAAT---
AATTTG---CTGAAA---GCTATAGAGGCGCAACAGCATATGTTGCAACTCACAGTCTGGGGCATT
>MH705137
TTTTATTGCAATACATCACAACTGTTTAATGGTACAATTACA------------------------------
----

ACAGATGGTTTAAACATCACAATCCCATGCAGAATAAAACAAATTATAAACATGTGGCAGGAGGTAGGACGAG
CAATGTATGCTCCTCCCATTGCGGGAAATATAATATGTAACTCAAGT---
ATCACAGGAATACTACTGACACGCGATGGAGGA--------------------------------------
----

GAGAATACTACAGAGACATTCAGACCTGGAGGAGGAGATATGAGGGACAATTGGAGAAGTGAATTATATAAAT

ATAAAGTAGTAGAAATTAAGCCATTA---GGAATAGCACCCACCAAGGCAAAAAGGAGAGTGGTG--------
-------------------------------GAGAGAGAAAAAAGAGCA---GTGACA---CTAGGAGCT---
------TTGTTCCTTGGGTTCTTGGGAGCA---GCAGGAAGCACTATG---GGCGCAGCGTCA---
ATAACGCTGACGGTACAG---GCCAGACAA---TTGTTG---TCTGGTATAGTGCAACAGCAAAGC---
AATTTG---CTGAGA---GCTATAGAGGCGCAACAGCATATGTTGCAACTCACAGTCTGGGGCATT
>MG989567
TTCTATTGTAACACAGCAGGACTGTTCAATGCAACATACAATAGTACAGAAGGAGATACA-------------
----

AACATTACAAACATCACACTCCCATGCAGAATAAGACAAATTATAAATATGTGGCAGGAAGTAGGAAGAGCAA
TATATGCCTCTCCCATTGAAGGAAATATAGCTTGTAACTCAAGT---
ATCACAGGACTACTATTGGTACGAGATGGAGGAAAT-------------------------------------
----

AGCTCAAATGAGACATTTAGACCTATAGGAGGAGATATGAAGAACAATTGGAGAAATGAATTATATAAATATA
AAGTGGTAGAAATTAAGCCATTG---GGAGTAGCCCCCACTGAAGCAAGAAGGAGAGTGGTG-----------
----------------------------GAGAGAGAAAAAAGAGCA---GTAGGA---CTGGGAGCT------
---GTGTTCCTTGGGTTCCTGGGAGCA---GCAGGAAGCACTATG---GGCGCGGCGTCA---
ATAACGCTGACGGTACAG---GCCAGACAA---TTACTG---TCTGGTATAGTGCAACAGCAAAGT---
AATTTG---CTGAGA---GCTATAGAGGCGCAACAGCATTTGTTGCAACTCACAGTCTGGGGCATT
>HM215433
TTCTATTGCAATACATCAAAACTGTTTAATAGTACATACAATCCTAATGGAACAAAAAATGATACAAAC----
----

TCAAGTGCATACATCATCCTTCCATGCAGAATAAAACAGATTATAAACATGTGGCAGGAGGTAGGACGAGCAA
TGTATGCCAATCCCATTGAAGGATACATAACATGTAGATCAAAT---
ATCACAGGACTACTATTAGTACGTGATGGAAGCACAAATAGTAGT----------------------------
----

AACGAGACATTCAGACCTGGAGGAGGAAATATGAAGGATAATTGGAGAAGTGAATTATATAAATATAAAGTGG
TAGAAATTAAGCCATTG---GGAATAGCACCCACTGAAGCAAAAAGGAGAGTGGTG-----------------
----------------------GAGAGAGAAAAAAGAGCA---GTGGGA---ATAGGAGCT---------
GTGTTCCTTGGGTTCTTGGGAGCA---GCAGGAAGCACTATG---GGCGCGGCGTCA---
ATGACGCTGACGGTACAG---GCCAGACAA---TTGTTG---TCTGGTATAGTGCAACAGCAAAGC---
AATTTG---CTGAGG---GCTATAGAGGCGCAACAGCATTTGTTGCAACTCACGGTCTGGGGCATT
>HQ659585
TTCTATTGCAATACATCAGGGCTGTTTGGTAGTACATACAGGAATACA-------------------------
----

GAAAATGGTACAATCATACTCCAATGCAGAATAAAGCAAATTATAAACATGTGGCAGGAGGTAGGAAGAGCAA
TGTATGCTCCTCCCATTGCAGGAAACATAACATGTAAGTCAAAT---
ATCACAGGATTACTATTGACGCGTGATGGAGGAACAAAT----------------------------------
----

GAAACAGAAATATTCAGACCTGGAGGAGGAGATATGAGGGACAATTGGAGAAGTGAATTATACAAATATAAAG
TGGTAGAAATTAAGCCATTA---GGAATAGCACCCACTGGGGCAAAAAGGAGAGTGGTG--------------
-------------------------GAGAGAGGAAAAAGAGCA---GTGGGA---ATAGGAGCT---------
GTGTTCCTTGGGTTCTTGGGAGCA---GCAGGAAGCACTATG---GGCGCGGCGTCA---
ATAACGCTGACGGTACAG---GCCAGACAA---TTATTG---TCTGGCATAGTGCAACAGCAAAAC---
AATTTG---CTGAGG---GCTATAGAGGCGCAACAGCATATGTTGCAACTCACGGTCTGGGGCATT
>HQ697934
TTCTATTGCAATACATCAAAACTGTTTAATGGTATATACAACTCTAATAATACAAGAAATGAT----------
----

TCAAATTCAACCATCATACTCCCATGCAGAATAAAACAAATTGTAAACATGTGGCAGGAGGTAGGACGAGCAA
TGTATGCCCCTCCCATTGCAGGAAACATAACATGTAAATCACAA---
ATCACAGGGCTACTATTGACATGGGATGGAGGCAAAGACAATAACTCAACA----------------------
----

GAGGATAACAATACAGAGATATTCAGACCTGGAGGAGGAGATATGAGGGACAATTGGAGAAGTGAATTATATA

AATATAAAGTGGTAGAAATTAAGCCATTA---GGAGTAGCACCCACTGGGGCAAAAAGGAGAGTGGTG-----
----------------------------------GAGAGAGAAAAAAGAGCA---GTGGGA---
ATAGGAGCT---------GTGTTCCTTGGGTTCTTGGGAGCA---GCAGGAAGCACTATG---
GGCGCGGCGTCA---ATCACGCTGACGGTACAG---ACCAGACAA---TTATTG---
TCTGGTATAGTGCAACAGCAAAGC---AATTTG---CTGAAG---
GCTATAGAGGCGCAACAGCATATGTTGCAGCTCACAGTCTGGGGCATT
>HQ697983
TTCTATTGCAATACATCAAACCTGTTTAATAATAACATAACT-------------------------------
----

TTAAACGGCACACTCACACTCCCATGCAGGATAAAACAAATTGTAAACATGTGGCAGGGGGTAGGACAAGCAA
TGTATGCCCCTCCCATTGAAGGAAACATAACATGTAAATCAAAC---
ATCACAGGAATATTATTGACACGGGATGGAGGGACAAATAGGACTGAA-------------------------
----

GGGAATAATAATGAGACATTCAGACCTGGAGGAGGAGACATGAGGGACAATTGGAGAAGTGAATTATATAAAT
ATAAAGTAGTAGAAATTAAGCCACTA---GGATTAGCACCCACCGAGGCAAAAAGGAGAGTGGTG--------
-------------------------------GAGAGAGAAAAAAGAGCA---GTGGGA---ATAGGAGCT---
------GTGTTCCTTGGGTTCTTGGGAGCA---GCAGGAAGCACTATG---GGCGCGGCGTCA---
ATAACGCTGACGGTACAG---GCCAGACAA---TTGTTG---TCTGGTATAGTGCAACAGCAAAGC---
AATTTG---CTGAGG---GCTATAGAGGCGCAACAGCATATGTTGCAACTCACGGTCTGGGGCATT
>HQ698009
TTCTATTGCAATACATCAGGACTGTTTCTGTTTAACAAATCAAGACCATTTACA-------------------
----

AAAAATGGGACAATCATACTCCCATGCAGAATAAAACAAATTATAAACATGTGGCAGGGGGTAGGACGAGCAA
TGTATGCCCCTCCCATTGCAGGAAACATAACATGTAACTCAAAT---
ATCACAGGACTATTATTGACACGTGATGGGGGAGGAAAAGGTAAT----------------------------
----

AACACAGAAACATTTAGACCTGGAGGAGGAGATATGAGGGACAATTGGAGAAGTGAATTATATAAATATAAAG
TAGTAGAAATTAAACCATTA---GGAATAGCACCCACTAAGGCAAAAAGGAGAGTGGTG--------------
-------------------------GAGAGAGAAAAAAGAGCA---GCA------ATAGGAGCT---------
GTGTTCCTTGGGTTCTTGGGAGCA---GCAGGAAGCACTATG---GGCGCGGCGTCA---
ATAACGCTGACGGTACAG---GCCAGACAA---CTGTTG---TCTGGTATAGTGCAACAGCAAAGC---
AATTTG---CTGAGA---GCTATAGAAGCGCAACAGCATCTGTTGCAGCTCACGGTCTGGGGCATT
>HM215286
TTCTATTGCAATACATTAGACCTGTTTAATAGTACATATGATAGTACATACGGGTCTAATAGTACA-------
----

GATAATTCAACCATCACACTCCACTGCAGAATAAAACAAATTATAAACATGTGGCAGGAGGTAGGACGAGCAA
TGTATGCCCCTCCCATTGAAGGAAACATAACATGTATATCAAAT---
ATTACAGGACTACTATTAACACGTGATGGAGGAGATAGAAATGATACA-------------------------
----

AATGACACAGAGACATTCAGACCTGGAGGAGGAGACATGAGGGATAATTGGAGAAGTGAATTATATAAATATA
AAGTAGTAGAAATTAAACCATTA---GGAATAGCACCCACACAGGCAAGAAGGAGAGTGGTG-----------
----------------------------GAGAGAGAAAAAAGAGCA---GTGGGA---ATAGGAGCT------
---GTGTTCCTTGGGTTCTTGGGAGCA---GCAGGAAGCACTATG---GGCGCGGCGTCA---
ATAACGCTGACGGTACAG---GCCAGACAA---TTGTTG---TCTGGTATAGTGCAACAGCAAAGC---
AATTTG---CTGAGG---GCCATAGAGGCGCAACAGCATATGTTGCAACTCACGGTCTGGGGCATT
>HM215302
TTCTATTGCAATACATCAAACCTGTTTAATGAAAGTTTATTTAATAGTACAGAAAATAGTAGT----------
----

AACAACACCATCATCACCCTCCCATGCAGAATAAAACAAATTATAAACATGTGGCAGGAGGTAGGACGAGCCA
TATATGCCCCTCCCATCGCAGGAAACATAACATGTAAATCAAAT---
ATTACAGGACTAATTTTGACACGTGATGGAGGAAACTCAAGTGAAACC-------------------------
----

AACCACACAGAGACATTCAGACCTGGAGGAGGAAATATGAGGGACAATTGGAGAAGTGAATTATATAAATATA
AAGTGGTAGAAATTAAGCCATTG---GGAATAGCACCTACCAAGGCAAAAAGGAGAGTGGTG----------
----------------------------GAGAGAGAAAAAAGAGCA---GTGGGA---ATGGGAGCT------
---ATGTTCCTTGGTCTCTTGAGTGCA---GCAGGAAGCACTATG---GGCGCGGCGTCA---
ATAACGCTGACGGTACAG---GCCAGACAA---CTGTTG---TCTGGTATAGTGCAGCAGCAAAGC---
AATTTG---CTGCGG---GCTATAGAGGCGCAACAGCATATGTTGCAACTCACAGTCTGGGGCATT
>AY253304
TTCTATTGCAATACATCAAAACTGTTTACAGGTGAATATAATGGTACTGAGTATAATGATACAGAATCAAATT
CA--

ACTTCATCAATCATCACACTCCCATGTAGAATAAAACAAATTATAAACATGTGGCAGAAGGTAGGACGAGCAA
TGTATGCCCCTCCTATTGAAGGAAACATAACGTGTAACTCAAGT---
ATCACAGGACTACTATTGACACGTGATGGAGGAAACAATACTAAT---------------------------
----

AACACAGAGACATTCAGACCTGGAGGAGGAGATATGAGGGATAATTGGAGAAGTGAATTATATAAATATAAAG
TGGTAGAAATTAAACCATTA---GGAATAGCACCCACTAGTGCAAAAAGGAGAGTGGTG-------------
------------------------GAGAGAGAGAAAAGAGCA---GTGGGA---ATAGGAGCT---------
TTGTTCCTTGGGTTCTTAGGAGCA---GCAGGAAGCACTATG---GGCGCAGCATCA---
ATAACGCTGACGGTACAG---GCCAGACAA---TTATTG---TCTGGTATAGTGCAACAGCAGAGC---
AATTTG---CTGAGG---GCCATAGAGGCGCAACAGCATATGTTGCAACTCACAGTCTGGGGCATT
>AY253308
TTCTATTGCGATACATCAGACCTGTTCAATAGAACATACGTGATGCCTAATGATACAGAAAATACAGAAGGTA
CG--

GCAAATACAACCATCAGACTCCAATGCAGAATAAAACAAATTGTAAATATGTGGCAGGGGGTAGGACGAGCAA
TGTATGCCCCTCCCATTGAAGGAAATATAGAATGTATATCAAAC---
ATCACAGGACTACTATTGACATGGGATGGAGGAAATGAAGCTAAT---------------------------
----

GACACAGAAGAGACATTCAGACCTGGAGGAGGAGACATGAGGGATAATTGGAGAAGTGAATTATATAAATATA
AAGTGGTAGAAATTAAGCCATTA---GGAATAGCACCCAGTATGGCAAAAAGGAGAGTGGTG----------
----------------------------GAGAGAGAAAAAAGAGCA---GTGGGA---ATAGGAGCT------
---GTGCTCCTTGGGTTCTTGGGAGCA---GCAGGAAGCACTATG---GGCGTGGCGTCA---
ATGACGCTGACGGTACAG---GCCAGACAA---TTGTTG---TCTGGTATAGTGCAACAGCAAAGC---
AATTTG---CTGAGG---GCTATAGAGGCGCAACAGCATATGTTGCAACTCACAGTCTGGGGCATT
>AY253310
TTCTATTGCAATACATCACAACTATTTAATAGTATATATAATAATAGTACATTTAATAGTAATAGTACATCTA
ATAGT---
-------------------ACA---
AATAATACTGAATACATCACACTCCAATGTAGAATAAAACAAATTATAAACATGTGGCAGGAGGTAGGACGAG
CAATGTATGCCCCTCCCGTTGCAGGAAACATAACATGTATATCAAAT---
ATTACAGGACTACTATTGACACGTGATGGAGGAAGCAGTGGAGGAAACAAT---------------------
----

AGCGAAGAGACAGAGGAGACATTCAGACCTGGAGGAGGAGATATGAGGGACAATTGGAGAAGTGAATTATACA
AGTATAAAGTGGTAGAAATTAAGCCATTG---GGAATAGCGCCCACTACAGCAAAAAGGAGAGTGGTG-----
----------------------------------GAGAGAGAAAAAAGAGCA---GTTGGA---
ATAGGAGCT---------GTGCTCTTTGGGTTCTTGGGAGCA---GCAGGAAGCACTATG---
GGCGCGGCGTCA---ATAACGCTGACGGTACAG---GCCAGACAA---GTGTTG---
TCTGGTATAGTGCAACAACAAAGC---AATCTG---CTGAGG---
GCTATAGAGGCGCAACAGCATATGTTGCAACTCACGGTCTGGGGCATT
>AY253312
TTCTATTGCAATACATCAGGGCTGTTCAATGGTACATACAATGGCACATACAATGGTACA------------
----

ACAAATTCAACCATCACACTCCAATGCAGAATAAAACAAATTATAAACATGTGGCAGAAGGTAGGACGAGCAA
TGTATGCCCCTCCCATTGCAGGAAACATAACATGTAACTCAACT---
ATCACAGGACTAATATTGACACGGGATGGAGGAAACGGAAACGTT---------------------------
----

ACAGGAAACGAAACATTCAGACCTGCAGGAGGAGATATGAAAGACAATTGGAGAAGTGAATTATATAAATATA
AAGTGGTAGAAGTTAAGCCATTA---GGAATAGCACCCACTAAGGCACAAAGAAAAGTGGTG-----------
----------------------------CAGAGAGAGAAAAGAGCA---GTGGGA---ATAGGAGCT------
---GTGTTCCTTGGGTTCTTGGGAGCA---GCAGGAAGCACTATG---GGCGCGGCATCA---
ATAACGCTGACGGTACAG---GCCAGACAA---TTATTG---TCTGGTATAGTGCAACAGCAAAGC---
AATTTG---CTGAGA---GCTATAGAGGCGCAACAGCATATGTTGCAACTCACGGTCTGGGGCATT
>AY253303
TTCTATTGCAATACATCAAACTTGTTTAATAAGACAATTTTGGCTAATATAACGGGTAATGGT----------
----

ACAAATGCAAACATCACACTCCCATGCAGGATAAAACAAATTATAAACATGTGGCAGGGGGTAGGACGAGCAA
TGTATGCCCCTCCCATCGCAGGAGAAATAACATGTAAATCAAAT---
ATCACAGGATTACTATTGACACGTGATGGAGGAAATAATAAGACA----------------------------
----

AACGAATCAGAGATATTTAGACCTATAGGAGGAGATATGAGGGACAATTGGAGAAATGAATTATATAAATATA
AAGTGGTAGAAATTAAACCATTA---GGAGTAGCACCCACTGGTGCAAAAAGGAGAGTGGTG-----------
----------------------------GAGAGAGAAAAAAGAGCA------------GCGGGAGTT------
---GTGCTCCTTGGGTTCTTGGGAGCA---GCAGGAAGCACTATG---GGCGCAGCATCA---
ATGTGGCTGACGGTACAG---GCCAGACAA---TTACTG---TCTGGTATAGTGCAACAGCAAAGT---
AATTTG---CTGAGG---GCTATAGAGGCGCAACAGCATCTGTTGCAACTCACAGTCTGGGGCATT
>AY253313
TTCTATTGCAATACATCAATACTGTTTAATAATAGCATGCTTAATGGTACAGAAGGTACTCCAAAT-------
----

TCAACTGAAGATATCACAATCCCATGTAGAATAAAACAAATTATAAACATGTGGCAGGGGGTAGGACGAGCAA
TGTATGCCCCTCCCATTGCAGGAAGCATAACATGTAAGTCAAAT---
ATCACAGGACTATTATTGACACGTGATGGAGGAATAAATGAGGAT----------------------------
----

AATACAACAGAGATATTCAGGCCTGGAGGAGGAGATATGAGGGATAATTGGAGAAGTGAATTATATAAATATA
AAGTGGTAGAAATTAAGCCATTA---GGAGTAGTACCCACTAATGCAAAAAGGAGAGTGGTG-----------
----------------------------GAGAGAGAAAAAAGAGCA---GTGGGA---ATAGGAGCT------
---GTGTTCCTTGGGTTCTTAGGAGCA---GCAGGAAGCACTATG---GGCGCAGCATCA---
ATAACGCTGACGGTACAG---GCCAGACAA---TTATTG---TCTGGTATAGTGCAACAGCAAAGC---
AATTTG---CTGAGG---GCTATAGAGGCGCAACAGCATATGTTGCAACTCACAGTCTGGGGCATT
>AY253322
TTCTATTGCAACACATCAGGTCTGTTTAATAGTACATACAACTCTAATGGTACCAATAGTACA----------
----

ACAAACTCAACCATCACACTTCCATGCAGAATAAAACAAATTATAAACATGTGGCAGGGGGTAGGACGAGCAA
TGTATGCCAATCCCATTGCAGGAAACATAACATGTAAATCAAAT---
ATCACAGGACTATTATTGGTACGTGATGGAGGTACAGATAATGAA----------------------------
----

ACAGAAACATTCAGACCTGGAGGAGGAAATATGAGGGACAATTGGAGAAGTGAATTATATAAATATAAAGTGG
TAGAAATTAGGCCATTG---GGAATAGCACCCACTAAGGCAAAAAGGAGAGTGGTG-----------------
----------------------GAGAGAGAAAAAAGAGCA---GTGGGA---ATAGGAGCT---------
GTGCTCCTTGGGTTCTTGGGAGCA---GCAGGAAGCACTATG---GGCGCGGCGTCA---
ATGACGCTGACGGTACAG---GCCAGACAA---CTGTTG---TCTGGTATAGTGCAACAGCAAAGC---
AATTTG---CTGAGA---GCTATAGAGGCGCAACAGCATCTGTTGCAACTCACGGTCTGGGGCATT
>MK501601
TTCTATTGCAACACATCAGGTCTGTTTAATAGTACATACAACTCTGCATACAAGTCTAATGATACA-------
----

ACAAACTCAACCATCACACTTCCATGCAGAATAAAACAAATTGTAAACATGTGGCAGGAGGTAGGACGAGCAA
TGTATGCCCCTCCCATTGCAGGAAACATAACATGTAAATCAAAT---
ATCACAGGATTATTATTAGTGCGTGATGGAGGAGGTGAAAGTAAT----------------------------
----

GAAACAGAAATATTCAGACCTGGAGGAGGAGATATGAGGGACAATTGGAGAAGTGAATTATATAAATACAAAG
TGGTAGAAGTTAAACCATTG---GGAATAGCACCCACTGAAGCAAAAAGGAGAGTGGTG--------------
-------------------------AAGAGAGATAAAAGAGCA---GTAGGA---ATAGGAGCT---------
ATGTTCCTTGGGTTCTTGGGAGCA---GCAGGAAGCACTATG---GGCGCGGCGTCA---
ATGACGCTGACGGTACAG---GCCAGACAA---GTGCTG---TCTGGTATAGTGCAACAGCAAAGC---
AATTTG---CTGAGG---GCTATAGAGGCGCAACAGCATATGTTGCAACTCACAGTCTGGGGCATT
>AY734550
TTCTATTGCAATACATCAAGCCTGTTTAATGATAGCCTGGTTAATGATACAGAAAGTGAGAATAACACAAAAA
GT---

GATAATGGTACAATCACACTCCCATGCAGAATAAAACAAATTATAAACCTGTGGCAGGAAGTAGGACGAGCAA
TGTATGCCCCTCCCATTGCAGGAAACATAACATGTAAATCAAAT---
ATCACAGGATTACTCTTGACACGTGATGGAGGAGACAAAAATAACACA-------------------------
----

AACAATACCGCAGAGATATTCAGACCTGGAGGAGGAAATATGAGGGACAACTGGAGAAGTGAATTATATAAAT
ATAAAGTGGTAGAAATTAAACCATTG---GGAATAGCACCCACTGCGGCAAAAAGGAGAGTGGTG--------
-------------------------------GAGAGAGAAAAAAGAGCA---GTAGGA---ATAGGAGCT---
------GTGTTCCTTGGGTTCTTGGGAGCA---GCTGGAAGCACTATG---GGCGCAGCGTCG---
ATAACGCTGACGGTACAG---GCCAGACAA---CTGTTG---TCTGGTATAGTGCAGCAGCAAAGC---
AATTTG---CTGAGG---GCTATAGAGGCGCAACAGCATATGTTGCAACTCACGGTCTGGGGCATT
>AY734551
TTCTATTGCAATACAACAATACTGTTTAATAGGACATACAGCCCTAATGGCACAGACAATAATGGCACATACA
AT---

AATAGTGATTCGACCATCACAATCCCATGCAGAATAAAGCAAATTATAAACATGTGGCAGGAGGTAGGACGAG
CAATGTATGCCCCTCCCATTGCAGGAAACATAACATGTAATTCAAGT---
ATCACAGGACTAATATTGACACGTGATGGTGGTAATAAAAATGATACA-------------------------
----

AATGATACAGAGATATTTAGACCTGAAGGAGGAAATATGAAAGACAATTGGAGAAGTGAATTGTATAAATATA
AGGTGGTAGAAATTAAGCCATTA---GGAATAGCACCCACTAATGCAAAAAGGAGAGTGGTG-----------
----------------------------GAGAGAGAGAAAAGAGCA---GTGGGA---ATAGGAGCT------
---GTGTTCCTTGGGTTCTTGGGAGCA---GCAGGAAGCACTATG---GGCGCAGCATCA---
ATAACGCTGACGGTACAG---GCCAGACAA---TTATTG---TCTGGTATAGTGCAACAGCAAAGC---
AATTTG---CTGAGG---GCTATAGAGGCGCAACAACATATGTTGCAACTCACGGTCTGGGGCATT
>AY734558
TTCTACTGCAATACATCAAAGCTGTTTAATAGTCTTAATGATACAGAC-------------------------
----

AATAGTACAGACATCACACTCCCATGCAGAATAAAACAAATTATAAACATGTGGCAGGGGGTAGGACGAGCAA
TGTATGCCCCTCCCATTGCAGGAAACATAACATGTAAATCAAAT---
ATCACAGGACTACTATTGACACGAGATGGAGGAACAAACAATACA----------------------------
----

GATACAGAGATATTCAGGCCTGGAGGAGGAGATATGAGGGACAATTGGAGAAGTGAATTATATAAATATAAAG
TGGTAGAAATTAAGCCATTA---GGAATAGCACCAACCACGGCAAAAAGGAGAGTGGTG--------------
-------------------------GAGAGAGAAAAAAGAGCA---GTGGGA---GTAGGAGCT---------
GTGTTCCTTGGGTTTTTAGGAGCA---GCCGGAAGCACTATG---GGCGCAGCATCA---
ATAACGCTGACGGTACAG---GCCAGACAA---TTATTG---TCTGGTATAGTGCAACAGCAAAGC---
AATTTG---CTGAGG---GCTATAGAGGCGCAACAGCATATGTTGCAACTCACAGTCTGGGGCATT
>AY734559
TTCTATTGCAATACATCAGGCCTGTTCAATAGTACGTTTAATGAGGGATATAATAATACAAGAAATACTTCAG
AAAAAACA---
-------------------AAT---
AATACTTCAGAAACCATCACACTCCAATGCAGAATAAAACAATTTATAAACATGTGGCAGGGGGTAGGACGAG
CAATGTATGCCCCTCCCATTGCAGGAAACATAACATGTAAATCAAAT---
ATCACAGGATTATTATTAACACGTGATGGAGGAGAAGGAAACGGTACAGAT----------------------
----

ACAAATACAACAGAGATATTCAGACCACAAGGAGGAAATATGAAGGACAATTGGAGAAGTGAATTATATAAAT
ATAAAGTAGTAGAAATTAGACCATTA---GGAATAGCACCTACTAATTCAAAAAGGAGAGTGGTG--------
------------------------------CAGAGAGAGAAAAGAGCA---GTGGGAGTAATGGGAGCT---
------ATGTTCCTTGGGTTCTTGGGAGCT---GCAGGAAGCACTATG---GGCGCGGCATCA---
ATAACGCTGACGGTACAG---GCCAGACAA---TTATTG---TCTGGTATAGTGCAACAGCAAAAT---
AATTTG---CTGAAA---GCTATAGAAGCGCAACAGCATATGTTGCAACTCACGGTCTGGGGCATT
>AY734560
TTCTATTGTAATACATCAAACCTGTTTAATAATTCACTGTTTAATGATACTTCA------------------
----

AATGATACAGTCATCACACTCCCCTGCAGAATAAAACAAATTATAAACATGTGGCAGGAGGTAGGACGAGCCA
TATATGCCCCTCCCATTGCTGGAAATATAACATGTAGATCAAAT---
ATCACAGGACTATTGTTGACACGTGATGGAGGAGTTAATGCCAGT---------------------------
----

AAAGAAACATTCAGACCTGGAGGAGGAGATATGAGGGATAATTGGAGAAGTGAATTATATAAATATAAAGTAG
TAGAAATTAATCCATTA---GGAGTAGCACCAACTAAGGCAAAAAGGAGAGTGGTG---------------
---------------------GAGAGAGAGAAAAGAGCA---GCGGGA---ATAGGAGCT---------
TTGATCCTTGGGTTCTTAGGAGCA---GCAGGAAGCACTATG---GGCGCAGCATCA---
ATAACGCTGACGGTACAG---GCCAGACAA---TTGTTG---TCTGGTATAGTGCAACAGCAGAGC---
AATTTG---CTGAGG---GCTATAGAGGCGCAACAGCATATGTTGCAACTCACAGTCTGGGGCATT
>KX907356
TTCTATTGTAATACAACACAACTATTTAATAATATTAGTATAGGAAGATTCAATACTAGTATAGAA-------
----

AATGATACAAACATCATACTCCCATGCAGAATAAAACAAATTATAAATATGTGGCAGGAGGTAGGACGAGCAA
TGTATGCCCCTCCTATTGAAGGGAACATAACATGCAGATCAAAC---
ATCACAGGAATACTATTAACACGTGATGGAGGCAATGGAGGCGCT---------------------------
----

GACAATTCAACAGAGACATTCAGACCTGGAGGAGGAAATATGAAGGACAATTGGAGAAGTGAATTATATAAAT
ATAAAGTGGTAGAAATCAAGCCATTA---GGAATAGCACCCACTAGGGCAAAAAGGAGAGTGGTG--------
------------------------------GAGAGAGAAAAAAGAGCA---GTGGGA---ATAGGAGCT---
------GTGTTCCTTGGGTTCTTGGGAGCA---GCTGGAAGCACTATG---GGCGCGGCGTCA---
ATAACGCTGACGGTACAG---GCCAGACAA---CTGTTG---TCTGGTATAGTGCAACAGCAAAGC---
AATTTG---CTGAGG---GCTATAGAGGCACAGCAACATATGTTGCAGCTCACTGTCTGGGGCATT
>KX907388
TTCTATTGCAATACATCAAAACTGTTTAATGATACATACAATGGGACAGAA---------------------
----

AGTAACTCAACCATCACACTCCCATGCAGAATAAAACAAATTATAAACATGTGGCAGGGGGTAGGACGAGCAA
TGTATGCCCCTCCCATTGCAGGAAACATAACATGTAAATCAATG---
ATCACAGGACTACTATTGGTACGGGATGGGGGCACGGGTAAAAAT---------------------------
----
-
GACACAGAAAATGCCACAGAAACATTCAGACCTGAAGGAGGAGATATGAGGGATAATTGGAGAAGTGAATTAT
ATAAATATAAAGTGGTAGAAATTAAGCCATTG---GGAATAGCACCCACTGAGGCAAAAAGGAGAGTGGTG--
------------------------------------GAGAGAGAAAAAAGAGCA---GTGGGA---
ATAGGAGCT---------GTGTTCCTTGGGTTCTTGGGAGCA---GCAGGAAGCACTATG---
GGCGCAGCGTCA---ATAACGCTGACGGTACAG---GCCAGACAA---CTGTTG---
TCTGGTATAGTGCAACAGCAAAAC---AATTTG---CTGAGG---
GCTATAGAGGCGCAACAGCATATGTTGCAACTCACAGTCTGGGGCATT
>KX907407
TTCTATTGCAACACATCAAAACTGTTTAATAGTACATACATGCCTGGGTACTGGCCTAATGCTACAGAAAGAA
AT--

TCAACCTCAAATATCACACTCCATTGCAGAATAAAACAAATTATAAACATGTGGCAGGGAGTAGGACGGGCAA
TGTATGCCCCTCCCATTAGAGGAAACATAACATGTATATCAAAT---
ATCACAGGACTACTATTGACATGGGATGGAGATAAAACAAGCAAT---------------------------

----

GACCCAGACACAGACGTATTCAGACCTGGAGGAGGAAATATGAAGGATAATTGGAGAAGTGAATTATATAAAT
ATAAAGTGGTAGAAATTAAGCCATTA---GGAATAGCACCCACCAGGGCAAAAAGGAGAGTGGTG--------
-------------------------------GAGAGAGAAAAAAGAGCA---GTGGGA---ATAGGAGCT---
------GTGCTCCTTGGGTTCTTGGGAGCA---GCCGGAAGCACTATG---GGCGTCGCGTCA---
ATAACGCTGACGGTACAG---GCCAGACAA---TTATTG---TCCGGTATAGTGCAACAGCAAAGT---
AATTTG---CTGAGG---GCTATAGAGGCGCAACAGCATATGTTGCAACTCACAGTCTGGGGCATT
>KX907424
TTCTATTGCAATACATCAAAATTGTTTAATAATAATGATACAGAA----------------------------
----

AATAATTTAACCATCACACTCCCATGCAGAATAAAGCAAATTGTAAACATGTGGCAGGGGGTAGGACGAGCAA
TGTATGCCCCTCCCATTGAAGGAAACATAACATGTAAATCAAAT---
ATCACAGGATTATTATTGACACGTGATGGAGGCAATGGCACGGAG----------------------------
----

AGGAATGAGACATTCAGACCTGGAGGAGGAGATATGAGGAACAATTGGAGAAGTGAATTATATAAATATAAAG
TGGTAGAAATTAAACCATTG---GGAATAGCACCCACTGGGGCAAAAAGGAGAGTGGTG--------------
-------------------------GAGAGAGAAAAGAGAGCA---GTGGGC---ATGGGAGCT---------
GTGTTCCTTGGGTTCTTGGGAGCA---GCAGGAAGCACTATG---GGCGCGGCGTCA---
CTAACGCTGACGGTACAA---GCCAGACAA---GTGTTG---TCTGGTATAGTGCAGCAGCAAAGC---
AATTTG---CTGAGA---GCTATAGAGGCGCAACAACATATGTTGCAACTCACAGTTTGGGGCATT
>KX907426
TTTTATTGCAATACATCAAGCCTGTTTAAGGGTACATTTAATGGTACGGTTACAGATACCTCAAATTTAACAG
AA---

ATCACACTCCACTGTAGAATAAAACAAATTGTAAACATGTGGCAAGGAGTAGGACGAGCAATGTATGCCCCTC
CCATTGCAGGAAATATAACTTGTAAATCAAAT---
ATCACAGGATTACTATTGACACGTGATGGAGGATGTGATAACAAC----------------------------
----

GGTACAGAGATATTCAGGCCTGGAGGAGGAGATATGAGGGATAATTGGAGAAGTGAGTTATACAAATATAAAG
TAGTAGAAATTAAGCCATTA---GGAATAGCACCCACTAAGGCAAAAAGGAGAGTGGTG--------------
-------------------------GAGAGAGAAAAAAGAGCA---GTGGGA---ATAGGAGCT---------
GTGTTCCTTGGGTTCTTAGGAGCA---GCAGGAAGCACTATG---GGCGCAGCATCA---
ATAACGCTGACGGTACAG---GCCAGACAA---TTATTG---TCTGGTATAGTGCAACAGCAAAAC---
AATTTG---CTGAGG---GCTATAGAGGCGCAACAGCATATGTTGCAACTCACAGTCTGGGGCATT
>KX907345
TTCTATTGCAATACATCAAAACTGGTTAATGAGAATGAGACAGACCCTAATAGAGCTAAT-------------
----

AGTAGTGAGCCAATCACACTCCAATGCAGAATAAAACAAATTATAAACATGTGGCAGGGGGTAGGACGAGCAA
TGTATGCCCCTCCCATTGAAGGAAACATAACATGTAGGTCAAAT---
ATCACAGGACTGCTATTGACACGCGACGGAGGTGCCAATAGTACC----------------------------
----

ACAAATGAAACATTCAGATTTGAAGGAGGAAATATGAAGGACAATTGGAGAAGTGAATTATATAAATATAAGG
TGGTAGAGATTAAGCCATTA---GGAATAGCACCCACTAAGGCAAAAAGGAGAGTGGTG--------------
-------------------------GAGAGAGAAAAAAGAGCA---GTGGGA---ATAGGAGCT---------
GTGTTCCTTGGGTTCTTGGGAGCA---GCAGGAAGCACTATG---GGCGCAGCATCA---
ATAACGCTGACGGTACAG---GCCAGACAA---TTGTTG---TCTGGTATAGTGCAACAGCAAAGC---
AATTTG---CTGAGG---GCCATAGAGGCGCAACAGCACATGTTGCAACTCACGGTCTGGGGCATT
>KX907384
TTCTATTGTAACACATCACAACTATTTAATGGTACCAGTACAGGATTTAGTAATAAGAGTACA----------
----

GGAAATGAAACCTTCACACTCCCATGCAGAATAAAACAAATTATAAACATGTGGCAGGAGGTAGGACGAGCAA
TGTATGCCCCTCCCATTGCAGGAAACATAACATGTGTATCAAAT---
ATCACAGGACTACTATTGACACGCGATGGAGGAGATAATAATACT----------------------------

----

AGAACTGAGACATTCAGACCTGGAGGAGGAGATATGAGGGACAATTGGAGAAGTGAATTATATAAATATAAAG
TAGTAGAAGTTAAACCACTA---GGAGTAGCACCCTCTGAGGCAAGAAGGAGAGTGGTG--------------
-------------------------GAGAGAGAAAAAAGAGCA---GTGGGA---CTAGGAGCT---------
GTGTTCCTTGGGTTCTTGGGAGCA---GCCGGAAGCACTATG---GGCGCGGCGTCG---
ATAACGCTGACGGTACAG---GCCAGACAA---CTGTTG---TCTGGTATAGTGCAACAGCAAAGC---
AATTTG---CTGAGA---GCTATAGAAGCACAGCAGCATATGTTGCAGCTCACGGTCTGGGGCATT
>KX907392
TTCTATTGCAATACATCAAAACTGTTTAATGATACAAACTTCACCACGTCT----------------------
----

GATGATGAAAACATCACACTCCCATGCAGAATAAAACAAATTATAAACATGTGGCAGGAGGTAGGACGAGCAA
TGTATGCCCCTCCCATTGCAGGAAACATAACATGCACATCAAAA---
ATCACAGGACTACTATTGACACGTGATGGGGGAACCACTGGCCCC----------------------------
----

AATAATACCGAGACATTCAGACCTATAGGAGGAAATATGAAGGACAATTGGAGAAGTGAATTATACAAATATA
AGGTGGTGGAAATTAAGCCATTA---GGAGTAGCACCCACTAAGGCAAAAAGGAGAGTGGTG-----------
----------------------------GAGAGAGAAAAAAGAGCA---GTGGGA---ATAGGAGCT------
---ATGTTCCTTGGGTTCTTGGGGGCA---GCAGGAAGCACTATG---GGCGCGGCGTCA---
ATAACGCTGACGGTACAG---GCCAGACAA---GTGTTG---TCTGGTATAGTGCAACAGCAAAGC---
AATTTG---CTGAGG---GCTATAGAGGCGCAACAGAATATGTTGCAACTCACGGTCTGGGGCATT
>KX907394
TTCTATTGCAATACATCAAAACTGTTTAATGGTACATACAGGTCTAATAGTACAGAAAAGAAT----------
----

TCAAATGCAAACATCACACTCCCATGCAGAATAAAACAAATTGTAAACATGTGGCAGGAGGTAGGGCGAGCAA
TGTATGCCCCTCCCATTGCAGGAAACATAACATGTAAATCAAAT---
ATCACAGGACTACTATTGACACGTGATGGGGGAAAGGATAATAATACT-------------------------
----

AATACCACAGAGACATTCAGACCAGGAGGAGGAGATATGAGGGACAATTGGAGAAGTGAATTATATAAATATA
AAGTAGTAGAAGTTAATCCACTA---GGAATAGCACCCACTACTGCAAAAAGGAGAGTGGTG-----------
----------------------------GAGAGAGAGAAAAGAGCA---GTGGGA---ATAGGAGCT------
---TTGTTCCTTGGGTTCTTGGGAGCA---GCAGGAAGCACTATG---GGCGCGGCGTCA---
ATGGCGCTGACGGTACAG---GCCAGACAA---TTATTG---TCTGGTATAGTGCAACAGCAAAGC---
AATTTG---CTGAGA---GCTATAGAGGCGCAACAACATATGTTGCAACTCACAGTCTGGGGCATT
>KX907395
TTCTATTGCAATACATCAAAACTGTTTAATGGTACAGAAAAG-------------------------------
----

AATTCAAATGCAAACATCACACTCCCATGCAGAATAAAACAAATTGTAAACATGTGGCAGGGGGTAGGGCGAG
CAATGTATGCCCCTCCCATTGCAGGAAACCTAACATGTAAATCAAAA---
ATCACAGGACTACTATTGACACGTGATGGGGGACCAAATGAGAAT----------------------------
----

GACACAGAGATATTCAGACCTGGAGGAGGAGACATGAGGGACAATTGGAGAAGTGAATTATATAAATATAAAG
TAGTAGAAATTAATCCATTA---GGAGTAGCACCCACTACTGCAAAAAGGAGAGTGGTG--------------
-------------------------GAGAGAGAGAAAAGAGCA---GTGGGA---GTAGGAGCT---------
ATGTTCCTTGGGTTCTTGGGAGCA---GCAGGAAGCACTATG---GGCGCGGCGTCA---
CTAACGCTGACGGTACAG---GCCAGACAA---TTATTG---TCTGGTATAGTGCAACAGCAAAGC---
AATTTG---CTGAGG---GCCATAGAGGCGCAACAACATATGTTGCAACTCACAGTCTGGGGCATT
>KX907405
TTCTATTGCAATACATCACAACTGTTTAATGAGACACACACTAATGGT-------------------------
----

ACCAATAGTACCATCATACTCCCATGCAGAATAAAACAAATTATAAACATGTGGCAGGAGGTAGGACGAGCAA
TGTATGCCCCTCCCATTGCAGGGAACATAACATGTACATCAAAT---
ATTACAGGACTATTATTGGAACGGGATGGAGGCAAGGACAATAAT----------------------------

----

ATGACTGAGATATTCAGACCTGGAGGAGGAGATATGAGGGATAATTGGAGAAGTGAACTATATAAATATAAAG
TGGTAGAAATTAAACCATTG---GGAGTAGCACCCACTAAGGCAAAAAGGAGAGTGGTG-------------
-------------------------GAGAGAGAAAAAAGAGCA---GCA------CTAGGAGCT--------
GTGTTCCTTGGGTTCTTGGGGGCA---GCAGGAAGCACTATG---GGCGCGGCGTCA---
ATAACGCTGACGGTACAG---GCCAGACAG---TTGTTG---TCTGGTATAGTGCAGCAGCAAAGC---
AATTTG---CTAAGA---GCTATAGAGGCGCAACAACATCTGTTGCAACTCACGGTTTGGGGCATT
>KX907413
TTCTATTGCAATACATCAGCACTGTTTAATAGTACATATAATGAAACATATAGATATAATGCTACAAACGAT-
----

ACAAATCCAACCATCACACTCAAATGCAGAATAAAGCAAATTATAAACATGTGGCAGGAGGTAGGACGAGCAA
TGTATGCCCCTCCCATTGCAGGAAACATAACGTGTAGATCAAAT---
ATCACAGGATACTATTGACACGTGATGGAGGACCTAACAGTACT----------------------------
----

AATGAAACATTCAGACCTGAAGGAGGAGATATGAGGAATAATTGGAGAAGTGAACTATATAAATATAAAGTGG
TAGAAATTAAACCATTG---GGAATAGCACCCACCAAGGCAAAAAGGAGAGTGGTG----------------
----------------------GAGAGAGAAAAAAGAGCA---GTCGGA---ATAGGAGCC---------
TTGTTCCTTGGTTTCTTGGGAGCA---GCAGGAAGCACTATG---GGCGCAGCGTCA---
ATAACGCTGACGGTACAG---GCCAGACAA---TTGTTG---TCTGGTATAGTGCAACAGCAAAGC---
AATTTG---CTGAGG---GCTATAGAGGCGCAACAGCATATGTTGCAACTCACTGTCTGGGGCATT
>KX907422
TTCTATTGCAACACATCACAACTGTTTAATAAACTGTTTAATGGTACTAATGATACAGAAAGTACTAAT---
----

TCAACTAAGAATATCACACTCCAATGTAAAATAAAACAAATTATAAATATGTGGCAGGGGGTAGGACGAGCAA
TATATGCTCCTCCCATTGAAGGAAACATCACATGTAAATCAAAC---
ATCACAGGACTAATATTAACACGAGATGGAGGAAGAAATAATAACACT------------------------
----

AACATAACAGAGATATTCAGACCTGGAGGAGGAGATATGAGGGACAATTGGAGAAGTGAATTATATAAATATA
AAGTAGTAGAAATTAAGCCATTA---GGAATAGCACCCACTGGAGCAAAAAGGAGAGTGGTG----------
----------------------------GAGAGAGAAAAAAGAGCA---GTGGGA---ATGGGAGCT------
---GTGCTCCTTGGGTTCTTAGGAGCA---GCAGGAAGCACTATG---GGCGCGGCGTCA---
ATAACGCTGACGGTACAG---GCCAGACAA---CTGCTG---TCTGGTATAGTGCAACAGCAAGAC---
AACTTG---CTGAGG---GCTATAGAGGCGCAACAGCATATGTTGCAACTCACAGTCTGGGGCATT
>KX907338
TTCTATTGCAATACATCAGGTCTGTTTAATGGTACAAATTCAACTAAT------------------------
----

CCAAATGCAACTATCATACTCCCATGCAGAATAAAACAAATTATTAATATGTGGCAGGAAGTAGGACGAGCAA
TATATGCCCCTCCCATTGCAGGAAGCATATCATGCACATCAAAT---
ATTACAGGACTACTATTGACACGTGATGGAGGAAATAACACAAAC---------------------------
----

AATGAGACATTCAGACCCGGAGGAGGAGATATGAGAAATAATTGGAGAAGTGAATTATATAAATATAAAGTGG
TAGAAATTAAACCATTA---GGACTAGCACCCACTAAGGCAAAAAGGAGAGTGGTG----------------
----------------------GAGAGAGAAAAAAGAGGA------------ATAGGAGCT---------
GTACTCCTTGGGTTCTTGGGAGCT---GCAGGAAGCACTATG---GGCGCGGCGTCA---
ATAGCGCTGACGGTACAG---GCCAGACAG---CTATTG---TCTGGTATAGTGCAACAGCAAAGC---
AATTTG---CTGAGG---GCTATAGAGGCGCAACAGCATATGTTGCAACTCACAGTCTGGGGCATT
>KX907363
TTCTATTGCAATACAACAAAACTGTTTACTGGTATTAGATACATTAATGGTACATACATAACTAATGATACAG
AAATAGCT--
----------AATGATACA------
GAGAATAATTCAACCATCACACTCCAATGCAGAATAAAACAGATTATAAACATGTGGCAGGGGGTAGGAAAAG
CAATGTATGCCCCTCCCATTGCAGGAAACATAATATGTAAATCAAAG---
ATCACAGGACTACTATTGACACGTGATGGAGAAGGGAAAATGGAACA-------------------------

----

AATGAGACTGAGACATTCAGACCTGGAGGAGGAGATATGAGGGACAATTGGAGAAGTGAATTGTATAAATATA
AAGTGGTAGAAATTAAGCCATTA---GGAATAGCACCCGCTGAGGCAAAAAGGAGAGTGGTG-----------
----------------------------GAGAGAGAAAAAAGAGCA---GTGGGA---ATGGGAGCT------
---GTGCTCTTTGGGTTCTTGGGAGCA---GCAGGAAGCACTATG---GGCGCAGCATCA---
GTGATGCTGACGGTACAG---GCCAGACAA---TTGTTG---TCTGGTATAGTGCAACAGCAAAGC---
AATTTG---CTGAGA---GCTATAGAGGCGCAACAGCATATGTTGCAACTCACAGTCTGGGGCATT
>KX907374
TTCTATTGCAACACATCAGCACTGTTTAATAAACTGTTTAATAATACTAATGATACAGAAAGTAATACT----
----

TCAGATACAACTATCACACTCCAGTGCAAAATAAAACAAATTATAAACATGTGGCAGGGGGTAGGACGAGCAA
TATATGCTCCTCCCATTGAAGGAATCATCAATTGTACCTCAAAC---
ATCACAGGACTACTGTTGACACGTGATGGTGGGAATAACAGTGATGGGAATAAC-------------------
----

AATAACACAGAAACATTCAGACCTGGAGGAGGAGATATGAGGGACAATTGGAGAAGTGAATTATATAAATATA
AAGTGGTAGAAATTAAACCATTA---GGAATAGCACCCACTAAGGCAAAAAGGAGAGTGGTG-----------
----------------------------GAGAGAGAAAAAAGAGCA---GTGAGC---CTAGGAGCT------
---GTGTTCCTTGGGTTCTTGGGAGCA---GCAGGAAGCACTATG---GGCGCGGCGTCA---
GTGACGCTGACGGTACAG---GCCAGACAA---TTGTTG---TCTGGTATAGTGCAACAGCAAAGC---
AATTTG---CTAAGA---GCTATAGAGGCGCAACAGCATCTGTTGCAACTCACGGTCTGGGGCATT
>KX907380
TTTATTGCAATACATCAGGCCTGTTTAATAATACACTGCTTAATGATACATTGACAATAGAC-----------
----

AATAATGAAACCATCACACTCCCATGCAGAATAAAACAAATTATAAACATGTGGCAGGAGGTAGGACGAGCAA
TGTATGCCCCTCCCATTGCAGGAAACATAACATGTAAATCAAAT---
ATCACAGGACTATTATTGGTACGGGATGGAGGCATATCAAATGGGACAGAT----------------------
----

AACAAAAATGAGACATTCAGACCTGGAGGAGGAGATATGAGGAATAATTGGAGAAGTGAATTATACAAATATA
AAGTGGTAGAAATTAAACCATTG---GGAGTGGCACCCACCAGGGCAAGAAGGAGAGTGGTG-----------
----------------------------GAGAGAGAAAAAAGAGCA---GTGGGA---ATAGGAGCT------
---GTGATCTTTGGGTTCTTAGGAGCA---GCAGGAAGCACTATG---GGCGCAGCGTCA---
ATAGCGCTGACGGTACAG---GCCAGACAA---CTGTTG---TCTGGTATAGTGCAACAGCAGGGC---
AACCTG---CTGAGG---GCTATAGAGGCGCAACAGCATCTGTTGCAACTCACGGTCTGGGGCATT
>KX907416
TTCTATTGCGATACATCAAATCTGTTTAATAGTACATACAGCCCTAACAGTACAGATGATGGAGCGCAAGATG
TA---

AATGACACAATAATCACACTCACATGCAAAATAAAACAAATTGTAAACATGTGGCAGGGAGTAGGAAAAGCAA
TGTATGCCCCTCCCATTAGAGGAAACATAACATGCAGATCAAAT---
ATTACAGGACTACTACTGACAAGGGATGGAGGAGACTCAGAGGGG----------------------------
----

ACAGAAACATTTAGGCCTGGAGGGGGAGATATGAAGGACAATTGGAGAAGTGAATTATATAAATATAAAGTGG
TAGAAGTTAAACCATTA---GGAGTAGCACCAACTACTGCAAAAAGGAGAGTGGTG-----------------
----------------------GAGAGAGAAAAAAGAGCA---GTGGGA---ATAGGAGCT---------
GTGTTCCTTGGGTTCTTGGGAGCA---GCAGGAAGCACTATG---GGCGCAGCGTCA---
ATAACGCTGACGGTACAG---GCCAGACAA---TTATTG---TCTGGTATAGTGCAACAACAAAAC---
AATTTG---CTAAGA---GCTATAGAGGCGCAACAGCATATGTTGCAACTCACGGTCTGGGGCATT
>KX907375
TTCTATTGCAATACAACAAAACTGTTTAATGATAGCATGTCAAATGAAAGCTCAAACATC-------------
----

ACAAACAGCACACTCACACTCCCATGCAGAATAAAACAAATTATAAATATGTGGCAGGAGGTAGGAAGAGCAA
TGTATGCCCCTCCCGTTGAAGGAAATATAACATGTAAATCAAAT---
ATTACAGGACTACTATTGGTACGGGATGGGGGT--

GTGTCAAATGATACAAAT---
GGGACAGAGACATTCAGACCTGGAGGAGGAGATATGAGGGATAATTGGAGAAGTGAATTATATAAATATAAAG
TAGTAGAAATTAAGCCATTG---GGAGTAGCACCAACTGGGGCAAAAAGGAGAGTGGTG--------------
-------------------------GAGAGAGAAAAAAGAGCA---GTGGGA---ATAGGAGCT---------
GTGTTCCTTGGGTTCTTGGGAGCG---GCAGGAAGCACTATG---GGCGCGGCGTCA---
ATAGCGCTGACGGCACAG---GCCAGACAA---GTGTTG---TCTGGTATAGTGCAACAGCAAAAC---
AATTTG---CTGAGA---GCTATAGAGGCGCAACAGCATCTGTTGCAACTCACAGTCTGGGGCATT
>KX907390
TTCTATTGCAATACATCAAAACTGTTTAATACTACATACCTGTATAATGATACAGAAGGTAAC----------
----

TCAACCATCACACTCACACTCCCATGCAGAATAAAACAAATTATAAACTTGTGGCAGGAGGTAGGACGAGCAA
TGTATGCCCCTCCCATTAAAGGAGACATAATATGTAACTCAAGT---
ATCACAGGGCTATTATTGACACGTGATGGAGGAGACAATAGAACA----------------------------
----

AAGGAGACATTCAGACCTGGAGGAGGAGATATGAGGGACAATTGGAGAAGTGAATTATACAAATATAAGGTAA
TACAAATTAAGCCACTA---GGAATAGCACCCACTGCGGCAAGAAGGAGAGTGGTG-----------------
----------------------GAGAGAGAAAAAAGAGCA---ATTGGA---CTAGGAGCC---------
ATGTTCCTTGGGTTCTTGGGAGCA---GCAGGAAGCACTATG---GGCGCGGCGTCA---
ATAACGCTGACGGGACAG---GCCAGACAA---TTGTTG---TCTGGTATAGTGCAACAGCAAAGC---
AATTTG---CTGAGA---GCTATAGAGGCGCAACAGCATATGTTGCAACTCACAGTCTGGGGCATT
>KX907391
TTCTATTGCAATACATCAAACCTATTCAATGGTACCAATTCT-------------------------------
----

TCAGGCAGTAACATCACACTCCCATGTAGAATAAAACAAATTATAAACATGTGGCAGGGAGTAGGAAGAGCAA
TGTATGCTCCTCCCATTGCAGGAGAAATAAAATGTGCATCAAAT---
ATCACAGGGCTAATATTAACATGGGATGGAGGAAAGAACAATAAT----------------------------
----

ACCACAGAGACATTCAGACCTGGAGGAGGGGATATGAGGGATAATTGGAGAAGTGAATTATATAAATATAAAG
TGGTAGAAATTAAGCCATTA---GGAATAGCACCCACTGAGGCAAAAAGGAGAGTGGTG--------------
-------------------------GAGAGAGGAAAAAGAGCA---GTGGGA---ATAGGAGCC---------
GTGTTCCTTGGGTTCTTGGGAGCA---GCAGGAAGCACTATG---GGCGCAGCGTCA---
ATAACGCTGACGGTACAG---GCCAGACAA---CTGTTG---TCTGGTATAGTGCAACAGCAAAGC---
AATTTG---CTGAGG---GCTATAGAGGCGCAACAGCATATGTTGCAACTCACAATCTGGGGCATT
>KX907399
TTCTATTGCGATACAACAAAACTGTTTAATAGTACTAATGGTACTAATAGTTTTAAC----------------
----

TCAAATGAAACTATCATACTCCAATGTAGAATAAAACAAATTATAAATATGTGGCAGGGGGTAGGACGAGCAA
TATATGCTCCTCCCATTGAAGGAAACGTCACATGTACATCAAAC---
ATCACAGGACTACTATTGACACGTGATGGAGGAAATGTAACTGAC----------------------------
----

ACAGAAAAATTCAGACCTGGAGGAGGAGATATGAGGGACAATTGGAGAAGTGAATTATACAAATATAAAGTGG
TAGAAATTAAGCCATTA---GGAATAGCACCCACTGGGGCAAAAAGGAGAGTGGTG-----------------
----------------------GAGAGAGAAAAAAGAGCA---GTGGGA---ATAGGAGCT---------
GTGTTCCTTGGGTTCTTGGGAGCA---GCAGGAAGCACTATG---GGCGCGGCGTCA---
ATAACGCTGACGGTACAG---GCCAGACAA---CTATTG---TCTGGCATAGTGCAACAGCAAAGT---
AATATG---CTGAGA---GCTATAGAGGCGCAACAGCATATGTTGCAACTTACAGTCTGGGGCATA
>KC156220
TTCTATTGTAATACATCAAAACTGTTTAATAATAATATAACAAGCGAT-------------------------
----

AATTCAAGCAACATCACAATCCCATGCAAAATAAAACAAATTATAAACATGTGGCAGAAGGTAGGAAAAGCAA
TGTATGCCCCTCCCATTGCAGGGAACATAACATGTACCTCGAAT---
ATCACAGGAATACTATTAACACGTGATGGAGGAAATAATAGTGAC----------------------------

----

AGTGATCCAGAGACATTCAGACCTACAGGAGGAGATATGAGGGACAATTGGAGAAGTGAATTATATAAATATA
AAGTAGTAGAAGTAGAACCATTA---GGAATAGCACCCACTGGAGCAAAAAGGAGAGTGGTG-----------
-----------------------------GAGAGACAGAAAAGAGCA---GTGGGA---ATGGGAGCT------
---ATGTTCCTTGGGTTCTTGGGAGCA---GCAGGAAGCACTATG---GGCGCAGCGTCA---
ATAACGCTGACGGTACAG---GCCAGACAA---TTATTG---TCTGGTATAGTGCAACAGCAAAGC---
AATTTG---CTGAGG---GCTATAGAGGCGCAACAGCATATATTGCAACTCACAGTCTGGGGCATT
>AF361874
TTCTATTGCGATACATCAAGGCTATTTAATGAAAGTCTTAATGAAAGACTGTTTAATGAAAGTAGTGAG----
----

TCAAATGACACCATCACACTCCAATGTAGAATAAAACAAATTATAAACATGTGGCAGAAGGTAGGACAAGCAA
TATATGCCCCTCCCATAAAAGGAAACATATCATGTACATCAAAC---
ATCACAGGACTACTATTGACACGTGATGGAGGAAATGATGATGGAGGAAATGAT--------------------
----

ACAGGGTCAAGGCCAGAGACATTCAGACCTGGAGGAGGAGATATGAGGGACAATTGGAGAAGTGAATTATATA
AATATAAAGTGGTAGAAATTAAGCCATTA---GGAATAGCACCCACTGCGGCAAAAAGAAGAGTGGTG-----
-----------------------------------GAGAGAGAAAAAAGAGCA---GTGGGA---
ATAGGAGCT---------GTGTTCCTTGGGTTCTTGGGAGCA---GCAGGAAGCACTATG---
GGCGCGGCGTCA---ATAACGCTGACGGTACAG---GCCAGACAA---CTATTG---
TCTGGTATAGTGCAACAGCAAAGC---AATTTG---CTGAGA---
GCTATAGAGGCGCAACAGCATATGTTGCAACTCACGGTCTGGGGCATT
>AF286234
TTCTATTGCAATACATCAAATCTGTTTACATCAAAACTGTTTAATAGTACTAATACTACAGAGAATGCCACAC
AA--

AGAAACGAAACCCTCACACTCCAATGCAGAATAAAACAGATTATAAACATGTGGCAGGAGGTAGGACGAGCAA
TATATGCCCCTCCCATTGCAGGGAGCATAACATGTACATCAAAT---
ATCACAGGAATACTATTGACACGAGATGGAGAAAATGCAGGAAATAAC--------------------------
----

ACAGGGAATGAGACATTCAGACCTGGAGGAAGAAATATGAGGGATAATTGGAGAAGTGAATTATATAAATATA
AAGTGGTAGAAATTAAGCCATTG---GGAATAGCACCCACTGAGGCAAAAAGGAGAGTGGTG-----------
-----------------------------GAGAGAGAAAAAAGAGCA---GTGGGA---CTAGGAGCT------
---GTGTTCCTTGGGTTCTTGGGAGCA---GCCGGAAGCACTATG---GGCGCGGCGTCA---
ATAACGCTGACGGTACAG---GCCAGACAA---TTATTG---TCTGGTATAGTGCAACAGCAAAGC---
AATCTG---CTGAGG---GCTATAGAGGCGCAACAGCATCTGTTGCAACTCACGGTCTGGGGCATT
>AF286235
TTCTATTGCAATACATCAGGCCTGTTTAATGAAAGTCTGGTTAAAAGTAATAAT--------------------
----

TCAAATGGAACTATCGAACTCCAATGTAGAATAAAACAAATTATAAACATGTGGCAGGAGGTAGGACGAGCAA
TATATGCCCCTCCCATTAGAGGAAACATAACATGTATATCAAAC---
ATCACAGGACTATTATTGACACGTGATGGAGGAAATAATGGCACT-----------------------------
----

AACGCAACGGAGATATTCAGACCTGGAGGAGGGGATATGAGGGACAATTGGAGAAGTGAATTATACAAATATA
AAGTGGTAGAAATTAAACCATTA---GGAATAGCACCCACTGGGGCAAAAAGAAGAGTGGTG-----------
-----------------------------GAGAGAGAAAAAAGAGCA---GTGGGA---ATGGGAGCT------
---GTGCTCCTTGGGTTCTTGGGAGCG---GCAGGAAGCACTATG---GGCGCGGCGTCG---
ATGGCGCTGACGGTACAG---GCCAGACAA---CTATTG---TCTGGTATAGTGCAACAGCAAAGC---
AATTTG---CTGAGG---GCTATAGAGGCGCAACAGCATATGTTGCAACTCACGGTCTGGGGCATT
>L22948
TTCTATTGCAATACATCAAGCCTGTTTAATAGTAGTGATAATAAT-----------------------------
----

AATAGTACCATCATCACACTCCCATGCAGAATAAAACAAATTATAAACATGTGGCAGGGGGTAGGACGAGCAA
TGTATGCCCCTCCCATTAAAGGAAAAATAACATGTAGATCAAAT---

ATCACAGGACTACTACTGACACGTGATGGAGGGGAGACAAGTGAGACA-------------------------
----

AATAGCACAGAGACATTCAGACCTGGAGGAGGAGATATGAGGGACAATTGGAGAAGTGAATTATATAAATATA
AAGTGGTAGAAGTTAAGCCACTA---GGAGTAGCACCCACTAAGGCAAAAAGGAGAGTGGTG-----------
----------------------------GAGAGAGAAAAAAGAGCA---GTGGGA---ATAGGAGCT------
---GTGTTCCTTGGGTTCTTGGGAGCC---GCAGGAAGCACTATG---GGCGCAGCATCA---
ATAACGCTGACGGTACAG---GCCAGACAA---TTATTG---TCTGGTATAGTGCAACAGCAAAAC---
AATTTG---CTGAGG---GCTATAGAGGCGCAACAGCATATGTTGCAACTCACGGTCTGGGGCATT
>EU663619
TTCTATTGCGATACATCAGGACTGTTCAATAGGACATACTTGCTAGCAAATGTTACAGGAAATATTTCAGACA
CAGACCGC---
-------------------AAC---
GTCACAGACCGCAACATCACACTTTCATGCAGAATAAAACAAATTATAAACACGTGGCAGGAGGTAGGAAAAG
CAATGTATGCTCCTCCCATTGCAGGAAACATAACATGTACATCAAAC---
ATCACAGGACTACTATTGACACGTGATGGAGGTATAAGTAATGACACC-------------------------
----

AATAACACAGAGACATTCAGACCTGGAGGAGGAGATATGAAGAACAATTGGAGAAGTGAATTATATAAATATA
AAGTGGTAGAAATTAAGCCATTA---GGATTAGCACCCACTGCAGCAAAAAGGAGAGTGGTG-----------
----------------------------GAGAGAGAAAAAAGAGCA---GTAGGA---ATAGGAGCT------
---GTACTCCTTGGGTTCTTGGGAGCA---GCAGGAAGCACTATG---GGCGCGGCGTCA---
ATAACGCTGACGGTACAA---GCCAGACAA---GTATTG---TCTGATATAGTGCAACAGCAAAGC---
AATTTG---CTGAAG---GCTATAGATGCGCAACAGCATATGTTGCAACTCACAGTCTGGGGCATT
>AY444800
TTCTATTGCAATACATCAGGGCTGTTTAATGGTACGTATAATGATACAAGGGGTGAG----------------
----

CCAAATGGAACCATCACACTCCCATGCAGAATAAAACAAATTATAGACATGTGGCAGAAGGTAGGACGAGCAA
TGTATGCTCCTCCCATTGAAGGAAATATAACATGTAAATCAAAT---
ATCACAGGACTATTATTGACACGTGATGGAGGCCTGTTAGATAAT----------------------------
----

AGTAGCACAGAGACATTCAGACCTGCAGGAGGAGATATGAGGGACAATTGGAGAAGTGAATTATATAAATATA
AAGTGGTGGAAATTAAGCCATTA---GGAGTAGCACCCACTGCTGCAAAAAGGAGAGTGGTG-----------
----------------------------GAGAGAGAAAAAAGAGCA---GTGGGA---ATAGGAGCT------
---GTGTTCCTTGGGTTCTTGGGAGCA---GCAGGAAGCACTATG---GGCGCGGCGTCA---
ATAACGCTGACGGTACAG---GCCAAACAA---TTGTTG---TCTGGTATAGTGCAGCAGCAAAGC---
AATTTG---CTGAGA---GCTATAGAGGCGCAACAGCATATGTTGCAACTCACGGTCTGGGGCATT
>AY444801
TTCTATTGCAATACATCAGTCCTGTTTAATAGGTCATATAATCTTACTGATACAAATAGTAATAATATAAATA
GC---

ACTTCAACCGACACACTCACACTCCCATGCAGAATAAAACAAATTATAAACATGTGGCAGGAGGTAGGACGGG
CAATGTATGCCCCTCCCATTGCAGGAAACATAACATGTAACTCAAGC---
ATCACAGGACTACTATTGACATGGGATGGAGGCGAAGAGAAAAACAACACA----------------------
----

GAGAAAAACAACACAGAGATATTCAGACCTGGAGGAGGAGATATGAGGGACAATTGGAGAAGTGAACTATATA
AATATAAAGTGGTAGAAATTAAGCCATTA---GGAATAGCACCCACCAAGGCAAAAAGGAGAGTGGTG-----
----------------------------------GAGAGAGAAAAAAGAGCA---GTGGGA---
ATAGGAGCT---------GTGCTCCTTGGGTTCTTGGGAGCA---GCAGGAAGCACTATG---
GGCGCGGCGTCA---ATAACGCTGACGGCACAG---GCCAGACAA---GTGTTG---
TCTGGTATAGTGCAACAGCAAAGT---AATTTG---CTGAGA---
GCTATAGAGGCGCAACAGCAAATGTTGCAACTCACAGTCTGGGGCATT
>AY563169
TTCTATTGCAATACATCAAGCCTGTTTAATAGTACATACAATACAACAGGGACAGAAAGCAGTAATGGGACAG
AAAGC--
-------------------AGT---
AATGAAACAGGAAGCATTACAATCCCATGCAGAATAAAACAAGTTATAAACATGTGGCAAGGGGTAGGACGAG

CAATGTATGCCCCTCCCATTGAAGGAAACATAACATGTAGGTCAAAT---
ATCACAGGATTACTACTGACACGGGATGGAGGAGACGGGAATGGC----------------------------
----

ACAACAGAGACATTCAGACCTGGAGGAGGAGATATGAGGGACAATTGGAGAAGTGAATTATACAAATATAAAG
TGGTAGAAATTAAACCATTA---GGAATAGCACCCACTACTGCAAAAAGGAGAGTGGTG--------------
-------------------------GAGAGAGAAAAAAGAGCA---GCG------CTAGGAGCT---------
ATGTTCCTTGGGTTCTTGGGAGCA---GCAGGAAGCACTATG---GGCGCAGCGTCA---
ATAACGCTGACGGTACAG---GCCAGACAA---TTATTG---TCTGGGATAGTGCAACAGCAAAGC---
AATTTG---CTGAAG---GCTATAGAGGCGCAACAGCATATGTTGCAACTCACGGTCTGGGGCATT
>AY795906
TTCTATTGCAACACATCAAAATTGTTTAATAGGACATACATGTTTAATAGTACATACATGCCTAATAGTACTA
ATAGT--

ACTTCAAATTCAACCATCACACTCCCTTGCAGAATAAAACAAATTATAAACATGTGGCAGGGGGTAGGACGAG
CAATGTATGCCCCTCCTATTGCAGGAAACATAACATGTACATCAAAC---
ATCACAGGACTACTATTGACACGTGATGGAGGAGGAAAAGACAAC----------------------------
----

GAGTCAGAGACATTCAGACCTGGAGGAGGAGATATGAGGGATAATTGGAGAAGTGAATTATATAAATATAAAG
TGGTAGAAATTAAACCATTA---GGAATAGCACCCACTGCAGCAAAGAGGAGAGTGGTG--------------
-------------------------GAGAGAGAAAAAAGAGCA---GCAGGA---ATGGGAGCT---------
TTGTTCCTTGGGTTCTTGTCAGCA---GCAGGAAGCACTATG---GGCGCAGCATCA---
ATAACGCTGACGGTACAG---GCCAGGCAA---TTGTTG---TCTGGTATAGTGCAACAGCAAAGC---
AATTTG---CTGAGG---GCTATAGAGGCGCAACAGCATCTGTTGCAACTCACGGTCTGGGGCATT
>AY529667
TTCTATTGCAATACATCAAACCTGTTTAATGAGAGTAATATAGAAAGG-------------------------
----

AATGATTCAATCATCACACTCCCATGCAGAATAAAACAAATTATAAACATGTGGCAGGAGGTAGGACGAGCAA
TATATGCTCCTCCCATTGCAGGAAACATAACATGTAGATCAAAT---
ATCACAGGACTACTATTGACACGTGATGGAGGGTCAAATAATGGGGTG-------------------------
----

CCAAATGACACAGAGACATTCAGACCTGGAGGAGGCGATATGAGGAACAATTGGAGAAGTGAATTATATAAAT
ATAAAGTGGTAGAAGTTAAACCATTG---GGAGTAGCACCCACTGAGGCAAAAAGGCGAGTGGTG--------
-------------------------------GAGAGAGAAAAAAGAGCA---GTAGGA---ATAGGAGCT---
------GTGTTCCTTGGGATCTTGGGAGCA---GCCGGAAGCACTATG---GGCGCAGCATCA---
ATAACGCTGACGGTACAG---GCCAGACAA---TTGCTG---TCTGGTATAGTGCAACAGCAAAGC---
AATTTG---CTGAGG---GCTATAGAGGCGCAACAGCATATGTTGCAACTCACAGTCTGGGGCATT
>AY463217
TTCTATTGCAATACAACACAACTGTTTGAAAATATTACTAAT-------------------------------
----

AATGTAAGTAACATCATACTCCCATGCAGAATAAAACAATTTATAAACATGTGGCAGGAGGTAGGACGAGCAA
TGTATGCCCCTCCCATTGCAGGAAACATAACATGTACCTCAAAT---
ATCACAGGACTGCTATTGGTACGTGATGGTGGAAACACAAATGGCACA-------------------------
----

AATAACTCAGAAATATTCAGACCTGCAGGAGGAAATATGAAGGACAATTGGAGAAGTGAATTATATAAATATA
AAGTGATAGAACTTAAGCCATTG---GGAGTAGCACCCACTGAGGCAAGAAGGAGAGTGGTG-----------
----------------------------GAGAGAGAAAAAAGAGCA---GTGGGA---ATAGGAGCT------
---GTGTTCCTTGGGTTCTTGGGAGCA---GCAGGAAGCACTATG---GGCGCGGCGTCA---
ATAACGCTGACGGTACAG---GCCAGACAA---CTGTTG---TCTGGTATAGTGCAACAGCAAAGC---
AATTTG---CTGAGA---GCTATAGAGGCGCAACAGCATATGTTGCAACTCACGGTCTGGGGCATT
>AY463230
TTCTATTGCAATACATCACAACTGTTTAATGGCACATACAATGGCACATATAGTAAT----------------
----

ATCACAGACCTCATCACCCTCCCATGCAAGATAAAACAAATTATAAACCTGTGGCAGGGGGTAGGACGAGCAA

TGTATGCCCCTCCCATTGCAGGAAACATAACATGTAACTCAAAT---
ATCACAGGAATACTATTGACACGTGATGGAGGACACGAAAGGAATAACACA----------------------
----

AAGAATGACACAGAGATATTCAGACCTGGAGGAGGAAATATGAAGGACAATTGGAGAAGTGCATTATATAAAT
ATAAAGTGGTAGAAGTTAAGCCATTA---GGAATAGCACCAACTGAGGCAAAAAGGAGAGTGGTG--------
-------------------------------GAGAGAGAAAAAAGAGCA---GTGGGA---ATGGGAGCT---
------GTACTCCTTGGGTTCTTGGGAGCA---GCAGGAAGCACTATG---GGCGCAGCGTCA---
ATAACGCTGACGGTACAG---GCCAGGCAA---CTGTTG---TCTGGTATAGTGCAACAGCAAAAT---
AATTTG---CTGAGA---GCTATAGAGGCGCAACAGCATATGTTGCAACTCACGGTCTGGGGCATT
>AY463231
TTCTATTGTAATACATCAAACCTGTTTAATGATAATGGTACAGAAAGT-------------------------
----

AATTCAACACACATTACACTTCCATGCAGAATAAAACAAATTATAAACATGTGGCAGGGGGTAGGGCGAGCAA
TGTATGCCCCTCCCATTGAAGGAAATATAACATGTAACTCAAGT---
ATCACAGGACTACTACTGACACGTGATGGAGGAGACATAATAAATGGCACA----------------------
----

GACAATGAAACAGAGACATTCAGACCTGGAGGAGGAAATATGAAGGACAATTGGAGAAGTGAATTATATAAAT
ATAAAGTGGTAGAAATTAAGCCATTA---GGGATAGCACCCACTAAGGCAAAAAGGAGAGTGGTG--------
-------------------------------GAGAGAGAAAAAAGAGCA---GTAGGA---TTAGGAGCT---
------GTGATCTTTGGGTTCTTGGGAGCA---GCAGGAAGCACTATG---GGCGCGGCGTCA---
ATAACGCTGACGGTACAG---GCCAGACAA---CTGTTG---TCTGGTATAGTGCAACAGCAAAGC---
AATTTG---CTGAGA---GCTATAGAGGCGCAACAGCATATGTTGCAACTCACGGTCTGGGGCATT
>AY463225
TTCTATTGCAATACGTCAGACTTGTTTAATAGTAATAATAAGACAGAA-------------------------
----

AGCAATTCAACTATCACACTTCCATGCAGAATAAAACAAATTATAAACATGTGGCAGGGGGTAGGACGAACAA
TGTATGCCCCTCCCATTGCAGGAAACATAACATGTAACTCAAAT---
ATCACAGGACTACTATTGACACGTGATGGAGGGAAAAATGACACA----------------------------
----

GGGGAGGAGATATTCAGACCTGGAGGAGGAAATATGAAGGACAATTGGAGAAATGAATTATATAAATATAAAG
TGGTAGAAATTAAGCCATTG---GGAGTAGCACCCACTGAGGCAAAAAGGAGAGTGGTG--------------
-------------------------GAGAGAGAAAAAAGAGCA---GTGGGA---ATAGGAGCT---------
GTGATCCTTGGGTTCTTGGGAGCA---GCAGGAAGCACTATG---GGCGCGGCGTCA---
ATAACGCTGACGGTACAG---GCCAGACAA---CTGTTG---TCTGGTATAGTGCAACAGCAAAAT---
AATTTG---CTGAGA---GCTATAGAGGCGCAACAGCATATGTTGCAACTCACGGTCTGGGGCATT
>AY463232
TTCTATTGCAATACATCAAACCTGTTTAATAGTACATTGCTTAATAGTACATTGTCTAATAGTACAAATAAT-
----

ACTAATGGTATCATCATACTCCCATGCAGAATAAAACAAATTATAAATATGTGGCAGGGGGTAGGACGGGCAA
TGTATGCCCCTCCCATTGCAGGAAATATAACATGTAAATCAAAT---
ATCACAGGACTAATATTGACACGTGATGGAGGAGAACCTAATATA----------------------------
----

ACGGAGGAGACATTCAGACCTGGAGGAGGAGATATGAGAGACAATTGGAGAAGTGAATTATATAAATACAAAG
TGGTAGAAATTAATCCATTA---GGAGTAGCTCCCACTAGAGCAAAAAGGAGAGTGGTG--------------
-------------------------GAGAGAGAGAAAAGAGCA---GTGGGA---ATAGGAGCT---------
GTATTCCTTGGGTTCTTAGGAGCA---GCAGGAAGCACTATG---GGCGCAGCATCA---
ATAACGCTGACGGTACAG---GCCAGACAA---TTATTG---TCTGGTATAGTGCAACAGCAAAGC---
AATTTG---CTGAGG---GCTATAGAGGCGCAACAGCATATGTTGCAACTCACAGTCTGGGGCATT
>AY463218
TTCTATTGCAATACAACAAAACTGTTCACTAGTACTAATATG-------------------------------
----

ACAAACACAACCATCATGATCCCATGCAGAATAAGGCAAATCGTACGCCTGTGGCAGGAGGTAGGACGAGCAA

TGTATGCCCCTCCCATTGAAGGAAACATAACATGTAGCTCAAAT---
ATCACGGGACTACTATTGGTACGTGATGGAGGAAAAGGAAACGAAACA-------------------------
----

CAGAATAACACAGAAATATTCAGACCTGGAGGAGGAAATATGAAGGACAATTGGAGAAGTGAATTATATAAAT
ATAAGGTGGTAGAGCTTAAGCCATTG---GGAATAGCACCCACTGATGCAAAAAGGAGAGTGGTG--------
-------------------------------GAGAGAGAAAAAAGAGCA---GTGGGAGTAATAGGAGCT---
------GTGCTCCTTGGGTTCTTGGGAGCA---GCAGGAAGCACTATG---GGCGCGGCGTCA---
ATAACGCTGACGGTACAG---GCCAGACAA---CTGTTG---TCTGGTATAGTGCAACAGCAAAGC---
AATTTG---CTGAGA---GCTATTGAGGCGCAACAGCATTTGTTGCAACTCACGGTCTGGGGCATT
>AY463233
TTCTATTGCAATACATCAAAACTGTTTAATAGTACATACAATAGTACATACAATAGTACATACAATAGTAGTA
CA---

GGAAATTCAACCATTACACTCCAATGCAGAATAAAACAAATTATAAATATGTGGCAGGGGGTAGGACGAGCAA
TGTATGCCCCTCCCATTGCAGGAAATATAACATGTAAATCAAAT---
ATCACAGGACTACTATTGACAAGCGATGGAGGGGGGAATAATGAA----------------------------
----

ACAGAGACATTTAGACCTGGAGGAGGAAATATGAGGGACAATTGGAGAAGTGAATTGTATAAATATAAAGTGG
TAGAAATTAAGCCATTA---GGAGTAGCACCCACTGAGGCAAAAAGGAGAGTGGTG-----------------
----------------------GAGAGAGAAAAAAGAGCA---GTGGGA---ATAGGAGCT---------
GTACTCCTTGGATTCTTGGGAGCC---GCAGGAAGCACTATG---GGCGCAGCATCA---
ATAACGCTGACGGTACAG---GCCAGACAA---TTGTTG---TCTGGTATAGTGCAACAGCAAAGC---
AATTTG---CTGAGG---GCTATAGAGGCGCAACAGCATATGTTGCAACTCACGGTCTGGGGCATT
>AY463219
TTCTATTGCAATACAACAGGACTGTTTAATAACAATGCAGCT-------------------------------
----

AACACAACAACCATCAACATCCCATGCAGAATAAAACAAATTGTAAACATGTGGCAAGAGGTAGGACGAGCAA
TGTATGCCCCTCCTATTGCAGGAAACATAACATGTAAATCAAAC---
ATCACAGGATTACTATTGCTACGTGATGGAGGAAACAATAGTAAC----------------------------
----

ACAGAGATATTTAGACCTGGAGGAGGAAATACGAAGGACAATTGGAGAAGTGAATTATATAAATACAAAGTAG
TAGAAATTAAGCCATTG---GGAGTAGCACCCACTAAGGCAAAAAGGAGAGTGGTG-----------------
----------------------GAGAGAGAAAAAAGAGCA---GTGGGA---ATAGGAGCT---------
GTGCTCCTTGGGTTCTTGGGAGCA---GCAGGAAGCACTATG---GGCGCGGCGTCA---
ATAACGCTGACGGTACAG---GCCAGACAA---TTGTTG---TCTGGTATAGTGCAACAGCAAAGC---
AATTTG---CTGAGA---GCTATAGAGGCGCAACAGCATATGTTGCAACTCACTGTCTGGGGCATT
>AY463220
TTCTATTGCAATACATCAAAGCTGTTTAATATATCTACTAGTGAAAATGTAACT-------------------
----

AGTGAAAATGTAACTACACTCCCATGCAGAATAAAACAAATTATAAGGATGTGGCAGGGAGTAGGACAAGCAA
TGTATGCCCCTCCCATTGAAGGAAACATAACATGTAACTCAAGT---
ATCACAGGACTACTACTGACACGGGATGGAGGAAACAACACGACT----------------------------
----

AATGGCACGGAGATATTCAGACCTGGAGGAGGAAATATGAAAGACAATTGGAGAAGTGAATTATATAAATATA
AAGTGGTAGAAATTAAGCCATTG---GGAGTAGCACCCACAAAGGCAAAAAGGAGAGTGGTG-----------
----------------------------GAGAAAGAAAAAAGAGCA---GTGGGA---ATAGGAGCT------
---GTGTTCCTCGGGTTCTTGGGAGCA---GCAGGAAGCACTATG---GGCGCGGCGTCA---
ATAACGCTGACGGTACAG---GCCAGACAA---CTGTTG---TTTGGTATAGTGCAACAGCAAAGC---
AATTTG---CTGAGA---GCTATAGAGGCGCAACAGCATATGTTGCAACTCACAGTCTGGGGCATT
>AY463234
TTCTATTGCAATACAACAGAACTGTTTAATGATAATAACAGTGAT----------------------------
----

GACAACGCAACCATCACACTCCCATGCAGAATAAAACAAATCATAAACATGTGGCAAGGGGTAGGACGAGCAA

TGTATGCCCCTCCCATTGAAGGAAGCATAACATGTAACTCAAAT---
ATCACAGGACTACTATTGACACGTGATGGAGGAACAAACAACACA----------------------------
----

GATGAGACATTCAGACCTGGAGGAGGAAATATGAAGGACAATTGGAGAAGTGAATTATATAAATATAAAGTGG
TAGAAATTAAACCATTG---GGAATAGCACCCACAGGGGCAAAAAGGAGAGTGGTG-----------------
----------------------GAGAGAGAAAAAAGAGCA---GTGGGA---CTAGGAGCT---------
GTGTTCCTTGGGTTCTTGGGAGCA---GCAGGAAGCACTATG---GGCGCGGCGTCA---
ATAACGCTGACGGTACAG---GCCAGACAA---CTGTTG---TCTGGTATAGTGCAACAGCAAAGC---
AATTTG---CTGAGA---GCTATAGAGGCGCAACAGCATATGTTGCAACTCACGGTCTGGGGCATT
>AY463221
TTCTATTGCAATACAACAAACCTGTTTAATGAAAGTCTGGTTAATACA-------------------------
----

AGCACAGGAGACATCACGCTCCCATGCAAAATAAAACAAATTATAAACATGTGGCAGGAAGTAGGGCGAGCAA
TGTATGCCCCTCCCATTGAAGGAAGCATAACATGTAAATCAAAT---
ATCACAGGACTACTATTGCTACGTGATGGAGGAAAGAATAACACA----------------------------
----

AATGAGACATTCAGGCCTGGAGGAGGAAATATGAAAGACAATTGGAGAAGTGAATTATATAAATATAAAGTGG
TAGGACTTAAGCCATTG---GGAATAGCACCCACTGATTCAAAAAGGAGAGTGGTG-----------------
----------------------GAGAGAGAAAAAAGAGCA---GTGGGA---ATAGGAGCT---------
ATGTTCCTTGGGTTCTTGGGAGCA---GCAGGAAGCACTATG---GGCGCGGCGTCG---
ATAACGCTGACGGTACAG---GCCAGACAA---CTGCTG---TCTGGTATAGTGCAACAGCAAAGC---
AATTTG---CTGAGA---GCTATAGAGGCGCAACAGCATTTGTTGCAACTCACGGTCTGGGGCATT
>AY463236
TTCTATTGCGATACAGCAGCCCTGTTTAATGGCACATATAATGGCACATATGGTAAT----------------
----

GAGACTTCAAACATCACACTCCAATGCAGAATAAAACAGATGATAAACATGTGGCAGGGGGTAGGACGAGCAA
TGTATGCTCCTCCCATTGCAGGAAACATAACATGTAGATCGAAT---
ATCACAGGACTACTATTGACACGTGATGGAAGTAACGATACTGGG----------------------------
----

CCAGAGATATTCAGACCTGGAGGAGGAAATATGAAGGACAATTGGAGAAGTGAATTATATAAATACAAAGTGG
TAGAAATTAAGCCATTA---GGAATAGCACCCACTAAAGCAAAAAGGAGAGTGGTG-----------------
----------------------GAGAGAGAGAAAAGAGCA---GTGGGA---ATAGGAGCT---------
GTGTTCCTTGGGTTCTTAGGAGCA---GCAGGAAGCACTATG---GGCGCAGCATCA---
ATAACGCTGACGGTACAG---GCCAGACAA---TTATTG---TCTGGTATAGTGCAACAGCAAAGC---
AATTTG---CTGAGA---GCTATAGAGGCGCAACAGCATATGTTGCAACTCACAGTCTGGGGCATT
>AY463226
TTCTATTGCAATACCACAAACCTGTTTGCCACAGAACTGGGTTCTAATAGAAGTGAGTCAAACATCACA----
----

AAGGAGTCAAACATCACACTCCCATGTAGGATAAAACAAATTATAAACATGTGGCAGGGGGTAGGACGAGCAA
TGTATGCCCCTCCCATTGAAGGAAACATAATGTGTAACTCAAGT---
ATCACAGGAATACTATTAACACGTGATGGAGGAGGAGGAGGAGAC----------------------------
----

AATCTCACAGAGATATTCAGACCTGGAGGAGGAAATATGAAGGACAATTGGAGAAATGAATTATATAAATATA
AAGTGGTAGAAATTAAGCCATTG---GGAGTGGCACCCACTGAATCAAAAAGGAGAGTGGTG-----------
----------------------------GAGAGAGAAAAAAGAGCA---GTGGGA---ATAGGAGCT------
---GTACTCCTTGGGTTCTTGGGAGCA---GCAGGAAGCACTATG---GGCGCGGCGTCA---
ATAACGCTGACGGTACAG---GCCAGACAA---TTGTTG---TCTGGTATAGTGCAACAGCAAAGC---
AATTTG---CTGAGA---GTCATAGAGGCGCAACAGCATATGTTGCAACTCACGGTCTGGGGCATT
>AY463222
TTCTATTGCAATACAACAATACTGTTTAATGAAACAAAACCT-------------------------------
----

TGTGAAAACACAACCATCCCATGCAGAATAAAACAAATTATAAACATATGGCAGGAGGTAGGAAGAGCAATGT

ATGCCCCTCCCATTGCAGGACACATAACATGTAACTCAACT---
ATCACAGGACTACTATTGTCCCGCGATGGAGGAGCCACAAACGAT----------------------------
----

ACCGAGATATTCAGGCCTGGAGGAGGAAATATGAAGGATAATTGGAGAAGTGAATTATATAAGTATAAAGTGG
TAGAAATTAAGCCATTA---GGAATAGCACCCACTACGGCAAAGAGGAGAGTGGTG-----------------
----------------------GAGAGAGAAAAAAGAGCA---GTGGGA---ATAGGAGCT---------
GTGTTCCTTGGGTTCTTGGGAGCA---GCAGGAAGCACTATG---GGCGCGGCGTCA---
ATAACGCTGACGGTACAG---GCCAGACAA---CTGCTG---TCTGGTATAGTGCAACAGCAAAGC---
AATTTG---CTGAGG---GCTATAGAGGCGCAACAGCATATGTTGCAACTCACGGTCTGGGGCATT
>JX845606
TTCTATTGCAATACAACAAACCTGTTTAACGAAAATATAACACAACTGTTTAATAAAAGTGAA----------
----

GCAAACGGAACCATCACACTCCCATGCAGGATAAAACAAATTATAAACATGTGGCAGAAGGTAGGACGAGCAA
TGTATGCCCCTCCTATTGCAGGAAATATAACATGTAAATCAAAT---
ATCACAGGACTACTATTGACACGCGATGGAGAAGGATACAACACA----------------------------
----

CCGAATGAGACATTCAGACCTACAGGAGGAAATATGAAAGATAATTGGAGAAGTGAATTATATAAATATAAAG
TGGTAGAAATTAAGCCATTA---GGAATAGCACCCACTGAGTCAAAAAGGAGAGTGGTG--------------
-------------------------GAGAGAGAAAAAAGAGCA---GTGGGA---CTAGGAGCT---------
GTACTCCTTGGGTTCTTGGGAGCA---GCAGGAAGCACTATG---GGCGCGGCGTCA---
ATAACGCTGACGGTACAG---GCCAGACAA---CTGTTG---TCTGGTATAGTGCAACAGCAAAGC---
AATTTG---CTGAGA---GCTATAGAGGCGCAACAGCATATGTTGCAACTCACAGTCTGGGGCATT
>KF146947
TTCTATTGCAATACATCAAAACTGTTTAATAGTACATTGAATAGTACATATATGCCTAATGGTACAGAA----
----

AGTAATTCAAACATCACACTCCCATGCAGAATAAGACAATTTGTAAACATGTGGCAGGAAGTAGGACGAGCAA
TGTATGCCCCTCCCATTGAAGGAAAGATAACATGTAACTCAAAT---
ATTACAGGACTACTATTGTTACGTGATGGAGGAATAAATAATGACAGC-------------------------
----

AGTGAGAATGAGACATTCAGACCGGGAGGAGGAGATATGAGGGACAATTGGAGAAGTGAATTATATAAATATA
AAGTGATAGAAATTAAGCCATTG---GGAATAGCACCCACTGGAGCAAAAAGGAGAGTGGTG-----------
----------------------------GAGAAAGAAAAAAGAGCA---GTGGGA---ATAGCAGCT------
---GTGTTCCTTGGGTTCTTGGGAGCA---GCAGGAAGCACTATG---GGCGCGGCGTCA---
TTAACGCTGACGGTACAG---GCCAGACAA---TTGTTG---TCTGGTATAGTGCAGCAGCAAAGC---
AATTTG---CTGAGG---GCTATAGAGGCGCAACAGCATATGTTGCAACTCACGGTCTGGGGCATT
>AY838568
TTCTATTGCAATACATCAAAACTATTTAAATTTAATAGTTCGGAACCATTTAATAGTACAGAAAGTAATAGTA
CA---

GAAAGTAATAAAAATATCACAATTCCATGCAGAATAAAACAAATTATAAACATGTGGCAGGAGGTAGGACGAG
CAATGTATGCCCCTCCCATTGCAGGGAACATAACATGTACATCAAAC---
ATCACAGGACTACTATTAGTACGTGATGGGGGCAAAGAAAATGACCAA-------------------------
----

AATAACACAGAAATATTCAGACCTGGAGGAGGAAATATGAAGGACAATTGGAGAAGTGAATTATATAAATATA
AAGTGGTAGAAATTAAGCCATTA---GGAATAGCACCCACTGGAGCAAAAAGGAGAGTGGTG-----------
----------------------------CAGAGAGAGAAAAGAGCA---GTGGGA---ATAGGAGCT------
---ATACTCCTTGGGTTCTTGGGAGCA---GCAGGAAGCACTATG---GGCGCAGCGTCA---
ATAGCGCTGACGGCACAG---GCCAGACAA---GTGTTG---TCTGGTATAGTGCAACAGCAAAAC---
AATCTG---CTGAGA---GCTATAGAGGCGCAACAGCATATGTTGCAACTCACGGTCTGGGGCATT
>AY463228
TTCTATTGCAATACAACAAAATTGTTCCTGTTTAATGAAAGTGATGGAAGTGCT-------------------
----

CTAAACAAAACCATCACACTCCCATGCAGAATAAAACAGATTATAAACATGTGGCAGGAGGTAGGACGAGCAA

```
TGTATGCCCCTCCCATTGCAGGAAACATAACATGTAACTCAAGT---
ACCACAGGATTACTATTGCTACGTGATGGCGGAAACCAAAACAAT----------------------------
-------------------------------------------------------------------------
-------------
GAGACAGAGATATTCAGACCTGGAGGAGGAAATATGAAGGACAATTGGAGAAGTGAATTATATAAATATAAAG
TGGTAGAACTTAAGCCATTA---GGAGTAGCACCCACTGAGGCAAAAAGGAGAGTGGTG--------------
-------------------------GAGAGAGAAAAAAGAGCA---GTGGGA---ATAGGAGCT---------
GTGTTCCTTGGGTTCTTGGGAGCA---GCAGGAAGCACTATG---GGCGCGGCGTCA---
ATAACGCTGACGGTACAG---GCCAGACAA---CTGCTG---TCTGGTATAGTGCAACAGCAAAGC---
AATTTG---CTGAGA---GCTATAGAGGCGCAACAGCATATGTTGCAACTCACGGTCTGGGGCATT
>EU161645
TTCTATTGCAATACATCAGGACTGTTTAATAGCATTTCAAATGAAACTACCACAAATGGAACC----------
-------------------------------------------------------------------------
----------------------------
ACAAATGGAACCATCACAATTCCATGCAGAATAAAACAAATTATAAACATGTGGCAGGAGGTAGGACGAGCAA
TGTATGCCCCTCCCATTGCAGGAAACATAACATGTAAATCAAAT---
ATCACAGGACTACTATTATCAAGTGATGGAGGCACAGATAATAGCACA-------------------------
-------------------------------------------------------------------------
-------
AAGCCAGAGACAGAGACCTTCAGACCTGGAGGAGGAGATATGAGGGATAACTGGAGAAGTGAATTATATAAAT
ATAAAGTGGTAGAAATTAAACCATTA---GGAATAGCACCCACTGAGGCAAAAAGGAGAGTGGTG--------
-------------------------------GAGAGAGAAAAAAGAGCA---GTGGGA---ATAGGAGCT---
------GTACTCCTTGGGTTCTTGGGAGCA---GCCGGAAGCACTATG---GGCGCGGCGTCA---
ATAACGCTGACGGTACAG---GCCAGACAA---TTGTTG---TCTGGTATAGTGCAACAGCAAAGC---
AATTTG---CTGAAG---GCTATAGAGGCGCAACAGCATATGTTGCAACTCACAGTCTGGGGCATT
>FJ846632
TTCTATTGCAATACAACAAATCTGTTTAATATAAGCATGACCAATGAC-------------------------
-------------------------------------------------------------------------
----------------------------
ACAGACACATTCATCACACTCCCATGTAGAATAAAACAAATTATAAATATGTGGCAGAGGGTAGGACGAGCAA
TGTATGCCCCTCCCATTACAGGAAACATAACATGTAACTCAAGT---
ATCACAGGACTACTATTGACACGTGATGGAGGAACAACAAATGGCACAGAG----------------------
-------------------------------------------------------------------------
-------
AATAATAACACAGAGATATTCAGACCTGCAGGAGGAAATATGAAGGATAATTGGAGAAGTGAATTATATAAAT
ATAAAGTGGTAGAGATTAAGCCATTA---GGAGTAGCACCCACTGGAGCAAAAAGGAGAGTGGTG--------
-------------------------------GAGAGAGAAAAAAGAGCA---GTGGGA---ATAGGAGCT---
------GTGTTCCTTGGGTTCTTGGGAGCA---GCAGGAAGCACTATG---GGCGCGGCGTCA---
ATCACGCTGACGGTACAG---GCCAGACAA---CTGTTG---TCTGGTATAGTGCAACAGCAAAGC---
AATTTG---CTGAGA---GCTATAGAGGCGCAACAGCATATGTTGCAACTCACGGTCTGGGGCATT
>AY529672
TTCTATTGTGATACATCAAGATTATTTAATAGAACATATAATGGTACAGAAGATAACTCAGGA----------
-------------------------------------------------------------------------
----------------------------
GATAACACAACTATTACAATTCCATGCAGAATAAGACAAATTATAAACATGTGGCAGGAGGTAGGACGAGCAA
TGTATGCCCCTCCCATTGCAGGAAACATAACATGTAAATCAAAT---
ATCACAGGACTACTATTGGTGAGTGATGGAGGCAACAGAAGCAGC----------------------------
-------------------------------------------------------------------------
-------------
TCAAATGAAACATTTAGACCTGGAGGGGGAAACATGAAGGACAATTGGAGAAGTGAATTATATAAATATAAAG
TGGTAGAAATTAGGCCATTG---GGAATAGCACCCACTAAAGCAAAAAGGAGAGTGGTG--------------
-------------------------GAGAAAGAAAAAAGAGCA---GTGGGAATAATAGGAGCT---------
GTGTTCCTTGGGATCTTGGGAATA---GCAGGAAGCACTATG---GGCGCAGCGTCA---
ATAACGCTGACGGCACAG---GCCAGACAA---GTGATG---TCTGGTATAGTGCAACAGCAAAGC---
AATTTG---CTGAGG---GCTATAGAGGCGCAACAGCATATGTTGCAACTCACAGTCTGGGGCATT
>AY463237
TTCTATTGCAATACATCAAAACTGTTTAATAGTACAAGTAATGATACACAA----------------------
-------------------------------------------------------------------------
----------------------------
AGTAACACAACCATCACACTCCCATGCAGAATAAAACAAATTATAAACATATGGCAGGAGGTAGGACGAGCAA
```

TGTATGCCCCTCCCATTGCAGGAAACATAACATGTAACTCAAGC---
ATCACAGGACTGCTGTTGCTACGTGATGGAGGAGAGGGGAACAAC----------------------------
----

AGCACAGAGATATTCAGACCTGGAGGAGGGAATATGAAGGACAATTGGAGAAGTGAATTATATAAATATAAAG
TGGTAGAAATTGAGCCATTG---GGAATAGCACCCACTACGGCAAAAAGGAGAGTGGTG--------------
-------------------------GAGAGAGAAAAAAGAGCA---GTGGGA---ATAGGAGCT---------
GTGCTCCTTGGGTTCTTGGGAGCA---GCAGGAAGCACTATG---GGCGCGGCGTCA---
ATAACGCTGACGGTACAG---GCCAGACAA---CTGTTG---TCTGGTATAGTGCAACAGCGAAGC---
AATTTG---CTGAGA---GCTATAGAGGCGCAACAGCATATGTTGCAACTCACGGTCTGGGGCATT
>KC863203
TTCTATTGCAATACATCAAAACTATTTAATAATACATACAGTCCTACATACAGTCCTAATGGTACAGGA----
----

GGAAATTCAACCATCAAAATTCCATGCAGAATAAAACAAATTATAAACATGTGGCAGGGGGTAGGACGAGCAA
TGTATGCCAATCCCATTGCAGGGAACATAACATGTAAATCAAAT---
ATCACAGGACTACTATTAGTACGTGATGGAGGCACAAACAACAGC----------------------------
----

ATGGAAATATTCAGACCTGGAGGAGGAAATATGAAGGACAATTGGAGAAGTGAATTATATAAATATAAAGTGG
TAGAAATTAAGCCATTG---GGAATAGCACCCACTGGAGCAAAAAGGAGAGTGGTG-----------------
----------------------GAGAGAGAAAAAAGAACA---GCGGGA---ATAGGAGCT---------
GTGTTCCTTGGGTTCTTGGGAGCA---GCAGGAAGCACTATG---GGCGCGGCGTCA---
TTGACGCTGACGGTACAG---GCTAGACAA---ATATTG---TCTGGTATAGTGCAACAGCAAAGC---
AATTTG---CTGAGG---GCTATAGAGGCGCAACAGCATATGTTGCAACTCACGGTCTGGGGCATT
>AY463223
TTCTACTGCAATACAACAGAACTGTTTAATTCAACAAAACTGTTCAATGTAACTAAGGTAACTAAT-------
----

GCAACTGAAAATATCACTCTCCCATGCAGAATAAAACAAATTGTAAACATGTGGCAGGAGGTGGGACGAGCAA
TGTATGCCCCTCCCATTGCAGGAAAAATAACATGTAGGTCAAAT---
ATCACAGGACTACTATTGTCACGTGATGGAGGCATGGATAATAACACGAAT----------------------
----

AGCACAAAAGAGGAGATATTCAGACCTGGAGGAGGAAATATGAAGGACAATTGGAGAAGTGAATTATATAAAT
ATAAAGTGGTAGAAGTTAAGCCATTG---GGAGTAGCACCCACGGAGGCAAAAAGGCGAGTGGTG--------
-------------------------------GAGAGAGAAAAAAGAGCA---GTGGGA---ATAGGAGCT---
------GTGTTCCTTGGGTTCTTGGGAGCA---GCAGGAAGCACTATG---GGCGCAGCGTCA---
ATAACGCTGACGGCACAG---GCCAGGCAA---CTGTTG---TCTGGTATAGTGCAACAGCAAAGC---
AATTTG---CTGAGA---GCTATAGAGGCGCAACAGCATATGTTGCAACTCACGGTCTGGGGCATT
>DQ351235
TTCTATTGCAATACATCAGGCCTGTTTAATGGTACGTACAATGGTACAGACATGTCTAATAGT----------
----

ACAGAAAGTACCATCACACTCCCATGCAGAATAAAACAAATTATAAACATGTGGCAGGGGGTAGGACGAGCAA
TGTATGCCCCTCCCATTGCAGGAAACATAACCTGTAAATCAAAC---
ATTACAGGATTACTATTGACACATGATGGAGGAAGAGAGGGTAAT----------------------------
----

AGCACTGAAACATTCAGACCTGGAGGAGGAAATATGAGGGATAATTGGAGAAGTGAATTATATAAATATAAAG
TAGTAGAAATCAAACCACTA---GGAATAGCACCCACAGAGGCAAAAAGGAGAGTGGTG--------------
-------------------------GAGAGAGAAAAAAGAGCA---GTGGGA---CTGGGAGCT---------
GTGTTCCTTGGGTTCTTGGGAGCA---GCAGGAAGCACTATG---GGCGCAGCGTCA---
ATAACGCTGACGGTACAG---GCCAGACAG---TTATTG---TCTGGTATAGTGCAACAGCAAAGC---
AATTTG---CTGAAG---GCTATAGAGGCGCAACAGCATATGTTGCAACTCACGGTCTGGGGCATT
>DQ351222
TTCTATTGCGATACAACAAGACTGTTTAATAGCAGTACATTTGGT----------------------------
----

GAAGGTGAAATCATCACACTCCCGTGCAAAATAAAACAAATTATAAACATGTGGCAGGGGGTAGGACGAGCAA

TGTATGCCCCTCCCGTTGAAGGAAATATAACATGTAACTCAAGT---
ACCACAGGAATACTATTGACACGTGATGGAGGAGACCAGGACAAT----------------------------
----

ACCACAGAGAAATTCAGACCTGGAGGAGGGAATATGAAGGACAATTGGAGAAGTGAATTATATAAATATAAAG
TGGTAGAAATTAATCCAGTG---GGAGTAGCACCCACGGAGGCAAAGAGGAGCGTGGTG--------------
-------------------------GCAAGAGAAAAAAGAACA---GTA------ATAGGAGCT---------
ATGATCCTTGGGTTCTTGGGAGCA---GCAGGAAGCACTATG---GGCGCGGCGTCA---
ATAACGCTGACGGTACAG---GCCAGACAA---CTGCTG---TCTGGTATAGTGCAACAGCAAAGC---
AATTTG---CTGAGA---GCTATAGAGGCGCAACAGCATCTGTTGCAACCCACGGTCTGGGGCATT
>DQ351218
TTCTATTGCAATACAACAAACCTGTTCAGTACAACAATCCTGGTCAACAACTCTACT----------------
----

GAAAAGGAAAACATCACACTCCCATGCAGAATAAAACAAATTATAAACATGTGGCAGGAGGTAGGACGAGCAA
TGTATGCCCCTCCCATTGAAGGAAACATAACATGTACATCAAAG---
ATCACAGGAATACTACTGACACGTGATGGAGGAGACAACACAACAACA-------------------------
----

AGAAATAACGGGACATTCAGACCTGGAGGAGGAAATATGAAGGACAATTGGAGAAATGAATTATATAAATATA
AAGTAGTAGAAATTAAACCATTG---GGAGTAGCACCCACTAAGGCAAAAAGGAGAGTGGTG-----------
----------------------------GAGAGAGAAAAAAGAGCA---GTGGGA---ATAGGAGCT------
---GTACTCCTTGGGTTCTTGGGAGCA---GCAGGAAGCACTATG---GGCGCAGCGTCA---
CTAACGCTGACGGTACAG---GCCAGACAA---CTGTTG---TCTGGTATAGTGCAACAGCAAAGC---
AATTTG---CTGAGG---GCTATAGAGGCGCAACAGCATATGTTGCAACTCACGGTCTGGGGCATT
>DQ447269
TTCTATTGCGATACCTCAAACTTGTTTAATAGGACATACCCATTGAATAATAGTAGTGCAGGAAATGACAATA
GT---

AGCAGTGATTCAACCATCACACTCCAATGCAGGATAAAACAAATTATAAACATGTGGCAGGGGGTAGGACGAG
CAATGTATGCCCCTCCCATTGAAGGAAACATAACATGTAAGTCAAAT---
ATCACAGGACTACTATTGACACGTGATGGAGGACCAAGTAATGAA----------------------------
----

AGTGAGACATTCAGACCTGCAGGAGGGGATATGAGGGACAATTGGAGAAGTGAATTATATAAATACAAAGTGG
TGGAAGTGAATCCATTA---GGAATAGCACCCACCAAGGCAAAAAGGAGAGTGGTG-----------------
----------------------GAGAGAGAGAAAAGAGCA---GTGGGA---ATAGGAGCT---------
GTGTTCCTTGGGTTCTTGGGAGCA---GCAGGAAGCACTATG---GGCGCAGCATCA---
ATAACGCTGACGGTACAG---GCCAGACAA---TTATTG---TCTGGTATAGTGCAACAGCAAAGC---
AATTTG---CTGAGG---GCTATAGAGGCGCAACAGCATATGTTGCAACTCACAGTCTGGGGCATT
>DQ369978
TTCTATTGCAATACAACACCACTGTTTAATATGTCTGCTGACAATAACAGCACC-------------------
----

AATAATGGCACCATCACACTCCAATGCAAGATAAAACAAATTATAAACATGTGGCAGGGGGTAGGACGAGCAA
TGTATGCCCCTCCCATTGCAGGAAACATAACATGTGCGTCAAAT---
ATCACAGGACTACTATTGACACATGACGGAGGCATAAAAAATGAC----------------------------
----

ACAGTCCCAGAGGTATTTAGACCTGGAGGAGGAAATATGAAGGACAATTGGAGAAGTGAATTATATGAATATA
AAGTGGTAGAAATTAAGCCATTG---GGAATAGCCCCCACTGAGTCAAGAAGGATAGTGGTG-----------
----------------------------GAGAGAGAAAAAAGAGCA---GTGGGA---ATGGGAGCT------
---GTGATCTTTGGGTTCTTAGGAGCA---GCAGGAAGCACTATG---GGCGCGGCGTCA---
ACGGCGCTGACGGTACAG---GCCAGACAA---CTGTTG---TCTGGTATAGTGCAACAGCAAAGC---
AATTTG---CTGAGA---GCTATAGAGGCGCAACAGCATATGTTGCAACTCACAGTCTGGGGCATT
>DQ351225
TTCTATTGCAATACATCAAAACTGTTTAACGGTACATACAGCCATAATGTCAGTACAAATAAC----------
----

AGTACAGAACCCATCACAATTCCATGCAGGATAAAACAAATTATAAACATGTGGCAAGAGGTAGGACGAGCAA

TGTATGCCCCTCCCATTGCAGGAAACATAACATGTAAATCAAAT---
ATCACAGGACTACTATTAACACGTGATGGAGGGAACACAAACACAAGC-------------------------
----

AATAACACAGAGACATTCAGACCTGGAGGAGGAAATATGAAGGACAATTGGAGAAATGAATTATATAAATATA
AAGTGGTAGAAATTAAGCCATTG---GGAGTAGCACCCACTAAGGCAAAAAGGAGAGTGGTG-----------
----------------------------GAGAGAGAAAAAAGAGCA---GTGGGA---ATAGGAGCT------
---GTGCTCCTTGGGTTCTTGGGAGCA---GCAGGAAGCACTATG---GGCGCGGCGTCA---
ATAACGCTGACGGTACAG---GCCAGACAA---CTGCTG---TCTGGTATAGTGCAACAGCAAAGC---
AATTTG---CTGAGA---GCCATAGAGGCGCAACAGCATATGTTGCAACTCACGGTCTGGGGCATT
>DQ396367
TTCTATTGCAATACAACATACCTGTTTTCTAATAAGACAGGCAACACAACC----------------------
----

GACAACGCAACCATCACGCTCCCATGCAGGATAAAACAATTTATAAACATGTGGCAGGAGGTAGGACGAGCAA
TATATGCCCCTCCCATTGCAGGAAAAATAACATGTAACTCAAAT---
ATCACAGGACTACTATTGACACGTGATGGAGGTGTGAATACTACGAATAATTCG------------AGT----
----
AATACAAGTAAAACA---
GAGACAGAGATATTCAGACCTGGAGGAGGAAATATGAAAGACAATTGGAGAAGTGAATTATATAAATACAAAG
TGGTAGAAATTAATCCATTG---GGAGTAGCACCCACTAATGCAAAAAGGAGAGTGGTG--------------
-------------------------GAGAGAGAAAAAAGAGCA---GTGGGG---ATAGGAGCT---------
GTACTCCTTGGGTTCTTGGGAGCC---GCAGGAAGCACTATG---GGCGCGGCGTCA---
ATAACGCTGACGGTACAG---GCCAGACAA---TTGTTG---TCTGGTATAGTGCAACAGCAAAGC---
AATTTG---CTGAGA---GCTATAGAGGCGCAACAGCATATGTTGCAACTCACAGTCTGGGGCATT
>DQ351226
TTCTATTGTAATACAACAAACCTGTTTAACATAAACCTGACTAACTGGAGGTCTGATAGTAAGGGA-------
----

AATTCCACAATTATCACACTCCCATGTAGAATAAGACAATTTGTAAACCTGTGGCAGGGGGTAGGAAGGGCAA
TGTATGCCCCTCCCATTGAAGGTAATATAACATGTAAGTCAAAT---
ATCACAGGACTACTATTGACACGTGATGGAGGCAATGTCACAAAT----------------------------
----

AATGGCACAGAGATATTCAGACCTGGAGGAGGAGATATGAGGGACAATTGGAGAAGTGAATTATATAAATATA
AAGTGGTAGAAATTAAGCCATTG---GGAGTAGCACCCACAGAGGCAAAAAGGAGAGTGGTG-----------
----------------------------GAGAGAGGAAAAAGAGCA---GTGGGA---ATGGGAGCT------
---GTGATCCTTGGTTTCTTGGGAGCA---GCAGGAAGCACTATG---GGCGCGGCGTCA---
ATAACGCTGACGGTACAG---GCCAGACAA---CTGTTG---TCTGGTATAGTGCAACAGCAAAGC---
AATTTG---CTGAGA---GCTATAGAGGCGCAACAGCACATGTTGAAACTCACAGTCTGGGGCATT
>DQ369985
TTCTATTGCAATACATCAAACCTGTTTAATAGTACAGTTAATGGTAATAGTACAGTTAATGGTACACACCTGG
GTAATAGT---
-------------------AAT---
AGTACAAATAACAACATCACACTCCAATGCAGAATAAAACAAATTATAAACATGTGGCAGGGGGTAGGACGAG
CAATGTATGCCCCTCCTATTGCAGGAAACATAACCTGTGTATCAAAT---
ATCACAGGACTACTATTGACACGTGATGGTGGCAGGGAGAATGGCACA-------------------------
----

AATAACACAGAGATATTCAGACCTGCAGGAGGAAACATGAAGGACAATTGGAGAAGTGAATTATATAAATATA
AAGTGGTAGAAATTAATCCATTG---GGAGTAGCACCCACTGAGGCAAAAAGGAGAGTGGTG-----------
----------------------------GAGAGAGAAAGAAGAGCA---GTGGGA---ATAGGAGCT------
---GTGTTCCTTGGGTTCTTGGGAGCA---GCAGGAAGCACTATG---GGCGCAGCGTCA---
ATAACGCTGACGGTACAG---GCCAGACAA---CTGTTG---TCTGGTATAGTGCAACAGCAAAGC---
AATTTG---CTGAGG---GCTATAGAGGCGCAACAGCATATGTTGCAACCCACGGTCTGGGGCATT
>DQ369996
TTCTATTGCAATACATCAAAACTGTTTAATAGTACATACAGTAGTACATACAATAATACATCTGATAATACT-
----

TCACATCCAAACATCACACTCCCATGCAGAATAAAACAAATTATAAACCTGTGGCGGGAGGTAGGACGGGCAA

TGTATGCCCCTCCCATTGCAGGAAACATAACATGTAACTCAAGT---
ATCACAGGACTACTATCGACACGTGATGGGGGAGAAAATAACTCA----------------------------
----

ACAACAGAGACATTCAGACCTGGAGGAGGAGATATGAGGGACAATTGGAGAAGTGAATTATATAAATATAAAG
TGGTAGGAATTGAGCCATTA---GGAGTAGCACCCACCAAGGCAAAAAGGAGAGTGGTGGAG-----------
----------------------AGGGAGAGAGAAAAAAGAGCA---ATGGGA---ATAGGAGCT---------
GTGTTCCTTGGGTTCTTGGGAGCA---GCAGGAAGCACTATG---GGCGCGGCGTCA---
ATAACGCTGACGGTACAG---GCCAGACAA---CTGTTG---TCTGGTATAGTGCAACAGCAAAGC---
AATTTG---CTGAAG---GCTATAGAGGCGCAACAGCATCTGTTGCAACTCACAGTCTGGGGCATT
>DQ445633
TTCTATTGCAATACAACAAACTTGTTTAATAATATAAAAAATGATGAGGATACAAAAAAT-------------
----

GGTAGTGATCCAATCATACTCCCATGCAGAATAAAACAAATTATAAACATGTGGCAGGGGGTAGGACGAGCAA
TGTATGCCCCTCCCATTGCAGGAAACATAACATGTAACTCAAGT---
ATCACAGGACTACTATTGACACGTGATGGAGGGAATGGTACTAAT----------------------------
----

AATACTGAGATATTCAGACCTGGAGGAGGAAATATGAAGGACAACTGGAGAAATGAATTATACAAATATAAAG
TGGTAGAAATTAAACCATTA---GGAATAGCACCCACCAAGGCAAAAAGGAGAGTGGTG--------------
-------------------------GAGAGAGAAAAAAGAGCA---GTGGGA---ATAGGAGCT---------
GTGTTCCTTGGGTTCTTGGGAGCA---GCAGGAAGCACTATG---GGCGCGGCGTCT---
ATCACGCTGACGGTACAG---GCCAGACAA---CAGTTG---TCTGGTATAGTGCAACAGCAAAGC---
AATTTG---CTGAGA---GCTATAGAGGCGCAACAGCATATGTTGCAACTCACGGTCTGGGGCATT
>DQ369979
TTCTATTGCAATACAACAAGACTGTTCAACACAACTAGCACAAGAGAG-------------------------
----

TCAAACAAAACCATCACACTCCCATGCAGAATAAAACAAATTATAAACATGTGGCAGGGGGTAGGACGAGCAA
TGTATGCCCCTCCCATTGCAGGAAACATAACATGTAACTCAAGT---
ATCACAGGAATACTATTGCAACGTGATGGAGGAAACACGAGTGAG----------------------------
----

CCCACAGAGATATTCAGACCTGGAGGAGGAAATATGAAGGACAATTGGAGAAGCGAATTATATAAATATAAAG
TGGTAGAAATTAAGCCATTG---GGAATAGCACCCACGAAGGCAAAAAGGAGAGTGGTG--------------
-------------------------GAGAGAGAAAAAAGAGCA---GTGGGA---ATAGGAGCT---------
GTGTTCCTTGGGTTCTTGGGAGCA---GCAGGAAGCACTATG---GGCGCGGCGTCA---
ATAACGCTGACGGTACAG---GCCAGACAA---CTGTTG---TCTGGTATAGTGCAACAGCAAAGC---
AATTTG---CTGAGA---GCTATAGAGGCGCAACAGCATCTGTTGCAACTCACGGTCTGGGGCATT
>DQ369984
TTCTACTGCAATACAACAAAACTGTTTAATACAACAAGACTGTTAAAAGAAAGTGATTCA-------------
----

AACACAAACACCACCACACTCCCATGCAGGATAAAACAGATTGTAAACATGTGGCAGGAGGTAGGACGAGCAA
TGTATGCCCCTCCCATTGCAGGAAACATAACATGTAAATCAAAT---
ATCACAGGACTACTATTGACACGTGATGGAGGGAATAACACTGACACAGAT----------------------
----

GGCGATAACACAGAAACATTCAGACCTGGAGGAGGAAATATGAAGGACAATTGGAGAAGTGAATTATATAAGT
ATAAGGTGATAAAAATTAAGCCATTA---GGAATAGCACCCACTGAGGCAAAAAGAAGAGTGGTG--------
-------------------------------AAGAGAGAAAAAAGAGCA---GTGGGA---ATAGGAGCT---
------ATGATCTTTGGGTTCTTGGGAGCA---GCAGGAAGCACTATG---GGCGCGGCGTCA---
ATAACGCTGACGGTACAG---GCCAGACAA---CTGTTG---TCTGGTATAGTGCAACAGCAAAGC---
AATTTG---CTGAGA---GCTATAGAGGCGCAACAGCATATGTTGCAACTCACGGTCTGGGGCATT
>DQ396364
TTCTATTGCGATACAACAGGACTGTTTAATAGTACATACATGCCTGAATACTGGCCTAATAGTACAGAAAGTA
AT---

TCAAGCTCAGTAATCACACTCCCATGCAGAATAAAACAAATTATAAACATGTGGCAGGAGGTAGGAAGAGCAA

TGTATGCTCCTCCCATTGCAGGAAACATAACATGTAGATCAAAT---
ATCACAGGATTACTATTGGTGCGTGATGGAGGAACAAATAACACA----------------------------
----

AATAACACAGAGACCTTCAGACCTGGAGGAGGAGATATGAGGGACAATTGGAGAAGTGAATTATACAAATATA
AAGTGGTAGAAATTAAGCCATTG---GGAATAGCACCTACTGGGGCAAAAAGGAGAGTGGTG-----------
----------------------------GAGAGAGAAAAAAGAGCA---GTTGGA---ATAGGAGCT------
---GTGTTCCTTGGGTTCTTGGGAGCA---GCAGGAAGCACTATG---GGCGCAGCGTCA---
ATAACGCTGACGGTACAG---GCCAGACAA---TTGTTG---TCTGGTATAGTGCAACAGCAAAGT---
AATTTG---CTGAGG---GCCATAGAGGCGCAACAGCATATGTTGCAACTCACGGTCTGGGGCATC
>DQ369986
TTCTATTGCAACACATCAGGACTATTTAATGGTACATACACGAGTAACATGACAGGAAATGGAAATGAT----
----

TCAAACTCAAACATCACACTTCCATGCAGAATAAAACAAATTATAAACATGTGGCAAGAGGTAGGACGAGCAA
TGTATGCCCCTCCCATTAAAGGAAACATAACATGTAGATCAAAT---
ATCACAGGACTACTATTAGTACGTGATGGAGGCAACCACAACAACAGC-------------------------
----

AACAACACAGAGACATTCAGACCTGGAGGAGGAAATATGAAGGACAATTGGAGAAGTGAATTGTATAAATATA
AAGTGGTAGAAATTAAACCATTA---GGAATAGCACCCACTACTGCAAAAAGGAGAGTGGTG-----------
----------------------------GAGAGAGAAAAAAGAGCA---GTGGGA---ATAGGAGCT------
---GTATTCCTTGGGTTCTTGGGAACA---GCAGGAAGCACTATG---GGCGCAGCGTCA---
ATAACGCTGACGGTACAG---GCCAGACAA---CTGTTG---TCTGGTATAGTGCAACAGCAAAGC---
AATTTG---CTGAGA---GCTATAGAGGCGCAACAGCATCTGTTGCAACTCACGGTCTGGGGCATT
>DQ369980
TTCTATTGCAATACATCAAACCTGTTTAATAGTACATACAAGCCTAATGGTACAAGAAGTACT----------
----

TCAAATTTAACCATCACACTCCAGTGCAGAATAAAACAAATTATAAACATGTGGCAGGAGGTGGGACGAGCAA
TGTATGCCCCTCCCATTGCAGGAAACATAACATGTAAATCAAAT---
ATCACAGGGCTACTATTGACACGTGACGGAGGAAAAGGAAATGAT----------------------------
----

ACGTCTGAAACATTCAGACCTGGAGGAGGAGATATGAGGGACAATTGGAGAAGTGAATTATATAAATATAAGG
TGGTAGAAATTAAGCCATTA---GGATTAGCACCCACTAAGGCAAAAAGGAGAGTGGTG--------------
-------------------------CAGAGAGAGAAAAGAGCA---GTGGGA---ATAGGAGCT---------
GTGTTCCTTGGGTTCTTGGGGGCA---GCAGGAAGCACTATG---GGCGCGGCGTCA---
ATAACGCTGACGGTACAG---GCCAGACAA---TTATTG---TCTGGCATAGTGCAACAGCAAAGC---
AATTTG---CTGAAG---GCTATAGAGGCGCAACAACATCTGTTGCAACTCACAGTCTGGGGCATT
>DQ396385
TTCTATTGTAATACAACAAAGCTGTTTAATGGTACATACAGGCCTGCATACAATAATGGTACAGGA-------
----

AGTAATTCAAGCATCACACTCCAATGCAGGATAAAACAAATTATAAACATGTGGCAGCAGGTAGGACGAGCAA
TGTATGCTCCTCCCATTGAAGGAAACATAACATGTAAATCAACT---
ATCACAGGACTACTATTGACACGTGATGGAGGGGAAAATGGCACA----------------------------
----

GAAAATGAGACATTCAGACCTGGAGGAGGAAATATGAGGGACAATTGGAGAAGTGAATTATACAAATATAAAG
TGGTAGAAGTTAAACCATTG---GGAATAGCACCTACTGGGGCAAAAAGGAGAGTGGTG--------------
-------------------------GAGAGAGAAAAAAGAGCA---GTGGGA---ATAGGAGCT---------
GTGTTCCTTGGGTTCTTGGGGGCA---GCAGGAAGCACTATG---GGCGCGGCGTCA---
CTGACGCTGACGGTACAG---GCCAGACAA---CTATTG---TCTGGTATAGTGCAACAGCAAAGC---
AATTTG---CTGAGG---GCCATAGAGGCGCAACAGCATATGTTGCAACTCACGGTCTGGGGCATT
>DQ369987
TTCTATTGCAATACATCAAGACTGTTTAATAGCACATTTAATAGTACATACATGCCTAATGCTACAGAAAGTA
AT---

TCAAGTTCAGTCATCACGATTCCCTGCAGAATAAAACAAATTATAAACATGTGGCAGGAGATAGGACGGGCAA

TGTATGCCCCTCCTATTGCAGGAAACATAACATGTAAATCAAAT---
ATCACAGGACTACTATTAGTACGTGATGGAGGCAACAGTAACAAT----------------------------
----

GACACAGAGATATTCAGACCTGGAGGAGGAAATATGAAGGACAATTGGAGAAGTGAATTATACAAATATAAAG
TGGTAGAAATTAAGCCATTG---GGAATAGCACCCACGAAGGCAAAAAGGAGAGTGGTG--------------
-------------------------GAGAGAGAAAAAAGAGCA---GTAGGA---ATAGGAGCT---------
GTGTTCCTTGGGTTCTTGGGAGCA---GCAGGAAGCACTATG---GGCGCGGCGTCA---
ATAACGCTGACGGCACAG---GCCAGACAA---TTGTTG---TCTGGTATAGTGCAACAGCAAAGC---
AATTTG---CTGAGG---GCTATAGAGGCGCAACAGCATATGTTGCAACTCACGGTCTGGGGCATT
>DQ369988
TTCTATTGCGAAACAACAAGACTGTTTAATACAACACAACTTAATACAACA----------------------
----

CAAAATGGAACAATCATACTCCCATGTAGAATAAAGCAAATTATAAACATGTGGCAGGGGGTAGGACGAGCAA
TGTATGCCCCTCCCATTGCAGGAATGATAACATGTAACTCAAGT---
ATCACAGGACTACTATTGGTACGCGATGGAGGCACGAATGACACA----------------------------
----

GAGATATTCAGACCTGGAGGAGGAAATATGAAGGACAATTGGAGAAGTGAATTATATAAATATAAAGTGGTAG
AAATTAAGCCATTG---GGAATAGCACCCACTGGGGCAAAAAGGAGAGTGGTG--------------------
-------------------GAGAGAGAAAAAAGAGCA---GTGGGA---ATAGGAGCT---------
GTACTCCTTGGGTTCTTGGGAGCA---GCAGGAAGCACTATG---GGCGCAGCATCA---
ATAACGCTGACGGTACAG---GCCAGACAA---CTGTTG---TCTGGTATAGTGCAACAGCAAAGC---
AATTTG---CTGAGG---GCTATAGAGGCGCAACAGCATATGTTGCAACTCACGGTCTGGGGCATT
>DQ396373
TTCTATTGCAATACATCACAACTGTTTAATAGTACATACAACAACCATACATACACGAATAATGGGACA----
----

GGAAATTCAAACATCACACTTCCATGCAAAATAAAACAGATTATAAATATGTGGCAGGGGGTAGGACGAGCAA
TGTATGCCCCTCCCATTGCAGGAAACATAACATGTATCTCAAAT---
ATCACAGGACTAATATTGACACGTGATGGAGGAGAAGAAGGAAACAAC-------------------------
----

AACAAAACAGAGATATTCAGACCTGGAGGAGGAAATATGAAGGATAATTGGAGAAATGAATTATATAAATATA
AAGTGGTAGAAATTAAGCCATTG---GGAATAGCACCCACTAAGGCAAAAAGAAGAGTGGTG-----------
----------------------------GAGAGAGAAAAAAGAGCA---GTGGGA---CTAGGAGCT------
---GTGTTCCTTGGGTTCTTGGGAGCA---GCAGGAAGCACTATG---GGCGCGGCGTCA---
ATAACGCTGACGGTACAA---GCCAGACAA---CTGTTA---GCTGGTATAGTGCAACAGCAAAAT---
AATTTG---CTGAGG---GCTATAGAGGCGCAACAGCATATGTTGCAACTCACGGTCTGGGGCATT
>DQ396374
TTCTATTGCAATACATCAAAACTGTTTAATACAACAGTTAATGGAACAGAGTTAGAGTTTAACACAACAGGAA
GG---

TCTAATAAAACCATCACTCTCCAATGCAAGATAAAACAAATTATAAACATGTGGCAAGAGGTAGGACGAGCAA
TGTATGCCCCTCCCATTGCAGGAAACATAACATGTAGATCAAAT---
ATCACAGGATTACTATTGACATGGGATGGACCAACAAACAATGGC----------------------------
----

ACAGACATATTTAGACCTGGAGGAGGAAATATGAAAGACAATTGGAGAAGTGAATTATATAAATATAAAGTGG
TAGAAATTAAGCCATTA---GGAATAGCACCCACTGAATCAAAAAGGAGAGTGGTG-----------------
----------------------GAAAGAGAAAAAAGAGCA---GTGGGA---CTAGGAGCT---------
TTGATCCTTGGGTTCTTGGGAACA---GCAGGAAGCACTATG---GGCGCGGCGTCA---
ATAGCGCTGACGGTACAG---GCCAGACAA---CTGTTG---TCTGGTATAGTGCAGCAGCAAAAC---
AATTTG---CTGAGA---GCTATAGAGGCGCAACAGCATATGTTGCAACTCACAGTCTGGGGCATT
>DQ369989
TTCTATTGCAATACATCAGAACTATTTAACAGTACATACAATAAGGATACAAAGAGTAATGGTACAAAG----
----

AGTAATTTAACCATCACAATTCCATGCAGGATAAAACAAATTATAAACATGTGGCAGCAGGTGGGACGAGCAA

```
TGTATGCTCCTCCCATTGCAGGAAACATAACATGTAAATCAAAT---
ATCACAGGACTACTATTGGTGCGTGATGGAGGAACAAATAACATA----------------------------
-------------------------------------------------------------------------
----------
AATAACACAGAGACATTCAGACCTGGAGGAGGAGATATGAGGGATAATTGGAGAAGTGAATTATACAAATATA
AAGTGGTAGAAATTAAACCATTG---GGAATGGCACCAACTAAGGCAAAACGGAGAGTGGTG-----------
----------------------------GAGAGAGAAAAAAGAGCA---GTGGGA---ATAGGAGCT------
---GTGCTCTTTGGGTTCTTGGGAGCA---GCAGGAAGCACTATG---GGCGTGGCGTCA---
ATAACGCTGACGGTACAG---GCCAGACAA---TTGTTG---TCTGGTATAGTGCAACAGCAAAGC---
AATTTG---CTGAGA---GCCATAGAGGCGCAACAGCGTATGTTGCAACTCACGGTCTGGGGCATT
>DQ396375
TTCTATTGCAATACAACAGAACTGTTTAATAATAGTGAAGTAAACATAACTGCAGCTACTGCA----------
-------------------------------------------------------------------------
----------------------------
ACAAATGAAACCTTCACACTCCCATGCAAAATAAAACAAATTATAAACATGTGGCAGGGGGTAGGACGAGCAA
TGTATGCCCCTCCCATTAAAGGAAACATAACATGTAAATCAAAC---
ATCACAGGAATACTATTGACATGGGATGGAGGAAATGAAACTAGGACAGATAAT-------------------
-------------------------------------------------------------------------
-
AGGACAAATAATAAAACAGAGATATTCAGACCTGGAGGAGGAAATATGAAGGACAATTGGAGAAATGAATTAT
ATAAATATAAAGTGGTAGAAATTAAGCCATTG---GGAGTAGCACCCACTACGGCAAAAGGAGAGTGGTG--
-------------------------------------GAGAGAGAAAAAAGAGCA---GTAGGA---
ATAGGAGCT---------GTGCTCCTTGGGTTCTTGGGAGCA---GCAGGAAGCACTATG---
GGCGCGGCGTCA---ATAACGCTGACGGTACAG---GCCAGACAA---CTGTTG---
TCTGGTATAGTGCAACAGCAAAGC---AATTTG---CTGAGA---
GCTATAGAGGCGCAACAGCATATGTTGCAACTCACAGTCTGGGGCATT
>DQ396389
TTCTATTGCAATACATCAAGACTGTTTAATGGTACATACAACAGCACAGAAAACAGCACAGAAAAT-------
-------------------------------------------------------------------------
----------------------------
GCAAATAGTACCATCACACTCCCATGTAGAATAAAACAAATTGTAAACATGTGGCAGGGAGTAGGACAAGCAA
TGTATGCCCCTCCCATTGCAGGAAACATAACATGTAACTCAAAT---
ATCACAGGACTACTATTGACACGTGATGGGGGGAACAACAATAAC----------------------------
-------------------------------------------------------------------------
-------------
GGGACAGAGACATTCAGACCTGGAGGAGGAGATATGAGGGACAATTGGAGAAGTGAATTATATAAATATAAAG
TGGTAGAAATTAAGCCATTA---GGAATAGCACCCACTAAGGCACAAAGGAGAGTGGTG--------------
-------------------------CAGAGAGAAAAAAGAGCA---GTAGGA---ATAGGAGCT---------
GTACTCCTTGGGTTCTTGTCAGCA---GCAGGAAGCACTATG---GGCGCGGCGTCG---
ATAACGCTGACGGTACAG---GCCAGACAA---GTGCTG---TCTGGTATAGTGCAACAGCAGAGC---
AATCTG---CTGAGG---GCTATAGAGGCGCAACAGCATATGTTGCAACTCACGGTCTGGGGCATT
>DQ396376
TTCTATTGCAATACAACAAAACTATTTAATGACAATGAGACA-------------------------------
-------------------------------------------------------------------------
-------------------------------
AACGAATACATCATACTCCCATGCAGAATAAAACAAATTATAAACATGTGGCAGGGGGTAGGACGAGCCATGT
ATGCCCCTCCCATTGAAGGAAACATAACATGTAAATCAAAT---
ATCACAGGACTATTATTGACACGTGATGGAGGAGGCGAAAATGAA----------------------------
-------------------------------------------------------------------------
----------
ACCAAAATCGAGATATTCAGACCTGGAGGAGGAAATATGAAGGACAATTGGAGAAGTGAATTATATAAATATA
AAGTGGTAGAACTTAAGCCATTG---GGCATAGCACCCACTGAGGCAAAAAGGAGAGTGGTG-----------
----------------------------GAGAGAGAAAAAAGAGCA---GTGGGA---ATGGGAGCT------
---GTGCTCCTTGGGTTCTTGGGAGCA---GCGGGAAGCACTATG---GGCGCGGCGTCA---
ATAACGCTGACGGTACAG---GCCAGACAA---CTGTTG---TCTGGTATAGTGCAACAGCAAAGC---
AATTTG---CTGAGG---GCTATAGAGGCGCAACAACATATGTTGCAACTCACGGTCTGGGGCATT
>DQ093591
TTCTACTGCAATACAAGCGGACTGTTCAATGCAACTTTCAATACAACTAATACA-------------------
-------------------------------------------------------------------------
----------------------------
```

ACTGATGCAAACATCACACTCCCATGCAGAATAAAACAAATTATAAACATGTGGCAGGAGGTAGGACGAGCAA
TGTATGCCCCTCCCATTGAAGGAAACATAATATGTAGATCAAAC---
ATCACAGGACTGCTATTGCAACGTGATGGAGGCAAGGGCAATGGCACT-------------------------
----

GACAACGAAACAGAGATATTCAGACCTGGAGGAGGAAATATGAAAGACAATTGGAGAAGTGAATTATATAAAT
ATAAGGTGGTAGCAATTAAGCCATTA---GGAGTAGCACCCACGGATGCACAAAGGAGAGTGGTG--------
-------------------------------GAGAGAGAAAAAAGAGCA---GTGGGA---ATAGGAGCT---
------ATGCTCTTTGGGTTCTTAGGAGCA---GCAGGAAGCACTATG---GGCGCAGCGTCA---
ATAACGCTGACGGTACAG---GCCAGACAA---CTGTTG---TCTGGTATAGTGCAACAGCAAAGC---
AATTTG---CTGAGA---GCTATAGAGGCGCAACAGCATCTGTTGCAACTCACAGTCTGGGGCATT
>DQ351219
TTCTATTGTAATACAACAGGACTGTTTTCTAATAATGGAAGTGCAGGA-------------------------
----

AACGCAACCACCATCACACTCCCATGCAGAATAAAACAAATTATAAACATGTGGCAGGGGGTAGGACGAGCGA
TGTATGCCCCTCCCATTGCAGGAAACATAACATGTACCTCAAAT---
ATCACAGGACTACTATTGACACGGGATGGAGGAATAAAGGATAACACA-------------------------
----

ACAGACATAACAGAGACATTCAGACCTGGAGGAGGAAATATGAAGGACAATTGGAGAAATGAATTATATAAAT
ATAAAGTGGTAGAAATTAAACCATTG---GGAGTAGCACCCACTGAGGCAAAAAGGAGAGTGGTG--------
-------------------------------GATAGAGAAAAAAGAGCA---GTA------CTAGGAGCT---
------ATGTTCCTTGGGTTCTTGGGAGTA---GCAGGAAGCACTATG---GGCGCAGCGTCA---
GTAACGCTGACGGTACAG---GCTAGACAA---TTATTG---TCTGGTATAGTGCAACAGCAAAGC---
AATTTG---CTGAGG---GCTATAGAGGCGCAACAGCATATGCCGCAACTCACAGTCTGGGGCATT
>DQ351229
TTCTATTGCAATACATCAAAACTGTTTAATAGTACATTTAATAGTACATATATGCATAATGATACAGGAAGTA
AT---

ACAAGCTCAACAATCACAATCCCATGCAAAATAAAACAAATTATAAACATGTGGCAGGAGGTAGGAAGAGCAA
TGTATGCTCCTCCCATTGCAGGAAACATAACATGTAAATCAAAT---
ATCACAGGACTACTATTGACACGTGATGGGGGAAATTCAACTGAT----------------------------
----

AATGAAACAGAAACATTCAGACCTGAAGGAGGAGACATGAGGGACAATTGGAGAAATGAATTATACAAATATA
AAGTGGTAGAAATTAAGCCATTG---GGAATAGCACCCACTGAGGCAAAGAGGAGAGTGGTG-----------
----------------------------GAGAGAGAAAAAAGAGCA---GTGGGA---ATAGGAGCT------
---GTGCTCCTTGGGTTCTTGGGAGCA---GCAGGAAGCACTATG---GGCGCGGCGTCA---
ATAACGCTGACGGTACAA---GCCAGACAA---CTGTTG---TCTGGTATAGTGCAACAGCAAAGC---
AATTTG---CTGAGA---GCTATAGAGGCGCAACAGCATATGTTGCAACTCACGGTCTGGGGCATT
>DQ275654
TTCTATTGCAATACATCACAACTATTTAATGATAGTAATACAGAATTTAATATTAGTAGTACAGAA-------
----

AATAATAAAACCATCACAATTCTATGCAGAATAAAACAATTTATAAACATGTGGCAGAAGGTAGGACAAGCAA
TGTATGCCCCTCCCATAGCAGGAAACATAACATGTAACTCAAGC---
ATCACAGGATTATTACTGACACGTGATGGTGGAAATGAAAGTATCACA-------------------------
----

AATAACACAGAGATATTCAGACCTGGAGGAGGAAATATGAAGGACAATTGGAGAAGTGAATTATATAAATATA
AAGTGGTAGAAATTATGCCATTG---GGAATAGCACCCACTACTGCAAAAAGGAGAGTGGTG-----------
----------------------------GAGAGAGAAAAAAGAGCA---GTGGGA---ATAGGAGCT------
---GTGATCTTTGGGTTCTTGGGAGCA---GCAGGAAGCACTATG---GGCGCGGCGTCA---
ATAACGCTGACGGTACAG---GCCAGACAA---CTGCTG---TCTGGTATAGTGCAACAGCAAAGT---
AATTTG---CTGAGA---GCTATAGAGGCGCAACAACATATGTTGCAACTCACGGTCTGGGGCATT
>DQ351216
TTTTATTGCGATACATCGCGGCTGTTTACTAATGTAAGCAATGAAAATGACACAATCACCAATGTAAGCAAT-
----

AAAAATGACACAATCACACTCCCATGCAGAATAAAACAAATTGTAAACATGTGGCAGGGGGTAGGACGAGCAA
TGTATGCCCCTCCCATTGCAGGACAAATAACATGTAGCTCAAAT---
ATCACAGGACTACTATTGACACGTGATGGAGGAAACACAACTGTT----------------------------
----

ACTAACACAGAGGTATTCAGACCTGAAGGAGGAAATATGAAGGACAATTGGAGAAGTGAACTATATAAATATA
AAGTGGTAGAAATTAAACCATTG---GGAGTAGCACCCACTAAGGCAAAAAGGAGAGTGGTG-----------
----------------------------GAGAGAGAAAAAAGAGCA---GTGGGA---ATAGGAGCT------
---GTACTCCTTGGGTTCTTGGGAGCA---GCAGGAAGCACTATG---GGCGCGGCGTCT---
ATAACGCTGACGGTACAG---GCCAGACAA---CTGTTG---TCTGGTATAGTGCAACAGCAAAAC---
AATTTG---CTGAGA---GCTATAGAGGCGCAACAGCATATGTTGCAACTCACGGTCTGGGGCATT
>DQ275645
TTCTACTGCAATACAACAGAACTGTTTAATGTAAACAATGATCCA----------------------------
----

AACGATTCAAACATCACGCTCCCATGCAGAATAAAGCAAATTATAAACATGTGGCAGGGGGTAGGACGAGCAA
TGTATGCCCCTCCCATTGAAGGAGAGATAACATGTAAATCAAAC---
ATTACAGGACTACTATTGGAGCGTGATGGAGGAAAAACAAATGAC----------------------------
----

ACGAAAGAGACATTTAGACCTGGGGGAGGAAATATGAAGGACAATTGGAGAAGTGAATTATATAAATATAAAG
TGGTAGAAATTAAACCATTG---GGAGTAGCACCCACAGAGGCAAAAAGGAGAGTGGTG--------------
-------------------------GAGAGAGAAAAAAGAGCA---GTAACA---ATAGGAGCT---------
ATGTTCCTTGGGTTCTTGGGAGCA---GCAGGAAGCACTATG---GGCGCGGCGTCA---
ATAACGCTGACGGTACAG---GCCAGACAA---CTGTTG---TCTGGTATAGTGCAACAGCAAAGC---
AATTTG---CTGAGA---GCTATAGAGGCGCAACAGCATATGTTGCAACTCACAGTCTGGGGCATT
>DQ351230
TTCTATTGCAATACAACAAGACTGTTTAATAATGATACAGGCGAACTGAATGATAATAAT-------------
----

AAAACAACAAACATCACTCTCCCATGCAGAATAAAACAAATTATAAACATGTGGCAAGAGGTAGGACGAGCAA
TGTATGCCCCTCCCATTGAAGGAAACATAACATGTATATCAAAT---
ATCACAGGACTACTATTGCTACGAGATGGAGGAACAACAAATAACACAATTAAT-------------------
----
-
GGCACAGGGAAAAAGAATGATACAGTCAGACCTGGAGGAGGAAATATGAAAGACAATTGGAGAAGTGAATTAT
ATAAATATAAAGTGATAGAACTTAAACCATTG---GGAATAGCACCCACTGAGGCAAGAAGGAGAGTGGTG--
-------------------------------------GAGAGAGAAAAAAGAGCA---GTGGGA---
ATAGGAGCT---------GTGTTCCTTGGGTTCTTGGGAGCA---GCAGGAAGCACTATG---
GGCACGGCGTCA---ATAACGCTGACGGTACAG---GCCAGACAA---CTGCTG---
TCTGGTGTAGTGCAACAGCAAAGC---AATTTG---CTGAGA---
GCTATAGAGGCGCAACAACATCTGTTGCAACTCACAGTCTGGGGCATA
>DQ275656
TTCTATTGCAATACATCACAATTGTTTAATAGTACATACACACATAATAGTACATATAATAGTACAGAA----
----

ACTAATGGAACCATCACACTTTCATGCAGAATAAAGCAATTTATAAACATGTGGCAGAGGGTAGGACGAGCAA
TGTATGCCCCTCCCATTGCAGGAAACATAACATGTAACTCAAGT---
ATCACAGGACTACTATTAGTACGTGATGGAGGCGATACAAATAAA----------------------------
----

AGTAACACAGAGACATTCAGACCTGAAGGAGGAAATATGAAGGACAATTGGAGAAGTGAATTATATAAATATA
AAGTAGTAGAAATTAAGCCATTG---GGAATAGCACCCACTAATGCAAAAAGGAGAGTGGTG-----------
----------------------------GAGAGAGAAAAAAGAGCA---GTGGGA---ATAGGAGCT------
---GTGTTCCTTGGGTTCTTGGGAGCA---GCAGGAAGCACTATG---GGCGCAGCGTCA---
ATAACGCTGACGGTACAG---GCCAGACAA---TTGTTG---TCCGGTATAGTGCAACAGCAAAGC---
AATTTG---CTGAGA---GCTATAGAGGCGCAACAACATCTGTTGCAACTCATGGTCTGGGGCATT
>DQ445632
TTCTATTGCAATACATCAAGTCTGTTTAATGGTACATACAAGGCTAATAGTAATAGTACAAACAGTACTTCAA
AA---

```
---------------------------
GTAAATGAAACCATCACACTCCCATGCAGAATAAAACAAATTATAAACATGTGGCAGGGGGTAGGACGCGCAA
TGTATGCCCCTCCAATTGCAGGAACCATAACATGTAAATCAAAT---
ATCACAGGAATACTATTGACACGTGATGGAGGCAGTGGCGATAAT----------------------------
-------------------------------------------------------------------------
----------
GAAACAGAAGAGATATTCAGACCTGCAGGAGGAGATATGAGGGACAATTGGAGAAGTGAATTATATAAATATA
AAGTGGTAGAAATTAAGCCATTG---GGACCAGCTCCTACTGAGGCAAAAAGGAGAGTAGTG-----------
----------------------------GAGAGAGAAAAAAGAGCA---GTGGGA---ATAGGAGCT------
---GTGTTCCTTGGGTTCTTGGGAGCA---GCAGGAAGCACTATG---GGCGCAGCATCA---
ATAACGCTGACGGTACAG---ACCAGACAA---TTGTTG---TCTGGTATAGTGCAACAGCAAAGC---
AATTTG---CTGAAG---GCTATAGAGGCGCAACAACATCTGTTGCAGCTCACGGTCTGGGGCATT
>DQ396378
TTCTATTGCAATACAACAAACCTGTTCAGCACAAGTGTTAACATAACAAATAGT-------------------
-------------------------------------------------------------------------
---------------------------
ACAAATAGTAGCATCACACTCCCATGCAGAATAAAACAAGTTATAAACATGTGGCAGGGGGTAGGTCGAGCAA
TGTATGCCCCTCCCATTGCAGGAAACATAACATGTAACTCTACT---
ATCACAGGACTACTACTGACACGAGATGGAGGACAGGGAACAGAAACC-------------------------
-------------------------------------------------------------------------
----------
AATGACACAGAGATATTCAGGCCTGGAGGAGGAGATATGAGGGACAATTGGAGAAGTGAATTATATAAATACA
AAGTGGTAGAAATTAAGCCATTG---GGACTAGCACCCACTGAGGCAAAAAGGAGAGTGGTG-----------
----------------------------GAGAGAGAAAAAAGAGCA---GTAGGA---ATAGGAGCT------
---GTACTCCTTGGGTTCTTGGGAGCA---GCAGGAAGCACTATG---GGCGCGGCGTCA---
ATAACGCTGACGGTACAG---GCCAGACAA---CTGTTG---TCTGGTATAGTGCAACAGCAAAGC---
AATTTG---CTGAGA---GCTATAGAGGCGCAACAGCATATGTTGCAACTCACGGTCTGGGGCATT
>DQ396386
TTCTATTGCAACACATCATCCCTGTTTAATAGAACATACCCGCTCAATAGTGCAGGAAACAGTTCAACAGGG-
-------------------------------------------------------------------------
---------------------------
AATAATTCAATCATCACACTCCAATGCAGAATAAAACAATTTATAAACATGTGGCAGGAGGTAGGACGAGCAA
TGTATGCCCCTCCCATTGCAGGAAACATAACATGTAAATCAAAT---
ATCACAGGACTACTATTGGTACGTGATGGAGGAACCACAAATGACTCA-------------------------
-------------------------------------------------------------------------
----------
AATAACAAAGAGACATTCAGACCTGGAGGAGGAGATATGAGGGACAATTGGAGAAGTGAATTATATAAATATA
AAGTGGTAGAAATTAAGCCATTA---GGAATAGCACCCACTAAGGCAAAAAGGAGAGTGGTG-----------
----------------------------GAGAGAGAAAAAAGAGCA---GTGGGA---ATAGGAGCT------
---GTGTTCCTTGGGTTCTTGGGAGCA---GCAGGAAGCACTATG---GGCGCAGCATCA---
ATAACGCTGACGGTACAG---GCCAGACAA---TTGCTG---TCTGGAATAGTGCAACAGCAAAGC---
AATTTG---CTGAGG---GCTATAGAGGCGCAACAGCATATGTTGCAACTCACGGTCTGGGGCATT
>DQ396365
TTCTATTGTGATACATCACAACTGTTTAATGATACATTTAATAATACAGGA----------------------
-------------------------------------------------------------------------
---------------------------
TCTAATGCTACACTTACACTCCCCTGTAGAATAAAACAAATTATAAACATGTGGCAGGGGGTAGGACGAGCAA
TGTATGCCCCTCCCATTGCAGGAAATATAACATGTAAATCGAAG---
ATCACAGGACTACTATTGACAAGTGATGGAGGAAAAGACTCAAAT----------------------------
-------------------------------------------------------------------------
-------------
GCCACAGAGACGTTCAGACCTGGGGGAGGAAATATGAGAGACAATTGGAGAAGTGAATTATATAAATATAAAG
TGGTAGAAATTAATCCATTA---GGAGTAGCTCCCACTGGAGCAAAAAGGAGAGTGGTG--------------
-------------------------GAGAGAGAAAGAAGAGCA---GTGGGA---ATAGGAGCT---------
GTGTTCCTTGGGTTCTTAGGAGCA---GCAGGAAGCACTATG---GGCGCAGCATCA---
ATAACGCTGACGGTACAG---GCCAGACAA---TTATTG---TCTGGTATAGTGCAACAGCAAAGC---
AATTTG---CTGAGG---GCTATAGAGGCGCAACAGCATATGTTGCAACTCACAGTCTGGGGCATT
>DQ445635
TTCTATTGCAATACAACAAAACTATTTTCTAATGAGAGTGAG-------------------------------
-------------------------------------------------------------------------
```

TCAAACAAAACTATCACACTCCCATGCAGGGTAAAACAAATTATAAACATGTGGCAGGGAGTAGGACGAGCAA
TGTATGCCCCTCCTATTGCAGGAAATATAACATGTAAATCAAAT---
ATCACAGGACTACTATTGACACGAGATGGAGGAAACACAGGAGAA----------------------------
----

AACATAACAGAGATATTCAGACCGGGGGGAGGAAACATGAAAGACAATTGGAGAAATGAGTTATATAAATACA
AAGTGGTAGAAATTAAGCCATTG---GGAATAGCACCCACTGAAGCAAAAAGGAGAGTGGTG-----------
----------------------------GAGAGAGAAAAAAGAGCA---GTGGGA---ATAGGAGCT------
---GTGTTTCTTGGGTTCTTGGGAGCA---GCAGGAAGCACTATG---GGCGCAGCGTCA---
GTGGCGCTGACGGTACAG---GCCAGACAA---CTGCTG---TCTGGTATAGTGCAACAGCAAAGC---
AATTTG---CTGAGA---GCTATAGAGGCGCAACAGCATATGTTGCAACTCACAGTCTGGGGCATT
>DQ396368
TTCTATTGCAATACAACAAATCTGTTCAATGAACTGGATAATAAT----------------------------
----

AGTAGTGGAAACATCACACTCCCATGCAAGATAAAGCAATTTATAAACATGTGGCAACAAGTGGGACGAGCAA
TGTATGCCCCTCCCATTGAAGGAAAAATTACATGTAACTCAAAT---
ATCACAGGAATTCTATTGACAAGGGATGGAGGGATTAATGGTACA----------------------------
----

GAAACATTCAGACCTACAGGAGGAAATATGAAGGACAATTGGAGAAATGAATTATATAAATATAAAGTAGTAG
AAATTAAGCCATTG---GGAATAGCACCCACTGAAGCAAAAAGGAGAGTGGTG--------------------
-------------------GAGAGAGAAAAAAGAGCA---GTGGGA---ATAGGAGCT---------
GTATTCTTTGGGTTCTTGGGAGCA---GCAGGAAGCACTATG---GGCGCGGCGTCA---
ATAACGCTGACGGTACAG---GCCAGACAA---CTGTTG---TCTGGTATAGTGCAACAGCAAAGC---
AATTTG---CTGAGA---GCTATAGAGGCGCAACAGCATCTGTTGCAACTCACGGTCTGGGGCATT
>DQ396370
TTCTATTGCAATACGACAAGACTGTTTAATGAAAGTAATTCAGATATA-------------------------
----

ACAAATACCACCATCACACTCCCATGCAGAATAAAACAAATTATAAATATGTGGCAGGGAGTAGGACGAGCAA
TGTATGCCCCTCCCATTGAAGGAAACATAACATGTAACTCAAGT---
ATCACAGGATTACTATTGCTACGTGATGGAGGAAACAAGAATGATACA-------------------------
----

AATGATACAAACGAGATATTCAGACCTGGAGGAGGAAATATGAAGGACAATTGGAGAAGTGAATTATATAAAT
ATAAAGTGATAGAAATTAAGCCATTG---GGGGTAGCCCCCACCGAGGCAAAAAGAAGAGTGGTG--------
-------------------------------GAGAGAGAAAAAAGAGCA---GTGGGA---ATAGGAGCT---
------GTACTCCTTGGGTTCTTGGGAGCA---GCAGGAAGCACTATG---GGCGCGGCGTCA---
ATAACGCTGACGGTACAG---GCCAGACAA---CTGTTG---TCTGGTATAGTGCAACAGCAAAGC---
AATTTG---CTGAGA---GCTATAGAGGCGCAACAGCATATGTTGCAACTCACGGTCTGGGGCATT
>DQ396369
TTCTATTGTAATACAACAAAACTGTTTAATAACACACAGTGGTTGAACCTGACA-------------------
----

AACAATGACACAATCACACTCCAATGCAGAATAAAACAAATTATAAACATGTGGCAGGAGGTAGGACGAGCAA
TGTATGCCCCTCCCATTGCAGGTAACATAACGTGTAACTCAAGT---
ATCACAGGATTACTATTGACACGTGATGGAGGCAATAACGACACA----------------------------
----

AAGAATGAGACATTCAGGCCTGCAGGAGGAAATATGAAGGACAATTGGAGAAGTGAATTATATAAATATAAAG
TGGTAGAAATTAAACCATTG---GGAGTAGCACCCACGGAGGCAAAAAGGAGAGTGGTG--------------
-------------------------GAGAGAGAAAAAAGAGCA---GTGGGA---ATGGGAGCT---------
GTGATCCTTGGGTTCTTGGGAGCA---GCAGGAAGCACTATG---GGCGCGGCGTCA---
ATAACGCTGACGGTACAG---GCCAGACAA---CTGTTG---TCTGGTATAGTGCAACAGCAAAGC---
AATTTG---CTGAAA---GCTATAGAAGCGCAACAGCATATGTTGCAACTCACGGTCTGGGGCATT
>DQ369976
TTTTATTGCAGTACATCACGACTGTTTAATAGTACAGAAAGTGCAAATAGC----------------------
----

ACTAATGGTACCATCACACTTCCATGCAGAATAAAGCAAATTATAAACATGTGGCAGAAGGTAGGGCGAGCAA
TATATGCCCCTCCCATTGCAGGAAACATAACATGTAATTCAAGT---
ATCACAGGACTAATATTGGTACGTGATGGAGGAACAAATACAACAAAT-------------------------
----

AATAATAATGAGATATTCAGACCTGAAGGAGGAGACATGAGAGATAATTGGAGAAGTGAATTATATAAATATA
AAGTGGTAGAAATTGAGCCATTA---GGAATAGCACCCACTGGGGCAAAAAGGAGAGTGGTG-----------
----------------------------GAGAGAGAAAAAAGAGCA---GTGGGA---ATAGGAGCT------
---ATGTTCCTTGGGTTCTTGGGAGCA---GCAGGAAGCACTATG---GGCGCGGCGTCA---
ATGACGCTGACGGTACAG---GCCAGACAA---CTGTTG---TCTGGTATAGTGCAGCAGCAAAGC---
AATTTG---CTGAGG---GCTATAGAGGCGCAACAGCATATGTTGCAACTCACAGTCTGGGGCATT
>DQ396390
TTCTATTGCAATACAACAAAACTATTTAATAGTACAGAACTATTTAATAGTACAATCAATAACACAAACAGA-
----

AACATCACAAACATCACAATTCCATGCAGAATAAAACAAATCATAAACATGTGGCAAAAGGTAGGGCGAGCAA
TGTATGCCAATCCCATTGCAGGAAACATAATATGTAGATCAAAT---
ATCACAGGACTACTATTAGTACGTGATGGAGGCAGTAACAGTAAC----------------------------
----

ACAACAGAAATATTCAGACCTGGAGGAGGAAATATGAAGGACAATTGGAGAAGTGAATTATATAAATATAAAG
TGGTAGAAATTAAGCCATTG---GGAATAGCACCCACTGGAGCAAAAAGGAGAGTGGTG--------------
-------------------------GAGAGAGAAAAAAGAGCA---GTGGGA---ATAGGAGCT---------
GTACTCCTTGGGTTCTTGGGAGCA---GCAGGAAGCACTATG---GGCGCAGCGTCA---
ATAGCGCTGACGGCACAG---GCCAGACAA---GTGCTG---TCTGGTATAGTGCAACAGCAAAGC---
AATTTG---CTGAGG---GCTATAGAGGCGCAACAGCATCTGTTGCAACTCACGGTCTGGGGCATT
>DQ275657
TTCTATTGCAATACAACAAACCTGTTCAATACAACAAACCTGTTCAATACAACAAACCTGTTCAATGCCACTA
CT---

AATGAAAATGATAACATCACACTCCAATGCAAAATAAAACAAATTATAAACATGTGGCAGGGAGTAGGACGAG
CAATGTATGCCCCTCCCATTGAAGGAAACATAACATGTAACTCTAAT---
ATCACAGGACTACTATTGACACGTGATGGAGGAACAAACGCAACC----------------------------
----

AATAACACAGAGATATTCAGACCTGGAGGAGGAAATATGAAGGACAATTGGAGAAATGAATTATATAAATATA
AAGTAGTAGAAGTTAAGCCATTG---GGAGTAGCACCCACCAAGGCAAAAAGGAGAGTGGTG-----------
----------------------------GAGAGAGAAAAAAGAGCA---GTGGGA---CTAGGAGCT------
---GTGTTCCTTGGGTTCTTGGGAGCA---GCAGGAAGCACTATG---GGCGCGGCGTCA---
ATAACGCTGACGGTACAG---GCCAGACAA---CTATTG---TCTGGTATAGTGCAACAGCAAAAT---
AATTTG---CTGAGG---GCTATAGAGGAGCAACAGCATATGTTGCAACTCACGGTCTGGGGCATT
>DQ275643
TTCTATTGCAATACAACAAATCTGTTCAATACAACAAATCTGTTTGGAAATACTTCAACCGCAAACATCACA-
----

AATACAACAAAAATCACACTCCCATGCAGGATAAAACAAATTATAAACATGTGGCAGGGGGTAGGACGAGCAA
TGTATGCCCCTCCCGTTGAAGGAAACATAACATGTGAGTCAAAT---
ATCACAGGACTACTATTGACACGTGATGGAGGAAAAAACACAACA----------------------------
----

GAGGAAACAGAGACTTTTAGACCTGGAGGAGGAAATATGAAAGACAATTGGAGAAGTGAATTATATAAATATA
AAGTGGTAGAAATTAAACCATTG---GGAATAGCACCCACTGAGGCAAAAAGGATAGTGGTG-----------
----------------------------GAGAGAGAAAAAAGAGCA---GTGGGA---ATAGGAGCT------
---GTGCTCCTTGGGTTCTTGGGAGCA---GCAGGAAGCACTATG---GGCGCAGCGTCA---
ATAACGCTGACGGTACAG---GCCAGACAA---CTGTTG---TCTGGTATAGTGCAGCAGCAAAGC---
AATTTG---CTGAGA---GCTATAGAGGCGCAACAGCATATGTTGCAACTCACAGTCTGGGGCATT
>DQ396380
TTCTATTGCAATACATCAGGCCTGTTTAATAGTTCCAACAATACAGCAGTTACTGAT----------------
----

AATAATGGCACTATTACACTCCAATGCAGAATAAAACAAATTATAAACATGTGGCAGAAGGTAGGACGAGCAA
TGTATGCCCCTCCCATTGCAGGAAACATAACATGTAGATCAAAC---
ATCACAGGACTACTATTGACACGTGATGGAGGAAATAATAGTGAA---------------------------
----

AATGCAGAGATATTCAGACCTGGAGGAGGAGATATGAGGGACAATTGGAGAAGTGAATTATATAAGTATAAAG
TGGTAGAAATTAAGCCATTA---GGAGTAGCACCCACTACTGCAAAGAGGAGAGTGGTG-------------
-------------------------GAGAGACAGAAAAGAGCA---GTGGGA---CTAGGAGCT--------
GCGTTCTTCGGGTTCTTGGGAGCA---GCAGGAAGCACTATG---GGCGCGGCATCG---
ATAACGCTGACGGTACAG---GCCAGACGA---TTATTG---TCTGGTATAGTGCAACAGCAAAGC---
AATTTG---CTGAAA---GCCATAGAGGCGCAACAGCATATGTTGCAACTCACGGTCTGGGGCATT
>DQ275646
TTCTATTGTAATACAACAAACCTGTTTACAGAACTGTCTAATACAACAAAGGGGGAAGCTACAAATAAA----
----

AGTGATGAAAACATCACACTCCAGTGCAGAATAAAACAAATTATAAACATGTGGCAGGGGGTAGGACGAGCAA
TGTATGCCCCTCCCATTGAAGGAAACATAACATGTAACTCGAGT---
ATCACAGGACTGCTATTGACACGTGATGGAGGAAGAAATGACACA---------------------------
----

GAGACATTCAGACCTGGAGGAGGAAATATGAAAGACAATTGGAGAAGTGAATTATATAAATATAAAGTGGTAG
AAATTAAGCCATTG---GGAATAGCACCCACTACGGCAAAAAGGAGAGTGGTG-------------------
-------------------GAGAGAGAAAAAAGAGCA---GTAGTAGGAATAGGAGCT---------
GTACTCCTTGGGTTCTTGGGAGCA---GCAGGAAGCACTATG---GGCGCGGCGTCA---
ATAACGCTGACGGTACAG---GCCAGACAA---CTGTTG---TCTGGTATAGTGCAACAGCAAAGT---
AATTTG---CTGAGG---GCTATAGAGGCGCAACAGCATATGTTGCAACTCACGGTCTGGGGCATT
>DQ351231
TTCTATTGCAATACATCACAACTGTTTAAGGGTACATACAATGGTACATACAATGGTACAGAA----------
----

AGTAATTCAACCATCACACTTCCATGCAGAATAAAACAAATTATAAACATGTGGCAGGAGGTAGGACGAGCAA
TGTATGCCCCTCCCATTGAAGGAAATATAACATGTAACTCAAGT---
ATCACAGGACTACTATTAGTACGTGATGGAGGCAACAATAACACA---------------------------
----

AACGAGACATTCAGACCTATAGGAGGAGATATGAAGGACAATTGGAGAAGTGAATTATATAAATACAAAGTGG
TAGAAATTAAGCCATTG---GGACTAGCACCCACTGGAGCAAAAAGGAGAGTGGTG-----------------
----------------------GAGAGAGAAAAAAGAGCA---GTAGGA---ATAGGAGCT---------
GTGTTCCTTGGGTTCTTGGGAGCA---GCAGGAAGCACTATG---GGCGCGGCATCA---
ATAACGCTGACGGTACAG---GCCAGACAA---CTGTTG---TCTGGTATAGTGCAACAGCAAAAT---
AATTTG---CTGAGG---GCTATAGAGGCGCAACAGCATATGTTGCAACTCACGGTCTGGGGCATT
>DQ369981
TTCTATTGCAACACATCAGGACTGTTCAATAGTATATACAGTCCTACATACAGGCCTAATGGGACA-------
----

AGTGATTCAACCATCACACTCCCATGCAGAATAAAACAGATTATAAACATGCGGCAGGGGGTAGGACGAGCAA
TGTATGCCCCTCCCATTGAAGGAAGCATAACATGTAACTCAACC---
ATCACAGGACTACTATTGACACGTGATGGAGGAAAAGCAATGAAT---------------------------
----

AACACAGAGATATTCAGACCTGGAGGAGGAAATATGAAGGACAATTGGAGAAGTGAATTATACAAATATAAAG
TGGTAGAAATTAAGCCATTG---GGGGTAGCACCCACTGGGGCAAAAGGGAGAGTGGTG-------------
-------------------------GAGAAAGAAAAAAGAGCA---GTGGGA---ATAGGAGCT---------
GTGCTCCTTGGGTTCTTGGGAGCA---GCAGGAAGCACTATG---GGCGCGGCGTCA---
ATAACGCTGACGGTACAG---GCCAGACAA---CTGTTG---TCTGGTATAGTGCAACAGCAAAAT---
AATTTG---CTGAGA---GCTATAGAGGCGCAACAGCATATGTTGCAACTCACGGTCTGGGGCATT
>DQ396392
TTCTATTGCAATACATCAGGCCTGTTTAATGGTACATATAATAGTACAAATACATATAATAGTACAAATAGTA
CT--

GAAAATTCAAACATCACACTCCCTTGCAGAATAAAACAAATTGTAAACATGTGGCAGGAGGTAGGACAAGCAA
TGTATGCCCCTCCCATTGCAGGAAACATAACATGTAAATCAAAT---
ATCACAGGATTACTCTTGACACGTGATGGCGGAAACAATACAAAT---------------------------
----

GGGACAGAGACATTCAGACCTGGAGGAGGAGATATGAAGAACAATTGGAGAAGTGAATTATATAAATATAAAG
TGGTAGAAATTAAGCCATTA---GGAGTAGCACCCACTAAGGCAAGAAGGATGGTGGTG-------------
------------------------GAGAGAGAGAAAAGAGCA---GTAGGA---ATAGGAGCT---------
GTGTTCCTTGGGTTCTTGGGAGCA---GCAGGAAGCACTATG---GGCGCGGCGTCA---
ATAACGCTGACGGTACAA---GCCAGACAA---TTATTG---TCTGGTATAGTGCAACAGCAAAGC---
AATTTG---CTGAGG---GCTATAGAGGCGCAGCAGCATATGTTGCAACTCACGGTCTGGGGCATT
>DQ396399
TTTTATTGCAATACATCAGGCCTGTTTAATAGTACACTGTTTAATAGTACTTCAAACAGTACTTCA-------
----

AACAGTACTGATATCATACTCCCATGCAGAATAAAACAAATTATAAACATGTGGCAGGAGGTAGGACGAGCAA
TGTATGCCCCTCCCATTGCAGGAAACATAACATGTAAATCAAAT---
ATCACAGGACTATTATTGACACGAGATGGAGGAAAAAACATAAGC---------------------------
----

AAAACCGAGACATTCAGACCTGGAGGAGGAAATATGAAGGACAATTGGAGAAGTGAATTATATAAATATAAAG
TAGTAGAAATTAAGCCATTA---GGAATAGCACCCACATCGGCAAAAAGGAGAGTGGTG-------------
------------------------GAGAGAGAGAAAAGAGCA---GCA------ATAGGAGCT---------
ATGCTCCTTGGGTTCTTGGGAGCA---GCCGGAAGCACTATG---GGCGCAGCGTCA---
ATAGCGCTGACGGTACAG---GCCAGACAA---CTGCTG---TCTGGTATAGTGCAACAGCAAAGC---
AATTTG---CTGAGA---GCTATAGAGGCGCAACAGCATATGTTGCAACTCACGGTCTGGGGCATT
>AY901965
TTCTATTGCAATACATCAAAGCTATTTAATAATACATACAATGGTACATACTGGATATTTAATAATAATACAT
ACTGGAAT--
-------------------AGC---
AGCACAAACGAGAACATTACAATTCCATGCAGAATAAAACAAATTATAAACATGTGGCAGGAGGTAGGACGAG
CAATGTATGCCCCTCCCATTGCAGGAAACATAACATGTAACTCAAGT---
ATCACAGGATTACTATTGACACGTGATGGAGGAGACGAAACAAAC---------------------------
----

AATACAGAAACATTCAGACCTGGAGGAGGAAATATGAAGGACAATTGGAGAAGTGAATTATATAAATATAAAG
TGGTAGAAATTAAGCCATTG---GGAATAGCACCCACTGGAGCAAAAAGGAGAGTGGTG-------------
------------------------GAGAGAGAAAAAAGAGCA---GTGGGA---ATAGGAGCT---------
GTACTCCTTGGGTTCTTGGGAGCA---GCAGGAAGCACTATG---GGCGCGGCGTCA---
ATAACGCTGACGGTACAG---GCCAGACAA---CTGTTG---TCTGGTATAGTGCAACAGCAAAGC---
AATTTG---CTGAGA---GCTATAGAGGCGCAACAGCATATGTTGCAACTCACAGTCTGGGGCATT
>DQ275660
TTCTATTGCAATACAACAGGCCTGTTTAACACAACACTGATTACTAATAGTAGTGAGGAG------------
----

TCAAACGAAACCATCACACTCCCATGCAGAATAAAACAAATTGTAAACATGTGGCAGGAGGTAGGACGAGCAA
TGTATGCCCCGCCCATTGCAGGAAACATAACATGTATATCAAAT---
ATCACAGGAATACTATTGACATGGGATGGAGGAAATCTTAGTGAAACA------------------------
----

AGCAATAGGAGTGAAATATTCAGACCTGCAGGAGGAAATATGAAGGATAATTGGAGAAGTGAATTATATAAAT
ATAAAGTAGTAGAAATTAAACCATTG---GGAGTAGCACCCACTGGGGCAAAAAGGAGAGTGGTG--------
------------------------------GAGAGAGAAAAAAGAGCA---GTGGGA---CTAGGAGCT---
------GTGTTCCTTGGGTTCTTGGGAGCA---GCAGGAAGCACTATG---GGCGCGGCGTCA---
ATCACGCTGACGGTACAG---GCCAGACAA---CTGCTG---TCTGGTATAGTGCAACAGCAAAGC---
AATTTG---CTGAGA---GCTATAGAGGCGCAACAGCATATGTTGCAACTCACGGTCTGGGGCATT
>DQ351233
TTCTATTGTAATACATCACAACTGTTTAATAATACACACTGGTTTAATAATACACACGGGATTAATAGCACAA
AA--

GGTAATGAAAACATCACACTCCCATGTAGAATAAAACAAATTATAAACATGTGGCAGAAGGTAGGACAAGCAA
TGTATGCCCCTCCCATTGCAGGAAACATAACATGTGCCTCAAAT---
ATCACAGGACTACTATTGGTACATGATGGAGGAAATGAAACAAAA---------------------------
----

GAGATATTCAGACCTGGAGGAGGAGATATGAGAGACAATTGGAGAAGTGAACTATATAAATATAAGGTGGTAG
AAGTTAAGCCATTG---GGAGTAGCACCAACCAAGGCAAGAAGGAGAGTGGTG-------------------
-------------------GAGAGAGAAAAAAGAGCAGTAGCAGGAGGACTGGGAGCT---------
TTGTTCCTTGGGTTCTTGGGAGCA---GCAGGAAGCACTATG---GGCGCAGCATCA---
ATAACGCTGACGGTACAG---GCCAGACAA---TTGTTG---TCCGGCATAGTGCAACAGCAAAGC---
AATTTG---TTGAAG---GCTATAGAGGCGCAACAGCATTTGTTGCAACTCACGGTCTGGGGCATT
>AY878063
TTCTATTGCAATACATCAAAACTGTTTAATATAACAAGTGAGTCAATCAATATAAGTAATGAC---------
----

AGTAATGTAAACATCACACTCCCATGCAGAATAAAACAAATTATAAACATGTGGCAAGGGGTAGGAAAAGCAA
TGTATGCCCCTCCCATTAAAGGAAACATAACATGTAAATCAAAT---
ATCACAGGACTACTATTGACATGGGATGGAGGACACAAAGGGAATGACACA---------------------
----

GATAAGAACACAGAAACATTCAGACCTGGAGGAGGAAATATGAAGGACAATTGGAGAAGTGAATTATATAAAT
ATAAAGTAGTAGAAATTAAGCCATTG---GGGGTAGCACCCACCAAGGCAAAAAGGAGAGTGGTG-------
------------------------------GAGAGAGAAAAAAGAGCA---GTGGGA---ATAGGAGCT---
------GTACTCCTTGGGTTCTTGGGAGCA---GCCGGAAGCACTATG---GGCGCGGCGTCA---
ATAACGCTGACGGTACAG---GCCAGACAA---CTGTTG---TCTGGTATAGTGCAACAGCAAAGC---
AATTTG---CTGAGA---GCTATAGAGGCGCAACAGCATATGTTGCAACTCACAGTCTGGGGCATT
>AY878064
TTCTACTGCAATACAACAAATCTGTTTAATACAACAGATTTGGTTAGTGCAACAAAGAAGGCTGGT------
----

CCGAATGATAACATCACACTTCCATGCAGAATAAAACAAATTATAAACATGTGGCAGGAGGTAGGACGAGCAA
TGTATGCCCCTCCCATTGAAGGAAACATAACATGTAAATCAAAT---
ATCACAGGACTATTATTGACACGTGATGGAGGAATCACAAATGAT---------------------------
----

AATAAAACAGAGATATTCAGACCTGGAGGAGGAAATATGAAAGACAATTGGGGAAATGAATTATATAAATATA
AAGTGGTAGAGATTAAGCCATTG---GGAGTAGCACCCACTAAGGCAAAAAGGAGAGTGGTG----------
---------------------------GAGAGAGAAAAAAGAGCA---GTGGGA---ATAGGAGCT------
---GTGCTCCTTGGGTTCTTGGGAGCA---GCAGGAAGCACTATG---GGCGCGGCGTCA---
ATGGCGCTGACGGTACAG---GCCAGACAG---TTGTTG---TCTGGTATAGTGCAACAGCAAAGC---
AATTTG---CTGAGG---GCTATAGAGGCGCAACAGCATATGTTGCAACTCACGGTCTGGGGCATT
>AY878065
TTCTATTGCAATACAACAAACTTGTTTAATAGGACATACACGCCTGAATACGTGTTTAATGGTACAAATAGC-
----

AATTCAAGCGTAATCACCCTCCCATGCAGGATAAAACAAATTATAAATATGTGGCAGCAGGTAGGACGAGCAA
TGTATGCTCCTCCCATTGCAGGAAACATAACATGTAACTCAACA---
ATCACAGGATTACTATTGATGCGTGATGGAGGAATAAGTAATAACACA------------------------
----

AATAAAACAGAGACATTCAGACCTGGAGGAGGAGATATGAGGGACAATTGGAGAAGTGAATTATACAAATATA
AAGTGGTAGAAATTAAGCCATTA---GGAATAGCACCTACTGGGGCAAAAAGGAGAGTGGTG----------
---------------------------GAGAGAGAAAAAAGAGCA---GTGGGA---ATAGGAGCT------
---GTGTTCCTTGGGTTCTTAGGAGCA---GCAGGAAGCACTATG---GGCGCGGCATCA---
ATAACGCTGACGGTACAG---GCCAGACAA---TTGTTG---TCTGGTATAGTGCAACAGCAAAGC---
AATTTG---CTGAGG---GCCATAGAGGCGCAACAGCATATGTTGCAACTCACGGTCTGGGGCATT
>AY878068
TTCTATTGCAATACATCAGGACTGTTTAATAGTACATACCGTCCTACATACAGGCAGAATGGACAGAC----
----

AATAATTCAACCATCACACTGCCATGCAGAATAAAACAAATTATAAACATGTGGCAGGAGGTAGGACGAGCAA
TGTATGCCCCTCCTATTGCAGGAAAAATAACATGTAACTCAAGT---
ATCACAGGACTACTATTGACACGTGATGGAGGAACCAATAACACAGAG-------------------------
----

AATAACACAGAGATATTCAGACCTGGAGGAGGAAATATGAAGGACAATTGGAGAAGTGAATTATATAAATATA
AAGTGGTAGAAATTAAGCCACTA---GGAATAGCACCCACTACGGCAAAAAGGAGAGTGGTG-----------
----------------------------GAGAGAGAAAAAAGAGCA---GTGGGA---CTAGGAGCT------
---TTGTTCCTTGGGTTCTTGGGAGTA---GCAGGAAGCACTATG---GGCGCGGCGTCA---
ATAACGCTGACGGTACAG---GCCAGACAA---CTGTTG---TCTGGTATAGTGCAACAGCAAAAT---
AATTTG---CTGAGA---GCTATAGAGGCGCAACAGCATATGTTGCAACTCACGGTCGGGGGCATT
>AY878069
TTCTATTGTAATACATCAAAGCTGTTTAATAGTACAGAGCTGTTTAATAGTACAGAA----------------
----

AATAATTCAACAATCACACTCCCATGCAGGATAAAACAGATTATAAACATGTGGCAACAGGTAGGACGAGCAA
TGTATGCTCCTCCCATTGCAGGAAACATAACATGTAACTCAAGT---
ATCACAGGATTACTATTAGTGCGTGATGGAGGAACAAATGCCACA----------------------------
----

AATGAGACATTCAGACCTGGAGGAGGAGATATGAGGGACAATTGGAGAAGTGAATTATACAAGTATAAAGTGG
TAGAAATTAAACCATTG---GGAATAGCTCCAACTGATGCAAGAAGGAGAGTGGTG-----------------
----------------------GAGAGAGAAAAAAGAGCA---GTGGGC---TTTGGAGCT---------
TTGATCATTGGGTTCTTGGGAGCA---GCAGGAAGCACTATG---GGCGCGGCGTCA---
ATAACGCTGACGGTACAG---GCCAGACAA---CTGTTG---TCTGGTATAGTGCAACAGCAAAGC---
AATTTG---CTGAGG---GCCATAGAGGCGCAACAGCATATGTTGCAACTCACGGTCTGGGGCATT
>AY901967
TTCTATTGCGATACAACAAACCTGTTTAATCTGACTAAAAAAGACAGAACCATCAAGGATGGA----------
----

AATGAAACCATCATCACACTCCCATGCAGAATAAAACAAATTGTAAGGATGTGGCAGGGGGTAGGACAAGCAA
TGTATGCCCCTCCCATTGCAGGAAACATAACATGTAGATCAAAT---
ATCACAGGACTACTATTGACACGTGATGGAGGAAAAACAAATGGCACAGGGAAC-------------------
----

AATAAAACAGAGACAGAGATATTCAGACCTGCAGGAGGGAATATGAAGGACAATTGGAGAAGTGAATTATACA
AATATAAAGTGGTAGAAATTAAGCCATTG---GGAATAGCACCCGCCAAGGCAAAAAGGAGAGTGGTG-----
----------------------------------GAGAGAGAAAAAAGAGCA---GTGGGA---
ATGGGAGCT---------GTACTCCTTGGGTTCTTGGGAGCA---GCTGGAAGCACTATG---
GGCGCGGCGTCA---ATCACGCTGACGGTACAG---GCCAGACAA---GTGCTG---
TCTGGTATAGTGCAACAGCAAAGT---AATTTG---CTGAGA---
GCTATAGAGGCGCAACAGCATATGTTGCAACTCACGGTCTGGGGCATT
>AY901968
TTCTATTGCAATACAACACAACTGTTTAATGATAGTGTGGCAAAAGACAAC----------------------
----

AACACTAACATCACTACTATCCCATGCAAGATAAAACAAATTGTAAACATGTGGCAGGGGGTAGGACAAGCAA
TGTATGCCCCTCCCATTGCAGGAAACATAACATGTGAGTCAAAT---
ATCACAGGACTATTATTGACACGTGATGGAGGAAATGCAGGAAAAAAATACT---------------------
----

ACAGATAATACAGAGATATTCAGACCTGGAGGAGGAAATATGAAGGACAATTGGAGAAGTGAATTATATAAAT
ATAAAGTGGTAGAAATTAATCCATTG---GGAGTAGCACCCACTGAGGCAAAAAGGAGAGTGGTG--------
-------------------------------GAGAGAGAAAAAAGAGCA---GTGGGA---CTAGGAGCT---
------GTGTTCCTTGGGTTCTTGGGAGCA---GCAGGAAGCACTATG---GGCGCGGCGTCA---
ATAACGCTGACGGTACAG---GCCAGACAA---CTGTTG---TCTGGTATAGTGCAACAGCAAAGC---
AATTTG---CTGAGA---GCTATAGAGGCGCAACAGCATATGTTGCAACTCACAGTCTGGGGCATT
>DQ164113

TTCTATTGCAATACAACATACCTGTTTAATAATACAAGTGAT-------------------------------
----

CTAAATAACACCATCACACTCCCATGCAGAATAAAACAAATTATAAACATGTGGCGGGAGGTAGGGAGAGCAA
TGTATGCCCCTCCCATTGCAGGAAACATAACATGTAACTCAAGT---
ATCACAGGACTACTATTGACACGTGATGGAGGAAGCACAAACGACACGAAT----------------------
----

AATACAAACCCAGAGATATTCAGACCTGGAGGAGGAAACATGAAAGACAATTGGAGAAGTGAATTATATAAAT
ATAAAGTGGTAGAAATTAAGCCATTG---GGAGTAGCACCCACGAAAGCAAAAAGGAGAGTGGTG--------
-------------------------------CAGAGAGAAAAAAGAGCA---GTGGGA---ATGGGAGCT---
------GTGCTCCTTGGGTTCTTGGGAGCA---GCAGGAAGCACTATG---GGCGCGGCGTCA---
ATAACGCTGACGGTACAG---GCCAGACAA---CTGTTG---TCTGGTATAGTGCAACAGCAAAGC---
AATTTG---CTGAGA---GCTATAGAGGCGCAACAACATATGTTGCAACTCACGGTCTGGGGCATT
>AY901969
TTCTATTGCAATACATCAAAACTGTTTAATAGTACATATAATAGTACATATAATAGTAACAGTACAAATAGTA
AT---

TCAAACTCAACCATCACACTCCAATGCGGAATAAAACAAATTATAAACATGTGGCAGGGGGTAGGACAAGCAA
TGTACGCCCCTCCTATTGCAGGAAACATAACCTGTATATCAAAT---
ATCACAGGACTACTATTGACACGTGATGGAGGAGAAAACAGGTCA----------------------------
----

GAGGAGACATTCAGACCTGGAGGAGGAGATATGAGGGACAATTGGAGAAGTGAATTATATAAATATAAAGTGG
TGGAAATTAAACCATTA---GGAATAGCACCCACTAAGGCAAAAAGAAGAGTGGTG-----------------
----------------------GAGAGAGAAAAAAGAGCA---GTGGGA---ATAGGAGCT---------
GTGTTCCTTGGGTTCTTGGGAGCA---GCAGGAAGCACTATG---GGCGCGGCATCA---
CTGACGCTGACGGTACAG---GCCAGACAA---TTATTG---TCTGGTATAGTGCAACAGCAAAGC---
AATTTG---CTGAGA---GCTATAGAGGCGCAACAGCATATGTTGCAGCTCACAGTCTGGGGCATT
>DQ093593
TTCTATTGCAATACATCACAACTGTTTAACAGTACAGATAACAGTACACACATTGAAACAGGTAAGGCAAACA
TCACA--

GAAAGTGATGCAAACATCACACTGCCATGCAGAATAAAGCAAATTATAAACATGTGGCAGGAGGTAGGACGAG
CAATGTACGCCCCTCCCATTGCAGGAAACATAATATGTAACTCAAGT---
ATCACAGGACTCCTATTGACACGAGATGGAGGAAAGGGAGAAAACACA-------------------------
----

ACTAACATGACAGAGACATTCAGACCTGGAGGAGGAAATATGAAGGACAATTGGAGAAATGAATTATATAAAT
ATAAAGTGGTAGAAATTAAACCATTG---GGAATAGCACCCACTAAAGCAAAAAGGAGAGTGGTG--------
-------------------------------GAGAGAGAAAAAAGAGCA---GTGGGA---ATAGGAGCT---
------GTGCTCTTTGGGTTCTTGGGAGCA---GCAGGAAGCACTATG---GGCGCGGCGTCA---
ATAACGCTGACGGTACAG---GCCAGACAA---CTGTTG---TCTGGTATAGTGCAACAGCAAAGT---
AATTTG---CTGAGG---GCTATAGAGGCGCAACAGCATATGTTGCAACTCACGGTCTGGGGCATT
>AY901970
TTCTATTGTGATACAACAAAACTATTTAATGAAACATTATTTAATGATACAACAGAACTAGTTAATGGCACAA
CA---

AGTAATTCAAGCAACATCACACTCCCCTGCAGGATAAAACAAATTATAAACATGTGGCAGCAGGTAGGACGAG
CAATGTATGCTCCTCCCATTGCAGGAAACATAACATGTAACTCAAAT---
ATCACAGGATTACTATTGGTGCGTGATGGAGGAACACATAACAAAACAGAGACA------------AAT----
----
-
ACCACAGAAAATGAAACAGAGACATTCAGACCTGGAGGAGGAGATATGAGGGACAATTGGAGAAGTGAATTAT
ACAAATATAAAGTGGTAGAAATTAAACCATTA---GGAATAGCACCTACCAAGGCAAAACGGAGAGTGGTG--
-------------------------------------GAGAGAGAAAAAAGA---------GGA---
CTAGGAGCT---------GTGATCTTTGGGTTCTTGGGAGCA---GCAGGAAGCACTATG---
GGCGCAGCGTCA---ATAGCGCTGACGGCACAG---GCCAGACAA---GTGTTG---
TCTGGTATAGTGCAACAGCAAAGC---AATTTG---CTGAGG---
GCCATAGAGGCGCAACAGCATATGTTGCAACTCACGGTCTGGGGCATT

>AY901981
TTCTATTGTAATACAACAAAACTGTTTGATGATAGTGAAAGGATAAACACG----------------------
----

ACAACCACAACCATCATACTCCCATGCAGAATAAAACAAATTGTAAACATGTGGCAGGGGGTAGGACGAGCAA
TGTATGCCCCGCCCATTGAAGGAAACATAACATGTAAATCAAAT---
ATCACAGGATTACTATTGACACGTGATGGAGGAAACAATGGAACA----------------------------
----

AATGAGACATTCAGACCTGAAGGAGGAAATATGAAGGACAATTGGAGAAGTGAATTATATAAATATAAAGTAG
TGGAAATTAAGCCATTG---GGAGTAGCACCCACTGAGTCAAAAAGAAGAGTGGTG-----------------
----------------------GAGAGAGAAAAAAGAGCA---TTGGGA---ATAGGAGCC---------
GTGTTCCTTGGGTTCTTAGGAGCA---GCAGGAAGCACTATG---GGCGCGGCGTCA---
ATCACGCTGACGGTACAG---GCCAGACAA---GTGTTG---TCTGGTATAGTGCAACAGCAAAGT---
AATTTG---CTGAGA---GCTATAGAGGCGCAACAGCATATGTTGCAACTCACGGTCTGGGGCATT
>AY878057
TTCTATTGCAATACAACAAACCTGTTTAACATTAATGGAAGTGGAAGT-------------------------
----

AGTGGTAACAGCACAACACTCCCATGCAGGATAAAACAAATTATAAACATGTGGCAGGGGGTAGGACGAGCAA
TGTATGCCCCTCCCATAGAAGGAAACATAACATGTATATCAAAT---
ATCACAGGACTACTATTGACACGTGATGGAGGATACAATGTAACAGGAGAG----------------------
----

AATAATAAATCAACAGAGATATTCAGACCAGGAGGAGGAAATATGAAGGACAATTGGAGAAATGAATTATATA
AATATAAAGTGGTAGAAATTAAGCCATTG---GGAGTAGCACCCACTGAGGCAAAAAGGAGAGTGGTG-----
----------------------------------GAGAGAGAAAAAAGAGCA---GTGGGA---
ATAGGAGCT---------GTGTTCCTTGGGTTCTTGGGAGCG---GCAGGAAGCACTATG---
GGCGCGGCGTCA---ATAACGCTGACGGTACAG---GCCAGACAA---CTATTG---
TCTGGTATAGTGCAACAGCAAAGC---AATTTG---CTAAGA---
GCTATAGAGGCGCAACAGCACATGTTGCAACTCACGGTCTGGGGCATT
>AY878060
TTCTATTGCAATACACCAGGACTGTTTAATGGGACATACAATGAGAGTAGTGAAATA----------------
----

AGTAATGATACCATCAGACTCCCATGCAGAATAAAACAAATTATAAACATGTGGCAAGGGGTAGGACGAGCAA
TGTATGCCCCTCCCATTGCAGGGAACATAACTTGTAGCTCAAGT---
ATCACAGGACTACTATTGACACGGGATGGAGGAAAGGATAATGATAAT-------------------------
----

AATGGGAATGAGACATTCAGACCTGGAGGAGGAAATATGAAGGACAATTGGAGAAGTGAATTATATAAATATA
AAGTGGTAGAAATTAAGCCATTG---GGAATAGCACCCACTGGGGCAAAAAGGAGAGTGGTG-----------
----------------------------GAAAGAGAAAAAAGAGCA---GTGGGA---ATAGGAGCT------
---GTGTTCCTTGGGTTCTTGGGAGCT---GCAGGAAGCACTATG---GGCGCGGCGTCA---
ATAACGCTGACGGTACAG---GCCAGACAA---CTGTTG---TCTGGTATAGTGCAACAGCAAAGC---
AATTTG---CTGAGA---GCTATAGAGGCGCAACAGCATATGTTGCAACTCACGGTCTGGGGCATT
>AY878061
TTCTATTGCAATACATCAGAGCTGTTTAATAATAGTACAAGTAATGCAACTACA-------------------
----

AGTAATGCAACTATTACACTCCCATGCAGAATAAAACAAATTATAAACATGTGGCAGGGGGTAGGACGAGCAA
TGTATGCCCCTCCCATTGCAGGAAACATAACATGTAACTCAAGT---
ATCACAGGACTACTATTGACACGTGATGGAGCAATTAACAATACA----------------------------
----

GAGACATTCAGACCTGGAGGAGGAGATATGAGGGACAATTGGAGAAGTGAATTATATAAATATAAAGTGGTAG
AAATTAAACCATTA---GGAGTAGCACCCACTGATGCAAAAAGGAGAGTGGTG--------------------
-------------------GAGAGAGGAAAAAGAACA---GTGGGA---ATAGGAGCT---------
GTGTTCCTTGGGTTCTTGGGAGCA---GCAGGAAGCACTATG---GGCGCGGCGGCA---

GTAACGCTGACGGTACAG---GCCAGACAA---CTATTG---TCTGGTATAGTGCAACAGCAAAGC---
AATTTG---CTGAGA---GCTATAGAGGCGCAACAGCACATGTTGCAACTCACGGTCTGGGGCATT
>DQ396395
TTCTATTGCAATACAACAAAACTGTTTTCTTCTGAGTGGTTAAACAACACCAAGGAA----------------
----

AACATCACAACCATCACACTCCAATGCAGGATAAAACAATTTATAAACATGTGGCAGAAGGTAGGAAGAGCAA
TGTATAACCCTCCTATTGAAGGAAACATAACATGTAAATCAAAT---
ATCACAGGGCTATTATTGACAAGGGATGGAGGCGAAGGAGACAATACA-------------------------
----

AGAACAACAGAGATATTCAGACCTACAGGAGGAAATATGAAGGATAATTGGAGAAGTGAATTATATAAATATA
AGGTGGTAGAAATTAAGCCATTG---GGAGTAGCTCCCACTGAATCAAAAAGGAGAGTGGTG-----------
----------------------------GAGAGAGAAAAAAGAGCA---GTGGGA---ATAGGAGCT-------
---GTACTCCTTGGGTTCTTGGGAGCA---GCAGGAAGCACTATG---GGCGCGGCGTCA---
ATAACGCTGACGGTACAG---GCCAGACAA---CTGTTG---TCTGGTATAGTGCAACAGCAAAGC---
AATTTG---CTGAGA---GCCATAGAGGCGCAACAGCATATGTTGCAACTCACAGTCTGGGGCATT
>DQ056410
TTCTATTGCAATACATCAGGACTATTTAATGGTACATACAATAGTACAGAAAGTAATTCA-------------
----

ACAGATAAAAACACCACAATGCAATGCAGAATAAAACAAATTATAAACATGTGGCAGGGGGTAGGACGAGCAA
TGTATGCCCCTCCCATTGCAGGAAACATAACATGTAAATCAAAC---
ATCACAGGACTACTATTAGTACGTGATGGAGGCAACGACAACAAAACT-------------------------
----

AATGACACAGAAACATTCAGACCTGGAGGAGGAGATATGAGGGACAATTGGAGAAGTGAATTATATAAATATA
AAGTGGTAGAAATTAAGCCATTG---GGAATAGCTCCAACTGAAGCAAGAAGGAGAGTGGTG-----------
----------------------------GAGAGAGAGAAAAGAGCA---GTAGGA---ATAGGAGCG-------
---TTGATCCTTGGGTTCTTGGGAGCA---GCAGGAAGCACTATG---GGCGCGGCGTCA---
ATAGCGCTGACGGCACAG---GCCAGACAA---GTGCTG---TCTGGTATAGTGCAACAGCAAAAC---
AATTTG---CTGAGA---GCTATAGAGGCACAACAGCATCTGTTGCAACTCACAGTCTGGGGCATT
>DQ056411
TTCTACTGTAATACAACAGAACTGTTTACAAACAATATCACAACAAATGGCACCAACACA-------------
----

ACAAATGGCAACATCACCATCCCATGCAGGATAAAACAAATTGTAAACATGTGGCAGCAGGTAGGACGAGCAA
TGTATGCCCCTCCCATTGCAGGAAACATAACATGTAGATCAAAT---
ATTACAGGACTACTATTGACACGTGATGGTGGTATCACAAATGGC----------------------------
----

ACAGAGACATTCAGACCTGAAGGAGGAAATATGAAGGACAATTGGAGAAGTGAATTATATAAATATAAAGTGG
TAGAACTTAAGCCATTG---GGAATAGCACCCACCAAGGCAAAAAGGAGAGTGGTG-----------------
----------------------GAGAGAGGAAAGAGAGCA---GTGGGA---ATAGGAGCC----------
TTGTTCCTTGGGCTCTTGGGAGTA---GCAGGAAGCACTATG---GGCGCGGCGTCA---
GTGACGCTGACGGCACAG---GCCAGACAA---CTGTTG---TCTGGTATAGTGCAACAGCAAAAC---
AATTTG---CTGAGA---GCTATAGAGGCGCAACAACATATGTTGCAACTCACGGTCTGGGGCATT
>DQ056404
TTCTATTGCAATGCATCACAACTGTTTAACGAGACACTGCTTAACGAGACTATGACTAATGGTACA-------
----

ACCAATGCAACCATCACACTCCCATGCAGAATAAAACAAATTATAAACATGTGGCAGGAGGTAGGACGAGCAA
TATATGCCCCTCCCATTGAAGGAAACATAACATGTACATCAAAT---
ATCACAGGACTACTATTGGTACGTGATGGAGGAAATGATGGAGGAAAT-------------------------
----

AACACACAGACCGAGACATTCAGACCTGGAGGAGGAGATATGAGGAACAATTGGAGAAGTGAATTATATAAAT
ATAAAGTGGTAGAAATTAAGCCACTG---GGAGTAGCACCCACTGAGGCAAAAAGGAGAGTGGTG--------
-------------------------------GAGAGAGAAAAAAGAGCA---GCA------CTAGGAGCT---
------ATGCTCTTTGGGTTCTTGGGAGCA---GCCGGAAGCACTATG---GGCGCAGCATCA---

ATAACGCTGACGGTACAG---GCCAGACAA---TTGTTG---TCTGGAATAGTGCAACAGCAAAGC---
AATTTG---CTGAGG---GCTATAGAGGCGCAACAGCATATGTTGCAACTGACGGTCTGGGGCATT
>DQ351237
TTCTATTGCAATACATCAGGCCTGTTTAATGTTAATAGTAGTACACTTAAAAACAGTACAGGT----------
----

GCTAATGGTACAATCACACTCCAATGCAGAATAAAACAAATTATAAACATGTGGCAGAAGGTAGGACGAGCAA
TGTATGCCCCTCCCATTGCGGGAAATATAACATGTAACTCAACT---
ATCACAGGACTCCTATTGACACGCGATGGAGGAACAGGAGAAGAA----------------------------
----

AATACTACAGAGACATTCAGACCTGGAGGAGGAGATATGAGGGACAATTGGAGAAGTGAATTATATAAATATA
AAGTGGTAGAAGTTAAGCCATTA---GGAATAGCACCCACTACTGCAAAAAGGAGAGTGGTG-----------
----------------------------GAGAGAGAGAAAAGAGCA---GTGGGA---ATAGGAGCT------
---ATGTTCCTTGGGATCTTGGGAGCA---GCAGGAAGCACTATG---GGCGCAGCATCA---
ATAACGCTGACGGTACAG---GCCAGACAA---TTATTG---TCTGGTATAGTGCAACAGCAAAGC---
AATTTG---CTGAGA---GCTATAGAGGCGCAACAGCATATGTTGCAACTCACGGTCTGGGGCATA
>DQ056408
TTCTATTGTAATACAACAAAGCTGTTTAATAGTACATACATGCCTAATGGTACATACATGTACATGCCTAATG
ATACAGAA---
-------------------AGT---
AATTCAAGTGAAGTAATCACACTCCCATGCAGGATAAAACAAATTATAAACATGTGGCAGCAGGTAGGACGAG
CAATGTATGCTCCTCCCATTGCAGGGAACATAACATGCATATCAAAT---
ATCACAGGATTACTATTGGTGAGAGATGGAGGAAAAGGAAATAGCACAGATAAT-------------------
----
-
AAAGAGGCAGAAAAACCAGAGATATTCAGACCTGAAGGAGGAGATATGAGAAACAATTGGAGAAGTGAATTAT
ACAAATATAAAGTAGTAGAAGTTAAACCATTG---GGAATAGCACCTACTAAGGCAAAAAGAAGAGTGGTG--
-------------------------------------GAGAGAGAAAAAAGAGCA---GTGGGA---
CTAGGAGCT---------GTGTTCCTTGGGTTCTTGGGAGCA---GCAGGAAGCACTATG---
GGCGCGGCGTCA---ATAACGCTGACGGTACAG---GCCAGACAA---TTGTTG---
TCTGGTATAGTGCAACAGCAAAGC---AATTTG---CTGAGG---
GCCATAGAGGCGCAACAGCATATGTTGCAACTCGCGGTCTGGGGCATT
>DQ011169
TTCTATTGCAATACAACAGGTCTGTTTAGTAATAATGGAAGTACC----------------------------
----

CAAAATGAGACCATCACACTCCCATGCAGAATAAAACAAATTGTAAACATGTGGCAGGGGGTAGGACGAGCAA
TGTATGCCCCTCCCATTGCAGGAACCATAACATGTAAATCAAAT---
ATCACAGGATTATTATTGCTACGTGATGGAGGAGACACCAGCACG----------------------------
----

AATGGCACAGAGATATTCAGACCTGGAGGAGGAAATATGAAAGACAATTGGAGAAGTGAATTATATAAATATA
AAGTGGTAGAAATTAAGCCATTA---GGAGTAGCACCCACGACGGCAAAAAGGAGAGTGGTG-----------
----------------------------GAGAGAGAAAAAAGAGCA---GTGGGA---ATAGGAGCT------
---GTGCTCTTTGGGTTCTTGGGAGCA---GCAGGAAGCACTATG---GGCGCGGCGTCA---
ATAACGCTGACGGTACAG---GCCAGACAA---CTGTTG---TCTGGTATAGTGCAACAGCAAAGC---
AATTTG---CTGAGA---GCTATAGAGGCGCAACAGCATATGTTGCAACTCACGGTCTGGGGCATT
>DQ011176
TTCTATTGCAATACAACAAAACTGTTTAATGCAACAGACAAAGACAATATAACACAATGGGCCAACACAACAA
AC---

AGTACTGAAACCATCACAATCCCATGCAGAATAAAACAAATTGTAAACATGTGGCAGGGGGTAGGACGAGCAA
TGTATGCCCCTCCCATTGCAGGAAACATAACATGTAAATCAAAT---
ATCACAGGACTACTACTGACATGGGATGGTCCGTATAACAAAAGC----------------------------
----

AGTACGAATGAGACATTCAGACCTGGAGGAGGAAATATGAAGGACAATTGGAGAAGTGAATTATATAAATATA
AAGTGGTAGAAATTAAGCCATTG---GGAGTAGCACCCACTGAGGCAAAAAGGAGAGTGGTG-----------
----------------------------GGGAGAGAAAAAAGAGCA---GCA------CTAGGAGCT------

---TTGCTCCTTGGGTTCTTGGGAGCA---GCAGGAAGCACTATG---GGCGCGGCGTCA---
ATAACGCTGACGGTACAT---GCCAAACAA---CTGATG---GCTGGTATAGTGCAACAGCAAAGC---
AATTTG---CTGAGA---GCTATAGAGGCGCAACAGCATATGTTGCAACTCACAGTTTGGGGCATT
>DQ093601
TTCTATTGCAATACAACAAAACTGTTTTCTAATTCTAATGACAGTGGTTCAAAC-------------------
----

ACAACTACCATAACCACCATCCCATGTAGGATAAAACAAATTGTAAACTTGTGGCAGGGGGTAGGAAGAGCAA
TGTATGCCCCTCCCATTGAAGGGAACATAACATGTAACTCAAGT---
ATCACAGGATTACTATTGACACGTGATGGTGGCTTCAACAACACA----------------------------
----

ACAGAGATATTCAGACCTGAAGGAGGAAATATGAAGGACAATTGGAGAAGTGAATTATATAAATATAAAGTGG
TAGAAATTAAGCCATTG---GGAGTAGCACCCACTAAGGCAAAAAGGAGAGTGGTG-----------------
----------------------GAGAGAGAAAAGAGAGCA---GTGGGG---ATAGGAGCT---------
TTGTTCCTTGGGTTCTTGGGAGCA---GCAGGAAGCACTATG---GGCGCGGCGTCA---
ATGACGCTGACGGTACAG---GCCAGACAA---CTGTTG---TCCGGTATAGTGCAACAGCAAAAC---
CATTTG---CTGAGA---GCCATAGAGGCGCAACAGCATATGTTGCAACTCACGGTCTGGGGCATT
>DQ093596
TTCTATTGCAATACATCAAACCTGTTTAGTGACATGTATAAT-------------------------------
----

GATACCATCACACTCCCATGCAAAATAAAGCAAATCATAAACATGTGGCAGGAGGTAGGACGAGCAATGTATG
CCCCTCCCATTGCAGGAAACATAACATGTAATTCAAGT---
ATCACAGGATTACTACTGACACGTGATGGAGGCACGAATGGAGGCACG-------------------------
----

AATGACACCACAGAAACATTCAGACTTGGAGGAGGAAATATGAAGGACAACTGGAGAAGTGAATTATATAAGT
ACAAGGTGGTAGAAATTAAGCCATTA---GGAGTAGCACCCACTGGAGCAAAAAGGAGAGTGGTG--------
-------------------------------GAGAGAGAAAAAAGAGCA---GTGGGA---ATGGGAGCT---
------GTGCTCCTTGGGTTCTTGGGAGCA---GCAGGAAGCACTATG---GGCGCGGCGTCA---
ATAACGCTGACGGTACAG---GCCAGACAA---CTGTTG---TCTGGTATAGTGCAACAGCAAAGC---
AATTTG---CTGAGA---GCTATAGAGGCGCAACAGCATCTGTTGCAACTCACGGTCTGGGGCATT
>DQ093607
TTCTATTGTGACACATTAAAACTGTTTAATAATAGTCTGGTTAATGATAGTGCT-------------------
----

ACAAACGACTCAATCACCATCCCATGCAGAATAAAGCAAATTATAAATATGTGGCAAAAGGTAGGACGAGCAA
TGTATGCCCCTCCCATTGCAGGAAACATAACATGTAGATCAAAT---
ATCACAGGACTACTATTGACACGTGATGGAGGAACAATTAATGCAACT-------------------------
----

GAAAATAATATAGAGACATTCAGACCTGGAGGAGGAGATGTGAGGGACAATTGGAGAAGTGAATTATATAAAT
ATAAAGTGGTAGAAATTAAGCCATTA---GGAATAGCACCCACTGAAGCAAAAAGGAGAGTGGTG--------
-------------------------------GAGAGAGAAAAAAGAGCA---GTGGGA---ATAGGAGCC---
------GTGTTCCTTGGGTTCTTGGGAGCA---GCAGGAAGCACTATG---GGCGCAGCGTCA---
ATAACGCTGACGGTACAG---GCCAGACAA---CTATTG---TCTGGTATAGTGCAACAGCAAAGC---
AATTTG---CTGAGG---GCTATAGAGGCGCAACAGCATATGTTGCAACTCACGGTCTGGGACATT
>DQ164108
TTCTATTGCAATACATCAGCACTGTTTAATGGTACATACAATGGGACAGAAAGTAATTCA-------------
----

ACTGAGTCAAACATCACACTTCCATGCAGAATAAAACAAATTGTAAACATGTGGCAGGAGGTAGGACGAGCAA
TGTATGCCCCTCCCATTGAAGGAATAATAACATGTAACTCAAGT---
ATCACAGGACTACTATTGACACGTGATGGAGGACAAACAAACAAAACA-------------------------
----

AATGGCACAGAGATATTCAGACCTGGAGGAGGAAATATGAAGGACAATTGGAGAAATGAATTGTATAAATATA
AAGTGGTAGAAATTAAGCCATTG---GGAATAGCACCCTCTAAGGCAAAAAGGAGAGTGGTG-----------
----------------------------GAGAGAGAAAAAAGAGCA---GCAGGA---ATGGGAGCT------

---GTGCTCCTTGGGTTCTTGGGAGCA---GCAGGAAGCACTATG---GGCGCGGCGTCA---
ATAACGCTGACGGTACAG---GCCAGACAA---CTGTTG---TCTGGTATAGTGCAACAGCAAAAT---
AATTTG---CTGAGG---GCTATTGAAGCGCAACAGCATATGTTGCAACTCACGGTCTGGGGCATT
>DQ351234
TTCTATTGCAACACATCAAAACTGTTTAATAGTACCATGTCTAATGGTACCATGTTTAATAGTACAGGA----
----

AATAATTCAGTCATCACACTCCAATGTAGAATAAAACAAATTATAAATATGTGGCAGGAGGTAGGACGAGCAA
TGTATGCCCCTCCCATTGCAGGAACCATCACATGTAACTCTAAT---
ATCACAGGACTACTATTGACACGTGATGGTGGAAATAATGAGACA----------------------------
----

GAAACGTTCAGACCTGAAGGAGGAGATATGAGGGATAATTGGAGAAGTGAATTATATAAATATAAAGTGGTAG
AAATTAAGCCATTA---GGAGTAGCACCCACTGAGGCAAAAAGGAGAGTGGTG--------------------
-------------------GAGAGAGAAAAAAGAGCA---GTGGGA---ATGGGAGCT---------
GTGTTCCTTGGGTTCTTGGGAGCA---GCAGGAAGCACTATG---GGCGCAGCGGCA---
GTGACGCTGACGGTACAG---GCCAGACAA---TTGCTG---TCTGGAATAGTGCAACAGCAAAGC---
AATTTG---CTGAGA---GCTATAGAGGCGCAACAGCATATGTTGAAACTCACGGTCTGGGGCATT
>DQ275650
TTTTATTGCAATACATCAGCACTATTTAATAGTTCGTTTAATGGTACATACATGAACAGTACAGAA-------
----

GGTAATTCAAACATCACAATCCCACGCAGAATAAAACAAATTATAAACATGTGGCAGGAGGTGGGAAAAGCAA
TGTATGCCCCTCCCATTGCAGGAAACATAACATGTAAATCAAAT---
ATCACAGGACTACTATTGATACGTGATGGAGGAACAGGCAATGAAATA-------------------------
----

GACAATACAACAGAGACATTCAGACCTGGAGGAGGAGATATGAGGGACAATTGGAGAAGTGAATTATATAAAT
ATAAAGTGGTAGAAATTAAGCCATTA---GGAATAGCACCCACTGAGGCAAAAAGGAGAGTGGTG--------
-------------------------------CAGAGGGAGAAAAGAGCA---GTGGGA---ATAGGAGCT---
------GTGCTCCTTGGGTTCTTGGGAGCA---GCAGGAAGCACTATG---GGCGCAGCATCA---
ATAACGCTGACGGTACAG---GCCAGACAA---TTATTG---TCTGGTATAGTGCAACAGCAAAGC---
AATTTG---CTGAGA---GCTATAGAGGCGCAACAACATATGTTGCAACTCACAGTCTGGGGCATT
>KT183301
TTCTATTGTAATACATCAAGACTATTTAATGGTAATGGTACACACACACAAGATAATAGTACAAAAGAG----
----

ACCAACAAGACCATCACAATTACATGCAAAATAAAACAAATAATAAATATGTGGCAAGAGGTAGGACGAGCAA
TGTATGCCCCTCCCATTGCAGGAAACATAACATGTAAATCAAAT---
ATCACAGGACTACTGTTGACACGTGATGGAGGAGGAAATAGTACT----------------------------
----

GATGAGATCTTCAGACCTGGAGGAGGAGATATGAGAGACAATTGGAGAAGTGAATTATATAAATATAAAGTGG
TAGAAATTAAACCATTA---GGAATAGCACCCACTAGTGCAAAAAGGAGAGTGGTG-----------------
----------------------GAGAGAGAAAAAAGAGCA---GTGGGA---ATAGGAGCT---------
GTGTTCCTTGGGTTCTTGGGAACA---GCAGGAAGCACTATG---GGCGCGGCGTCA---
ATAACGCTGACGGTACAG---GCCAGACAA---TTATTG---TCTGGTATAGTGCAGCAGCAAAGC---
AATTTG---CTGAGA---GCCATAGAGGCGCAACAGCATCTGTTGCAACTCACGGTCTGGGGCATT
>KC863229
TTCTATTGCAATACATCAAAACTGTTTAATAAGAATGGTACA-------------------------------
----

GAAAAGAATACAACCATCACACTCCCATGCAGAATAAAACAAATTATAAACATGTGGCAGGAGGTAGGACGAG
CAATATATGCCCCTCCCATTAAAGGAAACATAACATGTAGCTCAAAC---
ATCACAGGACTACTATTGACACGGGATGGAGGAGAAACAAACATAAAAAACACA-------------------
----
-
GAGACAGACAATACCACAGAGACATTCAGACCTGAAGGAGGAGATATGAGAAATAATTGGAGAAGTGAATTGT
ATAAATATAAAGTGGTAGAAGTTAAGCCACTA---GGAGTAGCACCTACTGAGGCAAAAAGGAGAGTGGTG--
-------------------------------------GAGAGAGAAAAAAGAGCA---GTGGGA---

ATAGGAGCT---------GTGTTCCTTGGGTTCTTGGGAGCA---GCAGGAAGCACTATG---
GGCGCGGCGTCA---ATAACGCTGACGGTACAG---GCCAGACAA---TTGTTG---
TCTGGAATAGTGCAACAGCAAAGC---AATTTG---CTGAAG---
GCTATAGAGGCGCAACAGCATATGTTGCAACTCACGGTCTGGGGCATT
>AY772690
TTCTATTGTGATACAACAAAACTATTTAATGAAAGTGAGTTT-------------------------------
----

AATGAGGACGCAATCACGCTTCCATGCAAGATAAAACAATTTATAAACATGTGGCAGGGGGTGGGACGGGCAA
TGTACGCCCCTCCCATTGAAGGAAACATAACATGTAACTCAAGT---
ATCACAGGATTACTATTGACACGTGATGGAGGCAATACCAGTAGC----------------------------
----

CGAGGAGAGATATTCAGACCTGGAGGAGGAAATATGAAGGACAATTGGAGAAATGAATTATATAAATATAAAG
TGGTAGAAATTAAGCCATTG---GGAGTAGCACCCACTGATGCAAAAAGGAGAGTGGTG--------------
-------------------------GAGAGAGAAAAAAGAGCA---GTGGGA---ATAGGAGCT---------
GTGTTCCTTGGGTTCTTGGGAGCA---GCAGGAAGCACTATG---GGCGCGGCGTCA---
ATAACGCTGACGGTACAG---GCCAGACAA---CTGTTG---TCTGGTATAGTGCAACAGCAAAGC---
AATTTG---CTGAGA---GCTATAGAGGCGCAACAGCATATGTTGCAACTCACAGTCTGGGGCATT
>AY772691
TTCTATTGCAATACATCAGGCCTGTTTAATGGTACATATGATGTTAATAATACAAACAGTACTTCAAATACAA
GC---

AGTAATTCATCCATCATACTTAAATGCCGAATAAAACAAATTATAAACATGTGGCAGGGGGTAGGACGAGCGA
TGTATGCCCCTCCCATTGCAGGAAACATAACATGTAAATCAAAT---
ATTACAGGATTACTATTGACACGTGATGGAGGAACGGGAAATGACACT-------------------------
----

GACACAGAAACAGAAACATTCAGACCTGGAGGAGGAAATATGAGGGACAATTGGAGAAGTGAATTATATAAAT
ATAAGGTAGTAGAAATTAAACCACTA---GGAATAGCACCCACTGAGGCAAAAAGGAGAGTGGTG--------
-------------------------------GAGAGAGAAAAAAGAGCA---GTGGGA---CTAGGAGCT---
------GTGTTCCTTGGGTTCTTAGGAGCA---GCAGGAAGCACTATG---GGCGCAGCATCA---
ATAACGCTGACGGTACAG---GCCAGACAA---CTATTA---TCTGGTATAGTGCAACAGCAAAAC---
AATCTG---CTGAGA---GCTATAGAGGCGCAACAACATATGTTGCAACTCACAGTCTGGGGCATT
>AY703908
TTCTATTGCAATACAGCAGACCTGTTTACTAATGTAAGTAGA-------------------------------
----

AATGGGACCATCACACTCCCATGCAGAATAAAACAAATTGTAAACATGTGGCAGGGGGTAGGACGAGCAATGT
ATGCCCCTCCCATTGAAGGAAACATAACATGTAAATCAAAT---
ATCACAGGATTATTATTGCTACGTGATGGAGGAAACATGGGAGGAGACACCAGT-------------------
----
-
ACGAATGGCATAAAGAACGAGACATTCAGACCTGGGGGAGGAAATATGAAAGACAATTGGAGAAGTGAACTAT
ATAAATATAAAGTGGTAGAAATTAAGCCATTG---GGAGTGGCACCCACAGGGGCAAAAAGGACAGTGGTG--
-------------------------------------GAGAGAGAAAAAAGAGCA---GTGGGG---
ATAGGAGCT---------GTGCTCTTTGGGTTCTTGGGAGCA---GCAGGAAGCACTATG---
GGCGCGGCGTCA---ATAACGCTGACGGTACAG---GCCAGACAA---TTGTTG---
TCTGGTATAGTGCAACAGCAAAGC---AATTTG---CTGAGA---
GCTATAGAGGCGCAACAGCATATGTTGCAACTCACAGTCTGGGGCATT
>AY772693
TTCTATTGCGATACAACAAACCTGTTTAATGAAAGTATAGTTTCTAGTAAAAGTGAG----------------
----

TTAAACGAAACCATCATACTCCCATGCAGGATAAAACAAATTATAAACATGTGGCAGGGGGTAGGACGAGCAA
TGTATGCCCCTCCCATTGCAGGAAACATAACATGTAAATCAAAT---
ATCACAGGACTACTATTGACACGTGATGGAGGAAAAAACACAACA----------------------------
----

AAAGAGATATTCAGACCTGGAGGAGGAAATATGAAAGACAATTGGAGAAGTGAATTATATAAATATAAAGTAG

TAGAAATTAAGCCATTG---GGAATAGCACCCACTGGGGCAAAAAGGAGAGTGGTG-----------------
----------------------GAGAGAGAAAAAAGAGCA---GTGGGA---ATAGGAGCT---------
GTGTTCCTTGGGTTCTTGGGAGCG---GCAGGAAGCACTATG---GGCGCGGCGTCG---
ATAACGCTGACGGTACAG---GCCAGACAA---CTGTTG---TCTGGTATAGTGCAACAGCAAAGT---
AATTTG---CTGAGA---GCTATAGAGGCGCAACAACATATGTTGCAACTCACAGTCTGGGGCATT
>AY772700
TTCTATTGTAATACAACAAACCTGTTTAATCCTAATGGTACAGAGGGAAGT----------------------
----

CCAAACTCAACCATCACACTCCAATGCAGAATAAAACAAATTATAAACATGTGGCAGGAGGTAGGACGAGCAA
TGTATGCTCCGCCCATTGCAGGAAACATAACATGTAAATCAAAC---
ATCACAGGAATACTATTGACACGTGATGGAGGAAACAATGATAATAATACA----------------------
----

GATAATAATACAGAGACATTCAGACCTGGAGGAGGAAATATGAAGGATAATTGGAGAAGTGAATTATATAAAT
ATAAAGTGGTAGAAATTAAACCATTG---GGAGTAGCACCAACTGAAGCAAAAAGGAGAGTGGTG--------
-------------------------------GAGAGAGAAAAAAGAGCA---GTGGGA---ATAGGAGCT---
------GTGTTCCTTGGGTTCTTGGGAGCA---GCAGGAAGCACTATG---GGCGCGGCGTCA---
ATAACGCTGACGGTACAG---GCCAGACAA---CTGTTG---TCTGGTATAGTGCAACAGCAAAGC---
AATTTG---CTGAGA---GCTATAGAGGCGCAACAACATATGTTGCAACTCACGGTCTGGGGCATT
>AY772694
TTCTATTGCAATACATCAGGACTGTTTAATAGTACACACAATTCTACATTCTGGAATGAGACACATTGGCCAT
ACTGGGAG--
-------------AATACA------
ACAGATAATTTAAACATCACACTCCCATGCAGAATAAAACAAATTATAAACATGTGGCAGGAGGTAGGACGAG
CAATGTATGCTCCTCCCATTGCAGGAAACATAACATGTAAATCAAAT---
ATCACAGGACTACTATTAGTACGTGATGGAGGCAAACACAACAACACA-------------------------
----

AACGAGACAGAGAGATATTCAGACCTGCAGGAGGAGATATGAAGGACAATTGGAGAAGTGAATTATATAAATATA
AAGTGGTAGAAGTCAAGCCATTG---GGGATAGCACCCACTGGAGCAAAAAGGAGAGTGGTA-----------
----------------------------AAGAGAGAAAAAAGAGCA---GTGGGA---ATAGGAGCT------
---GTGCTCCTTGGTTTCTTGGGAGCA---GCCGGAAGCACTATG---GGCGCGGCGTCA---
ATAACGCTGACGGTACAG---GCCAGACAG---CTGCTG---TCTGGTATAGTGCAACAGCAAAAT---
AATTTG---CTGAGG---GCTATAGAGGCGCAACAGCATATGTTGCAACTCACAGTCTGGGGCATT
>AY772701
TTCTATTGTAATACAACAGAACTATTTGATACAACAAAAATATTTAATGGCACAGAA----------------
----

GGTAATTCAAACATCACACTCCCCTGCAGGATAAAACAAGTTATAAACATGTGGCAGCAGGTAGGACGAGCAA
TGTATGCTCCTCCCATTGCAGGAAATATAACATGTAACTCAAGT---
ATCACAGGATTACTATTGGTGCGTGATGGAGGAACAAGTAACACAAATAAC----------------------
----

ACAGAGAATAACACAGAGACATTCAGACCTGGAGGAGGAGATATGAGGGACAATTGGAGAAGTGAGTTATACA
AATATAAAGTGGTAGAAATTAAACCATTA---GGAATAGCACCTACCAAGGCAAAAAGGAGAGTGGTG-----
------------------------------------GAGAGAGAAAAAAGAGCA---GCGGGA---
ATAGGGGCT---------GTGATCCTTGGGTTCTTGGGAGCA---GCAGGAAGCACTATG---
GGCGCAGCGTCA---ATAGCGCTGACGGCACAG---GCCAGACAA---TTGTTG---
TCTGGTATAGTGCAACAGCAAAGC---AATTTG---CTGAGG---
GCCATAGAGGCGCAACAGCATATGTTGCAACTCACGGTTTGGGGCATC
>AY772695
TTCTATTGTAATACAACAAACCTGTTCAGTAAGAATGTAAGT-------------------------------
----

AATGATAACATCATACTCCCATGCAGAATAAAACAAATTATAAATATGTGGCAGAAGGTGGGACGAGCAATGT
ATGCCCCTCCCATTGCAGGAAACATAACATGTAACTCAAAT---
ATCACAGGACTACTACTGACACGTGATGGAGGCACAAATAACACA----------------------------
----

GAGATATTCAGACCTGGAGGAGGAAATATGAAGGACAATTGGAGAAATGAATTATATAAATATAAAGTGGTAG
AAATTAAGCCATTG---GGAGTAGCACCCACTGAGGCAAAAAGAATAGTGGTG--------------------
-------------------GAGAGAGAAAAAAGAGCA---GTAGGA---ATAGGAGCT---------
GTACTCCTTGGGTTCTTGGGAGCA---GCAGGAAGCACTATG---GGCGCGGCGTCA---
GTGACGCTGACGGTACAG---GCCAGACAA---CTGTTG---TCTGGTATAGTGCAACAGCAAAGC---
AATTTG---CTGAGA---GCTATAGAGGCGCAACAGCATATGTTGCAACTCACAGTCTGGGGCATT
>AY772696
TTTTATTGCGATACAACAAACCTGTTCAATCGTTCTAAGGATAAT----------------------------
----

GAAAATGATACCATCATACTCCCATGCAGAATAAAACAATTTATAAACATGTGGCAGGGGGTAGGACGAGCAA
TGTATGCCCCTCCCATTGAAGGATACATAACATGTAACTCAAGT---
ATCACAGGACTACTACTGACACGTGATGGAGGTGGTAATGAGACA----------------------------
----

AGGAAAAAATGAGACATTCAGACCTGGAGGAGGAAATATGAAGGACAATTGGAGAAGTGAATTATATAAATATA
AAGTGGTAGAAATTAAGCCATTG---GGAATAGCACCCACTGAGGCAAAAAGGAGAGTGGTG-----------
----------------------------GAGAGAGAAAAAAGAGCA---GTTGGA---ATAGGAGCT------
---GTGCTCCTTGGGTTCTTGGGAGCA---GCAGGGAGCACTATG---GGCGCAGCGTCA---
GTAACGCTGACGGTACAT---GCCAGAAAA---CTGTTG---GCTGGTATAGTGCAACAGCAAAGC---
AATTTG---CTGAGA---GCTATAGAGGCGCAACAGCACATGTTGCAACTCACAGCCTGGGGCATT
>DQ164115
TTCTATTGCAATACATCAAATCTGTTCAATACAACCACTAAT-------------------------------
----

AACATTACAAACATCACACTCCCATGCAGAATAAAACAATTTATAAACATGTGGCAGGAGGTAGGACGAGCAA
TGTATGCCCCGCCCATTGAAGGAAACATAACATGTAGGTCAAAT---
ATCACAGGACTACTATTGGTACGTGATGGAGGAAAAAGGAATAACACGAAT----------------------
----

GACAATGCCACAGAGATATTCAGACCTGGAGGAGGAAATATGAAGGACAATTGGAGAAGTGAATTATATAAAT
ATAAAGTAGTAGAAATTAAACCATTG---GGAATAGCACCCACAAAAGCAAAAAGGAGAGTGGTG--------
-------------------------------GAGAGAGAAAAAAGAGCA---GTGGGA---ATAGGAGCT---
------GTACTCCTTGGGTTCTTGGGAGCA---GCAGGAAGCACTATG---GGCGCGGCGTCA---
ATAACGCTGACGGTACAG---GCCAGACAA---CTGTTG---TCTGGTATAGTGCAACAGCAAAGC---
AATTTG---CTGAGA---GCTATAGAGGCGCAACAGCATATGTTGCAACTCACGGTCTGGGGCATT
>DQ351221
TTCTATTGTGATACAGCACAACTGTTTAACGAAAGTGAGGCAAACATAACCAGT-------------------
----

GAGGCAAACATAACCACTCTCCAATGCAGGATAAAACAAATTATAAACATGTGGCAGGGGGTAGGACGAGCAA
TGTATGCCCCTCCCATTGCAGGAAACATAACATGTAAATCAAAT---
ATCACAGGACTATTGTTGACACGTGATGGAGGAAACGCAGAAAAC----------------------------
----

ACAACGGAGACATTCAGACCTGGAGGAGGAAATATGAAGGACAATTGGAGAAGTGAATTATATAAATATAAAG
TGGTAGAAATTAAGCCATTG---GGAGTAGCACCCACTGAGGCAAAAAGGAGAGTGGTG--------------
-------------------------GAGAGAGAAAAAAGAGCA---GTGGGA---CTAGGAGCT---------
GTGTTCCTTGGGTTCTTGGGAGCA---GCAGGAAGCACTATG---GGCGCGGCGTCA---
ATAACGCTGACGGTACAG---GCCAGACAG---CTGTTG---TCTGGTATAGTGCAACAGCAAAGC---
AATTTG---CTGAGA---GCTATAGAGGCGCAACAGCATATGTTGCAACTCACGGTCTGGGGCATT
>DQ164121
TTCTATTGCAATACGTCAAATCTGTTTAATAGTACATATACTAGCAACATTACA-------------------
----

GGAAATTCAAGCATCACACTCCCATGCAGAATAAAACAAATTATAAACATGTGGCAGGAGGTAGGACAAGCAA
TGTATGCCCCTCCCATTAGAGGAAACATAACATGTATCTCAAAG---
ATCACAGGACTACTATTGACACGTGATGGAGGAAACAATAGGACT----------------------------
----

AATGAGACCTTCAGACCTGGAGGAGGAGATATGAGGGACAATTGGAGAAATGAATTATATAAATATAAAGTGG
TAGAAATTAAGCCATTG---GGAATAGCACCCACTGAGGCAAAAAGGACAGTGGTG-----------------
----------------------GAGAGAGAAAAAAGAGCA---GTGGGA---ATAGGAGCT---------
GTGTTCCTTGGGTTCTTAGGAGCA---GCAGGAAGCACTATG---GGCGCGGCGTCA---
ATAACGCTGACGGTACAG---GCCAGACAA---CTGTTG---TCTGGTATAGTGCAACAGCAAAGC---
AATTTG---CTGAGA---GCTATAGAGGCGCAACAGCATATGTTGCAACTCACAGTCTGGGGCATT
>DQ396393
TTCTATTGCAATACATCACTACTGTTTAATAGTATAGCAAGTAATAGTACAGAAAGTAATAGTACAAAAAGCA
GT---

AGTACAGAAAAGAAGATCACACTTCTATGCAGAATAAAACAAATTATAAACATGTGGCAGGAGGTGGGACGAG
CAATGTATGCCCCTCCCATTGCCGGAAACATAACATGTATATCAAAT---
ATAACAGGGATAATATTAACACGTGATGGAGGAGAACAGACAAAT----------------------------
----

GACACAGAGACATTCAGACCTGGAGGAGGAGATATGAGGGACAATTGGAGAAGTGAATTATATAAATATAAAG
TGGTAGAAATTAAGCCATTG---GGAGTAGCACCAACTGCGGCAAAAAGGAGAGTGGTG--------------
-------------------------AAGAGAGAAAAAAGAGCA---GTGGGA---ATAGGAGCT---------
GTGTTCCTTGGGTTCTTGGGAGCA---GCAGGAAGCACTATG---GGCGCGGCGTCA---
ATAACGCTGACGGTACAG---GCCAGACAA---CTGTTG---TCTGGTATAGTGCAACAGCAAAGC---
AATTTG---CTGAGG---GCTATAGAGGCGCAACAGCATATGTTGCAACTCACTGTCTGGGGCATT
>DQ396394
TTCTATTGCAATACATCAGGCTTGTTTAATAATGCTACAGGAAATGCATCAGGCTTGGTTAATAATGCTACAG
GA---

GATGGGTCAGTCATCACACTGCCATGCAAAATAAAACAAATAATAAACATGTGGCAGGAGGTAGGACGAGCAA
TGTATGCCCCTCCCATTGCAGGAAACATAACATGTAACTCAAGT---
ATCACAGGACTACTATTGACACGGGATGGAGGAAATACCAATGGC----------------------------
----

ACTGAAATATTCAGACCTGGAGGAGGAAATATGAAGGACAATTGGAGAAGTGAATTATATAAATATAAAGTAG
TAGAAATCAAGCCATTA---GGAGTAGCACCCACCGAGGCAAAAAGGAGAGTGGTG-----------------
----------------------GAGAGAGAAAAAAGAGCA---GTGGGA---ATAGGAGCT---------
GTTCTCCTTGGGTTCTTGGGAGCA---GCAGGAAGCACTATG---GGCGCGGCGTCA---
ATAACGCTGACGGTACAG---GCCAGACAA---CTGTTG---TCTGGTATAGTGCAACAGCAAAGC---
AATTTG---CTGAGA---GCTATAGAGGCGCAACAGCATATGTTGCAACTCACAGTCTGGGGCATT
>DQ164122
TTCTATTGCAGTACATCAAAGCTGTTTAATGCTACAATGGGTAATGATAAGTTGCTTAATGATACTTCA----
----

GATAACTCAACCATCACACTCCCATGCAGAATAAAACAATTTATAAACATGTGGCAGGGGGTTGGACGAGCAA
TGTATGCCCCGCCCATTGCAGGAAACATAACATGTAGATCAAAT---
ATCACAGGACTACTATTGACACGTGATGGAGGAGTAGGTAATGGGACA-------------------------
----

GAGAATAATACAGAGATATTCAGACCTGGAGGAGGAGATATGAAGGACAATTGGAGAAGTGAATTATATAAAT
ACAAAGTGGTAGAAGTGATGCCATTG---GGAATAGCACCCACTGGAGCAAAAAGGAGAGTGGTG--------
-------------------------------GAGAGAGAAAAAAGAGCA---GTGGGA---ATAGGAGCT---
------GTGTTCCTTGGGTTCTTGGGAGCA---GCAGGAAGCACTATG---GGCGCAGCGTCA---
ATAACGCTGACGGTACAG---GCCAGACAA---TTGTTG---TCTGGTATAGTGCAACAGCAAAAC---
AATTTG---CTGAGG---GCTATAGAGGCGCAACAGCATATGTTGCAACTCACGGTCTGGGGCATT
>DQ164117
TTCTATTGCAATACATCAGGACTATTTAATGGGACATTTGATGGTACAGAAAGTAATTCAACA----------
----

AGTAATGCAACCATCACAATTCCATGCAGAATAAAACAAATTATAAACATGTGGCAGGGGGTAGGACGAGCAA
TGTATGCCCCTCCCATTGCAGGAAACATAACATGTAAATCAAAT---
ATCACAGGACTACTATTAGTACGTGATGGAGGCAACACAGACAACACA-------------------------
----

AATGGCACAGAAATATTCAGACCTGGAGGAGGAGATATGAAGGACAATTGGAGAAGTGAATTATATAAATATA
AAGTGGTAGAAATTAAGCCATTG---GGAATAGCACCCACTGGAGCAAAAAGGAGAGTGGTG----------
---------------------------AAGAGAGAAAAAAGAGCA---GTGGGA---ATAGGAGCT------
---GTGCTCTTTGGGTTCTTGGGAGCA---GCAGGAAGCACTATG---GGCGCAGCGTCA---
ATAGCGCTGACGGCACAA---GCCAGACAA---GTATTG---TCTGGTATAGTGCAACAGCAAAAC---
AATTTG---CTGAGG---GCTATAGAGGCGCAACAGCATATGTTGCAACTCACGGTCTGGGGCATT
>DQ164118
TTCTATTGTAATACATCAGACCTGTTTAATAGGCCTATAAATGAGTCTAATGGTACAGAG------------
----

GATAATTCAACCATCACACTTCCATGCAGAATAAAACAAATTATAAACCTGTGGCAGGAGGTAGGACGAGCAA
TGTATGCCCCTCCCATTGAAGGAAACATAACATGTAAATCAAAT---
ATCACAGGACTACTATTGGTACGTGATGGAGGCAACACAACTGAA---------------------------
----

AACACACCTGAGATATTCAGACCTGGAGGAGGAGATATGAGGGACAATTGGAGAAATGAATTATACAAATATA
AAGTAGTAGAAATTGAGCCATTG---GGAATAGCACCCACTAAAGCAAAAAGGAGAGTGGTG----------
---------------------------GAGAGAGAAAAAAGAGCA---GTGGGA---ATAGGAGCT------
---GTGTTCCTTGGGTTCTTGGGAGCA---GCAGGAAGCACTATG---GGCGCGGCGTCA---
CTAACGCTGACGGTACAG---GCCAGACAA---CTGTTG---TCTGGTATAGTGCAACAGCAAAGC---
AATTTG---CTGAGA---GCCATAGAGGCGCAACAGCATATGTTGCAACTCACGGTATGGGGCATT
>DQ445637
TTCTACTGCAATACGACAGAACTGTTCAACATCACTAGCAAT------------------------------
----

ACAACTACCATCACACTCCCATGCAGAATAAAACAAATTATAAACATGTGGCAGGAGGTAGGACGAGCAATGT
ATGCCCCTCCCATTGCAGGAAACATAACATGTAACTCAAGT---
ATCACAGGATTACTATTAACACGTGATGGAGGCGGCACCCCAAATAACAAA---------------------
----

TCAGAGGGTAACGAAACATTCAGACCTGGAGGAGGAAATATGAAGGATAATTGGAGAAGTGAATTATATAAAT
ATAAAGTGGTAGAAATTAAGCCATTG---GGGATAGCACCCACTAATGCAAAAAGGAGAGTGGTG-------
------------------------------GCGAGAGAAAAAAGAGCA---GTGGGA---GTAGGAGTT---
------ATGTTCCTTGGGTTCTTGGGAGCA---GCAGGAAGCACTATG---GGCGCAGCGGCA---
GTGACGCTGACGGTACAG---GCCAGACAA---CTGTTG---TCTGGTATAGTGCAACAGCAAAAC---
AATTTG---CTGAGA---GCTATAGAGGCGCAACAGCATCTGTTGCAGCTCACGGTCTGGGGCATT
>DQ164110
TTCTATTGCAATACAACAGAACTGTTTTCTAATAAAAGTAATTCAGGCAACACAACA---------------
----

GACAAAGCAACCATCACACTCCCATGCAGAATAAAACAAATTGTAAACATGTGGCAGGAGGTAGGACGAGCAA
TGTATGCCCCTCCCATTGAAGGAAAAATAACATGCAATTCAAGT---
ATCACAGGACTACTATTGACACGTGATGGGGGAGAAGGAGGAAACACA------------------------
----

ACCAACGAAACAGAGATATTTAGACCTGCAGGAGGAAATATGAAGGACAATTGGGGAAGTGAATTATATAAAT
ATAAAGTGGTAGAAATCAAGCCATTG---GGAATAGCTCCCACTGAGGCAAGAAGGAGAGTAGTG-------
------------------------------GAGAGAGAAAAAAGAGCA---GCA------CTAGGAGCT---
------GTGTTCCTTGGGTTCTTGGGAGCA---GCAGGAAGCACTATG---GGCGCGGCGTCA---
ATAACGCTGACGGTACAG---GCCAGACAA---CTGTTG---TCTGGTATAGTGCGACAGCAAAGC---
AATTTG---CTGAGA---GCTATAGAGGCGCAACAGCACCTGTTGCAACTCACGGTCTGGGGCATT
>DQ093598
TTTTATTGCAATACATCAGGCCTGTTTAATACATCAGGTCTGTCTAGGAATGGTACAGAA------------
----

AATAATGCAAGCATCACACTCCCATGTAAAATAAAACAAATTATAAACATGTGGCAGGAGGTAGGACGAGCAA
TGTATGCCCCTCCCATTGCAGGGAACATAACATGCTCCTCAAAT---
ATCACAGGACTACTATTGGTACGTGATGGAGGCGGAGATAATAAC---------------------------
----

ACAGAAACATTCAGACCTGGAGGAGGGAATATGAAGGACAATTGGAGAAGTGAATTATATAAATATAAAGTGG
TAGAAATTAGGCCATTG---GGAGTAGCACCCACCAAGGCAAAAGGAGAGTGGTG------------------
----------------------GAGAGAGAAAAAAGAGCA---GTGGGA---CTGGGAGCT---------
GTGCTCTTTGGGTTCTTGGGAGCA---GCAGGAAGCACTATG---GGCGCGGCGTCA---
ATGACGTTGACGGTACAG---GCCAGACAA---TTGTTG---TCTGGTATAGTGCAACAGCAAAGC---
AATTTG---CTGAAG---GCTATAGAGGCGCAACAGCATCTGTTGCAACTCACGGTCTGGGGCATT
>DQ093599
TTCTATTGTGATACAACAAAGCTATTTAATAGTACATATAATCCTAATGTTACAACAAATGCAGCA-------
----

AATGGGTCAGTAATCACACTCCCATGCAGGATAAAACAATTTATAAATATGTGGCAGCAGGTAGGACGAGCAA
TGTATGCTCCTCCCATTGCAGGAAACATAACATGTAAATCAAAC---
ATCACAGGATTACTATTGGTGCGTGATGGAGGAAATCGCACAGAAACA-------------------------
----

GATAACACAGAGATATTCAGACCTGGAGGAGGAGATATGAGGGACAATTGGAGAAGTGAATTATACAAATATA
AAGTGGTAGAACTTAAACCATTG---GGAATAGCACCTACTAAGGCAAAAGGAGAGTGGTG-----------
----------------------------GAGAGAGAAAAAAGAGCA---GTGGGA---ATAGGAGCT------
---GTGTTCCTTGGGTTCTTGGGATTA---GCAGGAAGCACTATG---GGCGCGGCGTCA---
ATAACGCTGACGGTACAG---GCCAGACAA---TTGTTG---TCTGGTATAGTGCAACAGCAAAGC---
AATTTG---CTGAGG---GCCATAGAGGCGCAACAGCATATGTTGCAACTCACGGTCTGGGGCATT
>DQ164126
TTCTATTGCAATACATCAGCACTGTTTAATAGTACATACAATGGTACATACATTAATGAGACAAAA-------
----

AGTAATGGGACCATCACACTTCCATGCAGAATAAAGCAAATTATAAACATGTGGCAGGGGGTAGGACGAGCAA
TGTATGCTAATCCCATTGAAGGAAATATAACATGTAAATCAAAT---
ATCACAGGAATACTATTAGTACGTGATGGAGGAAATGAAGACAGT----------------------------
----

ACAAACGAGACATTCAGACCTATAGGAGGAAATATGAAGGACAATTGGAGAAGTGAATTATATAAATATAAAG
TGGTAGAAATTAAGCCATTG---GGAATAGCACCCACTAGAGCAAAAAGGAGAGTGGTG--------------
-------------------------GACAGAGCAAAAAGAGCA---GTGGGA---CTAGGAGCT---------
GTGATCGTTGGGTTCTTGGGAGCA---GCAGGAAGCACTATG---GGCGCGGCGTCA---
ATAACGCTGACGGCACAG---GCCAGACAA---CTGTTG---TCAGGTATAGTGCAACAGCAGAAT---
AATCTG---CTGAGA---GCTATAGAGGCGCAACAACATCTGCTGCAACTCACGGTCTGGGGCATT
>DQ093588
TTCTATTGCAATACAACAGATCTGTTTAATGAAAGGTTTAATGAA----------------------------
----

AGTAATCCAAACATCACACTCCTATGCAGAATAAAACAGATTATAAACATGTGGCAGGGGGTAGGACGAGCAA
TGTATGCCCCTCCCATTAAAGGAAACATAACATGTACCTCAAAT---
ATCACAGGACTACTATTGACACATGATAGAGAAAGAAATGGCACA----------------------------
----

GAGACATTCAGACCTGGAGGAGGAAATATGAAGGACAATTGGAGAAATGAATTATATAAATATAAGGTGGTAG
AAGTTAAGCCATTG---GGAGTAGCACCCACTGAGGCAAAAAGGAGAGTGGTG--------------------
-------------------GAGAGAGAAAAAAGAGCA---GTGGGA---ATAGGAGCT---------
GTACTCCTTGGGTTCTTGGGAGCA---GCAGGAAGCACTATG---GGCGCGGCGTCA---
ATAACGCTGACGGTACAG---GCCAGACAA---CTGTTG---TCTGGTATAGTGCAACAGCAAAGC---
AATTTG---CTGAGA---GCTATAGAGGCGCAACAGCATATGTTGCAACTCACGGTCTGGGGCATT
>DQ164119
TTCTATTGTGATACAACAGAACTGTTTAATTCAACAGAACAGTCTAGTACAACAGAAGAGGAGAATAAG----
----

AATAATAATACAATCACACTCCAATGCAGAATAAAACAAATTGTAAACATGTGGCAGGGGGTAGGAAGAGCAA
TGTATGCCCCTCCCATTGCAGGAAATATAACATGTAACTCAACC---
ATCACAGGATTACTATTGACACGTGATGGTGGAATAATCAATAACACAGAA----------------------
----

GGTAACAAAACAGAGACATTCAGACCAGGAGGAGGAGATATGAGGGACAATTGGAGAGGTGAATTATATAAAT
ATAAAGTGGTAGAAATTAGACCATTG---GGAATAGCACCCACTGAGGCAAAAAGGAGAGTGGTG--------
-------------------------------GAGAGAGGAAAAAGAGCA---GTGGGA---ATAGGAGCT---
------GTGCTCTTTGGGTTCTTGGGAGCA---GCAGGAAGCACTATG---GGCGCGGCGTCA---
GTGGCGCTGACGGTACAG---GCCAGACAA---CTGTTG---ACTGGCATAGTGCAACAGCAAAGT---
AATTTG---CTGAGG---GCTATAGAGGCGCAACAGCATATGTTGCAACTCACGGTCTGGGGCATT
>DQ093585
TTCTATTGTAACACATCAACACTGTTTAATAATACAAATATC-------------------------------
----

ACAGGCATAGCCACAATCCCATGCAGAATAAAACAAATTATAAACATGTGGCAGGAGGTAGGACGAGCAACAT
ATGCCCCTCCCATTGCAGGAAACATAACATGTATCTCAAAT---
ATCACAGGACTACTATTGCAACGTGATGGAGGAAACATCACAGATGAAAAG----------------------
----

AACAGGTCCATAGAGACATTCAGACCTATAGGAGGAAATATGAAGGACAATTGGAGAAGTGAATTATATAAAT
ATAAAGTAGTAGAAATTAAGCCATTG---GGAATAGCACCCACGGGGTCAAAAAGGAGAGTGGTG--------
-------------------------------GAGAGAGAAAAAAGAGCA---GTGGGA---GTAGGAGCT---
------GTACTCCTTGGGTTCTTGGGAGCA---GCAGGAAGCACTATG---GGCGCGGCGGCA---
GTGACGCTGACGGTACAG---GCCAGACAA---CTGCTG---TCTGGTATAGTGCAACAGCAAAGC---
AATTTG---CTGAGA---GCTATAGAGGCGCAACAGCATATGTTGCAGCTCACGGTCTGGGGCATT
>DQ056412
TTCTATTGCAATACATCAAACCTGTTCCAGGTCAATAACTCTACA----------------------------
----

ACTAATGATACCATCACACTTCCATGCAGAATAAAACAAATTATCAACATGTGGCAGGGGGTAGGACGAGCAA
TGTATGCCCCTCCCATTGCAGGAAACATAACATGTAACTCAAGC---
ATCACAGGACTACTACTGACACGTGATGGAGGAGACAAAAATAGC----------------------------
----

ACAGACCCAGAGACATTCAGACCTGGAGGAGGAAATATGAAGGACAATTGGAGAAGTGAATTATATAAATATA
AAGTGGTAGAAATTAAGCCATTA---GGAATAGCCCCCACTAAGGCAAAAAGGAGAGTGGTG-----------
----------------------------GAGAGAGAAAAAAGAGCA---GTGGGA---CTAGGAGCT------
---GTACTCCTTGGGTTCTTGGGAGCG---GCAGGAAGCACTATG---GGCGCAGCGTCA---
ATAACGCTGACGGTACAG---GCCAGACAA---TTGCTG---TCTGGTATAGTGCAACAGCAAAGC---
AATTTG---CTGAGA---GCTATAGAGGCGCAACAGCATATGTTGCAACTCACAGTCTGGGGCATT
>AY878072
TTCTATTGCAATACAACACAACTGTTTAATGAAAGTCTGTTCAAT----------------------------
----

AACACAACAAACATCACACTCCCATGCAGAATAAAACAAATTATAAACATGTGGCAGGAAGTAGGACGAGCAA
TGTATGCCCCTCCCATTGCAGGAAACATAACATGTAACTCAAGT---
ATCACAGGACTACTATTGCTACGTGATGGAGGAACAGAGAATAAT----------------------------
----

GACACAGAGACATTCAGGCCTGGAGGAGGAAATATGAAAGACAATTGGAGAAGTGAATTGTATAAATATAAAG
TGGTAGGACTTAAGCCATTG---GGAGTAGCACCCACTGAGGCAAAAAGGAGAGTGGTG--------------
-------------------------GAGAGAGAAAAAAGAGCA---GTGGGA---CCGGGAGCT---------
GTGCTCTTTGGGTTCTTGGGAGCA---GCAGGAAGCACTATG---GGCGCGGCGTCA---
ATATCGCTGACGGTACAG---GCCAGACAA---TTGCTG---TCTGGTATAGTGCAGCAGCAAAGC---
AATTTG---CTGAGA---GCTATAGAGGCGCAACAGCATATGTTGCAGCTCACGGTCTGGGGCATT
>AY772699
TTCTATTGCAATACATCAAATCTGTTTAATATAACTGCAAGTAATGCAAGTGATGCA----------------
----

AACAACAACACCATCACACTCCCATGCAAAATAAAACAAATTATAAACATGTGGCAGGAGGTAGGACGAGCAA
TGTATGCCCCTCCCATTGCAGGAAACATAACATGTAACTCAAGT---
ATCACAGGACTACTATTGACACGTGATGGAGGCAATAACAATGACACAGGC----------------------
----

AATAACAATGACACAGAGATATTCAGACCTGGAGGAGGAAATATGAAGGACAATTGGAGAAGTGAGTTATATA
AATATAAAGTGGTAGAAATTAAGCCATTA---GGAATAGCACCCACTAAGGCAAAAGGAGAGTGGTG------
----------------------------------GAGAGAGAGAAAAGAGCA---GTGGGA---
CTAGGAGCT---------GTACTCCTTGGGTTCTTAGGAACA---GCAGGAAGCACTATG---
GGCGCGGCGTCA---ATAACGCTGACGGTACAG---GCCAGGCAA---CTGTTG---
TCTGGTATAGTGCAACAGCAAAGC---AATTTG---CTGAGG---
GCTATAGAGGCGCAACAGCACATGTTGCAACTCACGGTCTGGGGCATT
>DQ164114
TTTTATTGCAATACAACACAACTGTTTAATGAAGATGTTTTAAACACCACACTGTTTAATAAA----------
----

ACAGACACCAACATCACACTCCCATGCCGAATAAAACAAATTATAAACATGTGGCAGGGAGTAGGAAAAGCAA
TGTATGCCCCCCCCATTGAAGGACAAATAATATGTAACTCAAGT---
ATCACAGGAATACTACTGACACGGGATGGAGGAGCAGTAACGAATGGC-------------------------
----

ACGAATGTCACAGAGACATTCAGACCTGGGGGAGGAAATATGAAGGACAATTGGAGAAGTGAATTATATAAAT
ATAAAGTGGTAGAAATTAAGCCATTA---GGGGTAGCACCCACTGGGGCAAAAGGAGAGTGGTG---------
-------------------------------GAGAGAGAAAAAAGAGCA---GTAGGA---ATAGGAGCT---
------GTGTTCCTTGGGTTCTTGGGAGCC---GCAGGAAGCACTATG---GGCGCGGCGTCA---
ATGACGCTGACGGTACAG---GCCAGACAA---CTGTTG---TCCGGTATAGTGCAACAGCAAAGC---
AATTTG---CTGAGA---GCCATAGAGGCGCAACAACATATGTTGCAACTCACGGTCTGGGGCATT
>AY901977
TTCTATTGTAATACAACAAACCTGTTTAATGAAAGTACGTCA-------------------------------
----

AACACAACCATCGGATTCCCATGCAGAATAAAACAAATTATAAACATGTGGCAGGGGGTAGGGCGAGCAATGT
ATGCCCCTCCCATTGAAGGAAACATAACATGTAAATCAAGT---
ATCACAGGGTTACTATTGGTACGTGATGGAGGAATAAATGACACA----------------------------
----

AAAGAGATATTCAGACCTGGAGGAGGAAATATGAAGGACAATTGGCGAAGTGAATTATATAAATATAAAGTGG
TAGAAATTAAGCCATTG---GGAGTAGCACCCACTGAGGCAAAAGGAGAGTGGTG------------------
----------------------GAGAGAGAAAAAAGAGCA---GTGGGA---ATAGGAGCT---------
GTGATCCTTGGGTTCTTGGGAGCA---GCAGGAAGCACTATG---GGCGCAGCGTCA---
ATAACGCTGACGGTACAG---GCCAGACAA---CTGTTG---TCTGGTATAGTGCAACAGCAAAGC---
AATTTG---CTGAGA---GCTGTAGAGACGCAACAGCATATGCTGCAACTCACGGTCTGGGGCATT
>AY901978
TTCTATTGCAATACAACAAAACTGTTTTCTAATGAAAGTGATGCT----------------------------
----

GACAACGGAACAATCACAATCCCATGCAGGATAAAACAAATCATAAGAATGTGGCAGGGGGTAGGACAAGCAA
TGTATGCCCCTCCCATTGTAGGAAACATAACATGTAACTCAAAT---
ATCACAGGACTACTCTTGGTACGTGATGGAGGAAAAAGCAATAAT----------------------------
----

AACACAGAGATATTTAGACCTGGAGGAGGAAATATGAAGGACAATTGGAGAAGTGAATTATATAAATATAAAG
TGGTAGAAGTTAAGCCATTG---GGAATAGCACCCACTAGAGCAAAAGGAGAGTGGTG---------------
-------------------------GAGAGAGAAAAAAGAGCA---GTGGGA---ATAGGAGCT---------
GTGTTCCTTGGGTTCTTGGGAGCA---GCAGGAAGCACTATG---GGCGCGGCGTCA---
ATAACGCTGACGGTACAG---GCCAGACAA---CTGTTG---TCTGGTATAGTGCAACAGCAAAGC---
AATTTG---CTGAGA---GCCATAGAGGCGCAACAGCATATGTTGCAACTCACAGTCTGGGGCATT
>DQ011171
TTCTATTGCAATACATCAAACCTGTTTAATAAAACTATAACAGCAAATTCAACAAATTCAAATTCAACAAAT-
----

TCAAGTATGCCCATCACACTCCAATGTAGAATAAAACAGATTATAAACATGTGGCAGGGGGTAGGACAAGCAA
TGTATGCCCCTCCCATTAAAGGAAACATAACATGTAACTCAAGC---
ATCACAGGACTACTATTGACACGTGATGGAGGAGCAAACAACGGT----------------------------
----

AGCGAAACATTCAGACCTGAAGGAGGAAATATGAAGGACAATTGGAGAAGTGAATTATATAAATATAAAGTGG
TAGAAATTAGGCCATTA---GGAATAGCACCCACTAAGGCAAAAAGGAGAGTGGTG-----------------
----------------------GAGAGAGAAAAAAGAGCA---GTGGGA---ATGGGAGCT---------
GTGCTCTTTGGGTTCTTAGGAGCA---GCAGGAAGCACTATG---GGCGCGGCGTCA---
ATAACGCTGACGGTACAG---GCCAGACAA---TTGTTG---TCTGGTATAGTGCAACAGCAAAGT---
AATTTG---CTGAGA---GCTATAGAGGCGCAACAGCATTTGTTGCAACTTACAGTCTGGGGCATT
>DQ011179
TTCTATTGCAATACATCACAACTGTTTAACGAAAGTAGGTGGTCAAACAACACA-------------------
----

AGCGACACAAACATCACAATCCCATGCAGGATAAAACAAATTATAAACATGTGGCAGGGGGTAGGAAGAGCAA
TGTATGCCCCTCCCATTGCAGGAAACATAACATGTAAATCAAAT---
ATCACAGGACTACTATTGACAAGGGATGGAGGAGTAAGCAATGGCACA-------------------------
----

AACAATAACGAGACATTCAGACCTGCAGGAGGAGATATGAAGGACAATTGGAGAAGTAAATTATATAAATATA
AAGTGGTAGAAATTAAGCCATTG---GGAATAGCACCCACTGGAGCAAAAAGGAGAGTGGTG-----------
----------------------------GGAAGAGAAAAAAGAGCA---GCA------ATAGGAGCT------
---ATGCTCCTTGGGTTCTTGGGAGCA---GCAGGAAGCACTATG---GGCGCGGCGTTA---
ATAACGCTGACGGTACAG---GCCAGACAA---CTGTTG---TCTGGTATAGTGCAACAGCAAAGC---
AATTTG---CTGAGA---GCTATAGAGGCGCAACAACATATGTTGCAACTCACGGTCTGGGGCATT
>DQ011173
TTCTATTGTAATACATCAGGACTATTTAATGGTACATACACAAAC----------------------------
----

GAGGATTCAACCATCACAATTCCATGCAGAATAAAACAAATTATAAACATGTGGCAGGGGGTAGGACGAGCAA
TGTATGCCCCTCCCATTGCAGGAAACATAATATGTAACTCAAAT---
ATCACAGGATTACTGTTAACACGTGATGGTGGTGTTGGTAATGAATCA-------------------------
----

TTAAATAACACAGAGATATTTAGACCTGGAGGAGGAAATATGAAGGACAATTGGAGAAGTGAATTATATAAAT
ATAAAGTGGTAGAAATTAAACCATTG---GGAATAGCACCCACTGAGGCAAAAAGGAGAGTGGTG--------
-------------------------------GAGAGAGAAAAAAGAGCA---GCGGGA---ATAGGAGCT---
------TTGTTCCTTGGGTTCTTGGGAACA---GCAGGAAGCACTATG---GGCGCGGCGTCA---
ATAACGCTGACGGTACAG---GCCAGACAA---CTGTTG---TCTGGTATAGTGCAACAGCAAAGC---
AATTTG---CTGAAA---GCTATAGAGGCACAACAGCATATGTTGCAACTCACGGTCTGGGGCATT
>DQ011170
TTCTATTGCAATACAACAAAACTGTTTAATGAAAGCGGGTTAAACGAAACAAATATCAATGAAAGCGGGTCA-
----

AACAAAACAAACATCACACTCCCGTGCAGGATAAAACAAATTATAAACATGTGGCAGGAGGTAGGAAGAGCAA
TGTACGCCCCTCCTATTGCAGGAAACATAACATGTAGATCAAAT---
ATCACAGGACTACTATTGACACGTGATGGGGGAACAAACAACACA----------------------------
----

GAGATATTCAGACCTGGAGGAGGAAATATGAAGGACAATTGGAGAAGTGAATTATATAAGTACAAAGTGGTAG
AAATTAAGCCATTG---GGAGTAGCTCCCACTAAATCAAAAAGGAGAGTGGTG--------------------
-------------------GAGAGAGAAAAAAGAGCA---GTGGGA---ATGGGAGCT---------
GTACTCCTTGGGTTCTTGGGAGCA---GCAGGAAGCACTATG---GGCGCGGCGTCA---
ATAACGCTGACGGTACAG---GCCAGACAA---CTGTTG---TCTGGTATAGTGCAACAGCAAAGC---
AATTTG---CTGAGA---GCTATAGAGGCGCAACAGCATATGTTGCAACTCACGGTCTGGGGCATT
>AY901979
TTCTATTGTAATACAACAAATCTGTTTAATAGTACATACATGCCTGAATACCCGCATAATGGTACAGAAAGTA
AT---

TCAAGCACAGAAATCACACTCCCATGCAGGATAAAGCAAATTATAAACATGTGGCAGCAGGTAGGACGAGCAA
TGTATGCTCCTCCCATTGCAGGGAGCATAACATGTAACTCAAAT---
ATCACAGGAATACTATTGATGCGTGATGGAGGAAATAACTCAAATAAC-------------------------
----

ACAACTAAGATAGAGATATTCAGACCTGGAGGAGGAGATATGAGGGACAATTGGAGAAGTGAATTATACAAAT
ATAAAGTGGTAGAAATTAAGCCATTG---GGAATAGCACCTACCAAGGCAAAAAGGAGAGTGGTG--------
-------------------------------GAGAGAGAAAAGAGAGCA---GTGGGA---ATAGGAGCT---
------GTGTTCCTTGGGTTCTTGGGAGCA---GCCGGAAGCACTATG---GGCGCGGCGTCA---
ATAACGCTGACGGTACAG---GCCAGACAA---TTGTTG---TCTGGTATAGTGCAACAGCAAAGC---
AATTTG---CTGAGG---GCCATAGAGGCGCAACAGCATATGTTGCAACTCACGGTCTGGGGCATT
>DQ396387
TTCTATTGCAATACATCAGAGCTGTTTAATACATCAAAGCTGTTAGATGCTAATGAAACAGAA----------
----

AGTAATTCAACAATCATACTCCCATGCAGAATAAAACAAATTATAAACATGTGGCAAGAGGTAGGACGAGCAA
TATATGCCCCTCCCATTGCAGGAAACATAACATGTAGATCAAAT---
ATCACAGGACTATTATTGACACGTGATGGAGGGACAAATGGGACA----------------------------
----

GATAAGACAGAGATATTCAGACCTGGAGGAGGAGATATGAGGGACAATTGGAGAAGTGAATTATATAAATATA
AAGTGGTAGAAATTAAACCACTG---GGAGTAGCACCCACTGAGGCAAAAAGGAGAGTGGTG-----------
----------------------------GAGAGAGAAAAAAGAGCA---GTGGGA---ATAGGAGCT------
---GTGTTCCTTGGGTTCTTGGGAGCA---GCAGGAAGCACTATG---GGCGCAGCATCA---
ATAACGCTGACGGTACAG---GCCAGACAA---TTGTTG---TCTGGAATAGTGCAACAGCAAAGC---
AATTTG---CTGAGG---GCTATAGAGGCGCAACAGCATCTGTTGCAACTCACGGTCTGGGGCATT
>DQ164127
TTCTATTGTGATACATCAAACCTGTTTAATAGAACATACCCGTCTAACCACACAGATAATAGTACAAAA----
----

GGGAATGAAACCATTACACTTCCATGCAGAATAAAACAAATTGTAAACATGTGGCAGGGGGTAGGACAAGCAA
TATATGCCCCTCCCATTGCAGGAAACATAACATGTAACTCAAGC---
ATCACAGGACTACTATTAGTACGTGATGGAGGGAATGTGAGTGCC----------------------------
----

ACAAACGAGACATTCAGGCCTGGAGGAGGAAATATGAAGGACAATTGGAGAAGTGAATTATATAAATATAAAG
TGGTAGAAATTAAGCCATTG---GGAGTAGCACCCACTGAGGCAAAAAGGAGAGTGGTG--------------
-------------------------GAGAGAGAAAAAAGAGCA---GTGGGC---ATAGGAGCT---------
GTACTCCTTGGGTTCTTGGGCGCA---GCAGGAAGCACTATG---GGCGCGGCGTCA---
ATAACGCTGACGGTACAG---GCCAGACAA---CTGTTG---TCTGGTATAGTGCAACAGCAAAGC---
AATTTG---CTGAGA---GCTATAGAGGCGCAACAACACATGTTGCAACTCACAGTCTGGGGCATT
>AY878058
TTCTATTGTAATACAACAGCATTGTTTGATGAAAGTATTTTTAATAAAAGTGAT-------------------
----

TCAAACAGCACCATCACCCTCCCATGCAGAATAAAGCAAATTATAAACATGTGGCGGAAAGTAGGACGAGCAA
TGTATGCCCCTCCCATTGAAGGAAACATAACATGTAAATCAAAT---
ATCACGGGACTATTATTGGAACGTGATGGAGGAAAGGTAAATAAT----------------------------
----

AAAACCGAGATATTCAGACCTGGAGGAGGAAATATGAAGGACAATTGGAGAAGTGAATTATATAAATATAAAG
TGGTAGAACTTAAGCCATTG---GGAGTAGCACCCACTAAGGCAAAAAGGATAGTGGTG--------------
-------------------------GAGAGAGAAAAAAGAGCA---GTGGGA---CTAGGAGCT---------
GTGTTCTTTGGGTTCTTGGGAGCA---GCAGGAAGCACTATG---GGCGCGGCGTCA---
ATAACGCTGACGGTACAG---GCCAGACAA---TTGTTG---TCTGGTATAGTGCAGCAGCAAAGC---
AATTTG---CTGAGA---GCTATAGAGGCGCAACAGCATATGTTGCAACTCACGGTCTGGGGCATT
>DQ396381
TTCTATTGCAATACATCACGACTATTTAATAGTACATACACACAGAATAATACAGAA----------------
----

GATAATTCAAACATCGCACTTTCATGCAAAATAAAACAAATTATAAACATGTGGCAGAGGGTAGGGCGAGCAA
TGTATGCCCCTCCCATTGCAGGAAACATAACATGTAGATCAAAT---
ATCACAGGACTACTATTAGTACGTGATGGAGGCACAGAAGACGAA----------------------------
----

AATAAGACAGAAACATTCAGACCTGGAGGAGGAAATATGAAGGACAATTGGAGAAGTGAATTATATAAATATA
AAGTGGTAGAAATTAAGCCATTG---GGAATAGCACCCACTGAAGCAAAAAGGAGAGTGGTG-----------
----------------------------GAGAGAGAAAAAAGAGCA---GTGGGA---ATAGGAGCT------
---GTGTTCCTTGGGTTCTTGGGAGCA---GCAGGAAGCACTATG---GGCGTGGCGTCA---
ATGACGCTGACGGTACGA---GCCAGACAG---TTGTTG---TCTGGTATAGTGCAACAGCAAAGC---
AATTTG---CTGAGG---GCTATAGAGGCGCAACAGCATATGTTGCAACTCACGGTCTGGGGCATT
>DQ093595
TTCTATTGTAATACATCACATCTATTTAATACATCACAGCTATTTAATAGTACATTGGCTAATAGTACAGGAG
AT---

TCTAATGGCAACATCACACTCCAATGCAGAATAAAACAAATTATAAACATGTGGCAGGGGGTAGGACGAGCAA
TGTATGCCCCTCCCATTGCAGGAAATATAACATGCAGATCAAAT---
ATCACAGGGCTACTATTGACACGTGATGGAGGAAAAACTAACACAAGC-------------------------
----

AATGAGACAGAGACATTCAGACCTGGAGGAGGAGACATGAGAGACAATTGGAGAAGTGAATTATATAAATATA
AAGTGGTAGAAATTAATCCATTA---GGAGTAGCTCCCACTGGAGCAAAAAGGAGAGTGGTG-----------
----------------------------GAGAGAGAGAAAAGAGCA---GTGGGA---ATAGGAGCT------
---GTGTTCCTTGGGTTCTTAGGAACA---GCAGGAAGCACTATG---GGCGCAGCATCA---
ATAACGCTGACGGTACAG---GCCAGACAA---TTATTG---TCTGGTATAGTGCAACAGCAAAGC---
AATTTG---CTGAGA---GCTATAGAGGCGCAACAGCATATGTTGCAGCTCACAGTCTGGGGCATT
>DQ164129
TTTTATTGTGATACATCAAAGCTGTTTAAGAGGACATACATGGATAATGGTACAGAAAATAATTCAACCACA-
----

AACAGTAATTCAACCATCATTCCATGCAGAATAAAACAAATTATAAACATGTGGCAGGAGGTAGGACGAGCAA
TGTATGCACCTCCCATTGCAGGAAAAATAACATGTAAATCAAAT---
ATCACAGGACTACTATTGACACGTGATGGAGGGTTAGATGATTCAAATAAG----------------------
----

CCAAATGATAGTGAGATATTCAGACCTGGAGGAGGAGATATGAGGAACAATTGGAGAAGTGAATTATATAAAT
ATAAAGTGGTAGAAATTAAGCCATTG---GGAGTAGCACCCACCAAGGCACAAAGGGAAGTGGTG--------
-------------------------------GGGAGAGAAAAAAGAGCA---CTGGGAATAGTAGGAGCT---
------ATGTTCCTTGGGTTCTTGGGAGCA---GCAGGAAGCACTATG---GGCGCAGCGTCA---
ATAACGCTGACGGCACAG---GCCAGACAA---GTGTTG---TCTGGTATAGTGCAACAGCAAAGC---
AATTTG---CTGAGG---GCTATAGAGGCGCAACAGCATATGTTGCAACTCACGATCTGGGGCATT
>DQ056416
TTCTATTGCAATACATCAGGATTGTTTAATGGTATATACAACGGTACAAAAAGTAATTCAAGTACAAAA----
----

AGTAATTTAACCATCACACTCCCATGCAGAATAAAACAAATTGTAAACATGTGGCAGAAGGTAGGACAAGCAA
TATATGCCCCTCCTATTGCAGGAAACATAACATGTACATCAAAG---
ATCACAGGACTACTATTGACACGTGATGGAGGCATACATAACCCA----------------------------
----

ACTAATGAGACATTCAGACCTGGAGGAGGAGATATGAGGGATAATTGGAGAAGTGAATTATATAAATATAAAG
TGGTAGAAATTAAGCCATTA---GGAATAGCACCCACTAATGCAAAAAGAAGAGTGGTG--------------
-------------------------GAGAGAGAAAAAAGAGCA---GTGGGA---ATAGGAGCT---------
GTGTTCCTTGGGTTCTTGGGAGCA---GCAGGAAGCACTATG---GGCGCGGCGTCA---
ATATCGCTGACGGTACAG---GCCAGACAA---TTGTTG---TCTGGTATAGTGCAACAGCAAAGC---
AATTTG---CTGAGG---GCTATAGAGGCGCAACAGCATATGTTGCAACTCACAGTCTGGGGCATT
>AY878062
TTCTATTGTAATACATCAGACCTGTTTAATGAATCCATAACTAATAGGACAGGAAATAATAGTACAGGA----
----

AATAGTTCAGTCATCACACTCCCATGCAGAATAAAACAAATTATAAACATGTGGCAGAAGGTAGGACGAGCAA
TGTATGCCCCTCCCATTGCAGGAAACATAACATGTAAATCAAAT---
ATCACAGGACTACTATTGACACGTGATGGAGGAATAAATGGGACT----------------------------
----

AATAACATAGGGACATTCAGACCTGGAGGAGGAGATATGAGGGACAATTGGAGAAGTGAATTATATAAATATA
AAGTGGTAGAGATTAGGCCATTG---GGAATAGCACCCACTGGAGCAAAGAGGAGAGTGGTG-----------
----------------------------GAGAGAGAAAAAAGAGCA---GTGGGA---ATAGGAGCT------
---GTGTTCCTTGGGTTCTTGGGAGCA---GCAGGAAGCACTATG---GGCGCAGCATCA---
ATAACGCTGACGGTACAA---GCCAGACAA---TTGTTG---TCTGGGATAGTGCAACAGCAAAGC---
AATTTG---CTGAGA---GCGATAGAGGCGCAACAGCATATGTTGCAACTCACGGTCTGGGGCATT
>DQ056409
TTCTATTGCAATACATCAAAACTGTTTAATGGTACATACAATGGTAATGGTACATACAATGGTACAGGTACAG
AC---

ACTAATTCAACCATCACCATCCCATGCAAGATAAAACAAATAGTAAACATGTGGCAGGGGGTAGGACGAGCAA
TGTATGCCCCTCCCATTGCAGGAAACATAACATGTAAATCAAAT---
ATCACAGGACTACTATTGACACGTGATGGAGGAGAAAATAGTACA----------------------------
----

GAGACATTCAGACCTGGAGGAGGAAATATGAAGGACAATTGGAGAAGTGAATTATATAAATATAAAGTAGTAG
AAATTAAGCCATTG---GGAATAGCACCCACTGGAGCAAAAAGGAGAGTGGTG--------------------
-------------------GAGAGAGAAAAAAGAGCA---GTGGGA---ATAGGAGCT---------
GTGTTCCTTGGGTTCTTGGGAGCA---GCAGGAAGCACTATG---GGCGCGGCGTCG---
ATAACGCTGACGGTACAA---GCCAGACAA---CTGTTG---TCTGGTATAGTGCAACAGCAAAGC---
AATTTG---CTGAGA---GCTATAGAGGCGCAACAGCATATGTTGCAACTCACAGTCTGGGGCATT
>DQ369993
TTTTATTGCAACACATCAAACCTGTTTAATGGTACATACAATAGTACATACAATAGTACATACAATAGT----
----

ACATCCAGAAACATCACACTCCAATGCAGAATAAAACAAATTATAAACATGTGGCAGGGAGTAGGGCAAGCAA
TGTATGCCCCTCCCATTAAAGGAAACATAACATGTAAATCAAAT---
ATCACAGGAATACTATTGACACGTGATGGAGGACATGAAAATGAC----------------------------
----

AACAACACAGAGACATTCAGACCTGGAAGAGGAAATATGAAGGACAATTGGAGAAGTGAATTATATAAATATA
AAGTGGTAGAAATTAAGCCATTG---GGAATTGCCCCCACTGAAGCAAAAAGGAGAGTGGTG-----------
----------------------------GAGAGAGAAAAAAGAGCA---GTGGGA---ATAGGAGCT------
---GTGTTCCTGGGGTTCTTGGGAGCA---GCAGGAAGCACTATG---GGCGCGGCGTCA---
ATAACGCTGACGGTACAG---GCCAGACAA---CTGTTG---TCTGGTATAGTGCAACAGCAAAAT---
AATTTG---CTGAGA---GCTATAGAGGCGCAACAACATATGTTGCAACTCACAGTCTGGGGCATT
>DQ396382
TTCTATTGCAACACAACAGGACTGTTTAATATCAGTAACAGTAAA----------------------------
----

GATGATTCAAACATCACACTCCCATGCAGAATAAAACAAATTATAAACATGTGGCAGGGGGTAGGACGAGCAA
TGTATGCTCCTCCCATTGCAGGAAAGATAACATGTACCTCAAAT---
ATCACAGGAATACTATTGACACGTGATGGAGGCCCAGAGAATAAC----------------------------
----

ACAAAGATATTCAGACCTGGAGGAGGAGATACGAGGGACAATTGGAGAAGTGAATTATATAAATATAAAGTGG
TAGAAGTTAAGCCATTG---GGAGTAGCACCCACTGAGGCAAGAAGGAGAGTGGTG-----------------
----------------------GAGAGAGAAAAAAGAGCA---GTGGGA---ATAGGAGCT---------
GTGTTCCTTGGGTTCTTGGGAGCA---GCAGGAAGCACTATG---GGCGCAGCGTCA---
ATAACGCTGACGGTACAG---GCCAGACAA---CTATTG---TCTGGTATAGTGCAACAGCAAAGT---
AATTTG---CTGAGA---GCTATAGAGGCGCAACAACATATGTTGCAACTCACGGTCTGGGGCATT
>DQ396396
TTCTATTGCAATACAACAAATCTGTTCAATACAACAGATCTGTTTAATAATAGCAATGATTTA----------
----

AACACAAGTACCATCACACTCCCATGCAGAATAAAGCAAATTATAAACATGTGGCAGGGGGTAGGACGAGCCA
TGTACGCCCCTCCCGTAGCAGGAAACATAACATGTAACTCAAGT---
ATCACAGGACTACTATTGGTACGTGATGGAGGAAGGAATAATAAT----------------------------
----

ACAAATGAGACATTCAGACCTGGGGGAGGAAATATGAAGGACAATTGGAGAAGTGAATTATATAAATATAAAG
TGGTAGAAATTAAGCCATTG---GGAGTGGCACCCACTGAGGCAAAAAGAAGAGTGGTG--------------
-------------------------GAGAGAGAAAAAAGAGCA---GTGGGA---ATAGGAGCT---------
GTATTCCTCGGGTTCTTGGGAGCA---GCCGGAAGCACTATG---GGCGCAGCGTCA---
ATAACGCTGACGGTACAG---GCCAGACAA---CTGTTG---TCTGGTATAGTGCAACAGCAAAGC---
AATTTG---CTGAGA---GCTATAGAGGCGCAACAGCACATGTTGCAACTCACGGTCTGGGGCATT
>DQ056413
TTCTATTGCAATACATCAAAACTATTTAATAGCACATATGTTAATAGGACAGACATGAATGATGATACAGGA-
----

AATAATTCAACCATCACACTTCCATGCAGAATAAAACAAATTATAAACATGTGGCAGGAGGTAGGACGAGCAA
TGTATGCCCCTCCCATTGAAGGAAACATAACATGTAAATCAAAT---
ATCACAGGAATACTATTAGTACGTGATGGAGGAATAAATAAGACA----------------------------
----

AATAACACAGAGACATTCAGACCAGGAGGAGGAAATATGAAGGACAATTGGAGAAGTGAATTATATAAATATA
AAGTGGTAGAAATTAAGCCATTA---GGAATAGCACCCACTGAAGCAAAAAGGAGAGTGGTG-----------
----------------------------GAGAGAGAAAAAAGAGCA---GTAGGA---ATGGGAGCT------
---GTGTTCCTTGGGTTCTTGGGAGTA---GCAGGAAGCACTATG---GGCGCAGCGTCA---
ATAGCGCTGACGGCACAG---GCCAGACAA---GTGTTG---TCTGGTATAGTGCAACAGCAAAGC---
AATTTG---CTGAGG---GCTATAGAGGCGCAACAGCATATGTTGCAACTCACGGTCTGGGGCATT
>DQ396383
TTCTATTGCAATACAACAAAACTGTTTGATAACAGAACGTTTAAT----------------------------
----

GATGGAAGCACAATCACAATCCCATGTAGAATAAAACAAATTATAAATATGTGGCAGGGGGTAGGACGAGCAA
TGTATGCCCCTCCCATTGAGGGCAGCATAACATGTAACTCAAAT---
ATCACAGGGCTACTATTGCTACGTGATGGAGGAAACGTGGATAGC----------------------------
----

TCAGAGGAGGTATTCAGACCTGGAGGAGGAAATATGAAGGACAATTGGAGAAGTGAATTATATAAATATAAAG
TGGTAGCAATTAAGCCCTTG---GGAATAGCACCCACGACGGCAAAAAGAAGGGTGGTG--------------
-------------------------GAGAGAGAAAAAAGAGCG---GGA------ATAGGAGCT---------
GTGTTCCTTGGGTTCTTGGGAGCA---GCAGGAAGCACTATG---GGCGCGGCGTCA---
ATAACGCTGACGGTACAG---GCCAGAAAA---CTGTTG---TCTGGTATAGTGCAACAGCAAAGC---
AATTTG---CTGAGA---GCTATAGAGGCGCAACAGCATATGTTGCAACTCACGGTCTGGGGCATT
>DQ396397
TTCTATTGTAATACATCAGAACTGTTTAAGAGTACATTAGATAATTTAACAGCAAATAGTTCAATAGAA----
----

GATAATACAATCATCACAATTCCATGCAGAATAAAACAAATTATAAACATGTGGCAGGGGGTAGGAAGAGCAA
TGTATGCCCCTCCCATTGCAGGAAACATAACATGTAATTCAAGC---
ATCACAGGACTACTATTAGTACGTGATGGTGGCGATACAAATGAC----------------------------
----

ACAGAAGAAATATTCAGACCTGAAGGAGGAAATATGAAGGACAATTGGAGAAGTGAATTATATAAATATAAAG
TGGTAGAAATTAAGCCATTA---GGAATAGCACCCACTAAAGCAAAAAGGAGAGTGGTG--------------
-------------------------GAGAGAGAAAAAAGAGCA---GTGGGA---TTAGGAGCT---------
TTGTTCCTTGGGTTCTTGGGAGCA---GCAGGAAGCACTATG---GGCGCGGCGTCA---
ATAACGCTGACGGTACAA---GCCAGACAA---GTATTG---TCTGGTATAGTGCAACAGCAAAAC---
AATTTG---CTGAGG---GCTATAGAGGCGCAACAGCATATGTTGCAACTCACAGTCTGGGGCATT
>DQ011180
TTCTATTGTAATACATCAGGCCTGTTTAATAGTACATACAATACATCAGACCTGTTTAATGATACATACAACA
CA---

GAAAGTAATTCCATTATCACACTCCCATGCAGAATAAAACAAATCATAAACATGTGGCAGGAGGTAGGACGAG
CAATGTATGCTCCTCCCATTGCAGGCACCATAACATGTAAATCAAAC---
ATCACAGGATTACTATTGGAGCGTGATGGAGGAAGGGAAAGTAAT----------------------------
----

GGCAGCGAGATATTCAGACCTGGAGGAGGAGATATGAAGAACAATTGGAGAAGTGAATTATATAAATATAAAG
TGGTAGAAATTAAGCCATTG---GGAGTTGCACCCACTGAGGCAAAAAGGGCAGTGGTG-------------
-------------------------AAGAGAGAAAAAAGAGCA---GTAGTGGGACTAGGAGCT--------
ATGATCTTTGGGTTCTTGGGAGCA---GCAGGAAGCACTATG---GGCGCGGCATCA---
ATAACGCTGACGGTACAG---GCCAGACAA---CTACTG---TCTGGTATAGTGCAACAGCAAAGC---
AATTTG---CTGAGG---GCTATAGAGGCGCAACAGCATATGTTGCAACTCACGGTCTGGGGCATT
>DQ056414
TTCTATTGCAATACATCAAGCCTGTTTAATGGTATATATAATGGTACATATAATGATACG------------
----

TCTGATAAAACCATCACATTCTCATGCAGAATAAAACAAATTATAAACATGTGGCAGAAGGTAGGACGAGCAA
TATATGCCCCTCCCATTGCAGGAAACATAACATGTAAATCAAAT---
ATCACAGGACTACTATTGACACGTGATGGAGGAGGTAGAAATAAT---------------------------
----

AATATAACAGAGACATTCAGACCTGGAGGAGGAGAGATGAGGGATAATTGGAGAAGTGAATTATATAAATACA
AGGTGGTAGAAATTAAGCCATTA---GGAGTAGCACCCACCAAGGCAAAAAGGAGAGTGGTG----------
----------------------------GAGAGAGGAAAAAGAGCA---GTAGGA---ATAGGAGCT-----
---ATGATCTTTGGGTTCTTGGGAGCA---GCAGGAAGCACTATG---GGCGCAGCATCA---
ATAACGCTGACGGTACAG---GCCAGACAA---TTGTTG---TCTGGTATAGTGCAACAGCAAAGC---
AATTTG---CTGAGG---GCTATAGAGGCGCAACAGCATATGTTGCAACTCACGGTCTGGGGCATT
>DQ056415
TTCTATTGCAATACATCAAGACTGTTTAATAGTACATACAATCCTAATGGTACATACAACTCTACATACACAT
CATACAATTTT---
-------------AGTGGT------
ACATCTAATGATACCATCACACTCCCATGCAGAATAAAACAAATTATAAACATGTGGCAGGAAGTAGGGAGAG
CAATGTATGCCCCTCCCATTACAGGAAACATAACATGTAAATCAAAT---
ATCACAGGACTACTATTGTTACGTGATGGAGGAAACAGCTCTAAT---------------------------
----

GAGACATTCAGACCTGGAGGAGGAGATATGAGGGATAATTGGAGAAGTGAACTATATAAATATAAGGTAGTAG
AAATTAAGCCATTA---GGAATAGCACCCACTACAGTAAAAAGGAGAGTGGTG-------------------
-------------------GAGAAAGAAAAAAGAGCA---GTGGGA---ATAGGAGCT--------
GTGTTCCTTGGGTTCTTGGGAGCA---GCAGGAAGCACTATG---GGCGCGGCGTCA---
ATAACGCTGACGGTACAG---GCCAGACAA---TTGTTG---TCTGGTATAGTGCAACAGCAAAGC---
AATTTG---CTGAGG---GCTATAGAGGCGCAACAGCATATGTTGCAACTCACAGTCTGGGGCATT
>DQ056406
TTCTATTGCAATACAACAGAACTGTTTAATAATACAAATAACAGTACA------------------------
----

AATATAACAGACATCATACTCCCATGCAGAATAAAACAGATTATAAACATGTGGCAGGGGGTAGGACGAGCAA
TGTATGCCCCTCCCATTGCAGGAAACATAACATGTACCTCAAAG---
ATCACAGGACTACTATTGACACGTGATGGAGGAGGAAACGAAACA---------------------------
----

AATAACACAGAAATATTCAGACCTGGAGGAGGAAATATGAAGGACAATTGGAGAAGTGAATTATATAAATATA
AAGTGGTAGAAATTAAGCCATTG---GGAATAGCACCCACTGAGGCAAAAAGGAGAACGGTG----------
----------------------------AAGAAAGAAAAAAGAGCA---GTGGGA---ATAGGAGCT-----
---GTACTCCTTGGGTTCTTGGGAGCA---GCAGGAAGCACTATG---GGCGCGGCGTCA---
ATAACGCTGACGGTACAG---GCCAGACAA---CTATTG---TCTGGTATAGTGCAACAGCAAAAC---
AATTTG---CTGAGA---GCTATAGAGGCGCAACAGAGTATGTTGCAACTCACGGTCTGGGGCATT
>DQ056417
TTCTATTGCAATACATCAGGCCTATTTAATGGTACATACAATCCAGCATACACGTATAATGCGACA------
----

AATAACTCAACCATCACACTCCAATGCAGAATAAGACAAATTATAAACATGTGGCAGAAGGTGGGAAGAGCAA
TGTATGCCCCTCCCATTGCAGGAAACATAACATGTAATTCGAGC---
ATCACAGGACTACTGTTGGTACGTGATGGAGGCAAAAACACAAGT---------------------------
----

GGAATAGAAACATTCAGACCCGGAGGAGGAAACATGAAGGACAATTGGAGAAGTGAATTATATAAATATAAAG
TGGTAGAAATTAAGCCATTG---GGAGTAGCACCCACGAGTGCAAAAAGGAGAGTGGTG--------------
-------------------------GAGAGAGAAAAAAGAGCA---GTGGGA---CTAGGAGCT---------
GTGTTCCTTGGGTTCTTGGGAGCA---GCAGGAAGCACTATG---GGCGCGGCGTCA---
ATAACGCTGACGGTACAG---GCCAGACAA---TTATTG---TCTGGTATAGTGCAGCAGCAAAGT---
AATTTG---CTGAGA---GCTATAGAGGCGCAACAACATATGTTGCAACTCACAGTCTGGGGCATT
>DQ093605
TTCTATTGTAATACATCAGGACTGTTTAATGGTACAGTAGGTAACTCAACTTCAAATAATTCAACT-------
----

TTAAGCGGCACAATCACACTCCCATGCAGAATAAAACAGATTATAAACATGTGGCAGACGGTAGGGCGAGCAA
TGTATAACCCTCCCATTGCAGGAAACATAACATGTAAATCAAAT---
ATCACAGGACTACTGTTGACACGGGATGGAGGGAACGACAATGAAACA-------------------------
----

AAAGAAACAGAGACATTCAGACCTGGAGGAGGAGATATGAGAGACAATTGGAGAAGTGAATTATACAAATATA
AAGTGGTAGAAATTAAGCCATTA---GGAATAGCACCCACTAAGGCACGAAGGAGAGTGGTG-----------
----------------------------GAGAGAGAGAAAAGAGCA---GTAGGA---ATAGGAGCT------
---GTGTTCCTTGGGTTCTTGGGAGCA---GCTGGAAGCACTATG---GGCGCAGCATCA---
ATAACGCTGACGGTACAG---GCCAGACAA---TTGTTG---TCTGGTATAGTGCAACAGCAAAGT---
AATTTG---CTGAGG---GCTATAGAGGCGCAACAGCATTTGTTGCAACTCACGGTCTGGGGCATT
>GQ999973
TTCTATTGCAATACAACAAAACTGTTTAACAGTACAGATAATGAGACAAAAAGTACAGAAAGTAATAATGAGA
TA---

GGAAATAATTCAATCATCACACTTCCATGCAGAATAAAACAAATTATAAACATGTGGCAGGAGGTAGGACGAG
CAATGTATGCCCCTCCCATTGCAGGAAACATAACATGTAGATCAGAG---
ATCACAGGATTATTATTGACACGTGATGGAGGGAGAAACACTACA----------------------------
----

GAGGAGACATTCAGACCTGGAGGAGGAAATATGAAGGACAATTGGAGAAGTGAATTATATAAATATAAAGTGG
TAGAAATTAAACCATTG---GGAATAGCACCCACCAAGGCAAAAAGGAGAGTGGTG-----------------
----------------------GAGAGAGAAAAAAGAGCA---GTGGGA---ATAGGAGCT---------
TTGTTCCTTGGGTTCTTGGGAGCA---GCAGGAAGCACTATG---GGCGCGGCGTCA---
ATAACGCTGACGGTACAG---GCCAGACAA---TTGTTG---TCTGGTATAGTGCAACAGCAAAAT---
AATTTG---CTGAGA---GCTATAGAGGCGCAACAGCATATGTTGCAACTCACGGTCTGGGGCATT
>GQ999975
TTTTATTGCAATACATCAGGCCTGTTTAATGGTACATTTAATGGTACAGACATGTCTAATGAG----------
----

ACAAATTCAACCATCACACTTCCATGCAAAATAAAACAAATTATAAACATGTGGCAAAAGGTAGGACGGGCAA
TGTATGCCCCTCCCATTGCAGGAAATATAACATGTACAGCAAAT---
ATCACAGGACTGCTATTGACACGTGATGGAGGGGACAGTAGCAGT----------------------------
----

ACAAACGAGACATTCAGACCTGGAGGAGGAAATATGAAGGACAATTGGAGAAGTGAATTATATAAATATAAAG
TGGTAGAAATCAAGCCATTG---GGGATAGCACCAACTGAAGCAAAAAGGAGAGTGGTG--------------
-------------------------GAGAGAGAAAAAAGAGCA---GTGGGA---ATAGGAGCT---------
TTGTTCCTTGGGTTCTTGGGAGCA---GCAGGAAGCACTATG---GGCGCGGCGTCA---
ATAACGCTGACGGCACAG---GCCAGACAA---CTGTTG---TCTGGTATAGTGCAACAGCAAAAC---
AATTTG---CTGAGA---GCTATAGAGGCGCAACAGCATATGTTGCAACTCACGGTCTGGGGCATT
>AY703909
TTCTATTGCAATACATCAAAACTGTTTAATAATAATAAGACAGAA----------------------------
----

TGGAATTCAACCATCATACTGCCATGCAGAATAAAACAAATTATAAACATGTGGCAGGAGGTAGGAAGAGCAA
TATATGCCCCTCCCATTGCAGGAAACATAACATGTATATCAAAT---
ATCACAGGACTACTATTGGAACGGGATGGAGGAGATAATTCAACAGAG-------------------------
----

AATGGCACAGAGATATTCAGACCTATAGGAGGAGATATGAGGAACAATTGGAGAAGTGAATTATATAAATATA
AAGTGGTAGAAATTAAGCCACTG---GGAGTAGCACCCACTAAGGCAAAAAGGAGAGTGGTG----------
----------------------------GAGAGAGAAAAAAGAGCA---GTGGGA---CTAGGAGCT------
---TTGTTCCTTGGGTTCTTGGGAGCA---GCAGGAAGCACTATG---GGCGCAGCATCA---
ATAACGCTGACGGTACAG---GCCAGACAA---GTGCTG---TCTGGAATAGTGCAACAGCAAAGC---
AATTTG---CTGAGA---GCTGTAGAGGCGCAACAGCATATGTTGCAACTCACTGTCTGGGGCATT
>AY703910
TTCTATTGTAATACATCAAAACTGTTTAATAATAATAGCACAAACAGCACT---------------------
----

TCAAATGGAACCATCACACTCCCATGCAGAATAAGACAAATTATAAACATGTGGCAGGGAGTAGGACGGGCAA
TGTATGCCCCGCCCATTGCAGGAAACATAACATGTACGTCAAAT---
ATTACAGGACTACTACTGACACGTGATGGAGGAAATAACGAAACA----------------------------
----

GAGACAGAAACATTCAGACCTGGAGGAGGAGATATGAGGGACAATTGGAGAAGTGAGTTATATAAATACAAAG
TGGTAGAAATTAAGCCATTA---GGAATAGCACCTACTAGGGCAAAAAGGAGAGTGGTG-------------
-------------------------GAGAGAGAAAAAAGAGCA---GCA------ATAGGAGCT---------
GTGTTCCTTGGGTTCTTGGGAGCA---GCAGGAAGCACTATG---GGCGCAGCATCA---
ATAACGCTGACGGTACAG---GCCAGACAA---TTGTTG---TCTGGTATAGTGCAACAGCAAAGC---
AATTTG---CTGAGG---GCTATAGAGGCGCAACAGCATATGTTGCAACTCACGGTCTGGGGCATT
>AY703911
TTCTATTGCAATACATCAGATCTGTTTACAGAATTTAATGGTACAACAGAATATGATAATGCAACCATAGAT-
----

GATAATGCAACCATCACACTCCAATGTAGAATAAAACAGATTATAAACATGTGGCAGGGGGTAGGACAAGCAA
TGTATGCCCCTCCCATTGAAGGAAACATAACATGTAACTCAAAT---
ATCACAGGACTACTATTGACACGTGATGGAGGACCAGATAATAGA----------------------------
----

ACGGAGACATTCAGACCTGGAGGAGGAAATATGAAGGACAATTGGAGAAGTGAATTATATAAATATAAAGTGG
TAGAAATTAAGCCATTA---GGAATAGCACCCACTAAGGCACAAAGGAGAGTGGTG-----------------
----------------------GAAAGAGAAAAAAGAGGA---GCAGCA---CTGGGAGCT---------
ATGTTCCTTGGGTTCTTGGGAGCA---GCAGGAAGCACTATG---GGCGCGGCATCA---
ATAACGCTGACGGTACAG---GCCAGACGA---TTGTTG---TCTGGTATAGTGCAACAGCAAAGC---
AATTTG---CTGAGA---GCTATAGAGGCGCAACAGCATATGTTGCAACTCACAGTCTGGGGCATT
>DQ382369
TTCTATTGTAACACAACAGAACTGTTTAAAAATGATTCAAGCACAAAA-------------------------
----

AATGATACATACATCACACTCCCATGCAGAATAAAACAAATTATAAACATGTGGCAAGAGGTAGGACGAGCAA
TGTATGCTCCTCCCATTGCAGGAAACATAACATGTAGATCAAAT---
ATCACAGGATTGCTATTGGTACGTGATGGAGGAAACGAGAATAAC----------------------------
----

ATCACAGAGATATTCAGACCTGGAGGAGGAAATATGAAGGACAATTGGAGAAGTGAACTATATAAATACAAAG
TGGTAGAAATTAAACCATTA---GGAATAGCACCCACAACGGCAAAAAGGAGAGTGGTG-------------
-------------------------CAGAGAGAAAAAAGAGCA---GTAGGA---ATGGGAGCT---------
GTACTCCTTGGGTTCTTGGGAGCA---GCAGGAAGCACTATG---GGCGCGGCGTCA---
ATGACGCTGACGGTACAG---GCCAGACAA---CTGTTG---TCTGGTATAGTGCAACAGCAAAGC---
AATTTG---CTGAGA---GCTATAGAGGCGCAACAGCATCTGTTGCAACTCACAGTCTGGGGCATT
>DQ382370
TTCTATTGCAATACAACAAAACTGTTTAAGGAAAATAGTACAAAT----------------------------
----

GAAAATGATACAATCACACTCCCATGCAGGATAAAACAAATTGTAAACATGTGGCAGACAGTAGGACAAGCAA
TGTATGCCCCTCCCATTGCAGGAAACATAACATGTAACTCAAGT---
ATCACAGGGTTACTATTGACACGTGATGGAGGAAATGGGAATAGT----------------------------
----

ACTAATGAGACATTCAGACCTACAGGAGGAGATATGAAAGACAATTGGAGAAGTGAATTATATAAATATAAAG
TGGTAGAAATTAAGCCATTG---GGAGTAGCACCCACAGAGTCAAAAAGGAGAGTGGTG--------------
-------------------------GAGAGAGAAAAAAGAGCA---GCGGGA---ATAGGAGCT---------
GTACTCCTTGGGTTCTTGGGAGCA---GCAGGAAGCACTATG---GGCGCAGCGTCA---
ATAACGCTGACGGTACAG---GCCAGACAA---CTGTTG---TCTGGTATAGTGCAACAGCAAAGC---
AATTTG---CTGAGA---GCTATAGAGGCGCAACAGCATATGTTGCAACTCACGGTCTGGGGCATT
>DQ382371
TTCTATTGCAATACATCAAAACTGTTTAATAATAGTGAAGACAAGGATAGTCCTTTTAATTCAACC-------
----

AGAAATGCAACCATTATAATCCAATGCAGAATAAGACAATTTATAAACATGTGGCAGGAGGTAGGACGAGCAA
TGTATGCCCCTCCCATTGCAGGAAACATAACATGTAAATCAAAC---
ATCACAGGACTACTATTGACACGTGATGGAGGAGAGAATGACACA----------------------------
----

AACCCAGAGATATTCAGACCTGGAGGAGGAGATATGAGGGACAATTGGAGAAGTGAATTATATAAATACAAAG
TGGTAGAAATTAAGCCATTA---GGAATAGCACCCACTGGAGCAAAAAGGAGAGTGGTG--------------
-------------------------GAGAGAGAAAAAAGAGCA---GTGGGA---ATAGGAGCT---------
GTGCTCCTTGGGTTCTTGGGAACA---GCAGGAAGCACTATG---GGCGCGGCGTCA---
TTAGCGCTGACGGTACAG---GCCAGACAA---CTGTTG---TCTGGTATAGTGCAACAGCAAAGC---
AATTTG---CTGAGG---GCTATAGAGGCGCAACAGCATATGTTGCAACTCACGGTCTGGGGCATT
>DQ382372
TTCTATTGCAACACATCAAACCTGTTTAATAGGACATACAATAGTACAGATAAGTCAGATAGGTTTAATAGTA
CA---

GATAATTCAACCATCACAATCCAATGCAGAATAAGACAATTTATAAACATGTGGCAGAGGGTAGGACGAGCAA
TGTATGCCCCTCCCATTGAAGGAAACATAACATGTAAATCAAAA---
ATCACAGGACTACTATTGACACGTGATGGAGGCGAGGGGAATAAGAATGAC----------------------
----

ACAAATGACACAGAGATATTCAGACCTGGAGGAGGAGATATGAGGGACAATTGGAGAAGTGAATTATATAAAT
ATAAAGTGGTAGAAATTAAGCCATTA---GGACTAGCACCCACTAAAGCAAAAAGGAGAGTGGTG--------
-------------------------------GAGAGAGAAAAAAGAGCA---GTGGGA---ATAGGAGCG---
------TTGTTCCTTGGGGTCTTGGGAGTA---GCAGGAAGCACTATG---GGCGCAGCATCA---
ATAACGTTGACGGTACAG---GCCAGACAA---TTGTTG---TCTGGTATAGTGCAACAGCAAAAC---
AATTTG---CTGAGG---GCTATAGAGGCGCAACAGCATATGTTGCAACTCACGGTCTGGGGCATT
>DQ382375
TTCTATTGCAATACAGCAGGCCTGTTTAATACAAGTCTGATTAATAGTACTAAGAGTACAAGTACAAAGAAT-
----

ACACAAGACCCCATCACACTCACATGCAGAATAAAGCAATTTATAAACATGTGGCAGCAGGTAGGACGAGCAA
TGTATGCTCCTCCCATTGCAGGAAACATAACATGTAAATCAAAT---
ATCACAGGATTGCTATTGATGTATGATGGAGGAAGGAATGAGACA----------------------------
----

AATGCGACCTTCAGACCTGGAGGAGGAGATATGAGAGACAATTGGAGAAGTGAATTATACAAATATAAAGTGG
TAGAAATTAAGCCATTG---GGAATAGCACCCACCAAGGCAAAAAGGAGAGTGGTG-----------------
----------------------GAGAGAGAAAAAAGAGCA---GTGGGA---ATAGGAGCT---------
GTGCTCTTTGGGTTCTTGGGAGCA---GCAGGAAGCACTATG---GGCGCGGCGTCA---
ATAACGCTGACGGTACAG---GCCAGACAA---TTGTTG---TCTGGTATAGTGCAACAGCAAAGC---
AATTTG---CTGAGA---GCTATAGAGGCGCAACAGCATATGTTGCAACTCACGGTCTGGGGCATT
>DQ382377
TTCTATTGTAATACATCAAAACTGTTTAATGGTACATACAATGGTACATACAATGCTACAGAAAGAAACATT-
----

GGTCCCGGAACCCTCTGCCTCCAATGCCGAATAAAACAAATTATAAACATGTGGCAACAGGTGGGACGAGCAA
TGTATGCTCCTCCCATTGCAGGAAACATAACATGTACATCAAAT---
ATCACAGGACTACTATTGGTACGTGATGGAGGAAACTCAAGTAAC----------------------------
----

CTAAAGATAGAGATATTCAGACCTAACAGAGGAGAGATGAGGGACAACTGGAGAAGTAAATTATATGAGTATA
AGGTGGTAAAAATTAAACCATTA---GGAGTAGCTCCCACTGAAGCAAAAAGGAGAGTGGTG-----------
----------------------------GAGAGACAAAAAAGAGCA---GCAGGA---ATAGGAGCT------
---GTGTTCCTTGGGTTCTTGGGAGTA---GCAGGAAGCACTATG---GGCGCGGCGTCA---
ATGACGCTGACGGTACAG---GCCAGACAA---CTGTTG---TCTGGTATAGTGCAACAGCAAAGC---
AATTTG---CTGAGG---GCTATAGAGGCGCAACAGCATATGTTGCAACTCACGGTCTGGGGCATT
>DQ382378
TTCTATTGCGATACAACAAACCTGTTTAATATTTCTAATATTAAC----------------------------
----

AGTACAAGCAACATTACACTCCAATGCAAGATAAAACAAATTATAAACATGTGGCAAAAGGTAGGACGAGCAA
TGTATGCCCCTCCCATTGAAGGAACCATAACATGTATATCAAAC---
ATCACAGGACTATTATTGACACGTGATGGAGGTAGTAGTAATGAGACTAGT----------------------
----

AGTATTAATAAGACAGAGATTTTTAGACCTGGAGGAGGAAATATGAAAGACAATTGGAGAAGTGAATTGTATA
AATATAAGGTGGTAGAAATTAAGCCATTG---GGAATAGCACCCACTGGAGCAAAAAGGAGAGTGGTG-----
----------------------------------GAGAGAGAAAAAAGAGCA---GTGGGA---
ATAGGAGCT---------GTGTTCCTTGGGTTCTTGGGAGCA---GCAGGAAGCACTATG---
GGCGCGGCGTCA---ATAACGCTGACGGTACAG---GCCAGACAA---CTGCTG---
TCTGGTATAGTGCAACAGCAAAGC---AATTTG---CTGAGA---
GCTATAGAGGCGCAACAGCATATGTTGCAACTCACAGTCTGGGGCATT
>DQ382361
TTCTACTGCAATACATCAAGACTGTTTAATAGTACATGGCTTAATAGTACAGAGCCTACAGACCAGGCTAAT-
----

GATACAAGTGTAATAACACTCCCATGCAGAATAAAACAAATTATAAACATGTGGCAGGAGGTAGGACGGGCAA
TGTATGCTCCTCCCATTGCAGGAAACATAACATGTAGGTCAAAT---
ATCACAGGATTACTATTGGTGAGGGATGGAGGAAAGGGCAATAGCACA-------------------------
----

GACAATAAATCTGAGATATTCAGACCTGGAGGAGGAGATATGAGGGACAATTGGAGAAGTGAATTATACAAAT
ATAAAGTGGTAGAAGTTAAGCCATTG---GGAATAGCACCCACCCAGGCAAAAAGGAGAGTGGTG--------
-------------------------------GAGAGAGAAAAAAGAGCA---GTGGGA---ATAGGAGCT---
------GTGTTCCTTGGGTTCTTGGGAGCC---GCAGGAAGCACTATG---GGCGTGGCGTCA---
ATGGCGCTGACGGTACAG---GCCAGAAAT---TTATTG---TCTGGTATAGTGCAACAGCAAAGT---
AATTTG---CTGAGG---GCTATAGAGGCGCAACAGCATATGTTGCAACTCACGGTCTGGGGCATC
>DQ382362
TTCTATTGCAATACATCAGACCTGTTTAAGGATAATATTACTATAACTAATAGTACT----------------
----

AACAATACAGTTATTACACTCCAATGCAGAATAAAACAAATTATAAACATGTGGCAAAGGGCAGGACAGGCAA
TATATGCCCCTCCCATTAGAGGAAACATAACATGTAATTCAAAT---
ATCACAGGACTACTATTGACACGTGATGGAGGAAAAGATAATAAAACAAAT----------------------
----

AATGAGAATAAAACAGAGATATTCAGACCTGGAGGAGGAGATATGAGGGACAATTGGAGAAGTGAATTATATA
AATACAAAGTAGTAGAAATTAAGCCATTA---GGAATAGCACCCACTACTGCAAAAAGGAGAGTGGTG-----
----------------------------------GAGAGAGAGAAAAGAGCA---GTGGGA---
ATAGGAGCT---------GTGCTCCTTGGGTTCTTGGGAGCA---GCAGGAAGCACTATG---
GGCGCAGCGTCA---ATAACGCTGACGGCACAG---GCCAGACAA---GTATTG---
TCTGGTATAGTGCAACAGCAAAGT---AATTTG---CTGAGA---
GCTATAGAGGCGCAACAGCATATGTTGCAGCTCACAGTCTGGGGCATT
>DQ382364
TTCTATTGCAATACATCAAAACTGTTTAATGGTACATACAATGGTACAGACATGTCTAATAATACAGAA----
----

AGTAATTCAACCATCACACTCCAATGCAGAATAAAACAAATTATAAACATGTGGCAGGAGGTAGGACGAGCAA
TGTATGCCCCTCCCATTGCAGGAAACATAACATGTAGATCAAAT---

ATCACAGGGCTACTATTGCTACGTGATGGAGGTTTCTGCCAAAAT----------------------------
----

AATACCACAGAGACATTCAGGCCGGGAGGGGGAGATATGAGGAACAATTGGAGAAGTGAATTATATAAGTATA
AAGTGGTAGAAATTAAGCCATTG---GGAGTAGCACCCACTAAGGCAAAAAGGAGAGTGGTG-----------
----------------------------GAGAGAGAAAAAAGAGCA---GTGGGA---ATAGGAGCT------
---GTGTTCCTTGGGTTCTTGGGAGCA---GCAGGAAGCACTATG---GGCGCGGCGTCA---
ATAACGCTGACGGTACAG---GCCAGACAA---CTGTTG---TCTGGCATAGTGCAACAGCAGAGC---
AATTTG---CTGAGA---GCTATAGAGGCGCAACAGCATCTGTTGCAACTCACAGTCTGGGGCATT
>DQ382365
TTCTATTGCAATACATCAAGCCTGTTTAATGGGACATACAATAGTACACGCAATAGT----------------
----

ACAGGAAGGACTATCACACTCCAATGCAGAATAAAACAAATTATAAACATGTGGCAGGAGGTGGGACGAGCAA
TGTATGCCCCTCCCATTGCAGGGAACATAACATGTAATTCAAAT---
ATCACAGGACTAATATTGACACGTGATGGAGGATATGATAATAATAATGCCACT------
AAAGAAAATAAGACTACTAATAATACT--

AATAGTAATAAAACAGAGAAATTCAGACCTGTAGGAGGAGAGATGAGGGATAATTGGAGAAGTGAATTATATA
AATATAAAGTGGTAGAAATTAAGCCATTA---GGAATAGCACCCACTAAGGCAAAAAGGAGAGTGGTG-----
----------------------------------GAAAGGGAGAAAAGAGCA---GTGGGA---
CTAGGAGCT---------GTGTTCCTTGGGTTCTTGGGAGCA---GCAGGAAGCACTATG---
GGCGCGGCGTCA---ATAACGCTGACGGTACAG---GCCAGACAA---TTATTG---
TCTGGTATAGTGCAACAGCAAAGC---AATTTG---CTGAGA---
GCTATAGAGGCGCAACAGCATCTGTTGCAACTCACGGTCTGGGGCATT
>DQ382366
TTCTATTGCGATACATCAAAATTGTTTAATAGGACATTTAATAGTACTAATACAAAAGGTAATAGTACAGATG
CTAGA--
-------------------GGT---
AATAGTAATGCAACAATCACACTCCCATGCAGGATAAAACAATTTATACACATGTGGCAGCCAGTAGGAAGGG
CAATGTATGCTCCGCCCATTGCAGGAAAAATAACATGTAAATCAAAT---
ATCACAGGATTACTATTGGCGAGAGATGGAGGAAACACAAATAAC----------------------------
----

TCAGAGACATTCAGACCTGGAGGAGGAAATATGAAGGACAATTGGAGAAGTGAATTATACAAATATAAAGTGG
TAGAAGTTAAGCCATTG---GGAATAGCACCCACTGGAGCAAAAAGGAGAGTGGTG-----------------
----------------------GAGAGAGAAAAAAGAGCA---GTGGGA---ATAGGAGCT---------
GTGTTCCTTGGGTTCTTGGGAGCA---GCAGGAAGCACTATG---GGCGCGGCGTCA---
ATAACGCTGACGGTACAG---GCCAGACAA---CTGTTG---TCTGGTATAGTGCAACAGCAAGGC---
AATTTG---CTGAGA---GCTATAGAGGCGCAACAGCATATGTTGCAACTCACGGTCTGGGGCATT
>DQ382367
TTCTATTGTAATACATCACAACTGTTCAATAGCACATACAATAGCAATGAGACAACAAGTGAT----------
----

TCAAATTCAACCATCACACTCCCATGTACAATAAAACAAATTATAAATATGTGGCAGGAGGTGGGACGAGCAA
TGTATGCCCCTCCCATTGCAGGAAACATAACATGTGTATCAAAC---
ATCACAGGAATACTATTGACACGTGATGGAGGACAAAATGGAGGAAACAAC----------------------
----

ACAAATGTCCCAGAGATATTCAGACCTGCAGGAGGAGATATGAGGCTCAATTGGAGAAGTGAATTATTTAAAT
ATAAAGTAGTAGAAATTAAGCCATTG---GGAATAGCACCCACTGAGGCAAAAAGGAGAGTGGTG--------
-------------------------------GAGAGGGAAAAAAGAGCA---GTGGGA---ATAGGAGCT---
------GTGTTCCTTGGGTTCTTGGGAGCA---GCAGGAAGCACTATG---GGCGCGGCGTCA---
GTGACGCTGACGGTACAG---GCCAGACAA---TTGTTG---TCTGGTATAGTGCAACAGCAAAGC---
AATTTG---CTGAGG---GCTATAGAGGCGCAACAGCACATGTTGCAACTCACGGTCTGGGGCATT
>DQ396372
TTCTATTGCAATACAACAAAACTGTTTAATGAAACGCTTTTTTCGAAGGAA----------------------
----

ACTAATGAAACCATAACCATCCAATGCAGGATAAAACAAATTATAAACATGTGGCAGGGGGTAGGACGAGCAA

TGTATGCCCCTCCCGTTGCAGGAAACATAACATGTAACTCAAGT---
ATCACAGGAATACTATTAACACGTGATGGCGGTTTAAAAAACGACACC-------------------------
----

AATGACACAGAGACATTCAGACCTGGAGGAGGAAATATGAAGGACAATTGGAGAAGTGAATTATATAAATATA
AAGTGGTAGAAATTAAGCCTTTG---GGAATGGCACCCACTAGGGCAAAAAGGAGAGTGGTG-----------
----------------------------GAGAGAGAAAAAAGAGCA---GTGGGA---ATAGGAGCT------
---GTGTTCCTTGGGTTCTTGGGAGCA---GCAGGAAGCACTATG---GGCGCAGCGTCA---
ATAACGCTGACGGTACAG---GCCAGACAA---CTGTTG---TCTGGTATAGTGCAACAGCAGAGC---
AATCTG---CTGAGA---GCTATAGAGGCGCAACAGCATATGTTGCAACTCACAGTCTGGGGCATT
>DQ369992
TTCTATTGCAATACATCAAAGCTGTTTACTGAATCGCTTAATAGTACA-------------------------
----

GACAATGAAACAATCACAATCCCATGTAAACTAAAACAGATTATAAACCTGTGGCAGGAAGTAGGGCGAGCAA
TGTATGCCCCTCCCATTGAAGGAAACATAACATGTAACTCAAGT---
ATCACAGGACTACTATTGACACGTGATGGAGGAAACACGAATGACACA-------------------------
----

AATGGCCCAGAGATATTCAGACCTGGAGGAGGGAATATGAAGGACAATTGGAGAAGTGAATTATATAAATATA
AAGTGGTAGAAATTAAGCCATTG---GGAATAGCACCCACTGGAGCAAAAAGGAGAGTGGTG-----------
----------------------------GAGAGAGAAAAAAGAGCA---GTGGGA---ATAGGAGCT------
---ATGATCCTTGGGTTCTTGGGAGCA---GCAGGAAGCACTATG---GGCGCGGCGTCA---
ATAACGCTGACGGTACAG---GCCAGACAA---TTGTTG---TCTGGTATAGTGCAACAGCAAAAT---
AATTTG---CTGAGG---GCTATAGAGGCGCAACAGCATATGTTGCAACTAACGGTCTGGGGCATT
>DQ369982
TTCTATTGTAATACATCAGGACTATTTAATGGTACATATAATAGTACAGGATCTAATAGTACAGGA-------
----

AATGGTTCAGTCATTACACTTCCATGCAGAATAAAACAAATTATAAACATGTGGCAGGAAGTAGGACGAGCAA
TGTATGCCCCTCCCATTGCAGGAAACATAACATGTGAGTCAAAT---
ATCACAGGACTACTATTAGTACGTGATGGTGGCAACACAAGTACA----------------------------
----

GAAGAAATATTCAGACCTGGGGGAGGAAATATGAAGGACAATTGGAGAAGTGAATTATATAAATATAAAGTGG
TAGAGGTTAAGCCATTG---GGAATAGCACCCACTAGAGCAAAAAGGAGAGTGGTG-----------------
----------------------GAGAGAGAGAAAAGAGCA---GTGGGA---ATAGGAGCT---------
GTGTTCCTTGGGTTCTTGGGAGCA---GCAGGAAGCACTATG---GGCGCAGCGTCA---
ATAGCGCTGACGGCACAG---GCCAGACAA---GTGCTG---TCTGGTATAGTGCAACAGCAAAGC---
AATTTG---CTGAGG---GCTATAGAGGCGCAACAGCATATGTTGCAACTCACAGTCTGGGGCATT
>DQ369983
TTCTACTGCAATACAACACAACTGTTCAATACAACAAAACTGTTCAATAGCACA-------------------
----

ACTAATGAAACTATCATACTCCCATGCAGAATAAAACAAATTATAAATATGTGGCAGGAGGTAGGACGAGCAA
TGTACGCCCCTCCCATTAGAGGAAACATAACATGTAACTCAAGT---
ATCACAGGGCTGCTATTGGAACGTGATGGAGGAAATGTAAACAGCAGC-------------------------
----

AATAGCACAGAGACATTCAGACCTGGAGGAGGAAATATGAAGGACAATTGGCGTAGTGAATTATATAAATATA
AAGTGGTAGAACTTAAGCCATTG---GGAATAGCACCCACGGATGCAAAAAGGAGAGCGGTG-----------
----------------------------GAGAGAGAAAAAAGAGCA---GTGGGA---ATAGGAGCT------
---GTGCTCCTTGGGTTCTTGGGAGCA---GCAGGAAGCACTATG---GGCGCGGCGTCA---
ATAACGCTGACGGTACAG---GCCAGGCAA---TTATTG---TCTGGTATAGTGCAACAGCAAAGC---
AGTTTG---CTGAGA---GCTATAGAGGCGCAACAGCATATGTCGCAACTCACGGTCTGGGGCATT
>DQ369994
TTCTATTGCAATACATCAAGACTGTTTACATCAAACCTGTCTAGTAAT-------------------------
----

ACAGAAGACATCATCACAATCCCATGCAGAATAAAACAGGTTATAAACATGTGGCAGAAGGTAGGACGAGCAA

TGTATGCCCCTCCCATTGCAGGAAACATAACATGTAGATCCAAC---
ATCACAGGACTACTATTGACACGTGATGGAGGATCAGATAACCATACC-------------------------
----

AATAACACAGAGACTTTCAGACCTGGAGGAGGAGACATGAGGGATAATTGGAGAAGTGAATTATATAAATATA
AAGTAGTAGAAATTAAGCCATTG---GGAATAGCACCCACCAAGGCAAAAAGGAGAGTGGTG-----------
----------------------------GAGAGAGAAAAAAGAGCA---GCGGGA---ATAGGAGCT------
---GTGTTCCTTGGGTTCTTGGGAGCA---GCAGGAAGCACTATG---GGCGCGGCGTCA---
ATAACGCTGACGGTACAG---GCCAGACAA---TTGTTG---TCTGGTATAGTGCAACAGCAAAGC---
AATTTG---CTGAAG---GCTATAGAGGCGCAACAGCATCTGTTGCAACTCACGGTCTGGGGCATT
>HQ615948
TTCTATTGCAATACATCAGGCCTATTTAATAGAAAATACTCCTTTAATGATACATACAATAGTACAGAA----
----

AATAGTTCAAACATCACACTCCAATGCAGAATAAAACAAATTATAAACATGTGGCAGGGGGTAGGACGAGCAA
TGTATGCCCCTCCCATTGAAGGAAATATAACATGTAAATCAAAG---
ATCACAGGACTACTATTAGTACGTGATGGAGGCAACCCTGACAATAGC-------------------------
----

AGTGGCACAGAGACATTCAGACCTGGAGGAGGAAACATGAAGGACAATTGGAGAAGTGAATTATATAAATATA
AAGTGGTAGAAATTAAACCATTA---GGAATAGCACCAACTAATGCAAAAAGGAGAGTGGTG-----------
----------------------------GAGAGAGAAAAAAGAGCA---GTGGGA---ATAGGAGCT------
---GTGTTCCTTGGGTTCTTGGGAGCA---GCAGGAAGCACTATG---GGCGCGGCGTCA---
GTGACGCTGACGGTACAG---GCCAGACAA---TTACTG---TCTGGTATAGTGCAACAGCAAAGC---
AATTTG---CTAAGG---GCTATAGAGGCGCAACAACATATGTTGCAACTCACGGTCTGGGGCATT
>JQ754193
TTCTATTGCAATACATCAAAACTGTTCAATAGTACATACATGGCTAATAATACAGACGTGTCTAATAGTACTT
TAAATAGTACT--

TCAAATGCAACCATCACACTCCCATGCAGAATAAAACAAATTATAAACATGTGGCAGGGGGTAGGACGGGCAA
TGTATGCCCCTCCCATTGCAGGAAACATAACATGTAAGTCAAAT---
ATCACAGGAGTACTATTGACACGTGATGGGGGAGAAAGCAATGGG----------------------------
----

ACAGAAGAAGTCTTCAGACCTGGAGGAGGAGATATGAGGGACAATTGGAGAAGTGAATTATATAAGTATAAAG
TGGTAGAAATTAAGCCATTA---GGAATAGCACCCACTGGGGCAAAAAGGAGAGTGGTG--------------
-------------------------GAGAGAGAAAAAAGAGCA---GTAGGA---ATAGGAGCT---------
GTGTTCCTTGGGTTCTTGGGAGCA---GCAGGAAGCACTATG---GGCGCAGCATCA---
ATAACGCTGACGGTACAG---GCCAGACAA---TTGTTG---TCTGGTATAGTGCAACAGCAAAGC---
AATTTG---CTGAGG---GCCATAGAGGCGCAACAACATTTGTTGCAACTCACGGTCTGGGGCATT
>GQ999981
TTCTATTGTAATACAGCAAAACTATTTAACACAACACAGTTTAATAACAAT----------------------
----

GATGCAAACACAACCACCATCCCATGCAGGATAAAACAAATTATAAACATGTGGCAGGGGGTAGGACGAGCAA
TGTATGCCCCTCCCATTGAAGGAAACATAACATGTAACTCAAGT---
ATCGCAGGACTACTATTGACACGTGATGGCGGCCAGCAGAATACA----------------------------
----

AATGAGACATTCAGACCTGGAGGAGGAAATATGAAGGACAATTGGAGAGGTGAATTATATAAATATAAAGTGG
TAGAAATTAAGCCATTG---GGGGTAGCACCCACTGAGGCAAAAAGGAGAGTGGTG-----------------
----------------------GAGAGAGAAAAAAGAGCA---GTGGGA---ATAGGAGCT---------
GTGCTCCTTGGGTTCTTGGGAGCA---GCAGGAAGCACTATG---GGCGCGGCGTCA---
ATAACGCTGACGGTACAG---GCCAGACAA---CTGTTG---TCTGGTATAGTACAACAGCAAAGC---
AATTTG---CTGAGA---GCTATAGAGGCGCAACAGCATATGTTGCAACTCACAGTCTGGGGCATC
>KC894135
TTCTATTGCGACACAACAACACTGTTTAATGACACACTGTTTAATAACACACTGTCTGATAAT----------
----

GAAAATGAGACAATCACACTCCCATGCAGAATAAAACAAATTATAAACATGTGGCAGGGGGTAGGACGAGCAA

TATATGCCCCTCCCATTGAAGGGAAAATAACATGTAACTCAACT---
ATCACAGGACTACTATTGACACGTGATGGAGGAGTAAATGCTAGT----------------------------
----

AACAAAGCAGAGACATTCAGACCTGGAGGAGGAAATATGAAGGACAATTGGAGAAGTGAATTATATAAGTATA
AAGTGGTAGAAATTAAGCCATTG---GGAGTAGCACCCACTGGGGCAAAAAGGAGAGTGGTG-----------
----------------------------GAGAGAGAAAAAAGAGCA---GTGGGA---ATGGGAGCT------
---GTGCTCCTTGGGTTCTTGGGAGCA---GCAGGAAGCACTATG---GGCGCAGCGTCA---
ATAACGCTGACGGTACAG---GCCAGACAA---CTGTTG---TCTGGTATAGTGCAACAGCAAAGC---
AATTTG---CTGAGA---GCTATAGAGGCGCAACAGCATATGTTGCAACTCACGGTCTGGGGCATT
>GQ999983
TTCTATTGCAATACCACAAAACTGTTTAATAGTACACATAATAGTACTGATAGTACAGTAAATAGTACTGATA
GTACAGCAGAA--
----------ACAGGAAAT------
TCAACAAATACAAACATCACGCTCCCATGCAGAATAAGGCAAATTATAAACATGTGGCAGGAGGTAGGACGAG
CAATGTATGCTCCTCCCAGTAAAGGGAACATAACATGTATATCAAAC---
ATCACAGGACTATTACTGACGCGTGATGGAGGAGAAAACAAAACAGAG-------------------------
----

AATAATGACACAGAGATATTCAGGCCTGGAGGAGGAGATATGAAGGACAATTGGAGAAGTGAATTATATAAAT
ATAAAGTAGTAGAAATTAAACCATTA---GGAGTAGCACCCACTAGAGCAAAAAGGAGAGTGGTG--------
-------------------------------GAGAGAGAAAAAAGAGCA---GTGGGA---ATAGGAGCT---
------GTGTTCCTTGGGTTCTTGGGAGCA---GCAGGAAGCACTATG---GGCGCAGCATCC---
ATAACGCTGACGGTACAA---GCCAGACAA---TTATTG---TCTGGTATAGTGCAGCAGCAAAGT---
AATTTG---CTGAGA---GCTATAGAGGCGCAACAACATATGTTGCAACTCACAGTCTGGGGCATT
>GQ999984
TTCTATTGCAATACAACAAAACTGTTTAATGAAAATGATACAAAATTGTTTAATGAAAGTGCTACAATTAAAA
ATGAA--
-------------------AGT---
AATACAAATGAAAACATCACAATCCCATGCAGAATAAAACAAATTATAAACATGTGGCAGGGGGTAGGACGAG
CAATGTATGCCCCTCCCATTGCAGGAAACATAACATGTAAATCAAAT---
[truncated: 555,191 more chars]
